# A resource of ribosomal RNA-depleted RNA-Seq data from different normal adult and fetal human tissues

**DOI:** 10.1038/sdata.2015.63

**Published:** 2015-11-10

**Authors:** Jocelyn Y.H. Choy, Priscilla L.S. Boon, Nicolas Bertin, Melissa J. Fullwood

**Affiliations:** 1 Cancer Science Institute of Singapore, National University of Singapore, Singapore 117599, Singapore; 2 School of Biological Sciences, Nanyang Technological University, Singapore 637551, Singapore; 3 Institute of Molecular and Cell Biology, Agency for Science, Technology and Research (A*STAR), Singapore 138673, Singapore; 4 Yale-NUS Liberal Arts College, Singapore 138527, Singapore

**Keywords:** Transcriptomics, Development, RNA sequencing

## Abstract

Gene expression is the most fundamental level at which the genotype leads to the phenotype of the organism. Enabled by ultra-high-throughput next-generation DNA sequencing, RNA-Seq involves shotgun sequencing of fragmented RNA transcripts by next-generation sequencing followed by *in silico* assembly, and is rapidly becoming the most popular method for gene expression analysis. Poly[A]+ RNA-Seq analyses of normal human adult tissue samples such as Illumina’s Human BodyMap 2.0 Project and the RNA-Seq atlas have provided a useful global resource and framework for comparisons with diseased tissues such as cancer. However, these analyses have failed to provide information on poly[A]−RNA, which is abundant in our cells. The most recent advances in RNA-Seq analyses use ribosomal RNA-depletion to provide information on both poly[A]+ and poly[A]−RNA. In this paper, we describe the use of Illumina’s HiSeq 2000 to generate high quality rRNA-depleted RNA-Seq datasets from human fetal and adult tissues. The datasets reported here will be useful in understanding the different expression profiles in different tissues.

## Background & Summary

**Figure 1:**
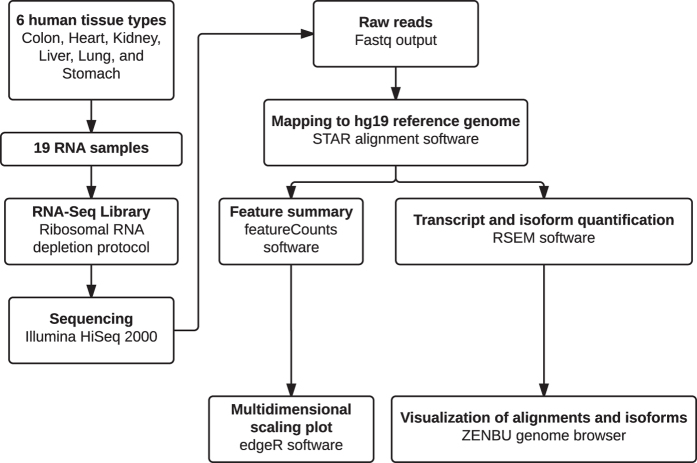
Flow Chart of the RNA-seq experiment and data analysis.

Next Generation Sequencing (NGS) technique uses short read lengths to enable the massive parallel sequencing of the genome. Currently with paired-end sequencing and multiplexing technology, NGS allows multiple samples to be studied at the same time, dramatically reducing the time spent generating data and simplifying the procedure. RNA-Seq is a technique used to study the transcriptome and involves the direct sequencing of transcripts by Next Generation Sequencing Technologies (Fig. 1). It allows the analysis of known transcripts and explores new transcripts.

There are various different databases on the web which provide information about the expression profiles of different tissues using RNA-Seq. Some of these databases include Illumina’s Human BodyMap 2.0 and the RNA-Seq Atlas. Illumina’s Human BodyMap 2.0 project data was generated in 2010 using its HiSeq 2000 instrument. This project consisted of 16 adult human tissue types including adrenal, adipose, brain, breast, colon, heart, kidney, liver, lung, lymph, ovary, prostate, skeletal muscle, testes, thyroid, and white blood cells from different individuals. RNA-Seq libraries were constructed using poly[A]+ mRNA and sequenced using small RNA-sequencing. For each tissue, the raw reads were aligned to the genome and then the exons were linked into tissue-specific transcript models using the reads that span an exon-exon boundary. A diverse RNA-Seq library database of 16 different tissues is available which makes it very comprehensive. However, there is no information about poly[A]− RNA.

The RNA-Seq Atlas data was generated from 11 healthy, human tissue samples pooled from multiple donors. The tissues include adipose, colon, heart, hypothalamus, kidney, liver, lung, ovary, skeletal muscle, spleen and testes. Both poly[A]+ and poly[A]− RNA were used for sequencing. The expression levels were estimated by mapping and counting reads to single gene sequences derived from the UCSC genome browser, followed by normalization to Reads Per Kilobase of exon model per Million (RPKM) mapped reads values^1^. However, this database lacks the information of fetal tissues as only adult tissues are included in this study. Hence, our study aims to address this situation and allow the comparison between adult and fetal tissues^2^.

Ribosomal RNA (rRNA) is the most abundant transcript in total RNA and constitutes about 80% of the total RNA, while poly[A]+ mRNA constitutes only about 5% of the total RNA present in an eukaryotic cell. rRNA provides little information about the transcriptome, hence it would be beneficial to remove rRNA to maximise the amount of information retrieved from a sequencing run. The use of ribosomal depleted RNA has been shown to recover more information about protein-coding genes, non-coding RNAs, snRNAs, snoRNAs and repeat elements^3^. The use of rRNA-depleted RNA-Seq on mouse cerebrum tissues has enabled more novel transcribed loci to be detected than mRNA-seq with the former yielding 9,428 novel transcribed loci and the latter yielding only 4,550 (ref. 3). The study also observed higher proportions of reads from intergenic (44%) and intronic (25%) regions from rRNA-depleted RNA-Seq compared to mRNA-seq (23% and 15% respectively) indicating better recovery of poly[A]− or bimorphic transcripts^3^. A comparison between Ribo-Zero-Seq and mRNA-Seq was demonstrated on fresh frozen tissues from multiple tumours, which included 11 human breast tumor samples as well as sets of tumors from The Cancer Genome Atlas and University of North Carolina. Transcript coverage is determined by measuring the variation at the 5′ and 3′ ends. This is achieved by evaluating the ratio of coverage at the 5′ end relative to the 3′ end for the 1,000 most highly expressed transcripts. It is shown that when applied to fresh frozen samples, rRNA-depletion RNA-Seq provides a less biased 5′-to-3′ coverage ratio compared to mRNA-Seq as it does not rely on the poly[A] selection step. Another advantage of rRNA-depleted RNA-Seq is its ability to measure immature transcripts (pre-mRNA), providing more information on splicing patterns and splice junctions^4^. Ribosomal depleted RNA datasets such as the characterization of the landscape of transcription in humans as part of the ENCODE consortium has allowed the further annotation of the human genome and discovery of novel transcripts^5^ and recently has been used to identify zero nucleotide recursive splicing in drosophila and humans^6^.

Our project aims to compare the expression between different fetal and adult tissues and to identify tissue-specific genes and expression profiles. Constructing ribosomal depleted RNA-Seq libraries in fetal tissues would provide insights into the early gene expression patterns in these tissues. Studying both fetal and adult gene expressions could give us a greater understanding of the regulated genes in different developmental stages and its effects on developmental functions. Study of these RNA-sequences will allow us to determine tissue-specific splice variants as well as non-coding RNA. Genome wide screens in different tissue types might also help us to identify polymorphisms and their effects on overall expression and splicing.

## Methods

### Total RNA preparation

2 different fetal normal tissues and 6 different adult normal tissues were purchased from different sources (Agilent Technologies, Biochain and OriGene). The qualities of these total RNA were tested using the Agilent Bioanalyser 2100 Eukaryote Total RNA Nano Series II. Only total RNAs with a RIN score of more than 7 were used for RNA-Seq library construction. Details of the total RNAs used were listed in Table 1 (available online only).

### rRNA depletion of total RNA

Ribosomal RNA (rRNA) was removed from total RNA using the RiboMinus Eukaryote Kit for RNA-Seq from Ambion. The ribosomal RNA depleted RNA fraction is termed the RiboMinus RNA fraction and is enriched in polyadenylated (polyA) mRNA, non-polyadenylated RNA, pre-processed RNA, tRNA, and may also contain regulatory RNA molecules such as microRNA (miRNA) and short interfering RNA (siRNA), snRNA, and other RNA transcripts of yet unknown function. Ambion RiboMinus rRNA depletion was performed as described in the manufacturer’s protocol (Pub. Part no.: 100004590, Rev. date 2 December 2011) following the standard protocol.

### TruSeq RNA sample preparation v2

TruSeq RNA Sample Preparation was performed on the RiboMinus RNA fraction as described in the manufacturer’s protocol (Pub. Part no.: 15026495, Rev. F March 2014, RS-122-9001DOC) following the low sample protocol. The libraries were sequenced on Illumina’s HiSeq 2000 instrument.

### Quality validation and read mapping

The quality of the RNA-seq libraries were evaluated using fastQC (http://www.bioinformatics.babraham.ac.uk/projects/fastqc/), picard tools (http://picard.sourceforge.net) RseQC^7^. The reads were aligned to the UCSC human genome release version hg19 reference genome using STAR^8^ and annotated using the Gencode version 19 annotation. Subsequent quantification of expression levels and isoforms was done using RSEM^9^. The STAR_RSEM.sh script was used for the processing of the data (https://github.com/ENCODE-DCC/long-rna-seq-pipeline/blob/master/DAC/STAR_RSEM.sh). The aligned reads were uploaded into ZENBU^10^ a genome browser for visualization. The program featureCounts^11^ was used to summarize the features of all the RNA-seq library. The output from featureCounts was inputted into the program edgeR^12^ to generate the multidimensional scaling plot.

## Data Records

The raw fastq files for the RNA-seq libraries were deposited at NCBI Sequence Read Archive (SRA) with BioProject accession PRJNA283012 (Data Citation 1: Sequence Read Archive PRJNA283012). The output from the quantification of transcripts by RSEM were deposited at Gene Expression Omnibus (GEO) with accession GSE69360 (Data Citation 2: Gene Expression Omnibus GSE69360). These files are tab-delimited files in the format suitable for viewing in the genome browser ZENBU. It contains the location of the transcripts on the genome, counts and normalized counts. The supplementary file GSE69360_RNAseq.counts.txt.gz contains counts used to construct the MDS plot.

## Technical Validation

### Quality control-RNA integrity

The integrity of the total RNA was measured by the RNA Integrity Number (RIN) algorithm; calculated by the Agilent Bioanalyzer software. It was determined by the entire electrophoretic trace of the RNA sample. This included the presence or absence of degradation products. The higher the RIN score, the better the integrity of the total RNA, with the highest RIN score of 10. All the total RNAs used for rRNA-depleted RNA-Seq had a RIN score of above 7 which showed the high integrity of total RNAs used. The Agilent Bioanalyzer profiles for the total RNA and RIN scores were listed in Supplementary File 1.

### Quality control-Library quality

All the rRNA-depleted RNA-Seq libraries had a library size range between 250 to 400 bp which was similar to the expected library size distribution as shown in the TruSeq RNA Sample Preparation manufacturer’s protocol (Pub. Part no.: 15026495, Rev. F March 2014, RS-122-9001DOC). This library size range was ideal for sequencing on the HiSeq 2000 instrument. The Agilent Bioanalyzer profiles for the rRNA-depleted RNA were listed in Supplementary File 2.

### Quality validation and analysis

**Figure 2:**
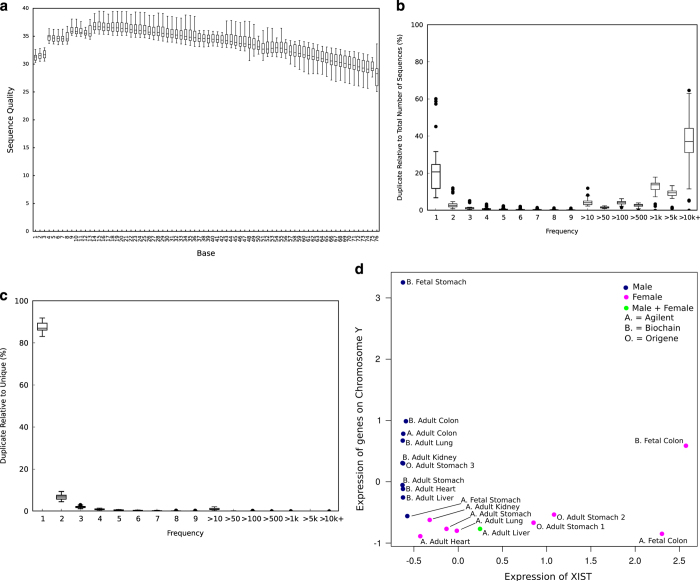
Summary of key quality control metrics. (**a**) Boxplot of average sequence quality per base per sample. (**b**) The distribution of duplicated reads relative to the total number of sequences for all libraries. (**c**) The distribution of unique reads for all libraries. (**d**) XIST versus ChrY: normalized expression of female specific transcript XIST (x-axis) versus sum of normalized expression of all Y chromosome transcripts excluding those in the pseudo-autosomal regions (y-axis).

To assess the quality of the RNA-seq libraries, the average quality score per base per library, duplication levels relative to total number of sequences, duplication levels relative to unique sequences of the raw fastq files were examined using fastQC (http://www.bioinformatics.babraham.ac.uk/projects/fastqc/). The average quality score per base per library for both the forward and reverse read was plotted. The average quality score per base was high with a median score above 30 indicating high quality sequences across all bases (Fig. 2a). There was no significant difference in the distribution of average quality score per base between the forward and reverse reads of all libraries (Supplementary Figs S1, S2 and Supplementary File 4). The duplication levels relative to the total number of sequences show a distribution that is skewed to the right of the graph (Fig. 2b). This is expected of deeply sequenced enriched libraries with 5 to 10 percent of the sequences duplicated 10 to 100 times due to highly expressed transcripts. However, the high levels of duplication of >1 k to >10 k+ indicates contamination of the library possibly from residual ribosomal RNA as observed from the report of overrepresented sequences (Supplementary File 3). However when the duplication levels relative to unique sequences were examined the number of unique contaminants was extremely low (Fig. 2c).

The RNA-seq libraries were aligned to the reference genome hg19 using STAR^8^ and gene expression and isoform levels were quantified using RSEM^9^. The mappability of the reads range from 55.5% to 96.3% (Table 1 (available online only)). The reads that were unable to be mapped were due to read sizes that were too short to be accurately mapped to the reference genome. To determine if there was any 5′ to 3′ bias in the data, a gene body coverage analysis was conducted. The gene body coverage of the mapped reads for all libraries were determined using the geneBodyCoverage.py script from the RseQC package and showed no significant 5′ or 3′ end bias (Supplementary Figs S3, S4 and Supplementary File 4). To check for sample swaps a XISTvsChrY analysis as published by ‘t Hoen *et al.* ^13^ was conducted to confirm the gender of each sample (Fig. 2d). The analysis shows a separation of the samples originating from males and females and correspond to the genders as labelled by the suppliers. However the biochain fetal colon sample show both high expression of XIST and ChrY genes and is indicative of possible contamination.

**Figure 3:**
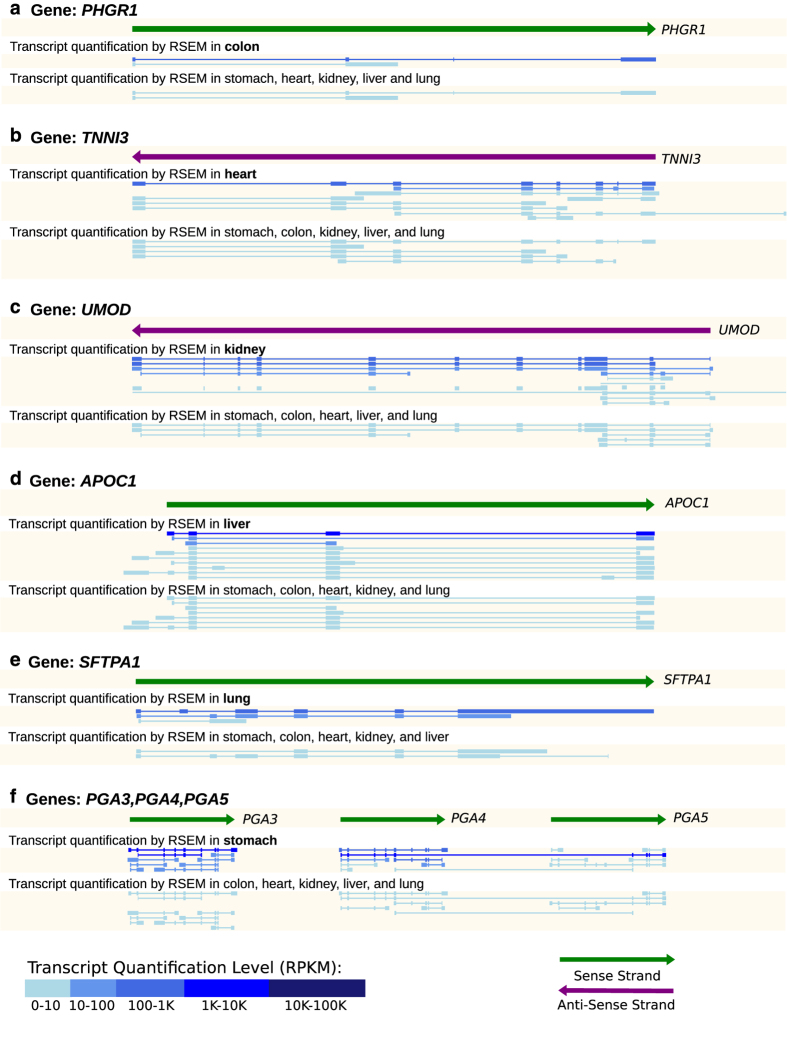
A representation of tissue specific genes with transcripts from corresponding RNA-seq libraries contrasted with transcripts from other tissue samples. (**a**) PHGR1, (**b**) TNNI3, (**c**) UMOD, (**d**) APOC1, (**e**) SFTPA1 and (**f**) PGA(3–5) are highly expressed in the colon, heart, kidney, liver, lung and stomach respectively. RNA-seq libraries from the same tissue type show higher expression of transcripts when compared to RNA-seq libraries of different tissue types.

**Figure 4:**
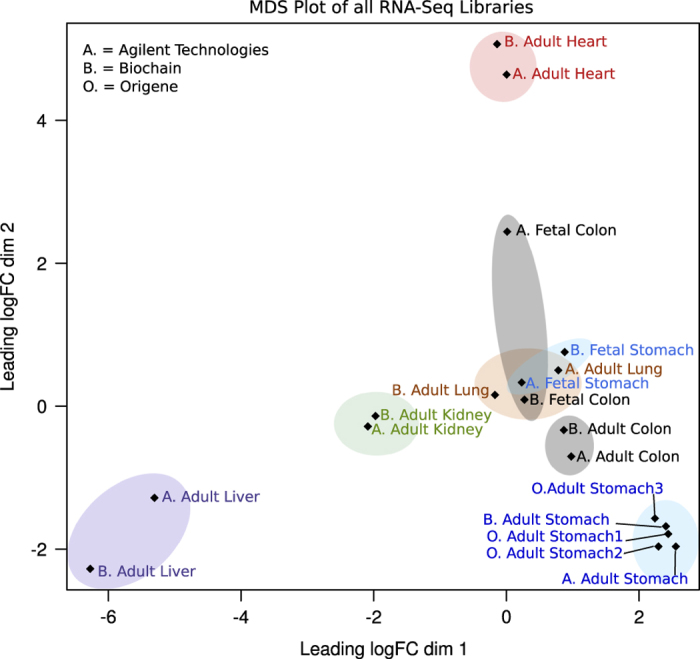
A multidimensional scaling plot for all libraries. The different tissue types are colored differently. Similar tissue types cluster together.

In order to further assess the quality of the RNA-seq libraries, the quantified transcript isoforms were visualized using ZENBU^10^ and the transcripts of known highly expressed tissue specific genes were examined. The libraries consistently showed enrichment of tissue specific genes only in the corresponding tissue libraries for both adult and fetal replicates. For example stomach tissue samples from both adult and fetal samples show the high expression of PGA3, PGA4 and PGA5 all of which are pepsinogen I genes that transcribes pepsinogen which is later converted in the stomach as the proteolytic enzyme pepsin (Fig. 3). A multidimensional scaling plot was plotted using the output from featureCounts to observe the clustering of the libraries. As expected, libraries originating from the same tissue type and stage of development (i.e., adult stomach or fetal stomach) cluster together (Fig. 4). However it is notable that the fetal stomach samples cluster close to the fetal colon and adult lung samples rather than to the adult stomach samples. This may be due to the difference in genes that are expressed during the developmental stage of the fetus compared with genes expressed in adult tissues. The difference in clustering of the fetal colon samples is possibly caused by difficulties in collection of fetal tissue.

## Usage Notes

As demonstrated above, the raw RNA-Seq fastq files may be aligned using popular genome aligners such as bowtie2 (ref. 14), TopHat2 (ref. 15) and STAR^8^ and viewed on genome browsers such as the UCSC genome browser^16^ or ZENBU^10^. Differential gene expression can be carried out by publicly available software such as edgeR^12^ and DESeq^17^.

A major advantage of this project is that total ribosomal RNA-depleted RNAs from the same tissue type in different individuals were sequenced. Biological replicates were important to prove that the abundant RNA-sequences observed was the true representation of the abundant RNA species and not from amplified cDNA libraries. However we note the large portion of transcripts (>30%) with mitochondrial origin in some libraries particularly the colon, heart and kidney. This is consistent with the findings of the GTEx consortium that reported high levels of transcripts with mitochondrial origin from tissues with high aerobic activity such as the heart and kidney^18^. One disadvantage of the RNA-Seq libraries constructed is that the libraries were not strand-specific. Hence, there is no information about the transcriptome annotation and the orientation of the transcripts^7^.

Several uses for the RNA-Seq libraries include identifying and validating new genes and transcripts and comparing expression in different fetal and adult tissues. In order to dissect out gene expression differences that arise due to different individuals, we profiled five normal stomachs from different people. Adult tissues which were used for this analysis included heart, lung, liver, kidney, stomach and colon. Fetal tissues included stomach and colon. In particular, the inclusion of liver and kidney will provide insights into drug toxicity, and the potential side effects of targeting particular drug targets. As normal samples, with no evidence of disease were used for these analyses, they are a useful complement to studies of cancer through RNA-Seq, which uses tissue adjacent to the cancer for comparison. While these tissues show no apparent cancer phenotype, they may not be completely normal, because cancer cells could infiltrate the adjacent tissues. In addition, the adjacent ‘normal’ cells could contain pre-malignant changes. Taken together, this dataset is a useful addition to the general biomedical community.

## Additional Information

Tables 1 is only available in the online version of this paper.

**How to cite this article:** Choy, J. *et al.* A resource of ribosomal RNA-depleted RNA-Seq data from different normal adult and fetal human tissues. *Sci. Data* 2:150063 doi: 10.1038/sdata.2015.63 (2015).

## Supplementary Material



Supplementary File 1

Supplementary File 2

Supplementary File 3

Supplementary File 4

## Figures and Tables

**Table 1:** Details of total RNA used for RNA-seq library construction, corresponding names used in the manuscript and key QC metrics

**Sample name**	**Source**	**Gender**	**Description**	**Sample name as appeared in manuscript**	**number of reads**	**% mapping**	**% reads mapping uniquely**	**% duplicated mapped reads**	**% mapped reads mapping to genes**	**% mapped reads mapping to rRNA**	**% mapped reads mapping to mitochondria**
Agilent_Fetal_Colon	Agilent Technologies	F	Single donor: 20 weeks gestation (Lot no.: 0006071667)	Agilent Fetal Colon	174262897	69.49	65.28	57.5	23.5	28.7	16.2
Agilent_Fetal_Stomach	Agilent Technologies	M	Pool of 10: 18 (2 donors), 19 (3 donors), 20 (4 donors), 21 weeks gestation (Lot no.: 0006049059)	Agilent Fetal Stomach	174661455	55.49	49.89	93.1	16.0	40.0	9.5
Biochain_Fetal_Colon	Biochain	F	Pool of 1 donor, 37 weeks old (Lot no.: B207218)	Biochain Fetal Stomach	78419198	95.35	82.83	27.8	68.4	1.2	1.1
Biochain_Fetal_Stomach	Biochain	M	Pool of 1 donor, 24 weeks old (Lot no.: B303126)	Biochain Fetal Stomach	81875607	96.28	88.62	16.0	48.9	1.0	0.7
Agilent_Adult_Colon	Agilent Technologies	M	Single donor; 82 years old (Lot no.: 0006055759)	Agilent Adult Colon	71105369	68.03	63.37	61.4	31.9	25.7	27.3
Agilent_Adult_Heart	Agilent Technologies	F	Single donor: 73 years old (Lot no.: 0006097996)	Agilent Adult Heart	67643565	65.39	60.58	78.5	31.5	32.4	34.3
Agilent_Adult_Kidney	Agilent Technologies	F	Single donor: 76 years old (Lot no.: 0006068269)	Agilent Adult Kidney	92807528	63.79	58.67	79.4	30.0	30.9	33.8
Agilent_Adult_Liver	Agilent Technologies	M+F	Pool of 3: 30, 44 and 55 years (Lot no.: 0006063161)	Agilent Adult Liver	78137222	68.63	62.94	62.6	36.9	23.7	20.6
Agilent_Adult_Lung	Agilent Technologies	F	Single donor: 40 years old (Lot no.: 0006106003)	Agilent Adult Lung	77032217	58.3	52.88	83.0	17.7	41.8	11.7
Agilent_Adult_Stomach	Agilent Technologies	F	Pool of 3 Donors, 39, 70, & 52 years old (Lot no.: 0006056559)	Agilent Adult Stomach	149287775	57.84	49.92	87.4	23.9	35.6	19.3
Biochain_Adult_Colon	Biochain	M	Single donor; 29 years old (Lot no.: 302060)	Biochain Adult Colon	68628998	65.95	62.3	69.7	24.7	32.4	32.3
Biochain_Adult_Heart	Biochain	M	Single donor; 24 years old (Lot no.: B604038)	Biochain Adult Heart	75787942	73.27	69.42	66.0	41.0	21.4	37.1
Biochain_Adult_Kidney	Biochain	M	Single donor; 26 years old (Lot no.: B106007)	Biochain Adult Kidney	80053022	69.81	65.83	68.5	30.2	27.4	36.3
Biochain_Adult_Liver	Biochain	M	Single donor; 64 years old (Lot no.: B510092)	Biochain Adult Liver	85220810	61.91	56.68	78.0	28.8	30.6	19.8
Biochain_Adult_Lung	Biochain	M	Single donor; 20 years old (Lot no.:B307203)	Biochain Adult Lung	82080766	62.28	57.92	56.4	21.0	25.7	17.3
Biochain_Adult_Stomach	Biochain	M	Single donor; 24 years old (Lot no.: A506301)	Biochain Adult Stomach	83186579	57.41	51.05	90.2	17.7	39.7	22
Origene_Adult_Stomach_0288	Origene	F	Single donor; 44 years old (Cat no.: CR560288)	Origene Adult Stomach 1	88595062	58.29	51.56	74.8	21.9	31.3	18.4
Origene_Adult_Stomach_0393	Origene	F	Single donor; 32 years old (Cat. no.: CR560393)	Origene Adult Stomach 2	89447266	61.15	54.74	66.0	24.7	27.8	18.4
Origene_Adult_Stomach_1840	Origene	M	Single donor; 59 years old (Cat. no.: CR561840)	Origene Adult Stomach 3	81103684	56.28	51.5	83.6	14.4	38.5	27.3
Total number of reads, percentage of reads mapped and percentage of reads mapping uniquely were obtained from the output of the alignment using STAR. The Picard MarkDuplicate tool was used to calculate the percentage of duplicated reads for each library. The percentages of mapped reads that map to genes and rRNA was obtained using RseQC. The percentage of mapped reads that map to mitochondrial chromosome was obtained from the output of featureCounts.											
