# Supplementary material for: A resource of ribosomal RNA-depleted RNA-Seq data from different normal adult and fetal human tissues
Source: Sci Data. 2015 Nov 10;2:150063. doi: 10.1038/sdata.2015.63 (PMC4640133; doi:10.1038/sdata.2015.63)
Supplement: Supplementary File 1 [file sdata201563-s2.pdf]

RNA Integrity Number (RIN) score of total RNA

Sample	Source	RIN score
**Fetal stomach**	Agilent	8.6
**Fetal stomach**	Biochain	7.3
**Fetal colon**	Agilent	8.3
**Fetal colon**	Biochain	7.4
**Adult heart**	Agilent	8.4
**Adult heart**	Biochain	7.4
**Adult lung**	Agilent	7.2
**Adult lung**	Biochain	8.2
**Adult liver**	Agilent	9.3
**Adult liver**	Biochain	8.3
**Adult kidney**	Agilent	9
**Adult kidney**	Biochain	7.1
**Adult stomach**	Agilent	7.5
**Adult stomach**	Biochain	8.7
**Adult stomach**	OriGene (Cat. no.: CR561840)	8.8
**Adult stomach**	OriGene (Cat no.: CR560288)	8.6
**Adult stomach**	OriGene (Cat. no.: CR560393)	8.3
**Adult colon**	Agilent	9.8
**Adult colon**	Biochain	7.5

Table above: RIN scores of the fetal and adult total RNA

Agilent Bioanalyser profiles of total RNA from 2 different fetal tissue types

Fetal stomach

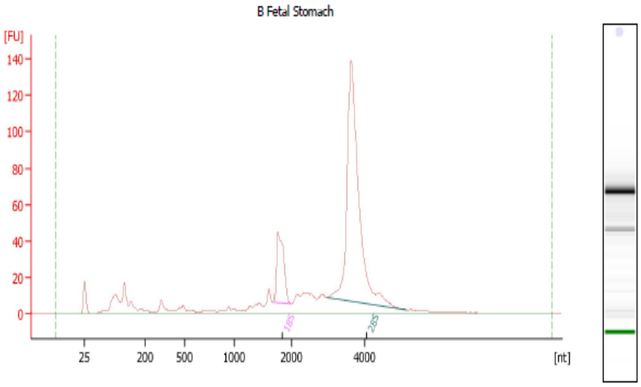

Overall Results for sample 2 : B Fetal Stomach

RNA Area:	821.3	RNA Integrity Number (RIN):	8.6 (B.02.05)
RNA Concentration:	478 ng/µl	Result Flagging Color:	
rRNA Ratio [28s / 18s]:	4.7	Result Flagging Label:	RIN: 8.60

Fragment table for sample 2 : B Fetal Stomach

Name	Start Size [nt]	End Size [nt]	Area	% of total Area
18S	1,689	2,015	76.8	9.3
28S	2,965	5,137	361.9	44.1

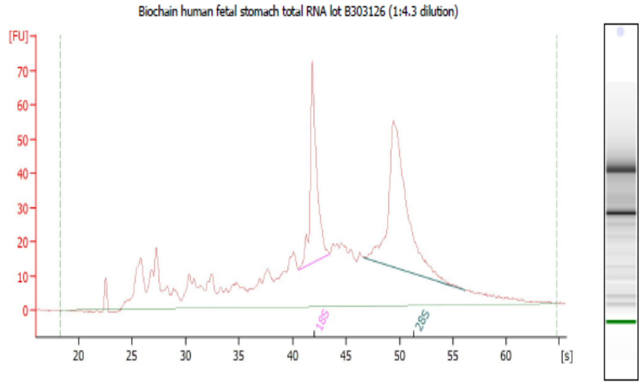

Overall Results for sample 2 : Biochain human fetal stomach total RNA lot B303126 (1:4.3 dilution)

RNA Area:	1,059.3	RNA Integrity Number (RIN):	7.3 (B.02.08)
RNA Concentration:	421 ng/µl	Result Flagging Color:	
rRNA Ratio [28s / 18s]:	1.8	Result Flagging Label:	RIN: 7.30

Fragment table for sample 2 : Biochain human fetal stomach total RNA lot B303126 (1:4.3 dilution)

Name	Start Time [s]	End Time [s]	Area	% of total Area
18S	40.55	43.45	99.7	9.4
28S	46.64	56.11	175.8	16.6

Fetal colon

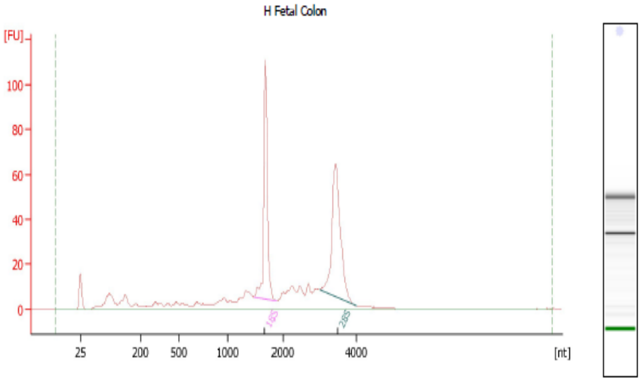

Overall Results for sample 8 : H Fetal Colon

RNA Area:	525.8	RNA Integrity Number (RIN):	8.3 (B.02.05)
RNA Concentration:	306 ng/µl	Result Flagging Color:	
rRNA Ratio [28s / 18s]:	1.2	Result Flagging Label:	RIN: 8.30

Fragment table for sample 8 : H Fetal Colon

Name	Start Size [nt]	End Size [nt]	Area	% of total Area
18S	1,478	1,877	102.4	19.5
28S	2,987	4,010	122.8	23.4

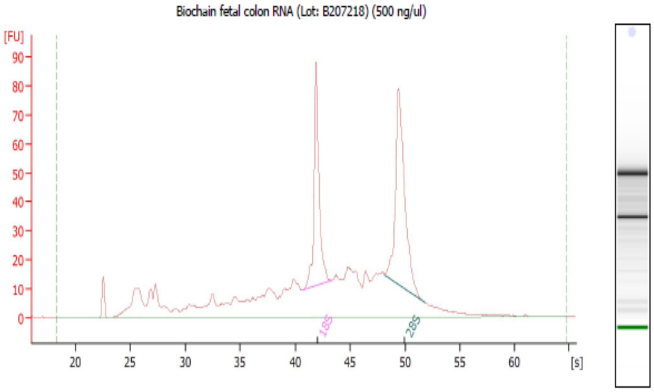

Overall Results for sample 3 : Biochain fetal colon RNA (Lot: B207218) (500 ng/ul)

RNA Area:	856.0	RNA Integrity Number (RIN):	7.4 (B.02.08)
RNA Concentration:	392 ng/µl	Result Flagging Color:	
rRNA Ratio [28s / 18s]:	1.3	Result Flagging Label:	RIN: 7.40

Fragment table for sample 3 : Biochain fetal colon RNA (Lot: B207218) (500 ng/ul)

Name	Start Time [s]	End Time [s]	Area	% of total Area
18S	40.64	43.36	99.7	11.6
28S	48.14	51.84	134.4	15.7

Agilent Bioanalyser profiles of total RNA from 6 different adult tissue types

Adult heart

Agilent Normal Adult heart total RNA (400ng/ul)

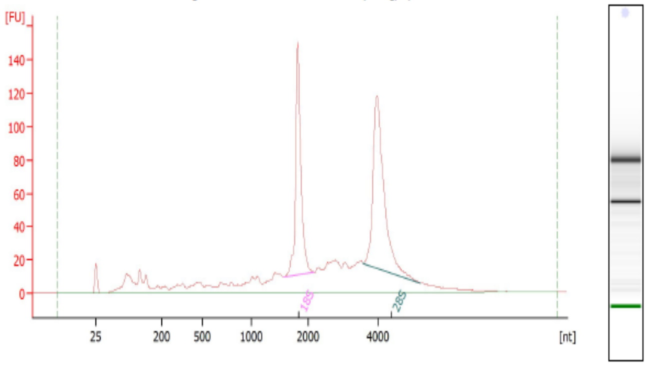

Biochain Normal Adult heart total RNA (400ng/ul)

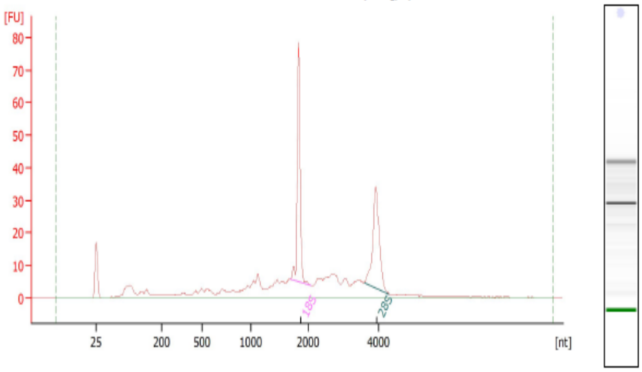

Overall Results for sample 1 : Agilent Normal Adult heart total RNA (400ng/ul)

RNA Area:	1,146.7	RNA Integrity Number (RIN):	8.4 (B.02.08)
RNA Concentration:	516 ng/µl	Result Flagging Color:	
rRNA Ratio [28s / 18s]:	1.3	Result Flagging Label:	RIN: 8.40

Fragment table for sample 1 : Agilent Normal Adult heart total RNA (400ng/ul)

Name	Start Size [nt]	End Size [nt]	Area	% of total Area
18S	1,564	2,163	194.6	17.0
28S	3,570	5,224	255.7	22.3

Overall Results for sample 2 : Biochain Normal Adult heart total RNA (400ng/ul)

RNA Area:	324.0	RNA Integrity Number (RIN):	7.4 (B.02.08)
RNA Concentration:	146 ng/µl	Result Flagging Color:	
rRNA Ratio [28s / 18s]:	0.8	Result Flagging Label:	RIN: 7.40

Fragment table for sample 2 : Biochain Normal Adult heart total RNA (400ng/ul)

Name	Start Size [nt]	End Size [nt]	Area	% of total Area
18S	1,676	2,066	56.9	17.6
28S	3,594	4,314	45.8	14.1

Adult liver

Agilent Normal Adult liver total RNA (400ng/ul)

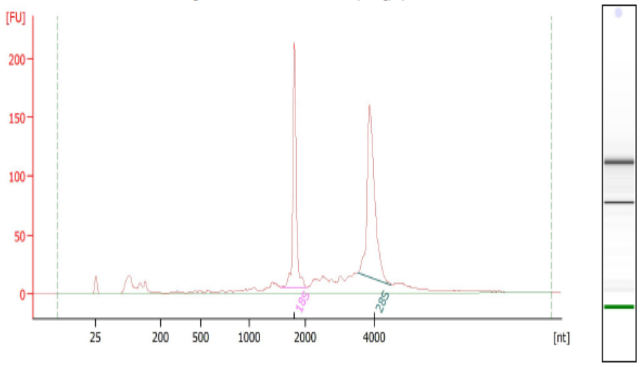

Biochain Normal Adult liver total RNA (400ng/ul)

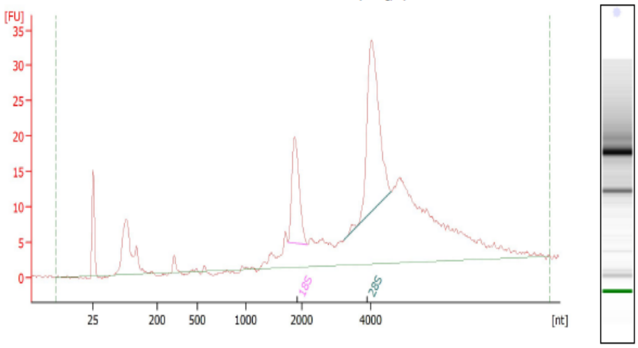

Overall Results for sample 5 : Agilent Normal Adult liver total RNA (400ng/ul)

RNA Area:	896.3	RNA Integrity Number (RIN):	9.3 (B.02.08)
RNA Concentration:	403 ng/µl	Result Flagging Color:	
rRNA Ratio [28s / 18s]:	1.3	Result Flagging Label:	RIN: 9.30

Fragment table for sample 5 : Agilent Normal Adult liver total RNA (400ng/ul)

Name	Start Size [nt]	End Size [nt]	Area	% of total Area
18S	1,555	2,033	188.8	21.1
28S	3,512	4,499	253.8	28.3

Overall Results for sample 6 : Biochain Normal Adult liver total RNA (400ng/ul)

RNA Area:	363.5	RNA Integrity Number (RIN):	8.3 (B.02.08, Anomaly Threshold(s) manually adapted)
RNA Concentration:	164 ng/µl		
rRNA Ratio [28s / 18s]:	2.2		
		Result Flagging Color:	
		Result Flagging Label:	RIN: 8.30

Fragment table for sample 6 : Biochain Normal Adult liver total RNA (400ng/ul)

Name	Start Size [nt]	End Size [nt]	Area	% of total Area
18S	1,740	2,161	29.5	8.1
28S	3,225	4,608	66.0	18.2

Adult colon

Agilent Human Normal Adult Colon (undiluted) total RNA

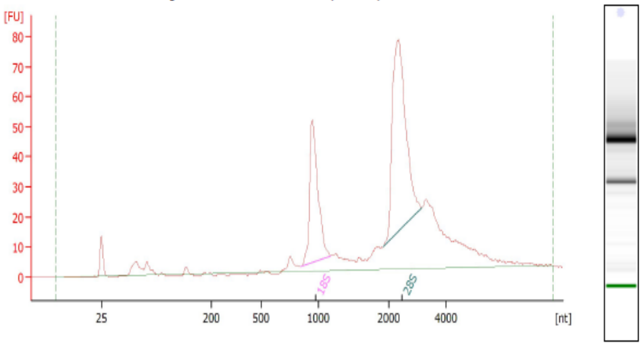

Overall Results for sample 3 : Agilent Human Normal Adult Colon (undiluted) total RNA

RNA Area:	617.9	RNA Integrity Number (RIN):	9.8 (B.02.07, Anomaly Threshold(s) manually adapted)
RNA Concentration:	853 ng/µl		
rRNA Ratio [28s / 18s]:	1.8		
		Result Flagging Color:	
		Result Flagging Label:	RIN: 9.80

Fragment table for sample 3 : Agilent Human Normal Adult Colon (undiluted) total RNA

Name	Start Size [nt]	End Size [nt]	Area	% of total Area
18S	845	1,182	100.4	16.3
28S	1,920	3,168	184.0	29.8

Biochain Human Normal Adult colon (400ng/ul) total RNA

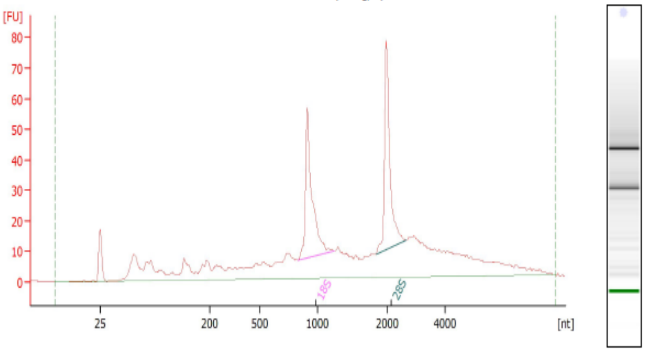

Overall Results for sample 4 : Biochain Human Normal Adult colon (400ng/ul) total RNA

RNA Area:	688.0	RNA Integrity Number (RIN):	7.5 (B.02.07, Anomaly Threshold(s) manually adapted)
RNA Concentration:	950 ng/µl		
rRNA Ratio [28s / 18s]:	1.0		
		Result Flagging Color:	
		Result Flagging Label:	RIN: 7.50

Fragment table for sample 4 : Biochain Human Normal Adult colon (400ng/ul) total RNA

Name	Start Size [nt]	End Size [nt]	Area	% of total Area
18S	830	1,245	83.4	12.1
28S	1,830	2,653	79.9	11.6

Adult lung

Agilent Normal Adult lung total RNA (400ng/ul)

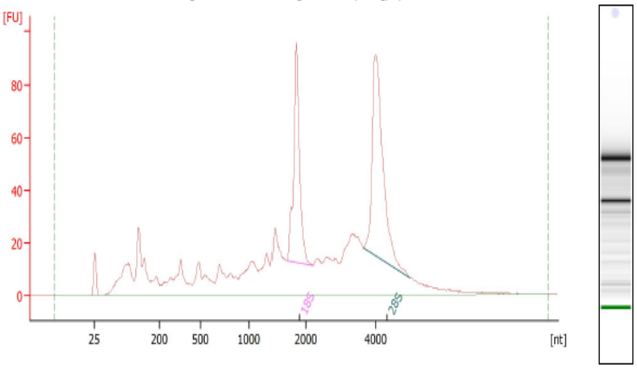

Overall Results for sample 3 : Agilent Normal Adult lung total RNA (400ng/ul)

RNA Area:	1,162.6	RNA Integrity Number (RIN):	7.2 (B.02.08)
RNA Concentration:	523 ng/µl	Result Flagging Color:	
rRNA Ratio [28s / 18s]:	1.4	Result Flagging Label:	RIN: 7.20

Fragment table for sample 3 : Agilent Normal Adult lung total RNA (400ng/ul)

Name	Start Size [nt]	End Size [nt]	Area	% of total Area
18S	1,651	2,197	129.0	11.1
28S	3,649	5,001	184.8	15.9

Biochain Normal Adult lung total RNA (400ng/ul)

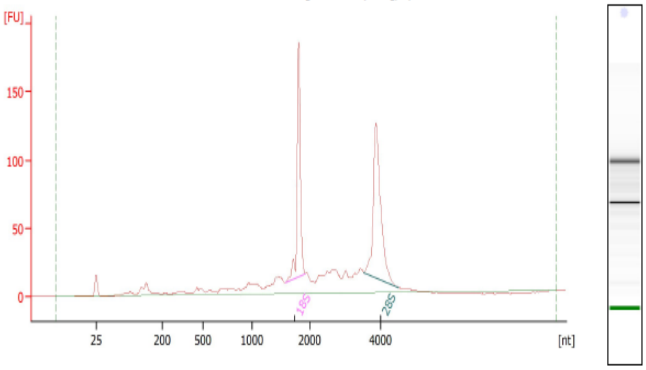

Overall Results for sample 4 : Biochain Normal Adult lung total RNA (400ng/ul)

RNA Area:	797.1	RNA Integrity Number (RIN):	8.2 (B.02.08)
RNA Concentration:	359 ng/µl	Result Flagging Color:	
rRNA Ratio [28s / 18s]:	1.3	Result Flagging Label:	RIN: 8.20

Fragment table for sample 4 : Biochain Normal Adult lung total RNA (400ng/ul)

Name	Start Size [nt]	End Size [nt]	Area	% of total Area
18S	1,560	1,916	144.8	18.2
28S	3,536	4,516	190.8	23.9

Adult kidney

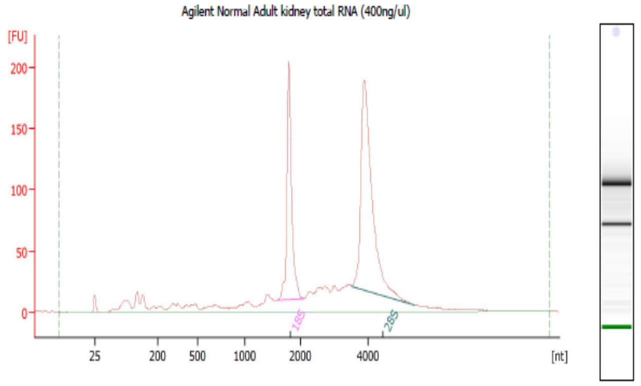

Overall Results for sample 7 : Agilent Normal Adult kidney total RNA (400ng/ul)

RNA Area:	1,423.3	RNA Integrity Number (RIN):	9 (B.02.08)
RNA Concentration:	641 ng/µl	Result Flagging Color:	
rRNA Ratio [28s / 18s]:	1.8	Result Flagging Label:	RIN:9

Fragment table for sample 7 : Agilent Normal Adult kidney total RNA (400ng/ul)

Name	Start Size [nt]	End Size [nt]	Area	% of total Area
18S	1,563	2,099	248.2	17.4
28S	3,507	5,369	445.3	31.3

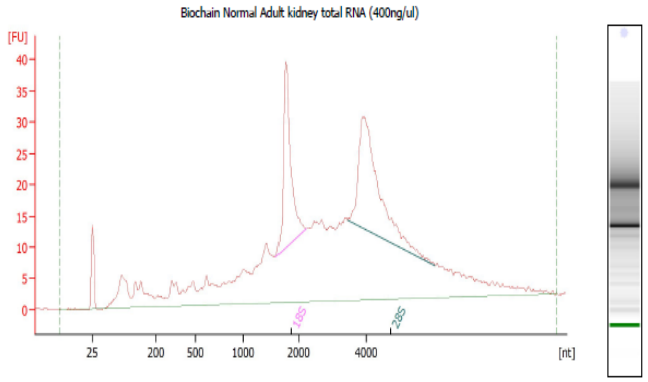

Overall Results for sample 9 : Biochain Normal Adult kidney total RNA (400ng/ul)

RNA Area:	686.0	RNA Integrity Number (RIN):	7.1 (B.02.08)
RNA Concentration:	309 ng/µl	Result Flagging Color:	
rRNA Ratio [28s / 18s]:	1.4	Result Flagging Label:	RIN: 7.10

Fragment table for sample 9 : Biochain Normal Adult kidney total RNA (400ng/ul)

Name	Start Size [nt]	End Size [nt]	Area	% of total Area
18S	1,580	2,225	57.9	8.4
28S	3,443	6,028	83.5	12.2

Adult stomach

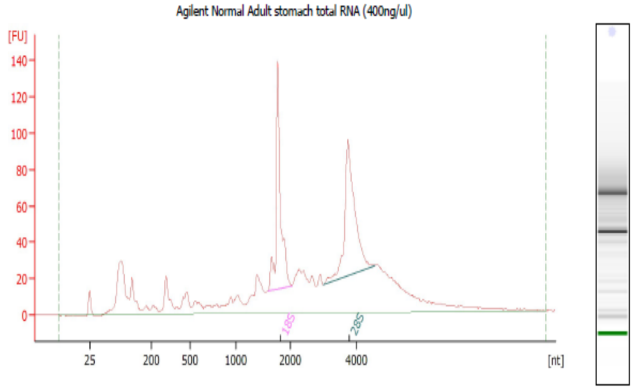

Overall Results for sample 10 : Agilent Normal Adult stomach total RNA (400ng/ul)

RNA Area:	1,361.9	RNA Integrity Number (RIN):	7.5 (B.02.07)
RNA Concentration:	613 ng/µl	Result Flagging Color:	
rRNA Ratio [28s / 18s]:	1.1	Result Flagging Label:	RIN: 7.50

Fragment table for sample 10 : Agilent Normal Adult stomach total RNA (400ng/ul)

Name	Start Size [nt]	End Size [nt]	Area	% of total Area
18S	1,602	2,043	156.2	11.5
28S	3,000	4,570	178.0	13.1

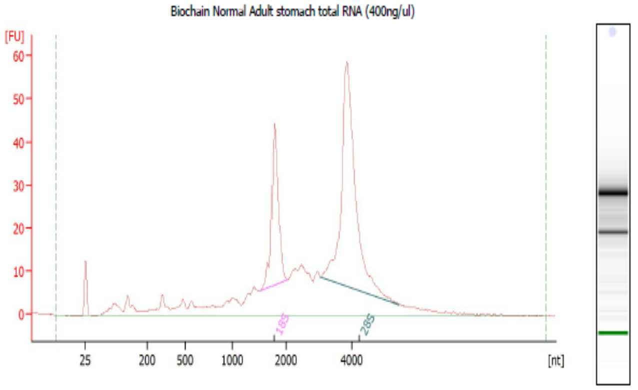

Overall Results for sample 11 : Biochain Normal Adult stomach total RNA (400ng/ul)

RNA Area:	584.5	RNA Integrity Number (RIN):	8.7 (B.02.08)
RNA Concentration:	263 ng/µl	Result Flagging Color:	
rRNA Ratio [28s / 18s]:	2.5	Result Flagging Label:	RIN: 8.70

Fragment table for sample 11 : Biochain Normal Adult stomach total RNA (400ng/ul)

Name	Start Size [nt]	End Size [nt]	Area	% of total Area
18S	1,522	2,053	68.0	11.6
28S	3,032	5,425	172.0	29.4

Gene ethics Normal Adult Stomach total RNA (R561840) (400ng/ul)

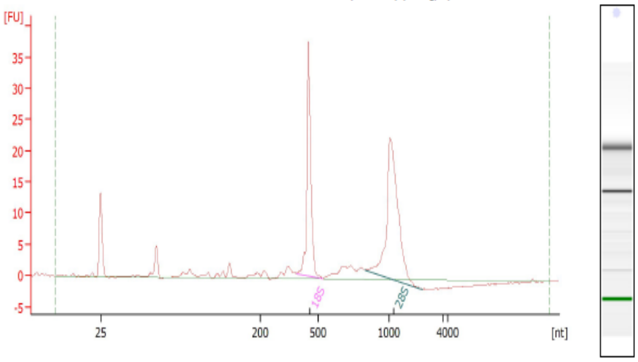

Overall Results for sample 4 : **Gene ethics Normal Adult Stomach total RNA (R561840) (400ng/ul)**

RNA Area:	138.8	RNA Integrity Number (RIN):	8.8 (B.02.07)
RNA Concentration:	219 ng/µl	Result Flagging Color:	
rRNA Ratio [28s / 18s]:	1.5	Result Flagging Label:	RIN: 8.80

Fragment table for sample 4 : **Gene ethics Normal Adult Stomach total RNA (R561840) (400ng/ul)**

Name	Start Size [nt]	End Size [nt]	Area	% of total Area
18S	385	534	37.9	27.3
28S	829	1,616	56.6	40.8

Gene ethics Normal Adult Stomach total RNA (R560288) (400ng/ul)

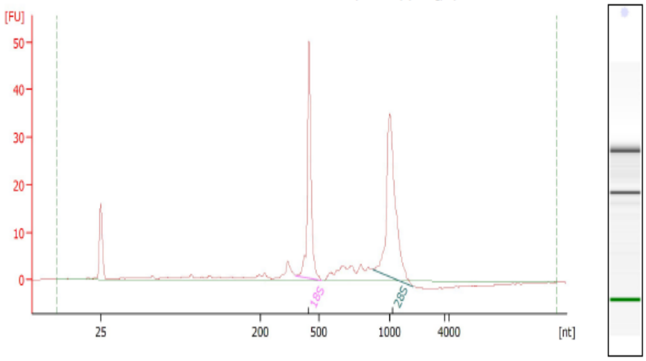

Overall Results for sample 5 : **Gene ethics Normal Adult Stomach total RNA (R560288) (400ng/ul)**

RNA Area:	186.2	RNA Integrity Number (RIN):	8.6 (B.02.07)
RNA Concentration:	293 ng/µl	Result Flagging Color:	
rRNA Ratio [28s / 18s]:	1.4	Result Flagging Label:	RIN: 8.60

Fragment table for sample 5 : **Gene ethics Normal Adult Stomach total RNA (R560288) (400ng/ul)**

Name	Start Size [nt]	End Size [nt]	Area	% of total Area
18S	378	522	48.8	26.2
28S	880	1,436	66.0	35.5

Origene Normal Adult stomach CR560393 total RNA (400ng/ul)

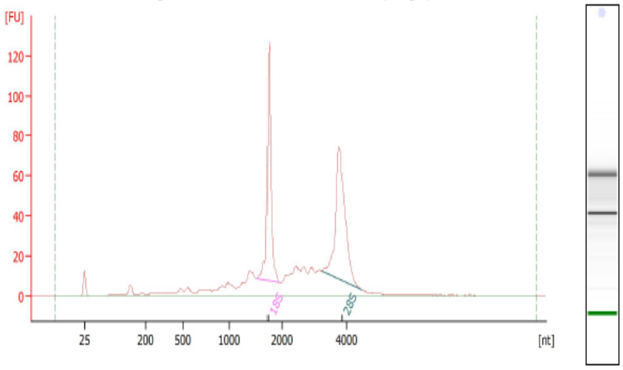

Overall Results for sample 12 : **Origene Normal Adult stomach CR560393 total RNA (400ng/ul)**

RNA Area:	647.8	RNA Integrity Number (RIN):	8.3 (B.02.08)
RNA Concentration:	292 ng/µl	Result Flagging Color:	
rRNA Ratio [28s / 18s]:	1.3	Result Flagging Label:	RIN: 8.30

Fragment table for sample 12 : **Origene Normal Adult stomach CR560393 total RNA (400ng/ul)**

Name	Start Size [nt]	End Size [nt]	Area	% of total Area
18S	1,533	1,950	121.5	18.8
28S	3,226	4,458	153.9	23.8

Figures above: Agilent Bioanalyser 2100 Eukaryote Total RNA Nano Series II profiles of fetal total RNA from stomach and colon as well as adult total RNA from heart, liver, colon, lung, kidney and stomach
