# Supplementary material for: A resource of ribosomal RNA-depleted RNA-Seq data from different normal adult and fetal human tissues
Source: Sci Data. 2015 Nov 10;2:150063. doi: 10.1038/sdata.2015.63 (PMC4640133; doi:10.1038/sdata.2015.63)
Supplement: Supplementary File 2 [file sdata201563-s3.pdf]

Agilent Bioanalyser profiles of rRNA- depleted RNA-Seq libraries from 2 different fetal tissue types

Fetal stomach

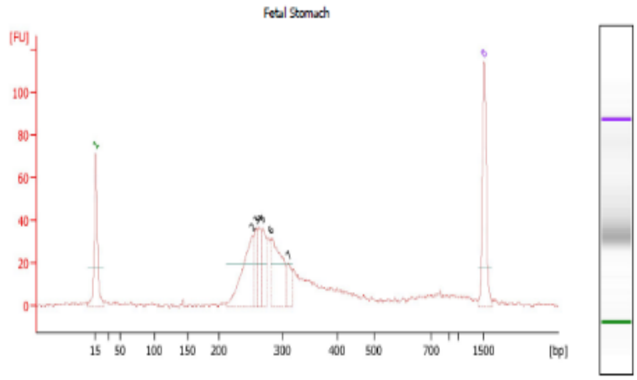

Overall Results for sample 2 : Fetal Stomach

Peak table for sample 2 : Fetal Stomach

Peak		Size [bp]	Conc. [ng/µl]	Molarity [nmol/l]	Observations
1	◀	15	4.20	424.2	Lower Marker
2		253	2.80	16.8	Number of peaks fou
3		258	0.79	4.7	
4		264	0.94	5.4	
5		269	1.24	7.0	
6		282	2.42	13.0	
7		310	0.74	3.6	
8	▶	1,500	2.10	2.1	Upper Marker

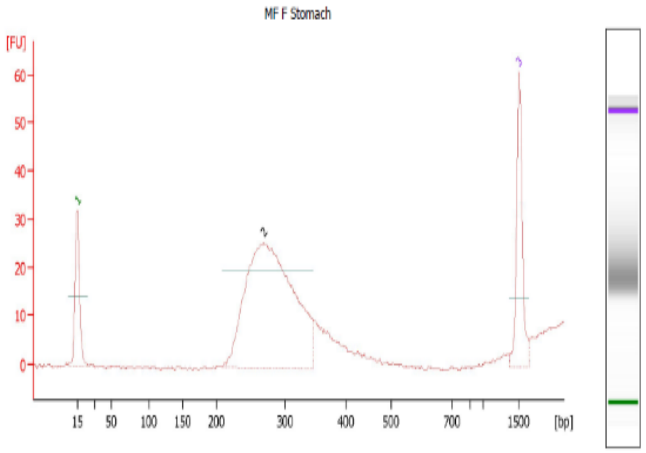

Overall Results for sample 12 : MF F Stomach

Peak table for sample 12 : MF F Stomach

Peak		Size [bp]	Conc. [ng/µl]	Molarity [nmol/l]	Observations
1	◀	15	4.20	424.2	Lower Marker
2		269	12.27	69.1	Number of peaks fou
3	▶	1,500	2.10	2.1	Upper Marker

Fetal colon

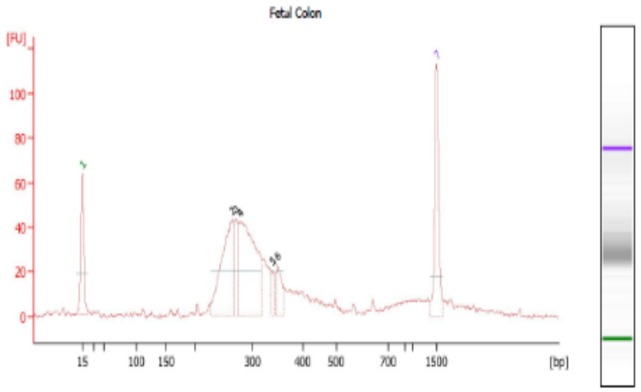

Overall Results for sample 8 : Fetal Colon

Peak table for sample 8 : Fetal Colon

Peak		Size [bp]	Conc. [ng/µl]	Molarity [nmol/l]	Observations
1	◀	15	4.20	424.2	Lower Marker
2		266	3.90	22.3	Number of peaks fou
3		272	1.17	6.5	
4		280	5.33	28.8	
5		341	0.32	1.4	
6		350	0.82	3.6	
7	▶	1,500	2.10	2.1	Upper Marker

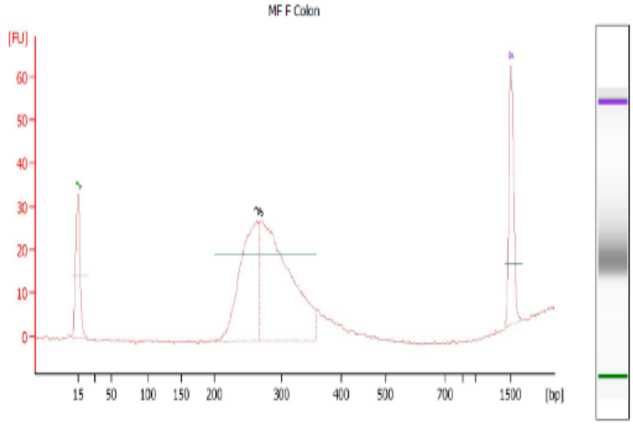

Overall Results for sample 11 : MF F Colon

Peak table for sample 11 : MF F Colon

Peak		Size [bp]	Conc. [ng/µl]	Molarity [nmol/l]	Observations
1	◀	15	4.20	424.2	Lower Marker
2		264	6.27	36.1	Number of peaks fou
3		269	10.01	56.5	
4	▶	1,500	2.10	2.1	Upper Marker

Agilent Bioanalyser profiles of of rRNA- depleted RNA-Seq libraries from 6 different adult tissue types

Adult heart

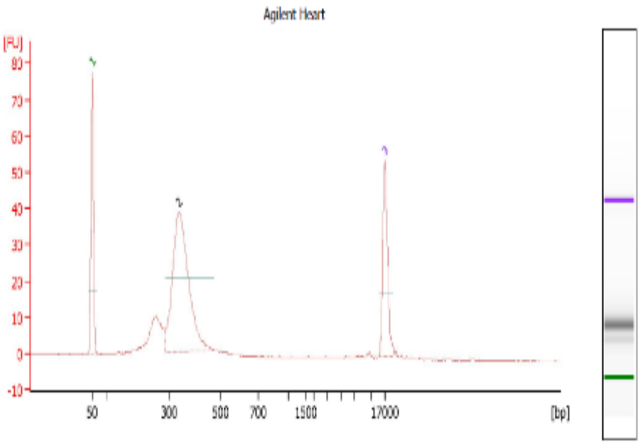

Overall Results for sample 1 : Agilent Heart

Peak table for sample 1 : Agilent Heart

Peak	Size [bp]	Conc. [ng/µl]	Molarity [nmol/l]	Observations
1	50	8.30	251.5	Lower Marker
2	337	14.88	66.8	Number of peaks fou
3	17,000	4.20	0.4	Upper Marker

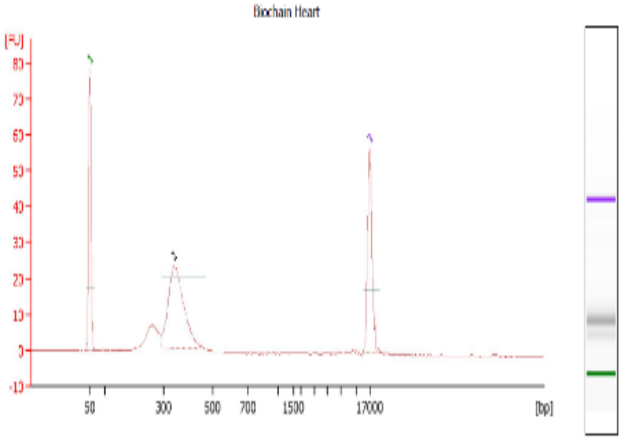

Overall Results for sample 2 : Biochain Heart

Peak table for sample 2 : Biochain Heart

Peak	Size [bp]	Conc. [ng/µl]	Molarity [nmol/l]	Observations
1	50	8.30	251.5	Lower Marker
2	343	8.48	37.4	Number of peaks fou
3	17,000	4.20	0.4	Upper Marker

Adult liver

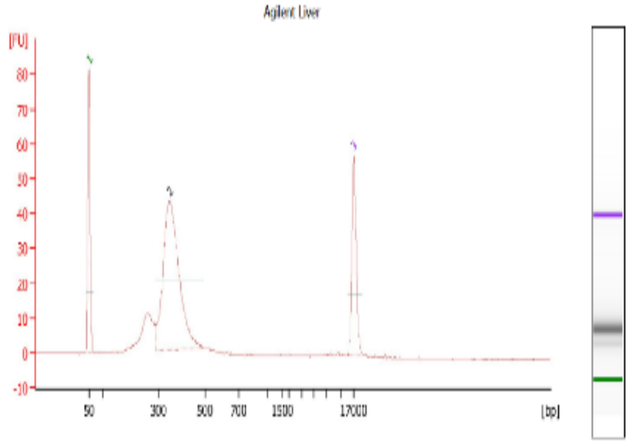

Overall Results for sample 5 : Agilent Liver

Peak table for sample 5 : Agilent Liver

Peak	Size [bp]	Conc. [ng/µl]	Molarity [nmol/l]	Observations
1	50	8.30	251.5	Lower Marker
2	346	17.08	74.8	Number of peaks fou
3	17,000	4.20	0.4	Upper Marker

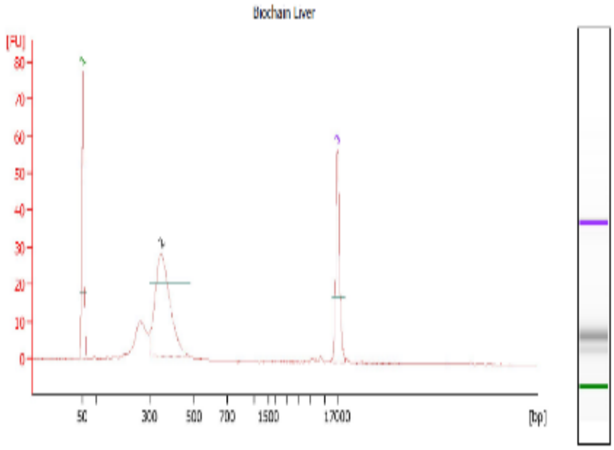

Overall Results for sample 6 : Biochain Liver

Peak table for sample 6 : Biochain Liver

Peak	Size [bp]	Conc. [ng/µl]	Molarity [nmol/l]	Observations
1	50	8.30	251.5	Lower Marker
2	349	11.22	48.6	Number of peaks fou
3	17,000	4.20	0.4	Upper Marker

Adult colon

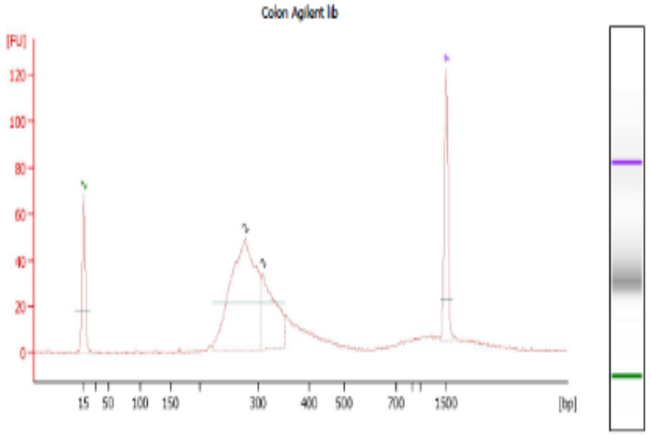

Overall Results for sample 1 : Colon Agilent lib

Peak table for sample 1 : Colon Agilent lib

Peak	Size [bp]	Conc. [ng/µl]	Molarity [nmol/l]	Observations
1	15	4.20	424.2	Lower Marker
2	278	9.89	53.9	
3	310	3.28	16.0	Number of peaks fou
4	1,500	2.10	2.1	Upper Marker

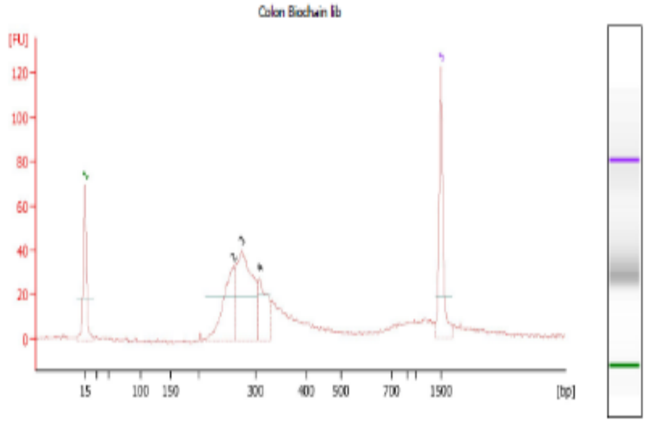

Overall Results for sample 2 : Colon Biochain lib

Peak table for sample 2 : Colon Biochain lib

Peak	Size [bp]	Conc. [ng/µl]	Molarity [nmol/l]	Observations
1	15	4.20	424.2	Lower Marker
2	261	2.60	15.1	Number of peaks fou
3	275	4.37	24.1	
4	307	1.67	8.2	
5	1,500	2.10	2.1	Upper Marker

Adult lung

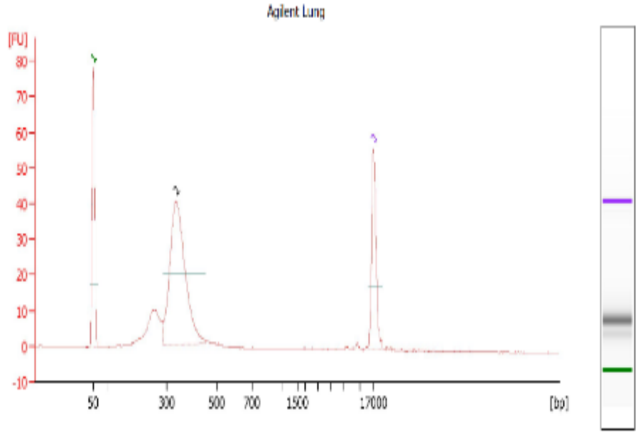

Overall Results for sample 3 : Agilent Lung

Peak table for sample 3 : Agilent Lung

Peak	Size [bp]	Conc. [ng/µl]	Molarity [nmol/l]	Observations
1	50	8.30	251.5	Lower Marker
2	337	15.50	69.7	Number of peaks fou
3	17,000	4.20	0.4	Upper Marker

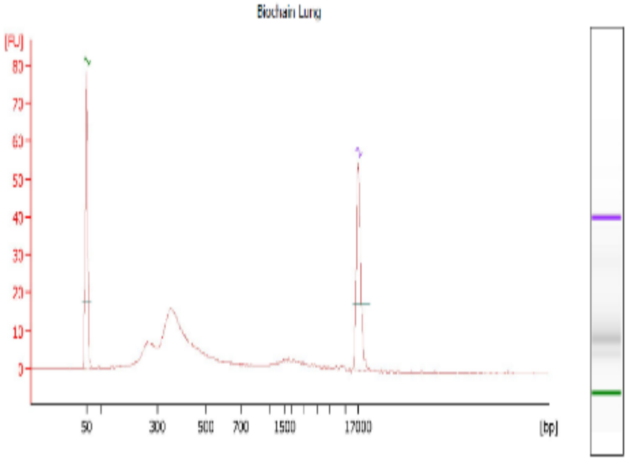

Overall Results for sample 4 : Biochain Lung

Peak table for sample 4 : Biochain Lung

Peak	Size [bp]	Conc. [ng/µl]	Molarity [nmol/l]	Observations
1	50	8.30	251.5	Lower Marker
2	17,000	4.20	0.4	Upper Marker Number of peaks fou

Adult kidney

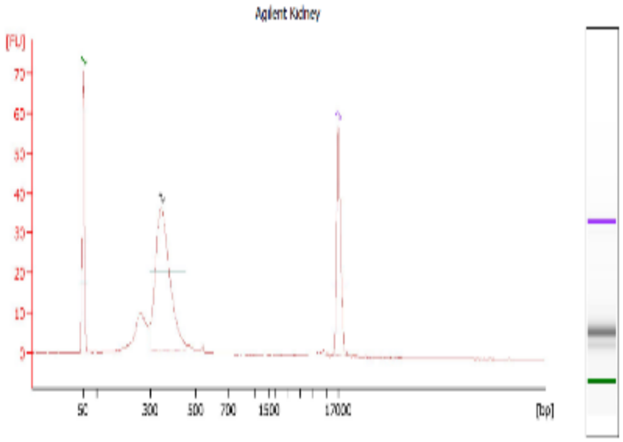

Overall Results for sample 7 : Agilent Kidney

Peak table for sample 7 : Agilent Kidney

Peak	Size [bp]	Conc. [ng/µl]	Molarity [nmol/l]	Observations
1	50	8.30	251.5	Lower Marker
2	350	14.55	63.1	Number of peaks fou
3	17,000	4.20	0.4	Upper Marker

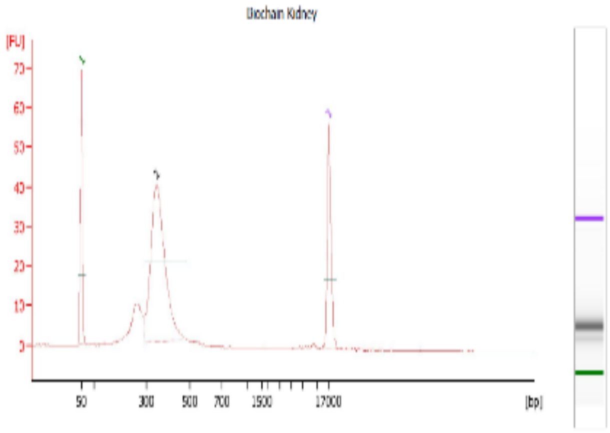

Overall Results for sample 8 : Biochain Kidney

Peak table for sample 8 : Biochain Kidney

Peak	Size [bp]	Conc. [ng/µl]	Molarity [nmol/l]	Observations
1	50	8.30	251.5	Lower Marker
2	347	16.54	72.1	Number of peaks fou
3	17,000	4.20	0.4	Upper Marker

Adult stomach

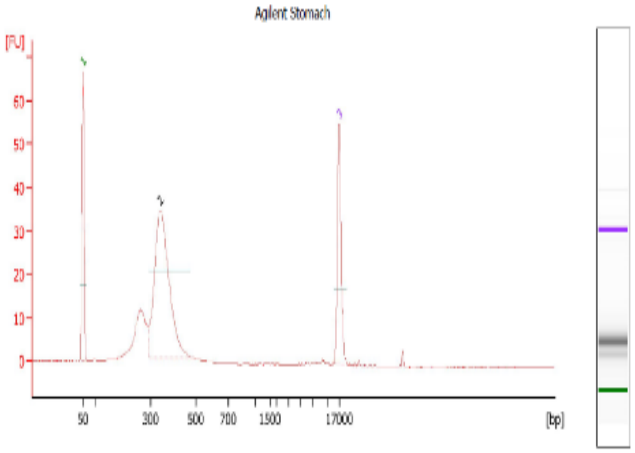

Overall Results for sample 9 : Agilent Stomach

Peak table for sample 9 : Agilent Stomach

Peak	Size [bp]	Conc. [ng/µl]	Molarity [nmol/l]	Observations
1	50	8.30	251.5	Lower Marker
2	343	14.62	64.5	Number of peaks fou
3	17,000	4.20	0.4	Upper Marker

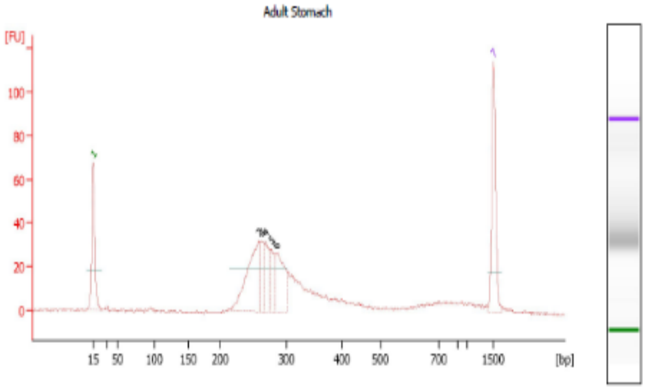

Overall Results for sample 1 : Adult Stomach

Peak table for sample 1 : Adult Stomach

Peak	Size [bp]	Conc. [ng/µl]	Molarity [nmol/l]	Observations
1	15	4.20	424.2	Lower Marker
2	259	3.07	18.0	Number of peaks fou
3	264	0.83	4.8	
4	270	1.10	6.2	
5	278	0.84	4.5	
6	285	1.58	8.4	
7	1,500	2.10	2.1	Upper Marker

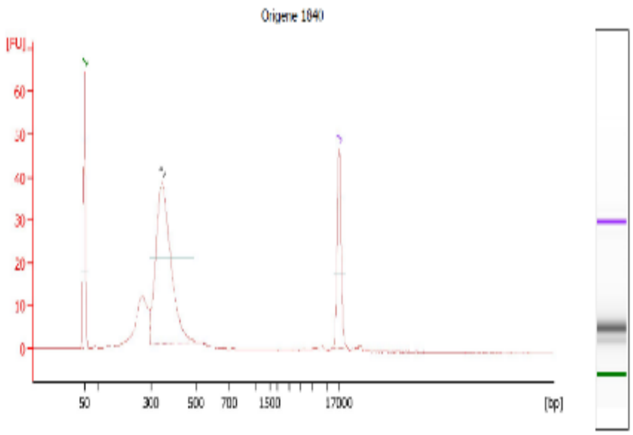

Overall Results for sample 10 : **Origene 1840**

Peak table for sample 10 : **Origene 1840**

Peak		Size [bp]	Conc. [ng/µl]	Molarity [nmol/l]		Observations
1	◀	50	8.30	251.5		Lower Marker
2		347	18.34	80.2	Number of peaks fou	
3	▶	17,000	4.20	0.4		Upper Marker

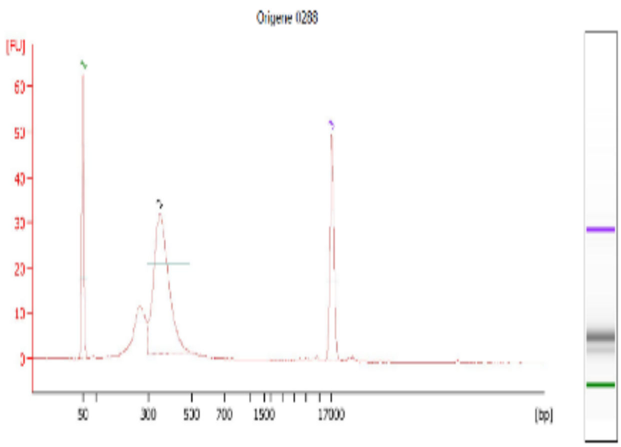

Overall Results for sample 11 : **Origene 0288**

Peak table for sample 11 : **Origene 0288**

Peak		Size [bp]	Conc. [ng/µl]	Molarity [nmol/l]		Observations
1	◀	50	8.30	251.5		Lower Marker
2		352	14.52	62.6	Number of peaks fou	
3	▶	17,000	4.20	0.4		Upper Marker

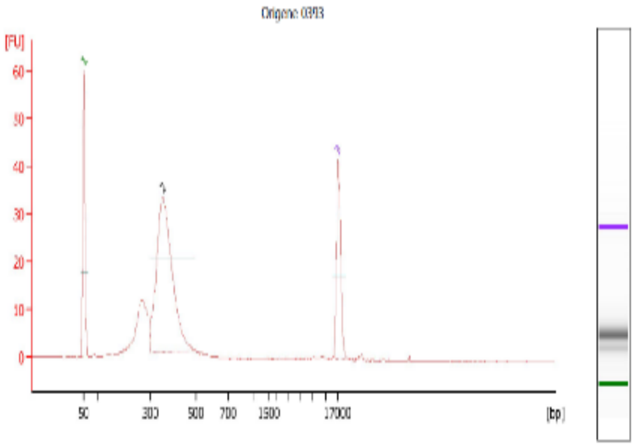

Overall Results for sample 12 : **Origene 0393**

Peak table for sample 12 : **Origene 0393**

Peak		Size [bp]	Conc. [ng/µl]	Molarity [nmol/l]		Observations
1	◀	50	8.30	251.5		Lower Marker
2		353	17.38	74.6	Number of peaks fou	
3	▶	17,000	4.20	0.4		Upper Marker

Figures above: Agilent Bioanalyser 2100 Eukaryote Total RNA Nano Series II profiles of the rRNA- depleted RNA-Seq libraries constructed from fetal and adult tissues.

Ideal Agilent Bioanalyser profile of final RNA-Seq library

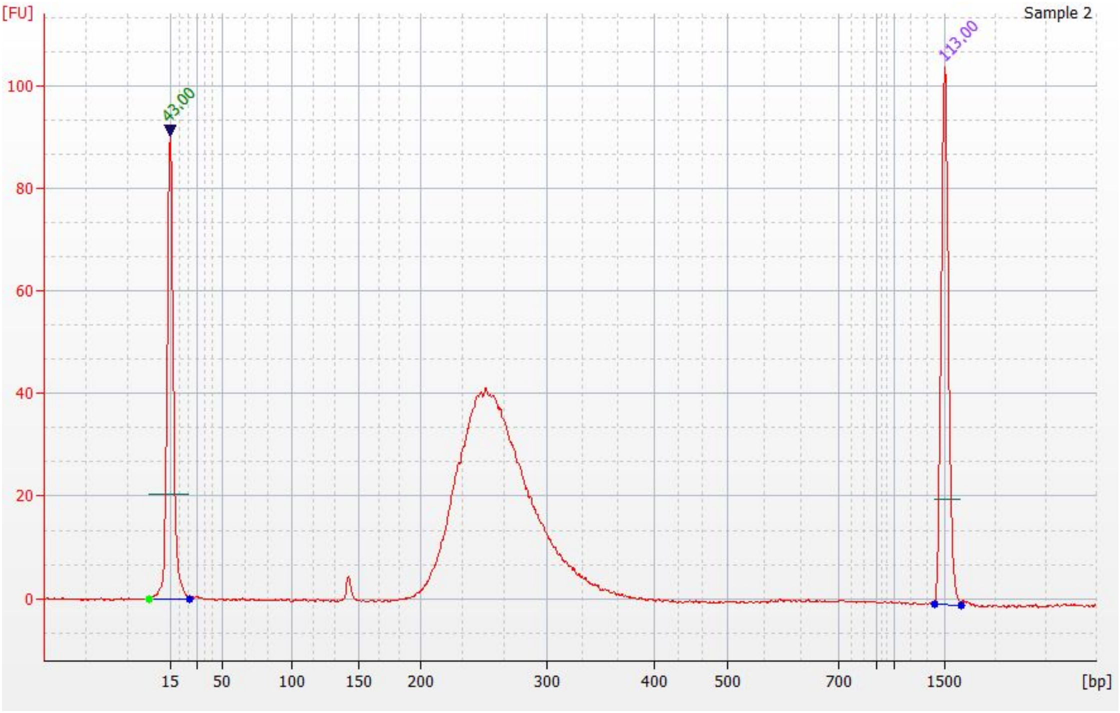

Figure above: Profile of the ideal rRNA- depleted RNA-Seq library. The ideal library would have a size at approximately 260bp.
