# Supplementary material for: A resource of ribosomal RNA-depleted RNA-Seq data from different normal adult and fetal human tissues
Source: Sci Data. 2015 Nov 10;2:150063. doi: 10.1038/sdata.2015.63 (PMC4640133; doi:10.1038/sdata.2015.63)
Supplement: Supplementary File 3 [file sdata201563-s4.pdf]

# FastQC Report

Mon 4 May 2015
Agilent_Adult_Colon_CGATGT_L007_R1.fastq.gz

## Summary


- Basic Statistics
- Per base sequence quality
- Per tile sequence quality
- Per sequence quality scores
- Per base sequence content
- Per sequence GC content
- Per base N content
- Sequence Length Distribution
- Sequence Duplication Levels
- Overrepresented sequences
- Adapter Content
- Kmer Content


## Basic Statistics

| Measure | Value |
|---|---|
| Filename | Agilent_Adult_Colon_CGATGT_L007_R1.fastq.gz |
| File type | Conventional base calls |
| Encoding | Sanger / Illumina 1.9 |
| Total Sequences | 71105369 |
| Sequences flagged as poor quality | 0 |
| Sequence length | 76 |
| %GC | 51 |

## Per base sequence quality

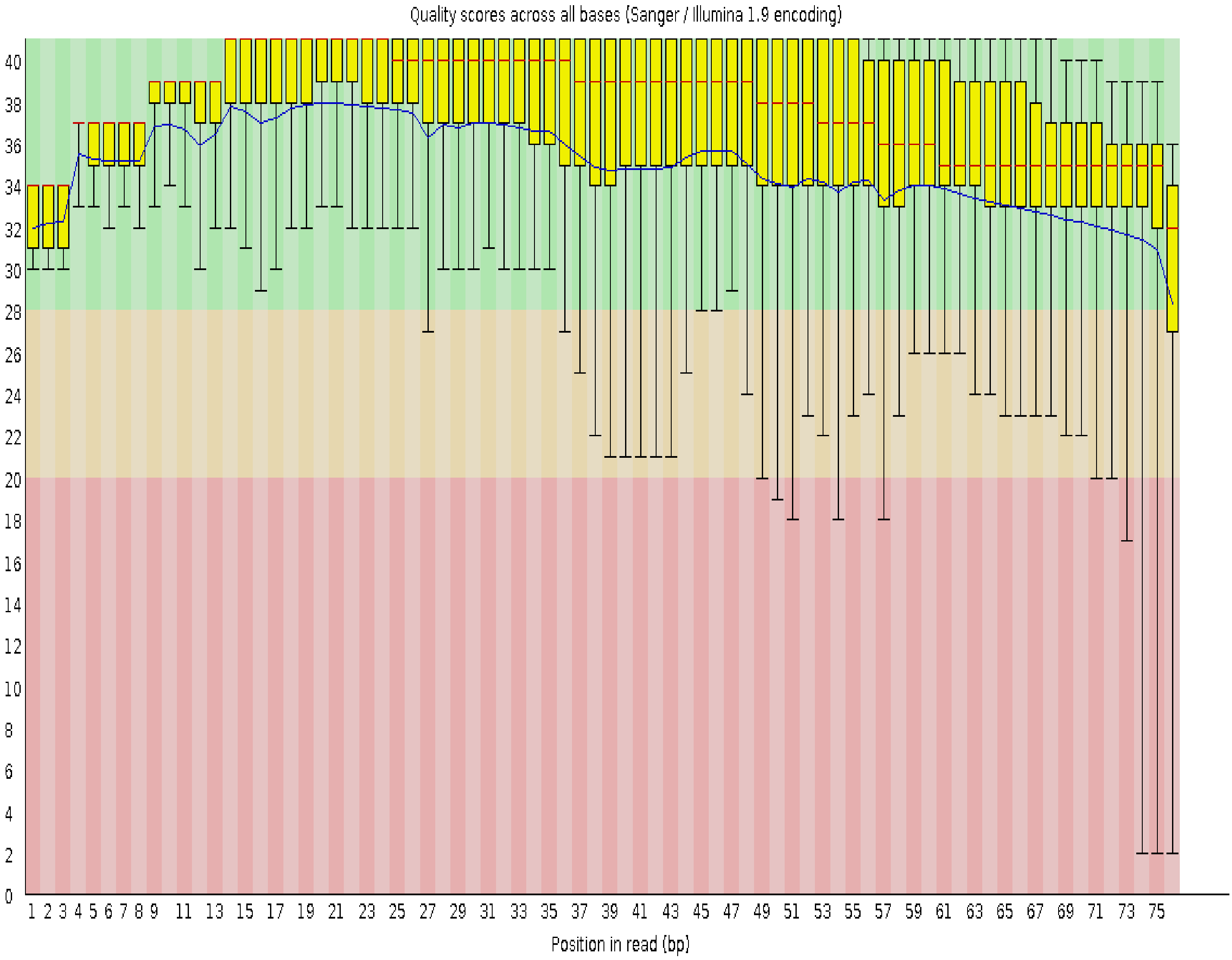


## Per tile sequence quality

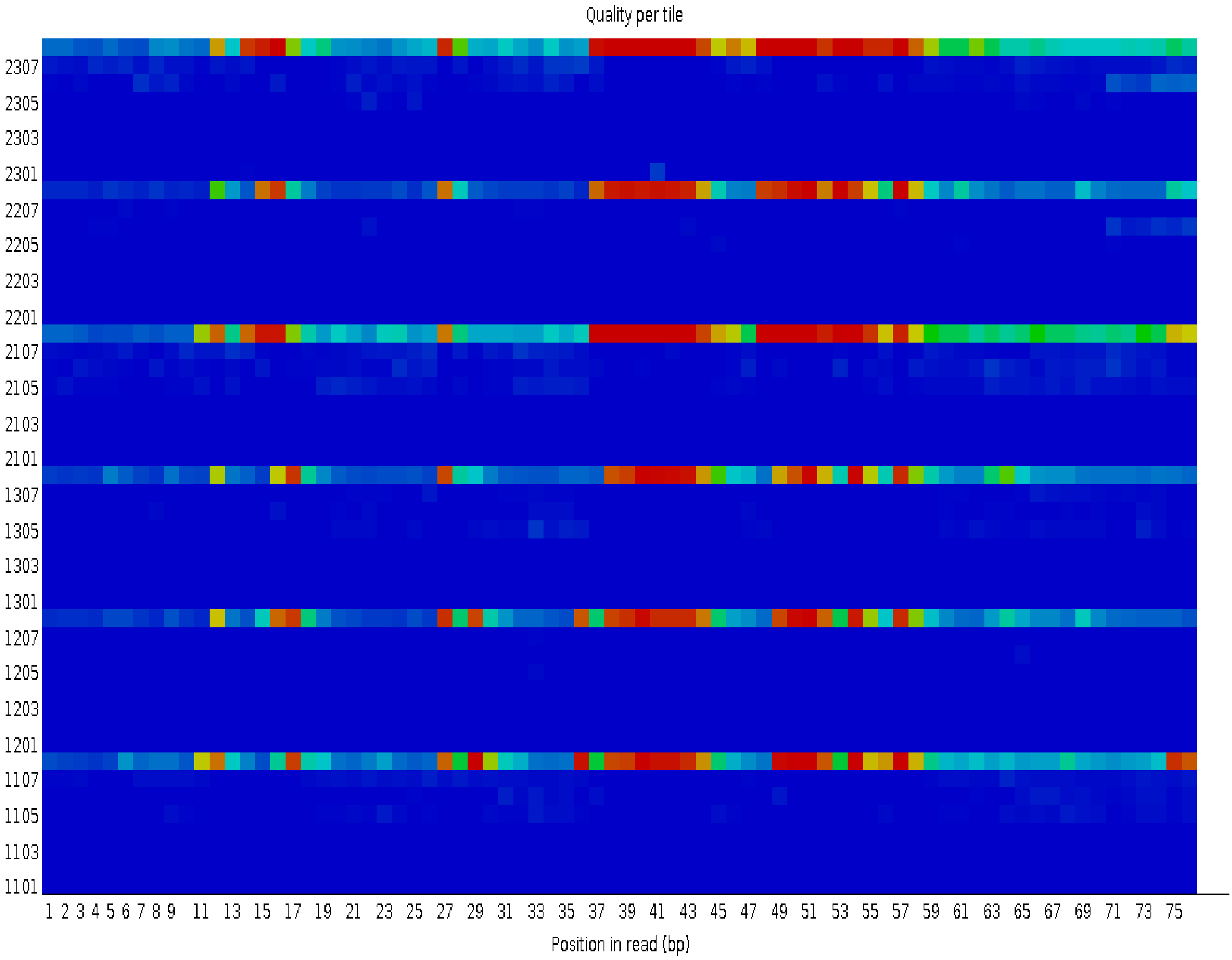


## Per sequence quality scores

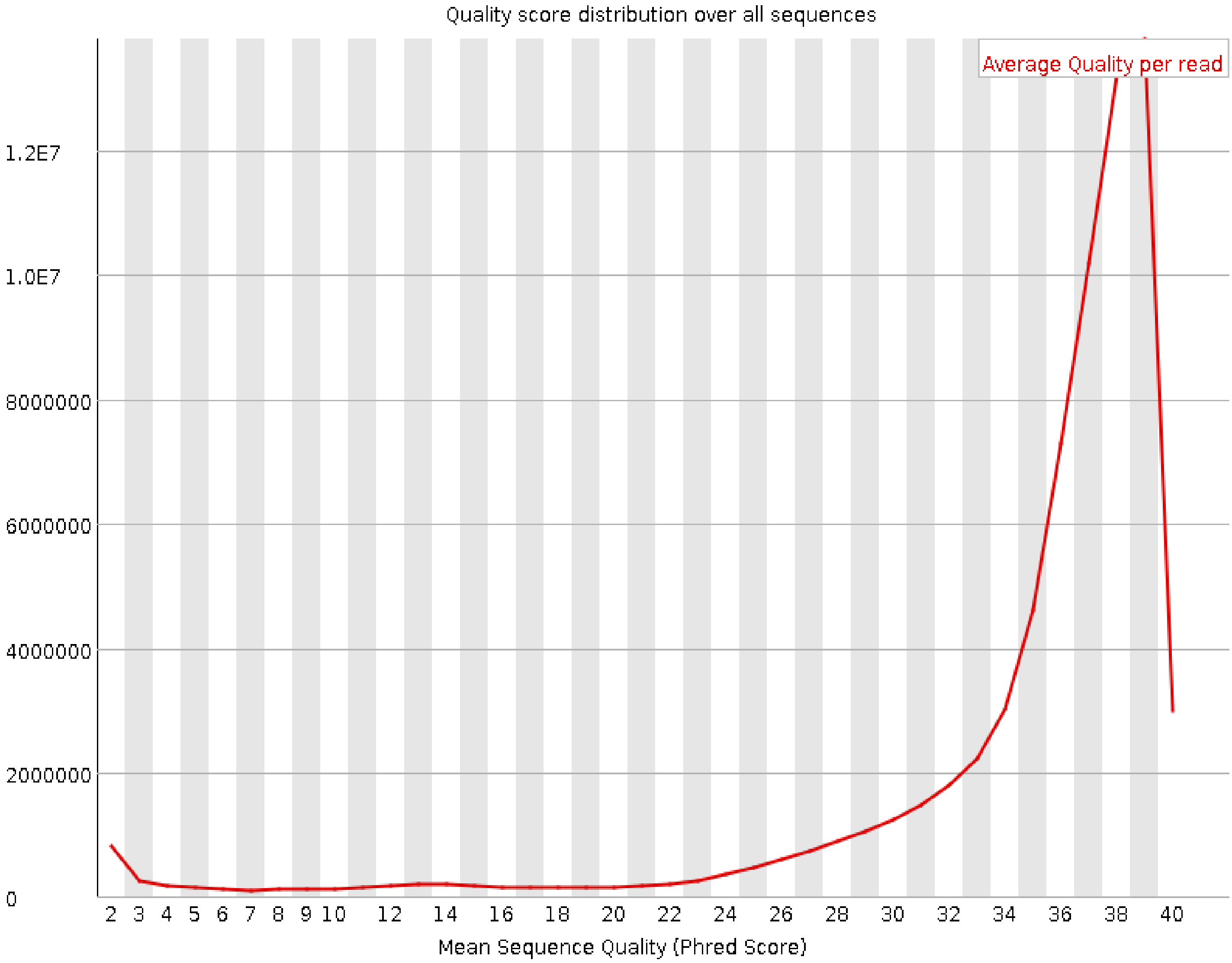


## Per base sequence content

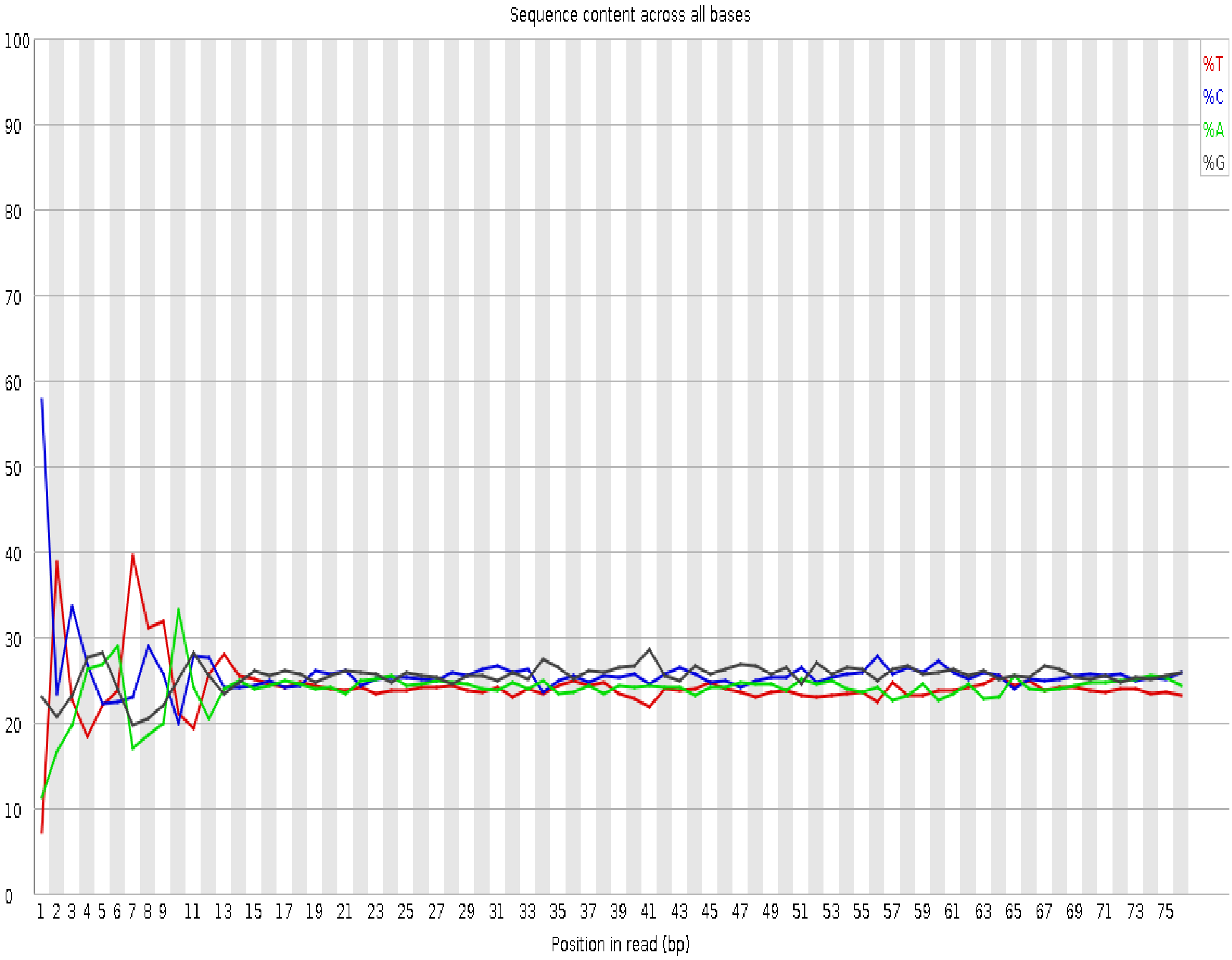


## Per sequence GC content

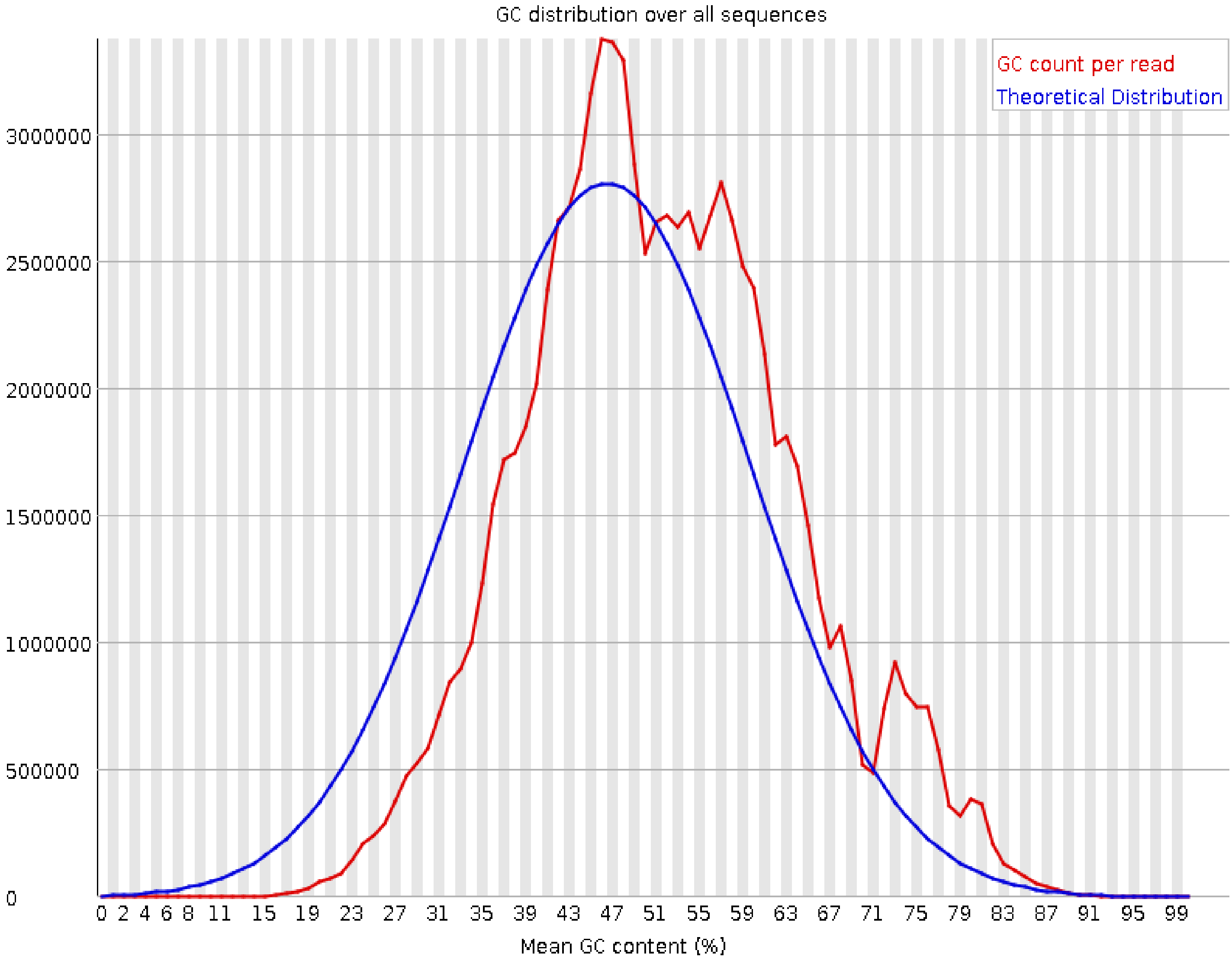


## Per base N content

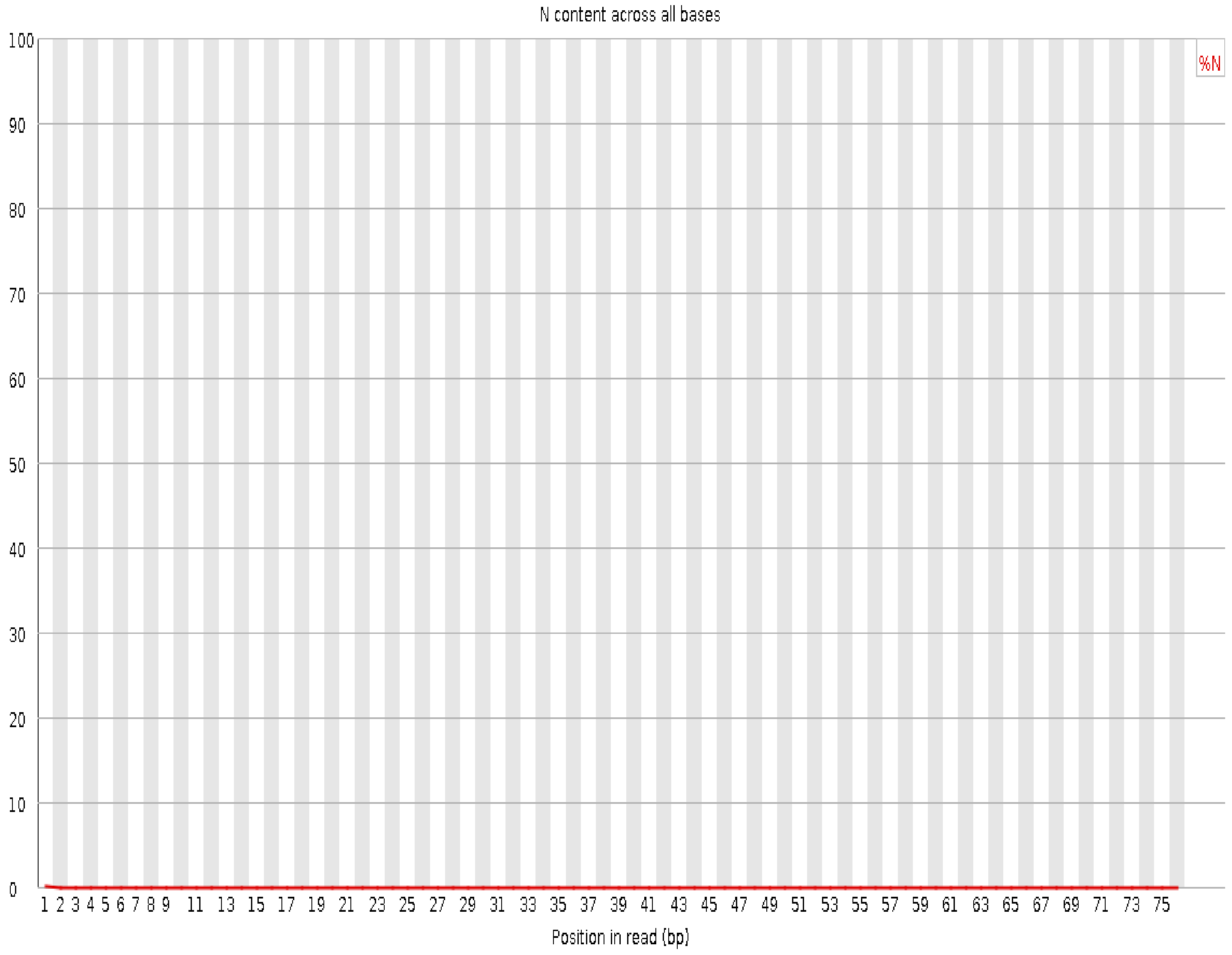


## Sequence Length Distribution

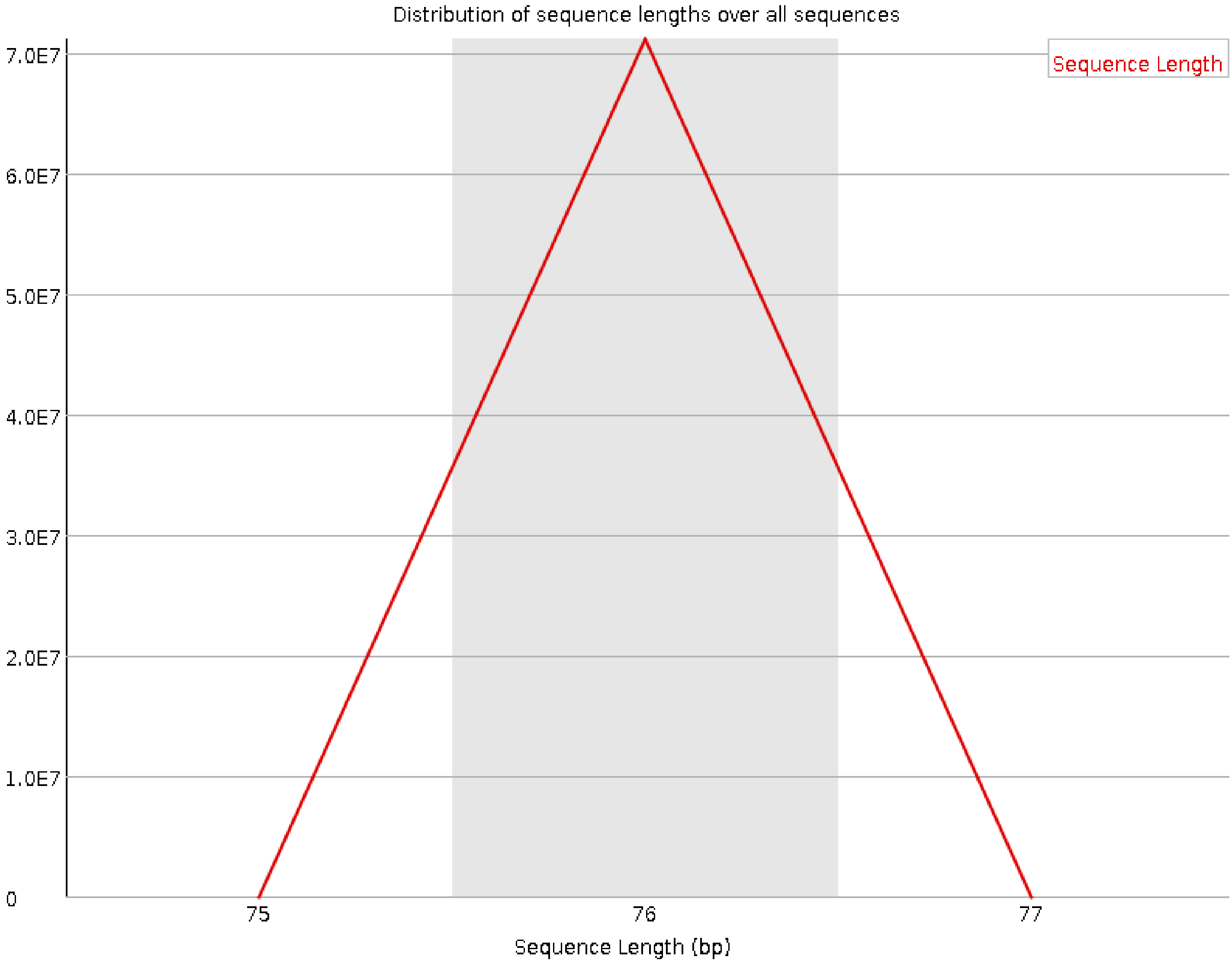


## Sequence Duplication Levels

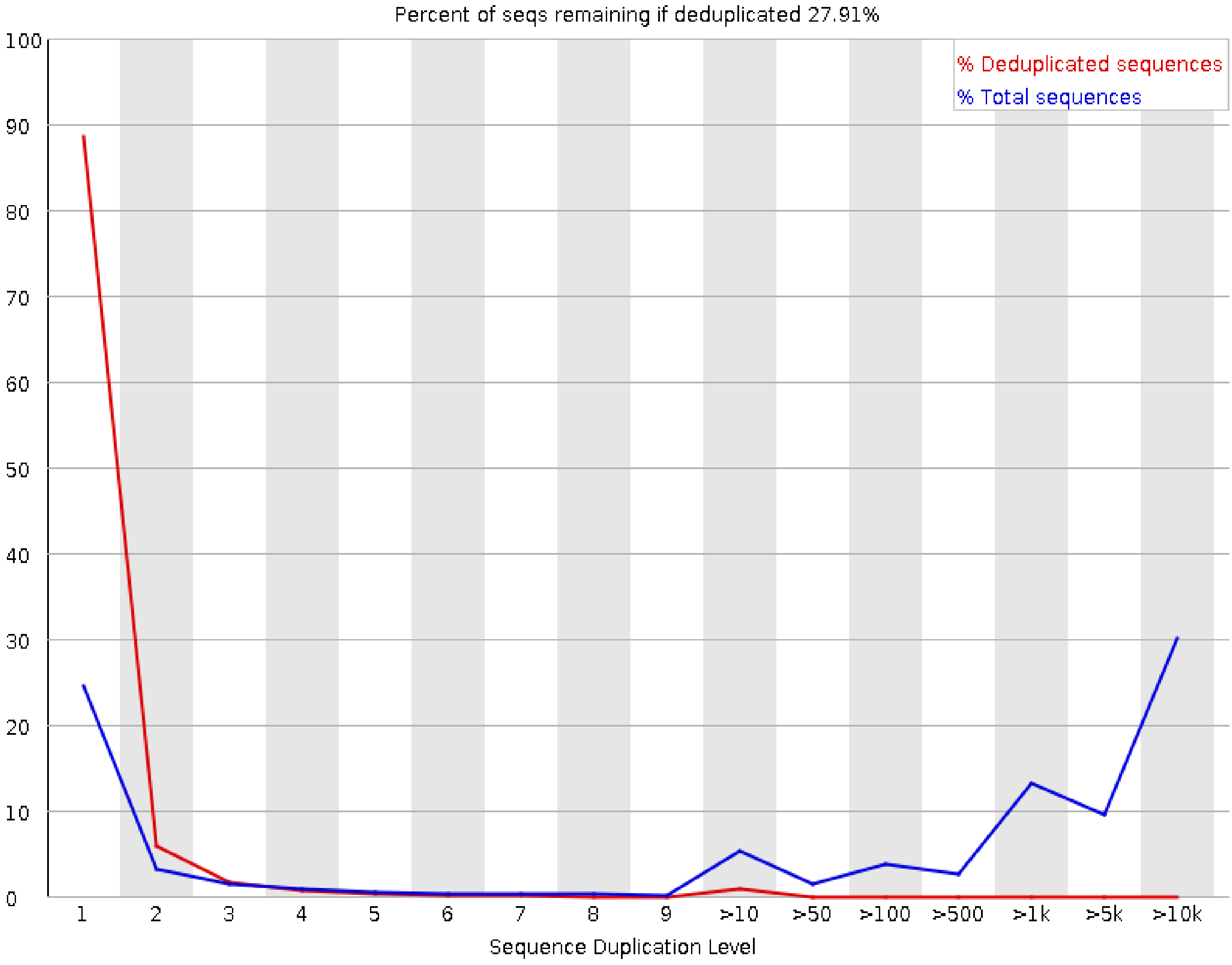


## Overrepresented sequences

| Sequence | Count | Percentage | Possible Source |
|---|---|---|---|
| CCCGTCGGCATGTATTAGCTCTAGAATTACCACAGTTATCCAAGTAGGAG | 312887 | 0.44003287571716276 | No Hit |
| CTTCCGTACGCCACATGTCCCGCGCCCCGCCGCGGGGCGGGGATTCGGCG | 256088 | 0.360152831778427З | No Hit |
| CTGCCAGTAGCATATGCTTGTCTCAAAGATTAAGCCATGCATGTCTAAGT | 223617 | 0.31448680056776024 | No Hit |
| CGCAGTTTTATCCGGTAAAGCGAATGATTAGAGGTCTTGGGGCCGAAACG | 200926 | 0.2825750049901295 | No Hit |
| CTCTCTTCAAAGTTCTTTTCAACTTTCCCTTACGGTACTTGTTGACTATC | 196276 | 0.276035414428409‌8 | No Hit |
| CCGGTATTTAGCCTTAGATGGAGTTTACCACCCGCTTTGGGCTGCATTCC | 185731 | 0.26120531067070335 | No Hit |
| CCTCACCCGGCCCGGACACGGACAGGATTGACAGATTGATAGCTCTTTCT | 156203 | 0.21967820742200211 | No Hit |
| CGCGTAACTAGTTAGCATGCCAGAGTCTCGTTCGTTATCGGAATTAACCA | 154220 | 0.21688938847923006 | No Hit |
| CTCCCTTTCGATCGGCCGAGGGCAACGGAGGCCATCGCCCGTCCCTTCGG | 148070 | 0.20824025257502005 | No Hit |
| CAAAGATTAAGCCATGCATGTCTAAGTACGCACGGCCGGTACAGTGAAAC | 134752 | 0.18951030266083002 | No Hit |
| CTTTAAATGGGTAAGAAGCCCGGCTCGCTGGCGTGGAGCCGGGCGTGGAA | 129429 | 0.182024229422113 | No Hit |

| Sequence | Count | Percentage | Possible Source |
|---|---|---|---|
| CTGCTGTCTATATCAACCAACACCTTTTCTGGGGTCTGATGAGCGTCGGC | 122051 | 0.1716480790641843 | No Hit |
| CAAACTTTAAATGGGTAAGAAGCCCGGCTCGCTGGCGTGGAGCCGGGCGT | 121591 | 0.17100115182582062 | No Hit |
| CTGGGGTCTGATGAGCGTCGGCATCGGGCGCCTTAACCCGGCGTTCGGTT | 113438 | 0.1595350697076 0534 | No Hit |
| CCGTCGGCATGTATTAGCTCTAGAATTACCACAGTTATCCAAGTAGGAGA | 111626 | 0.15698673893387713 | No Hit |
| CGGGTCTTCCGTACGCCACATGTCCCGCGCCCCGCCGCGGGGCGGGGATT | 110759 | 0.15576742172591776 | No Hit |
| CTTGGTTATAATTTTTCATCTTTCCCTTGCGGTACTATATCTATTGCGCC | 108386 | 0.15243012099409822 | No Hit |
| CCTGCCAGTAGCATATGCTTGTCTCAAAGATTAAGCCATGCATGTCTAAG | 105638 | 0.14856543392665608 | No Hit |
| CTCGCATTCCACGCCCGGCTCCACGCCAGCGAGCCGGGCTTCTTACCCAT | 101641 | 0.14294419877070044 | No Hit |
| CTTGTCTCAAAGATTAAGCCATGCATGTCTAAGTACGCACGGCCGGTACA | 101541 | 0.1428035624145344 | No Hit |
| CTGATGAGCGTCGGCATCGGGCGCCTTAACCCGGCGTTCGGTTCATCCCG | 98910 | 0.1391034198838065 | No Hit |
| CTGGATAGTAGGTAGGGACAGTGGGAATCTCGTTCATCCATTCATGCGCG | 98731 | 0.13885168080626936 | No Hit |
| CTCACCCGGCCCGGACACGGACAGGATTGACAGATTGATAGCTCTTTCTC | 98291 | 0.1382328808391389 | No Hit |
| GCCCTCTTGAACTCTCTCTTCAAAGTTCTTTTCAACTTTCCCTTACGGTA | 93624 | 0.1316693820968709 | No Hit |
| GTCGGCATGTATTAGCTCTAGAATTACCACAGTTATCCAAGTAGGAGAGG | 89355 | 0.12566561605214369 | No Hit |
| ATCAGACGTGGCGACCCGCTGAATTTAAGCATATTAGTCAGCGGAGGAAA | 89282 | 0.1255629515121425 | No Hit |
| CCCGAAGTTACGGATCCGGCTTGCCGACTTCCCTTACCTACATTGTTCCA | 88437 | 0.12437457430253965 | No Hit |
| CTTCACCGTGCCAGACTAGAGTCAAGCTCAACAGGGTCTTCTTTCCCCGC | 85672 | 0.12048597905454932 | No Hit |
| CTCCCGTCCACTCTCGACTGCCGGCGACGGCCGGGTATGGGCCCGACGCT | 85168 | 0.11977717181947259 | No Hit |
| CTCCGACTTTCGTTCTTGATTAATGAAAACATTCTTGGCAAATGCTTTCG | 83573 | 0.11753402193862464 | No Hit |
| CCACTCTCGACTGCCGGCGACGGCCGGGTATGGGCCCGACGCTCCAGCGC | 82345 | 0.11580700748490597 | No Hit |
| CCCTCCTTAGGCAACCTGGTGGTCCCCCGCTCCCGGGAGGTCACCATATT | 79437 | 0.11171730224759822 | No Hit |
| CTCCGTTTCCGACCTGGGCCGGTTCACCCCTCCTTAGGCAACCTGGTGGT | 78662 | 0.11062737048731158 | No Hit |
| CCCGCTTTGGGCTGCATTCCCAAGCAACCCGACTCCGGGAAGACCCGGGC | 77439 | 0.10890738785140121 | No Hit |
| CGTCGGCATGTATTAGCTCTAGAATTACCACAGTTATCCAAGTAGGAGAG | 77382 | 0.10882722512838658 | No Hit |
| CACAGTTATCCAAGTAGGAGAGGAGCGAGCGACCAAAGGAACCATAACTG | 77078 | 0.10839969060564189 | No Hit |
| GTTAATTGTCAGTTCAGTGTTTTAATCTGACGCAGGCTTATGCGGAGGAG | 76115 | 0.10704536249576316 | No Hit |
| CGCGTCACTAATTAGATGACGAGGCATTTGGCTACCTTAAGAGAGTCATA | 71798 | 0.10097409100007623 | No Hit |
| CCCAGGCATAGTTCACCATCTTTCGGGTCCTAACACGTGCGCTCGTGCTC | 71248 | 0.10020059104116315 | No Hit |

## Adapter Content

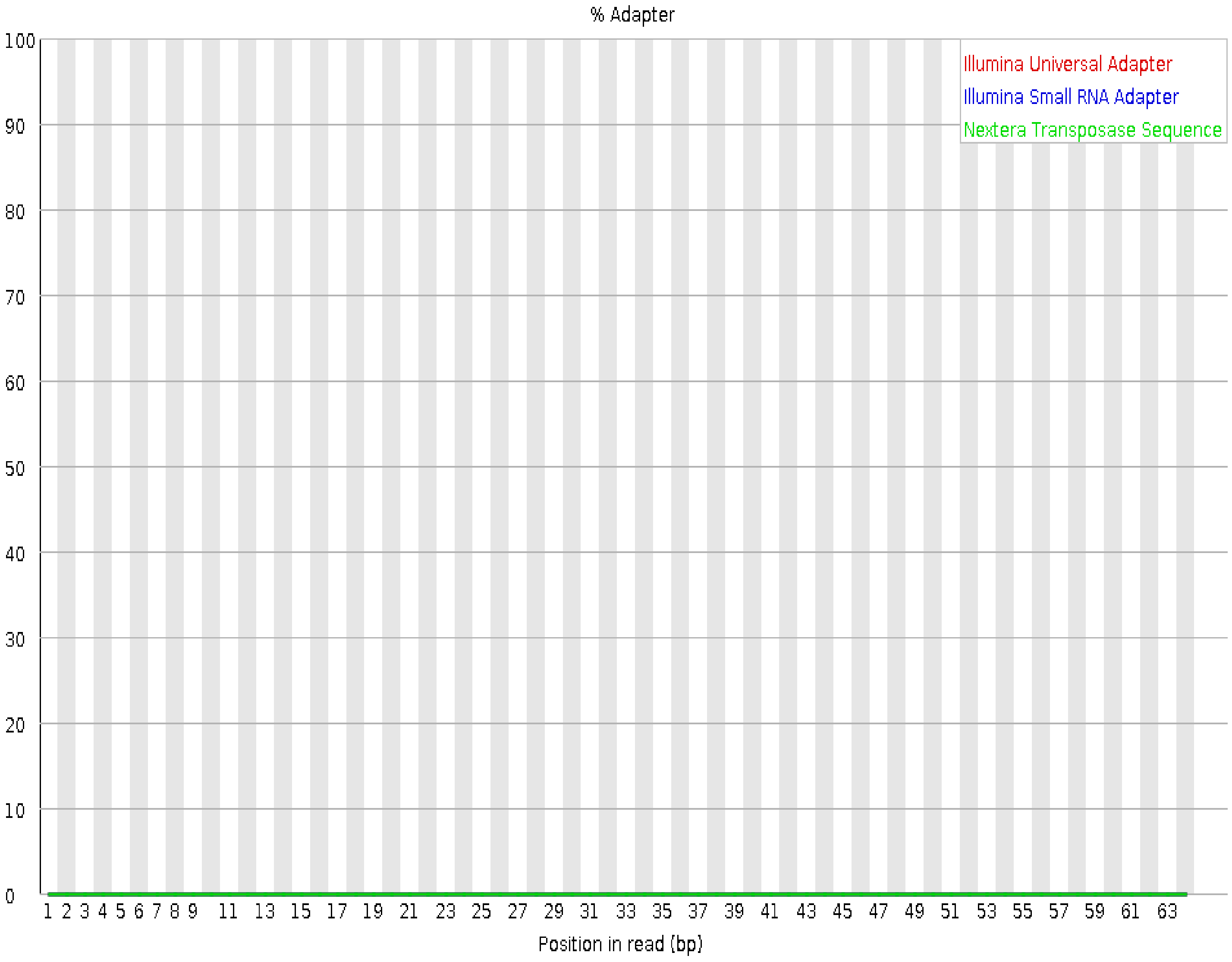


## Kmer Content

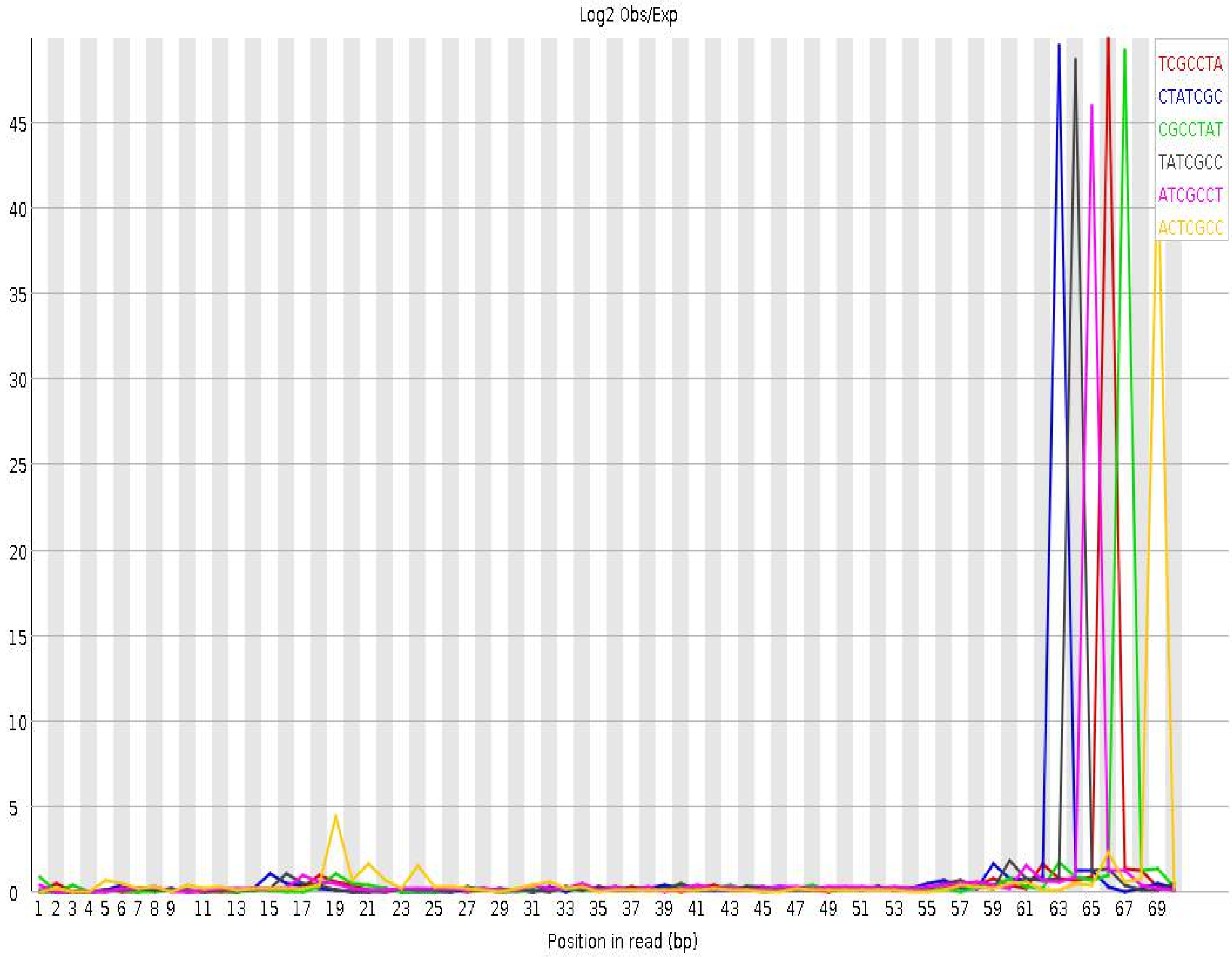


| Sequence | Count | PValue | Obs/Exp Max | Max Obs/Exp Position |
|---|---|---|---|---|
| TCGCCTA | 17125 | 0.0 | 49.795734 | 66 |
| CTATCGC | 17200 | 0.0 | 49.43251 | 63 |
| CGCCTAT | 17355 | 0.0 | 49.176414 | 67 |
| TATCGCC | 17555 | 0.0 | 48.571613 | 64 |
| ATCGCCT | 18560 | 0.0 | 45.944298 | 65 |
| ACTCGCC | 48155 | 0.0 | 42.906357 | 69 |
| CGCGTAA | 29250 | 0.0 | 42.847393 | 1 |
| TCACTCG | 51235 | 0.0 | 40.527557 | 67 |
| GCCTATA | 21630 | 0.0 | 39.68761 | 68 |
| CCCGTCG | 66210 | 0.0 | 39.35398 | 1 |
| GCGTAAC | 32790 | 0.0 | 38.427917 | 2 |
| TCAGACG | 29725 | 0.0 | 36.36145 | 2 |
| CCGTCGG | 74705 | 0.0 | 34.535244 | 2 |
| CTCGCCG | 66485 | 0.0 | 34.282387 | 70 |
| TGCCGTA | 12220 | 0.0 | 34.084858 | 2 |

| Sequence | Count | PValue | Obs/Exp Max | Max Obs/Exp Position |
|---|---|---|---|---|
| CGGAACG | 34145 | 0.0 | [illegible] | 48 |
| CGTAACT | 37715 | 0.0 | [illegible] | 3 |
| CTCGCTA | 7140 | 0.0 | 33.10871 | 1 |
| TAGTTAG | 36655 | 0.0 | 33.036846 | 9 |
| AGTTAGC | 36290 | 0.0 | 32.890156 | 10 |

**Produced by FastQC (version 0.11.2)**

# FastQC Report

Mon 4 May 2015
Agilent_Adult_Colon_CGATGT_L007_R2.fastq.gz

## Summary


- Basic Statistics
- Per base sequence quality
- Per tile sequence quality
- Per sequence quality scores
- Per base sequence content
- Per sequence GC content
- Per base N content
- Sequence Length Distribution
- Sequence Duplication Levels
- Overrepresented sequences
- Adapter Content
- Kmer Content


## Basic Statistics

| Measure | Value |
|---|---|
| Filename | Agilent_Adult_Colon_CGATGT_L007_R2.fastq.gz |
| File type | Conventional base calls |
| Encoding | Sanger / Illumina 1.9 |
| Total Sequences | 71105369 |
| Sequences flagged as poor quality | 0 |
| Sequence length | 76 |
| %GC | 51 |

## Per base sequence quality

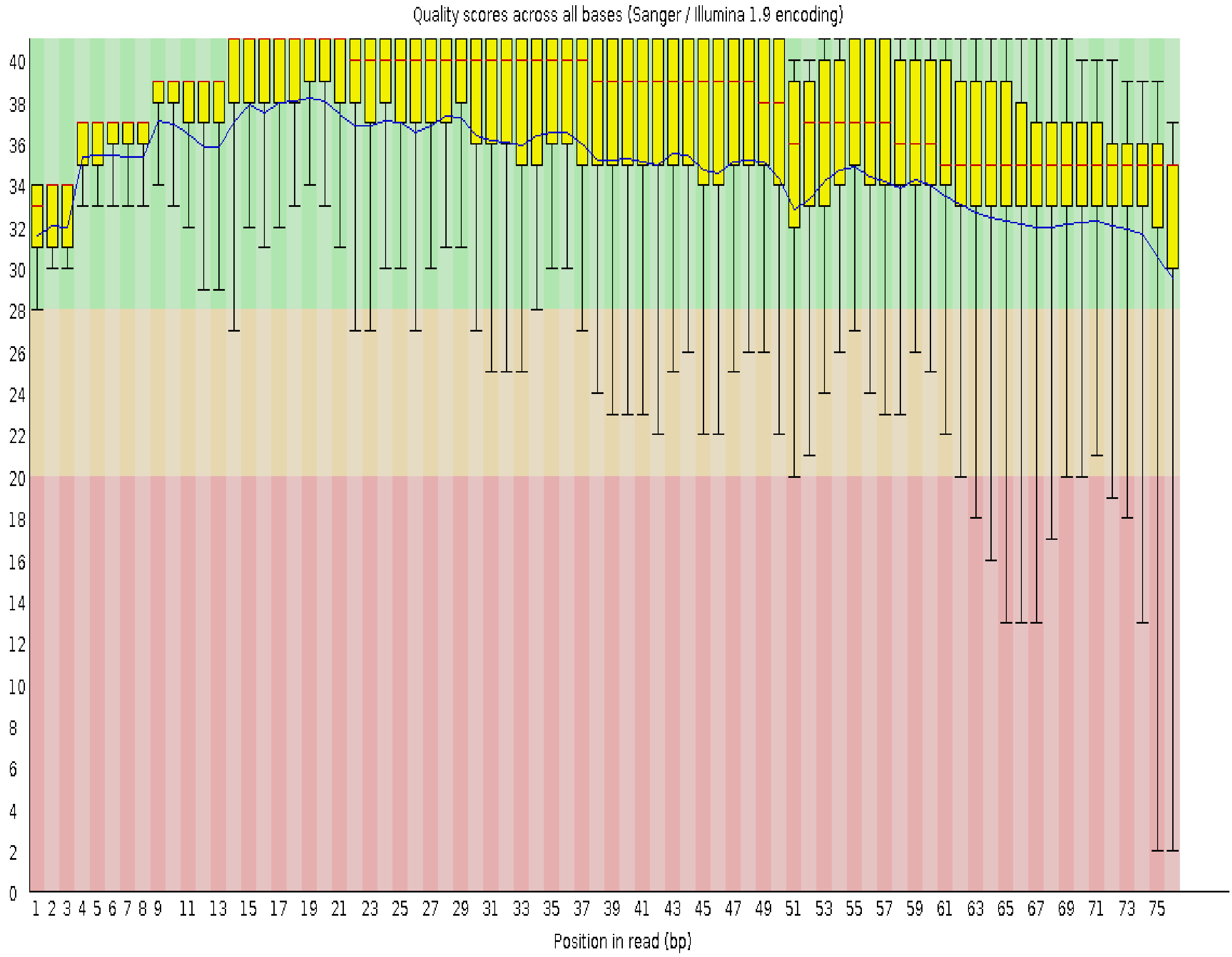


## Per tile sequence quality

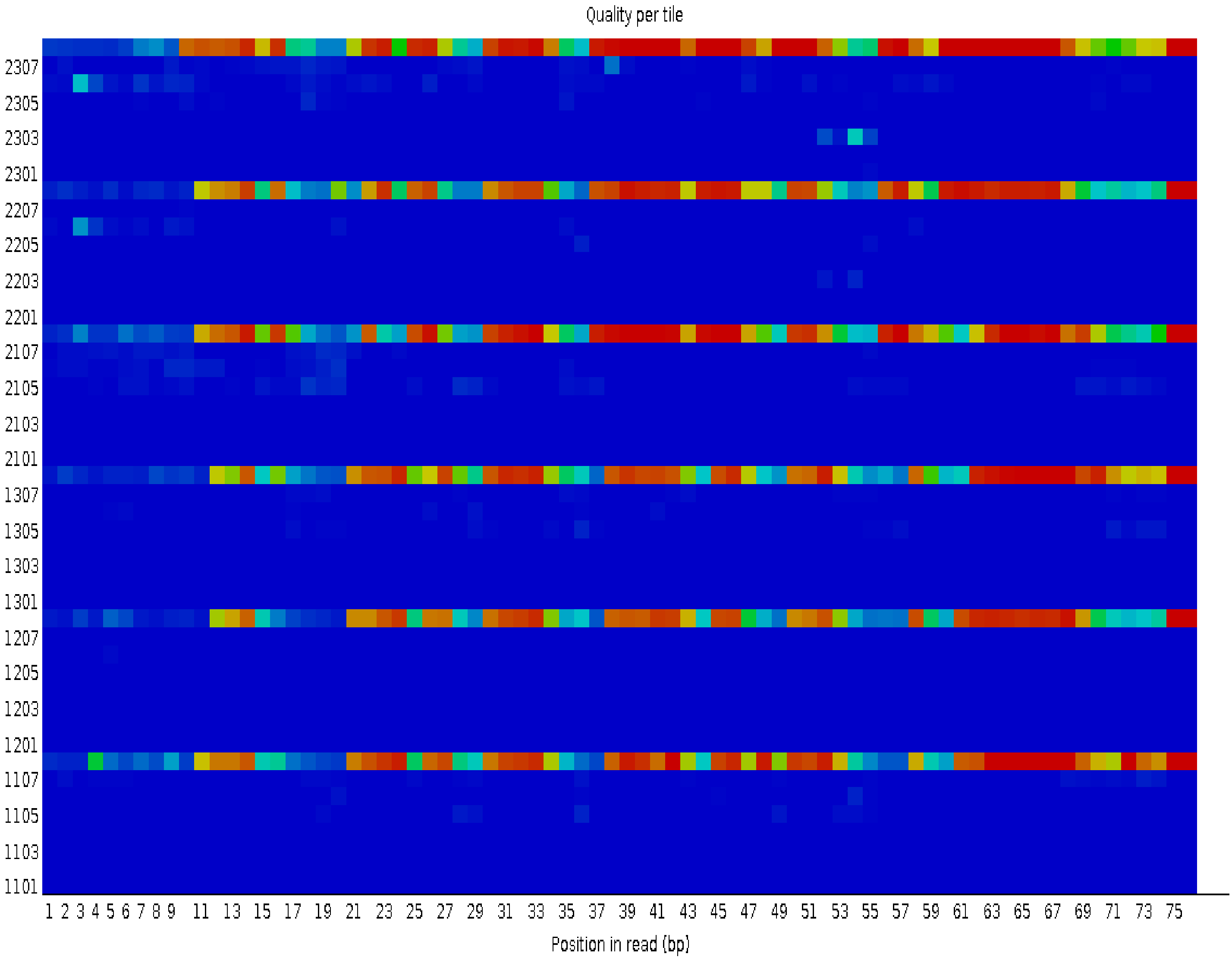


## Per sequence quality scores

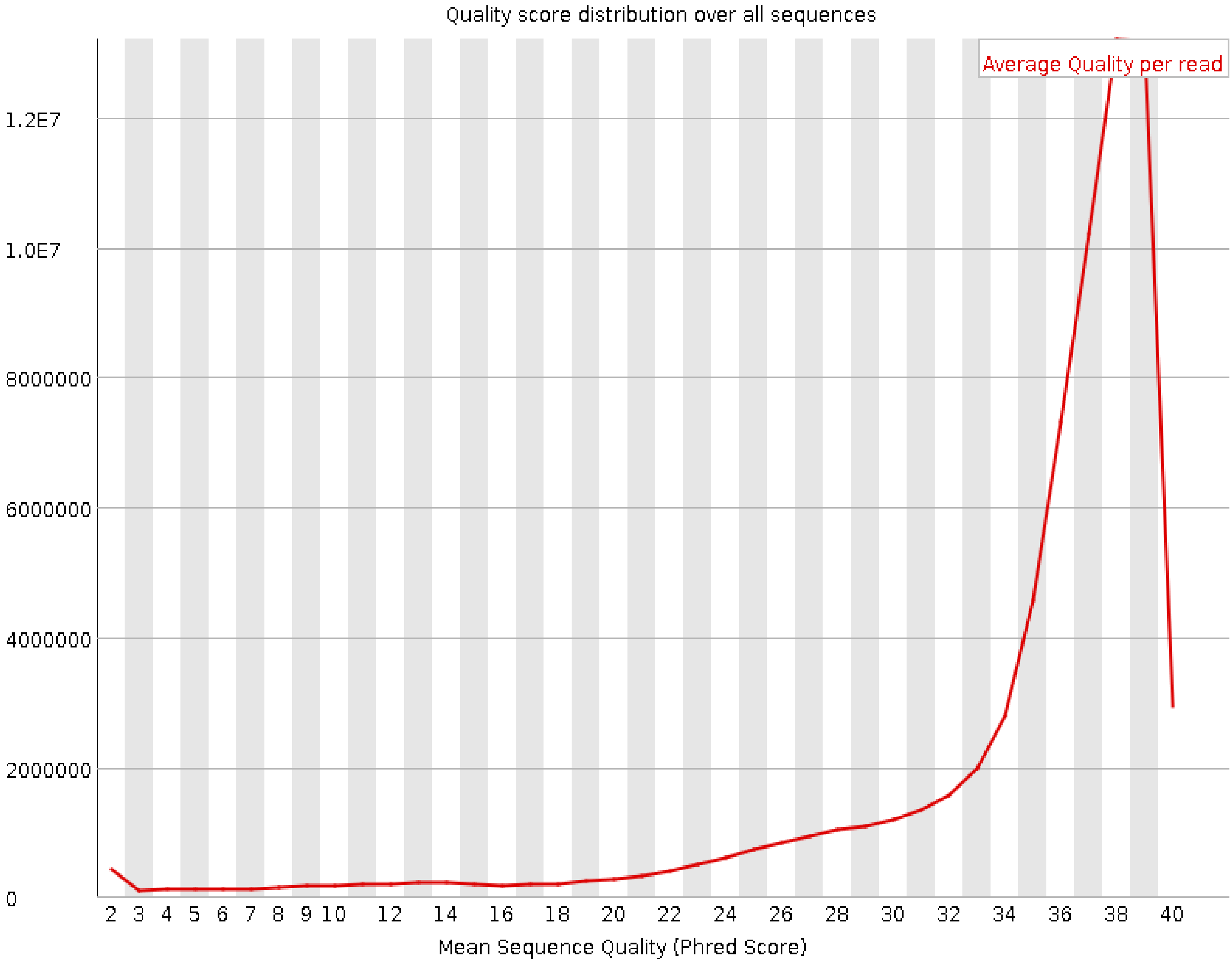


## Per base sequence content

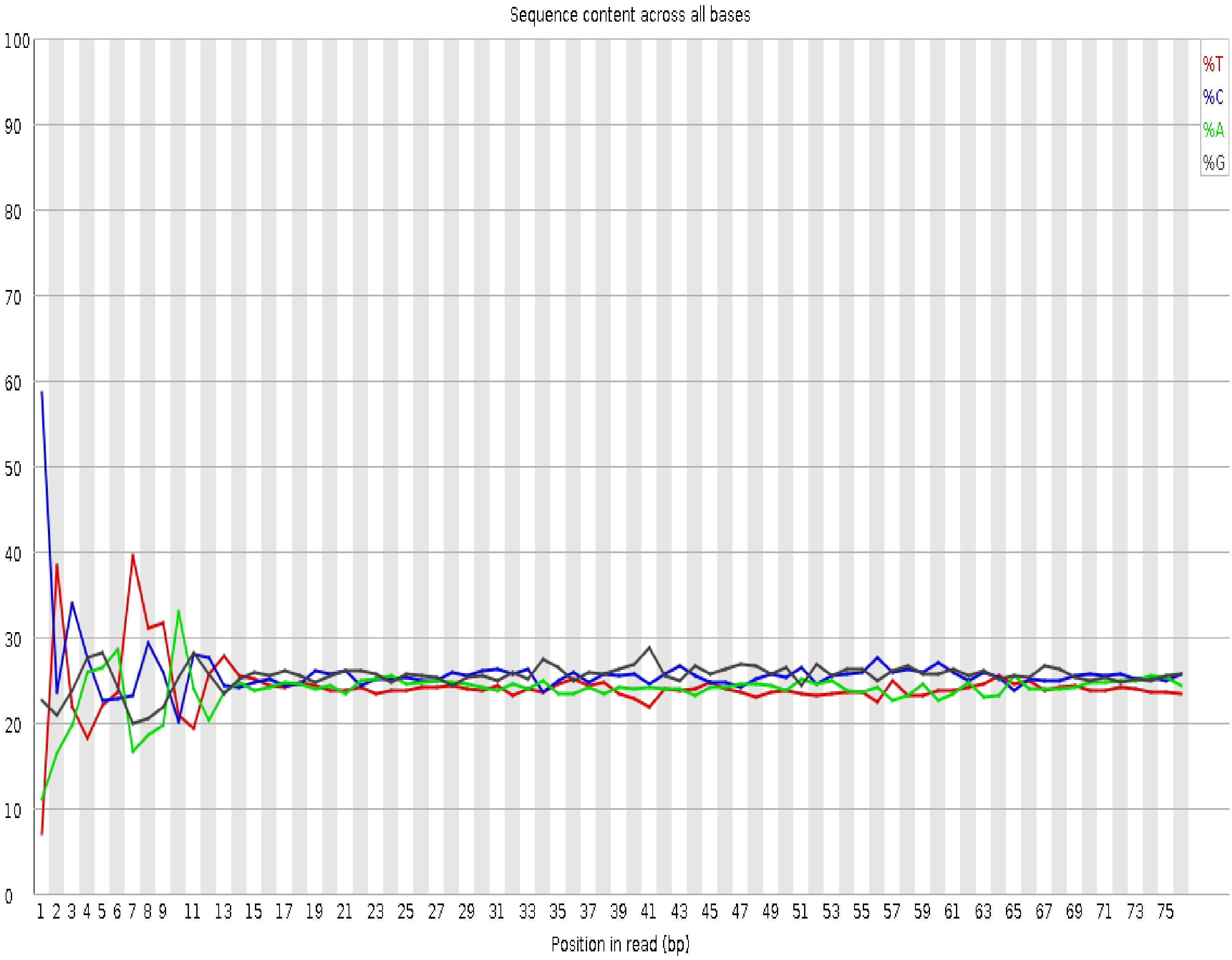


## Per sequence GC content

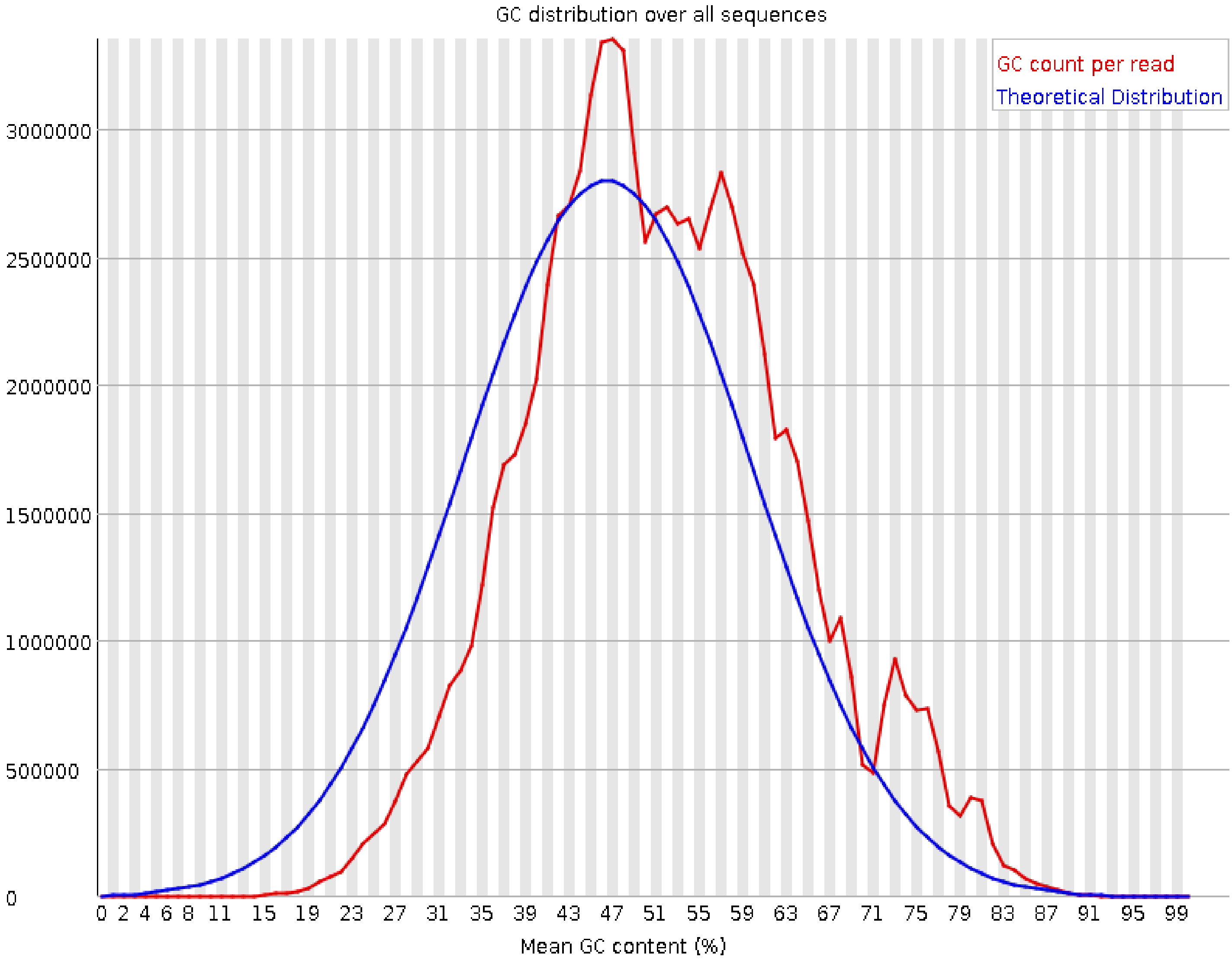


## Per base N content

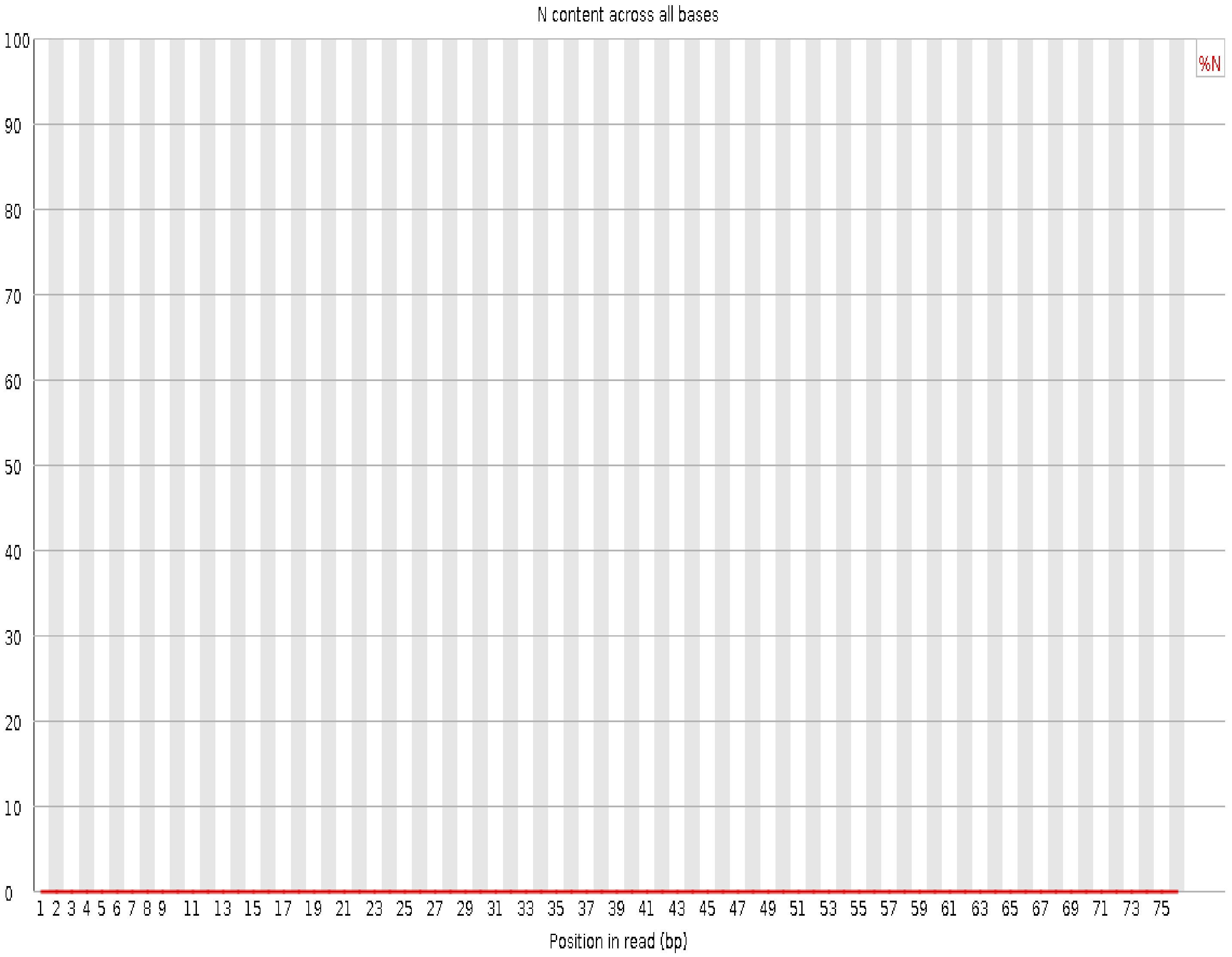


## Sequence Length Distribution

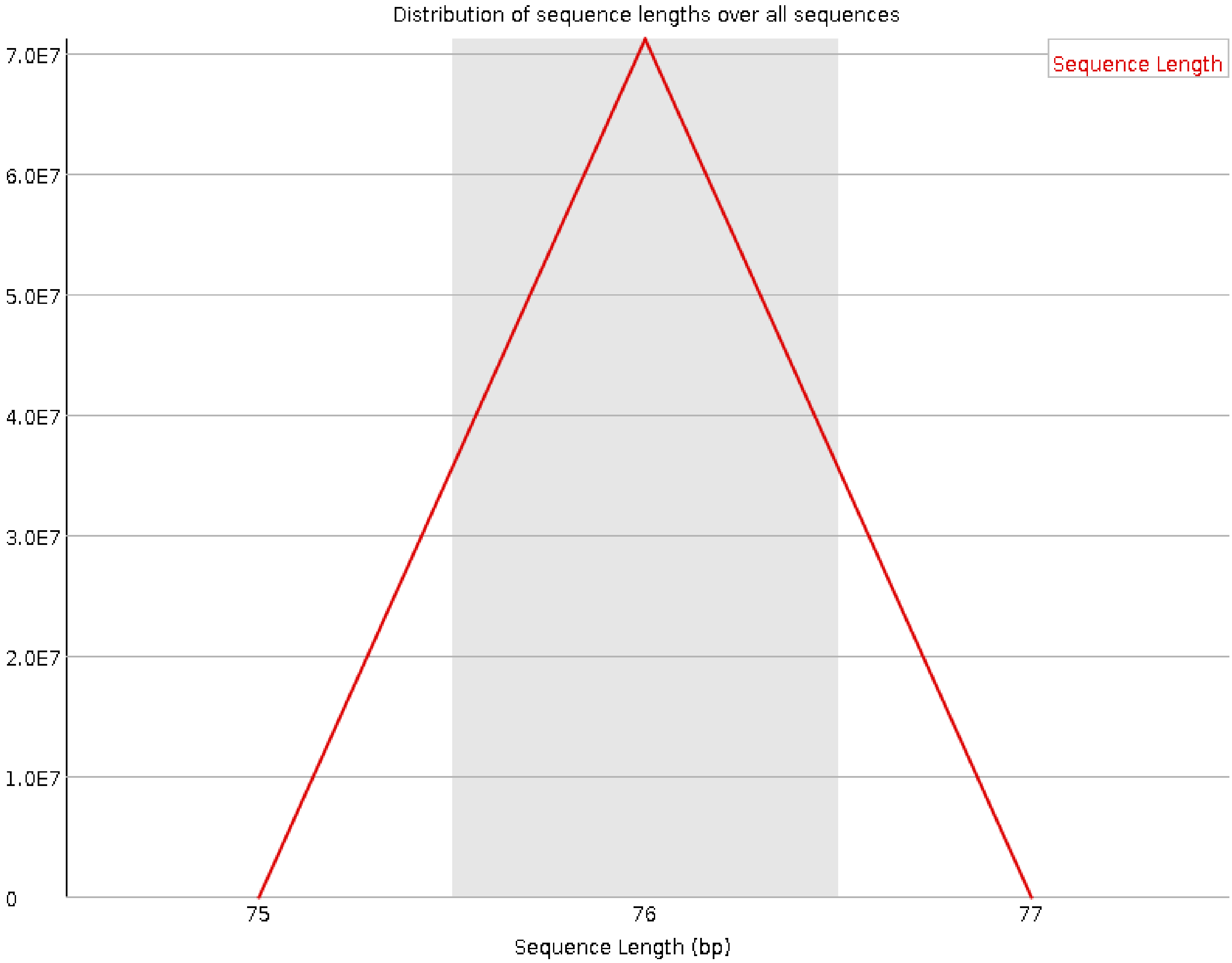


## Sequence Duplication Levels

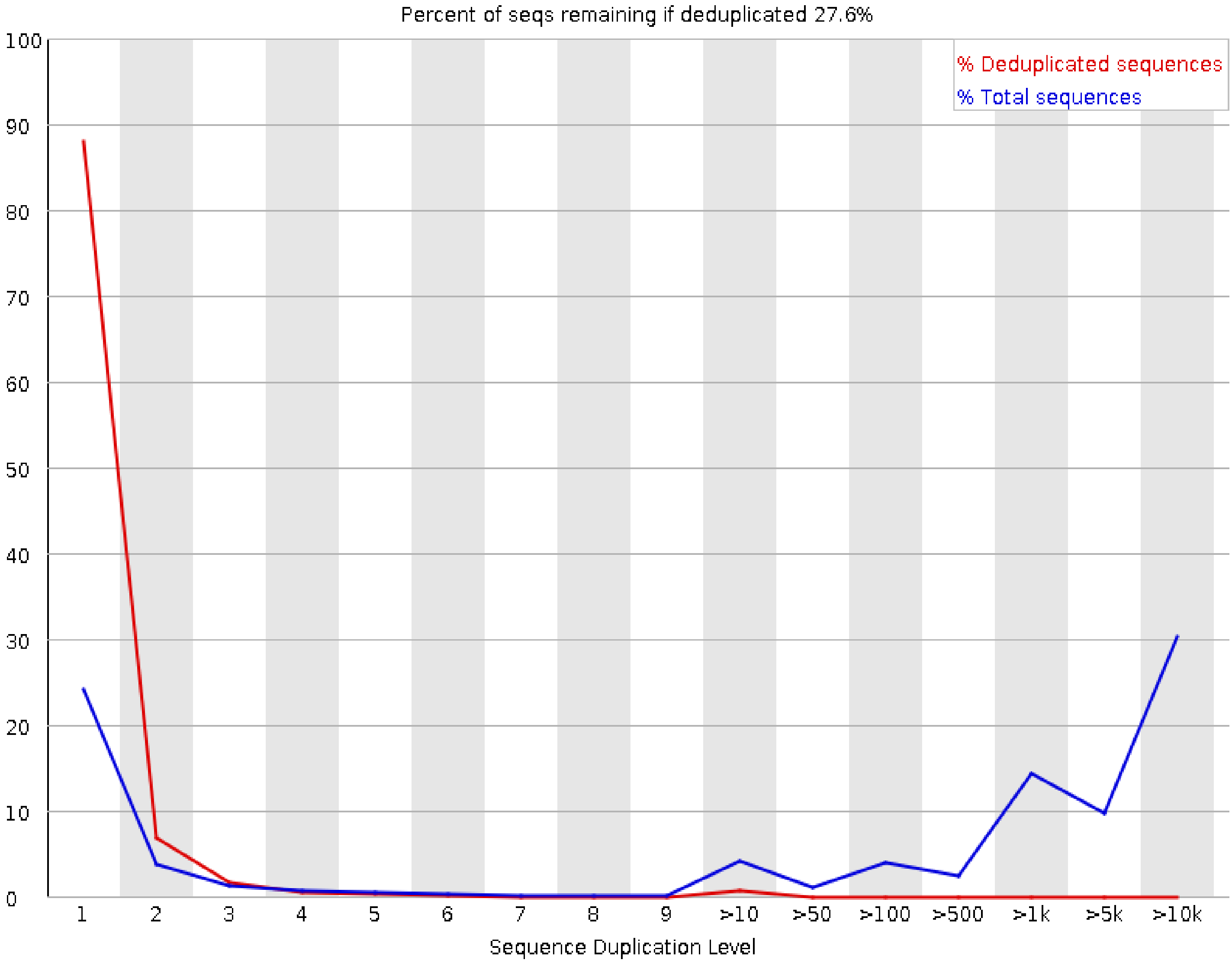


## Overrepresented sequences

| Sequence | Count | Percentage | Possible Source |
|---|---|---|---|
| CCCGTCGGCATGTATTAGCTCTAGAATTACCACAGTTATCCAAGTAGGAG | 304419 | 0.42812378907702453 | No Hit |
| CTTCCGTACGCCACATGTCCCGCGCCCCGCCGCGGGGCGGGGATTCGGCG | 258251 | 0.3631947961622982 | No Hit |
| CTGCCAGTAGCATATGCTTGTCTCAAAGATTAAGCCATGCATGTCTAAGT | 238942 | 0.33603932215020216 | No Hit |
| CGCAGTTTTATCCGGTAAAGCGAATGATTAGAGGTCTTGGGGCCGAAACG | 200996 | 0.2826734504394457 | No Hit |
| CTCTCTTCAAAGTTCTTTTCAACTTTCCCTTACGGTACTTGTTGACTATC | 186172 | 0.2618255170013955 | No Hit |
| CCGGTATTTAGCCTTAGATGGAGTTTACCACCCGCTTTGGGCTGCATTCC | 181930 | 0.2558597227728331 | No Hit |
| CCTCACCCGGCCCGGACACGGACAGGATTGACAGATTGATAGCTCTTTCT | 161468 | 0.22708271157414286 | No Hit |
| CTCCCTTTCGATCGGCCGAGGGCAACGGAGGCCATCGCCCGTCCCTTCGG | 160781 | 0.22611653980728233 | No Hit |
| CGCGTAACTAGTTAGCATGCCAGAGTCTCGTTCGTTATCGGAATTAACCA | 146191 | 0.2055976954426606 | No Hit |
| CAAAGATTAAGCCATGCATGTCTAAGTACGCACGGCCGGTACAGTGAAAC | 141742 | 0.19934078395683455 | No Hit |
| CGGGTCTTCCGTACGCCACATGTCCCGCGCCCCGCCGCGGGGCGGGGATT | 128928 | 0.18131964127772124 | No Hit |

| Sequence | Count | Percentage | Possible Source |
|---|---|---|---|
| CTGCTGTCTATATCAACCAACACCTTTTCTGGGGTCTGATGAGCGTCGGC | 124634 | 0.1752807161439525 | No Hit |
| CAAACTTTAAATGGGTAAGAAGCCCGGCTCGCTGGCGTGGAGCCGGGCGT | 118283 | 0.1663489011638484 | [illegible] |
| CTTTAAATGGGTAAGAAGCCCGGCTCGCTGGCGTGGAGCCGGGCGTGGAA | 116459 | 0.1637836940273807 | No Hit |
| CTGGGGTCTGATGAGCGTCGGCATCGGGCGCCTTAACCCGGCGTTCGGTT | 108408 | 0.15246106099245474 | No Hit |
| CCTGCCAGTAGCATATGCTTGTCTCAAAGATTAAGCCATGCATGTCTAAG | 106908 | 0.1503515156499645 | No Hit |
| CCGTCGGCATGTATTAGCTCTAGAATTACCACAGTTATCCAAGTAGGAGA | 105095 | 0.14780177851267462 | No Hit |
| CTCGCATTCCACGCCCGGCTCCACGCCAGCGAGCCGGGCTTCTTACCCAT | 102725 | 0.14446869687154004 | No Hit |
| CTTGTCTCAAAGATTAAGCCATGCATGTCTAAGTACGCACGGCCGGTACA | 102458 | 0.14409319780057678 | No Hit |
| CTCACCCGGCCCGGACACGGACAGGATTGACAGATTGATAGCTCTTTCTC | 101844 | 0.14322969057371743 | No Hit |
| CTGGATAGTAGGTAGGGACAGTGGGAATCTCGTTCATCCATTCATGCGCG | 99963 | 0.14058432071423468 | No Hit |
| CTTGGTTATAATTTTTCATCTTTCCCTTGCGGTACTATATCTATTGCGCC | 96281 | 0.13540609008020196 | No Hit |
| CTGATGAGCGTCGGCATCGGGCGCCTTAACCCGGCGTTCGGTTCATCCCG | 94923 | 0.13349624836346746 | No Hit |
| CTCCGACTTTCGTTCTTGATTAATGAAAACATTCTTGGCAAATGCTTTCG | 93248 | 0.1311405893976867 | No Hit |
| GCCCTCTTGAACTCTCTCTTCAAAGTTCTTTTCAACTTTCCCTTACGGTA | 92837 | 0.13056257397384435 | No Hit |
| CTCCCGTCCACTCTCGACTGCCGGCGACGGCCGGGTATGGGCCCGACGCT | 92123 | 0.12955843039081902 | No Hit |
| CTTCACCGTGCCAGACTAGAGTCAAGCTCAACAGGGTCTTCTTTCCCCGC | 91145 | 0.12818300682751538 | No Hit |
| CCACTCTCGACTGCCGGCGACGGCCGGGTATGGGCCCGACGCTCCAGCGC | 84559 | 0.11892069641042155 | No Hit |
| CTCCGTTTCCGACCTGGGCCGGTTCACCCCTCCTTAGGCAACCTGGTGGT | 83695 | 0.11770559829314717 | No Hit |
| GTCGGCATGTATTAGCTCTAGAATTACCACAGTTATCCAAGTAGGAGAGG | 82590 | 0.1161515665575127 | No Hit |
| ATCAGACGTGGCGACCCGCTGAATTTAAGCATATTAGTCAGCGGAGGAAA | 80616 | 0.11337540488679552 | No Hit |
| CCCGAAGTTACGGATCCGGCTTGCCGACTTCCCTTACCTACATTGTTCCA | 79928 | 0.11240782675637334 | No Hit |
| CCCTCCTTAGGCAACCTGGTGGTCCCCCGCTCCCGGGAGGTCACCATATT | 77811 | 0.10943055509633878 | No Hit |
| CCCGCTTTGGGCTGCATTCCCAAGCAACCCGACTCCGGGAAGACCCGGGC | 77557 | 0.1090733387516771 | No Hit |
| CTCCGCCACTCCGGATTCGGGGATCTGAACCCGACTCCCTTTCGATCGGC | 75589 | 0.10630561526232991 | No Hit |
| CGCGTCACTAATTAGATGACGAGGCATTTGGCTACCTTAAGAGAGTCATA | 72863 | 0.1024718681932443 | No Hit |
| CGTCGGCATGTATTAGCTCTAGAATTACCACAGTTATCCAAGTAGGAGAG | 72623 | 0.10213434093844587 | No Hit |
| CTCCGAGGTCGCCCCAACCGAAATTTTTAATGCAGGTTTGGTAGTTTAGG | 71894 | 0.10110910190199562 | No Hit |
| CTCGTGCCGGTATTTAGCCTTAGATGGAGTTTACCACCCGCTTTGGGCTG | 71642 | 0.10075469828445724 | No Hit |
| CTGCTTACCAAAAGTGGCCCACTAGGCACTCGCATTCCACGCCCGGCTCC | 71354 | 0.10034966557869913 | No Hit |
| CGACGACCCATTCGAACGTCTGCCCTATCAACTTTCGATGGTAGTCGCCG | 71343 | 0.10033419557952086 | No Hit |

## Adapter Content

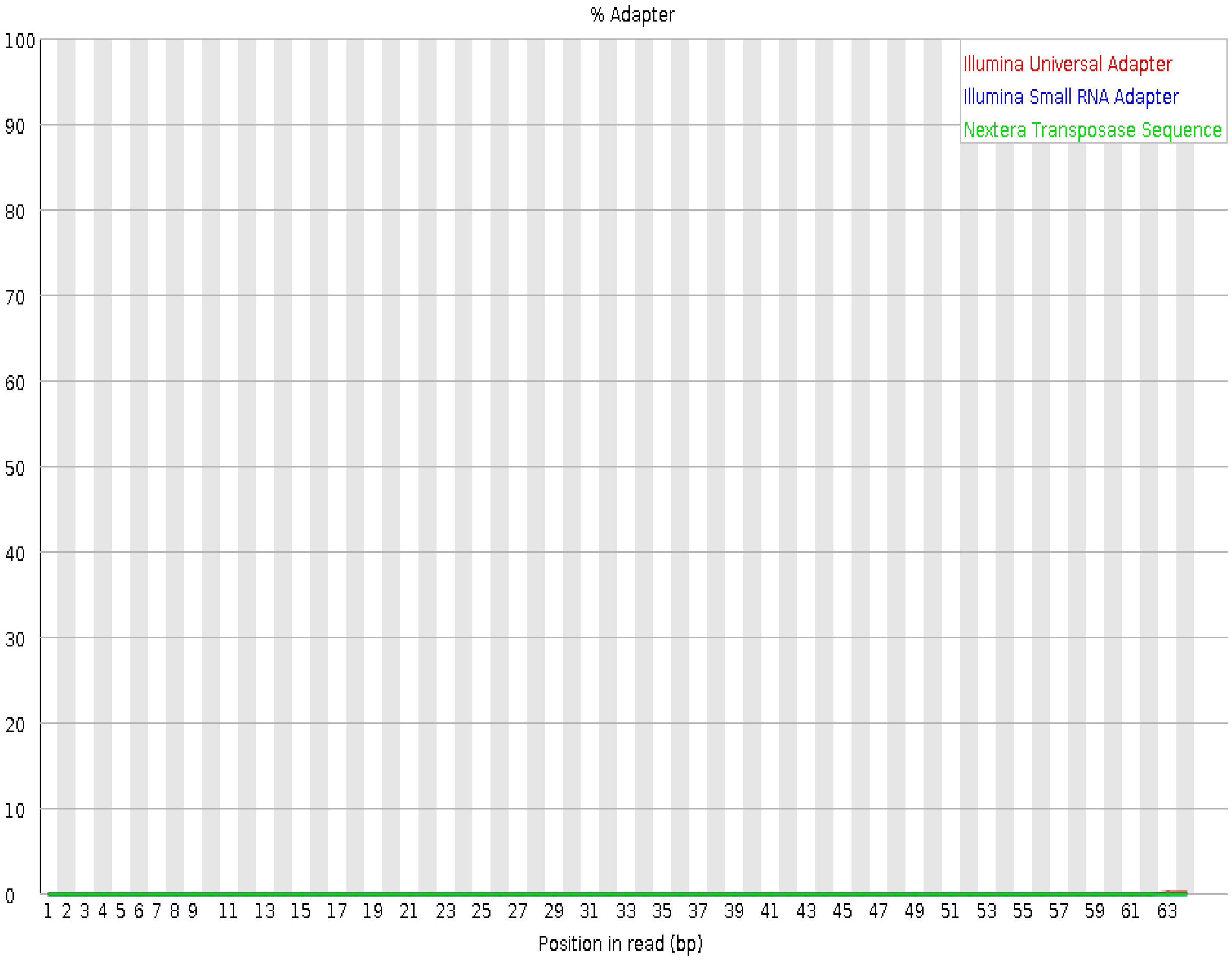


## Kmer Content

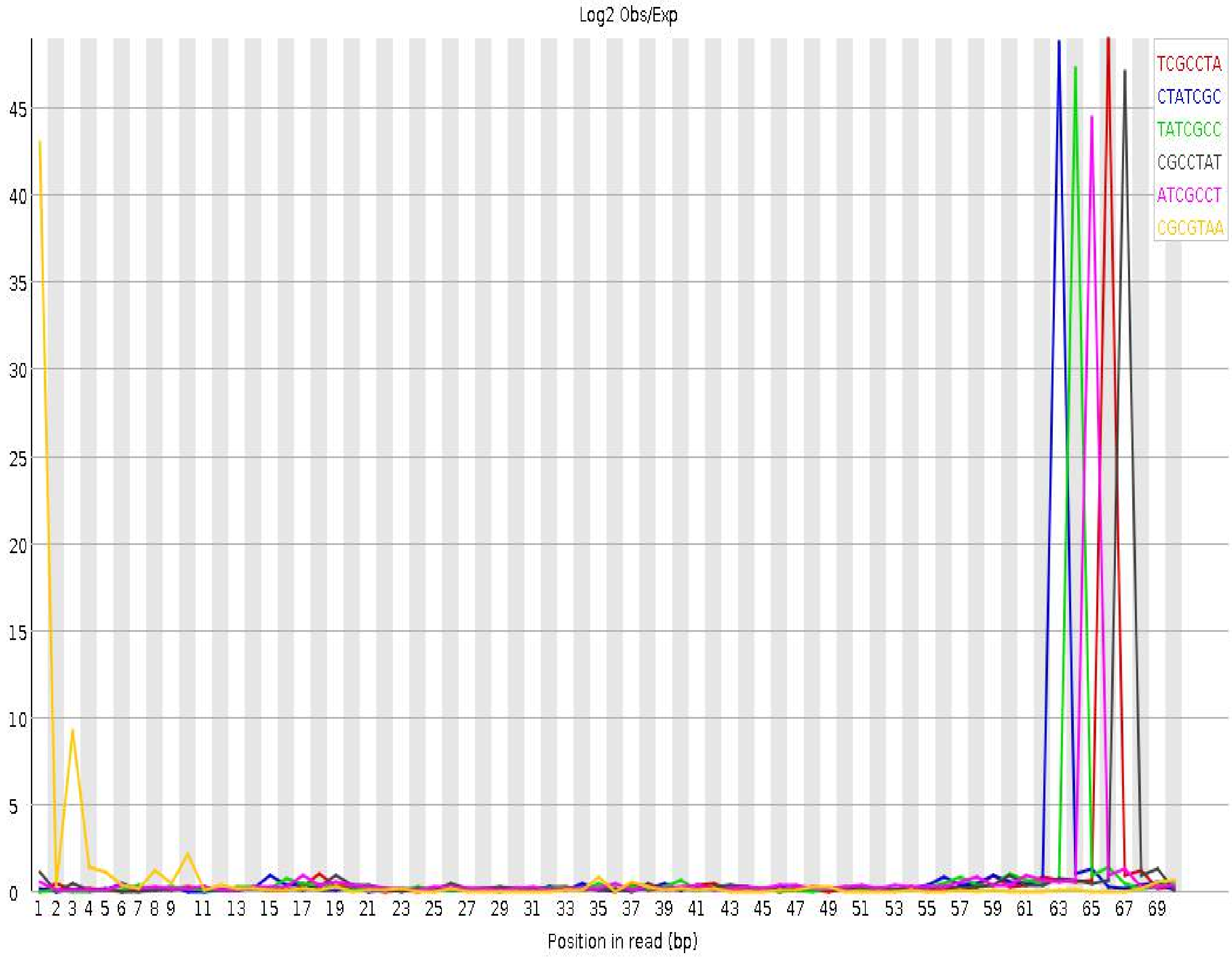


| Sequence | Count | PValue | Obs/Exp Max | Max Obs/Exp Position |
|---|---|---|---|---|
| TCGCCTA | 15460 | 0.0 | 48.87987 | 66 |
| CTATCGC | 15535 | 0.0 | 48.746227 | 63 |
| TATCGCC | 16005 | 0.0 | 47.191315 | 64 |
| CGCCTAT | 16185 | 0.0 | 47.014526 | 67 |
| ATCGCCT | 17015 | 0.0 | 44.451584 | 65 |
| CGCGTAA | 28595 | 0.0 | 42.983215 | 1 |
| ACTCGCC | 48575 | 0.0 | 42.033424 | 69 |
| TCACTCG | 50920 | 0.0 | 40.12708 | 67 |
| CCCGTCG | 65285 | 0.0 | 39.667423 | 1 |
| GCGTAAC | 32030 | 0.0 | 38.85547 | 2 |
| GCCTATA | 20815 | 0.0 | 37.89979 | 68 |
| CCGTCGG | 72595 | 0.0 | 35.308426 | 2 |
| CGGAACG | 37160 | 0.0 | 34.725372 | 48 |
| TCAGACG | 28390 | 0.0 | 34.673218 | 2 |
| CGTAACT | 36075 | 0.0 | 34.624157 | 3 |

| Sequence | Count | PValue | Obs/Exp Max | Max Obs/Exp Position |
|---|---|---|---|---|
| TGCCGTA | 12935 | 0.0 | [illegible] | 2 |
| GCCAGTA | 55770 | 0.0 | [illegible] | 3 |
| TAGTTAG | 35115 | 0.0 | 33.63356 | 9 |
| AGTTAGC | 35265 | 0.0 | 33.38315 | 10 |
| CTCGCCG | 66125 | 0.0 | 33.259922 | 70 |

**Produced by FastQC (version 0.11.2)**

# FastQC Report

Mon 4 May 2015
Agilent_Adult_Heart_ATCACG_L001_R1.fastq.gz

## Summary

- Basic Statistics
- Per base sequence quality
- Per tile sequence quality
- Per sequence quality scores
- Per base sequence content
- Per sequence GC content
- Per base N content
- Sequence Length Distribution
- Sequence Duplication Levels
- Overrepresented sequences
- Adapter Content
- Kmer Content

## Basic Statistics

| Measure | Value |
|---|---|
| Filename | Agilent_Adult_Heart_ATCACG_L001_R1.fastq.gz |
| File type | Conventional base calls |
| Encoding | Sanger / Illumina 1.9 |
| Total Sequences | 67643565 |
| Sequences flagged as poor quality | 0 |
| Sequence length | 76 |
| %GC | 54 |

## Per base sequence quality

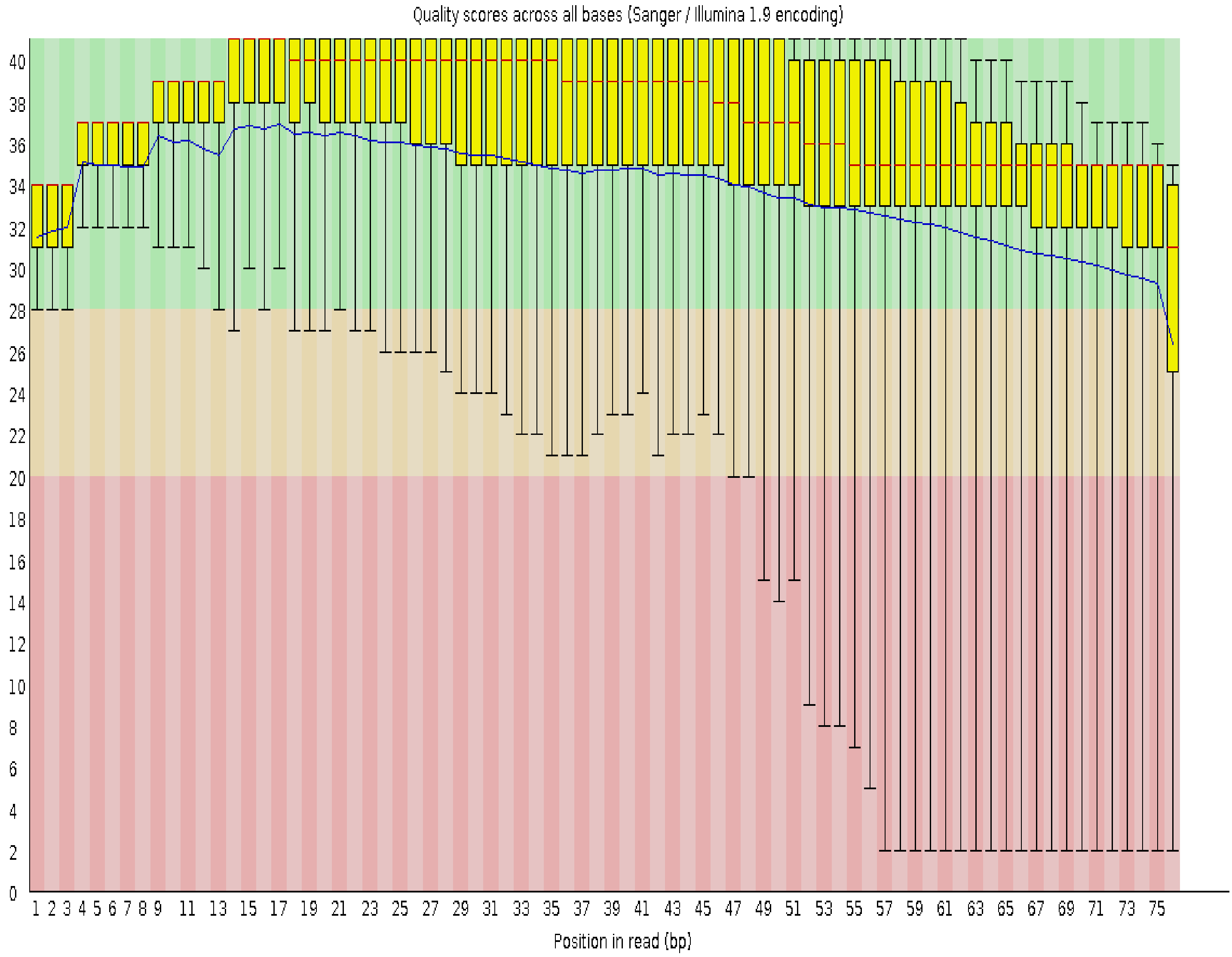


## Per tile sequence quality

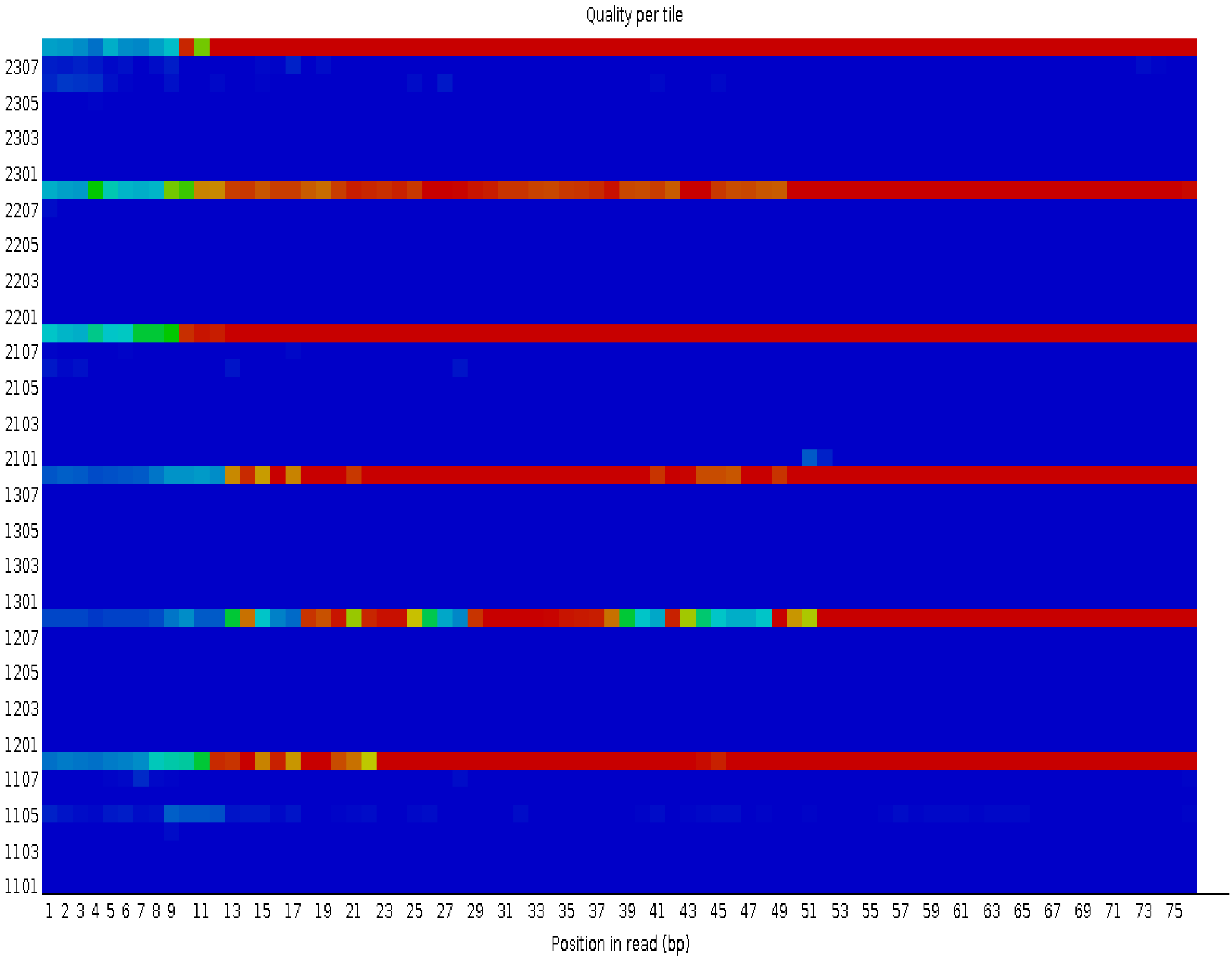


## Per sequence quality scores

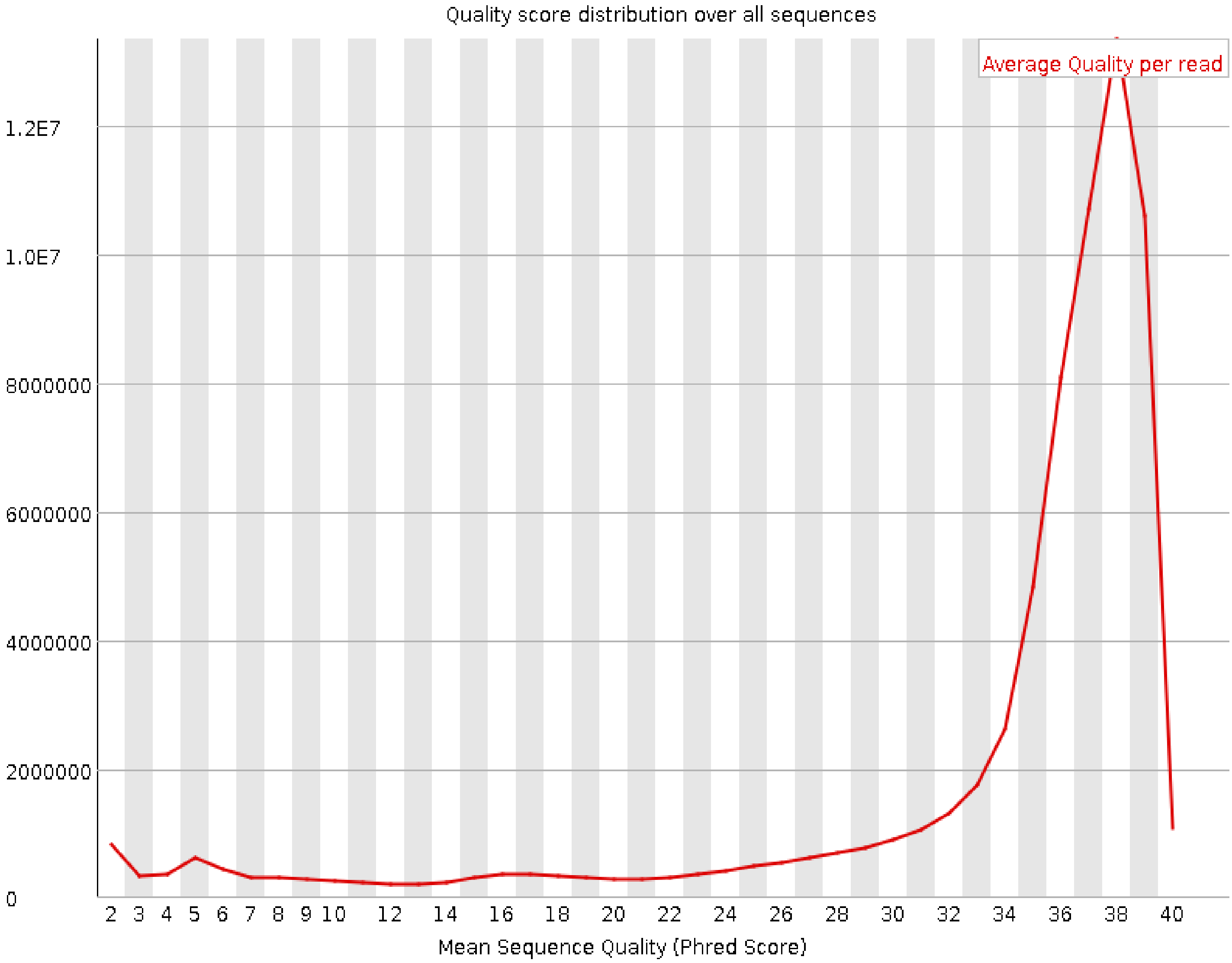


## Per base sequence content

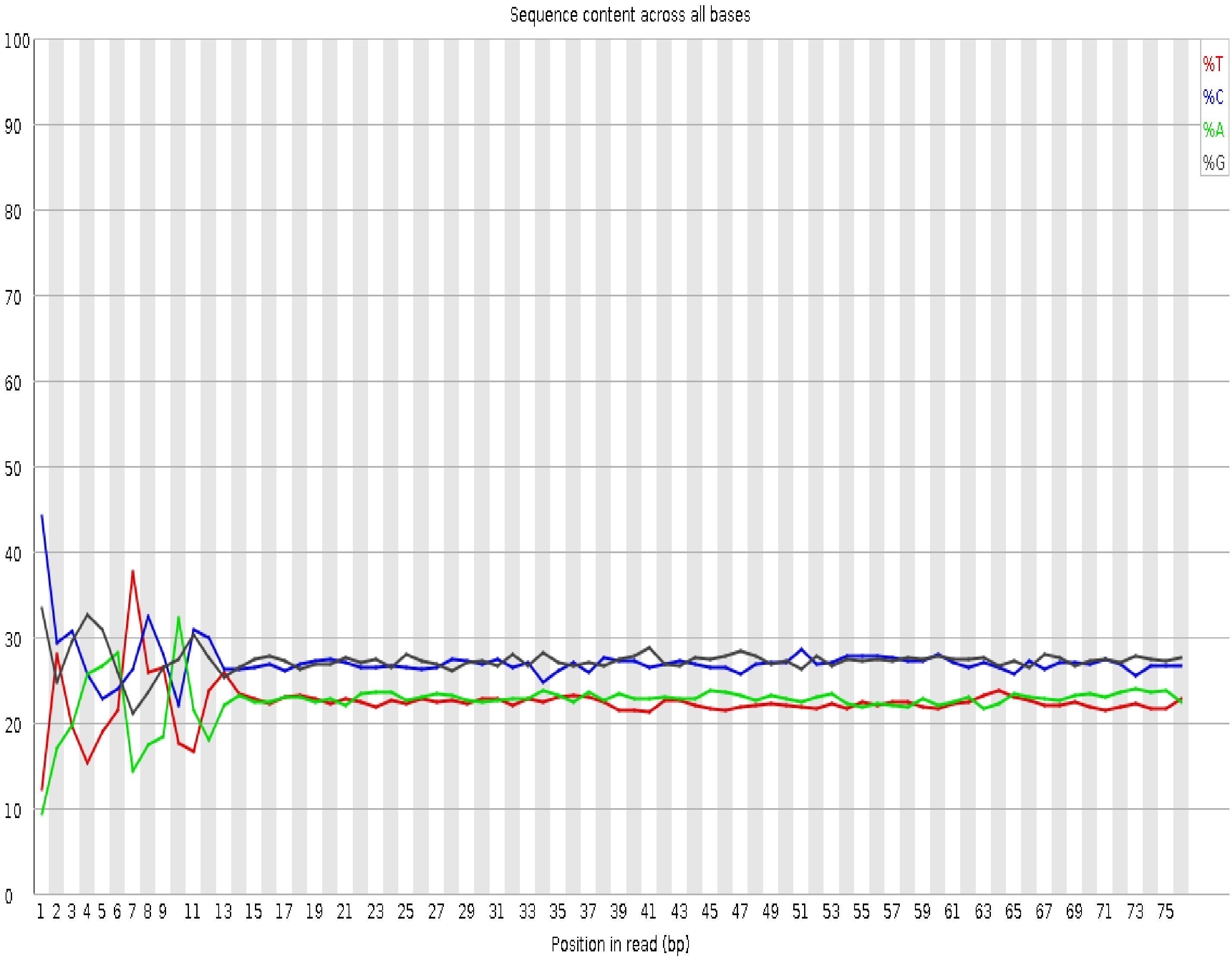


## Per sequence GC content

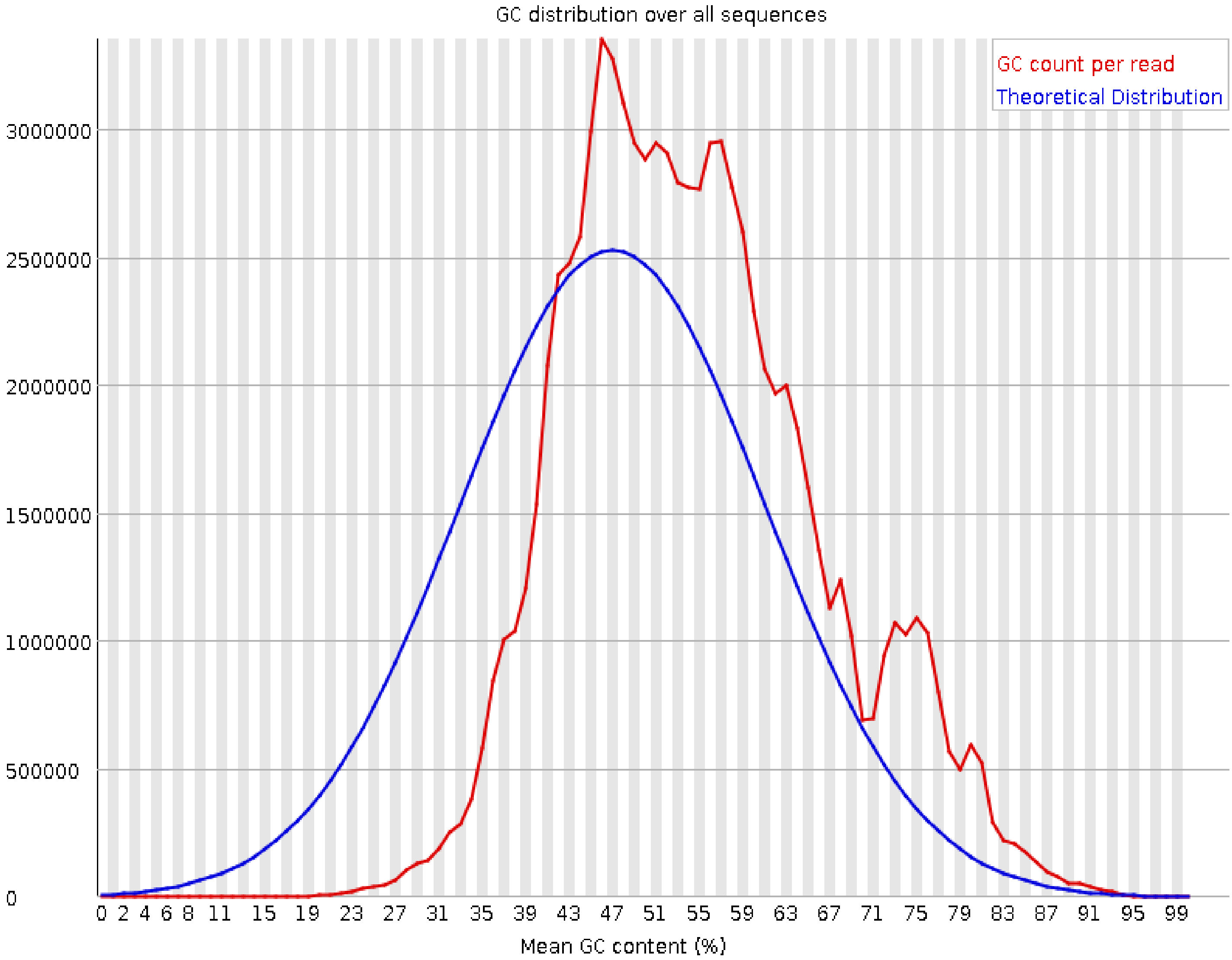


## Per base N content

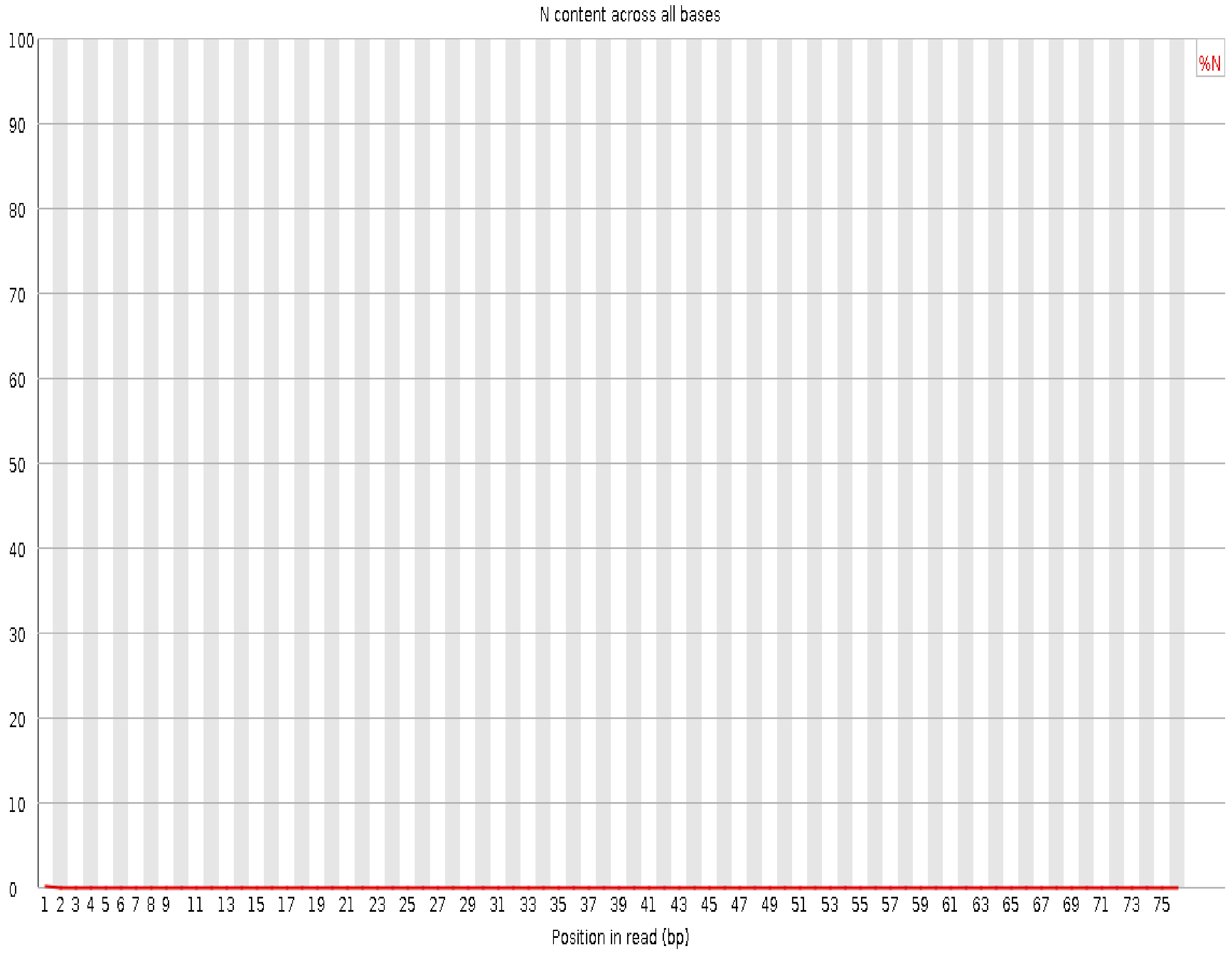


## Sequence Length Distribution

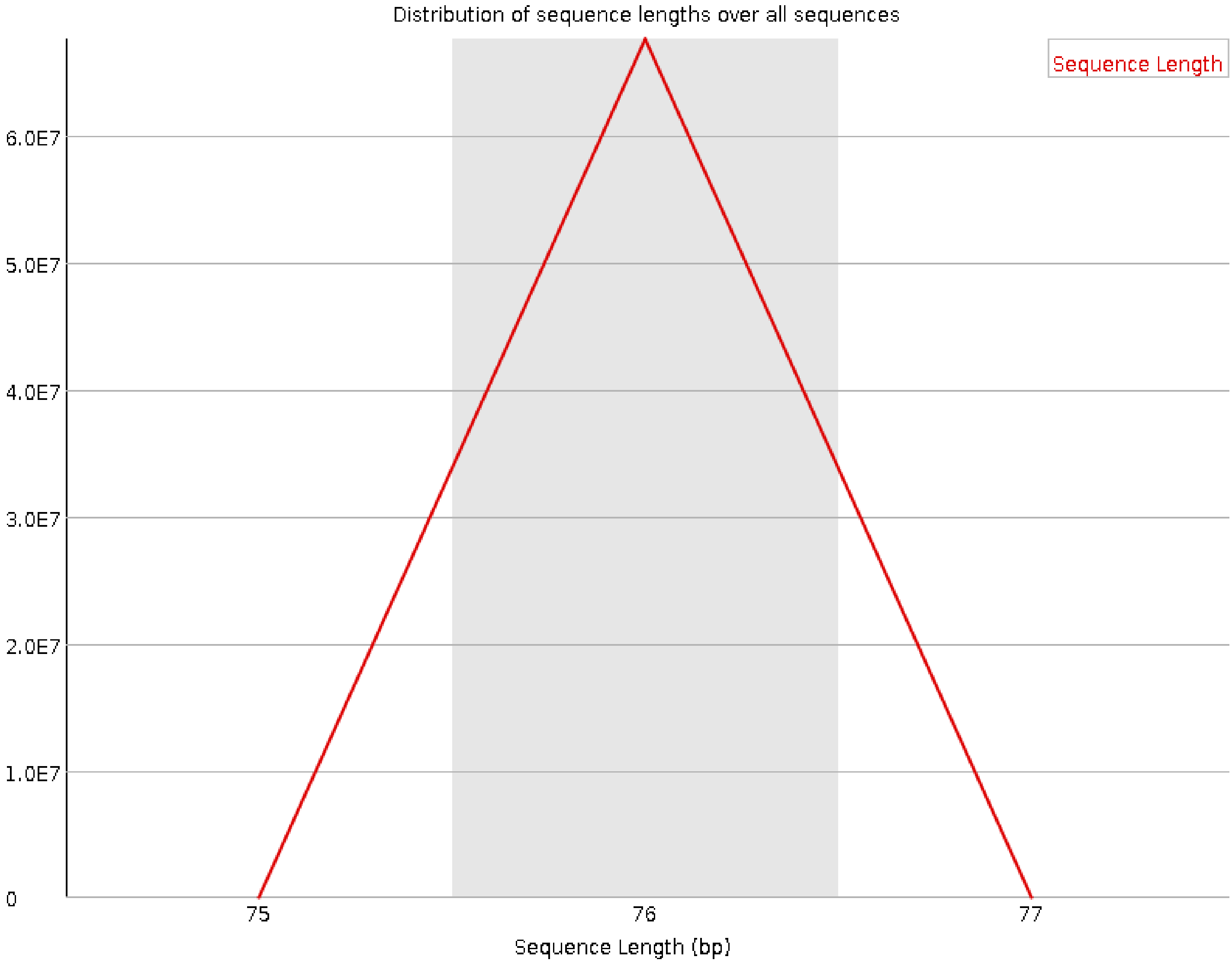


## Sequence Duplication Levels

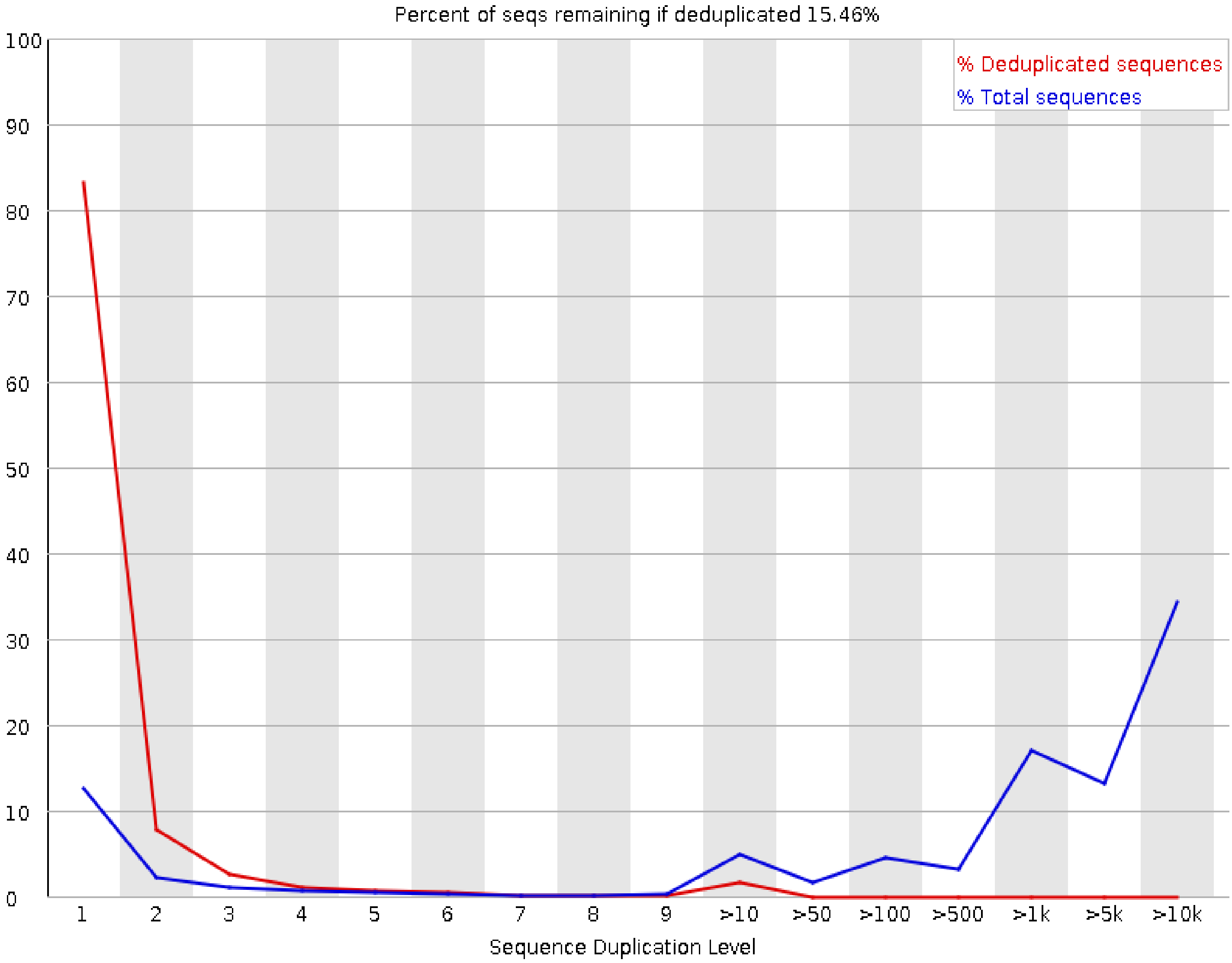


## Overrepresented sequences

| Sequence | Count | Percentage | Possible Source |
|---|---|---|---|
| CCGGTATTTAGCCTTAGATGGAGTTTACCACCCGCTTTGGGCTGCATTCC | 169510 | 0.250592942580717 | No Hit |
| CGCGTAACTAGTTAGCATGCCAGAGTCTCGTTCGTTATCGGAATTAACCA | 142843 | 0.21117012386913672 | No Hit |
| CTTCCGTACGCCACATGTCCCGCGCCCCGCCGCGGGCGGGGATTCGGCG | 140071 | 0.20707217308845266 | No Hit |
| GATCGGAAGAGCACACGTCTGAACTCCAGTCACATCACGATCTCGTATGC | 117747 | 0.17406977293405515 | TruSeq Adapter, Index 1 (100% over 50bp) |
| CTCTCTTCAAAGTTCTTTTCAACTTTCCCTTACGGTACTTGTTGACTATC | 114295 | 0.16896655284209222 | No Hit |
| CCTCACCCGGCCCGGACACGGACAGGATTGACAGATTGATAGCTCTTTCT | 102213 | 0.15110528252022198 | No Hit |
| CGCAGTTTTATCCGGTAAAGCGAATGATTAGAGGTCTTGGGGCCGAAACG | 100193 | 0.14811904133083464 | No Hit |
| GTCGGCATGTATTAGCTCTAGAATTACCACAGTTATCCAAGTAGGAGAGG | 94016 | 0.13898735230764375 | No Hit |

| Sequence | Count | Percentage | Possible Source |
|---|---|---|---|
| CTTGAACTCTCTCTTCAAAGTTCTTTTCAACTTTCCCTTACGGTACTTGT | 91230 | 0.13486870480584517 | No Hit |
| GTCAAAGTGAAGAAATTCAATGAAGCGCGGGTAAACGGCGGGAGTAACTA | 88511 | 0.13084910589 7952 | No Hit |
| CTGGATAGTAGGTAGGGACAGTGGGAATCTCGTTCATCCATTCATGCGCG | 85561 | 0.12648801109166852 | No Hit |
| CTGCCAGTAGCATATGCTTGTCTCAAAGATTAAGCCATGCATGTCTAAGT | 78784 | 0.11646931973499622 | No Hit |
| GCCCTCTTGAACTCTCTCTTCAAAGTTCTTTTCAACTTTCCCTTACGGTA | 77816 | 0.11503828930364626 | No Hit |
| GCGGGTCTTCCGTACGCCACATGTCCCGCGCCCCGCCGCGGGGCGGGGAT | 77225 | 0.11416459200516708 | No Hit |
| GCTGAATTTAAGCATATTAGTCAGCGGAGGAGAAGAAACTAACCAGGATT | 77131 | 0.11402562830625501 | No Hit |
| CGCGATGTGATTTCTGCCCAGTGCTCTGAATGTCAAAGTGAAGAAATTCA | 74504 | 0.11014203642282898 | No Hit |
| GGCGGGAGTAACTATGACTCTCTTAAGGTAGCCAAATGCCTCGTCATCTA | 73477 | 0.10862378409535335 | No Hit |
| CGGGTCTTCCGTACGCCACATGTCCCGCGCCCCGCCGCGGGGCGGGGATT | 72576 | 0.10729180225790878 | No Hit |
| GCTGGATAGTAGGTAGGGACAGTGGGAATCTCGTTCATCCATTCATGCGC | 72217 | 0.10676107919504242 | No Hit |
| CCCGTCGGCATGTATTAGCTCTAGAATTACCACAGTTATCCAAGTAGGAG | 72177 | 0.10670194570614366 | No Hit |
| CACCCGTTTACCTCTTAACGGTTTCACGCCCTCTTGAACTCTCTCTTCAA | 68493 | 0.10125575137856795 | No Hit |

## Adapter Content

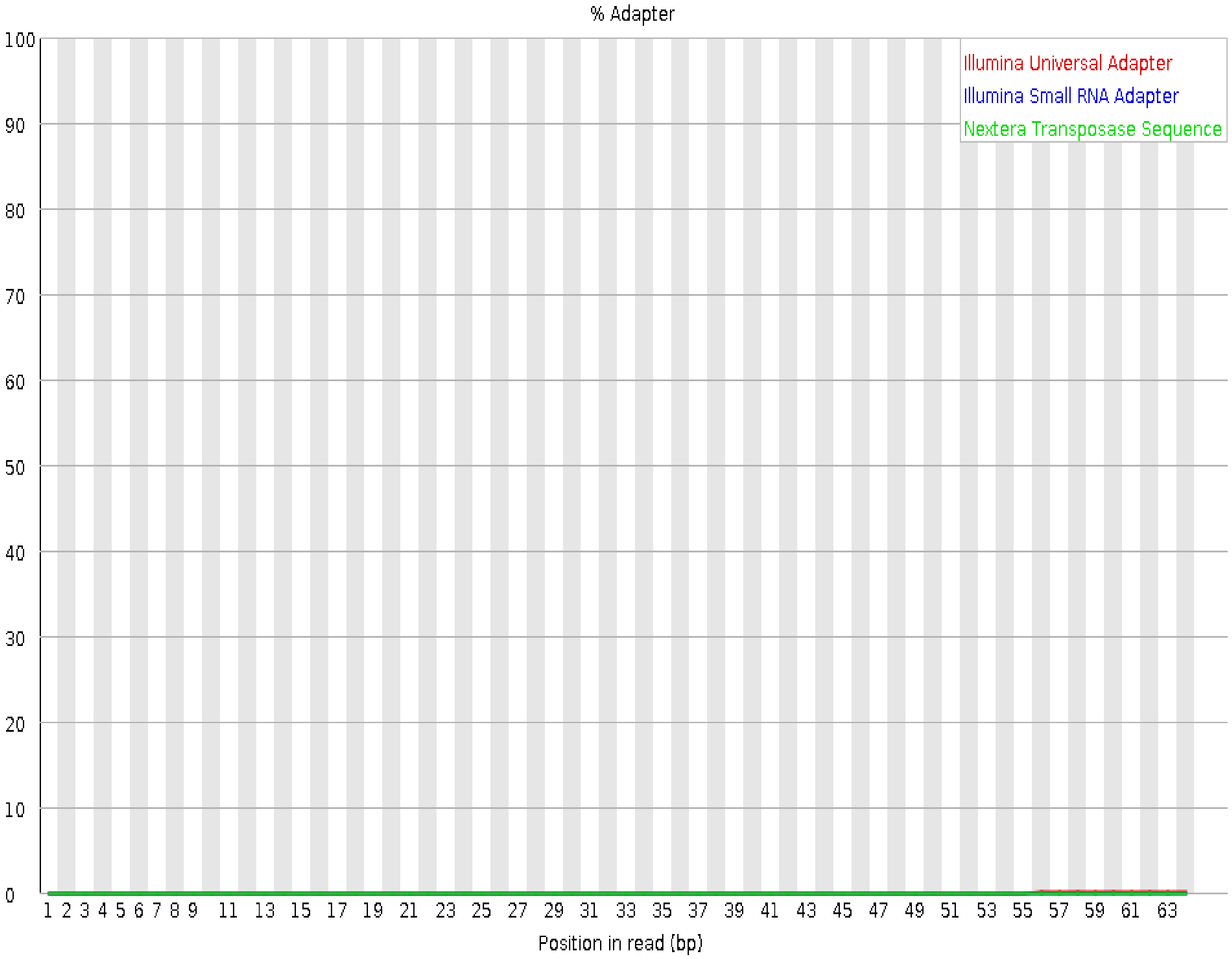

## Kmer Content

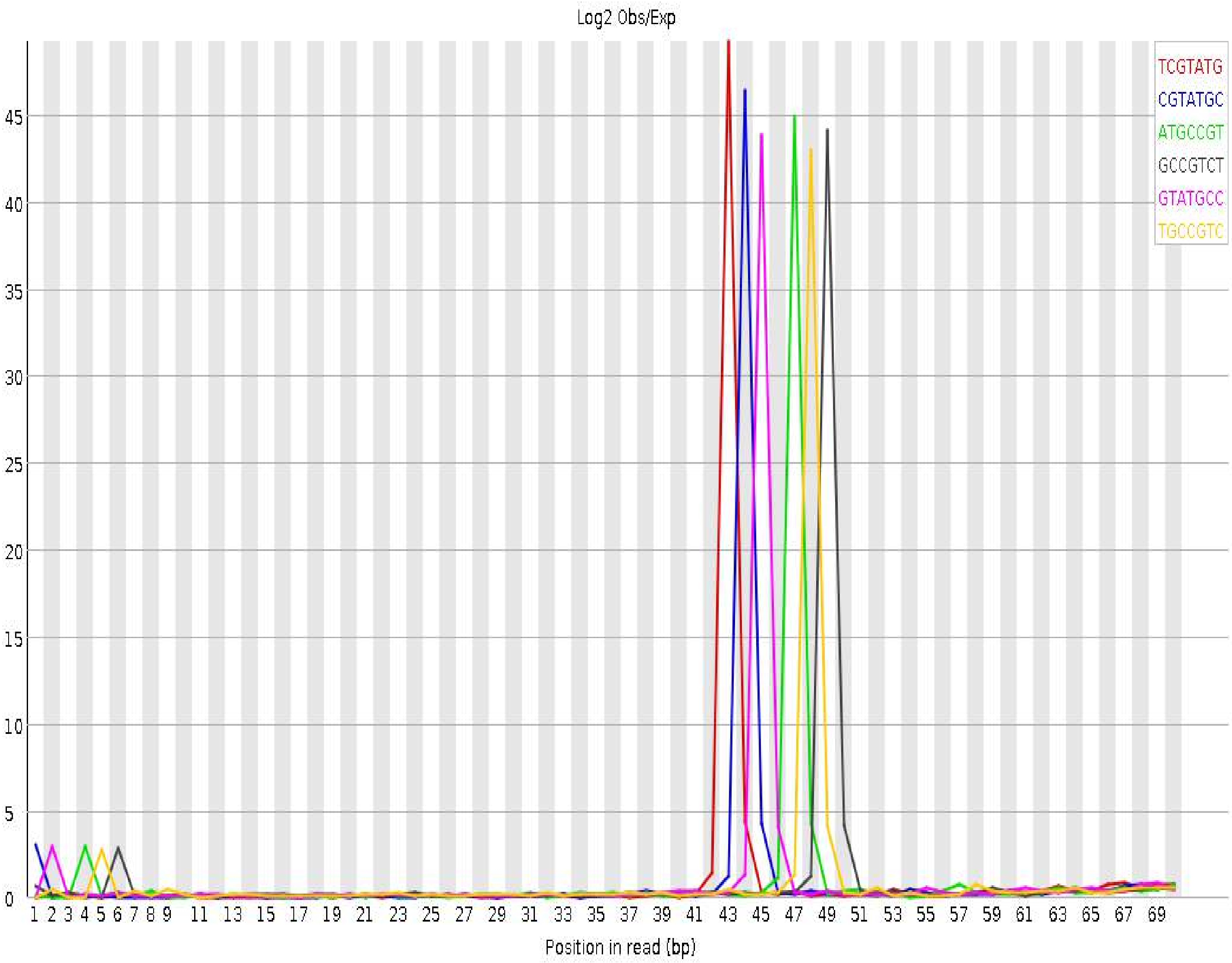


| Sequence | Count | PValue | Obs/Exp Max | Max Obs/Exp Position |
|---|---|---|---|---|
| TCGTATG | 21470 | 0.0 | 49.195374 | 43 |
| CGTATGC | 22695 | 0.0 | 46.38727 | 44 |
| ATGCCGT | 23100 | 0.0 | 44.878803 | 47 |
| GCCGTCT | 23590 | 0.0 | 44.11356 | 49 |
| GTATGCC | 23965 | 0.0 | 43.870804 | 45 |
| TGCCGTC | 24235 | 0.0 | 42.952927 | 48 |
| CGCGTAA | 28280 | 0.0 | 41.73094 | 1 |
| CTCGTAT | 24720 | 0.0 | 40.873283 | 42 |
| GATCTCG | 23820 | 0.0 | 40.507786 | 39 |
| TCTCGTA | 25900 | 0.0 | 37.390636 | 41 |
| GTCACAT | 28005 | 0.0 | 37.237633 | 29 |
| | | | | |

| Sequence | Count | PValue | Obs/Exp Max | Max Obs/Exp Position |
|---|---|---|---|---|
| CATCACG | 28165 | 0.0 | 36.817486 | 33 |
| [illegible] | [illegible] | [illegible] | 35.589935 | [illegible] |
| GCGTAAC | 33730 | 0.0 | 35.522415 | 2 |
| AGTCACA | 30490 | 0.0 | 34.36294 | 28 |
| CAGTCAC | 31055 | 0.0 | 33.565353 | 27 |
| ACACGTC | 31890 | 0.0 | 33.188435 | 13 |
| CACGTCT | 32930 | 0.0 | 31.983425 | 14 |
| CACACGT | 33005 | 0.0 | 31.84467 | 12 |
| TGCTTGA | 33140 | 0.0 | 31.18883 | 58 |

**Produced by FastQC (version 0.11.2)**

# FastQC Report

Mon 4 May 2015
Agilent_Adult_Heart_ATCACG_L001_R2.fastq.gz

## Summary


- Basic Statistics
- Per base sequence quality
- Per tile sequence quality
- Per sequence quality scores
- Per base sequence content
- Per sequence GC content
- Per base N content
- Sequence Length Distribution
- Sequence Duplication Levels
- Overrepresented sequences
- Adapter Content
- Kmer Content


## Basic Statistics

| Measure | Value |
| --- | --- |
| Filename | Agilent_Adult_Heart_ATCACG_L001_R2.fastq.gz |
| File type | Conventional base calls |
| Encoding | Sanger / Illumina 1.9 |
| Total Sequences | 67643565 |
| Sequences flagged as poor quality | 0 |
| Sequence length | 76 |
| %GC | 54 |

## Per base sequence quality

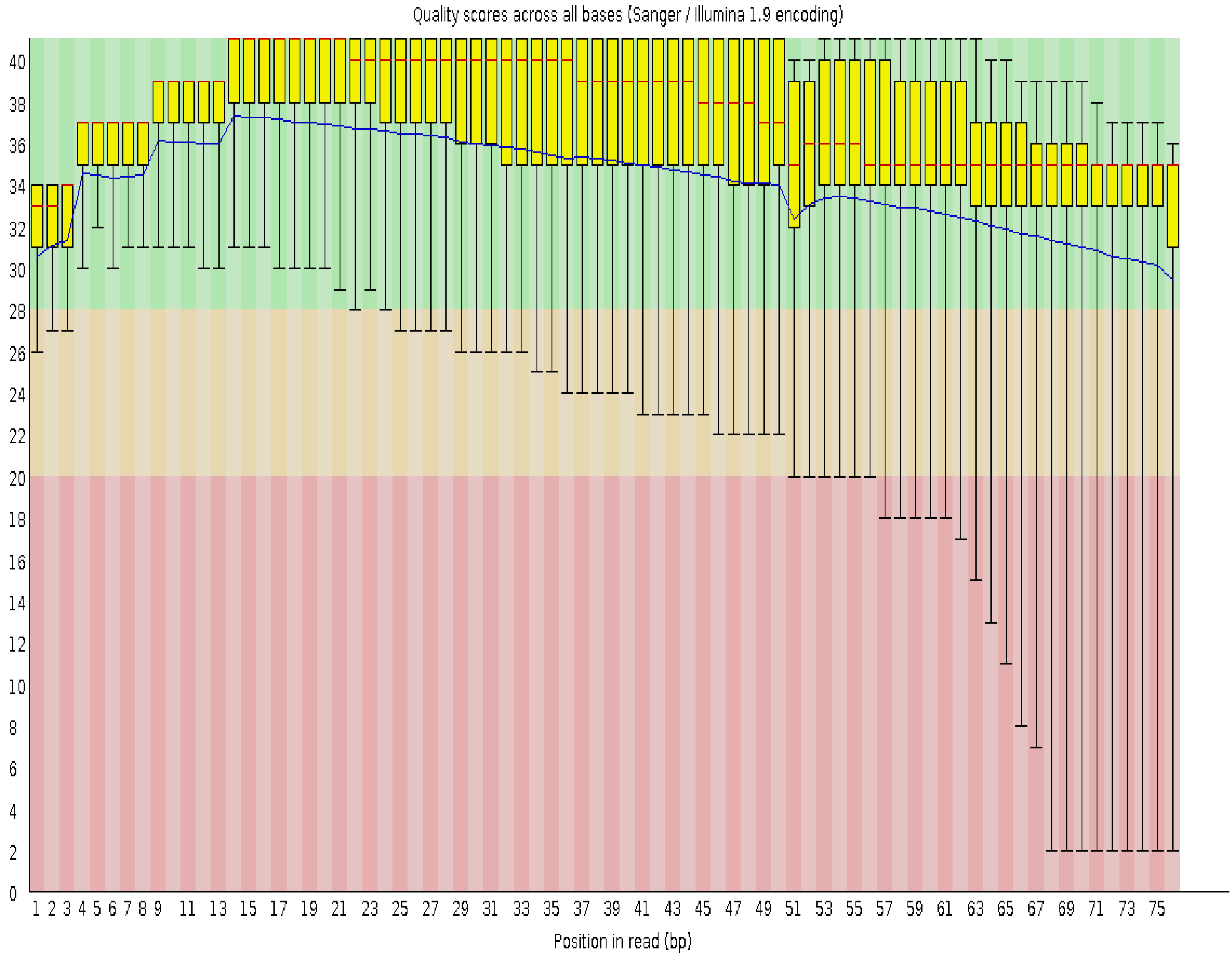


## Per tile sequence quality

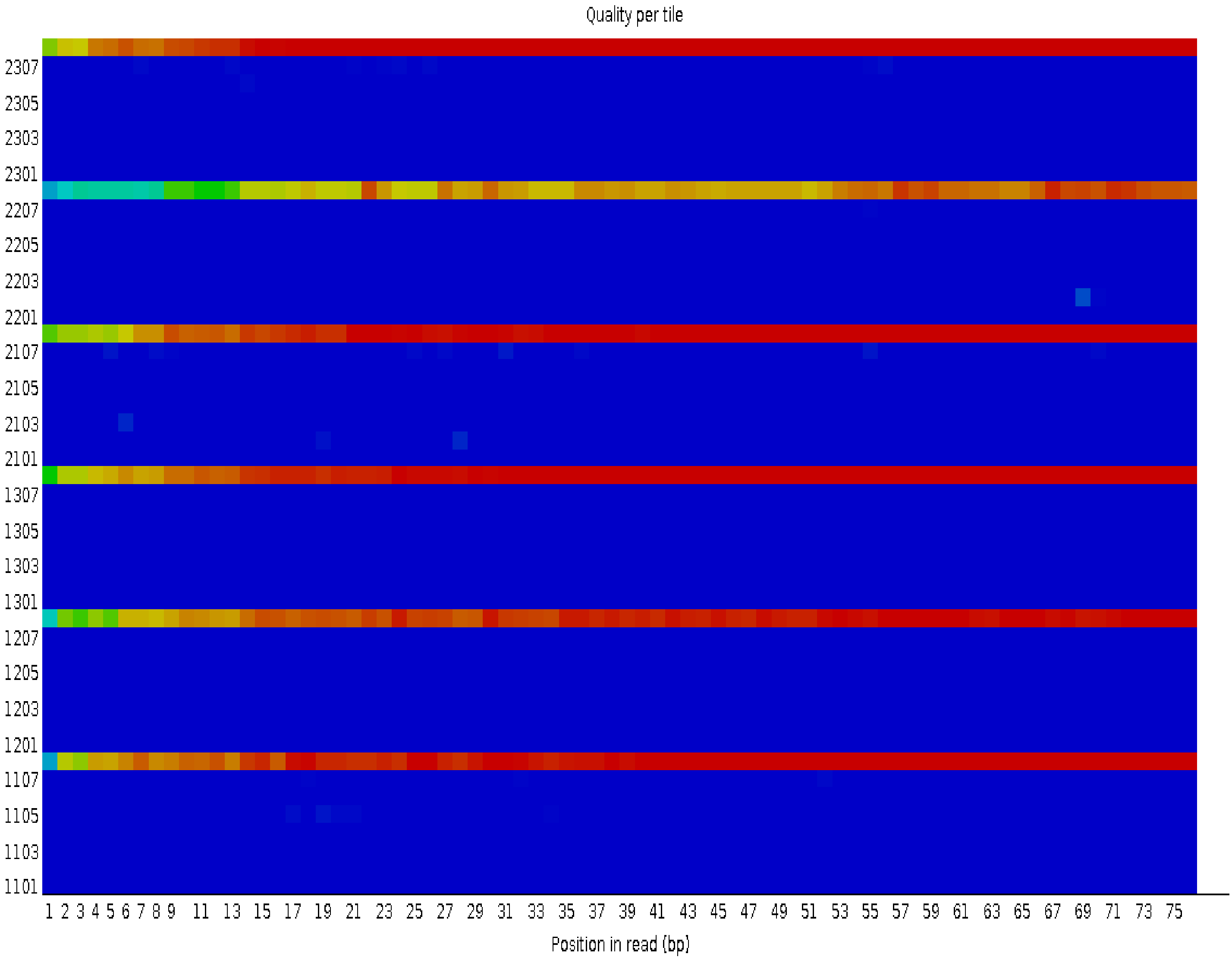


# Per sequence quality scores

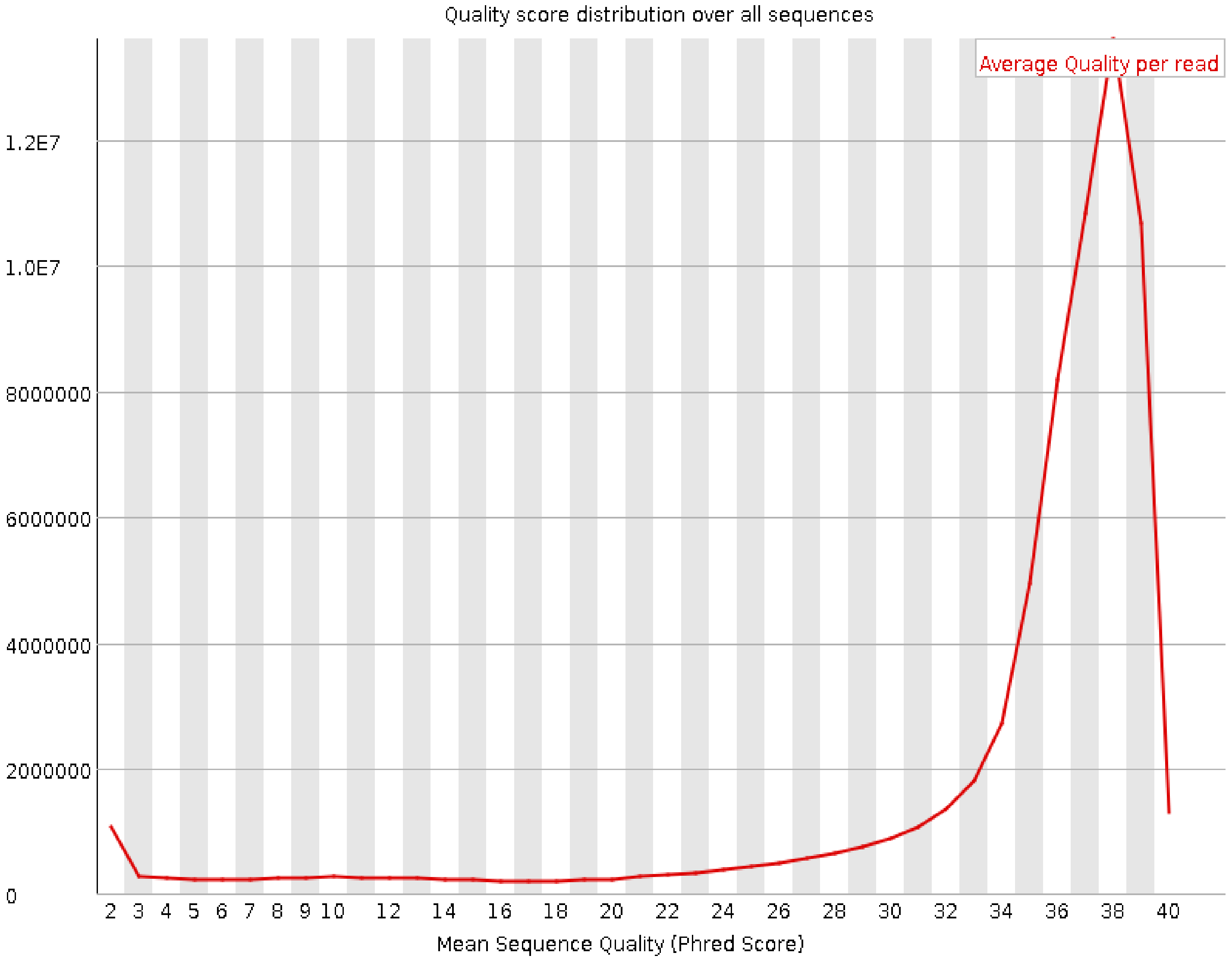


## Per base sequence content

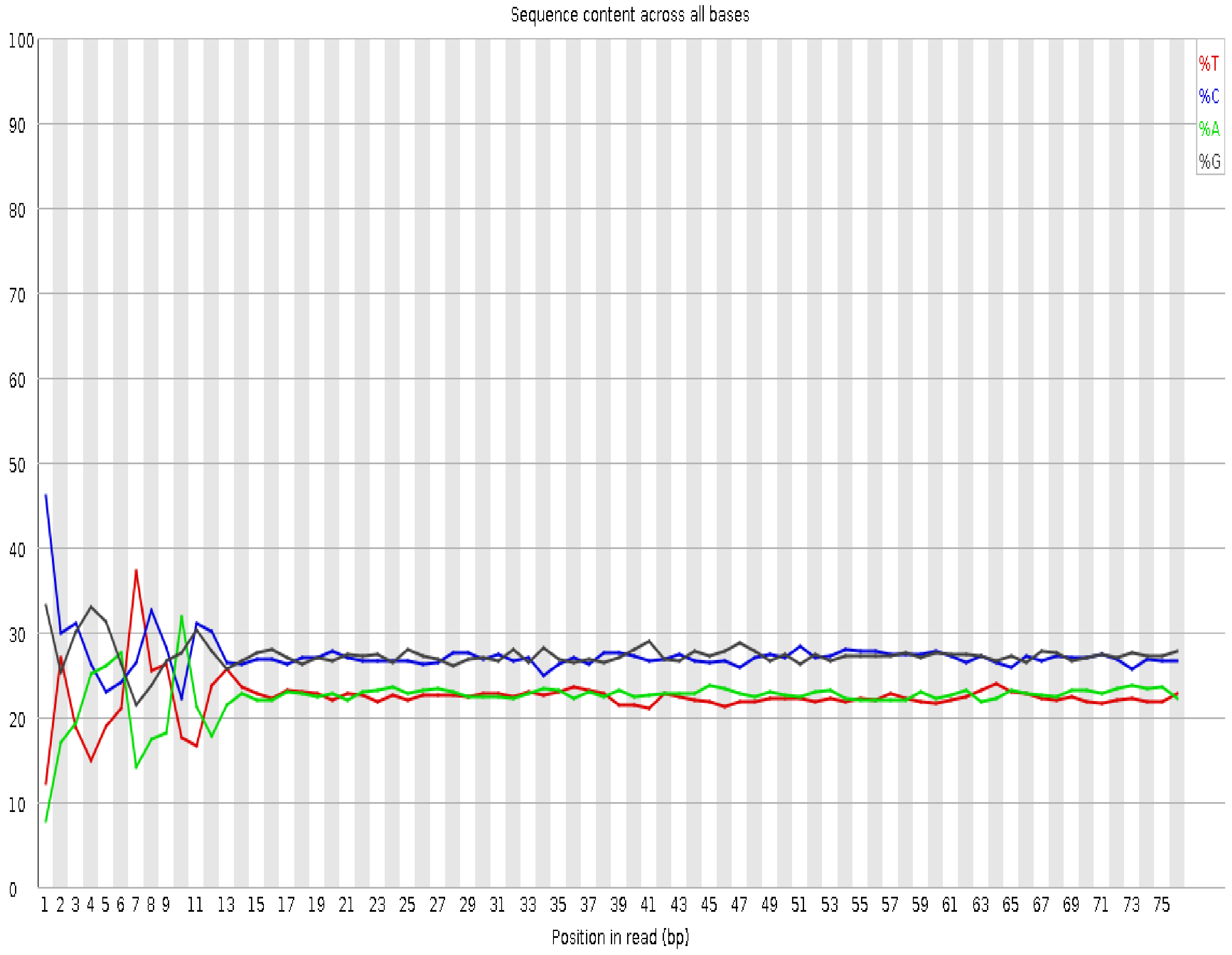


## Per sequence GC content

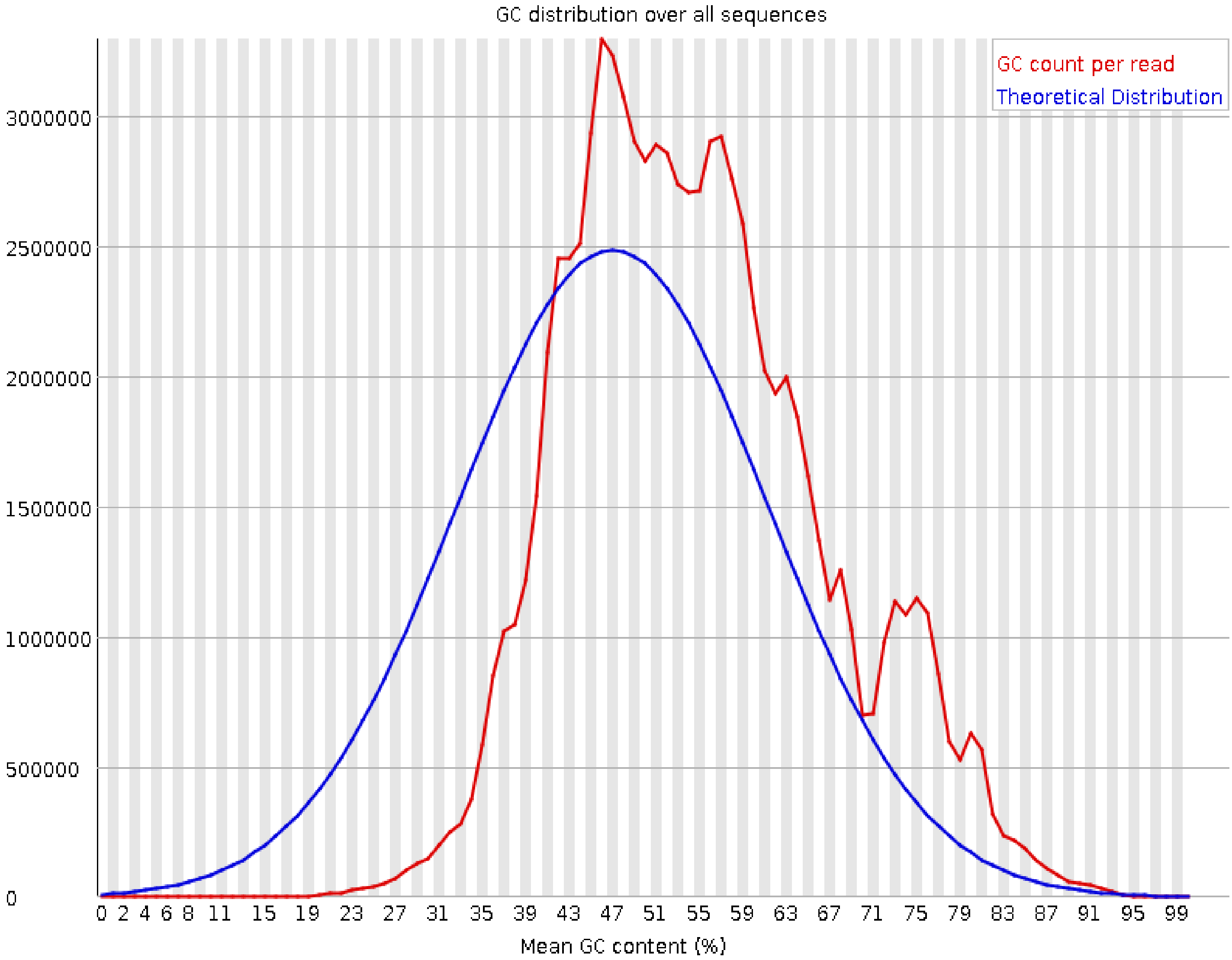


## Per base N content

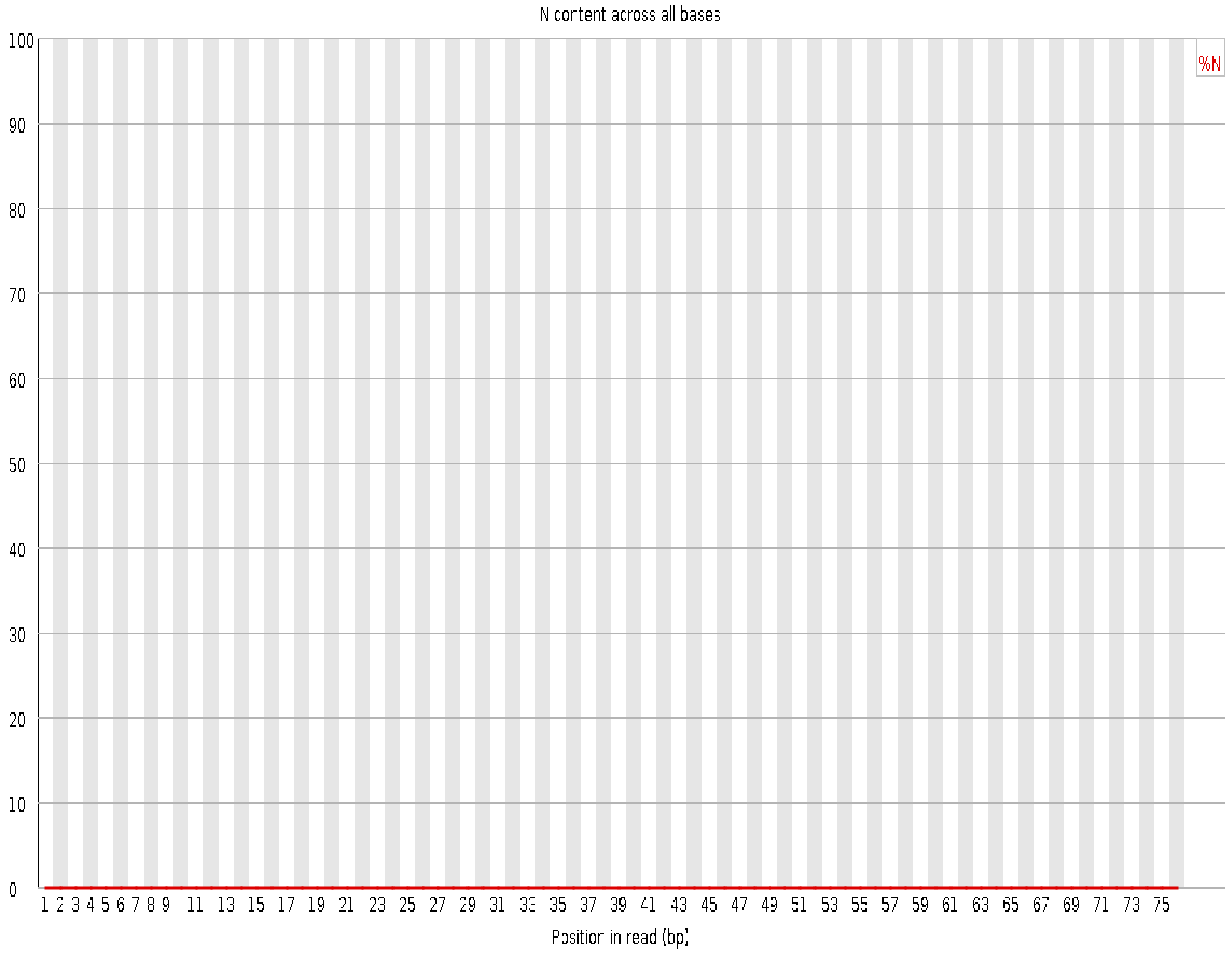


## Sequence Length Distribution

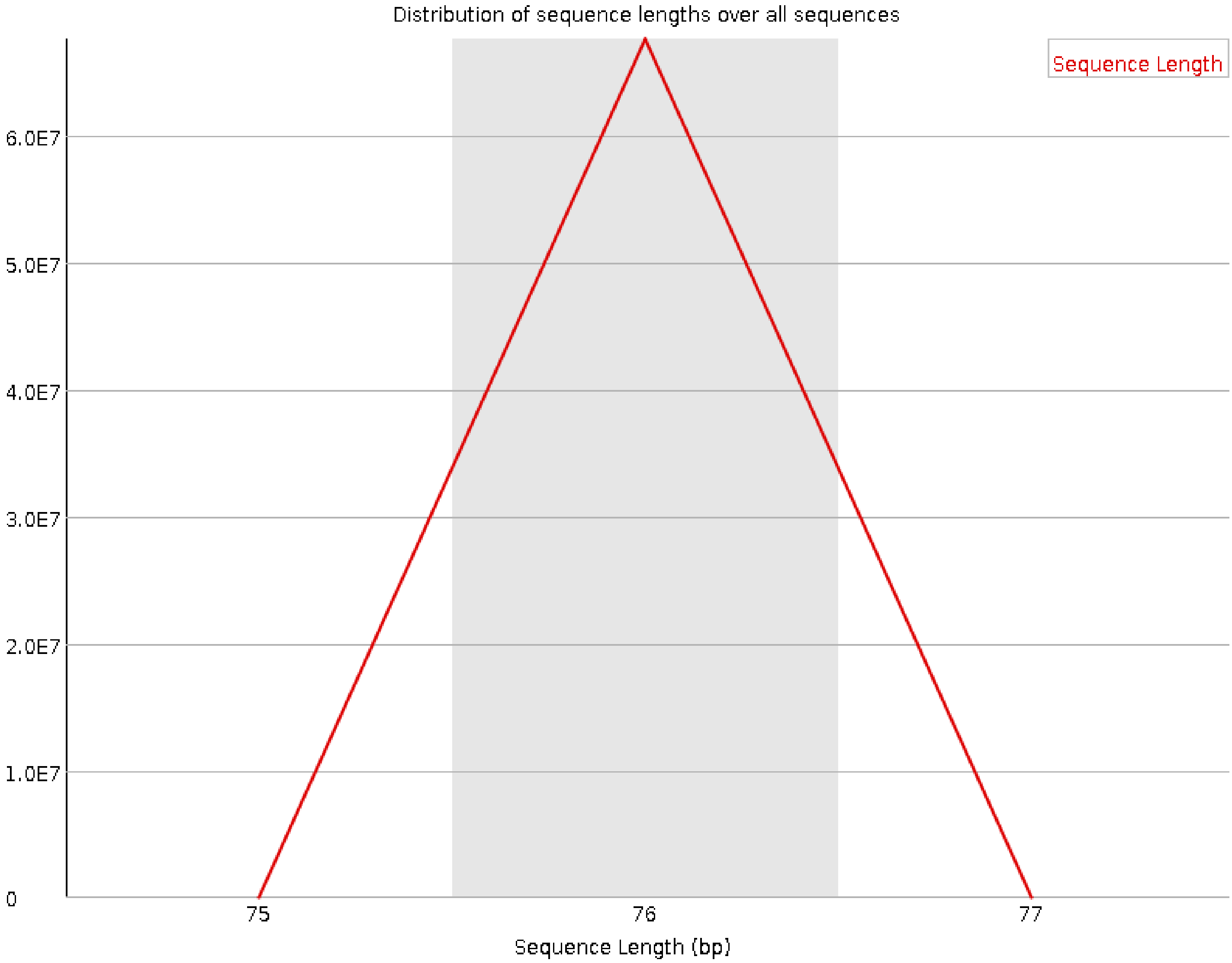


## Sequence Duplication Levels

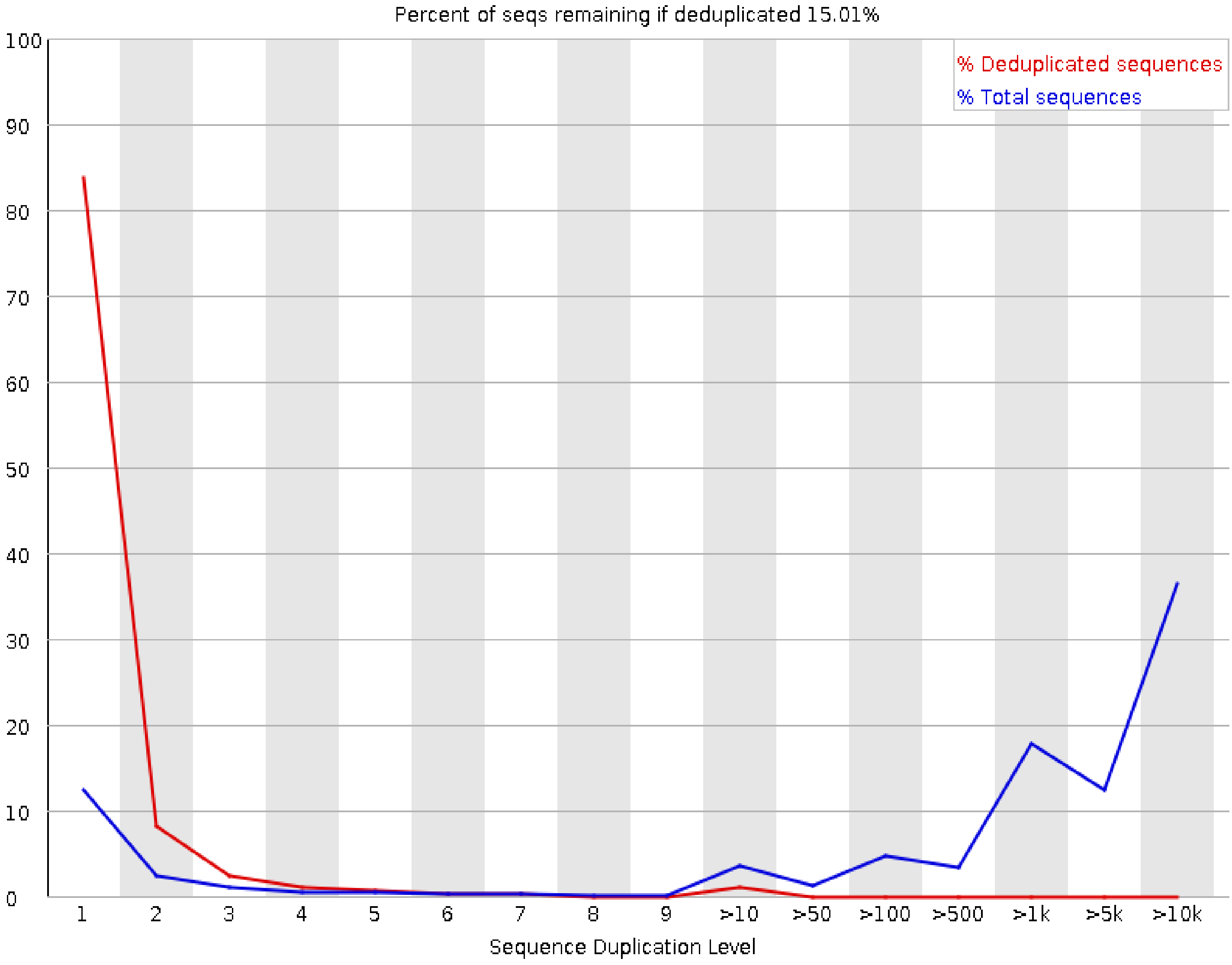


## Overrepresented sequences

| Sequence | Count | Percentage | Possible Source |
|---|---|---|---|
| CCGGTATTTAGCCTTAGATGGAGTTTACCACCCGCTTTGGGCTGCATTCC | 172407 | 0.25487568551420964 | No Hit |
| CTTCCGTACGCCACATGTCCCGCGCCCCGCCGCGGGGCGGGGATTCGGCG | 158249 | 0.23394538711849383 | No Hit |
| CGCGTAACTAGTTAGCATGCCAGAGTCTCGTTCGTTATCGGAATTAACCA | 148648 | 0.21975187144556915 | No Hit |
| CTCTCTTCAAAGTTCTTTTCAACTTTCCCTTACGGTACTTGTTGACTATC | 114651 | 0.1694928408932912 | No Hit |
| CCTCACCCGGCCCGGACACGGACAGGATTGACAGATTGATAGCTCTTTCT | 110518 | 0.1633828731528269 | No Hit |
| CGCAGTTTTATCCGGTAAAGCGAATGATTAGAGGTCTTGGGGCCGAAACG | 109157 | 0.1613708561930466 | No Hit |
| GTCGGCATGTATTAGCTCTAGAATTACCACAGTTATCCAAGTAGGAGAGG | 100724 | 0.14890403839596567 | No Hit |
| CTTGAACTCTCTCTTCAAAGTTCTTTTCAACTTTCCCTTACGGTACTTGT | 93657 | 0.1384566292447774 | No Hit |
| CTGGATAGTAGGTAGGGACAGTGGGAATCTCGTTCATCCATTCATGCGCG | 90559 | 0.1338767405295685 | No Hit |
| CGGGTCTTCCGTACGCCACATGTCCCGCGCCCCGCCGCGGGGCGGGGATT | 89458 | 0.13224909124763012 | No Hit |
| GCGGGTCTTCCGTACGCCACATGTCCCGCGCCCCGCCGCGGGGCGGGGAT | 87448 | 0.1292776334304675 | No Hit |

| Sequence | Count | Percentage | Possible Source |
|---|---|---|---|
| GTCAAAGTGAAGAAATTCAATGAAGCGCGGGTAAACGGCGGGAGTAACTA | 86556 | 0.12795895662802514 | No Hit |
| GCCCTCTTGAACTCTCTCTTCAAAGTTCTTTTCAACTTTCCCTTACGGTA | 84957 | 0.12559509540929725 | No Hit |
| CTGCCAGTAGCATATGCTTGTCTCAAAGATTAAGCCATGCATGTCTAAGT | 84244 | 0.12454104096967687 | No Hit |
| CCCGTCGGCATGTATTAGCTCTAGAATTACCACAGTTATCCAAGTAGGAG | 81977 | 0.12118965048633969 | No Hit |
| CGCGATGTGATTTCTGCCCAGTGCTCTGAATGTCAAAGTGAAGAAATTCA | 77970 | 0.11526595323590647 | No Hit |
| GGCGGGAGTAACTATGACTCTCTTAAGGTAGCCAAATGCCTCGTCATCTA | 76850 | 0.11361021554674121 | No Hit |
| CACCCGTTTACCTCTTAACGGTTTCACGCCCTCTTGAACTCTCTCTTCAA | 76426 | 0.11298340056441437 | No Hit |
| GCTGGATAGTAGGTAGGGACAGTGGGAATCTCGTTCATCCATTCATGCGC | 76358 | 0.11288287363328649 | No Hit |
| CTTCACCGTGCCAGACTAGAGTCAAGCTCAACAGGGTCTTCTTTCCCCGC | 76066 | 0.11245119916432553 | No Hit |
| GCTGAATTTAAGCATATTAGTCAGCGGAGGAGAAGAAACTAACCAGGATT | 71691 | 0.10598347381602373 | No Hit |
| CTCGATCAGAAGGACTTGGGCCCCCCACGAGCGGCGCCGGGGAGCGGGTC | 68593 | 0.10140358510081485 | No Hit |

# Adapter Content

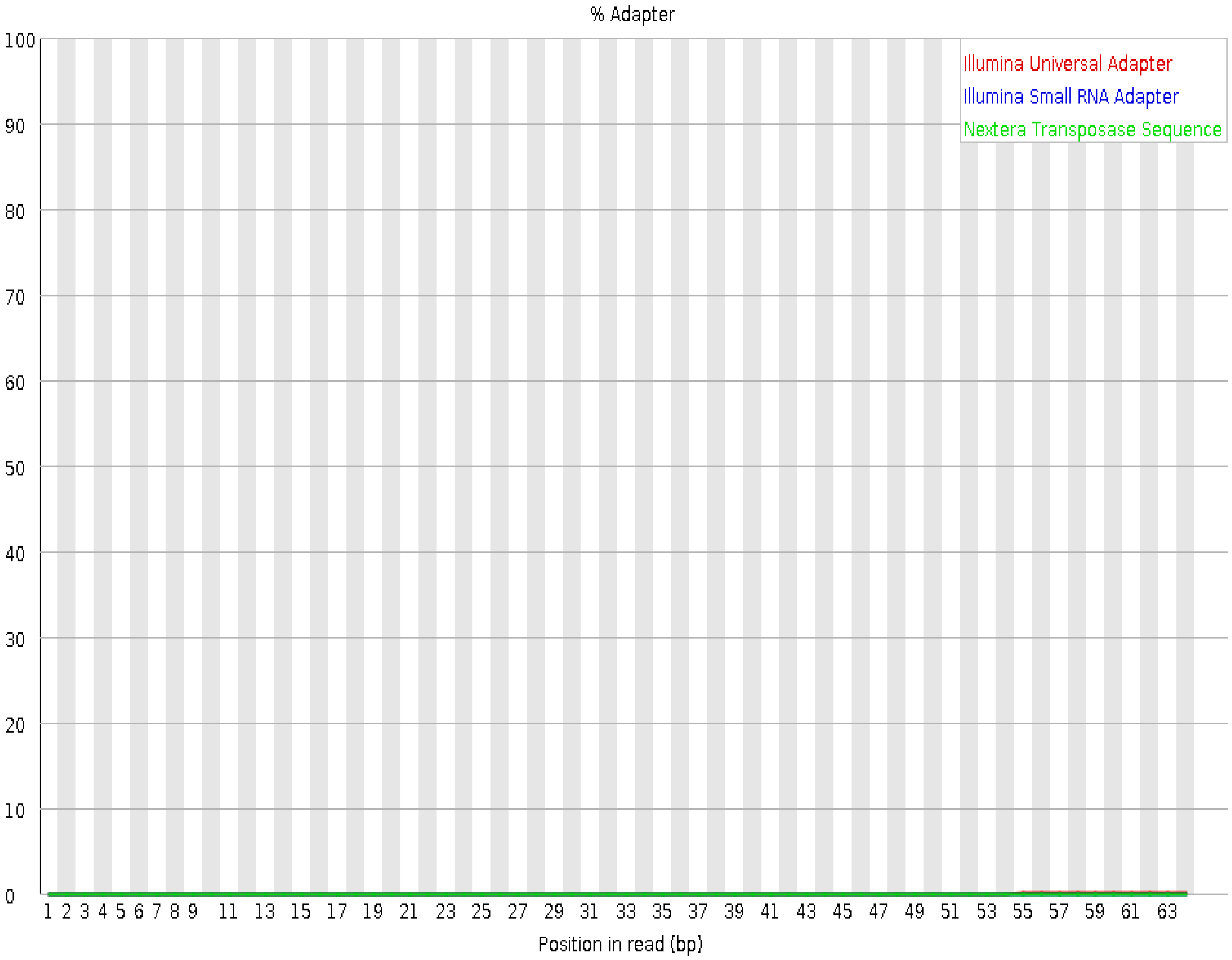

# Kmer Content

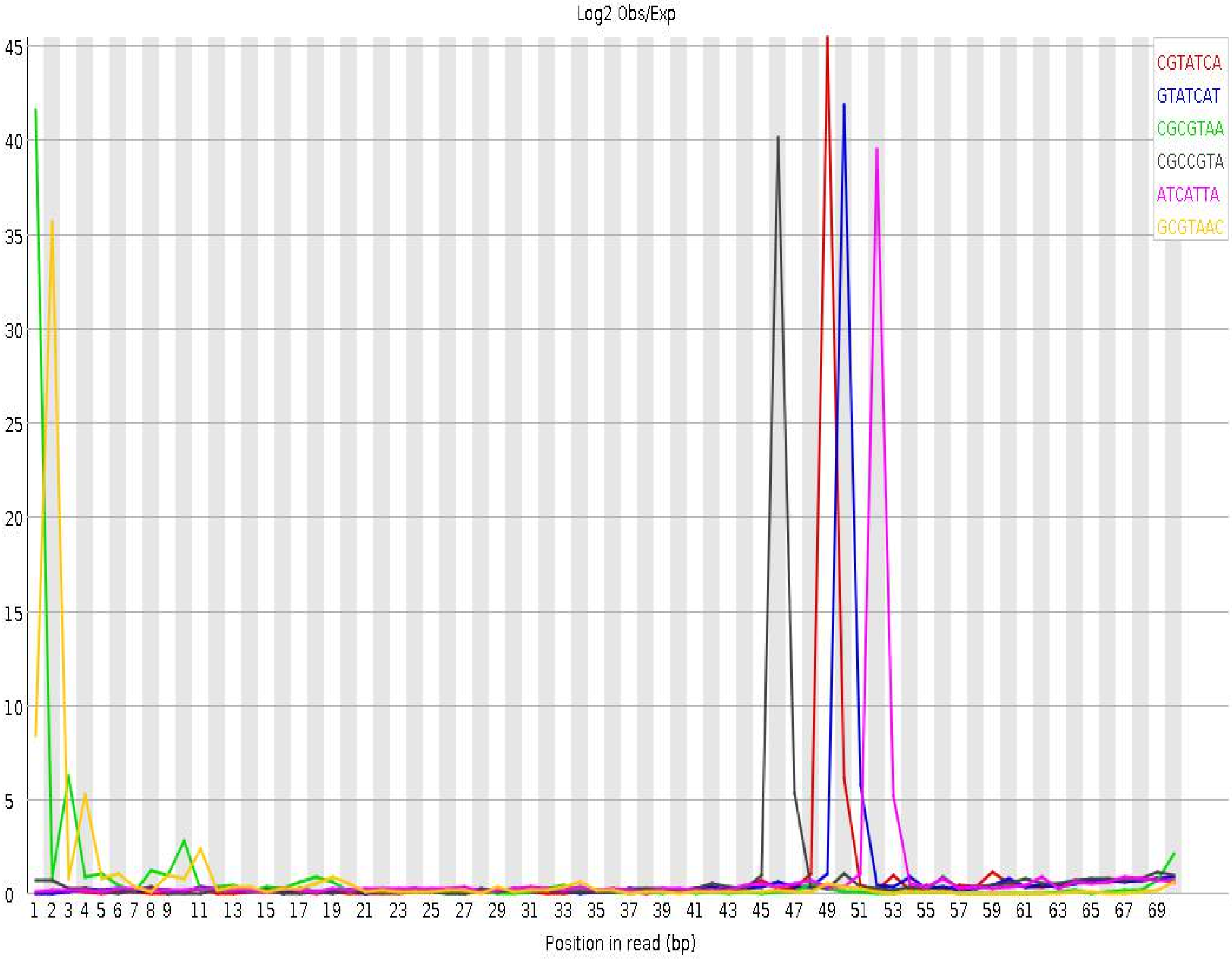


| Sequence | Count | PValue | Obs/Exp Max | Max Obs/Exp Position |
|---|---|---|---|---|
| CGTATCA | 15625 | 0.0 | 45.396896 | 49 |
| GTATCAT | 16995 | 0.0 | 41.861004 | 50 |
| CGCGTAA | 27435 | 0.0 | 41.588123 | 1 |
| CGCCGTA | 17910 | 0.0 | 40.151333 | 46 |
| ATCATTA | 19510 | 0.0 | 39.514286 | 52 |
| GCGTAAC | 32460 | 0.0 | 35.656853 | 2 |
| GATCTCG | 24875 | 0.0 | 32.369957 | 34 |
| GTAGATC | 22960 | 0.0 | 32.282112 | 31 |
| TAGATCT | 23530 | 0.0 | 31.454685 | 32 |
| GTGTCGA | 4700 | 0.0 | 31.207518 | 4 |
| AGTTAGC | 38055 | 0.0 | 31.080914 | 10 |
| TCTCGGT | 22090 | 0.0 | 31.048979 | 36 |
| TCGGTGG | 22745 | 0.0 | 30.986467 | 38 |
| | | | | |

| Sequence | Count | PValue | Obs/Exp Max | Max Obs/Exp Position |
|---|---|---|---|---|
| GCCGTAT | 23550 | 0.0 | 30.728256 | 47 |
| [illegible] | [illegible] | 0.0 | [illegible] | [illegible] |
| GTTAGCA | 38605 | 0.0 | 30.588167 | 11 |
| GTGGTCG | 20595 | 0.0 | 30.55177 | 41 |
| CTCGGTG | 22785 | 0.0 | 30.302235 | 37 |
| AGATCTC | 27320 | 0.0 | 30.03613 | 33 |
| CTATCGC | 10290 | 0.0 | 29.99633 | 63 |

**Produced by FastQC (version 0.11.2)**

# FastQC Report

Mon 4 May 2015
Agilent_Adult_Kidney_GCCAAT_L004_R1.fastq.gz

## Summary


- Basic Statistics
- Per base sequence quality
- Per tile sequence quality
- Per sequence quality scores
- Per base sequence content
- Per sequence GC content
- Per base N content
- Sequence Length Distribution
- Sequence Duplication Levels
- Overrepresented sequences
- Adapter Content
- Kmer Content


## Basic Statistics

| Measure | Value |
|---|---|
| Filename | Agilent_Adult_Kidney_GCCAAT_L004_R1.fastq.gz |
| File type | Conventional base calls |
| Encoding | Sanger / Illumina 1.9 |
| Total Sequences | 92807528 |
| Sequences flagged as poor quality | 0 |
| Sequence length | 76 |
| %GC | 53 |

## Per base sequence quality

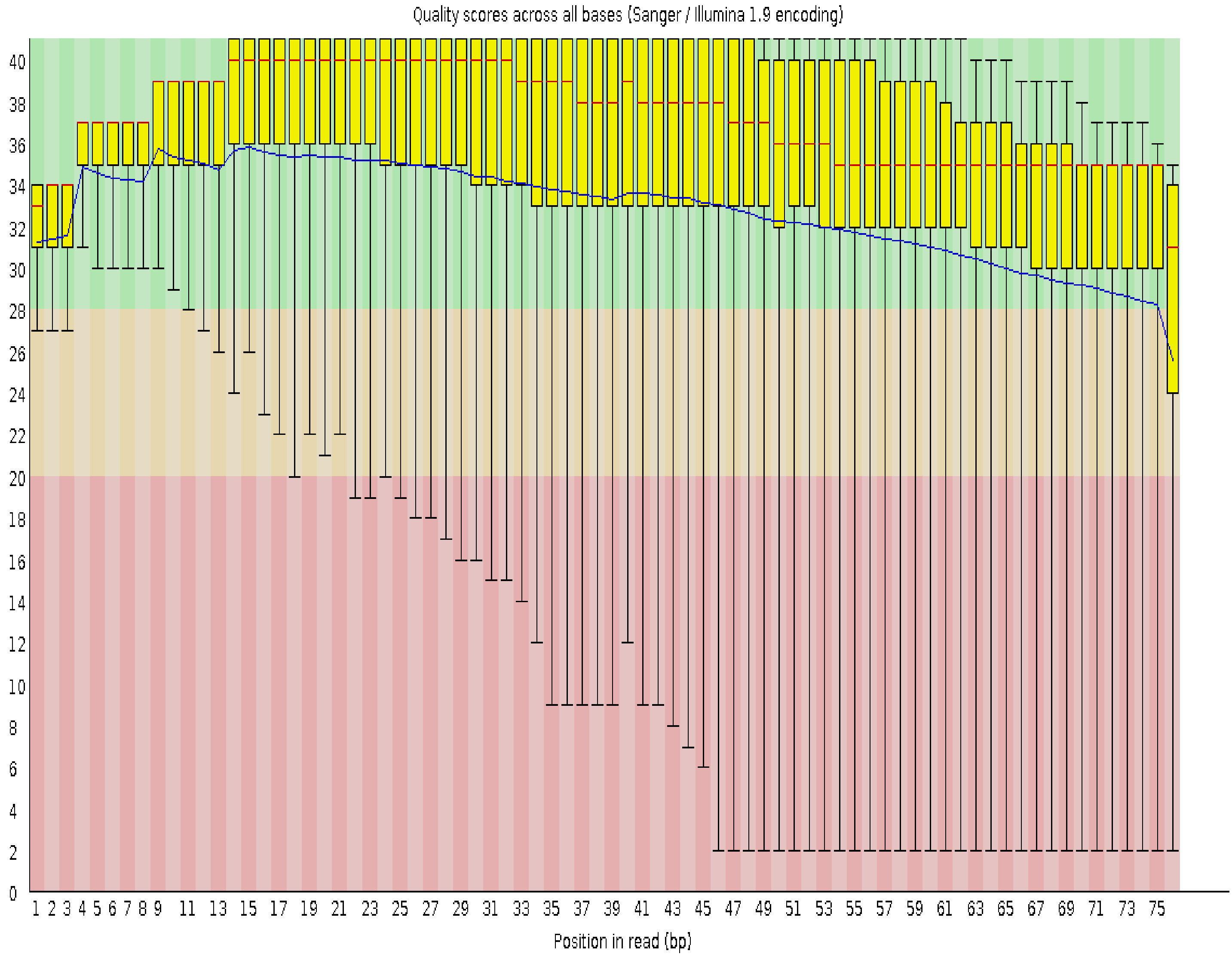


## Per tile sequence quality

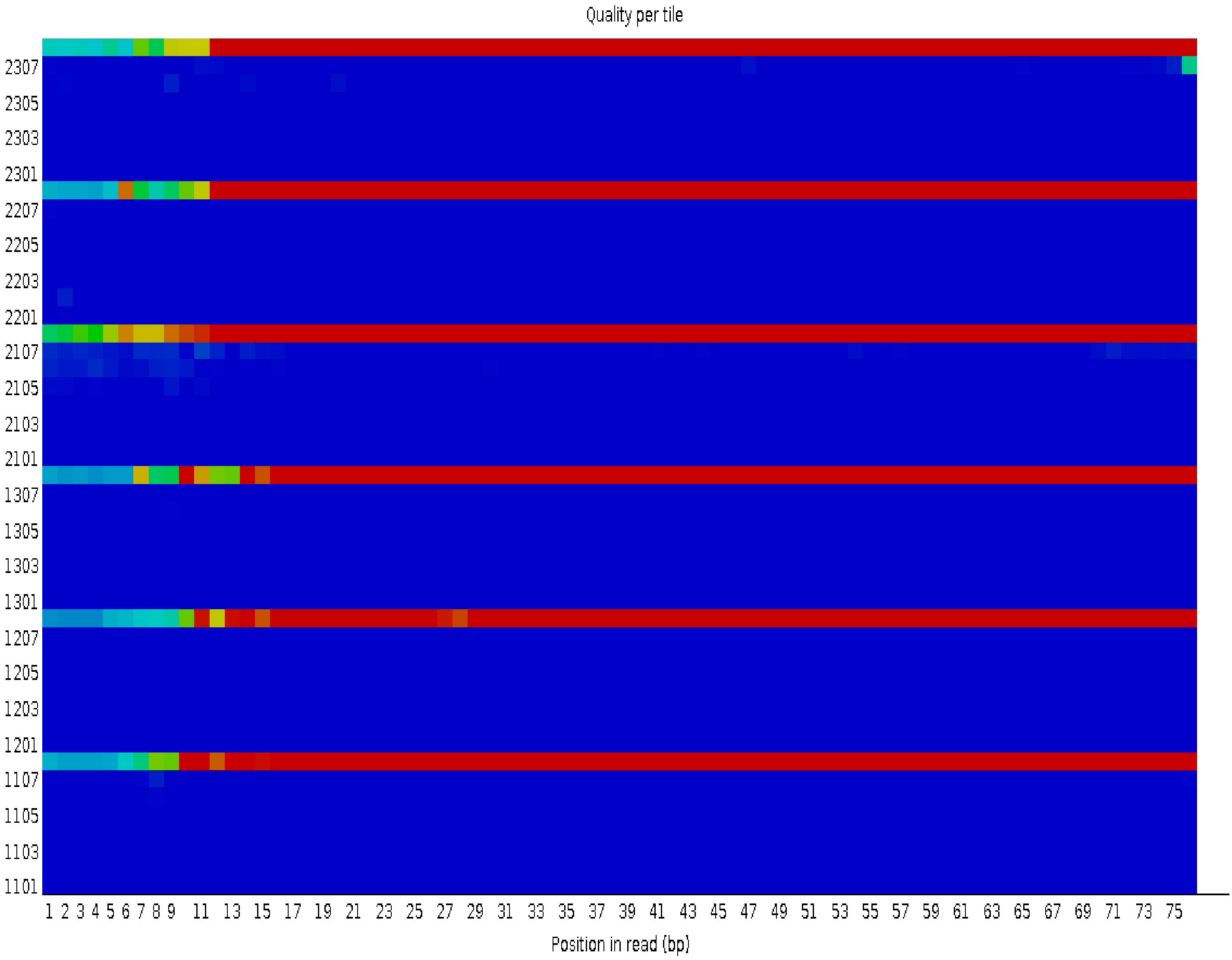


## Per sequence quality scores

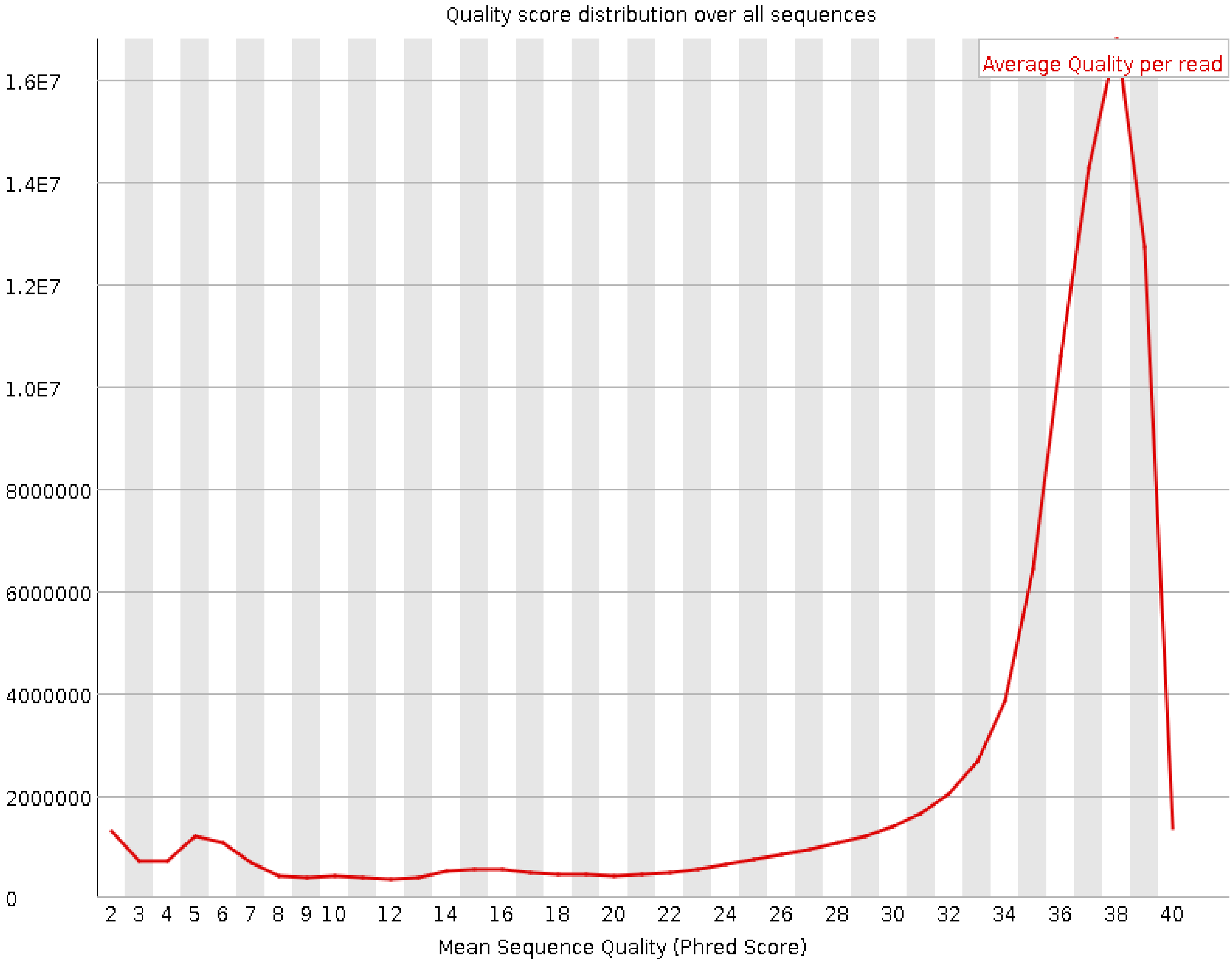


## Per base sequence content

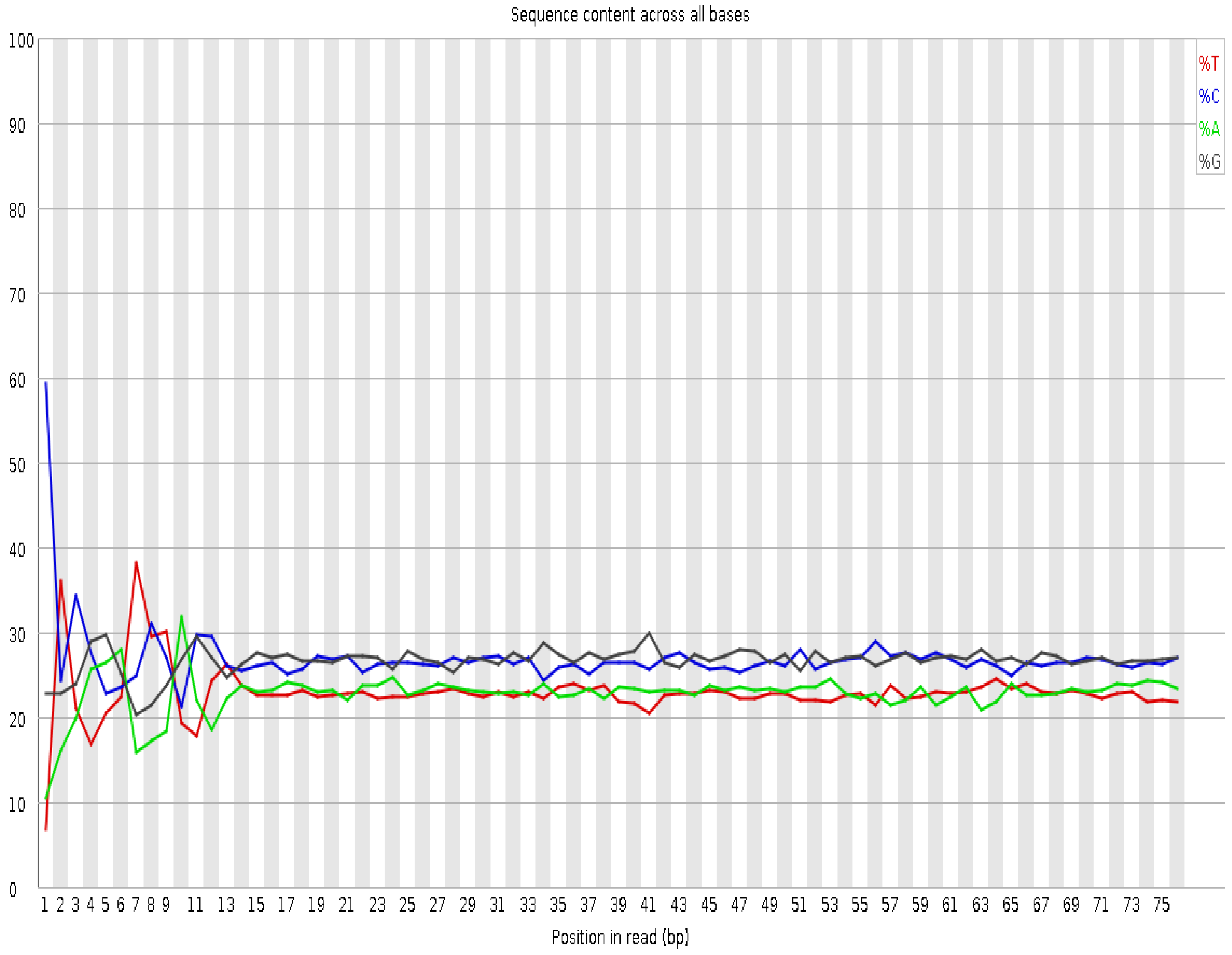


## Per sequence GC content

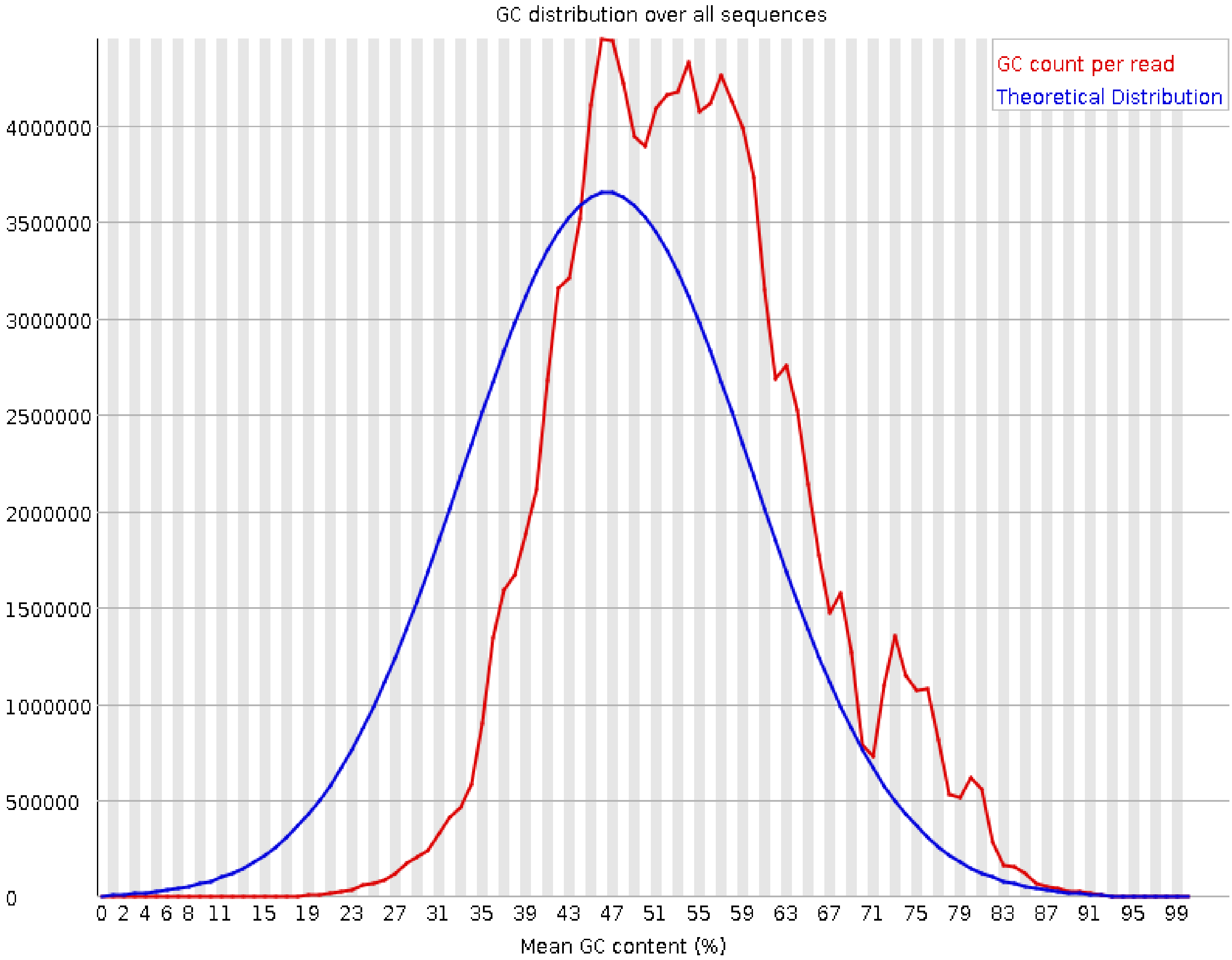


## Per base N content

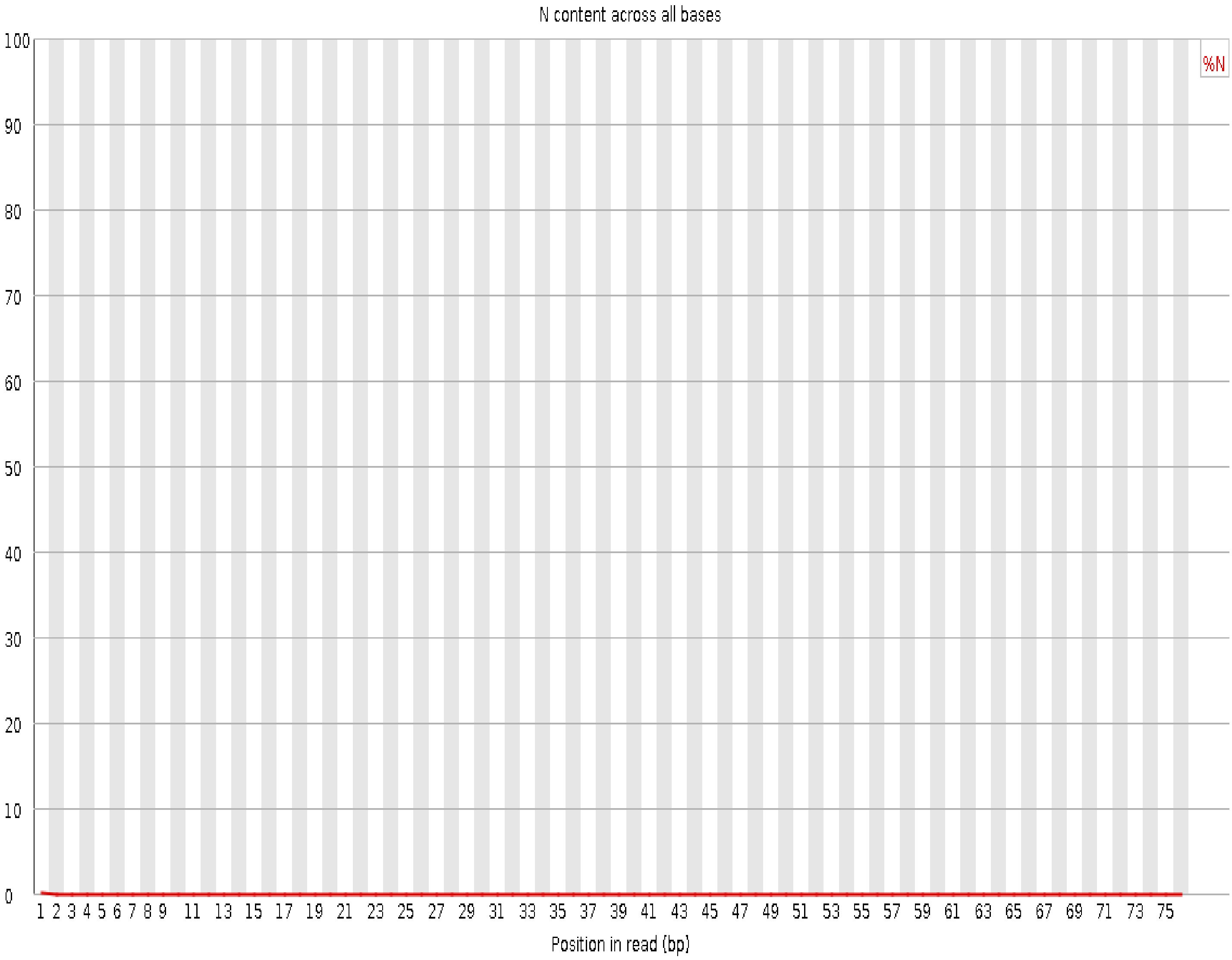


# Sequence Length Distribution

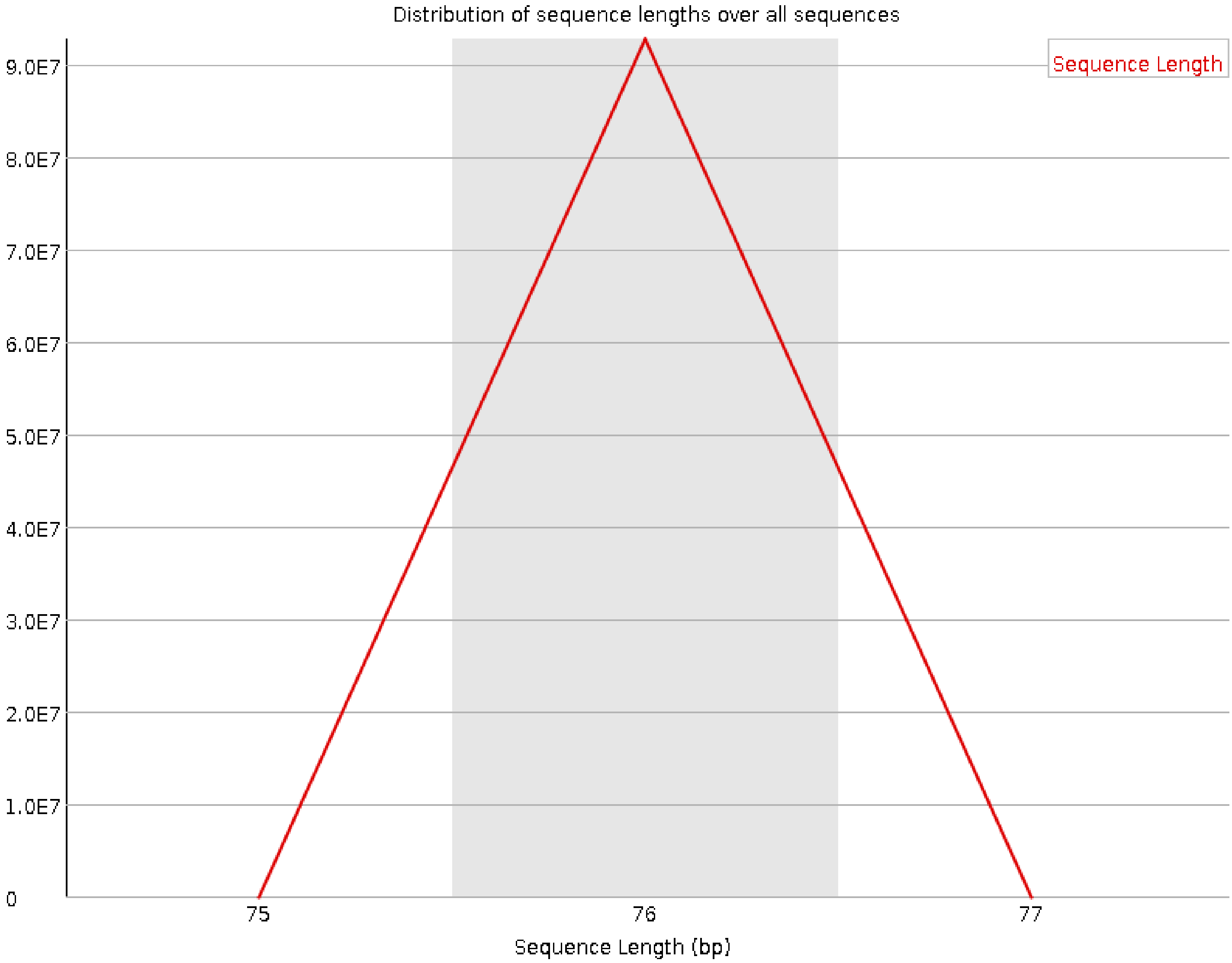


## Sequence Duplication Levels

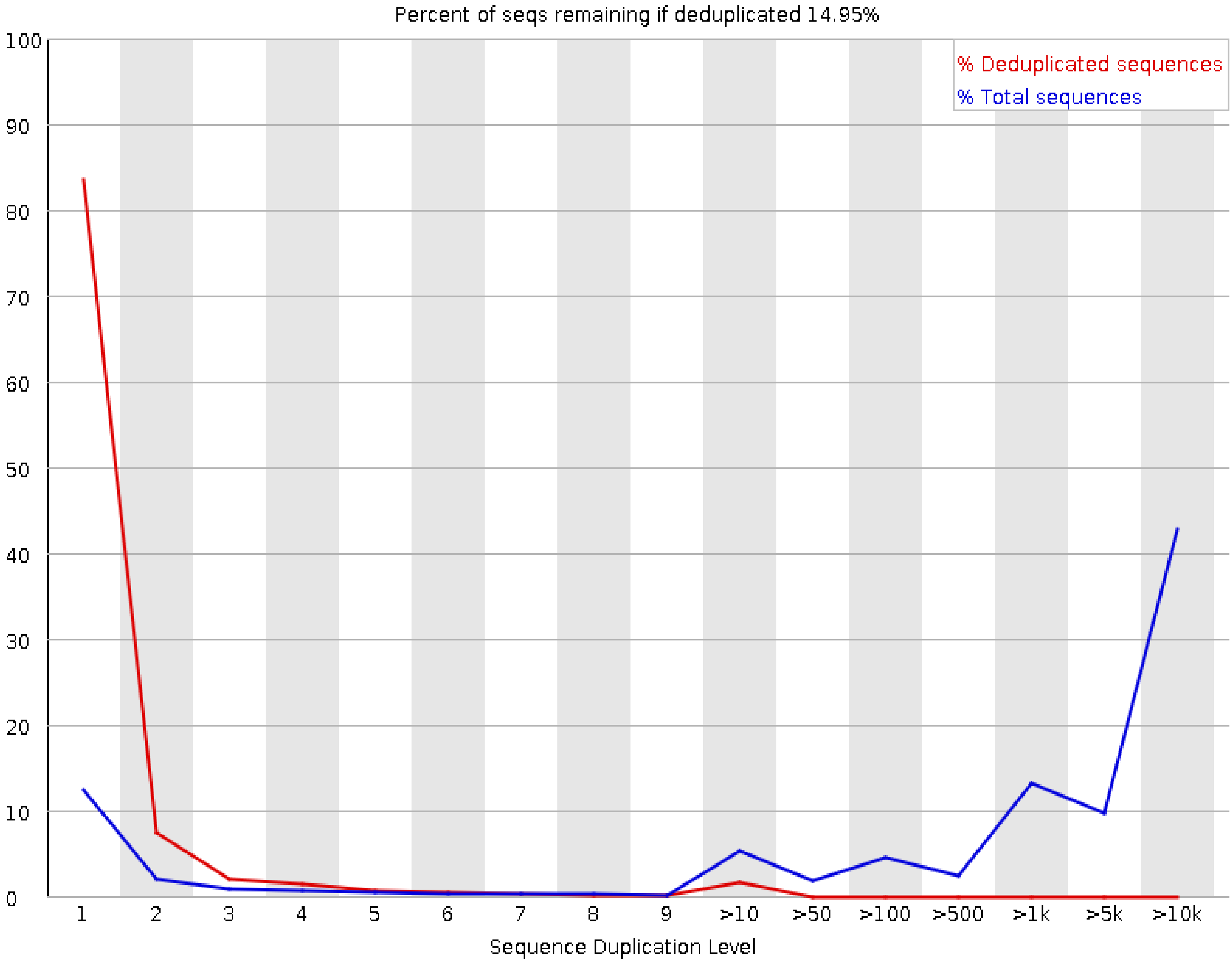


## Overrepresented sequences

| Sequence | Count | Percentage | Possible Source |
|---|---|---|---|
| CTTCCGTACGCCACATGTCCCGCGCCCCGCCGCGGGGCGGGGATTCGGCG | 391101 | 0.42141085796402206 | No Hit |
| CGCGTAACTAGTTAGCATGCCAGAGTCTCGTTCGTTATCGGAATTAACCA | 314035 | 0.3383723354855438 | No Hit |
| CCTCACCCGGCCCGGACACGGACAGGATTGACAGATTGATAGCTCTTTCT | 282802 | 0.3047188154822958 | No Hit |
| CGCAGTTTTATCCGGTAAAGCGAATGATTAGAGGTCTTGGGGCCGAAACG | 279360 | 0.301010064614586 | No Hit |
| CCGGTATTTAGCCTTAGATGGAGTTTACCACCCGCTTTGGGCTGCATTCC | 221233 | 0.23837829189890716 | No Hit |
| CTCTCTTCAAAGTTCTTTTCAACTTTCCCTTACGGTACTTGTTGACTATC | 206816 | 0.22284399170722446 | No Hit |
| CTCACCCGGCCCGGACACGGACAGGATTGACAGATTGATAGCTCTTTCTC | 175287 | 0.188871532059339 | No Hit |
| CGGGTCTTCCGTACGCCACATGTCCCGCGCCCCGCCGCGGGGCGGGGATT | 171043 | 0.18429862715446962 | No Hit |
| CTCCGACTTTCGTTCTTGATTAATGAAAACATTCTTGGCAAATGCTTTCG | 163958 | 0.17666454816036045 | No Hit |
| CTTTAAATGGGTAAGAAGCCCGGCTCGCTGGCGTGGAGCCGGGCGTGGAA | 158610 | 0.17090208458089734 | No Hit |
| CCCGTCGGCATGTATTAGCTCTAGAATTACCACAGTTATCCAAGTAGGAG | 155815 | 0.16789047543643226 | No Hit |

| Sequence | Count | Percentage | Possible Source |
|---|---|---|---|
| CTGCCAGTAGCATATGCTTGTCTCAAAGATTAAGCCATGCATGTCTAAGT | 153889 | 0.16581521274868996 | No Hit |
| CCCGAAGTTACGGATCCGGCTTGCCGACTTCCCTTACCTACATTGTTCCA | 153043 | 0.16490364876435457 | No Hit |
| CAAACTTTAAATGGGTAAGAAGCCCGGCTCGCTGGCGTGGAGCCGGGCGT | 149805 | 0.16141470765173274 | No Hit |
| CTTCACCGTGCCAGACTAGAGTCAAGCTCAACAGGGTCTTCTTTCCCCGC | 145158 | 0.15640757073068468 | No Hit |
| CTTGGTTATAATTTTTCATCTTTCCCTTGCGGTACTATATCTATTGCGCC | 141092 | 0.15202646061211758 | No Hit |
| CTGGATAGTAGGTAGGGACAGTGGGAATCTCGTTCATCCATTCATGCGCG | 135615 | 0.14612499968752535 | No Hit |
| CTCGCATTCCACGCCCGGCTCCACGCCAGCGAGCCGGGCTTCTTACCCAT | 130141 | 0.14022677125933147 | No Hit |
| CTCCCTTTCGATCGGCCGAGGGCAACGGAGGCCATCGCCCGTCCCTTCGG | 128136 | 0.13806638616643252 | No Hit |
| CTCCCACTTATTCTACACCTCTCATGTCTCTTCACCGTGCCAGACTAGAG | 127877 | 0.13778731397737476 | No Hit |
| CGACGACCCATTCGAACGTCTGCCCTATCAACTTTCGATGGTAGTCGCCG | 126955 | 0.1367938600842811 | No Hit |
| CCCATATCCGCAGCAGGTCTCCAAGGTGAACAGCCTCTGGCATGTTGGAA | 125359 | 0.13507417200035757 | No Hit |
| CTCTCATGTCTCTTCACCGTGCCAGACTAGAGTCAAGCTCAACAGGGTCT | 124819 | 0.13449232264865413 | No Hit |
| CTGCTGTCTATATCAACCAACACCTTTTCTGGGGTCTGATGAGCGTCGGC | 124172 | 0.1337951809254094 | No Hit |
| CTCTGGTCCGTCTTGCGCCGGTCCAAGAATTTCACCTCTAGCGGCGCAAT | 121057 | 0.13043877216512006 | No Hit |
| GTCAAAGTGAAGAAATTCAATGAAGCGCGGGTAAACGGCGGGAGTAACTA | 116540 | 0.1255717100880006 | No Hit |
| ATCAGACGTGGCGACCCGCTGAATTTAAGCATATTAGTCAGCGGAGGAGA | 115968 | 0.12495538077471474 | No Hit |
| CTCCCGTCCACTCTCGACTGCCGGCGACGGCCGGGTATGGGCCCGACGCT | 114518 | 0.12339300751551102 | No Hit |
| CCCCGCTTCGCGCCCCAGCCCGACCGACCCAGCCCTTAGAGCCAATCCTT | 112691 | 0.12142441720891435 | No Hit |
| GCCCTCTTGAACTCTCTCTTCAAAGTTCTTTTCAACTTTCCCTTACGGTA | 106526 | 0.11478163711029993 | No Hit |
| CAAAGATTAAGCCATGCATGTCTAAGTACGCACGGCCGGTACAGTGAAAC | 106106 | 0.11432908761453058 | No Hit |
| CGCGATGTGATTTCTGCCCAGTGCTCTGAATGTCAAAGTGAAGAAATTCA | 104891 | 0.11301992657319782 | No Hit |
| CTGAATTTAAGCATATTAGTCAGCGGAGGAGAAGAAACTAACCAGGATTC | 103139 | 0.11113214867655993 | No Hit |
| CCACTCTCGACTGCCGGCGACGGCCGGGTATGGGCCCGACGCTCCAGCGC | 102394 | 0.11032941207096907 | No Hit |
| CTTAGATGGAGTTTACCACCCGCTTTGGGCTGCATTCCCAAGCAACCCGA | 101322 | 0.10917433335795776 | No Hit |
| CCCAGGCATAGTTCACCATCTTTCGGGTCCTAACACGTGCGCTCGTGCTC | 100698 | 0.10850197410710044 | No Hit |
| CGCGTCACTAATTAGATGACGAGGCATTTGGCTACCTTAAGAGAGTCATA | 100148 | 0.10790934976740249 | No Hit |
| CACCCGTTTACCTCTTAACGGTTTCACGCCCTCTTGAACTCTCTCTTCAA | 99149 | 0.10683292846675109 | No Hit |
| CTTGAACTCTCTCTTCAAAGTTCTTTTCAACTTTCCCTTACGGTACTTGT | 98933 | 0.10660018872606972 | No Hit |
| CGAAGGCCCGCGGCGGGTGTTGACGCGATGTGATTTCTGCCCAGTGCTCT | 97397 | 0.10494515057011324 | No Hit |
| CCCACTTATTCTACACCTCTCATGTCTCTTCACCGTGCCAGACTAGAGTC | 96178 | 0.10363167953358267 | No Hit |
| CCCTCCTTAGGCAACCTGGTGGTCCCCCGCTCCCGGGAGGTCACCATATT | 93238 | 0.10046383306319721 | No Hit |

## Adapter Content

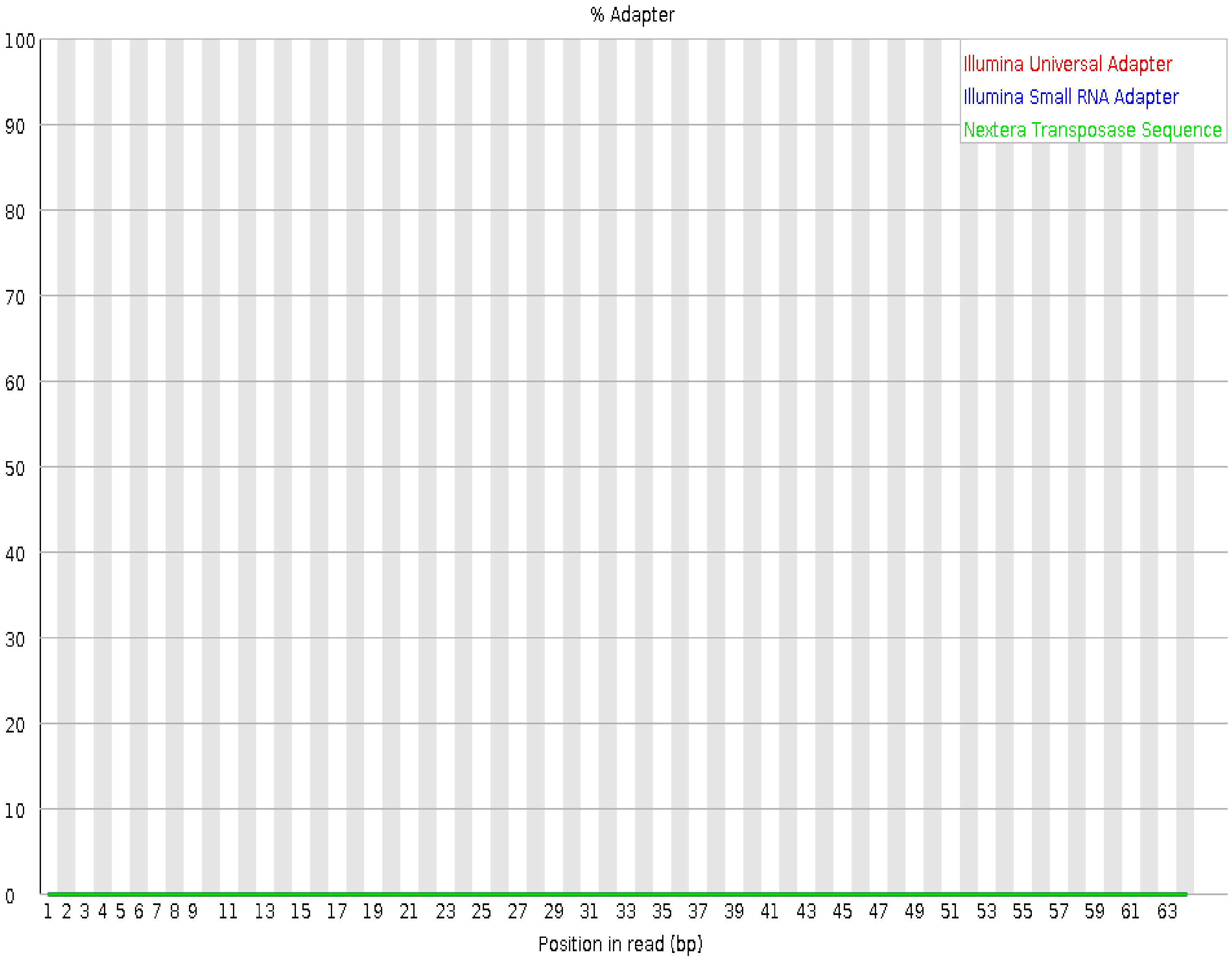


## Kmer Content

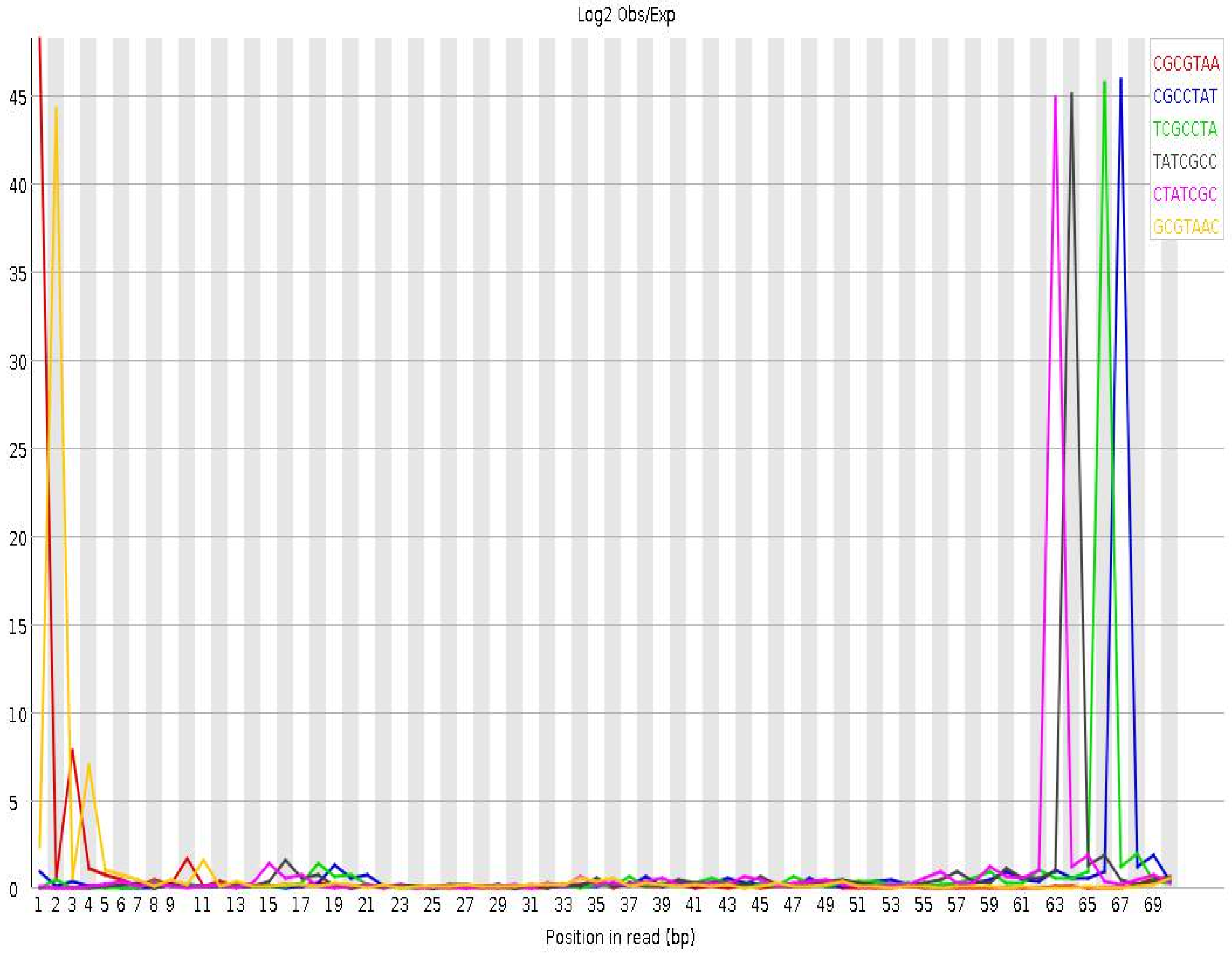


| Sequence | Count | PValue | Obs/Exp Max | Max Obs/Exp Position |
|---|---|---|---|---|
| CGCGTAA | 55010 | 0.0 | 48.18933 | 1 |
| CGCCTAT | 25755 | 0.0 | 45.92571 | 67 |
| TCGCCTA | 25810 | 0.0 | 45.733425 | 66 |
| TATCGCC | 26285 | 0.0 | 45.107113 | 64 |
| CTATCGC | 26415 | 0.0 | 44.9198 | 63 |
| GCGTAAC | 60330 | 0.0 | 44.283096 | 2 |
| ATCGCCT | 26770 | 0.0 | 44.224102 | 65 |
| ACTCGCC | 78085 | 0.0 | 40.845634 | 69 |
| GCCTATA | 30030 | 0.0 | 39.76125 | 68 |
| TCACTCG | 80640 | 0.0 | 39.368896 | 67 |
| AGTTAGC | 63025 | 0.0 | 39.333275 | 10 |
| TAGTTAG | 64445 | 0.0 | 38.993782 | 9 |
| CGACGAC | 27715 | 0.0 | 38.25429 | 1 |
| GTTAGCA | 64970 | 0.0 | 38.197124 | 11 |
| AACTAGT | 68925 | 0.0 | 37.5262 | 6 |

| Sequence | Count | PValue | Obs/Exp Max | Max Obs/Exp Position |
|---|---|---|---|---|
| ACTAGTT | 68490 | 0.0 | 37.47841 | 7 |
| TCAGACG | 42460 | 0.0 | 36.90[illegible] | 2 |
| CGTAACT | 72845 | 0.0 | 36.728695 | 3 |
| ACGACCC | 29135 | 0.0 | 36.405666 | 3 |
| TTCACTC | 89935 | 0.0 | 35.168106 | 66 |

**Produced by FastQC (version 0.11.2)**

# FastQC Report

Mon 4 May 2015
Agilent_Adult_Kidney_GCCAAT_L004_R2.fastq.gz

## Summary


- Basic Statistics
- Per base sequence quality
- Per tile sequence quality
- Per sequence quality scores
- Per base sequence content
- Per sequence GC content
- Per base N content
- Sequence Length Distribution
- Sequence Duplication Levels
- Overrepresented sequences
- Adapter Content
- Kmer Content


## Basic Statistics

| Measure | Value |
|---|---|
| Filename | Agilent_Adult_Kidney_GCCAAT_L004_R2.fastq.gz |
| File type | Conventional base calls |
| Encoding | Sanger / Illumina 1.9 |
| Total Sequences | 92807528 |
| Sequences flagged as poor quality | 0 |
| Sequence length | 76 |
| %GC | 54 |

## Per base sequence quality

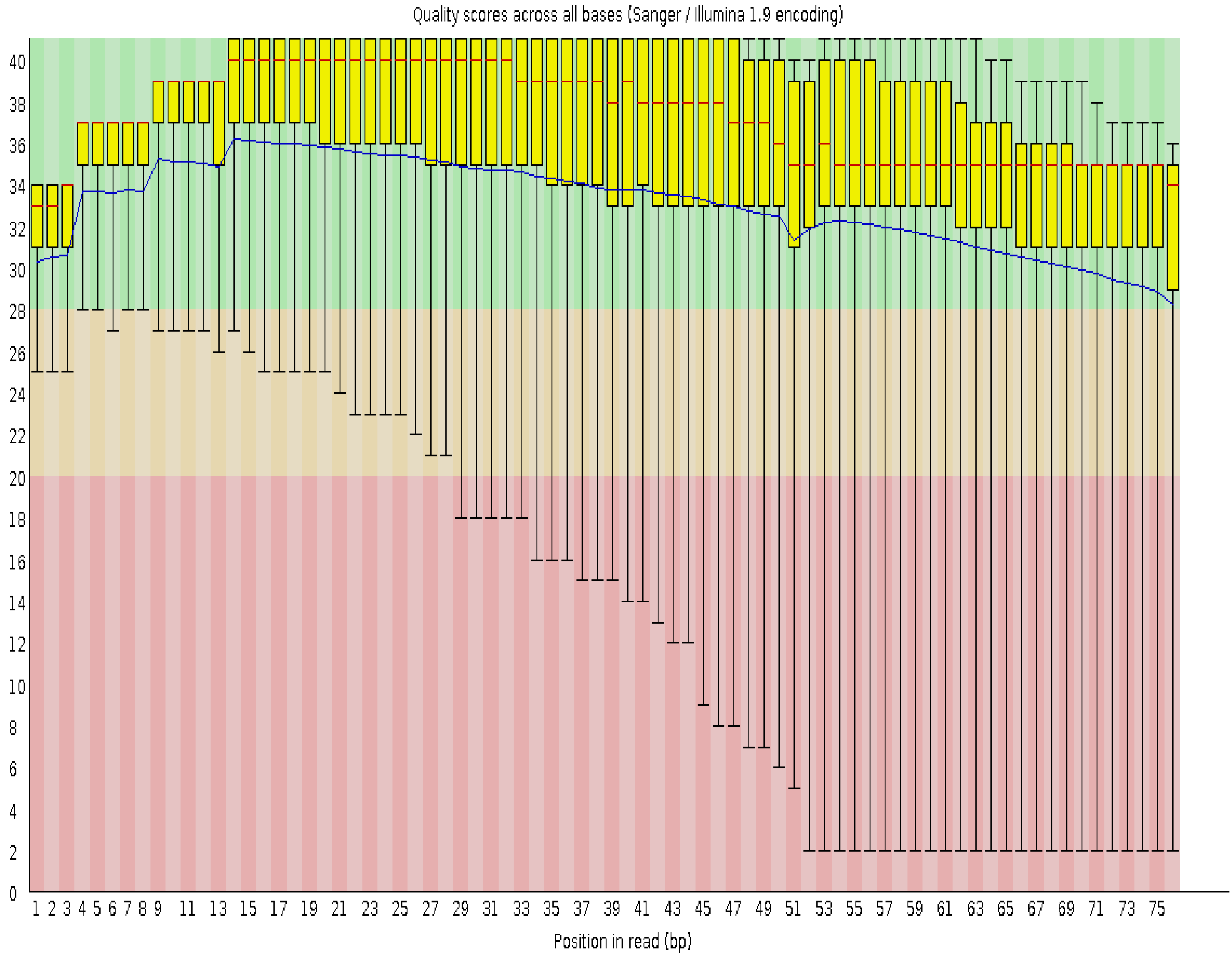


## Per tile sequence quality

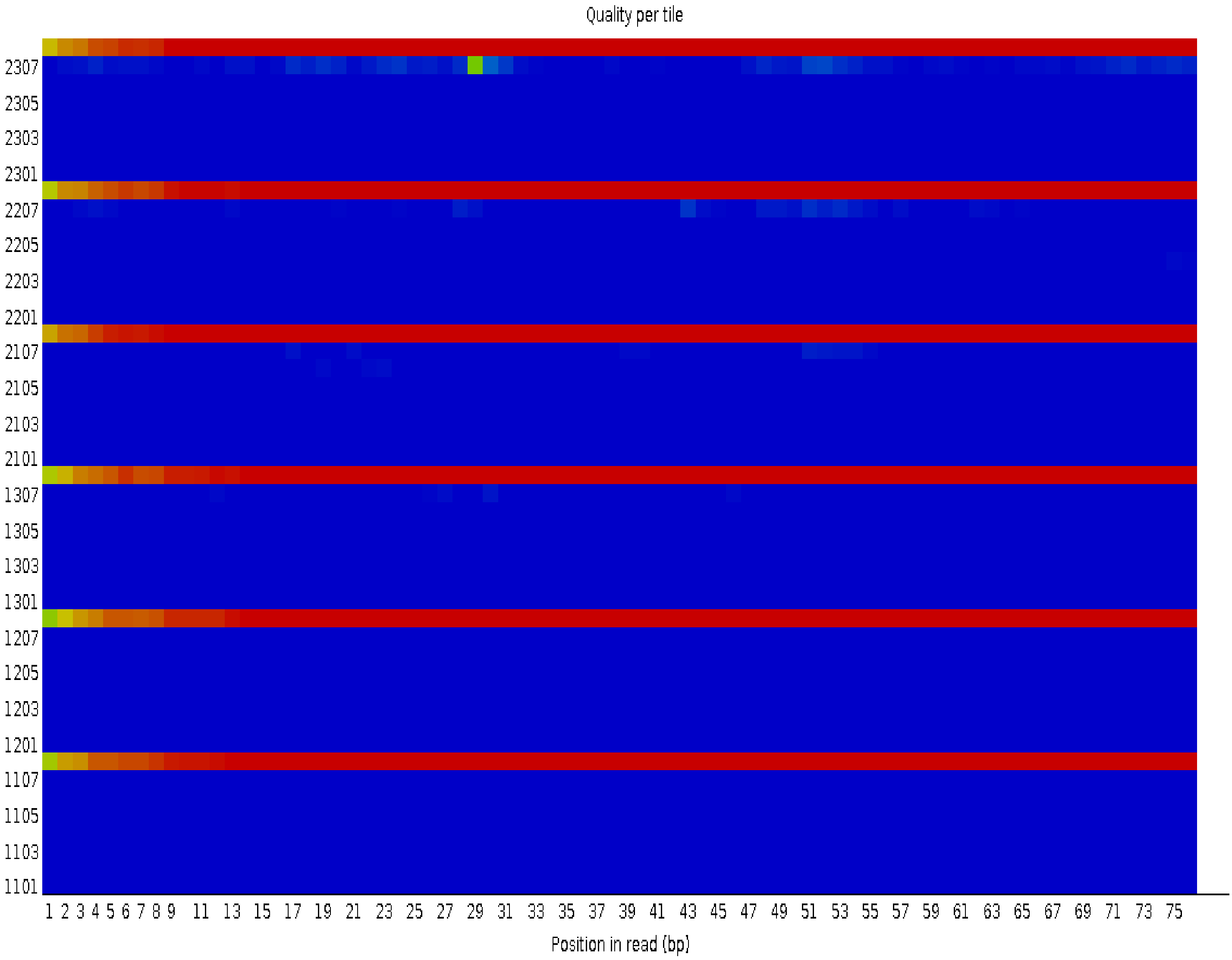


## Per sequence quality scores

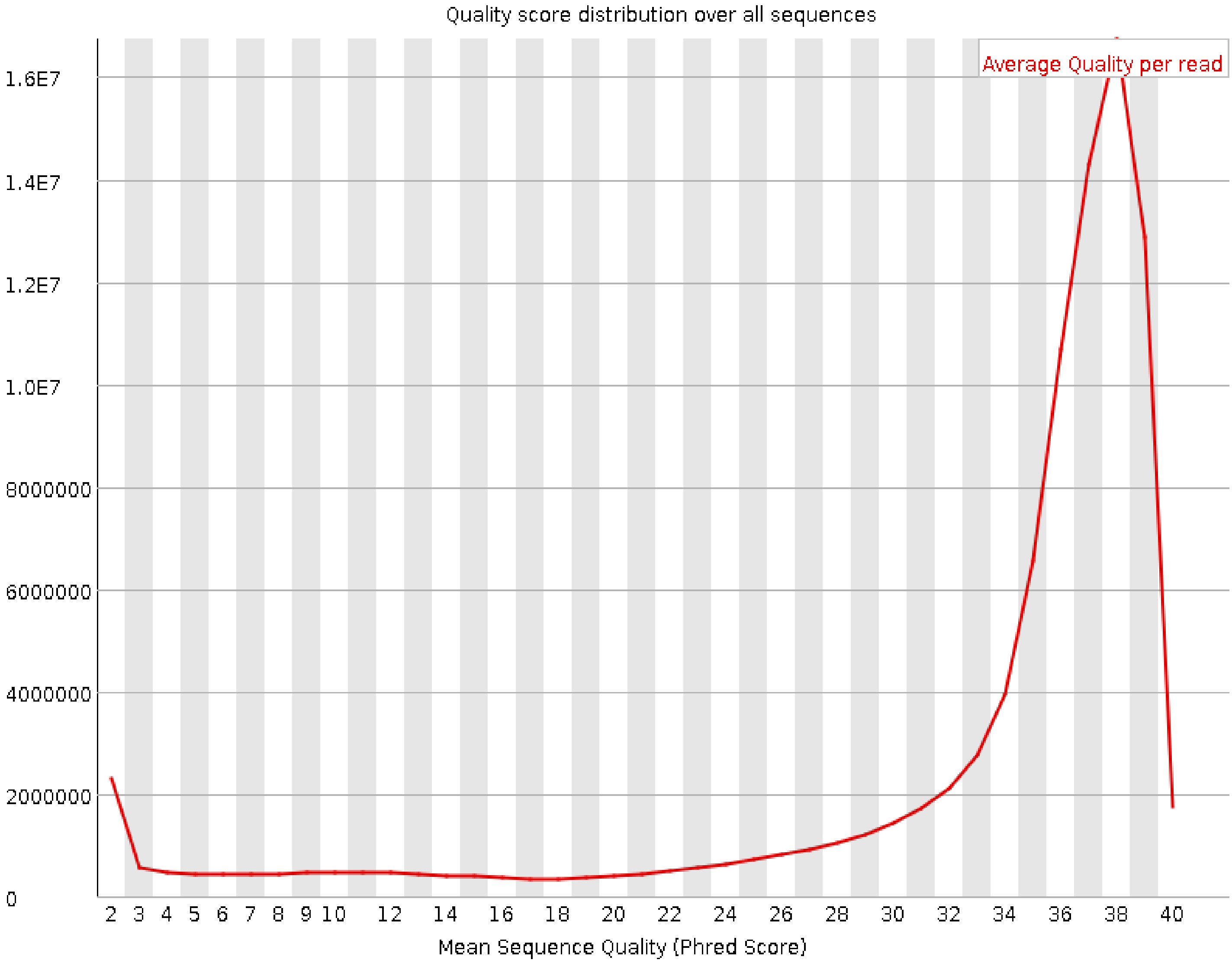


## Per base sequence content

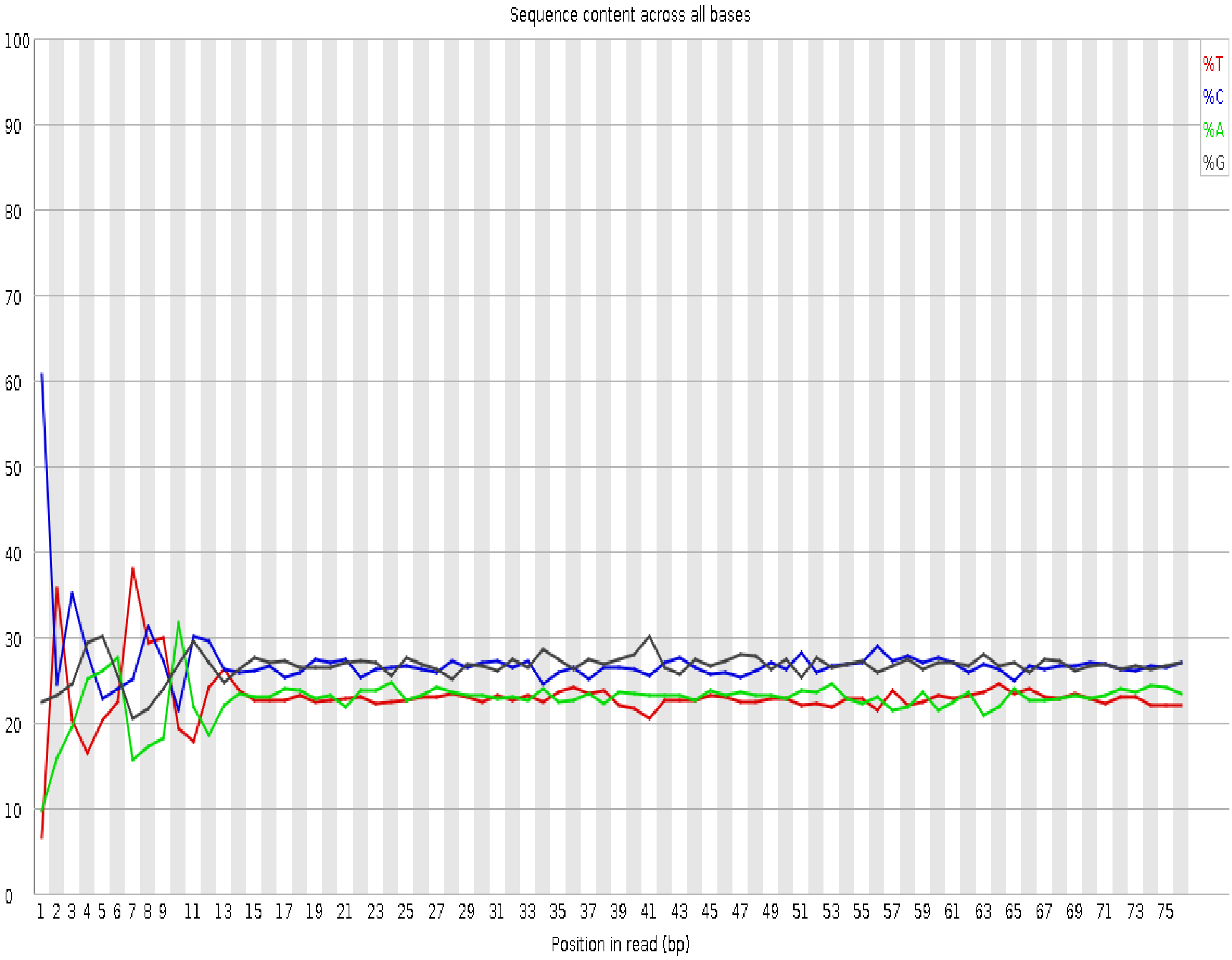


## Per sequence GC content

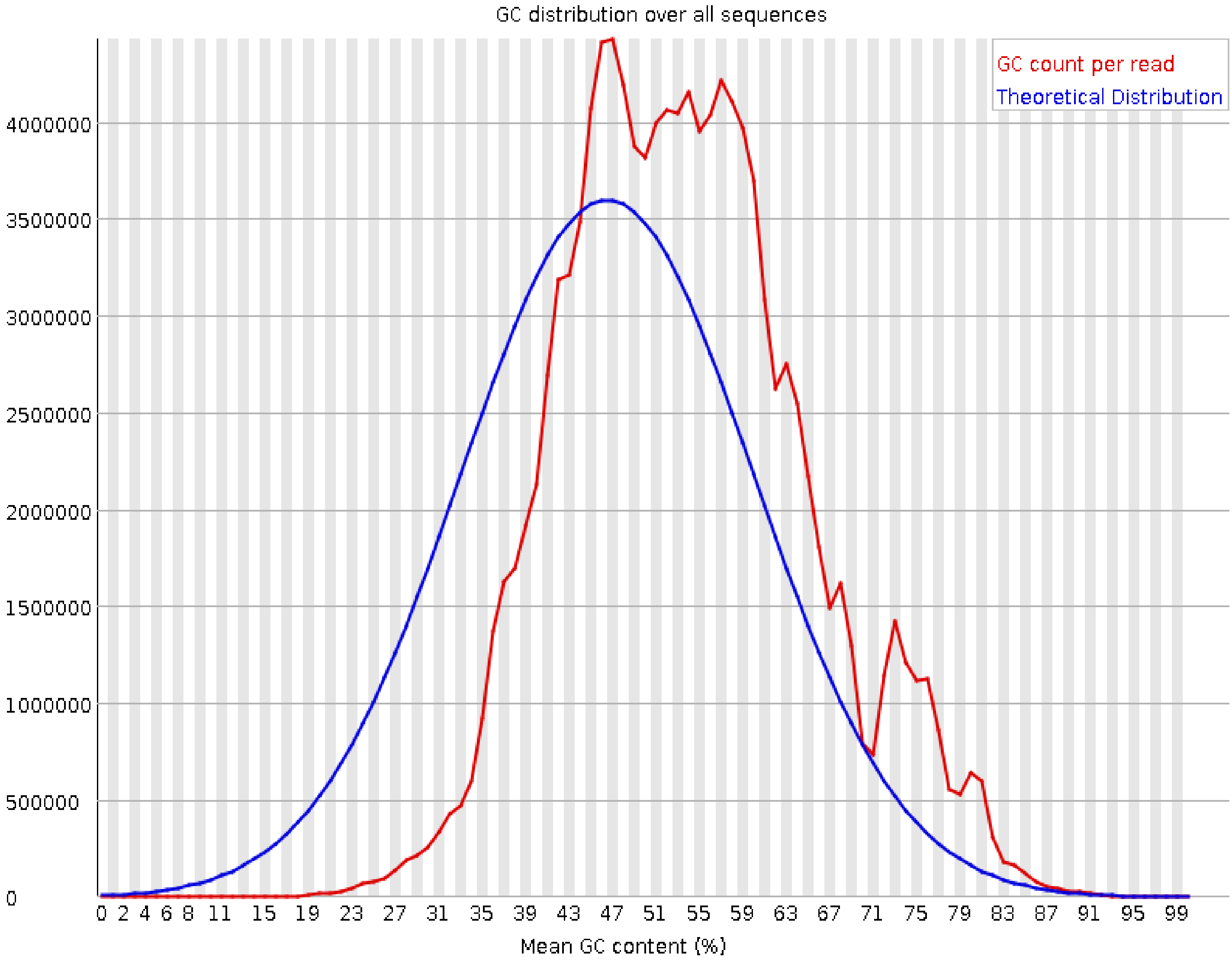


## Per base N content

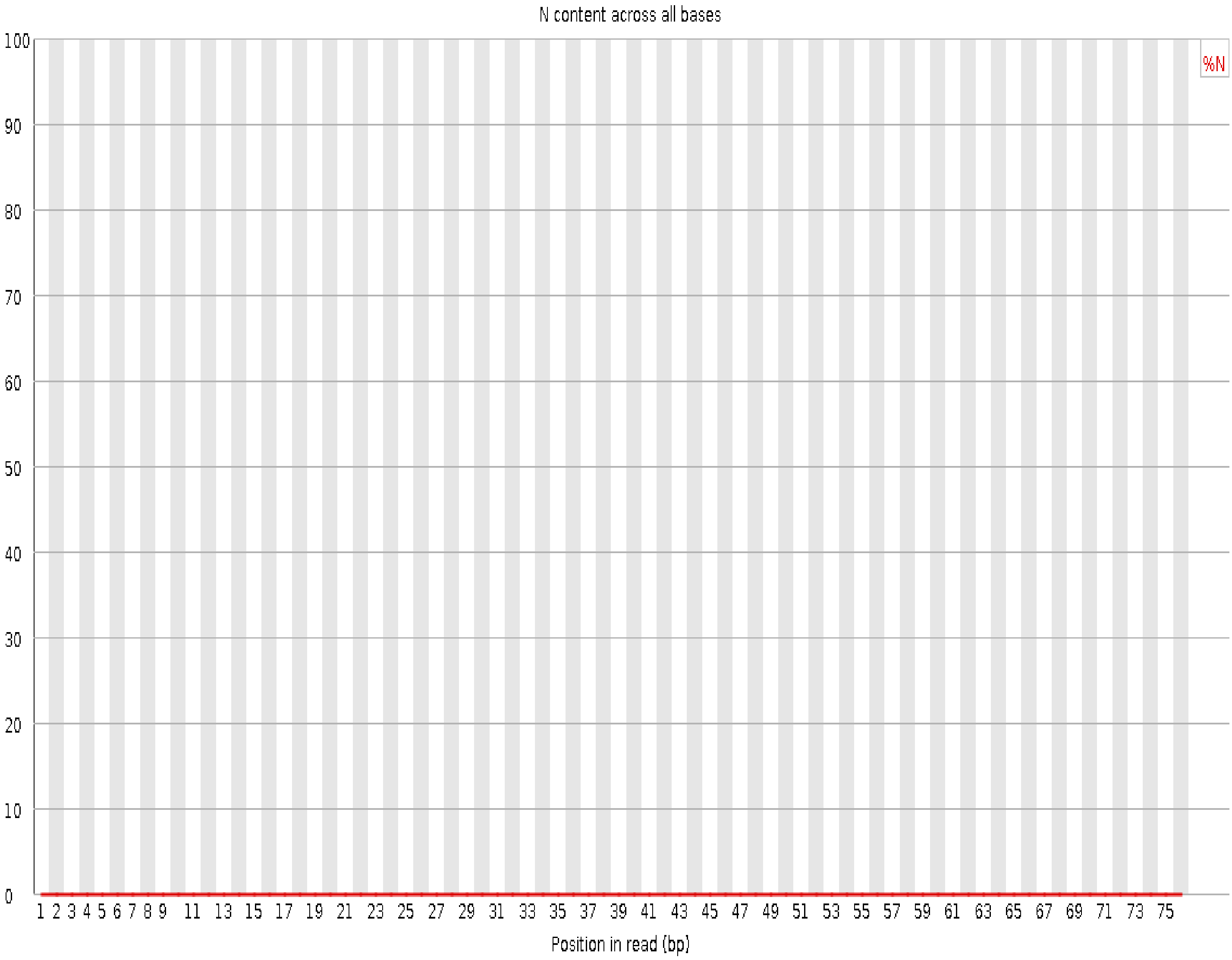


## Sequence Length Distribution

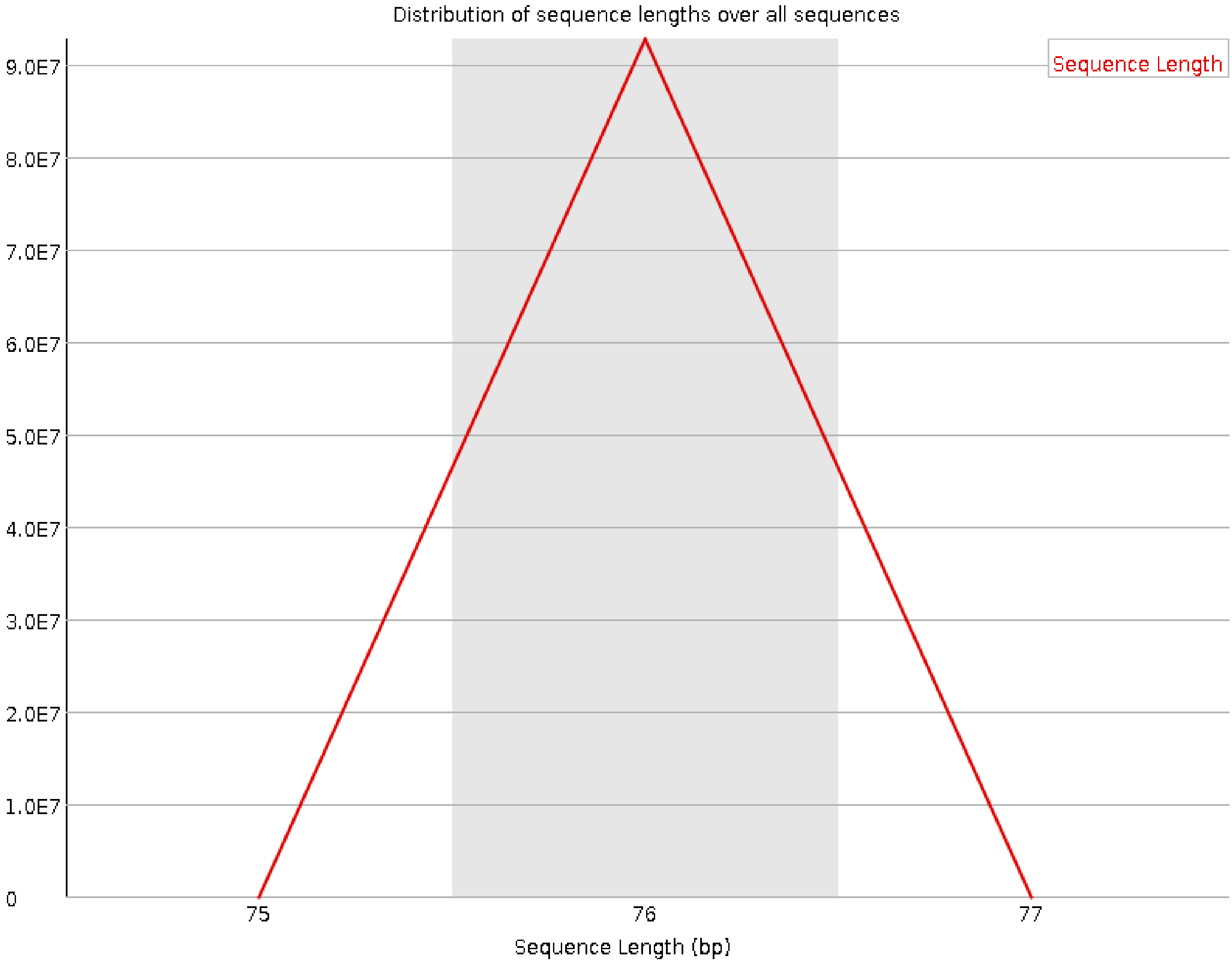


## Sequence Duplication Levels

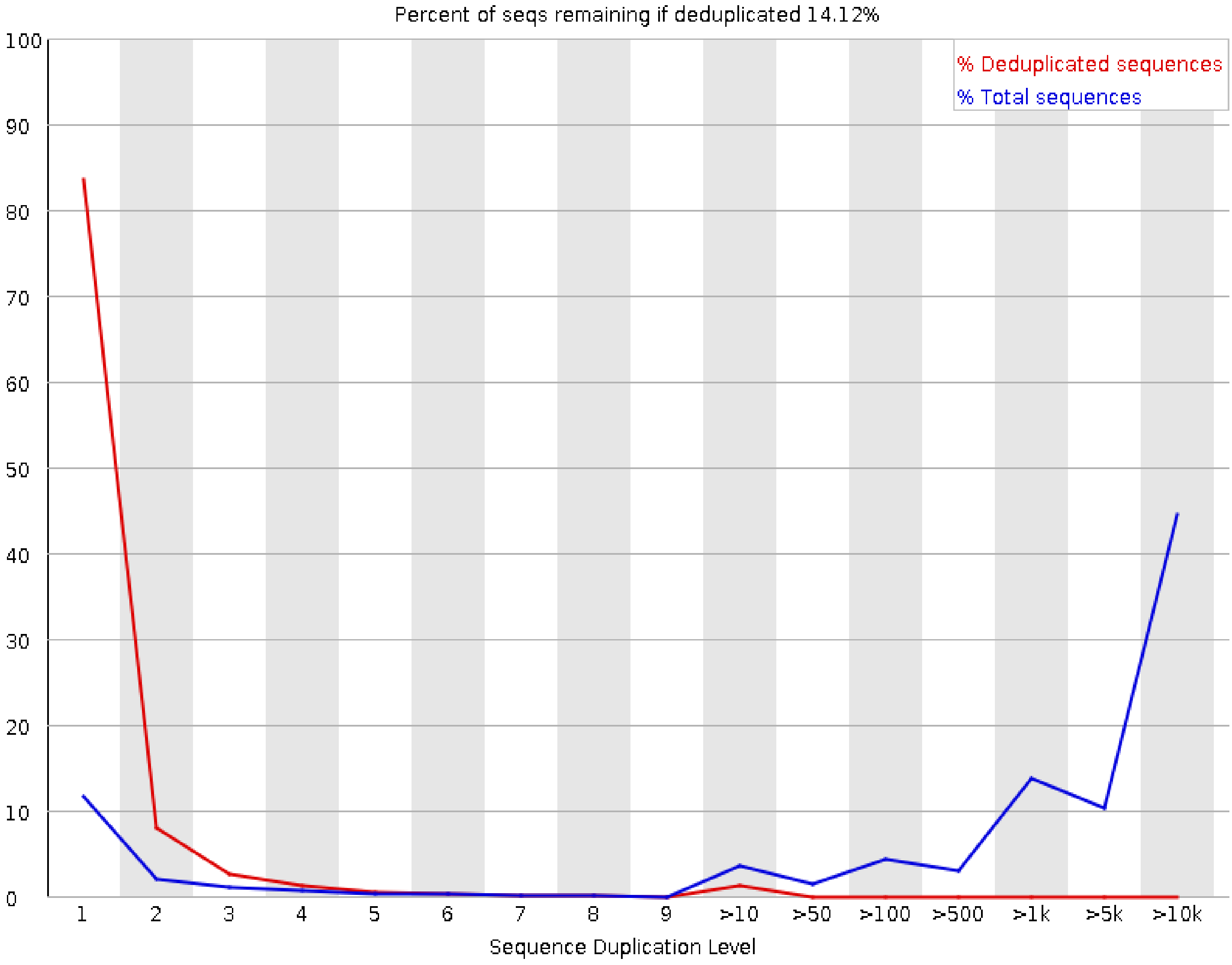


## Overrepresented sequences

| Sequence | Count | Percentage | Possible Source |
|---|---|---|---|
| CTTCCGTACGCCACATGTCCCGCGCCCCGCCGCGGGGCGGGGATTCGGCG | 423026 | 0.45581000713649006 | No Hit |
| CGCGTAACTAGTTAGCATGCCAGAGTCTCGTTCGTTATCGGAATTAACCA | 328591 | 0.3540564080103502 | No Hit |
| CGCAGTTTTATCCGGTAAAGCGAATGATTAGAGGTCTTGGGGCCGAAACG | 304390 | 0.3279798595648405 | No Hit |
| CCTCACCCGGCCCGGACACGGACAGGATTGACAGATTGATAGCTCTTTCT | 300682 | 0.3239844940164768 | No Hit |
| CCGGTATTTAGCCTTAGATGGAGTTTACCACCCGCTTTGGGCTGCATTCC | 228310 | 0.24600375090262075 | No Hit |
| CTCCGACTTTCGTTCTTGATTAATGAAAACATTCTTGGCAAATGCTTTCG | 207433 | 0.2235088084664856 | No Hit |
| CTCTCTTCAAAGTTCTTTTCAACTTTCCCTTACGGTACTTGTTGACTATC | 207206 | 0.22326421623901027 | No Hit |
| CGGGTCTTCCGTACGCCACATGTCCCGCGCCCCGCCGCGGGGCGGGGATT | 207082 | 0.22313060638787835 | No Hit |
| CTCACCCGGCCCGGACACGGACAGGATTGACAGATTGATAGCTCTTTCTC | 185993 | 0.20040723420625967 | No Hit |
| CCCGTCGGCATGTATTAGCTCTAGAATTACCACAGTTATCCAAGTAGGAG | 178500 | 0.1923335357019745 | No Hit |
| CTTCACCGTGCCAGACTAGAGTCAAGCTCAACAGGGTCTTCTTTCCCCGC | 164076 | 0.17679169301869563 | No Hit |

| Sequence | Count | Percentage | Possible Source |
|---|---|---|---|
| CTGCCAGTAGCATATGCTTGTCTCAAAGATTAAGCCATGCATGTCTAAGT | 161630 | 0.17415613095523888 | No Hit |
| CAAACTTTAAATGGGTAAGAAGCCCGGCTCGCTGGCGTGGAGCCGGGCGT | 149733 | 0.16133712773817227 | N[illegible] |
| CTTTAAATGGGTAAGAAGCCCGGCTCGCTGGCGTGGAGCCGGGCGTGGAA | 149166 | 0.16072618591888363 | No Hit |
| CTCCCTTTCGATCGGCCGAGGGCAACGGAGGCCATCGCCCGTCCCTTCGG | 147882 | 0.15934267746038877 | No Hit |
| CTGGATAGTAGGTAGGGACAGTGGGAATCTCGTTCATCCATTCATGCGCG | 143487 | 0.15460707023680234 | No Hit |
| CTCCCACTTATTCTACACCTCTCATGTCTCTTCACCGTGCCAGACTAGAG | 142924 | 0.15400043841271152 | No Hit |
| CGACGACCCATTCGAACGTCTGCCCTATCAACTTTCGATGGTAGTCGCCG | 140798 | 0.15170967596507903 | No Hit |
| CTCGCATTCCACGCCCGGCTCCACGCCAGCGAGCCGGGCTTCTTACCCAT | 138098 | 0.14880042920656178 | No Hit |
| CTTGGTTATAATTTTTCATCTTTCCCTTGCGGTACTATATCTATTGCGCC | 135542 | 0.14604634227516544 | No Hit |
| CCCGAAGTTACGGATCCGGCTTGCCGACTTCCCTTACCTACATTGTTCCA | 134974 | 0.14543432295707737 | No Hit |
| CTCTCATGTCTCTTCACCGTGCCAGACTAGAGTCAAGCTCAACAGGGTCT | 134962 | 0.14542139297148396 | No Hit |
| CTCTGGTCCGTCTTGCGCCGGTCCAAGAATTTCACCTCTAGCGGCGCAAT | 133748 | 0.14411330942895065 | No Hit |
| CCCCGCTTCGCGCCCCAGCCCGACCGACCCAGCCCTTAGAGCCAATCCTT | 132074 | 0.14230957643866995 | No Hit |
| CTGCTGTCTATATCAACCAACACCTTTTCTGGGGTCTGATGAGCGTCGGC | 131494 | 0.14168462713498844 | No Hit |
| CTCCCGTCCACTCTCGACTGCCGGCGACGGCCGGGTATGGGCCCGACGCT | 130877 | 0.14101981037572728 | No Hit |
| CCCATATCCGCAGCAGGTCTCCAAGGTGAACAGCCTCTGGCATGTTGGAA | 123885 | 0.13348593876996703 | No Hit |
| GTCAAAGTGAAGAAATTCAATGAAGCGCGGGTAAACGGCGGGAGTAACTA | 115149 | 0.1240729092579645 | No Hit |
| CGCGATGTGATTTCTGCCCAGTGCTCTGAATGTCAAAGTGAAGAAATTCA | 113974 | 0.12280684816860976 | No Hit |
| GCCCTCTTGAACTCTCTCTTCAAAGTTCTTTTCAACTTTCCCTTACGGTA | 110752 | 0.11933514703677917 | No Hit |
| CGCGTCACTAATTAGATGACGAGGCATTTGGCTACCTTAAGAGAGTCATA | 109101 | 0.11755619651888584 | No Hit |
| CCACTCTCGACTGCCGGCGACGGCCGGGTATGGGCCCGACGCTCCAGCGC | 107178 | 0.11548416632754187 | No Hit |
| CAAAGATTAAGCCATGCATGTCTAAGTACGCACGGCCGGTACAGTGAAAC | 106393 | 0.11463832976997296 | No Hit |
| ATCAGACGTGGCGACCCGCTGAATTTAAGCATATTAGTCAGCGGAGGAGA | 106016 | 0.11423211272257999 | No Hit |
| CTCCGTTTCCGACCTGGGCCGGTTCACCCCTCCTTAGGCAACCTGGTGGT | 105292 | 0.1134520035917776 | No Hit |
| CCCAGGCATAGTTCACCATCTTTCGGGTCCTAACACGTGCGCTCGTGCTC | 105134 | 0.11328175878146438 | No Hit |
| CACCCGTTTACCTCTTAACGGTTTCACGCCCTCTTGAACTCTCTCTTCAA | 104660 | 0.11277102435052466 | No Hit |
| CCCACTTATTCTACACCTCTCATGTCTCTTCACCGTGCCAGACTAGAGTC | 101354 | 0.10920881331954019 | No Hit |
| CTCGATCAGAAGGACTTGGGCCCCCCACGAGCGGCGCCGGGGAGCGGGTC | 100056 | 0.10781021987785301 | No Hit |
| CCCGCTTCGCGCCCCAGCCCGACCGACCCAGCCCTTAGAGCCAATCCTTA | 99392 | 0.10709476067501766 | No Hit |
| CTTAGATGGAGTTTACCACCCGCTTTGGGCTGCATTCCCAAGCAACCCGA | 98910 | 0.10657540625368235 | No Hit |
| CTTGAACTCTCTCTTCAAAGTTCTTTTCAACTTTCCCTTACGGTACTTGT | 98856 | 0.106517221318512 | No Hit |
| CGAAGGCCCGCGGCGGGTGTTGACGCGATGTGATTTCTGCCCAGTGCTCT | 98696 | 0.10634482151059987 | No Hit |
| CTGAATTTAAGCATATTAGTCAGCGGAGGAGAAGAAACTAACCAGGATTC | 98507 | 0.10614117423750366 | No Hit |
| CCCTCCTTAGGCAACCTGGTGGTCCCCCGCTCCCGGGAGGTCACCATATT | 98359 | 0.10598170441518495 | No Hit |
| CCCGCTTTGGGCTGCATTCCCAAGCAACCCGACTCCGGGAAGACCCGGGC | 94529 | 0.10185488401328822 | No Hit |
| CCACCGTCCTGCTGTCTATATCAACCAACACCTTTTCTGGGGTCTGATGA | 93959 | 0.10124070969760127 | No Hit |

## Adapter Content

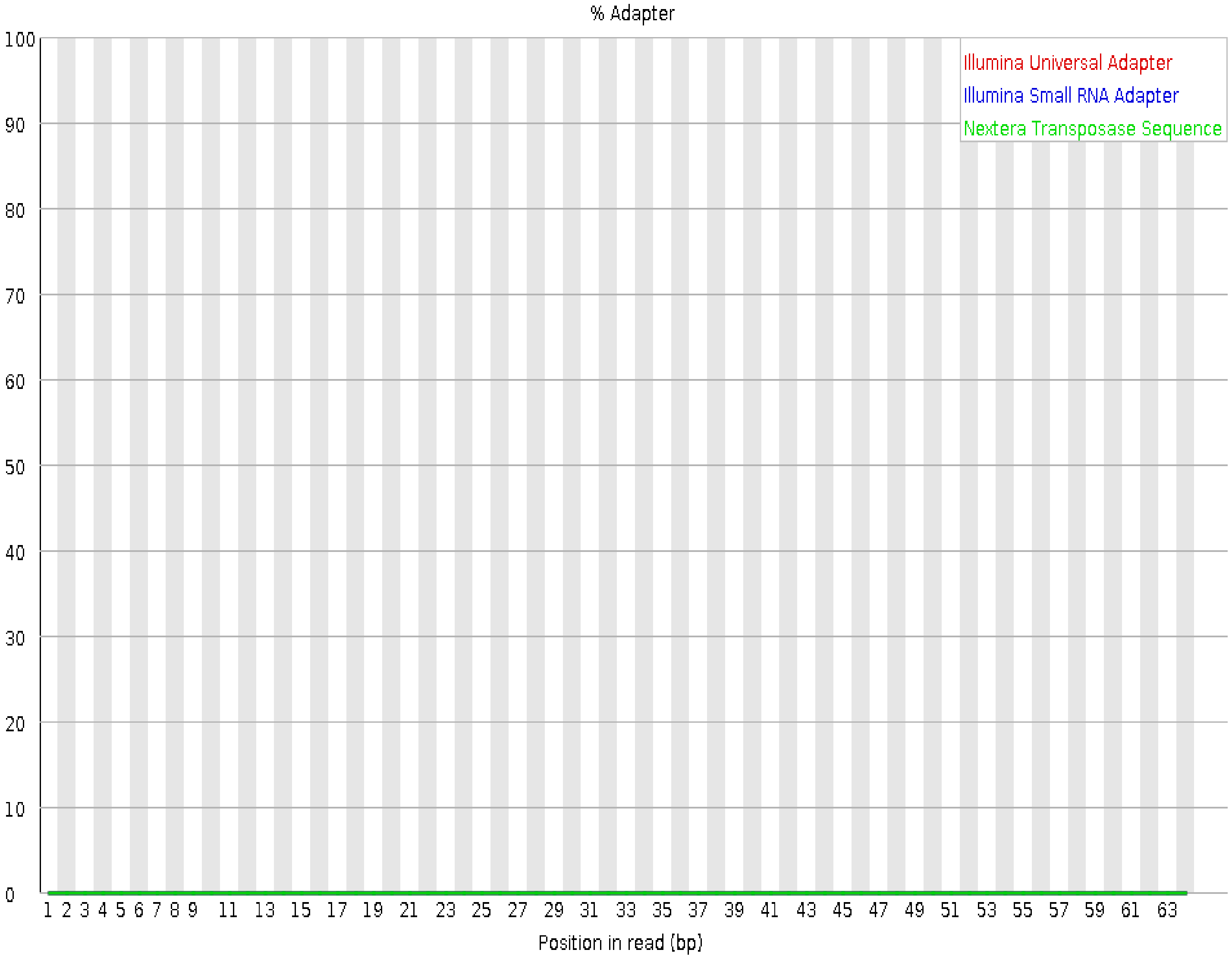


## Kmer Content

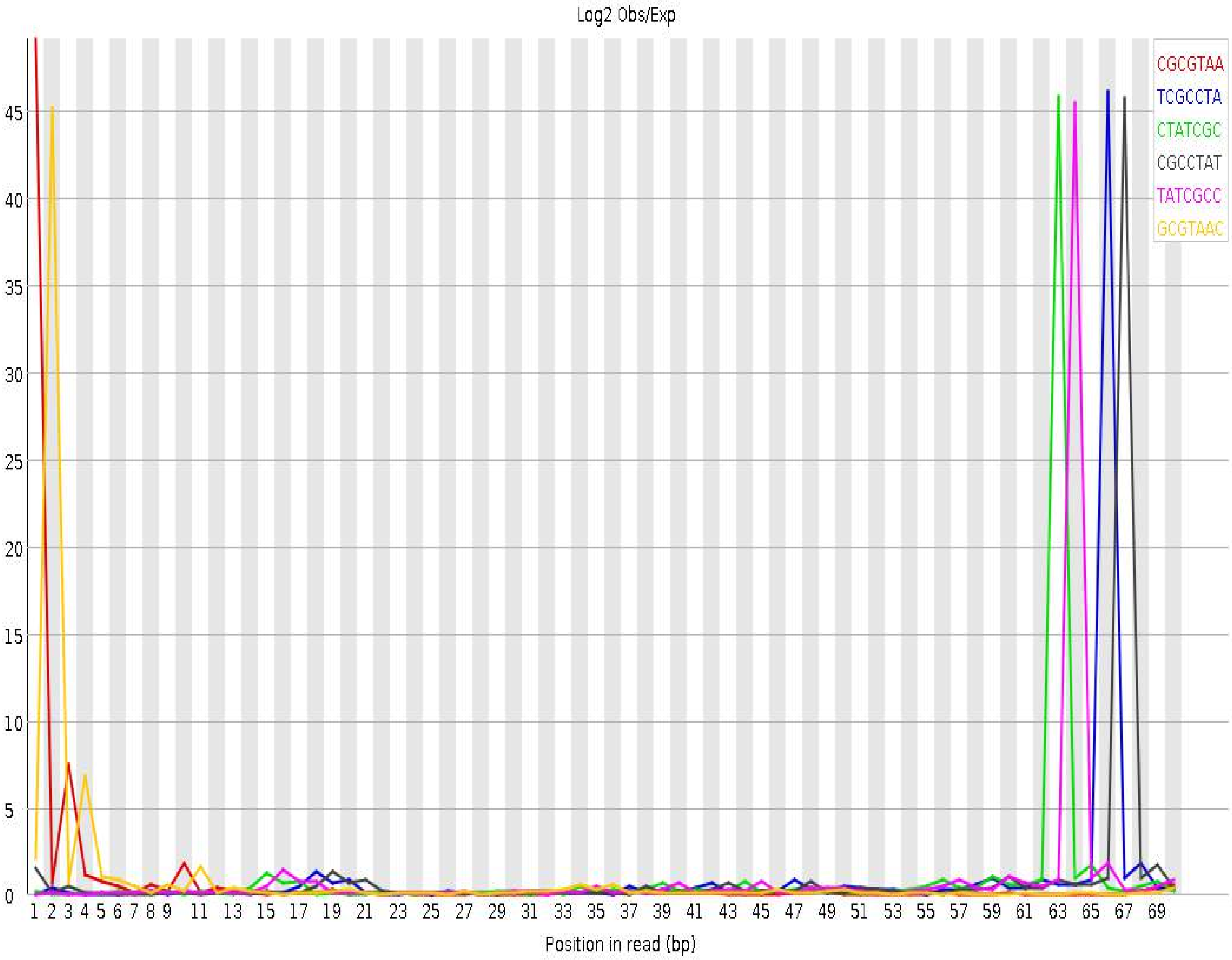


| Sequence | Count | PValue | Obs/Exp Max | Max Obs/Exp Position |
|---|---|---|---|---|
| CGCGTAA | 53410 | 0.0 | 49.10454 | 1 |
| TCGCCTA | 23320 | 0.0 | 46.146988 | 66 |
| CTATCGC | 23605 | 0.0 | 45.81115 | 63 |
| CGCCTAT | 23495 | 0.0 | 45.744946 | 67 |
| TATCGCC | 23810 | 0.0 | 45.4459 | 64 |
| GCGTAAC | 58715 | 0.0 | 45.175438 | 2 |
| ATCGCCT | 24450 | 0.0 | 44.3145 | 65 |
| AGTTAGC | 63350 | 0.0 | 41.433037 | 10 |
| TAGTTAG | 64590 | 0.0 | 40.88474 | 9 |
| ACTCGCC | 81135 | 0.0 | 40.664284 | 69 |
| TCACTCG | 81855 | 0.0 | 40.198864 | 67 |
| GTTAGCA | 65520 | 0.0 | 40.098186 | 11 |
| CGACGAC | 28010 | 0.0 | 39.07332 | 1 |
| GCCTATA | 27960 | 0.0 | 38.865692 | 68 |
| AACTAGT | 69665 | 0.0 | 38.484543 | 6 |

| Sequence | Count | PValue | Obs/Exp Max | Max Obs/Exp Position |
|---|---|---|---|---|
| ACTAGTT | 69255 | 0.0 | 38.[illegible] | 7 |
| CGTAACT | 70335 | 0.0 | 37.78[illegible] | 3 |
| ACGACCC | 29105 | 0.0 | 37.636475 | 3 |
| CTCGCTA | 13005 | 0.0 | 36.047462 | 1 |
| TTCACTC | 91285 | 0.0 | 35.99154 | 66 |

**Produced by FastQC (version 0.11.2)**

# FastQC Report

Mon 4 May 2015
Agilent_Adult_Liver_ATCACG_L003_R1.fastq.gz

## Summary


- Basic Statistics
- Per base sequence quality
- Per tile sequence quality
- Per sequence quality scores
- Per base sequence content
- Per sequence GC content
- Per base N content
- Sequence Length Distribution
- Sequence Duplication Levels
- Overrepresented sequences
- Adapter Content
- Kmer Content


## Basic Statistics

| Measure | Value |
|---|---|
| Filename | Agilent_Adult_Liver_ATCACG_L003_R1.fastq.gz |
| File type | Conventional base calls |
| Encoding | Sanger / Illumina 1.9 |
| Total Sequences | 78137222 |
| Sequences flagged as poor quality | 0 |
| Sequence length | 76 |
| %GC | 53 |

## Per base sequence quality

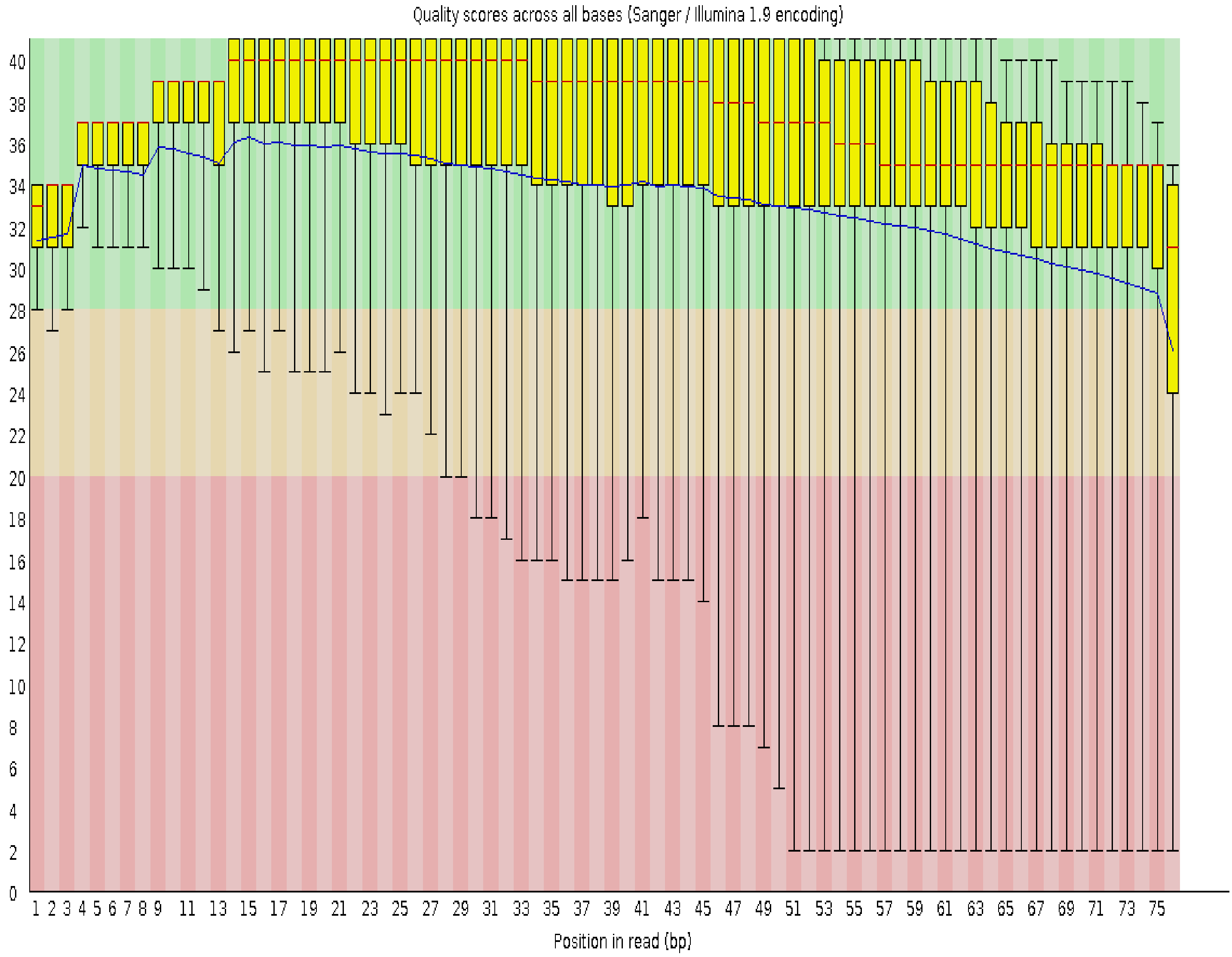


## Per tile sequence quality

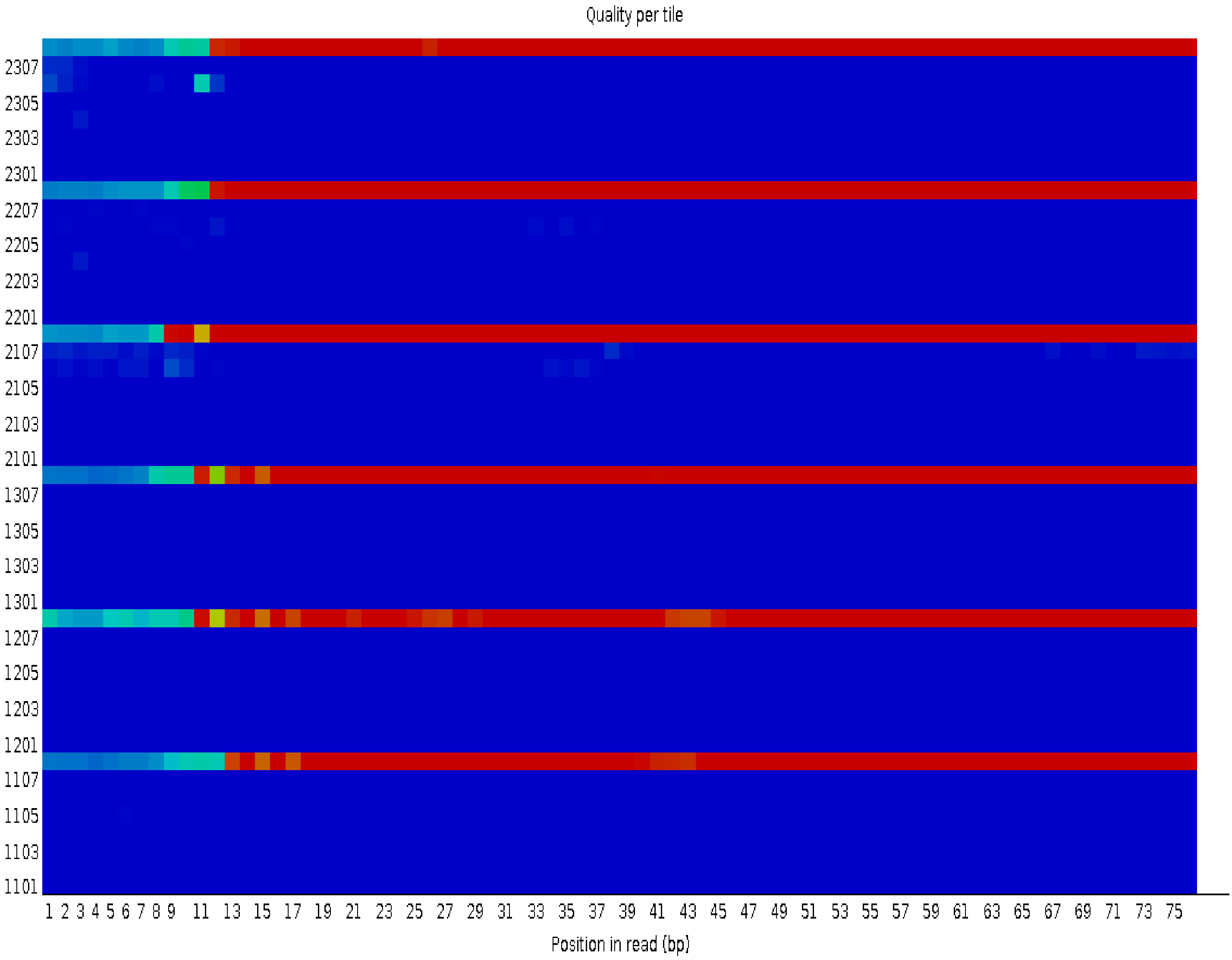


## Per sequence quality scores

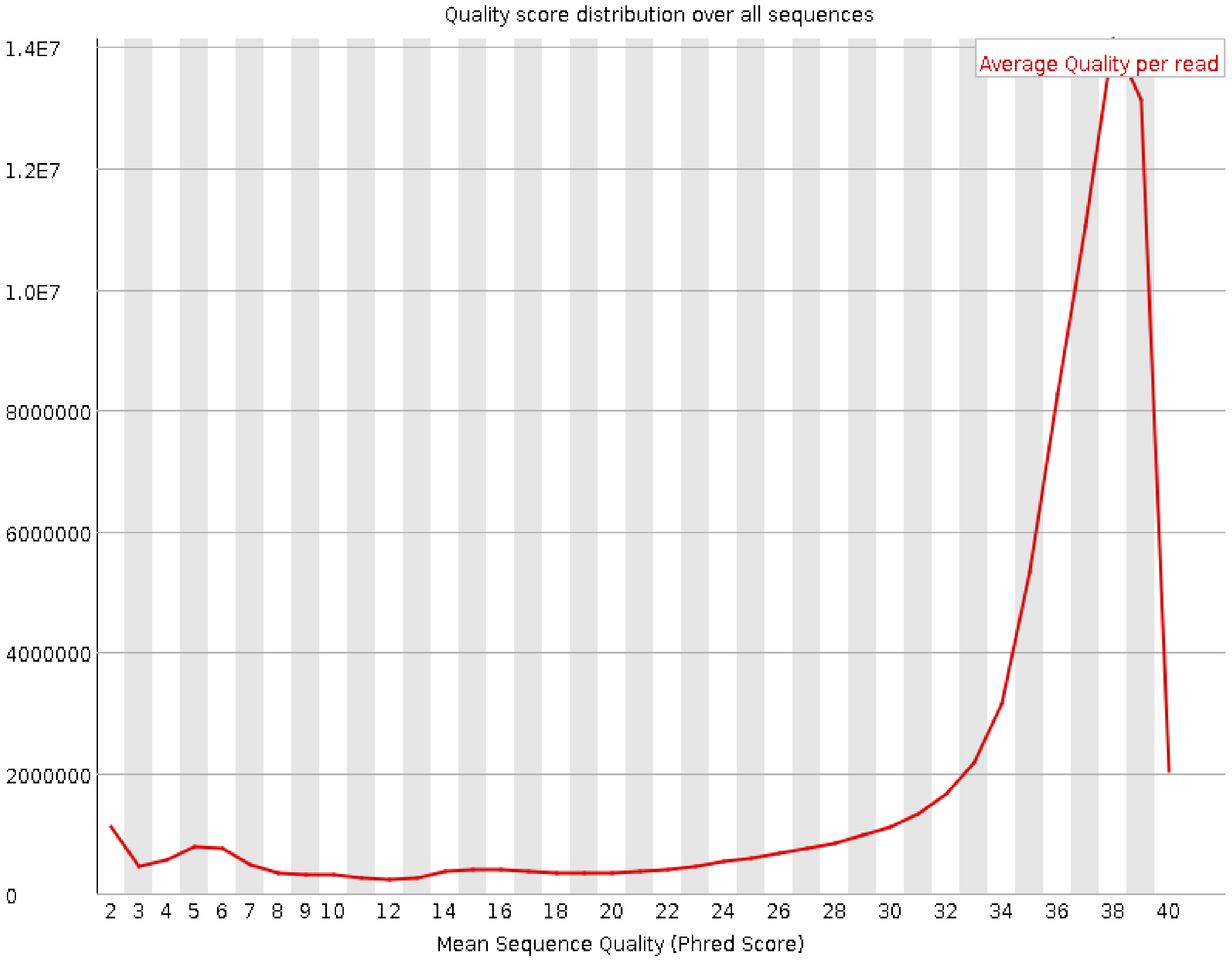


## Per base sequence content

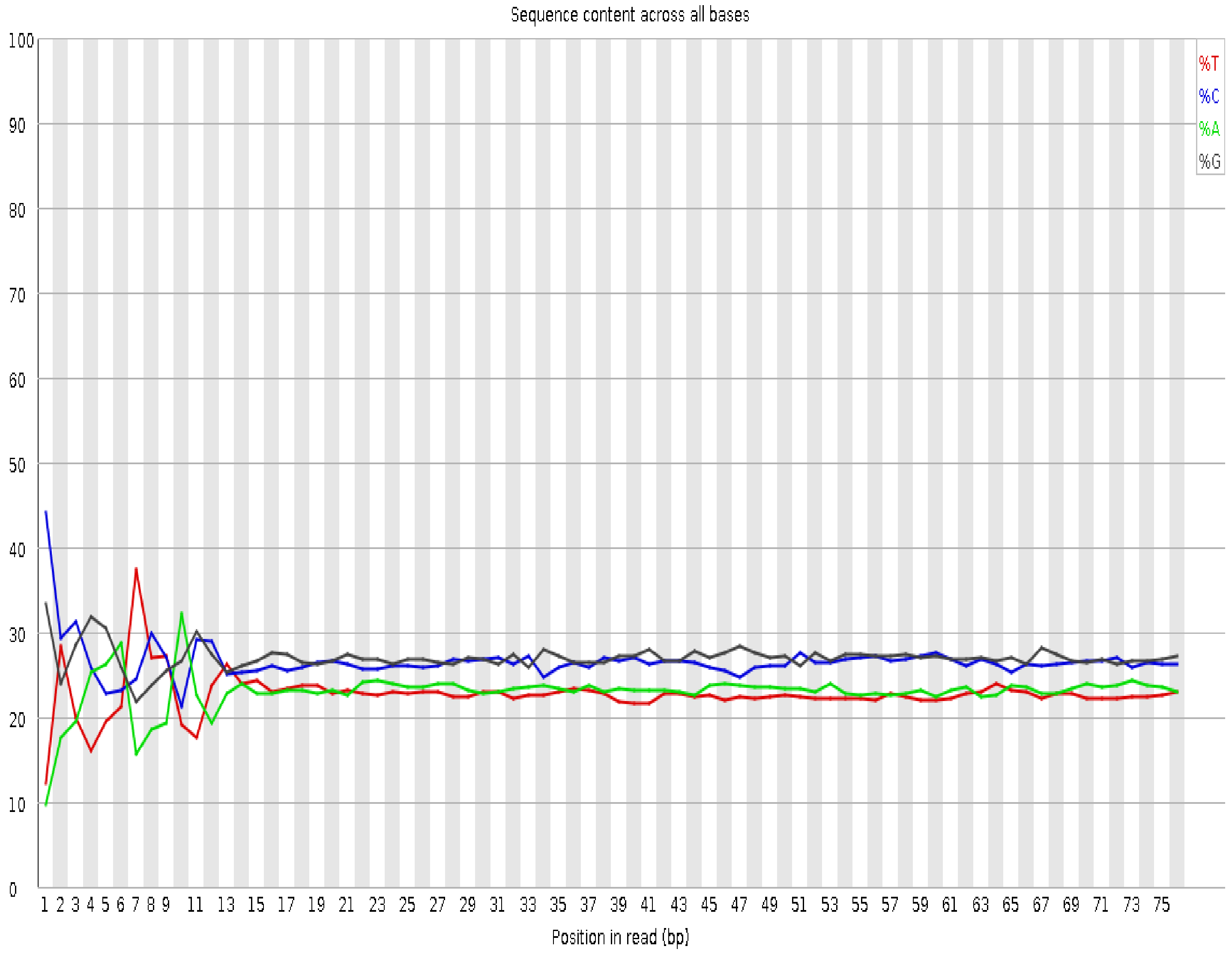


## Per sequence GC content

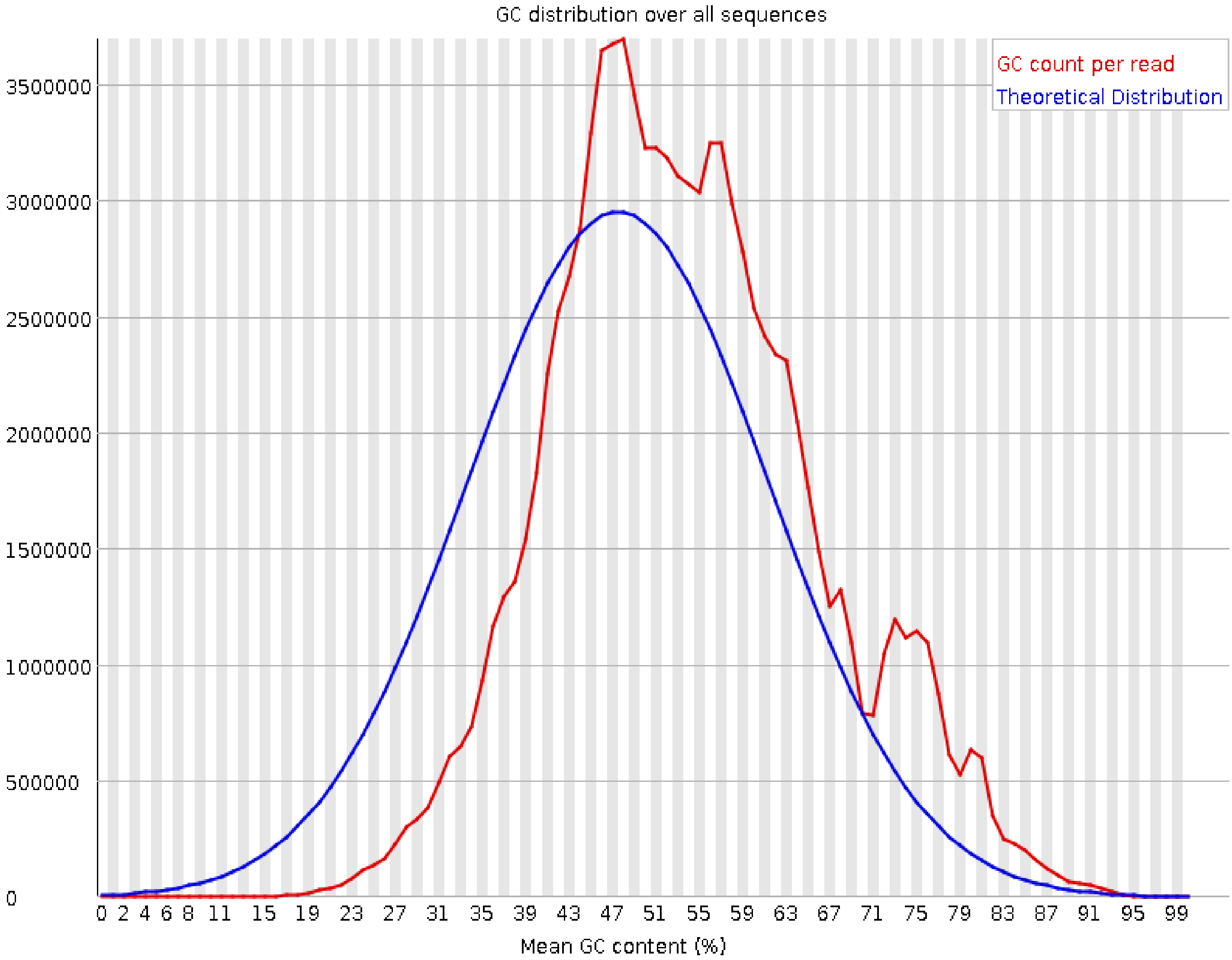


## Per base N content

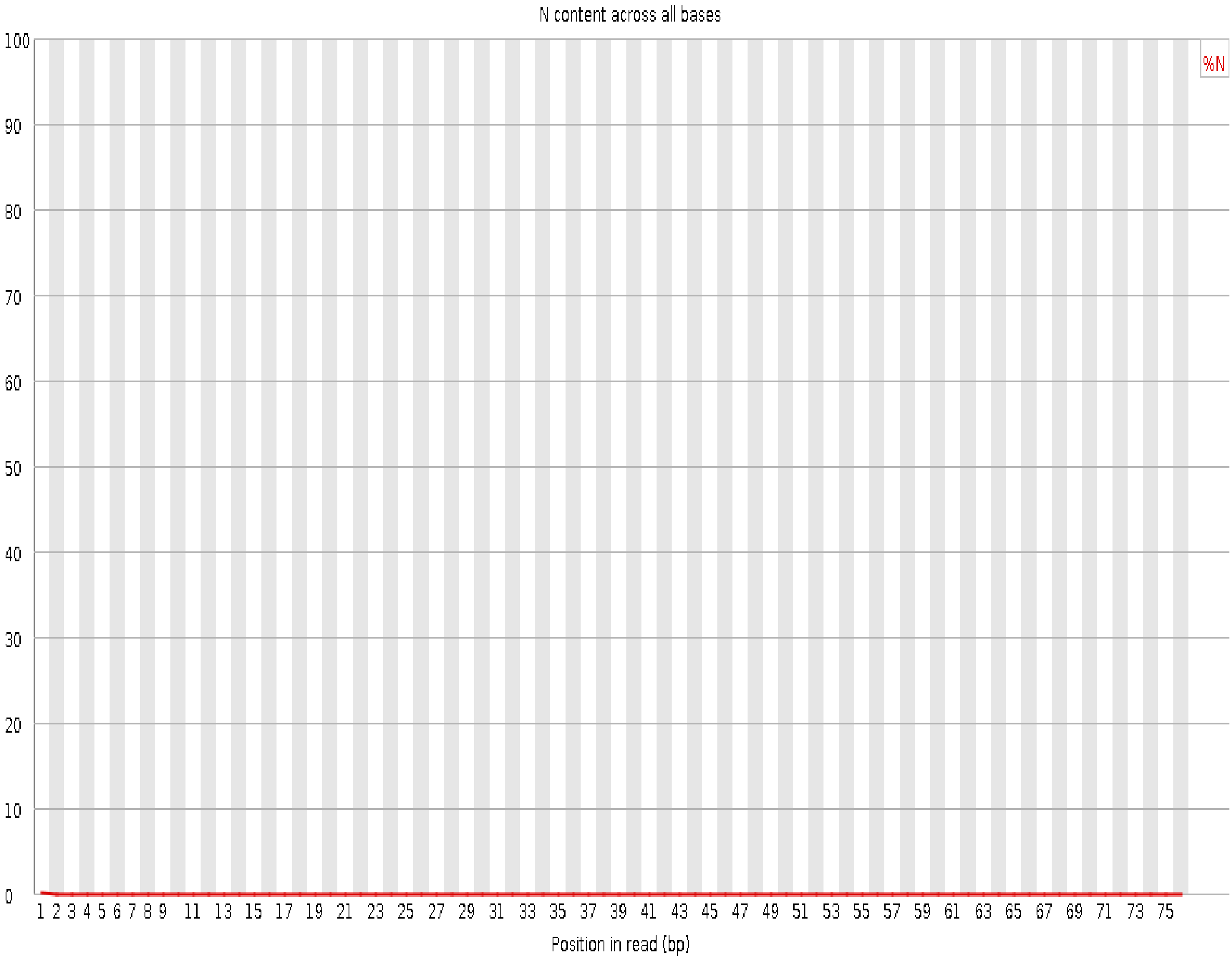


## Sequence Length Distribution

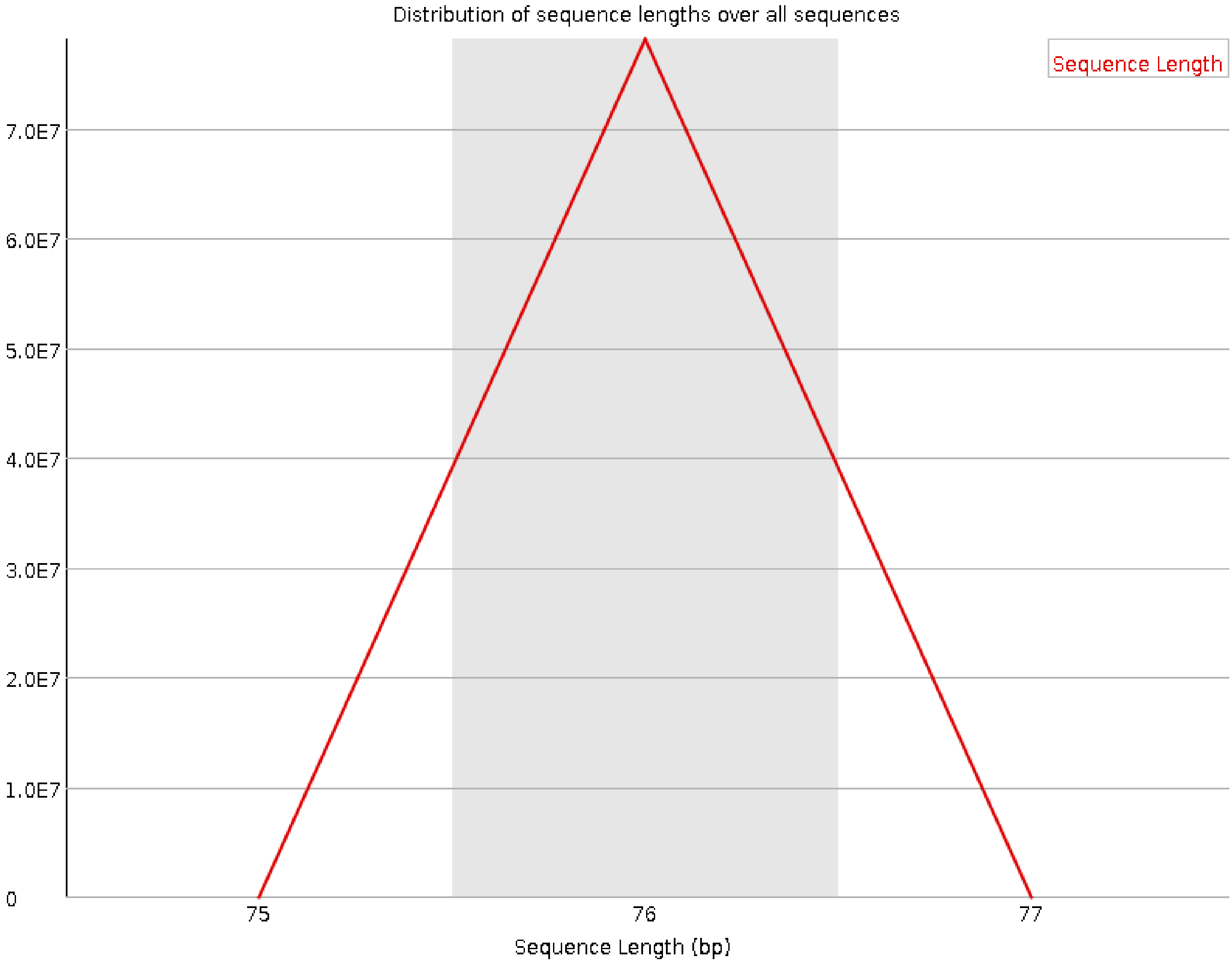


## Sequence Duplication Levels

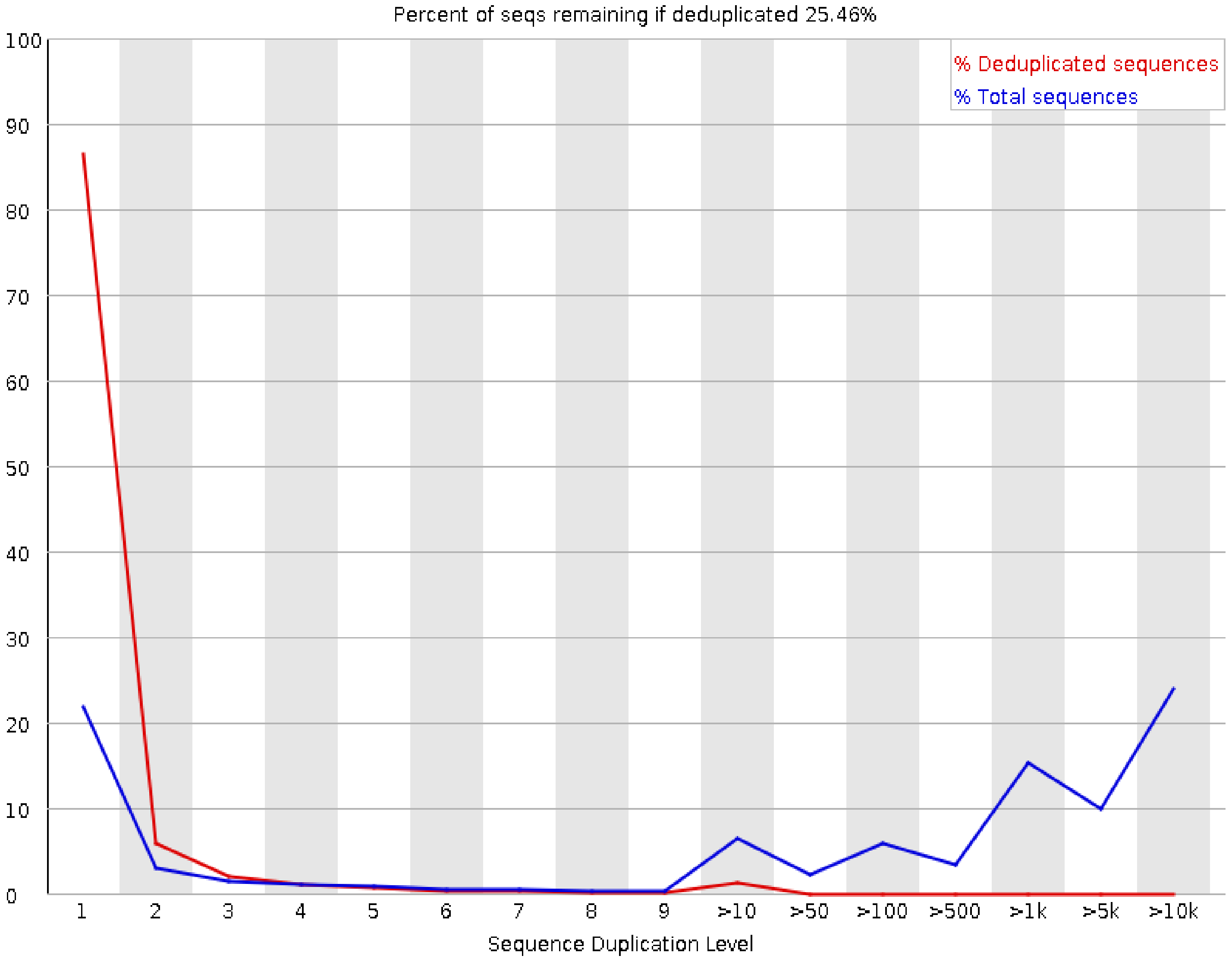


## Overrepresented sequences

| Sequence | Count | Percentage | Possible Source |
|---|---|---|---|
| CCGGTATTTAGCCTTAGATGGAGTTTACCACCCGCTTTGGGCTGCATTCC | 216447 | 0.27700882429631296 | No Hit |
| CCCGTCGGCATGTATTAGCTCTAGAATTACCACAGTTATCCAAGTAGGAG | 180707 | 0.23126877994203585 | No Hit |
| CTGCCAGTAGCATATGCTTGTCTCAAAGATTAAGCCATGCATGTCTAAGT | 158194 | 0.20245664735815666 | No Hit |
| CCGTCGGCATGTATTAGCTCTAGAATTACCACAGTTATCCAAGTAGGAGA | 147702 | 0.18902898800267048 | No Hit |
| CTTCCGTACGCCACATGTCCCGCGCCCCGCCGCGGGGCGGGGATTCGGCG | 147087 | 0.1882419111342351 | No Hit |
| GTCGGCATGTATTAGCTCTAGAATTACCACAGTTATCCAAGTAGGAGAGG | 138576 | 0.1773495351549611 | No Hit |
| GATCGGAAGAGCACACGTCTGAACTCCAGTCACATCACGATCTCGTATGC | 107620 | 0.13773205297726096 | TruSeq Adapter, Index 1 (100% over 50bp) |
| CTCTCTTCAAAGTTCTTTTCAACTTTCCCTTACGGTACTTGTTGACTATC | 104036 | 0.1331452505439725 | No Hit |

| Sequence | Count | Percentage | Possible Source |
|---|---|---|---|
| GCCCTCTTGAACTCTCTCTTCAAAGTTCTTTTCAACTTTCCCTTACGGTA | 96917 | 0.124034355866913 2 | [illegible] |
| GCTGAATTTAAGCATATTAGTCAGCGGAGGAGAAGAAACTAACCAGGATT | 96819 | 0.1239089354878799 | No Hit |
| GAAGAAACTAACCAGGATTCCCTCAGTAACGGCGAGTGAACAGGGAAGAG | 89873 | 0.11501944617380946 | No Hit |
| CGCAGTTTTATCCGGTAAAGCGAATGATTAGAGGTCTTGGGGCCGAAACG | 83821 | 0.10727409786849089 | No Hit |
| CCCGCTTTGGGCTGCATTCCCAAGCAACCCGACTCCGGGAAGACCCGGGC | 82720 | 0.10586503830402366 | No Hit |
| CCGACATCGAAGGATCAAAAAGCGACGTCGCTATGAACGCTTGGCCGCCA | 80077 | 0.10248252746943064 | No Hit |
| CAAAGATTAAGCCATGCATGTCTAAGTACGCACGGCCGGTACAGTGAAAC | 79173 | 0.10132558846281993 | No Hit |

## Adapter Content

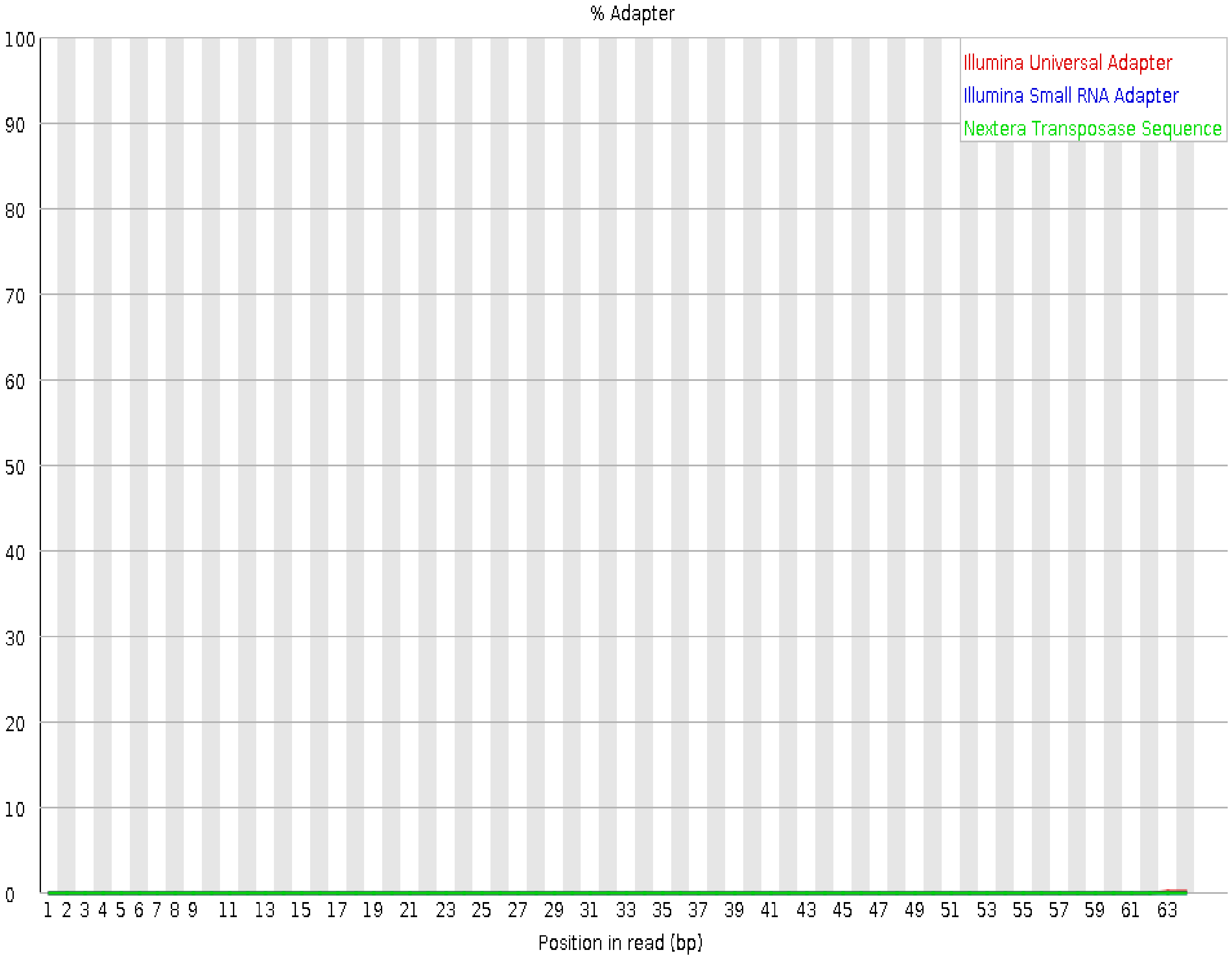


## Kmer Content

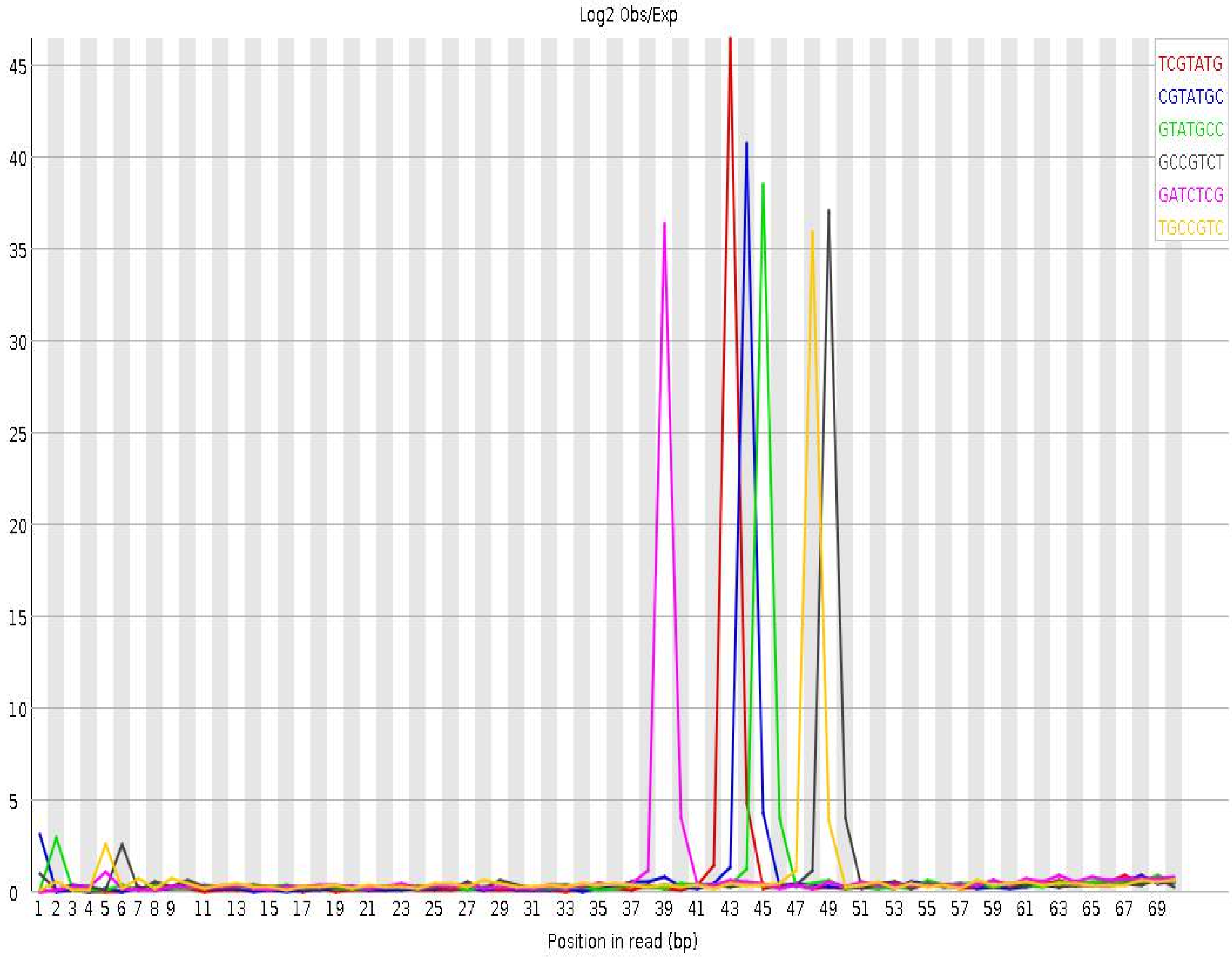


| Sequence | Count | PValue | Obs/Exp Max | Max Obs/Exp Position |
|---|---|---|---|---|
| TCGTATG | 20790 | 0.0 | 46.378635 | 43 |
| CGTATGC | 23700 | 0.0 | 40.713238 | 44 |
| GTATGCC | 25180 | 0.0 | 38.487038 | 45 |
| GCCGTCT | 26040 | 0.0 | 37.029762 | 49 |
| GATCTCG | 24085 | 0.0 | 36.370754 | 39 |
| TGCCGTC | 26705 | 0.0 | 35.88343 | 48 |
| ATGCCGT | 27165 | 0.0 | 35.494774 | 47 |
| CTCGTAT | 26785 | 0.0 | 35.201256 | 42 |
| CATCACG | 28565 | 0.0 | 33.179173 | 33 |
| ACACGTC | 31275 | 0.0 | 31.219368 | 13 |
| CGTCTGA | 31305 | 0.0 | 31.166454 | 16 |
| TCTCGTA | 28405 | 0.0 | 30.864704 | 41 |
| GTCACAT | 32550 | 0.0 | 29.740225 | 29 |
| CACACGT | 33010 | 0.0 | 29.345362 | 12 |
| TCAGACG | 26750 | 0.0 | 29.309858 | 2 |

| Sequence | Count | PValue | Obs/Exp Max | Max Obs/Exp Position |
|---|---|---|---|---|
| CACGTCT | 33645 | 0.0 | 28.[illegible]472 | 14 |
| TGGCGCG | 21170 | 0.0 | 28.4[illegible]7 | 2 |
| CTATCGC | 8340 | 0.0 | 27.113039 | 63 |
| TCGCCTA | 8385 | 0.0 | 27.086977 | 66 |
| TATCGCC | 8415 | 0.0 | 27.033157 | 64 |

**Produced by FastQC (version 0.11.2)**

# FastQC Report

Mon 4 May 2015
Agilent_Adult_Liver_ATCACG_L003_R2.fastq.gz

## Summary


- [PASS] Basic Statistics
- [PASS] Per base sequence quality
- [FAIL] Per tile sequence quality
- [PASS] Per sequence quality scores
- [FAIL] Per base sequence content
- [FAIL] Per sequence GC content
- [PASS] Per base N content
- [PASS] Sequence Length Distribution
- [FAIL] Sequence Duplication Levels
- [WARN] Overrepresented sequences
- [PASS] Adapter Content
- [FAIL] Kmer Content


## Basic Statistics

| Measure | Value |
|---|---|
| Filename | Agilent_Adult_Liver_ATCACG_L003_R2.fastq.gz |
| File type | Conventional base calls |
| Encoding | Sanger / Illumina 1.9 |
| Total Sequences | 78137222 |
| Sequences flagged as poor quality | 0 |
| Sequence length | 76 |
| %GC | 54 |

## Per base sequence quality

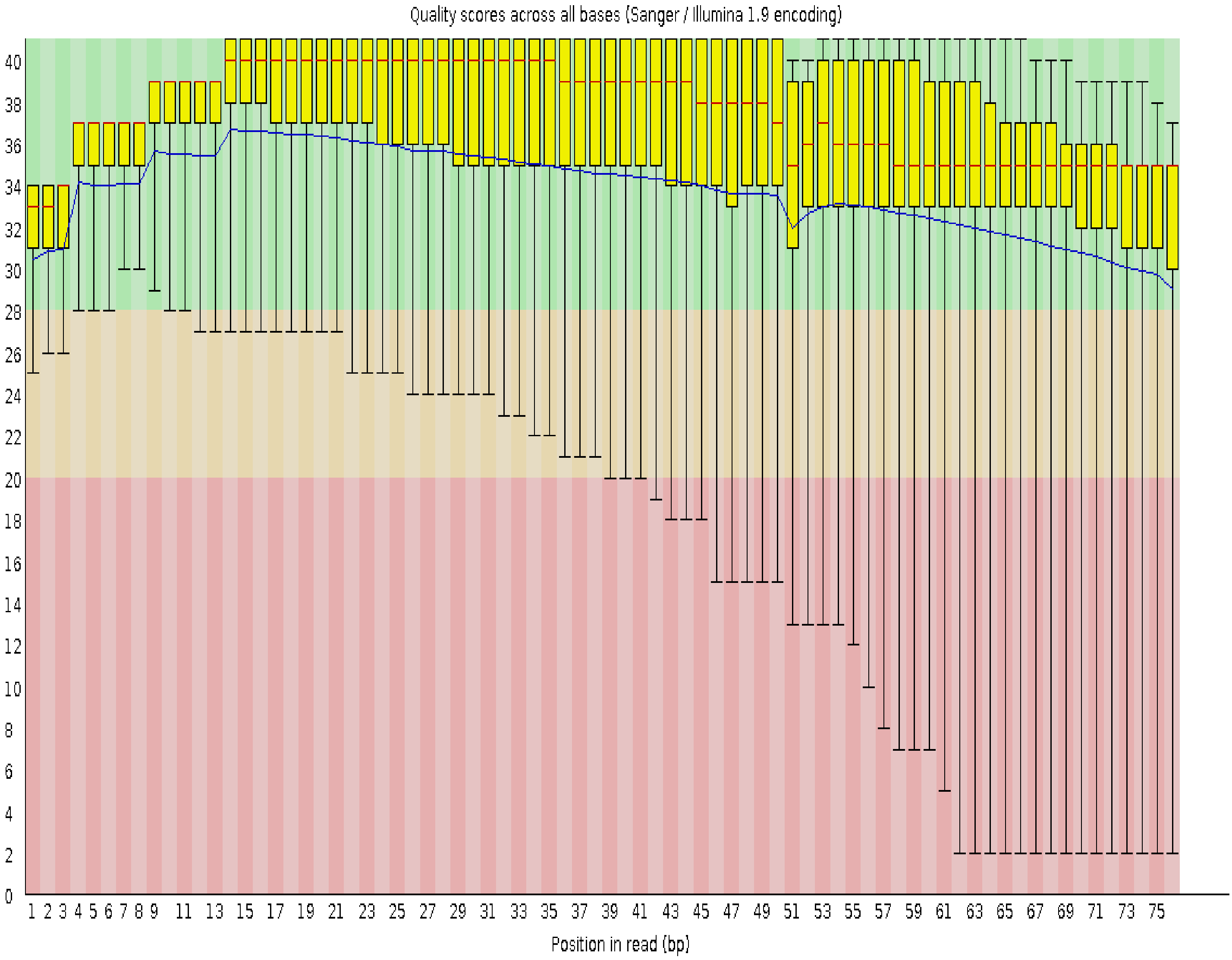


## Per tile sequence quality

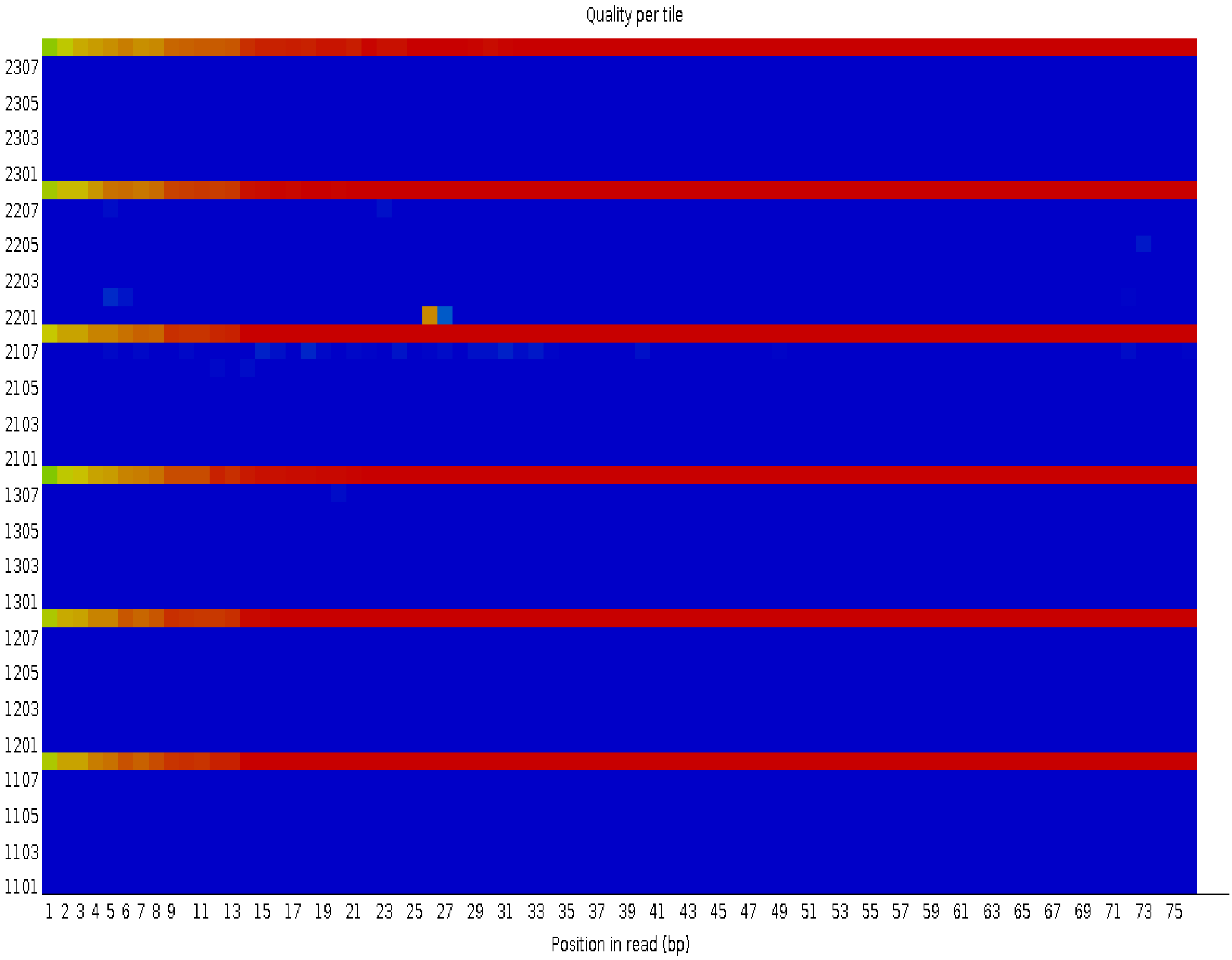


## Per sequence quality scores

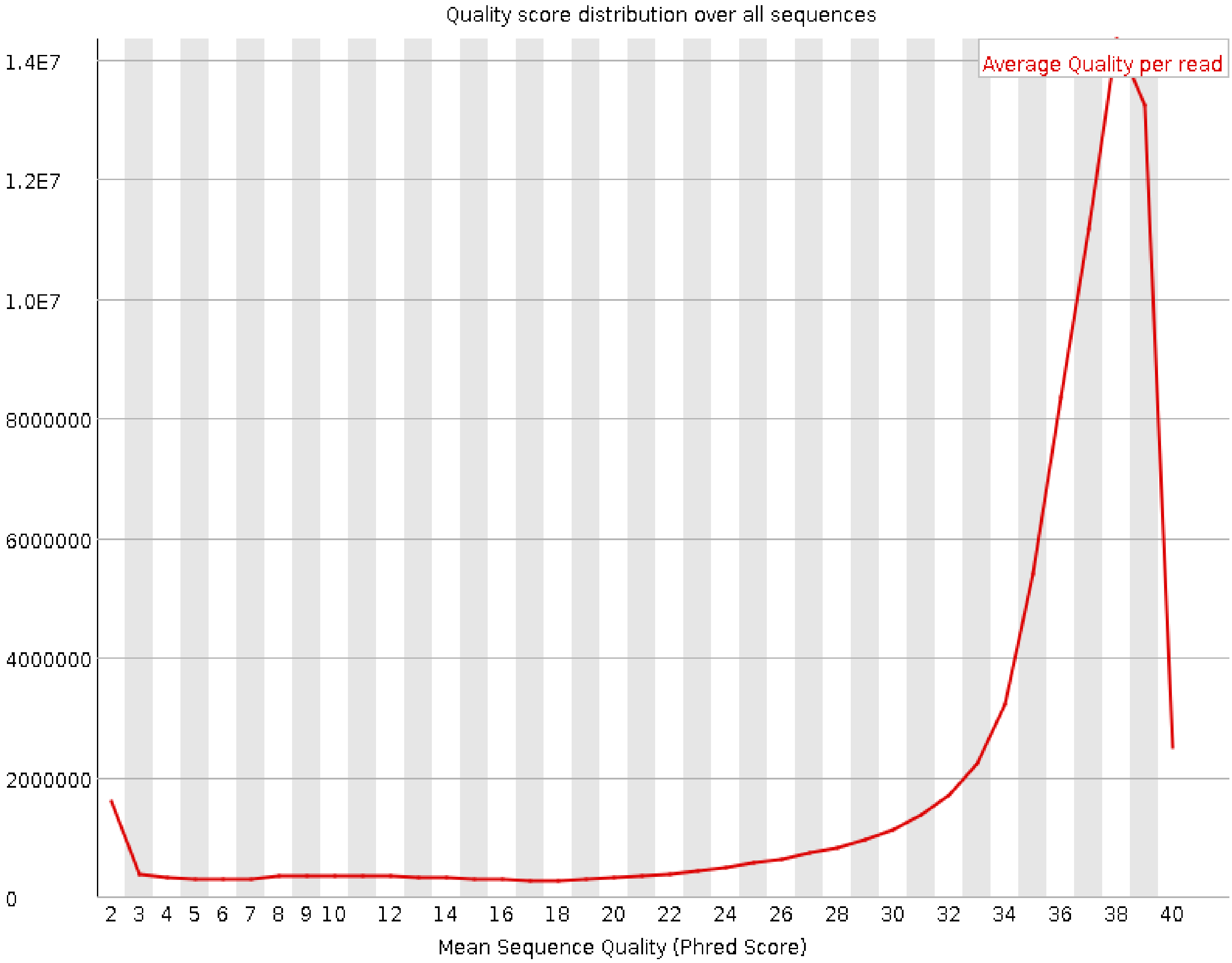


## Per base sequence content

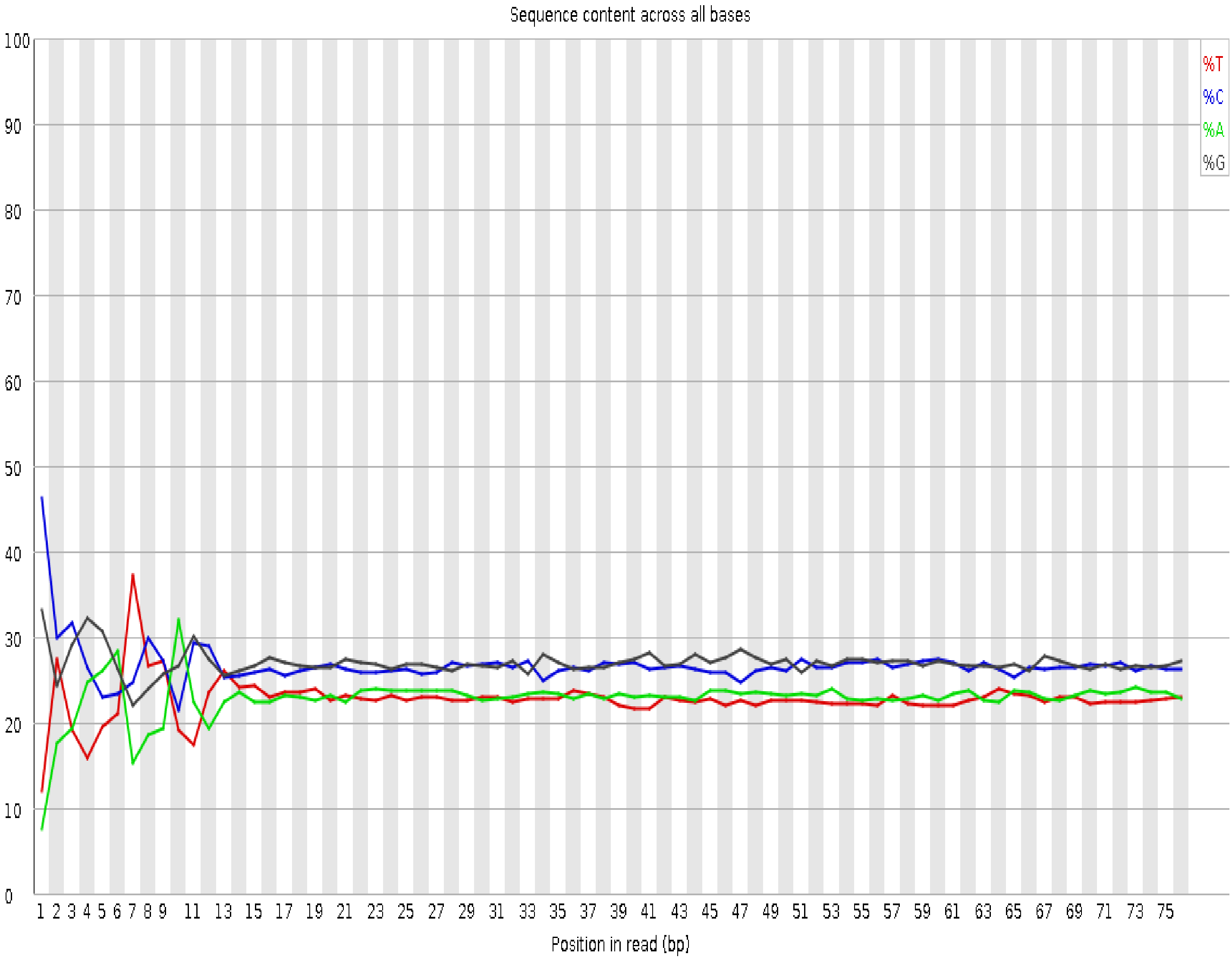


## Per sequence GC content

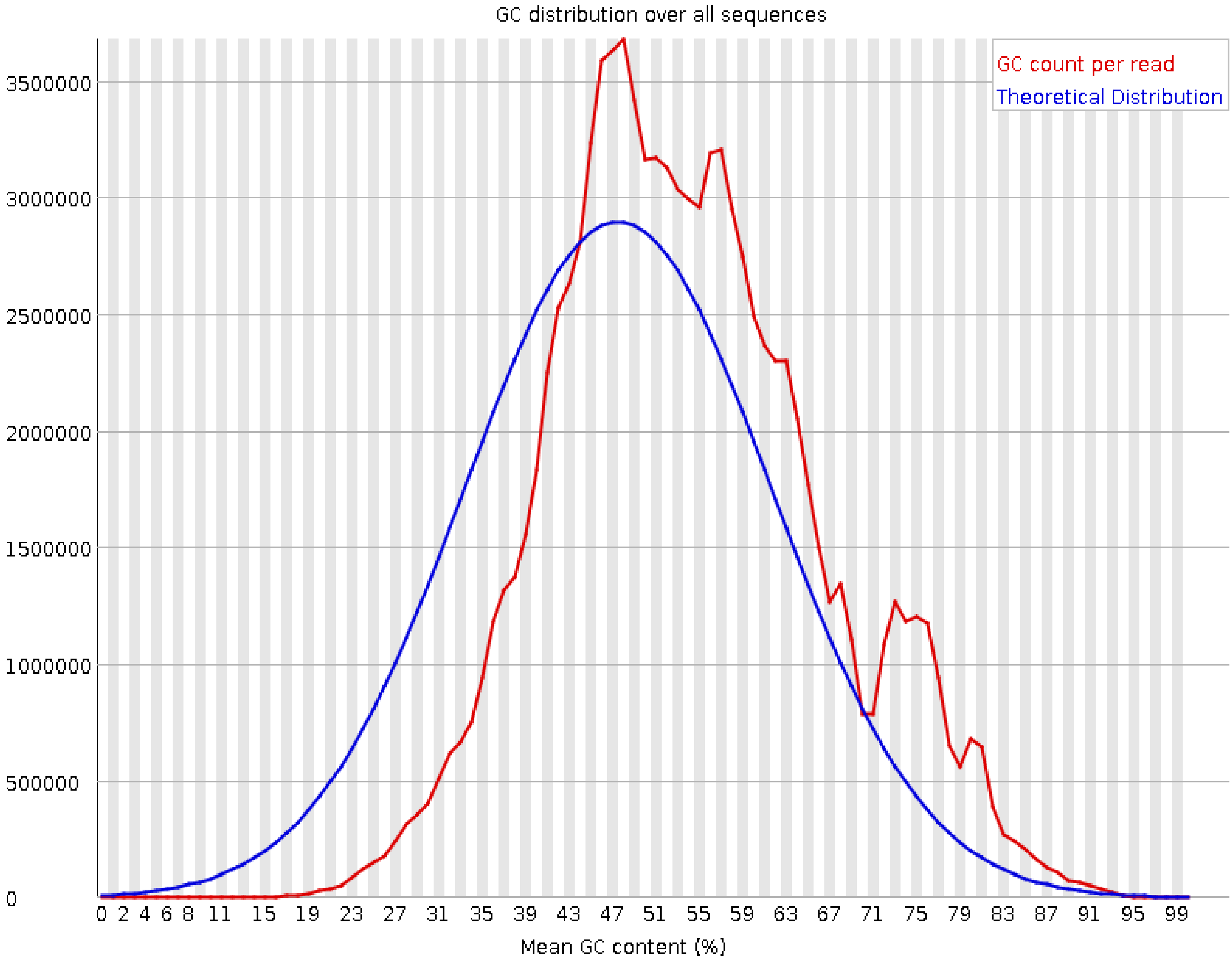


## Per base N content

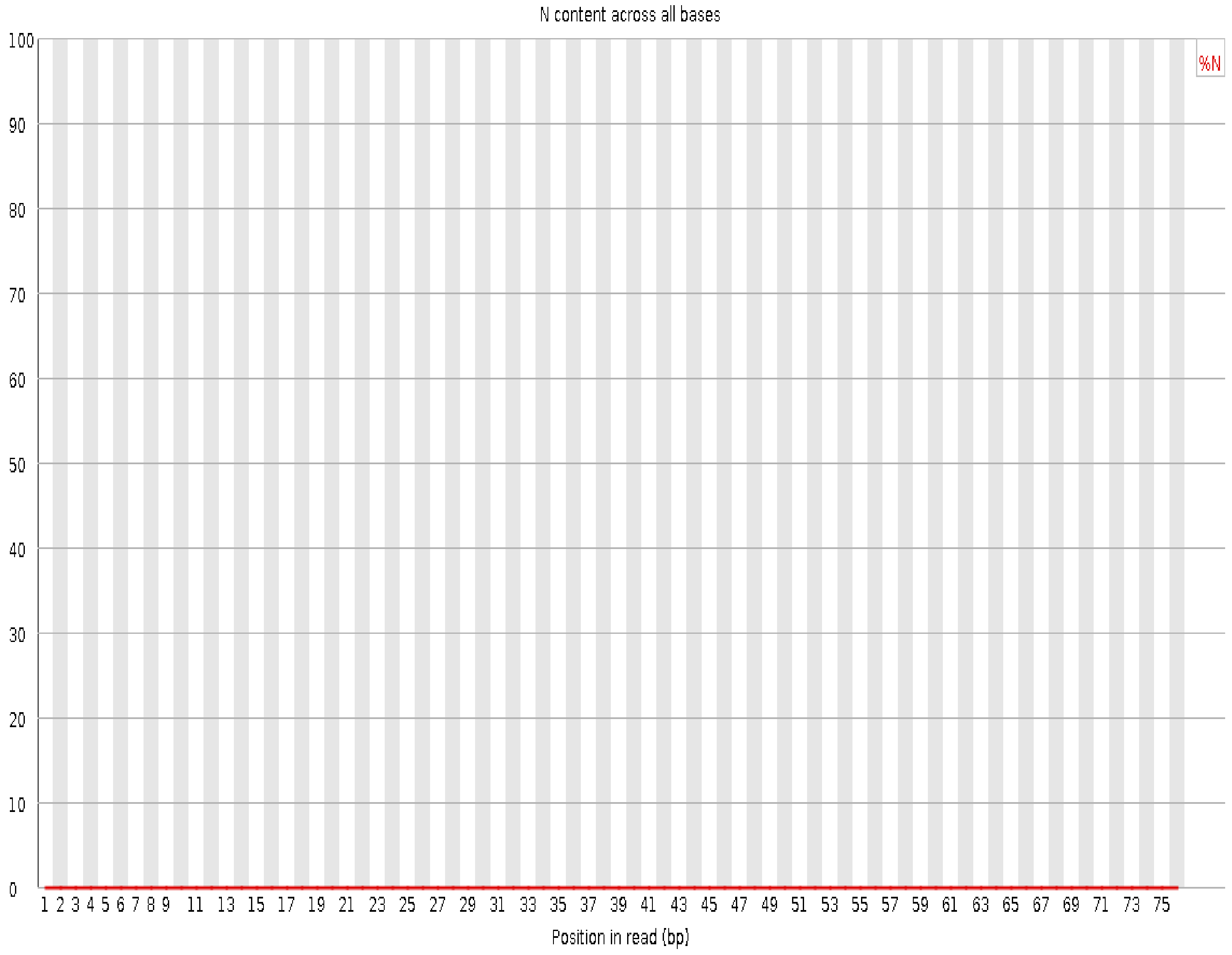


## Sequence Length Distribution

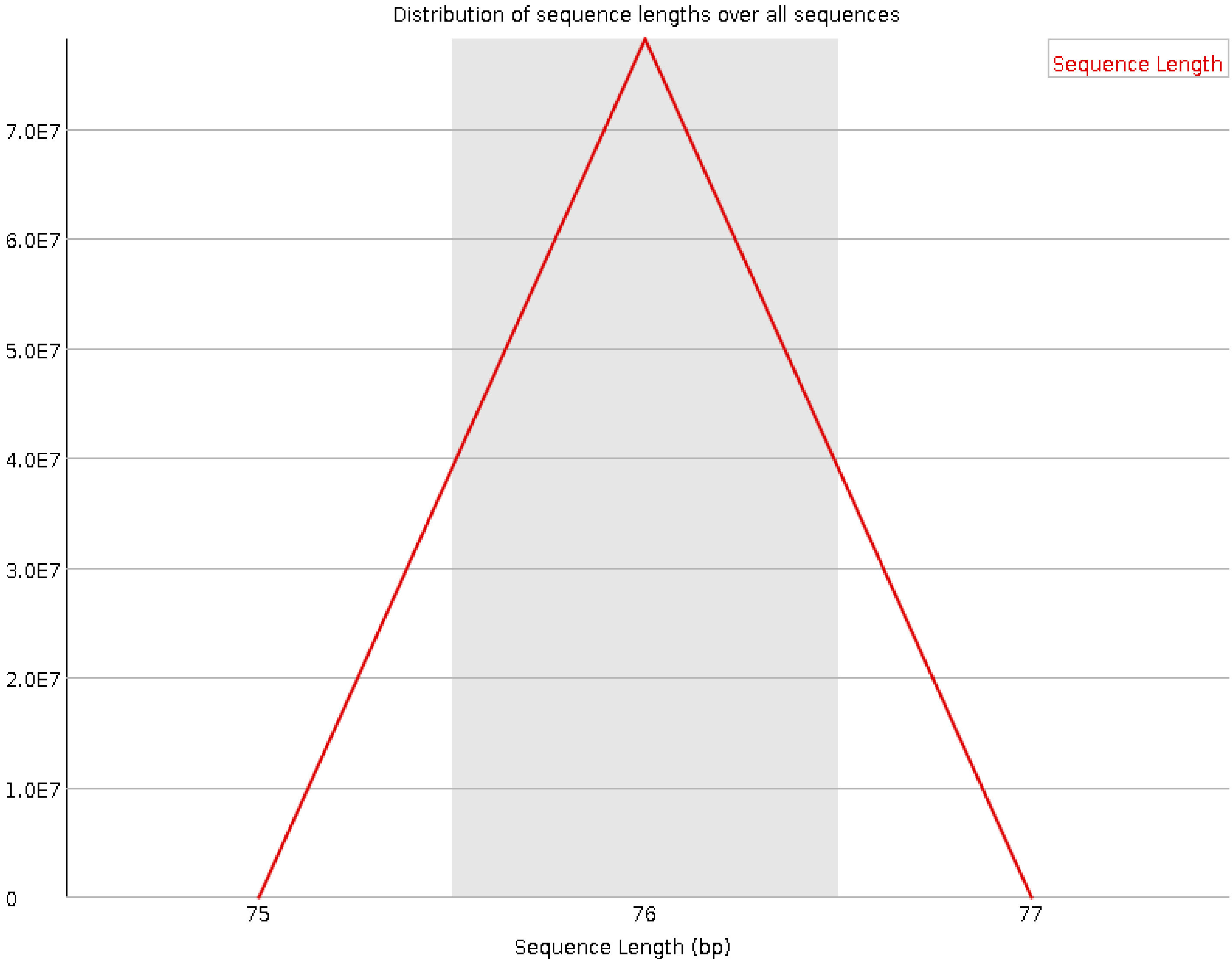


## Sequence Duplication Levels

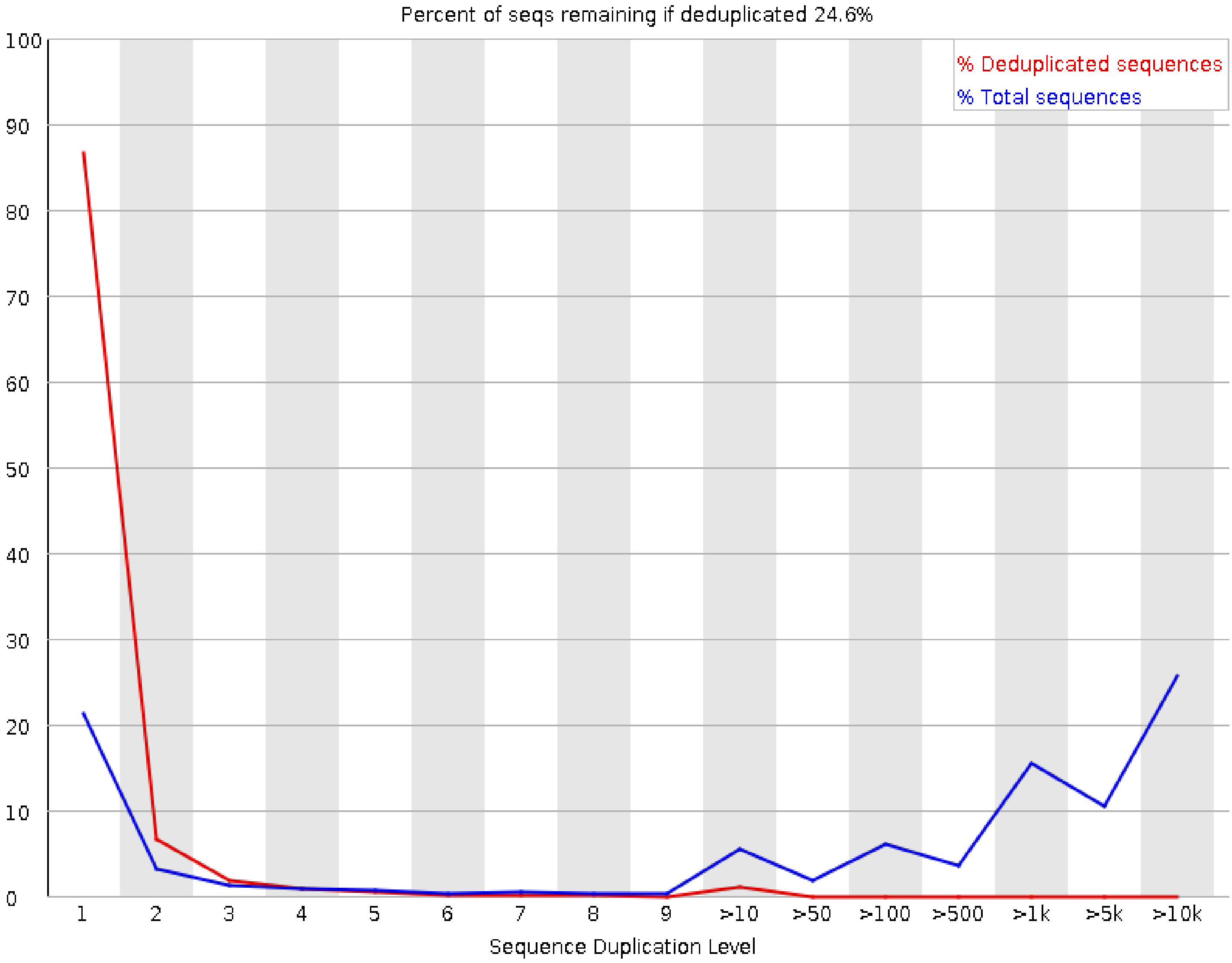


## Overrepresented sequences

| Sequence | Count | Percentage | Possible Source |
|---|---|---|---|
| CCGGTATTTAGCCTTAGATGGAGTTTACCACCCGCTTTGGGCTGCATTCC | 217784 | 0.2787199166102936 | No Hit |
| CCCGTCGGCATGTATTAGCTCTAGAATTACCACAGTTATCCAAGTAGGAG | 208220 | 0.2664799114562839 | No Hit |
| CTGCCAGTAGCATATGCTTGTCTCAAAGATTAAGCCATGCATGTCTAAGT | 170554 | 0.21827497271402868 | No Hit |
| CTTCCGTACGCCACATGTCCCGCGCCCCGCCGCGGGGCGGGGATTCGGCG | 166606 | 0.21322232315860937 | No Hit |
| CCGTCGGCATGTATTAGCTCTAGAATTACCACAGTTATCCAAGTAGGAGA | 161354 | 0.2065008146821498 | No Hit |
| GTCGGCATGTATTAGCTCTAGAATTACCACAGTTATCCAAGTAGGAGAGG | 148850 | 0.1904981981570832 | No Hit |
| GCCCTCTTGAACTCTCTCTTCAAAGTTCTTTTCAACTTTCCCTTACGGTA | 104080 | 0.13320156173455974 | No Hit |
| CTCTCTTCAAAGTTCTTTTCAACTTTCCCTTACGGTACTTGTTGACTATC | 103357 | 0.13227626648922838 | No Hit |
| CGCAGTTTTATCCGGTAAAGCGAATGATTAGAGGTCTTGGGGCCGAAACG | 91337 | 0.11689307306062147 | No Hit |
| CGGGTCTTCCGTACGCCACATGTCCCGCGCCCCGCCGCGGGGCGGGGATT | 91043 | 0.11651681192351579 | No Hit |
| CCCGCTTTGGGCTGCATTCCCAAGCAACCCGACTCCGGGAAGACCCGGGC | 90096 | 0.11530484152610392 | No Hit |

| Sequence | Count | Percentage | Possible Source |
|---|---|---|---|
| GCTGAATTTAAGCATATTAGTCAGCGGAGGAGAAGAAACTAACCAGGATT | 89112 | 0.1140455185366073 | No Hit |
| GAAGAAACTAACCAGGATTCCCTCAGTAACGGCGAGTGAACAGGGAAGAG | 85761 | 0.10975690945347404 | No Hit |
| CCGACATCGAAGGATCAAAAAGCGACGTCGCTATGAACGCTTGGCCGCCA | 85612 | 0.10956621928534904 | No Hit |
| GCGGGTCTTCCGTACGCCACATGTCCCGCGCCCCGCCGCGGGGCGGGGAT | 85212 | 0.10905429937091954 | No Hit |
| CAAAGATTAAGCCATGCATGTCTAAGTACGCACGGCCGGTACAGTGAAAC | 79780 | 0.10210242693296673 | No Hit |
| GGCGGGAGTAACTATGACTCTCTTAAGGTAGCCAAATGCCTCGTCATCTA | 79664 | 0.10195397015778218 | No Hit |
| GCTGGATAGTAGGTAGGGACAGTGGGAATCTCGTTCATCCATTCATGCGC | 78809 | 0.10085974134068908 | No Hit |
| CCCTCCTTAGGCAACCTGGTGGTCCCCCGCTCCCGGGAGGTCACCATATT | 78540 | 0.10051547519823523 | No Hit |

# Adapter Content

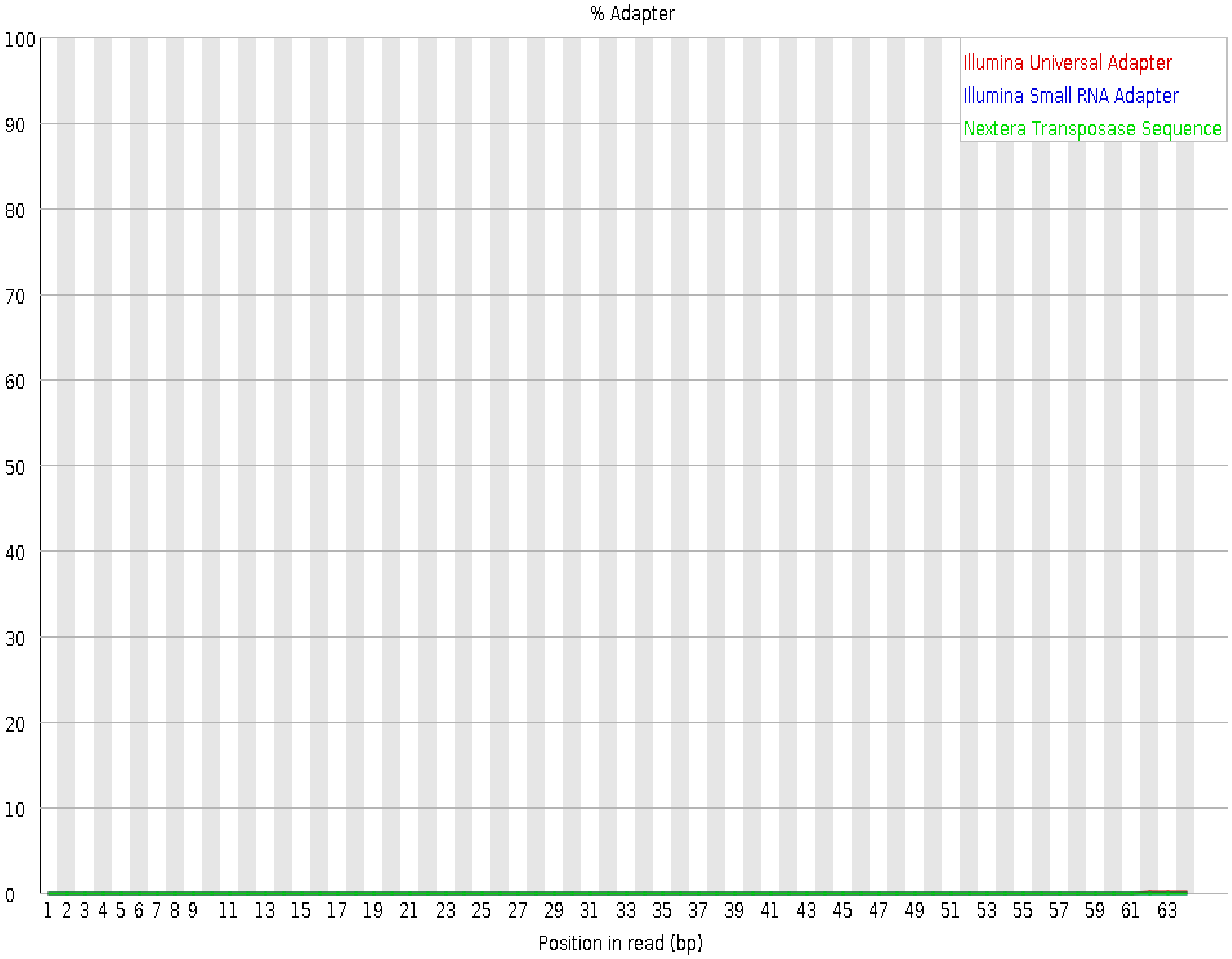


# Kmer Content

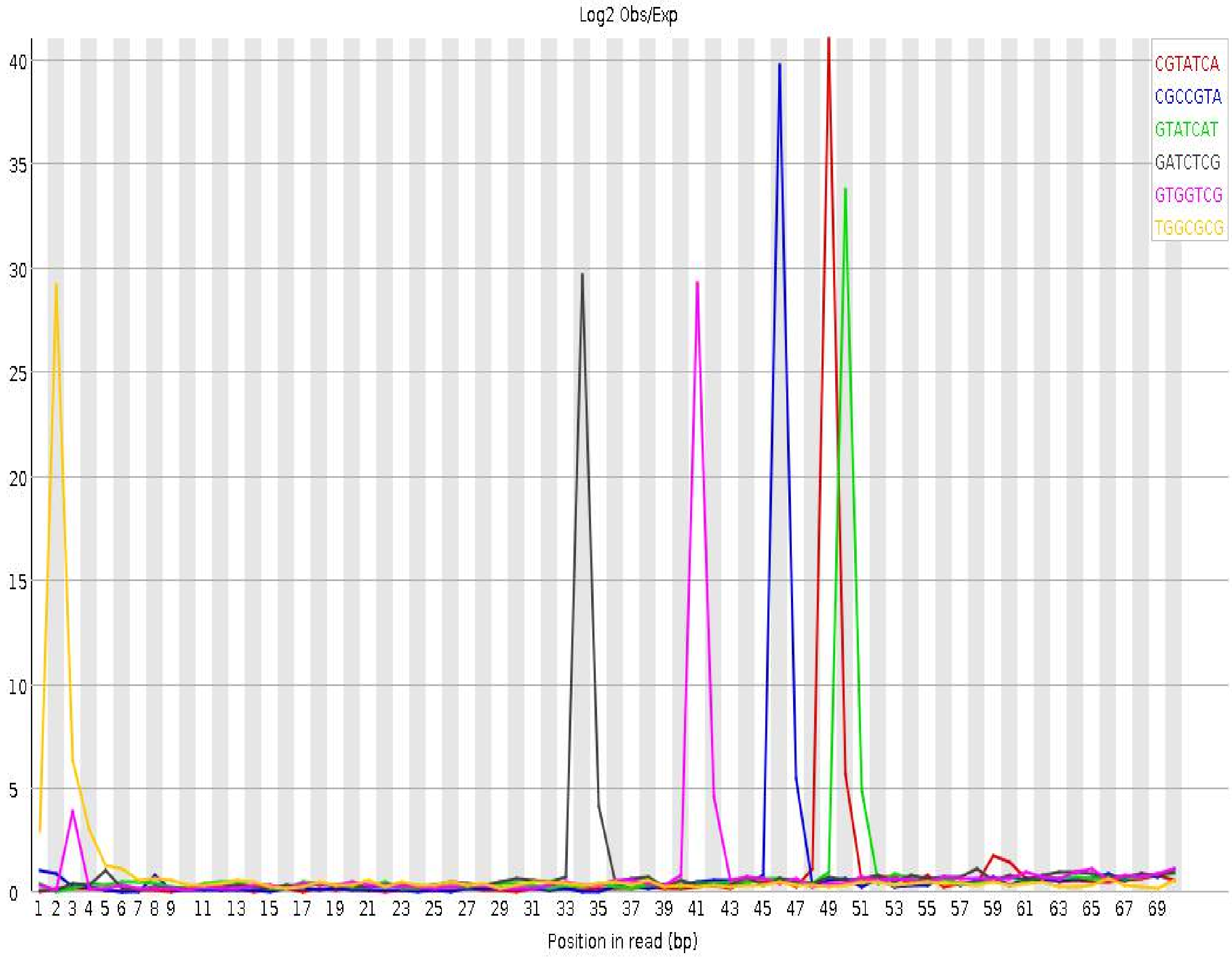


| Sequence | Count | PValue | Obs/Exp Max | Max Obs/Exp Position |
|---|---|---|---|---|
| CGTATCA | 17160 | 0.0 | 40.951572 | 49 |
| CGCCGTA | 17665 | 0.0 | 39.76039 | 46 |
| GTATCAT | 20970 | 0.0 | 33.744736 | 50 |
| GATCTCG | 25880 | 0.0 | 29.652456 | 34 |
| GTGGTCG | 20925 | 0.0 | 29.233768 | 41 |
| TGGCGCG | 19870 | 0.0 | 29.196468 | 2 |
| TCTCGGT | 23360 | 0.0 | 28.731863 | 36 |
| CTATCGC | 7525 | 0.0 | 28.506939 | 63 |
| TATCGCC | 7470 | 0.0 | 28.484312 | 64 |
| ATCATTA | 27005 | 0.0 | 28.341486 | 52 |
| TCGGTGG | 25175 | 0.0 | 27.75868 | 38 |
| GTAGATC | 25610 | 0.0 | 27.684204 | 31 |
| TCGCCTA | 7610 | 0.0 | 27.639244 | 66 |
| CTCGCTA | 11245 | 0.0 | 27.117773 | 1 |
| TCAGACG | 22770 | 0.0 | 26.984835 | 2 |

| Sequence | Count | PValue | Obs/Exp Max | Max Obs/Exp Position |
|---|---|---|---|---|
| TGCCAGT | 56260 | 0.0 | [illegible] | 2 |
| CTGCCAG | 58225 | 0.0 | [illegible] | 1 |
| TAGATCT | 27210 | 0.0 | 26.197205 | 32 |
| GCCAGTA | 57240 | 0.0 | 26.19323 | 3 |
| TTGGCCG | 27330 | 0.0 | 25.993528 | 41 |

**Produced by FastQC (version 0.11.2)**

# FastQC Report

Mon 4 May 2015
Agilent_Adult_Lung_ATCACG_L002_R1.fastq.gz

## Summary


- Basic Statistics
- Per base sequence quality
- Per tile sequence quality
- Per sequence quality scores
- Per base sequence content
- Per sequence GC content
- Per base N content
- Sequence Length Distribution
- Sequence Duplication Levels
- Overrepresented sequences
- Adapter Content
- Kmer Content


## Basic Statistics

| Measure | Value |
|---|---|
| Filename | Agilent_Adult_Lung_ATCACG_L002_R1.fastq.gz |
| File type | Conventional base calls |
| Encoding | Sanger / Illumina 1.9 |
| Total Sequences | 77032217 |
| Sequences flagged as poor quality | 0 |
| Sequence length | 76 |
| %GC | 57 |

## Per base sequence quality

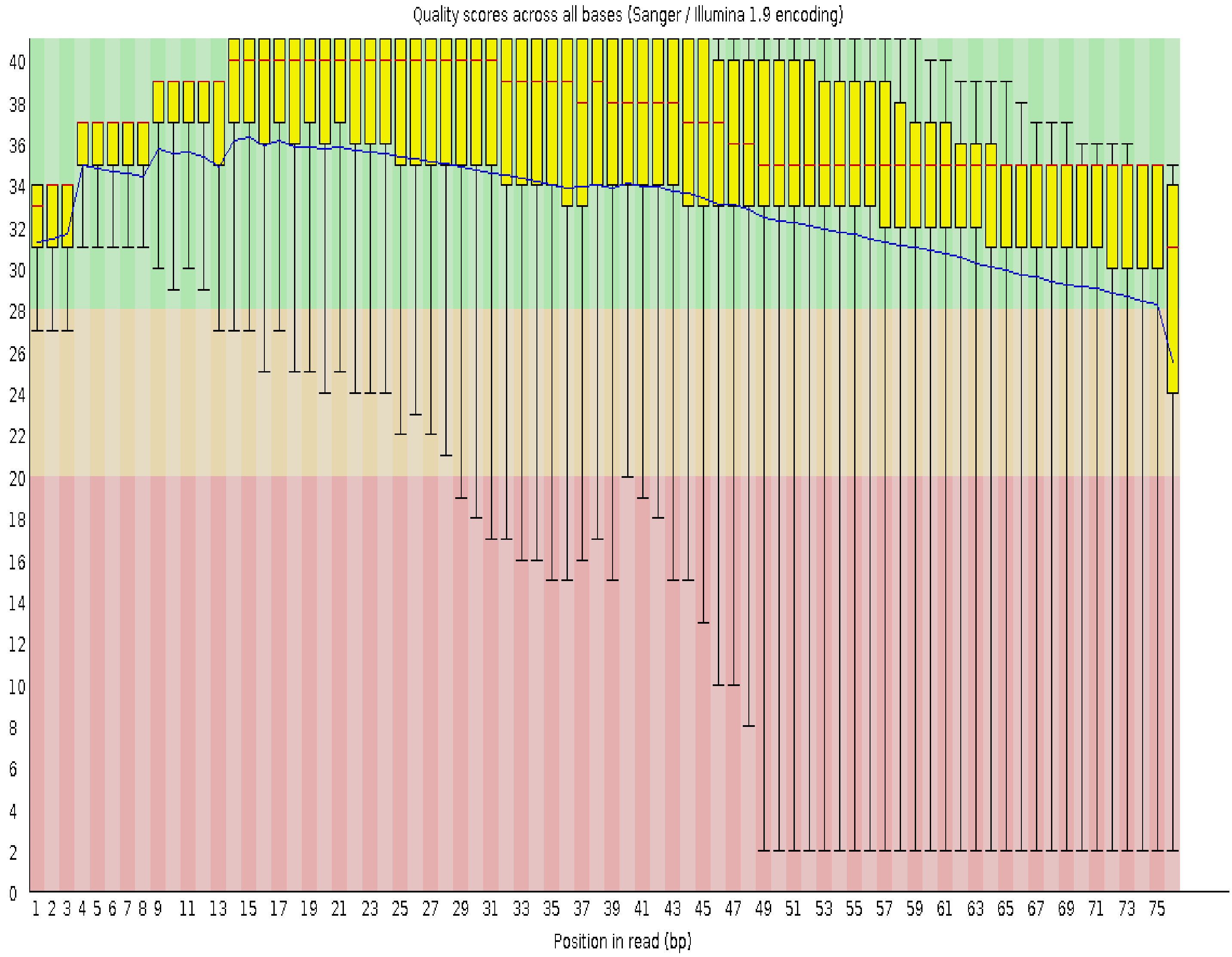


## Per tile sequence quality

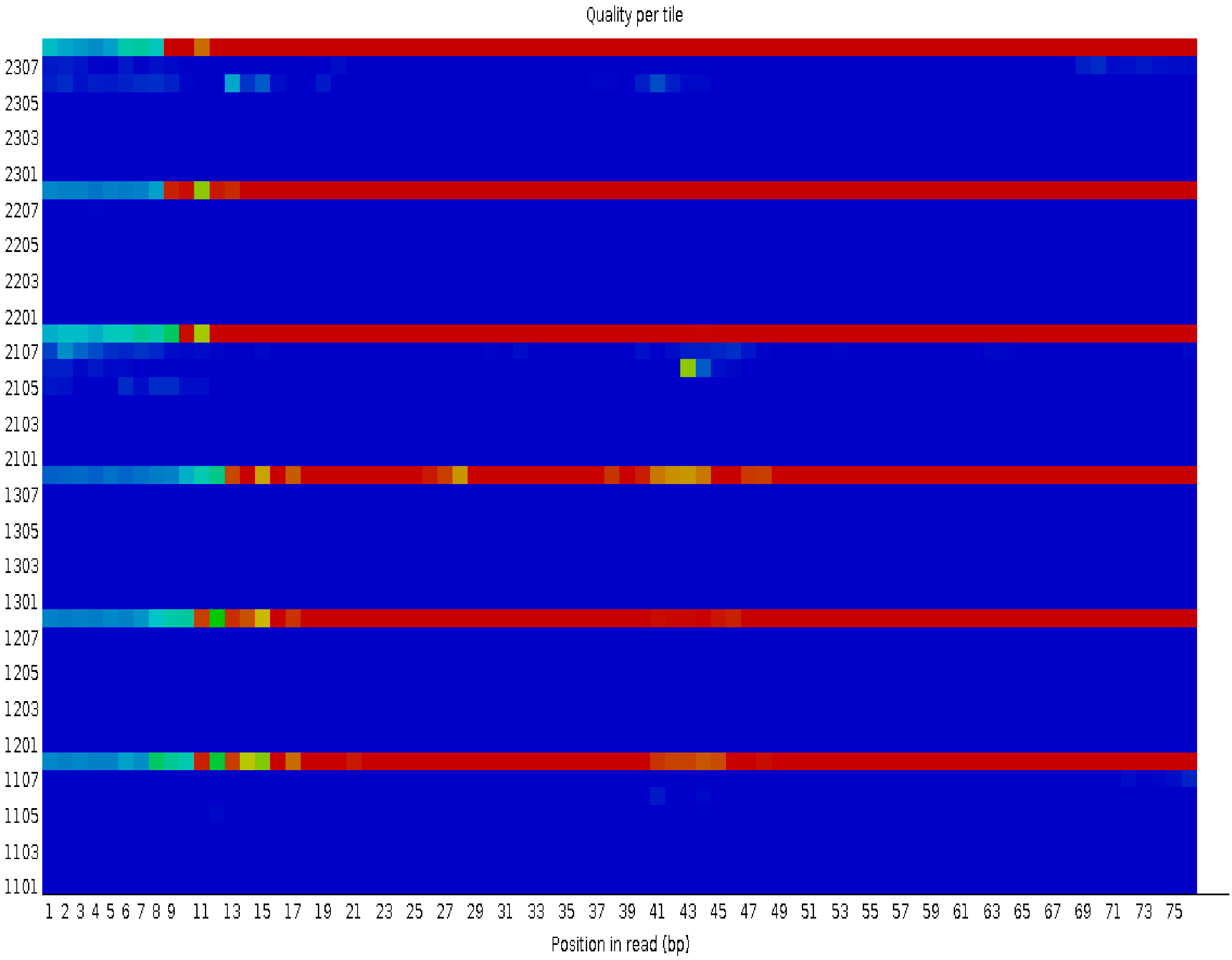


## Per sequence quality scores

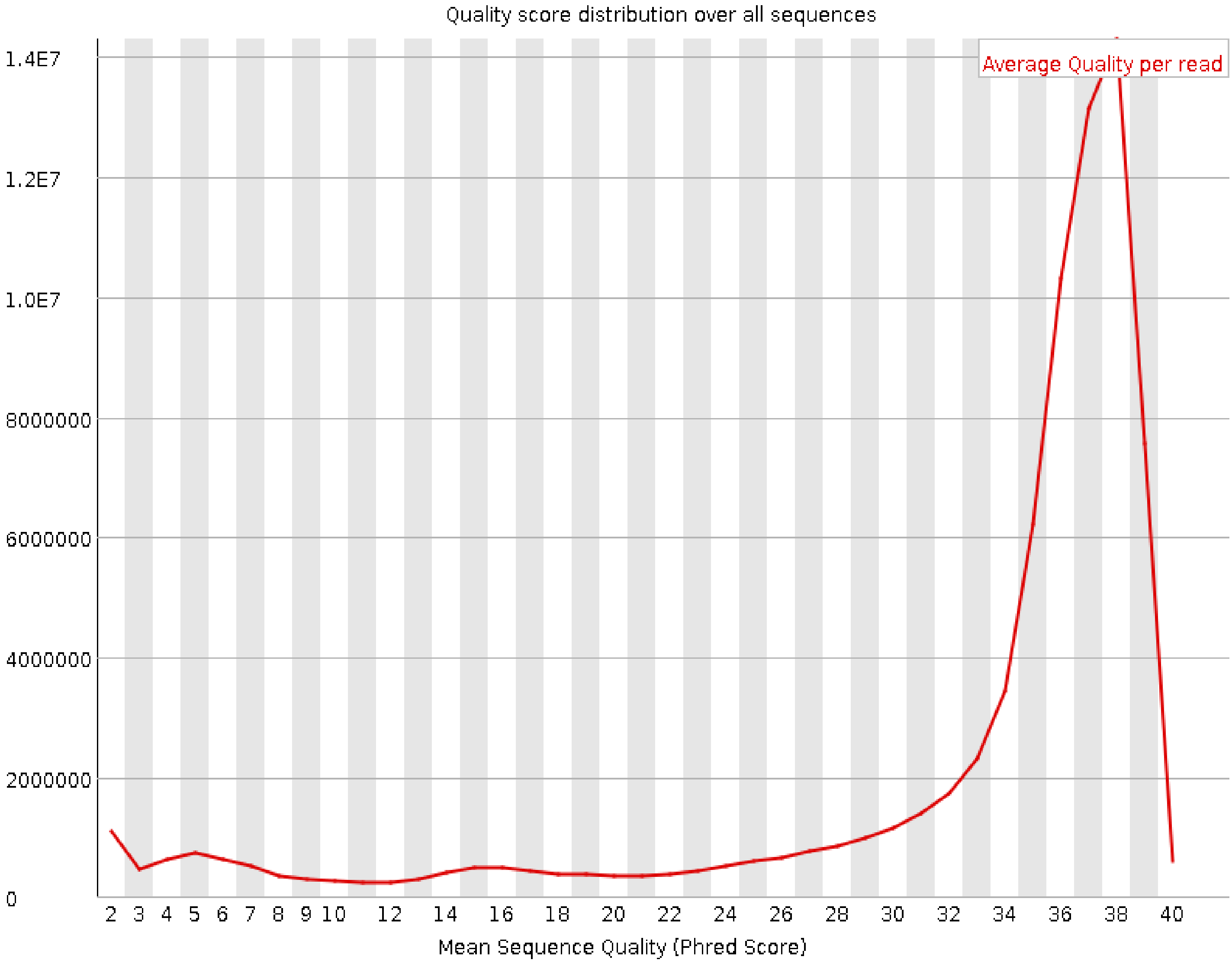


## Per base sequence content

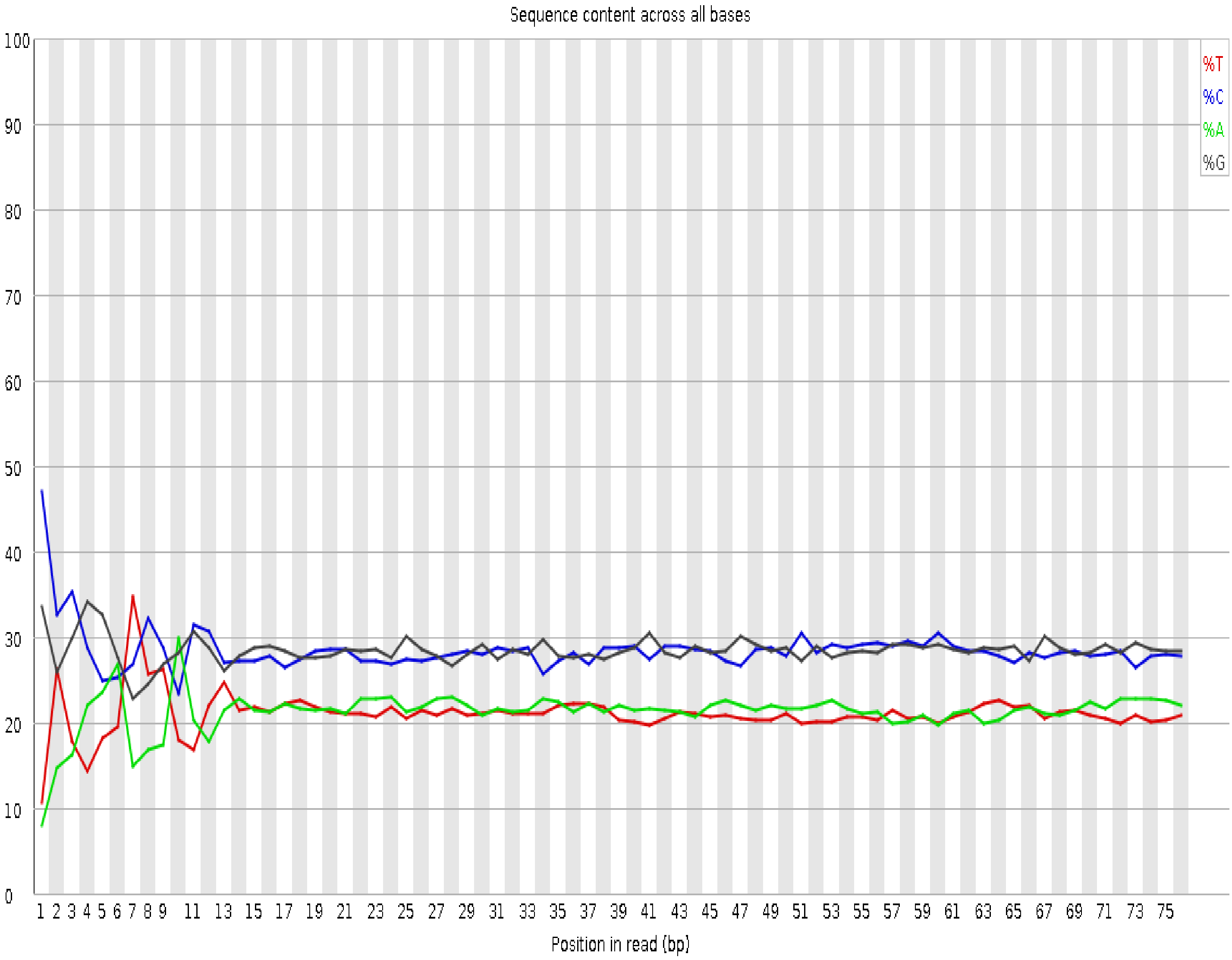


## Per sequence GC content

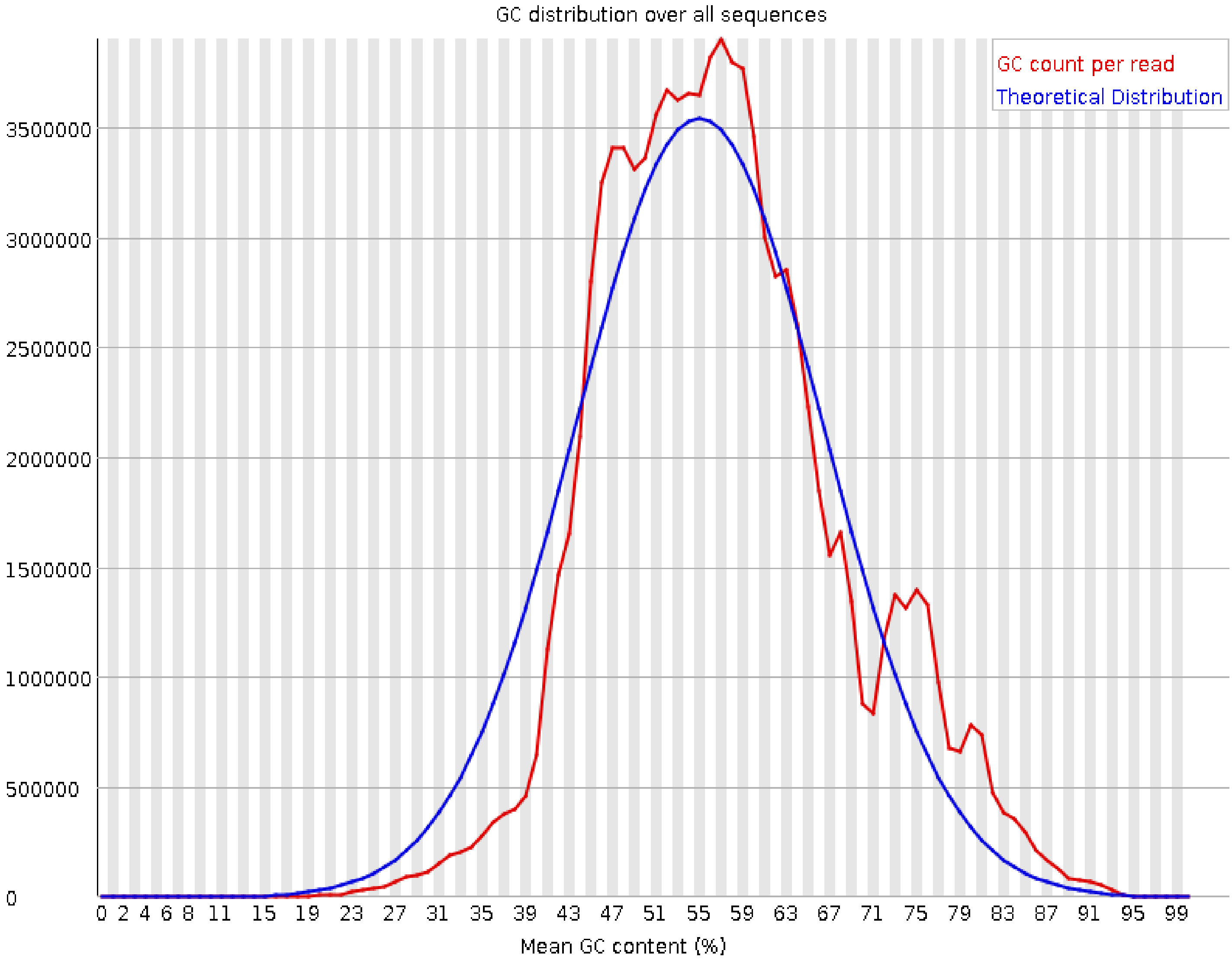


## Per base N content

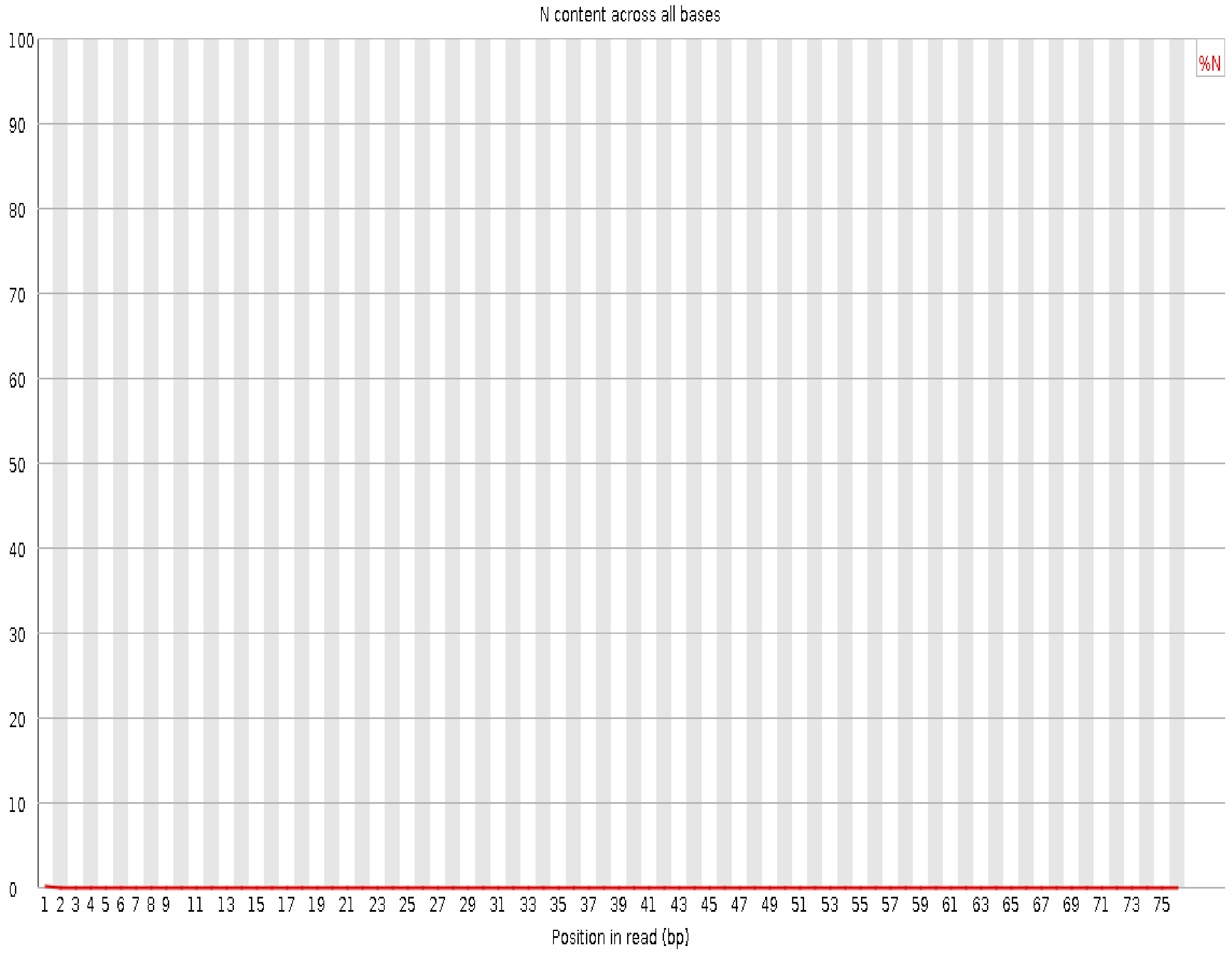


## Sequence Length Distribution

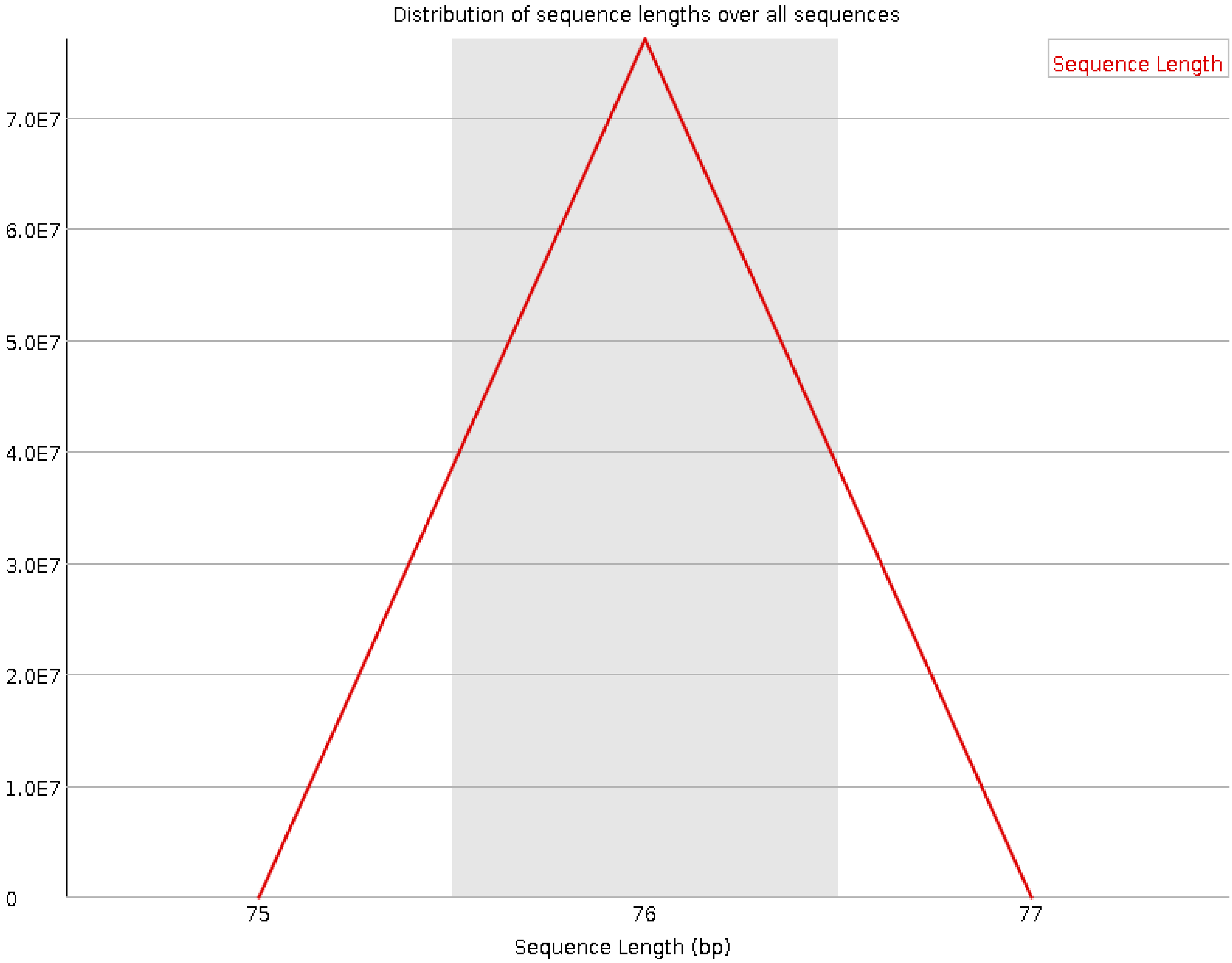


## Sequence Duplication Levels

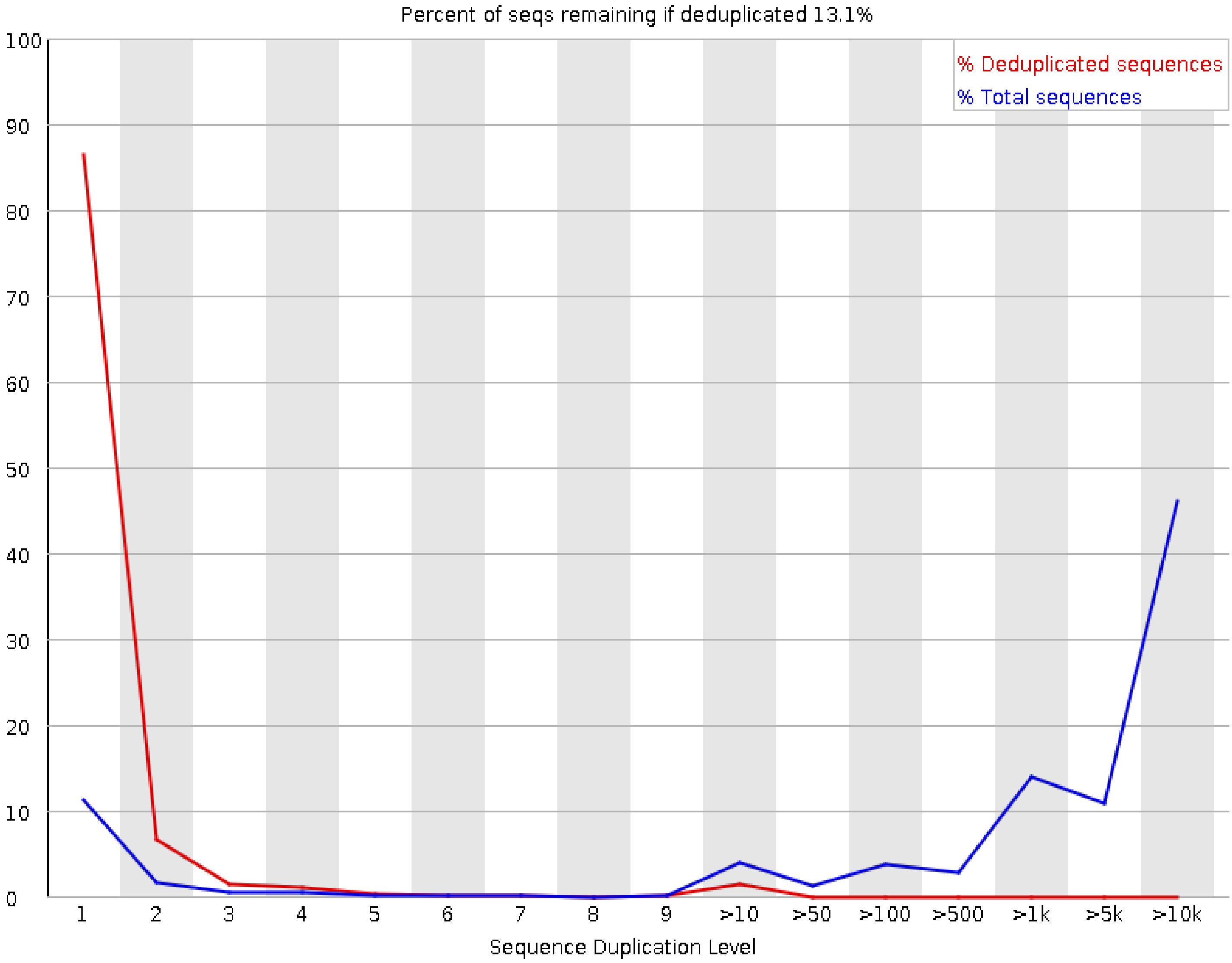


## Overrepresented sequences

| Sequence | Count | Percentage | Possible Source |
|---|---|---|---|
| CGCGTAACTAGTTAGCATGCCAGAGTCTCGTTCGTTATCGGAATTAACCA | 338618 | 0.439579715069086 | No Hit |
| CCTCACCCGGCCCGGACACGGACAGGATTGACAGATTGATAGCTCTTTCT | 306478 | 0.397856912257893 3 | No Hit |
| CTTCCGTACGCCACATGTCCCGCGCCCCGCCGCGGGGCGGGGATTCGGCG | 207324 | 0.2691393394532576 | No Hit |
| CCGGTATTTAGCCTTAGATGGAGTTTACCACCCGCTTTGGGCTGCATTCC | 204804 | 0.2658679809254354 | No Hit |
| CCCGTCGGCATGTATTAGCTCTAGAATTACCACAGTTATCCAAGTAGGAG | 181267 | 0.23531323264394688 | No Hit |
| GCCCTCTTGAACTCTCTCTTCAAAGTTCTTTTCAACTTTCCCTTACGGTA | 160624 | 0.20851535403687005 | No Hit |
| CGCAGTTTTATCCGGTAAAGCGAATGATTAGAGGTCTTGGGGCCGAAACG | 160398 | 0.2082219702958828 | No Hit |
| CTCACCCGGCCCGGACACGGACAGGATTGACAGATTGATAGCTCTTTCTC | 160327 | 0.20812980106751955 | No Hit |
| CTCCGACTTTCGTTCTTGATTAATGAAAACATTCTTGGCAAATGCTTTCG | 157786 | 0.20483118121863217 | No Hit |
| CTGCCAGTAGCATATGCTTGTCTCAAAGATTAAGCCATGCATGTCTAAGT | 153557 | 0.19934127041936234 | No Hit |
| GCTGAATTTAAGCATATTAGTCAGCGGAGGAGAAGAAACTAACCAGGATT | 153085 | 0.19872853977446867 | No Hit |

| Sequence | Count | Percentage | Possible Source |
|---|---|---|---|
| GATCGGAAGAGCACACGTCTGAACTCCAGTCACATCACGATCTCGTATGC | 150349 | 0.19517677908711883 | TruSeq Adapter, Index [illegible] (100% over 50bp) |
| GTCGGGTCTGCGAGAGCGCCAGCTATCCTGAGGGAAACTTCGGAGGGAAC | 135705 | 0.17616655119766317 | No Hit |
| GAAGAAACTAACCAGGATTCCCTCAGTAACGGCGAGTGAACAGGGAAGAG | 132282 | 0.17172295586403802 | No Hit |
| CCGTCGGCATGTATTAGCTCTAGAATTACCACAGTTATCCAAGTAGGAGA | 126711 | 0.16449091683288825 | No Hit |
| CGGGTCTTCCGTACGCCACATGTCCCGCGCCCCGCCGCGGGGCGGGATT | 122469 | 0.15898412997772088 | No Hit |
| GCGGGTCTTCCGTACGCCACATGTCCCGCGCCCCGCCGCGGGGCGGGGAT | 122038 | 0.1584246238168116 | No Hit |
| CAGACGTGGCGACCCGCTGAATTTAAGCATATTAGTCAGCGGAGGAGAAG | 114886 | 0.1491401967568972 | No Hit |
| GCTGGATAGTAGGTAGGGACAGTGGGAATCTCGTTCATCCATTCATGCGC | 114282 | 0.14835610923673662 | No Hit |
| GTCGGCATGTATTAGCTCTAGAATTACCACAGTTATCCAAGTAGGAGAGG | 113285 | 0.14706184556521332 | No Hit |
| GTGGCGACCCGCTGAATTTAAGCATATTAGTCAGCGGAGGAGAAGAAACT | 104542 | 0.13571204889507466 | No Hit |
| CTCGATCAGAAGGACTTGGGCCCCCCACGAGCGGCGCCGGGGAGCGGGTC | 104306 | 0.1354056835726278 | No Hit |
| CCGCGGGGCCCGAAGCGTTTACTTTGAAAAAATTAGAGTGTTCAAAGCAG | 103997 | 0.1350045527055258 | No Hit |
| CGCGGGGCCCGAAGCGTTTACTTTGAAAAAATTAGAGTGTTCAAAGCAGG | 96616 | 0.12542284743018625 | No Hit |
| CCCGAAGTTACGGATCCGGCTTGCCGACTTCCCTTACCTACATTGTTCCA | 96097 | 0.1247491033524324 | No Hit |
| TGGCGACCCGCTGAATTTAAGCATATTAGTCAGCGGAGGAGAAGAAACTA | 92940 | 0.12065081808563292 | No Hit |
| CTCTCTTCAAAGTTCTTTTCAACTTTCCCTTACGGTACTTGTTGACTATC | 89963 | 0.11678620128510646 | No Hit |
| CCCAGGCATAGTTCACCATCTTTCGGGTCCTAACACGTGCGCTCGTGCTC | 88966 | 0.11549193761358316 | No Hit |
| CTGGATAGTAGGTAGGGACAGTGGGAATCTCGTTCATCCATTCATGCGCG | 88732 | 0.11518816860742824 | No Hit |
| GTCGCGTAACTAGTTAGCATGCCAGAGTCTCGTTCGTTATCGGAATTAAC | 86634 | 0.11246463281720166 | No Hit |
| GTCAAAGTGAAGAAATTCAATGAAGCGCGGGTAAACGGCGGGAGTAACTA | 86575 | 0.11238804148658996 | No Hit |
| CCCTCCTTAGGCAACCTGGTGGTCCCCCGCTCCCGGGAGGTCACCATATT | 85866 | 0.11146764736110347 | No Hit |
| CTCCCTTTCGATCGGCCGAGGGCAACGGAGGCCATCGCCCGTCCCTTCGG | 85017 | 0.11036551109518242 | No Hit |
| CACCCGTTTACCTCTTAACGGTTTCACGCCCTCTTGAACTCTCTCTTCAA | 84667 | 0.109911155744096 | No Hit |
| GGCGGGAGTAACTATGACTCTCTTAAGGTAGCCAAATGCCTCGTCATCTA | 84215 | 0.10932438826212156 | No Hit |
| CTGAATTTAAGCATATTAGTCAGCGGAGGAGAAGAAACTAACCAGGATTC | 83096 | 0.10787174929679098 | No Hit |
| CCGTGCCAGACTAGAGTCAAGCTCAACAGGGTCTTCTTTCCCCGCTGATT | 81810 | 0.1062023179210849 | No Hit |
| ATCAGACGTGGCGACCCGCTGAATTTAAGCATATTAGTCAGCGGAGGAGA | 81064 | 0.10523389194419784 | No Hit |
| CGCCCATCTCTCAGGACCGACTGACCCATGTTCAACTGCTGTTCACATGG | 79830 | 0.10363196479208174 | No Hit |
| CTTGAACTCTCTCTTCAAAGTTCTTTTCAACTTTCCCTTACGGTACTTGT | 79333 | 0.10298678019353902 | No Hit |
| CTTCACCGTGCCAGACTAGAGTCAAGCTCAACAGGGTCTTCTTTCCCCGC | 78305 | 0.10165227361949092 | No Hit |
| CCGGGCTTCTTACCCATTTAAAGTTTGAGAATAGGTTGAGATCGTTTCGG | 77866 | 0.10108238219341395 | No Hit |

## Adapter Content

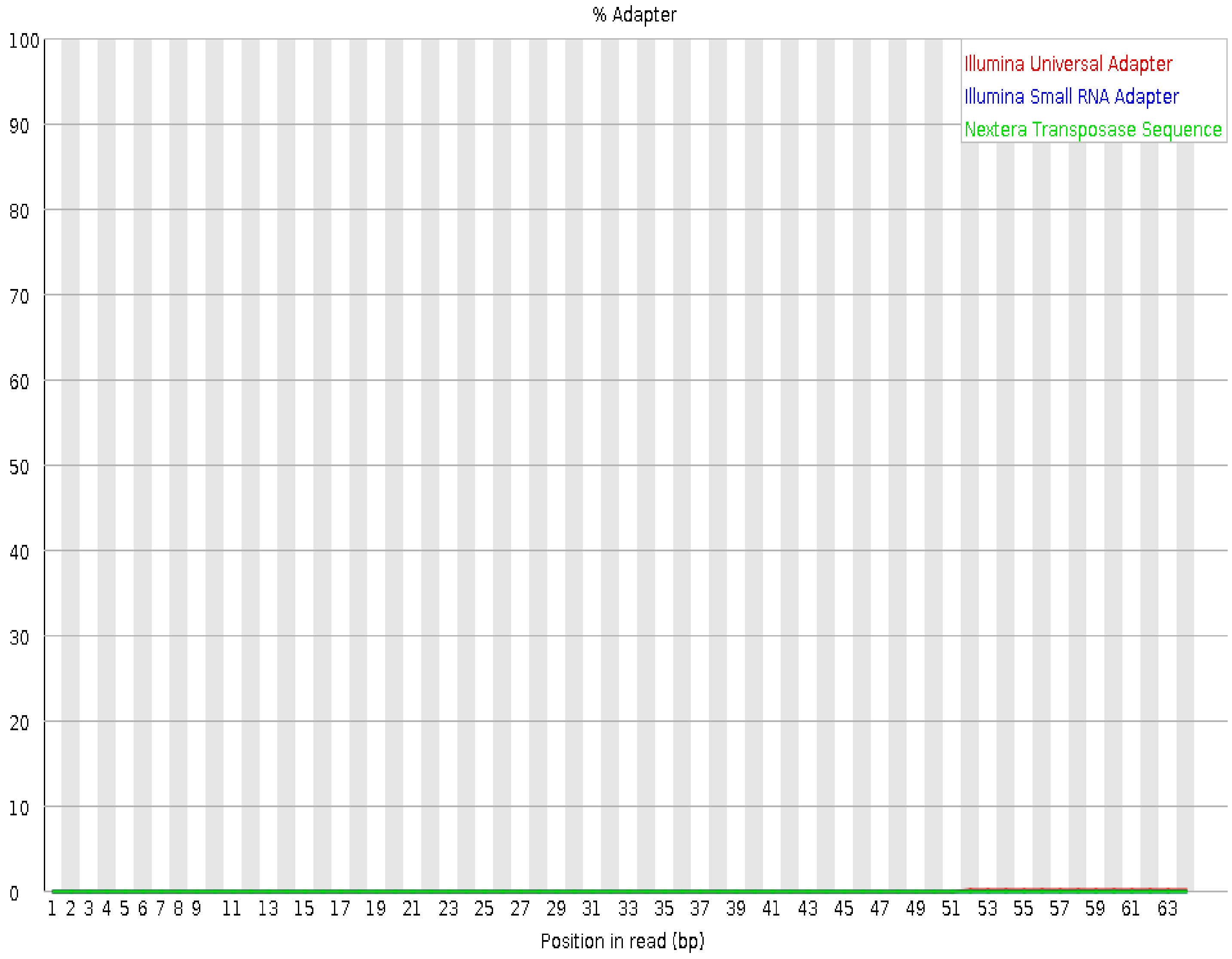


## Kmer Content

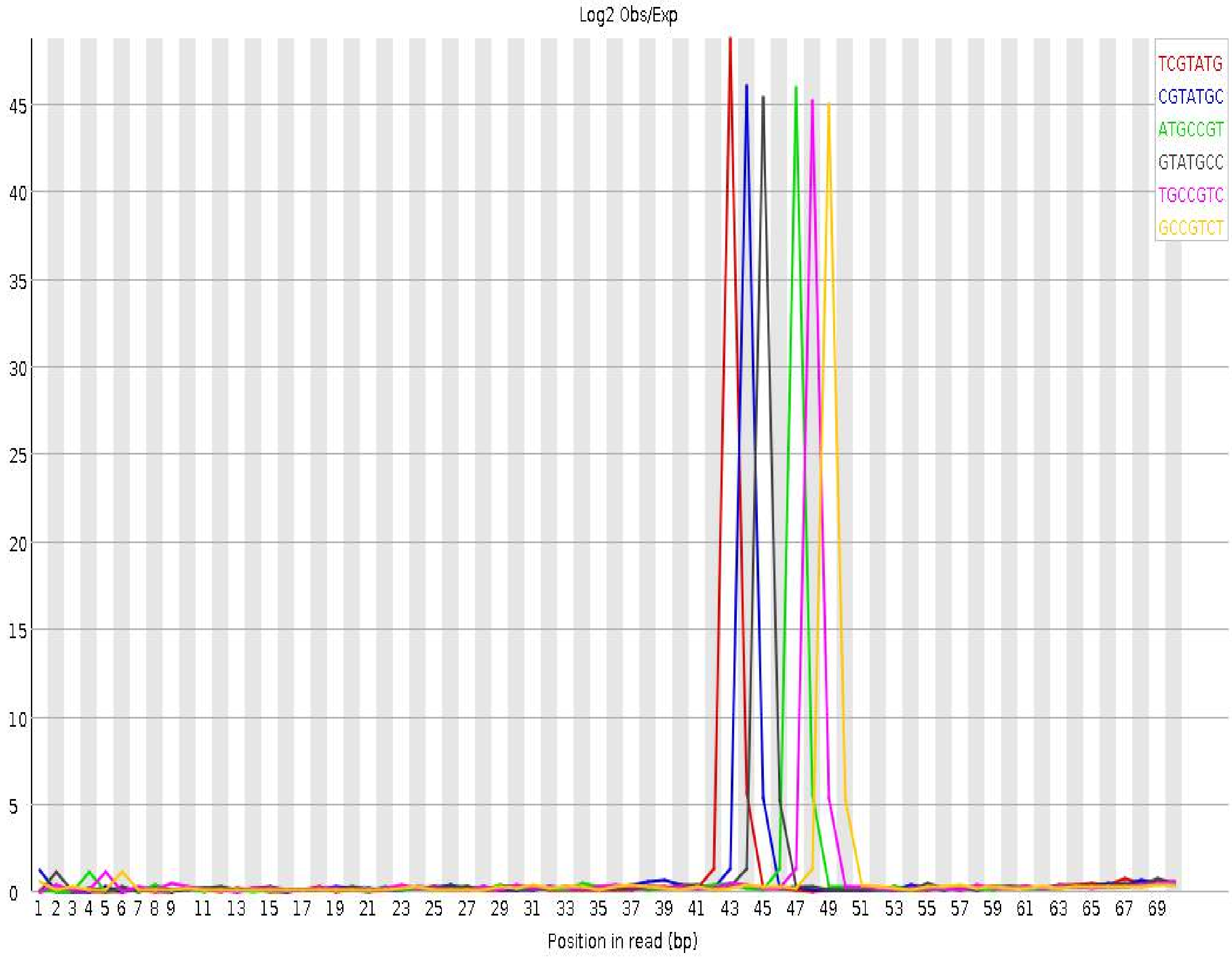


| Sequence | Count | PValue | Obs/Exp Max | Max Obs/Exp Position |
|---|---|---|---|---|
| TCGTATG | 27815 | 0.0 | 48.67264 | 43 |
| CGTATGC | 29445 | 0.0 | 45.967495 | 44 |
| ATGCCGT | 29065 | 0.0 | 45.878452 | 47 |
| GTATGCC | 29785 | 0.0 | 45.39391 | 45 |
| TGCCGTC | 29825 | 0.0 | 45.166916 | 48 |
| GCCGTCT | 30065 | 0.0 | 44.935463 | 49 |
| CGCGTAA | 67210 | 0.0 | 43.261307 | 1 |
| GATCTCG | 29935 | 0.0 | 42.558315 | 39 |
| CTCGTAT | 31200 | 0.0 | 42.370815 | 42 |
| TGCTTGA | 32580 | 0.0 | 41.92169 | 58 |
| CTGCTTG | 34470 | 0.0 | 40.80237 | 57 |
| GTCACAT | 34595 | 0.0 | 39.68433 | 29 |
| ACATCAC | 35570 | 0.0 | 38.775536 | 32 |
| GCTTGAA | 35110 | 0.0 | 38.373737 | 59 |
| TCTCGTA | 33145 | 0.0 | 38.167145 | 41 |

| Sequence | Count | PValue | Obs/Exp Max | Max Obs/Exp Position |
|---|---|---|---|---|
| GCGTAAC | 77295 | 0.0 | 38.[illegible] | 2 |
| AGTCACA | 36220 | 0.0 | 37.6[illegible] | 28 |
| CAGTCAC | 37685 | 0.0 | 35.932896 | 27 |
| TCTTCTG | 38460 | 0.0 | 35.768715 | 53 |
| CTGCCAG | 36270 | 0.0 | 35.742947 | 1 |

**Produced by FastQC (version 0.11.2)**

# FastQC Report

Mon 4 May 2015
Agilent_Adult_Lung_ATCACG_L002_R2.fastq.gz

## Summary


- Basic Statistics
- Per base sequence quality
- Per tile sequence quality
- Per sequence quality scores
- Per base sequence content
- Per sequence GC content
- Per base N content
- Sequence Length Distribution
- Sequence Duplication Levels
- Overrepresented sequences
- Adapter Content
- Kmer Content


## Basic Statistics

| Measure | Value |
|---|---|
| Filename | Agilent_Adult_Lung_ATCACG_L002_R2.fastq.gz |
| File type | Conventional base calls |
| Encoding | Sanger / Illumina 1.9 |
| Total Sequences | 77032217 |
| Sequences flagged as poor quality | 0 |
| Sequence length | 76 |
| %GC | 57 |

## Per base sequence quality

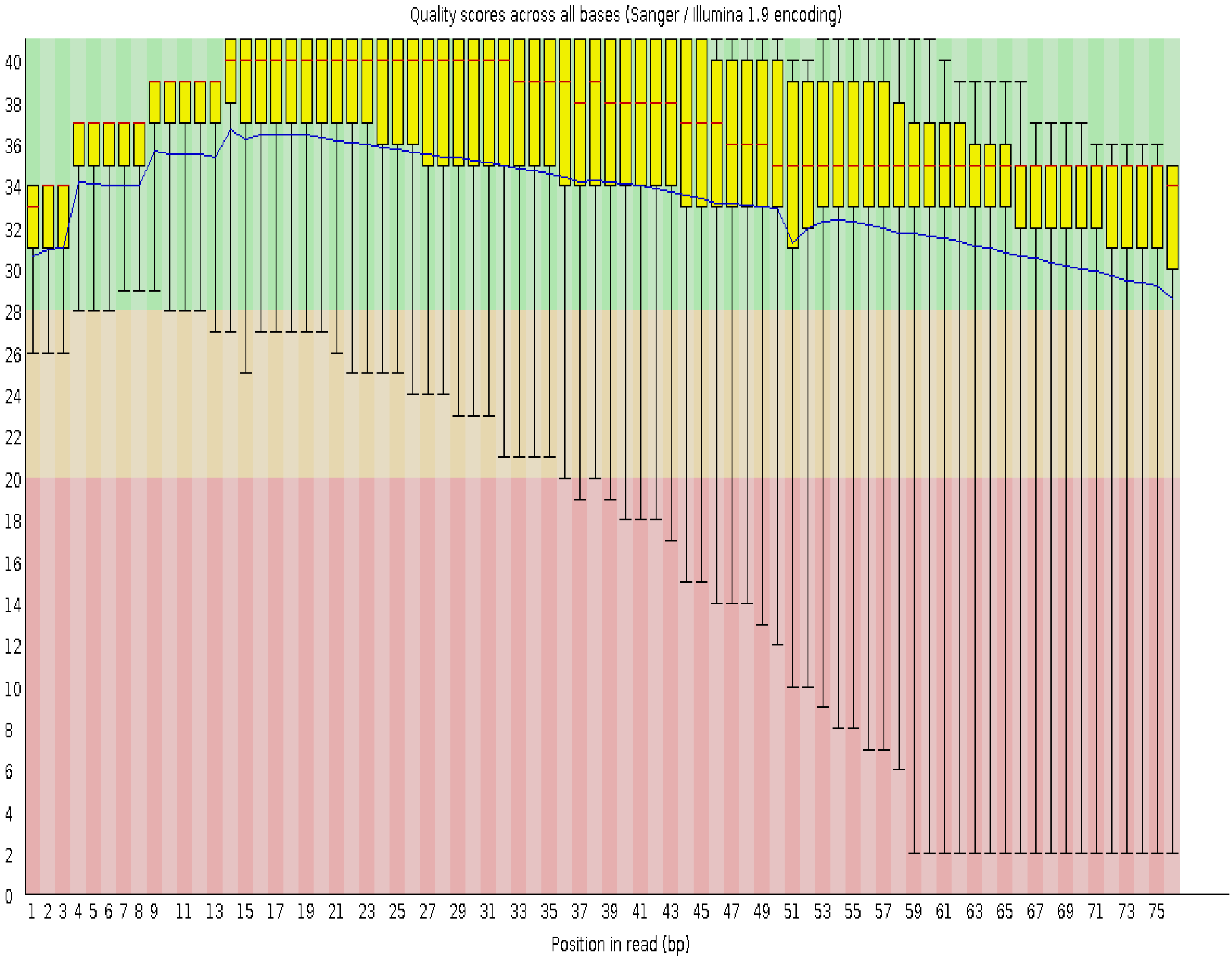


## Per tile sequence quality

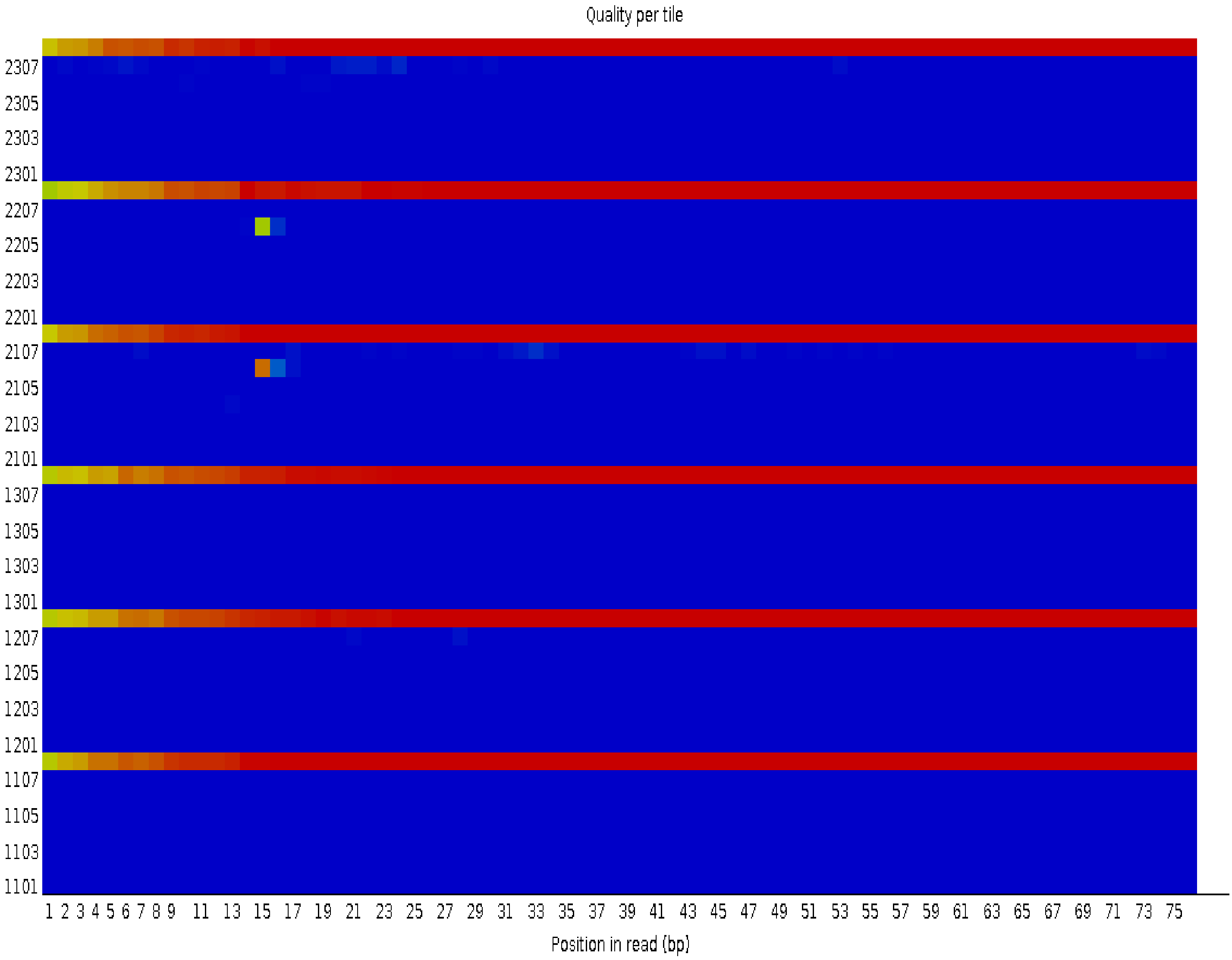


## Per sequence quality scores

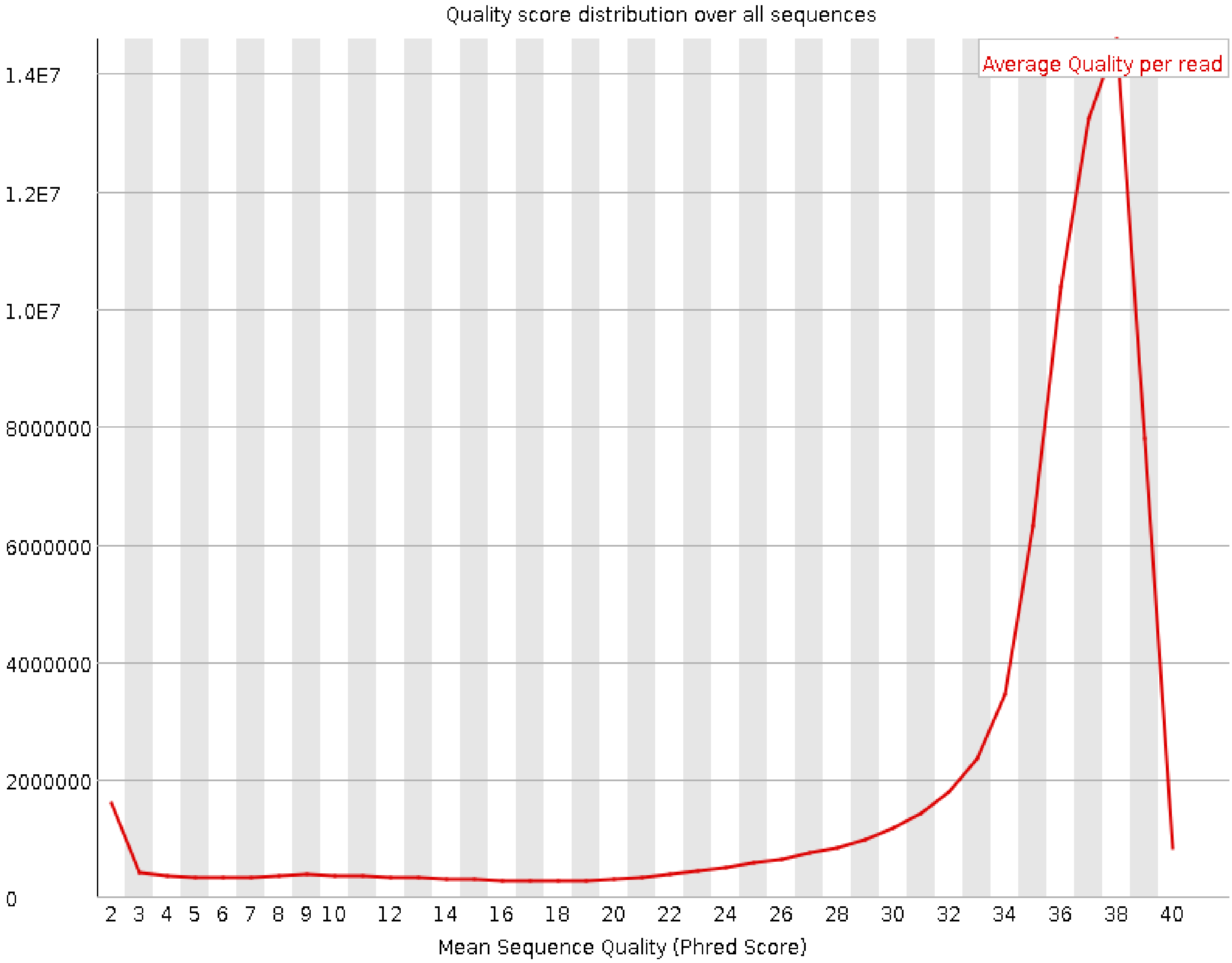


## Per base sequence content

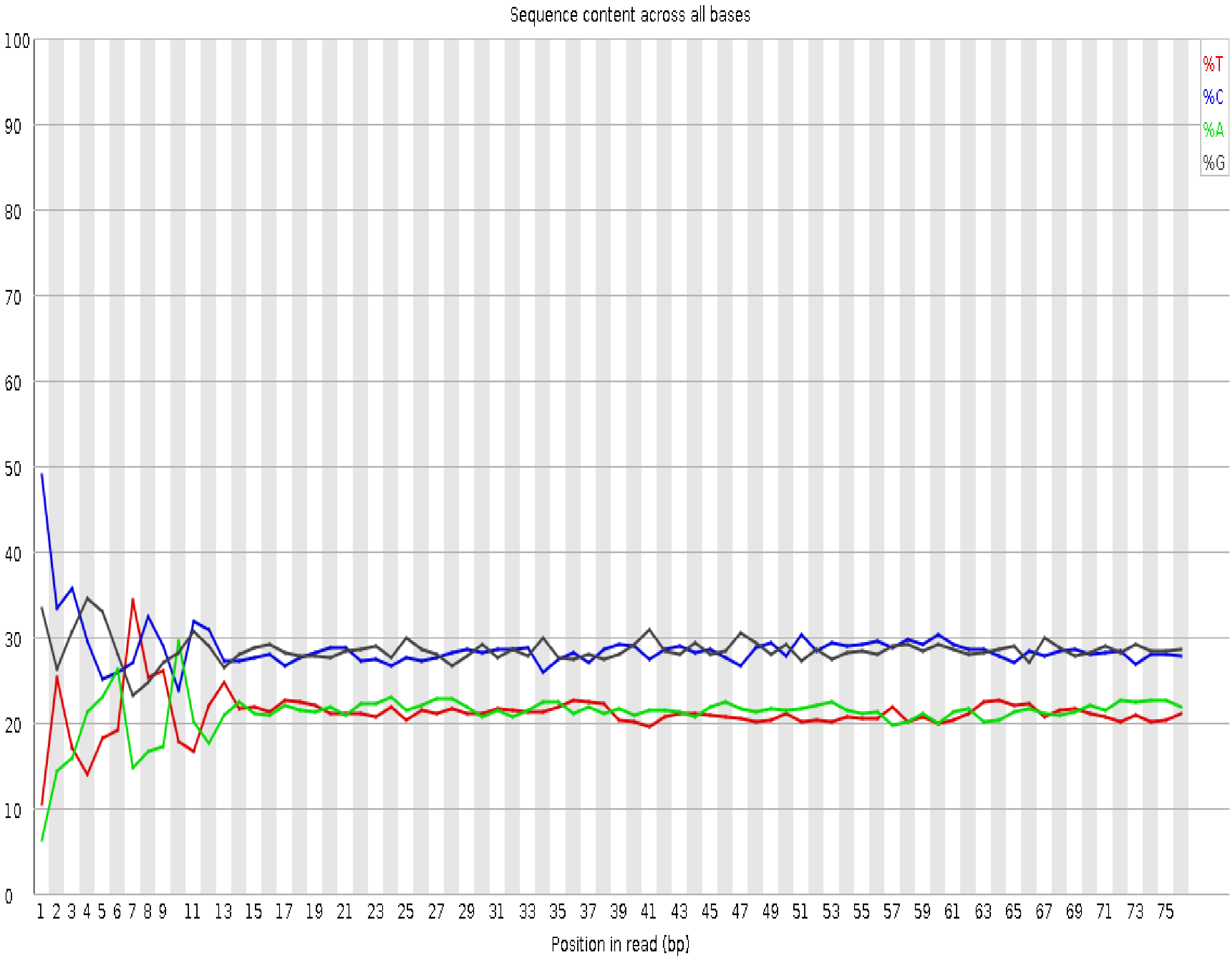


# Per sequence GC content

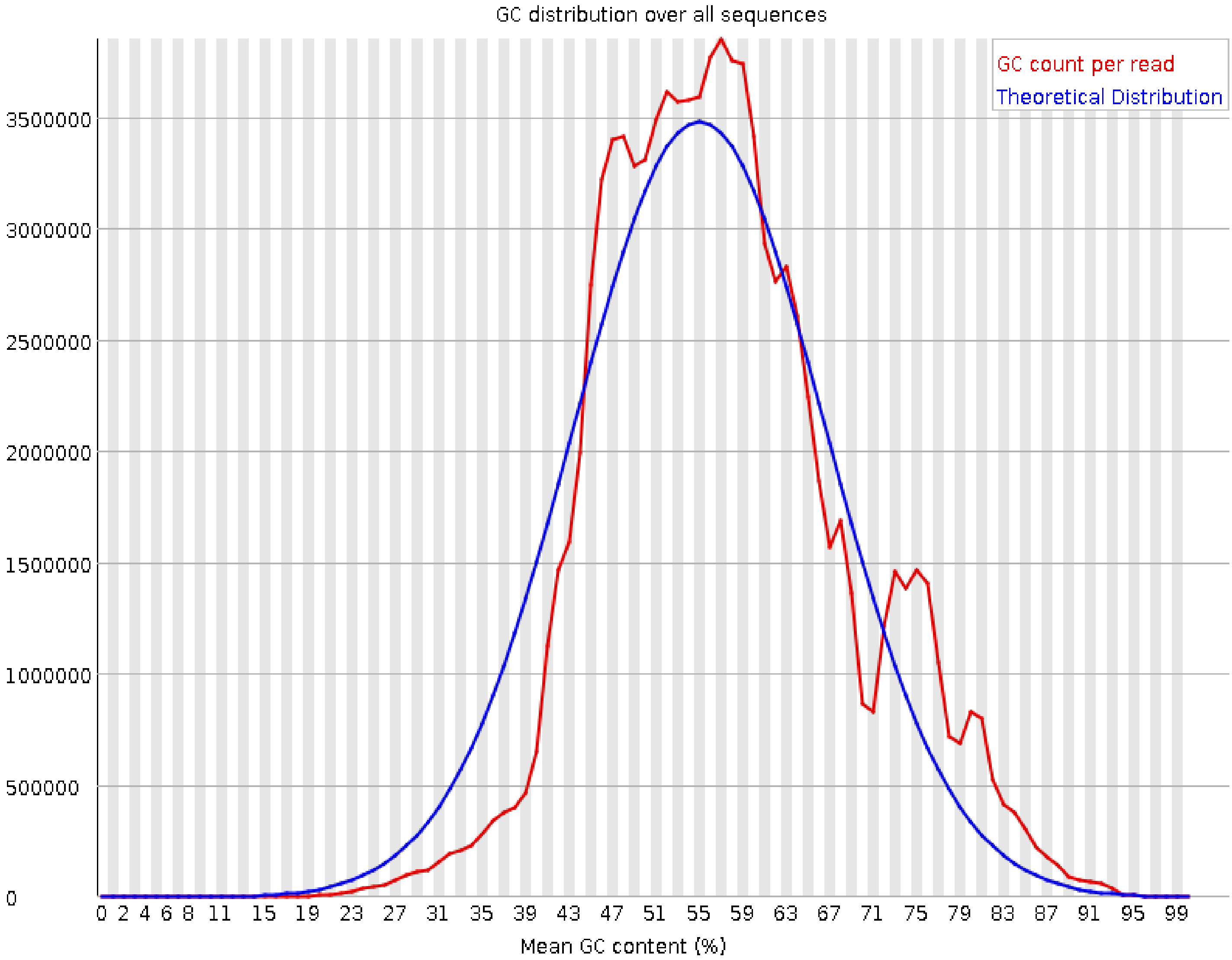


## Per base N content

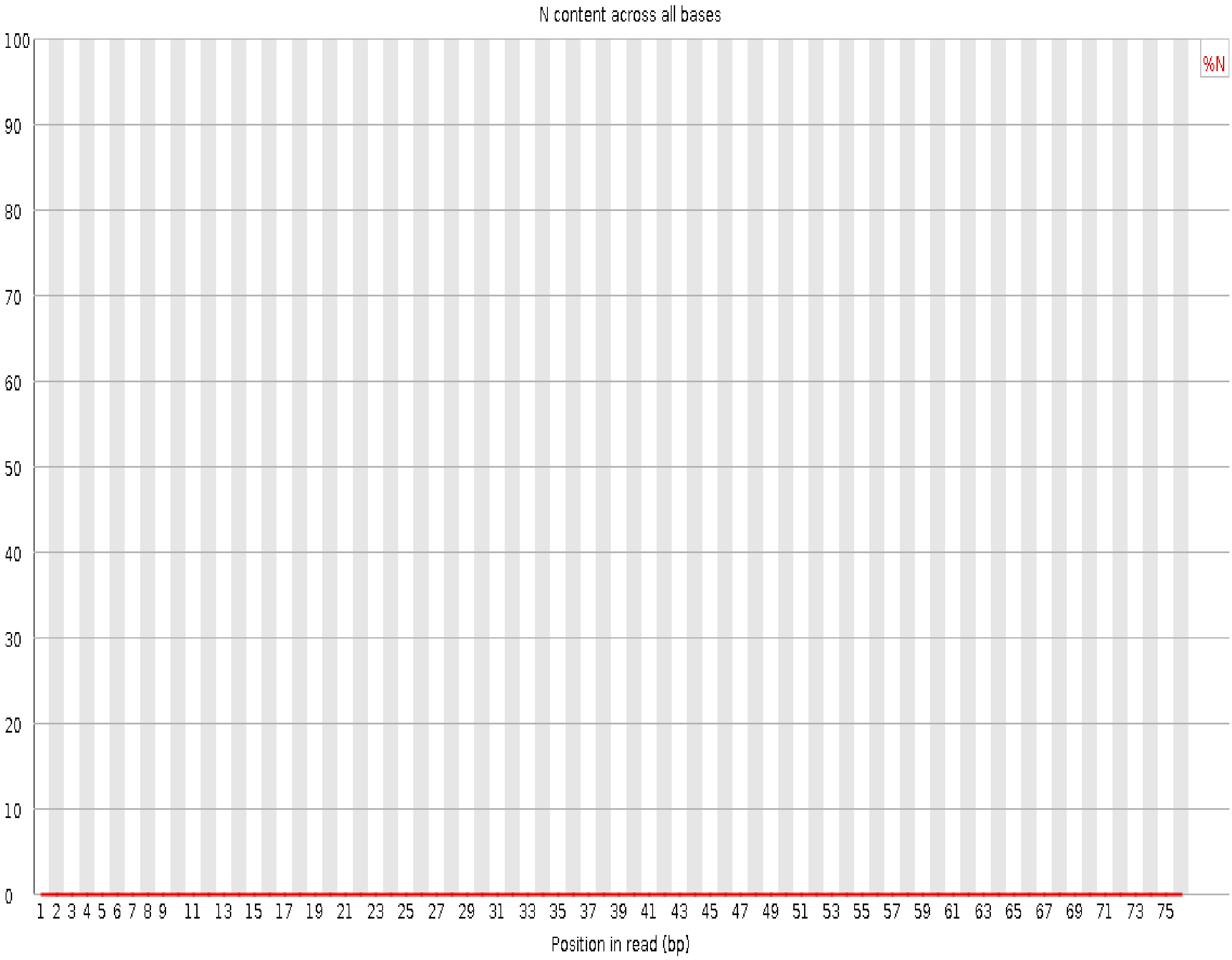


## Sequence Length Distribution

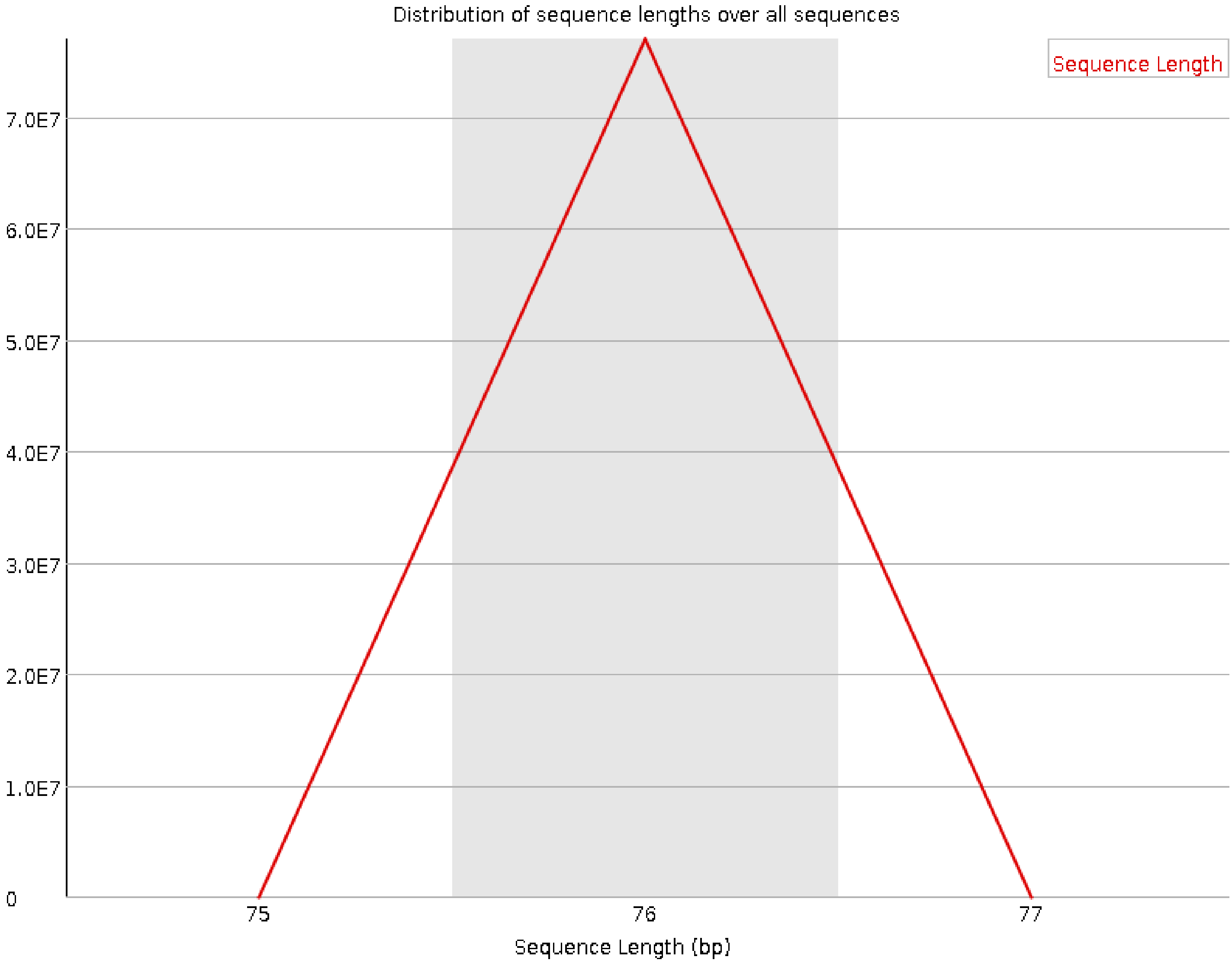


## Sequence Duplication Levels

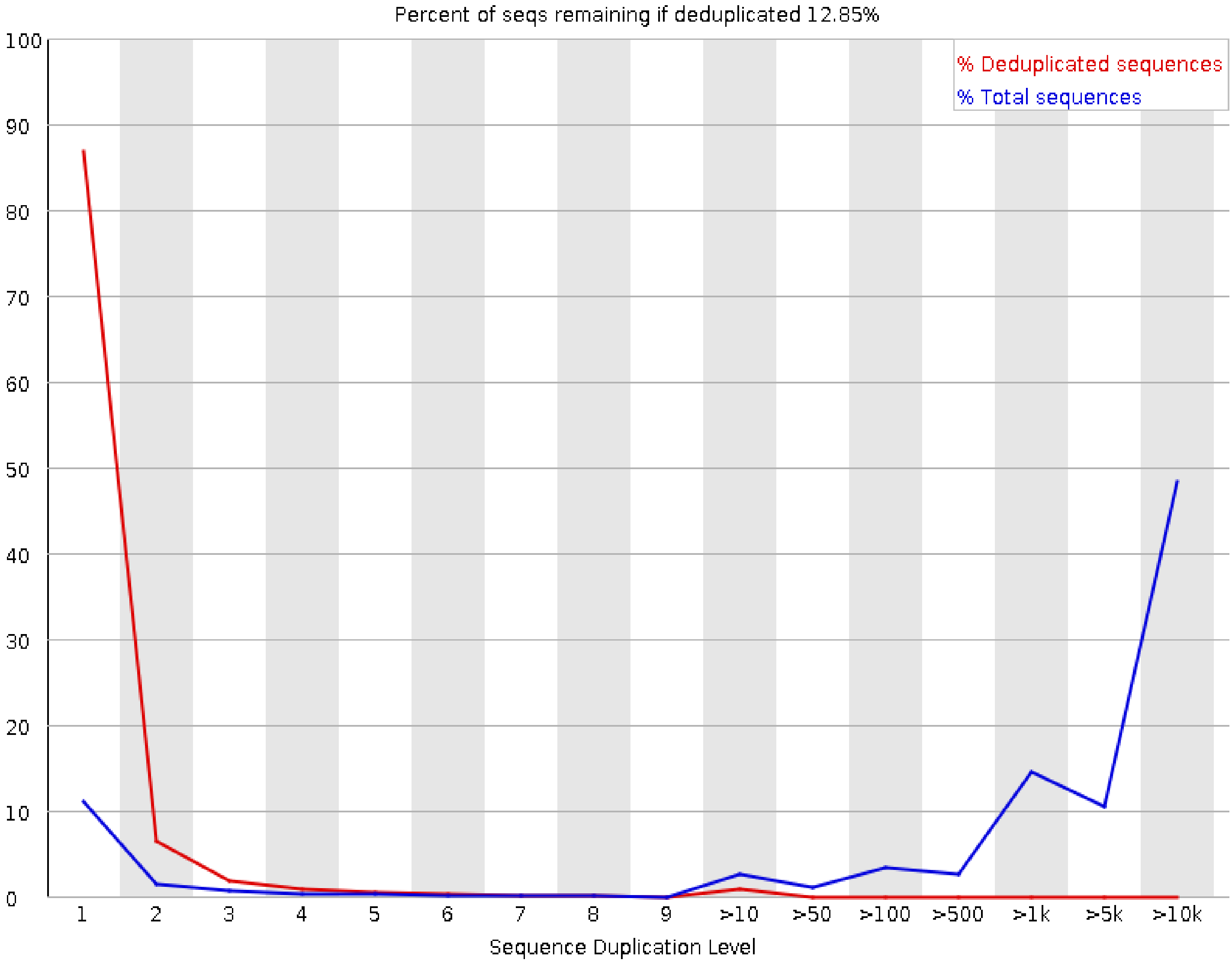


## Overrepresented sequences

| Sequence | Count | Percentage | Possible Source |
|---|---|---|---|
| CGCGTAACTAGTTAGCATGCCAGAGTCTCGTTCGTTATCGGAATTAACCA | 366396 | 0.4756399520475959 | No Hit |
| CCTCACCCGGCCCGGACACGGACAGGATTGACAGATTGATAGCTCTTTCT | 323203 | 0.4195686072490943 | No Hit |
| CTTCCGTACGCCACATGTCCCGCGCCCCGCCGCGGGGCGGGGATTCGGCG | 236844 | 0.3074609679220319 | No Hit |
| CCCGTCGGCATGTATTAGCTCTAGAATTACCACAGTTATCCAAGTAGGAG | 210607 | 0.2734011926464482 | No Hit |
| CCGGTATTTAGCCTTAGATGGAGTTTACCACCCGCTTTGGGCTGCATTCC | 208944 | 0.2712423556497147 | No Hit |
| CTCCGACTTTCGTTCTTGATTAATGAAAACATTCTTGGCAAATGCTTTCG | 185218 | 0.24044225547863954 | No Hit |
| GCCCTCTTGAACTCTCTCTTCAAAGTTCTTTTCAACTTTCCCTTACGGTA | 177716 | 0.2307034730676387 | No Hit |
| CGCAGTTTTATCCGGTAAAGCGAATGATTAGAGGTCTTGGGGCCGAAACG | 173950 | 0.2258146094899489 | No Hit |
| CTGCCAGTAGCATATGCTTGTCTCAAAGATTAAGCCATGCATGTCTAAGT | 164796 | 0.21393126982182015 | No Hit |
| CTCACCCGGCCCGGACACGGACAGGATTGACAGATTGATAGCTCTTTCTC | 162494 | 0.2109429097698175 | No Hit |
| CGGGTCTTCCGTACGCCACATGTCCCGCGCCCCGCCGCGGGGCGGGGATT | 149533 | 0.1941174820400145 | No Hit |

| Sequence | Count | Percentage | Possible Source |
|---|---|---|---|
| GCTGAATTTAAGCATATTAGTCAGCGGAGGAGAAGAAACTAACCAGGATT | 141350 | 0.18349465393161407 | No Hit |
| GTCGGGTCTGCGAGAGCGCCAGCTATCCTGAGGGAAACTTCGGAGGGAAC | 140961 | 0.18298967041283518 | [illegible] |
| CCGTCGGCATGTATTAGCTCTAGAATTACCACAGTTATCCAAGTAGGAGA | 140588 | 0.18250545742439167 | No Hit |
| GCGGGTCTTCCGTACGCCACATGTCCCGCGCCCCGCCGCGGGGCGGGGAT | 137987 | 0.17912894808674662 | No Hit |
| GAAGAAACTAACCAGGATTCCCTCAGTAACGGCGAGTGAACAGGGAAGAG | 126247 | 0.16388857145316224 | No Hit |
| GTCGGCATGTATTAGCTCTAGAATTACCACAGTTATCCAAGTAGGAGAGG | 123404 | 0.1601979078441946 | No Hit |
| CCGCGGGGCCCGAAGCGTTTACTTTGAAAAAATTAGAGTGTTCAAAGCAG | 122515 | 0.159043845252435l | No Hit |
| GCTGGATAGTAGGTAGGGACAGTGGGAATCTCGTTCATCCATTCATGCGC | 120863 | 0.15689928799530722 | No Hit |
| CAGACGTGGCGACCCGCTGAATTTAAGCATATTAGTCAGCGGAGGAGAAG | 118115 | 0.15333194941020586 | No Hit |
| CTCGATCAGAAGGACTTGGGCCCCCCACGAGCGGCGCCGGGGAGCGGGTC | 114134 | 0.14816398183113436 | No Hit |
| CGCGGGGCCCGAAGCGTTTACTTTGAAAAAATTAGAGTGTTCAAAGCAGG | 113612 | 0.14748634327894264 | No Hit |
| GTGGCGACCCGCTGAATTTAAGCATATTAGTCAGCGGAGGAGAAGAAACT | 103228 | 0.13400626909128166 | No Hit |
| CTCCCTTTCGATCGGCCGAGGGCAACGGAGGCCATCGCCCGTCCCTTCGG | 98218 | 0.12750249678001607 | No Hit |
| CCCGAAGTTACGGATCCGGCTTGCCGACTTCCCTTACCTACATTGTTCCA | 97957 | 0.1271636775039202 | No Hit |
| CACCCGTTTACCTCTTAACGGTTTCACGCCCTCTTGAACTCTCTCTTCAA | 94315 | 0.12243578553632956 | No Hit |
| CTGGATAGTAGGTAGGGACAGTGGGAATCTCGTTCATCCATTCATGCGCG | 93608 | 0.121517987727135 | No Hit |
| CCCAGGCATAGTTCACCATCTTTCGGGTCCTAACACGTGCGCTCGTGCTC | 93100 | 0.12085852338898671 | No Hit |
| CGCCCATCTCTCAGGACCGACTGACCCATGTTCAACTGCTGTTCACATGG | 92620 | 0.12023540747892535 | No Hit |
| CTCTCTTCAAAGTTCTTTTCAACTTTCCCTTACGGTACTTGTTGACTATC | 92614 | 0.12022761853004958 | No Hit |
| CCGTGCCAGACTAGAGTCAAGCTCAACAGGGTCTTCTTTCCCCGCTGATT | 92481 | 0.12005496349663675 | No Hit |
| TGGCGACCCGCTGAATTTAAGCATATTAGTCAGCGGAGGAGAAGAAACTA | 92463 | 0.12003159665000944 | No Hit |
| CCCTCCTTAGGCAACCTGGTGGTCCCCCGCTCCCGGGAGGTCACCATATT | 92333 | 0.11986283609103447 | No Hit |
| GGCGGGAGTAACTATGACTCTCTTAAGGTAGCCAAATGCCTCGTCATCTA | 89128 | 0.11570223923322888 | No Hit |
| GTCGCGTAACTAGTTAGCATGCCAGAGTCTCGTTCGTTATCGGAATTAAC | 88367 | 0.1147143408841524 | No Hit |
| CTTCACCGTGCCAGACTAGAGTCAAGCTCAACAGGGTCTTCTTTCCCCGC | 88014 | 0.1142560910586281 | No Hit |
| CGGCCTTCAAAGTTCTCGTTTGAATATTTGCTACTACCACCAAGATCTGC | 87349 | 0.11339281589156391 | No Hit |
| GATCGGAAGAGCGTCGTGTAGGGAAAGAGTGTAGATCTCGGTGGTCGCCG | 86231 | 0.1119414750843793 | Illumina Single End PCR Primer 1 (100% over 50bp) |
| CCGACTTTCGTTCTTGATTAATGAAAACATTCTTGGCAAATGCTTTCGCT | 86051 | 0.11170780661810628 | No Hit |
| CCGGGCTTCTTACCCATTTAAAGTTTGAGAATAGGTTGAGATCGTTTCGG | 84434 | 0.10960868489608706 | No Hit |
| GTCAAAGTGAAGAAATTCAATGAAGCGCGGGTAAACGGCGGGAGTAACTA | 83904 | 0.10892066107872761 | No Hit |
| CCACTCTCGACTGCCGGCGACGGCCGGGTATGGGCCCGACGCTCCAGCGC | 82195 | 0.10670210880727996 | No Hit |
| CTTGAACTCTCTCTTCAAAGTTCTTTTCAACTTTCCCTTACGGTACTTGT | 82050 | 0.10651387587611556 | No Hit |
| CGCGATGTGATTTCTGCCCAGTGCTCTGAATGTCAAAGTGAAGAAATTCA | 80069 | 0.10394222458896647 | No Hit |
| CTCCACTTCGGCCTTCAAAGTTCTCGTTTGAATATTTGCTACTACCACCA | 79056 | 0.10262719038710777 | No Hit |
| CTTCCGTCAATTCCTTTAAGTTTCAGCTTTGCAACCATACTCCCCCCGGA | 78846 | 0.10235457717645592 | No Hit |

| Sequence | Count | Percentage | Possible Source |
| --- | --- | --- | --- |
| CTGCTGTCTATATCAACCAACACCTTTTCTGGGGTCTGATGAGCGTCGGC | 77354 | 0.10041772522268182 | No Hit |
| GGAAACTCTGGTGGAGGTCC[illegible]TGACGTGCAAATCGGTCGTC | [illegible] | 0.10[illegible]7708 | No Hit |

# Adapter Content

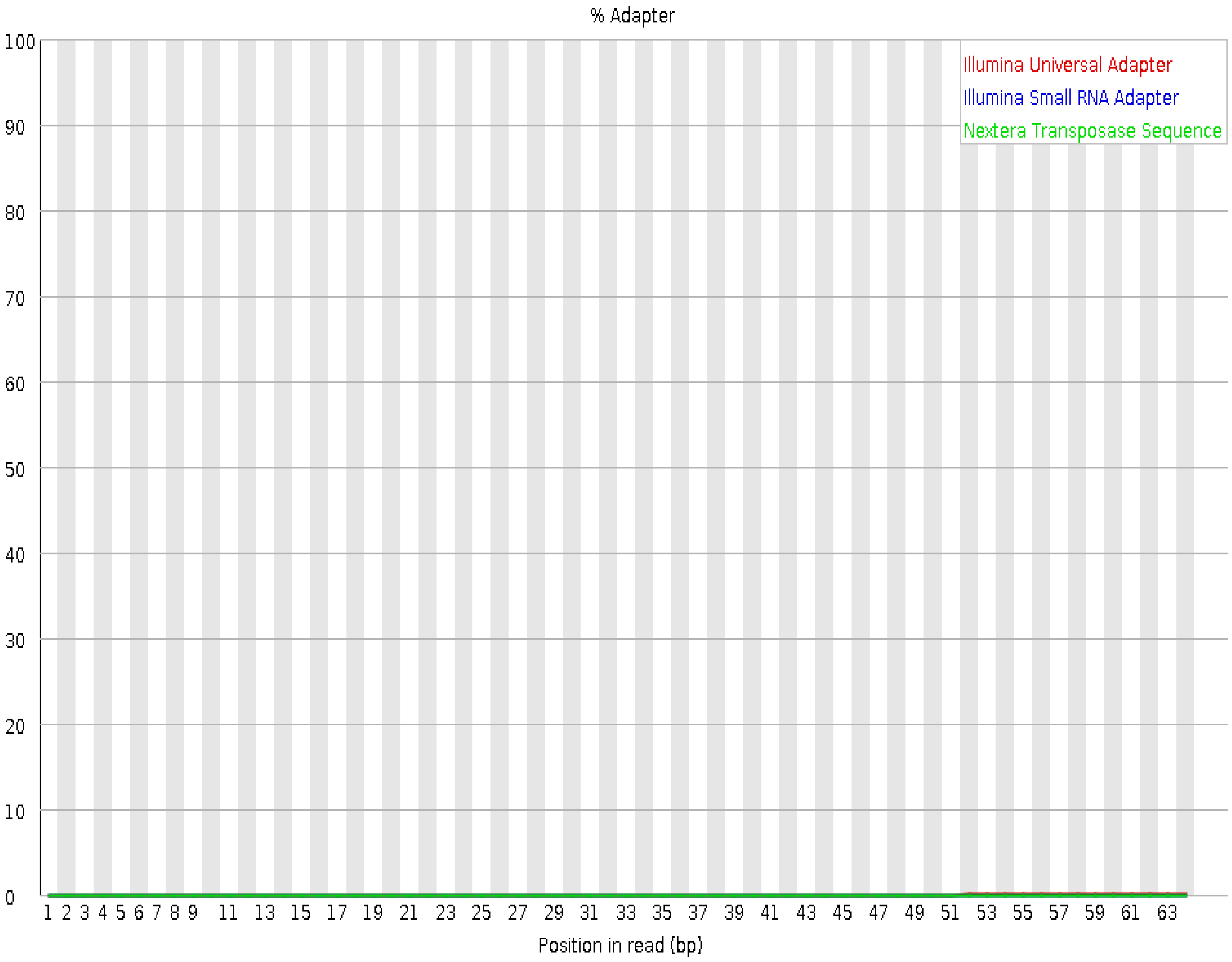


# Kmer Content

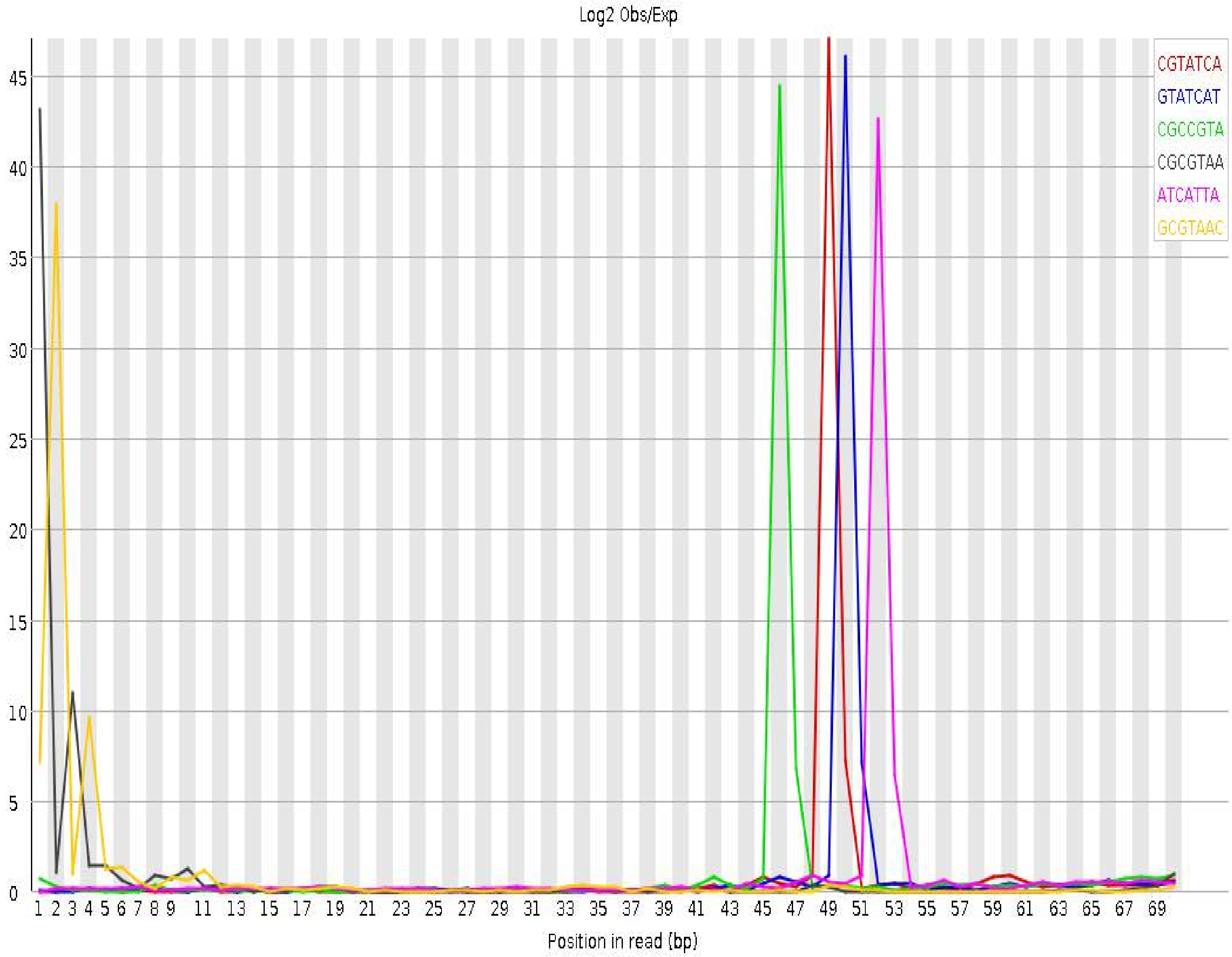


| Sequence | Count | PValue | Obs/Exp Max | Max Obs/Exp Position |
|---|---|---|---|---|
| CGTATCA | 21165 | 0.0 | 47.020824 | 49 |
| GTATCAT | 21435 | 0.0 | 46.06936 | 50 |
| CGCCGTA | 22585 | 0.0 | 44.419113 | 46 |
| CGCGTAA | 66515 | 0.0 | 43.201027 | 1 |
| ATCATTA | 24845 | 0.0 | 42.619938 | 52 |
| GCGTAAC | 76490 | 0.0 | 37.950584 | 2 |
| CTGCCAG | 34565 | 0.0 | 36.582474 | 1 |
| AGTTAGC | 81290 | 0.0 | 35.569183 | 10 |
| GTGGTCG | 24530 | 0.0 | 35.566456 | 41 |
| TAGTTAG | 81930 | 0.0 | 35.468876 | 9 |
| GTTAGCA | 81730 | 0.0 | 35.341206 | 11 |
| CTCGGTG | 27255 | 0.0 | 34.897907 | 37 |
| GTAGATC | 28685 | 0.0 | 34.85495 | 31 |
| TCGGTGG | 28335 | 0.0 | 34.75378 | 38 |
| TAGCATG | 83445 | 0.0 | 34.716084 | 13 |

| Sequence | Count | PValue | Obs/Exp Max | Max Obs/Exp Position |
|---|---|---|---|---|
| GATCTCG | 30885 | 0.0 | [illegible] | 34 |
| TTAGCAT | 84590 | 0.0 | [illegible] | 12 |
| TAGATCT | 29515 | 0.0 | 33.95443 | 32 |
| TCTCGGT | 28020 | 0.0 | 33.6436 | 36 |
| TGCCAGT | 37200 | 0.0 | 33.54775 | 2 |

**Produced by FastQC (version 0.11.2)**

# FastQC Report

Tue 28 Apr 2015
Agilent_Adult_Stomach_GCCAAT_R1.fastq.gz

## Summary


- Basic Statistics
- Per base sequence quality
- Per tile sequence quality
- Per sequence quality scores
- Per base sequence content
- Per sequence GC content
- Per base N content
- Sequence Length Distribution
- Sequence Duplication Levels
- Overrepresented sequences
- Adapter Content
- Kmer Content


## Basic Statistics

| Measure | Value |
|---|---|
| Filename | Agilent_Adult_Stomach_GCCAAT_R1.fastq.gz |
| File type | Conventional base calls |
| Encoding | Sanger / Illumina 1.9 |
| Total Sequences | 149287775 |
| Sequences flagged as poor quality | 0 |
| Sequence length | 74 |
| %GC | 56 |

## Per base sequence quality

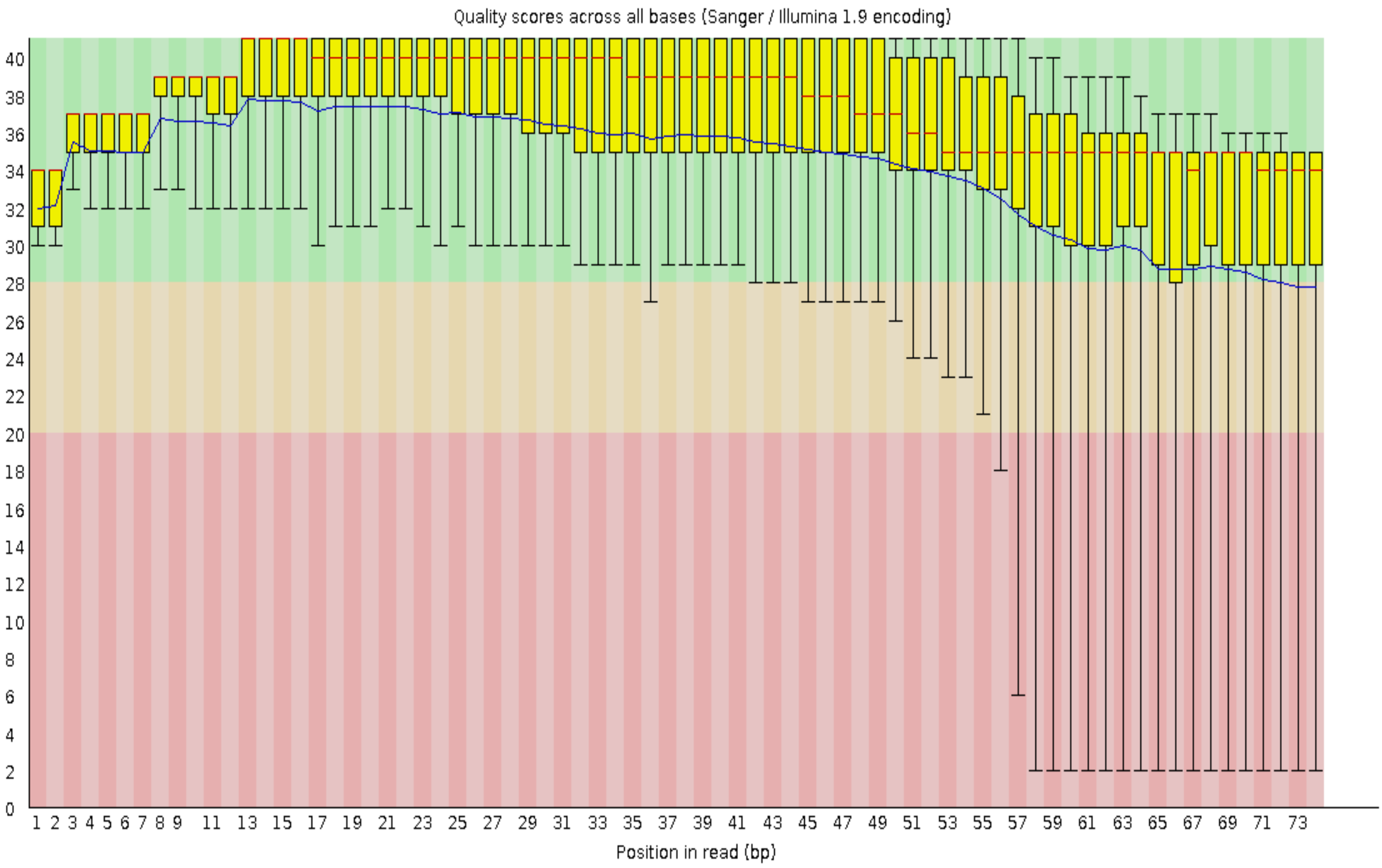


## Per tile sequence quality

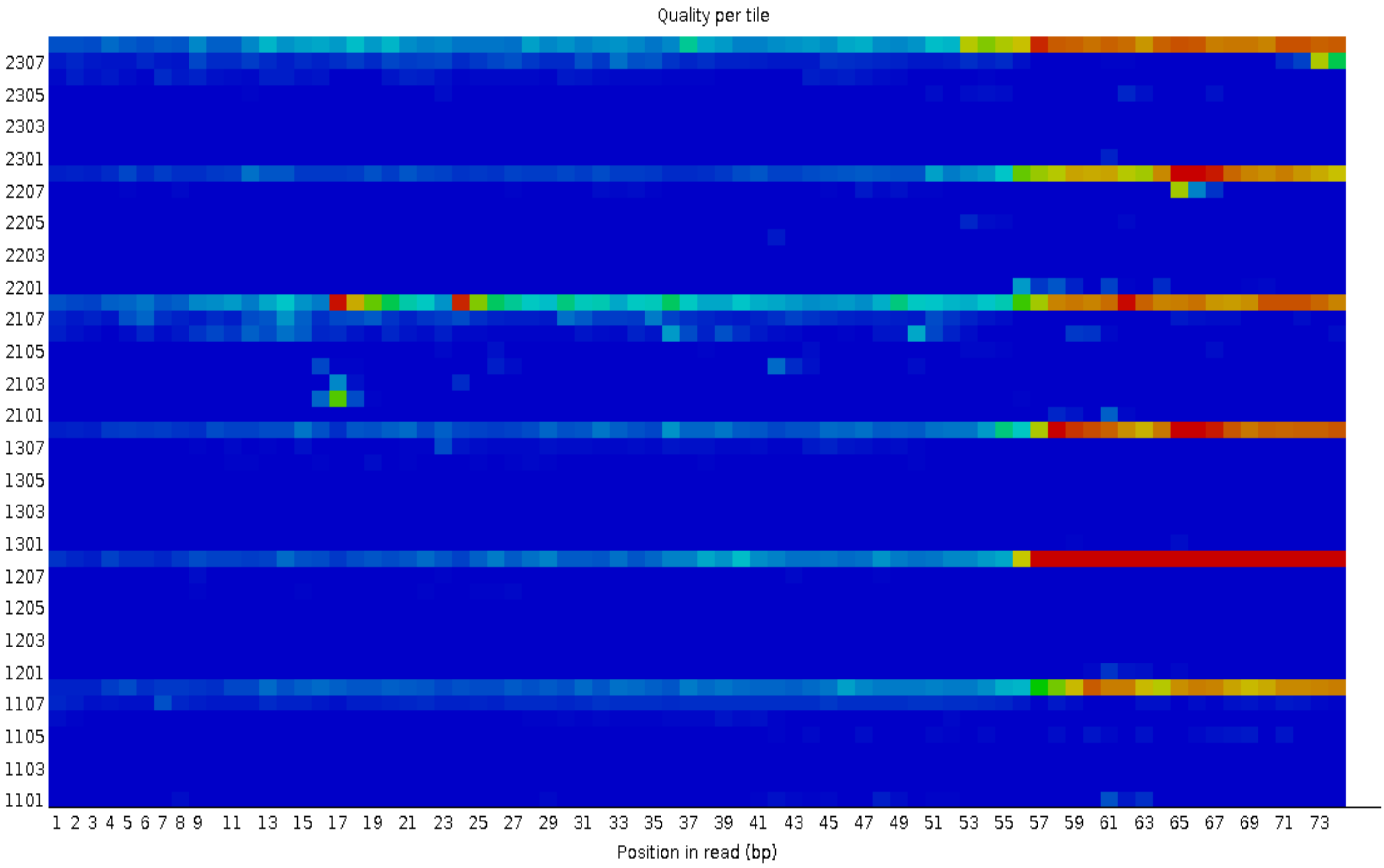

## Per sequence quality scores

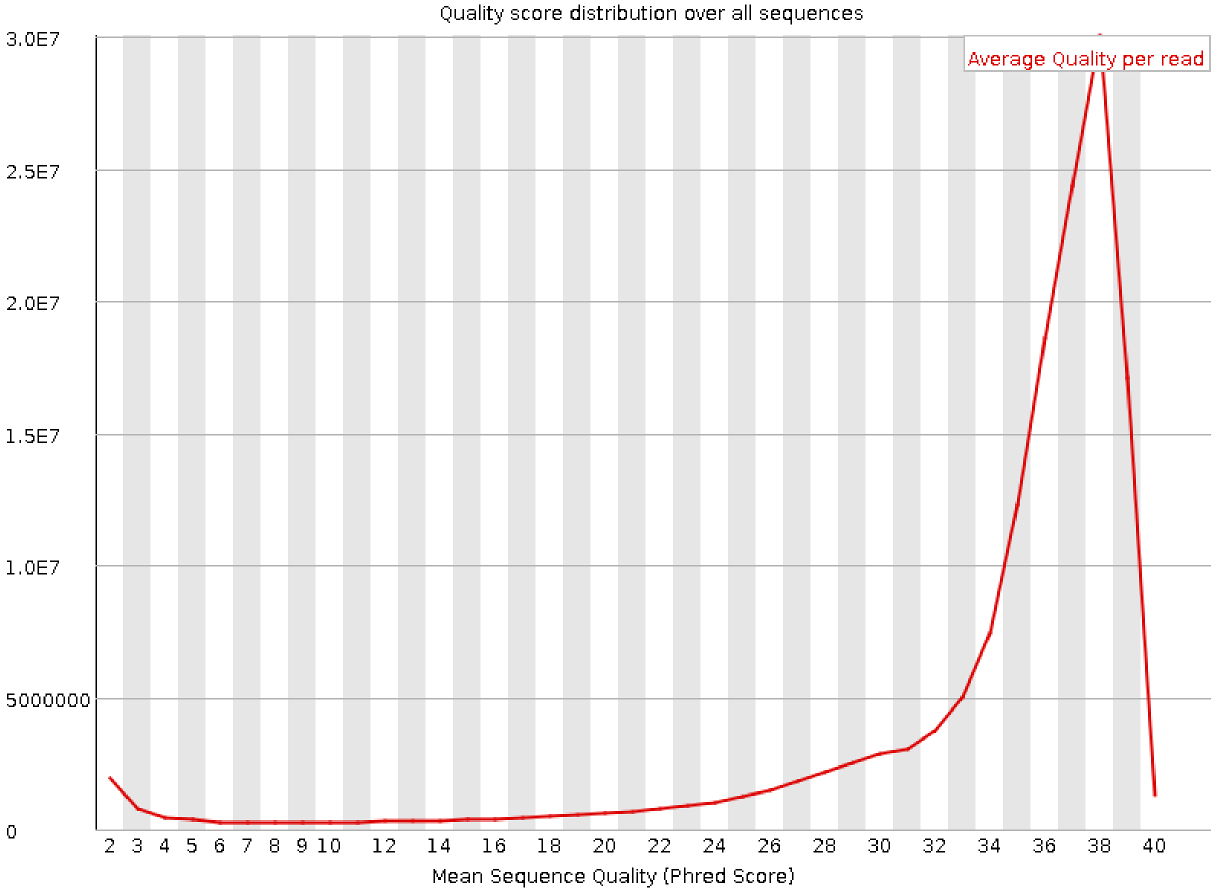


## Per base sequence content

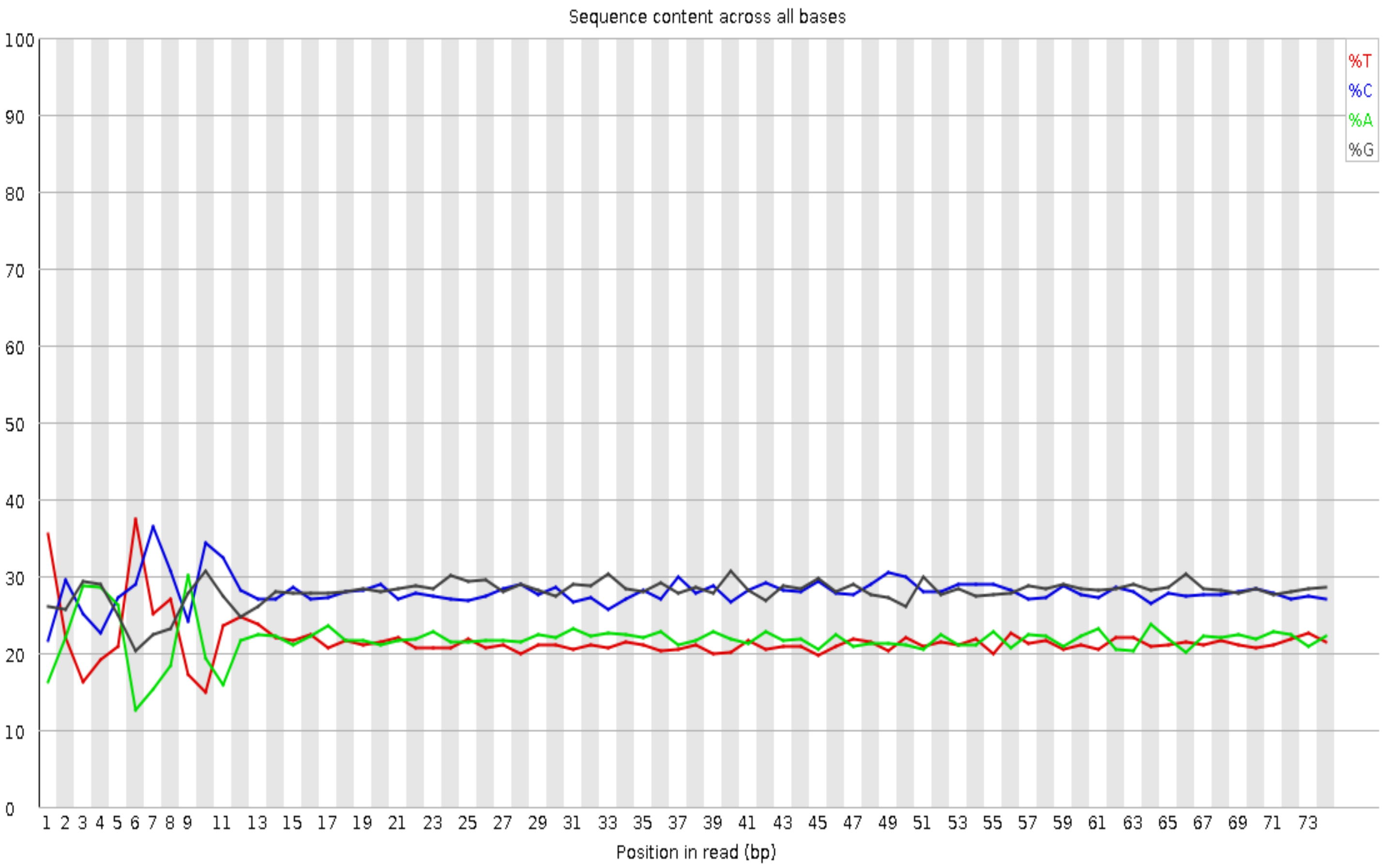


## Per sequence GC content

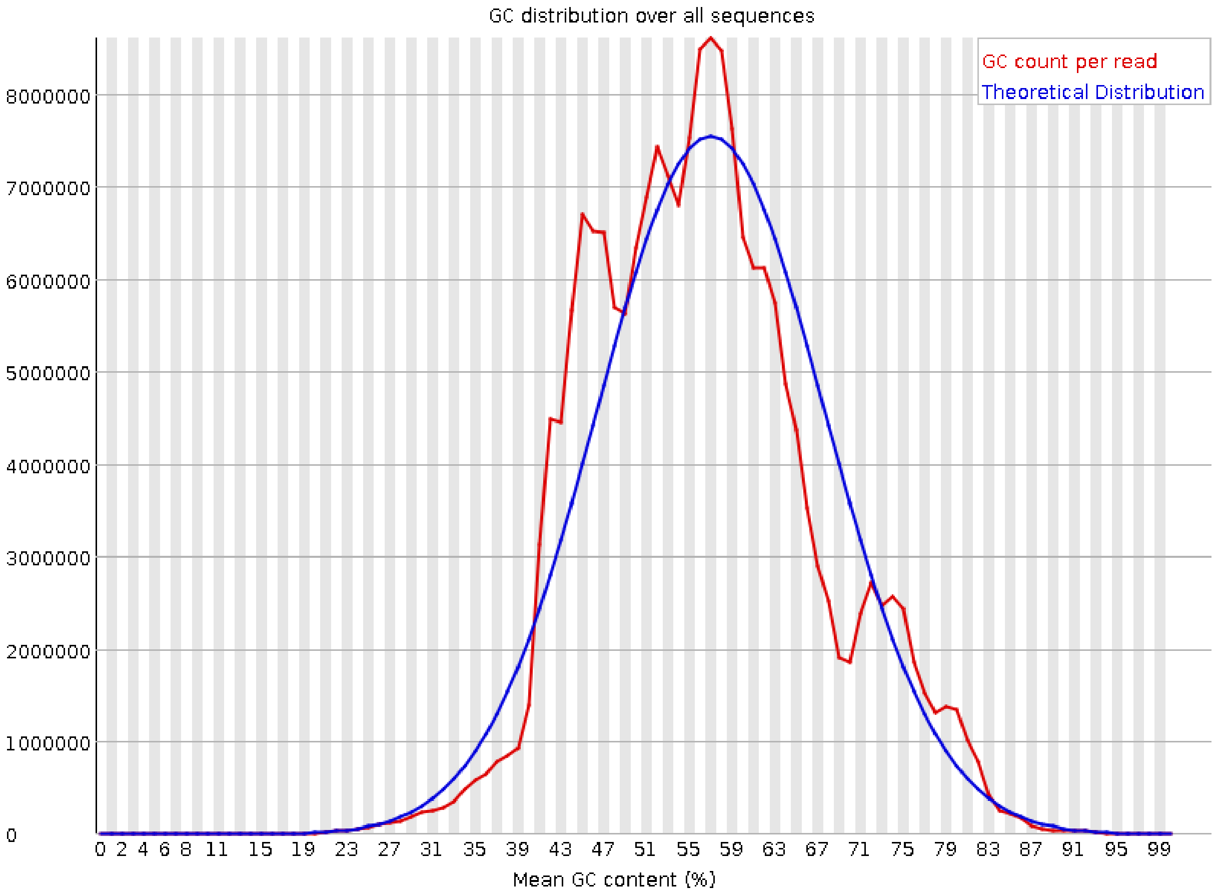

## Per base N content

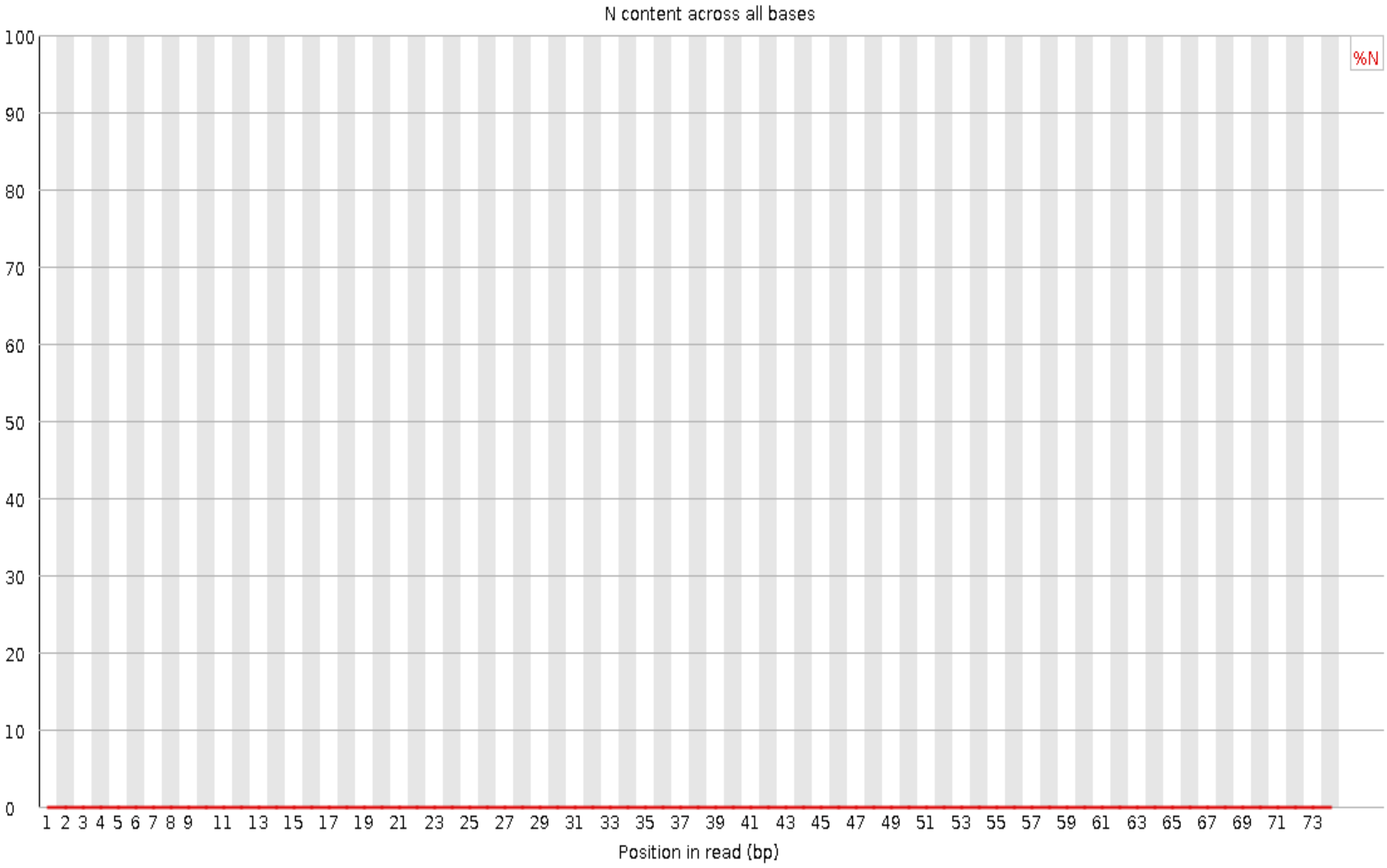


## Sequence Length Distribution

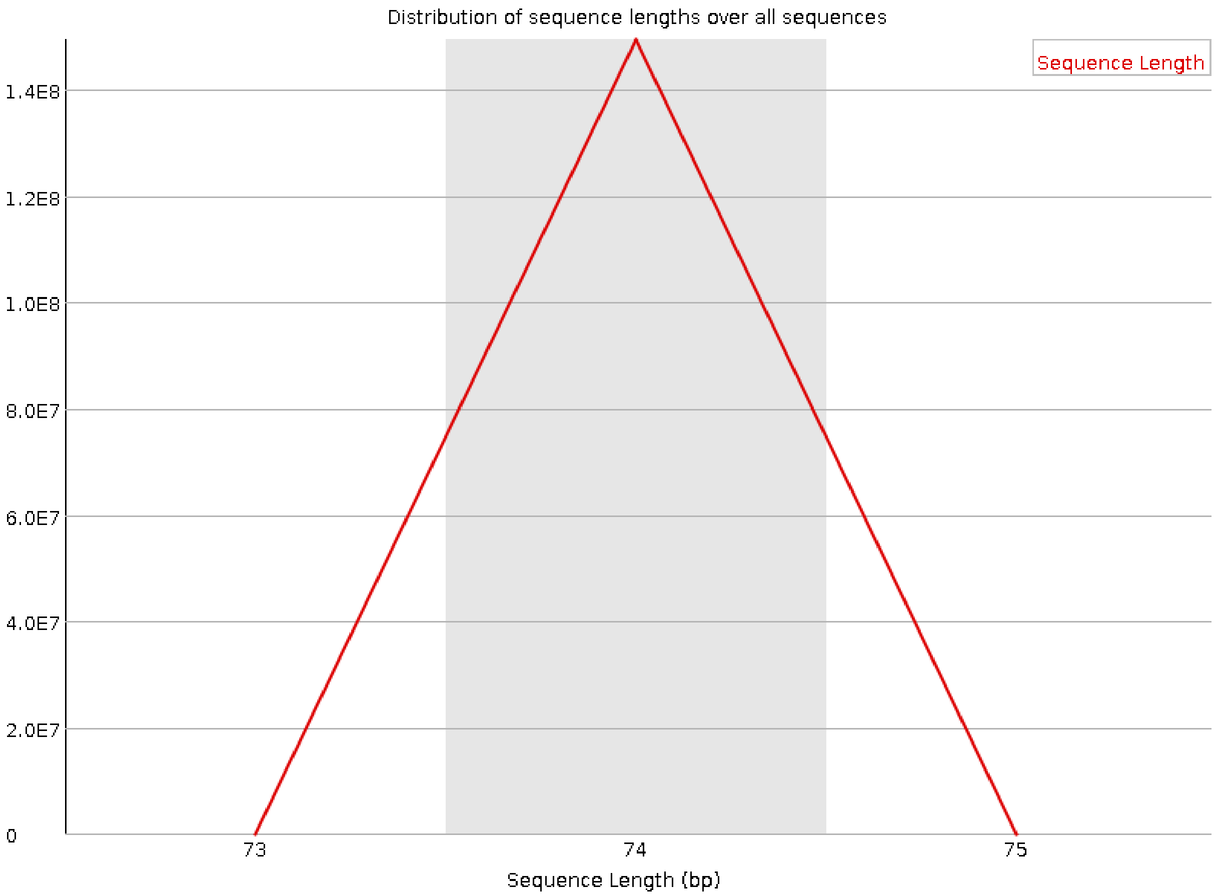


## Sequence Duplication Levels

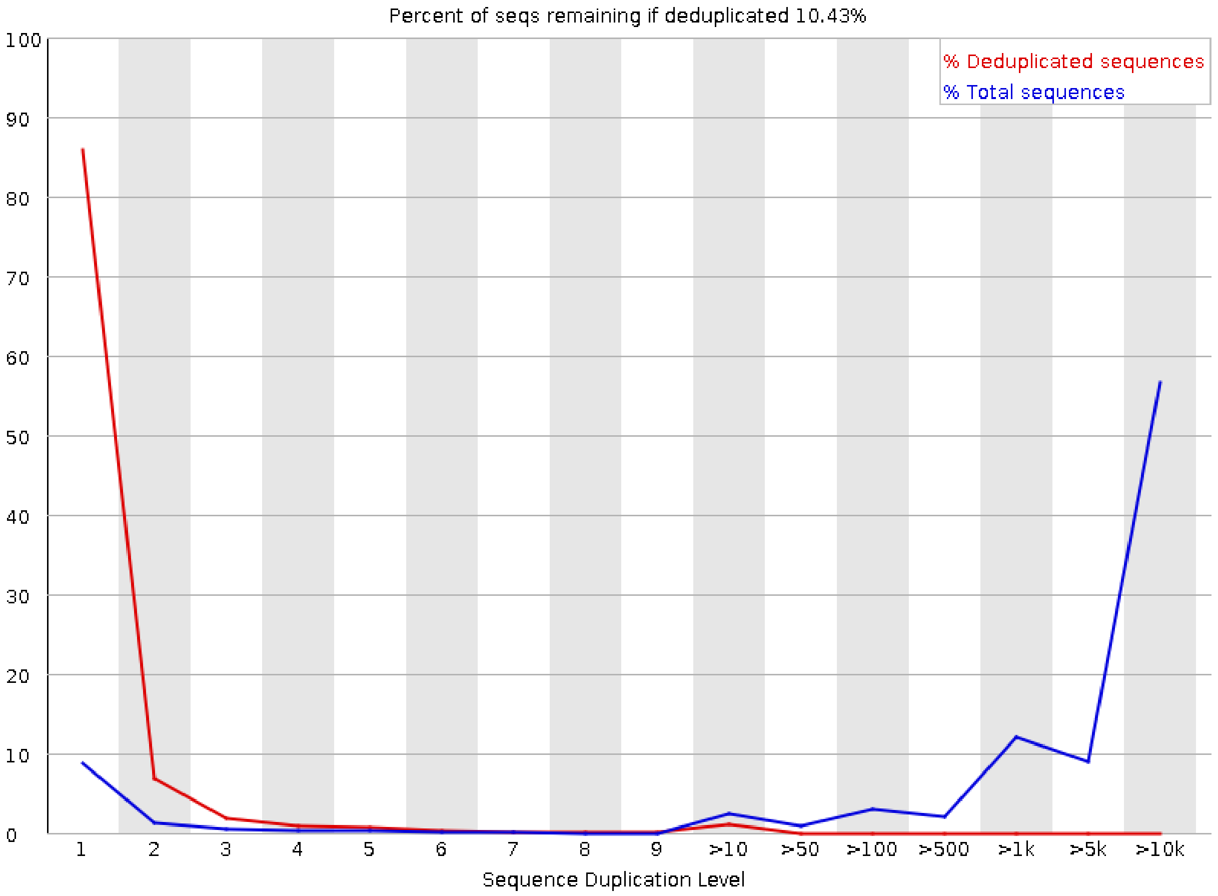

## Overrepresented sequences

| Sequence | Count | Percentage | Possible Source |
|---|---|---|---|
| TTGTCTCAAAGATTAAGCCATGCATGTCTAAGTACGCACGGCCGGTACAGTGAAACTGCGAATGGCTCATTAAA | 613563 | 0.4109934654729766 | No Hit |
| TGAATTTAAGCATATTAGTCAGCGGAGGAAAAGAAACTAACCAGGATTCCCTCAGTAACGGCGAGTGAACAGGG | 565083 | 0.3785192725928161 | No Hit |
| AAAGATTAAGCCATGCATGTCTAAGTACGCACGGCCGGTACAGTGAAACTGCGAATGGCTCATTAAATCAGTTA | 506093 | 0.3390049855053436 | No Hit |
| TTCACCGTGCCAGACTAGAGTCAAGCTCAACAGGGTCTTCTTTCCCCGCTGATTCCGCCAAGCCCGTTCCCTTG | 446325 | 0.2989695572862547 | No Hit |
| TGGATAGTAGGTAGGGACAGTGGGAATCTCGTTCATCCATTCATGCGCGTCACTAATTAGATGACGAGGCATTT | 420883 | 0.28192730449629916 | No Hit |
| TTGAACTCTCTCTTCAAAGTTCTTTTCAACTTTCCCTTACGGTACTTGTTGACTATCGGTCTCGTGCCGGTATT | 349793 | 0.23430786613304408 | No Hit |
| CGGTATTTAGCCTTAGATGGAGTTTACCACCCGCTTTGGGCTGCATTCCCAAGCAACCCGACTCCGGGAAGACC | 348922 | 0.23372442921063027 | No Hit |
| TTCCGTACGCCACATGTCCCGCGCCCCGCCGCGGGGCGGGGATTCGGCGCTGGGCTCTTCCCTGTTCACTCGCC | 342909 | 0.22969663791961534 | No Hit |
| TGAATTTAAGCATATTAGTCAGCGGAGGAGAAGAAACTAACCAGGATTCCCTCAGTAACGGCGAGTGAACAGGG | 331893 | 0.22231760102258877 | No Hit |
| TTTTCTGACACCTCCTGCTTAAAACCCAAAAGGTCAGAAGGATCGTGAGGCCCCGCTTTCACGGTCTGTATTCG | 328951 | 0.2203469105223117 | No Hit |
| TCTCTTCAAAGTTCTTTTCAACTTTCCCTTACGGTACTTGTTGACTATCGGTCTCGTGCCGGTATTTAGCCTTA | 325889 | 0.2182958383564897 | No Hit |
| TAGAATTACCACAGTTATCCAAGTAGGAGAGGAGCGAGCGACCAAAGGAACCATAACTGATTTAATGAGCCATT | 301178 | 0.2017432438791455 | No Hit |
| TAGAGTCAAGCTCAACAGGGTCTTCTTTCCCCGCTGATTCCGCCAAGCCCGTTCCCTTGGCTGTGGTTTCGCTG | 275352 | 0.18444376975944612 | No Hit |
| TCGACTGCCGGCGACGGCCGGGTATGGGCCCGACGCTCCAGCGCCATCCATTTTCAGGGCTAGTTGATTCGGCA | 267860 | 0.17942527444058967 | No Hit |
| TTTGGGCTGCATTCCCAAGCAACCCGACTCCGGGAAGACCCGGGCCCGGCGCGCCGGGGGCCGCTACCGGCCTC | 247821 | 0.1660022061417956 | No Hit |
| TTAGATGGAGTTTACCACCCGCTTTGGGCTGCATTCCCAAGCAACCCGACTCCGGGAAGACCCGGGCCCGGCGC | 245251 | 0.1642806988047079 | No Hit |
| GCGGTTCTATTTTGTTGGTTTTCGGAACTGAGGCCATGATTAAGAGGGACGGCCGGGGGCATTCGTATTGCGCC | 239811 | 0.16063672996667008 | No Hit |
| TTGGCTGTGGTTTCGCTGGATAGTAGGTAGGGACAGTGGGAATCTCGTTCATCCATTCATGCGCGTCACTAATT | 234506 | 0.15708319050237032 | No Hit |
| TCAAAGATTAAGCCATGCATGTCTAAGTACGCACGGCCGGTACAGTGAAACTGCGAATGGCTCATTAAATCAGT | 232888 | 0.15599937771193925 | No Hit |
| TTTAAATGGGTAAGAAGCCCGGCTCGCTGGCGTGGAGCCGGGCGTGGAATGCGAGTGCCTAGTGGGCCACTTTT | 220113 | 0.14744207956746627 | No Hit |
| GAGATTCCCACTGTCCCTACCTACTATCCAGCGAAACCACAGCCAAGGGAACGGGCTTGGCGGAATCAGCGGGG | 214693 | 0.14381150767368595 | No Hit |
| TTTAGCCTTAGATGGAGTTTACCACCCGCTTTGGGCTGCATTCCCAAGCAACCCGACTCCGGGAAGACCCGGGC | 211071 | 0.14138532106865415 | No Hit |
| TTAGAGCCAATCCTTATCCCGAAGTTACGGATCCGGCTTGCCGACTTCCCTTACCTACATTGTTCCAACATGCC | 208423 | 0.1396115656489622 | No Hit |
| CGACATCGAAGGATCAAAAAGCGACGTCGCTATGAACGCTTGGCCGCCACAAGCCAGTTATCCCTGTGGTAACT | 203198 | 0.13611161396169244 | No Hit |
| GAACGTCTGCCCTATCAACTTTCGATGGTAGTCGCCGTGCCTACCATGGTGACCACGGGTGACGGGGAATCAGG | 202902 | 0.13591333918668158 | No Hit |
| AGAAACCTCCCGTGGAGCAGAAGGGCAAAAGCTCGCTTGATCTTGATTTTCAGTACGAATACAGACCGTGAAAG | 202267 | 0.1354879862065062 | No Hit |
| CCACATGTCCCGCGCCCCGCCGCGGGGCGGGGATTCGGCGCTGGGCTCTTCCCTGTTCACTCGCCGTTACTGAG | 199167 | 0.13341145984659494 | No Hit |
| GGTATTTAGCCTTAGATGGAGTTTACCACCCGCTTTGGGCTGCATTCCCAAGCAACCCGACTCCGGGAAGACCC | 194324 | 0.13016738979464326 | No Hit |
| TTAAGGTAGCCAAATGCCTCGTCATCTAATTAGTGACGCGCATGAATGGATGAACGAGATTCCCACTGTCCCTA | 192218 | 0.12875669156432937 | No Hit |
| GACGCTCCAGCGCCATCCATTTTCAGGGCTAGTTGATTCGGCAGGTGAGTTGTTACACACTCCTTAGCGGATTC | 186609 | 0.12499951854731575 | No Hit |
| TTAGCTCTAGAATTACCACAGTTATCCAAGTAGGAGAGGAGCGAGCGACCAAAGGAACCATAACTGATTTAATG | 185584 | 0.12431292515411928 | No Hit |
| TCGCATTCCACGCCCGGCTCCACGCCAGCGAGCCGGGCTTCTTACCCATTTAAAGTTTGAGAATAGGTTGAGAT | 185304 | 0.12412536793451441 | No Hit |
| GGCATCGGGCGCCTTAACCCGGCGTTCGGTTCATCCCGCAGCGCCAGTTCTGCTTACCAAAAGTGGCCCACTAG | 184108 | 0.12332423066791637 | No Hit |
| ATGTATTAGCTCTAGAATTACCACAGTTATCCAAGTAGGAGAGGAGCGAGCGACCAAAGGAACCATAACTGATT | 177845 | 0.11912897757368278 | No Hit |
| TCAAAGTGAAGAAATTCAATGAAGCGCGGGTAAACGGCGGGAGTAACTATGACTCTCTTAAGGTAGCCAAATGC | 175425 | 0.11750794731852626 | No Hit |
| TTCTTTTCAACTTTCCCTTACGGTACTTGTTGACTATCGGTCTCGTGCCGGTATTTAGCCTTAGATGGAGTTTA | 173313 | 0.1160932300049351 | No Hit |
| TCATGTCTCTTCACCGTGCCAGACTAGAGTCAAGCTCAACAGGGTCTTCTTTCCCCGCTGATTCCGCCAAGCCC | 169393 | 0.11346742893046668 | No Hit |
| TGACACCTCCTGCTTAAAACCCAAAAGGTCAGAAGGATCGTGAGGCCCCGCTTTCACGGTCTGTATTCGTACTG | 168458 | 0.11284112178642892 | No Hit |
| GAAGGCCCGCGGCGGGTGTTGACGCGATGTGATTTCTGCCCAGTGCTCTGAATGTCAAAGTGAAGAAATTCAAT | 166394 | 0.11145855713905577 | No Hit |
| TTTTATCCGGTAAAGCGAATGATTAGAGGTCTTGGGGCCGAAACGATCTCAACCTATTCTCAAACTTTAAATGG | 156471 | 0.10481166324570113 | No Hit |
| CACTCTCGACTGCCGGCGACGGCCGGGTATGGGCCCGACGCTCCAGCGCCATCCATTTTCAGGGCTAGTTGATT | 156081 | 0.10455042283268004 | No Hit |
| TTAACCCGGCGTTCGGTTCATCCCGCAGCGCCAGTTCTGCTTACCAAAAGTGGCCCACTAGGCACTCGCATTCC | 155802 | 0.10436353546028802 | No Hit |
| CAGACTAGAGTCAAGCTCAACAGGGTCTTCTTTCCCCGCTGATTCCGCCAAGCCCGTTCCCTTGGCTGTGGTTT | 155453 | 0.1041297587829948 | No Hit |
| GCAGGTGTCCTAAGGCGAGCTCAGGGAGGACAGAAACCTCCCGTGGAGCAGAAGGGCAAAAGCTCGCTTGATCT | 150574 | 0.10086157423137962 | No Hit |

| Sequence | Count | Percentage | Possible Source |
|---|---|---|---|
| TTAGGCAACCTGGTGGTCCCCCGCTCCCGGGAGGTCACCATATTGATGCCGAACTTAGTGCGGACACCCGATCG | 150120 | 0.10055746359673456 | [illegible] |
| AACCAACACCTTTTCTGGGGTCTGATGAGCGTCGGCATCGGGCGCCTTAACCCGGCGTTCGGTTCATCCCGCAG | 149816 | 0.10035383004402068 | [illegible] |
| TTTAAGCATATTAGTCAGCGGAGGAAAAGAAACTAACCAGGATTCCCTCAGTAACGGCGAGTGAACAGGGAAGA | 149795 | 0.10033976325255031 | No Hit |

## Adapter Content

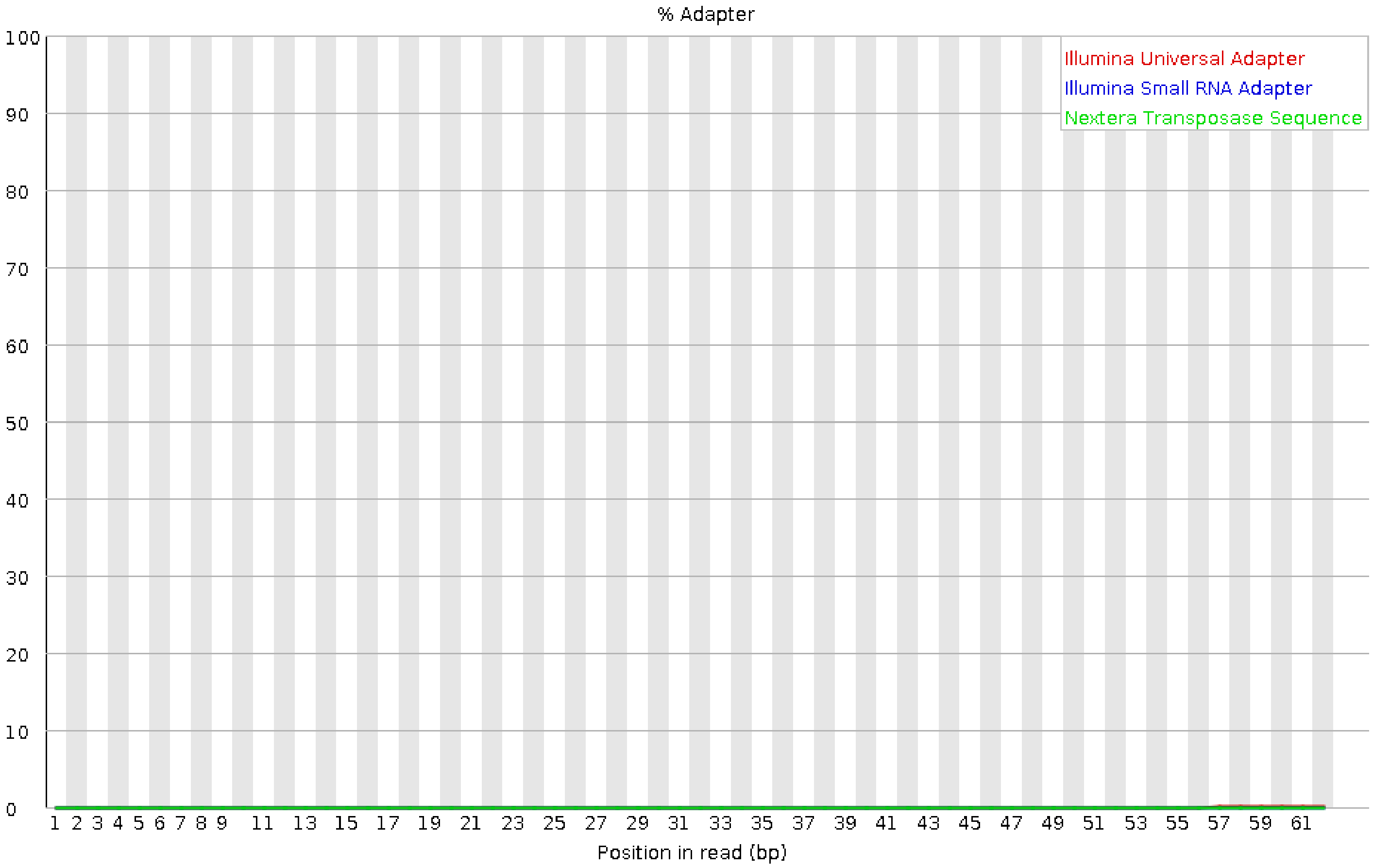


## Kmer Content

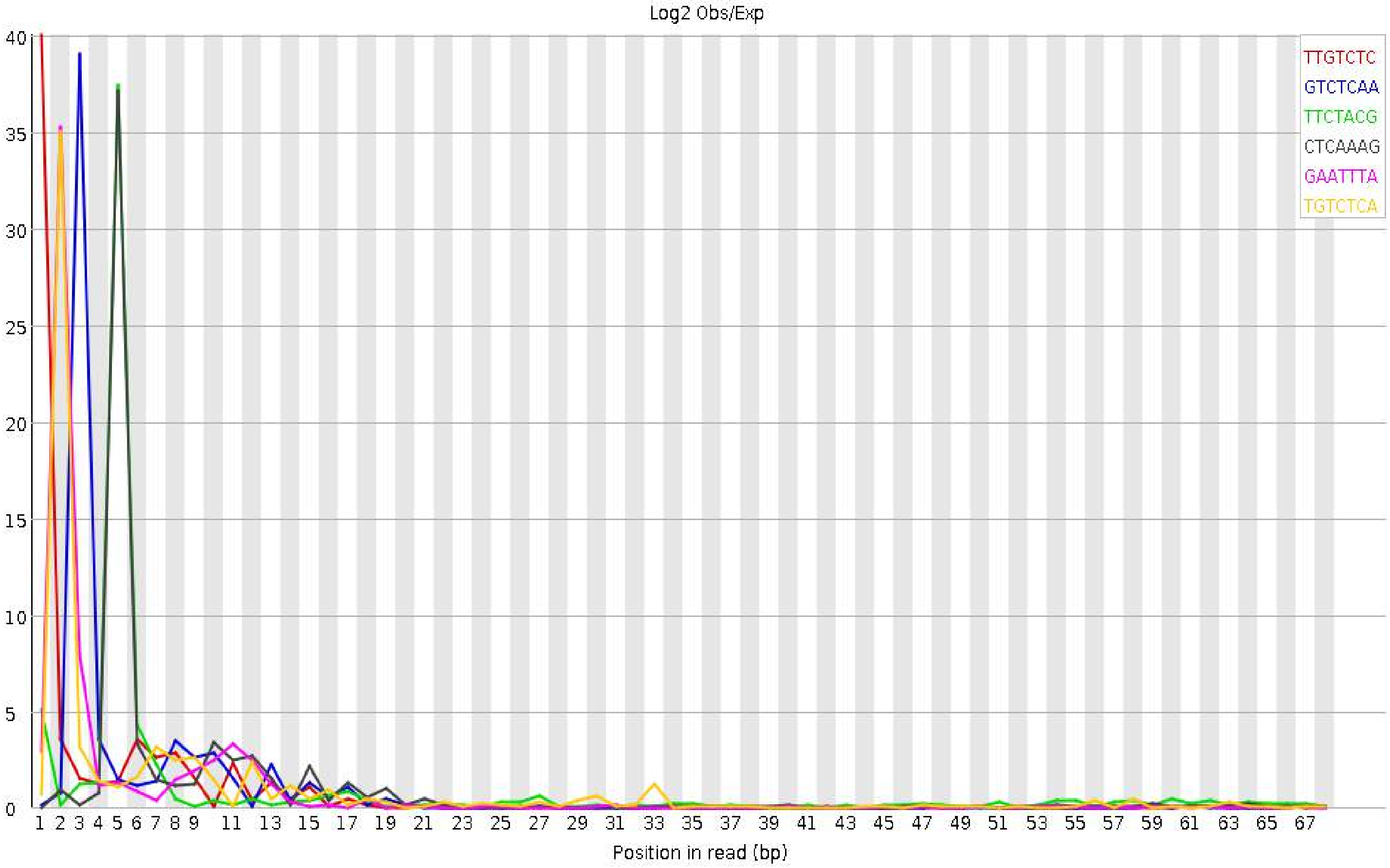


| Sequence | Count | PValue | Obs/Exp Max | Max Obs/Exp Position |
|---|---|---|---|---|
| TTGTCTC | 134460 | 0.0 | 40.025673 | 1 |
| GTCTCAA | 137280 | 0.0 | 39.041737 | 3 |
| TTCTACG | 12130 | 0.0 | 37.43733 | 5 |
| CTCAAAG | 144825 | 0.0 | 37.127415 | 5 |
| GAATTTA | 226875 | 0.0 | 35.272385 | 2 |
| TGTCTCA | 153460 | 0.0 | 35.062706 | 2 |
| ATTTAAG | 232215 | 0.0 | 34.3149 | 4 |
| TCTACGT | 13305 | 0.0 | 33.799118 | 6 |
| AATTTAA | 255795 | 0.0 | 31.554277 | 3 |
| TTTTTCG | 18340 | 0.0 | 31.437637 | 1 |
| GCCTGTA | 53080 | 0.0 | 31.141045 | 3 |
| TCAGTTA | 137755 | 0.0 | 30.395632 | 68 |
| ATCAGTT | 137505 | 0.0 | 30.3603 | 67 |
| TAAGCAT | 263880 | 0.0 | 30.142134 | 7 |
| CCTGTAG | 55485 | 0.0 | 29.589073 | 4 |
| GCGTAAC | 41425 | 0.0 | 28.911692 | 1 |
| AAGCATA | 278695 | 0.0 | 28.522787 | 8 |
| ACGACCC | 47315 | 0.0 | 28.520119 | 2 |
| CAAAGAT | 188460 | 0.0 | 28.50083 | 7 |
| GCATATT | 278025 | 0.0 | 28.452147 | 10 |

**Produced by FastQC (version 0.11.2)**

# FastQC Report

Tue 28 Apr 2015
Agilent_Adult_Stomach_GCCAAT_R2.fastq.gz

## Summary


- Basic Statistics
- Per base sequence quality
- Per tile sequence quality
- Per sequence quality scores
- Per base sequence content
- Per sequence GC content
- Per base N content
- Sequence Length Distribution
- Sequence Duplication Levels
- Overrepresented sequences
- Adapter Content
- Kmer Content


## Basic Statistics

| Measure | Value |
| --- | --- |
| Filename | Agilent_Adult_Stomach_GCCAAT_R2.fastq.gz |
| File type | Conventional base calls |
| Encoding | Sanger / Illumina 1.9 |
| Total Sequences | 149287775 |
| Sequences flagged as poor quality | 0 |
| Sequence length | 74 |
| %GC | 57 |

## Per base sequence quality

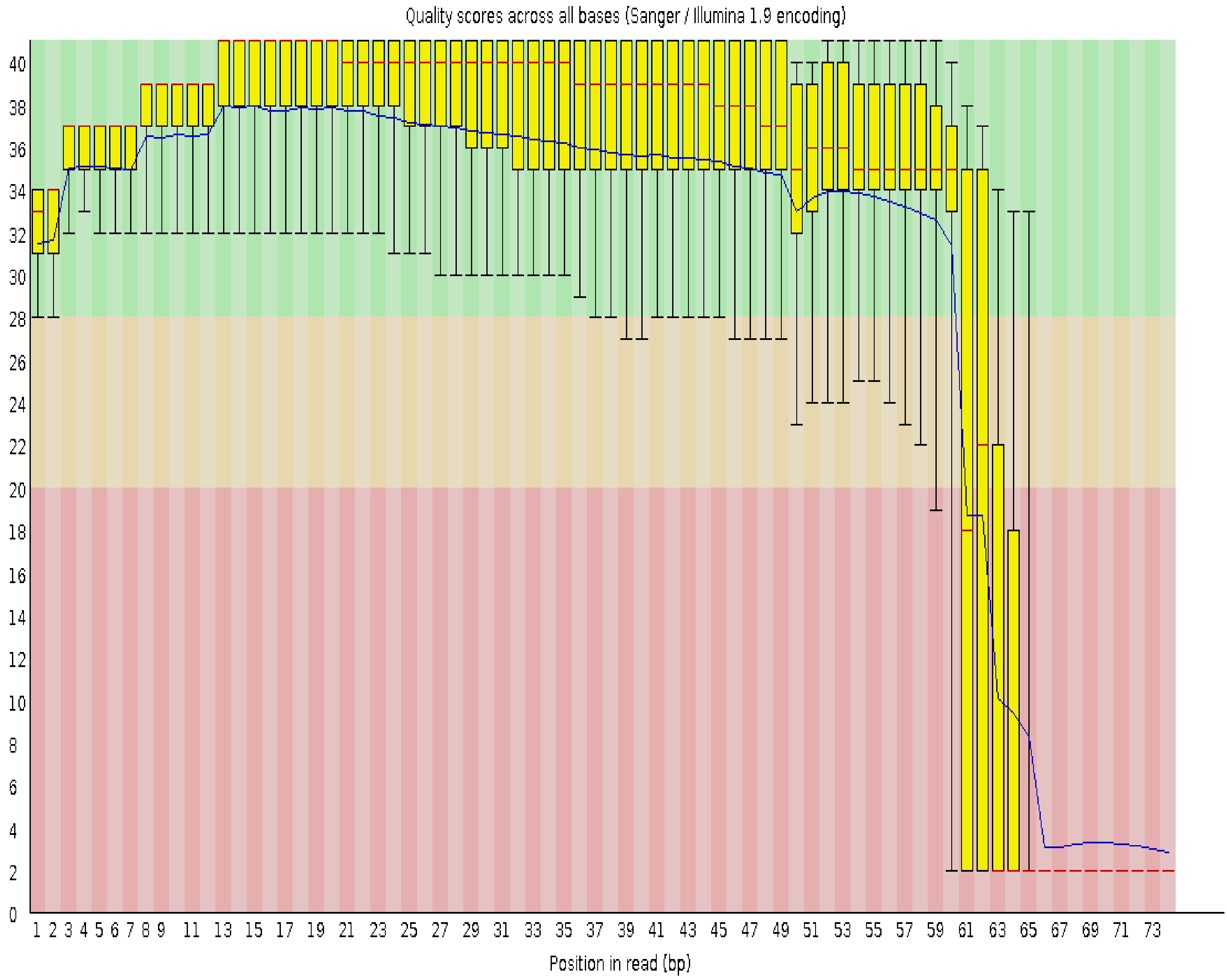


## Per tile sequence quality

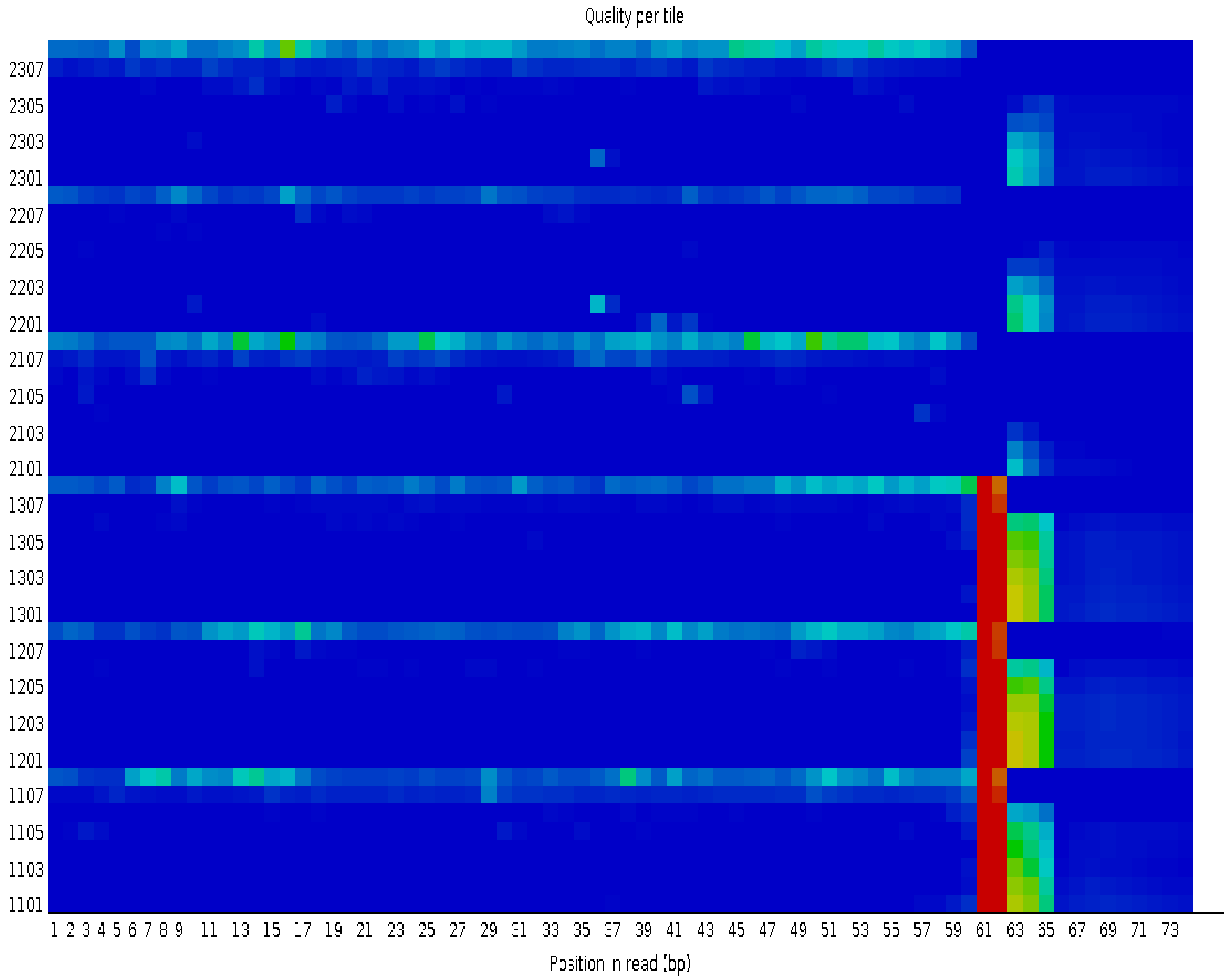


## Per sequence quality scores

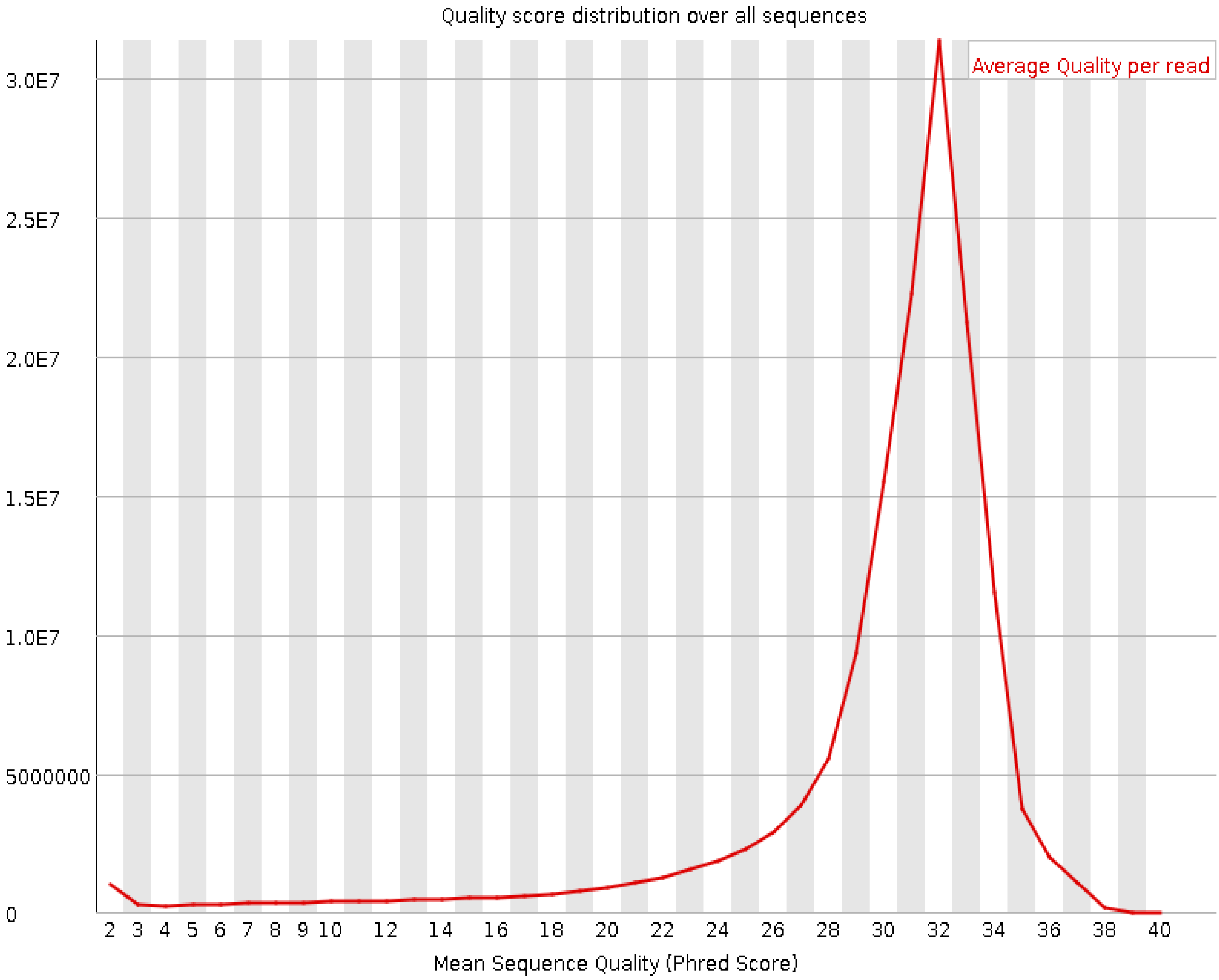


## Per base sequence content

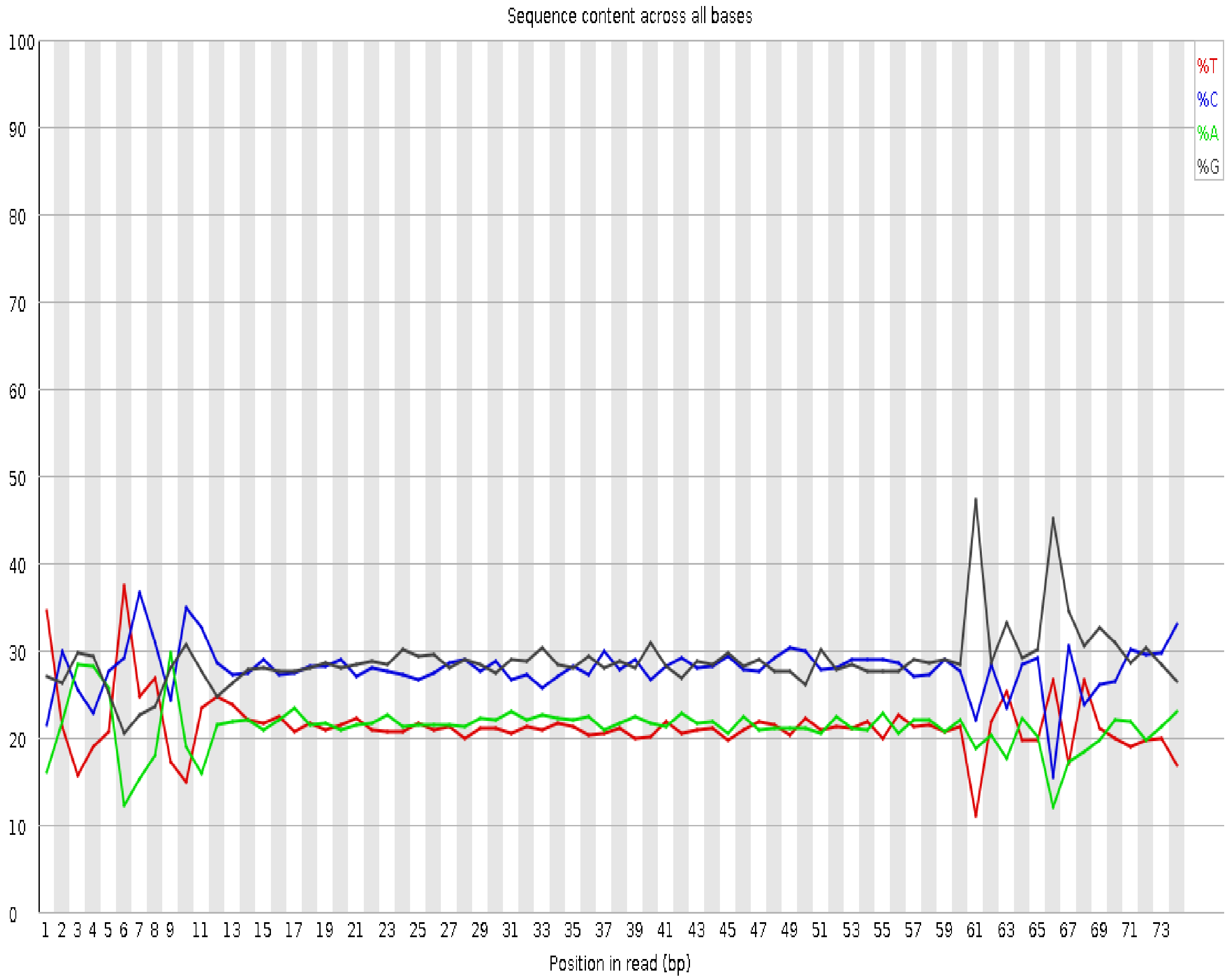


## Per sequence GC content

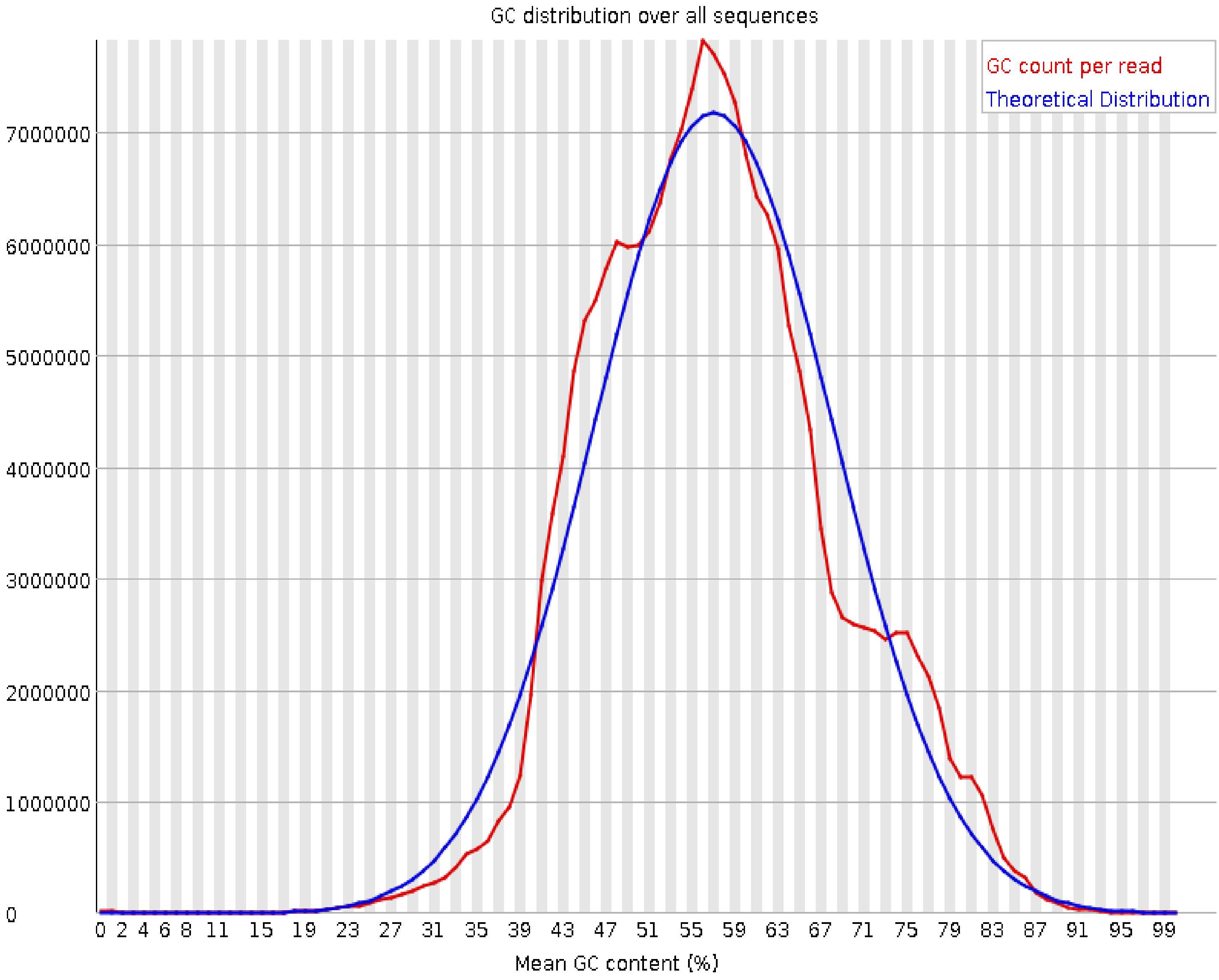


## Per base N content

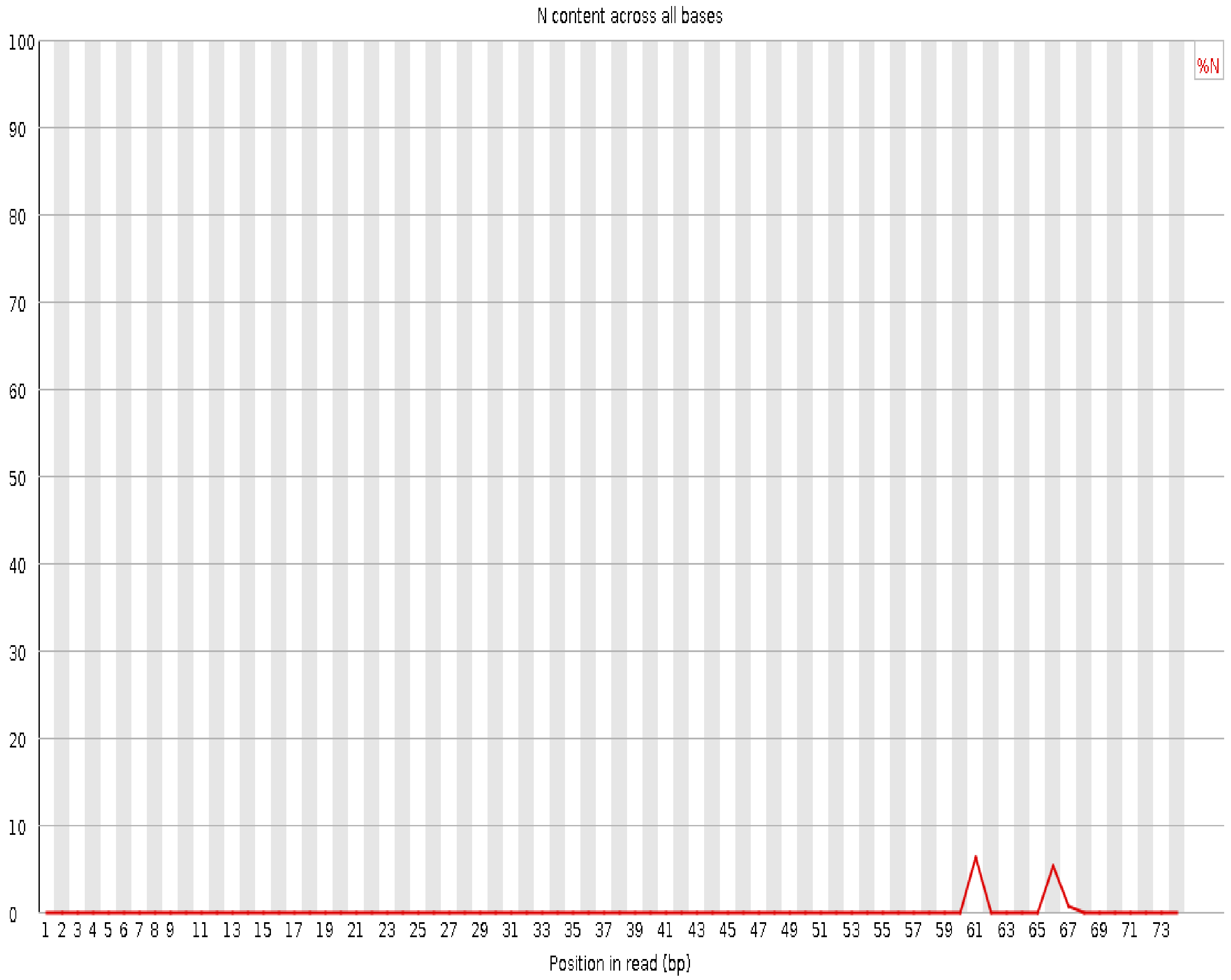


## Sequence Length Distribution

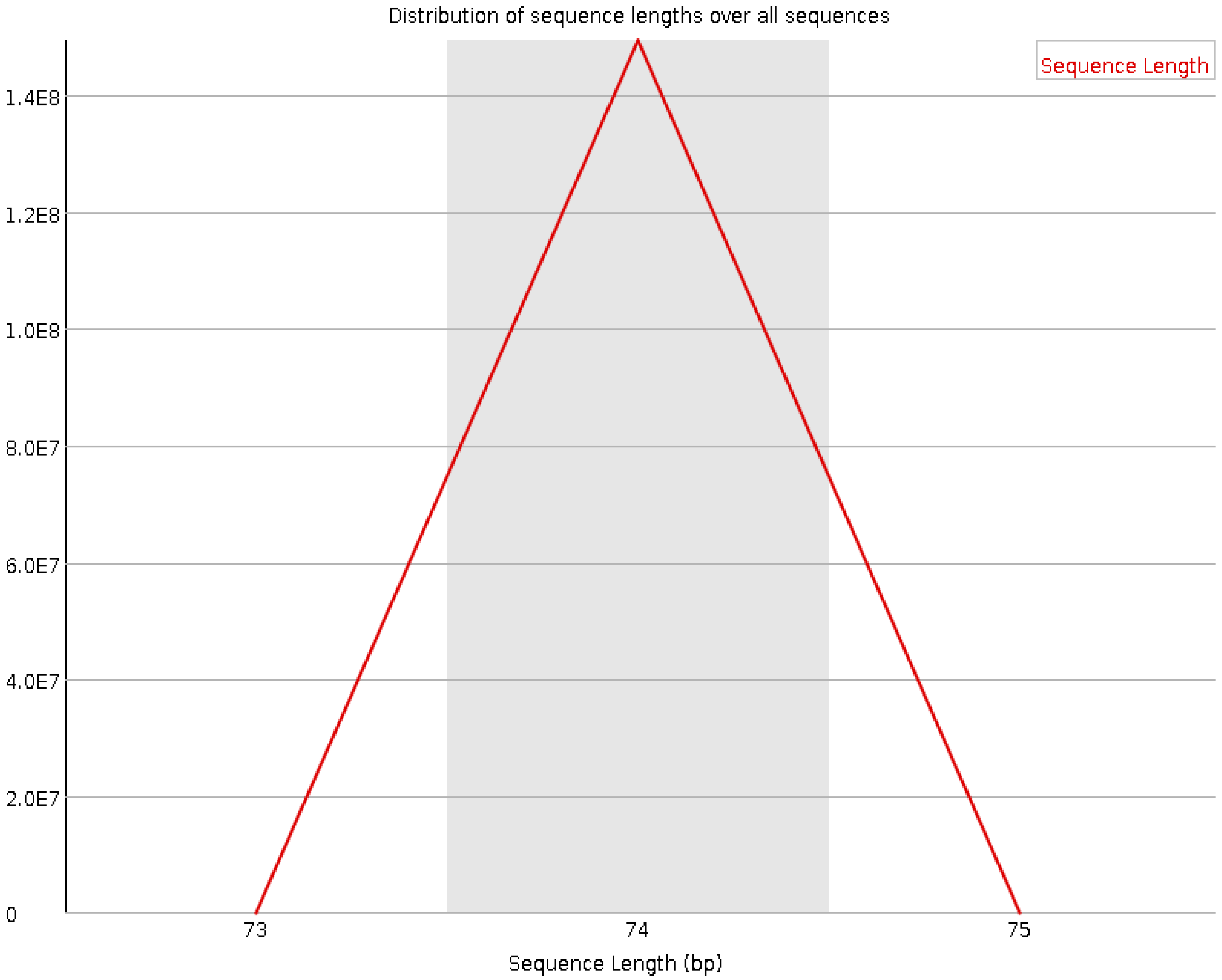


## Sequence Duplication Levels

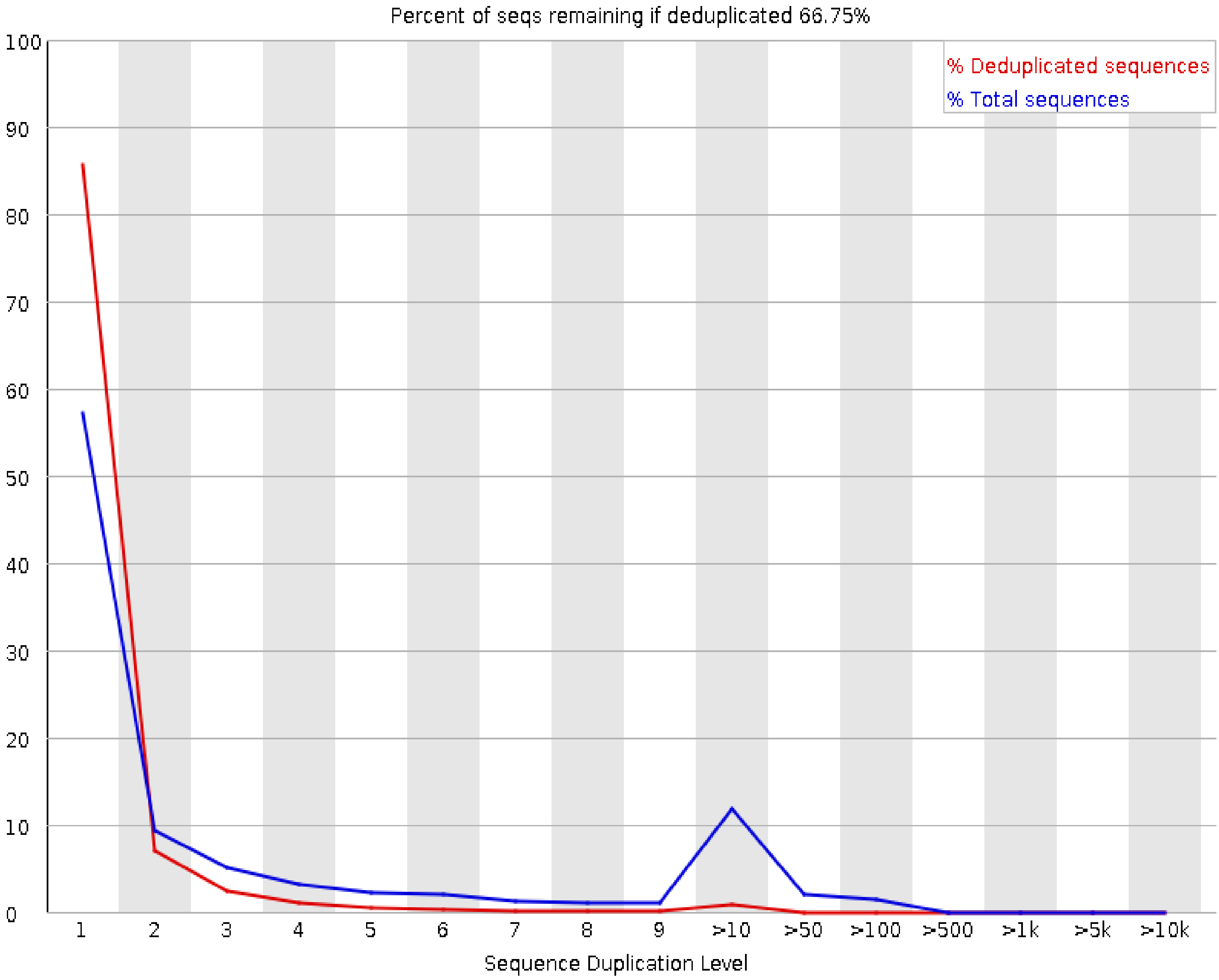


## Overrepresented sequences

No overrepresented sequences

## Adapter Content

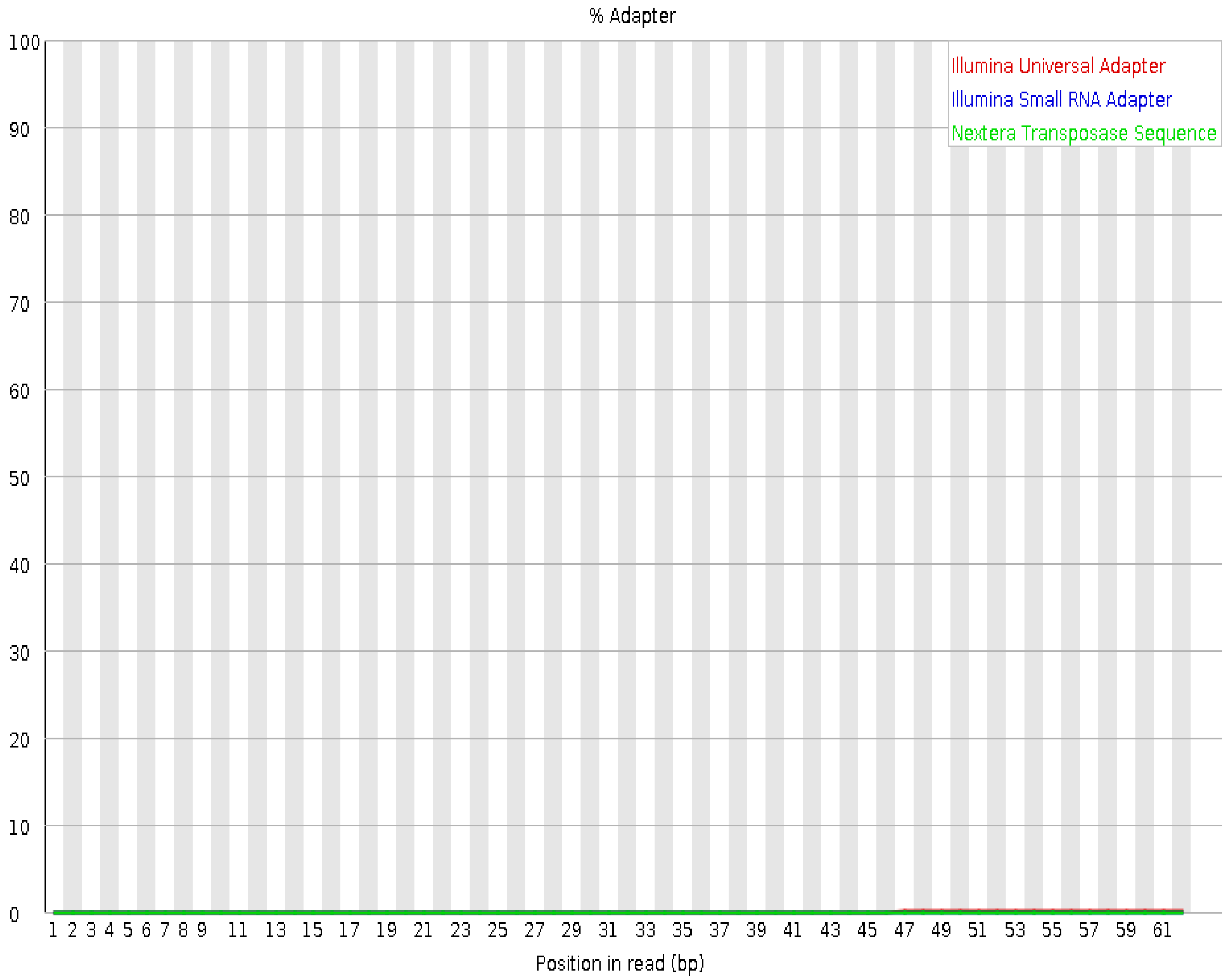


## Kmer Content

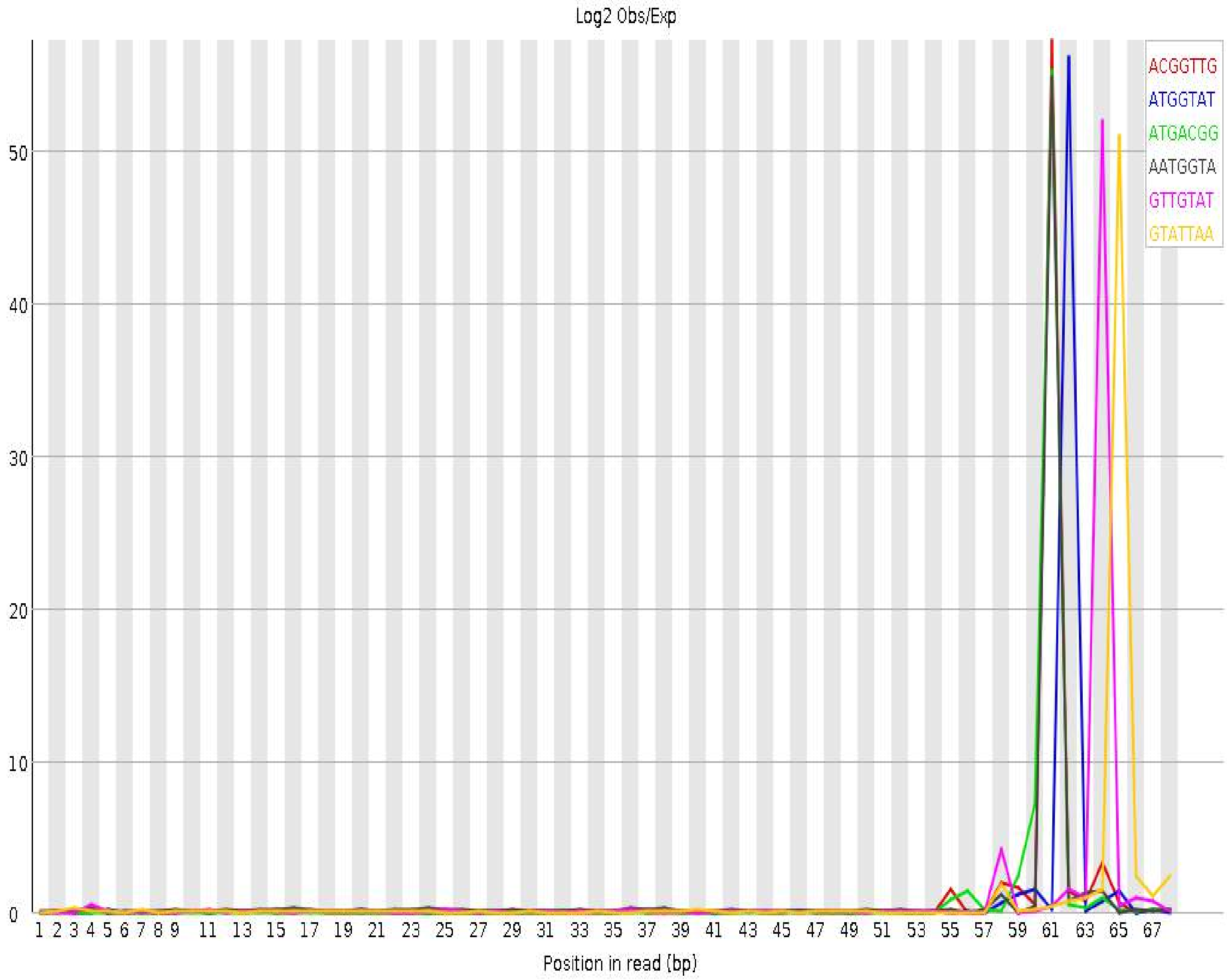


| Sequence | Count | PValue | Obs/Exp Max | Max Obs/Exp Position |
|---|---|---|---|---|
| ACGGTTG | 25190 | 0.0 | 57.262897 | 61 |
| ATGGTAT | 25655 | 0.0 | 56.16897 | 62 |
| ATGACGG | 41820 | 0.0 | 55.35652 | 61 |
| AATGGTA | 23015 | 0.0 | 54.89541 | 61 |
| GTTGTAT | 22320 | 0.0 | 52.08698 | 64 |
| GTATTAA | 30180 | 0.0 | 51.06212 | 65 |
| CAGTTAG | 17615 | 0.0 | 50.76837 | 55 |
| ACACACG | 15325 | 0.0 | 50.299267 | 55 |
| TGAGTTA | 18345 | 0.0 | 49.94202 | 58 |
| TCATTAC | 14485 | 0.0 | 49.75152 | 60 |
| CACGGTT | 30245 | 0.0 | 49.037476 | 60 |
| TCACGCG | 17055 | 0.0 | 47.785854 | 57 |
| GCGAGTT | 20240 | 0.0 | 47.67822 | 59 |
| GTTCACG | 18365 | 0.0 | 47.48356 | 55 |

| Sequence | Count | PValue | Obs/Exp Max | Max Obs/Exp Position |
|---|---|---|---|---|
| ACTGAGT | 22465 | 0.0 | 46.[illegible] | 56 |
| GGTTGTA | 25365 | 0.0 | 46.12[illegible]4 | 63 |
| TACAGAG | 17425 | 0.0 | 45.87804 | 60 |
| ATAGTTG | 16010 | 0.0 | 45.762264 | 63 |
| TTAGCCG | 13060 | 0.0 | 45.58596 | 55 |
| TATTCGA | 10495 | 0.0 | 45.49463 | 68 |

# FastQC Report

Mon 4 May 2015
Agilent_Fetal_Colon_CGATGT_L008_R1.fastq.gz

## Summary


- Basic Statistics
- Per base sequence quality
- Per tile sequence quality
- Per sequence quality scores
- Per base sequence content
- Per sequence GC content
- Per base N content
- Sequence Length Distribution
- Sequence Duplication Levels
- Overrepresented sequences
- Adapter Content
- Kmer Content


## Basic Statistics

| Measure | Value |
|---|---|
| Filename | Agilent_Fetal_Colon_CGATGT_L008_R1.fastq.gz |
| File type | Conventional base calls |
| Encoding | Sanger / Illumina 1.9 |
| Total Sequences | 174262897 |
| Sequences flagged as poor quality | 0 |
| Sequence length | 74 |
| %GC | 52 |

## Per base sequence quality

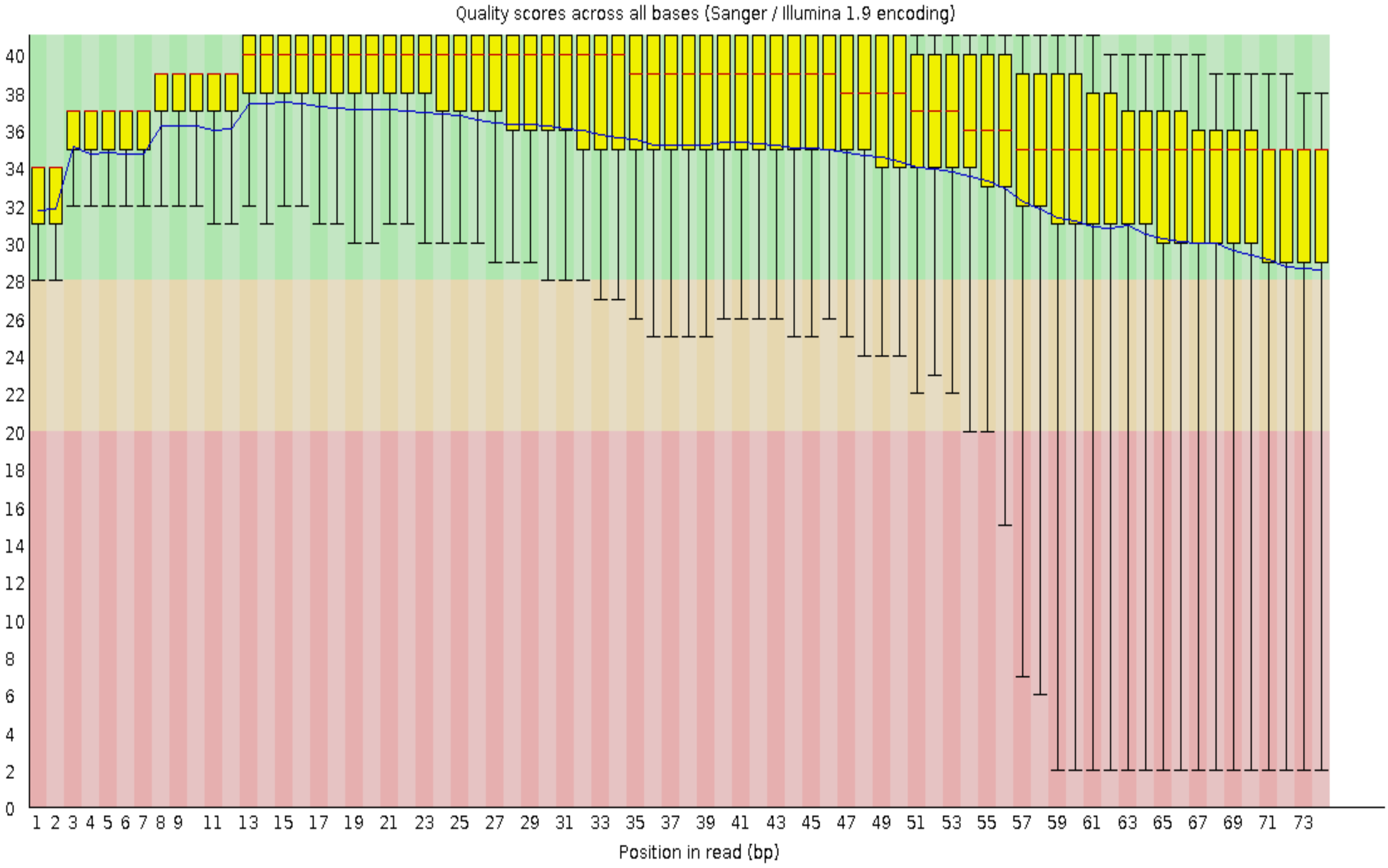


## Per tile sequence quality

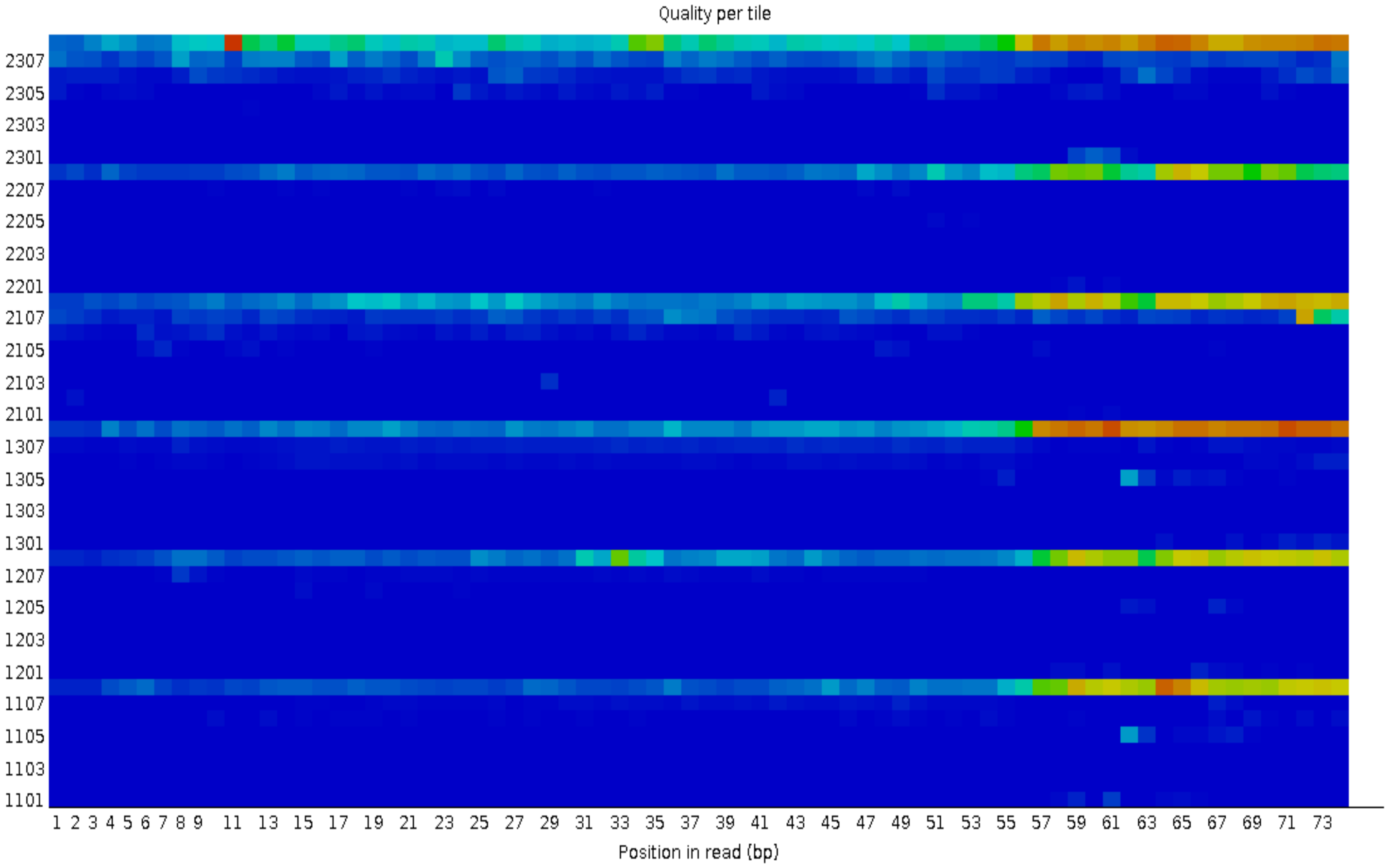

## Per sequence quality scores

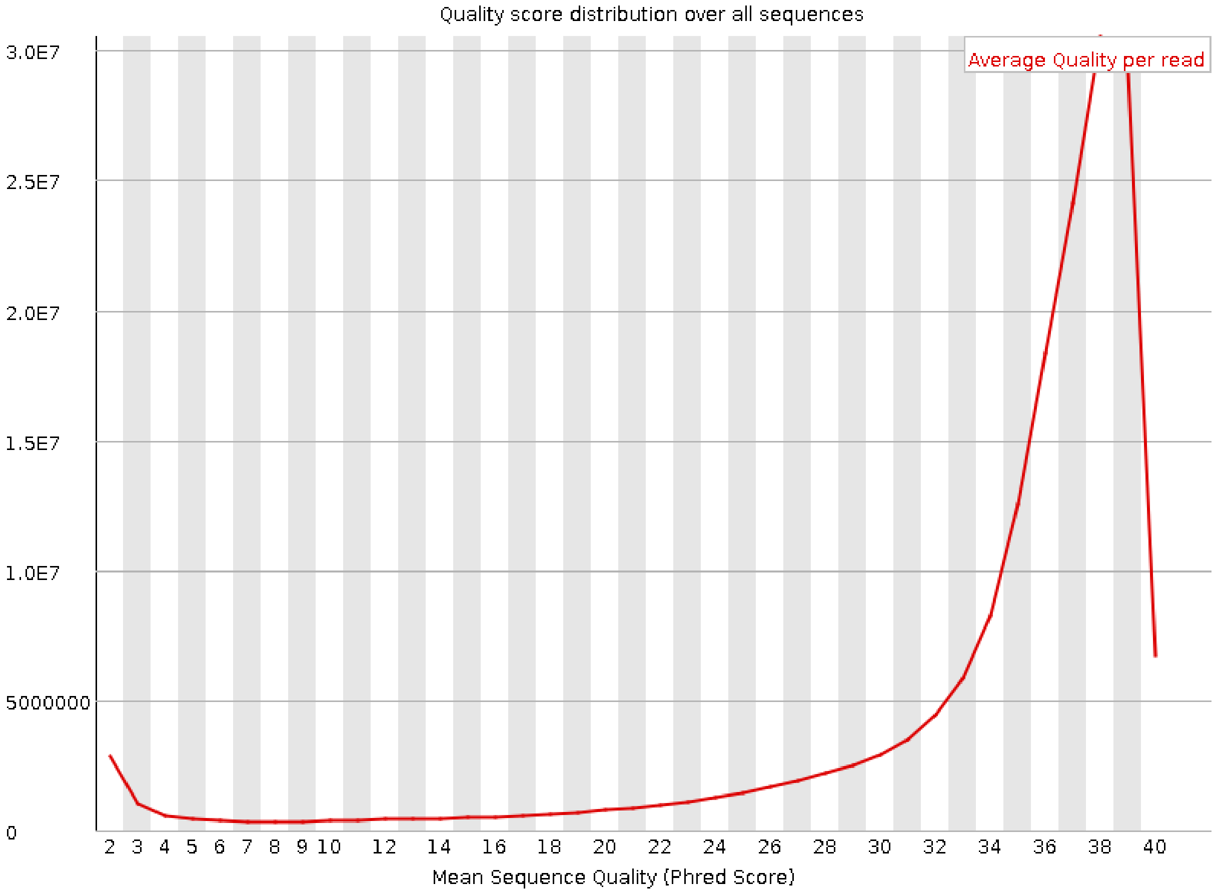


## Per base sequence content

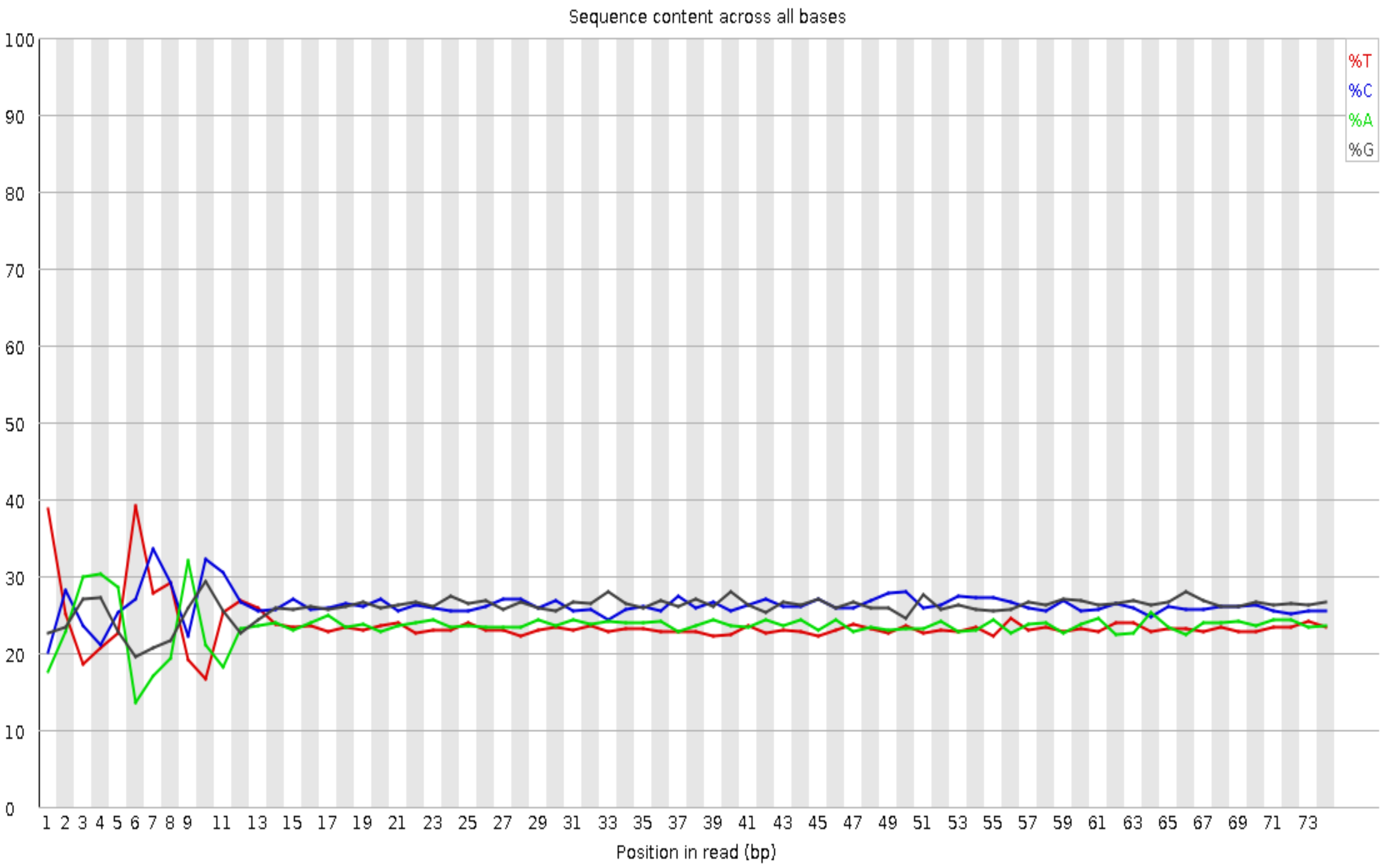


## Per sequence GC content

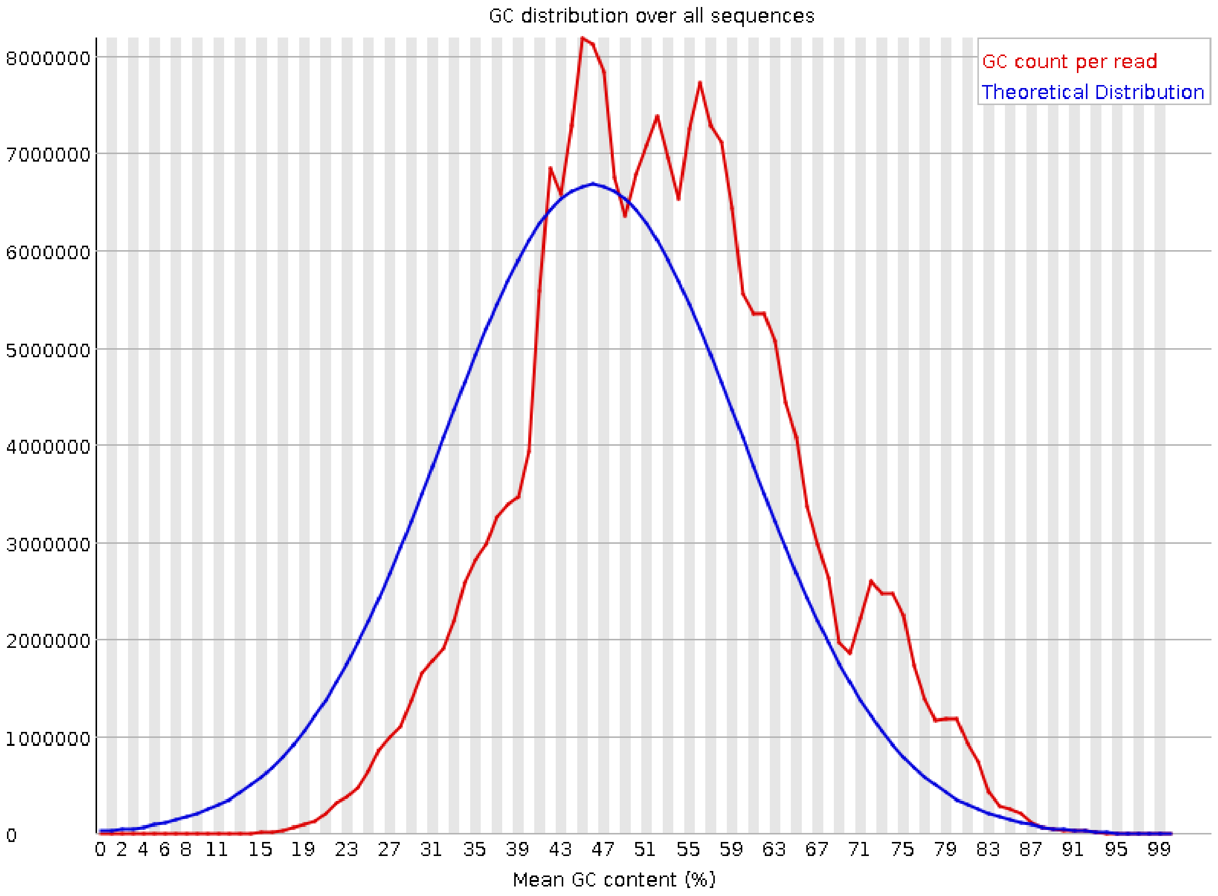

## Per base N content

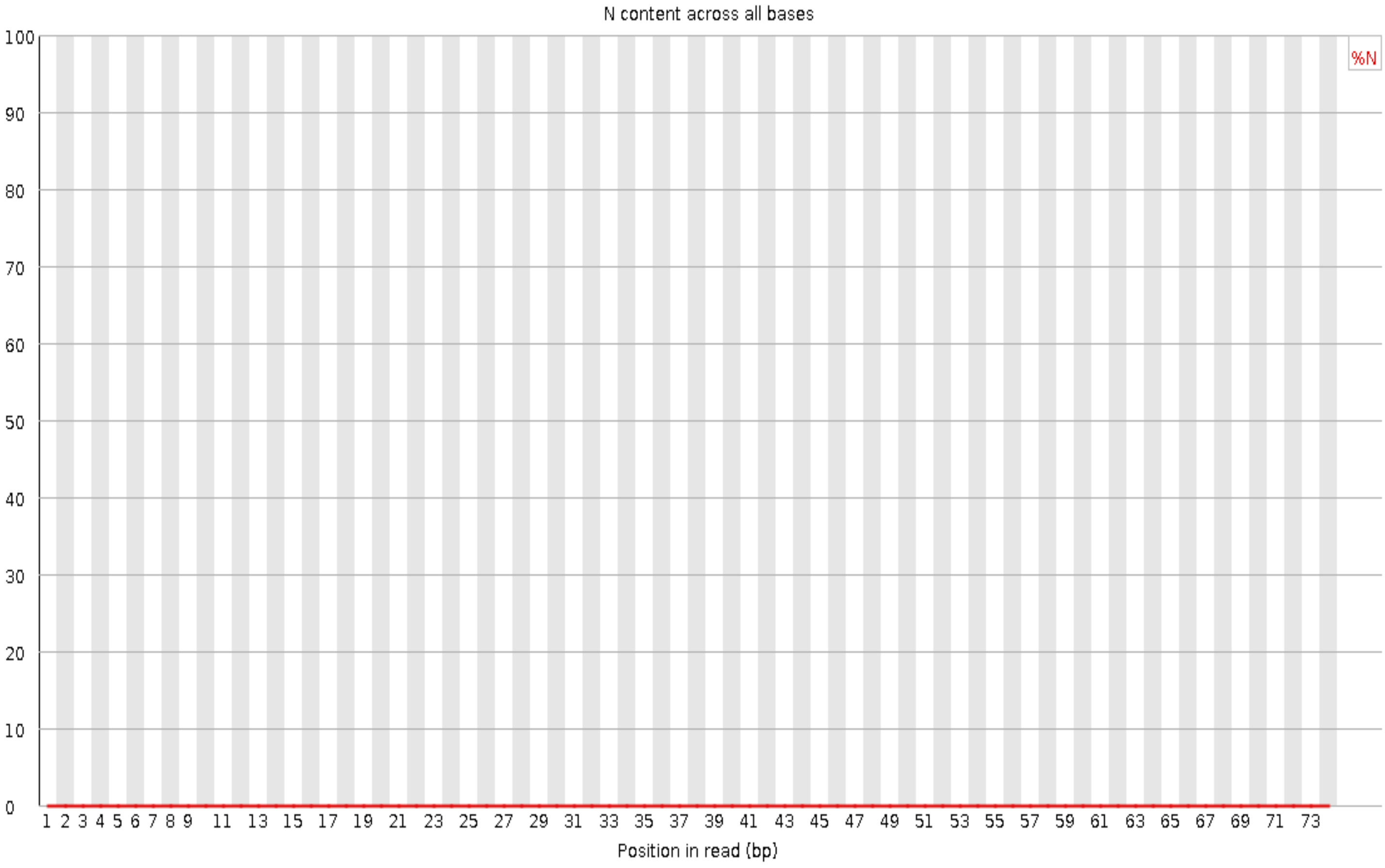


## Sequence Length Distribution

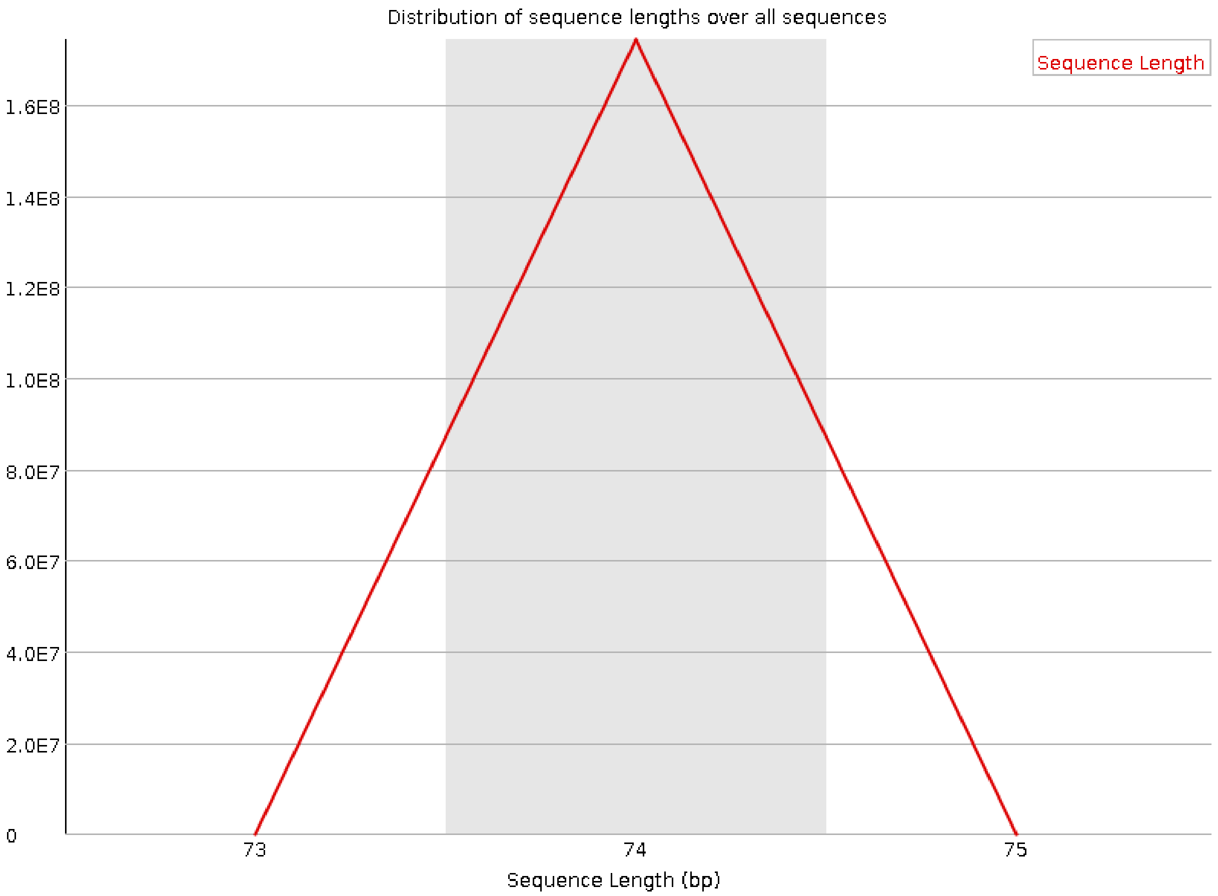


## Sequence Duplication Levels

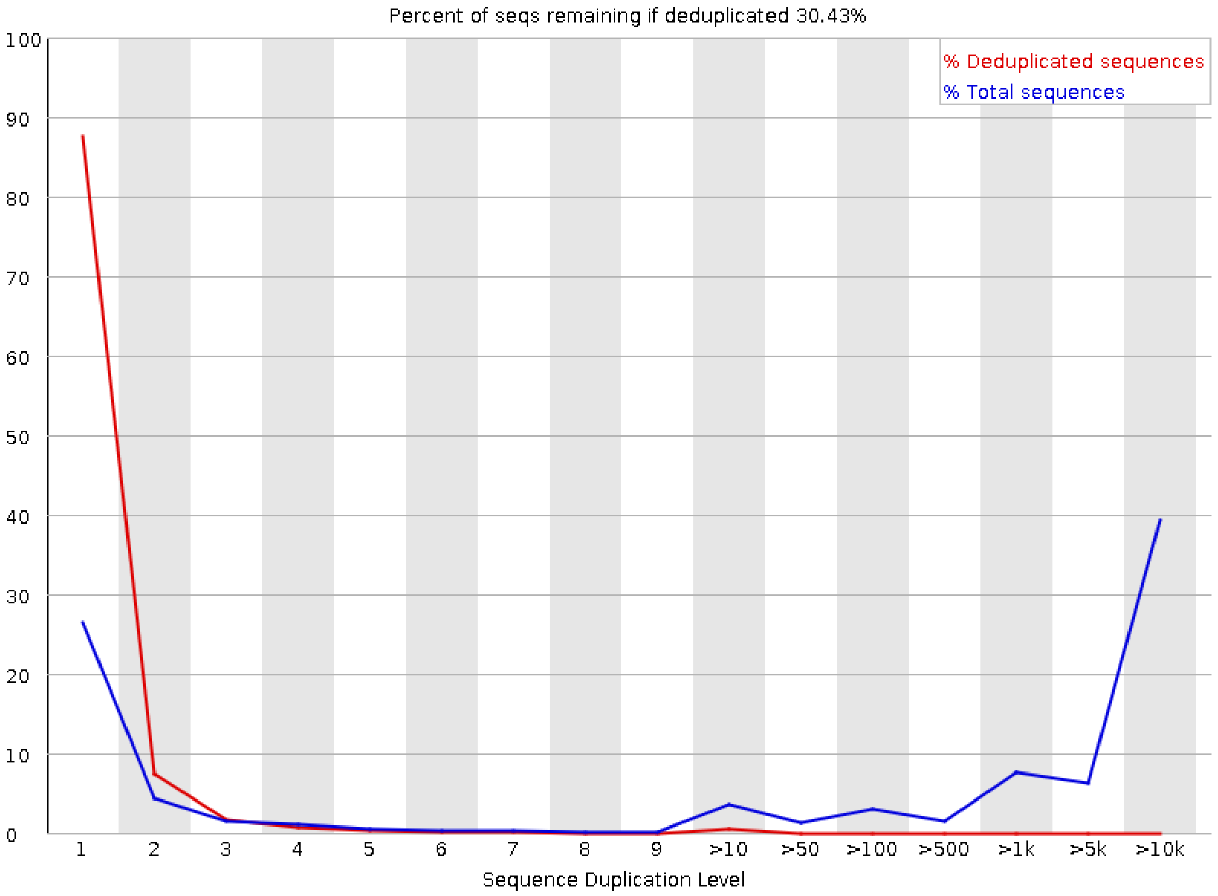

## Overrepresented sequences

| Sequence | Count | Percentage | Possible Source |
|---|---|---|---|
| TTGAACTCTCTCTTCAAAGTTCTTTTCAACTTTCCCTTACGGTACTTGTTGACTATCGGTCTCGTGCCGGTATT | 554221 | 0.31803729281511944 | No Hit |
| TGAATTTAAGCATATTAGTCAGCGGAGGAAAAGAAACTAACCAGGATTCCCTCAGTAACGGCGAGTGAACAGGG | 517365 | 0.2968876386807686 | No Hit |
| TTGTCTCAAAGATTAAGCCATGCATGTCTAAGTACGCACGGCCGGTACAGTGAAACTGCGAATGGCTCATTAAA | 494642 | 0.28384814467993147 | No Hit |
| CGGTATTTAGCCTTAGATGGAGTTTACCACCCGCTTTGGGCTGCATTCCCAAGCAACCCGACTCCGGGAAGACC | 470902 | 0.2702250496845579 | No Hit |
| TGGATAGTAGGTAGGGACAGTGGGAATCTCGTTCATCCATTCATGCGCGTCACTAATTAGATGACGAGGCATTT | 434532 | 0.24935428452104755 | No Hit |
| AAAGATTAAGCCATGCATGTCTAAGTACGCACGGCCGGTACAGTGAAACTGCGAATGGCTCATTAAATCAGTTA | 404901 | 0.23235066498406717 | No Hit |
| TTCCGTACGCCACATGTCCCGCGCCCCGCCGCGGGGCGGGGATTCGGCGCTGGGCTCTTCCCTGTTCACTCGCC | 398173 | 0.22848983165934628 | No Hit |
| TTCACCGTGCCAGACTAGAGTCAAGCTCAACAGGGTCTTCTTTCCCCGCTGATTCCGCCAAGCCCGTTCCCTTG | 366944 | 0.21056920682318278 | No Hit |
| TGAATTTAAGCATATTAGTCAGCGGAGGAGAAGAAACTAACCAGGATTCCCTCAGTAACGGCGAGTGAACAGGG | 348503 | 0.1999869197629602 | No Hit |
| GGTATTTAGCCTTAGATGGAGTTTACCACCCGCTTTGGGCTGCATTCCCAAGCAACCCGACTCCGGGAAGACCC | 267908 | 0.15373783209859068 | No Hit |
| TTTAGCCTTAGATGGAGTTTACCACCCGCTTTGGGCTGCATTCCCAAGCAACCCGACTCCGGGAAGACCCGGGC | 260403 | 0.14943112072789655 | No Hit |
| TTTGGGCTGCATTCCCAAGCAACCCGACTCCGGGAAGACCCGGGCCCGGCGCGCCGGGGGCCGCTACCGGCCTC | 256852 | 0.14739339493478062 | No Hit |
| TCTCTTCAAAGTTCTTTTCAACTTTCCCTTACGGTACTTGTTGACTATCGGTCTCGTGCCGGTATTTAGCCTTA | 249035 | 0.14290764373095438 | No Hit |
| TTAGATGGAGTTTACCACCCGCTTTGGGCTGCATTCCCAAGCAACCCGACTCCGGGAAGACCCGGGCCCGGCGC | 243336 | 0.13963729754819812 | No Hit |
| TAGAGTCAAGCTCAACAGGGTCTTCTTTCCCCGCTGATTCCGCCAAGCCCGTTCCCTTGGCTGTGGTTTCGCTG | 229119 | 0.13147893438268732 | No Hit |
| GAGATTCCCACTGTCCCTACCTACTATCCAGCGAAACCACAGCCAAGGGAACGGGCTTGGCGGAATCAGCGGGG | 225213 | 0.12923749339482174 | No Hit |
| TTTTCTGACACCTCCTGCTTAAAACCCAAAAGGTCAGAAGGATCGTGAGGCCCCGCTTTCACGGTCTGTATTCG | 218753 | 0.12553045069599642 | No Hit |
| TCGACTGCCGGCGACGGCCGGGTATGGGCCCGACGCTCCAGCGCCATCCATTTTCAGGGCTAGTTGATTCGGCA | 218666 | 0.12548052612714225 | No Hit |
| TTGGCTGTGGTTTCGCTGGATAGTAGGTAGGGACAGTGGGAATCTCGTTCATCCATTCATGCGCGTCACTAATT | 215567 | 0.12370217855382033 | No Hit |
| TAGAATTACCACAGTTATCCAAGTAGGAGAGGAGCGAGCGACCAAAGGAACCATAACTGATTTAATGAGCCATT | 215224 | 0.1235053495064988 | No Hit |
| TTAGAGCCAATCCTTATCCCGAAGTTACGGATCCGGCTTGCCGACTTCCCTTACCTACATTGTTCCAACATGCC | 204433 | 0.11731298143172726 | No Hit |
| TCGCATTCCACGCCCGGCTCCACGCCAGCGAGCCGGGCTTCTTACCCATTTAAAGTTTGAGAATAGGTTGAGAT | 196658 | 0.11285133174389957 | No Hit |
| ATGTATTAGCTCTAGAATTACCACAGTTATCCAAGTAGGAGAGGAGCGAGCGACCAAAGGAACCATAACTGATT | 193297 | 0.11092263661839617 | No Hit |
| TTTAAATGGGTAAGAAGCCCGGCTCGCTGGCGTGGAGCCGGGCGTGGAATGCGAGTGCCTAGTGGGCCACTTTT | 192905 | 0.11069768913574299 | No Hit |

## Adapter Content

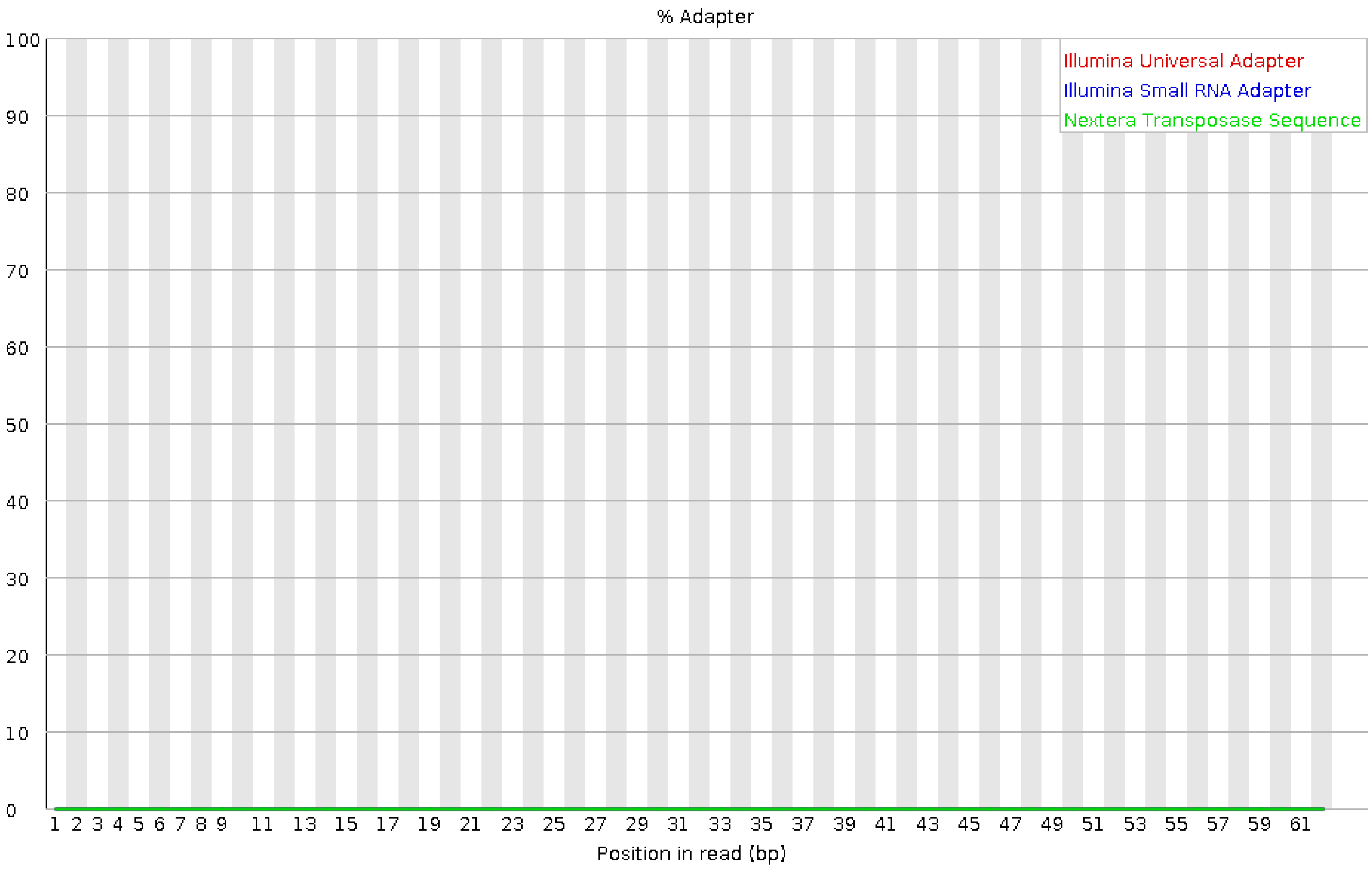


## Kmer Content

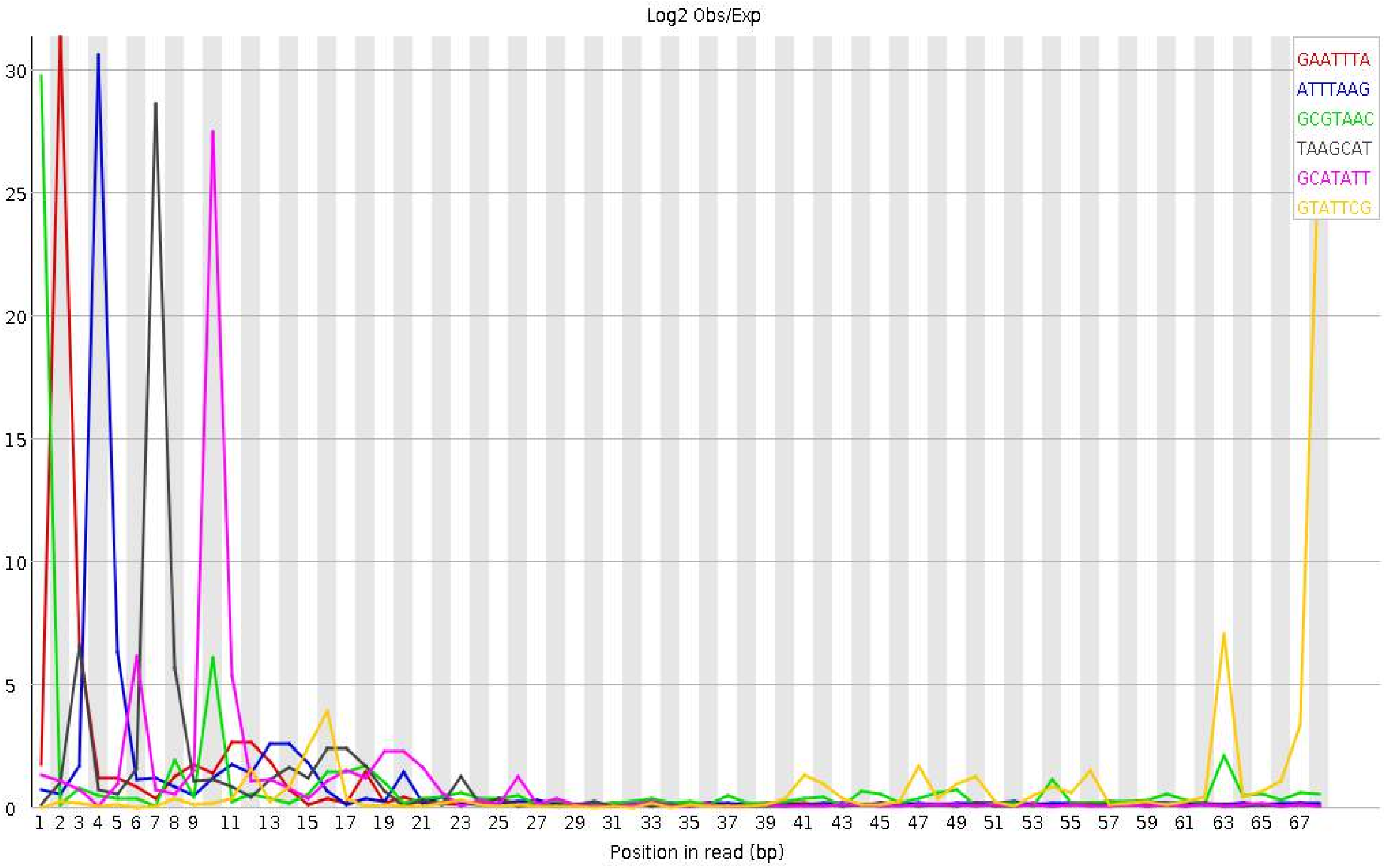

| Sequence | Count | PValue | Obs/Exp Max | Max Obs/Exp Position |
|---|---|---|---|---|
| GAATTTA | 245195 | 0.0 | 31.315096 | 2 |
| ATTTAAG | 250545 | 0.0 | 30.651836 | 4 |
| GCGTAAC | 43255 | 0.0 | 29.809504 | 1 |
| TAAGCAT | 254390 | 0.0 | 28.6546 | 7 |
| GCATATT | 265640 | 0.0 | 27.484364 | 10 |
| GTATTCG | 63755 | 0.0 | 27.481256 | 68 |
| CAGCGGA | 275185 | 0.0 | 27.454742 | 20 |
| TAGTCAG | 276935 | 0.0 | 27.278765 | 16 |
| ATATTAG | 269690 | 0.0 | 27.211527 | 12 |
| CATATTA | 268270 | 0.0 | 27.17944 | 11 |
| AGCGGAG | 278510 | 0.0 | 27.170914 | 21 |
| TTGTCTC | 155625 | 0.0 | 27.154072 | 1 |
| TTAGTCA | 280550 | 0.0 | 26.991615 | 15 |
| AAGCATA | 275290 | 0.0 | 26.45076 | 8 |
| AGTCAGC | 287340 | 0.0 | 26.261385 | 17 |
| TCGCCTA | 18045 | 0.0 | 26.094501 | 65 |
| AGCATAT | 282045 | 0.0 | 26.059294 | 9 |
| TATCGCC | 18370 | 0.0 | 25.781353 | 63 |
| CTATCGC | 18570 | 0.0 | 25.690569 | 62 |
| CGCCTAT | 18460 | 0.0 | 25.451992 | 66 |

# FastQC Report

Mon 4 May 2015
Agilent_Fetal_Colon_CGATGT_L008_R2.fastq.gz

## Summary


- Basic Statistics
- Per base sequence quality
- Per tile sequence quality
- Per sequence quality scores
- Per base sequence content
- Per sequence GC content
- Per base N content
- Sequence Length Distribution
- Sequence Duplication Levels
- Overrepresented sequences
- Adapter Content
- Kmer Content


## Basic Statistics

| Measure | Value |
|---|---|
| Filename | Agilent_Fetal_Colon_CGATGT_L008_R2.fastq.gz |
| File type | Conventional base calls |
| Encoding | Sanger / Illumina 1.9 |
| Total Sequences | 174262897 |
| Sequences flagged as poor quality | 0 |
| Sequence length | 74 |
| %GC | 52 |

## Per base sequence quality

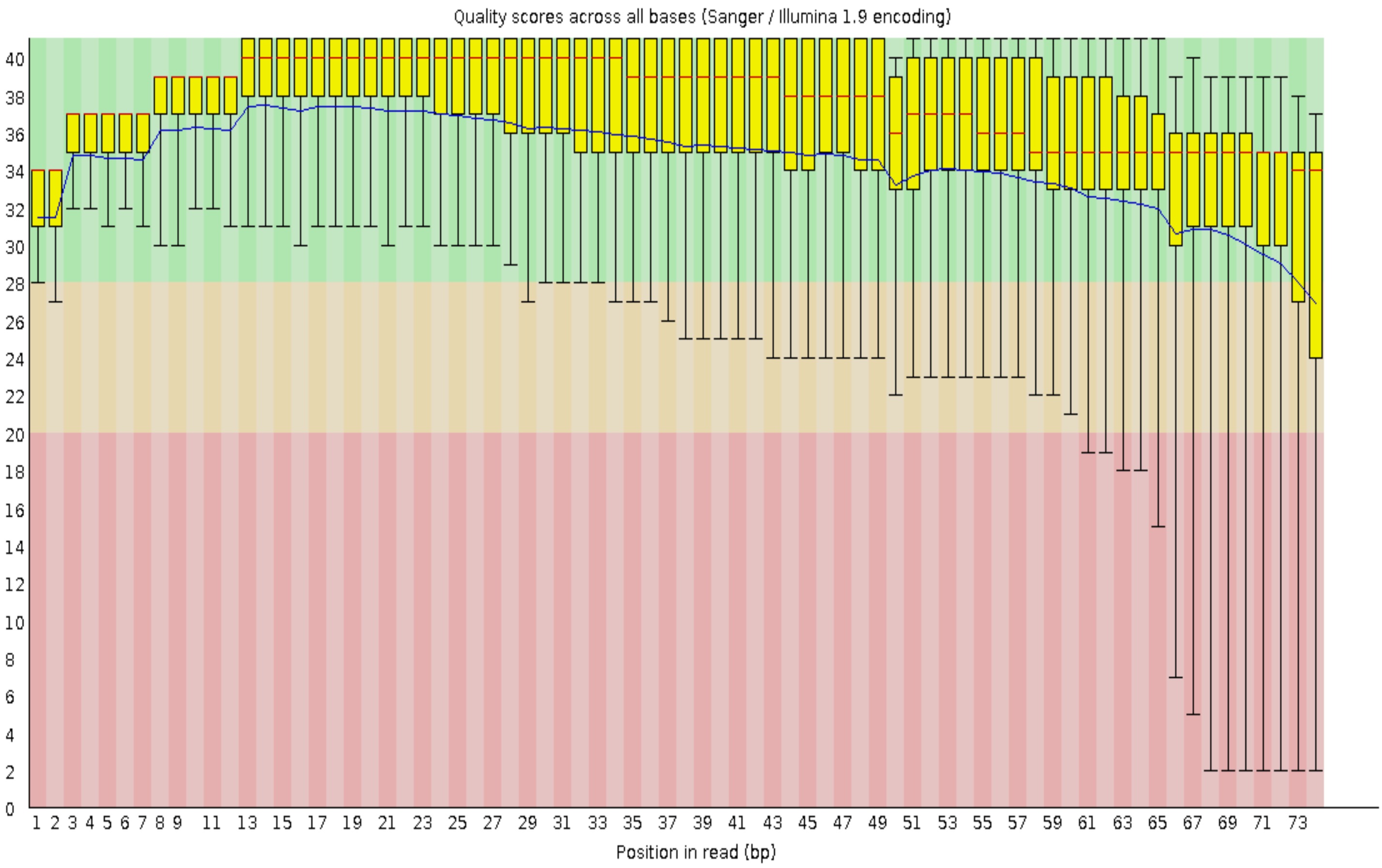


## Per tile sequence quality

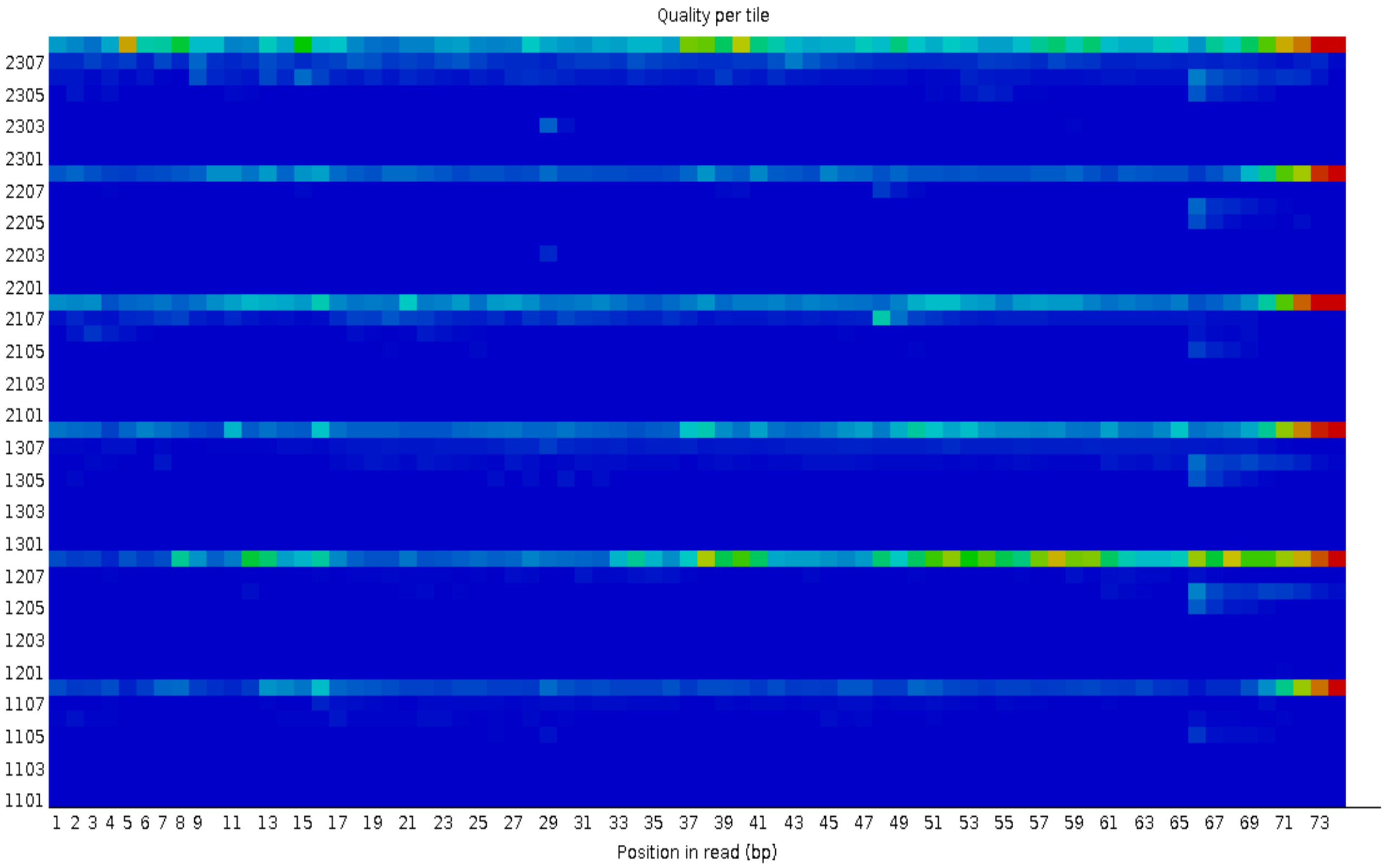

## Per sequence quality scores

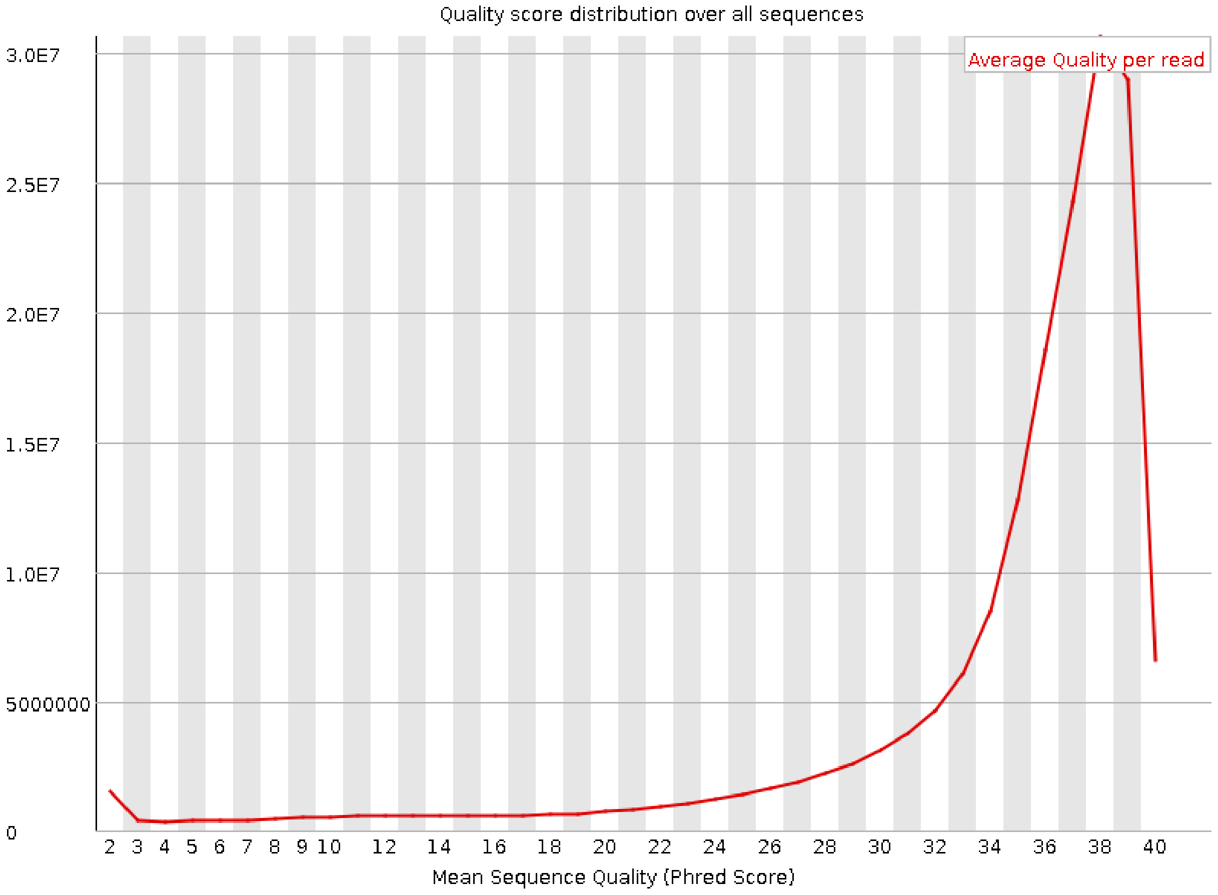


## Per base sequence content

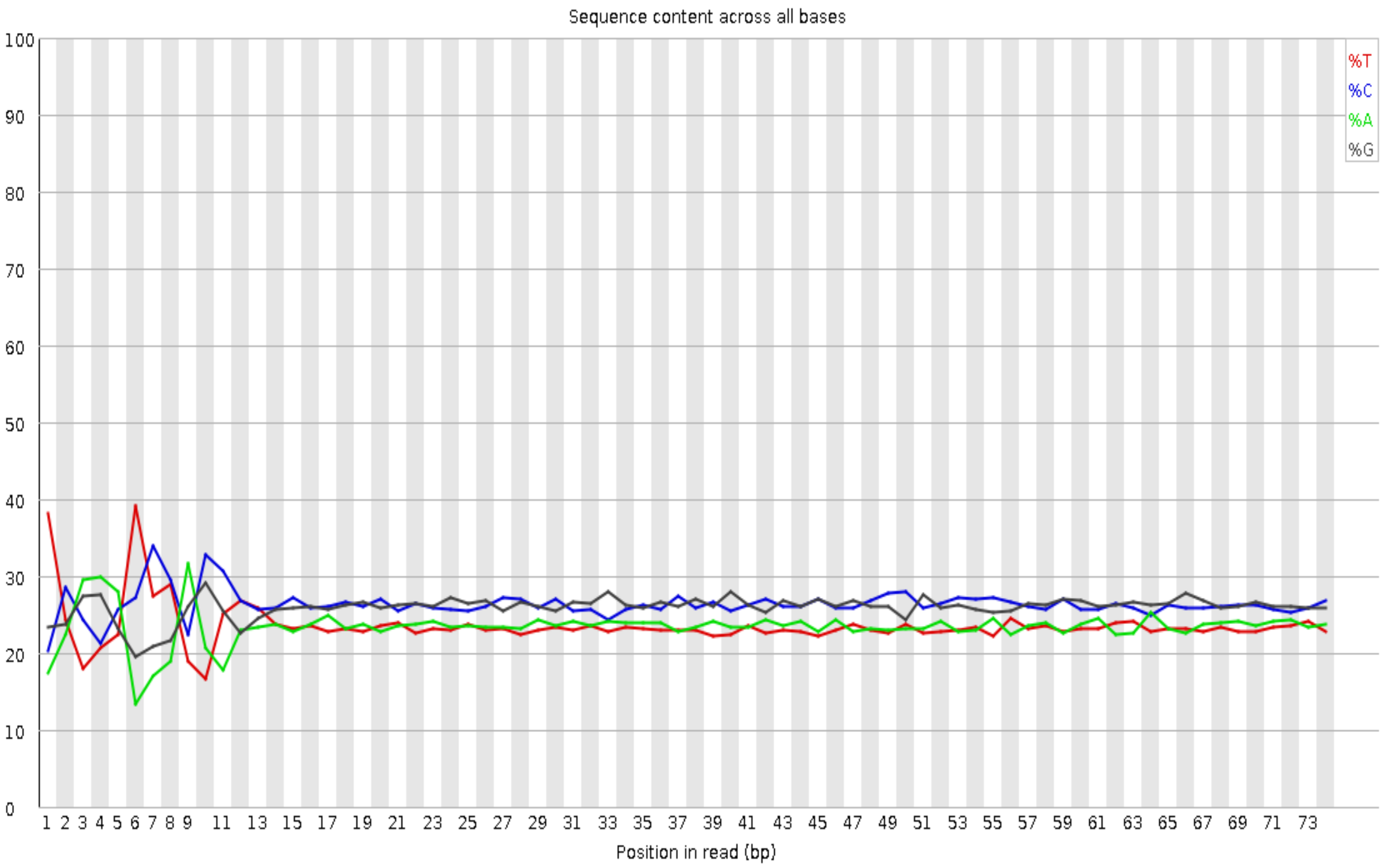


## Per sequence GC content

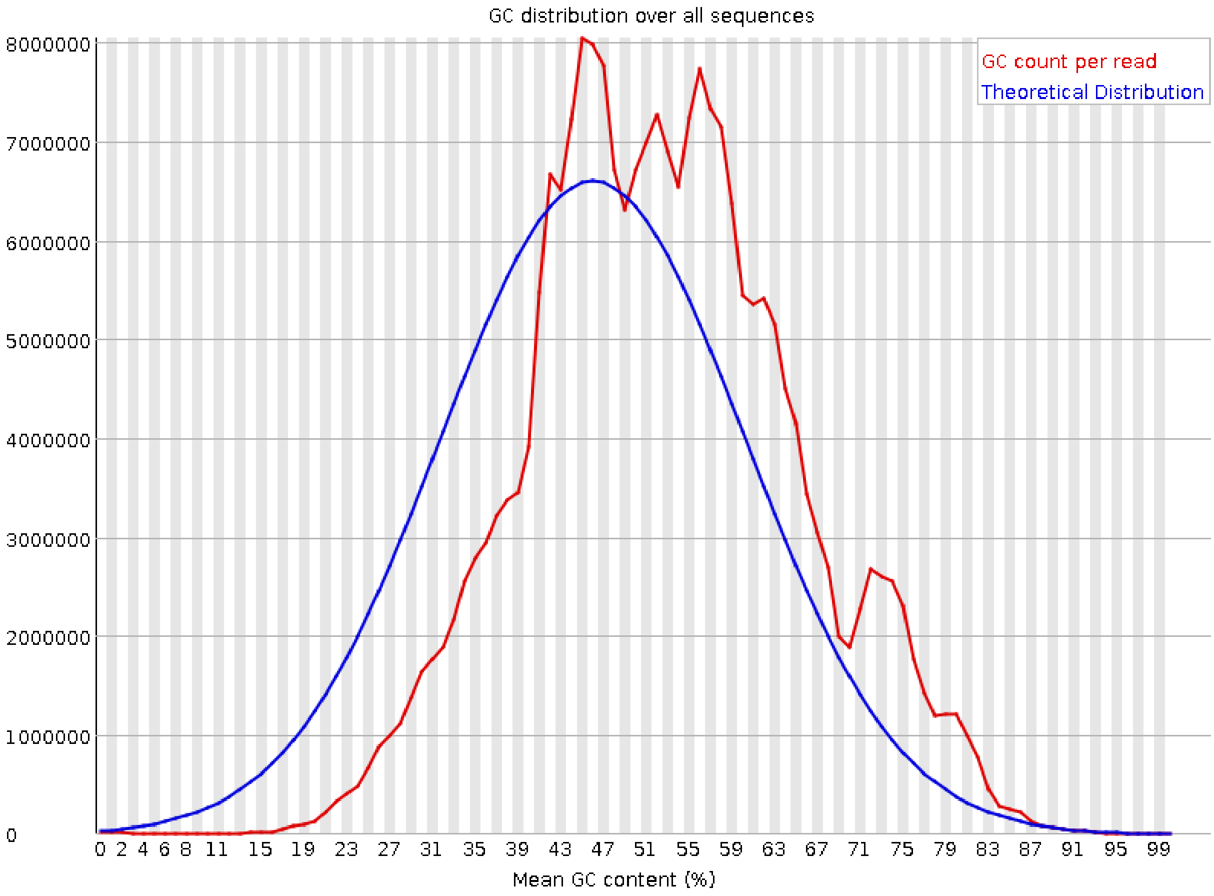

## Per base N content

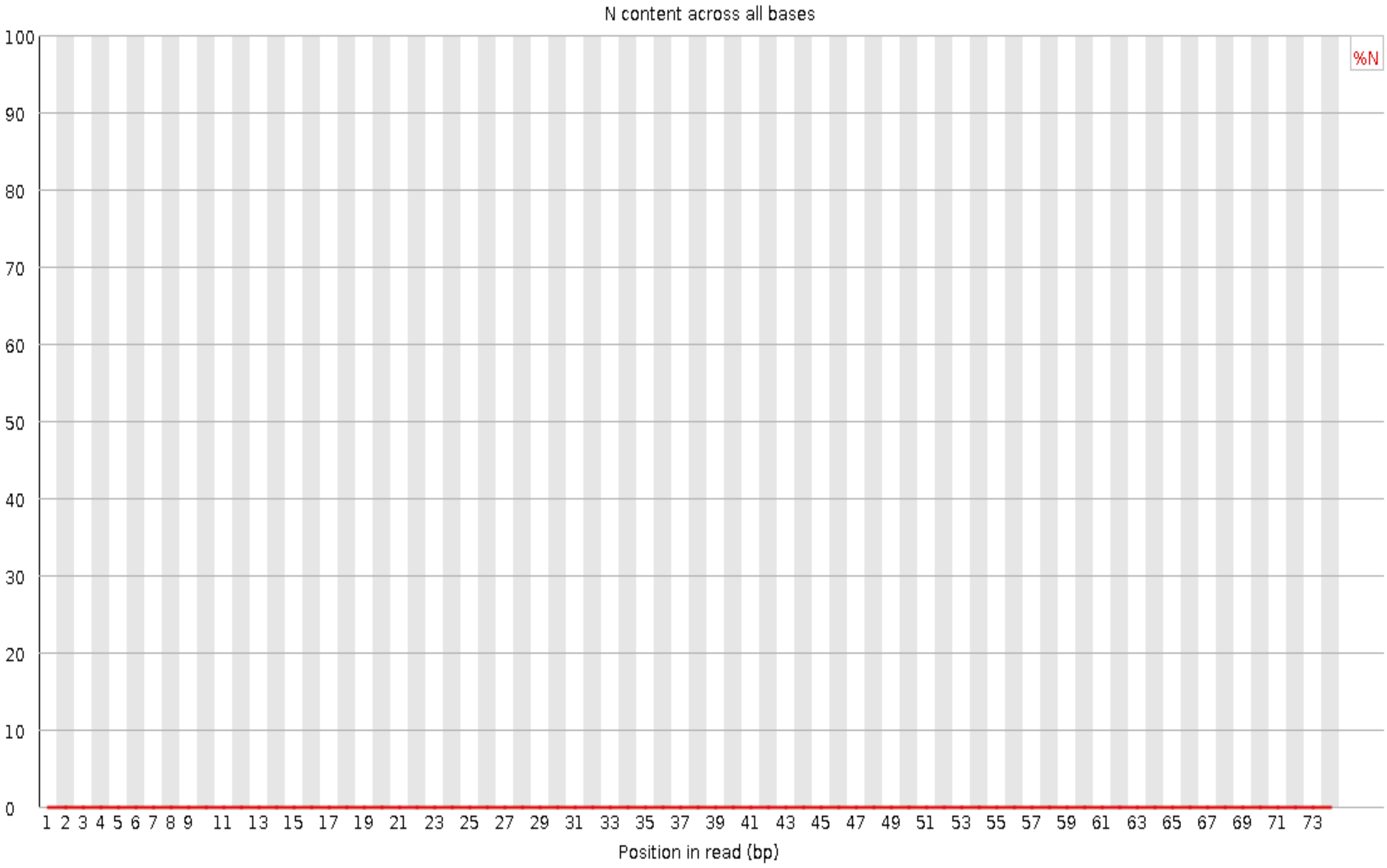


## Sequence Length Distribution

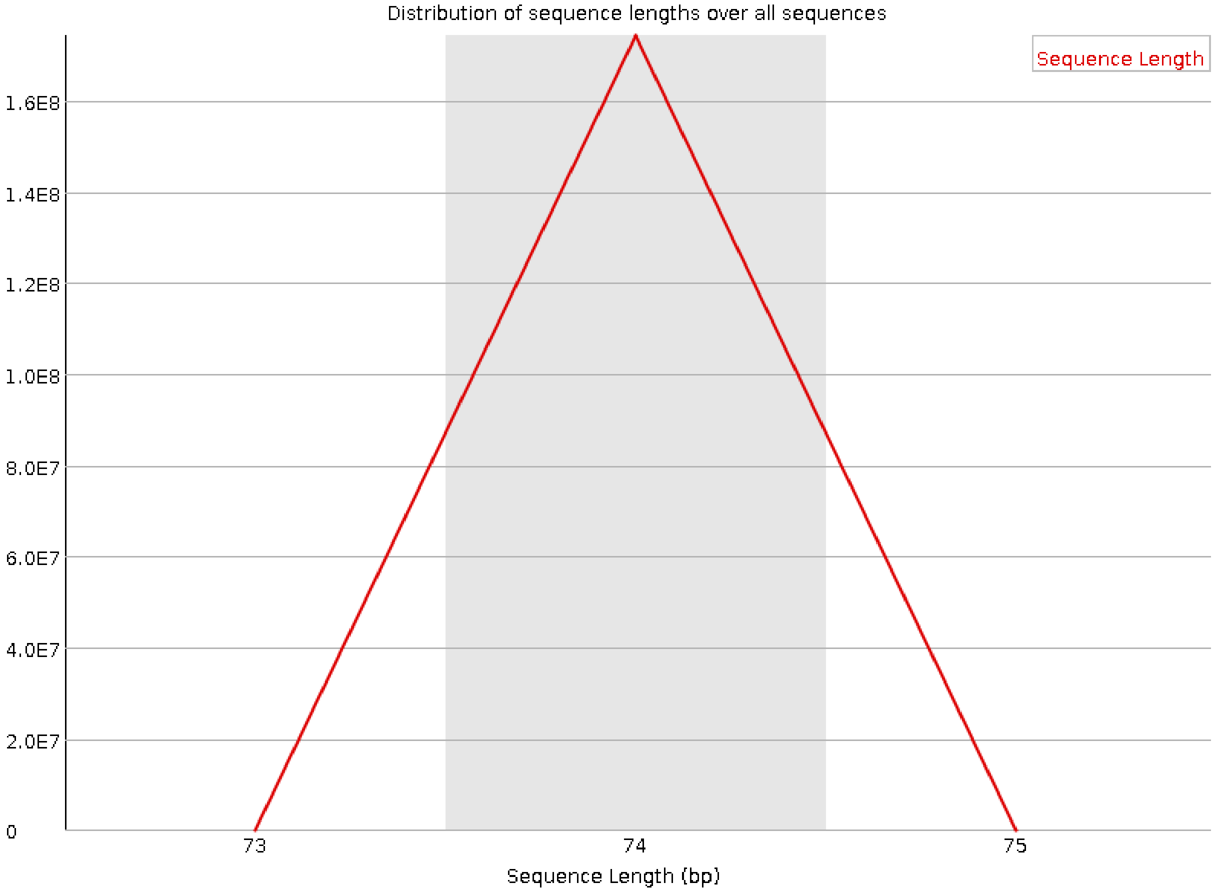


## Sequence Duplication Levels

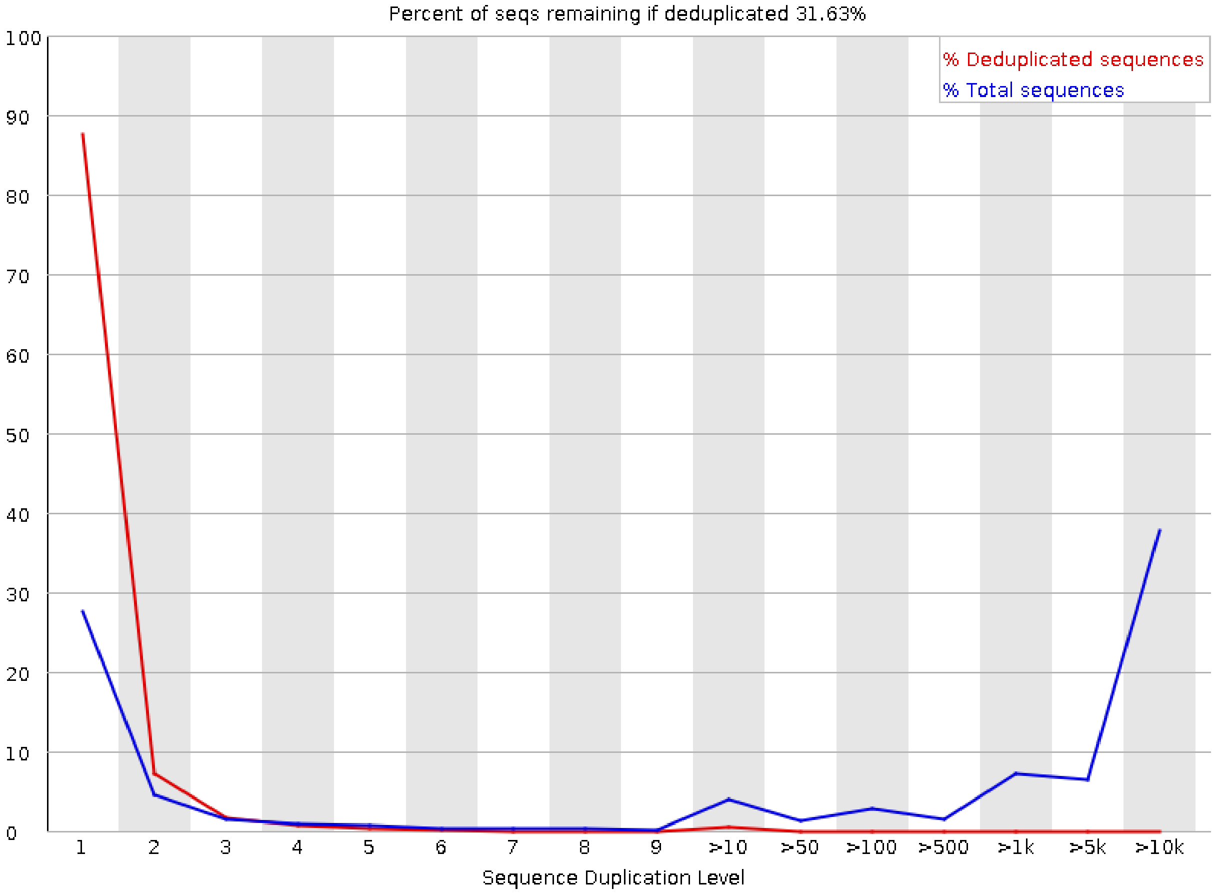

## Overrepresented sequences

| Sequence | Count | Percentage | Possible Source |
|---|---|---|---|
| TTGAACTCTCTCTTCAAAGTTCTTTTCAACTTTCCCTTACGGTACTTGTTGACTATCGGTCTCGTGCCGGTATT | 516106 | 0.296165167046431 | No Hit |
| TTGTCTCAAAGATTAAGCCATGCATGTCTAAGTACGCACGGCCGGTACAGTGAAACTGCGAATGGCTCATTAAA | 513159 | 0.2944740440071991 | No Hit |
| CGGTATTTAGCCTTAGATGGAGTTTACCACCCGCTTTGGGCTGCATTCCCAAGCAACCCGACTCCGGGAAGACC | 452514 | 0.25967317644214305 | No Hit |
| TGAATTTAAGCATATTAGTCAGCGGAGGAAAAGAAACTAACCAGGATTCCCTCAGTAACGGCGAGTGAACAGGG | 445283 | 0.2555236987710585 | No Hit |
| TTCCGTACGCCACATGTCCCGCGCCCCGCCGCGGGGCGGGATTCGGCGCTGGGCTCTTCCCTGTTCACTCGCC | 428239 | 0.2457430740405974 | No Hit |
| AAAGATTAAGCCATGCATGTCTAAGTACGCACGGCCGGTACAGTGAAACTGCGAATGGCTCATTAAATCAGTTA | 427274 | 0.24518931301824964 | No Hit |
| TGGATAGTAGGTAGGGACAGTGGGAATCTCGTTCATCCATTCATGCGCGTCACTAATTAGATGACGAGGCATTT | 421025 | 0.24160335174503614 | No Hit |
| TTCACCGTGCCAGACTAGAGTCAAGCTCAACAGGGTCTTCTTTCCCCGCTGATTCCGCCAAGCCCGTTCCCTTG | 385314 | 0.22111075084445542 | No Hit |
| TGAATTTAAGCATATTAGTCAGCGGAGGAGAAGAAACTAACCAGGATTCCCTCAGTAACGGCGAGTGAACAGGG | 304115 | 0.1745150604262019 | No Hit |
| GGTATTTAGCCTTAGATGGAGTTTACCACCCGCTTTGGGCTGCATTCCCAAGCAACCCGACTCCGGGAAGACCC | 259496 | 0.14891064275145155 | No Hit |
| TTTGGGCTGCATTCCCAAGCAACCCGACTCCGGGAAGACCCGGGCCCGGCGCGCCGGGGGCCGCTACCGGCCTC | 253802 | 0.14564316579679035 | No Hit |
| TCTCTTCAAAGTTCTTTTCAACTTTCCCTTACGGTACTTGTTGACTATCGGTCTCGTGCCGGTATTTAGCCTTA | 233355 | 0.13390974442482728 | No Hit |
| TAGAGTCAAGCTCAACAGGGTCTTCTTTCCCCGCTGATTCCGCCAAGCCCGTTCCCTTGGCTGTGGTTTCGCTG | 226370 | 0.12990143277602 | No Hit |
| TTTAGCCTTAGATGGAGTTTACCACCCGCTTTGGGCTGCATTCCCAAGCAACCCGACTCCGGGAAGACCCGGGC | 224655 | 0.12891728753941237 | No Hit |
| TTAGATGGAGTTTACCACCCGCTTTGGGCTGCATTCCCAAGCAACCCGACTCCGGGAAGACCCGGGCCCGGCGC | 221498 | 0.12710565692018766 | No Hit |
| TCGACTGCCGGCGACGGCCGGGTATGGGCCCGACGCTCCAGCGCCATCCATTTTCAGGGCTAGTTGATTCGGCA | 217655 | 0.12490036820631989 | No Hit |
| TTGGCTGTGGTTTCGCTGGATAGTAGGTAGGGACAGTGGGAATCTCGTTCATCCATTCATGCGCGTCACTAATT | 215139 | 0.12345657262888268 | No Hit |
| GAGATTCCCACTGTCCCTACCTACTATCCAGCGAAACCACAGCCAAGGGAACGGGCTTGGCGGAATCAGCGGGG | 212438 | 0.121906615611928 | No Hit |
| TTTTCTGACACCTCCTGCTTAAAACCCAAAAGGTCAGAAGGATCGTGAGGCCCCGCTTTCACGGTCTGTATTCG | 210231 | 0.12064013833076584 | No Hit |
| GGGTCTTCCGTACGCCACATGTCCCGCGCCCCGCCGCGGGGCGGGATTCGGCGCTGGGCTCTTCCCTGTTCAC | 202114 | 0.11598223344123564 | No Hit |
| TAGAATTACCACAGTTATCCAAGTAGGAGAGGAGCGAGCGACCAAAGGAACCATAACTGATTTAATGAGCCATT | 194101 | 0.11138400849608279 | No Hit |
| TCGCATTCCACGCCCGGCTCCACGCCAGCGAGCCGGGCTTCTTACCCATTTAAAGTTTGAGAATAGGTTGAGAT | 191700 | 0.11000620516483207 | No Hit |
| ATGTATTAGCTCTAGAATTACCACAGTTATCCAAGTAGGAGAGGAGCGAGCGACCAAAGGAACCATAACTGATT | 181222 | 0.10399345076881168 | No Hit |
| TTAGAGCCAATCCTTATCCCGAAGTTACGGATCCGGCTTGCCGACTTCCCTTACCTACATTGTTCCAACATGCC | 174529 | 0.10015270204075627 | No Hit |

## Adapter Content

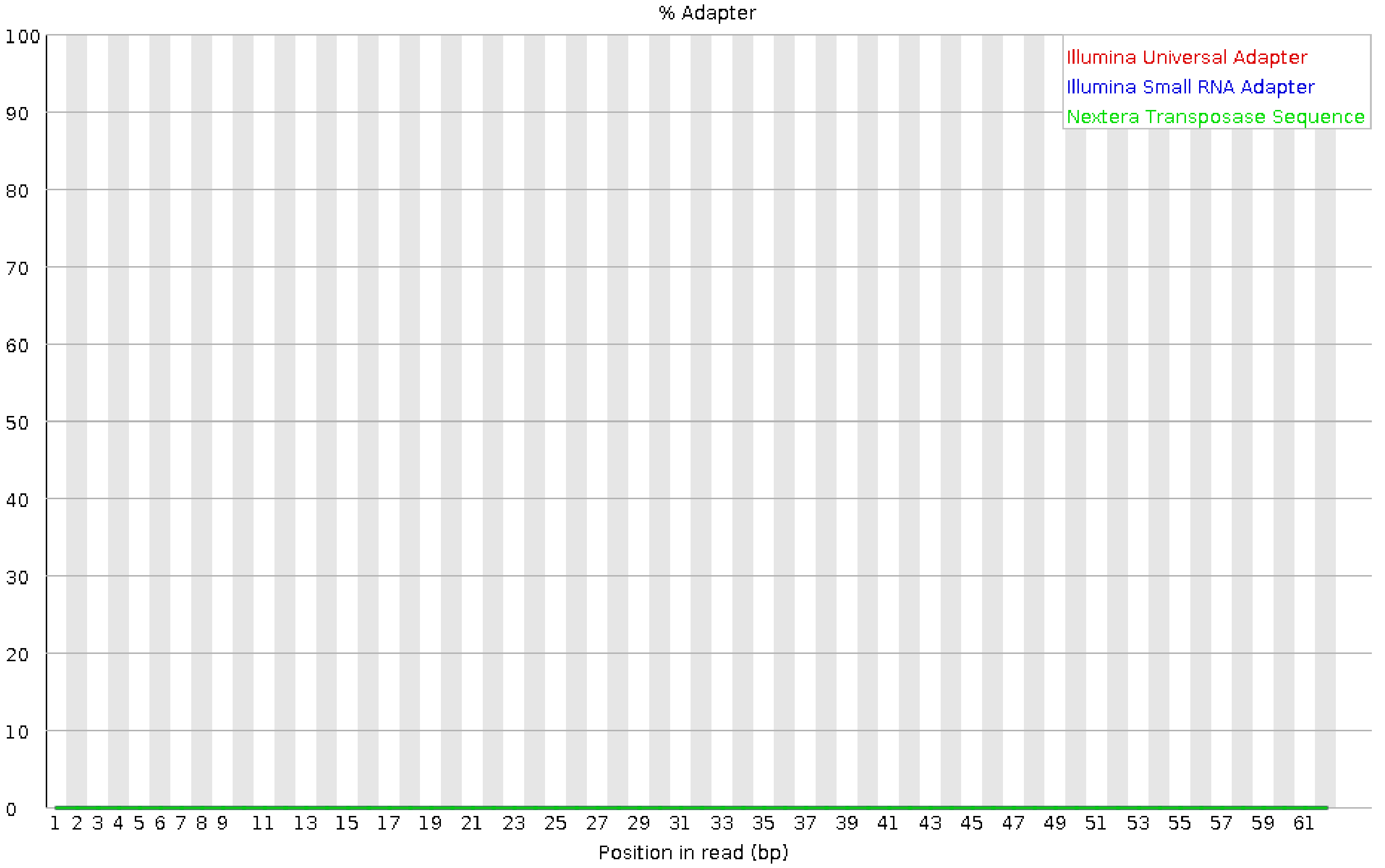


## Kmer Content

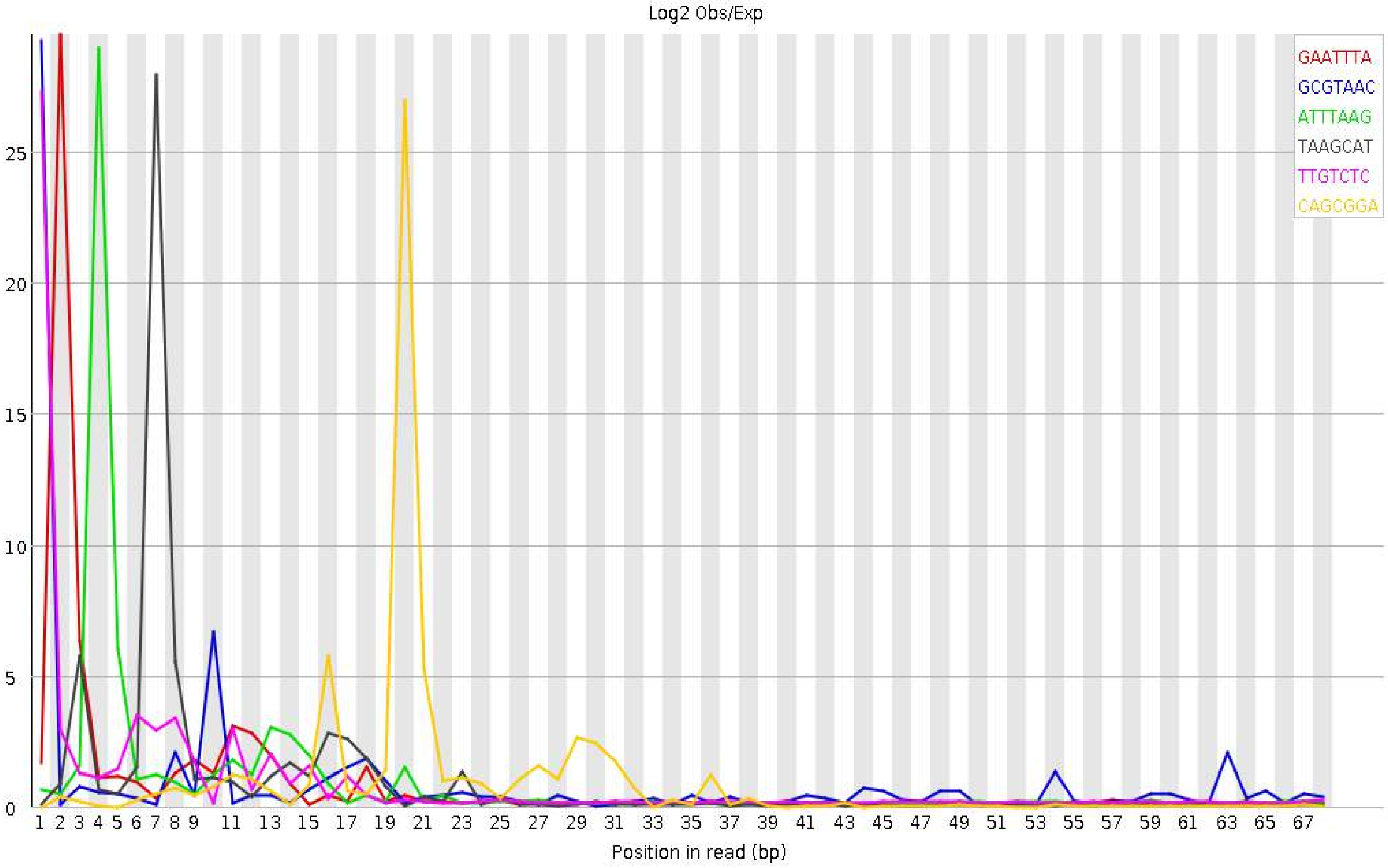

| Sequence | Count | PValue | Obs/Exp Max | Max Obs/Exp Position |
|---|---|---|---|---|
| GAATTTA | 228470 | 0.0 | 29.413599 | 2 |
| GCGTAAC | 42715 | 0.0 | 29.214697 | 1 |
| ATTTAAG | 233320 | 0.0 | 28.942663 | 4 |
| TAAGCAT | 232140 | 0.0 | 27.891031 | 7 |
| TTGTCTC | 155145 | 0.0 | 27.26455 | 1 |
| CAGCGGA | 250145 | 0.0 | 26.928152 | 20 |
| GCATATT | 241910 | 0.0 | 26.735518 | 10 |
| TAGTCAG | 252000 | 0.0 | 26.632952 | 16 |
| AGCGGAG | 254600 | 0.0 | 26.52777 | 21 |
| CATATTA | 245290 | 0.0 | 26.318695 | 11 |
| ATATTAG | 245000 | 0.0 | 26.312315 | 12 |
| GTATTCG | 60245 | 0.0 | 26.258482 | 68 |
| TTAGTCA | 255320 | 0.0 | 26.24538 | 15 |
| AAGCATA | 252500 | 0.0 | 25.694584 | 8 |
| AGTCAGC | 262375 | 0.0 | 25.600449 | 17 |
| TCGCCTA | 16360 | 0.0 | 25.589363 | 65 |
| CTATCGC | 16480 | 0.0 | 25.42369 | 62 |
| CTCAAAG | 167615 | 0.0 | 25.291342 | 5 |
| AGCATAT | 259000 | 0.0 | 25.201797 | 9 |
| GTCTCAA | 168155 | 0.0 | 25.162094 | 3 |

# FastQC Report

Mon 4 May 2015
Agilent_Fetal_Stomach_GCCAAT_L002_R1.fastq.gz

## Summary


- Basic Statistics
- Per base sequence quality
- Per tile sequence quality
- Per sequence quality scores
- Per base sequence content
- Per sequence GC content
- Per base N content
- Sequence Length Distribution
- Sequence Duplication Levels
- Overrepresented sequences
- Adapter Content
- Kmer Content


## Basic Statistics

| Measure | Value |
|---|---|
| Filename | Agilent_Fetal_Stomach_GCCAAT_L002_R1.fastq.gz |
| File type | Conventional base calls |
| Encoding | Sanger / Illumina 1.9 |
| Total Sequences | 174661455 |
| Sequences flagged as poor quality | 0 |
| Sequence length | 74 |
| %GC | 57 |

## Per base sequence quality

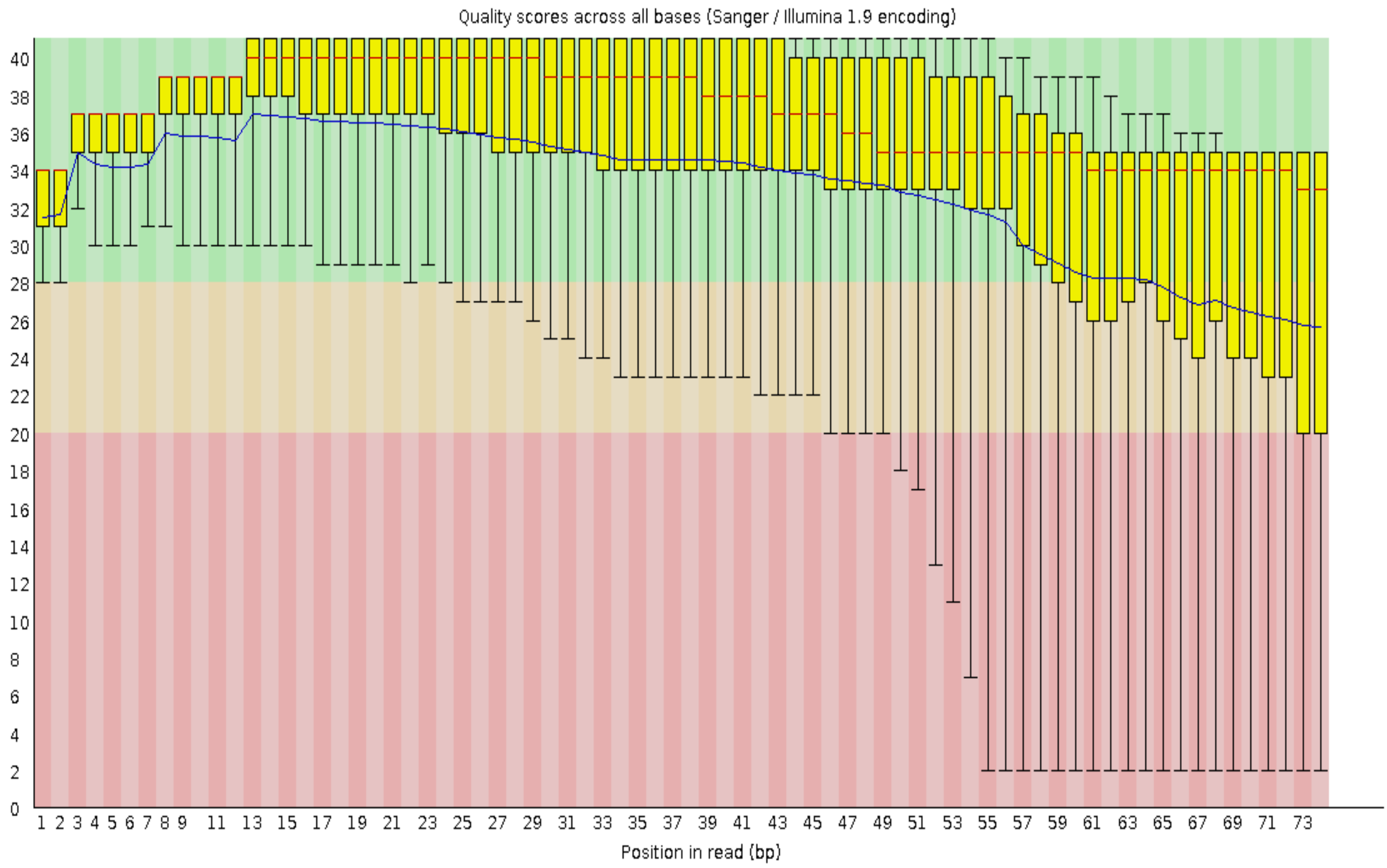


## Per tile sequence quality

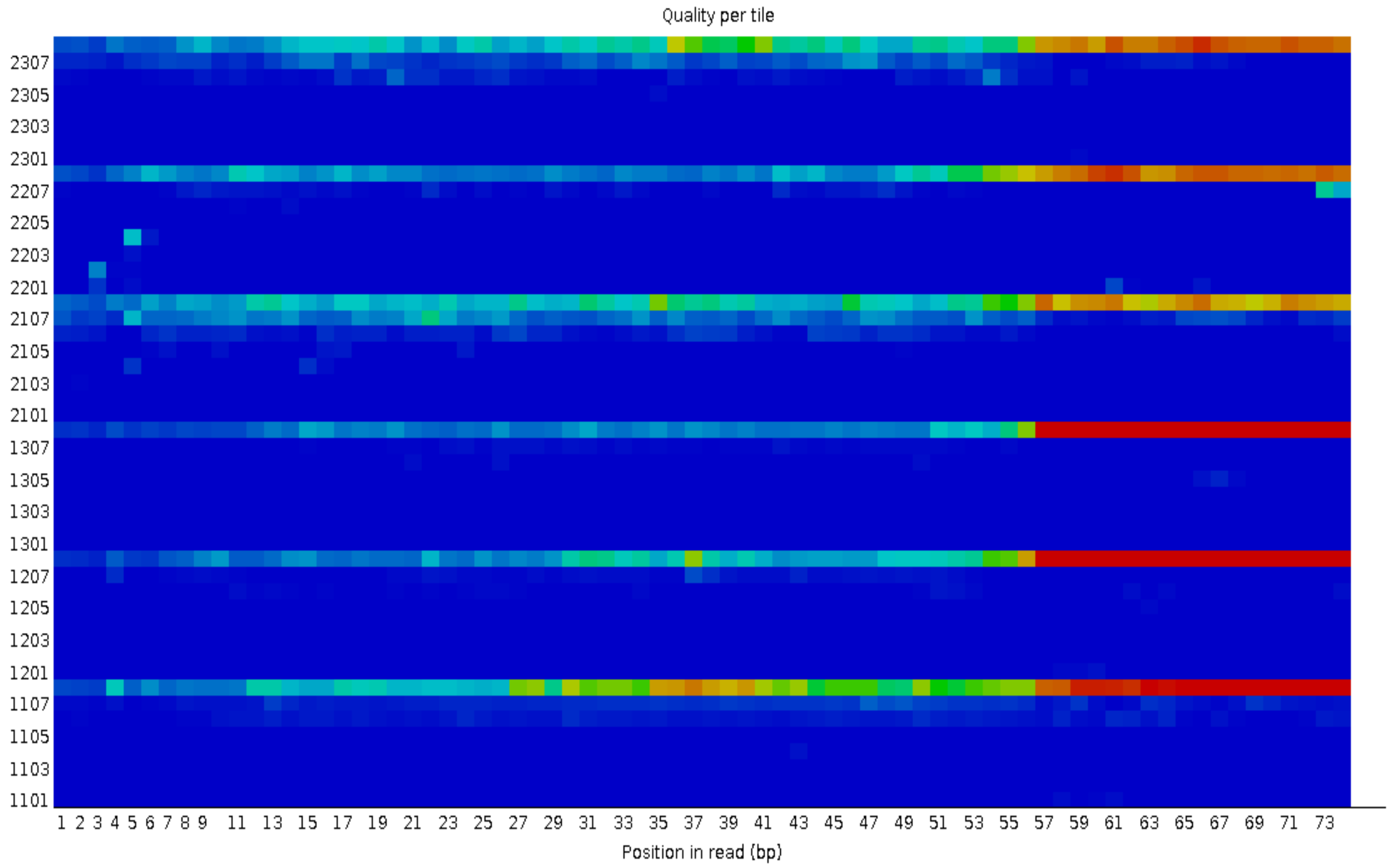

## Per sequence quality scores

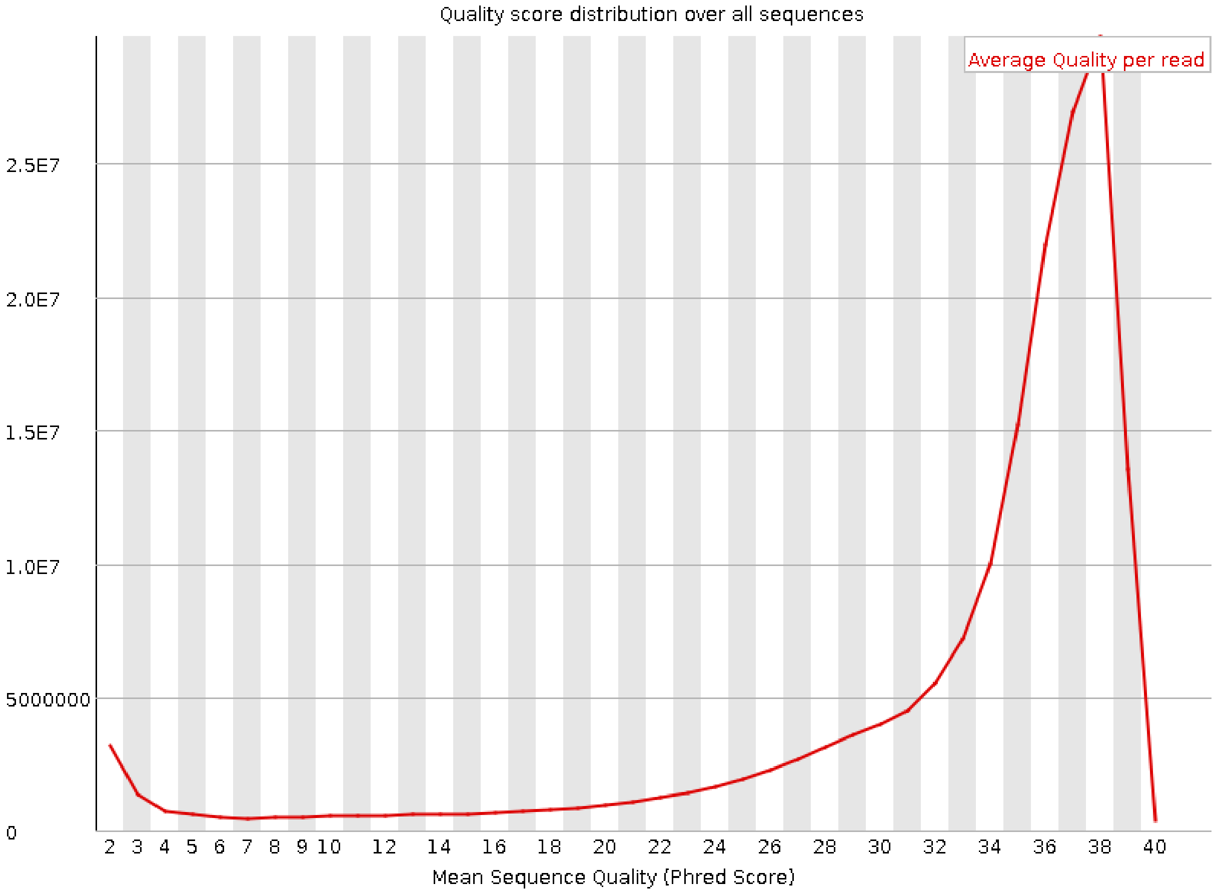


## Per base sequence content

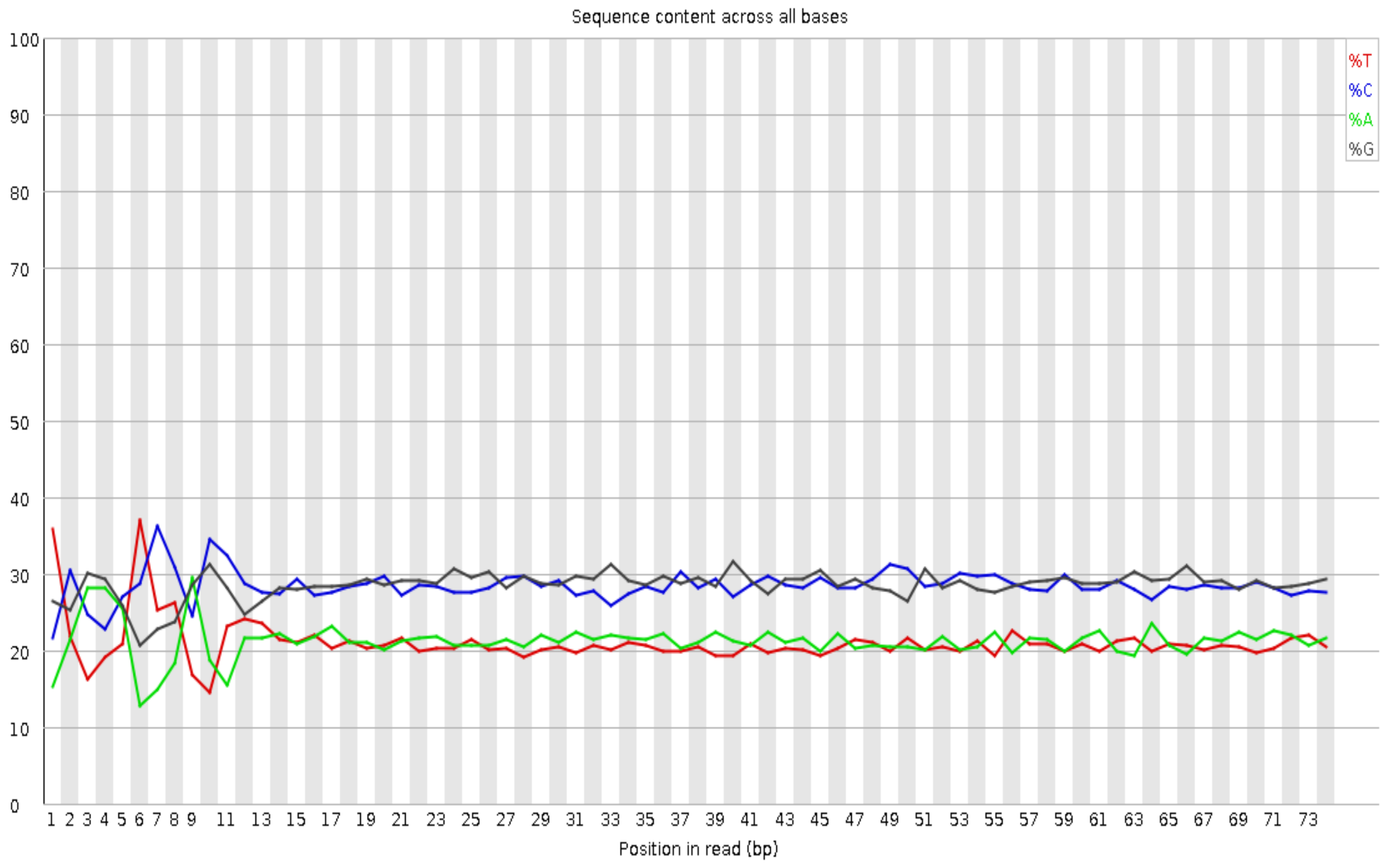


## Per sequence GC content

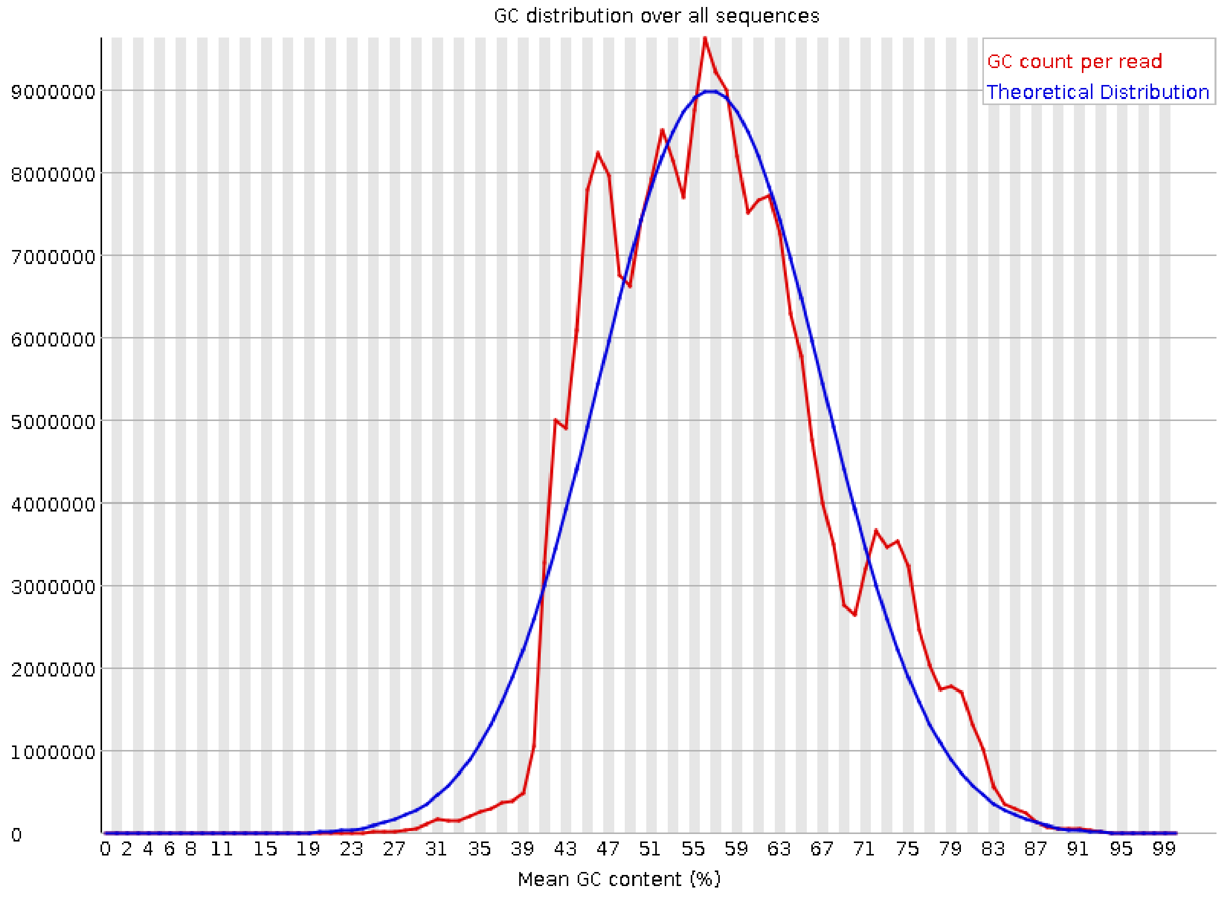

## Per base N content

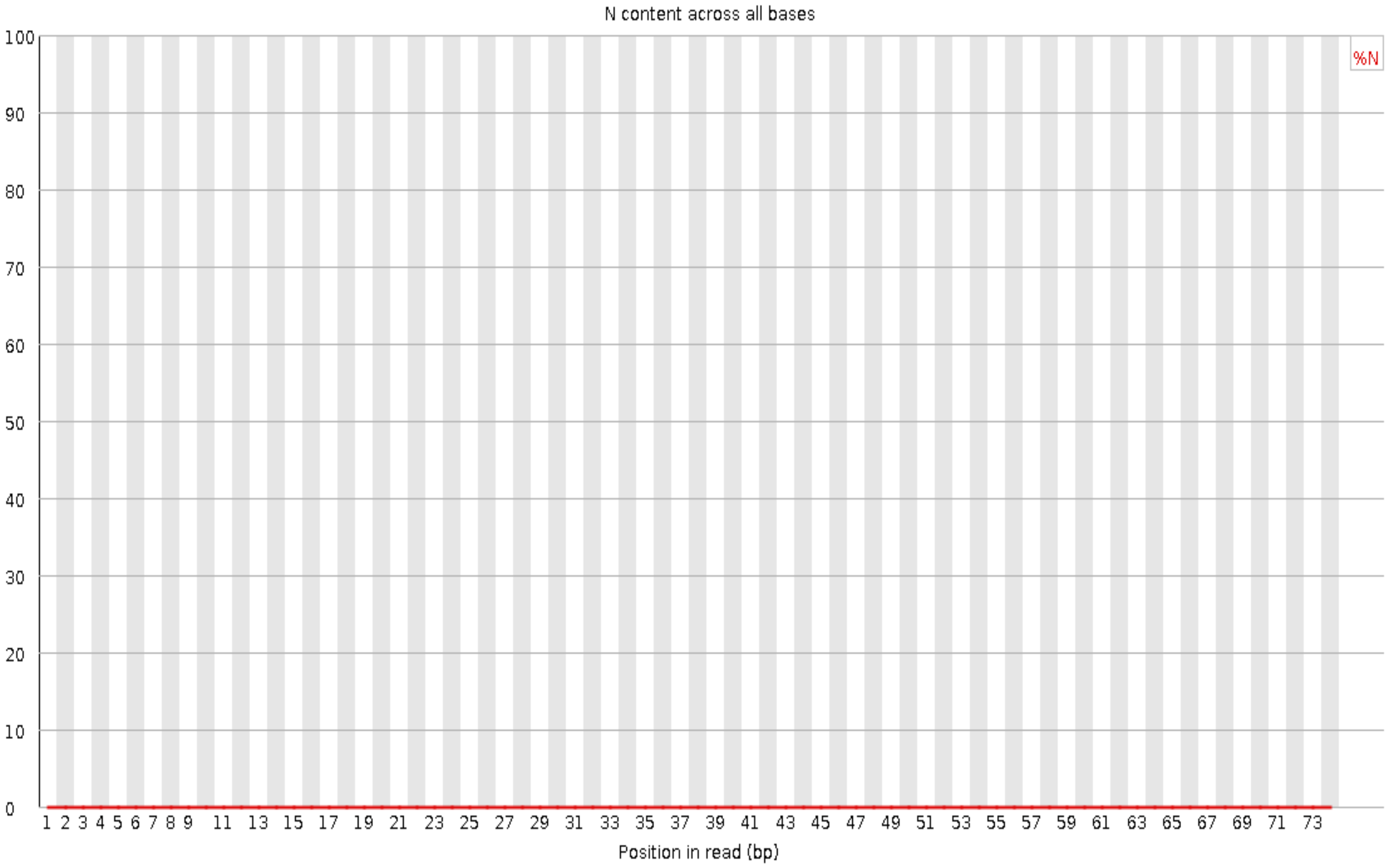


## Sequence Length Distribution

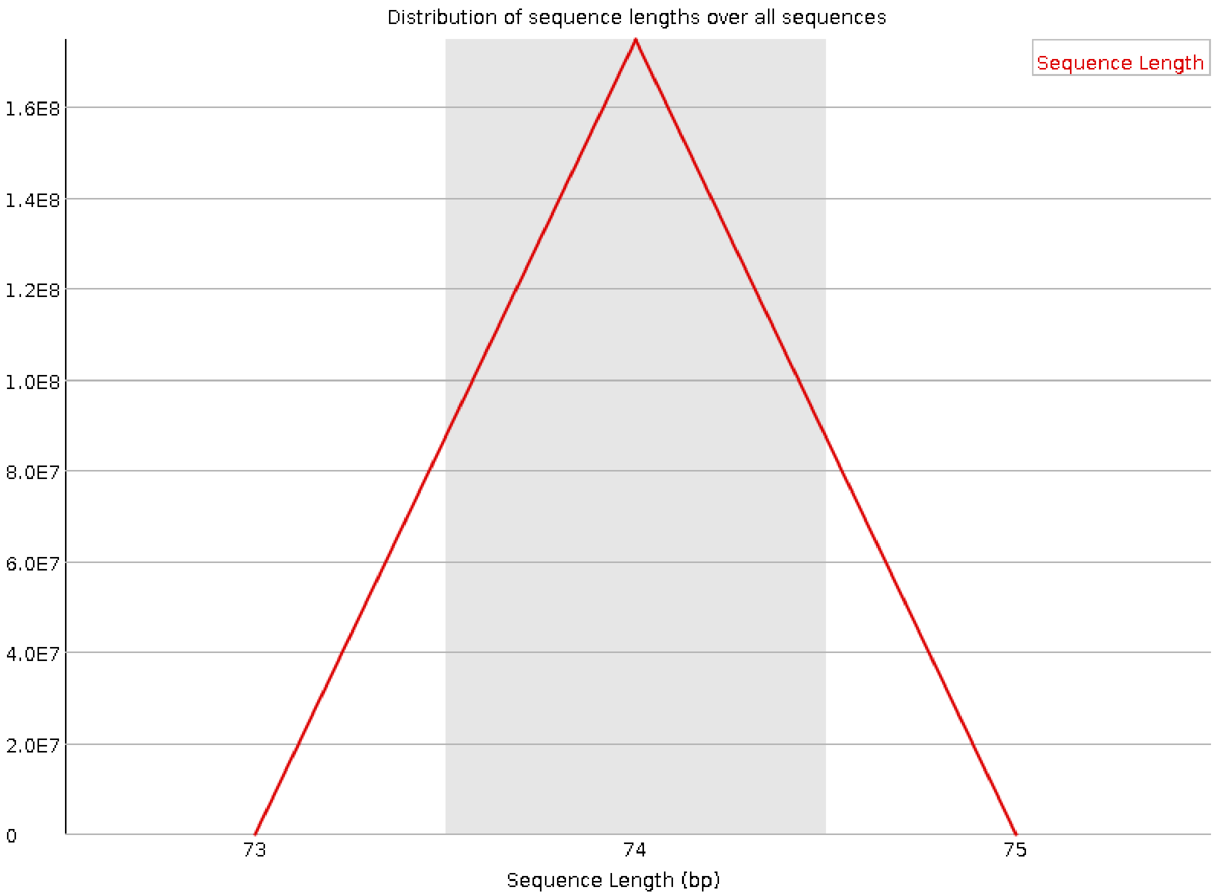


## Sequence Duplication Levels

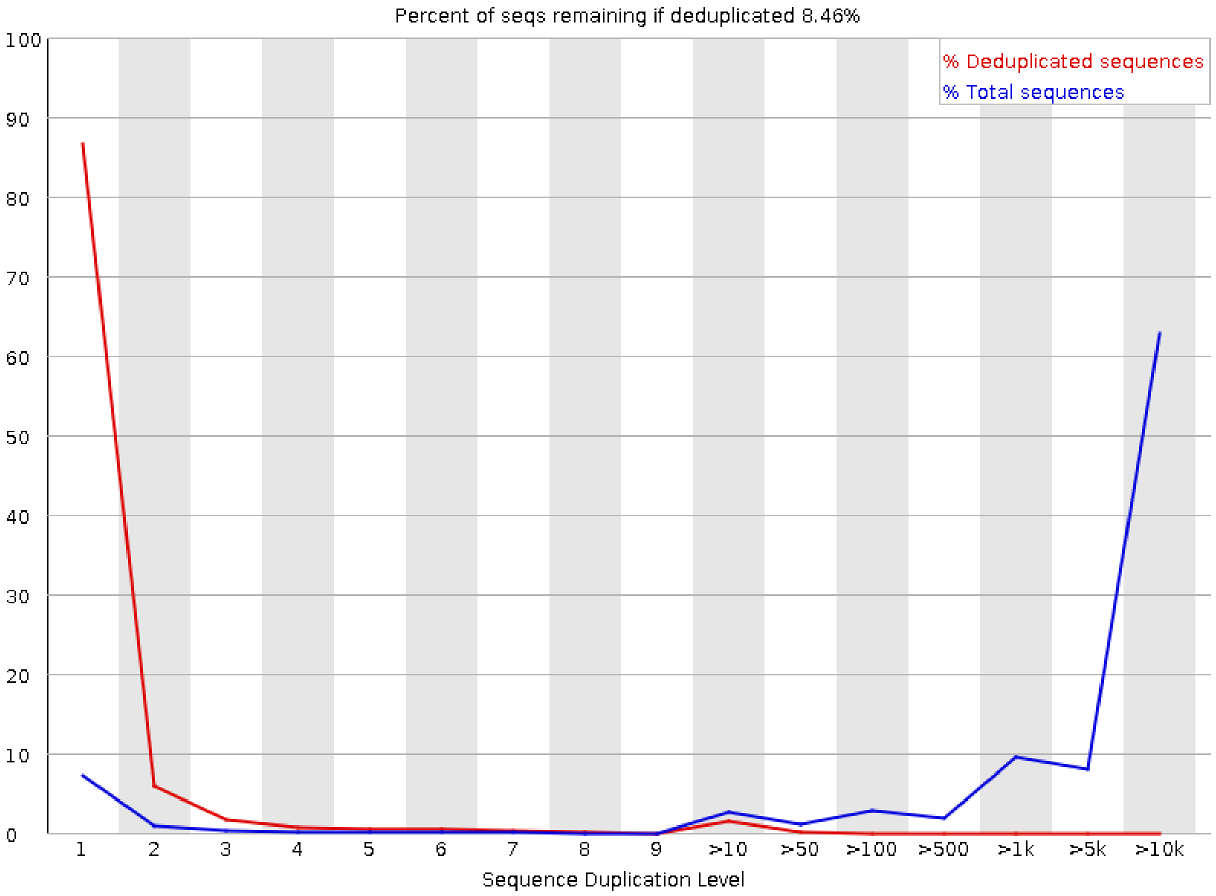

## Overrepresented sequences

| Sequence | Count | Percentage | Possible Source |
|---|---|---|---|
| TGAATTTAAGCATATTAGTCAGCGGAGGAGAAGAAACTAACCAGGATTCCCTCAGTAACGGCGAGTGAACAGGG | 731093 | 0.41857718407304007 | No Hit |
| TTGTCTCAAAGATTAAGCCATGCATGTCTAAGTACGCACGGCCGGTACAGTGAAACTGCGAATGGCTCATTAAA | 677837 | 0.3880861979536355 | No Hit |
| AAAGATTAAGCCATGCATGTCTAAGTACGCACGGCCGGTACAGTGAAACTGCGAATGGCTCATTAAATCAGTTA | 571009 | 0.32692330428599714 | No Hit |
| TTGAACTCTCTCTTCAAAGTTCTTTTCAACTTTCCCTTACGGTACTTGTTGACTATCGGTCTCGTGCCGGTATT | 560278 | 0.32077941867597515 | No Hit |
| TGGATAGTAGGTAGGGACAGTGGGAATCTCGTTCATCCATTCATGCGCGTCACTAATTAGATGACGAGGCATTT | 516425 | 0.2956719901365759 | No Hit |
| CGGTATTTAGCCTTAGATGGAGTTTACCACCCGCTTTGGGCTGCATTCCCAAGCAACCCGACTCCGGGAAGACC | 495678 | 0.2837935822760666 | No Hit |
| TTCCGTACGCCACATGTCCCGCGCCCCGCCGCGGGGCGGGGATTCGGCGCTGGGCTCTTCCCTGTTCACTCGCC | 484855 | 0.27759702333866393 | No Hit |
| TTCACCGTGCCAGACTAGAGTCAAGCTCAACAGGGTCTTCTTTCCCCGCTGATTCCGCCAAGCCCGTTCCCTTG | 445725 | 0.2551936831168617 | No Hit |
| TCTCTTCAAAGTTCTTTTCAACTTTCCCTTACGGTACTTGTTGACTATCGGTCTCGTGCCGGTATTTAGCCTTA | 417161 | 0.23883976003749655 | No Hit |
| TGAATTTAAGCATATTAGTCAGCGGAGGAAAAGAAACTAACCAGGATTCCCTCAGTAACGGCGAGTGAACAGGG | 395379 | 0.22636877724395457 | No Hit |
| TTTTCTGACACCTCCTGCTTAAAACCCAAAAGGTCAGAAGGATCGTGAGGCCCCGCTTTCACGGTCTGTATTCG | 394966 | 0.22613231980690876 | No Hit |
| TCGACTGCCGGCGACGGCCGGGTATGGGCCCGACGCTCCAGCGCCATCCATTTTCAGGGCTAGTTGATTCGGCA | 350488 | 0.2006670561630212 | No Hit |
| TTTGGGCTGCATTCCCAAGCAACCCGACTCCGGGAAGACCCGGGCCCGGCGCGCCGGGGGCCGCTACCGGCCTC | 339027 | 0.19410521915095694 | No Hit |
| TAGAATTACCACAGTTATCCAAGTAGGAGAGGAGCGAGCGACCAAAGGAACCATAACTGATTTAATGAGCCATT | 314925 | 0.18030595244955447 | No Hit |
| TTTAAATGGGTAAGAAGCCCGGCTCGCTGGCGTGGAGCCGGGCGTGGAATGCGAGTGCCTAGTGGGCCACTTTT | 295015 | 0.16890675736097585 | No Hit |
| TTAGATGGAGTTTACCACCCGCTTTGGGCTGCATTCCCAAGCAACCCGACTCCGGGAAGACCCGGGCCCGGCGC | 294690 | 0.16872068310664193 | No Hit |
| TTTAGCCTTAGATGGAGTTTACCACCCGCTTTGGGCTGCATTCCCAAGCAACCCGACTCCGGGAAGACCCGGGC | 282691 | 0.16185082163663414 | No Hit |
| TCGCATTCCACGCCCGGCTCCACGCCAGCGAGCCGGGCTTCTTACCCATTTAAAGTTTGAGAATAGGTTGAGAT | 272000 | 0.1557298374732994 | No Hit |
| TTGGCTGTGGTTTCGCTGGATAGTAGGTAGGGACAGTGGGAATCTCGTTCATCCATTCATGCGCGTCACTAATT | 271042 | 0.1551813478251398 | No Hit |
| TAGAGTCAAGCTCAACAGGGTCTTCTTTCCCCGCTGATTCCGCCAAGCCCGTTCCCTTGGCTGTGGTTTCGCTG | 270656 | 0.1549603488646078 | No Hit |
| GGTATTTAGCCTTAGATGGAGTTTACCACCCGCTTTGGGCTGCATTCCCAAGCAACCCGACTCCGGGAAGACCC | 267529 | 0.15317002826983206 | No Hit |
| TTAGGCAACCTGGTGGTCCCCCGCTCCCGGGAGGTCACCATATTGATGCCGAACTTAGTGCGGACACCCGATCG | 266009 | 0.15229977329571656 | No Hit |
| TCAAAGATTAAGCCATGCATGTCTAAGTACGCACGGCCGGTACAGTGAAACTGCGAATGGCTCATTAAATCAGT | 251309 | 0.14388349163815223 | No Hit |
| TTAGAGCCAATCCTTATCCCGAAGTTACGGATCCGGCTTGCCGACTTCCCTTACCTACATTGTTCCAACATGCC | 245753 | 0.14070248069329322 | No Hit |
| GAGATTCCCACTGTCCCTACCTACTATCCAGCGAAACCACAGCCAAGGGAACGGGCTTGGCGGAATCAGCGGGG | 245498 | 0.14055648397066198 | No Hit |
| AGAAACCTCCCGTGGAGCAGAAGGGCAAAAGCTCGCTTGATCTTGATTTTCAGTACGAATACAGACCGTGAAAG | 234315 | 0.13415381201307408 | No Hit |
| ATGTATTAGCTCTAGAATTACCACAGTTATCCAAGTAGGAGAGGAGCGAGCGACCAAAGGAACCATAACTGATT | 225219 | 0.12894602303639346 | No Hit |
| CACTCTCGACTGCCGGCGACGGCCGGGTATGGGCCCGACGCTCCAGCGCCATCCATTTTCAGGGCTAGTTGATT | 223163 | 0.12776888867666883 | No Hit |
| TCAAAGTGAAGAAATTCAATGAAGCGCGGGTAAACGGCGGGAGTAACTATGACTCTCTTAAGGTAGCCAAATGC | 219418 | 0.12562474073057503 | No Hit |
| TTTTATCCGGTAAAGCGAATGATTAGAGGTCTTGGGGCCGAAACGATCTCAACCTATTCTCAAACTTTAAATGG | 219347 | 0.12558409066270518 | No Hit |
| TTAGCTCTAGAATTACCACAGTTATCCAAGTAGGAGAGGAGCGAGCGACCAAAGGAACCATAACTGATTTAATG | 216114 | 0.1237330812342082 | No Hit |
| GACGCTCCAGCGCCATCCATTTTCAGGGCTAGTTGATTCGGCAGGTGAGTTGTTACACACTCCTTAGCGGATTC | 213568 | 0.12227540415256474 | No Hit |
| GGCATCGGGCGCCTTAACCCGGCGTTCGGTTCATCCCGCAGCGCCAGTTCTGCTTACCAAAAGTGGCCCACTAG | 210828 | 0.12070665505448813 | No Hit |
| TTAAGGTAGCCAAATGCCTCGTCATCTAATTAGTGACGCGCATGAATGGATGAACGAGATTCCCACTGTCCCTA | 201732 | 0.11549886607780749 | No Hit |
| CCACATGTCCCGCGCCCCGCCGCGGGGCGGGGATTCGGCGCTGGGCTCTTCCCTGTTCACTCGCCGTTACTGAG | 198822 | 0.11383278583131007 | No Hit |
| GCGGTTCTATTTTGTTGGTTTTCGGAACTGAGGCCATGATTAAGAGGGACGGCCGGGGGCATTCGTATTGCGCC | 193886 | 0.11100674731010342 | No Hit |
| TCATGTCTCTTCACCGTGCCAGACTAGAGTCAAGCTCAACAGGGTCTTCTTTCCCCGCTGATTCCGCCAAGCCC | 191441 | 0.10960689638134526 | No Hit |
| AACCAACACCTTTTCTGGGGTCTGATGAGCGTCGGCATCGGGCGCCTTAACCCGGCGTTCGGTTCATCCCGCAG | 191128 | 0.10942769256101754 | No Hit |
| TTCTTTTCAACTTTCCCTTACGGTACTTGTTGACTATCGGTCTCGTGCCGGTATTTAGCCTTAGATGGAGTTTA | 191074 | 0.10939677560798976 | No Hit |
| CAGGATTCCCTCAGTAACGGCGAGTGAACAGGGAAGAGCCCAGCGCCGAATCCCCGCCCCGCGGCGGGGCGCGG | 189749 | 0.1086381651864746 | No Hit |
| TTAACCCGGCGTTCGGTTCATCCCGCAGCGCCAGTTCTGCTTACCAAAAGTGGCCCACTAGGCACTCGCATTCC | 187033 | 0.10708315695641032 | No Hit |
| GAACGTCTGCCCTATCAACTTTCGATGGTAGTCGCCGTGCCTACCATGGTGACCACGGGTGACGGGGAATCAGG | 184332 | 0.10553673676885378 | No Hit |
| TCACCCGGCCCGGACACGGACAGGATTGACAGATTGATAGCTCTTTCTCGATTCCGTGGGTGGTGGTGCATGGC | 182922 | 0.10472946077312822 | No Hit |
| TGGAGTGCAGTGGCTATTCACAGGCGCGATCCCACTACTGATCAGCACGGGAGTTTGACCTGCTCCGTTTCCG | 179197 | 0.10259676355037807 | No Hit |

| Sequence | Count | Percentage | Possible Source |
|---|---|---|---|

## Adapter Content

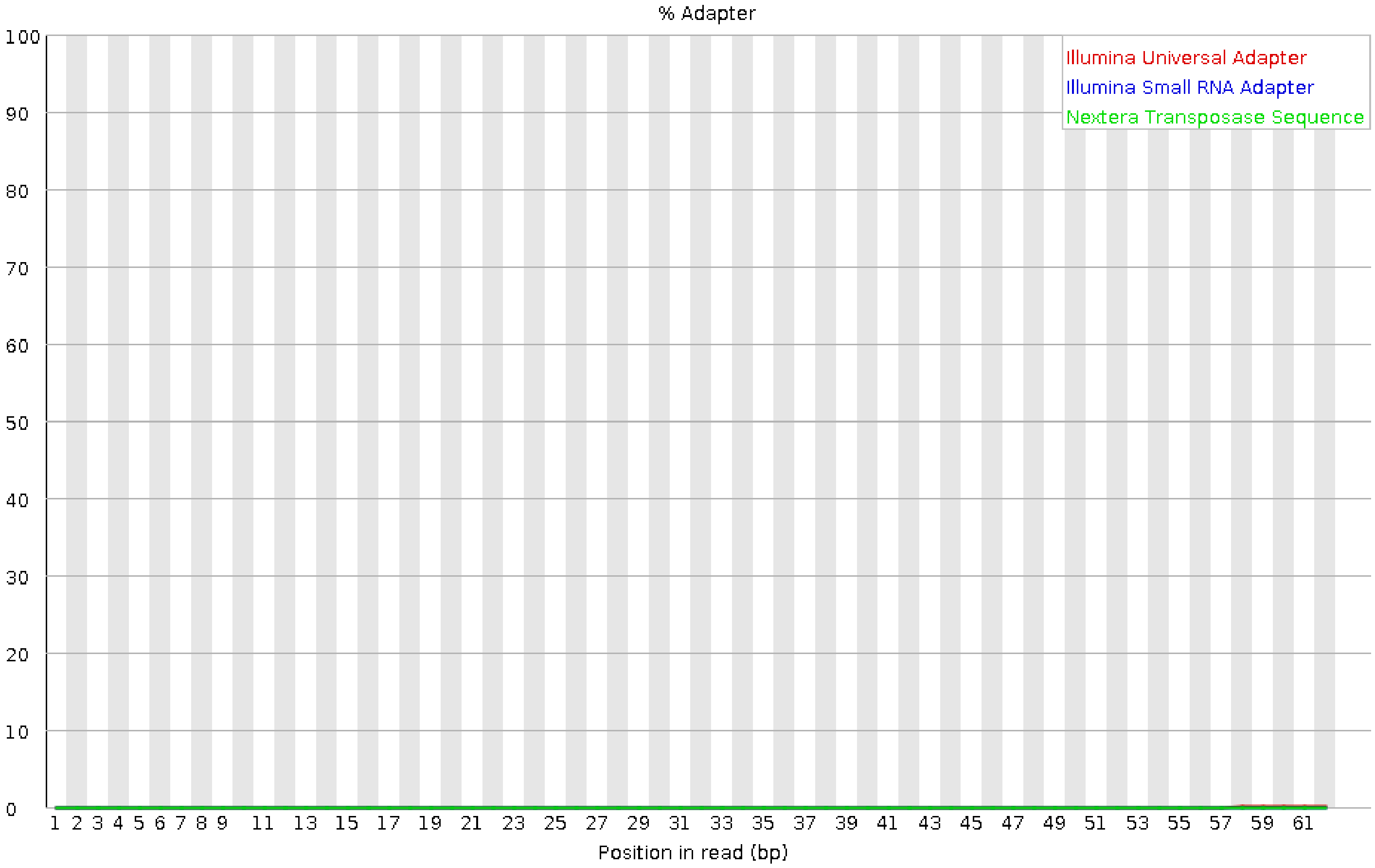


## Kmer Content

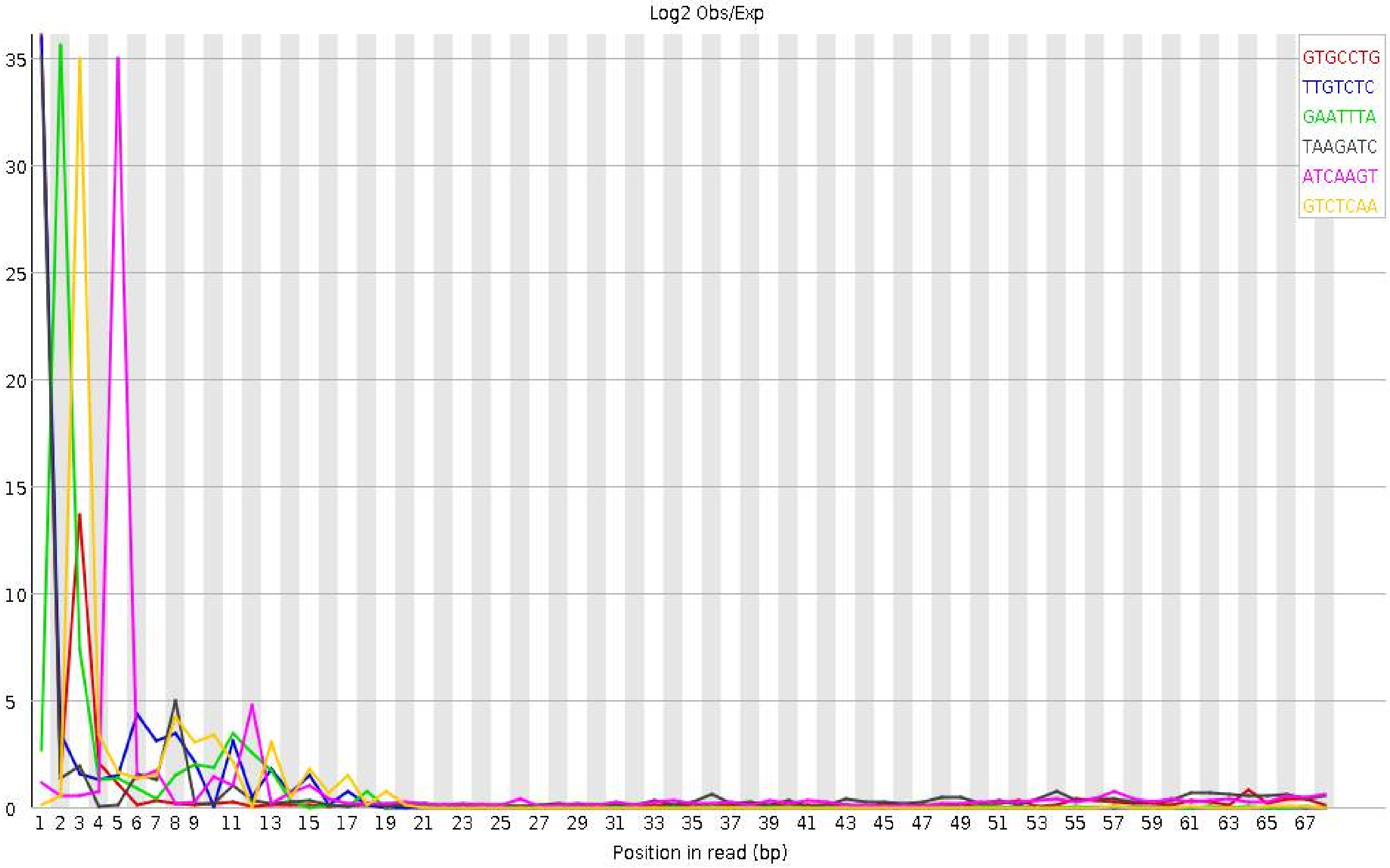


| Sequence | Count | PValue | Obs/Exp Max | Max Obs/Exp Position |
|---|---|---|---|---|
| GTGCCTG | 70405 | 0.0 | 36.09108 | 1 |
| TTGTCTC | 172205 | 0.0 | 35.98707 | 1 |
| GAATTTA | 294340 | 0.0 | 35.611076 | 2 |
| TAAGATC | 14795 | 0.0 | 35.17178 | 1 |
| ATCAAGT | 14210 | 0.0 | 35.04111 | 5 |
| GTCTCAA | 176035 | 0.0 | 35.003323 | 3 |
| ATTTAAG | 299280 | 0.0 | 34.916546 | 4 |
| AATTTAA | 304755 | 0.0 | 34.30376 | 3 |
| CTCAAAG | 181385 | 0.0 | 34.25758 | 5 |
| GTAGTAT | 14870 | 0.0 | 33.944912 | 12 |
| GCCTGTA | 76030 | 0.0 | 33.36283 | 3 |
| TAGTATC | 15500 | 0.0 | 32.411674 | 13 |
| AAGTGTA | 15315 | 0.0 | 32.048416 | 8 |
| CAAGTGT | 15585 | 0.0 | 31.863943 | 7 |
| TGCCTGT | 79870 | 0.0 | 31.746017 | 2 |
| TTTTTCG | 20900 | 0.0 | 31.663126 | 1 |
| GTGTAGT | 16230 | 0.0 | 31.204994 | 10 |
| TAAGCAT | 340210 | 0.0 | 30.985094 | 7 |
| TCAGTTA | 156670 | 0.0 | 30.471848 | 68 |
| CCTGTAG | 83265 | 0.0 | 30.357761 | 4 |

# FastQC Report

Mon 4 May 2015
Agilent_Fetal_Stomach_GCCAAT_L002_R2.fastq.gz

## Summary


- Basic Statistics
- Per base sequence quality
- Per tile sequence quality
- Per sequence quality scores
- Per base sequence content
- Per sequence GC content
- Per base N content
- Sequence Length Distribution
- Sequence Duplication Levels
- Overrepresented sequences
- Adapter Content
- Kmer Content


## Basic Statistics

| Measure | Value |
|---|---|
| Filename | Agilent_Fetal_Stomach_GCCAAT_L002_R2.fastq.gz |
| File type | Conventional base calls |
| Encoding | Sanger / Illumina 1.9 |
| Total Sequences | 174661455 |
| Sequences flagged as poor quality | 0 |
| Sequence length | 74 |
| %GC | 57 |

## Per base sequence quality

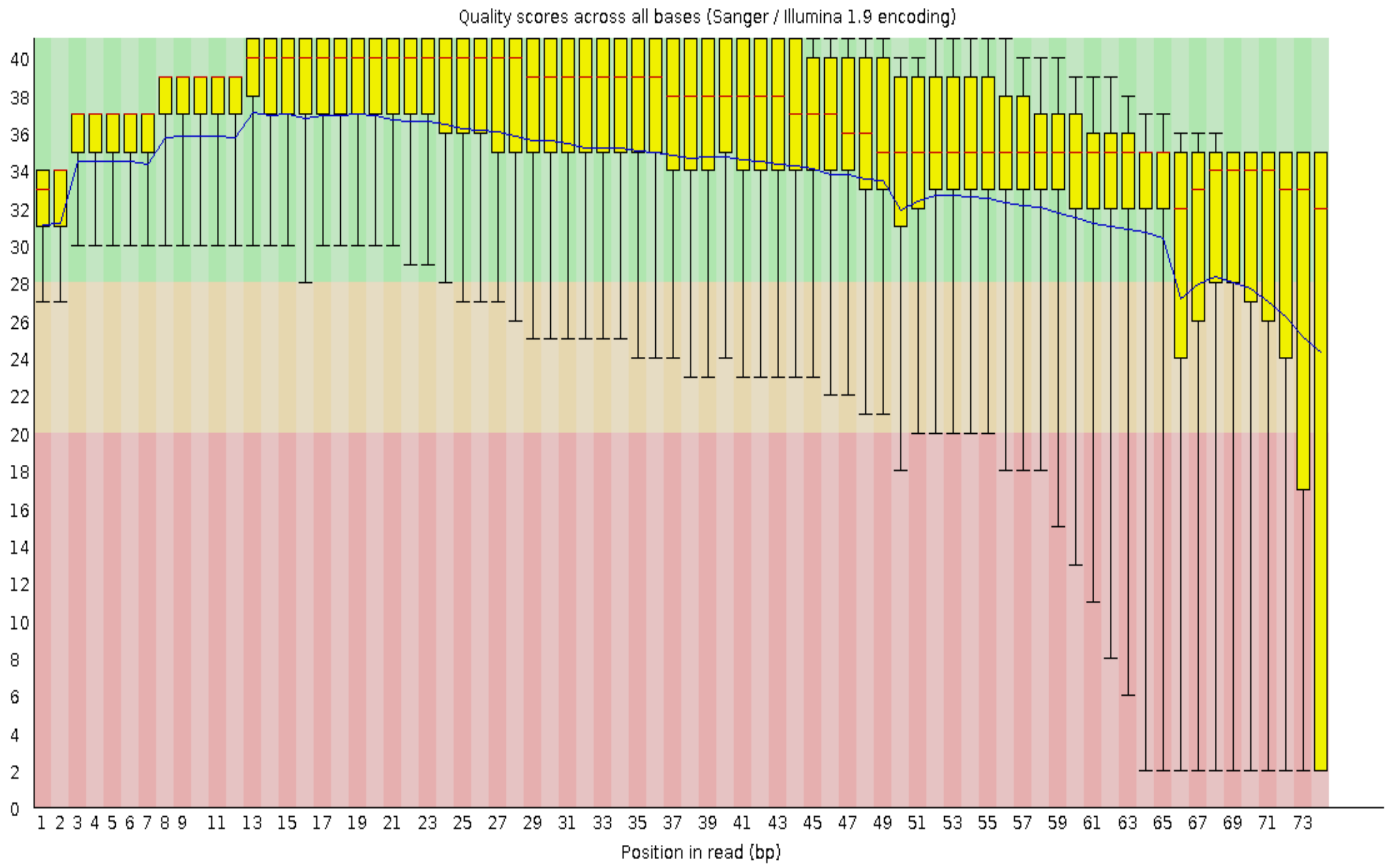


## Per tile sequence quality

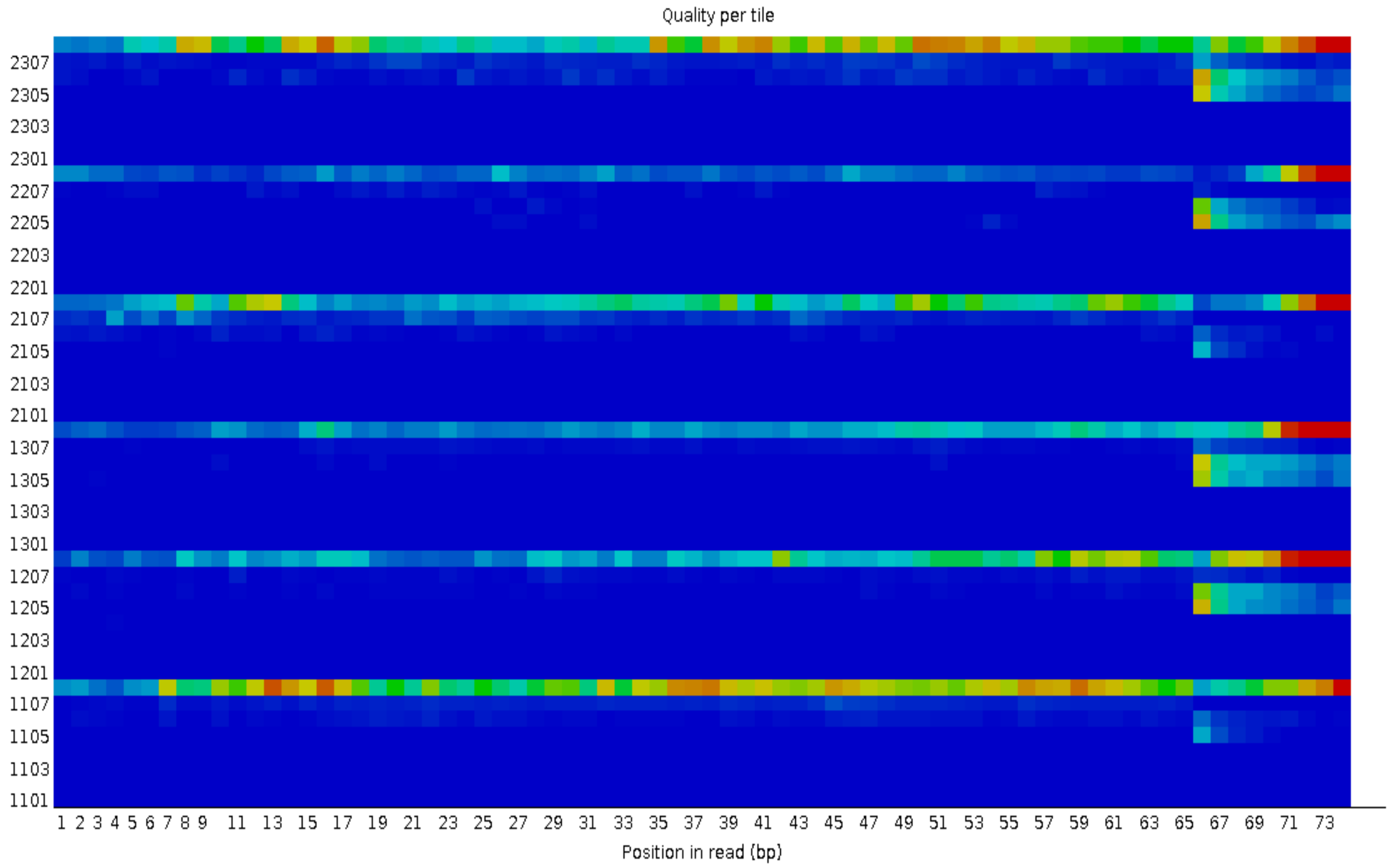

## Per sequence quality scores

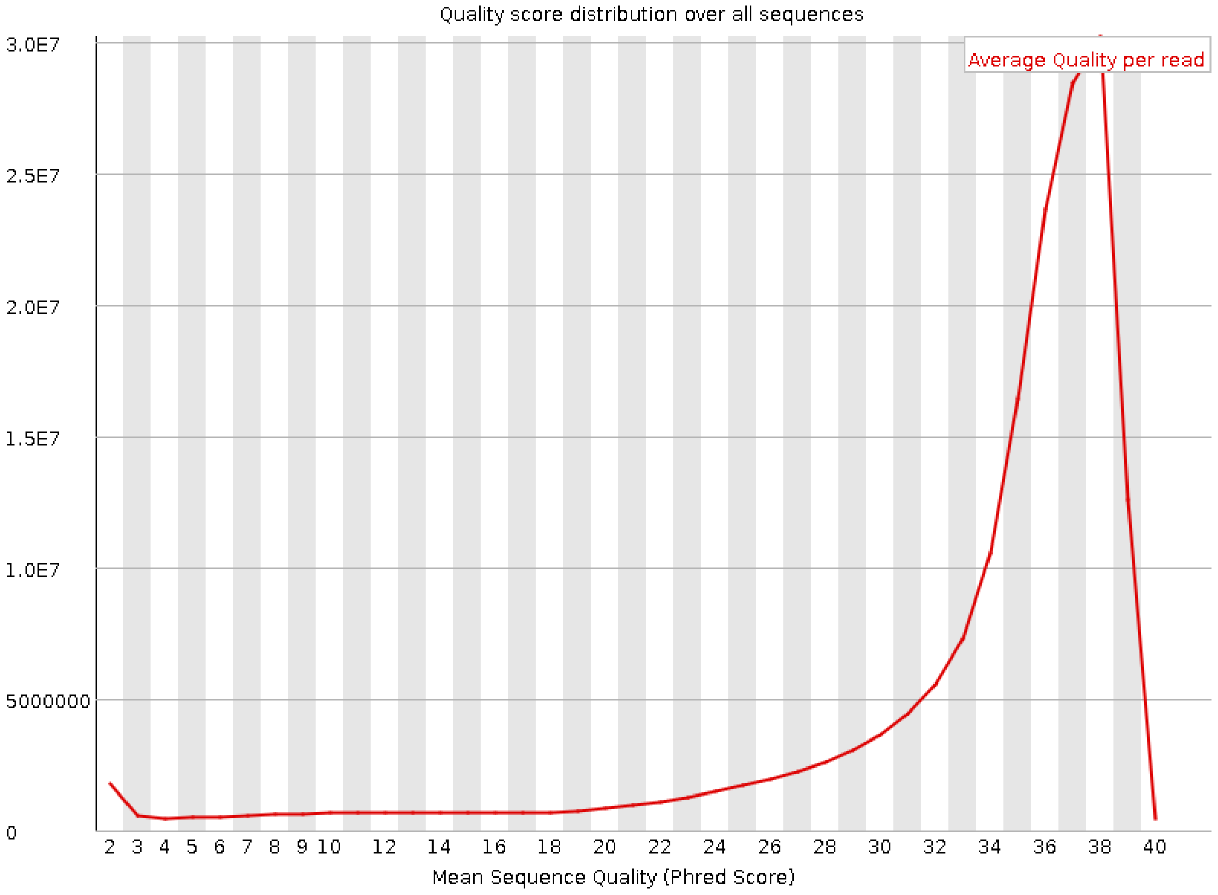


## Per base sequence content

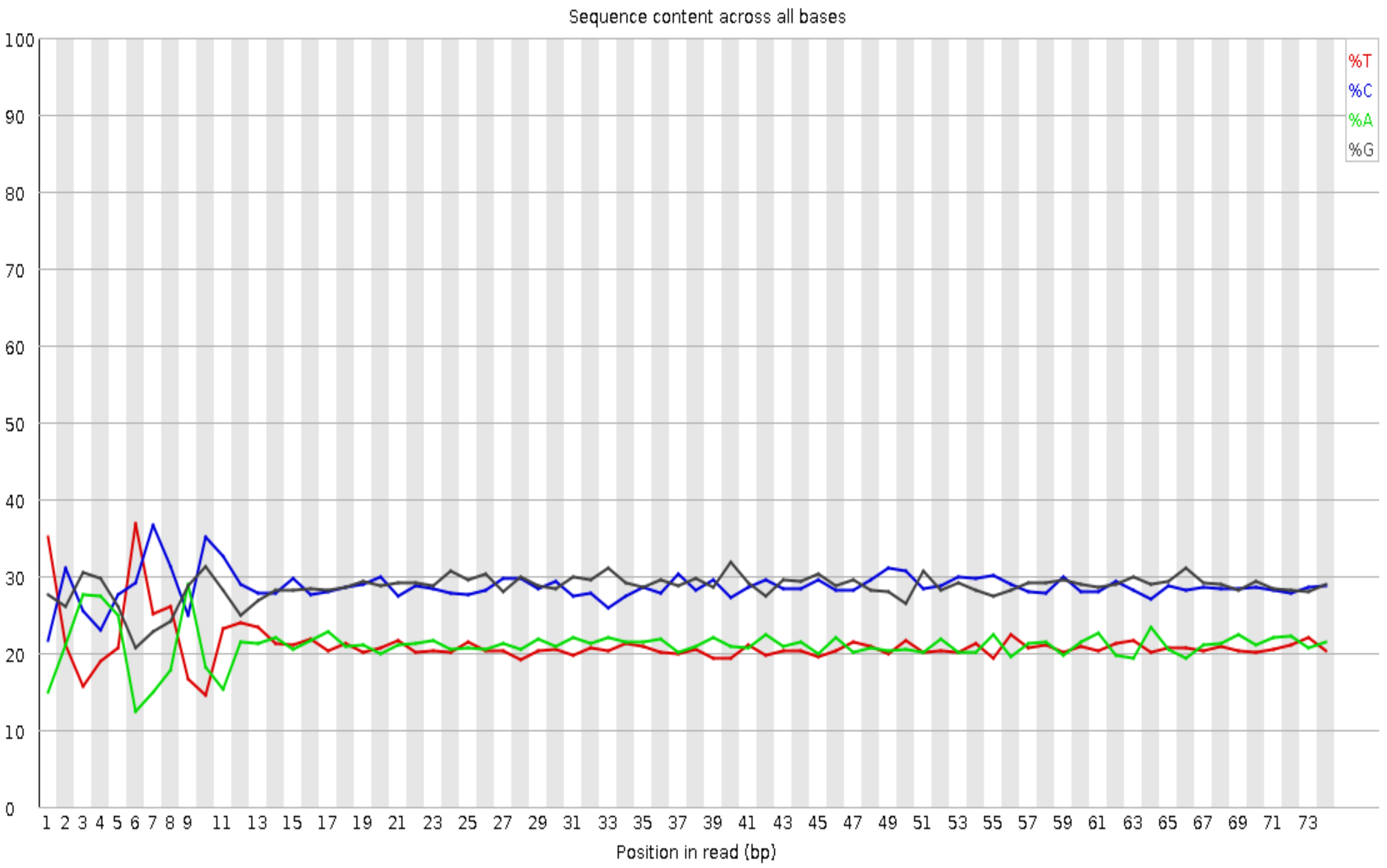


## Per sequence GC content

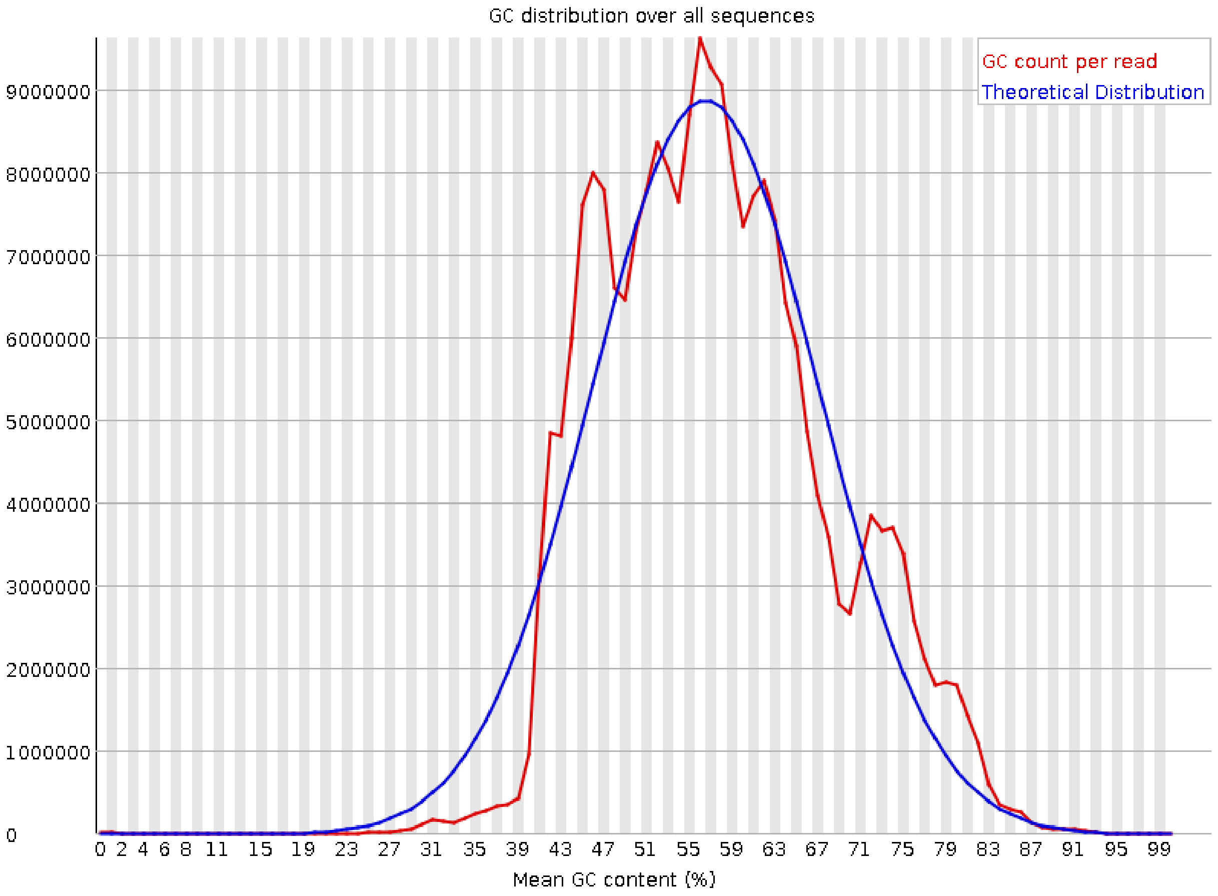

## Per base N content

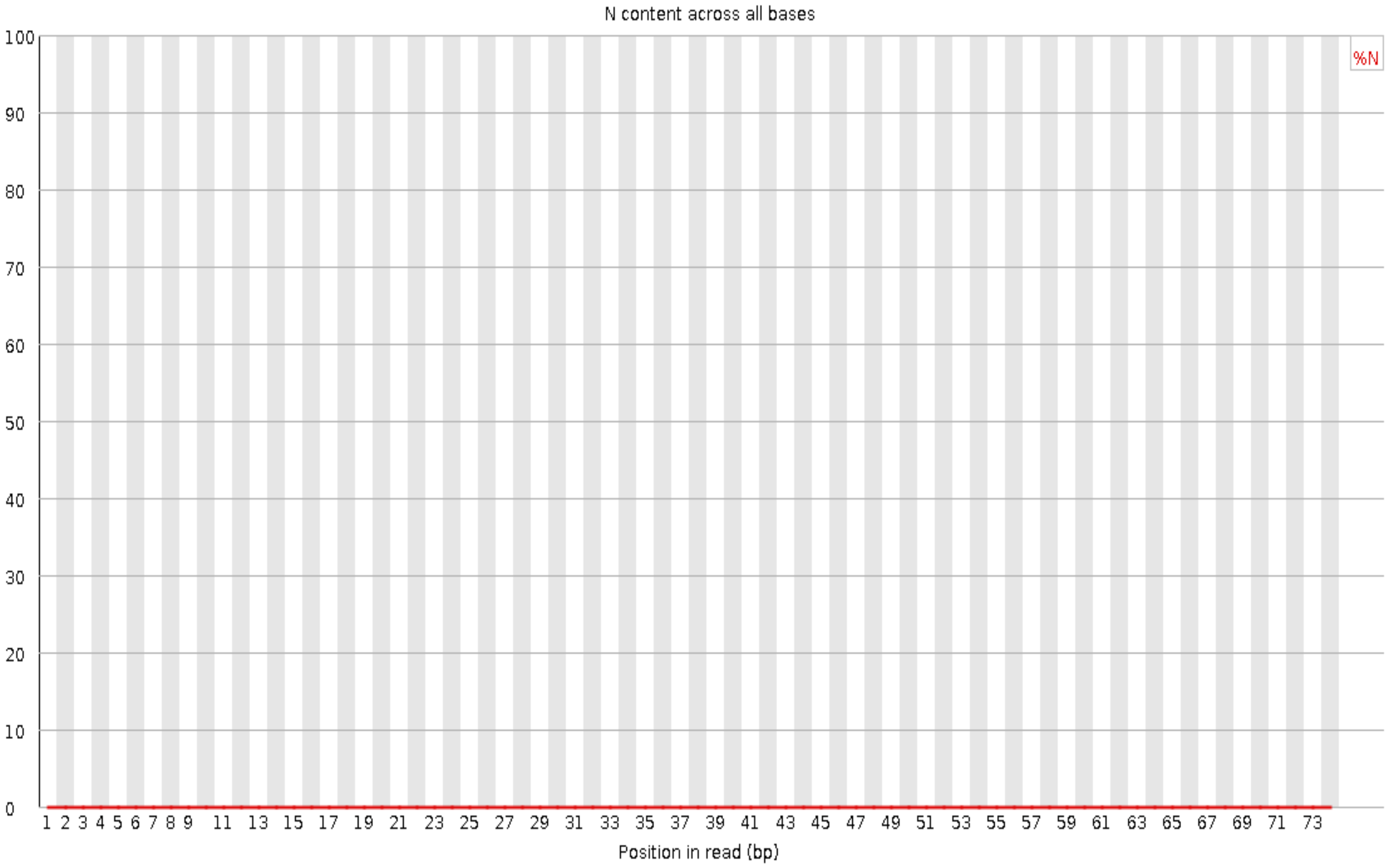


## Sequence Length Distribution

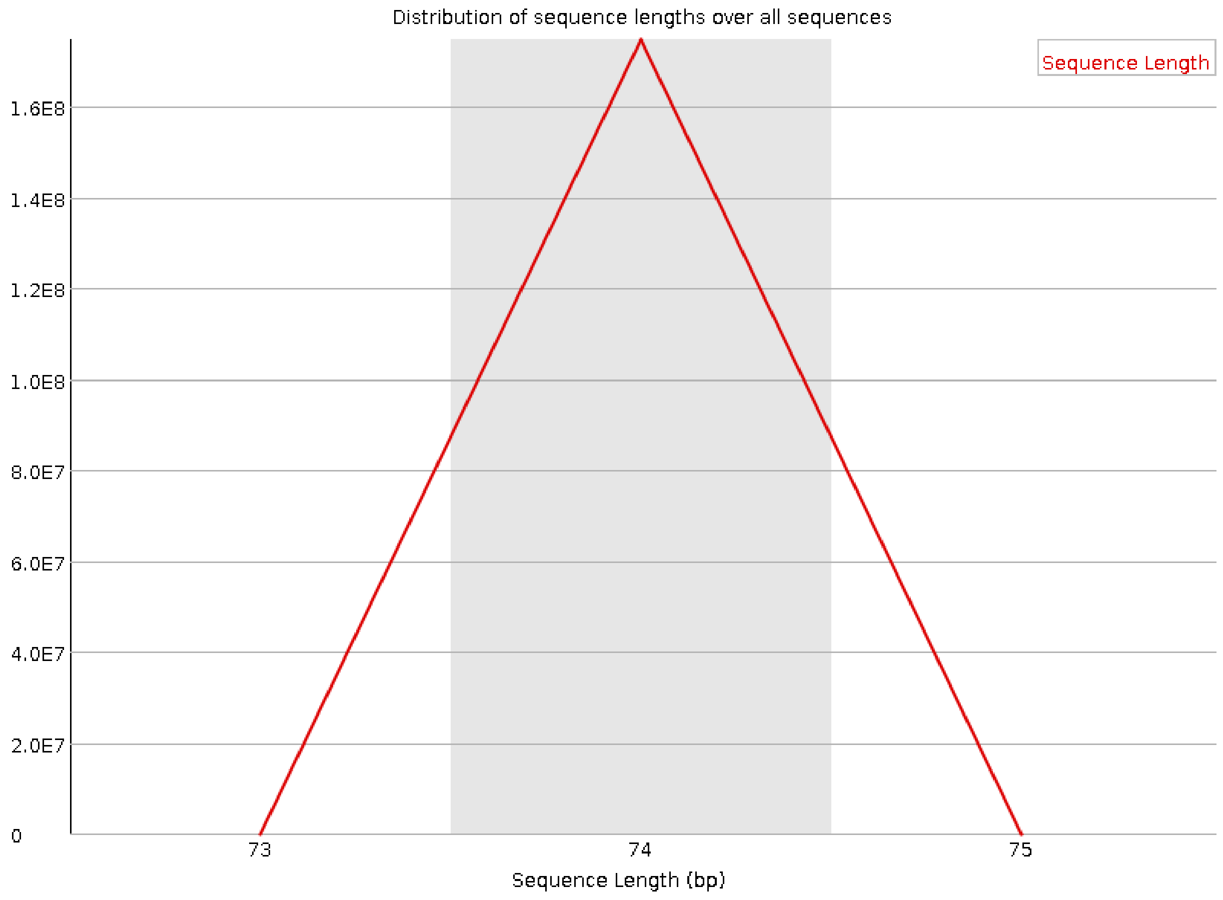


## Sequence Duplication Levels

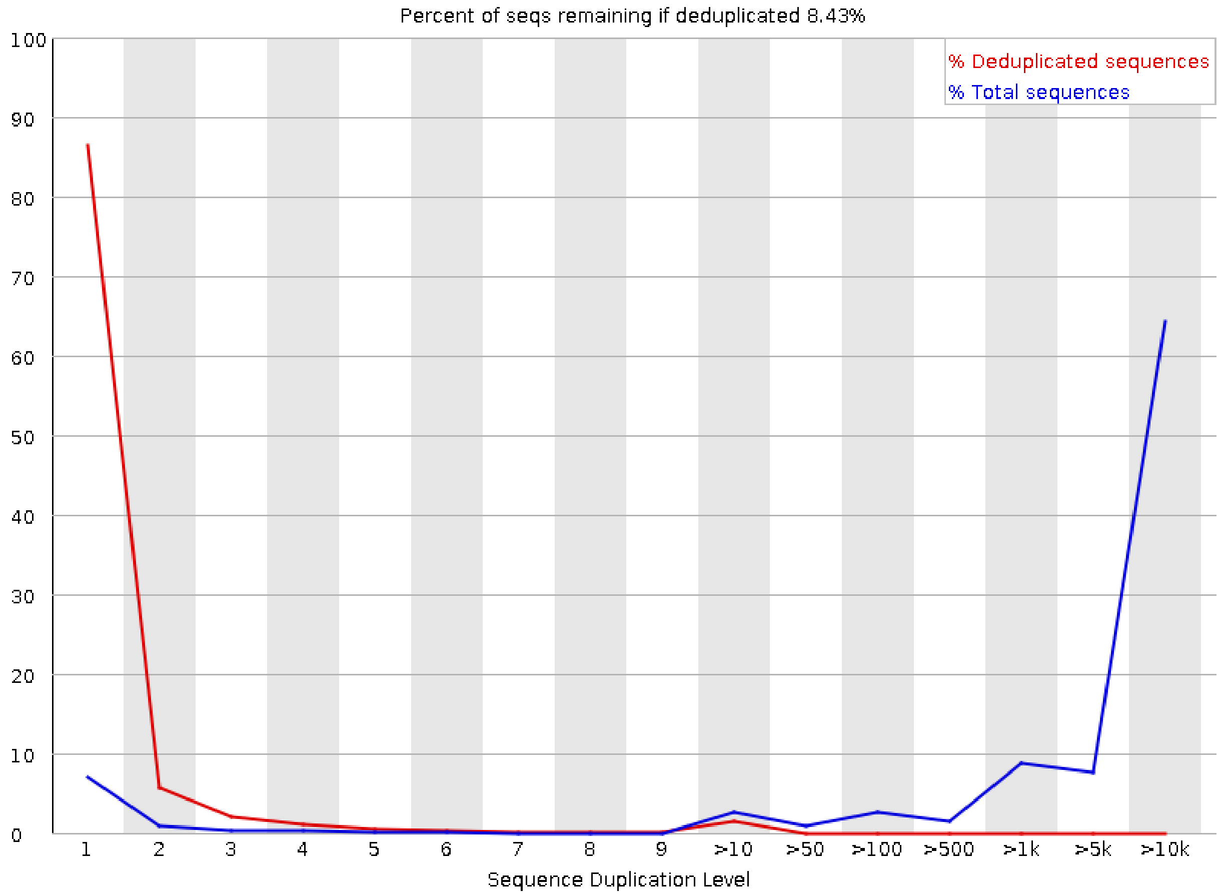

## Overrepresented sequences

| Sequence | Count | Percentage | Possible Source |
|---|---|---|---|
| TTGTCTCAAAGATTAAGCCATGCATGTCTAAGTACGCACGGCCGGTACAGTGAAACTGCGAATGGCTCATTAAA | 716590 | 0.41027369204041036 | No Hit |
| TGAATTTAAGCATATTAGTCAGCGGAGGAGAAGAAACTAACCAGGATTCCCTCAGTAACGGCGAGTGAACAGGG | 672832 | 0.38522065443689335 | No Hit |
| AAAGATTAAGCCATGCATGTCTAAGTACGCACGGCCGGTACAGTGAAACTGCGAATGGCTCATTAAATCAGTTA | 596696 | 0.34163004081238185 | No Hit |
| TTCCGTACGCCACATGTCCCGCGCCCCGCCGCGGGGCGGGATTCGGCGCTGGGCTCTTCCCTGTTCACTCGCC | 569646 | 0.3261429374901291 | No Hit |
| TTGAACTCTCTCTTCAAAGTTCTTTTCAACTTTCCCTTACGGTACTTGTTGACTATCGGTCTCGTGCCGGTATT | 562106 | 0.3218260147895825 | No Hit |
| TGGATAGTAGGTAGGGACAGTGGGAATCTCGTTCATCCATTCATGCGCGTCACTAATTAGATGACGAGGCATTT | 542626 | 0.3106730102528918 | No Hit |
| CGGTATTTAGCCTTAGATGGAGTTTACCACCCGCTTTGGGCTGCATTCCCAAGCAACCCGACTCCGGGAAGACC | 514502 | 0.2945710030870864 | No Hit |
| TTCACCGTGCCAGACTAGAGTCAAGCTCAACAGGGTCTTCTTTCCCCGCTGATTCCGCCAAGCCCGTTCCCTTG | 496738 | 0.2844004706132787 | No Hit |
| TCTCTTCAAAGTTCTTTTCAACTTTCCCTTACGGTACTTGTTGACTATCGGTCTCGTGCCGGTATTTAGCCTTA | 411172 | 0.23541084093224807 | No Hit |
| TTTTCTGACACCTCCTGCTTAAAACCCAAAAGGTCAGAAGGATCGTGAGGCCCCGCTTTCACGGTCTGTATTCG | 395513 | 0.22644549709035688 | No Hit |
| TCGACTGCCGGCGACGGCCGGGTATGGGCCCGACGCTCCAGCGCCATCCATTTTCAGGGCTAGTTGATTCGGCA | 372130 | 0.21305788389315775 | No Hit |
| TGAATTTAAGCATATTAGTCAGCGGAGGAAAAGAAACTAACCAGGATTCCCTCAGTAACGGCGAGTGAACAGGG | 363223 | 0.20795830425207437 | No Hit |
| TTTGGGCTGCATTCCCAAGCAACCCGACTCCGGGAAGACCCGGGCCCGGCGCGCCGGGGGCCGCTACCGGCCTC | 350348 | 0.20058690109961583 | No Hit |
| TAGAATTACCACAGTTATCCAAGTAGGAGAGGAGCGAGCGACCAAAGGAACCATAACTGATTTAATGAGCCATT | 312158 | 0.17872174487496398 | No Hit |
| TTGGCTGTGGTTTCGCTGGATAGTAGGTAGGGACAGTGGGAATCTCGTTCATCCATTCATGCGCGTCACTAATT | 289090 | 0.16551448057042695 | No Hit |
| TTAGATGGAGTTTACCACCCGCTTTGGGCTGCATTCCCAAGCAACCCGACTCCGGGAAGACCCGGGCCCGGCGC | 285541 | 0.1634825497131007 | No Hit |
| TAGAGTCAAGCTCAACAGGGTCTTCTTTCCCCGCTGATTCCGCCAAGCCCGTTCCCTTGGCTGTGGTTTCGCTG | 281047 | 0.16090957217778817 | No Hit |
| TTAGGCAACCTGGTGGTCCCCCGCTCCCGGGAGGTCACCATATTGATGCCGAACTTAGTGCGGACACCCGATCG | 280574 | 0.16063876257071144 | No Hit |
| TTTAAATGGGTAAGAAGCCCGGCTCGCTGGCGTGGAGCCGGGCGTGGAATGCGAGTGCCTAGTGGGCCACTTTT | 280089 | 0.16036108252962855 | No Hit |
| TCGCATTCCACGCCCGGCTCCACGCCAGCGAGCCGGGCTTCTTACCCATTTAAAGTTTGAGAATAGGTTGAGAT | 274823 | 0.15734610707325208 | No Hit |
| GGTATTTAGCCTTAGATGGAGTTTACCACCCGCTTTGGGCTGCATTCCCAAGCAACCCGACTCCGGGAAGACCC | 272048 | 0.15575731920932412 | No Hit |
| TCAAAGATTAAGCCATGCATGTCTAAGTACGCACGGCCGGTACAGTGAAACTGCGAATGGCTCATTAAATCAGT | 262955 | 0.15055124784114504 | No Hit |
| AGAAACCTCCCGTGGAGCAGAAGGGCAAAAGCTCGCTTGATCTTGATTTTCAGTACGAATACAGACCGTGAAAG | 260608 | 0.14920750545677064 | No Hit |
| GAACGTCTGCCCTATCAACTTTCGATGGTAGTCGCCGTGCCTACCATGGTGACCACGGGTGACGGGGAATCAGG | 255876 | 0.14649826431366897 | No Hit |
| GGCATCGGGCGCCTTAACCCGGCGTTCGGTTCATCCCGCAGCGCCAGTTCTGCTTACCAAAAGTGGCCCACTAG | 254233 | 0.1455575873909902 | No Hit |
| TTTAGCCTTAGATGGAGTTTACCACCCGCTTTGGGCTGCATTCCCAAGCAACCCGACTCCGGGAAGACCCGGGC | 251364 | 0.14391498112734719 | No Hit |
| GAGATTCCCACTGTCCCTACCTACTATCCAGCGAAACCACAGCCAAGGGAACGGGCTTGGCGGAATCAGCGGGG | 249561 | 0.1428826984179194 | No Hit |
| GACGCTCCAGCGCCATCCATTTTCAGGGCTAGTTGATTCGGCAGGTGAGTTGTTACACACTCCTTAGCGGATTC | 243079 | 0.13917151898225055 | No Hit |
| GGGTCTTCCGTACGCCACATGTCCCGCGCCCCGCCGCGGGGCGGGATTCGGCGCTGGGCTCTTCCCTGTTCAC | 239699 | 0.1372363467371779 | No Hit |
| CACTCTCGACTGCCGGCGACGGCCGGGTATGGGCCCGACGCTCCAGCGCCATCCATTTTCAGGGCTAGTTGATT | 233725 | 0.13381601567443716 | No Hit |
| ATGTATTAGCTCTAGAATTACCACAGTTATCCAAGTAGGAGAGGAGCGAGCGACCAAAGGAACCATAACTGATT | 233177 | 0.13350226585482183 | No Hit |
| TCAAAGTGAAGAAATTCAATGAAGCGCGGGTAAACGGCGGGAGTAACTATGACTCTCTTAAGGTAGCCAAATGC | 226475 | 0.1296651284623731 | No Hit |
| CCACATGTCCCGCGCCCCGCCGCGGGGCGGGATTCGGCGCTGGGCTCTTCCCTGTTCACTCGCCGTTACTGAG | 222677 | 0.12749063609941874 | No Hit |
| TTAGAGCCAATCCTTATCCCGAAGTTACGGATCCGGCTTGCCGACTTCCCTTACCTACATTGTTCCAACATGCC | 222029 | 0.1271196326630853 | No Hit |
| TTTTATCCGGTAAAGCGAATGATTAGAGGTCTTGGGGCCGAAACGATCTCAACCTATTCTCAAACTTTAAATGG | 221652 | 0.12690378652805795 | No Hit |
| TCACCCGGCCCGGACACGGACAGGATTGACAGATTGATAGCTCTTTCTCGATTCCGTGGGTGGTGGTGCATGGC | 208633 | 0.11944993816752529 | No Hit |
| TTAGCTCTAGAATTACCACAGTTATCCAAGTAGGAGAGGAGCGAGCGACCAAAGGAACCATAACTGATTTAATG | 208244 | 0.11922722159849178 | No Hit |
| GCGGTTCTATTTTGTTGGTTTTCGGAACTGAGGCCATGATTAAGAGGGACGGCCGGGGGCATTCGTATTGCGCC | 207675 | 0.11890144851936565 | No Hit |
| TCATGTCTCTTCACCGTGCCAGACTAGAGTCAAGCTCAACAGGGTCTTCTTTCCCCGCTGATTCCGCCAAGCCC | 205784 | 0.11781878262722592 | No Hit |
| AACCAACACCTTTTCTGGGGTCTGATGAGCGTCGGCATCGGGCGCCTTAACCCGGCGTTCGGTTCATCCCGCAG | 203894 | 0.11673668927125334 | No Hit |
| TTAAGGTAGCCAAATGCCTCGTCATCTAATTAGTGACGCGCATGAATGGATGAACGAGATTCCCACTGTCCCTA | 196443 | 0.11247072228958586 | No Hit |
| TTAACCCGGCGTTCGGTTCATCCCGCAGCGCCAGTTCTGCTTACCAAAAGTGGCCCACTAGGCACTCGCATTCC | 194911 | 0.1115935968814642 | No Hit |
| TCGGCATCGGGCGCCTTAACCCGGCGTTCGGTTCATCCCGCAGCGCCAGTTCTGCTTACCAAAAGTGGCCCACT | 191201 | 0.10946948770122177 | No Hit |
| GAAGGCCCGCGGCGGGTGTTGACGCGATGTGATTTCTGCCCAGTGCTCTGAATGTCAAAGTGAAGAAATTCAAT | 189221 | 0.1083358660902029 | No Hit |

| Sequence | Count | Percentage | Possible Source |
|---|---|---|---|
| CAGGATTCCCTCAGTAACGGCGAGTGAACAGGGAAGAGCCCAGCGCCGAATCCCCGCCCCGCGGCGGGGCGCGG | 188098 | 0.10769290797445837 | [illegible] |
| TCGATCAGAAGGACTTGGGCCCCCCACGAGCGGCGCCGGGGAGCGGGTCTTCCGTACGCCACATGTCCCGCGCC | 185753 | 0.10635031066241833 | [illegible] |
| TGGAGTGCAGTGGCTATTCACAGGCGCGATCCCACTACTGATCAGCACGGGAGTTTTGACCTGCTCCGTTTCCG | 185672 | 0.10630393523287664 | No Hit |
| TTCTTTTCAACTTTCCCTTACGGTACTTGTTGACTATCGGTCTCGTGCCGGTATTTAGCCTTAGATGGAGTTTA | 178370 | 0.10212327614011918 | No Hit |
| TCTGGTGGAGGTCCGTAGCGGTCCTGACGTGCAAATCGGTCGTCCGACCTGGGTATAGGGGCGAAAGACTAATC | 177255 | 0.10148489831371207 | No Hit |
| GCAGGTGTCCTAAGGCGAGCTCAGGGAGGACAGAAACCTCCCGTGGAGCAGAAGGGCAAAAGCTCGCTTGATCT | 175579 | 0.1005253276975163 | No Hit |
| CAGACTAGAGTCAAGCTCAACAGGGTCTTCTTTCCCCGCTGATTCCGCCAAGCCCGTTCCCTTGGCTGTGGTTT | 175568 | 0.10051902979967733 | No Hit |
| GCGTAACTAGTTAGCATGCCAGAGTCTCGTTCGTTATCGGAATTAACCAGACAAATCGCTCCACCAACTAAGAA | 175246 | 0.10033467315384496 | No Hit |
| CGACATCGAAGGATCAAAAAGCGACGTCGCTATGAACGCTTGGCCGCCACAAGCCAGTTATCCCTGTGGTAACT | 175219 | 0.10031921467733107 | No Hit |

## Adapter Content

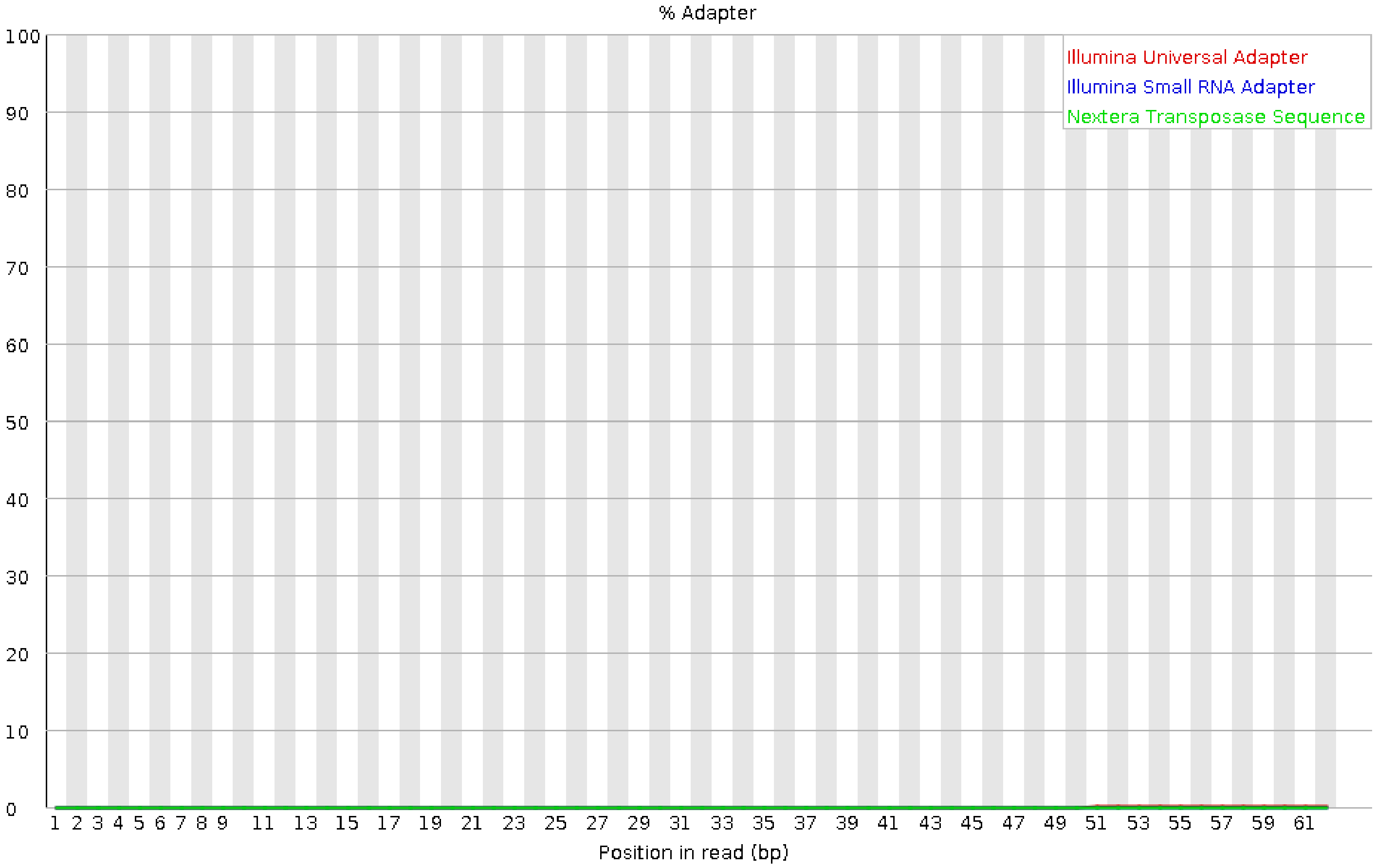


## Kmer Content

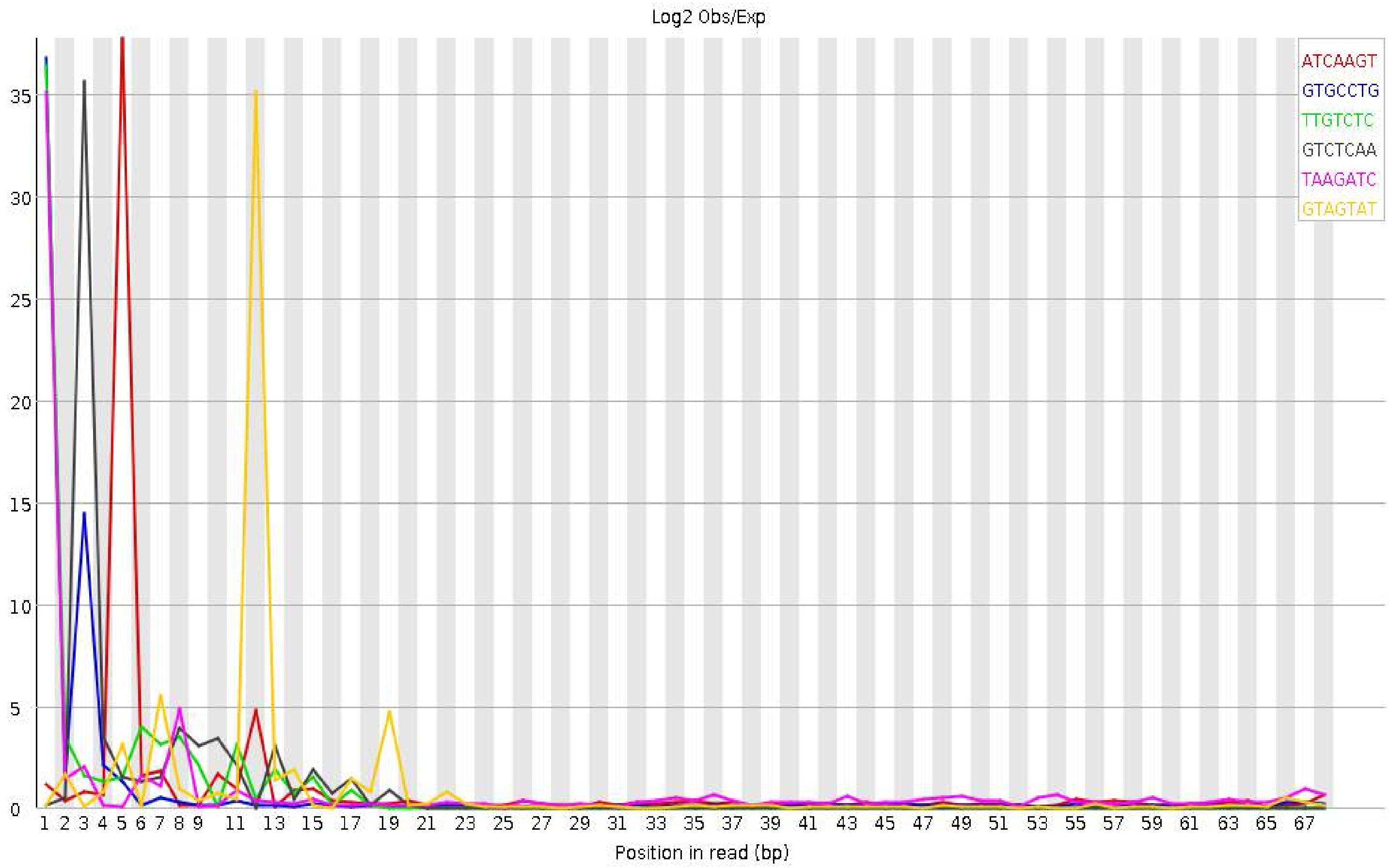


| Sequence | Count | PValue | Obs/Exp Max | Max Obs/Exp Position |
|---|---|---|---|---|
| ATCAAGT | 13055 | 0.0 | 37.742374 | 5 |
| GTGCCTG | 64975 | 0.0 | 36.833176 | 1 |
| TTGTCTC | 169635 | 0.0 | 36.44221 | 1 |
| GTCTCAA | 173370 | 0.0 | 35.645027 | 3 |
| TAAGATC | 14690 | 0.0 | 35.190037 | 1 |
| GTAGTAT | 14065 | 0.0 | 35.145073 | 12 |
| GAATTTA | 264080 | 0.0 | 34.910633 | 2 |
| CTCAAAG | 178445 | 0.0 | 34.8364 | 5 |
| ATTTAAG | 268925 | 0.0 | 34.27311 | 4 |
| GCCTGTA | 70885 | 0.0 | 34.046875 | 3 |
| TAGTATC | 14535 | 0.0 | 34.008484 | 13 |
| AATTTAA | 275260 | 0.0 | 33.524067 | 3 |
| GTGTCGA | 4945 | 0.0 | 33.15116 | 3 |
| CAAGTGT | 14550 | 0.0 | 33.086235 | 7 |
| AAGTGTA | 14515 | 0.0 | 33.00197 | 8 |
| GTGTAGT | 15005 | 0.0 | 32.988716 | 10 |
| TGTCGAG | 5000 | 0.0 | 32.7863 | 4 |
| TGCCTGT | 74430 | 0.0 | 32.04554 | 2 |
| AGTATCT | 15805 | 0.0 | 31.684086 | 14 |
| TCATTAC | 5375 | 0.0 | 30.951376 | 67 |

**Produced by FastQC (version 0.11.2)**

# FastQC Report

Mon 4 May 2015
Biochain_Adult_Colon_TGACCA_L007_R1.fastq.gz

## Summary


- Basic Statistics
- Per base sequence quality
- Per tile sequence quality
- Per sequence quality scores
- Per base sequence content
- Per sequence GC content
- Per base N content
- Sequence Length Distribution
- Sequence Duplication Levels
- Overrepresented sequences
- Adapter Content
- Kmer Content


## Basic Statistics

| Measure | Value |
|---|---|
| Filename | Biochain_Adult_Colon_TGACCA_L007_R1.fastq.gz |
| File type | Conventional base calls |
| Encoding | Sanger / Illumina 1.9 |
| Total Sequences | 68628998 |
| Sequences flagged as poor quality | 0 |
| Sequence length | 76 |
| %GC | 52 |

## Per base sequence quality

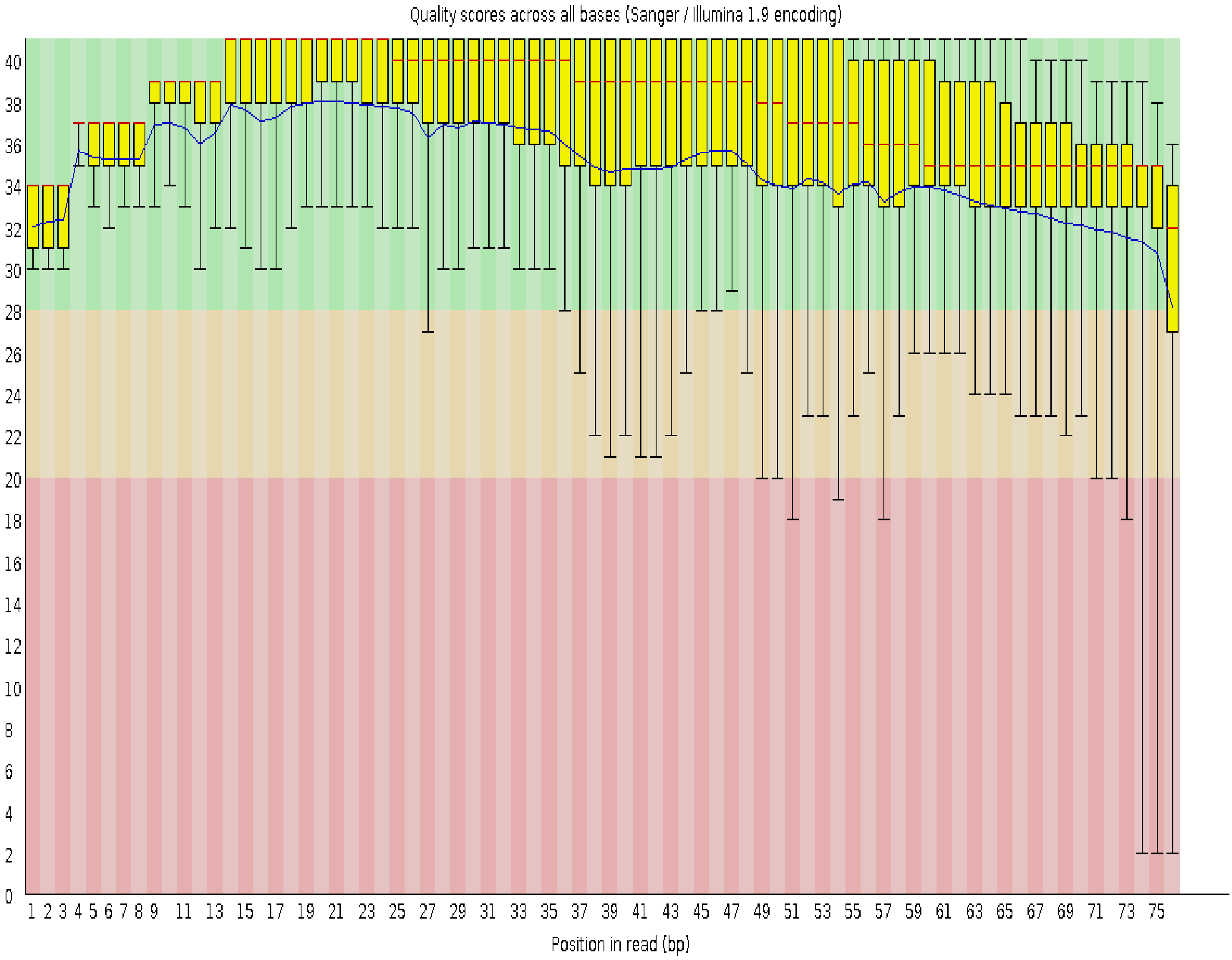


## Per tile sequence quality

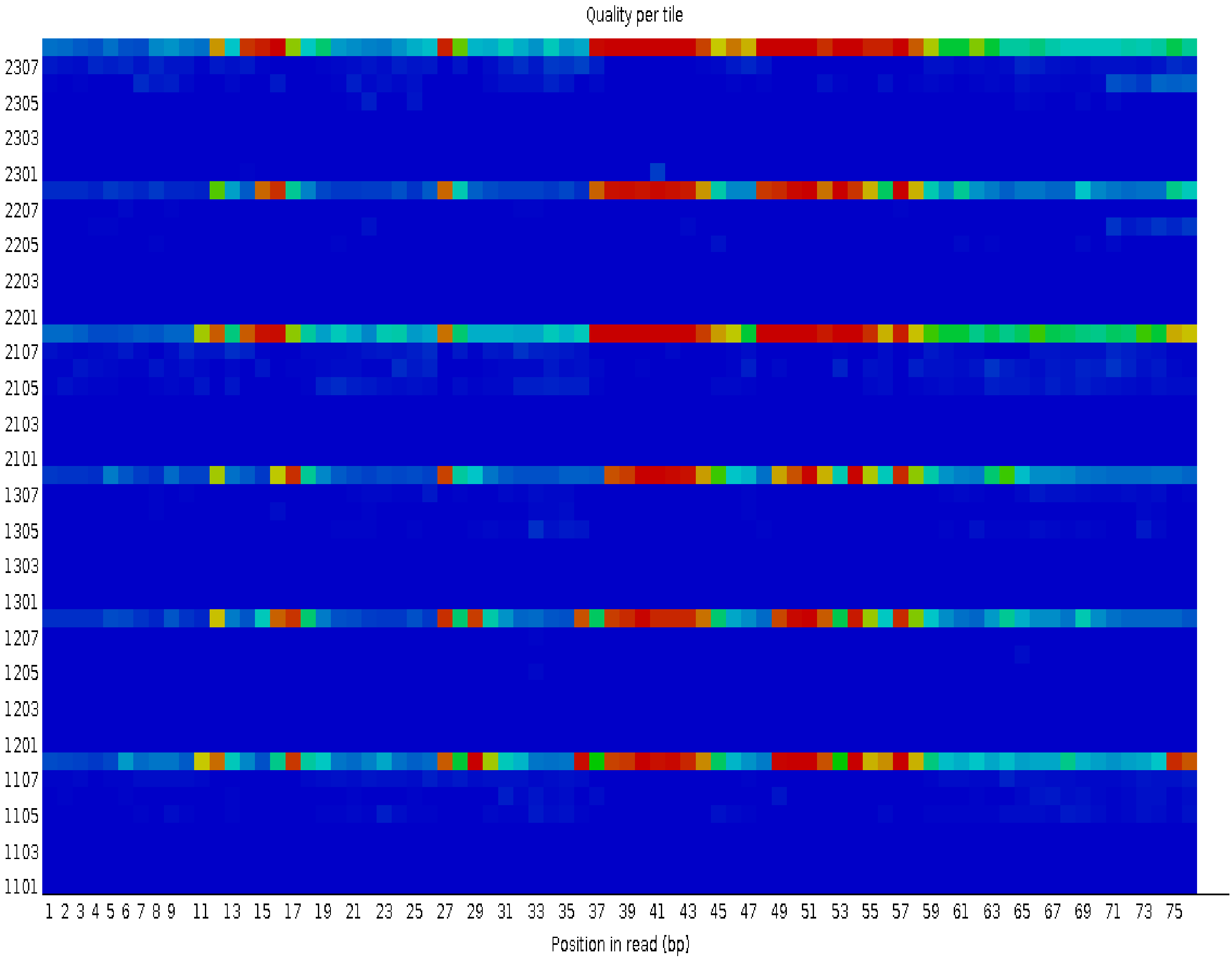


## Per sequence quality scores

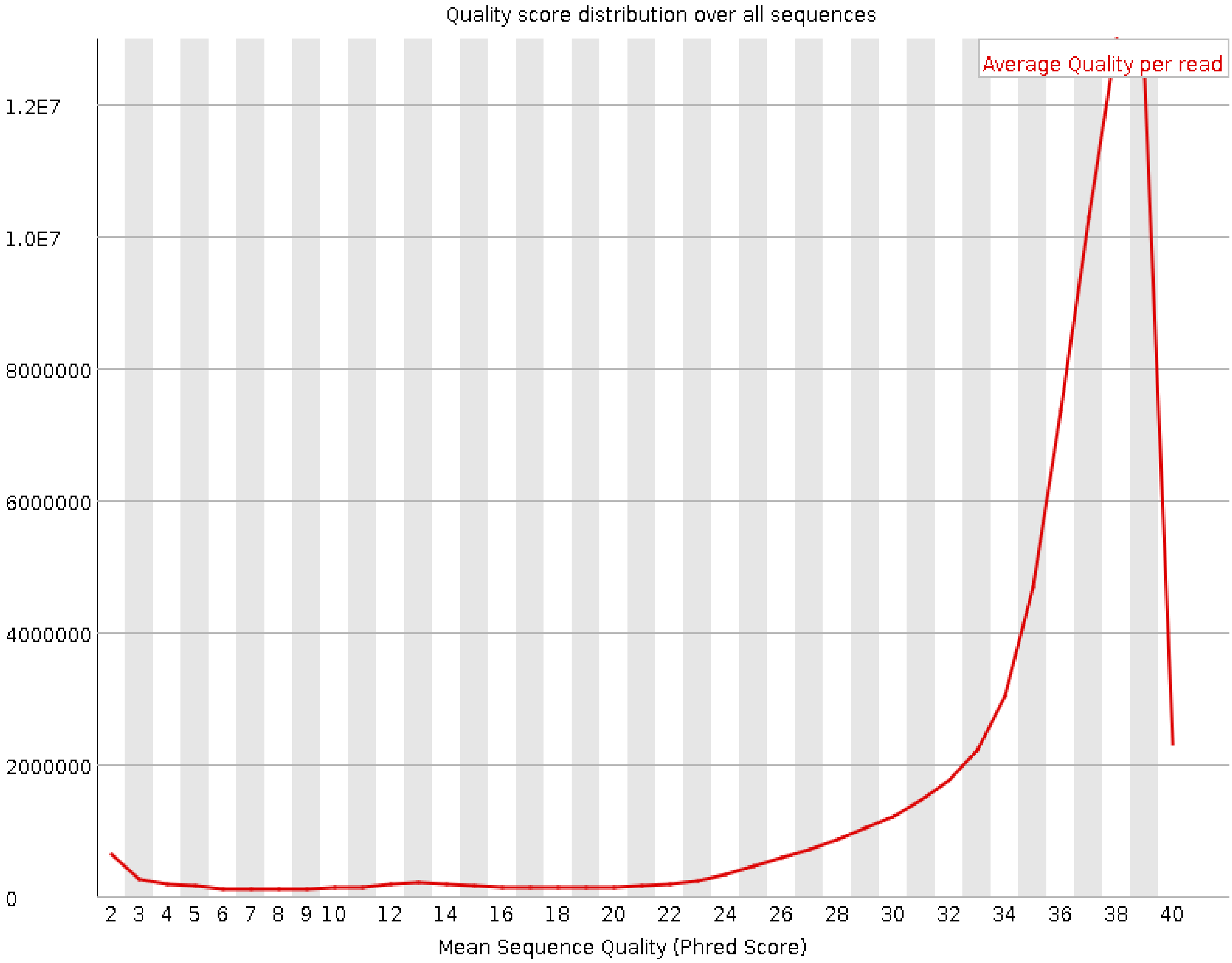


## Per base sequence content

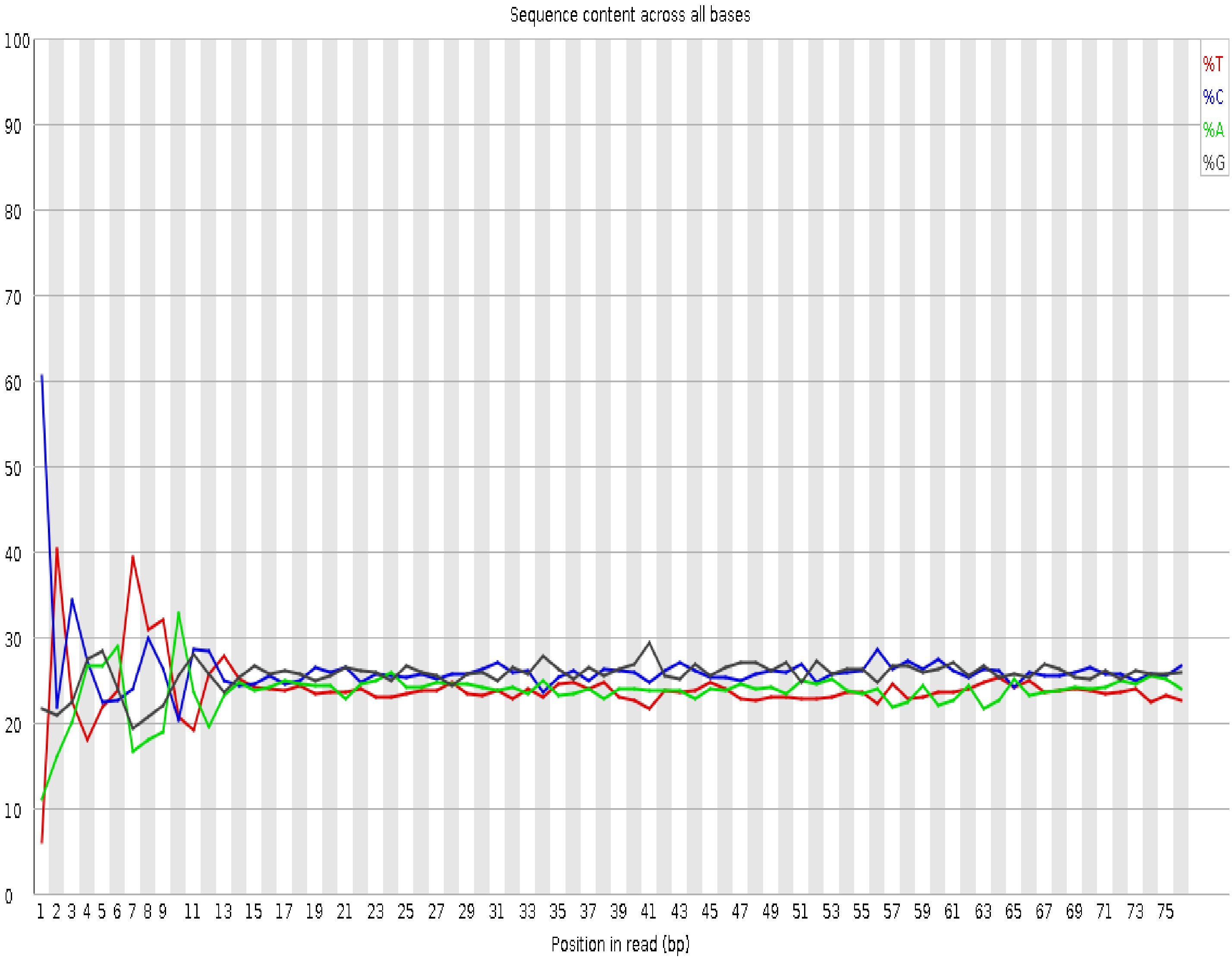


## Per sequence GC content

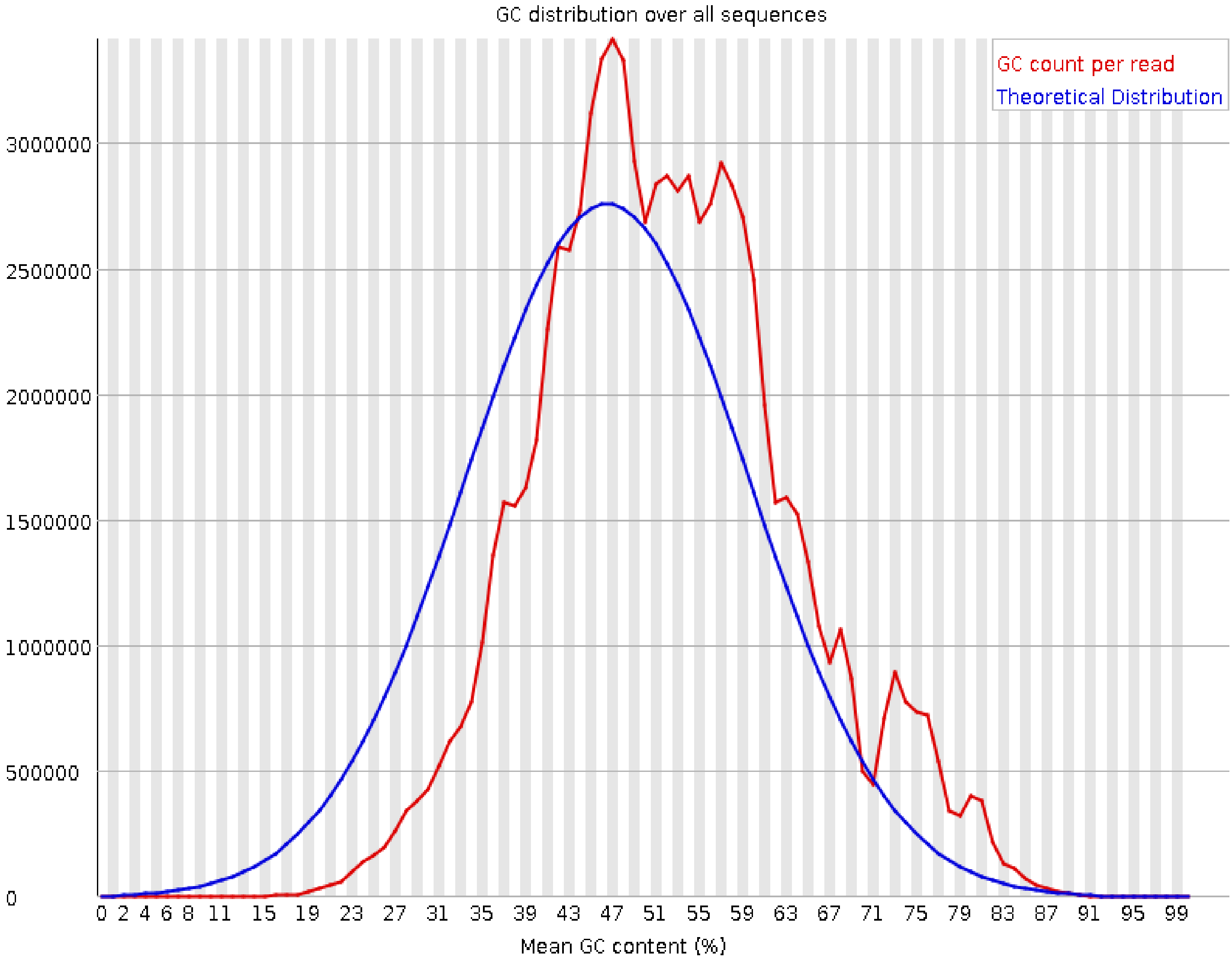


# Per base N content

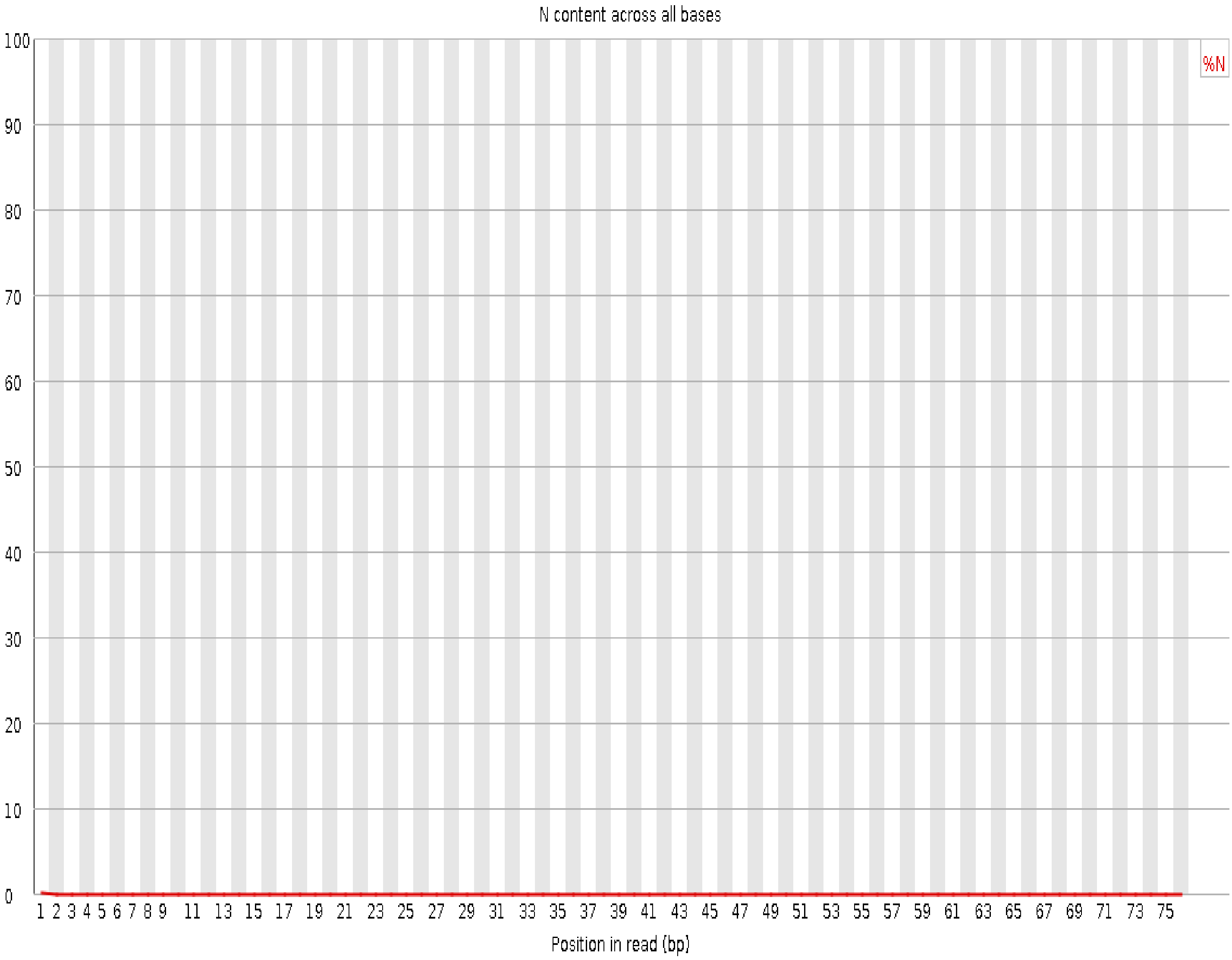


## Sequence Length Distribution

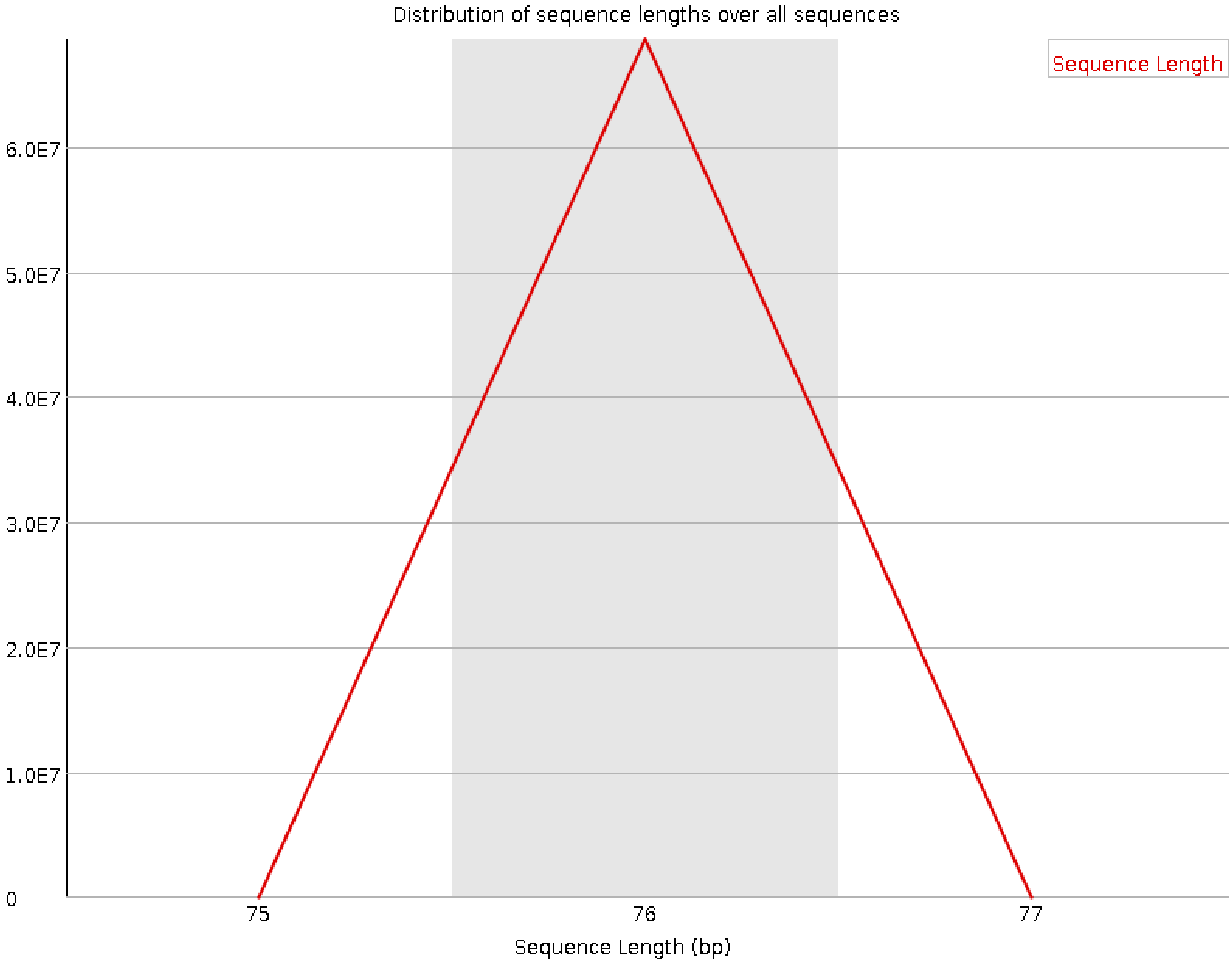


## Sequence Duplication Levels

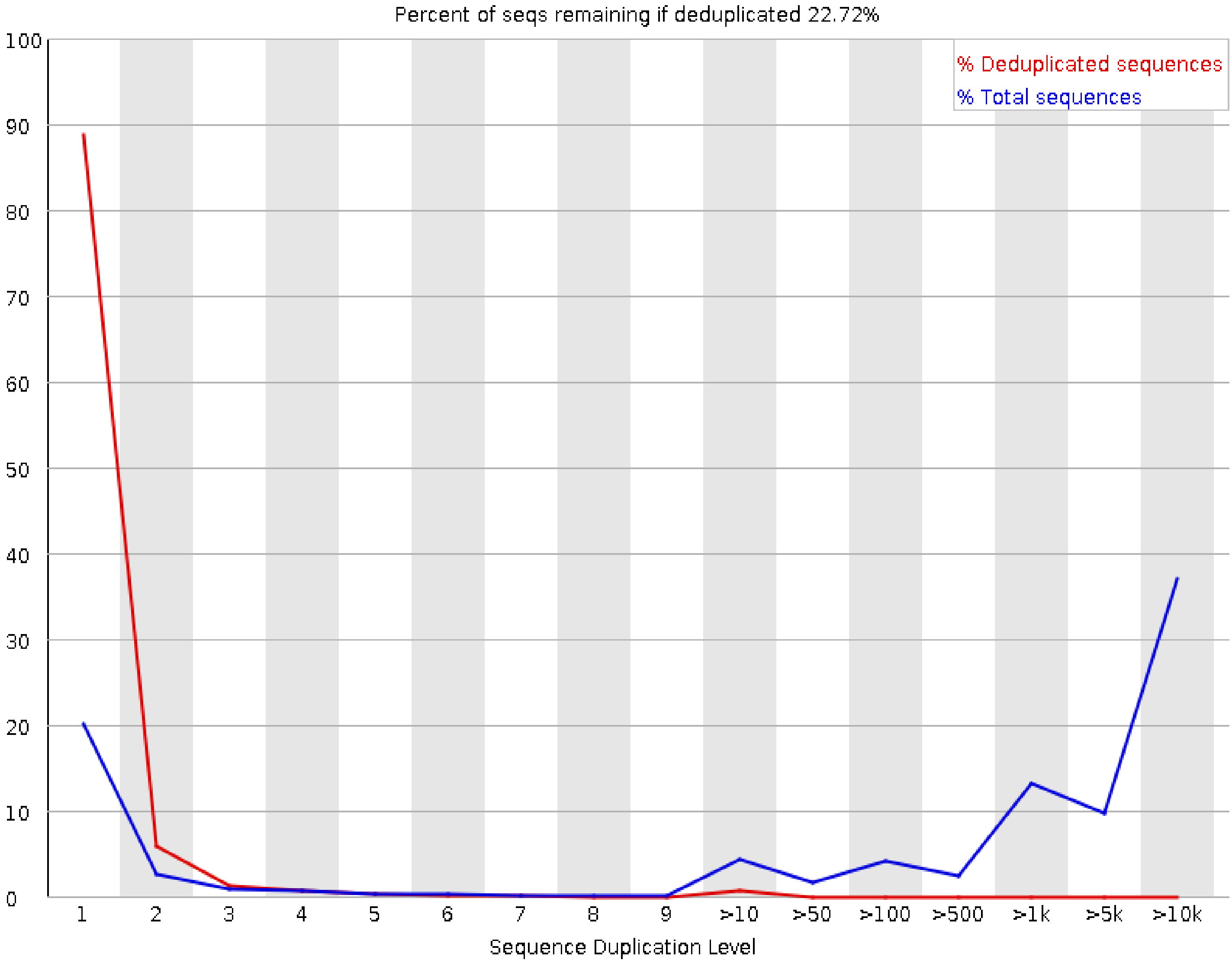


## Overrepresented sequences

| Sequence | Count | Percentage | Possible Source |
|---|---|---|---|
| CTTCCGTACGCCACATGTCCCGCGCCCCGCCGCGGGGCGGGGATTCGGCG | 291227 | 0.42434977704322596 | No Hit |
| CCCGTCGGCATGTATTAGCTCTAGAATTACCACAGTTATCCAAGTAGGAG | 270612 | 0.3943114541756825 | No Hit |
| CGCAGTTTTATCCGGTAAAGCGAATGATTAGAGGTCTTGGGGCCGAAACG | 256626 | 0.3739323135680926 | No Hit |
| CGCGTAACTAGTTAGCATGCCAGAGTCTCGTTCGTTATCGGAATTAACCA | 237926 | 0.34668435637075745 | No Hit |
| CCTCACCCGGCCCGGACACGGACAGGATTGACAGATTGATAGCTCTTTCT | 235104 | 0.34257239192097777 | No Hit |
| CTCCCTTTCGATCGGCCGAGGGCAACGGAGGCCATCGCCCGTCCCTTCGG | 195487 | 0.2848460646329122 | No Hit |
| CTCACCCGGCCCGGACACGGACAGGATTGACAGATTGATAGCTCTTTCTC | 191906 | 0.279628153685123 | No Hit |
| CTGCCAGTAGCATATGCTTGTCTCAAAGATTAAGCCATGCATGTCTAAGT | 188694 | 0.27494791633122784 | No Hit |
| CCGGTATTTAGCCTTAGATGGAGTTTACCACCCGCTTTGGGCTGCATTCC | 170182 | 0.24797389581587653 | No Hit |
| CTCTCTTCAAAGTTCTTTTCAACTTTCCCTTACGGTACTTGTTGACTATC | 153110 | 0.22309811371572116 | No Hit |
| CTGGATAGTAGGTAGGGACAGTGGGAATCTCGTTCATCCATTCATGCGCG | 152977 | 0.22290431808431763 | No Hit |

| Sequence | Count | Percentage | Possible Source |
|---|---|---|---|
| CTTGGTTATAATTTTTCATCTTTCCCTTGCGGTACTATATCTATTGCGCC | 142524 | 0.20767314714401044 | No Hi[illegible] |
| CTCCGACTTTCGTTCTTGATTAATGAAAACATTCTTGGCAAATGCTTTCG | 140420 | 0.20460738768180764 | N[illegible] |
| CTTCACCGTGCCAGACTAGAGTCAAGCTCAACAGGGTCTTCTTTCCCCGC | 138253 | 0.20144983028893998 | No Hit |
| CAAAGATTAAGCCATGCATGTCTAAGTACGCACGGCCGGTACAGTGAAAC | 129882 | 0.18925236239060345 | No Hit |
| CTCGCATTCCACGCCCGGCTCCACGCCAGCGAGCCGGGCTTCTTACCCAT | 123965 | 0.18063064245816324 | No Hit |
| CTGCTGTCTATATCAACCAACACCTTTTCTGGGGTCTGATGAGCGTCGGC | 123328 | 0.1797024633814412 | No Hit |
| CGGGTCTTCCGTACGCCACATGTCCCGCGCCCCGCCGCGGGGCGGGGATT | 123148 | 0.1794401835795417 | No Hit |
| CTGGGGTCTGATGAGCGTCGGCATCGGGCGCCTTAACCCGGCGTTCGGTT | 117180 | 0.1707441510365633 | No Hit |
| ATCAGACGTGGCGACCCGCTGAATTTAAGCATATTAGTCAGCGGAGGAGA | 110470 | 0.1609669428657548 | No Hit |
| GTTAATTGTCAGTTCAGTGTTTTAATCTGACGCAGGCTTATGCGGAGGAG | 107982 | 0.15734165315949972 | No Hit |
| CTCTCATGTCTCTTCACCGTGCCAGACTAGAGTCAAGCTCAACAGGGTCT | 107306 | 0.1563566467923661 | No Hit |
| CGACGACCCATTCGAACGTCTGCCCTATCAACTTTCGATGGTAGTCGCCG | 105663 | 0.15396261504502806 | No Hit |
| CTGATGAGCGTCGGCATCGGGCGCCTTAACCCGGCGTTCGGTTCATCCCG | 104655 | 0.15249384815439096 | No Hit |
| CTTTAAATGGGTAAGAAGCCCGGCTCGCTGGCGTGGAGCCGGGCGTGGAA | 103818 | 0.15127424707555834 | No Hit |
| CGAAGGCCCGCGGCGGGTGTTGACGCGATGTGATTTCTGCCCAGTGCTCT | 100159 | 0.1459426815469461 | No Hit |
| CGCGTCACTAATTAGATGACGAGGCATTTGGCTACCTTAAGAGAGTCATA | 99088 | 0.14438211672564416 | No Hit |
| CCCGAAGTTACGGATCCGGCTTGCCGACTTCCCTTACCTACATTGTTCCA | 97900 | 0.1426510700331076 | No Hit |
| CTCCCACTTATTCTACACCTCTCATGTCTCTTCACCGTGCCAGACTAGAG | 96646 | 0.14082385407987452 | No Hit |
| CTCCGCCACTCCGGATTCGGGGATCTGAACCCGACTCCCTTTCGATCGGC | 96329 | 0.14036195020652933 | No Hit |
| CTCGATCAGAAGGACTTGGGCCCCCCACGAGCGGCGCCGGGGAGCGGGTC | 92430 | 0.134680678275384 | No Hit |
| CTTCCGTCAATTCCTTTAAGTTTCAGCTTTGCAACCATACTCCCCCCGGA | 91248 | 0.13295837424291113 | No Hit |
| CTTGTCTCAAAGATTAAGCCATGCATGTCTAAGTACGCACGGCCGGTACA | 90633 | 0.13206225158642124 | No Hit |
| CAAACTTTAAATGGGTAAGAAGCCCGGCTCGCTGGCGTGGAGCCGGGCGT | 89431 | 0.1303108053537369 | No Hit |
| GCCCTCTTGAACTCTCTCTTCAAAGTTCTTTTCAACTTTCCCTTACGGTA | 88197 | 0.1285127316007149 | No Hit |
| CTCGTTCGTTATCGGAATTAACCAGACAAATCGCTCCACCAACTAAGAAC | 85730 | 0.12491804120468143 | No Hit |
| GTCAAAGTGAAGAAATTCAATGAAGCGCGGGTAAACGGCGGGAGTAACTA | 85362 | 0.12438182472079805 | No Hit |
| CCACTCTCGACTGCCGGCGACGGCCGGGTATGGGCCCGACGCTCCAGCGC | 85238 | 0.12420114307948951 | No Hit |
| CTGCTGCCTTCCTTGGATGTGGTAGCCGTTTCTCAGGCTCCCTCTCCGGA | 84523 | 0.12315930942194435 | No Hit |
| CCGTCGGCATGTATTAGCTCTAGAATTACCACAGTTATCCAAGTAGGAGA | 84243 | 0.122751318618989 | No Hit |
| CTCCCGTCCACTCTCGACTGCCGGCGACGGCCGGGTATGGGCCCGACGCT | 84189 | 0.12267263467841975 | No Hit |
| CCCACTTATTCTACACCTCTCATGTCTCTTCACCGTGCCAGACTAGAGTC | 82911 | 0.12081044808493344 | No Hit |
| CTTGAACTCTCTCTTCAAAGTTCTTTTCAACTTTCCCTTACGGTACTTGT | 82375 | 0.12002943711927717 | No Hit |
| CTACTATCCAGCGAAACCACAGCCAAGGGAACGGGCTTGGCGGAATCAGC | 81614 | 0.11892057640124659 | No Hit |
| CTGTATTGTTATTTTTCGTCACTACCTCCCCGGGTCGGGAGTGGGTAATT | 80704 | 0.11759460629164366 | No Hit |
| CGAGAACTTTGAAGGCCGAAGTGGAGAAGGGTTCCATGTGAACAGCAGTT | 80140 | 0.11677279624569194 | No Hit |
| CTACCTTAAGAGAGTCATAGTTACTCCCGCCGTTTACCCGCGCTTCATTG | 79987 | 0.11654985841407738 | No Hit |
| CGCGATGTGATTTCTGCCCAGTGCTCTGAATGTCAAAGTGAAGAAATTCA | 78656 | 0.11461044499003177 | No Hit |
| GTCGGCATGTATTAGCTCTAGAATTACCACAGTTATCCAAGTAGGAGAGG | 78635 | 0.11457984567981017 | No Hit |
| CACCGTGCCAGACTAGAGTCAAGCTCAACAGGGTCTTCTTTCCCCGCTGA | 78422 | 0.11426948124756243 | No Hit |

| Sequence | Count | Percentage | Possible Source |
|---|---|---|---|
| CTGAATTTAAGCATATTAGTCAGCGGAGGAGAAGAAACTAACCAGGATTC | 77849 | 0.11343455721151574 | No Hit |
| GTCGGGTCTGCGAGAGCGCC[illegible]GAGGGAAACTTCGGAGGGAAC | [illegible] | 0.112[illegible]6403 | No Hit |
| CTGCTTACCAAAAGTGGCCCACTAGGCACTCGCATTCCACGCCCGGCTCC | 74878 | 0.10910548337016374 | No Hit |
| CCCAGGCATAGTTCACCATCTTTCGGGTCCTAACACGTGCGCTCGTGCTC | 74192 | 0.1081059059029246 | No Hit |
| CTCCGAGGTCGCCCCAACCGAAATTTTTAATGCAGGTTTGGTAGTTTAGG | 74010 | 0.10784071188100401 | No Hit |
| CTTATTTCTCTTGTCCTTTCGTACAGGGAGGAATTTGAAGTAGATAGAAA | 73564 | 0.10719084081629751 | No Hit |
| AAACGATCTCAACCTATTCTCAAACTTTAAATGGGTAAGAAGCCCGGCTC | 71961 | 0.10485509346938154 | No Hit |
| CTCAAACTTTAAATGGGTAAGAAGCCCGGCTCGCTGGCGTGGAGCCGGGC | 69893 | 0.10184178996755862 | No Hit |
| CTGTGGTTTCGCTGGATAGTAGGTAGGGACAGTGGGAATCTCGTTCATCC | 69790 | 0.10169170763647169 | No Hit |
| CACCCGTTTACCTCTTAACGGTTTCACGCCCTCTTGAACTCTCTCTTCAA | 69484 | 0.10124583197324256 | No Hit |
| CCTGCCAGTAGCATATGCTTGTCTCAAAGATTAAGCCATGCATGTCTAAG | 69373 | 0.10108409276207121 | No Hit |

## Adapter Content

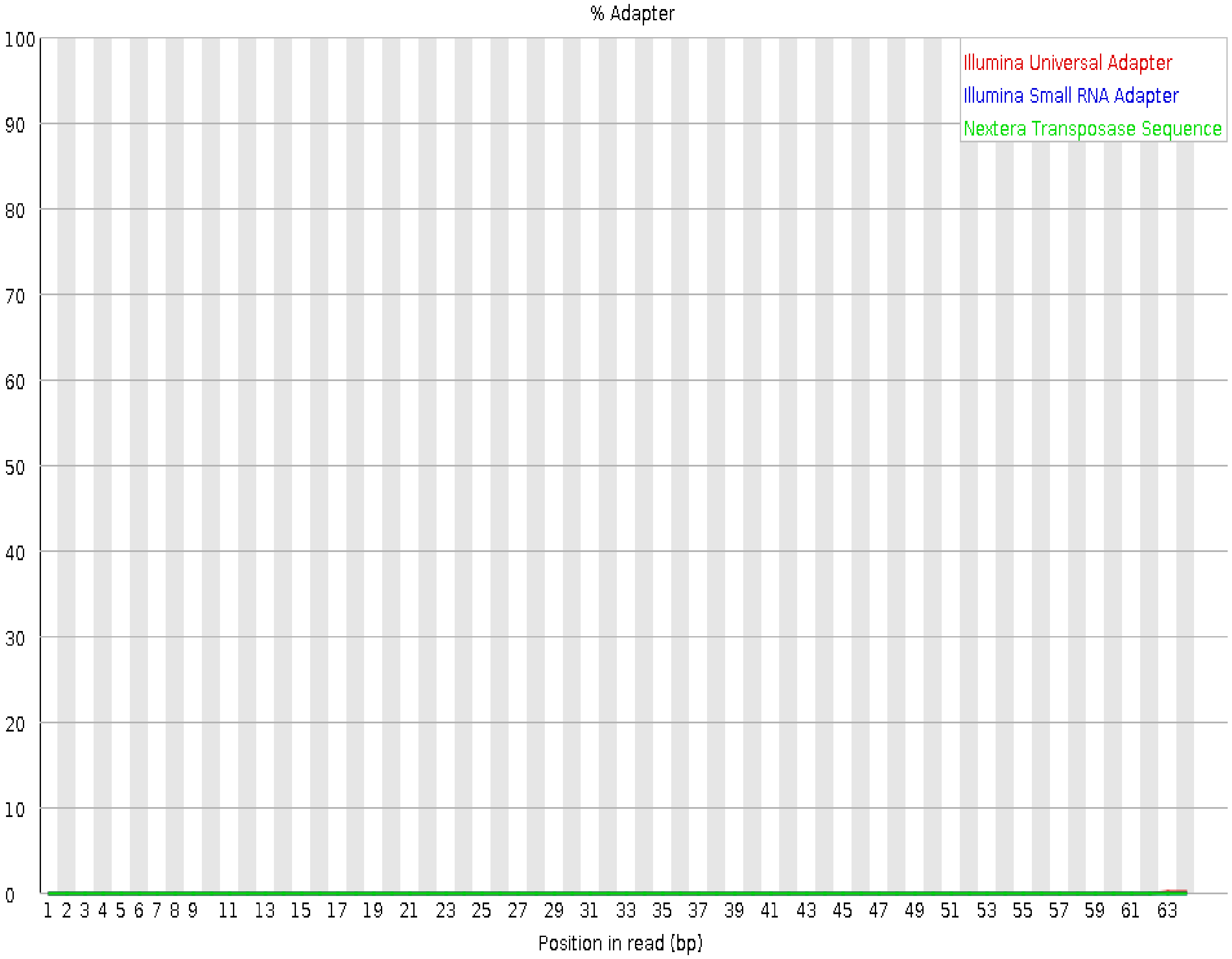

## Kmer Content

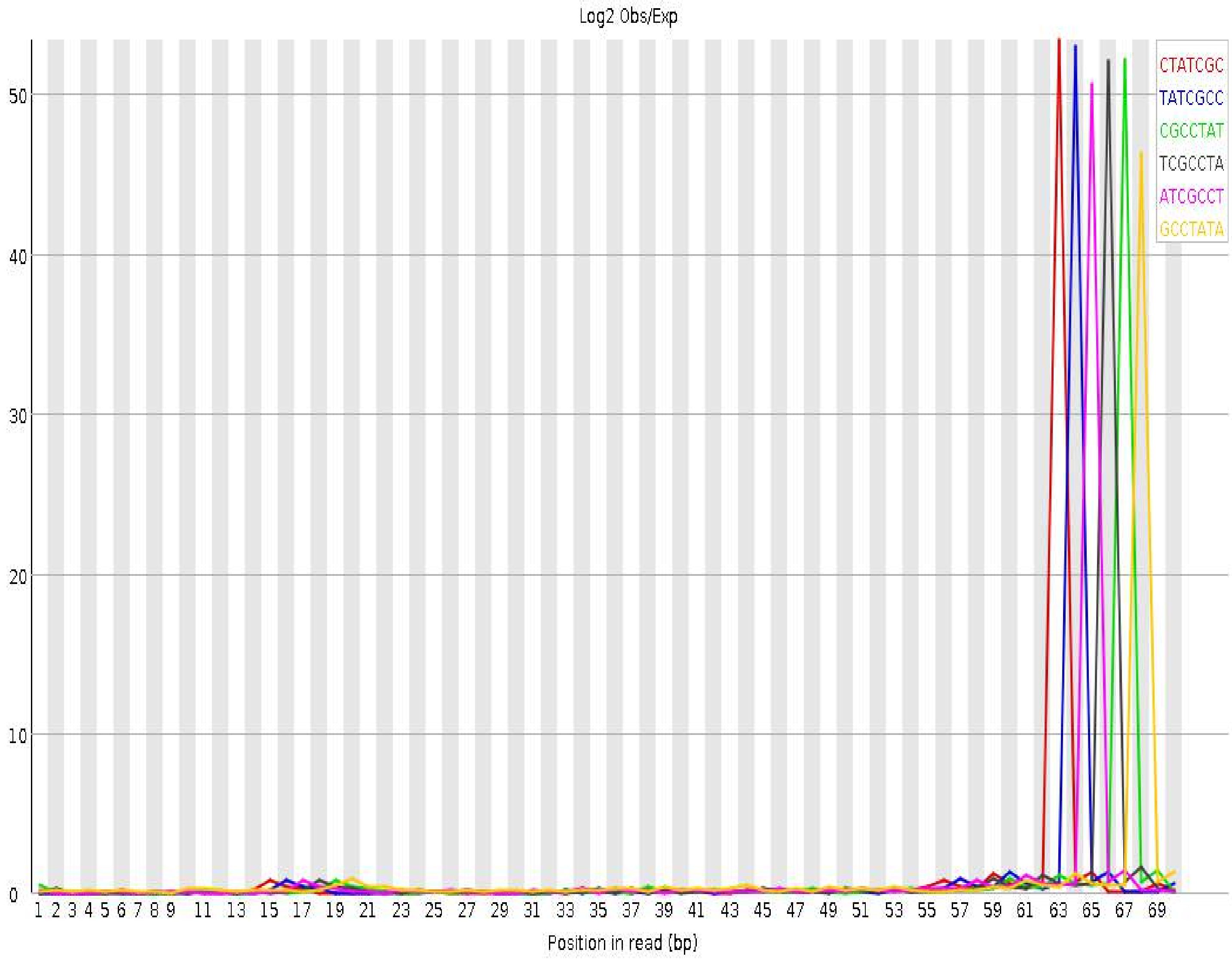


| Sequence | Count | PValue | Obs/Exp Max | Max Obs/Exp Position |
|---|---|---|---|---|
| CTATCGC | 21415 | 0.0 | 53.346058 | 63 |
| TATCGCC | 21575 | 0.0 | 53.01408 | 64 |
| CGCCTAT | 21930 | 0.0 | 52.16137 | 67 |
| TCGCCTA | 21965 | 0.0 | 52.093346 | 66 |
| ATCGCCT | 22590 | 0.0 | 50.64986 | 65 |
| GCCTATA | 24880 | 0.0 | 46.290676 | 68 |
| ACTCGCC | 51300 | 0.0 | 46.163322 | 69 |
| TCACTCG | 53780 | 0.0 | 44.19911 | 67 |
| CGCGTAA | 47250 | 0.0 | 42.92743 | 1 |
| TCAGACG | 29485 | 0.0 | 42.12031 | 2 |
| GCGTAAC | 51200 | 0.0 | 39.816265 | 2 |
| TGCCGTA | 11030 | 0.0 | 38.62106 | 2 |

| Sequence | Count | PValue | Obs/Exp Max | Max Obs/Exp Position |
|---|---|---|---|---|
| CCCGTCG | 59035 | 0.0 | 38.189373 | 1 |
| TCTATCG | 30305 | 0.0 | [illegible] | 62 |
| CTCGCCG | 68900 | 0.0 | 37.42851 | 70 |
| TTCACTC | 63860 | 0.0 | 37.145176 | 66 |
| CGTAACT | 55355 | 0.0 | 36.746403 | 3 |
| CGGAACG | 41405 | 0.0 | 36.366703 | 48 |
| AGTTAGC | 54005 | 0.0 | 35.889874 | 10 |
| ACGGCGC | 42840 | 0.0 | 35.8238 | 52 |

**Produced by FastQC (version 0.11.2)**

# FastQC Report

Mon 4 May 2015
Biochain_Adult_Colon_TGACCA_L007_R2.fastq.gz

## Summary


- Basic Statistics
- Per base sequence quality
- Per tile sequence quality
- Per sequence quality scores
- Per base sequence content
- Per sequence GC content
- Per base N content
- Sequence Length Distribution
- Sequence Duplication Levels
- Overrepresented sequences
- Adapter Content
- Kmer Content


## Basic Statistics

| Measure | Value |
|---|---|
| Filename | Biochain_Adult_Colon_TGACCA_L007_R2.fastq.gz |
| File type | Conventional base calls |
| Encoding | Sanger / Illumina 1.9 |
| Total Sequences | 68628998 |
| Sequences flagged as poor quality | 0 |
| Sequence length | 76 |
| %GC | 52 |

## Per base sequence quality

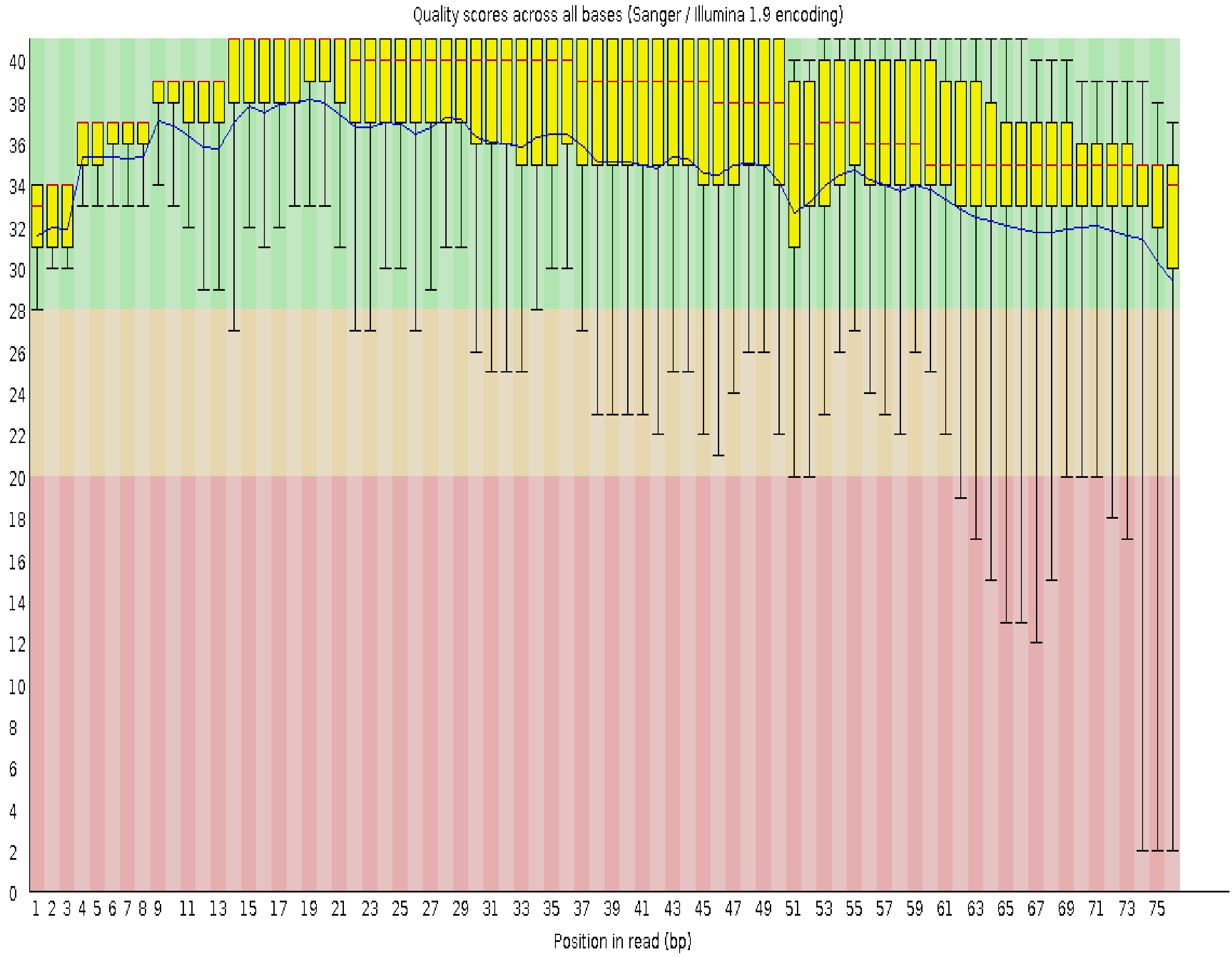


## Per tile sequence quality

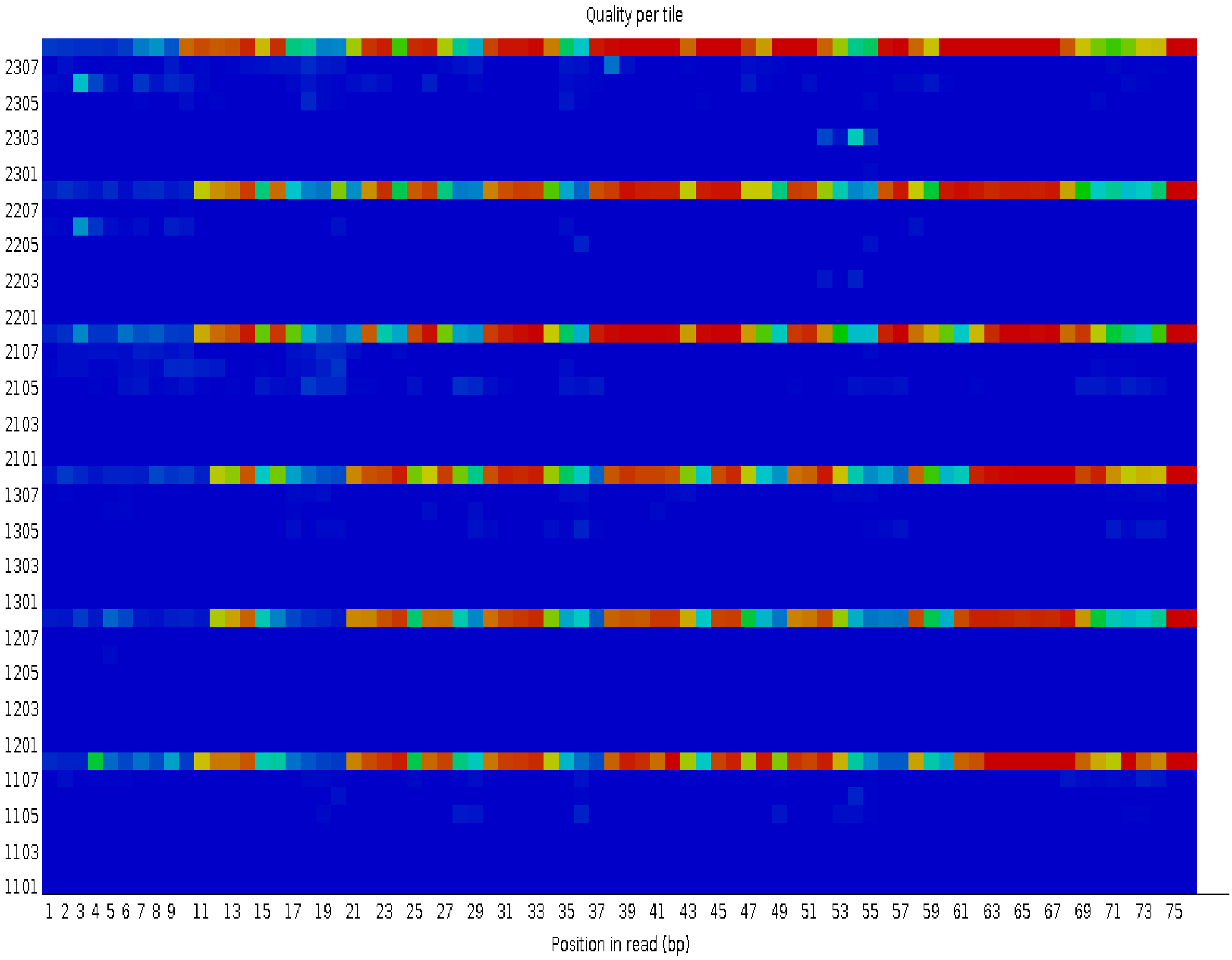


## Per sequence quality scores

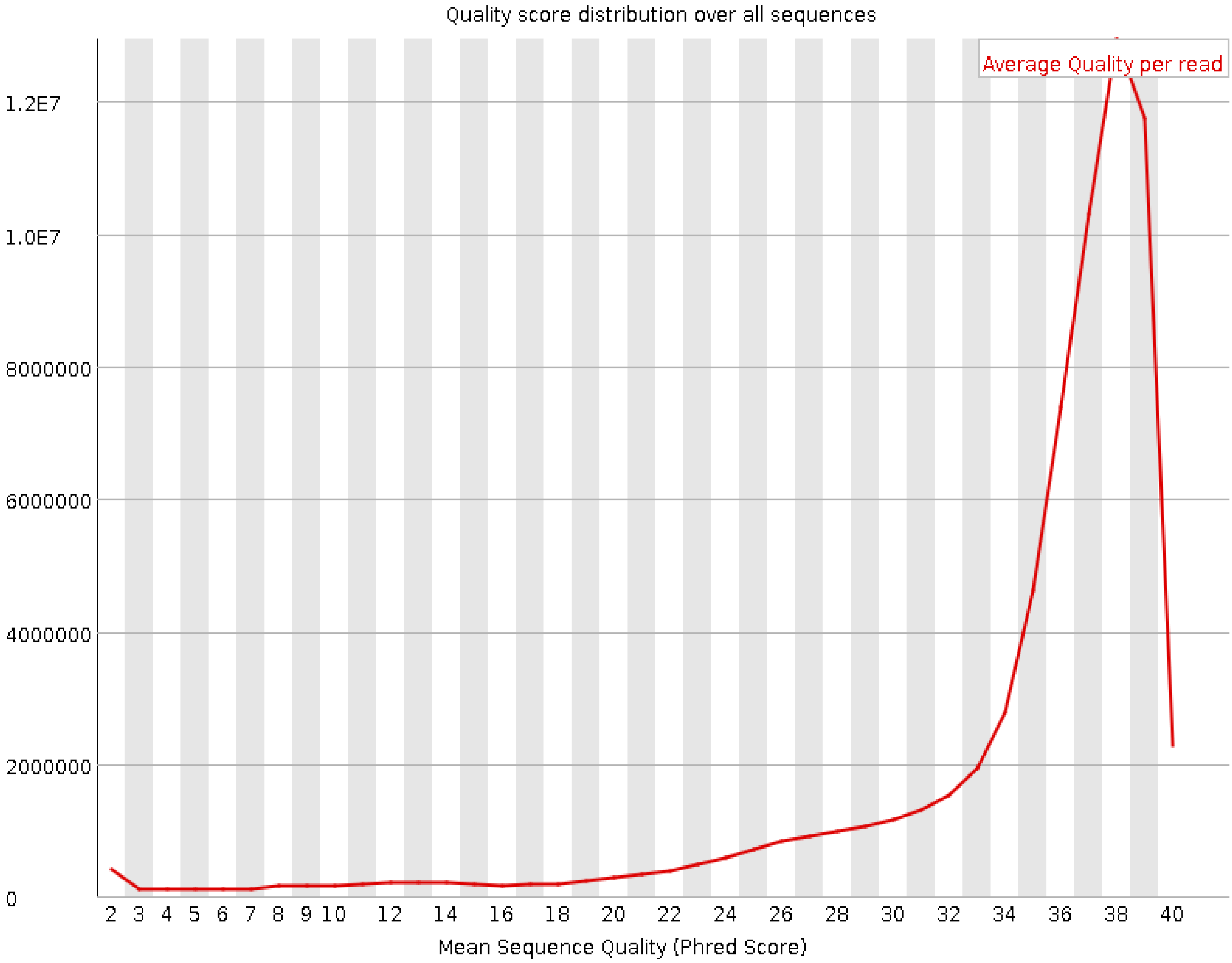


## Per base sequence content

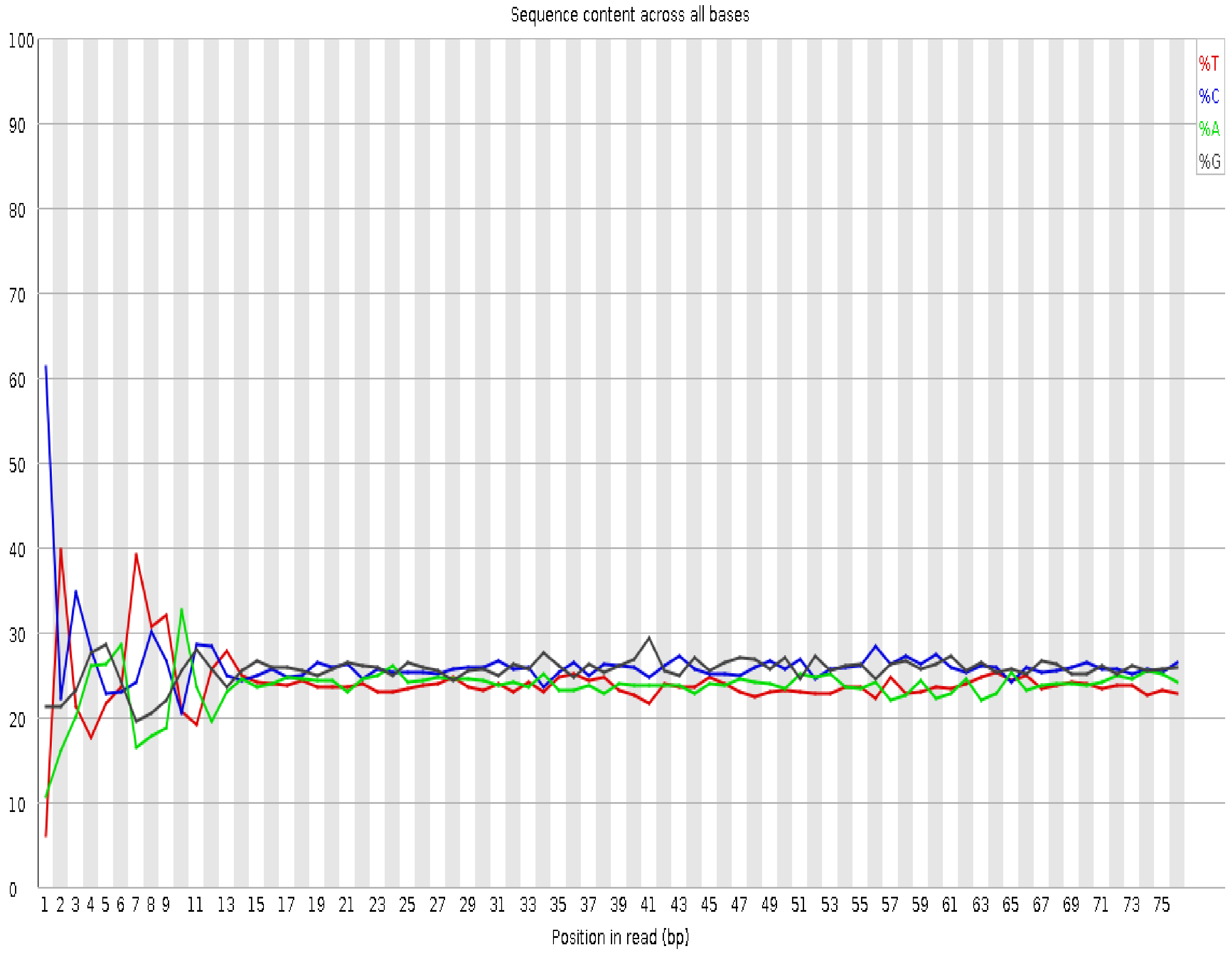


## Per sequence GC content

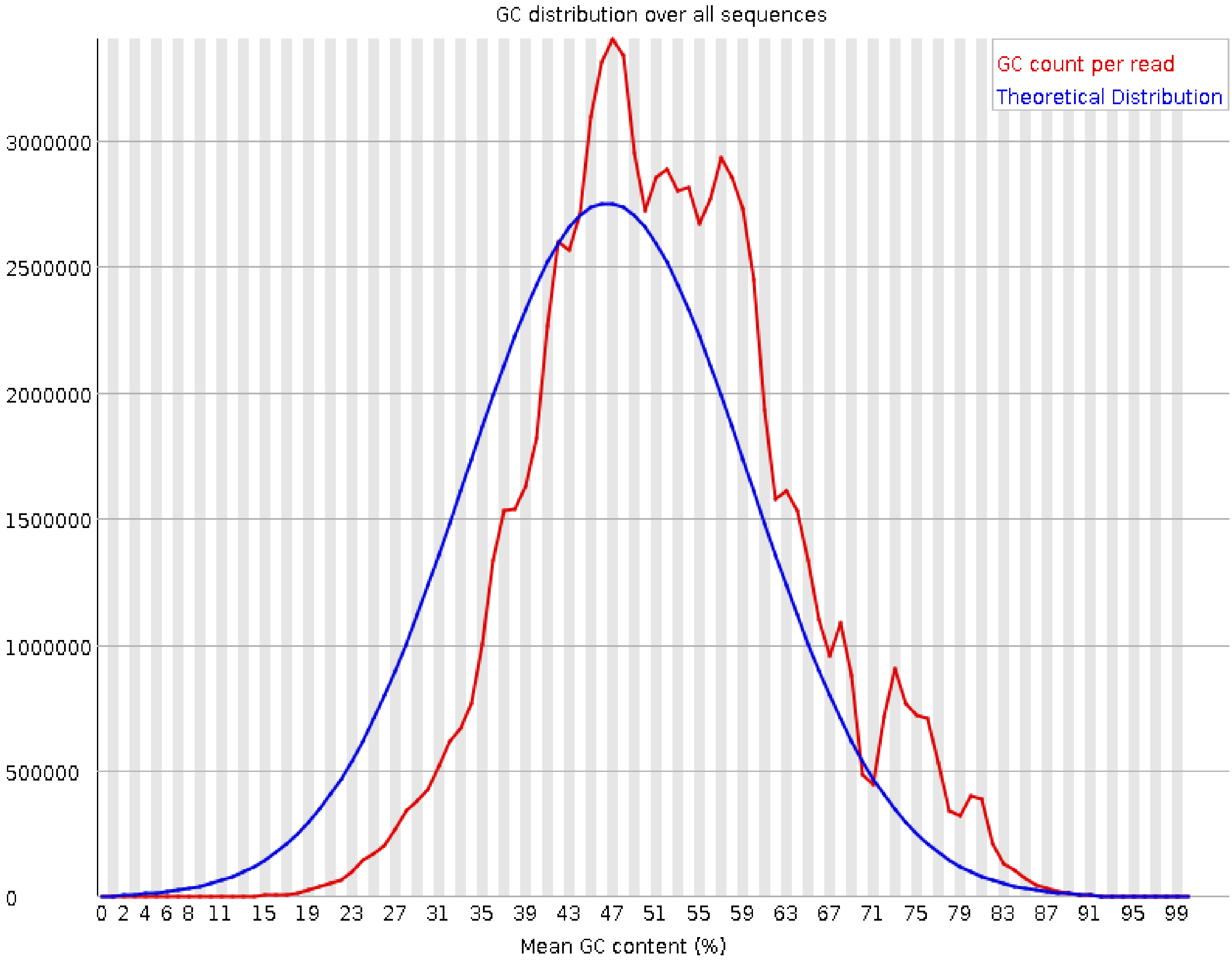


## Per base N content

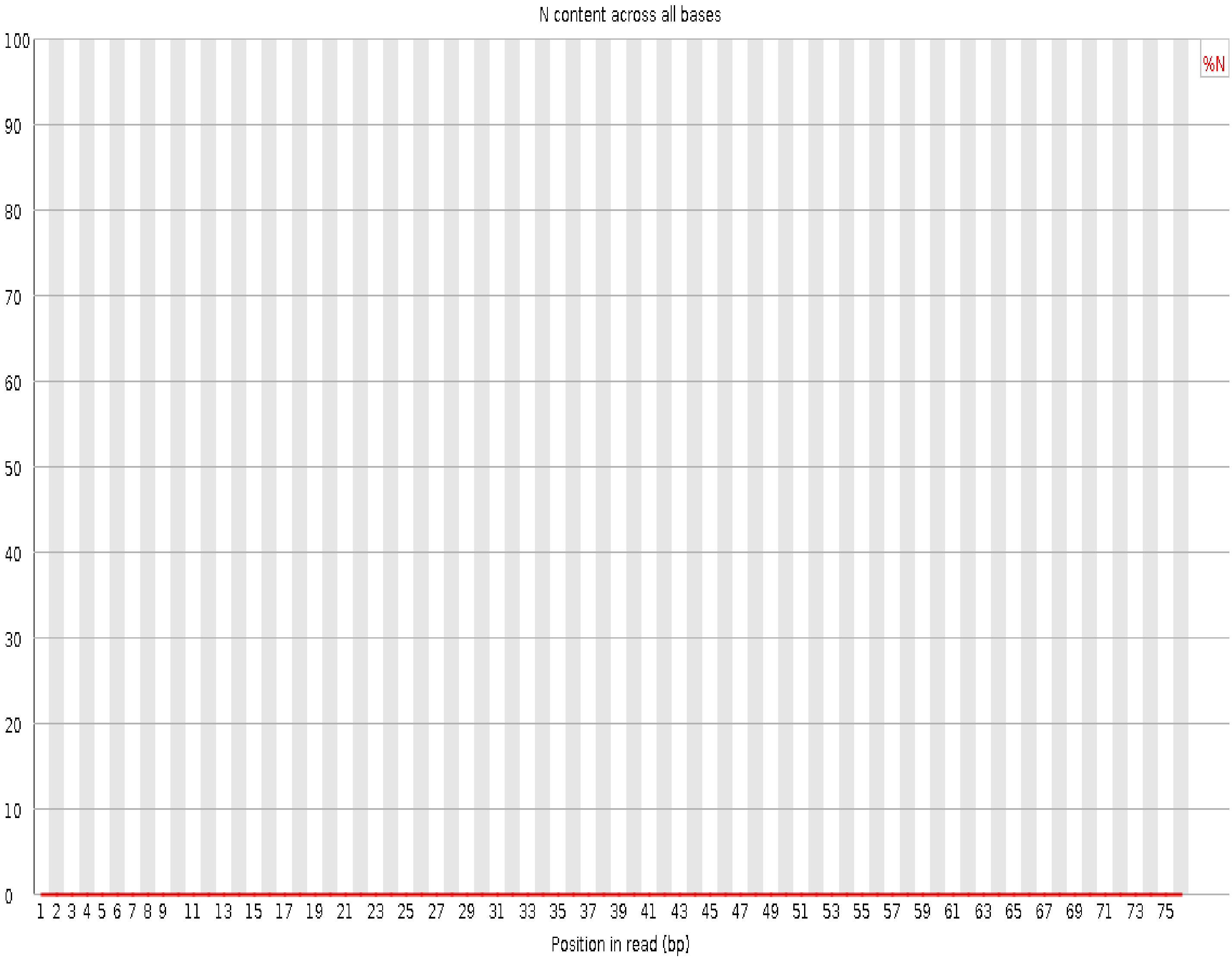


## Sequence Length Distribution

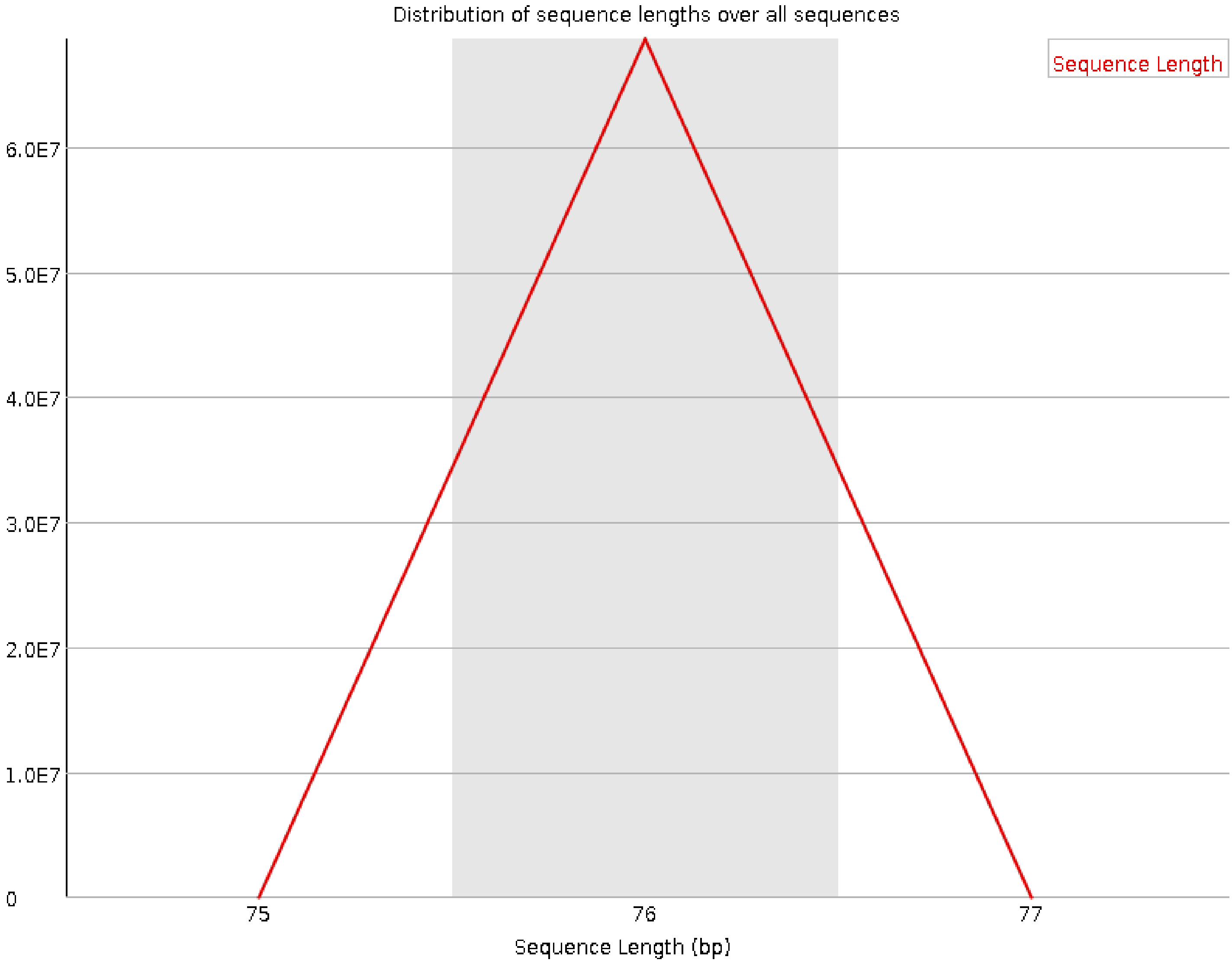


## Sequence Duplication Levels

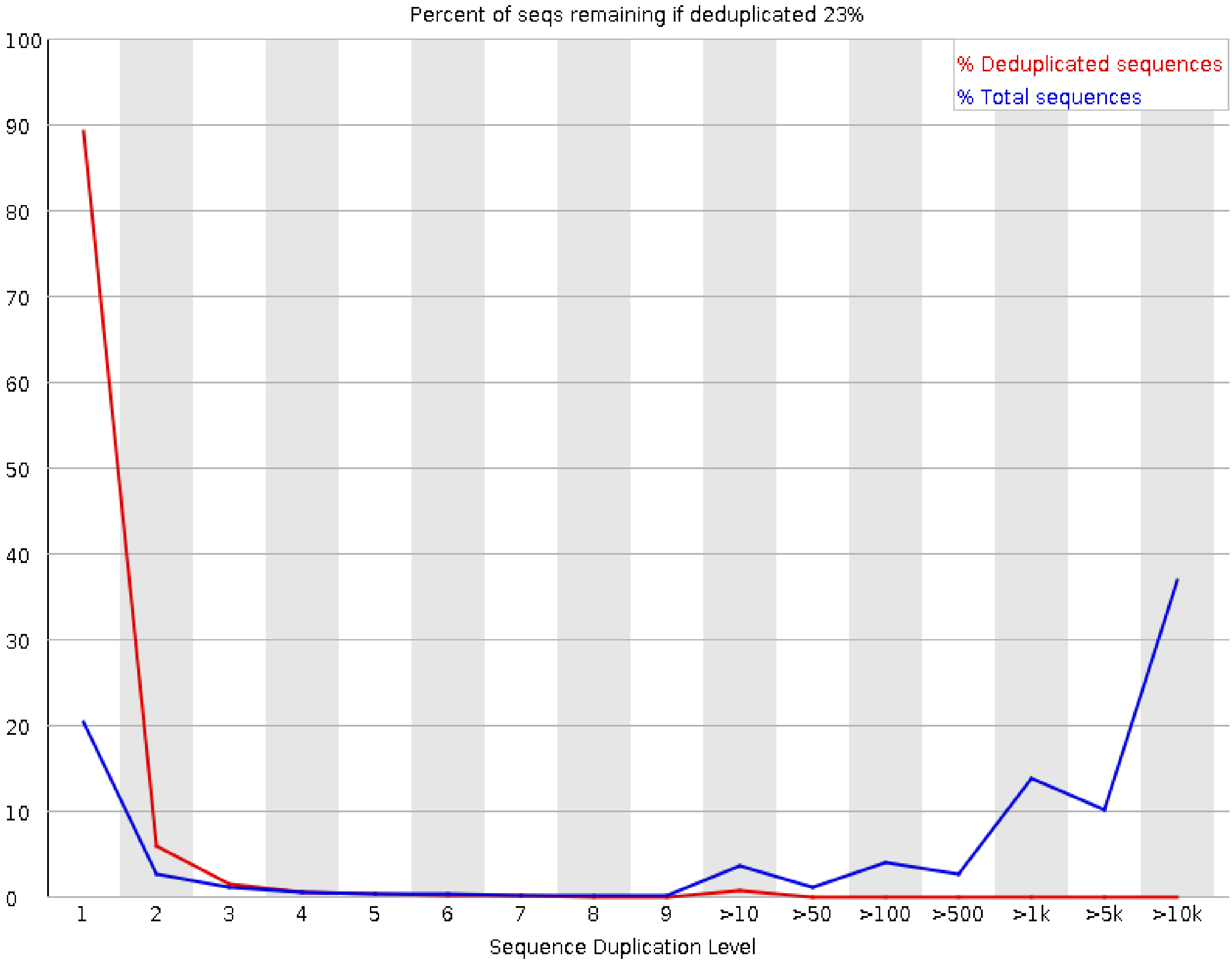


## Overrepresented sequences

| Sequence | Count | Percentage | Possible Source |
|---|---|---|---|
| CTTCCGTACGCCACATGTCCCGCGCCCCGCCGCGGGGCGGGGATTCGGCG | 286505 | 0.4174693035733962 | No Hit |
| CCCGTCGGCATGTATTAGCTCTAGAATTACCACAGTTATCCAAGTAGGAG | 265731 | 0.38719930021417476 | No Hit |
| CGCAGTTTTATCCGGTAAAGCGAATGATTAGAGGTCTTGGGGCCGAAACG | 260832 | 0.3800609182724772 | No Hit |
| CCTCACCCGGCCCGGACACGGACAGGATTGACAGATTGATAGCTCTTTCT | 236210 | 0.344183955592649 | No Hit |
| CGCGTAACTAGTTAGCATGCCAGAGTCTCGTTCGTTATCGGAATTAACCA | 228836 | 0.33343922637483353 | No Hit |
| CTCCCTTTCGATCGGCCGAGGGCAACGGAGGCCATCGCCCGTCCCTTCGG | 206672 | 0.3011438401009439 | No Hit |
| CTGCCAGTAGCATATGCTTGTCTCAAAGATTAAGCCATGCATGTCTAAGT | 199477 | 0.2906599335750174 | No Hit |
| CTCACCCGGCCCGGACACGGACAGGATTGACAGATTGATAGCTCTTTCTC | 195928 | 0.2854886501475659 | No Hit |
| CCGGTATTTAGCCTTAGATGGAGTTTACCACCCGCTTTGGGCTGCATTCC | 167741 | 0.24441709028011743 | No Hit |
| CTCCGACTTTCGTTCTTGATTAATGAAAACATTCTTGGCAAATGCTTTCG | 157069 | 0.22886681224749922 | No Hit |
| CTGGATAGTAGGTAGGGACAGTGGAATCTCGTTCATCCATTCATGCGCG | 154580 | 0.2252400654312336 | No Hit |

| Sequence | Count | Percentage | Possible Source |
|---|---|---|---|
| CTCTCTTCAAAGTTCTTTTCAACTTTCCCTTACGGTACTTGTTGACTATC | 145610 | 0.21216978863657604 | No Hit |
| CTTCACCGTGCCAGACTAGAGTCAAGCTCAACAGGGTCTTCTTTCCCCGC | 145052 | 0.21135672125068763 | [illegible] |
| CGGGTCTTCCGTACGCCACATGTCCCGCGCCCCGCCGCGGGGCGGGGATT | 142375 | 0.20745603775243812 | No Hit |
| CAAAGATTAAGCCATGCATGTCTAAGTACGCACGGCCGGTACAGTGAAAC | 133840 | 0.19501960381237096 | No Hit |
| CTTGGTTATAATTTTTCATCTTTCCCTTGCGGTACTATATCTATTGCGCC | 127484 | 0.18575821258529812 | No Hit |
| CTGCTGTCTATATCAACCAACACCTTTTCTGGGGTCTGATGAGCGTCGGC | 121488 | 0.17702138096202424 | No Hit |
| CTCGCATTCCACGCCCGGCTCCACGCCAGCGAGCCGGGCTTCTTACCCAT | 121291 | 0.17673433028994537 | No Hit |
| CGACGACCCATTCGAACGTCTGCCCTATCAACTTTCGATGGTAGTCGCCG | 115688 | 0.16857014290081868 | No Hit |
| CTGGGGTCTGATGAGCGTCGGCATCGGGCGCCTTAACCCGGCGTTCGGTT | 112211 | 0.163503771394127 | No Hit |
| CTCTCATGTCTCTTCACCGTGCCAGACTAGAGTCAAGCTCAACAGGGTCT | 110006 | 0.16029084382085834 | No Hit |
| CTCCGCCACTCCGGATTCGGGGATCTGAACCCGACTCCCTTTCGATCGGC | 107570 | 0.15674132383515202 | No Hit |
| CGAAGGCCCGCGGCGGGTGTTGACGCGATGTGATTTCTGCCCAGTGCTCT | 103510 | 0.15082545719230814 | No Hit |
| CGCGTCACTAATTAGATGACGAGGCATTTGGCTACCTTAAGAGAGTCATA | 101799 | 0.1483323419642525 | No Hit |
| CTGATGAGCGTCGGCATCGGGCGCCTTAACCCGGCGTTCGGTTCATCCCG | 98863 | 0.1440542669732698 | No Hit |
| ATCAGACGTGGCGACCCGCTGAATTTAAGCATATTAGTCAGCGGAGGAGA | 97870 | 0.142607356732791 | No Hit |
| CTCCCACTTATTCTACACCTCTCATGTCTCTTCACCGTGCCAGACTAGAG | 96940 | 0.14125224442297699 | No Hit |
| CTCGATCAGAAGGACTTGGGCCCCCCACGAGCGGCGCCGGGGAGCGGGTC | 93654 | 0.1364641809283009 | No Hit |
| CCCGAAGTTACGGATCCGGCTTGCCGACTTCCCTTACCTACATTGTTCCA | 91380 | 0.1331507127643041 | No Hit |
| CTTTAAATGGGTAAGAAGCCCGGCTCGCTGGCGTGGAGCCGGGCGTGGAA | 89990 | 0.13112532984963585 | No Hit |
| GCCCTCTTGAACTCTCTCTTCAAAGTTCTTTTCAACTTTCCCTTACGGTA | 88999 | 0.12968133382917815 | No Hit |
| CTCCCGTCCACTCTCGACTGCCGGCGACGGCCGGGTATGGGCCCGACGCT | 88371 | 0.1287662687425511 | No Hit |
| CTTCCGTCAATTCCTTTAAGTTTCAGCTTTGCAACCATACTCCCCCCGGA | 87964 | 0.12817322496825614 | No Hit |
| CTTGTCTCAAAGATTAAGCCATGCATGTCTAAGTACGCACGGCCGGTACA | 87115 | 0.1269361385692969 | No Hit |
| CAAACTTTAAATGGGTAAGAAGCCCGGCTCGCTGGCGTGGAGCCGGGCGT | 86446 | 0.12596133197223716 | No Hit |
| GTTAATTGTCAGTTCAGTGTTTTAATCTGACGCAGGCTTATGCGGAGGAG | 84704 | 0.12342304633385437 | No Hit |
| CCACTCTCGACTGCCGGCGACGGCCGGGTATGGGCCCGACGCTCCAGCGC | 83926 | 0.12228941474564439 | No Hit |
| CACCGTGCCAGACTAGAGTCAAGCTCAACAGGGTCTTCTTTCCCCGCTGA | 82408 | 0.12007752174962544 | No Hit |
| CGCGATGTGATTTCTGCCCAGTGCTCTGAATGTCAAAGTGAAGAAATTCA | 82398 | 0.1200629506495199 | No Hit |
| CCGTCGGCATGTATTAGCTCTAGAATTACCACAGTTATCCAAGTAGGAGA | 81071 | 0.11812936566551649 | No Hit |
| CTACCTTAAGAGAGTCATAGTTACTCCCGCCGTTTACCCGCGCTTCATTG | 80647 | 0.11751155102104215 | No Hit |
| CTCGTTCGTTATCGGAATTAACCAGACAAATCGCTCCACCAACTAAGAAC | 80153 | 0.11679173867582912 | No Hit |
| CTCCGAGGTCGCCCCAACCGAAATTTTTAATGCAGGTTTGGTAGTTTAGG | 79888 | 0.11640560452303268 | No Hit |
| CTACTATCCAGCGAAACCACAGCCAAGGGAACGGGCTTGGCGGAATCAGC | 79388 | 0.11567704951775633 | No Hit |
| GTCAAAGTGAAGAAATTCAATGAAGCGCGGGTAAACGGCGGGAGTAACTA | 79199 | 0.1154016557257618 | No Hit |
| CCCACTTATTCTACACCTCTCATGTCTCTTCACCGTGCCAGACTAGAGTC | 78405 | 0.11424471037738304 | No Hit |
| CTTGAACTCTCTCTTCAAAGTTCTTTTCAACTTTCCCTTACGGTACTTGT | 78049 | 0.11372597921362629 | No Hit |
| GTCGGGTCTGCGAGAGCGCCAGCTATCCTGAGGGAAACTTCGGAGGGAAC | 76706 | 0.11176908046945404 | No Hit |
| CTGCTGCCTTCCTTGGATGTGGTAGCCGTTTCTCAGGCTCCCTCTCCGGA | 76060 | 0.11082778740263702 | No Hit |
| CTTATTTCTCTTGTCCTTTCGTACAGGGAGGAATTTGAAGTAGATAGAAA | 75778 | 0.1104168823796614 | No Hit |

| Sequence | Count | Percentage | Possible Source |
|---|---|---|---|
| GTCGGCATGTATTAGCTCTAGAATTACCACAGTTATCCAAGTAGGAGAGG | 74751 | 0.10892043039882354 | No Hit |
| CGAGAACTTTGAAGGCCGAA[illegible]GGTTCCATGTGAACAGCAGTT | [illegible] | 0.10[illegible]9299 | [illegible] |
| CACCCGTTTACCTCTTAACGGTTTCACGCCCTCTTGAACTCTCTCTTCAA | 71759 | 0.10456075724724992 | No Hit |
| CCCAGGCATAGTTCACCATCTTTCGGGTCCTAACACGTGCGCTCGTGCTC | 71312 | 0.10390942907253288 | No Hit |
| CTGTGGTTTCGCTGGATAGTAGGTAGGGACAGTGGGAATCTCGTTCATCC | 70232 | 0.10233575026113598 | No Hit |
| AAACGATCTCAACCTATTCTCAAACTTTAAATGGGTAAGAAGCCCGGCTC | 70207 | 0.10229932251087216 | No Hit |
| CCTGCCAGTAGCATATGCTTGTCTCAAAGATTAAGCCATGCATGTCTAAG | 69774 | 0.10166839387630285 | No Hit |
| CTGCTTACCAAAAGTGGCCCACTAGGCACTCGCATTCCACGCCCGGCTCC | 69313 | 0.10099666616143807 | No Hit |
| CGGGTCTGCGAGAGCGCCAGCTATCCTGAGGGAAACTTCGGAGGGAACCA | 69007 | 0.10055079049820893 | No Hit |
| CTGTATTGTTATTTTTCGTCACTACCTCCCCGGGTCGGGAGTGGGTAATT | 68955 | 0.10047502077766018 | No Hit |
| CTGAATTTAAGCATATTAGTCAGCGGAGGAGAAGAAACTAACCAGGATTC | 68866 | 0.100345337986721 | No Hit |

# Adapter Content

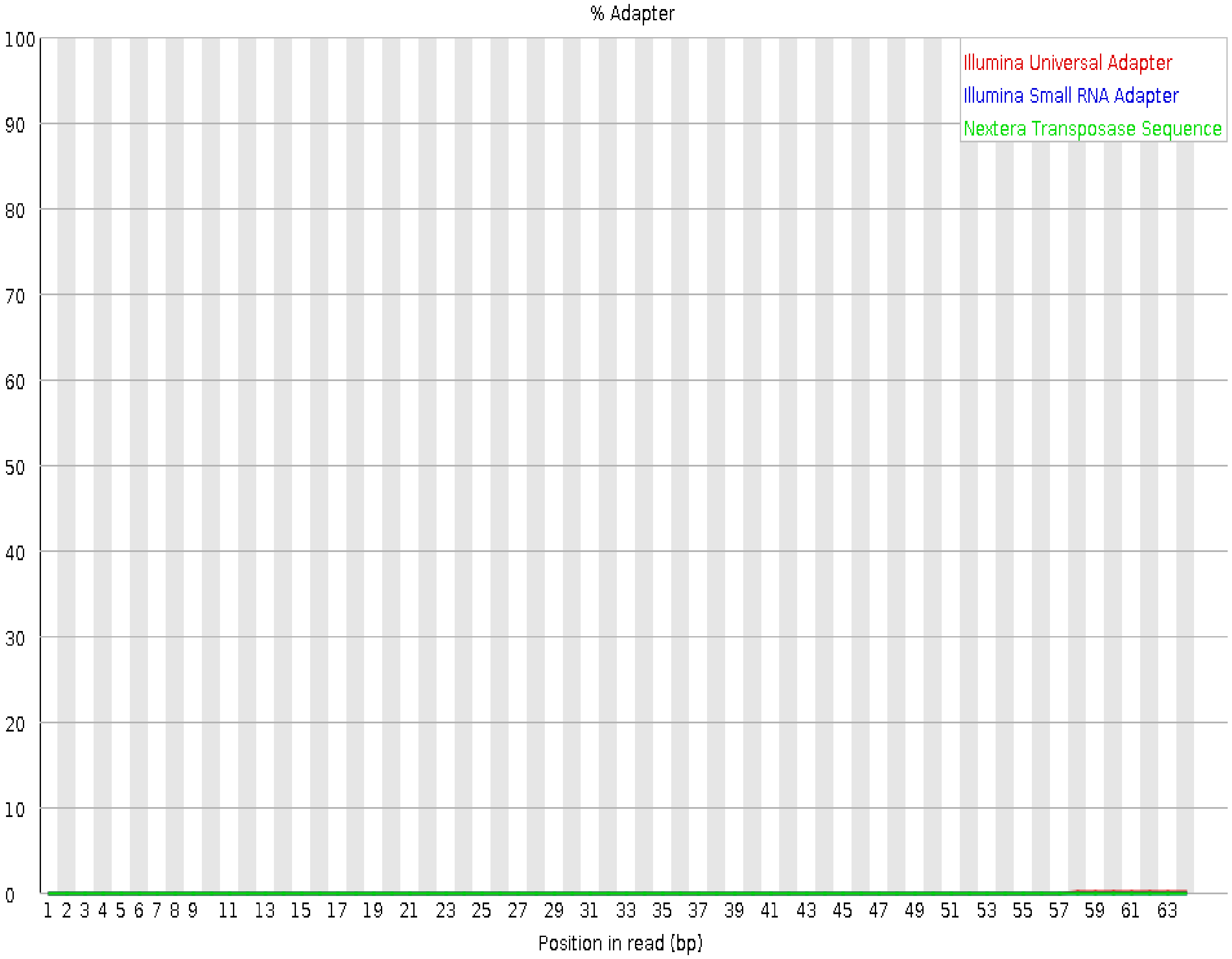

# Kmer Content

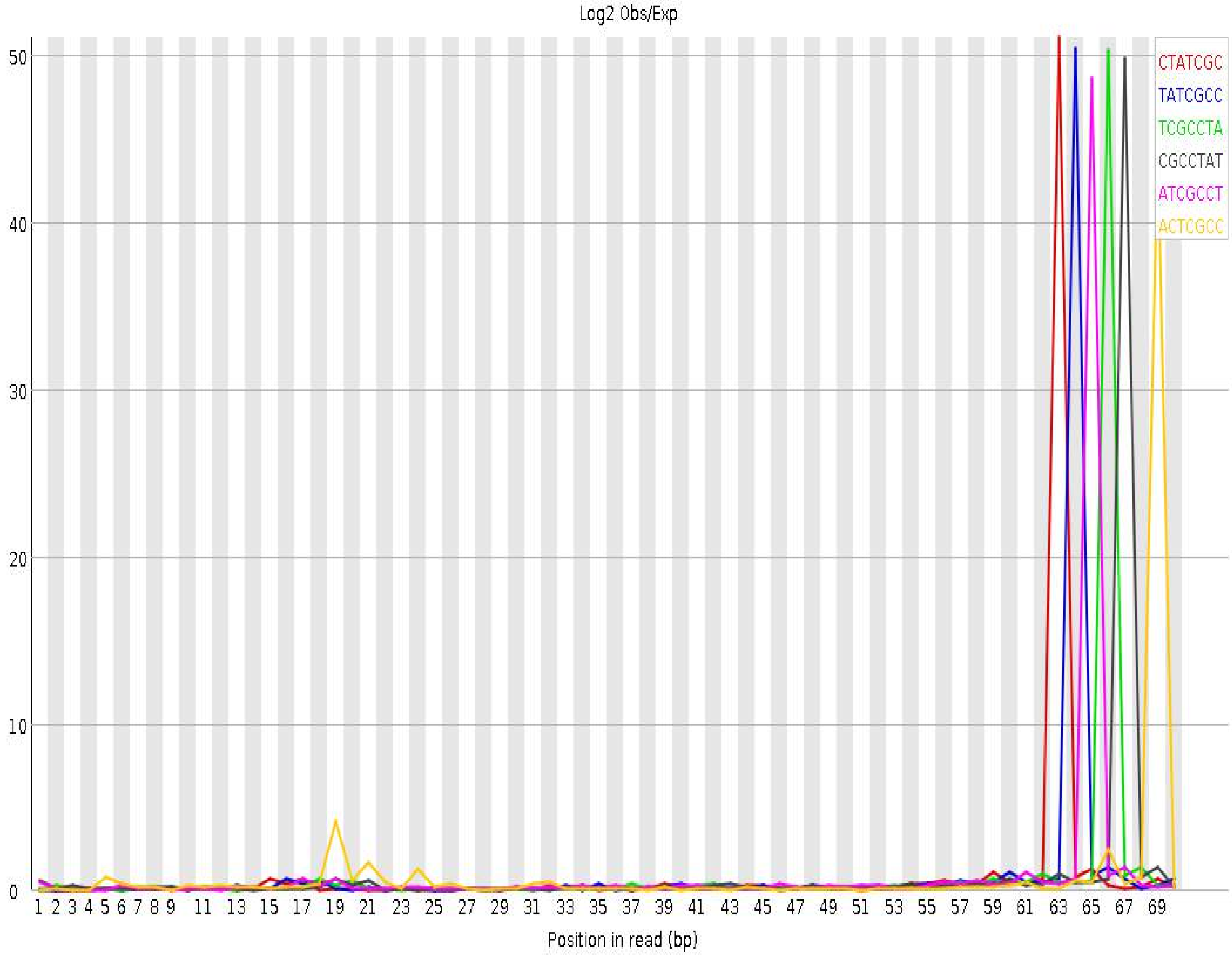


| Sequence | Count | PValue | Obs/Exp Max | Max Obs/Exp Position |
|---|---|---|---|---|
| CTATCGC | 19730 | 0.0 | 51.050205 | 63 |
| TATCGCC | 19955 | 0.0 | 50.412624 | 64 |
| TCGCCTA | 20080 | 0.0 | 50.32729 | 66 |
| CGCCTAT | 20450 | 0.0 | 49.77583 | 67 |
| ATCGCCT | 20690 | 0.0 | 48.68848 | 65 |
| ACTCGCC | 49885 | 0.0 | 44.564186 | 69 |
| GCCTATA | 23670 | 0.0 | 44.495617 | 68 |
| TCACTCG | 51575 | 0.0 | 43.356728 | 67 |
| CGCGTAA | 44730 | 0.0 | 42.148045 | 1 |
| TCAGACG | 28110 | 0.0 | 39.91021 | 2 |
| GCGTAAC | 48330 | 0.0 | 39.48641 | 2 |
| CCCGTCG | 58965 | 0.0 | 38.805176 | 1 |

| Sequence | Count | PValue | Obs/Exp Max | Max Obs/Exp Position |
|---|---|---|---|---|
| TGCCGTA | 11880 | 0.0 | 37.70884 | 2 |
| CGGAACG | 44930 | 0.0 | 37.41[illegible] | 48 |
| ACGGCGC | 46185 | 0.0 | 36.86681 | 52 |
| CGTAACT | 52305 | 0.0 | 36.665726 | 3 |
| GCCAGTA | 43470 | 0.0 | 36.555668 | 3 |
| CTGCCAG | 44580 | 0.0 | 36.280952 | 1 |
| TCTATCG | 28315 | 0.0 | 36.14943 | 62 |
| TTCACTC | 61425 | 0.0 | 36.09093 | 66 |

**Produced by FastQC (version 0.11.2)**

# FastQC Report

Mon 4 May 2015
Biochain_Adult_Heart_CGATGT_L001_R1.fastq.gz

## Summary


- Basic Statistics
- Per base sequence quality
- Per tile sequence quality
- Per sequence quality scores
- Per base sequence content
- Per sequence GC content
- Per base N content
- Sequence Length Distribution
- Sequence Duplication Levels
- Overrepresented sequences
- Adapter Content
- Kmer Content


## Basic Statistics

| Measure | Value |
|---|---|
| Filename | Biochain_Adult_Heart_CGATGT_L001_R1.fastq.gz |
| File type | Conventional base calls |
| Encoding | Sanger / Illumina 1.9 |
| Total Sequences | 75787942 |
| Sequences flagged as poor quality | 0 |
| Sequence length | 76 |
| %GC | 50 |

## Per base sequence quality

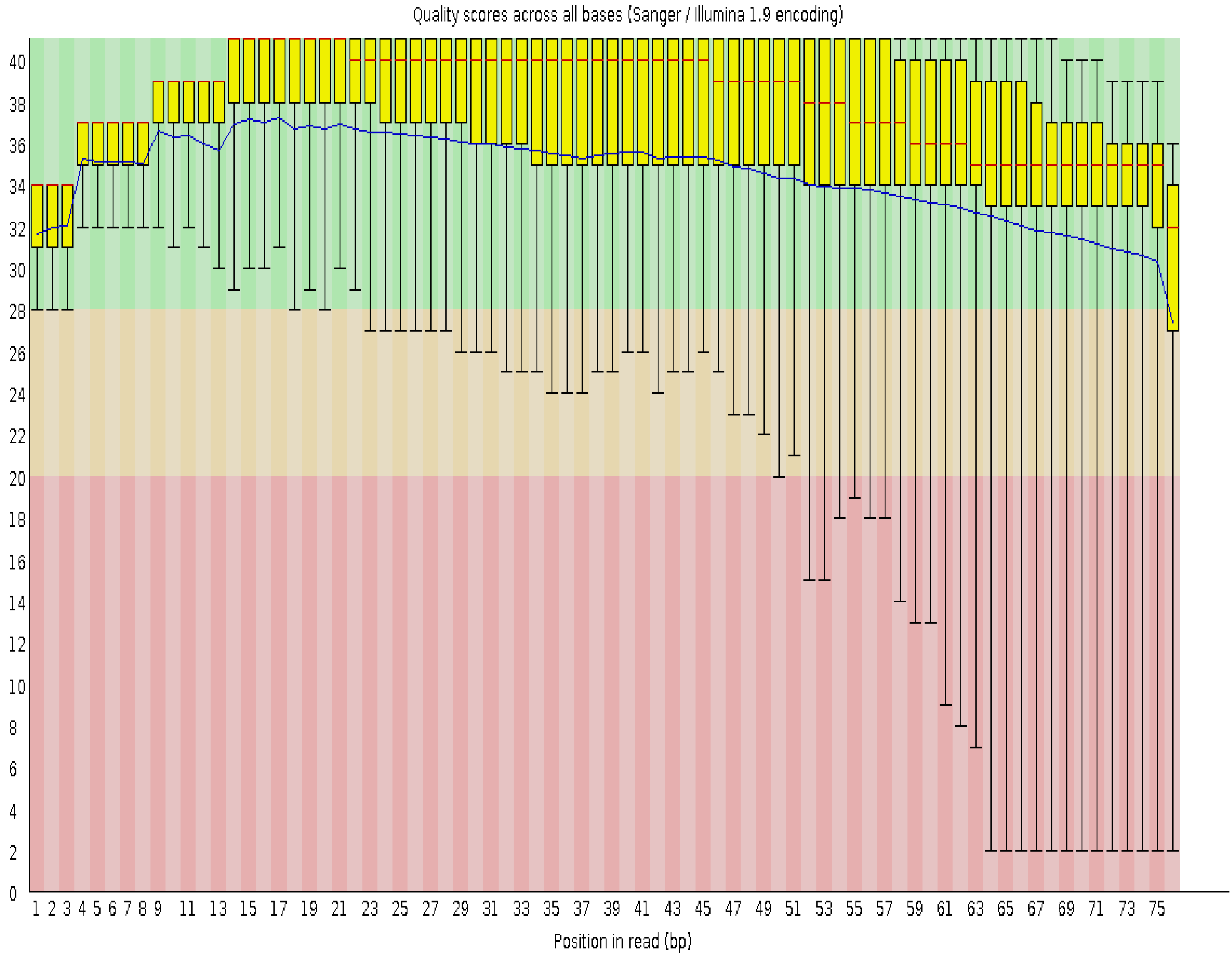


## Per tile sequence quality

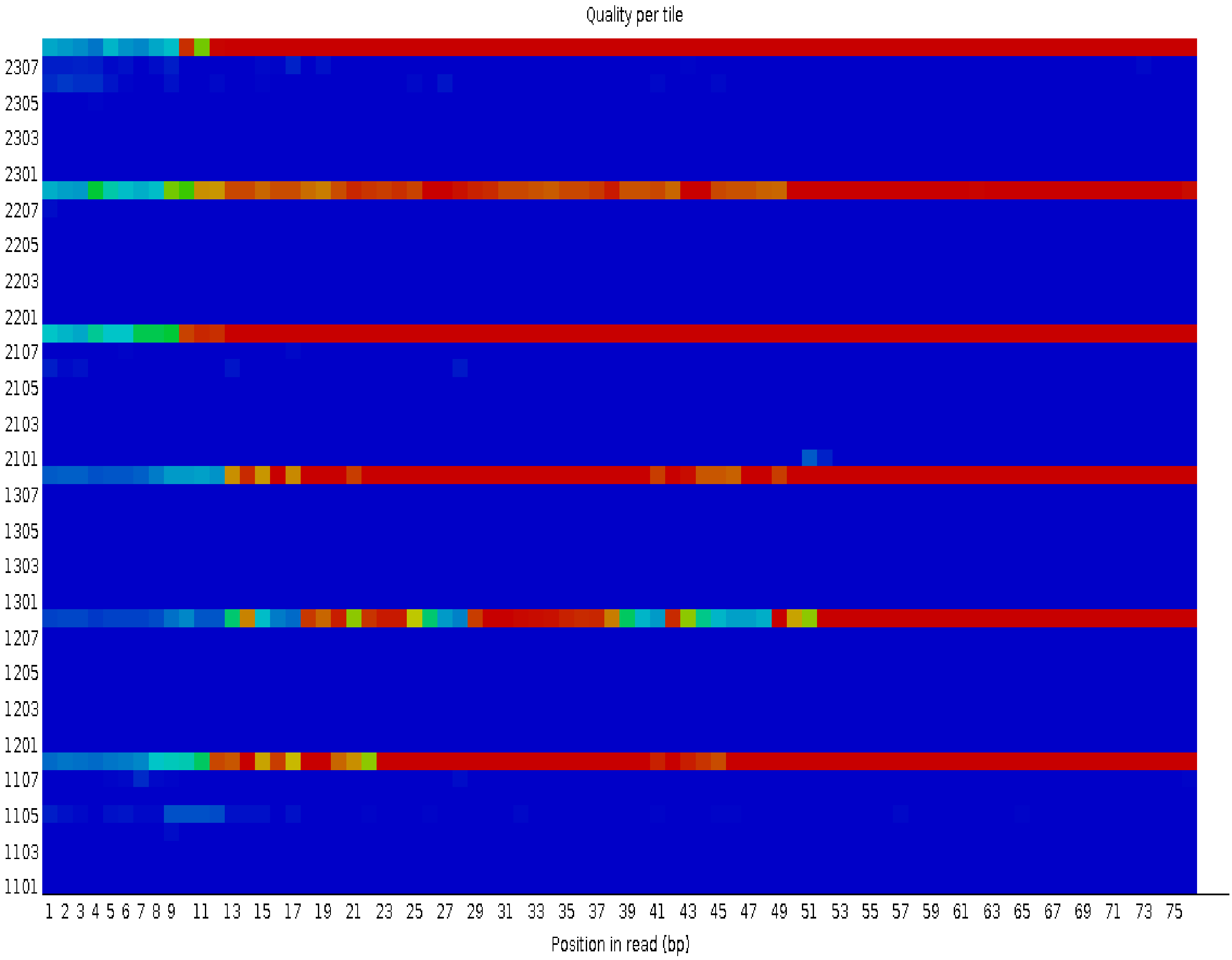


## Per sequence quality scores

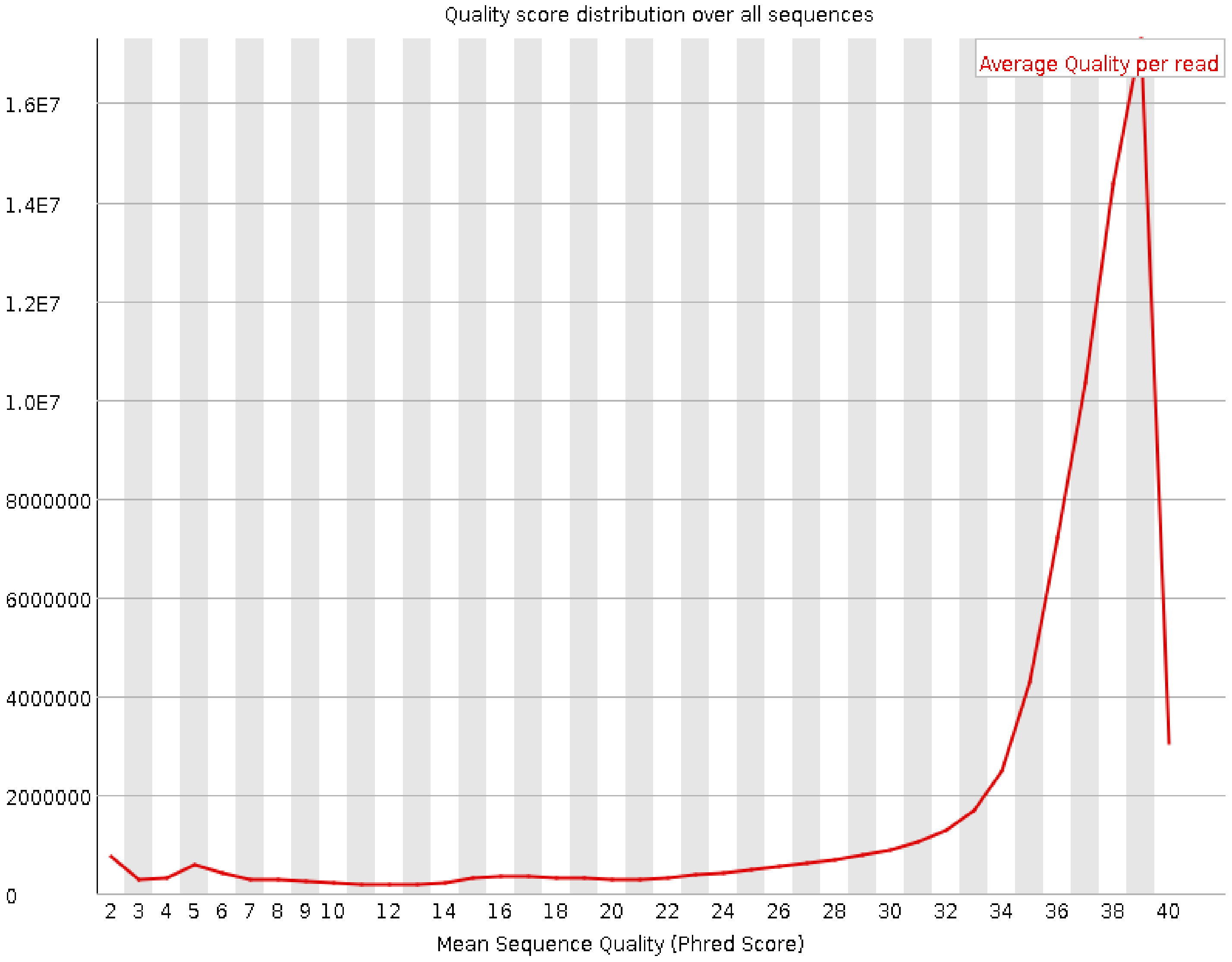


## Per base sequence content

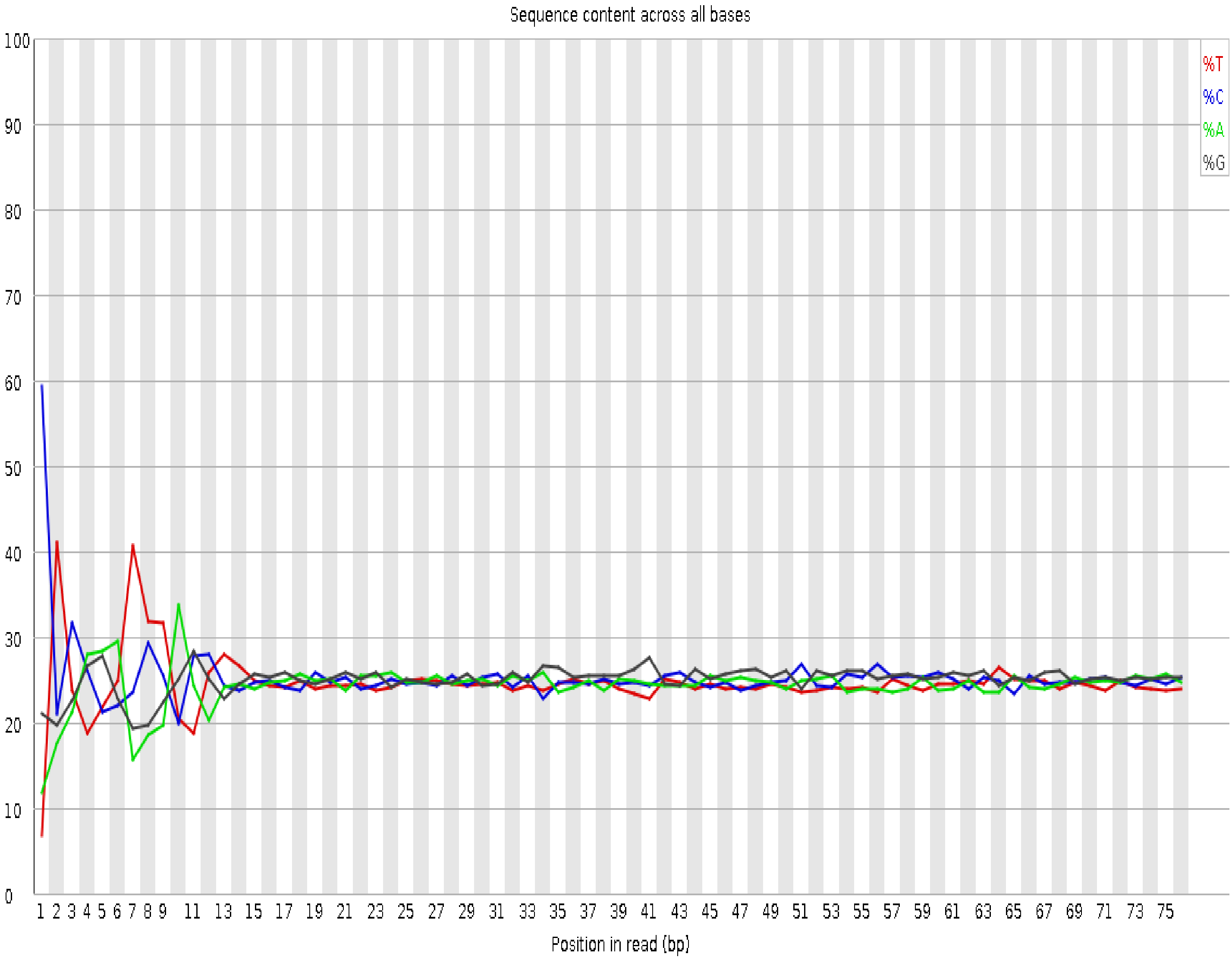


## Per sequence GC content

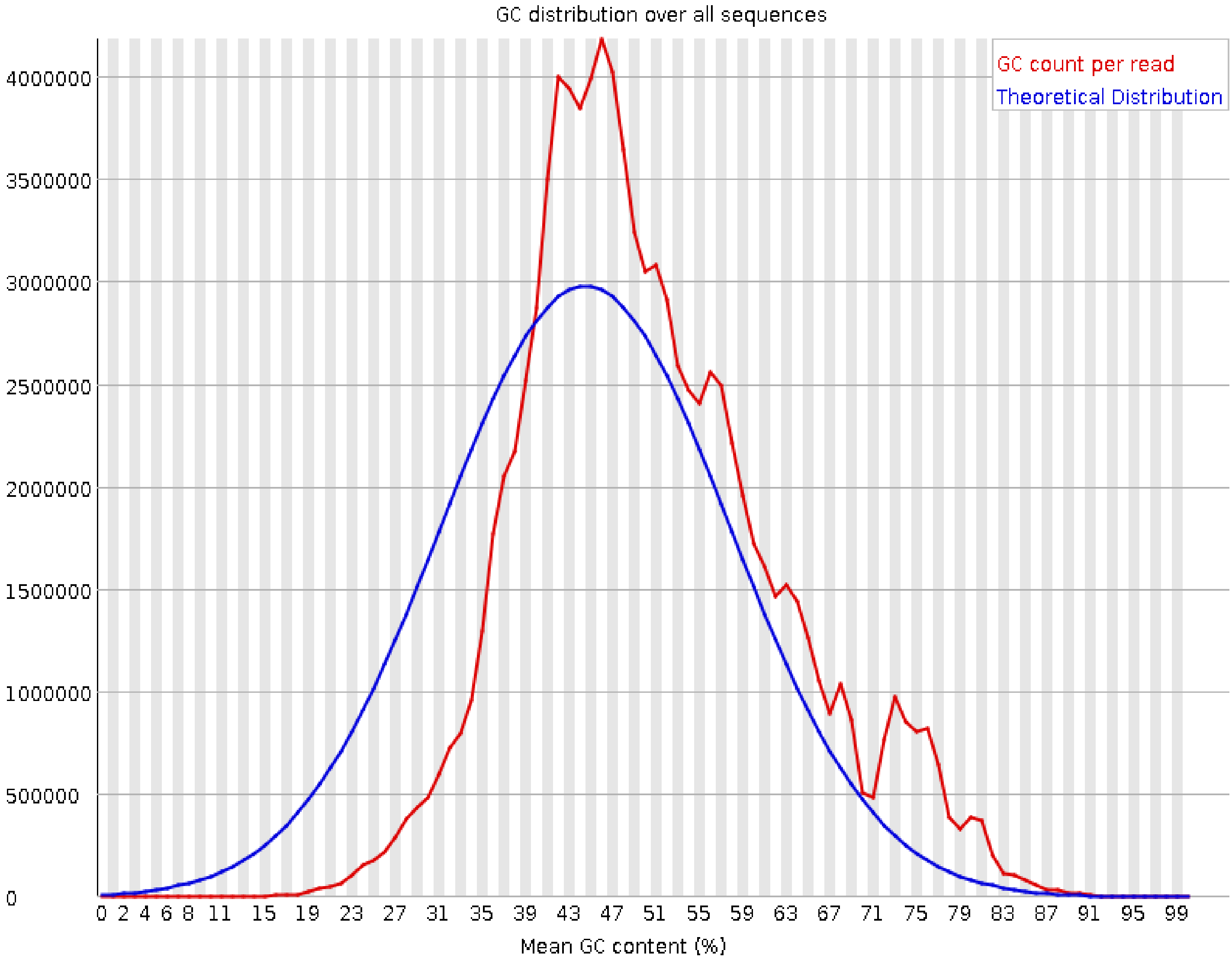


## Per base N content

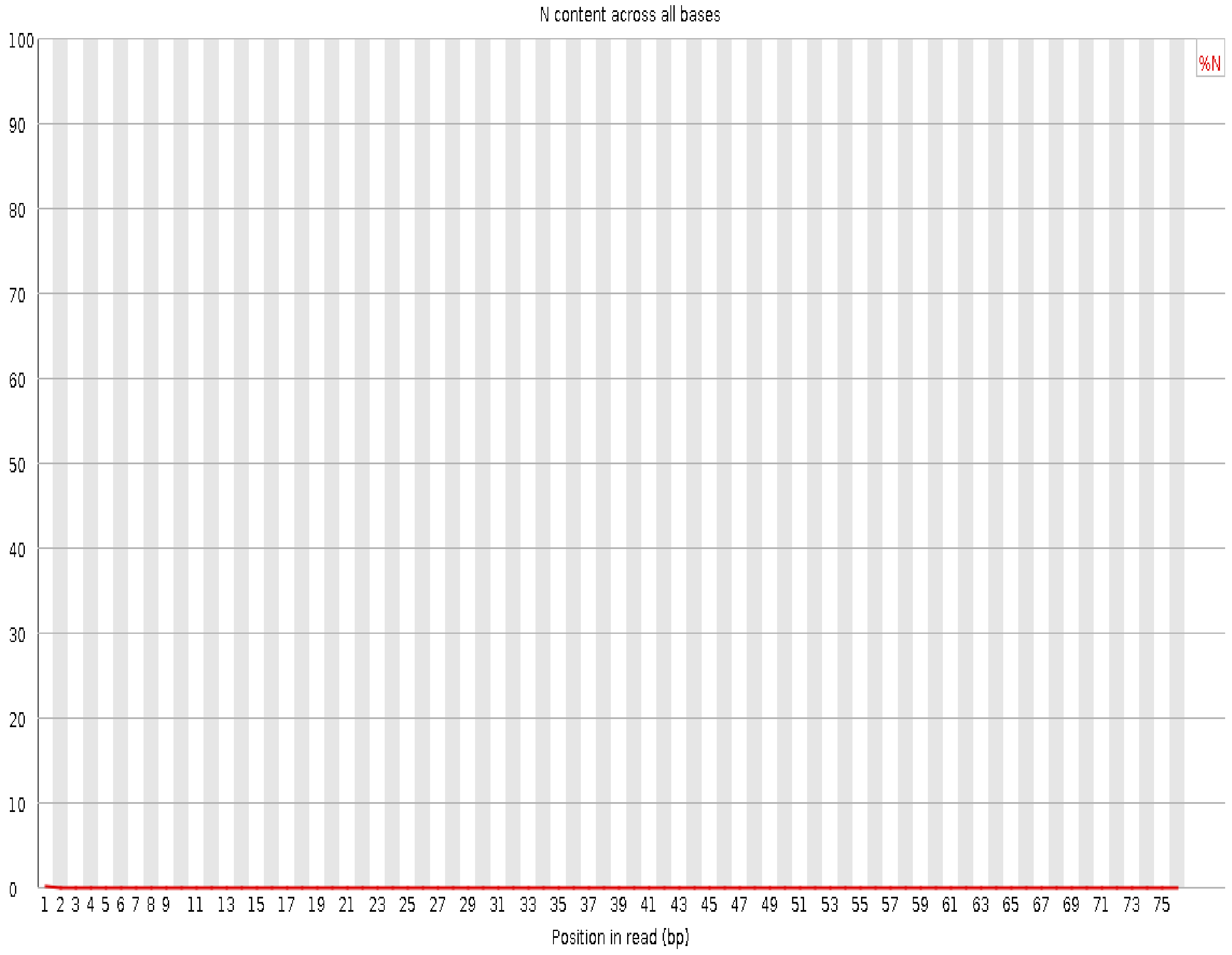


## Sequence Length Distribution

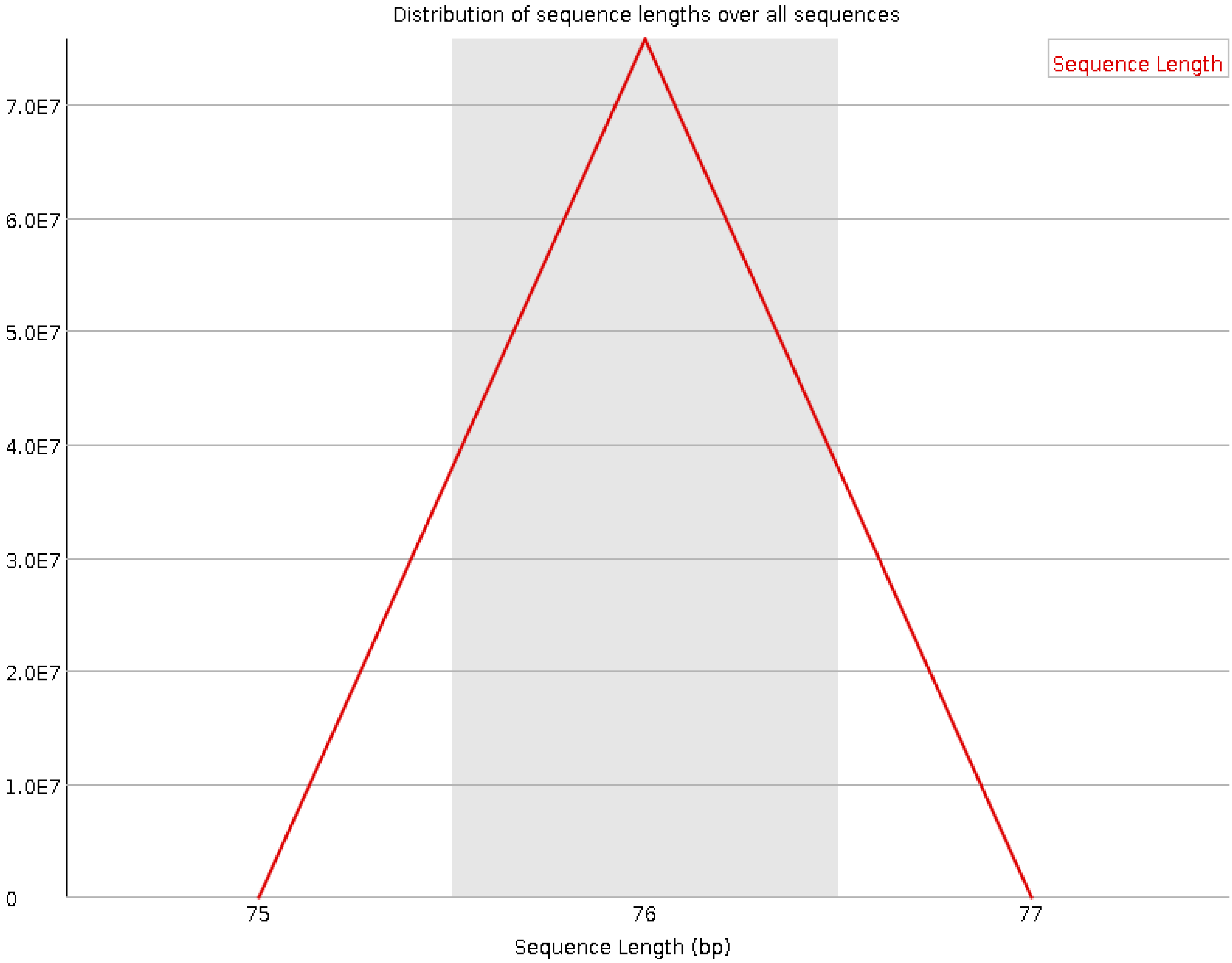


## Sequence Duplication Levels

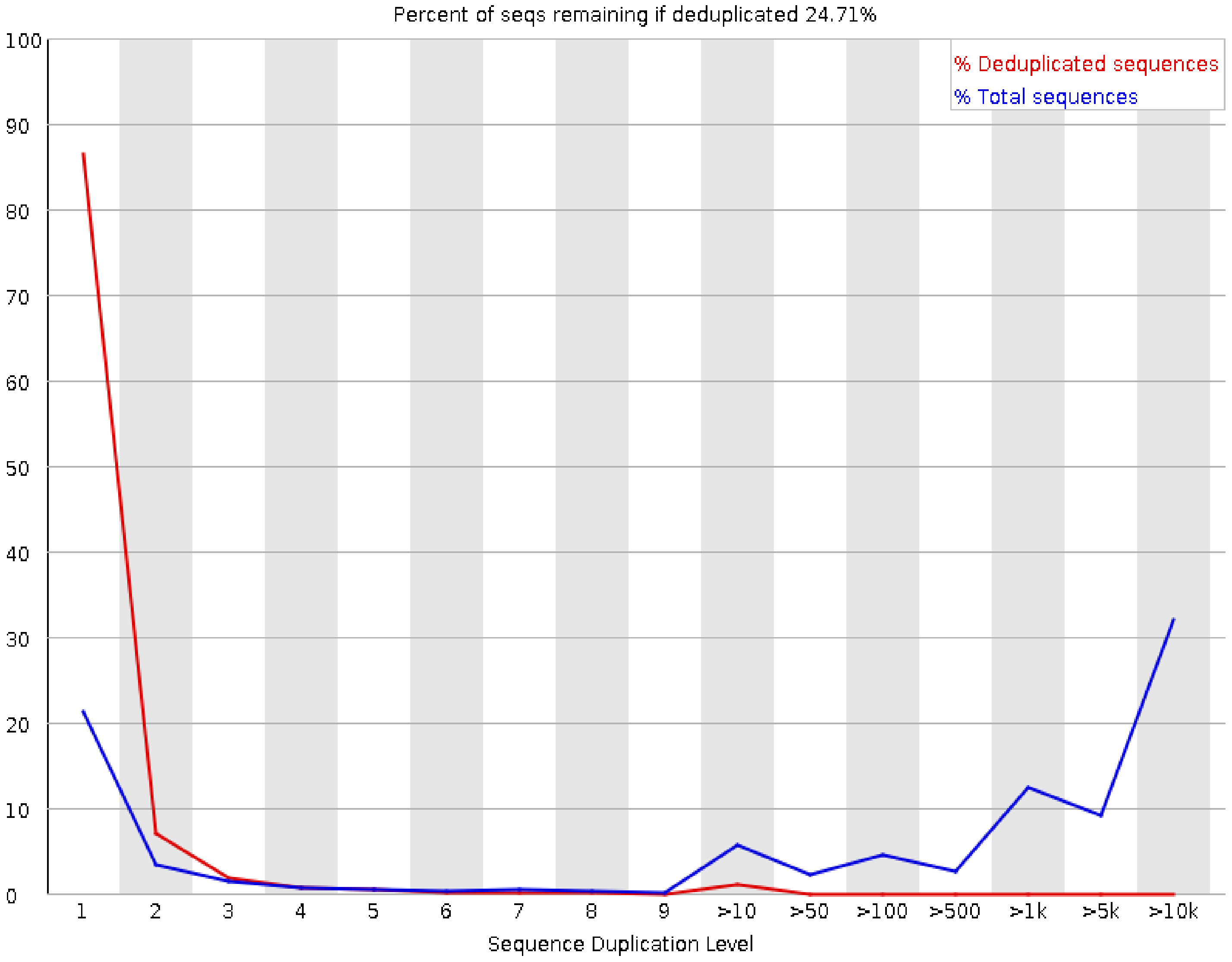


## Overrepresented sequences

| Sequence | Count | Percentage | Possible Source |
|---|---|---|---|
| CTTCCGTACGCCACATGTCCCGCGCCCCGCCGCGGGGCGGGGATTCGGCG | 290166 | 0.382865654275188 | No Hit |
| CCGGTATTTAGCCTTAGATGGAGTTTACCACCCGCTTTGGGCTGCATTCC | 223749 | 0.2952303415231938 | No Hit |
| CTCTCTTCAAAGTTCTTTTCAACTTTCCCTTACGGTACTTGTTGACTATC | 210637 | 0.2779294363211499 | No Hit |
| CTCCGAGGTCGCCCCAACCGAAATTTTTAATGCAGGTTTGGTAGTTTAGG | 189211 | 0.24965844830566847 | No Hit |
| CTCCCTTTCGATCGGCCGAGGGCAACGGAGGCCATCGCCCGTCCCTTCGG | 180419 | 0.23805765830136935 | No Hit |
| CGCAGTTTTATCCGGTAAAGCGAATGATTAGAGGTCTTGGGGCCGAAACG | 175879 | 0.23206725945929496 | No Hit |
| CTTATTTCTCTTGTCCTTTCGTACAGGGAGGAATTTGAAGTAGATAGAAA | 174725 | 0.23054458979767523 | No Hit |
| CCCGTCGGCATGTATTAGCTCTAGAATTACCACAGTTATCCAAGTAGGAG | 133893 | 0.1766679454101023 | No Hit |
| CTTGAACTCTCTCTTCAAAGTTCTTTTCAACTTTCCCTTACGGTACTTGT | 131224 | 0.1731462770159401 | No Hit |
| CTTGGTTATAATTTTTCATCTTTCCCTTGCGGTACTATATCTATTGCGCC | 126555 | 0.1669856664005997 | No Hit |
| CGGGTCTTCCGTACGCCACATGTCCCGCGCCCCGCCGCGGGGCGGGGATT | 119894 | 0.15819666933296592 | No Hit |

| Sequence | Count | Percentage | Possible Source |
|---|---|---|---|
| GTTAATTGTCAGTTCAGTGTTTTAATCTGACGCAGGCTTATGCGGAGGAG | 117363 | 0.154857087952065 | No Hit |
| CGCTGTTATCCCTAGGGTAACTTGTTCCGTTGGTCAAGTTATTGGATCAA | 115798 | 0.1527921156639931 | N[illegible] |
| CTGCCAGTAGCATATGCTTGTCTCAAAGATTAAGCCATGCATGTCTAAGT | 109790 | 0.14486473323157395 | No Hit |
| CTTAGATGGAGTTTACCACCCGCTTTGGGCTGCATTCCCAAGCAACCCGA | 107808 | 0.14224954149038643 | No Hit |
| CCCGCTTTGGGCTGCATTCCCAAGCAACCCGACTCCGGGAAGACCCGGGC | 100766 | 0.13295782592961822 | No Hit |
| CCCGAAGTTACGGATCCGGCTTGCCGACTTCCCTTACCTACATTGTTCCA | 93158 | 0.12291928972025655 | No Hit |
| CTGAATTTAAGCATATTAGTCAGCGGAGGAAAAGAAACTAACCAGGATTC | 93129 | 0.12288102505804949 | No Hit |
| GTATAATACTAAGTTGAGATGATATCATTTACGGGGGAAGGCGCTTTGTG | 92254 | 0.12172648783628404 | No Hit |
| GCCCTCTTGAACTCTCTCTTCAAAGTTCTTTTCAACTTTCCCTTACGGTA | 91432 | 0.12064188258337982 | No Hit |
| CTTACTTTTAACCAGTGAAATTGACCTGCCCGTGAAGAGGCGGGCATGAC | 89991 | 0.11874052471302096 | No Hit |
| CTGCTGTCTATATCAACCAACACCTTTTCTGGGGTCTGATGAGCGTCGGC | 88783 | 0.11714660361142938 | No Hit |
| CTCGCATTCCACGCCCGGCTCCACGCCAGCGAGCCGGGCTTCTTACCCAT | 88326 | 0.11654360531389016 | No Hit |
| CACCCGTTTACCTCTTAACGGTTTCACGCCCTCTTGAACTCTCTCTTCAA | 86415 | 0.11402209602155446 | No Hit |
| CTTTTAACCAGTGAAATTGACCTGCCCGTGAAGAGGCGGGCATGACACAG | 85411 | 0.11269734702652302 | No Hit |
| ATAATACTAAGTTGAGATGATATCATTTACGGGGGAAGGCGCTTTGTGAA | 83456 | 0.11011778100532141 | No Hit |
| CTGGATAGTAGGTAGGGACAGTGGGAATCTCGTTCATCCATTCATGCGCG | 82851 | 0.10931950098341502 | No Hit |
| CTCGATCAGAAGGACTTGGGCCCCCCACGAGCGGCGCCGGGGAGCGGGTC | 81153 | 0.10707903903763476 | No Hit |
| CTGAACTCCTCACACCCAATTGGACCAATCTATCACCCTATAGAAGAACT | 80679 | 0.10645360973121555 | No Hit |
| CTTGTCTCAAAGATTAAGCCATGCATGTCTAAGTACGCACGGCCGGTACA | 77538 | 0.10230915097285528 | No Hit |
| CAACAATAGGGTTTACGACCTCGATGTTGGATCAGGACATCCCGATGGTG | 77359 | 0.10207296564405985 | No Hit |
| CTAAAAGCAGCCACCAATTAAGAAAGCGTTCAAGCTCAACACCCACTACC | 76187 | 0.10052654550244947 | No Hit |
| CTGAATTTAAGCATATTAGTCAGCGGAGGAGAAGAAACTAACCAGGATTC | 76140 | 0.10046453036025177 | No Hit |

## Adapter Content

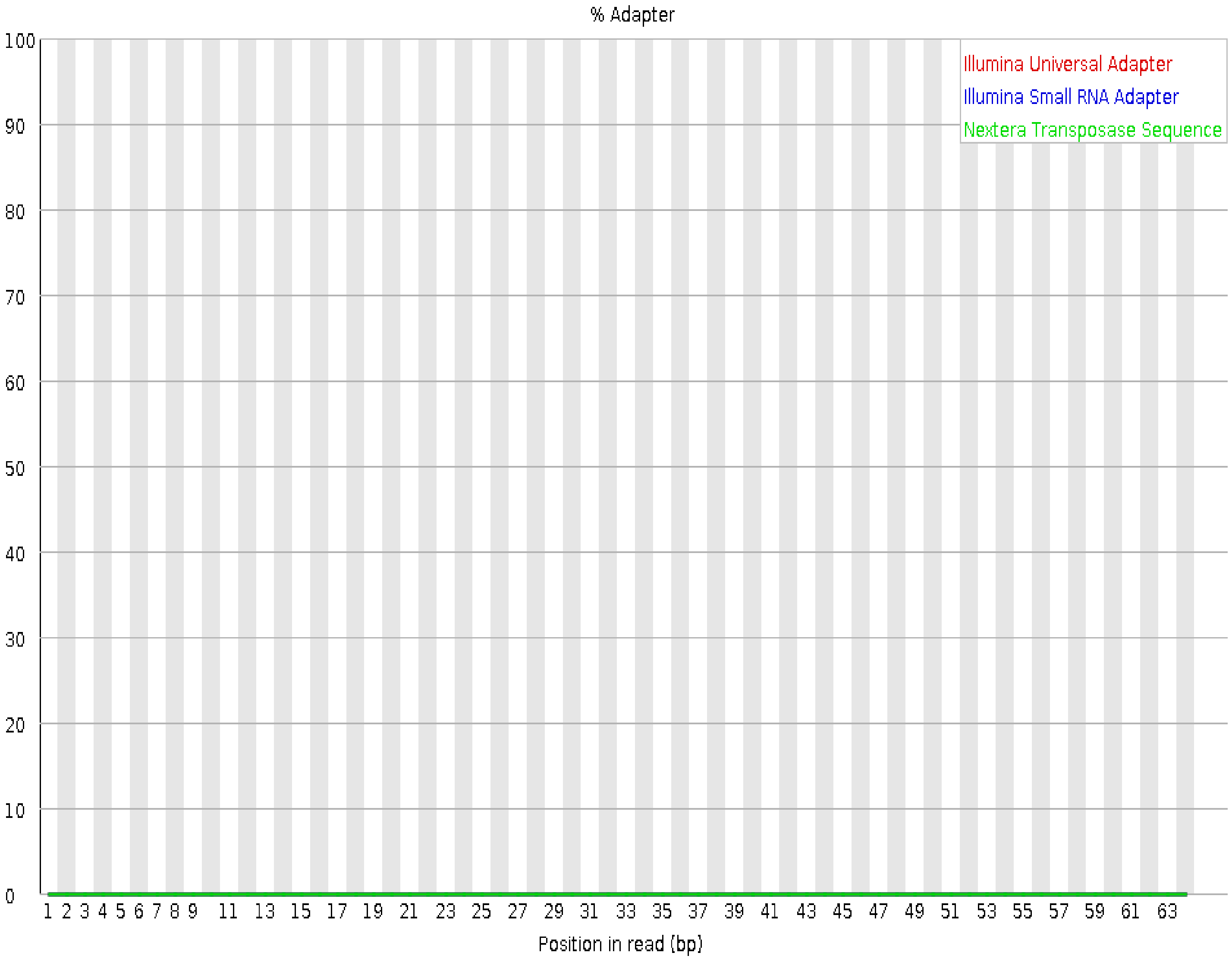


## Kmer Content

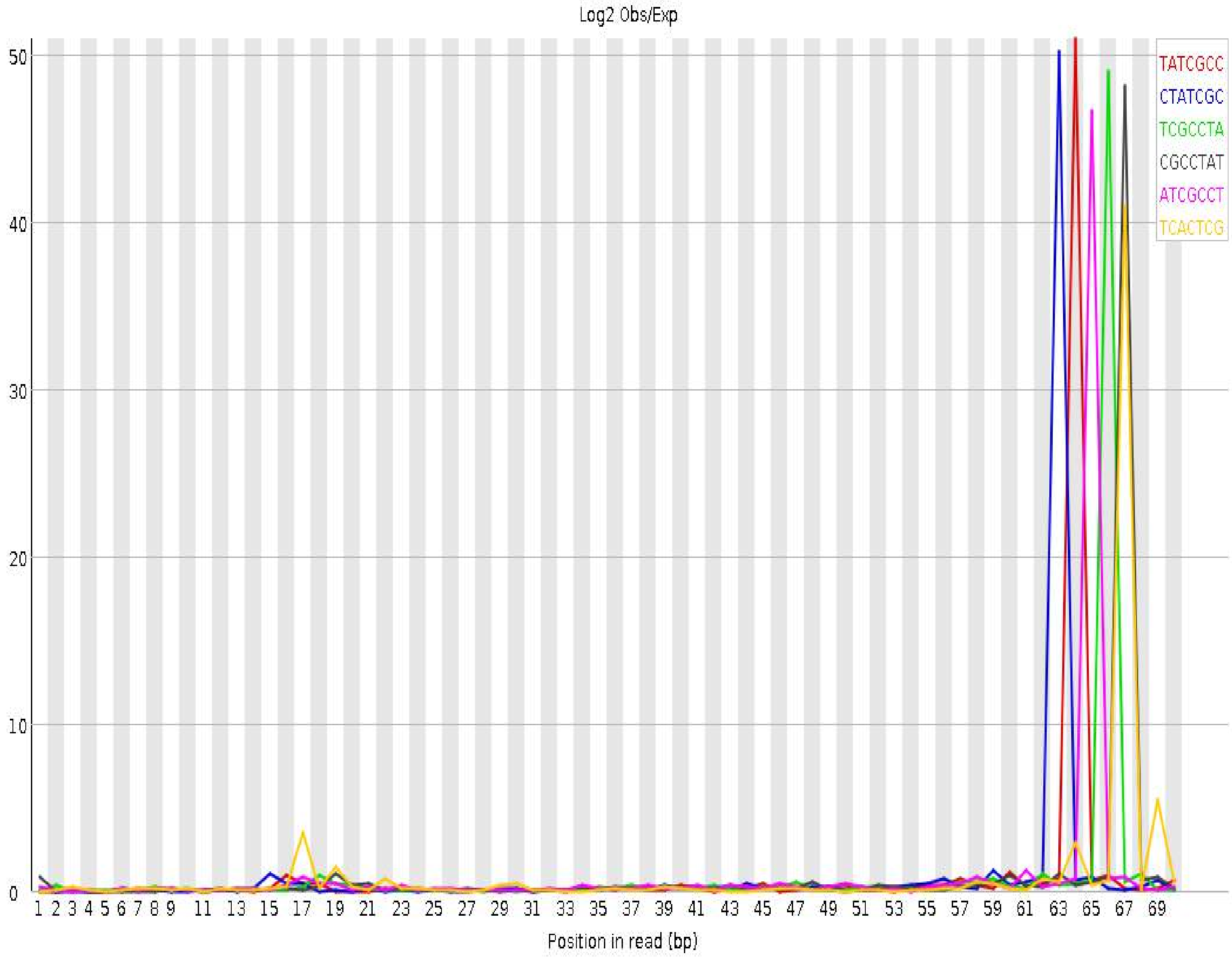


| Sequence | Count | PValue | Obs/Exp Max | Max Obs/Exp Position |
|---|---|---|---|---|
| TATCGCC | 20570 | 0.0 | 50.883484 | 64 |
| CTATCGC | 20825 | 0.0 | 50.151634 | 63 |
| TCGCCTA | 21180 | 0.0 | 48.9858 | 66 |
| CGCCTAT | 21595 | 0.0 | 48.077213 | 67 |
| ATCGCCT | 22415 | 0.0 | 46.646255 | 65 |
| TCACTCG | 55620 | 0.0 | 40.981865 | 67 |
| ACTCGCC | 56135 | 0.0 | 40.461308 | 69 |
| TCTATCG | 26285 | 0.0 | 39.962723 | 62 |
| CGCGTAA | 12790 | 0.0 | 35.417362 | 1 |
| CTCGCCG | 70095 | 0.0 | 35.198536 | 70 |
| CGGAACG | 41825 | 0.0 | 34.73808 | 48 |
| TCAGACG | 23280 | 0.0 | 34.432266 | 2 |
| TTCACTC | 66175 | 0.0 | 34.307446 | 66 |
| GCCTATA | 30805 | 0.0 | 34.19137 | 68 |
| ACGGCGC | 45010 | 0.0 | 33.15262 | 52 |

| Sequence | Count | PValue | Obs/Exp Max | Max Obs/Exp Position |
| --- | --- | --- | --- | --- |
| CCCGTCG | 35405 | 0.0 | 32.[illegible] | 1 |
| GCGTAAC | 14445 | 0.0 | 31.59[illegible] | 2 |
| CCGTCGG | 35470 | 0.0 | 31.559519 | 2 |
| CCTTCGG | 46175 | 0.0 | 31.554165 | 44 |
| AGGTCGC | 50230 | 0.0 | 30.586868 | 6 |

**Produced by FastQC (version 0.11.2)**

# FastQC Report

Mon 4 May 2015
Biochain_Adult_Heart_CGATGT_L001_R2.fastq.gz

## Summary


- Basic Statistics
- Per base sequence quality
- Per tile sequence quality
- Per sequence quality scores
- Per base sequence content
- Per sequence GC content
- Per base N content
- Sequence Length Distribution
- Sequence Duplication Levels
- Overrepresented sequences
- Adapter Content
- Kmer Content


## Basic Statistics

| Measure | Value |
|---|---|
| Filename | Biochain_Adult_Heart_CGATGT_L001_R2.fastq.gz |
| File type | Conventional base calls |
| Encoding | Sanger / Illumina 1.9 |
| Total Sequences | 75787942 |
| Sequences flagged as poor quality | 0 |
| Sequence length | 76 |
| %GC | 50 |

## Per base sequence quality

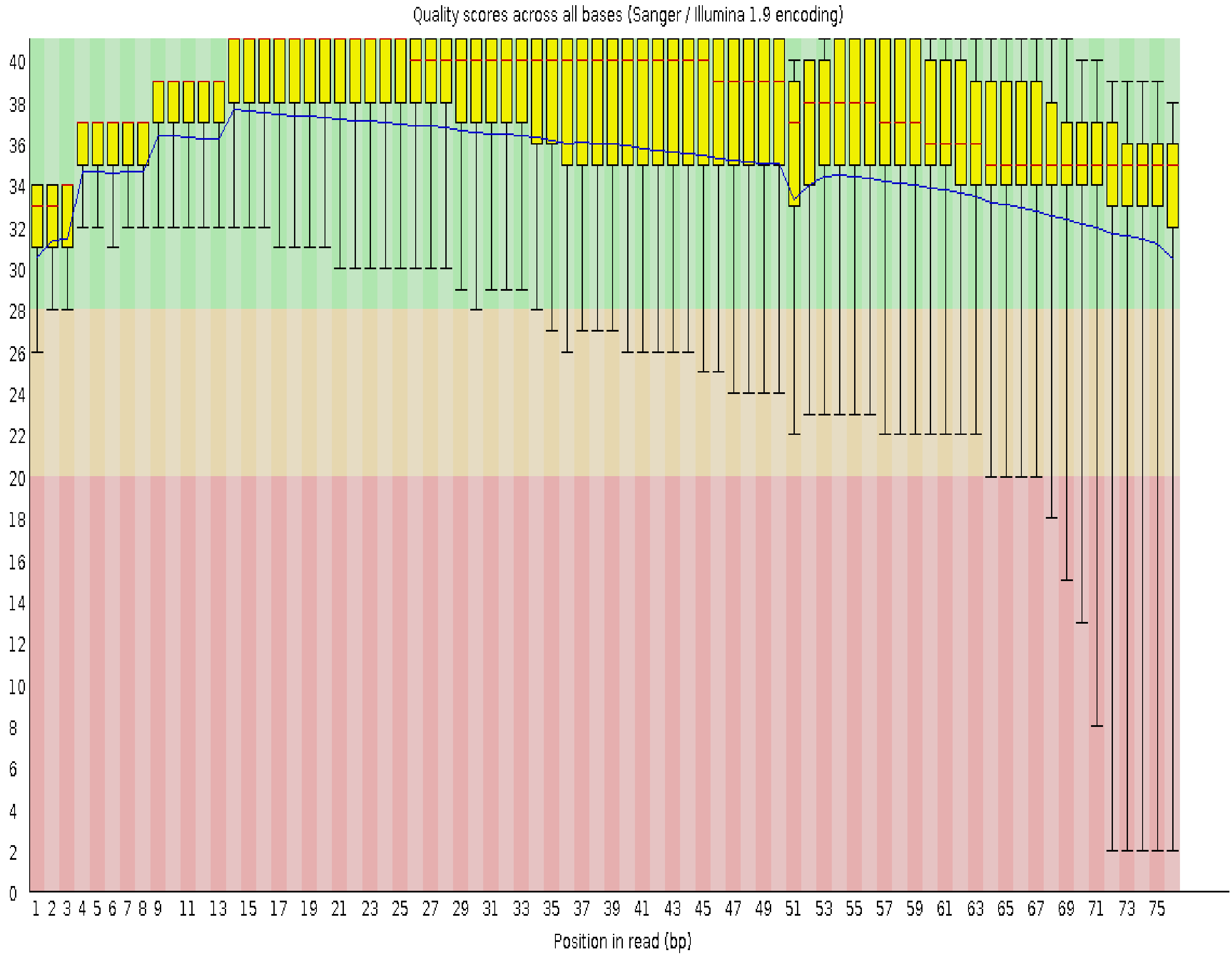


## Per tile sequence quality

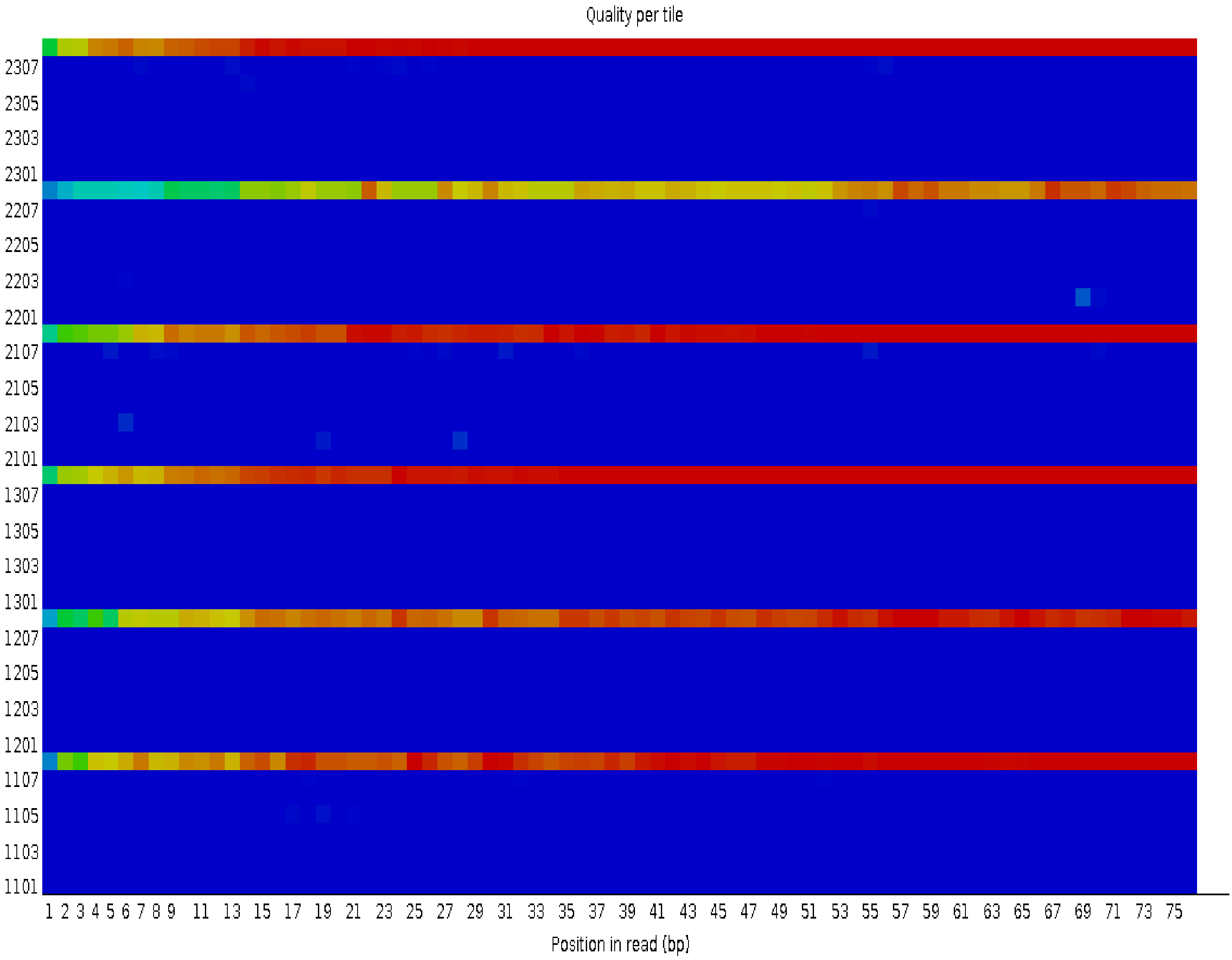


## Per sequence quality scores

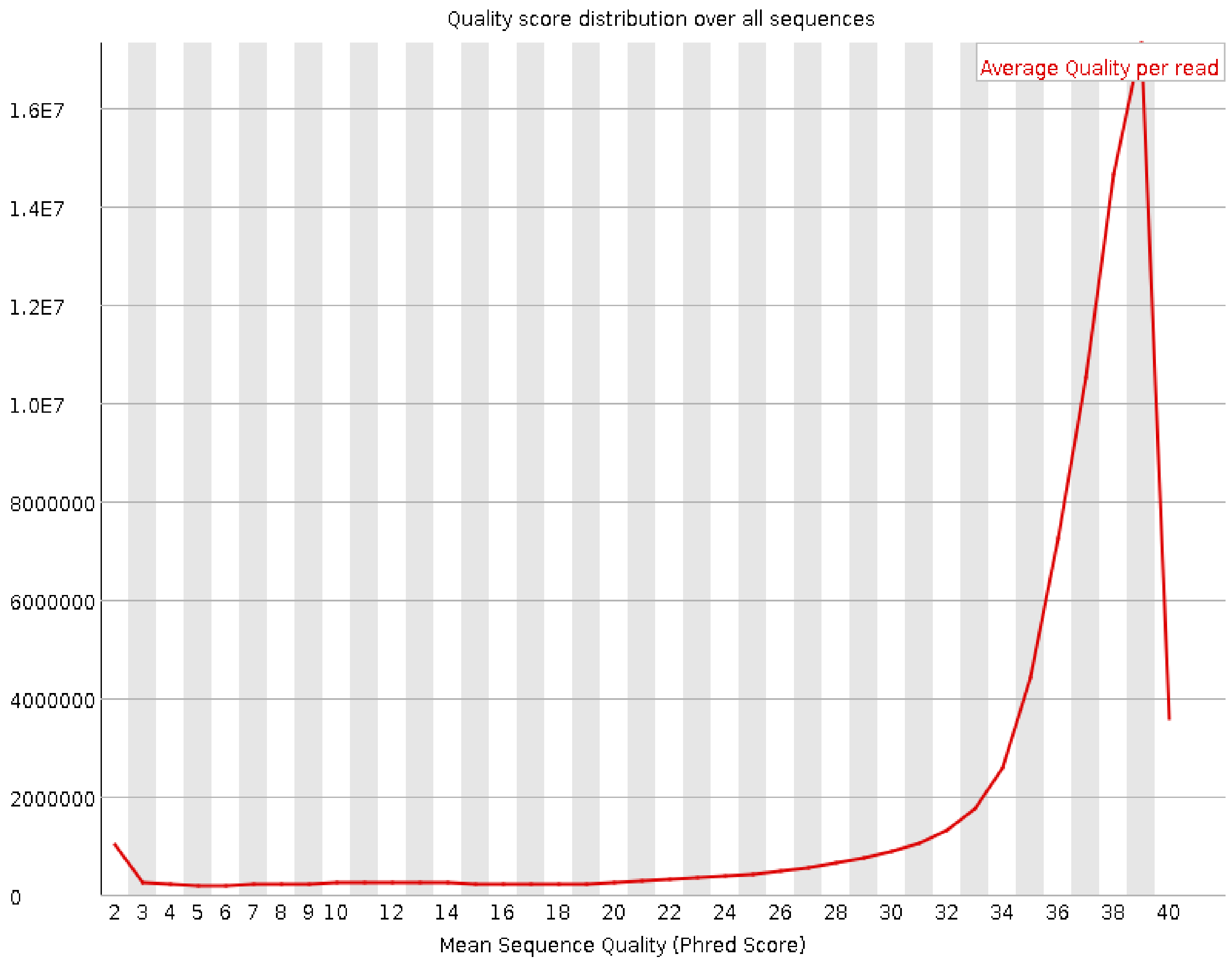


## Per base sequence content

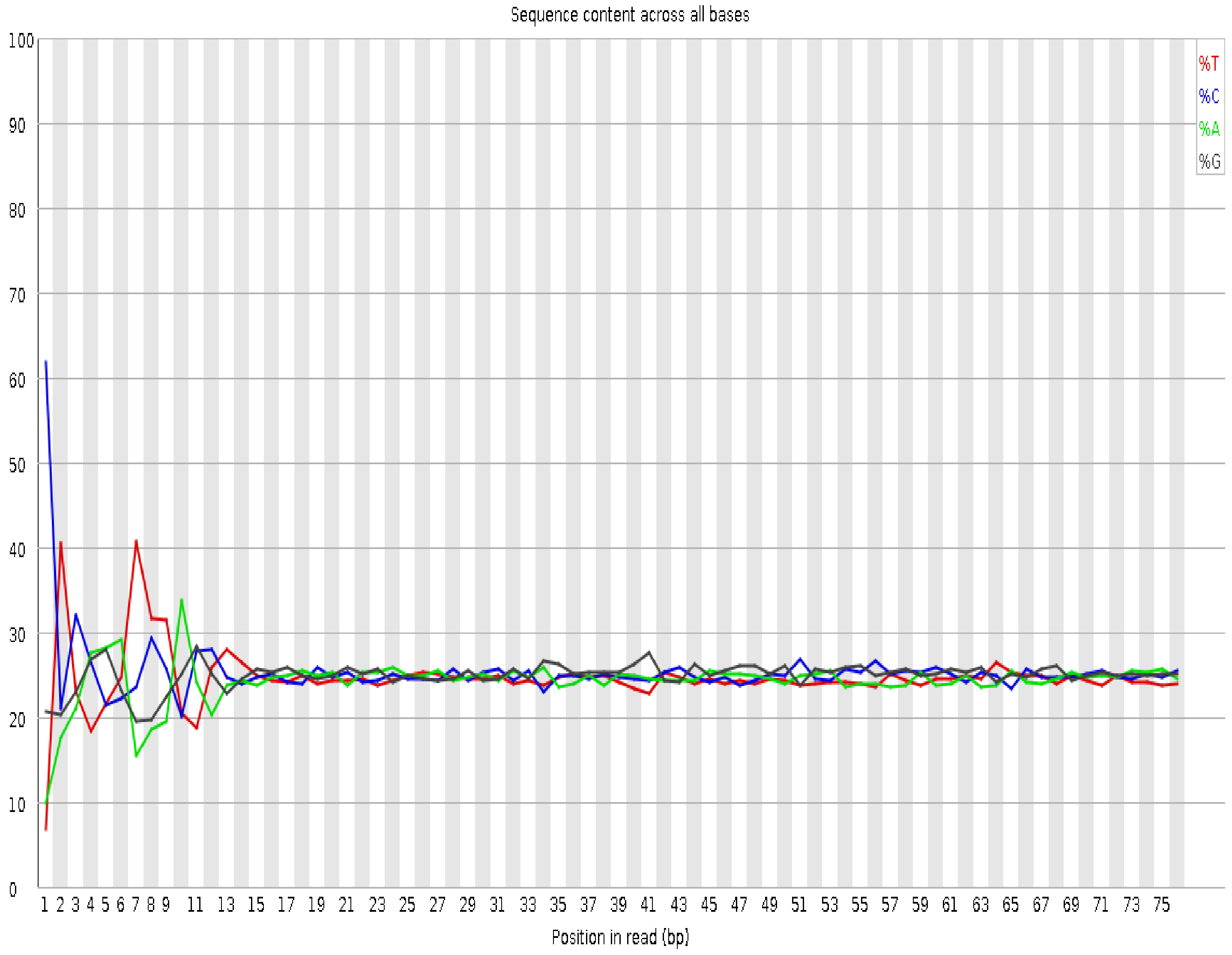


## Per sequence GC content

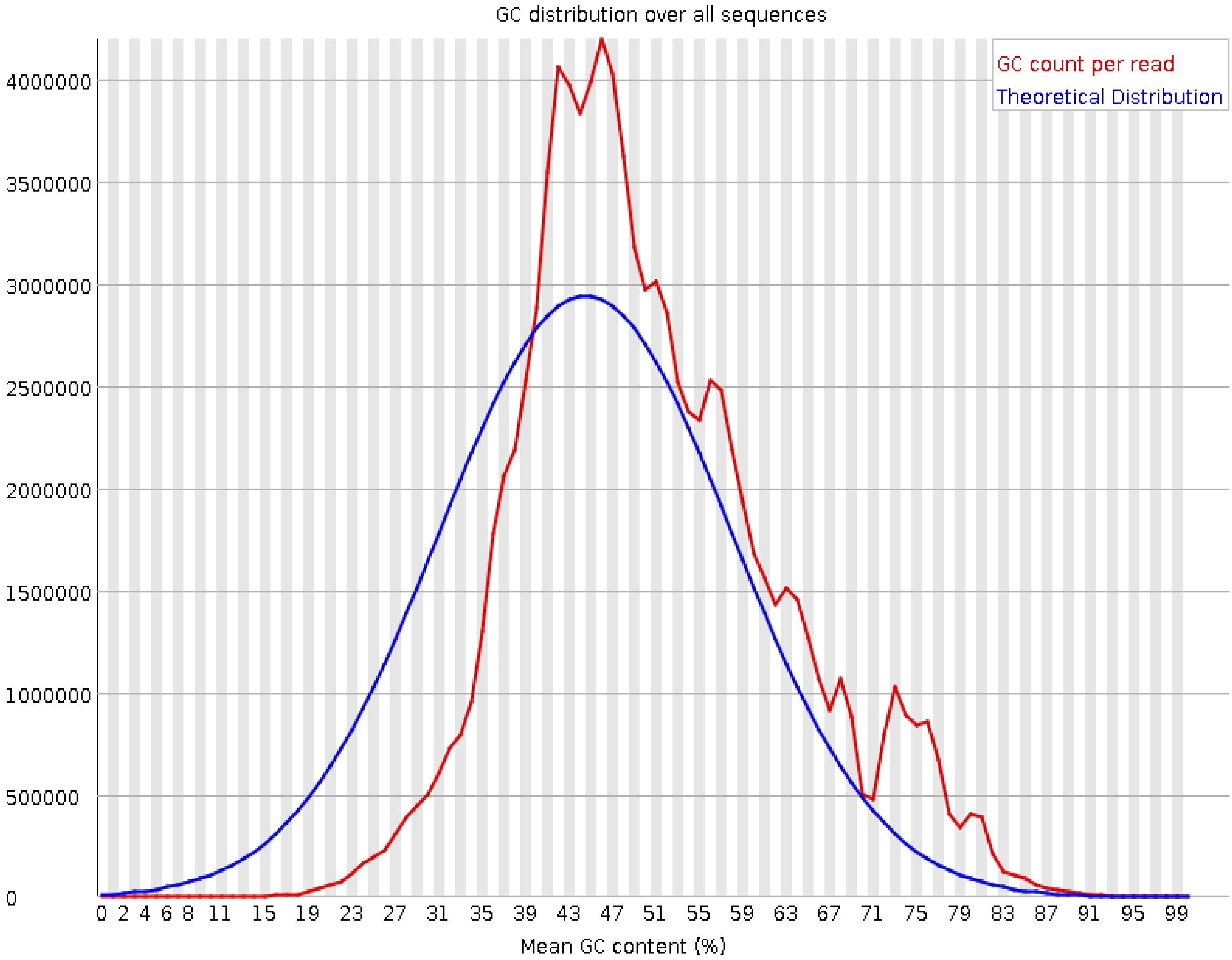


## Per base N content

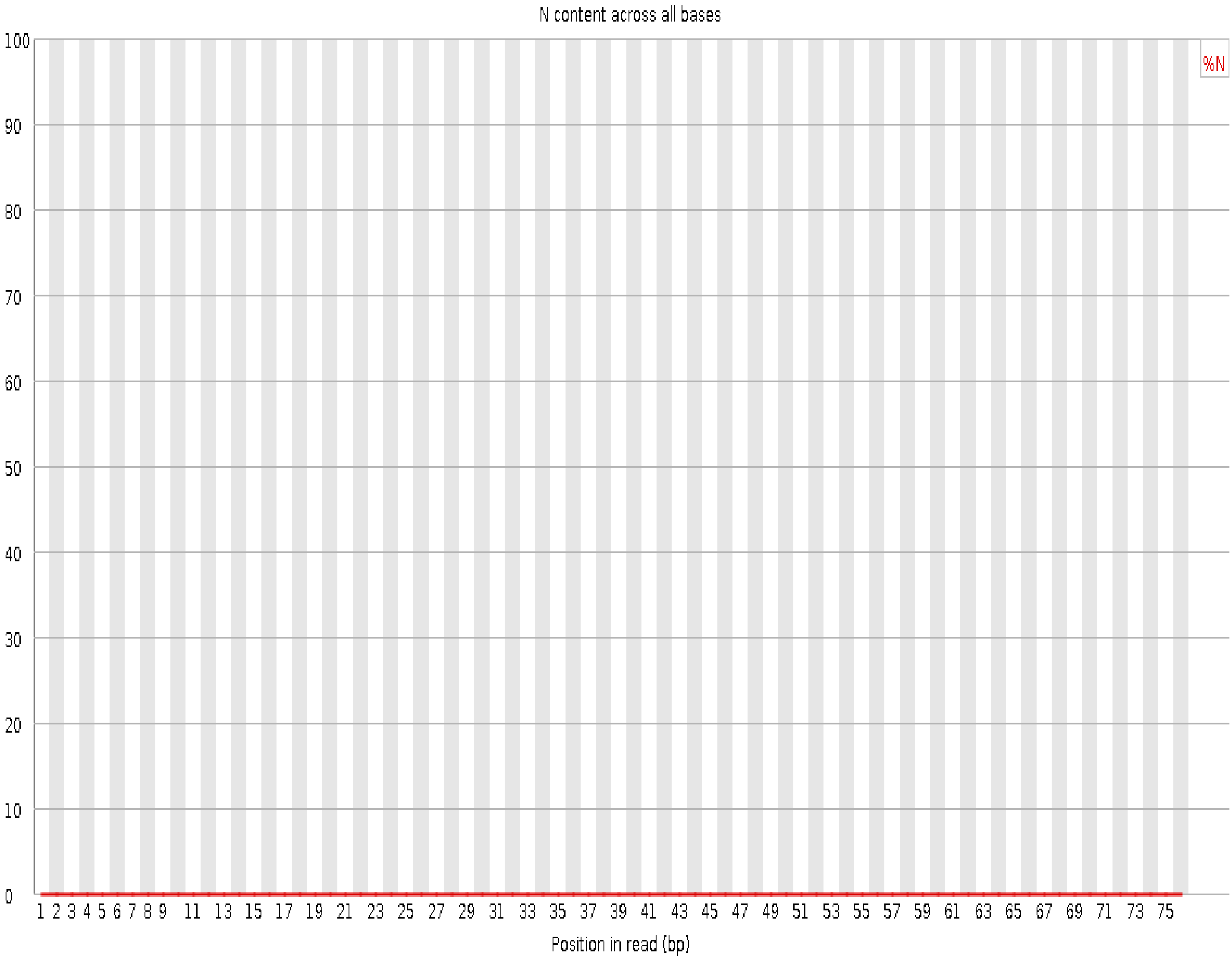


## Sequence Length Distribution

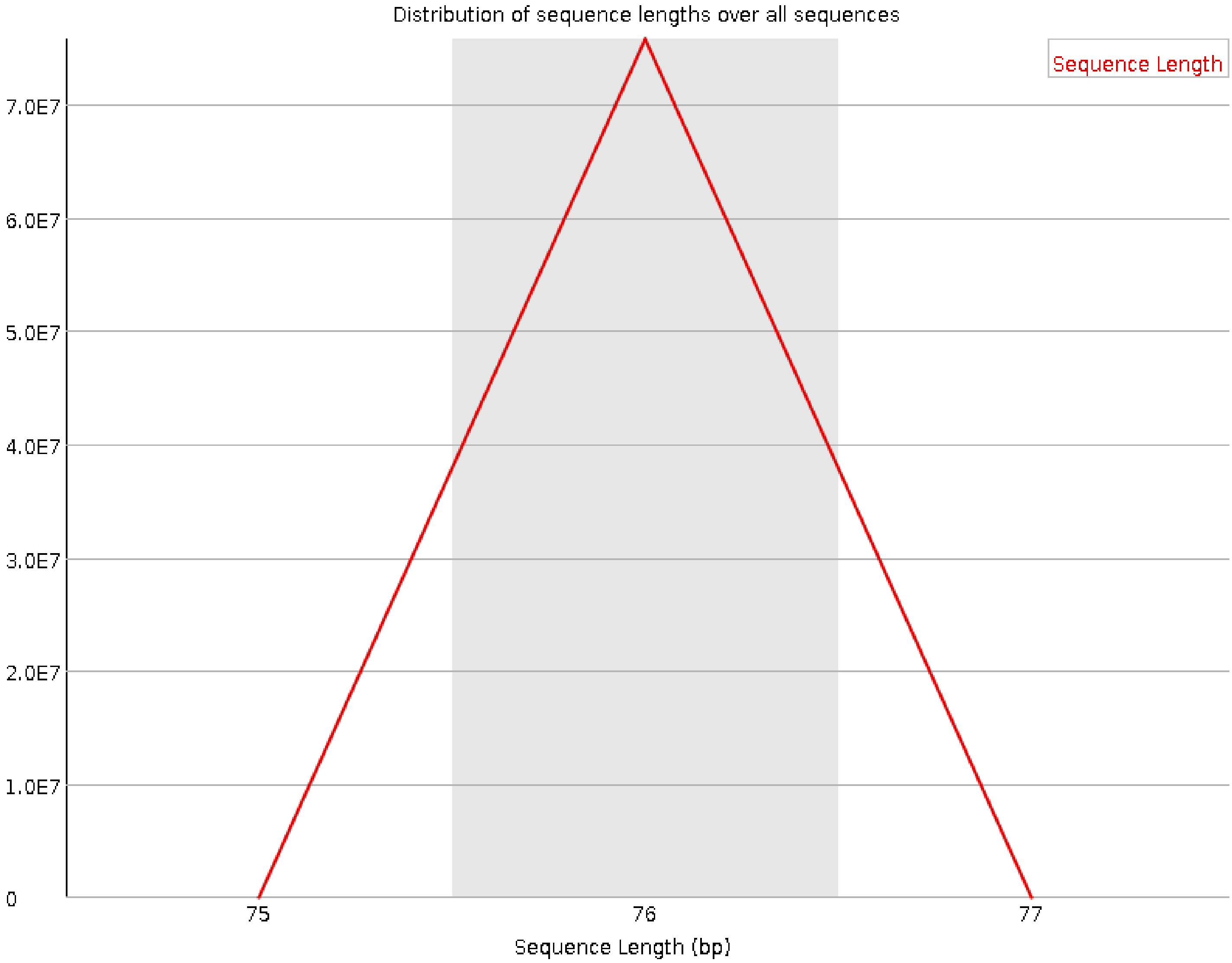


## Sequence Duplication Levels

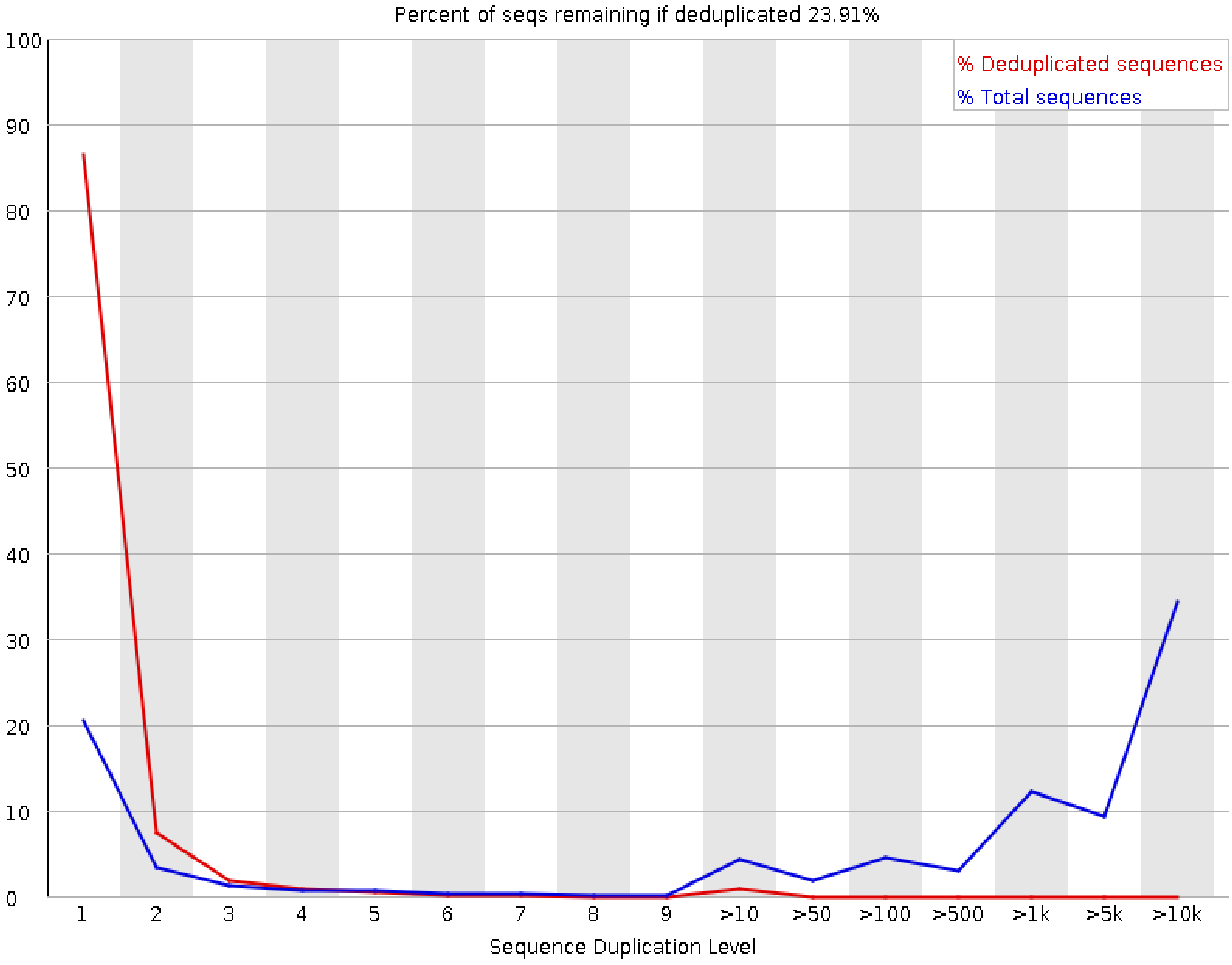


## Overrepresented sequences

| Sequence | Count | Percentage | Possible Source |
|---|---|---|---|
| CTTCCGTACGCCACATGTCCCGCGCCCCGCCGCGGGGCGGGGATTCGGCG | 306711 | 0.4046963037998842 | No Hit |
| CCGGTATTTAGCCTTAGATGGAGTTTACCACCCGCTTTGGGCTGCATTCC | 236023 | 0.311425529934564 | No Hit |
| CTCCGAGGTCGCCCCAACCGAAATTTTTAATGCAGGTTTGGTAGTTTAGG | 217206 | 0.2865970420466095 | No Hit |
| CTCTCTTCAAAGTTCTTTTCAACTTTCCCTTACGGTACTTGTTGACTATC | 211198 | 0.2786696596141904 | No Hit |
| CTCCCTTTCGATCGGCCGAGGGCAACGGAGGCCATCGCCCGTCCCTTCGG | 202643 | 0.2673815842631008 | No Hit |
| CGCAGTTTTATCCGGTAAAGCGAATGATTAGAGGTCTTGGGGCCGAAACG | 191806 | 0.2530824758376471 | No Hit |
| CTTATTTCTCTTGTCCTTTCGTACAGGGAGGAATTTGAAGTAGATAGAAA | 185128 | 0.24427104776113331 | No Hit |
| CCCGTCGGCATGTATTAGCTCTAGAATTACCACAGTTATCCAAGTAGGAG | 147565 | 0.1947077544340761 | No Hit |
| CGGGTCTTCCGTACGCCACATGTCCCGCGCCCCGCCGCGGGGCGGGGATT | 147123 | 0.1941245482031957 | No Hit |
| CTTGAACTCTCTCTTCAAAGTTCTTTTCAACTTTCCCTTACGGTACTTGT | 131969 | 0.17412928299332894 | No Hit |
| CGCTGTTATCCCTAGGGTAACTTGTTCCGTTGGTCAAGTTATTGGATCAA | 131956 | 0.17411212986889127 | No Hit |

| Sequence | Count | Percentage | Possible Source |
|---|---|---|---|
| CTTGGTTATAATTTTTCATCTTTCCCTTGCGGTACTATATCTATTGCGCC | 119193 | 0.15727172008444298 | No Hi[illegible] |
| CTGCCAGTAGCATATGCTTGTCTCAAAGATTAAGCCATGCATGTCTAAGT | 117556 | 0.15511174587640866 | N[illegible] |
| GTTAATTGTCAGTTCAGTGTTTTAATCTGACGCAGGCTTATGCGGAGGAG | 115327 | 0.15217064477090564 | No Hit |
| CTTAGATGGAGTTTACCACCCGCTTTGGGCTGCATTCCCAAGCAACCCGA | 104998 | 0.13854182766963116 | No Hit |
| CCCGCTTTGGGCTGCATTCCCAAGCAACCCGACTCCGGGAAGACCCGGGC | 104551 | 0.13795202408319782 | No Hit |
| GCCCTCTTGAACTCTCTCTTCAAAGTTCTTTTCAACTTTCCCTTACGGTA | 97821 | 0.1290719835089334 | No Hit |
| CTGAACTCCTCACACCCAATTGGACCAATCTATCACCCTATAGAAGAACT | 97011 | 0.12800321190935623 | No Hit |
| CACCCGTTTACCTCTTAACGGTTTCACGCCCTCTTGAACTCTCTCTTCAA | 93589 | 0.12348798176892045 | No Hit |
| CTCGCATTCCACGCCCGGCTCCACGCCAGCGAGCCGGGCTTCTTACCCAT | 93388 | 0.12322276807569205 | No Hit |
| CTGCTGTCTATATCAACCAACACCTTTTCTGGGGTCTGATGAGCGTCGGC | 91682 | 0.12097175036102709 | No Hit |
| GTATAATACTAAGTTGAGATGATATCATTTACGGGGGAAGGCGCTTTGTG | 91609 | 0.12087542896995408 | No Hit |
| CTGGATAGTAGGTAGGGACAGTGGGAATCTCGTTCATCCATTCATGCGCG | 88928 | 0.1173379269224648 | No Hit |
| CTGAATTTAAGCATATTAGTCAGCGGAGGAAAAGAAACTAACCAGGATTC | 88603 | 0.11690909881152334 | No Hit |
| CTCGATCAGAAGGACTTGGGCCCCCCACGAGCGGCGCCGGGGAGCGGGTC | 86726 | 0.11443245153694767 | No Hit |
| CCCGAAGTTACGGATCCGGCTTGCCGACTTCCCTTACCTACATTGTTCCA | 84443 | 0.11142009899147282 | No Hit |
| CTCCGCCACTCCGGATTCGGGGATCTGAACCCGACTCCCTTTCGATCGGC | 83520 | 0.11020222715639909 | No Hit |
| CTTCACCGTGCCAGACTAGAGTCAAGCTCAACAGGGTCTTCTTTCCCCGC | 83501 | 0.1101771572052979 | No Hit |
| CCCCAACCGAAATTTTTAATGCAGGTTTGGTAGTTTAGGACCTGTGGGTT | 83426 | 0.11007819687200372 | No Hit |
| CAACAATAGGGTTTACGACCTCGATGTTGGATCAGGACATCCCGATGGTG | 79266 | 0.10458919705195319 | No Hit |
| CTTGTCTCAAAGATTAAGCCATGCATGTCTAAGTACGCACGGCCGGTACA | 78850 | 0.10404029706994815 | No Hit |
| CTTACTTTTAACCAGTGAAATTGACCTGCCCGTGAAGAGGCGGGCATGAC | 78731 | 0.10388328000778804 | No Hit |
| CTGAGTTCAGACCGGAGTAATCCAGGTCGGTTTCTATCTACTTCAAATTC | 78224 | 0.10321430815471938 | No Hit |
| CTAAAAGCAGCCACCAATTAAGAAAGCGTTCAAGCTCAACACCCACTACC | 78140 | 0.10310347258142989 | No Hit |
| CTCCATCTAAGGCTAAATACCGGCACGAGACCGATAGTCAACAAGTACCG | 77096 | 0.10172594474197492 | No Hit |
| CTTTTAACCAGTGAAATTGACCTGCCCGTGAAGAGGCGGGCATGACACAG | 76477 | 0.10090919212452029 | No Hit |

## Adapter Content

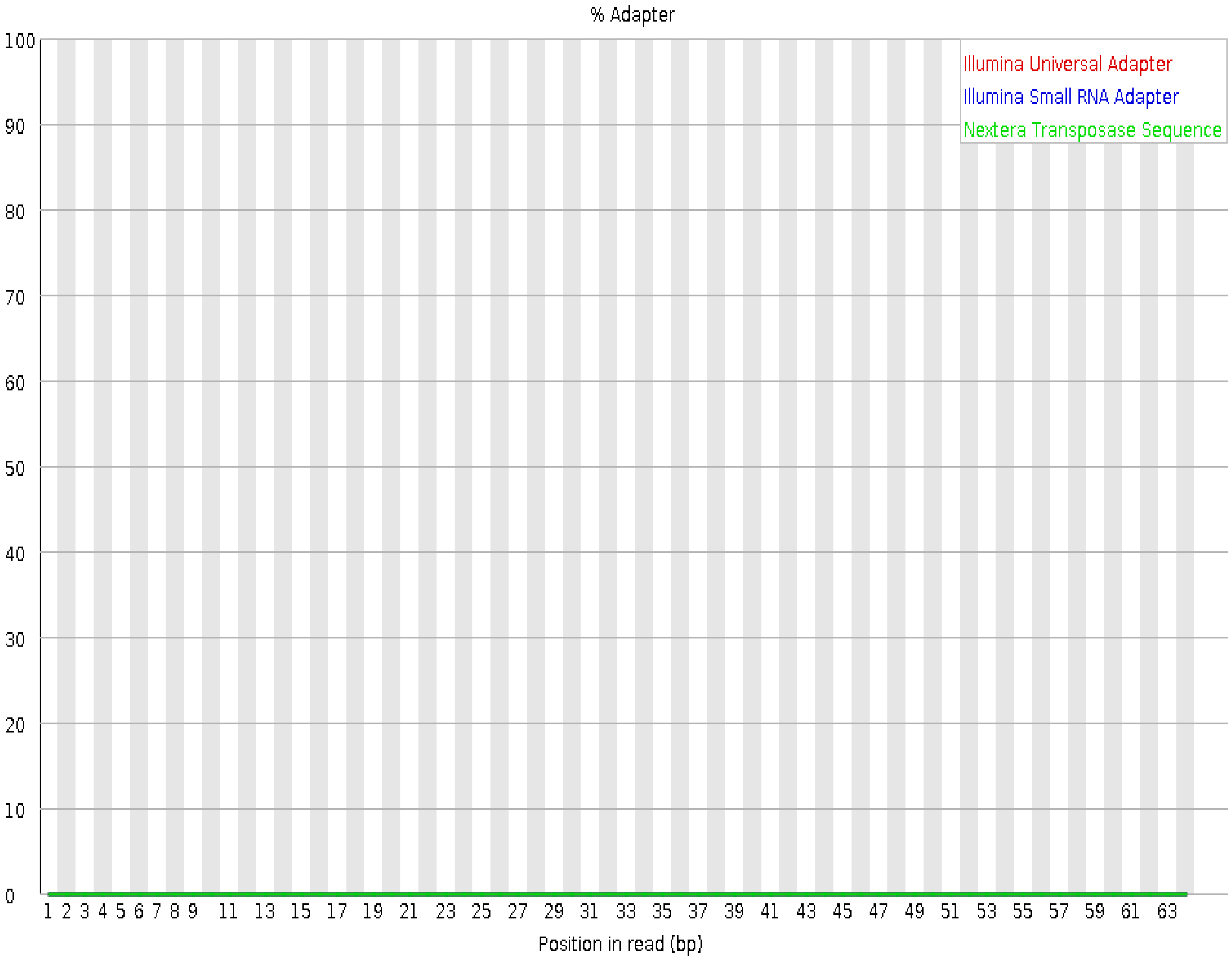


## Kmer Content

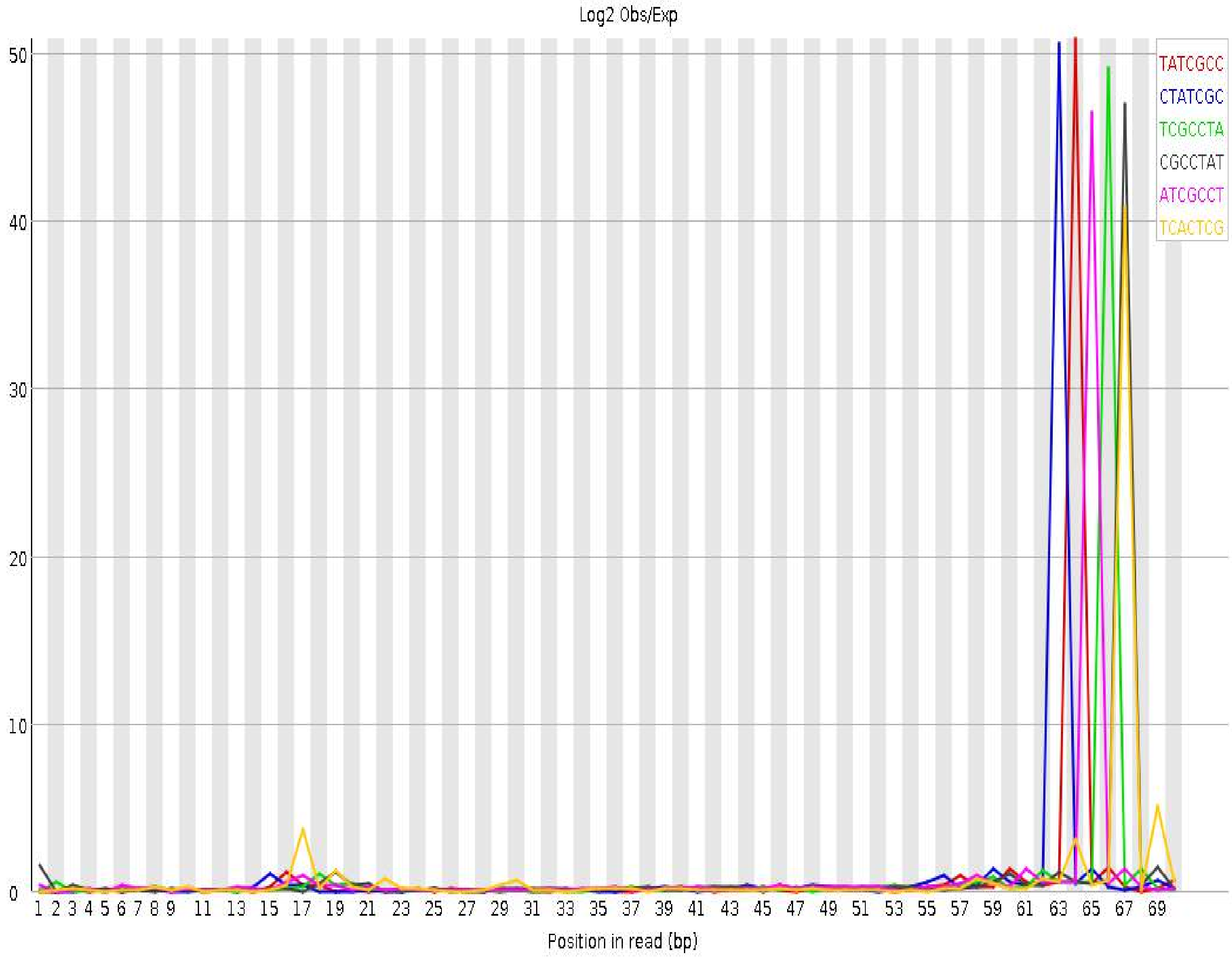


| Sequence | Count | PValue | Obs/Exp Max | Max Obs/Exp Position |
|---|---|---|---|---|
| TATCGCC | 18185 | 0.0 | 50.80289 | 64 |
| CTATCGC | 18315 | 0.0 | 50.53903 | 63 |
| TCGCCTA | 18735 | 0.0 | 49.087563 | 66 |
| CGCCTAT | 19580 | 0.0 | 46.988518 | 67 |
| ATCGCCT | 19895 | 0.0 | 46.48795 | 65 |
| TCACTCG | 56525 | 0.0 | 40.787483 | 67 |
| ACTCGCC | 57260 | 0.0 | 40.22098 | 69 |
| TCTATCG | 24020 | 0.0 | 38.768845 | 62 |
| CGGAACG | 45185 | 0.0 | 35.036407 | 48 |
| TTCACTC | 66985 | 0.0 | 34.495586 | 66 |
| CTCGCCG | 73210 | 0.0 | 34.41544 | 70 |
| CGCGTAA | 12725 | 0.0 | 33.759266 | 1 |
| ACGGCGC | 47090 | 0.0 | 33.449253 | 52 |
| CCCGTCG | 35880 | 0.0 | 33.235207 | 1 |
| TCAGACG | 20920 | 0.0 | 32.968983 | 2 |

| Sequence | Count | PValue | Obs/Exp Max | Max Obs/Exp Position |
|---|---|---|---|---|
| CCGTCGG | 35370 | 0.0 | 32.[illegible] | 2 |
| GCCTATA | 29015 | 0.0 | 32.16[illegible] | 68 |
| CCTTCGG | 50495 | 0.0 | 31.90173 | 44 |
| CTCGCTA | 6055 | 0.0 | 31.397268 | 1 |
| AGGTCGC | 53480 | 0.0 | 31.293455 | 6 |

**Produced by FastQC (version 0.11.2)**

# FastQC Report

Mon 4 May 2015
Biochain_Adult_Kidney_CAGATC_L004_R1.fastq.gz

## Summary


- Basic Statistics
- Per base sequence quality
- Per tile sequence quality
- Per sequence quality scores
- Per base sequence content
- Per sequence GC content
- Per base N content
- Sequence Length Distribution
- Sequence Duplication Levels
- Overrepresented sequences
- Adapter Content
- Kmer Content


## Basic Statistics

| Measure | Value |
|---|---|
| Filename | Biochain_Adult_Kidney_CAGATC_L004_R1.fastq.gz |
| File type | Conventional base calls |
| Encoding | Sanger / Illumina 1.9 |
| Total Sequences | 80053022 |
| Sequences flagged as poor quality | 0 |
| Sequence length | 76 |
| %GC | 51 |

## Per base sequence quality

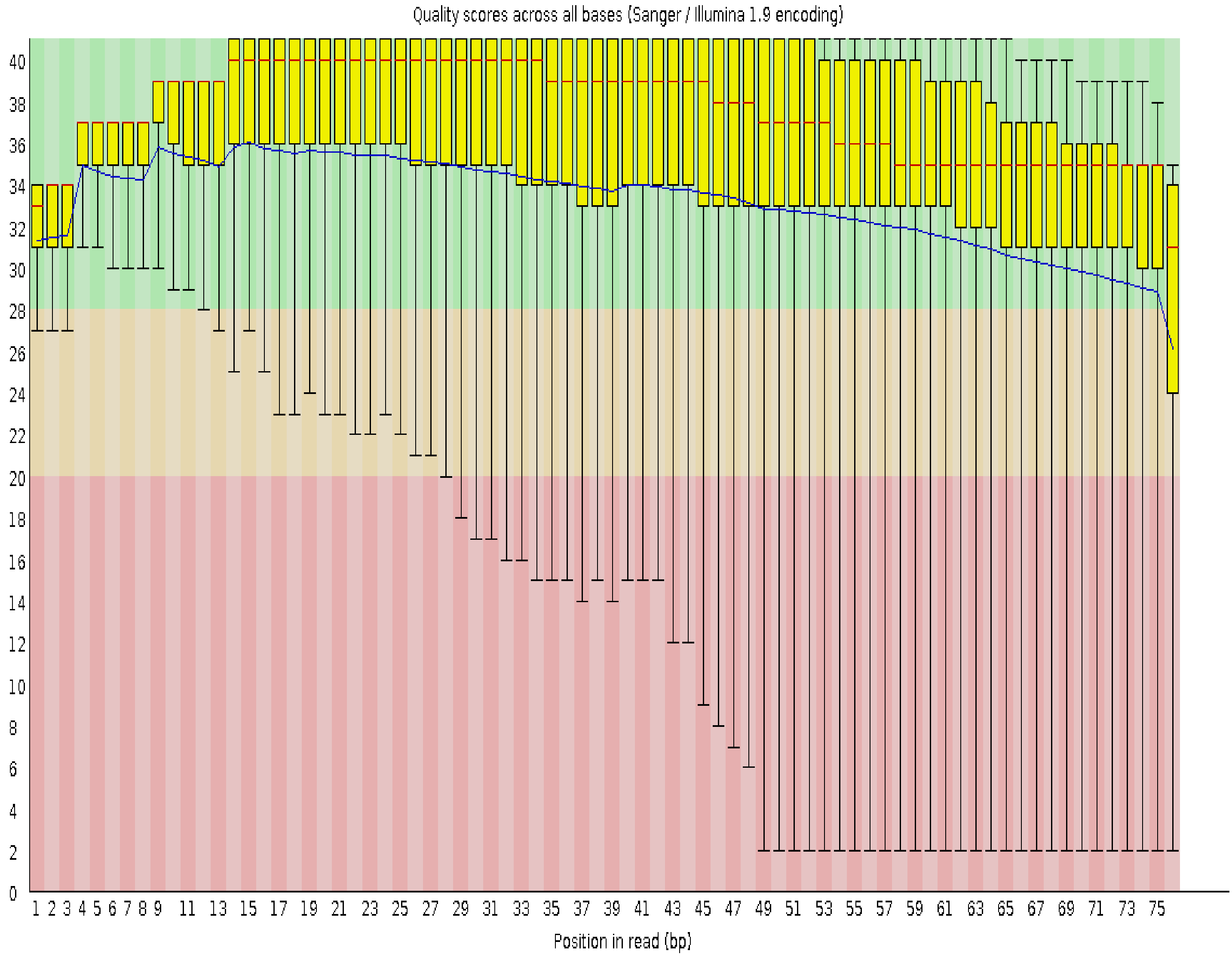


## Per tile sequence quality

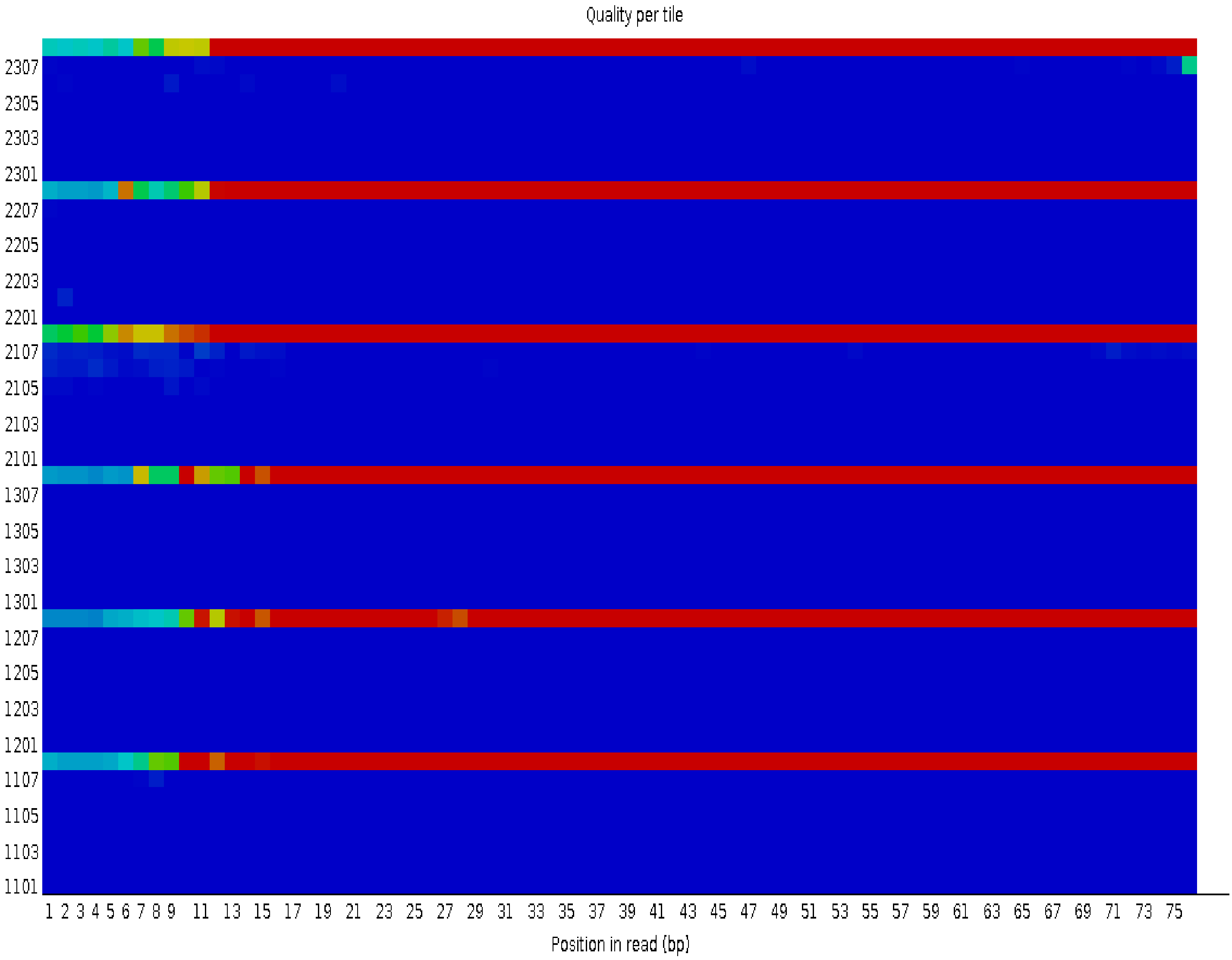


## Per sequence quality scores

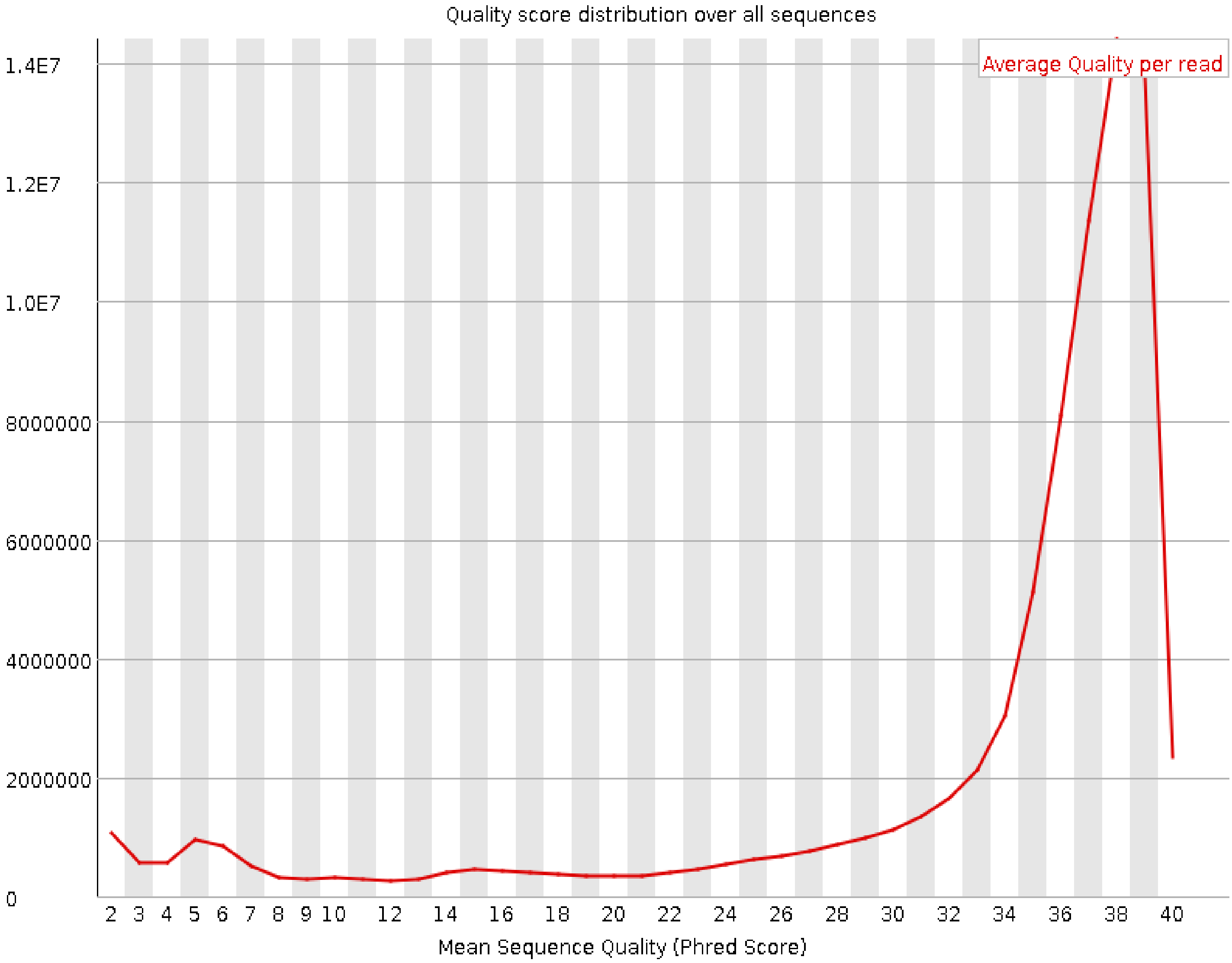


## Per base sequence content

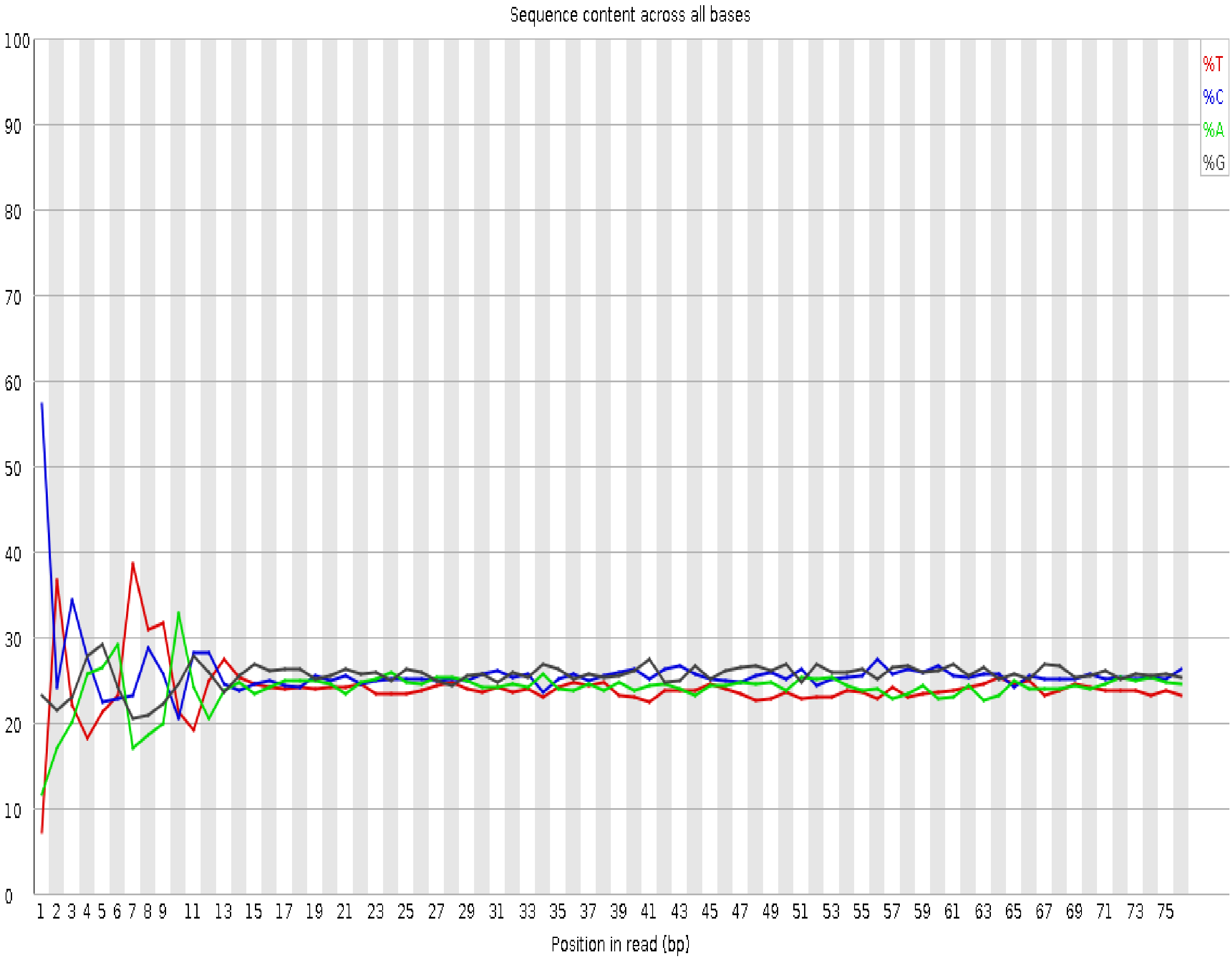


## Per sequence GC content

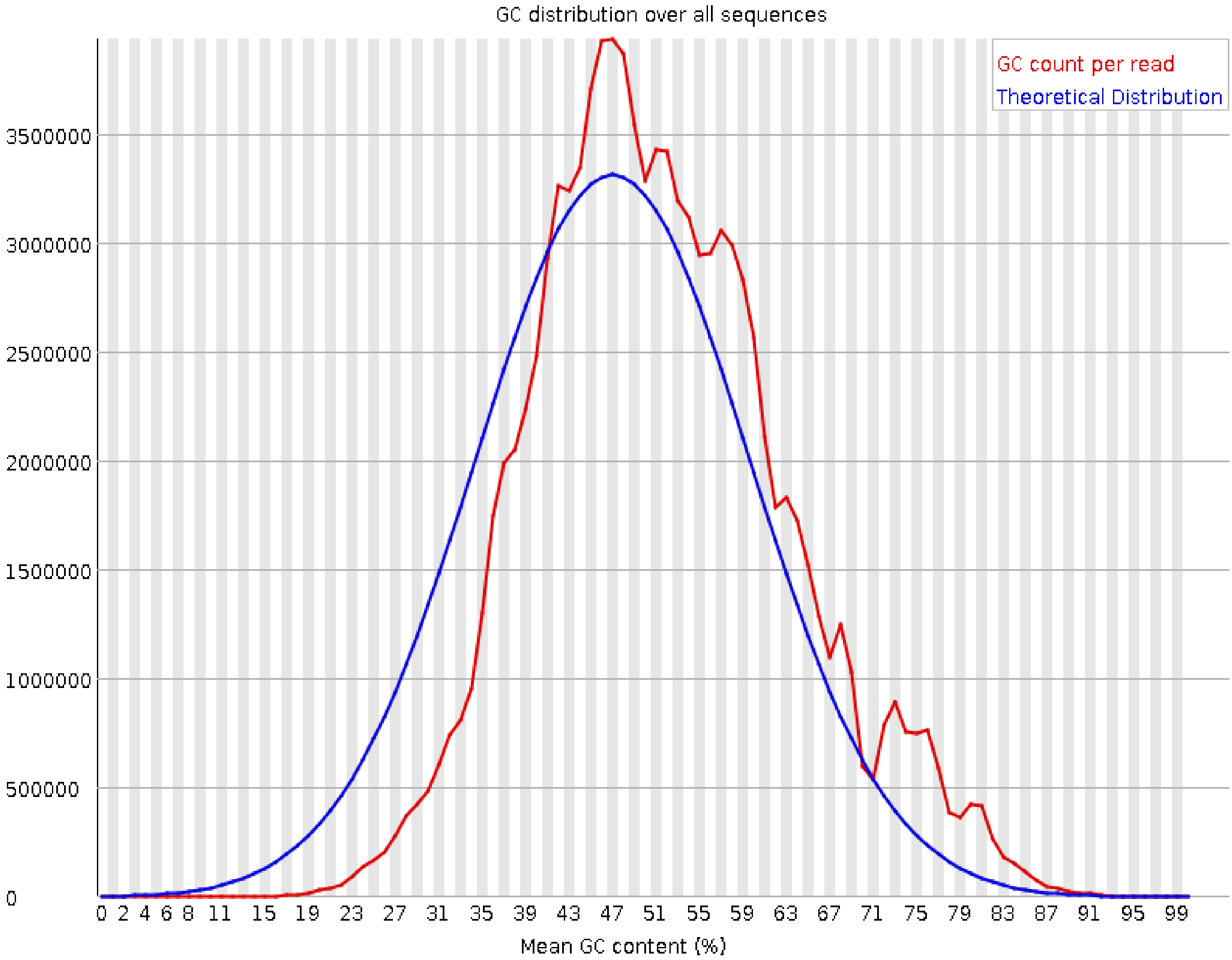


## Per base N content

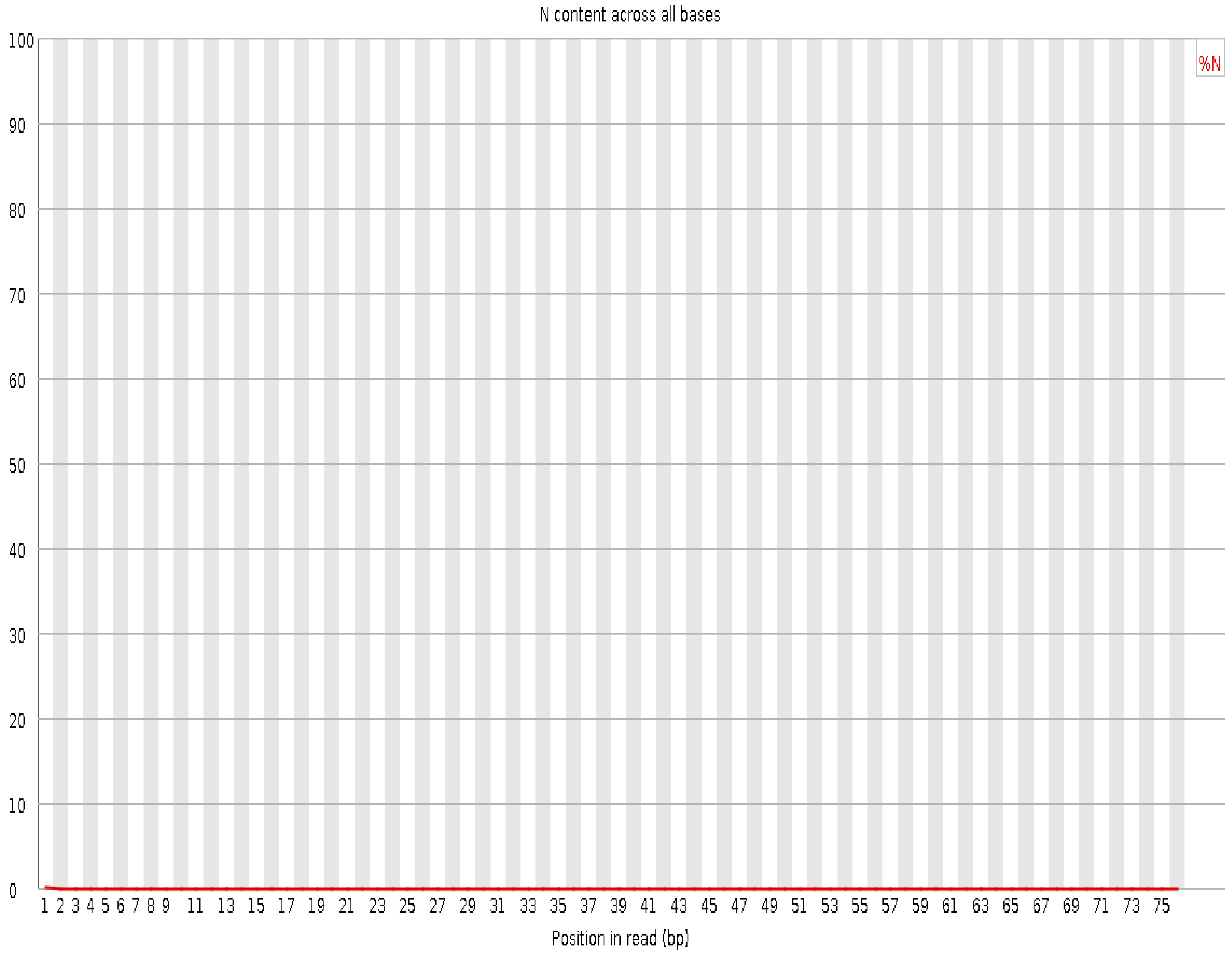


## Sequence Length Distribution

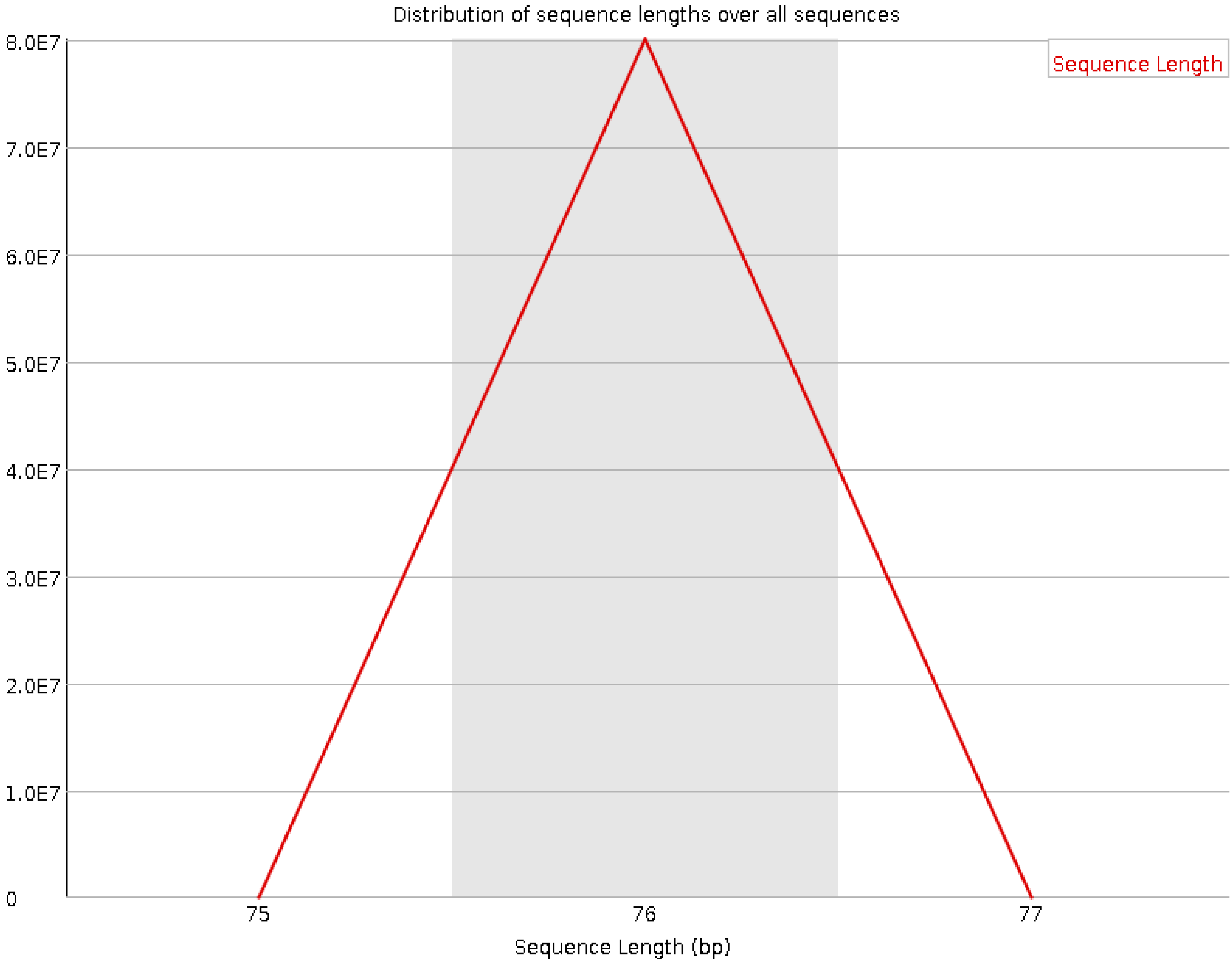


## Sequence Duplication Levels

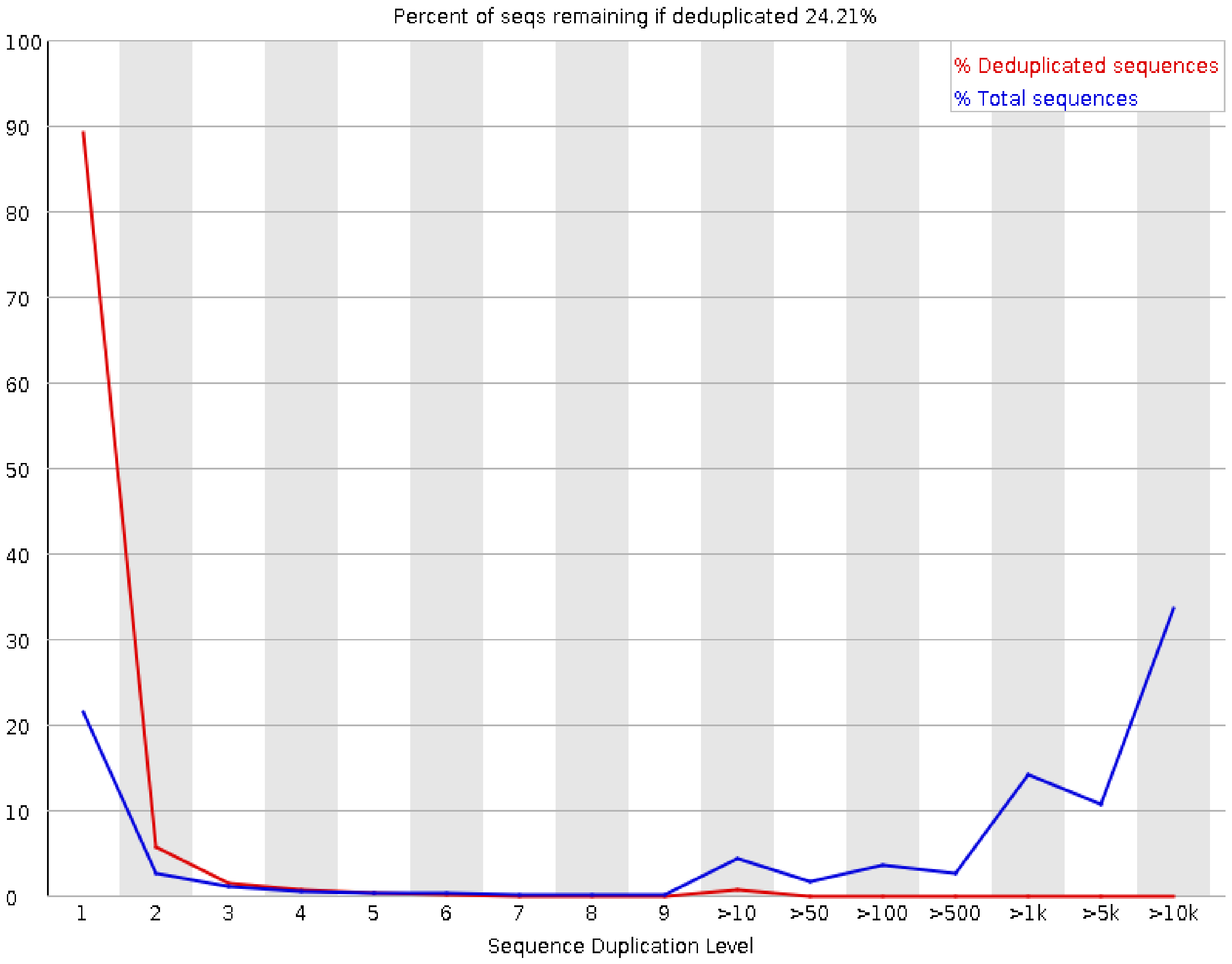


# Overrepresented sequences

| Sequence | Count | Percentage | Possible Source |
|---|---|---|---|
| CCCGTCGGCATGTATTAGCTCTAGAATTACCACAGTTATCCAAGTAGGAG | 294884 | 0.36836085963125786 | No Hit |
| CCTCACCCGGCCCGGACACGGACAGGATTGACAGATTGATAGCTCTTTCT | 225667 | 0.28189691577164944 | No Hit |
| CGCAGTTTTATCCGGTAAAGCGAATGATTAGAGGTCTTGGGGCCGAAACG | 206285 | 0.2576854625175799 | No Hit |
| CTCCCTTTCGATCGGCCGAGGGCAACGGAGGCCATCGCCCGTCCCTTCGG | 193117 | 0.2412363645684731 | No Hit |
| CTTCCGTACGCCACATGTCCCGCGCCCCGCCGCGGGGCGGGGATTCGGCG | 180492 | 0.22546556705879262 | No Hit |
| CTGCCAGTAGCATATGCTTGTCTCAAAGATTAAGCCATGCATGTCTAAGT | 179318 | 0.2239990390369023 | No Hit |
| CTTGGTTATAATTTTTCATCTTTCCCTTGCGGTACTATATCTATTGCGCC | 174185 | 0.2175870387503922 | No Hit |
| CGCGTAACTAGTTAGCATGCCAGAGTCTCGTTCGTTATCGGAATTAACCA | 164543 | 0.2055425215552762 | No Hit |
| CTCCGACTTTCGTTCTTGATTAATGAAAACATTCTTGGCAAATGCTTTCG | 155221 | 0.19389773942575209 | No Hit |
| CAAAGATTAAGCCATGCATGTCTAAGTACGCACGGCCGGTACAGTGAAAC | 140523 | 0.17553740819428404 | No Hit |
| CTCACCCGGCCCGGACACGGACAGGATTGACAGATTGATAGCTCTTTCTC | 132025 | 0.16492194385865908 | No Hit |

| Sequence | Count | Percentage | Possible Source |
|---|---|---|---|
| CTCGCATTCCACGCCCGGCTCCACGCCAGCGAGCCGGGCTTCTTACCCAT | 131236 | 0.16393634708755905 | No Hit |
| CCGGTATTTAGCCTTAGATGGAGTTTACCACCCGCTTTGGGCTGCATTCC | 128967 | 0.16110197563809645 | [illegible] |
| CCCGAAGTTACGGATCCGGCTTGCCGACTTCCCTTACCTACATTGTTCCA | 114528 | 0.14306517997534185 | No Hit |
| CGGGTCTTCCGTACGCCACATGTCCCGCGCCCCGCCGCGGGGCGGGGATT | 99680 | 0.12451747293187757 | No Hit |
| CCTGCCAGTAGCATATGCTTGTCTCAAAGATTAAGCCATGCATGTCTAAG | 99057 | 0.12373923872605334 | No Hit |
| GTTAATTGTCAGTTCAGTGTTTTAATCTGACGCAGGCTTATGCGGAGGAG | 98360 | 0.12286856578631099 | No Hit |
| CTTCCGTCAATTCCTTTAAGTTTCAGCTTTGCAACCATACTCCCCCCGGA | 93951 | 0.11736096608570255 | No Hit |
| CCGTCGGCATGTATTAGCTCTAGAATTACCACAGTTATCCAAGTAGGAGA | 92989 | 0.11615926254476688 | No Hit |
| CTGCTGTCTATATCAACCAACACCTTTTCTGGGGTCTGATGAGCGTCGGC | 92587 | 0.11565709536861705 | No Hit |
| CTCCGCCACTCCGGATTCGGGGATCTGAACCCGACTCCCTTTCGATCGGC | 92321 | 0.1153248155953438 | No Hit |
| CTGGATAGTAGGTAGGGACAGTGGGAATCTCGTTCATCCATTCATGCGCG | 91270 | 0.11401193573929039 | No Hit |
| CTCTCATGTCTCTTCACCGTGCCAGACTAGAGTCAAGCTCAACAGGGTCT | 89156 | 0.111371185962223 9 | No Hit |
| CTCCGAGGTCGCCCCAACCGAAATTTTTAATGCAGGTTTGGTAGTTTAGG | 86390 | 0.10791597598901388 | No Hit |
| CCCTGTGGTAACTTTTCTGACACCTCCTGCTTAAAACCCAAAAGGTCAGA | 85302 | 0.10655687676600141 | No Hit |
| CTCGATCAGAAGGACTTGGGCCCCCCACGAGCGGCGCCGGGGAGCGGGTC | 83299 | 0.1040547850898121 | No Hit |
| GCCCTCTTGAACTCTCTCTTCAAAGTTCTTTTCAACTTTCCCTTACGGTA | 83145 | 0.103862412589496 | No Hit |
| CTCTCTTCAAAGTTCTTTTCAACTTTCCCTTACGGTACTTGTTGACTATC | 82707 | 0.10331527521846708 | No Hit |
| ATCAGACGTGGCGACCCGCTGAATTTAAGCATATTAGTCAGCGGAGGAAA | 82049 | 0.10249331998984372 | No Hit |
| AAAGAAACTAACCAGGATTCCCTCAGTAACGGCGAGTGAACAGGGAAGAG | 81944 | 0.10236215692144639 | No Hit |
| CGAGAACTTTGAAGGCCGAAGTGGAGAAGGGTTCCATGTGAACAGCAGTT | 80168 | 0.10014362730741133 | No Hit |

## Adapter Content

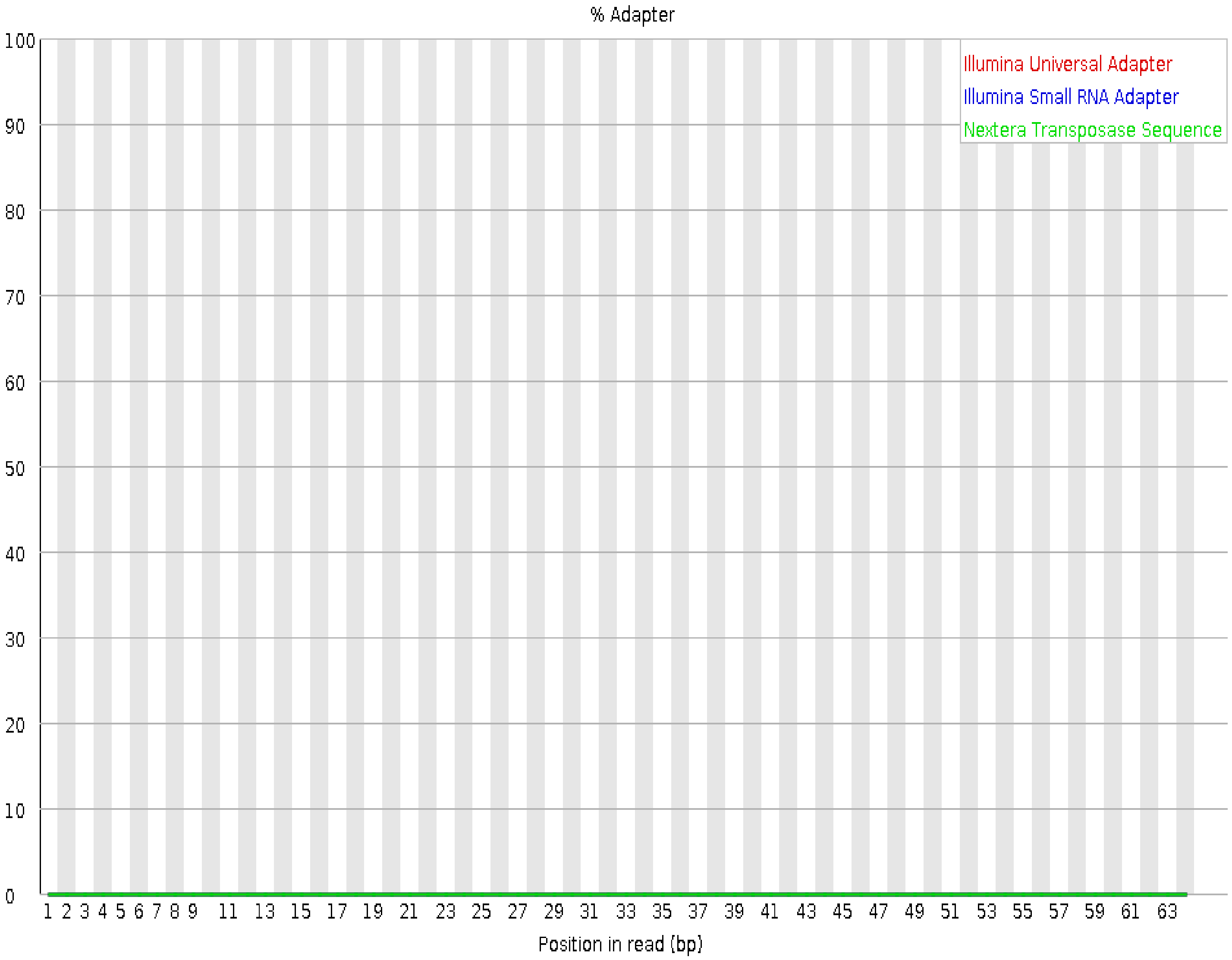


## Kmer Content

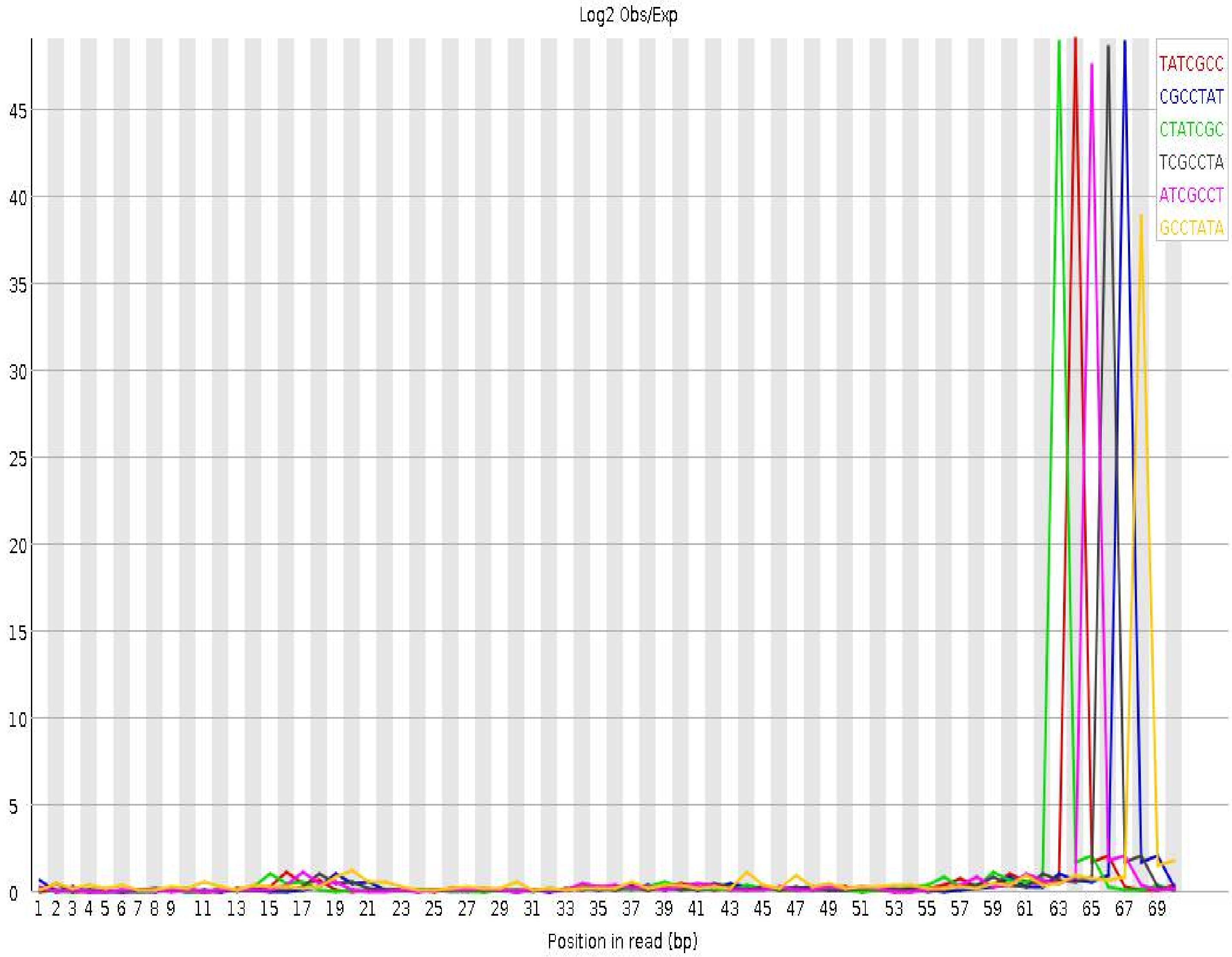


| Sequence | Count | PValue | Obs/Exp Max | Max Obs/Exp Position |
|---|---|---|---|---|
| TATCGCC | 29475 | 0.0 | 49.034836 | 64 |
| CGCCTAT | 29535 | 0.0 | 48.88712 | 67 |
| CTATCGC | 29655 | 0.0 | 48.886627 | 63 |
| TCGCCTA | 29685 | 0.0 | 48.652428 | 66 |
| ATCGCCT | 30390 | 0.0 | 47.615864 | 65 |
| GCCTATA | 37525 | 0.0 | 38.879242 | 68 |
| TCTATCG | 38920 | 0.0 | 37.60396 | 62 |
| CCCGTCG | 67780 | 0.0 | 37.576538 | 1 |
| ACTCGCC | 40995 | 0.0 | 36.860184 | 69 |
| TCACTCG | 42495 | 0.0 | 35.245865 | 67 |
| CCGTCGG | 73220 | 0.0 | 34.250355 | 2 |
| CGCGTAA | 44430 | 0.0 | 32.562977 | 1 |
| TGCCGTA | 11040 | 0.0 | 32.405674 | 2 |
| GCCAGTA | 47525 | 0.0 | 31.275614 | 3 |
| GCGTAAC | 46775 | 0.0 | 31.192715 | 2 |

| Sequence | Count | PValue | Obs/Exp Max | Max Obs/Exp Position |
|---|---|---|---|---|
| TTCGCTC | 41565 | 0.0 | 31.[illegible] | 47 |
| CCAGTAG | 47275 | 0.0 | 30.9[illegible] | 4 |
| CGGAACG | 50645 | 0.0 | 30.845581 | 48 |
| CGCACTT | 11380 | 0.0 | 30.669086 | 3 |
| ACGGCGC | 53695 | 0.0 | 30.243435 | 52 |

**Produced by FastQC (version 0.11.2)**

# FastQC Report

Mon 4 May 2015
Biochain_Adult_Kidney_CAGATC_L004_R2.fastq.gz

## Summary


- Basic Statistics
- Per base sequence quality
- Per tile sequence quality
- Per sequence quality scores
- Per base sequence content
- Per sequence GC content
- Per base N content
- Sequence Length Distribution
- Sequence Duplication Levels
- Overrepresented sequences
- Adapter Content
- Kmer Content


## Basic Statistics

| Measure | Value |
|---|---|
| Filename | Biochain_Adult_Kidney_CAGATC_L004_R2.fastq.gz |
| File type | Conventional base calls |
| Encoding | Sanger / Illumina 1.9 |
| Total Sequences | 80053022 |
| Sequences flagged as poor quality | 0 |
| Sequence length | 76 |
| %GC | 51 |

## Per base sequence quality

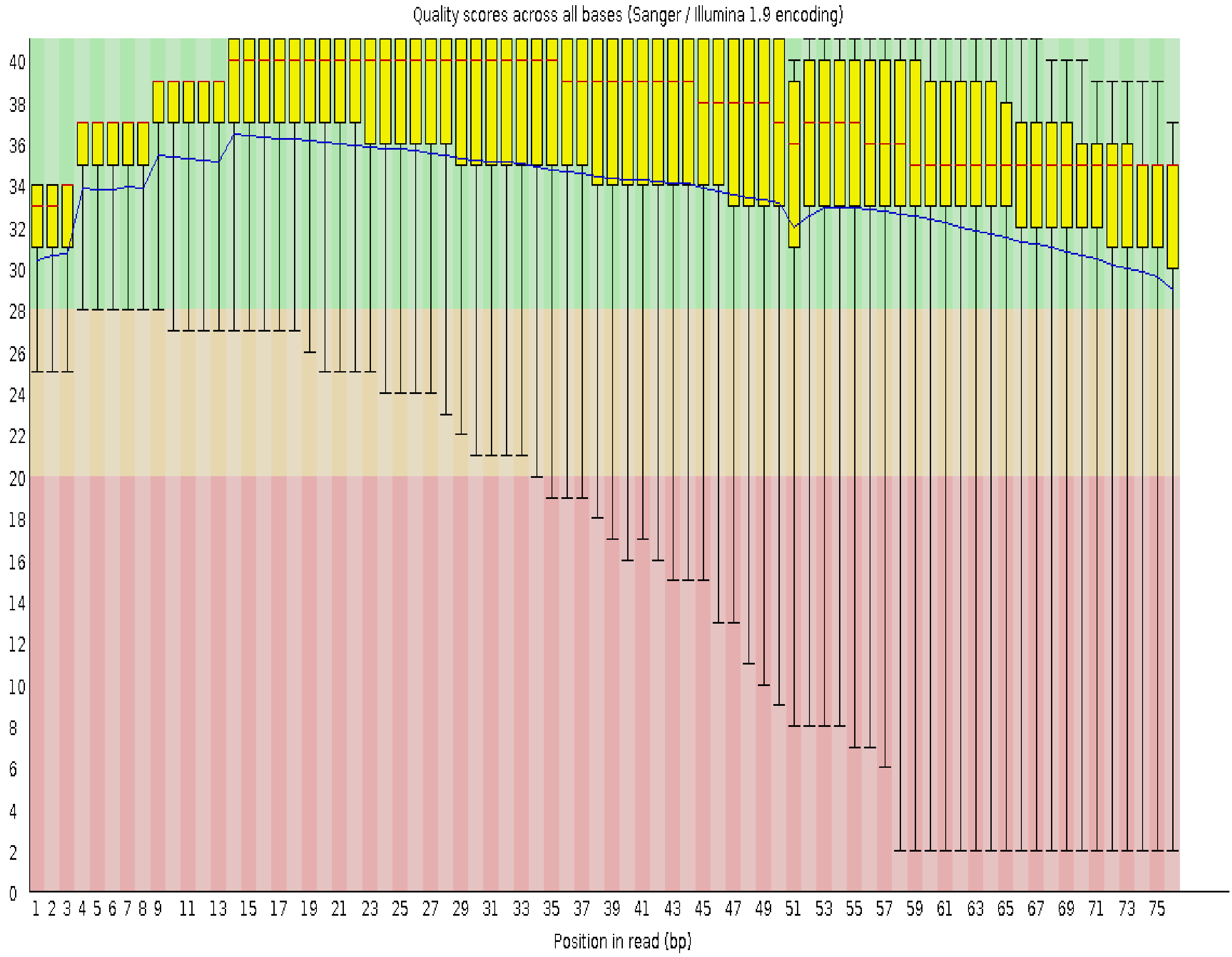


## Per tile sequence quality

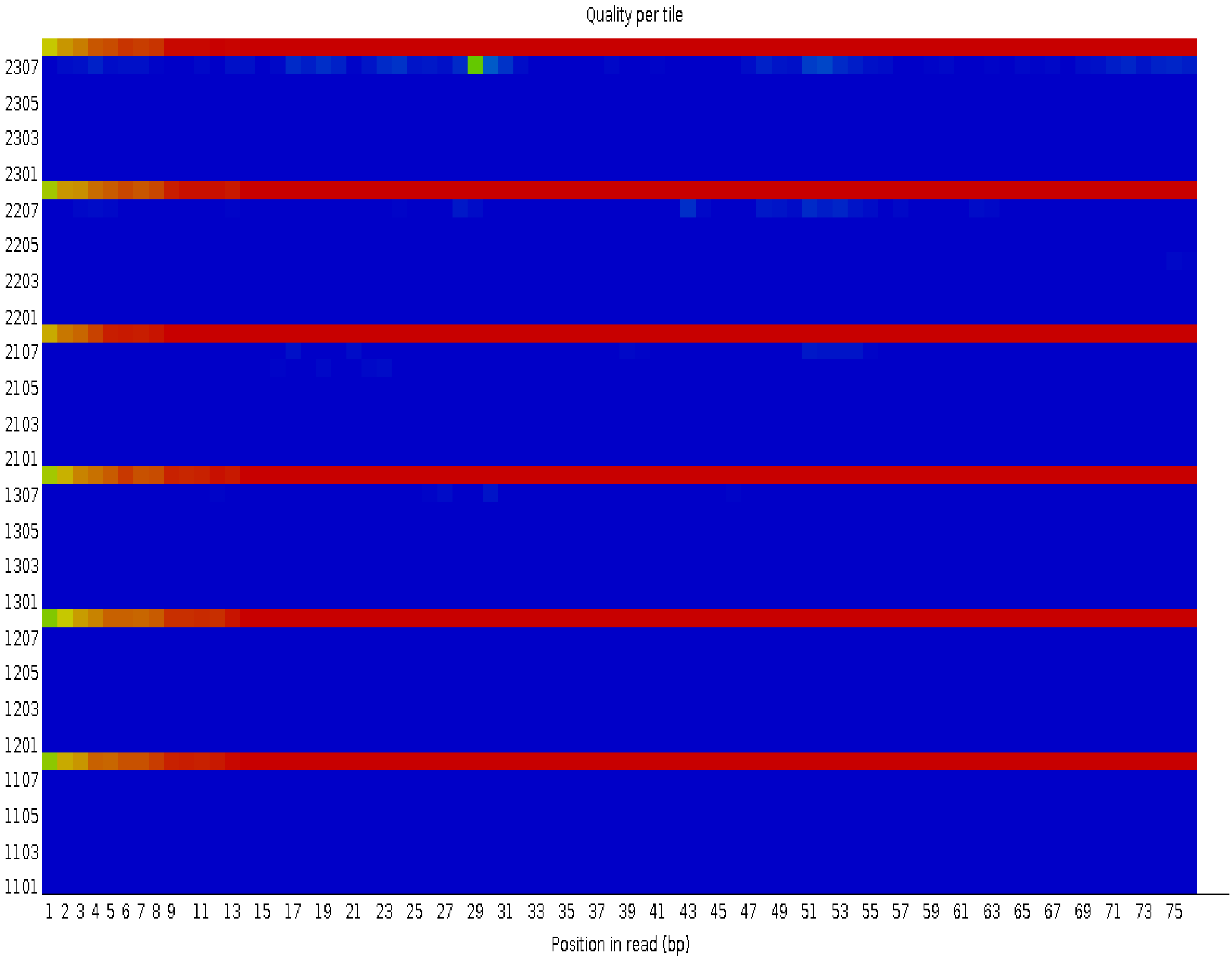


## Per sequence quality scores

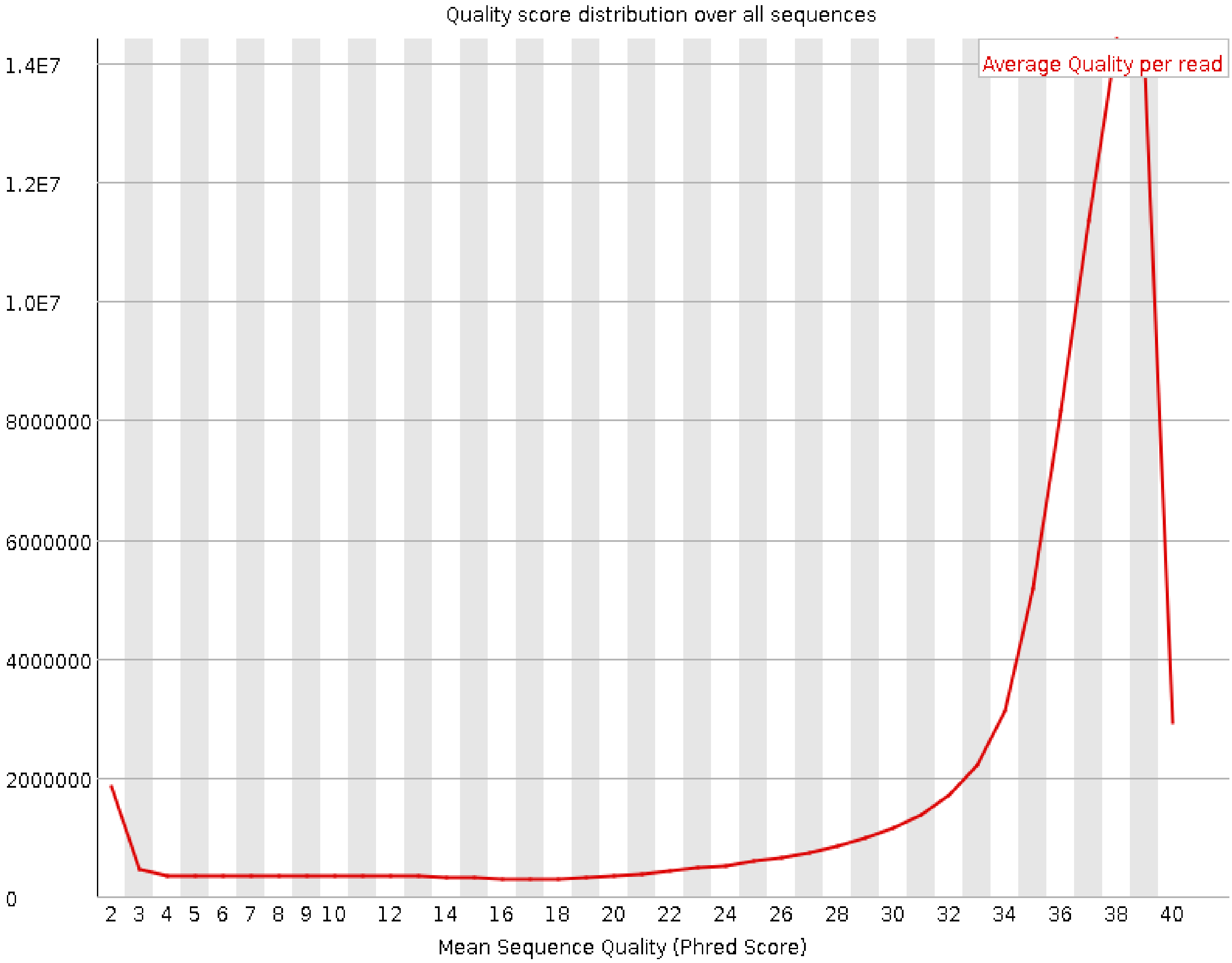


## Per base sequence content

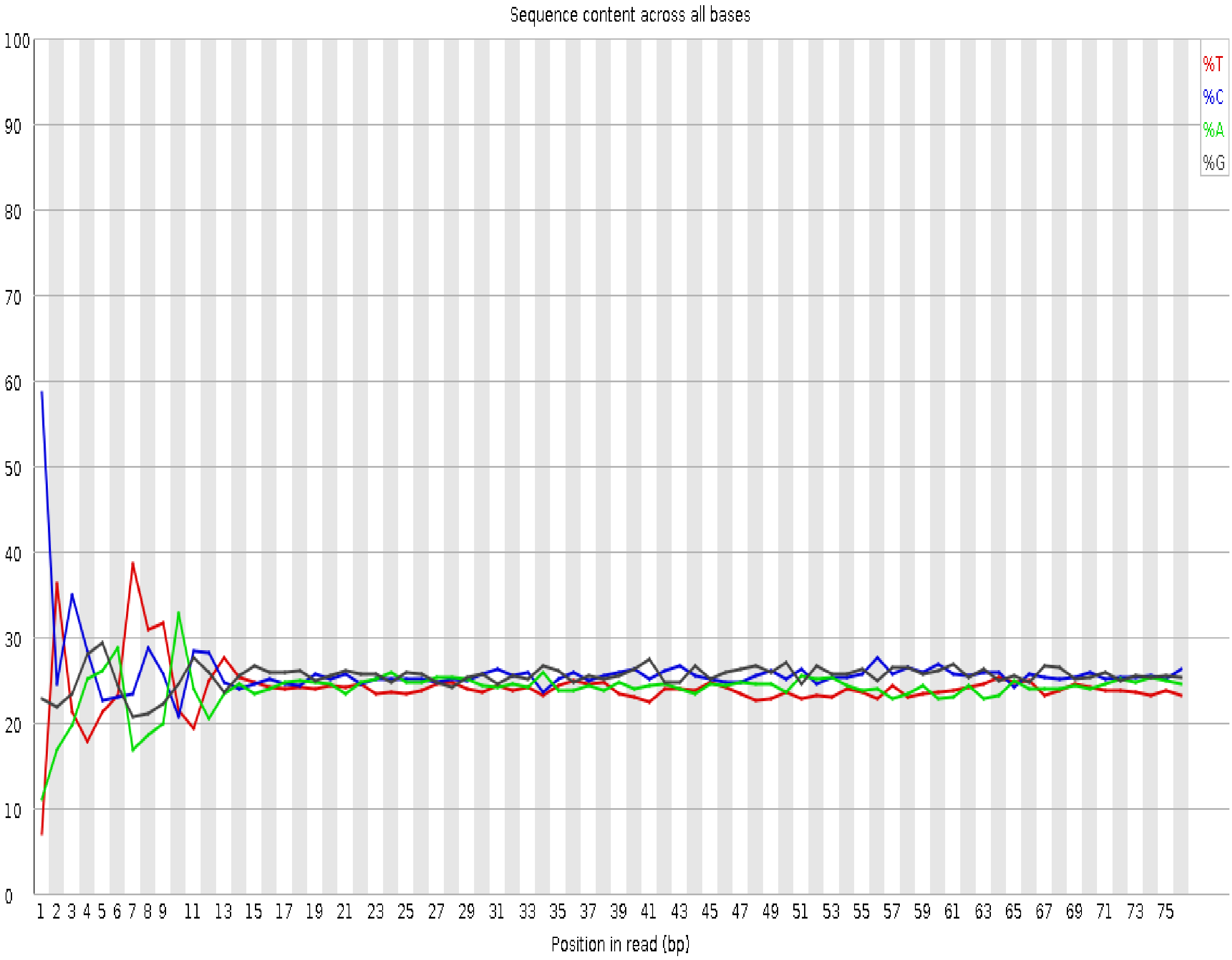


## Per sequence GC content

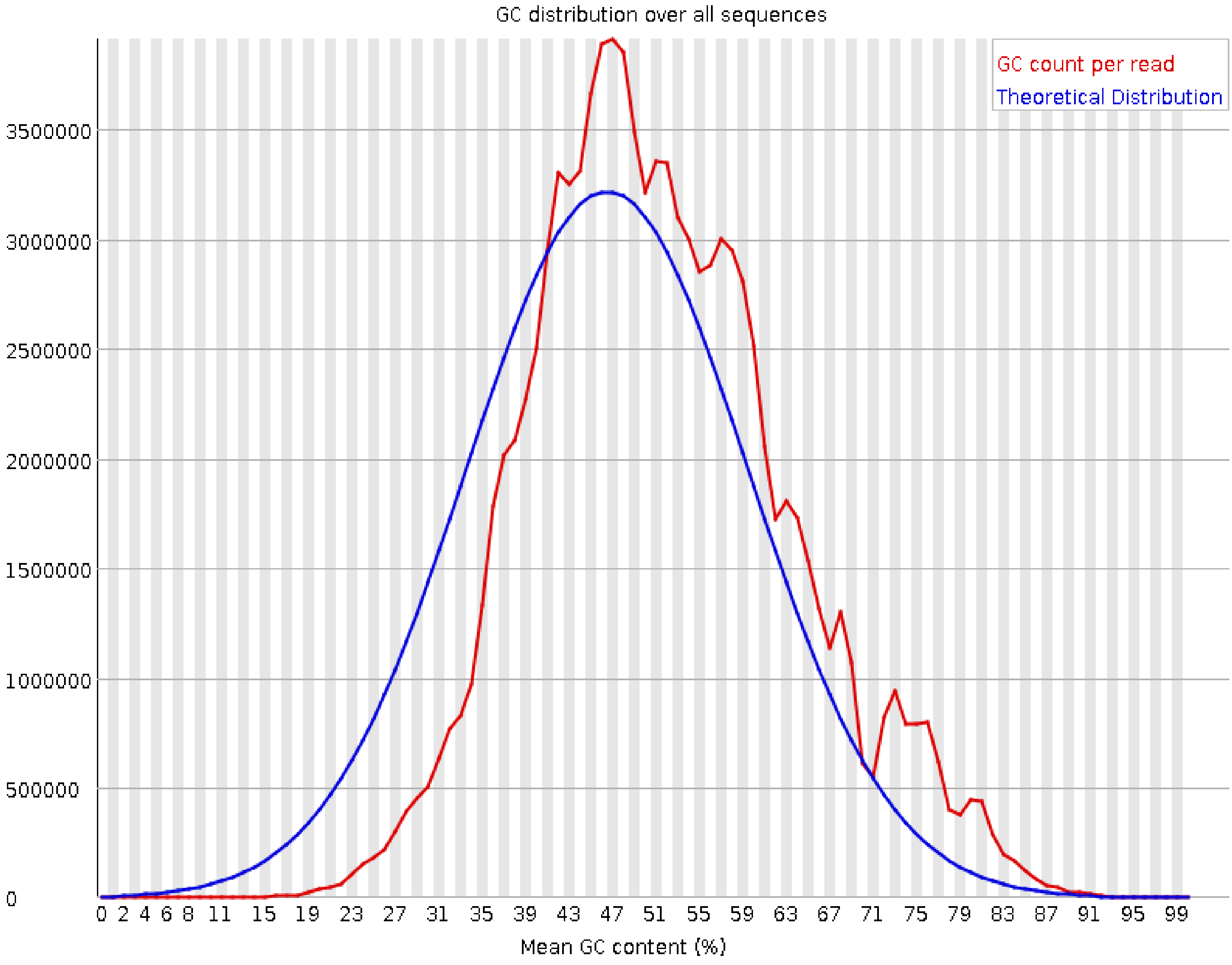


## Per base N content

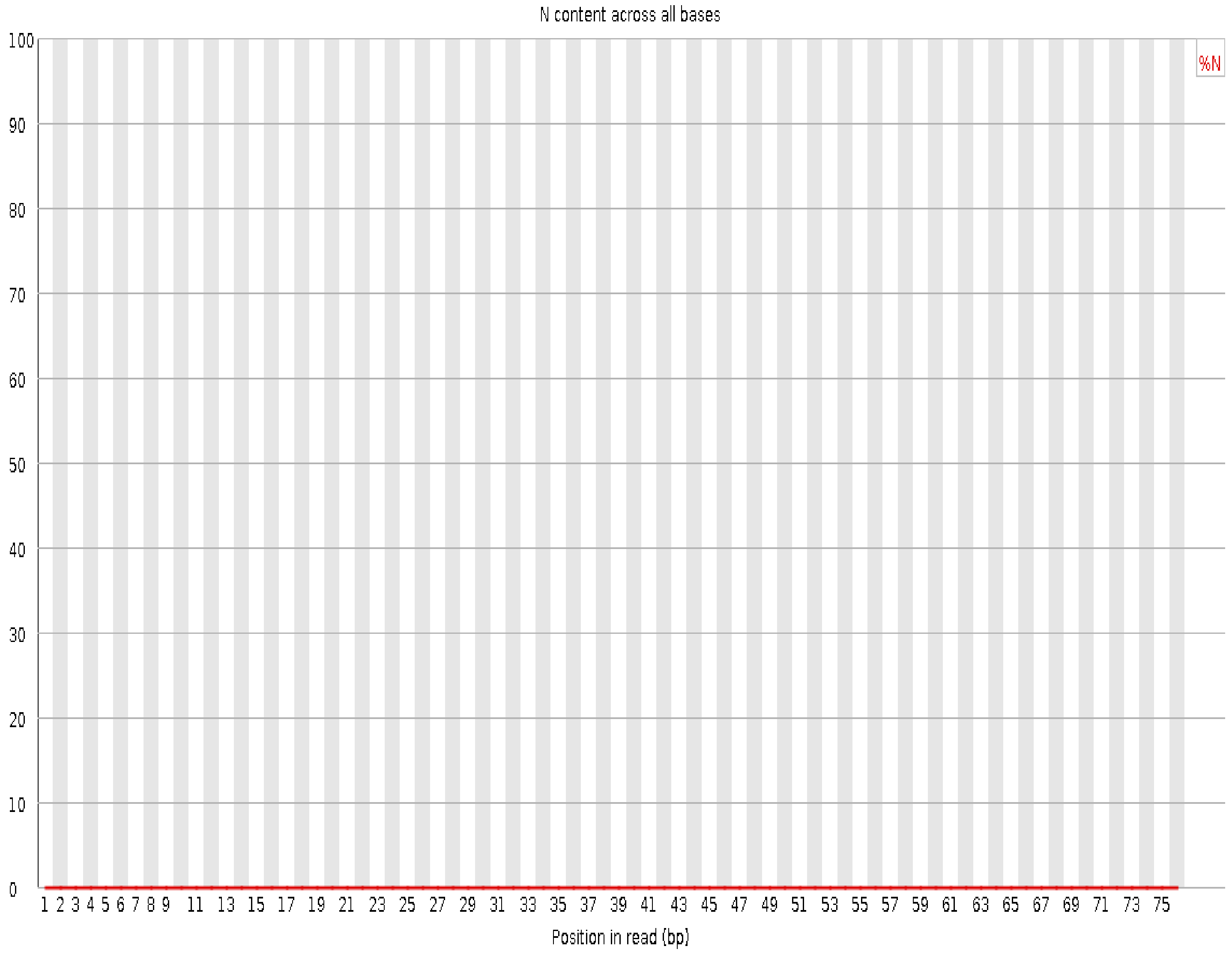


## Sequence Length Distribution

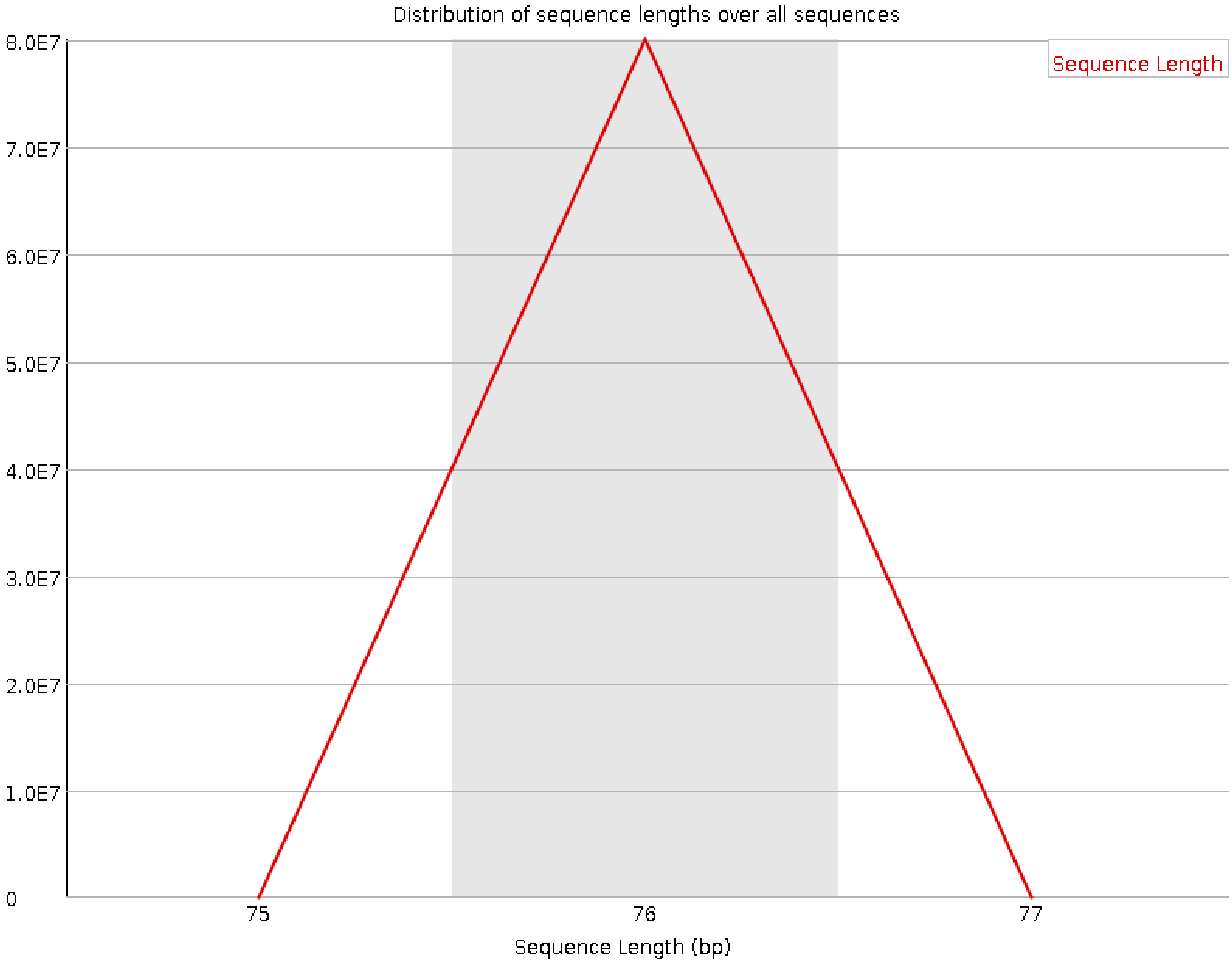


## Sequence Duplication Levels

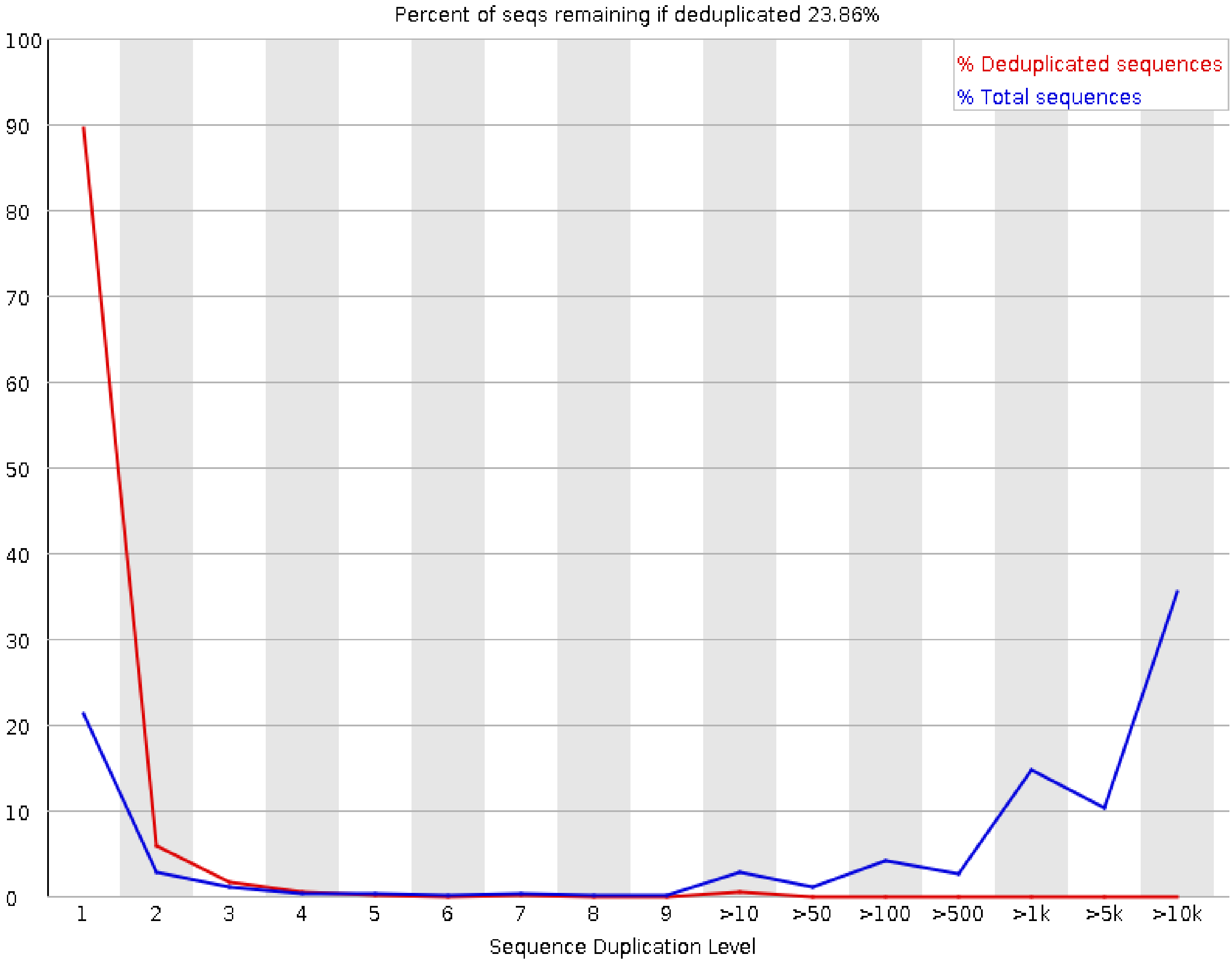


## Overrepresented sequences

| Sequence | Count | Percentage | Possible Source |
|---|---|---|---|
| CCCGTCGGCATGTATTAGCTCTAGAATTACCACAGTTATCCAAGTAGGAG | 336390 | 0.42020899598268757 | No Hit |
| CCTCACCCGGCCCGGACACGGACAGGATTGACAGATTGATAGCTCTTTCT | 236803 | 0.2958076960542476 | No Hit |
| CGCAGTTTTATCCGGTAAAGCGAATGATTAGAGGTCTTGGGGCCGAAACG | 228367 | 0.2852696803875811 | No Hit |
| CTCCCTTTCGATCGGCCGAGGGCAACGGAGGCCATCGCCCGTCCCTTCGG | 225872 | 0.2821529960480443 | No Hit |
| CTTCCGTACGCCACATGTCCCGCGCCCCGCCGCGGGGCGGGGATTCGGCG | 196256 | 0.2451575157275137 | No Hit |
| CTGCCAGTAGCATATGCTTGTCTCAAAGATTAAGCCATGCATGTCTAAGT | 189386 | 0.23657570353808755 | No Hit |
| CTCCGACTTTCGTTCTTGATTAATGAAAACATTCTTGGCAAATGCTTTCG | 188790 | 0.2358311969784226 | No Hit |
| CGCGTAACTAGTTAGCATGCCAGAGTCTCGTTCGTTATCGGAATTAACCA | 172369 | 0.21531854225315816 | No Hit |
| CTTGGTTATAATTTTTCATCTTTCCCTTGCGGTACTATATCTATTGCGCC | 164892 | 0.20597848261118737 | No Hit |
| CAAAGATTAAGCCATGCATGTCTAAGTACGCACGGCCGGTACAGTGAAAC | 139399 | 0.17413333877639248 | No Hit |
| CTCACCCGGCCCGGACACGGACAGGATTGACAGATTGATAGCTCTTTCTC | 138873 | 0.17347627426232579 | No Hit |

| Sequence | Count | Percentage | Possible Source |
|---|---|---|---|
| CTCGCATTCCACGCCCGGCTCCACGCCAGCGAGCCGGGCTTCTTACCCAT | 133369 | 0.16660083113414506 | No Hit |
| CCGGTATTTAGCCTTAGATGGAGTTTACCACCCGCTTTGGGCTGCATTCC | 132965 | 0.1660961656138353 | No Hit |
| CGGGTCTTCCGTACGCCACATGTCCCGCGCCCCGCCGCGGGGCGGGGATT | 121578 | 0.15187184313916344 | No Hit |
| CTCCGCCACTCCGGATTCGGGGATCTGAACCCGACTCCCTTTCGATCGGC | 111991 | 0.13989603040844603 | No Hit |
| CCCGAAGTTACGGATCCGGCTTGCCGACTTCCCTTACCTACATTGTTCCA | 106423 | 0.13294064026714694 | No Hit |
| CCGTCGGCATGTATTAGCTCTAGAATTACCACAGTTATCCAAGTAGGAGA | 102150 | 0.12760292796941508 | No Hit |
| CTTCCGTCAATTCCTTTAAGTTTCAGCTTTGCAACCATACTCCCCCCGGA | 100704 | 0.12579662514177167 | No Hit |
| CTCCGAGGTCGCCCCAACCGAAATTTTTAATGCAGGTTTGGTAGTTTAGG | 99595 | 0.12441129330507973 | No Hit |
| CCTGCCAGTAGCATATGCTTGTCTCAAAGATTAAGCCATGCATGTCTAAG | 99473 | 0.12425889431132281 | No Hit |
| CTGGATAGTAGGTAGGGACAGTGGGAATCTCGTTCATCCATTCATGCGCG | 98550 | 0.12310590848150617 | No Hit |
| CTCTCATGTCTCTTCACCGTGCCAGACTAGAGTCAAGCTCAACAGGGTCT | 97402 | 0.12167185893369521 | No Hit |
| CTGCTGTCTATATCAACCAACACCTTTTCTGGGGTCTGATGAGCGTCGGC | 96472 | 0.12051012889931875 | No Hit |
| GTTAATTGTCAGTTCAGTGTTTTAATCTGACGCAGGCTTATGCGGAGGAG | 94594 | 0.11816418373312627 | No Hit |
| CCCTGTGGTAACTTTTCTGACACCTCCTGCTTAAAACCCAAAAGGTCAGA | 94408 | 0.11793183772625099 | No Hit |
| CTCGATCAGAAGGACTTGGGCCCCCCACGAGCGGCGCCGGGGAGCGGGTC | 89291 | 0.11153982419302047 | No Hit |
| CTTCACCGTGCCAGACTAGAGTCAAGCTCAACAGGGTCTTCTTTCCCCGC | 89221 | 0.11145238214742224 | No Hit |
| GCCCTCTTGAACTCTCTCTTCAAAGTTCTTTTCAACTTTCCCTTACGGTA | 87260 | 0.10900275569859187 | No Hit |
| CTCTCTTCAAAGTTCTTTTCAACTTTCCCTTACGGTACTTGTTGACTATC | 83729 | 0.10459192908420122 | No Hit |
| CACCGTGCCAGACTAGAGTCAAGCTCAACAGGGTCTTCTTTCCCCGCTGA | 82495 | 0.10305045073751243 | No Hit |
| CTCCCGTCCACTCTCGACTGCCGGCGACGGCCGGGTATGGGCCCGACGCT | 81010 | 0.10119543019875002 | No Hit |

## Adapter Content

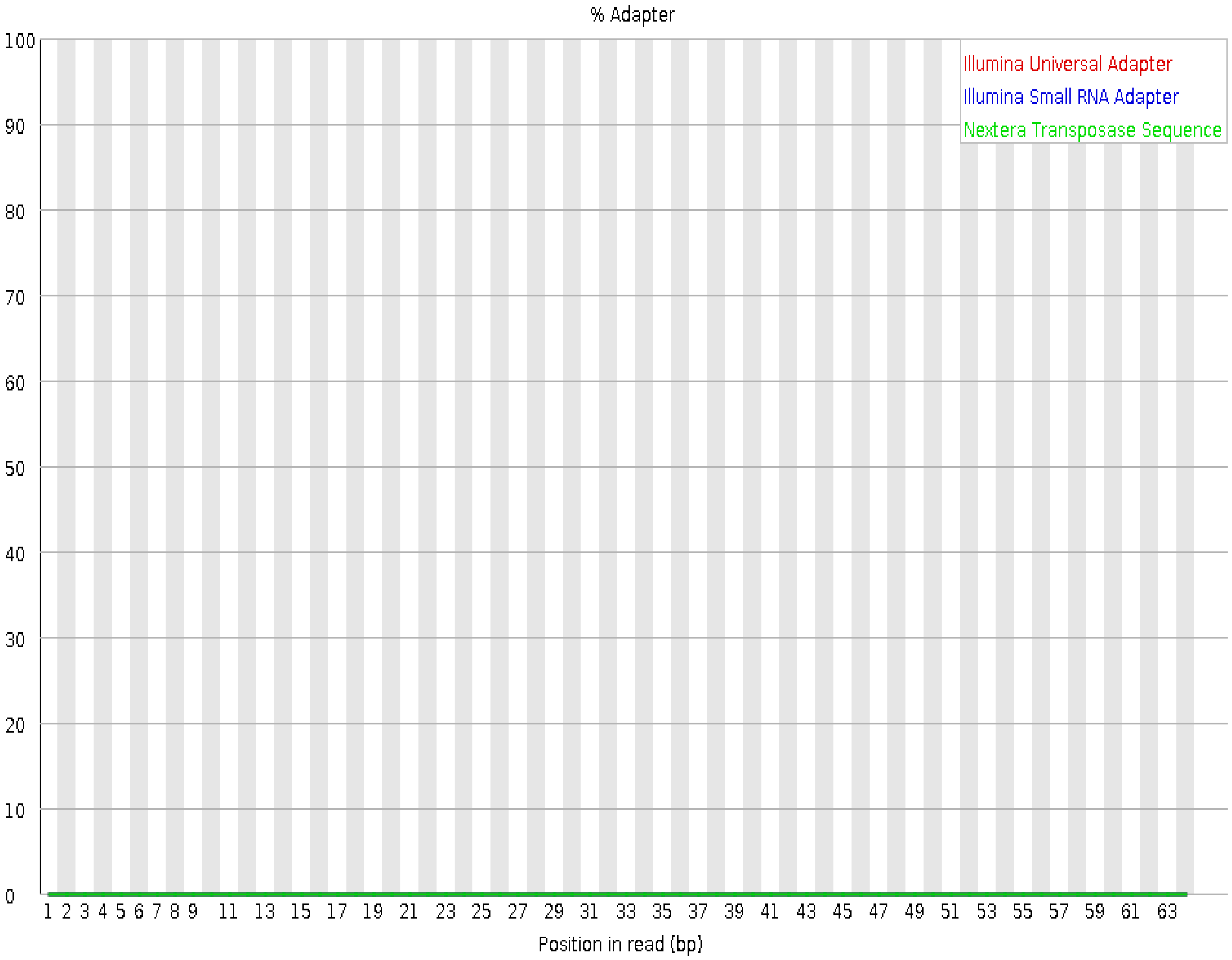


## Kmer Content

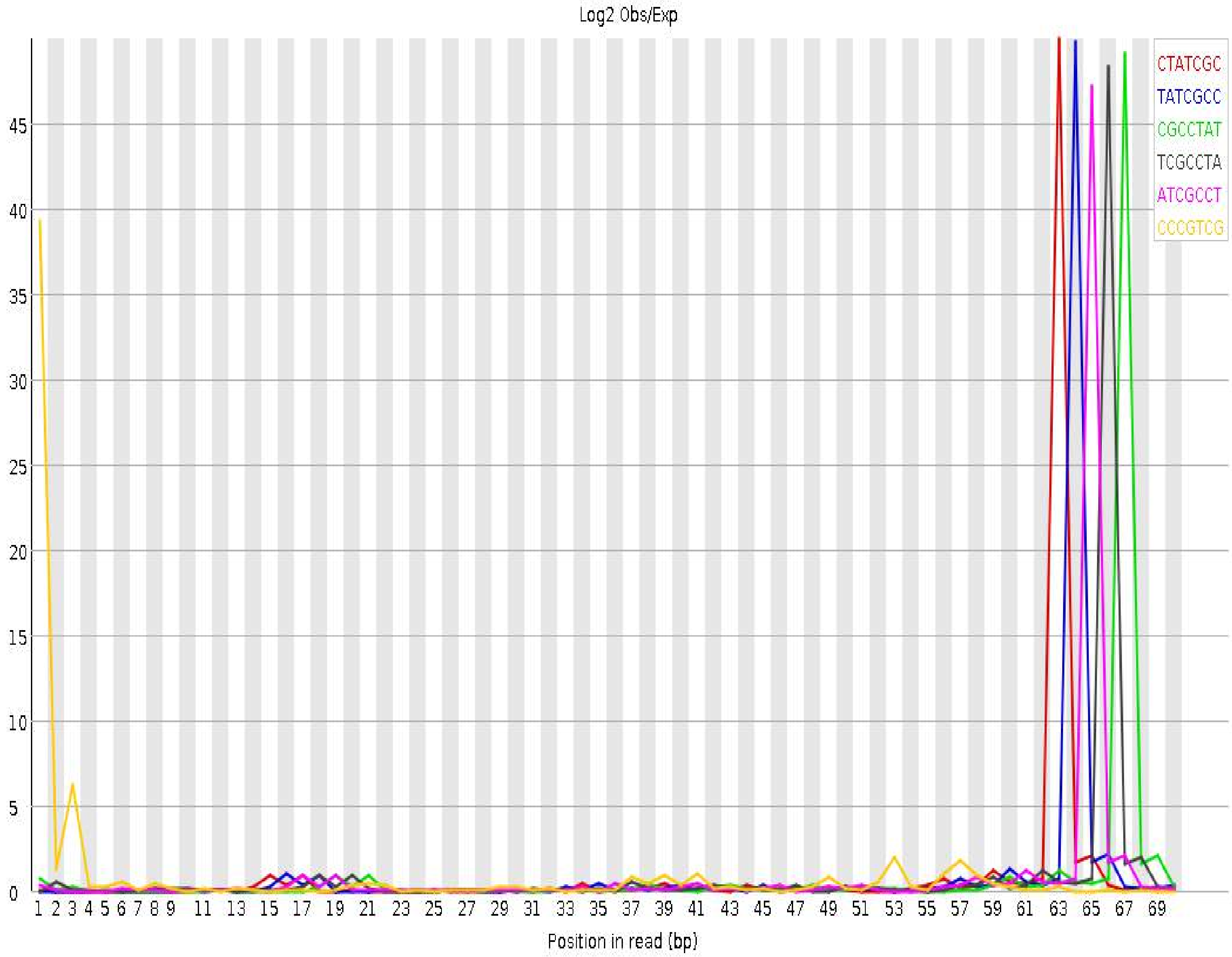


| Sequence | Count | PValue | Obs/Exp Max | Max Obs/Exp Position |
|---|---|---|---|---|
| CTATCGC | 26340 | 0.0 | 49.903564 | 63 |
| TATCGCC | 26435 | 0.0 | 49.79017 | 64 |
| CGCCTAT | 26730 | 0.0 | 49.060287 | 67 |
| TCGCCTA | 27190 | 0.0 | 48.31885 | 66 |
| ATCGCCT | 27900 | 0.0 | 47.20156 | 65 |
| CCCGTCG | 70550 | 0.0 | 39.3427 | 1 |
| GCCTATA | 35085 | 0.0 | 37.666527 | 68 |
| TCTATCG | 35790 | 0.0 | 36.84369 | 62 |
| ACTCGCC | 42125 | 0.0 | 36.639835 | 69 |
| CCGTCGG | 75975 | 0.0 | 36.09146 | 2 |
| TCACTCG | 43330 | 0.0 | 35.652416 | 67 |
| TGCCGTA | 11000 | 0.0 | 34.501587 | 2 |
| TTCGCTC | 44805 | 0.0 | 33.414707 | 47 |
| CGCACTT | 11045 | 0.0 | 33.343735 | 3 |
| CGGAACG | 55985 | 0.0 | 32.430435 | 48 |

| Sequence | Count | PValue | Obs/Exp Max | Max Obs/Exp Position |
|---|---|---|---|---|
| GCTTTCG | 46025 | 0.0 | 32.[illegible] | 44 |
| GCCAGTA | 46065 | 0.0 | 32.3[illegible] | 3 |
| CCAGTAG | 46100 | 0.0 | 32.279613 | 4 |
| TCGCTCT | 46500 | 0.0 | 32.13656 | 48 |
| ACGGCGC | 57260 | 0.0 | 31.756868 | 52 |

**Produced by FastQC (version 0.11.2)**

# FastQC Report

Mon 4 May 2015
Biochain_Adult_Liver_CGATGT_L003_R1.fastq.gz

## Summary


- Basic Statistics
- Per base sequence quality
- Per tile sequence quality
- Per sequence quality scores
- Per base sequence content
- Per sequence GC content
- Per base N content
- Sequence Length Distribution
- Sequence Duplication Levels
- Overrepresented sequences
- Adapter Content
- Kmer Content


## Basic Statistics

| Measure | Value |
|---|---|
| Filename | Biochain_Adult_Liver_CGATGT_L003_R1.fastq.gz |
| File type | Conventional base calls |
| Encoding | Sanger / Illumina 1.9 |
| Total Sequences | 85220810 |
| Sequences flagged as poor quality | 0 |
| Sequence length | 76 |
| %GC | 55 |

## Per base sequence quality

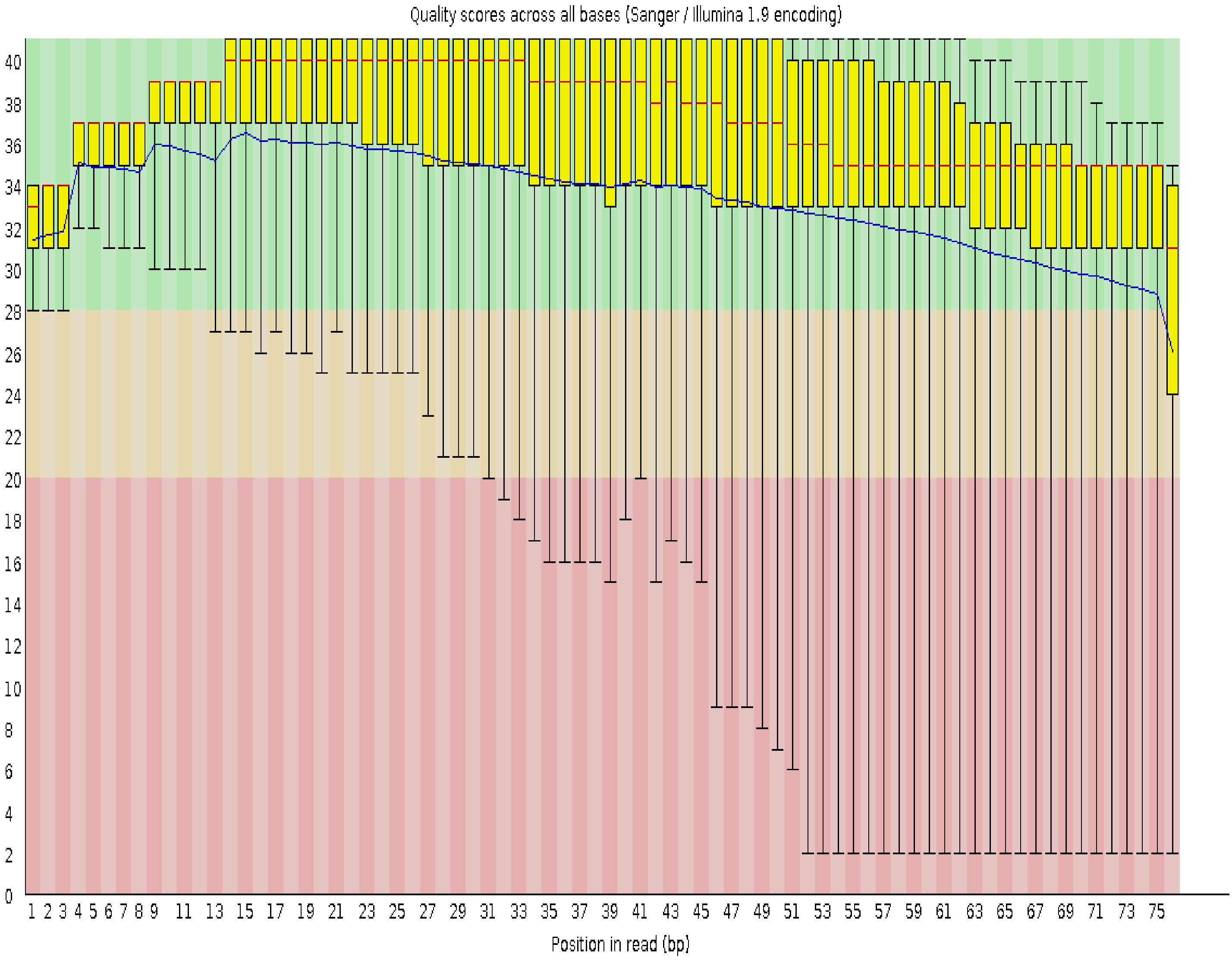


## Per tile sequence quality

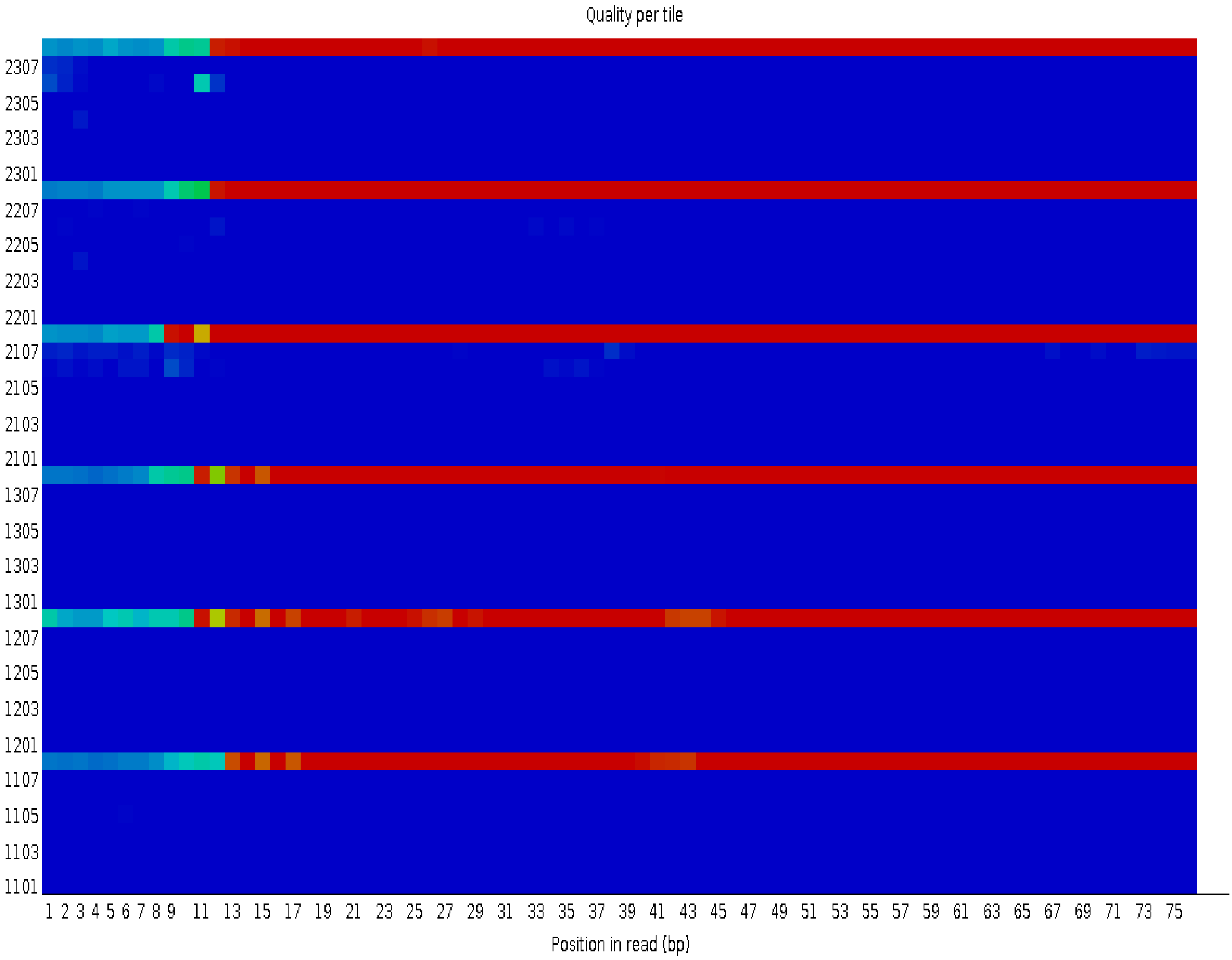


## Per sequence quality scores

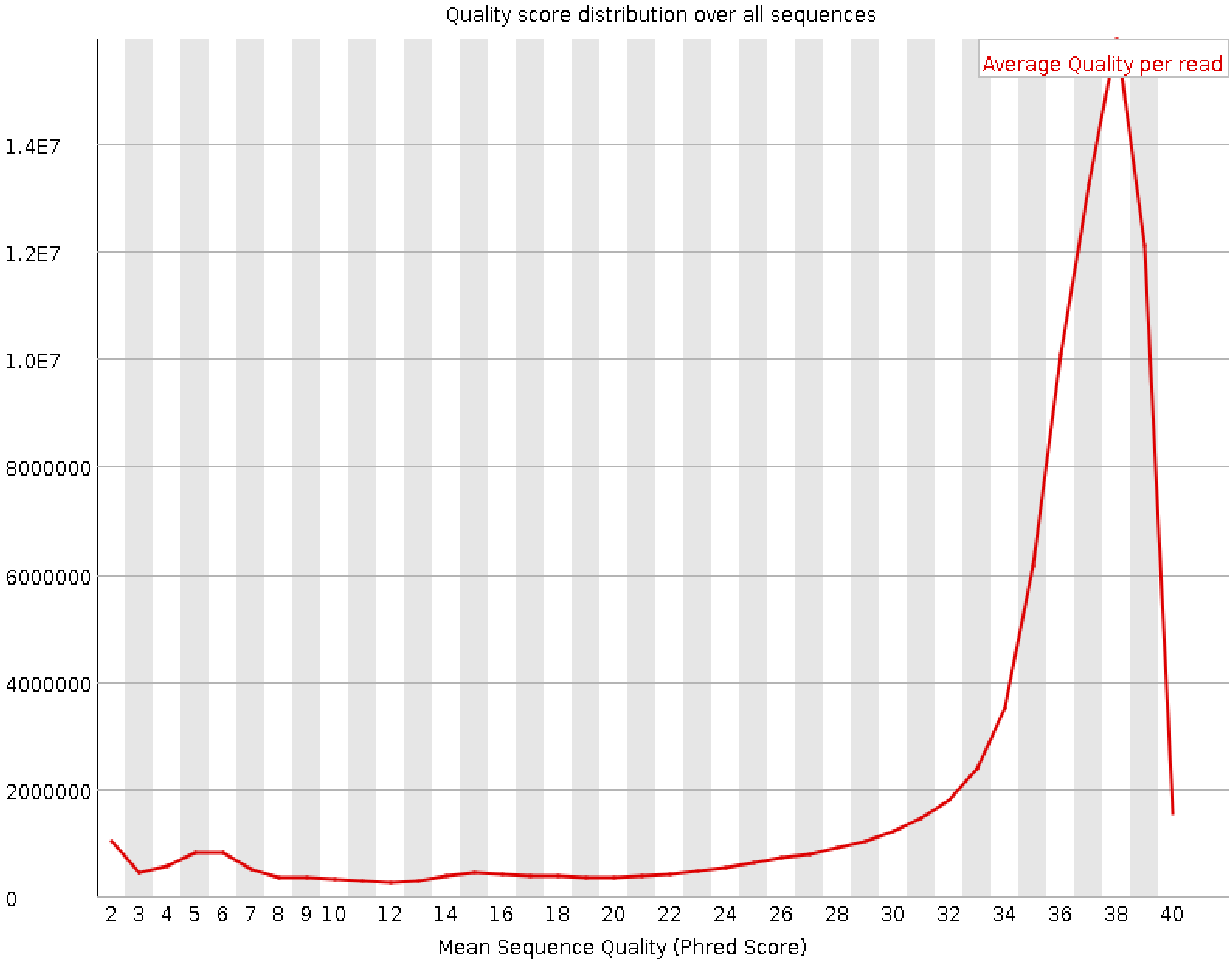


## Per base sequence content

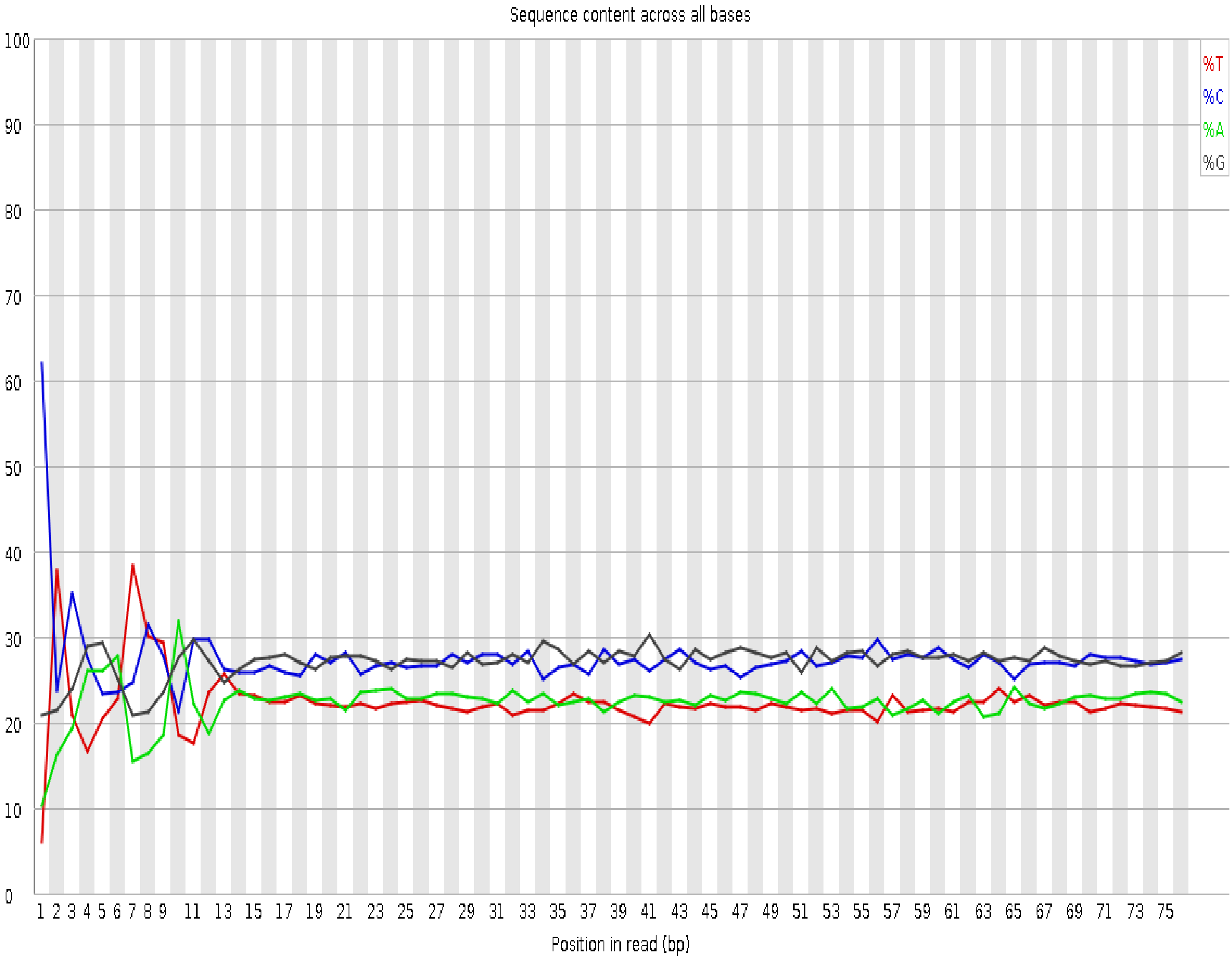


## Per sequence GC content

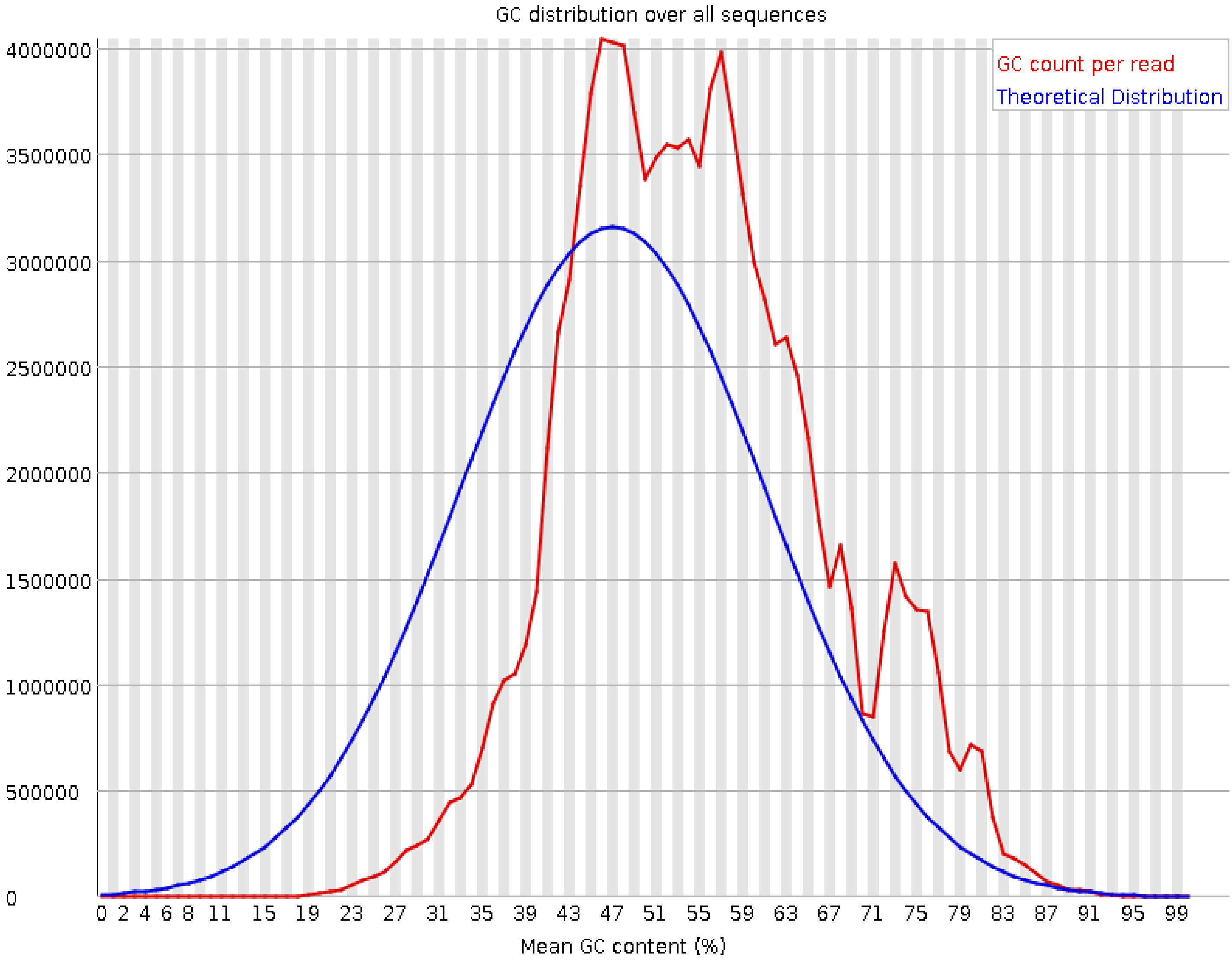


## Per base N content

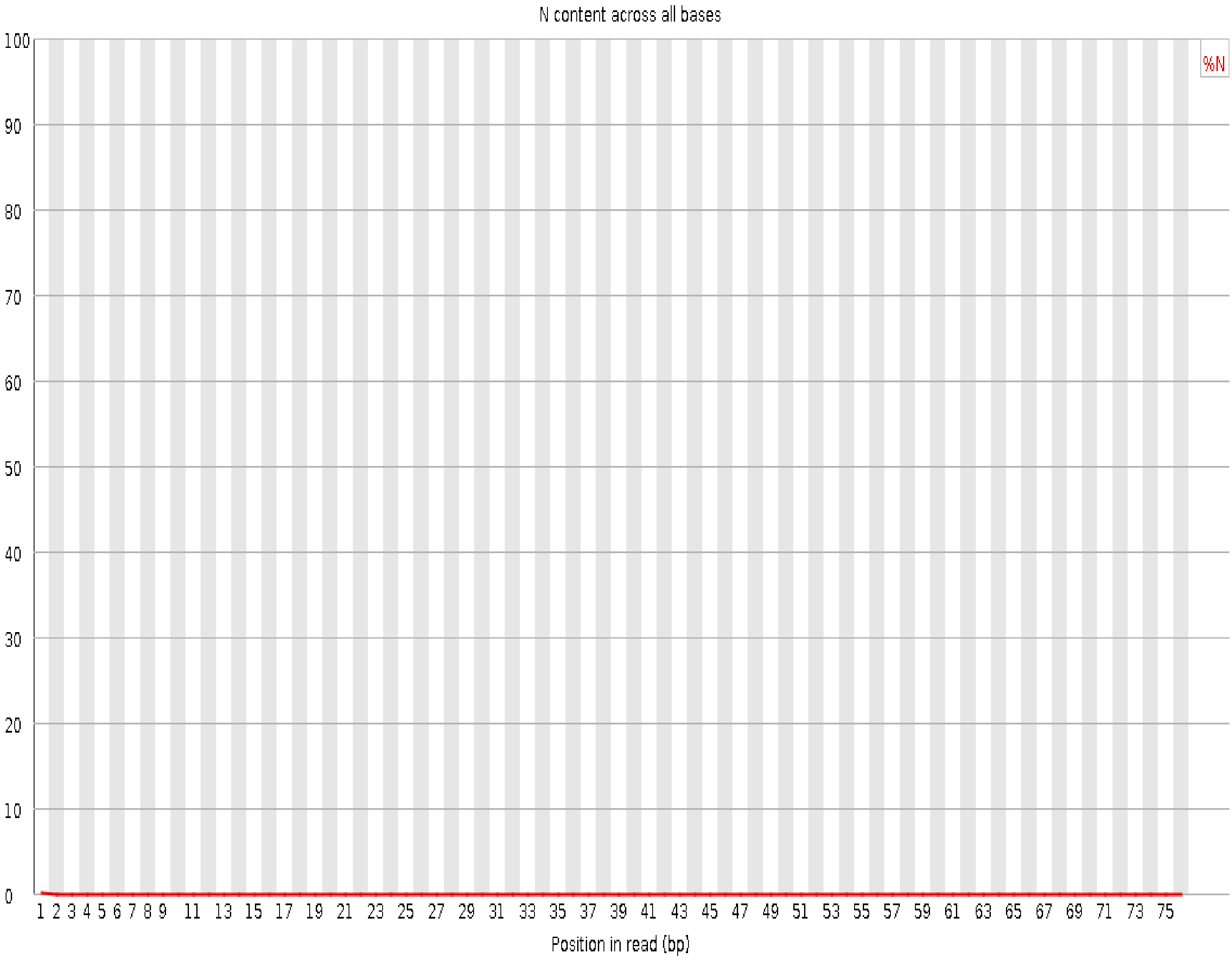


## Sequence Length Distribution

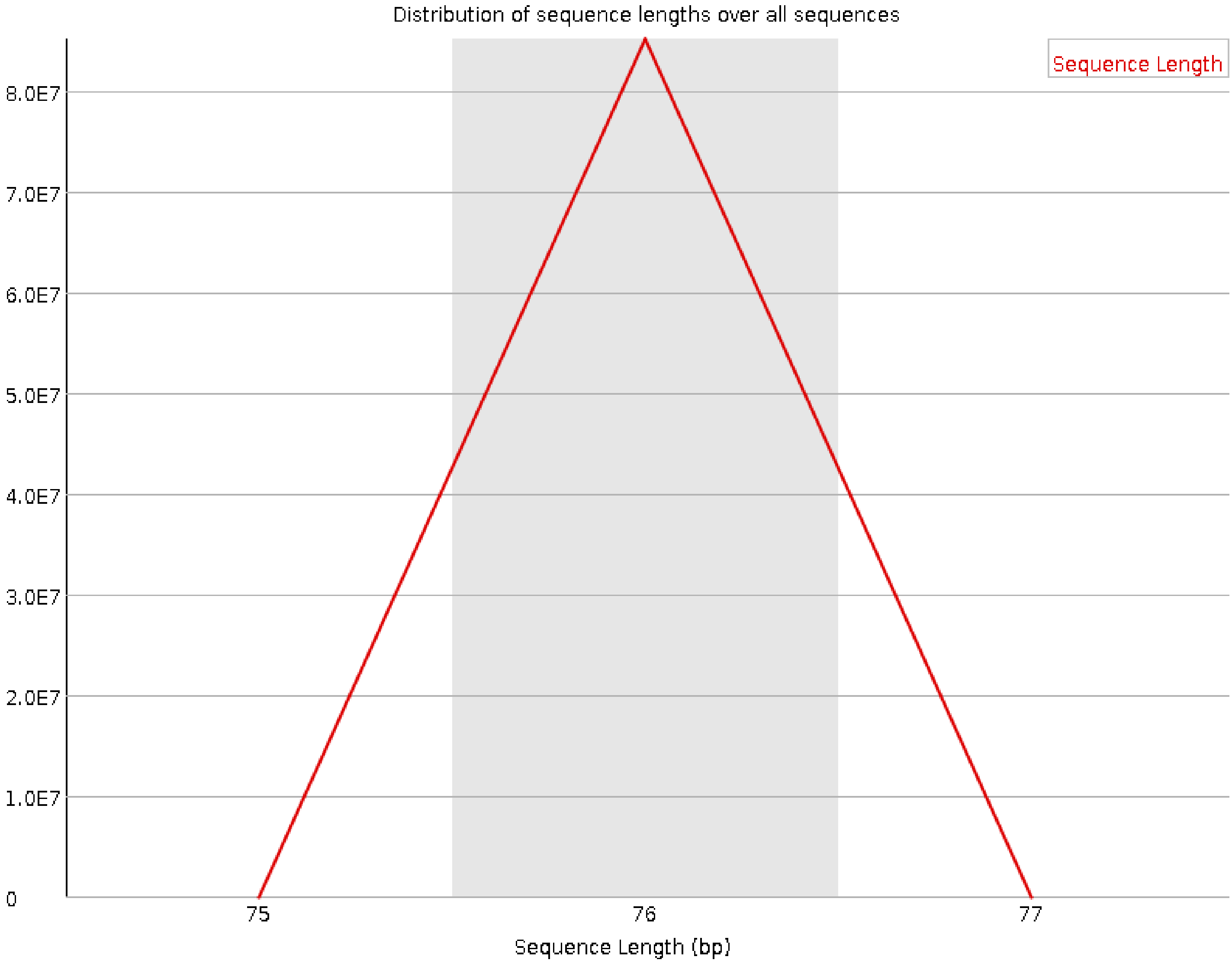


## Sequence Duplication Levels

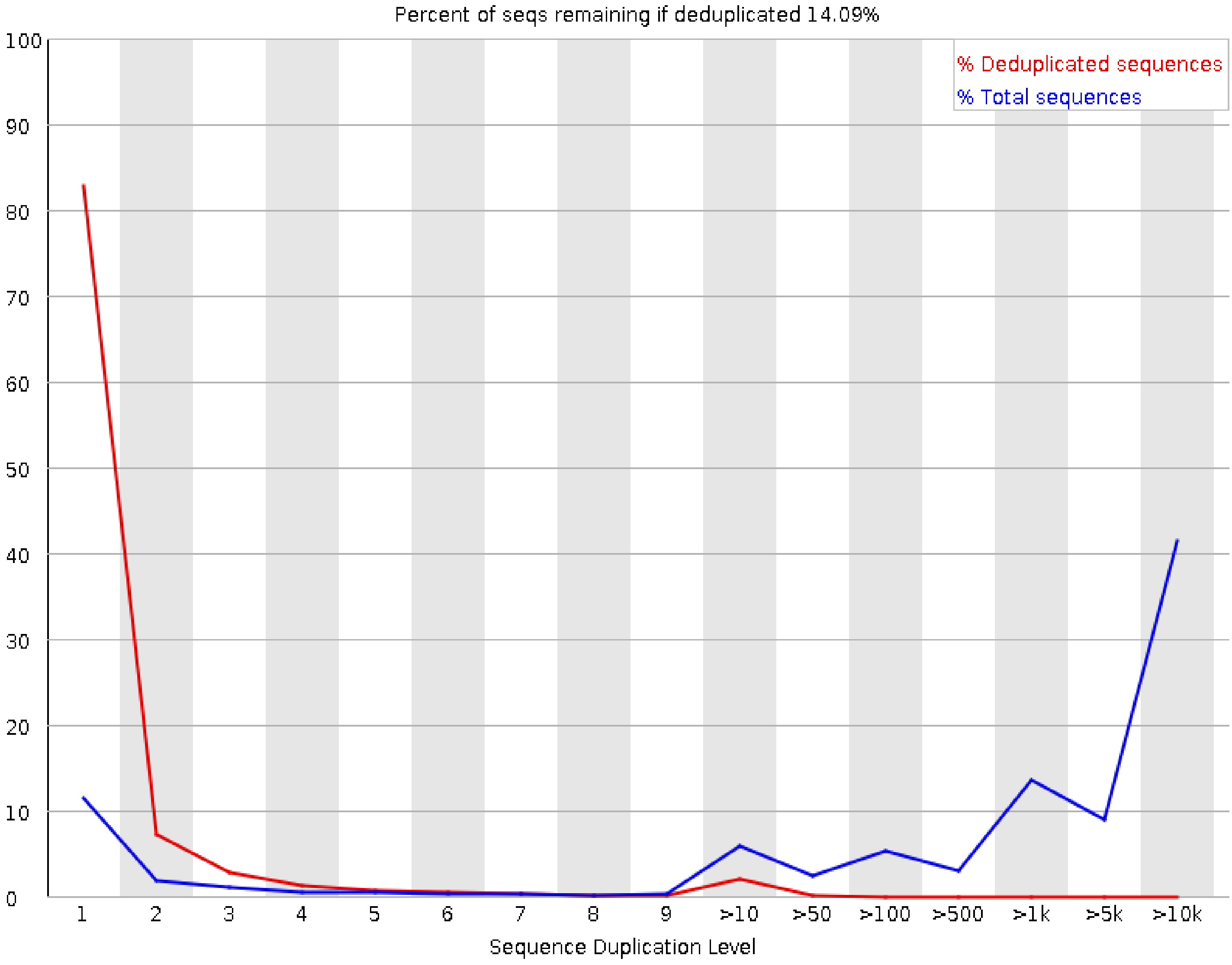


## Overrepresented sequences

| Sequence | Count | Percentage | Possible Source |
|---|---|---|---|
| CTTCCGTACGCCACATGTCCCGCGCCCCGCCGCGGGGCGGGGATTCGGCG | 428908 | 0.5032902174949991 | No Hit |
| CTCTCTTCAAAGTTCTTTTCAACTTTCCCTTACGGTACTTGTTGACTATC | 375495 | 0.4406142114819139 | No Hit |
| CGCAGTTTTATCCGGTAAAGCGAATGATTAGAGGTCTTGGGGCCGAAACG | 327394 | 0.38417142479636135 | No Hit |
| CCCGTCGGCATGTATTAGCTCTAGAATTACCACAGTTATCCAAGTAGGAG | 324181 | 0.380401218904162 | No Hit |
| CCGGTATTTAGCCTTAGATGGAGTTTACCACCCGCTTTGGGCTGCATTCC | 307664 | 0.36101980255761473 | No Hit |
| CTGCCAGTAGCATATGCTTGTCTCAAAGATTAAGCCATGCATGTCTAAGT | 285028 | 0.33445821507681045 | No Hit |
| CAAAGATTAAGCCATGCATGTCTAAGTACGCACGGCCGGTACAGTGAAAC | 228807 | 0.26848723920835765 | No Hit |
| CTCCCTTTCGATCGGCCGAGGGCAACGGAGGCCATCGCCCGTCCCTTCGG | 211476 | 0.24815065709889403 | No Hit |
| CTTAGATGGAGTTTACCACCCGCTTTGGGCTGCATTCCCAAGCAACCCGA | 191439 | 0.22463879421000577 | No Hit |
| CTTGTCTCAAAGATTAAGCCATGCATGTCTAAGTACGCACGGCCGGTACA | 190792 | 0.22387958997338794 | No Hit |
| CTCGCATTCCACGCCCGGCTCCACGCCAGCGAGCCGGGCTTCTTACCCAT | 182780 | 0.2144781304003095 | No Hit |

| Sequence | Count | Percentage | Possible Source |
|---|---|---|---|
| CGGGTCTTCCGTACGCCACATGTCCCGCGCCCCGCCGCGGGGCGGGATT | 150839 | 0.17699784829550436 | No Hit |
| CTTCACCGTGCCAGACTAGAGTCAAGCTCAACAGGGTCTTCTTTCCCCGC | 147212 | 0.1727418455656547 | N[illegible] |
| CTTGAACTCTCTCTTCAAAGTTCTTTTCAACTTTCCCTTACGGTACTTGT | 146236 | 0.1715965853880056 | No Hit |
| CCCGCTTTGGGCTGCATTCCCAAGCAACCCGACTCCGGGAAGACCCGGGC | 140152 | 0.16445748403470936 | No Hit |
| CTGGATAGTAGGTAGGGACAGTGGGAATCTCGTTCATCCATTCATGCGCG | 140092 | 0.16438707869591945 | No Hit |
| CCCGAAGTTACGGATCCGGCTTGCCGACTTCCCTTACCTACATTGTTCCA | 134502 | 0.15782764796532678 | No Hit |
| CTGAATTTAAGCATATTAGTCAGCGGAGGAGAAGAAACTAACCAGGATTC | 134117 | 0.15737588037475825 | No Hit |
| CTGCTGTCTATATCAACCAACACCTTTTCTGGGGTCTGATGAGCGTCGGC | 131787 | 0.15464180638508365 | No Hit |
| GTCGGCATGTATTAGCTCTAGAATTACCACAGTTATCCAAGTAGGAGAGG | 127527 | 0.1496430273310005 | No Hit |
| CTCCGTTTCCGACCTGGGCCGGTTCACCCCTCCTTAGGCAACCTGGTGGT | 123888 | 0.14537294353339283 | No Hit |
| CACAGTTATCCAAGTAGGAGAGGAGCGAGCGACCAAAGGAACCATAACTG | 123518 | 0.14493877727752177 | No Hit |
| CTCGATCAGAAGGACTTGGGCCCCCCACGAGCGGCGCCGGGGAGCGGGTC | 121421 | 0.14247811068681465 | No Hit |
| CTCTCATGTCTCTTCACCGTGCCAGACTAGAGTCAAGCTCAACAGGGTCT | 119988 | 0.14079659651204912 | No Hit |
| ATCAGACGTGGCGACCCGCTGAATTTAAGCATATTAGTCAGCGGAGGAGA | 115985 | 0.1360993869924494 | No Hit |
| CTTTAAATGGGTAAGAAGCCCGGCTCGCTGGCGTGGAGCCGGGCGTGGAA | 111587 | 0.13093867565914946 | No Hit |
| GTCAAAGTGAAGAAATTCAATGAAGCGCGGGTAAACGGCGGGAGTAACTA | 111562 | 0.13090934010132033 | No Hit |
| CTGCTTACCAAAAGTGGCCCACTAGGCACTCGCATTCCACGCCCGGCTCC | 109879 | 0.12893447034826352 | No Hit |
| CCGTCGGCATGTATTAGCTCTAGAATTACCACAGTTATCCAAGTAGGAGA | 107632 | 0.12629779041058165 | No Hit |
| CTGAATTTAAGCATATTAGTCAGCGGAGGAAAAGAAACTAACCAGGATTC | 107368 | 0.12598800691990605 | No Hit |
| CACCCGTTTACCTCTTAACGGTTTCACGCCCTCTTGAACTCTCTCTTCAA | 107055 | 0.1256207257358854 | No Hit |
| CCTCACCCGGCCCGGACACGGACAGGATTGACAGATTGATAGCTCTTTCT | 106405 | 0.12485800123232811 | No Hit |
| CCCATATCCGCAGCAGGTCTCCAAGGTGAACAGCCTCTGGCATGTTGGAA | 105325 | 0.12359070513410986 | No Hit |
| CGCGTAACTAGTTAGCATGCCAGAGTCTCGTTCGTTATCGGAATTAACCA | 103413 | 0.12134712167133825 | No Hit |
| AAACGATCTCAACCTATTCTCAAACTTTAAATGGGTAAGAAGCCCGGCTC | 102933 | 0.12078387896101903 | No Hit |
| CCCTCCTTAGGCAACCTGGTGGTCCCCCGCTCCCGGGAGGTCACCATATT | 100785 | 0.11826336783234048 | No Hit |
| GCCCTCTTGAACTCTCTCTTCAAAGTTCTTTTCAACTTTCCCTTACGGTA | 100696 | 0.1181589332464688 | No Hit |
| CGAGAACTTTGAAGGCCGAAGTGGAGAAGGGTTCCATGTGAACAGCAGTT | 100427 | 0.11784328264422739 | No Hit |
| CTCGTGCCGGTATTTAGCCTTAGATGGAGTTTACCACCCGCTTTGGGCTG | 97707 | 0.11465157395241844 | No Hit |
| CTCCCGTCCACTCTCGACTGCCGGCGACGGCCGGGTATGGGCCCGACGCT | 97229 | 0.11409067808672553 | No Hit |
| CCACTCTCGACTGCCGGCGACGGCCGGGTATGGGCCCGACGCTCCAGCGC | 97089 | 0.11392639896288241 | No Hit |
| CTCCATCTAAGGCTAAATACCGGCACGAGACCGATAGTCAACAAGTACCG | 96687 | 0.11345468319299008 | No Hit |
| AAGAAACTAACCAGGATTCCCTCAGTAACGGCGAGTGAACAGGGAAGAGC | 96418 | 0.11313903259074867 | No Hit |
| CGCGATGTGATTTCTGCCCAGTGCTCTGAATGTCAAAGTGAAGAAATTCA | 94789 | 0.1112275276426028 | No Hit |
| CTTTTCTTTGTGAAGGGCAGGGCGCCCTGGAATGGGTTCGCCCCGAGAGA | 94209 | 0.11054694270096704 | No Hit |
| CGAAGGCCCGCGGCGGGTGTTGACGCGATGTGATTTCTGCCCAGTGCTCT | 93908 | 0.11019374258470437 | No Hit |
| CTTTTGGTAAGCAGAACTGGCGCTGCGGGATGAACCGAACGCCGGGTTAA | 93282 | 0.10945918021666304 | No Hit |
| CTCAAACTTTAAATGGGTAAGAAGCCCGGCTCGCTGGCGTGGAGCCGGGC | 92474 | 0.10851105498762567 | No Hit |
| ATCAGACGTGGCGACCCGCTGAATTTAAGCATATTAGTCAGCGGAGGAAA | 92359 | 0.1083761114216117 | No Hit |
| CAAACTTTAAATGGGTAAGAAGCCCGGCTCGCTGGCGTGGAGCCGGGCGT | 89947 | 0.10554581680225757 | No Hit |

| Sequence | Count | Percentage | Possible Source |
|---|---|---|---|
| CTCAAAGATTAAGCCATGCATGTCTAAGTACGCACGGCCGGTACAGTGAA | 89446 | 0.10495793222336189 | No Hit |
| CAGAAACCTCCCGTGGAGCA[illegible]AGCTCGCTTGATCTTGATTT | [illegible] | 0.104[illegible]4736 | [illegible] |
| CGTCGGCATGTATTAGCTCTAGAATTACCACAGTTATCCAAGTAGGAGAG | 88625 | 0.10399455250425335 | No Hit |
| CTCCGCCACTCCGGATTCGGGGATCTGAACCCGACTCCCTTTCGATCGGC | 87733 | 0.10294785980091013 | No Hit |
| CCTGCCAGTAGCATATGCTTGTCTCAAAGATTAAGCCATGCATGTCTAAG | 86637 | 0.10166178894568123 | No Hit |
| CGAACGCCGGGTTAAGGCGCCCGATGCCGACGCTCATCAGACCCCAGAAA | 86204 | 0.10115369708408077 | No Hit |
| CTGGGGTCTGATGAGCGTCGGCATCGGGCGCCTTAACCCGGCGTTCGGTT | 85741 | 0.10061040255308533 | No Hit |

## Adapter Content

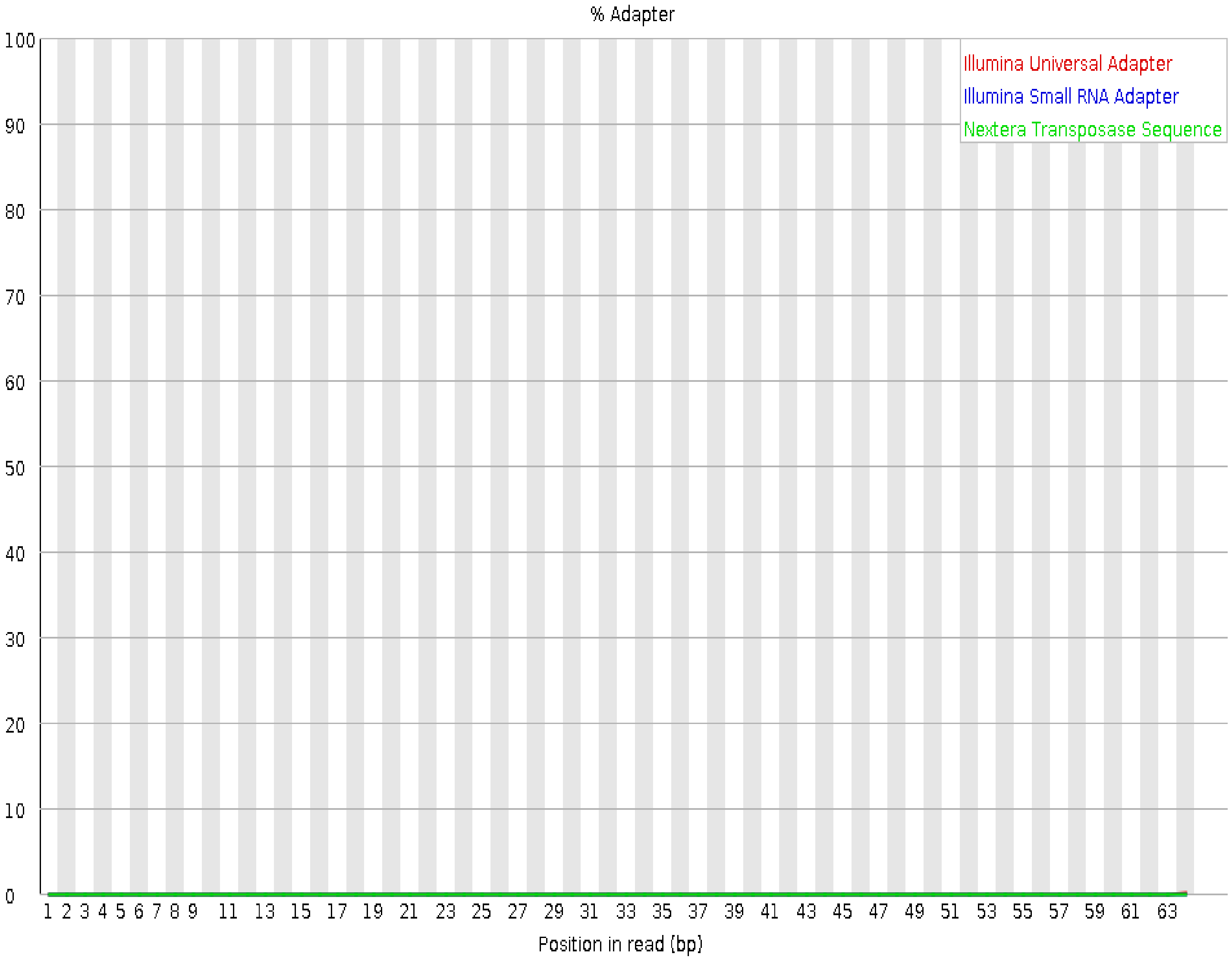


## Kmer Content

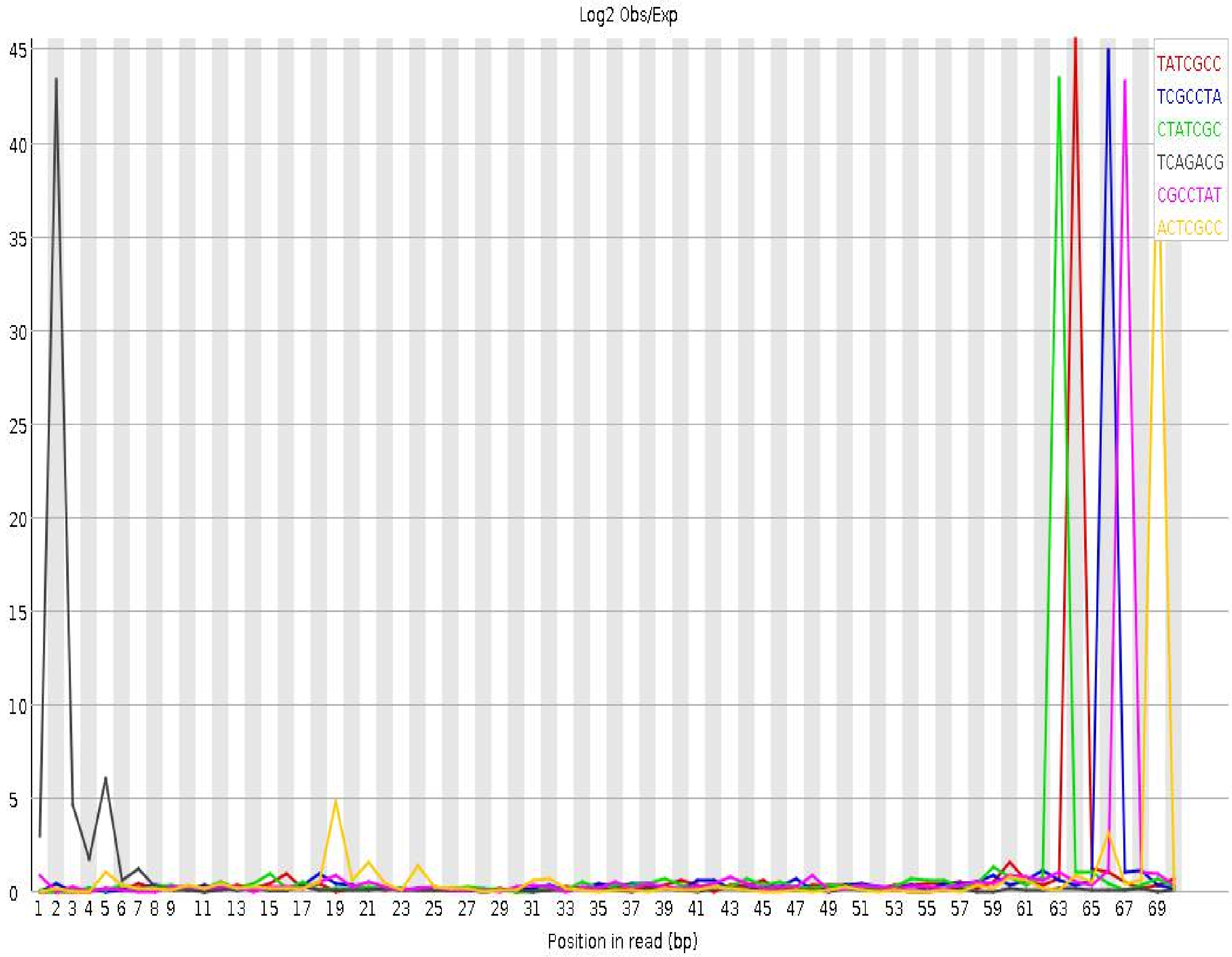


| Sequence | Count | PValue | Obs/Exp Max | Max Obs/Exp Position |
|---|---|---|---|---|
| TATCGCC | 10665 | 0.0 | 45.51566 | 64 |
| TCGCCTA | 10745 | 0.0 | 44.94704 | 66 |
| CTATCGC | 11210 | 0.0 | 43.49723 | 63 |
| TCAGACG | 41025 | 0.0 | 43.348736 | 2 |
| CGCCTAT | 11150 | 0.0 | 43.251663 | 67 |
| ACTCGCC | 82575 | 0.0 | 41.492023 | 69 |
| CTCGCTA | 14475 | 0.0 | 41.273197 | 1 |
| TCACTCG | 83155 | 0.0 | 41.23609 | 67 |
| ATCGCCT | 12215 | 0.0 | 39.738216 | 65 |
| CTGCCAG | 61640 | 0.0 | 38.473003 | 1 |
| TGTCGAG | 10610 | 0.0 | 37.276848 | 5 |
| TGCCAGT | 63265 | 0.0 | 37.172092 | 2 |
| TTCACTC | 92190 | 0.0 | 37.058125 | 66 |
| CGCGTAA | 24080 | 0.0 | 36.71268 | 1 |
| CCAGTAG | 64160 | 0.0 | 36.08624 | 4 |

| Sequence | Count | PValue | Obs/Exp Max | Max Obs/Exp Position |
|---|---|---|---|---|
| GTGTCGA | 11375 | 0.0 | 35.[illegible] | 4 |
| GCCAGTA | 65415 | 0.0 | 35.4[illegible] | 3 |
| TGTGTCG | 11515 | 0.0 | 35.198223 | 3 |
| CTCGCCG | 112290 | 0.0 | 34.0555 | 70 |
| GCCTATA | 14455 | 0.0 | 33.798687 | 68 |

**Produced by FastQC (version 0.11.2)**

# FastQC Report

Mon 4 May 2015
Biochain_Adult_Liver_CGATGT_L003_R2.fastq.gz

## Summary


- Basic Statistics
- Per base sequence quality
- Per tile sequence quality
- Per sequence quality scores
- Per base sequence content
- Per sequence GC content
- Per base N content
- Sequence Length Distribution
- Sequence Duplication Levels
- Overrepresented sequences
- Adapter Content
- Kmer Content


## Basic Statistics

| Measure | Value |
|---|---|
| Filename | Biochain_Adult_Liver_CGATGT_L003_R2.fastq.gz |
| File type | Conventional base calls |
| Encoding | Sanger / Illumina 1.9 |
| Total Sequences | 85220810 |
| Sequences flagged as poor quality | 0 |
| Sequence length | 76 |
| %GC | 55 |

## Per base sequence quality

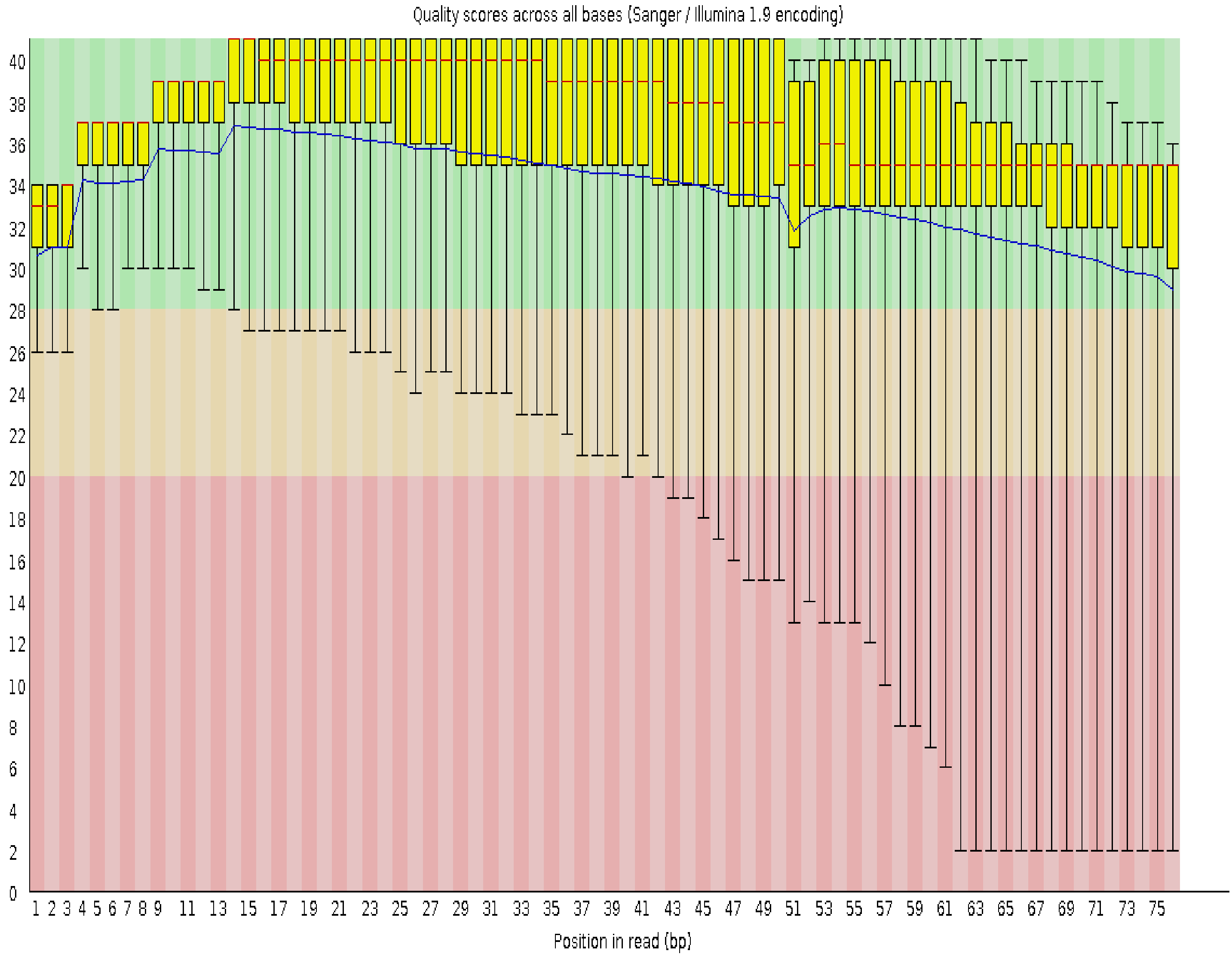


## Per tile sequence quality

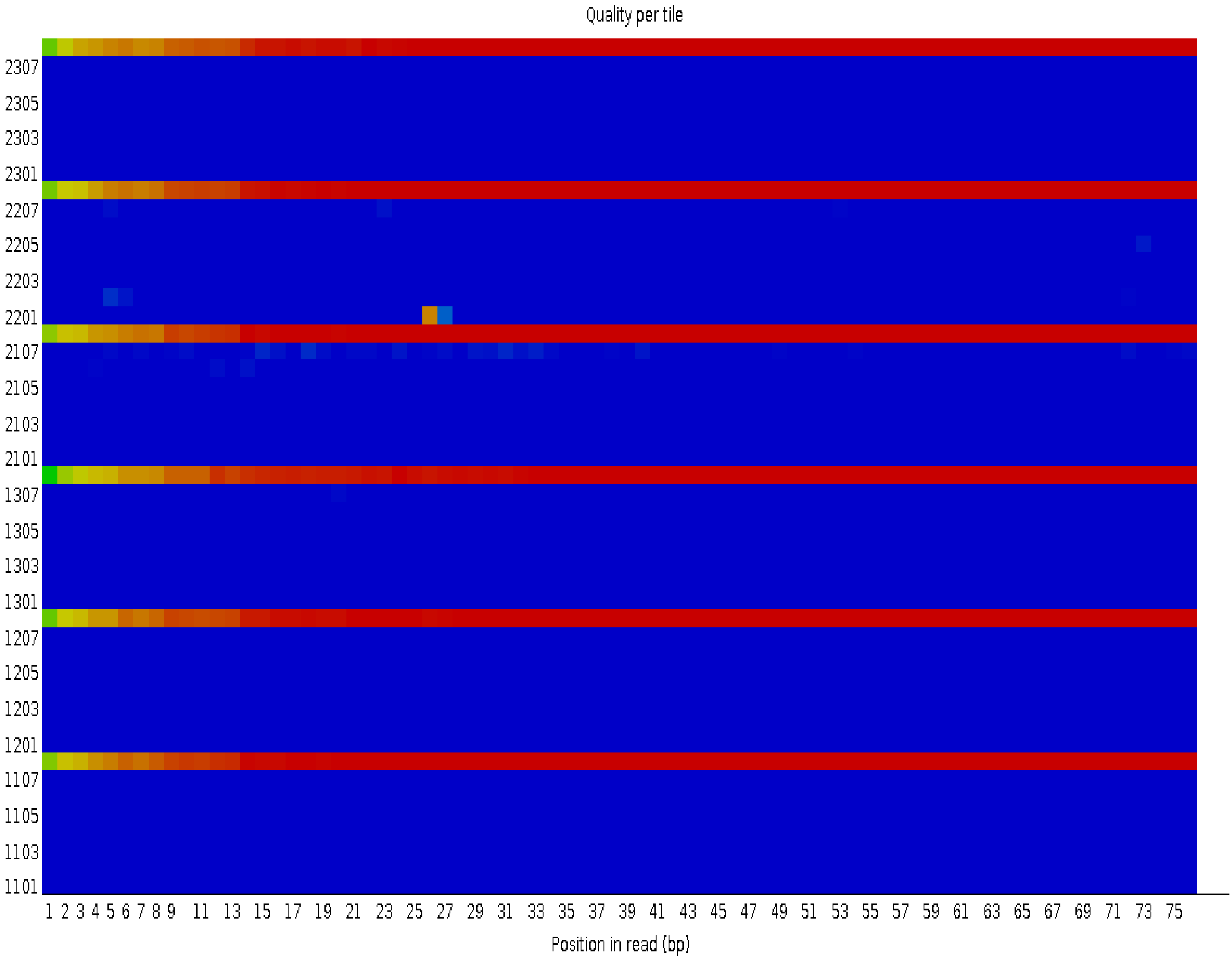


## Per sequence quality scores

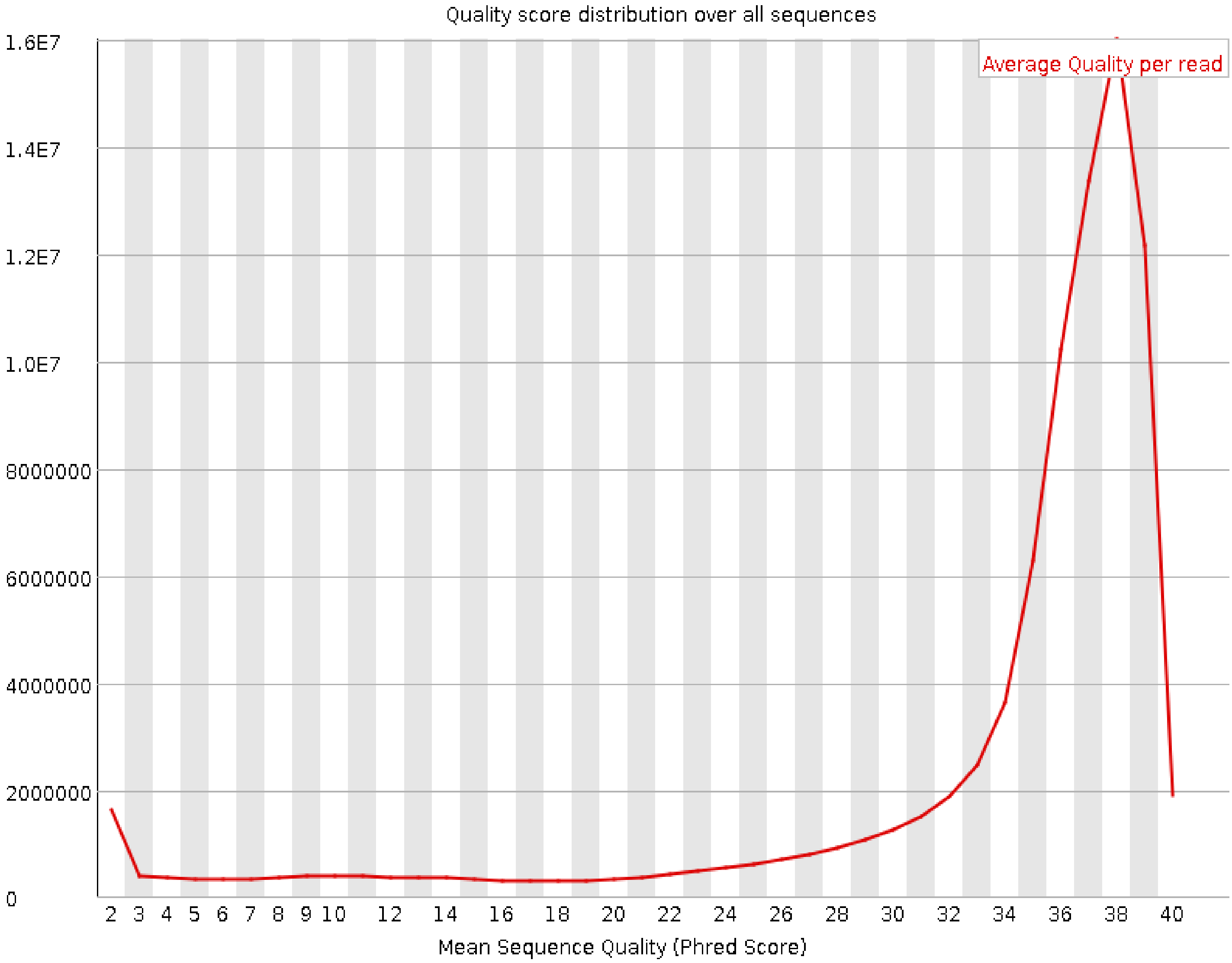


## Per base sequence content

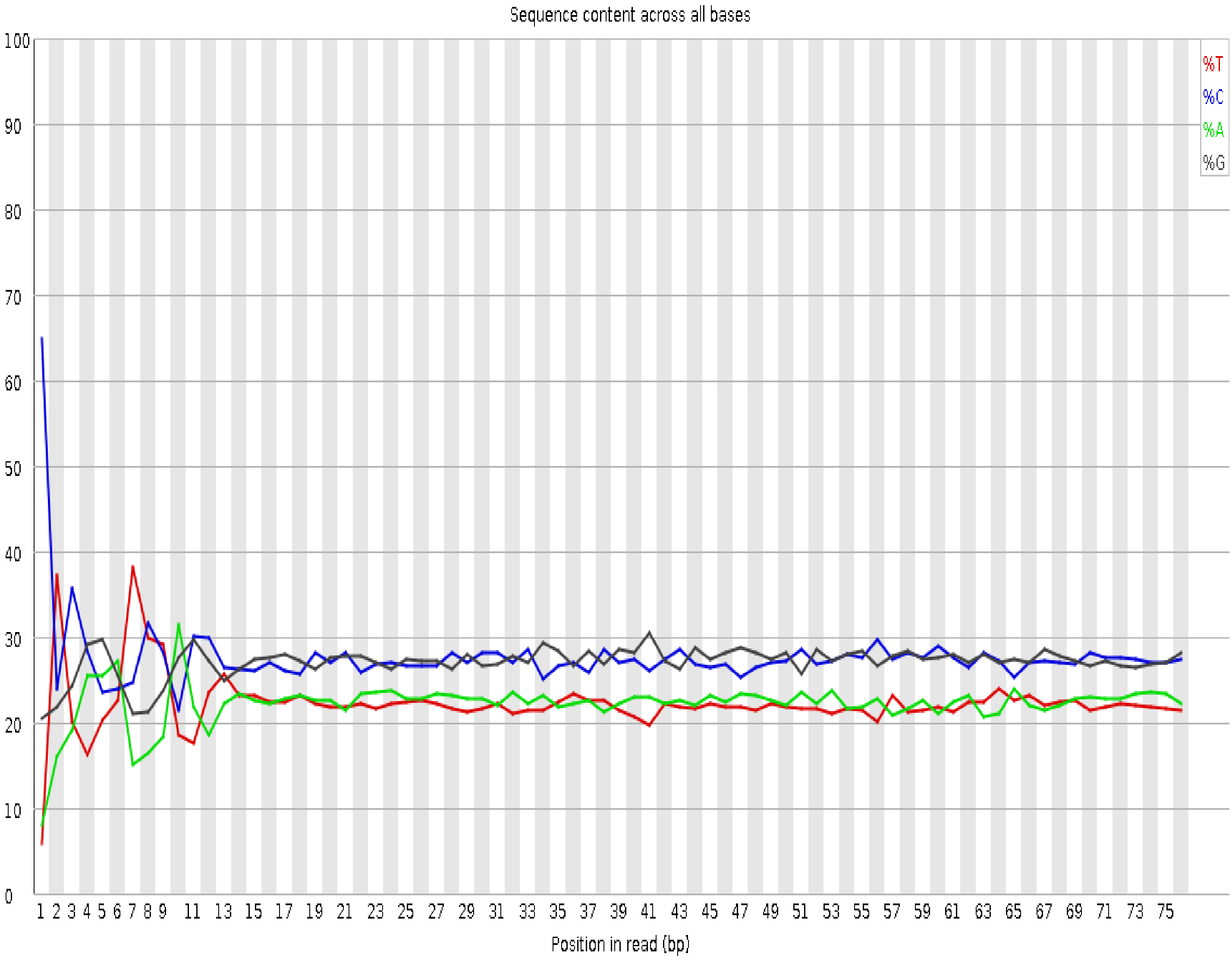


## Per sequence GC content

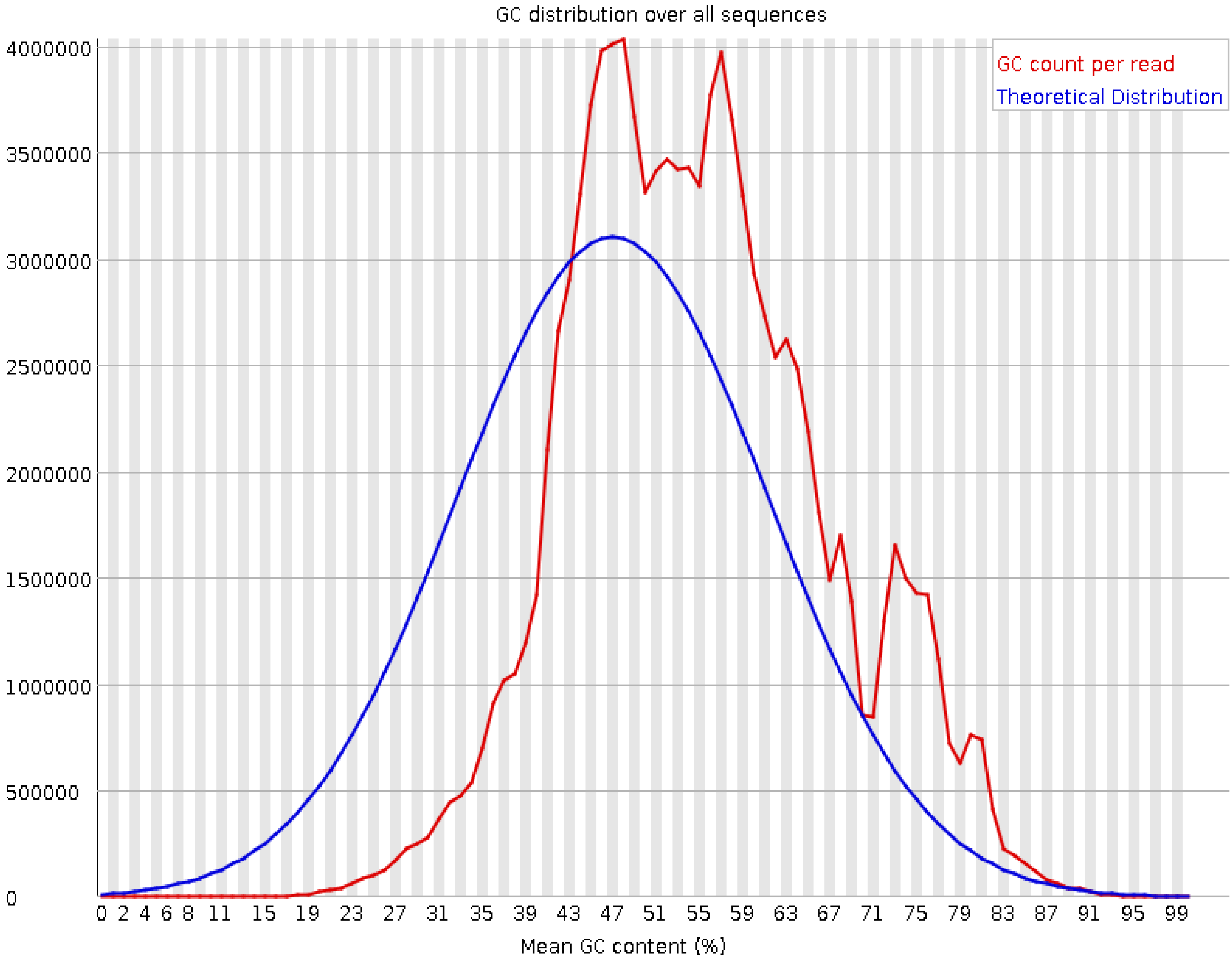


## Per base N content

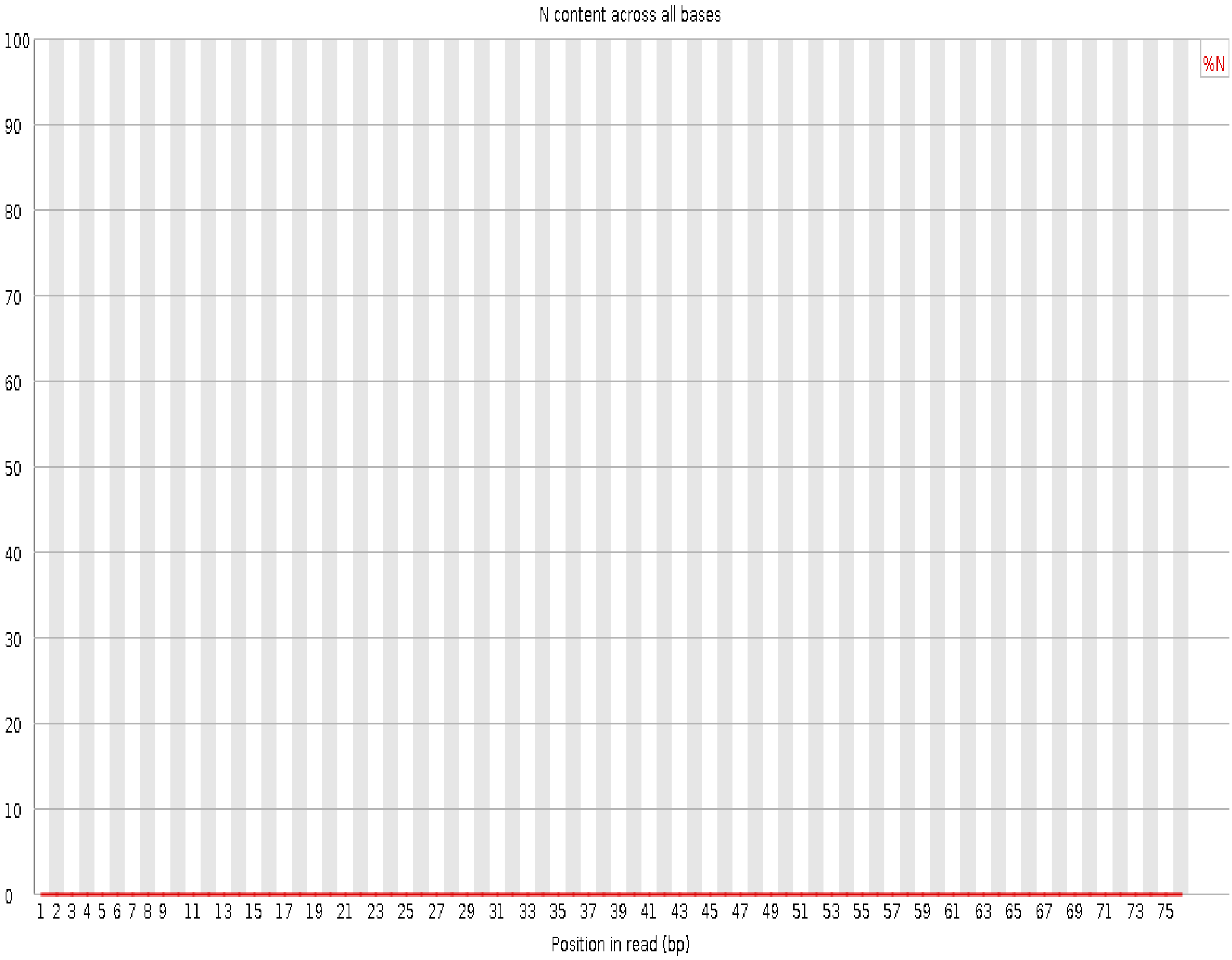


## Sequence Length Distribution

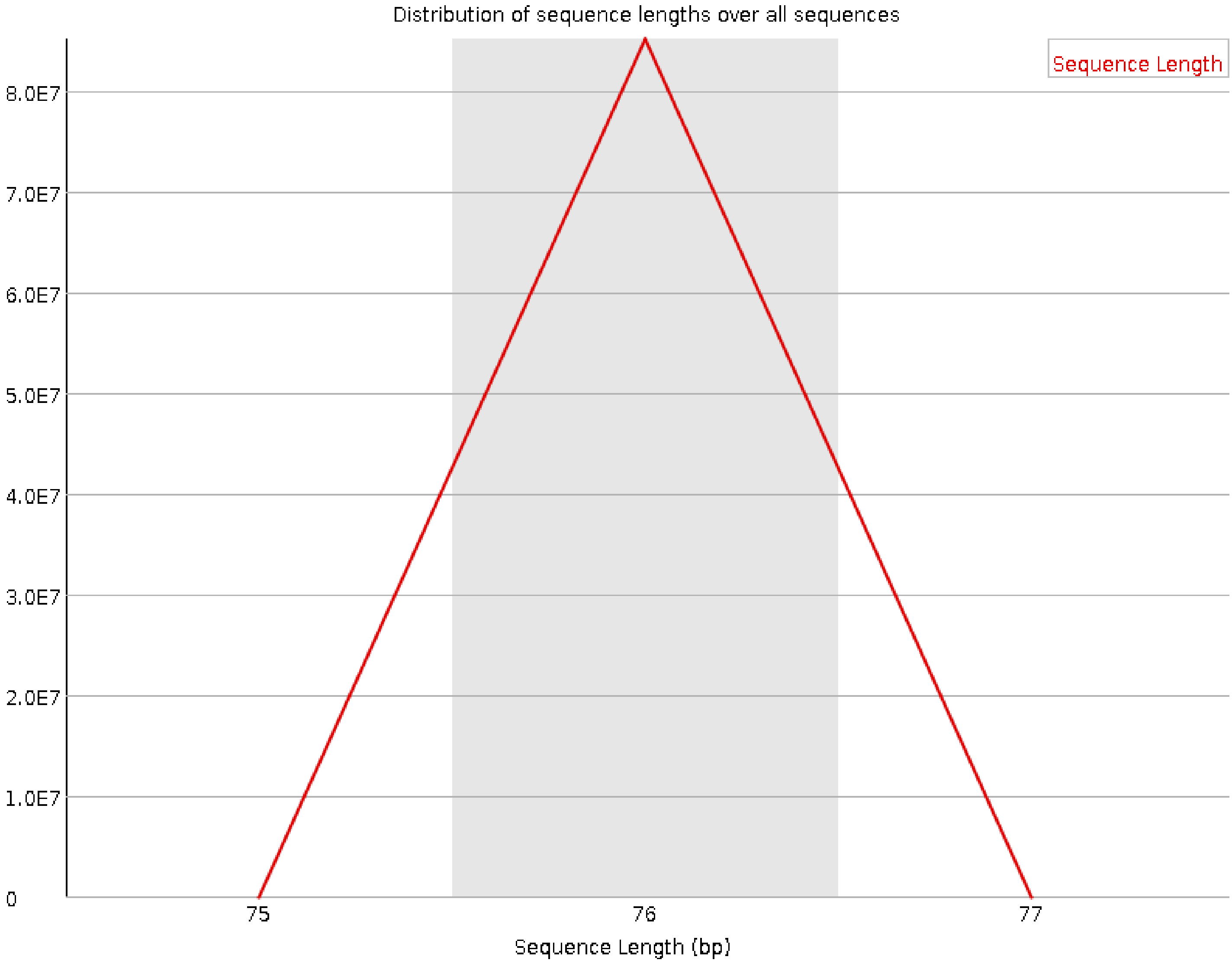


## Sequence Duplication Levels

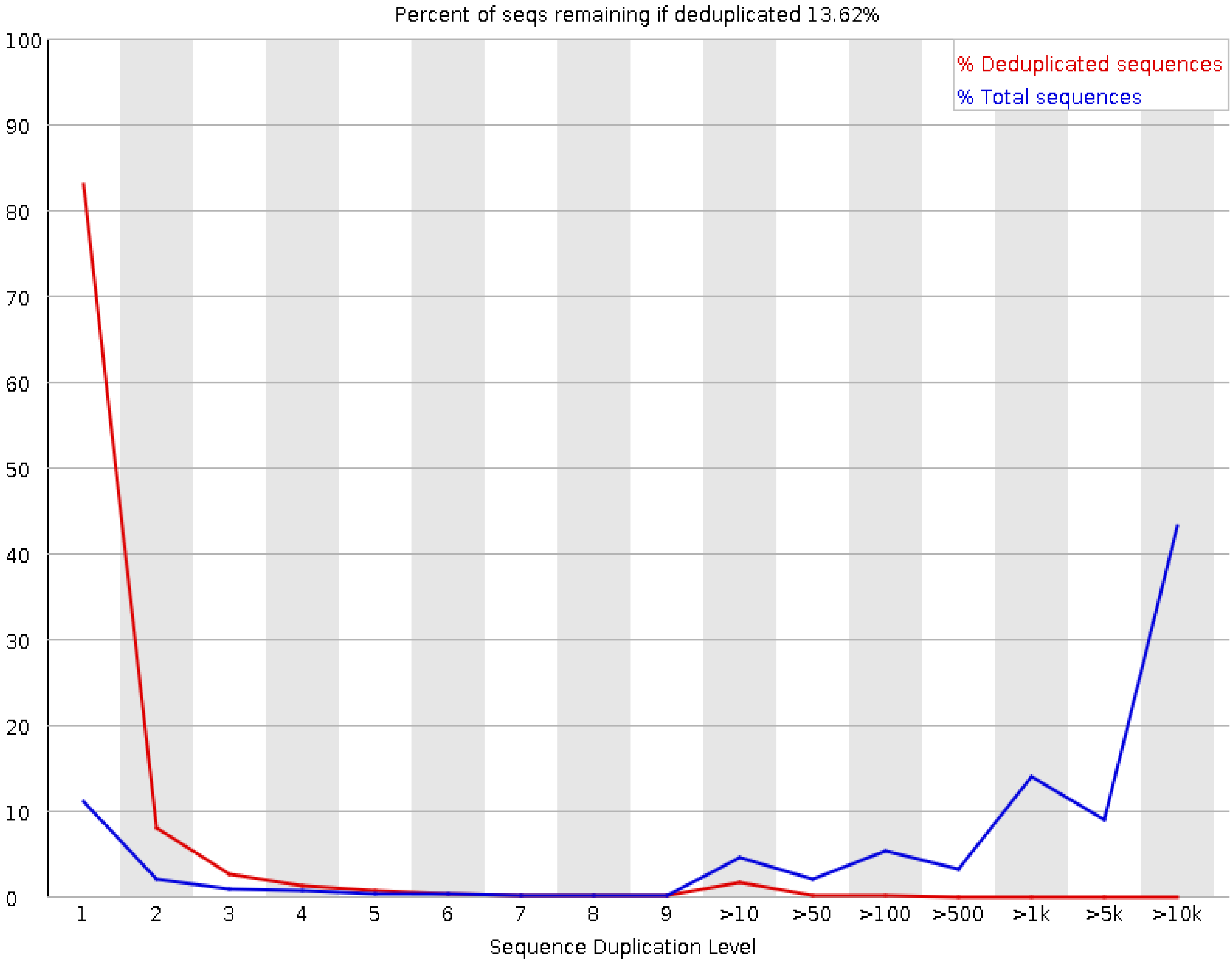


## Overrepresented sequences

| Sequence | Count | Percentage | Possible Source |
|---|---|---|---|
| CTTCCGTACGCCACATGTCCCGCGCCCCGCCGCGGGGCGGGGATTCGGCG | 458944 | 0.5385351300932248 | No Hit |
| CTCTCTTCAAAGTTCTTTTCAACTTTCCCTTACGGTACTTGTTGACTATC | 373863 | 0.43869918626682847 | No Hit |
| CCCGTCGGCATGTATTAGCTCTAGAATTACCACAGTTATCCAAGTAGGAG | 358921 | 0.4211659100635162 | No Hit |
| CGCAGTTTTATCCGGTAAAGCGAATGATTAGAGGTCTTGGGGCCGAAACG | 355043 | 0.41661537833306206 | No Hit |
| CCGGTATTTAGCCTTAGATGGAGTTTACCACCCGCTTTGGGCTGCATTCC | 321833 | 0.37764602331285047 | No Hit |
| CTGCCAGTAGCATATGCTTGTCTCAAAGATTAAGCCATGCATGTCTAAGT | 310721 | 0.36460695456896036 | No Hit |
| CAAAGATTAAGCCATGCATGTCTAAGTACGCACGGCCGGTACAGTGAAAC | 246349 | 0.28907141342589915 | No Hit |
| CTCCCTTTCGATCGGCCGAGGGCAACGGAGGCCATCGCCCGTCCCTTCGG | 241094 | 0.28290507917021673 | No Hit |
| CTCGCATTCCACGCCCGGCTCCACGCCAGCGAGCCGGGCTTCTTACCCAT | 197320 | 0.23153969083372947 | No Hit |
| CTTGTCTCAAAGATTAAGCCATGCATGTCTAAGTACGCACGGCCGGTACA | 195807 | 0.22976430287391073 | No Hit |
| CGGGTCTTCCGTACGCCACATGTCCCGCGCCCCGCCGCGGGGCGGGGATT | 187143 | 0.21959777195264865 | No Hit |

| Sequence | Count | Percentage | Possible Source |
|---|---|---|---|
| CTTAGATGGAGTTTACCACCCGCTTTGGGCTGCATTCCCAAGCAACCCGA | 183989 | 0.21589679797692604 | No Hit |
| CTTCACCGTGCCAGACTAGAGTCAAGCTCAACAGGGTCTTCTTTCCCCGC | 164980 | 0.19359121322597145 | [illegible] |
| CTGGATAGTAGGTAGGGACAGTGGGAATCTCGTTCATCCATTCATGCGCG | 150342 | 0.17641465740586132 | No Hit |
| CCCGCTTTGGGCTGCATTCCCAAGCAACCCGACTCCGGGAAGACCCGGGC | 148275 | 0.17398919348454914 | No Hit |
| CTTGAACTCTCTCTTCAAAGTTCTTTTCAACTTTCCCTTACGGTACTTGT | 146542 | 0.1719556526158341 | No Hit |
| CTCCGTTTCCGACCTGGGCCGGTTCACCCCTCCTTAGGCAACCTGGTGGT | 140167 | 0.16447508536940683 | No Hit |
| CTGCTGTCTATATCAACCAACACCTTTTCTGGGGTCTGATGAGCGTCGGC | 137722 | 0.16160606781371825 | No Hit |
| GTCGGCATGTATTAGCTCTAGAATTACCACAGTTATCCAAGTAGGAGAGG | 132774 | 0.15579997420817757 | No Hit |
| CTCGATCAGAAGGACTTGGGCCCCCCACGAGCGGCGCCGGGGAGCGGGTC | 130948 | 0.15365730506433817 | No Hit |
| CTCTCATGTCTCTTCACCGTGCCAGACTAGAGTCAAGCTCAACAGGGTCT | 130875 | 0.15357164523547712 | No Hit |
| CTGAATTTAAGCATATTAGTCAGCGGAGGAGAAGAAACTAACCAGGATTC | 127616 | 0.1497474619168722 | No Hit |
| CACAGTTATCCAAGTAGGAGAGGAGCGAGCGACCAAAGGAACCATAACTG | 124917 | 0.14658039509363968 | No Hit |
| CCCGAAGTTACGGATCCGGCTTGCCGACTTCCCTTACCTACATTGTTCCA | 122871 | 0.14417957304090398 | No Hit |
| CCGTCGGCATGTATTAGCTCTAGAATTACCACAGTTATCCAAGTAGGAGA | 115698 | 0.135762614788571 | No Hit |
| CACCCGTTTACCTCTTAACGGTTTCACGCCCTCTTGAACTCTCTCTTCAA | 115590 | 0.1356358851787492 | No Hit |
| CCTCACCCGGCCCGGACACGGACAGGATTGACAGATTGATAGCTCTTTCT | 113359 | 0.13301797999807793 | No Hit |
| GTCAAAGTGAAGAAATTCAATGAAGCGCGGGTAAACGGCGGGAGTAACTA | 109447 | 0.1284275519089762 | No Hit |
| CTCCCGTCCACTCTCGACTGCCGGCGACGGCCGGGTATGGGCCCGACGCT | 108806 | 0.12767538820623742 | No Hit |
| CTGCTTACCAAAAGTGGCCCACTAGGCACTCGCATTCCACGCCCGGCTCC | 108532 | 0.1273538704924302 | No Hit |
| CGCGTAACTAGTTAGCATGCCAGAGTCTCGTTCGTTATCGGAATTAACCA | 108438 | 0.12724356879499268 | No Hit |
| GCCCTCTTGAACTCTCTCTTCAAAGTTCTTTTCAACTTTCCCTTACGGTA | 107585 | 0.12624263956186288 | No Hit |
| CCCTCCTTAGGCAACCTGGTGGTCCCCCGCTCCCGGGAGGTCACCATATT | 106785 | 0.12530390171133085 | No Hit |
| CCCATATCCGCAGCAGGTCTCCAAGGTGAACAGCCTCTGGCATGTTGGAA | 104781 | 0.12295236339574807 | No Hit |
| CCACTCTCGACTGCCGGCGACGGCCGGGTATGGGCCCGACGCTCCAGCGC | 104671 | 0.12282328694129989 | No Hit |
| CTCGTGCCGGTATTTAGCCTTAGATGGAGTTTACCACCCGCTTTGGGCTG | 103529 | 0.12148323865966541 | No Hit |
| CTCCATCTAAGGCTAAATACCGGCACGAGACCGATAGTCAACAAGTACCG | 103519 | 0.12147150443653375 | No Hit |
| CTCCGCCACTCCGGATTCGGGGATCTGAACCCGACTCCCTTTCGATCGGC | 102533 | 0.12031451003575301 | No Hit |
| CTTTAAATGGGTAAGAAGCCCGGCTCGCTGGCGTGGAGCCGGGCGTGGAA | 102476 | 0.1202476249639026 | No Hit |
| CGCGATGTGATTTCTGCCCAGTGCTCTGAATGTCAAAGTGAAGAAATTCA | 101723 | 0.1193640379620893 | No Hit |
| CTGAATTTAAGCATATTAGTCAGCGGAGGAAAAGAAACTAACCAGGATTC | 100790 | 0.11826923494390632 | No Hit |
| CGAGAACTTTGAAGGCCGAAGTGGAGAAGGGTTCCATGTGAACAGCAGTT | 97777 | 0.11473371351433997 | No Hit |
| CGAAGGCCCGCGGCGGGTGTTGACGCGATGTGATTTCTGCCCAGTGCTCT | 97437 | 0.11433474992786387 | No Hit |
| CGTCGGCATGTATTAGCTCTAGAATTACCACAGTTATCCAAGTAGGAGAG | 96748 | 0.11352626195409313 | No Hit |
| CTCAAAGATTAAGCCATGCATGTCTAAGTACGCACGGCCGGTACAGTGAA | 94156 | 0.1104847513183693 | No Hit |
| CAGAAACCTCCCGTGGAGCAGAAGGGCAAAAGCTCGCTTGATCTTGATTT | 93469 | 0.10967861018922492 | No Hit |
| ATCAGACGTGGCGACCCGCTGAATTTAAGCATATTAGTCAGCGGAGGAGA | 92026 | 0.10798536179132774 | No Hit |
| CGAACGCCGGGTTAAGGCGCCCGATGCCGACGCTCATCAGACCCCAGAAA | 91737 | 0.10764624274282303 | No Hit |
| CCTGCCAGTAGCATATGCTTGTCTCAAAGATTAAGCCATGCATGTCTAAG | 90762 | 0.10650215598748708 | No Hit |
| CAAACTTTAAATGGGTAAGAAGCCCGGCTCGCTGGCGTGGAGCCGGGCGT | 90182 | 0.10582157104585135 | No Hit |

| Sequence | Count | Percentage | Possible Source |
|---|---|---|---|
| CCACCGTCCTGCTGTCTATATCAACCAACACCTTTTCTGGGGTCTGATGA | 89799 | 0.10537215029990915 | No Hit |
| CTTTTGGTAAGCAGAACTGG[illegible]ATGAACCGAACGCCGGGTTAA | [illegible] | [illegible] | [illegible] |
| CTCAAACTTTAAATGGGTAAGAAGCCCGGCTCGCTGGCGTGGAGCCGGGC | 89423 | 0.10493094351015908 | No Hit |
| AAACGATCTCAACCTATTCTCAAACTTTAAATGGGTAAGAAGCCCGGCTC | 88619 | 0.10398751197037437 | No Hit |
| CTGGGGTCTGATGAGCGTCGGCATCGGGCGCCTTAACCCGGCGTTCGGTT | 88079 | 0.10335386392126525 | No Hit |
| CTCATGTCTCTTCACCGTGCCAGACTAGAGTCAAGCTCAACAGGGTCTTC | 87755 | 0.10297367509179975 | No Hit |
| CGCGTCACTAATTAGATGACGAGGCATTTGGCTACCTTAAGAGAGTCATA | 86978 | 0.10206192595447051 | No Hit |
| CCCAGGCATAGTTCACCATCTTTCGGGTCCTAACACGTGCGCTCGTGCTC | 86949 | 0.10202789670738872 | No Hit |
| CTCCCACTTATTCTACACCTCTCATGTCTCTTCACCGTGCCAGACTAGAG | 86800 | 0.10185305678272712 | No Hit |
| CTCCGACTTTCGTTCTTGATTAATGAAAACATTCTTGGCAAATGCTTTCG | 85715 | 0.10057989357294304 | No Hit |
| CTTTTCTTTGTGAAGGGCAGGGCGCCCTGGAATGGGTTCGCCCCGAGAGA | 85689 | 0.10054938459280074 | No Hit |
| CCCACTTATTCTACACCTCTCATGTCTCTTCACCGTGCCAGACTAGAGTC | 85481 | 0.10030531275166243 | No Hit |

## Adapter Content

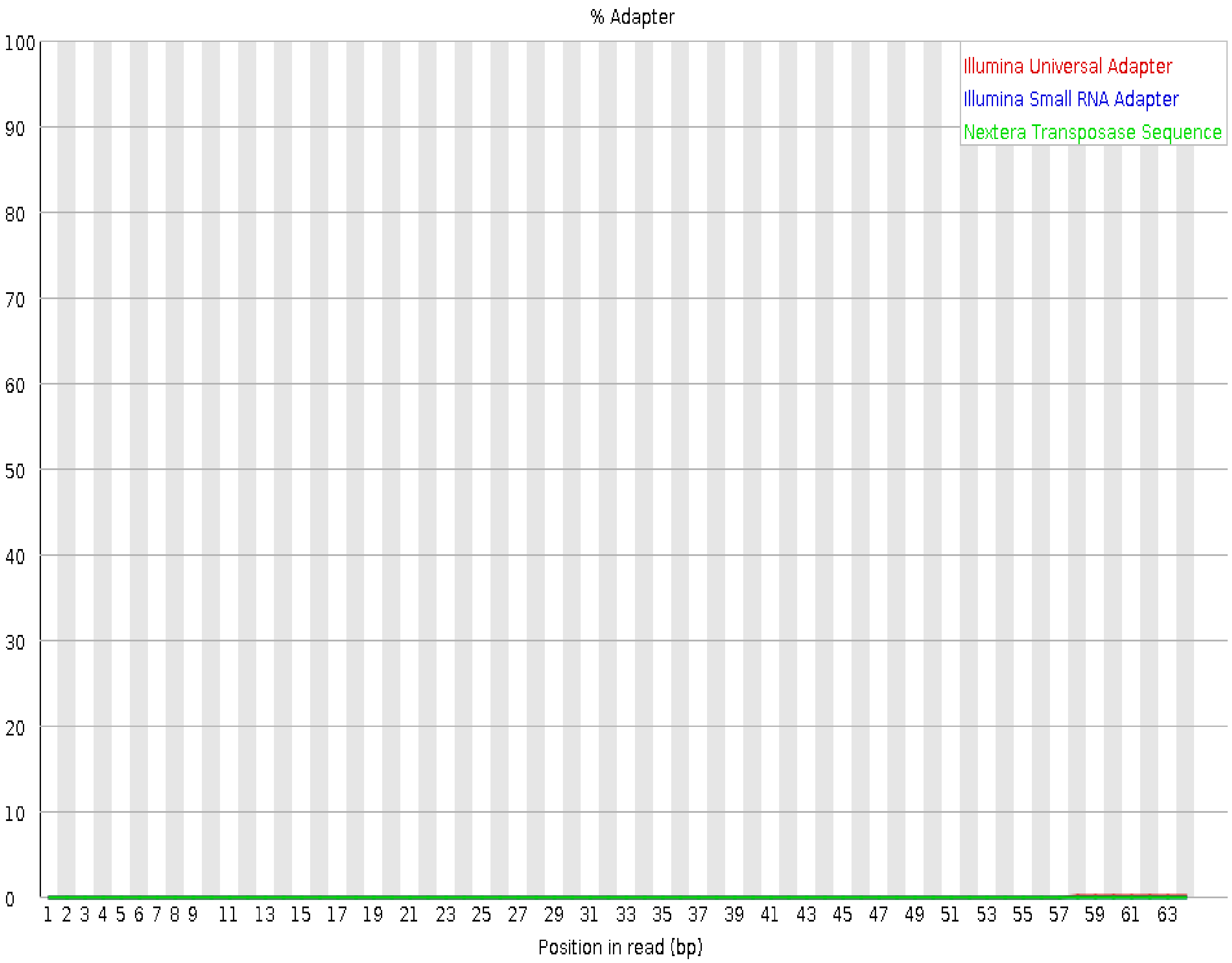

# Kmer Content

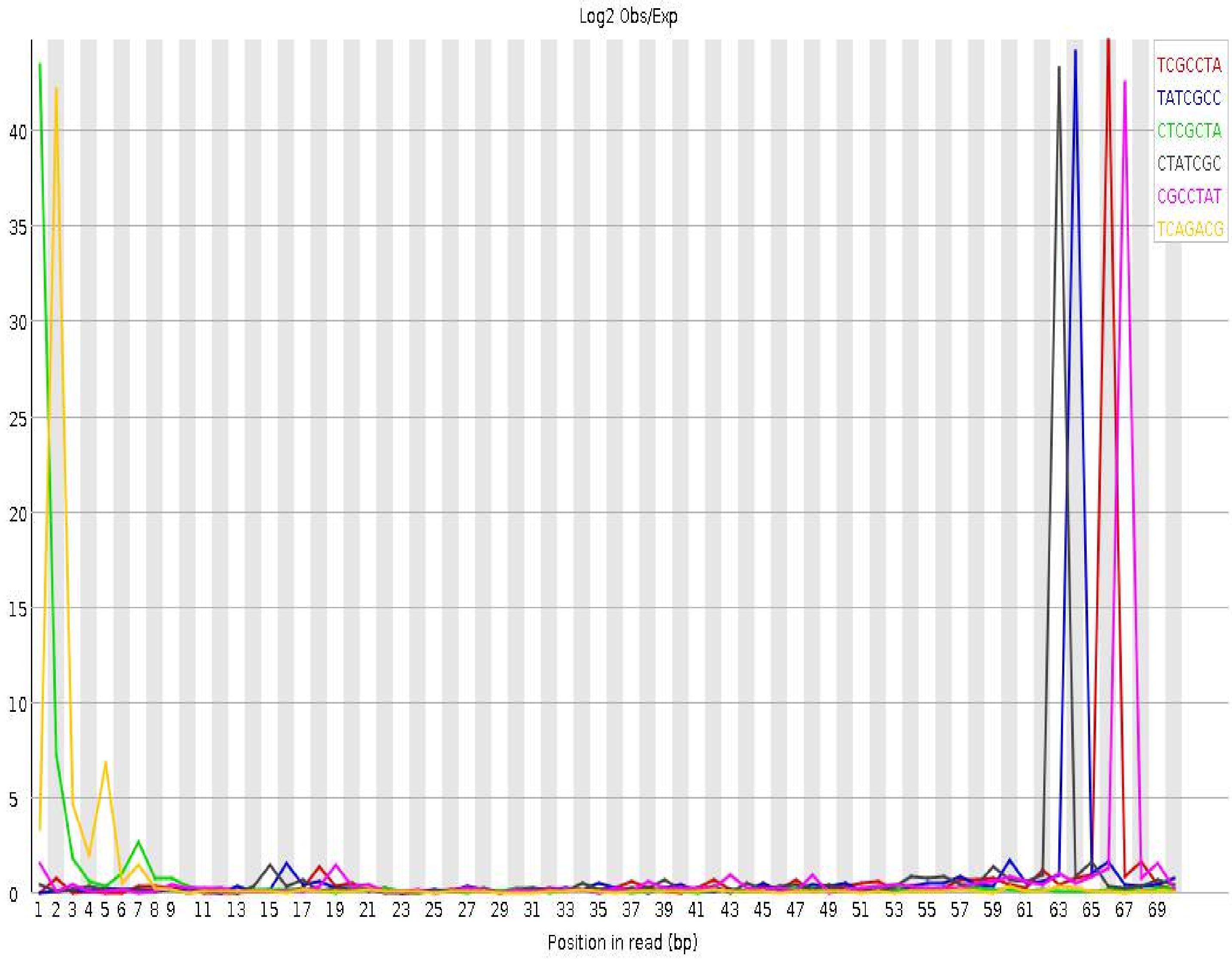


| Sequence | Count | PValue | Obs/Exp Max | Max Obs/Exp Position |
|---|---|---|---|---|
| TCGCCTA | 9820 | 0.0 | 44.69061 | 66 |
| TATCGCC | 9990 | 0.0 | 44.10343 | 64 |
| CTCGCTA | 13975 | 0.0 | 43.44008 | 1 |
| CTATCGC | 10130 | 0.0 | 43.284393 | 63 |
| CGCCTAT | 10350 | 0.0 | 42.470848 | 67 |
| TCAGACG | 36945 | 0.0 | 42.132706 | 2 |
| TCACTCG | 84475 | 0.0 | 41.284855 | 67 |
| ACTCGCC | 84490 | 0.0 | 41.146156 | 69 |
| CTGCCAG | 61595 | 0.0 | 39.486183 | 1 |
| ATCGCCT | 11445 | 0.0 | 38.650368 | 65 |
| TGCCAGT | 63380 | 0.0 | 37.85594 | 2 |
| CGCGTAA | 23145 | 0.0 | 37.51352 | 1 |
| | | | | |

| Sequence | Count | PValue | Obs/Exp Max | Max Obs/Exp Position |
|---|---|---|---|---|
| TTCACTC | 93715 | 0.0 | 37.370163 | 66 |
| TGTCGAG | 9705 | 0.0 | 37.[illegible] | 5 |
| CCAGTAG | 65100 | 0.0 | 36.89226 | 4 |
| GCCAGTA | 65365 | 0.0 | 36.583046 | 3 |
| GTGTCGA | 10260 | 0.0 | 35.277843 | 4 |
| TGTGTCG | 10450 | 0.0 | 34.3024 | 3 |
| GCGTAAC | 25670 | 0.0 | 34.08364 | 2 |
| CAGTAGC | 70645 | 0.0 | 33.990837 | 5 |

**Produced by FastQC (version 0.11.2)**

# FastQC Report

Mon 4 May 2015
Biochain_Adult_Lung_CGATGT_L002_R1.fastq.gz

## Summary


- Basic Statistics
- Per base sequence quality
- Per tile sequence quality
- Per sequence quality scores
- Per base sequence content
- Per sequence GC content
- Per base N content
- Sequence Length Distribution
- Sequence Duplication Levels
- Overrepresented sequences
- Adapter Content
- Kmer Content


## Basic Statistics

| Measure | Value |
|---|---|
| Filename | Biochain_Adult_Lung_CGATGT_L002_R1.fastq.gz |
| File type | Conventional base calls |
| Encoding | Sanger / Illumina 1.9 |
| Total Sequences | 82080766 |
| Sequences flagged as poor quality | 0 |
| Sequence length | 76 |
| %GC | 52 |

## Per base sequence quality

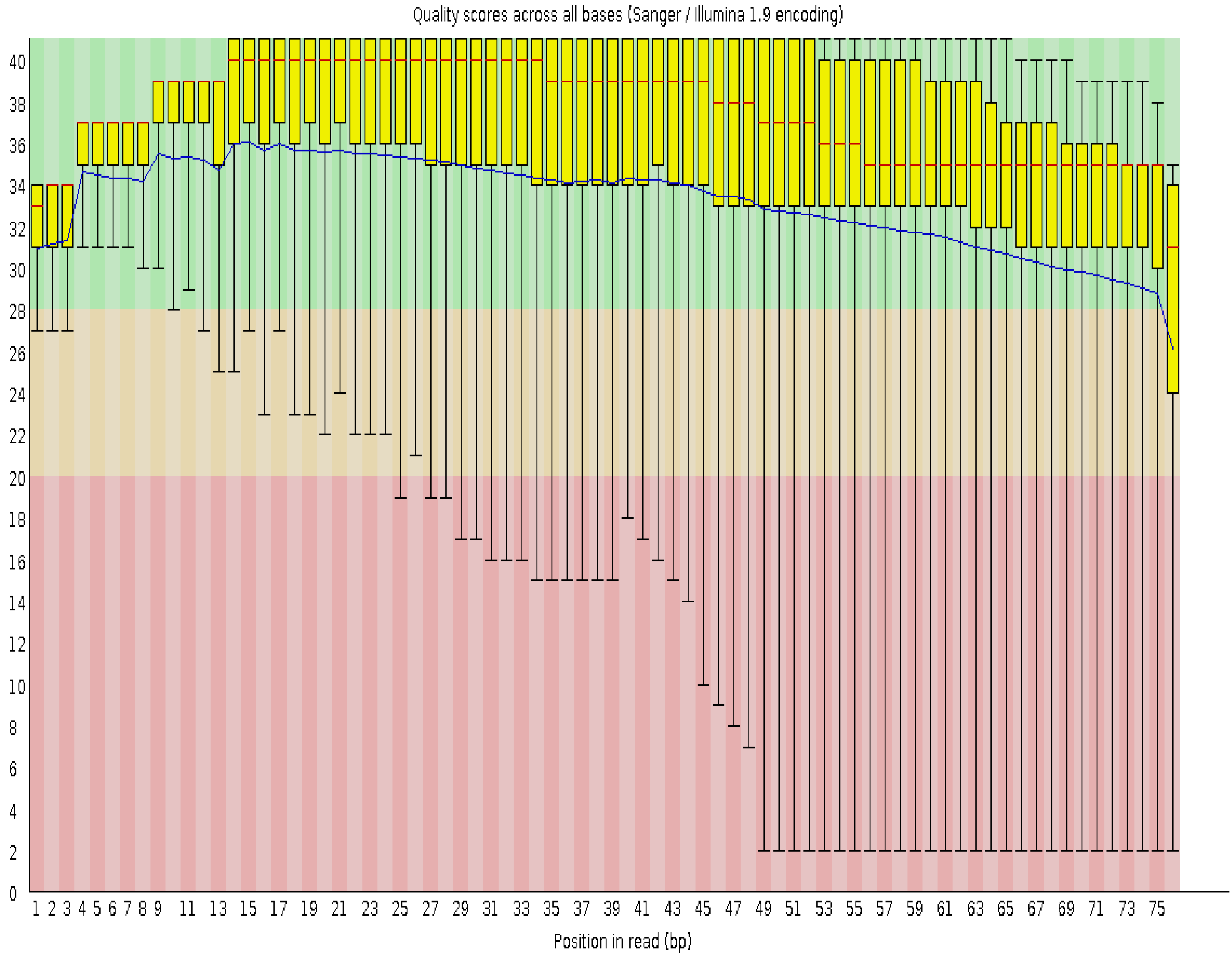


## Per tile sequence quality

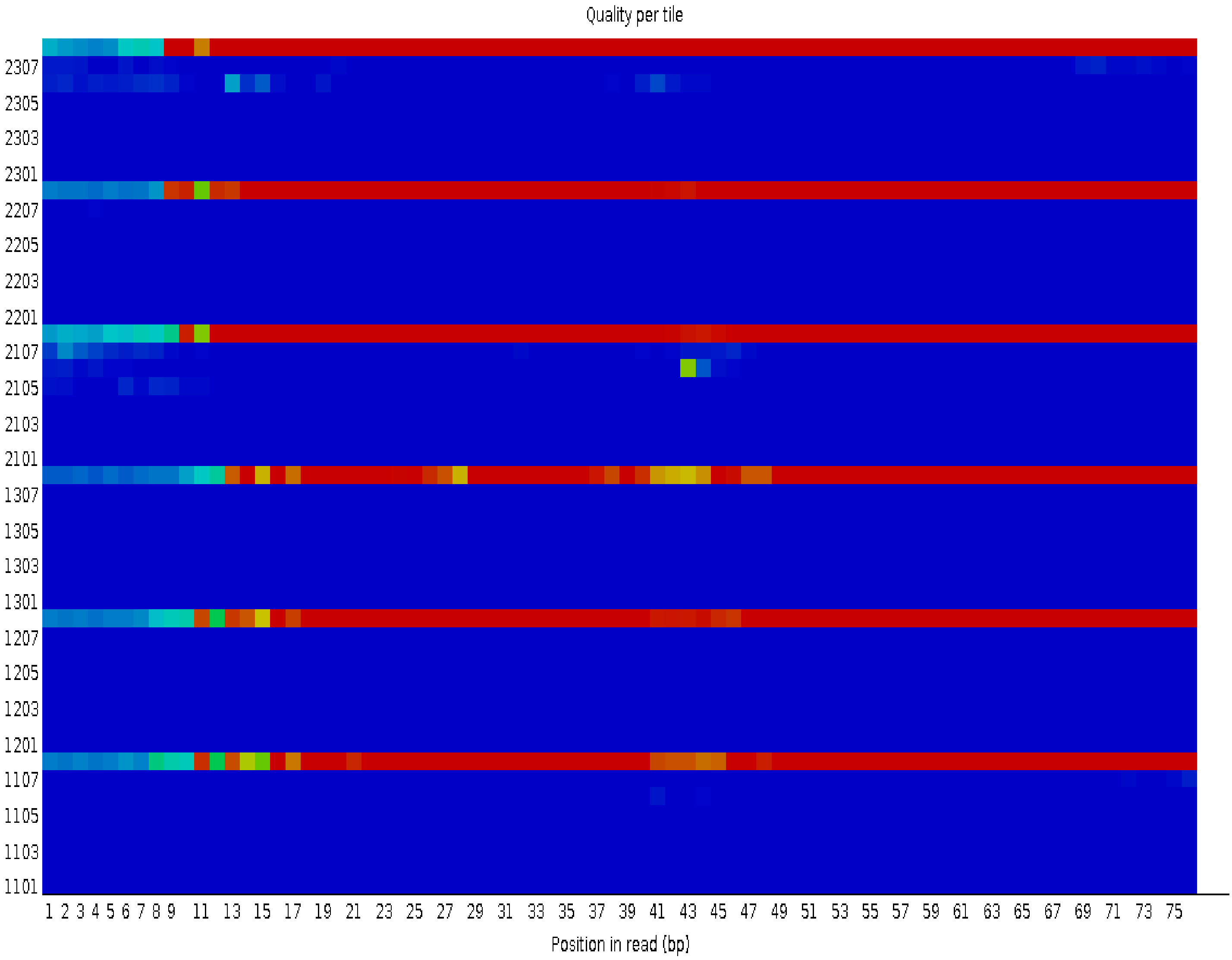


## Per sequence quality scores

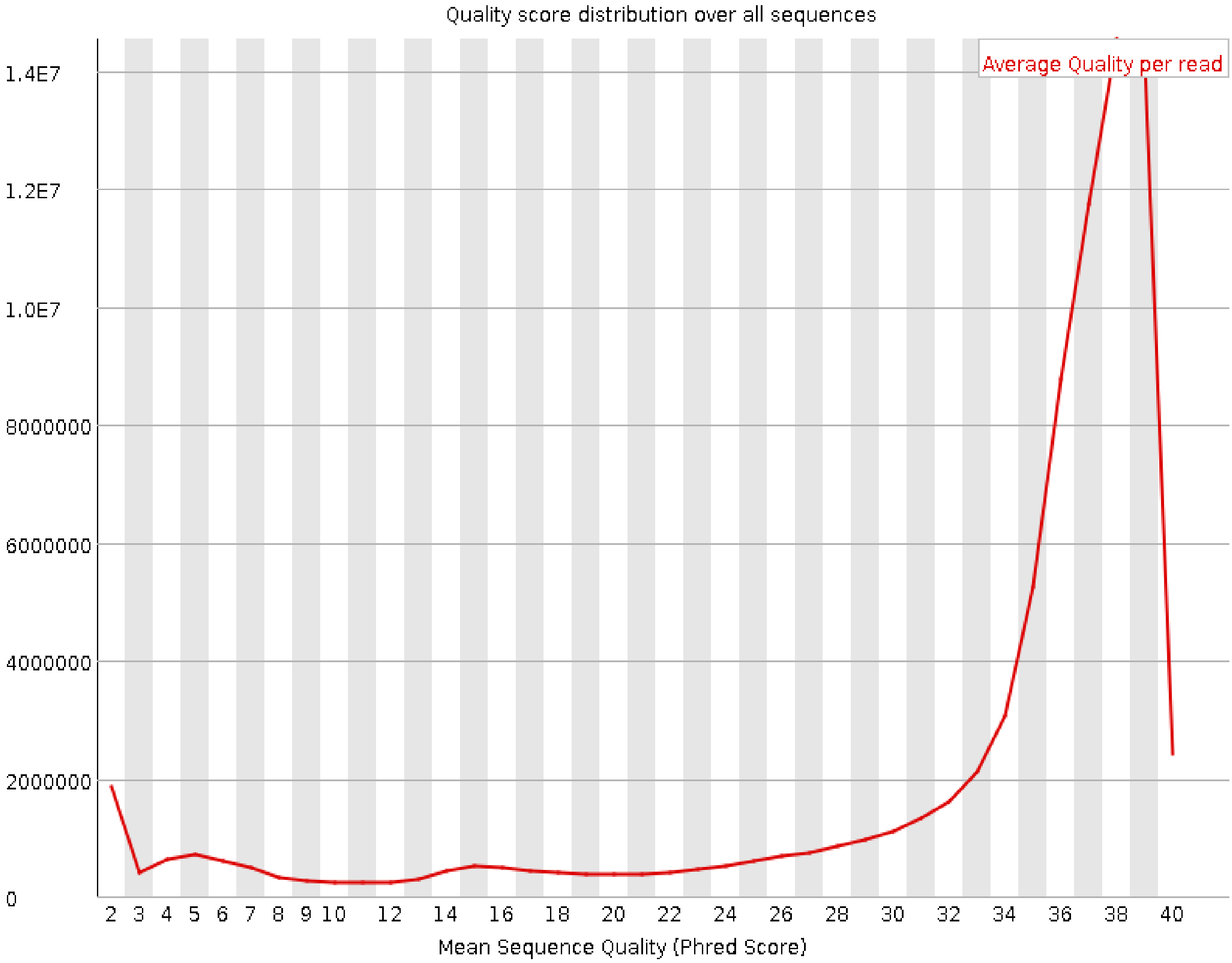


## Per base sequence content

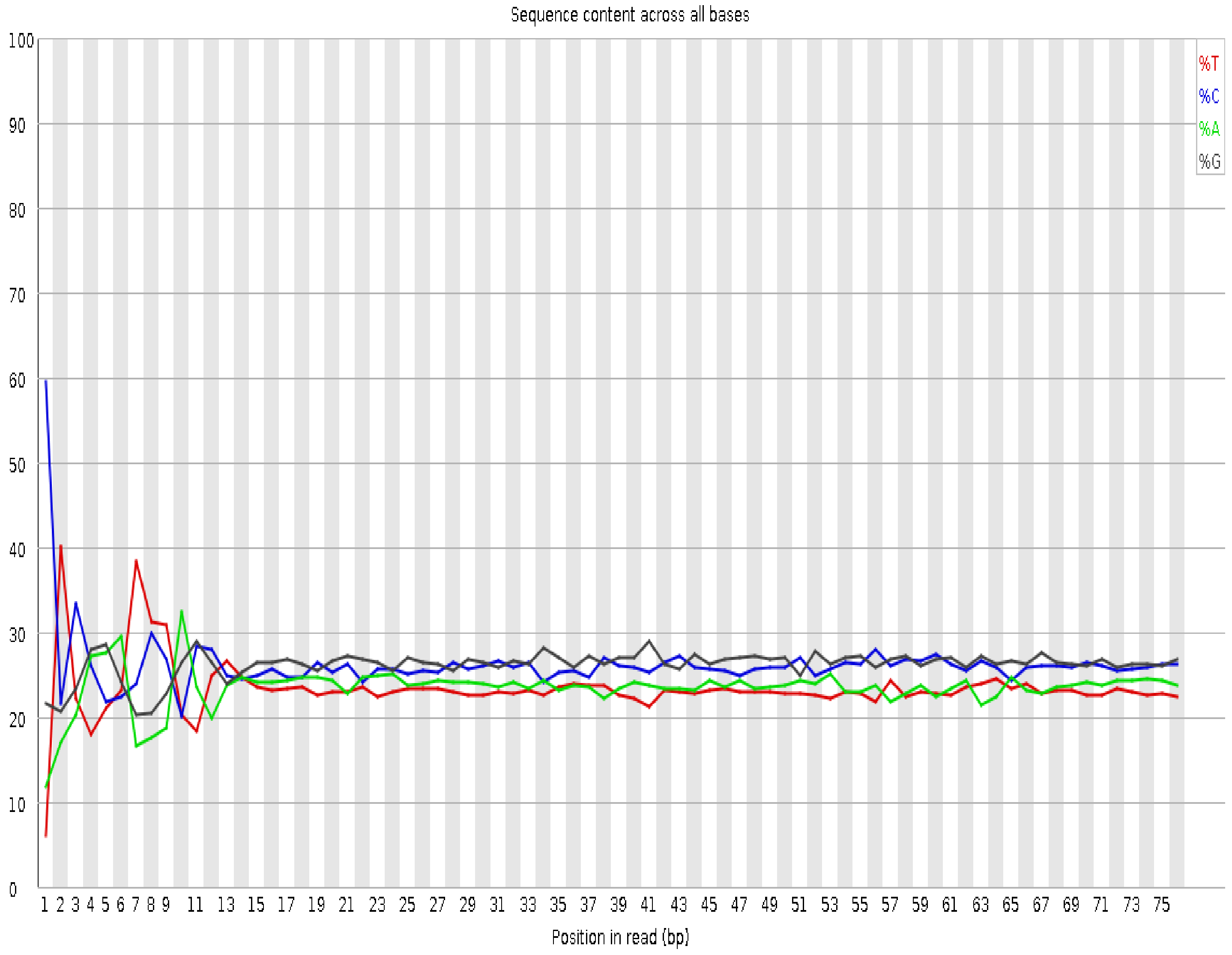


## Per sequence GC content

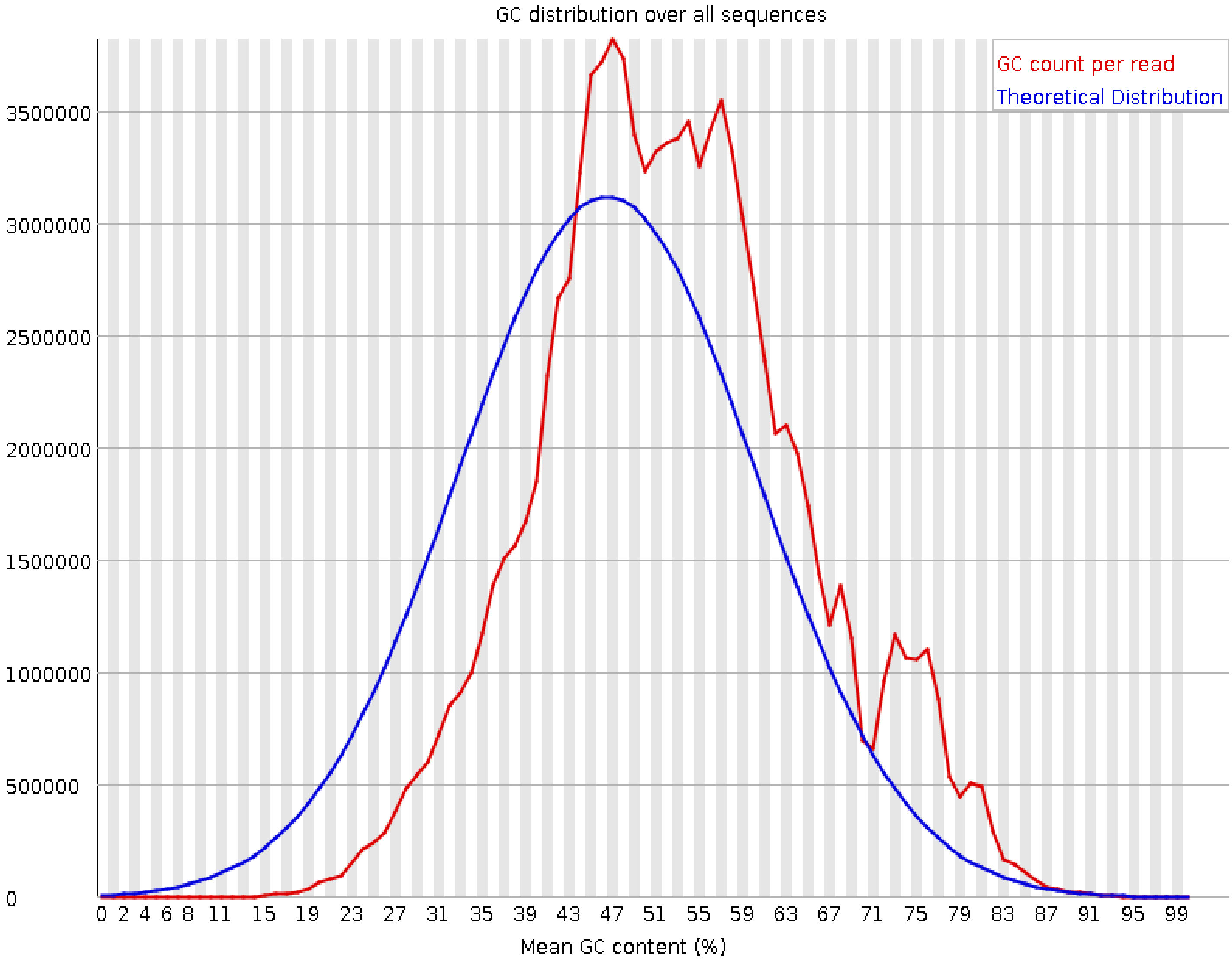


## Per base N content

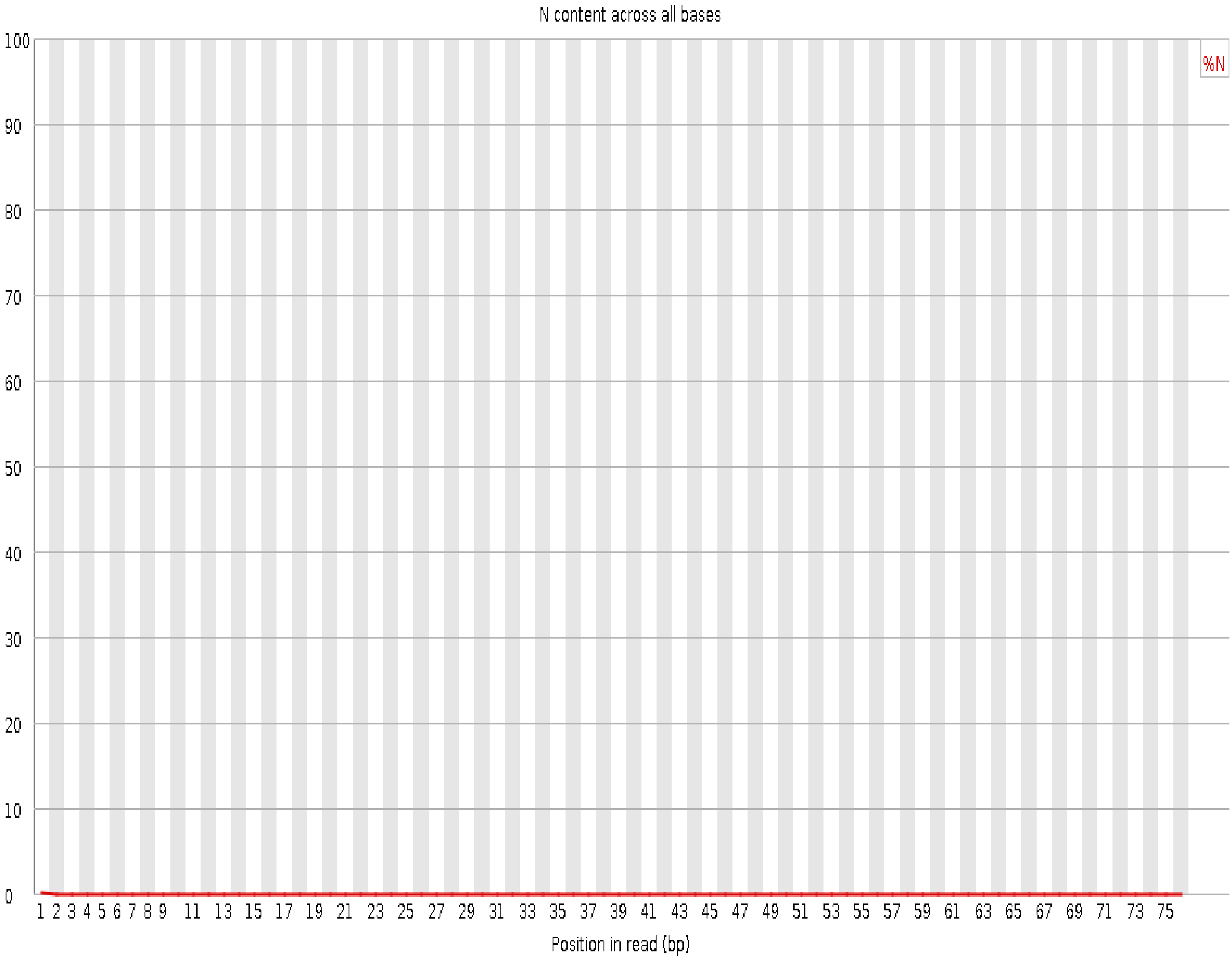


## Sequence Length Distribution

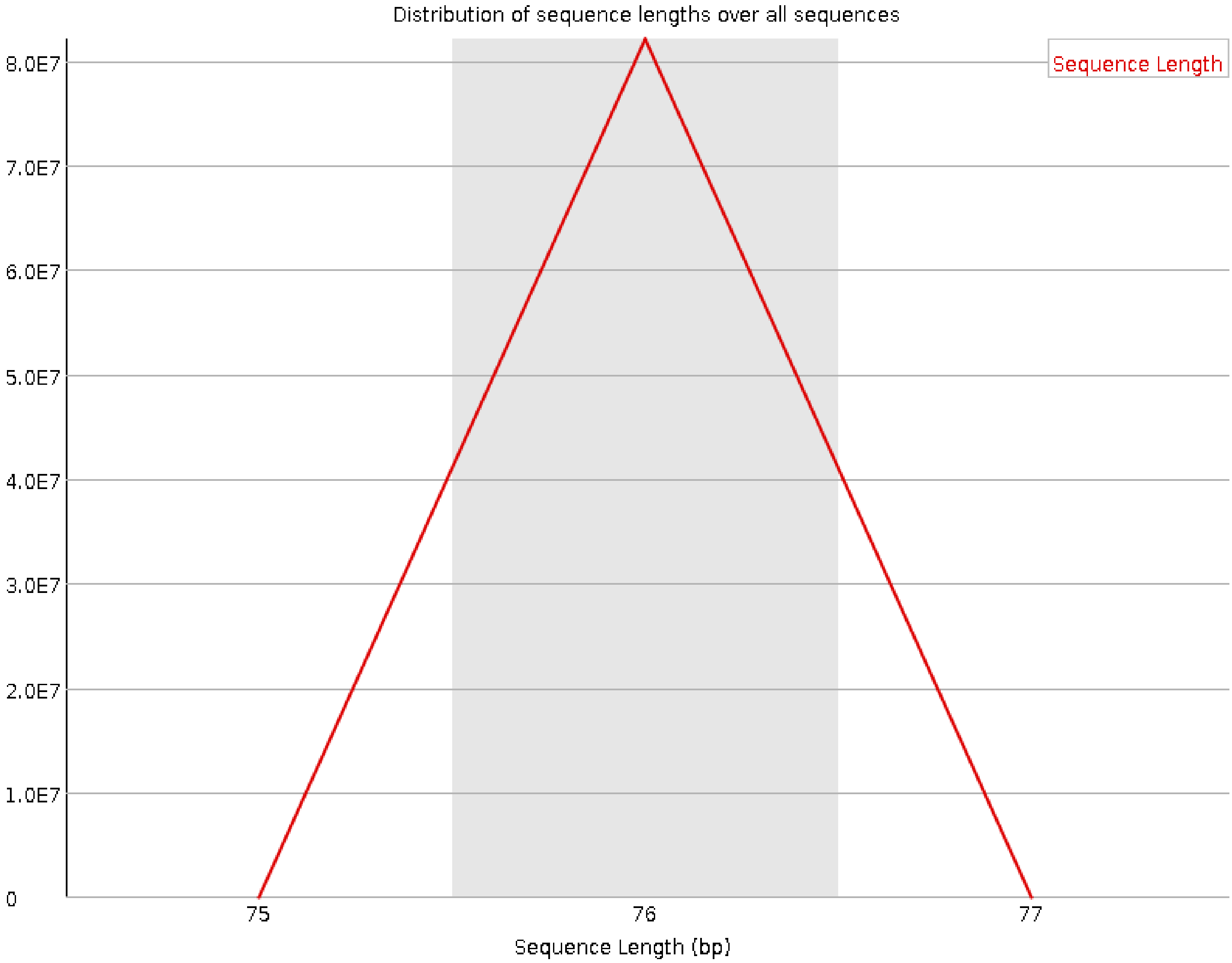


## Sequence Duplication Levels

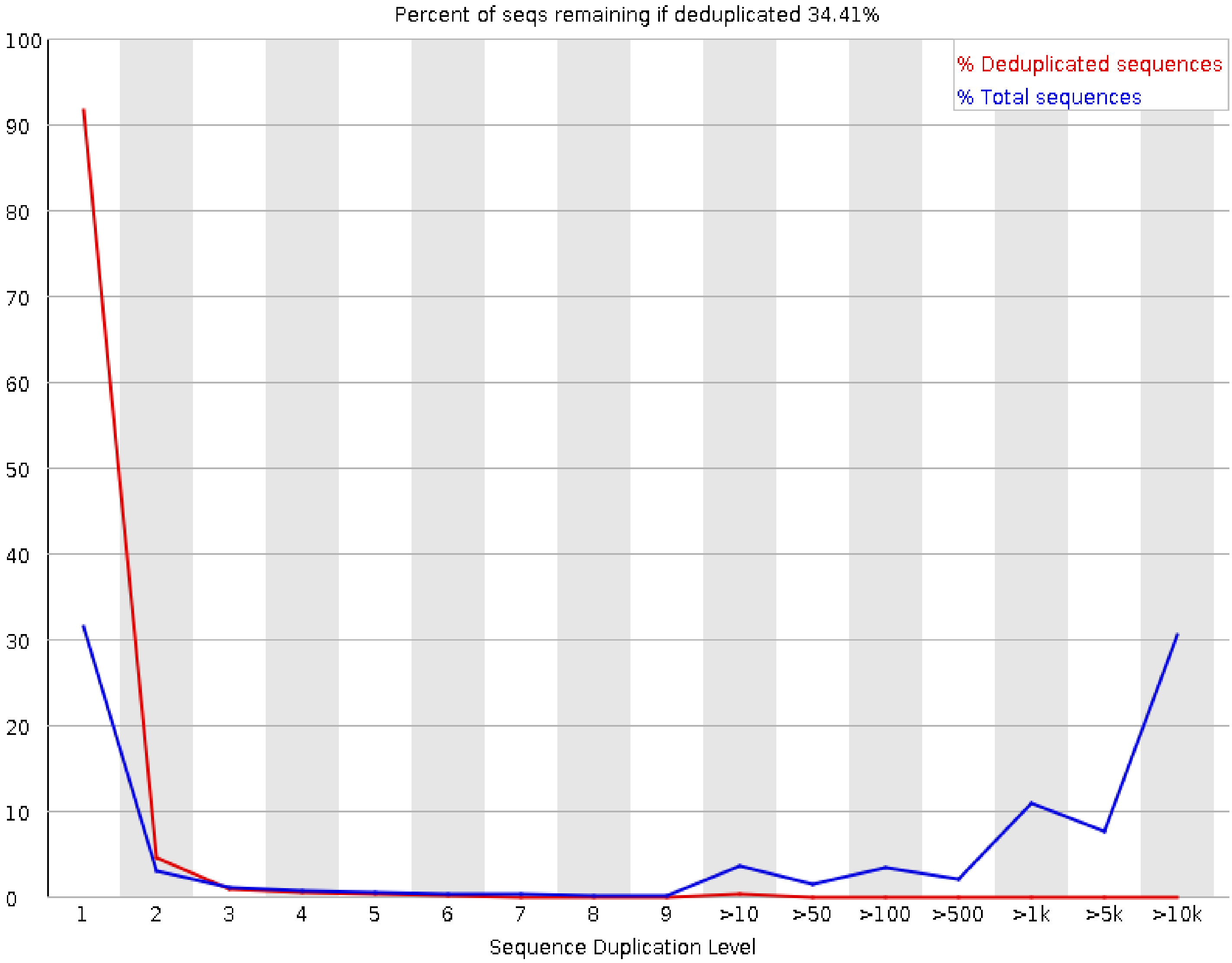


## Overrepresented sequences

| Sequence | Count | Percentage | Possible Source |
|---|---|---|---|
| CCGGTATTTAGCCTTAGATGGAGTTTACCACCCGCTTTGGGCTGCATTCC | 301970 | 0.3678937401729414 | No Hit |
| CTTCCGTACGCCACATGTCCCGCGCCCCGCCGCGGGGCGGGGATTCGGCG | 296195 | 0.3608579871196621 | No Hit |
| CTCCCTTTCGATCGGCCGAGGGCAACGGAGGCCATCGCCCGTCCCTTCGG | 227448 | 0.27710267713632203 | No Hit |
| CTCTCTTCAAAGTTCTTTTCAACTTTCCCTTACGGTACTTGTTGACTATC | 185711 | 0.2262539801346396 | No Hit |
| CGCAGTTTTATCCGGTAAAGCGAATGATTAGAGGTCTTGGGGCCGAAACG | 182553 | 0.22240655015329658 | No Hit |
| CCCGTCGGCATGTATTAGCTCTAGAATTACCACAGTTATCCAAGTAGGAG | 180944 | 0.22044628579611453 | No Hit |
| CTGGATAGTAGGTAGGGACAGTGGGAATCTCGTTCATCCATTCATGCGCG | 175472 | 0.21377968134459174 | No Hit |
| CTCCGACTTTCGTTCTTGATTAATGAAAACATTCTTGGCAAATGCTTTCG | 156278 | 0.19039539665114724 | No Hit |
| CTTCACCGTGCCAGACTAGAGTCAAGCTCAACAGGGTCTTCTTTCCCCGC | 140725 | 0.17144698673986547 | No Hit |
| GTCAAAGTGAAGAAATTCAATGAAGCGCGGGTAAACGGCGGGAGTAACTA | 139008 | 0.16935514466324547 | No Hit |
| CTTTAAATGGGTAAGAAGCCCGGCTCGCTGGCGTGGAGCCGGGCGTGGAA | 138363 | 0.16856933328326884 | No Hit |

| Sequence | Count | Percentage | Possible Source |
|---|---|---|---|
| CTGCCAGTAGCATATGCTTGTCTCAAAGATTAAGCCATGCATGTCTAAGT | 135938 | 0.16561492615700005 | No Hit |
| CTTAGATGGAGTTTACCACCCGCTTTGGGCTGCATTCCCAAGCAACCCGA | 135014 | 0.16448920566847536 | N[illegible] |
| CTTGAACTCTCTCTTCAAAGTTCTTTTCAACTTTCCCTTACGGTACTTGT | 132300 | 0.16118270631148837 | No Hit |
| CCTCACCCGGCCCGGACACGGACAGGATTGACAGATTGATAGCTCTTTCT | 131817 | 0.16059426151066863 | No Hit |
| CCCGAAGTTACGGATCCGGCTTGCCGACTTCCCTTACCTACATTGTTCCA | 131760 | 0.1605248177142986 | No Hit |
| CTGCTGTCTATATCAACCAACACCTTTTCTGGGGTCTGATGAGCGTCGGC | 128036 | 0.1559878230181234 | No Hit |
| CGCGTAACTAGTTAGCATGCCAGAGTCTCGTTCGTTATCGGAATTAACCA | 127207 | 0.15497784219021543 | No Hit |
| CTCTCATGTCTCTTCACCGTGCCAGACTAGAGTCAAGCTCAACAGGGTCT | 121625 | 0.14817722339482067 | No Hit |
| CTCGCATTCCACGCCCGGCTCCACGCCAGCGAGCCGGGCTTCTTACCCAT | 121434 | 0.1479445257613702 | No Hit |
| CAAAGATTAAGCCATGCATGTCTAAGTACGCACGGCCGGTACAGTGAAAC | 119953 | 0.14614020536796649 | No Hit |
| CGGGTCTTCCGTACGCCACATGTCCCGCGCCCCGCCGCGGGGCGGGGATT | 115287 | 0.14045556056336023 | No Hit |
| CTGAATTTAAGCATATTAGTCAGCGGAGGAAAAGAAACTAACCAGGATTC | 109992 | 0.13400459737424966 | No Hit |
| CTTTTCTTTGTGAAGGGCAGGGCGCCCTGGAATGGGTTCGCCCCGAGAGA | 109377 | 0.13325533536078354 | No Hit |
| CTTGTCTCAAAGATTAAGCCATGCATGTCTAAGTACGCACGGCCGGTACA | 108654 | 0.1323744956278795 | No Hit |
| CCCGCTTTGGGCTGCATTCCCAAGCAACCCGACTCCGGGAAGACCCGGGC | 108074 | 0.131667874542009 | No Hit |
| CTCCCACTTATTCTACACCTCTCATGTCTCTTCACCGTGCCAGACTAGAG | 106725 | 0.13002437136125167 | No Hit |
| CTCACCCGGCCCGGACACGGACAGGATTGACAGATTGATAGCTCTTTCTC | 103104 | 0.1256128628234293 | No Hit |
| CGAGAACTTTGAAGGCCGAAGTGGAGAAGGGTTCCATGTGAACAGCAGTT | 99333 | 0.12101860745305423 | No Hit |
| CGCGATGTGATTTCTGCCCAGTGCTCTGAATGTCAAAGTGAAGAAATTCA | 97908 | 0.1192825125438035 | No Hit |
| CTCCACTTCGGCCTTCAAAGTTCTCGTTTGAATATTTGCTACTACCACCA | 97012 | 0.11819090479735532 | No Hit |
| CTCGATCAGAAGGACTTGGGCCCCCCACGAGCGGCGCCGGGGAGCGGGTC | 94548 | 0.11518898349462284 | No Hit |
| GCCCTCTTGAACTCTCTCTTCAAAGTTCTTTTCAACTTTCCCTTACGGTA | 90058 | 0.10971876164021178 | No Hit |
| CAAACTTTAAATGGGTAAGAAGCCCGGCTCGCTGGCGTGGAGCCGGGCGT | 86097 | 0.1048930269486033 | No Hit |
| CCCATATCCGCAGCAGGTCTCCAAGGTGAACAGCCTCTGGCATGTTGGAA | 85153 | 0.10374294021573824 | No Hit |
| CACCCGTTTACCTCTTAACGGTTTCACGCCCTCTTGAACTCTCTCTTCAA | 84717 | 0.10321175608911837 | No Hit |
| CTCAAACTTTAAATGGGTAAGAAGCCCGGCTCGCTGGCGTGGAGCCGGGC | 84280 | 0.10267935365028148 | No Hit |
| CTCCGCCACTCCGGATTCGGGGATCTGAACCCGACTCCCTTTCGATCGGC | 84245 | 0.10263671272268585 | No Hit |
| CTGAATGTCAAAGTGAAGAAATTCAATGAAGCGCGGGTAAACGGCGGGAG | 83071 | 0.10120641417990667 | No Hit |

## Adapter Content

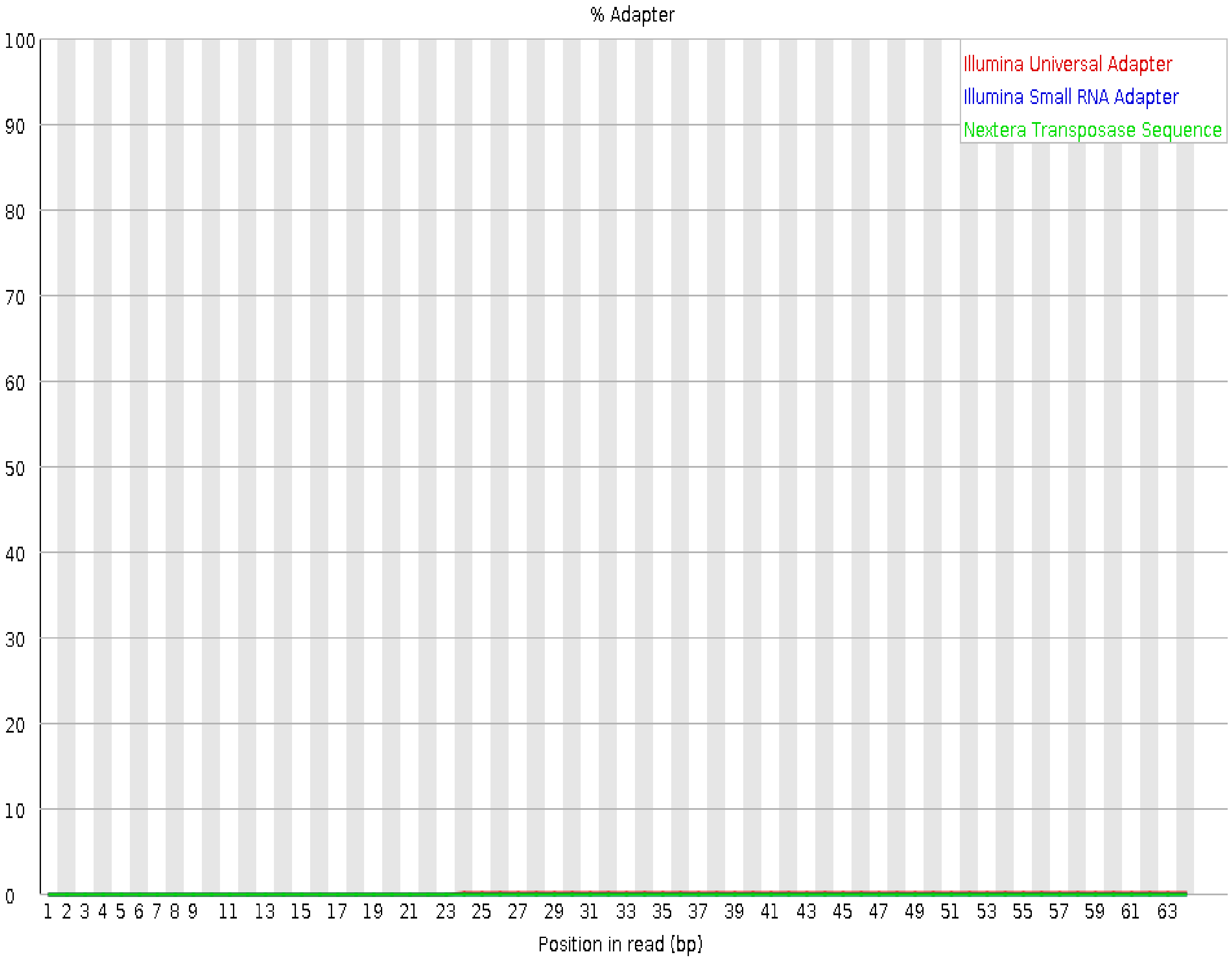


## Kmer Content

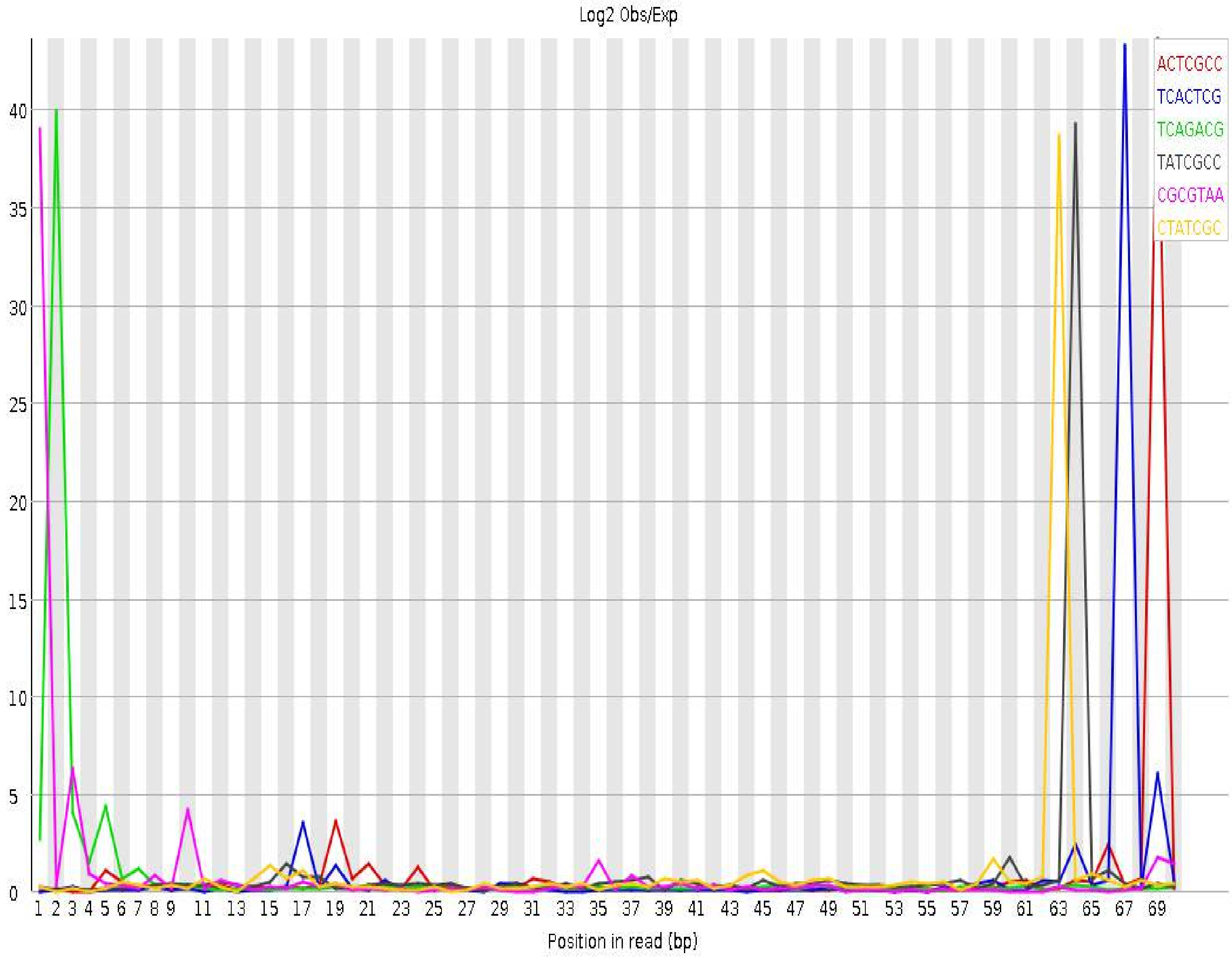


| Sequence | Count | PValue | Obs/Exp Max | Max Obs/Exp Position |
|---|---|---|---|---|
| ACTCGCC | 55660 | 0.0 | 43.598335 | 69 |
| TCACTCG | 55935 | 0.0 | 43.277927 | 67 |
| TCAGACG | 28050 | 0.0 | 39.98945 | 2 |
| TATCGCC | 8565 | 0.0 | 39.265972 | 64 |
| CGCGTAA | 28560 | 0.0 | 39.051292 | 1 |
| CTATCGC | 8715 | 0.0 | 38.677143 | 63 |
| TCGCCTA | 8805 | 0.0 | 38.151203 | 66 |
| CGCCTAT | 9235 | 0.0 | 36.375072 | 67 |
| GCGTAAC | 31925 | 0.0 | 35.27812 | 2 |
| CTCGCCG | 79110 | 0.0 | 34.66432 | 70 |
| TTCACTC | 71390 | 0.0 | 33.918377 | 66 |
| CGGAACG | 54600 | 0.0 | 33.90397 | 48 |
| ATCGCCT | 10255 | 0.0 | 33.030132 | 65 |
| ACGGCGC | 58170 | 0.0 | 32.79399 | 52 |
| CTCCGAC | 47065 | 0.0 | 31.809916 | 1 |

| Sequence | Count | PValue | Obs/Exp Max | Max Obs/Exp Position |
| --- | --- | --- | --- | --- |
| CCTTCGG | 58010 | 0.0 | [illegible] | 44 |
| GCCAGTA | 37265 | 0.0 | [illegible] | 3 |
| TCGCCCA | 63695 | 0.0 | 30.097893 | 59 |
| CTCGCTA | 7795 | 0.0 | 30.074612 | 1 |
| GTTCACT | 80640 | 0.0 | 30.023643 | 65 |

**Produced by FastQC (version 0.11.2)**

# FastQC Report

Mon 4 May 2015
Biochain_Adult_Lung_CGATGT_L002_R2.fastq.gz

## Summary


- Basic Statistics
- Per base sequence quality
- Per tile sequence quality
- Per sequence quality scores
- Per base sequence content
- Per sequence GC content
- Per base N content
- Sequence Length Distribution
- Sequence Duplication Levels
- Overrepresented sequences
- Adapter Content
- Kmer Content


## Basic Statistics

| Measure | Value |
|---|---|
| Filename | Biochain_Adult_Lung_CGATGT_L002_R2.fastq.gz |
| File type | Conventional base calls |
| Encoding | Sanger / Illumina 1.9 |
| Total Sequences | 82080766 |
| Sequences flagged as poor quality | 0 |
| Sequence length | 76 |
| %GC | 52 |

## Per base sequence quality

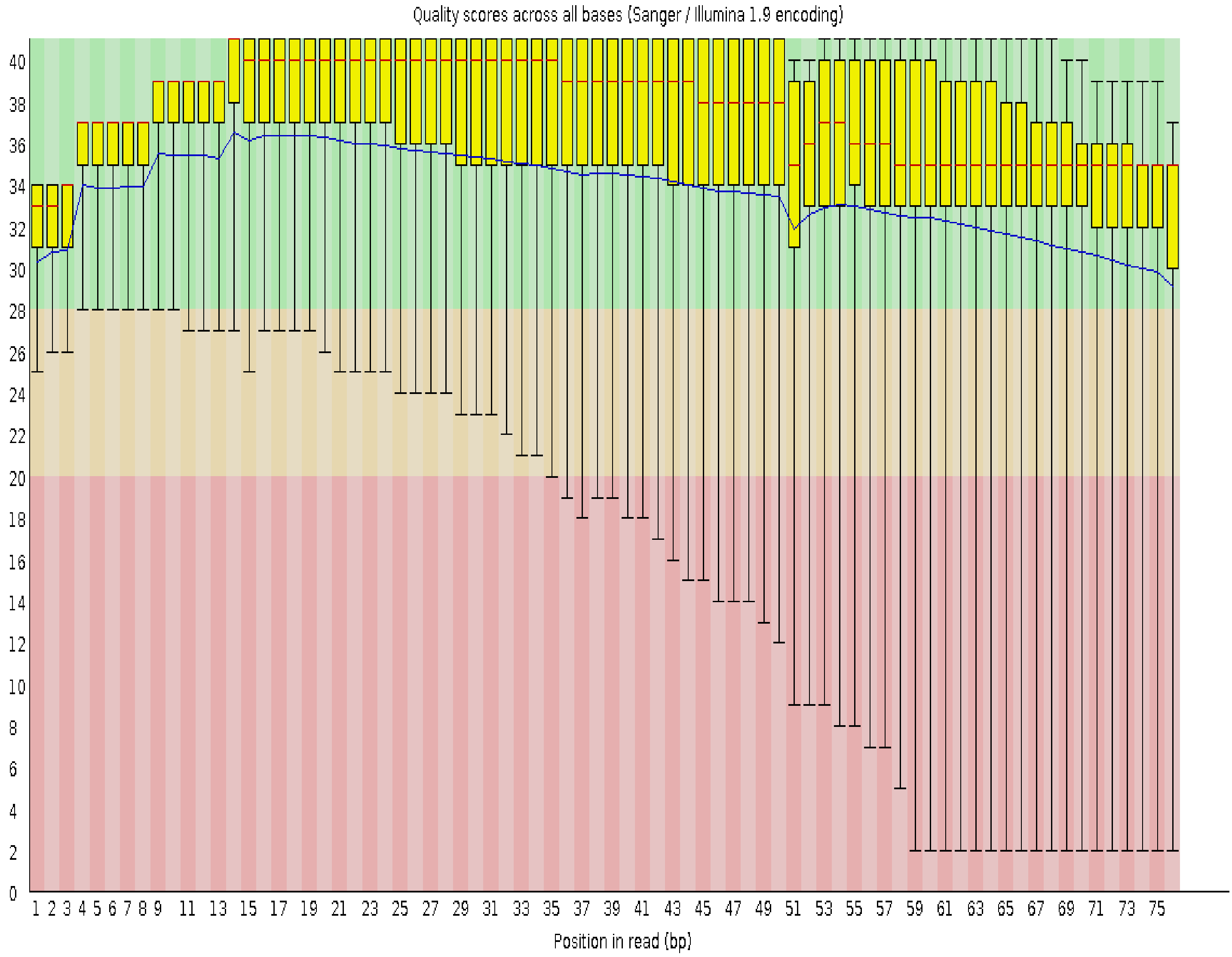


## Per tile sequence quality

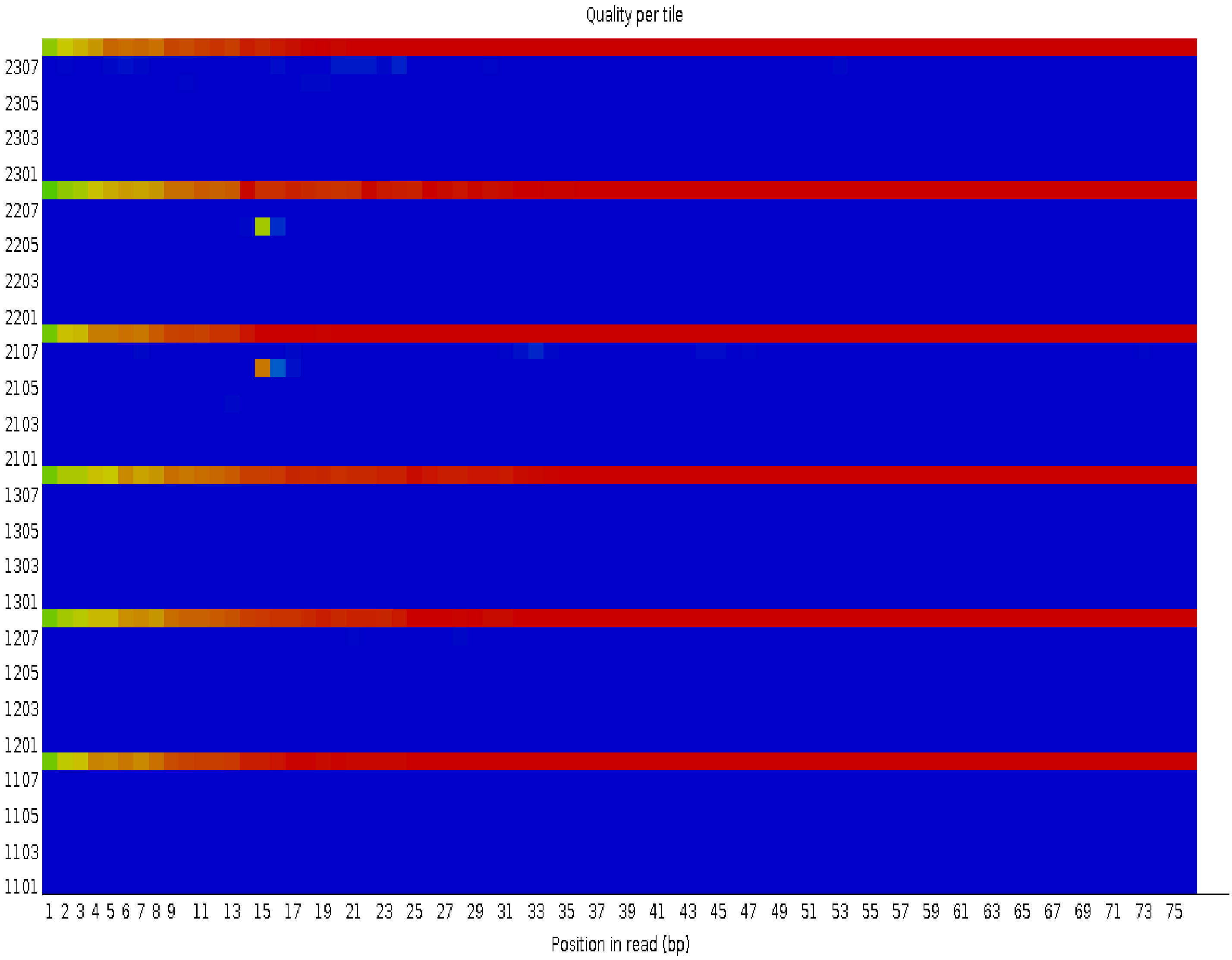


## Per sequence quality scores

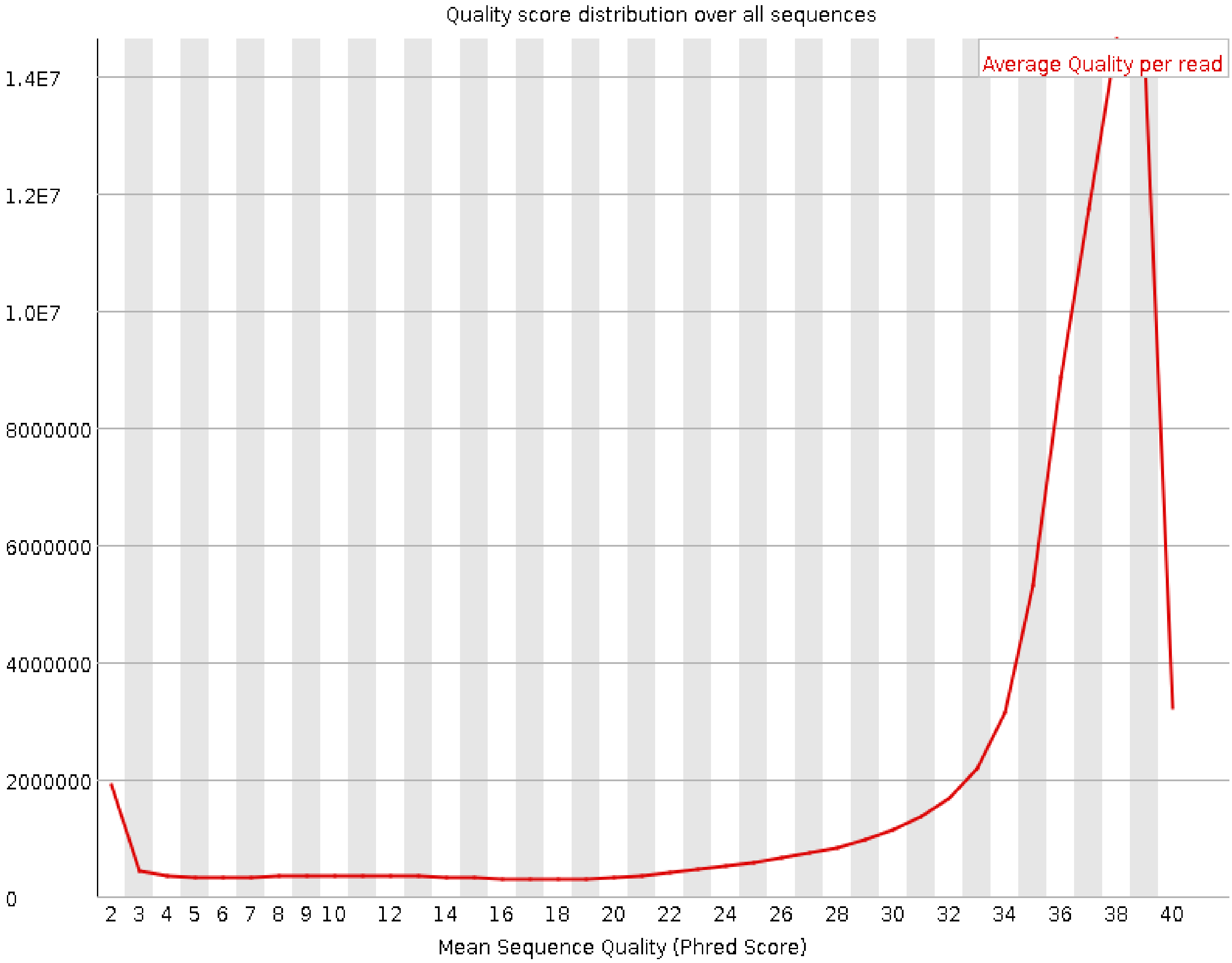


## Per base sequence content

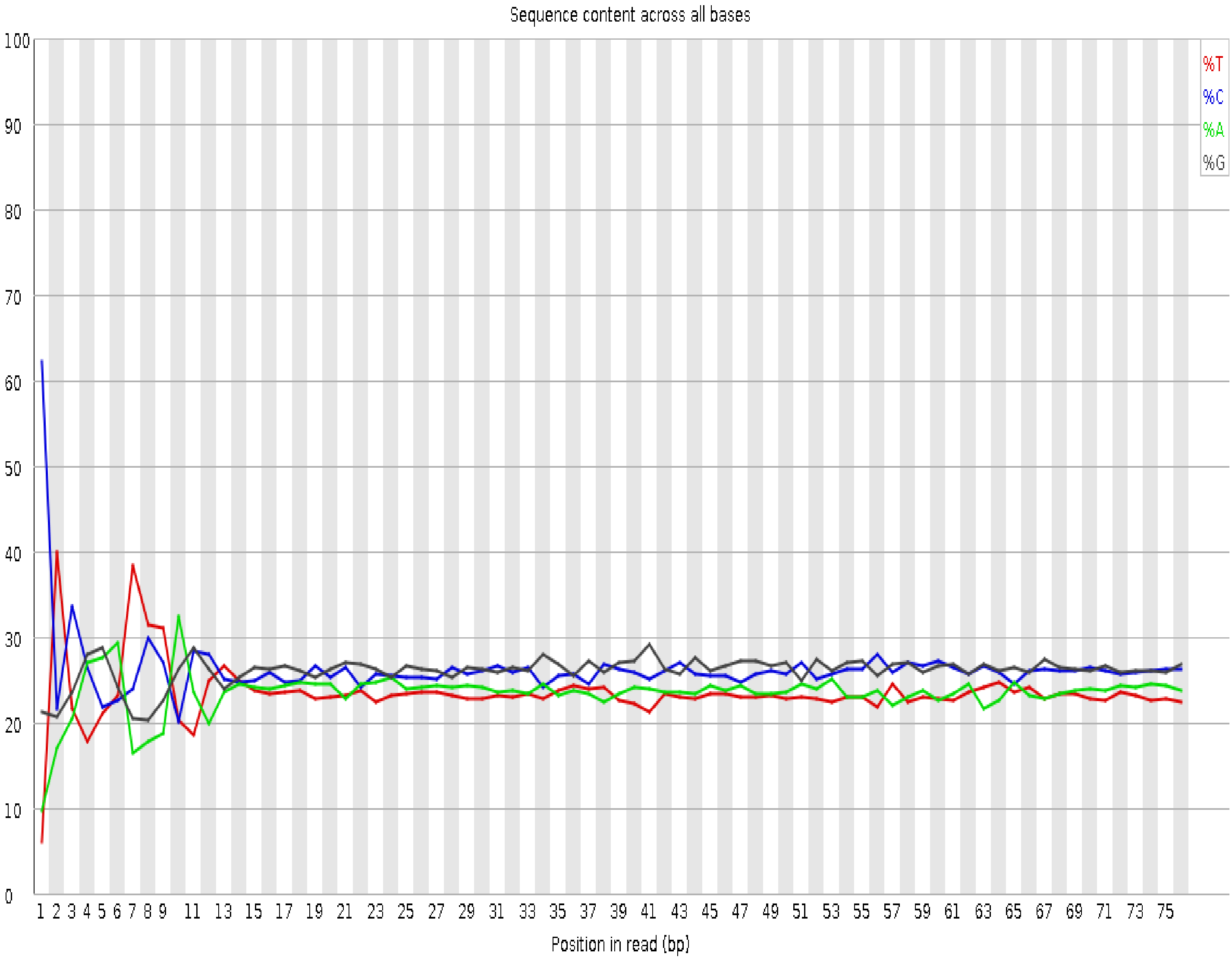


## Per sequence GC content

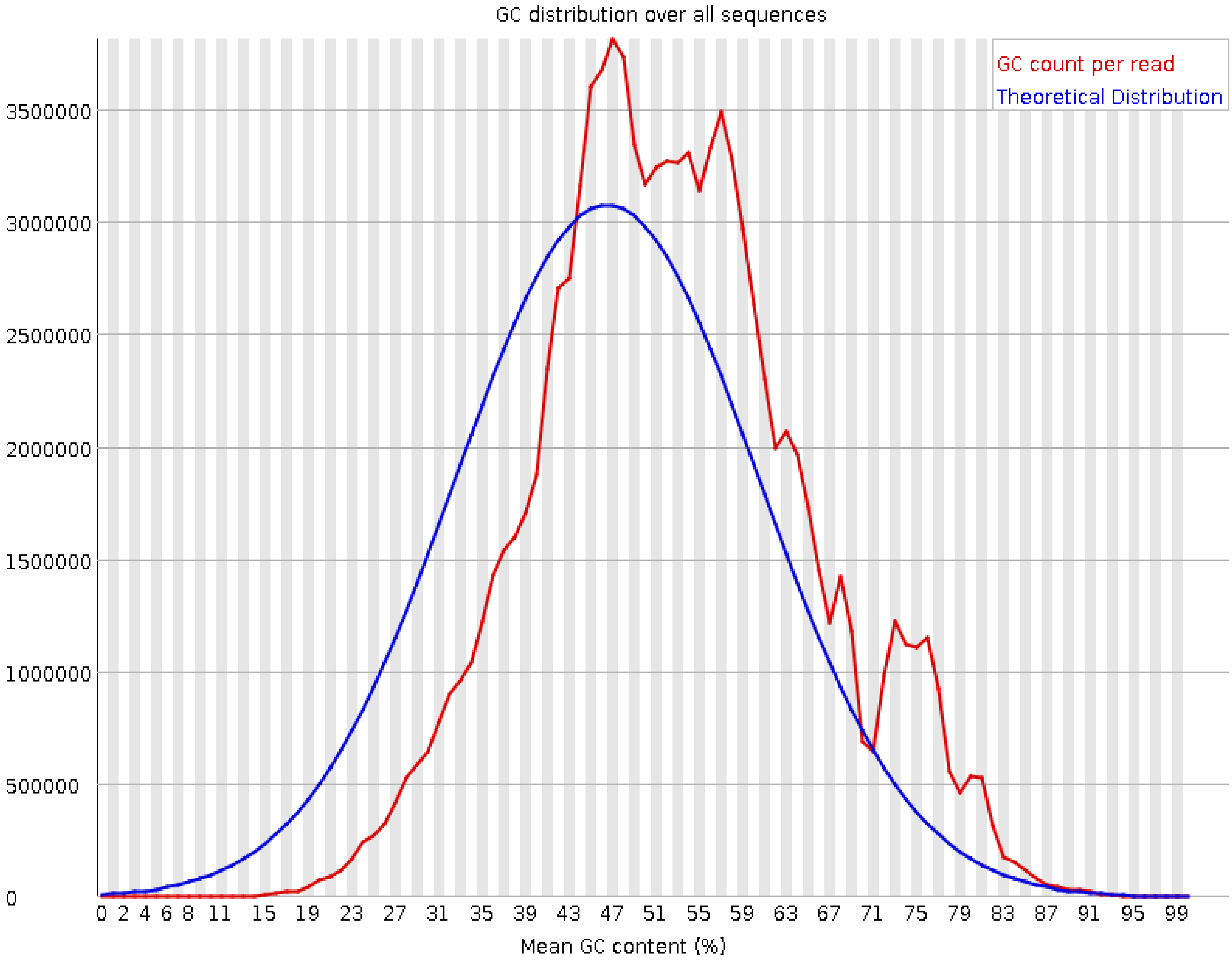


## Per base N content

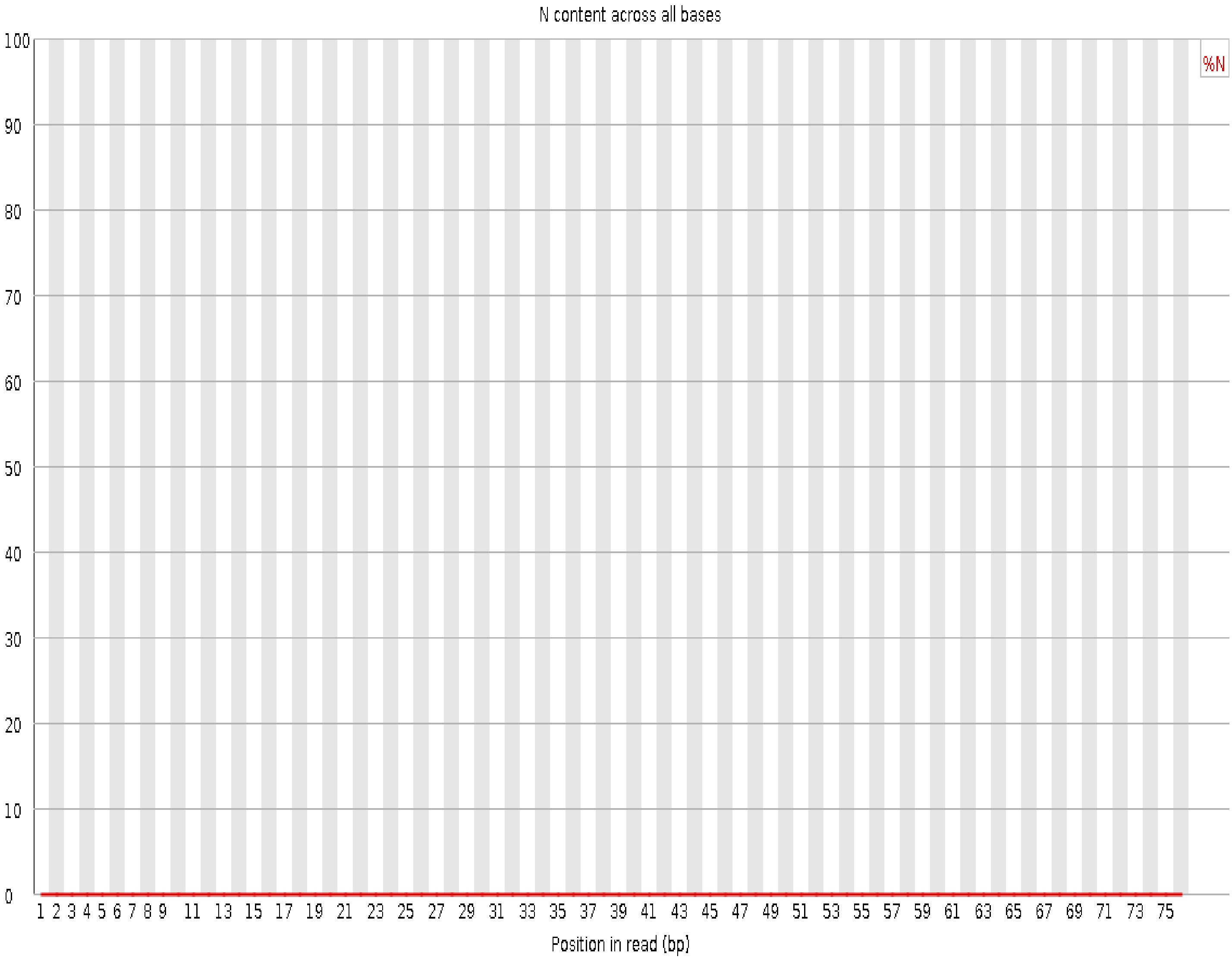


## Sequence Length Distribution

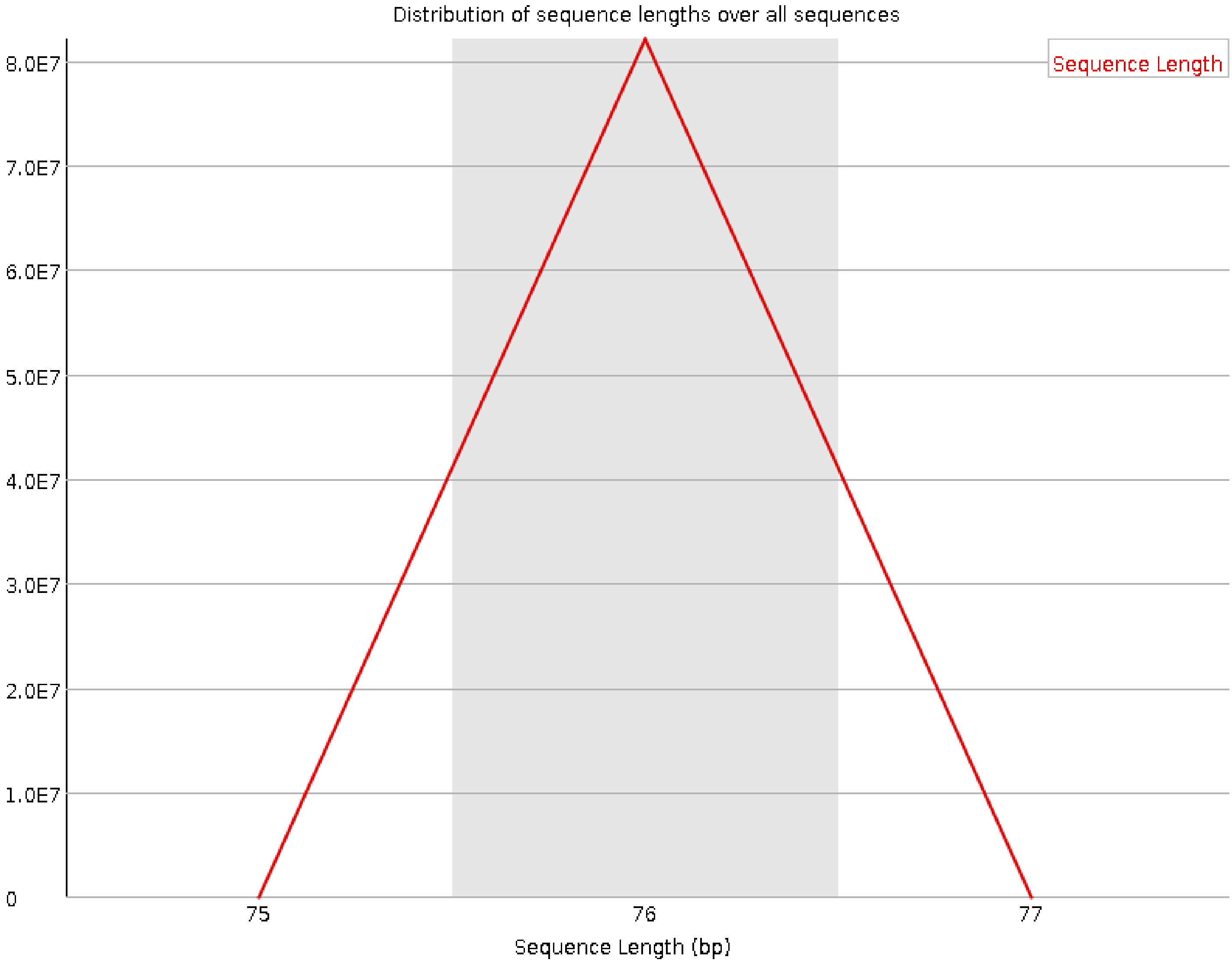


## Sequence Duplication Levels

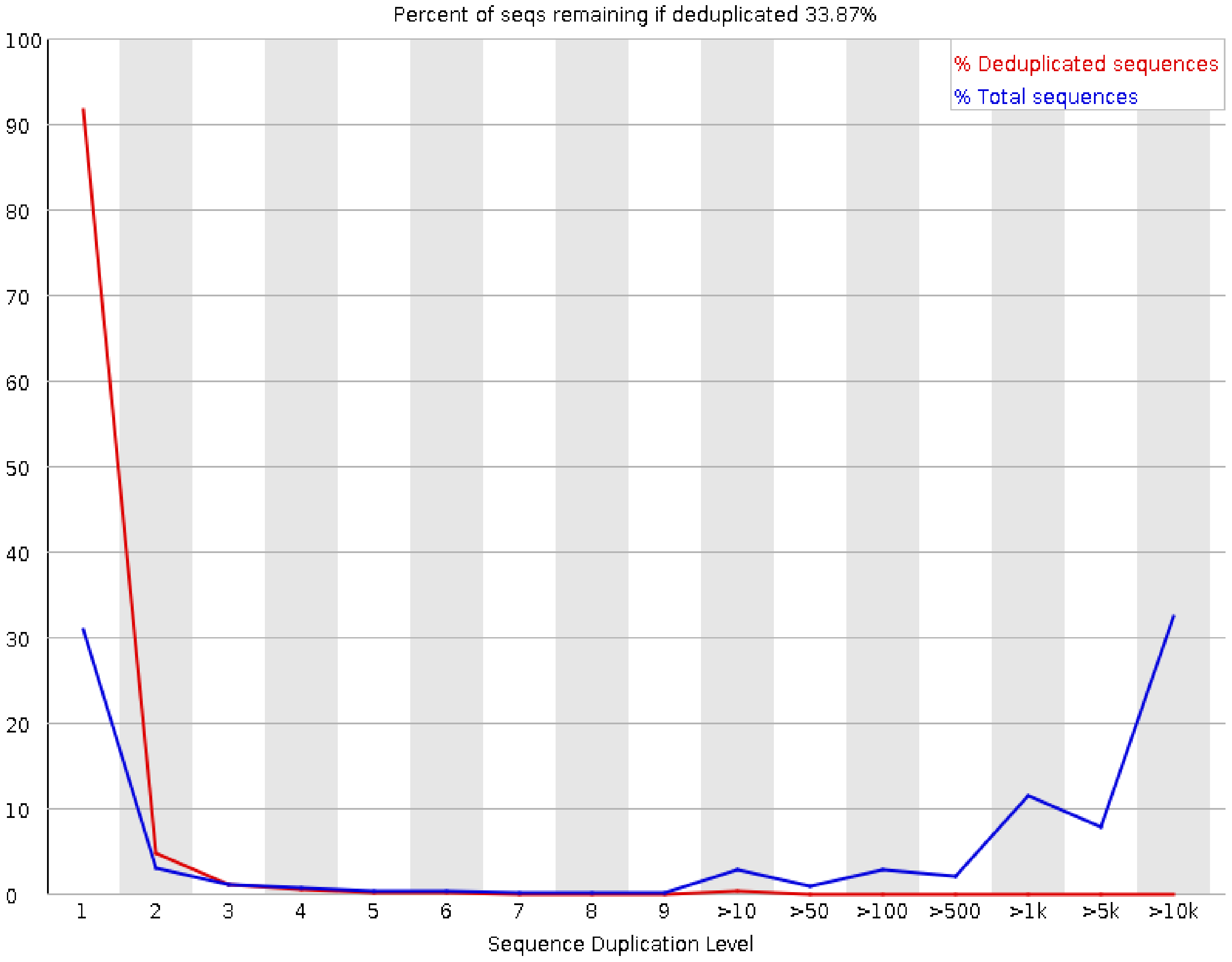


## Overrepresented sequences

| Sequence | Count | Percentage | Possible Source |
|---|---|---|---|
| CTTCCGTACGCCACATGTCCCGCGCCCCGCCGCGGGGCGGGGATTCGGCG | 322536 | 0.3929495492281347 | No Hit |
| CCGGTATTTAGCCTTAGATGGAGTTTACCACCCGCTTTGGGCTGCATTCC | 316314 | 0.3853692106138483 | No Hit |
| CTCCCTTTCGATCGGCCGAGGGCAACGGAGGCCATCGCCCGTCCCTTCGG | 258613 | 0.31507137737968965 | No Hit |
| CCCGTCGGCATGTATTAGCTCTAGAATTACCACAGTTATCCAAGTAGGAG | 213696 | 0.26034844752788977 | No Hit |
| CGCAGTTTTATCCGGTAAAGCGAATGATTAGAGGTCTTGGGGCCGAAACG | 200336 | 0.24407179630852863 | No Hit |
| CTGGATAGTAGGTAGGGACAGTGGGAATCTCGTTCATCCATTCATGCGCG | 187254 | 0.22813383588549843 | No Hit |
| CTCTCTTCAAAGTTCTTTTCAACTTTCCCTTACGGTACTTGTTGACTATC | 186559 | 0.2272871088946709 | No Hit |
| CTCCGACTTTCGTTCTTGATTAATGAAAACATTCTTGGCAAATGCTTTCG | 178515 | 0.21748700542097765 | No Hit |
| CTTCACCGTGCCAGACTAGAGTCAAGCTCAACAGGGTCTTCTTTCCCCGC | 151238 | 0.18425510307737625 | No Hit |
| CTGCCAGTAGCATATGCTTGTCTCAAAGATTAAGCCATGCATGTCTAAGT | 146651 | 0.17866670493791445 | No Hit |
| CGGGTCTTCCGTACGCCACATGTCCCGCGCCCCGCCGCGGGGCGGGGATT | 144310 | 0.17581463603787517 | No Hit |

| Sequence | Count | Percentage | Possible Source |
|---|---|---|---|
| CGCGTAACTAGTTAGCATGCCAGAGTCTCGTTCGTTATCGGAATTAACCA | 139244 | 0.16964266634646172 | No Hit |
| GTCAAAGTGAAGAAATTCAATGAAGCGCGGGTAAACGGCGGGAGTAACTA | 138951 | 0.16928570086687544 | No Hit |
| CCTCACCCGGCCCGGACACGGACAGGATTGACAGATTGATAGCTCTTTCT | 134437 | 0.16378623951925597 | No Hit |
| CTGCTGTCTATATCAACCAACACCTTTTCTGGGGTCTGATGAGCGTCGGC | 134234 | 0.1635389221392013 | No Hit |
| CTTGAACTCTCTCTTCAAAGTTCTTTTCAACTTTCCCTTACGGTACTTGT | 133852 | 0.1630735268723004 | No Hit |
| CTTAGATGGAGTTTACCACCCGCTTTGGGCTGCATTCCCAAGCAACCCGA | 130466 | 0.15894832170547726 | No Hit |
| CTTTAAATGGGTAAGAAGCCCGGCTCGCTGGCGTGGAGCCGGGCGTGGAA | 129505 | 0.1577775236649229 | No Hit |
| CTCTCATGTCTCTTCACCGTGCCAGACTAGAGTCAAGCTCAACAGGGTCT | 129054 | 0.15722806485504778 | No Hit |
| CCCGAAGTTACGGATCCGGCTTGCCGACTTCCCTTACCTACATTGTTCCA | 123798 | 0.1508246158424009 | No Hit |
| CTCGCATTCCACGCCCGGCTCCACGCCAGCGAGCCGGGCTTCTTACCCAT | 121526 | 0.14805661048533586 | No Hit |
| CTCCCACTTATTCTACACCTCTCATGTCTCTTCACCGTGCCAGACTAGAG | 120155 | 0.14638630443580414 | No Hit |
| CAAAGATTAAGCCATGCATGTCTAAGTACGCACGGCCGGTACAGTGAAAC | 118006 | 0.14376815148143232 | No Hit |
| CTCCACTTCGGCCTTCAAAGTTCTCGTTTGAATATTTGCTACTACCACCA | 113681 | 0.13849895114282923 | No Hit |
| CCCGCTTTGGGCTGCATTCCCAAGCAACCCGACTCCGGGAAGACCCGGGC | 112879 | 0.13752186474478076 | No Hit |
| CGCGATGTGATTTCTGCCCAGTGCTCTGAATGTCAAAGTGAAGAAATTCA | 108230 | 0.13185793124786385 | No Hit |
| CTTGTCTCAAAGATTAAGCCATGCATGTCTAAGTACGCACGGCCGGTACA | 105598 | 0.12865133349267233 | No Hit |
| CTGAATTTAAGCATATTAGTCAGCGGAGGAAAAGAAACTAACCAGGATTC | 104748 | 0.127615768108207 | No Hit |
| CTCACCCGGCCCGGACACGGACAGGATTGACAGATTGATAGCTCTTTCTC | 104654 | 0.12750124675980729 | No Hit |
| CTTTTCTTTGTGAAGGGCAGGGCGCCCTGGAATGGGTTCGCCCCGAGAGA | 103023 | 0.12551417953385083 | No Hit |
| CTCGATCAGAAGGACTTGGGCCCCCCACGAGCGGCGCCGGGGAGCGGGTC | 101817 | 0.12404489500012707 | No Hit |
| CGAGAACTTTGAAGGCCGAAGTGGAGAAGGGTTCCATGTGAACAGCAGTT | 99845 | 0.12164238330816747 | No Hit |
| GCCCTCTTGAACTCTCTCTTCAAAGTTCTTTTCAACTTTCCCTTACGGTA | 98319 | 0.11978323886499792 | No Hit |
| CTCCGCCACTCCGGATTCGGGGATCTGAACCCGACTCCCTTTCGATCGGC | 97195 | 0.11841385593306963 | No Hit |
| CACCCGTTTACCTCTTAACGGTTTCACGCCCTCTTGAACTCTCTCTTCAA | 91482 | 0.11145363823724551 | No Hit |
| CTGAATGTCAAAGTGAAGAAATTCAATGAAGCGCGGGTAAACGGCGGGAG | 88463 | 0.10777555365406799 | No Hit |
| CAAACTTTAAATGGGTAAGAAGCCCGGCTCGCTGGCGTGGAGCCGGGCGT | 88163 | 0.10741005998896258 | No Hit |
| CCCATATCCGCAGCAGGTCTCCAAGGTGAACAGCCTCTGGCATGTTGGAA | 87452 | 0.10654384000266275 | No Hit |
| CTCCCGTCCACTCTCGACTGCCGGCGACGGCCGGGTATGGGCCCGACGCT | 87234 | 0.10627824793935281 | No Hit |
| CCACCGTCCTGCTGTCTATATCAACCAACACCTTTTCTGGGGTCTGATGA | 84801 | 0.10331409431534788 | No Hit |
| CTATGACTCTCTTAAGGTAGCCAAATGCCTCGTCATCTAATTAGTGACGC | 84679 | 0.10316546022487168 | No Hit |
| CGCTGATTCCGCCAAGCCCGTTCCCTTGGCTGTGGTTTCGCTGGATAGTA | 83792 | 0.10208481728837668 | No Hit |
| CTCAAACTTTAAATGGGTAAGAAGCCCGGCTCGCTGGCGTGGAGCCGGGC | 83773 | 0.10206166935625334 | No Hit |

## Adapter Content

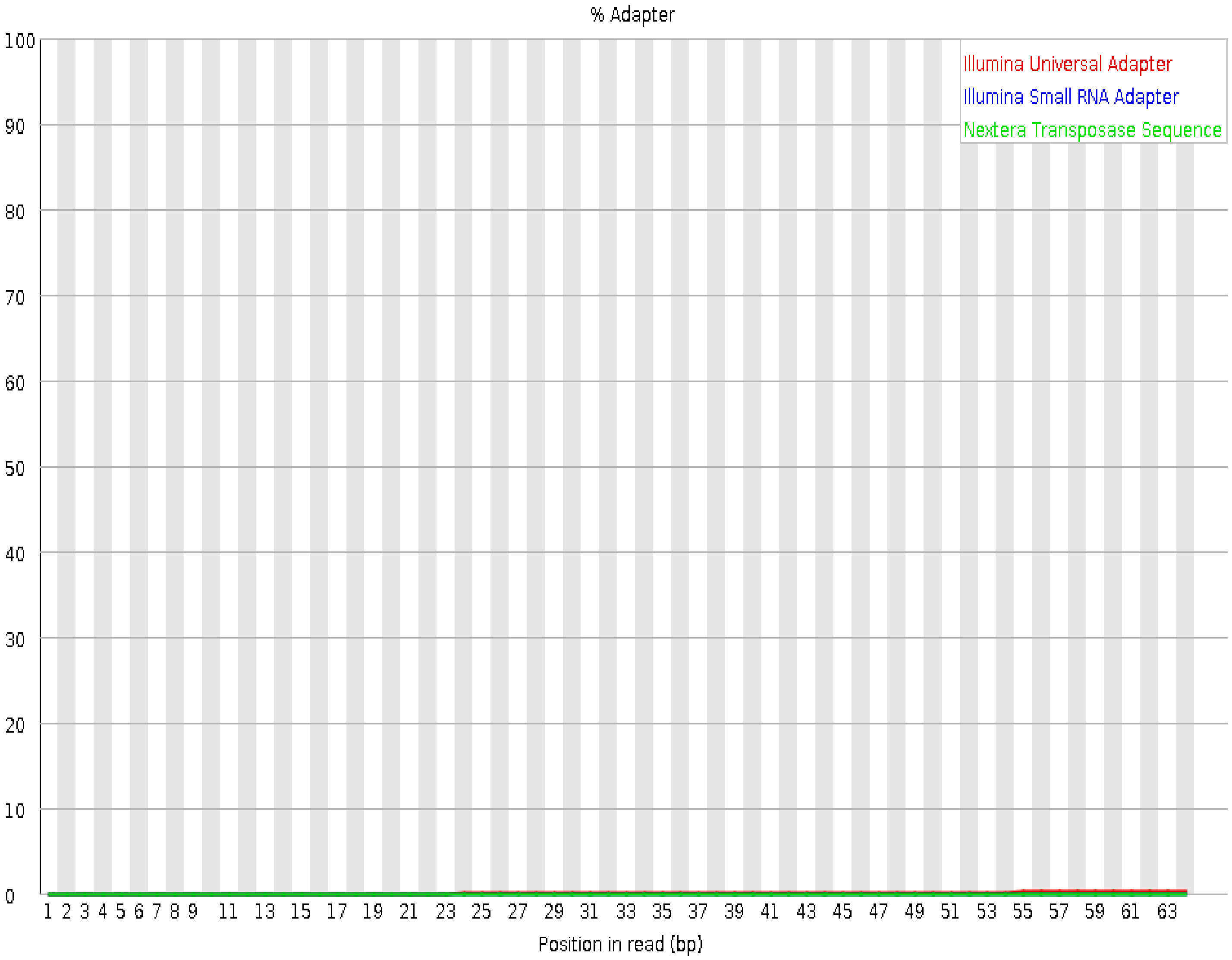


## Kmer Content

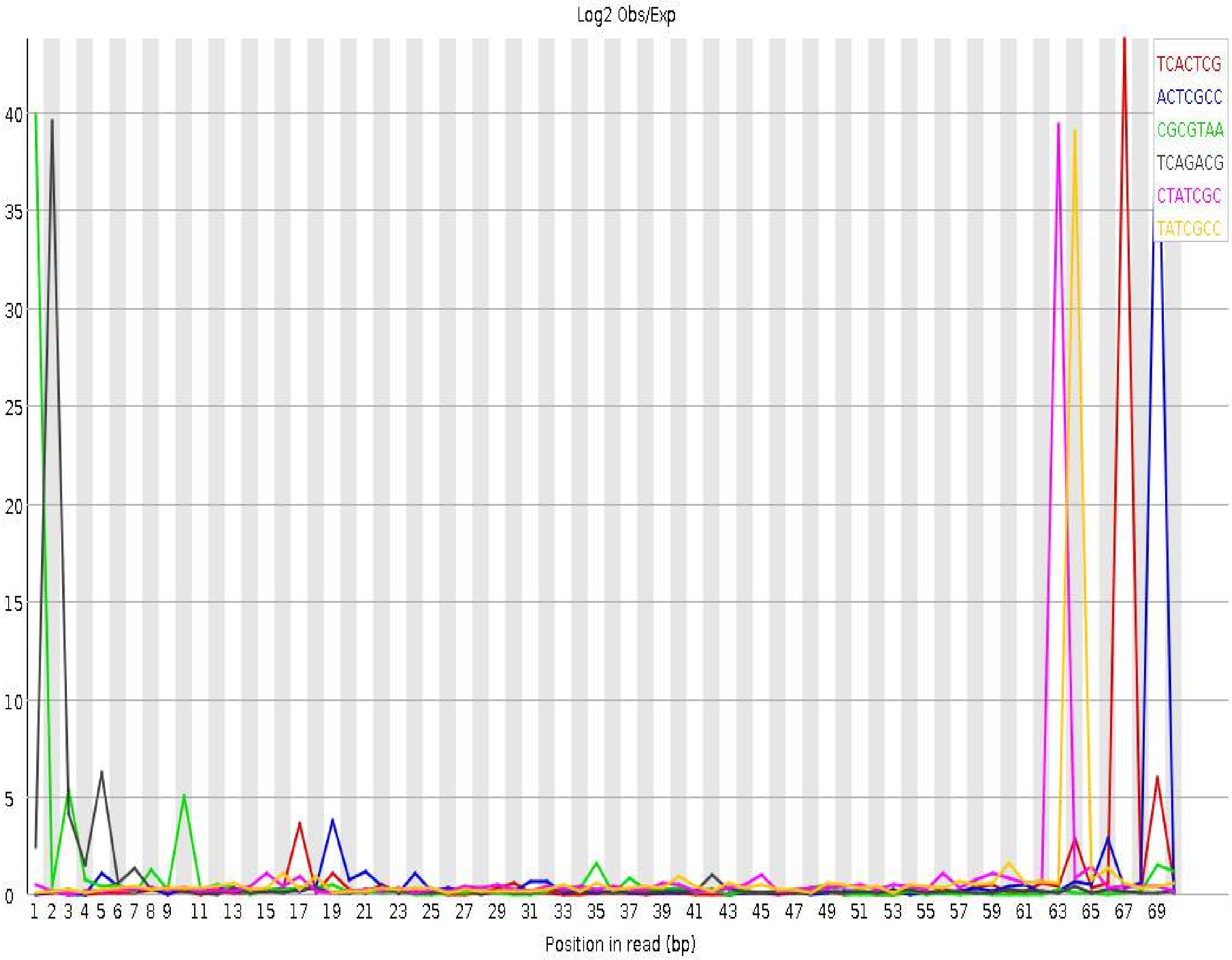


| Sequence | Count | PValue | Obs/Exp Max | Max Obs/Exp Position |
|---|---|---|---|---|
| TCACTCG | 56730 | 0.0 | 43.726475 | 67 |
| ACTCGCC | 56910 | 0.0 | 43.672844 | 69 |
| CGCGTAA | 27610 | 0.0 | 39.938347 | 1 |
| TCAGACG | 24990 | 0.0 | 39.598198 | 2 |
| CTATCGC | 7760 | 0.0 | 39.415253 | 63 |
| TATCGCC | 7840 | 0.0 | 39.059124 | 64 |
| TCGCCTA | 8015 | 0.0 | 38.209587 | 66 |
| GCGTAAC | 30575 | 0.0 | 36.41918 | 2 |
| CGCCTAT | 8420 | 0.0 | 36.332405 | 67 |
| CGGAACG | 58930 | 0.0 | 35.622032 | 48 |
| ACGGCGC | 60435 | 0.0 | 34.550545 | 52 |
| CTCGCTA | 7050 | 0.0 | 34.520695 | 1 |
| CTCGCCG | 80450 | 0.0 | 34.329205 | 70 |
| TTCACTC | 73210 | 0.0 | 34.149006 | 66 |
| CCTTCGG | 62490 | 0.0 | 33.572037 | 44 |

| Sequence | Count | PValue | Obs/Exp Max | Max Obs/Exp Position |
|---|---|---|---|---|
| TTCGCTC | 45950 | 0.0 | 32.[illegible] | 47 |
| ATCGCCT | 9630 | 0.0 | 31.8[illegible] | 65 |
| GCCAGTA | 34355 | 0.0 | 31.503439 | 3 |
| CTCCGAC | 51925 | 0.0 | 31.197008 | 1 |
| TCGCCCA | 67910 | 0.0 | 30.938734 | 59 |

**Produced by FastQC (version 0.11.2)**

# FastQC Report

Mon 4 May 2015
Biochain_Adult_Stomach_GCCAAT_L005_R1.fastq.gz

## Summary


- Basic Statistics
- Per base sequence quality
- Per tile sequence quality
- Per sequence quality scores
- Per base sequence content
- Per sequence GC content
- Per base N content
- Sequence Length Distribution
- Sequence Duplication Levels
- Overrepresented sequences
- Adapter Content
- Kmer Content


## Basic Statistics

| Measure | Value |
|---|---|
| Filename | Biochain_Adult_Stomach_GCCAAT_L005_R1.fastq.gz |
| File type | Conventional base calls |
| Encoding | Sanger / Illumina 1.9 |
| Total Sequences | 83186579 |
| Sequences flagged as poor quality | 0 |
| Sequence length | 76 |
| %GC | 56 |

## Per base sequence quality

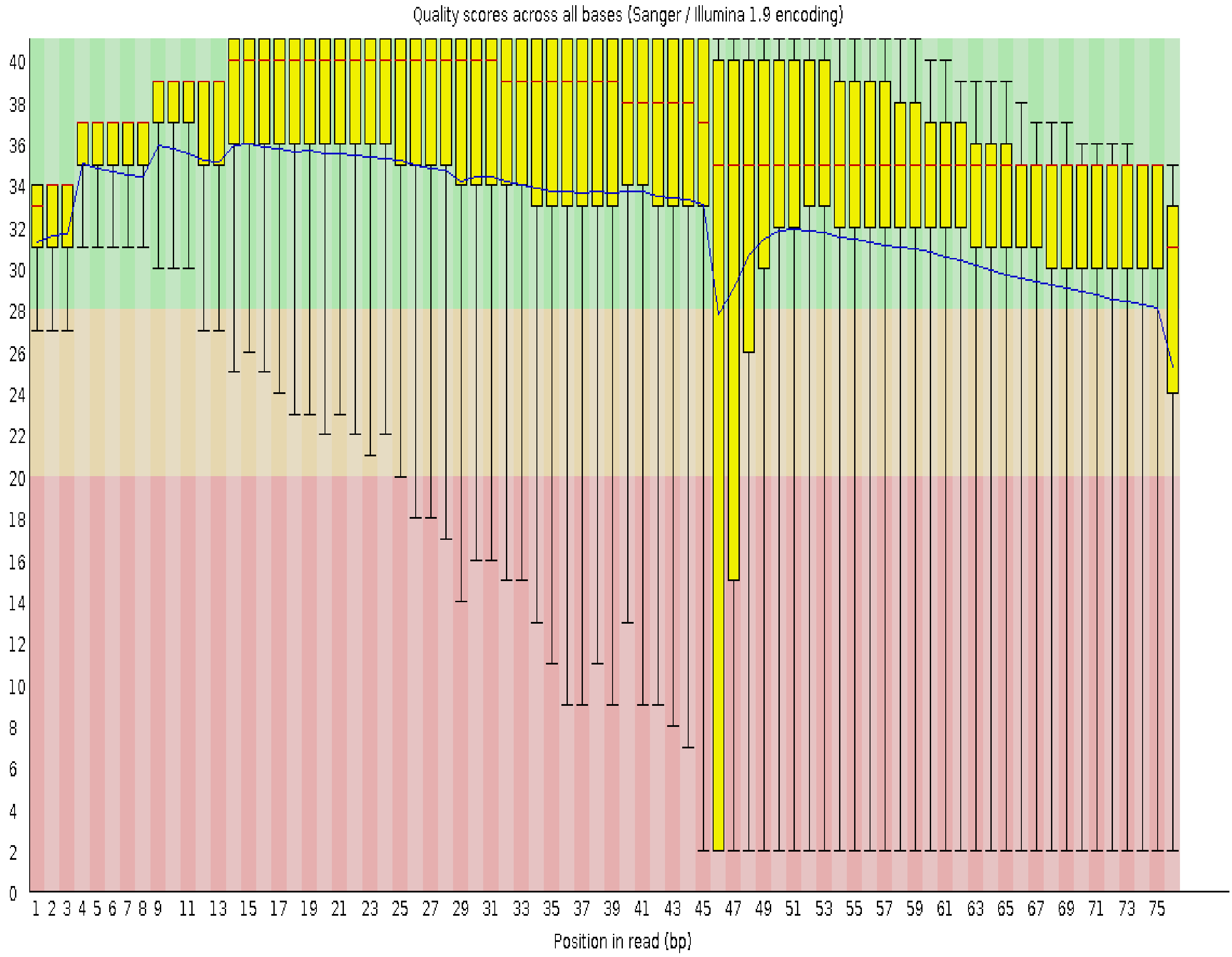


## Per tile sequence quality

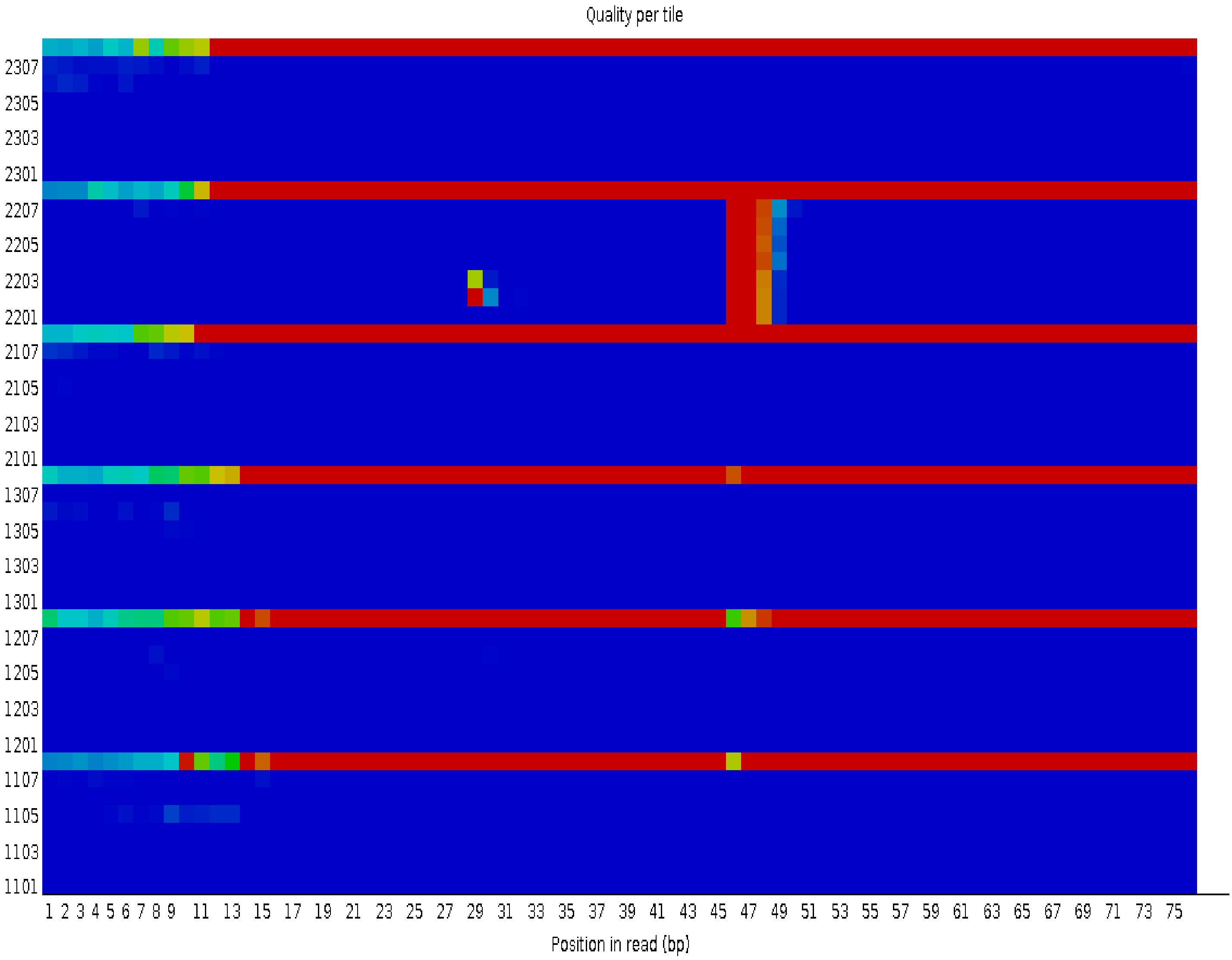


## Per sequence quality scores

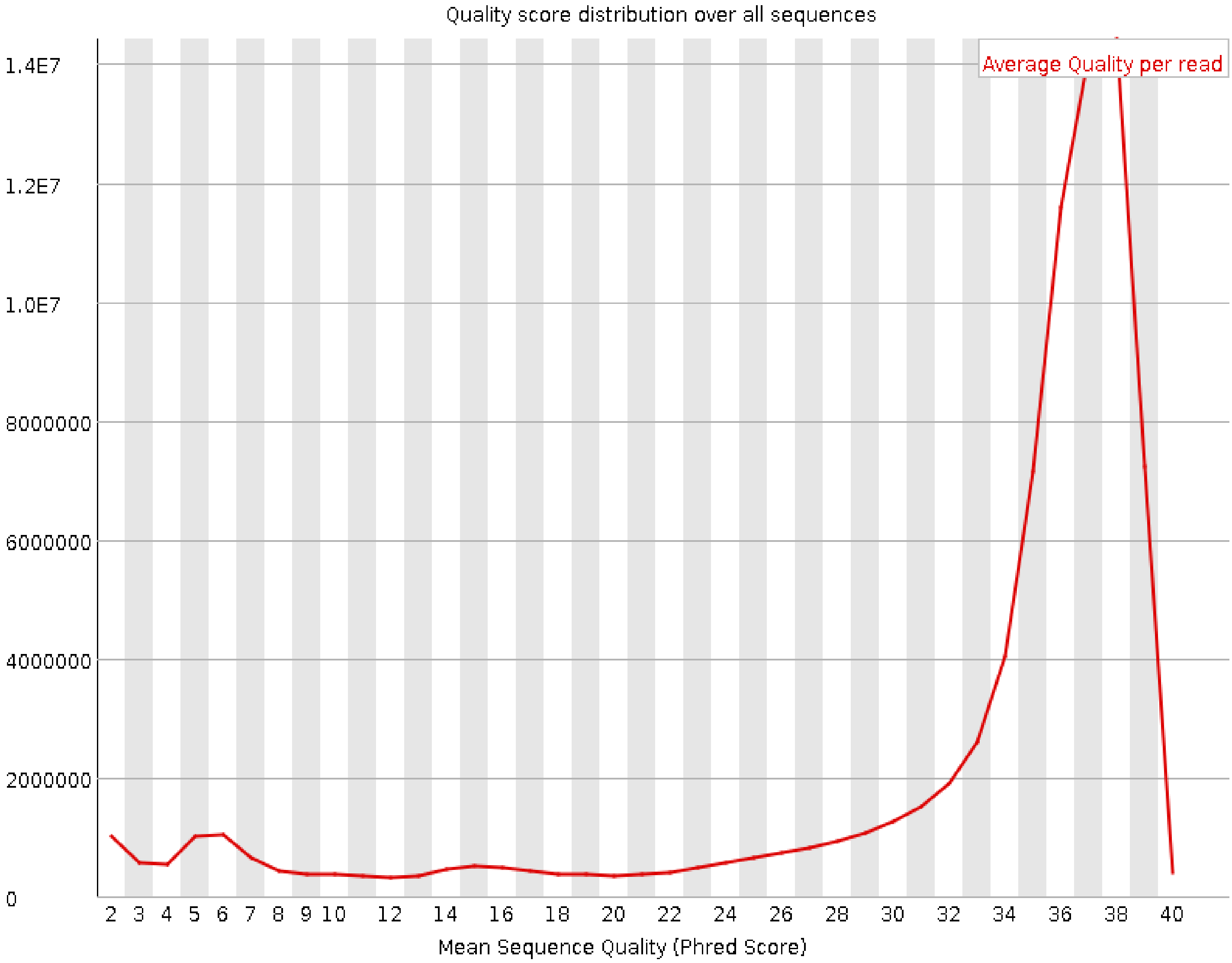


## Per base sequence content

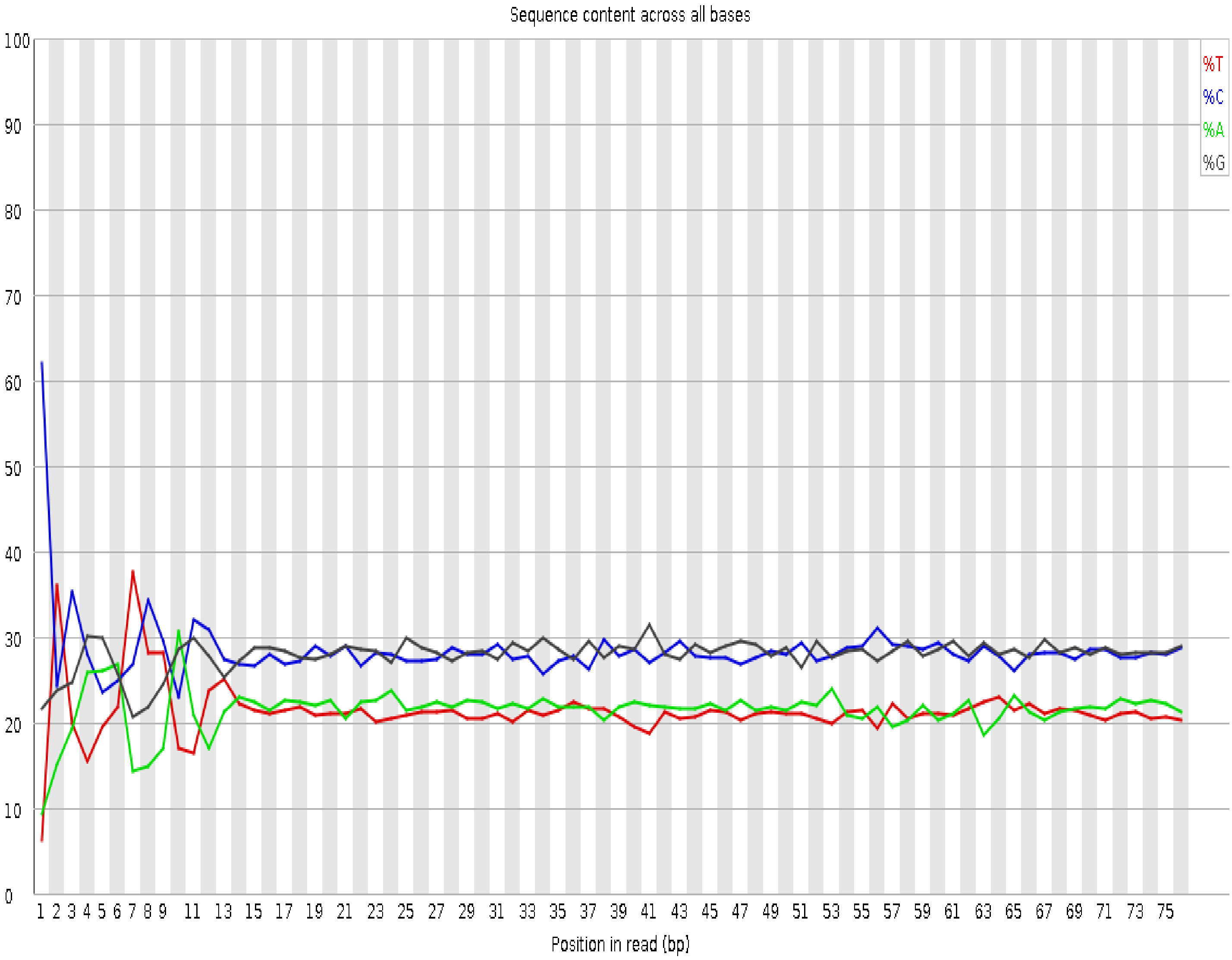


## Per sequence GC content

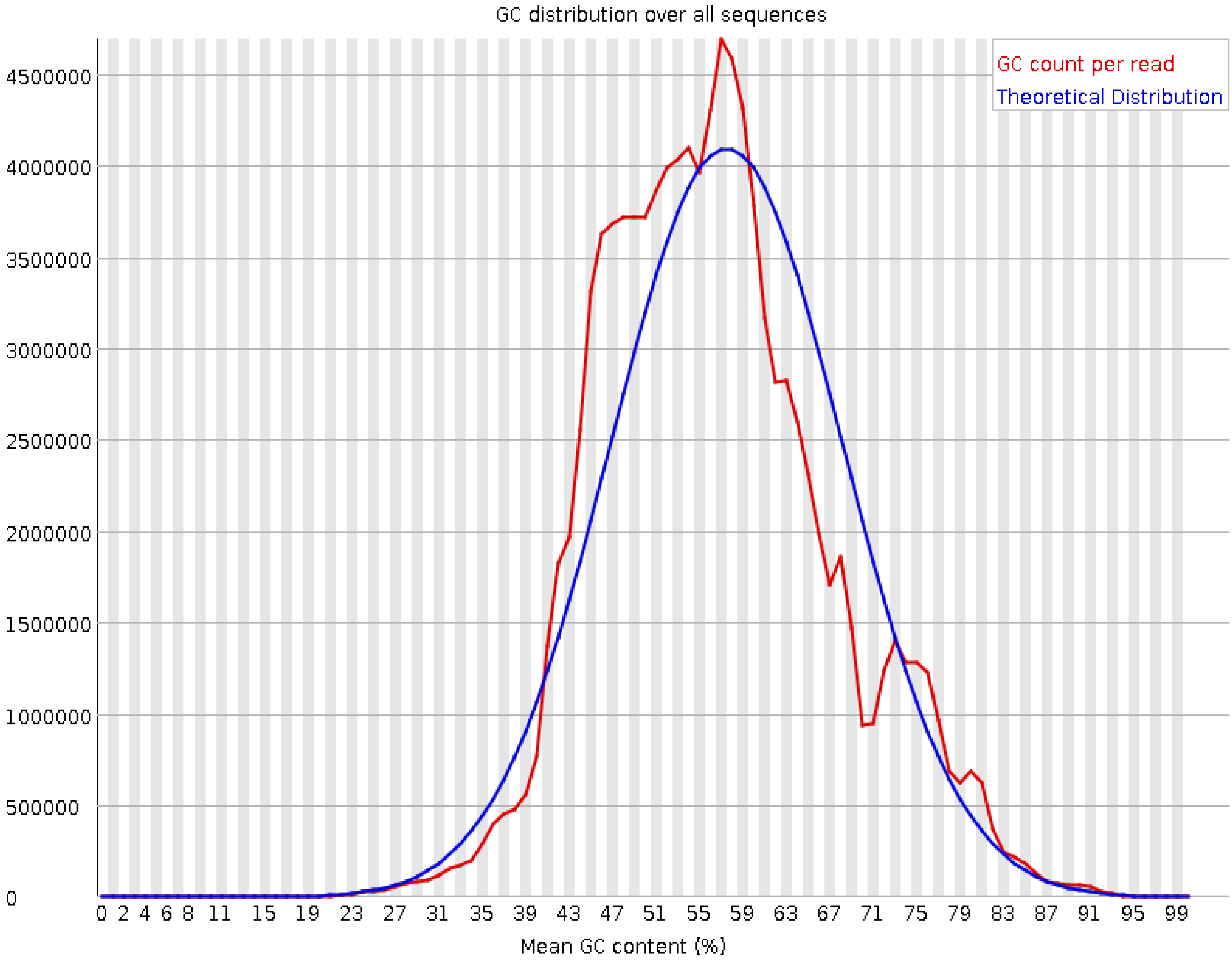


## Per base N content

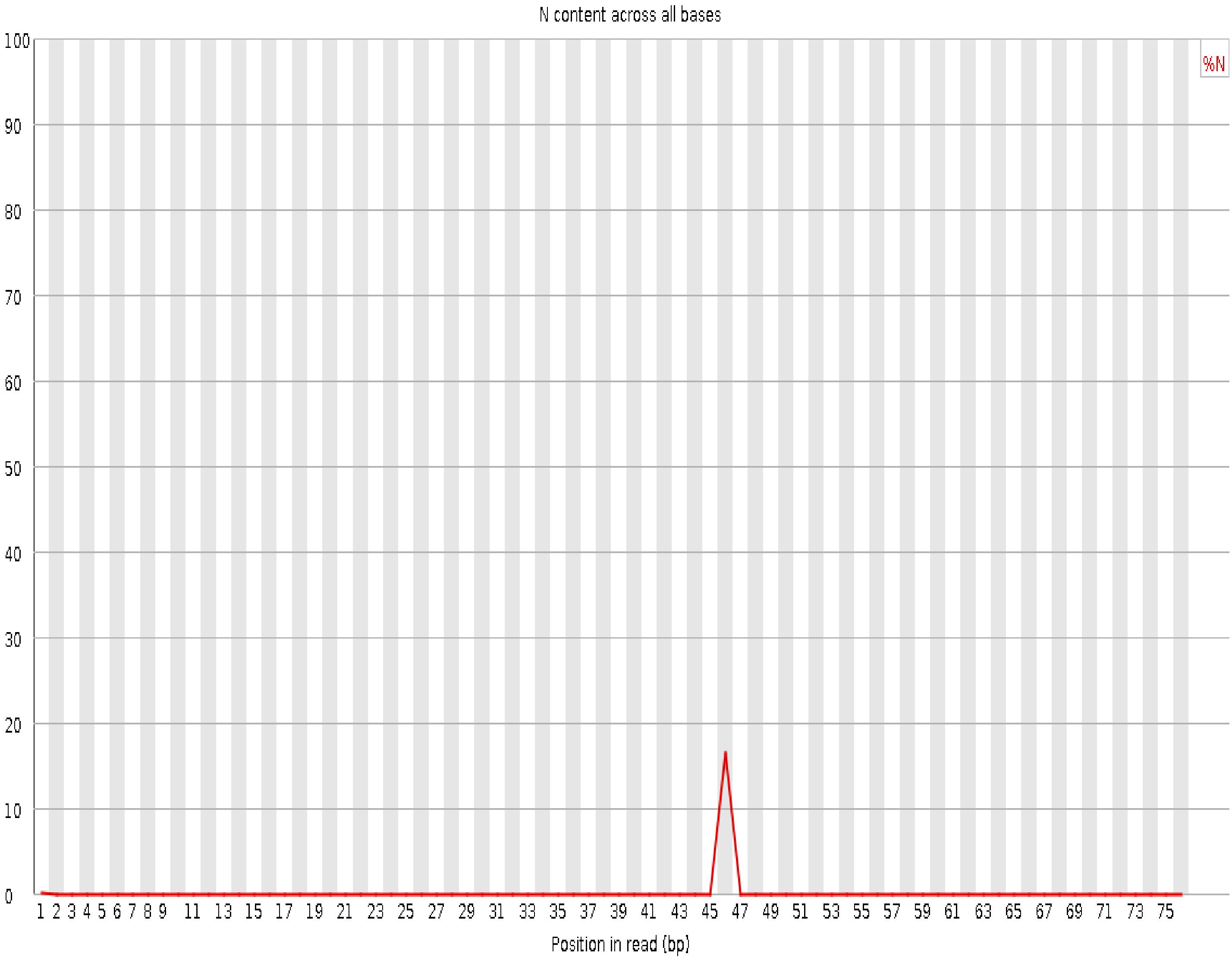


## Sequence Length Distribution

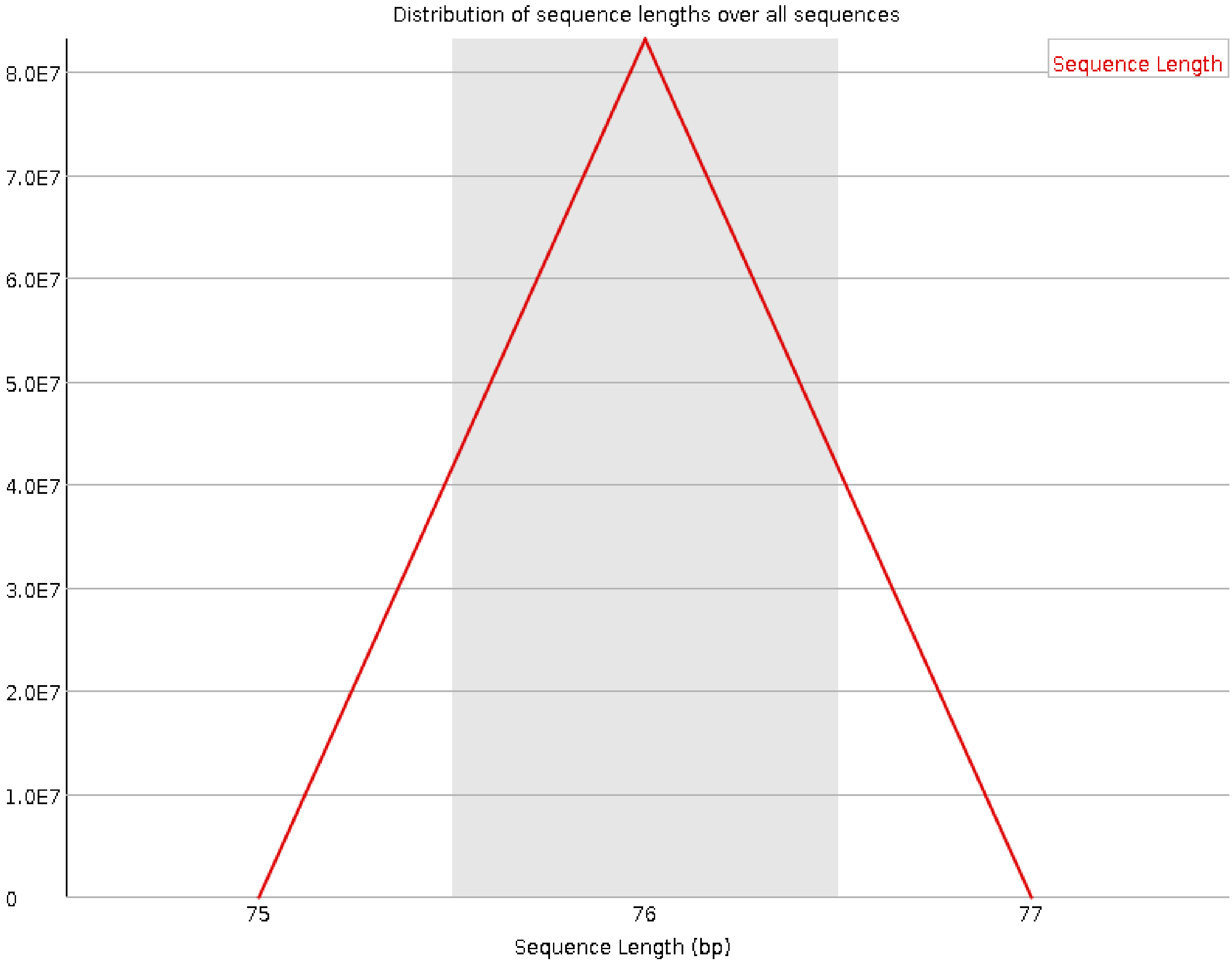


## Sequence Duplication Levels

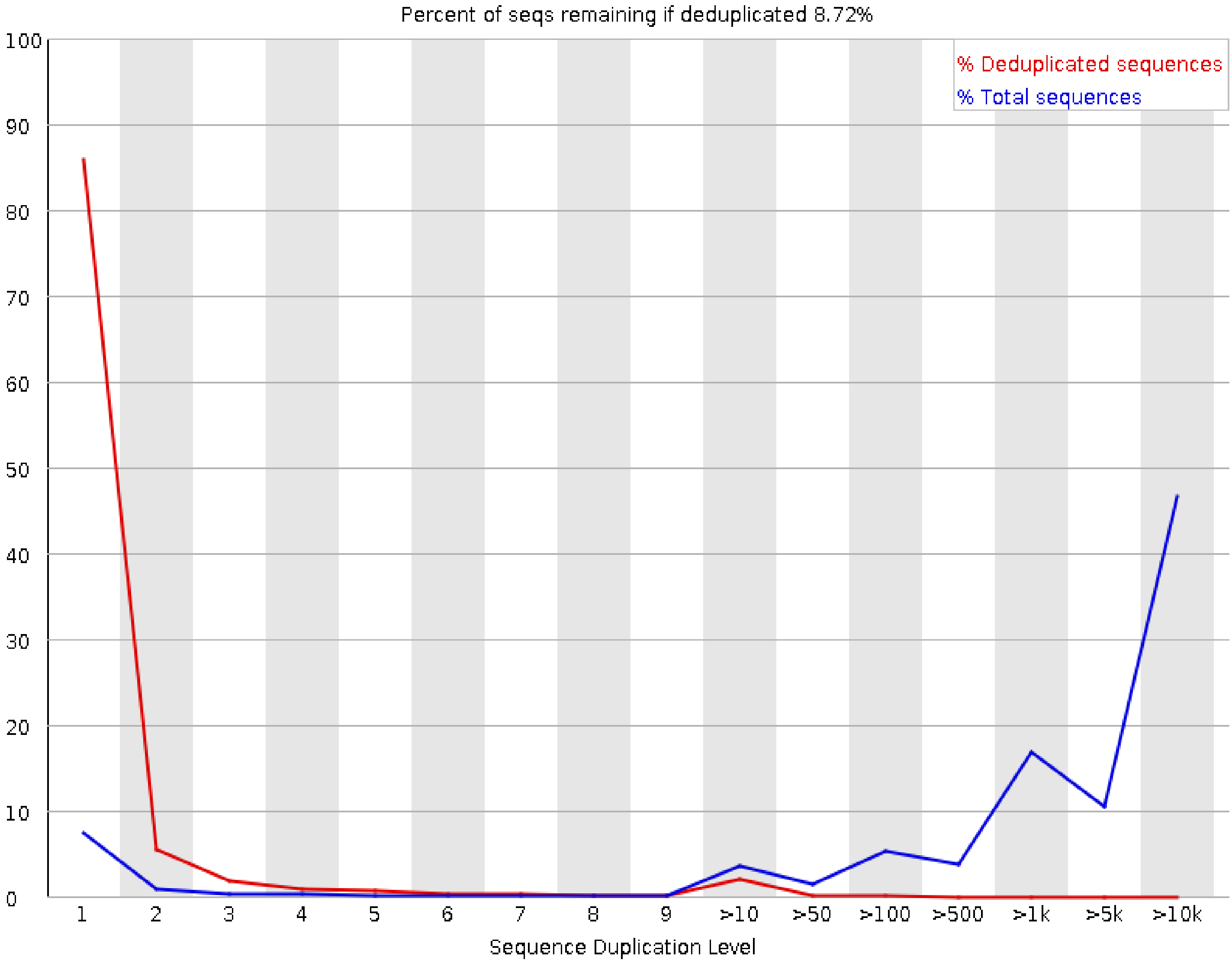


## Overrepresented sequences

| Sequence | Count | Percentage | Possible Source |
|---|---|---|---|
| CTTCCGTACGCCACATGTCCCGCGCCCCGCCGCGGGGCGGGGATTCGGCG | 301467 | 0.36 39860 784 3036 | No Hit |
| CTGGATAGTAGGTAGGGACAGTGGGAATCTCGTTCATCCATTCATGCGCG | 177 | 0. 66595889 390953 | No Hit |
| CCGGTATTTAGCCTTAGATGGAGTTTACCACCCGCTTTGGGCTGCATTCC | 1307 | 0. 660369048 331 9 | No Hit |
| CCTCACCCGGCCCGGACACGGACAGGATTGACAGATTGATAGCTCTTTCT | 10 15 | 0. 5 7030 31643496 | No Hit |
| CTTCACCGTGCCAGACTAGAGTCAAGCTCAACAGGGTCTTCTTTCCCCGC | 1988 | 0. 39007304 91 3668 | No Hit |
| CGCGTAACTAGTTAGCATGCCAGAGTCTCGTTCGTTATCGGAATTAACCA | 197937 | 0. 37943430754617 7 | No Hit |
| CGCAGTTTTATCCGGTAAAGCGAATGATTAGAGGTCTTGGGGCCGAAACG | 197840 | 0. 378 68 54065358 | o Hit |
| CTCTCTTCAAAGTTCTTTTCAACTTTCCCTTACGGTACTTGTTGACTATC | 184754 | 0. 0959 684549 | No Hit |
| CTCCCTTTCGATCGGCCGAGGGCAACGGAGGCCATCGCCCGTCCCTTCGG | 171105 | 0. 05688 766470538 | No Hit |
| CTCACCCGGCCCGGACACGGACAGGATTGACAGATTGATAGCTCTTTCTC | 1679 5 | 0. 0186549563481868 | No Hit |
| CTCTCATGTCTCTTCACCGTGCCAGACTAGAGTCAAGCTCAACAGGGTCT | 16 878 | 0.19579841118361171 | No Hit |

| Sequence | Count | Percentage | Possible Source |
|---|---|---|---|
| CCCGTCGGCATGTATTAGCTCTAGAATTACCACAGTTATCCAAGTAGGAG | 161564 | 0.194 188 945805477 | No Hit |
| CAGAAACCTCCCGTGGAGCAGAAGGCAAAAGCTCGCTTGATCTTGATTT | 154949 | 0.186 668 556569613 | N[illegible] |
| CTGCCAGTAGCATATGCTTGTCTCAAAGATTAAGCCATGCATGTCTAAGT | 151171 | 0.1817 5 757547 | o Hit |
| GTCAAAGTGAAGAAATTCAATGAAGCGCGGGTAAACGGCGGGAGTAACTA | 149996 | 0.18031 740111599 | No Hit |
| CTTGAACTCTCTCTTCAAAGTTCTTTTCAACTTTCCCTTACGGTACTTGT | 149703 | 0.1799605198333736 | No Hit |
| CCCGAAGTTACGGATCCGGCTTGCCGACTTCCCTTACCTACATTGTTCCA | 145390 | 0.174775789 53 1594 | No Hit |
| CCCATATCCGCAGCAGGTCTCCAAGGTGAACAGCCTCTGGCATGTTGGAA | 141195 | 0.16973 9084779409 | No Hit |
| CTTTAAATGGGTAAGAAGCCCGGCTCGCTGGCGTGGAGCCGGGCGTGGAA | 134039 | 0.161130559 937053 | No Hit |
| CTCGCATTCCACGCCCGGCTCCACGCCAGCGAGCCGGGCTTCTTACCCAT | 1309 6 | 0.15738836910 18415 | No Hit |
| CTCATGTCTCTTCACCGTGCCAGACTAGAGTCAAGCTCAACAGGGTCTTC | 1 8948 | 0.15501058169 4915 | No Hit |
| CTTGTCTCAAAGATTAAGCCATGCATGTCTAAGTACGCACGGCCGGTACA | 1 8073 | 0.1539587 93 8 009 | No Hit |
| CGAGATTCCCACTGTCCCTACCTACTATCCAGCGAAACCACAGCCAAGGG | 1 719 | 0.15 899664 595436 | No Hit |
| CGCGATGTGATTTCTGCCCAGTGCTCTGAATGTCAAAGTGAAGAAATTCA | 1 5896 | 0.1513417 0639816 | o Hit |
| CGGGTCTTCCGTACGCCACATGTCCCGCGCCCCGCCGCGGGGCGGGGATT | 1 101 | 0.1491839 06418141 | No Hit |
| CTTAGAGCCAATCCTTATCCCGAAGTTACGGATCCGGCTTGCCGACTTCC | 1 0 9 | 0.144553365994 9097 | No Hit |
| CGAAGGCCCGCGGCGGGTGTTGACGCGATGTGATTTCTGCCCAGTGCTCT | 1154 0 | 0.13874834304701966 | No Hit |
| CTCCGACTTTCGTTCTTGATTAATGAAAACATTCTTGGCAAATGCTTTCG | 115099 | 0.13836 6349305936 | No Hit |
| CTGCTGTCTATATCAACCAACACCTTTTCTGGGGTCTGATGAGCGTCGGC | 114900 | 0.1381 3 1194383 | No Hit |
| CCACTCTCGACTGCCGGCGACGGCCGGGTATGGGCCCGACGCTCCAGCGC | 11 63 | 0.134953 5970791514 | No Hit |
| GTAAATCTCGCGCCGGGCCGTACCCATATCCGCAGCAGGTCTCCAAGGTG | 111685 | 0.134 5843608738858 | No Hit |
| CTGAATTTAAGCATATTAGTCAGCGGAGGAGAAGAAACTAACCAGGATTC | 108441 | 0.1303587685701079 | o Hit |
| CAAAGATTAAGCCATGCATGTCTAAGTACGCACGGCCGGTACAGTGAAAC | 107756 | 0.1 95353184315946 | No Hit |
| CGCGTCACTAATTAGATGACGAGGCATTTGGCTACCTTAAGAGAGTCATA | 106950 | 0.1 856641 137 708 | No Hit |
| CTGAATTTAAGCATATTAGTCAGCGGAGGAAAAGAAACTAACCAGGATTC | 105467 | 0.1 678367 640 705 | No Hit |
| CCCACTTATTCTACACCTCTCATGTCTCTTCACCGTGCCAGACTAGAGTC | 104098 | 0.1 5137974480 3437 | No Hit |
| CTCCGCCACTCCGGATTCGGGGATCTGAACCCGACTCCCTTTCGATCGGC | 10 187 | 0.1 8407 891133077 | No Hit |
| CCGACATCGAAGGATCAAAAAGCGACGTCGCTATGAACGCTTGGCCGCCA | 98350 | 0.118 8 0601866557 | No Hit |
| CTTAGATGGAGTTTACCACCCGCTTTGGGCTGCATTCCCAAGCAACCCGA | 97903 | 0.1176908597 38985 | No Hit |
| CTACTATCCAGCGAAACCACAGCCAAGGGAACGGCTTGGCGGAATCAGC | 97154 | 0.116790474097991 | o Hit |
| CCTGTGGTAACTTTTCTGACACCTCCTGCTTAAAACCCAAAAGGTCAGAA | 97099 | 0.1167 35766351 04 | No Hit |
| CTCCCGTCCACTCTCGACTGCCGGCGACGGCCGGGTATGGGCCCGACGCT | 96938 | 0.1165308168 803665 | No Hit |
| CTCCCACTTATTCTACACCTCTCATGTCTCTTCACCGTGCCAGACTAGAG | 94999 | 0.11419991198339818 | No Hit |
| CTCCACTTCGGCCTTCAAAGTTCTCGTTTGAATATTTGCTACTACCACCA | 93440 | 0.11 3 5811595161 9 | No Hit |
| GTCGGCATGTATTAGCTCTAGAATTACCACAGTTATCCAAGTAGGAGAGG | 9 673 | 0.1114037878634 6 | No Hit |
| CGAGAACTTTGAAGGCCGAAGTGGAGAAGGGTTCCATGTGAACAGCAGTT | 86409 | 0.1038737 703474199 | No Hit |
| CTCGATCAGAAGGACTTGGGCCCCCCACGAGCGGCGCCGGGGAGCGGGTC | 86130 | 0.103538336394384 | No Hit |
| CAAACTTTAAATGGGTAAGAAGCCCGGCTCGCTGGCGTGGAGCCGGGCGT | 85587 | 0.10 8855868685 6 | No Hit |
| CACGAGCGCACGTGTTAGGACCCGAAAGATGGTGAACTATGCCTGGGCAG | 85059 | 0.10 508690975 593 | No Hit |
| CGACGACCCATTCGAACGTCTGCCCTATCAACTTTCGATGGTAGTCGCCG | 84350 | 0.101398568151 3988 | No Hit |

## Adapter Content

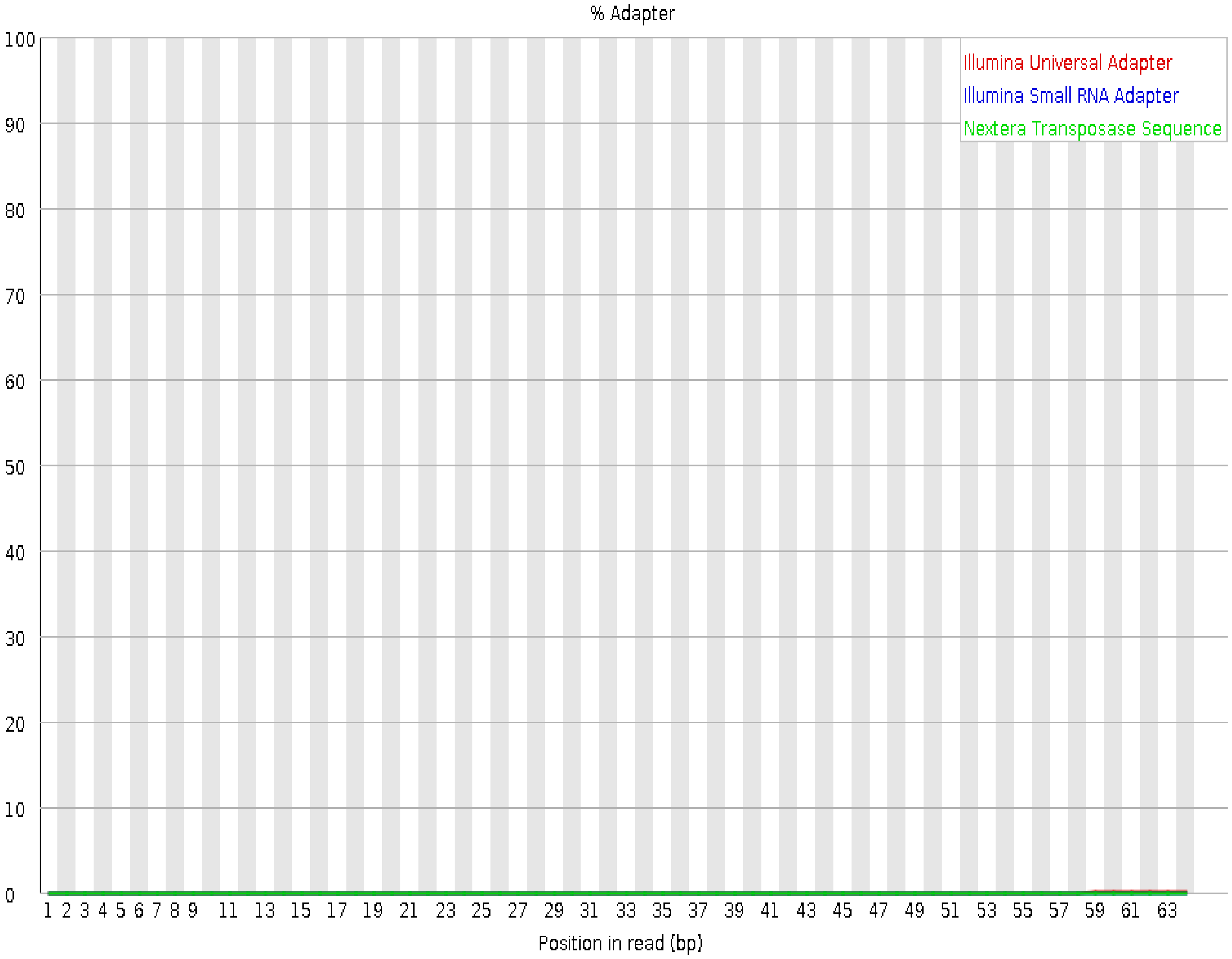


## Kmer Content

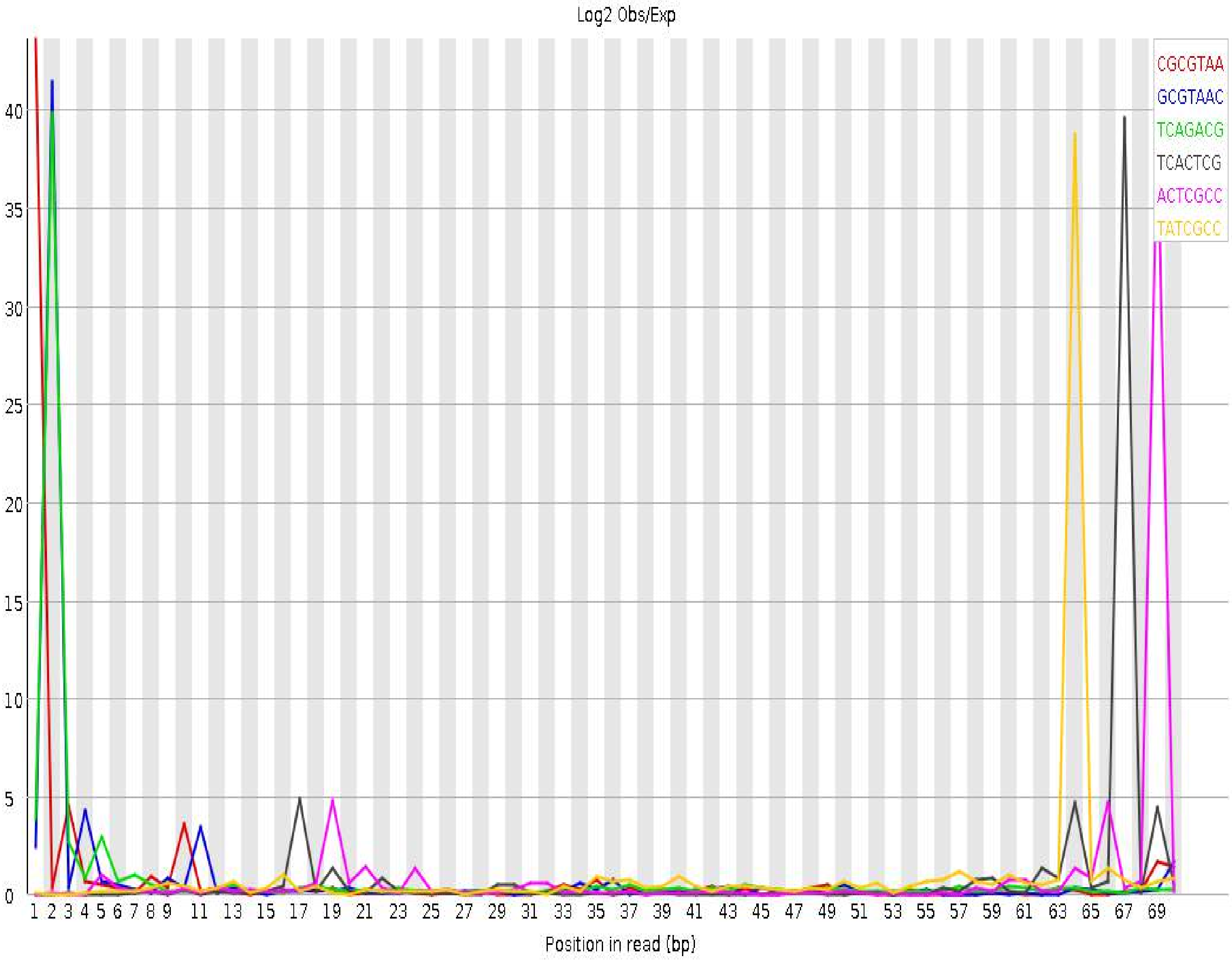


| Sequence | Count | PValue | Obs/Exp Max | Max Obs/Exp Position |
|---|---|---|---|---|
| CGCGTAA | 46535 | 0.0 | 43.590553 | 1 |
| GCGTAAC | 49535 | 0.0 | 41.4531 | |
| TCAGACG | 18   0 | 0.0 | 39.8395 3 | |
| TCACTCG | 7 3 0 | 0.0 | 39.641438 | 67 |
| ACTCGCC | 73675 | 0.0 | 39.1    04 | 69 |
| TATCGCC | 7145 | 0.0 | 38.768456 | 64 |
| TGTGTCG | 7085 | 0.0 | 38.4 618 | 3 |
| CGCCTAT | 7 90 | 0.0 | 38.1884 3 | 67 |
| TCGCCTA | 73 5 | 0.0 | 38.005768 | 66 |
| TTCACTC | 76560 | 0.0 | 37. 6607 | 66 |
| TGCCAGT | 0370 | 0.0 | 36.4045 | |
| CTATCGC | 7690 | 0.0 | 36. 08035 | 63 |
| GTGTCGA | 76 0 | 0.0 | 36.134796 | 4 |
| TGTCGAG | 7445 | 0.0 | 36.05866 | 5 |
| CCAGTAG | 40540 | 0.0 | 35.793774 | 4 |

| Sequence | Count | PValue | Obs/Exp Max | Max Obs/Exp Position |
|---|---|---|---|---|
| CTCGCTA | 8470 | 0.0 | 34.[illegible] | 1 |
| GTTAGCA | 54105 | 0.0 | 34.[illegible] | 11 |
| TAGTTAG | 54880 | 0.0 | 34.16435 | 9 |
| ACAATCC | 19030 | 0.0 | 34.14710 | 3 |
| AGTTAGC | 54350 | 0.0 | 34.060406 | 10 |

**Produced by FastQC (version 0.11.2)**

# FastQC Report

Mon 4 May 2015
Biochain_Adult_Stomach_GCCAAT_L005_R2.fastq.gz

## Summary


- Basic Statistics
- Per base sequence quality
- Per tile sequence quality
- Per sequence quality scores
- Per base sequence content
- Per sequence GC content
- Per base N content
- Sequence Length Distribution
- Sequence Duplication Levels
- Overrepresented sequences
- Adapter Content
- Kmer Content


## Basic Statistics

| Measure | Value |
| --- | --- |
| Filename | Biochain_Adult_Stomach_GCCAAT_L005_R2.fastq.gz |
| File type | Conventional base calls |
| Encoding | Sanger / Illumina 1.9 |
| Total Sequences | 83186579 |
| Sequences flagged as poor quality | 0 |
| Sequence length | 76 |
| %GC | 56 |

## Per base sequence quality

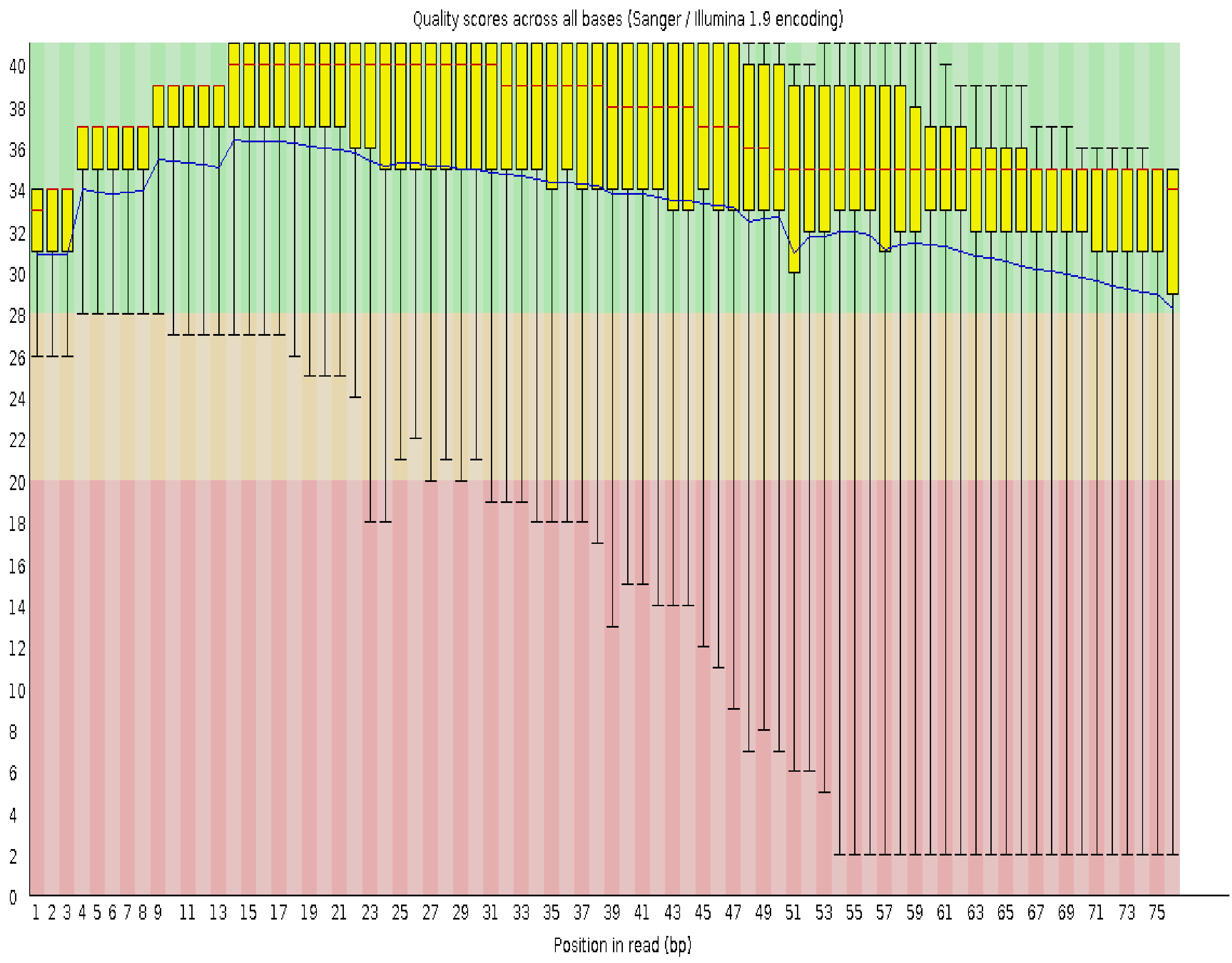


## Per tile sequence quality

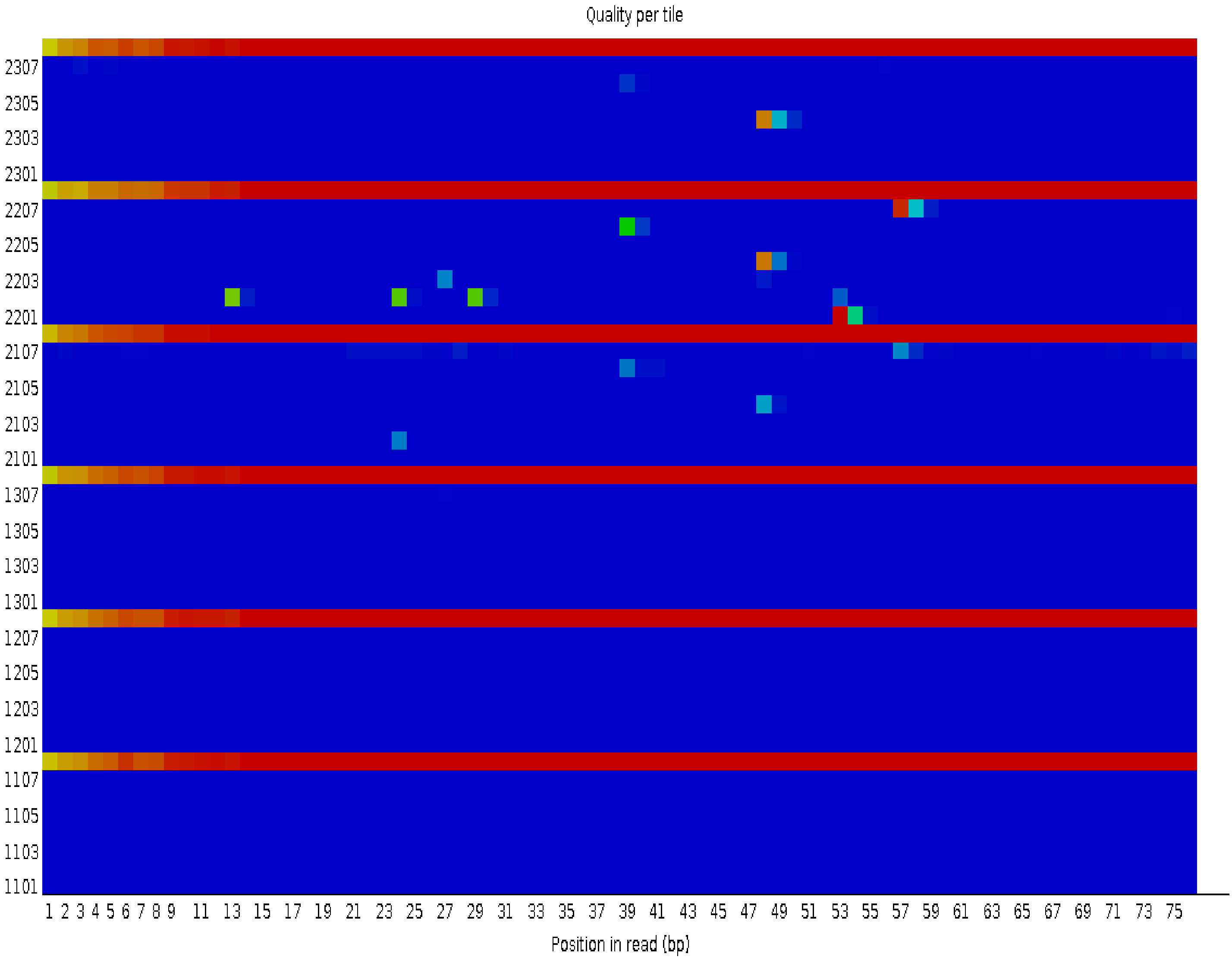


# Per sequence quality scores

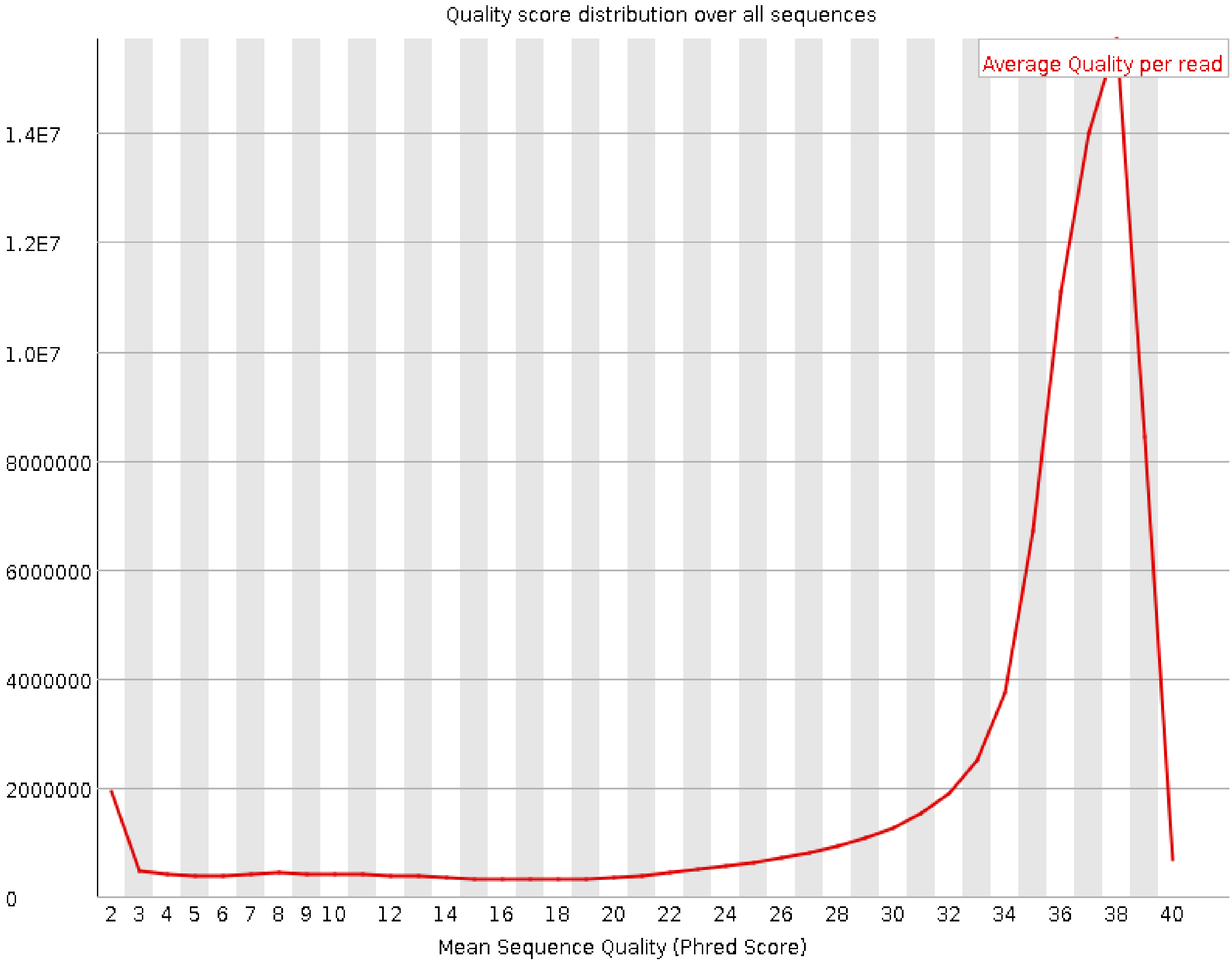


## Per base sequence content

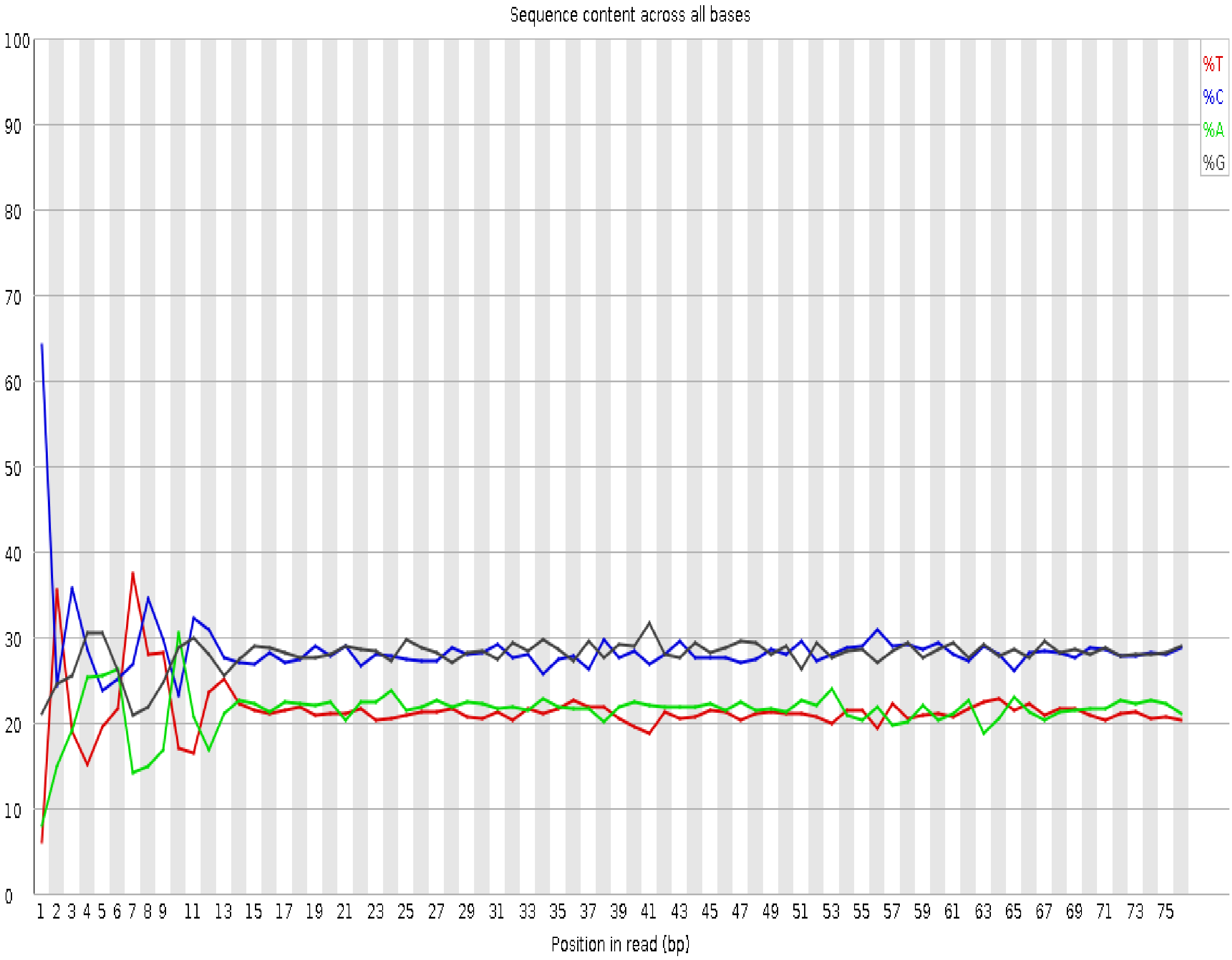


## Per sequence GC content

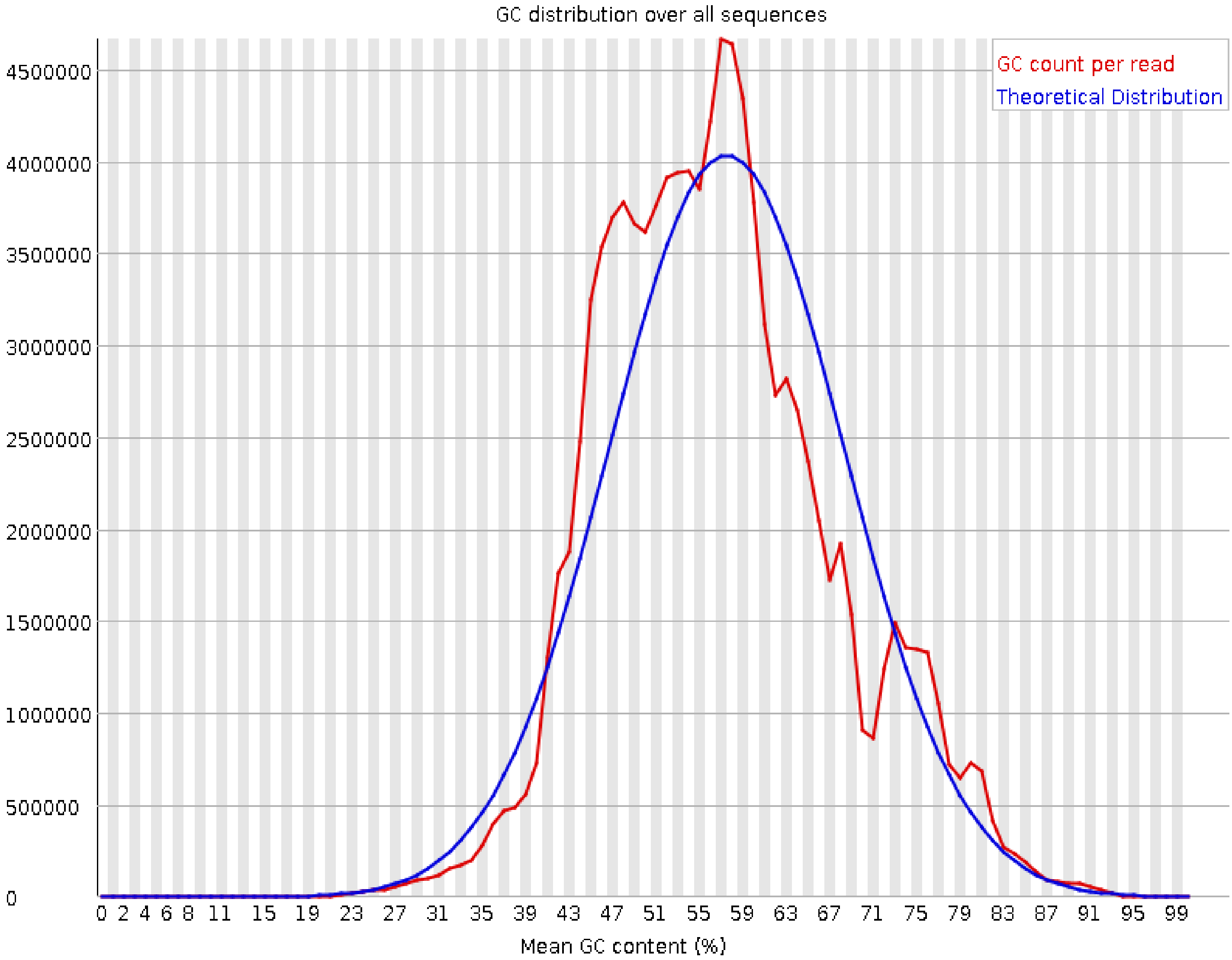


## Per base N content

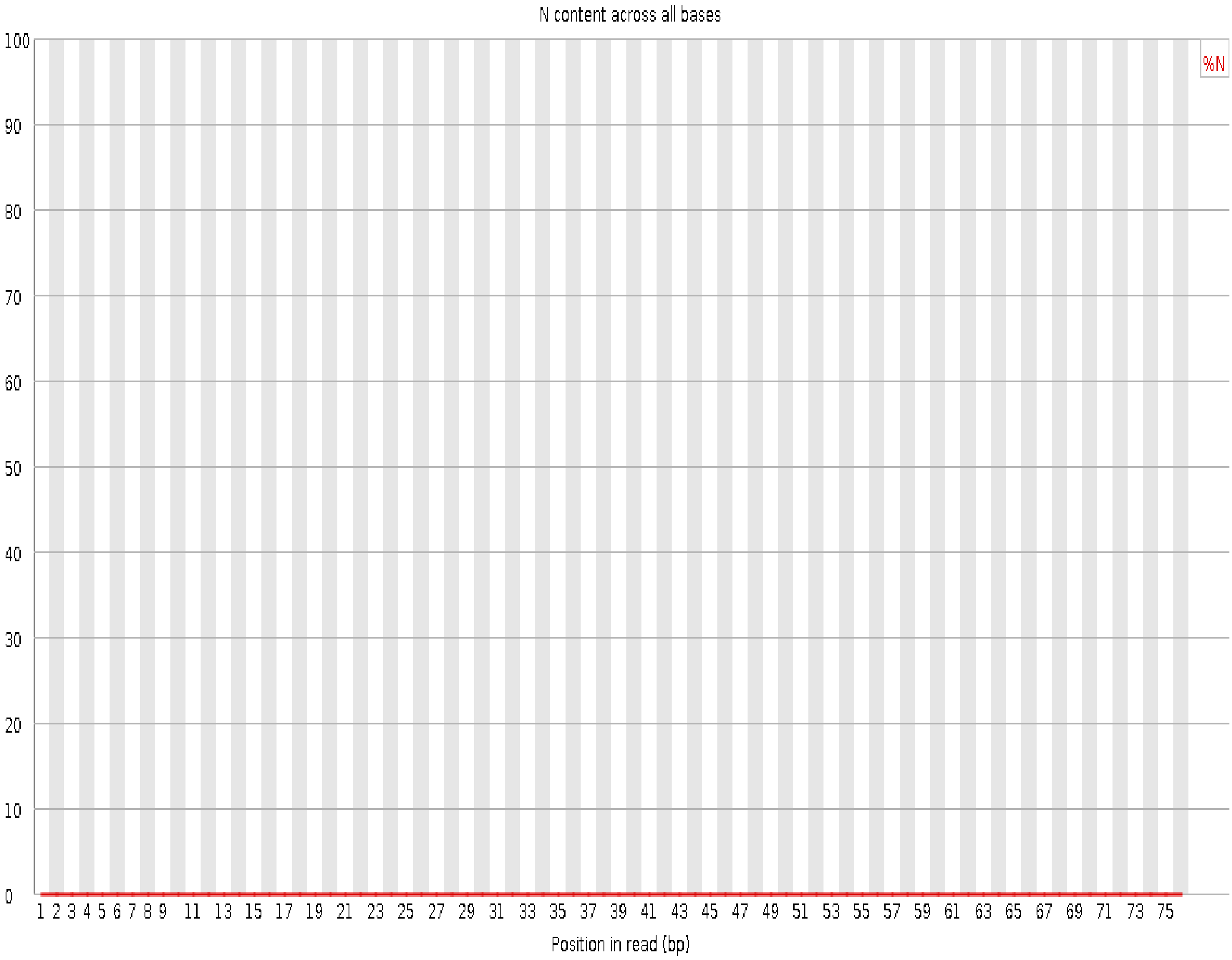


## Sequence Length Distribution

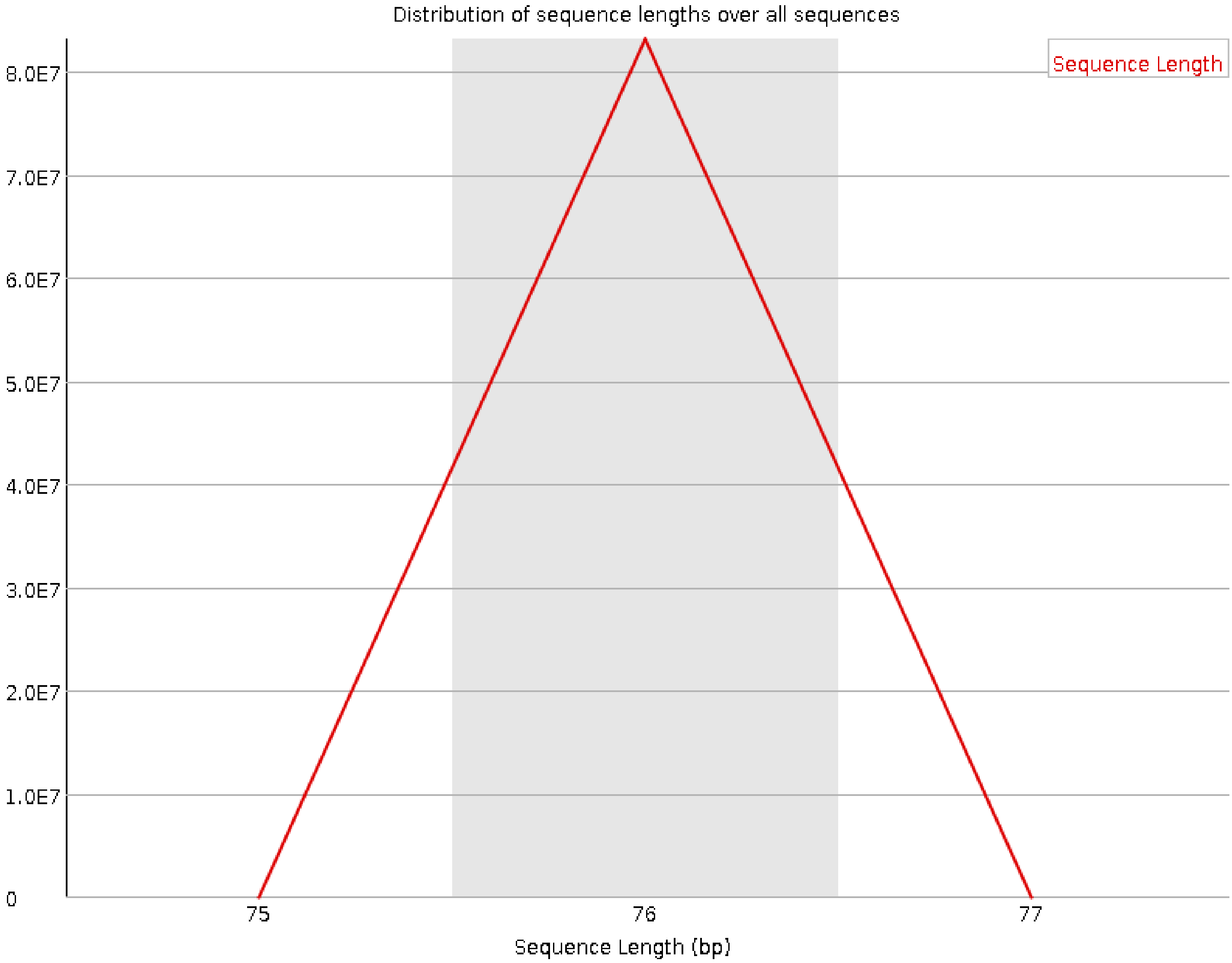


## Sequence Duplication Levels

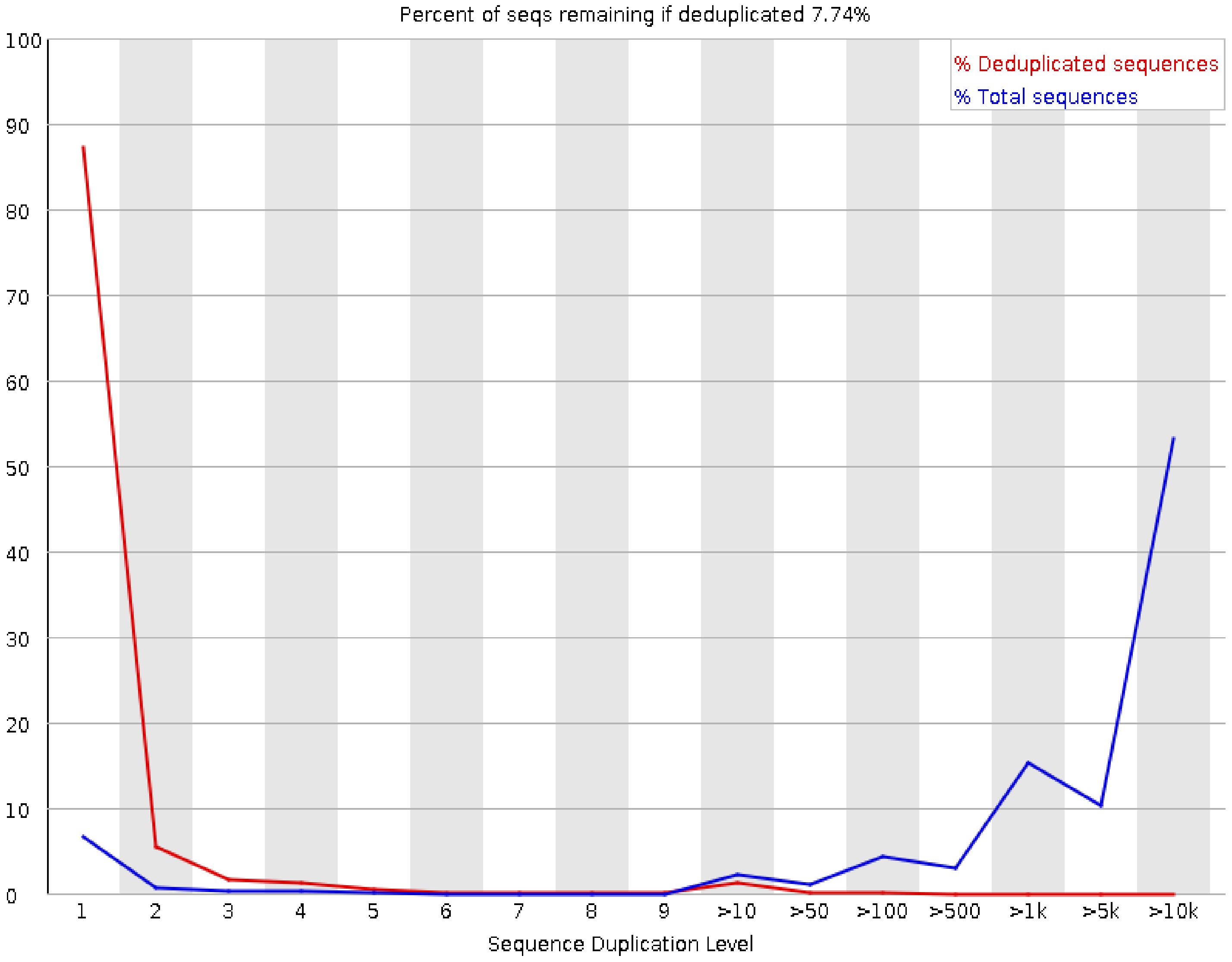


## Overrepresented sequences

| Sequence | Count | Percentage | Possible Source |
|---|---|---|---|
| CTTCCGTACGCCACATGTCCCGCGCCCCGCCGCGGGGCGGGGATTCGGCG | 367151 | 0.44135845519022965 | No Hit |
| CTGGATAGTAGGTAGGGACAGTGGGAATCTCGTTCATCCATTCATGCGCG | 271642 | 0.326545463541661 | No Hit |
| CCGGTATTTAGCCTTAGATGGAGTTTACCACCCGCTTTGGGCTGCATTCC | 261752 | 0.3146565265053152 | No Hit |
| CGCAGTTTTATCCGGTAAAGCGAATGATTAGAGGTCTTGGGGCCGAAACG | 247082 | 0.29702147025423414 | No Hit |
| CCTCACCCGGCCCGGACACGGACAGGATTGACAGATTGATAGCTCTTTCT | 245808 | 0.2954899732082984 | No Hit |
| CTTCACCGTGCCAGACTAGAGTCAAGCTCAACAGGGTCTTCTTTCCCCGC | 244794 | 0.2942710265799006 | No Hit |
| CGCGTAACTAGTTAGCATGCCAGAGTCTCGTTCGTTATCGGAATTAACCA | 237988 | 0.28608941834235063 | No Hit |
| CTCCCTTTCGATCGGCCGAGGGCAACGGAGGCCATCGCCCGTCCCTTCGG | 218591 | 0.2627719550770323 | No Hit |
| CCCGTCGGCATGTATTAGCTCTAGAATTACCACAGTTATCCAAGTAGGAG | 209450 | 0.25178340366659385 | No Hit |
| CTCTCTTCAAAGTTCTTTTCAACTTTCCCTTACGGTACTTGTTGACTATC | 207906 | 0.24992733503321493 | No Hit |
| CTCACCCGGCCCGGACACGGACAGGATTGACAGATTGATAGCTCTTTCTC | 201195 | 0.24185992790976535 | No Hit |

| Sequence | Count | Percentage | Possible Source |
|---|---|---|---|
| CTCTCATGTCTCTTCACCGTGCCAGACTAGAGTCAAGCTCAACAGGGTCT | 196856 | 0.2366439422878539 | No Hit |
| CAGAAACCTCCCGTGGAGCAGAAGGGCAAAAGCTCGCTTGATCTTGATTT | 187674 | 0.22560610408080373 | [illegible] |
| CTGCCAGTAGCATATGCTTGTCTCAAAGATTAAGCCATGCATGTCTAAGT | 185712 | 0.22324755054538306 | No Hit |
| CGGGTCTTCCGTACGCCACATGTCCCGCGCCCCGCCGCGGGGCGGGGATT | 175756 | 0.21127927378766231 | No Hit |
| CTTGAACTCTCTCTTCAAAGTTCTTTTCAACTTTCCCTTACGGTACTTGT | 169059 | 0.20322869630208018 | No Hit |
| GTCAAAGTGAAGAAATTCAATGAAGCGCGGGTAAACGGCGGGAGTAACTA | 168104 | 0.2020806745761236 | No Hit |
| CCCATATCCGCAGCAGGTCTCCAAGGTGAACAGCCTCTGGCATGTTGGAA | 159175 | 0.1913469719676776 | No Hit |
| CTCCGACTTTCGTTCTTGATTAATGAAAACATTCTTGGCAAATGCTTTCG | 157957 | 0.18988279347321158 | No Hit |
| CGCGATGTGATTTCTGCCCAGTGCTCTGAATGTCAAAGTGAAGAAATTCA | 157358 | 0.18916272539588386 | No Hit |
| CTCGCATTCCACGCCCGGCTCCACGCCAGCGAGCCGGGCTTCTTACCCAT | 155911 | 0.18742326211058638 | No Hit |
| CTCATGTCTCTTCACCGTGCCAGACTAGAGTCAAGCTCAACAGGGTCTTC | 155077 | 0.18642069654048402 | No Hit |
| CGAGATTCCCACTGTCCCTACCTACTATCCAGCGAAACCACAGCCAAGGG | 149558 | 0.1797862128697467 | No Hit |
| CCCGAAGTTACGGATCCGGCTTGCCGACTTCCCTTACCTACATTGTTCCA | 147146 | 0.17688670668858736 | No Hit |
| CTTGTCTCAAAGATTAAGCCATGCATGTCTAAGTACGCACGGCCGGTACA | 144454 | 0.17365060775007948 | No Hit |
| CTGCTGTCTATATCAACCAACACCTTTTCTGGGGTCTGATGAGCGTCGGC | 138087 | 0.1659967288713724 | No Hit |
| CTTTAAATGGGTAAGAAGCCCGGCTCGCTGGCGTGGAGCCGGGCGTGGAA | 136970 | 0.16465396419294992 | No Hit |
| CTCCGCCACTCCGGATTCGGGGATCTGAACCCGACTCCCTTTCGATCGGC | 136601 | 0.1642103830234442 | No Hit |
| CGAAGGCCCGCGGCGGGTGTTGACGCGATGTGATTTCTGCCCAGTGCTCT | 136587 | 0.16419355338557678 | No Hit |
| CGCGTCACTAATTAGATGACGAGGCATTTGGCTACCTTAAGAGAGTCATA | 135256 | 0.16259353567118082 | No Hit |
| CCACTCTCGACTGCCGGCGACGGCCGGGTATGGGCCCGACGCTCCAGCGC | 133112 | 0.16001619684348362 | No Hit |
| CTCCACTTCGGCCTTCAAAGTTCTCGTTTGAATATTTGCTACTACCACCA | 128609 | 0.1546030640351252 | No Hit |
| CAAAGATTAAGCCATGCATGTCTAAGTACGCACGGCCGGTACAGTGAAAC | 126351 | 0.15188868387050752 | No Hit |
| GTAAATCTCGCGCCGGGCCGTACCCATATCCGCAGCAGGTCTCCAAGGTG | 125691 | 0.15109528665675745 | No Hit |
| CCGACATCGAAGGATCAAAAAGCGACGTCGCTATGAACGCTTGGCCGCCA | 123913 | 0.1489579226475944 | No Hit |
| CTTAGAGCCAATCCTTATCCCGAAGTTACGGATCCGGCTTGCCGACTTCC | 123883 | 0.14892185913787848 | No Hit |
| CTCCCGTCCACTCTCGACTGCCGGCGACGGCCGGGTATGGGCCCGACGCT | 123798 | 0.1488196791936834 | No Hit |
| CCCACTTATTCTACACCTCTCATGTCTCTTCACCGTGCCAGACTAGAGTC | 119501 | 0.14365418248537423 | No Hit |
| CTCCCACTTATTCTACACCTCTCATGTCTCTTCACCGTGCCAGACTAGAG | 116741 | 0.14033633959151032 | No Hit |
| CTGAATTTAAGCATATTAGTCAGCGGAGGAGAAGAAACTAACCAGGATTC | 116144 | 0.13961867574816367 | No Hit |
| CTACTATCCAGCGAAACCACAGCCAAGGGAACGGGCTTGGCGGAATCAGC | 115648 | 0.13902242572086057 | No Hit |
| GTCGGCATGTATTAGCTCTAGAATTACCACAGTTATCCAAGTAGGAGAGG | 114402 | 0.1375245879506597 | No Hit |
| CCTGTGGTAACTTTTCTGACACCTCCTGCTTAAAACCCAAAAGGTCAGAA | 111643 | 0.13420794717378629 | No Hit |
| CTGAATTTAAGCATATTAGTCAGCGGAGGAAAAGAAACTAACCAGGATTC | 111164 | 0.13363213313532224 | No Hit |
| CGACGACCCATTCGAACGTCTGCCCTATCAACTTTCGATGGTAGTCGCCG | 107759 | 0.12953892478256618 | No Hit |
| CTTAGATGGAGTTTACCACCCGCTTTGGGCTGCATTCCCAAGCAACCCGA | 106156 | 0.12761193124674594 | No Hit |
| CTCGATCAGAAGGACTTGGGCCCCCCACGAGCGGCGCCGGGGAGCGGGTC | 106095 | 0.12753860211032358 | No Hit |
| CTCTGGTCCGTCTTGCGCCGGTCCAAGAATTTCACCTCTAGCGGCGCAAT | 103980 | 0.12499612467535177 | No Hit |
| CACGAGCGCACGTGTTAGGACCCGAAAGATGGTGAACTATGCCTGGGCAG | 103085 | 0.12392022996882705 | No Hit |
| CTTGGCTGTGGTTTCGCTGGATAGTAGGTAGGGACAGTGGGAATCTCGTT | 99501 | 0.11961184267476607 | No Hit |

| Sequence | Count | Percentage | Possible Source |
|---|---|---|---|
| CGAACGCCGGGTTAAGGCGCCCGATGCCGACGCTCATCAGACCCCAGAAA | 98955 | 0.11895548679793648 | No Hit |
| CACCCGTTTACCTCTTAACG[illegible]CCTCTTGAACTCTCTCTTCAA | [illegible] | 0.11[illegible]7776 | [illegible] |
| CGGCCTTCAAAGTTCTCGTTTGAATATTTGCTACTACCACCAAGATCTGC | 97660 | 0.1173987452951996 | No Hit |
| CAAACTTTAAATGGGTAAGAAGCCCGGCTCGCTGGCGTGGAGCCGGGCGT | 96904 | 0.11648994485035861 | No Hit |
| CGAGAACTTTGAAGGCCGAAGTGGAGAAGGGTTCCATGTGAACAGCAGTT | 96838 | 0.11641060512898362 | No Hit |
| CCCGCTTTGGGCTGCATTCCCAAGCAACCCGACTCCGGGAAGACCCGGGC | 96053 | 0.11546694329141723 | No Hit |
| CCCAGGCATAGTTCACCATCTTTCGGGTCCTAACACGTGCGCTCGTGCTC | 95347 | 0.11461824869610276 | No Hit |
| CCCTGTGGTAACTTTTCTGACACCTCCTGCTTAAAACCCAAAAGGTCAGA | 94777 | 0.11393304201150045 | No Hit |
| CTGGGGTCTGATGAGCGTCGGCATCGGGCGCCTTAACCCGGCGTTCGGTT | 93240 | 0.11208538819705519 | No Hit |
| CTGAATGTCAAAGTGAAGAAATTCAATGAAGCGCGGGTAAACGGCGGGAG | 92445 | 0.11112970518958352 | No Hit |
| CCACCGTCCTGCTGTCTATATCAACCAACACCTTTTCTGGGGTCTGATGA | 91163 | 0.10958859120772353 | No Hit |
| CTCGACTGCCGGCGACGGCCGGGTATGGGCCCGACGCTCCAGCGCCATCC | 89903 | 0.10807392379965522 | No Hit |
| CACCGTGCCAGACTAGAGTCAAGCTCAACAGGGTCTTCTTTCCCCGCTGA | 89618 | 0.10773132045735408 | No Hit |
| CTTCCGTCAATTCCTTTAAGTTTCAGCTTTGCAACCATACTCCCCCCGGA | 88903 | 0.10687180680912484 | No Hit |
| CCCCGCTTCGCGCCCCAGCCCGACCGACCCAGCCCTTAGAGCCAATCCTT | 88279 | 0.10612168580703385 | No Hit |
| CTCAAACTTTAAATGGGTAAGAAGCCCGGCTCGCTGGCGTGGAGCCGGGC | 87617 | 0.10532588435930271 | No Hit |
| CGCTGATTCCGCCAAGCCCGTTCCCTTGGCTGTGGTTTCGCTGGATAGTA | 86051 | 0.1034433691521321 | No Hit |
| CCCGCTTCGCGCCCCAGCCCGACCGACCCAGCCCTTAGAGCCAATCCTTA | 85060 | 0.10225207121451647 | No Hit |
| CACCTCTCATGTCTCTTCACCGTGCCAGACTAGAGTCAAGCTCAACAGGG | 84801 | 0.10194072291396909 | No Hit |
| GCCCTCTTGAACTCTCTCTTCAAAGTTCTTTTCAACTTTCCCTTACGGTA | 84709 | 0.10183012815084029 | No Hit |
| CTGCTGCCTTCCTTGGATGTGGTAGCCGTTTCTCAGGCTCCCTCTCCGGA | 84155 | 0.10116415533808645 | No Hit |
| CTTAAGAGAGTCATAGTTACTCCCGCCGTTTACCCGCGCTTCATTGAATT | 83980 | 0.10095378486474361 | No Hit |
| CTCCGTTTCCGACCTGGGCCGGTTCACCCCTCCTTAGGCAACCTGGTGGT | 83456 | 0.1003238755617057 | No Hit |

## Adapter Content

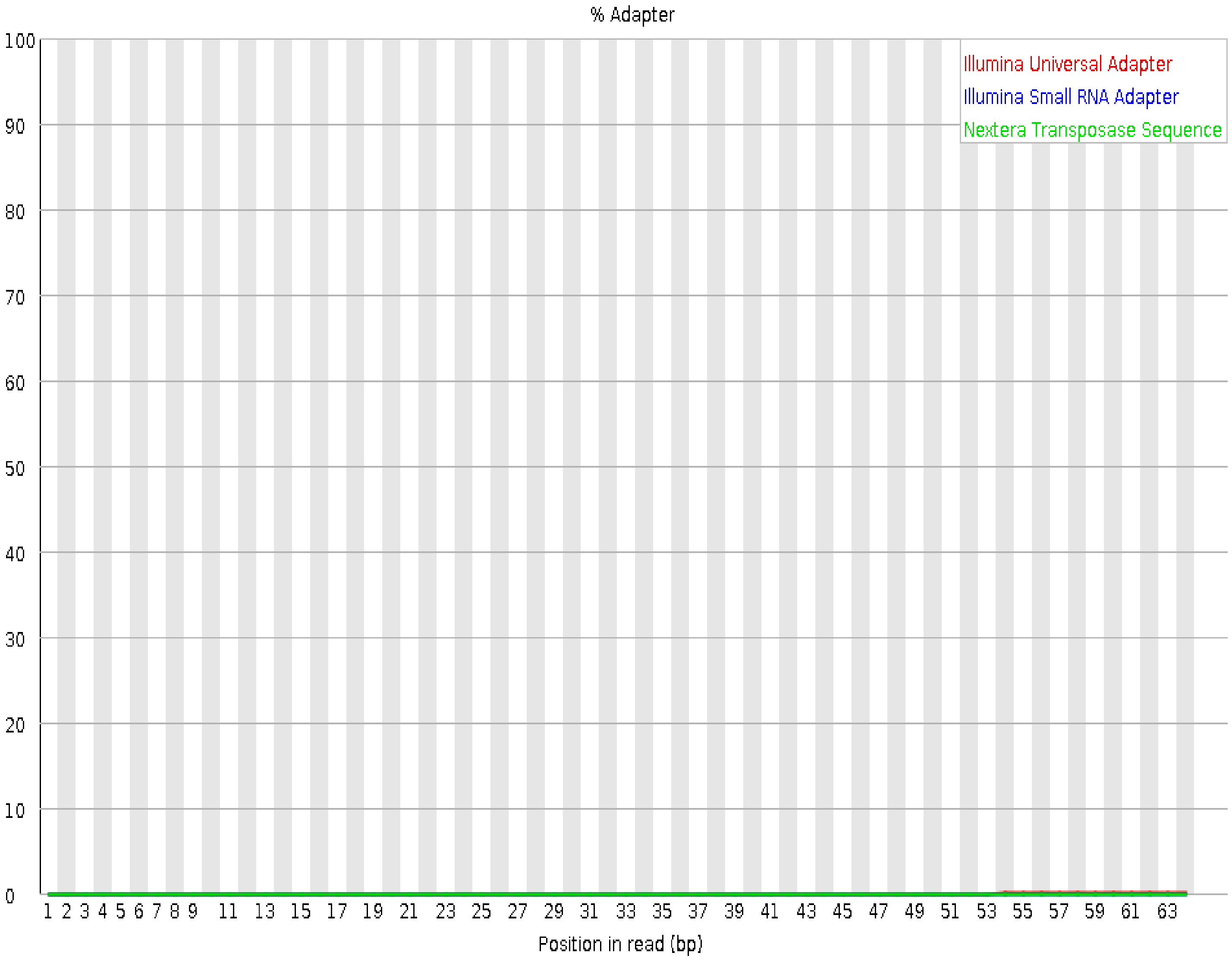


## Kmer Content

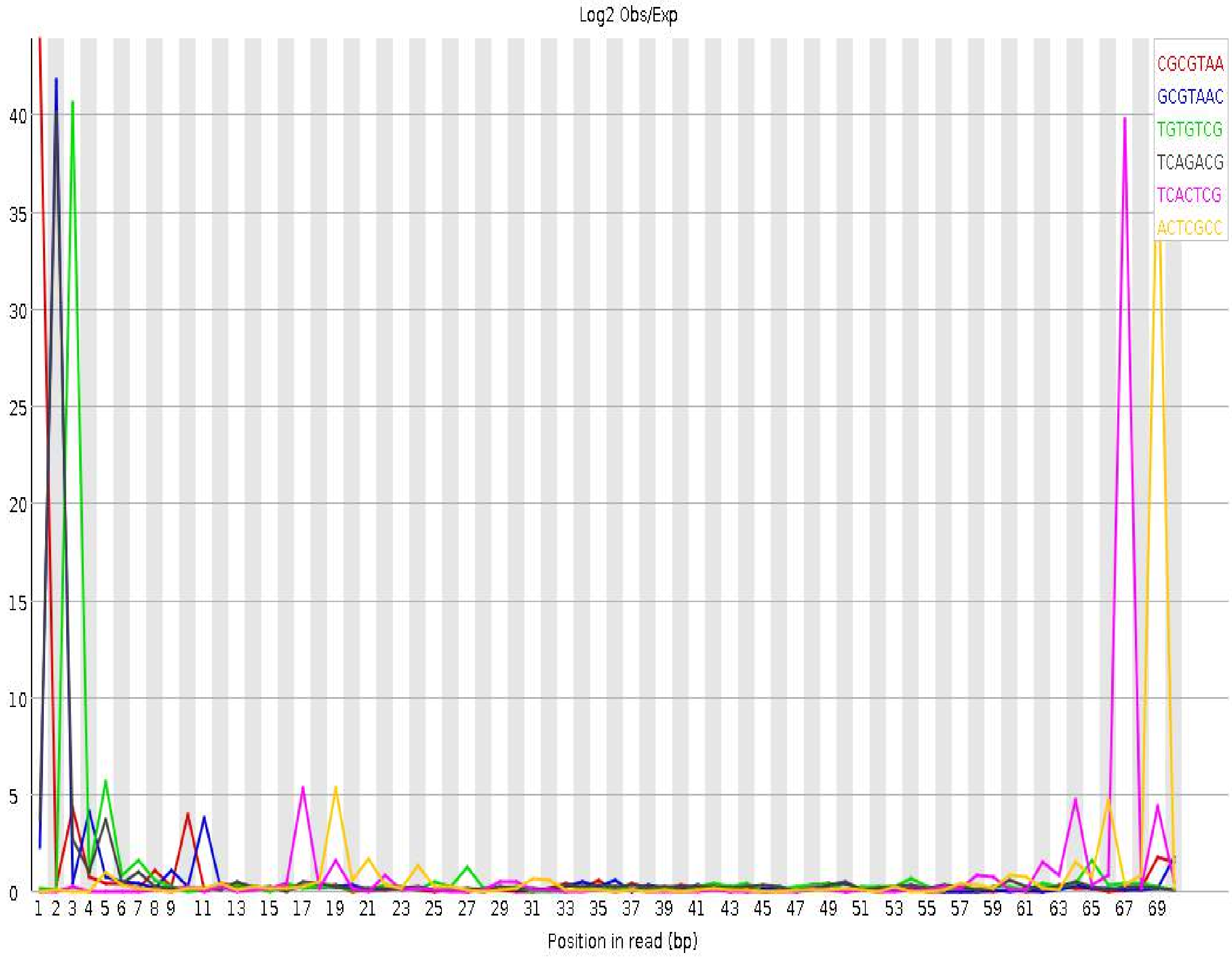


| Sequence | Count | PValue | Obs/Exp Max | Max Obs/Exp Position |
|---|---|---|---|---|
| CGCGTAA | 45595 | 0.0 | 43.881477 | 1 |
| GCGTAAC | 47900 | 0.0 | 41.84679 | 2 |
| TGTGTCG | 6310 | 0.0 | 40.67053 | 3 |
| TCAGACG | 15800 | 0.0 | 40.10917 | 2 |
| TCACTCG | 73895 | 0.0 | 39.809986 | 67 |
| ACTCGCC | 75325 | 0.0 | 39.185677 | 69 |
| CCAGTAG | 40040 | 0.0 | 37.790585 | 4 |
| TCGCCTA | 6410 | 0.0 | 37.615845 | 66 |
| TATCGCC | 6460 | 0.0 | 37.59637 | 64 |
| TTCACTC | 78190 | 0.0 | 37.439007 | 66 |
| TGTCGAG | 7140 | 0.0 | 37.31274 | 5 |
| TGCCAGT | 40935 | 0.0 | 37.129337 | 2 |
| CGCCTAT | 6520 | 0.0 | 36.928234 | 67 |
| GTGTCGA | 7280 | 0.0 | 36.837513 | 4 |
| CTCGCTA | 8145 | 0.0 | 35.890343 | 1 |

| Sequence | Count | PValue | Obs/Exp Max | Max Obs/Exp Position |
|---|---|---|---|---|
| AGTTAGC | 55960 | 0.0 | 35.[illegible] | 10 |
| GTTAGCA | 55910 | 0.0 | 35.7[illegible] | 11 |
| TAGTTAG | 57385 | 0.0 | 35.279213 | 9 |
| CTATCGC | 6955 | 0.0 | 35.17195 | 63 |
| CTTGTGT | 8045 | 0.0 | 34.508762 | 1 |

**Produced by FastQC (version 0.11.2)**

# FastQC Report

Mon 4 May 2015
Biochain_Fetal_Colon_ACAGTG_L006_R1.fastq.gz

## Summary


- Basic Statistics
- Per base sequence quality
- Per tile sequence quality
- Per sequence quality scores
- Per base sequence content
- Per sequence GC content
- Per base N content
- Sequence Length Distribution
- Sequence Duplication Levels
- Overrepresented sequences
- Adapter Content
- Kmer Content


## Basic Statistics

| Measure | Value |
|---|---|
| Filename | Biochain_Fetal_Colon_ACAGTG_L006_R1.fastq.gz |
| File type | Conventional base calls |
| Encoding | Sanger / Illumina 1.9 |
| Total Sequences | 78419198 |
| Sequences flagged as poor quality | 0 |
| Sequence length | 76 |
| %GC | 48 |

## Per base sequence quality

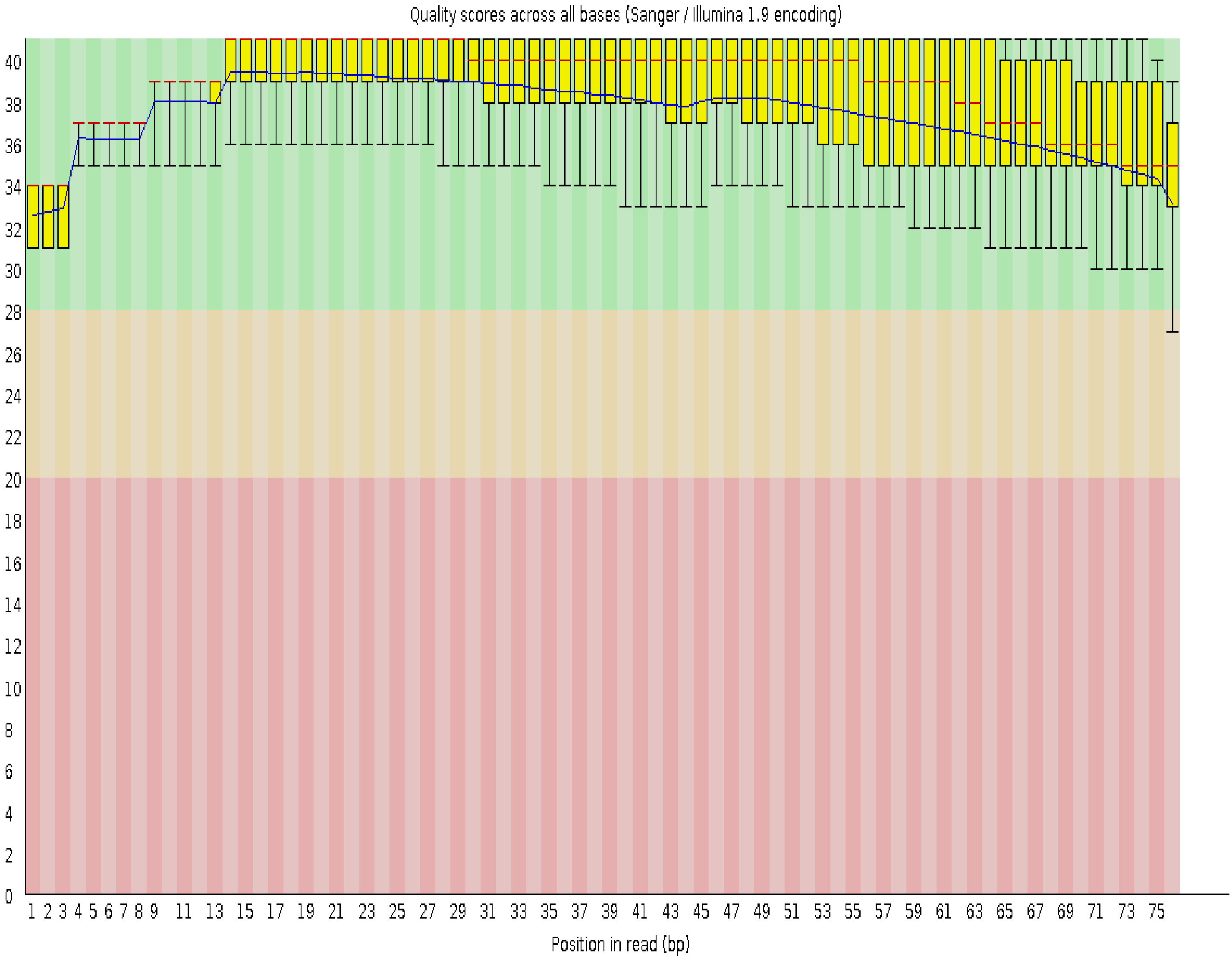


## Per tile sequence quality

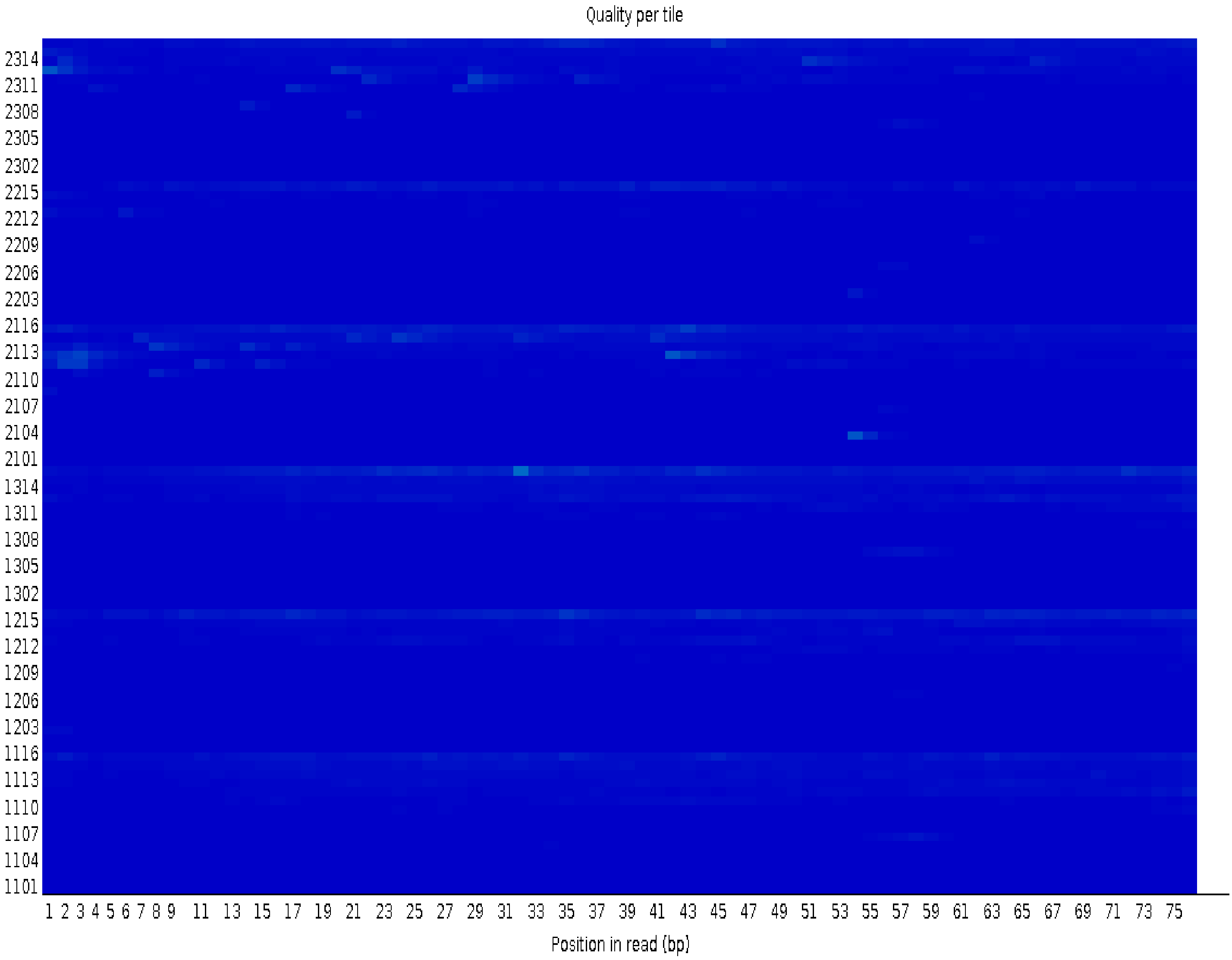


## Per sequence quality scores

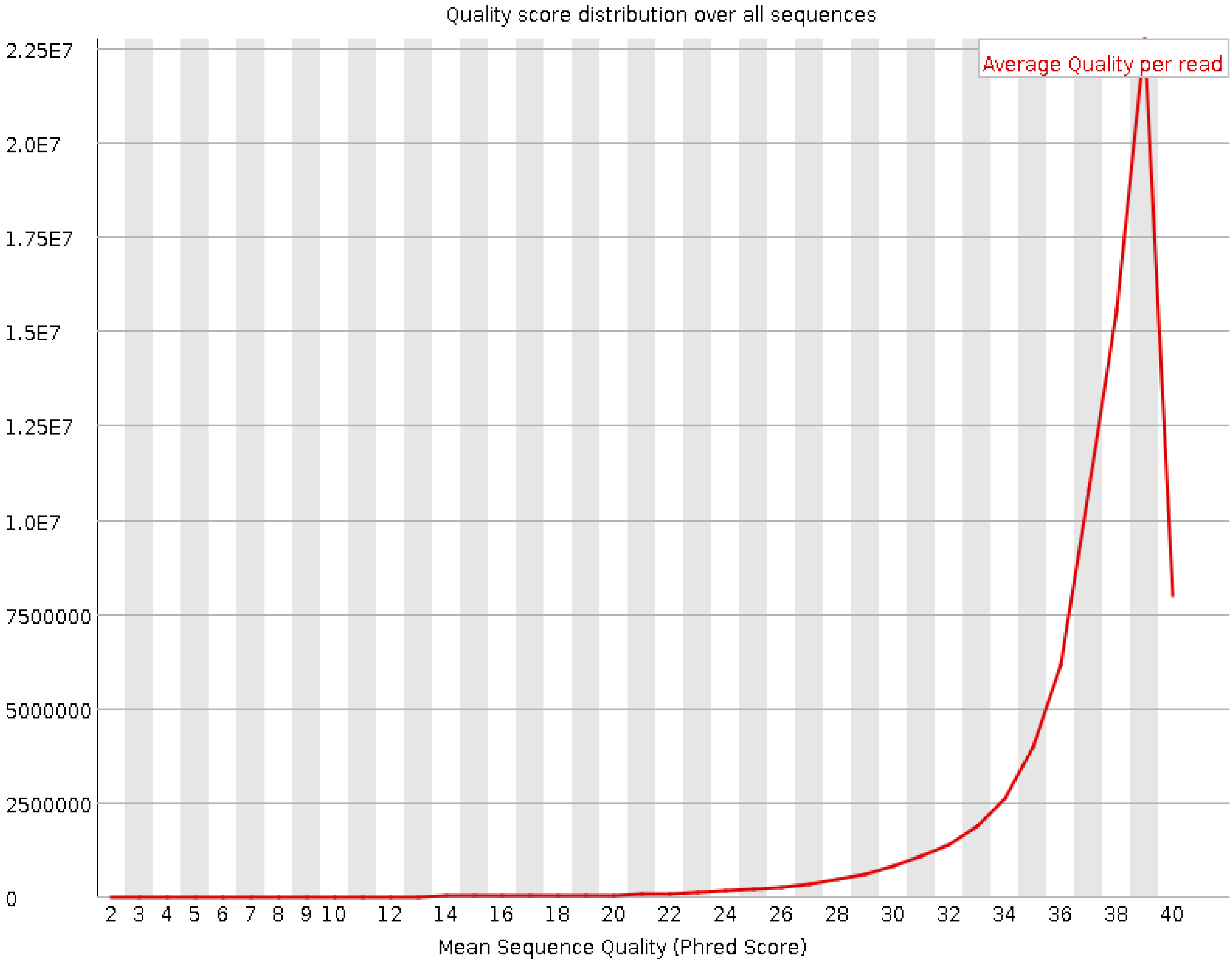


## Per base sequence content

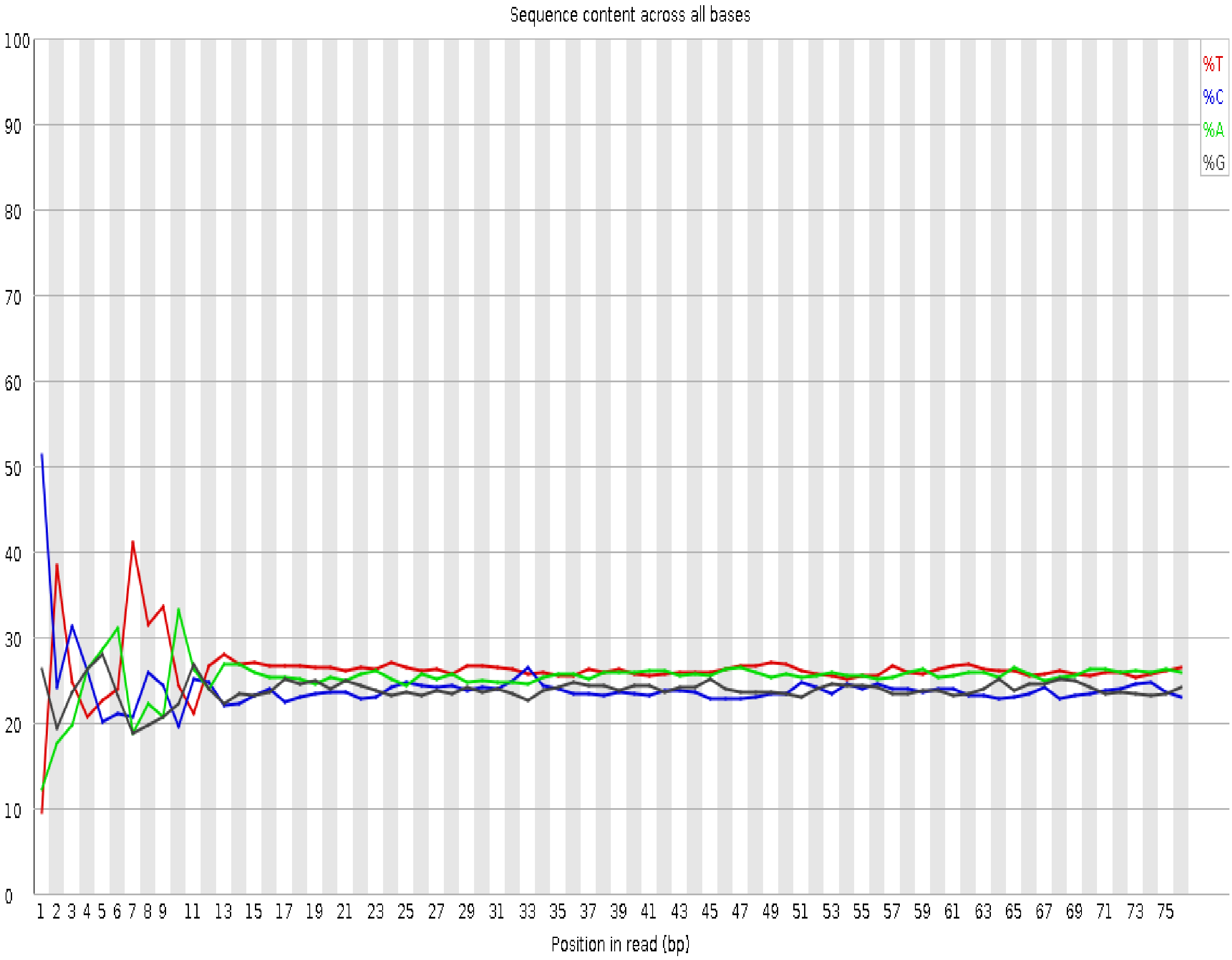


## Per sequence GC content

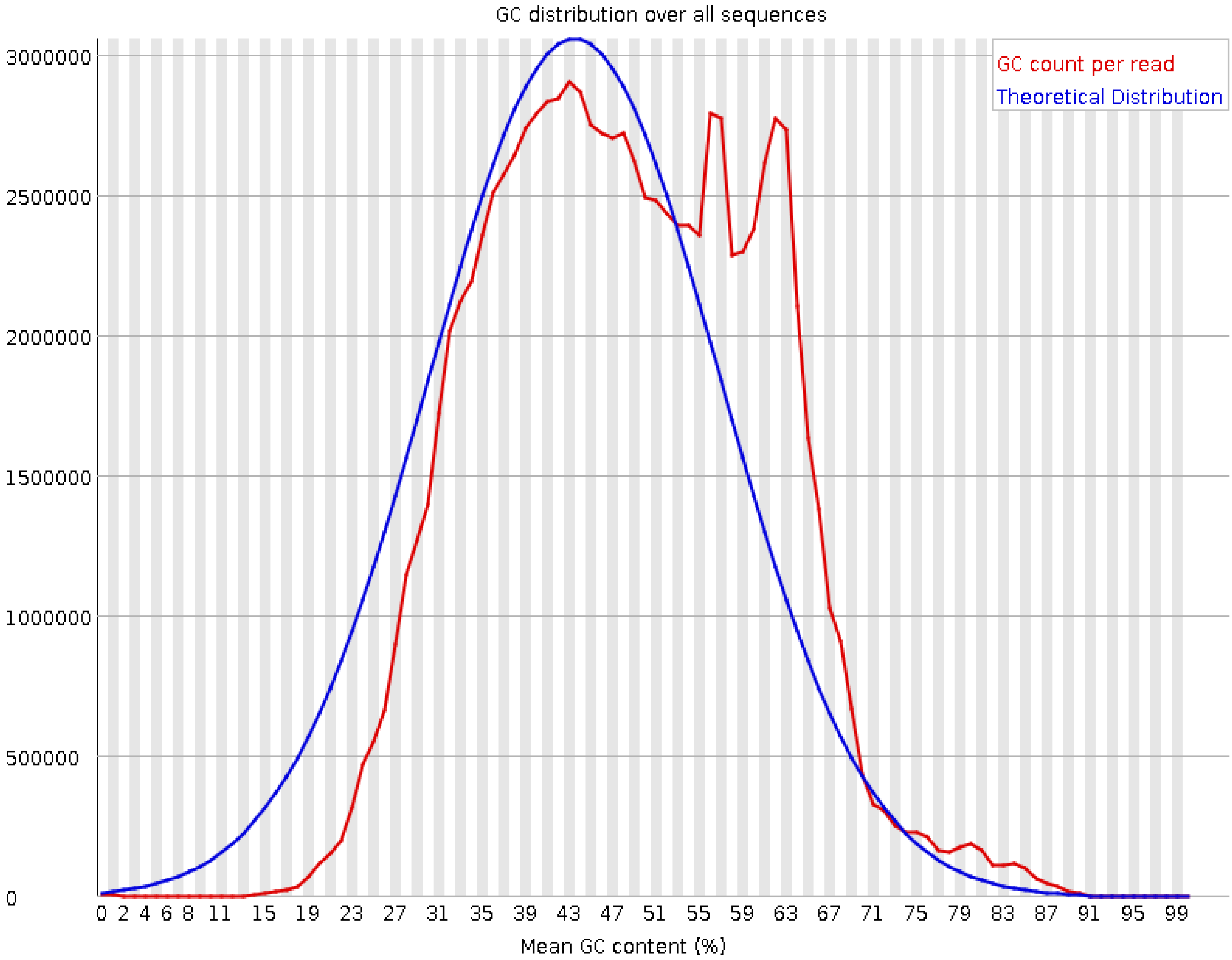


## Per base N content

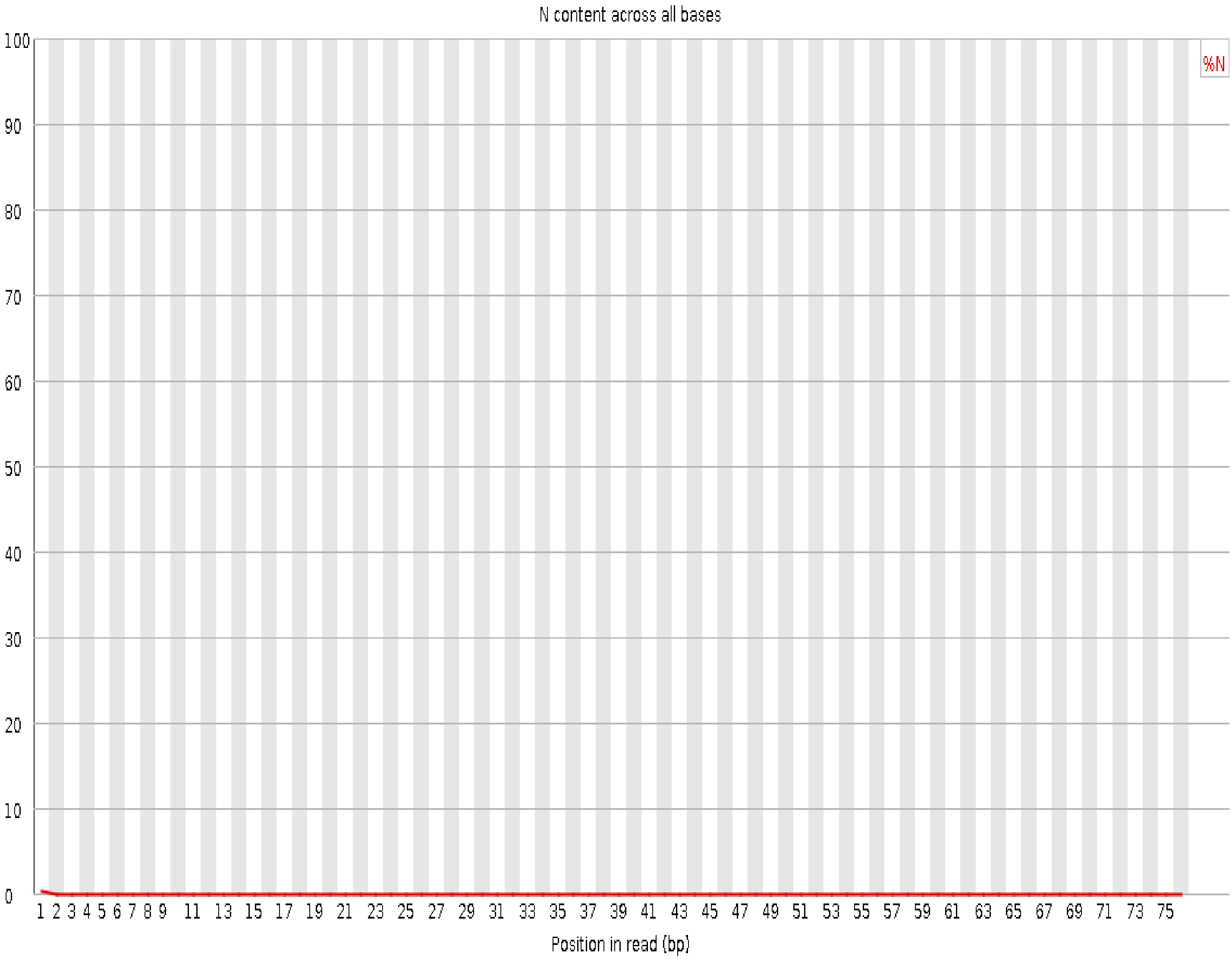


# Sequence Length Distribution

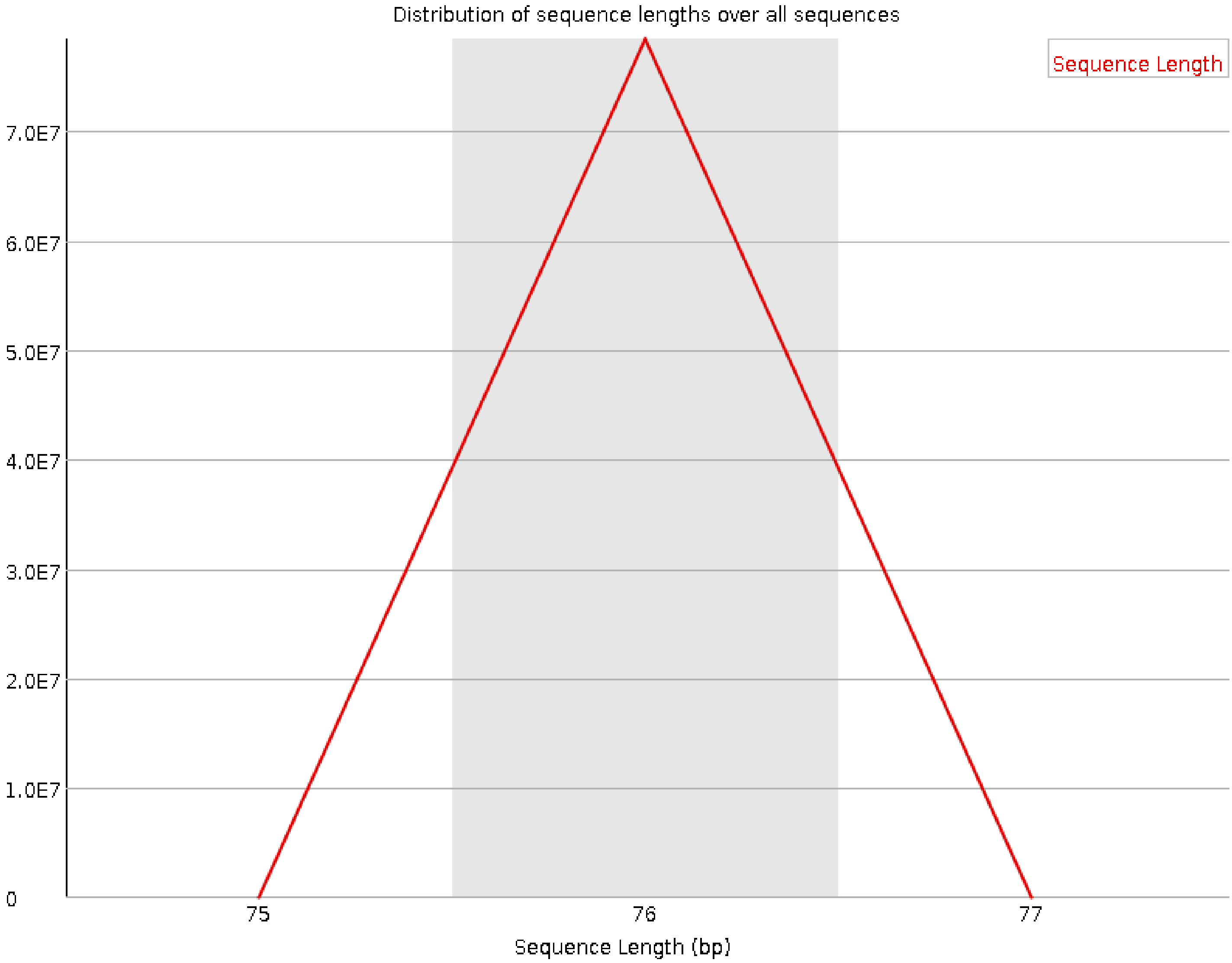


## Sequence Duplication Levels

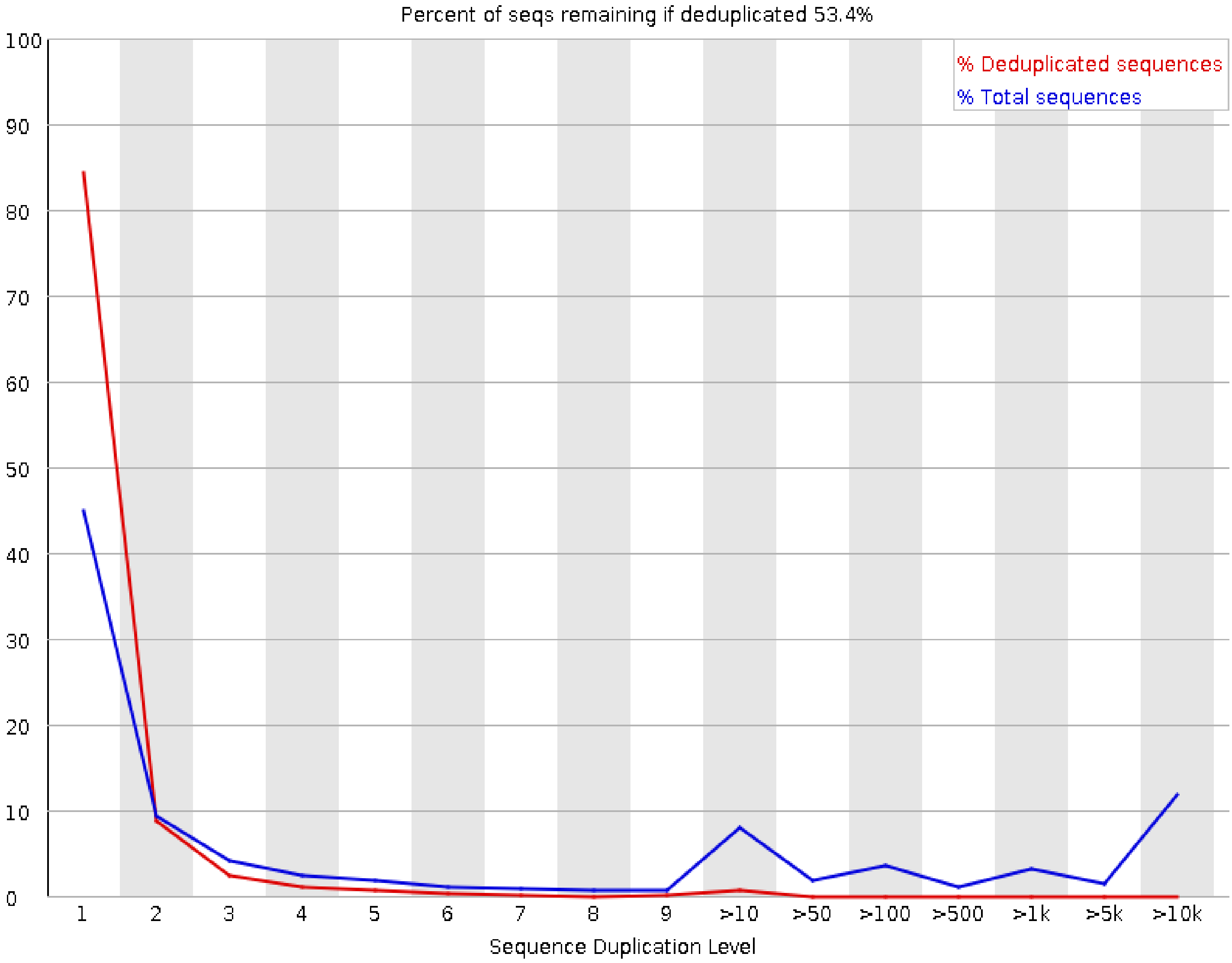


## Overrepresented sequences

| Sequence | Count | Percentage | Possible Source |
|---|---|---|---|
| CCCTCCTTAGGCAACCTGGTGGTCCCCCGCTCCCGGGAGGTCACCATATT | 289939 | 0.3697296164645805 | No Hit |
| CCCCACTACCACAAATTATGCAGTCGAGTTTCCCACATTTGGGGAAATCG | 262446 | 0.3346705994111289 | No Hit |
| CTCCGTTTCCGACCTGGGCCGGTTCACCCCTCCTTAGGCAACCTGGTGGT | 249757 | 0.3184896127093776 | No Hit |
| CTGGAGTCTTGGAAGCTTGACTACCCTACGTTCTCCTACAAATGGACCTT | 231794 | 0.2955832320549873 | No Hit |
| GGGAGATACCATGATCACGAAGGTGGTTTTCCCAGGGCGAGGCTTATCCA | 219713 | 0.28017756570272495 | No Hit |
| CCCCTCCTTAGGCAACCTGGTGGTCCCCCGCTCCCGGGAGGTCACCATAT | 183700 | 0.23425386217288272 | No Hit |
| CCCCCACTACCACAAATTATGCAGTCGAGTTTCCCACATTTGGGGAAATC | 183598 | 0.2341237919826724 | No Hit |
| CCTCCTTAGGCAACCTGGTGGTCCCCCGCTCCCGGGAGGTCACCATATTG | 175238 | 0.2234631371771999 | No Hit |
| CCATGATCACGAAGGTGGTTTTCCCAGGGCGAGGCTTATCCATTGCACTC | 162391 | 0.20708066920041698 | No Hit |
| GTGCGCTATGCCGATCGGGTGTCCGCACTAAGTTCGGCATCAATATGGTG | 152398 | 0.19433761615363626 | No Hit |
| CTGGCTGCGACATCTGTCACCCCATTGATCGCCAGGGTTGATTCGGCTGA | 148822 | 0.18977750830861598 | No Hit |

| Sequence | Count | Percentage | Possible Source |
|---|---|---|---|
| CCCACTACCACAAATTATGCAGTCGAGTTTCCCACATTTGGGGAAATCGC | 139070 | 0.1773417779661557 | No Hi[illegible] |
| GATCGGAAGAGCACACGTCTGAACTCCAGTCACACAGTGATCTCGTATGC | 124213 | 0.1583961621234637 | [illegible] Adapter, Index 5 (100% over 50bp) |
| CCAGGCTGGAGTGCAGTGGCTATTCACAGGCGCGATCCCACTACTGATCA | 119065 | 0.15183144311167274 | No Hit |
| GTGGCTATTCACAGGCGCGATCCCACTACTGATCAGCACGGGAGTTTTGA | 118400 | 0.15098343647941923 | No Hit |
| CTCAGGCTGGAGTGCAGTGGCTATTCACAGGCGCGATCCCACTACTGATC | 108749 | 0.13867650112922603 | No Hit |
| GTCCGCACTAAGTTCGGCATCAATATGGTGACCTCCCGGGAGCGGGGGAC | 106695 | 0.1360572445538145 | No Hit |
| GTGGCGCGTGCCTGTAGTCCCAGCTACTCGGGAGGCTGAGGTGGGAGGAT | 105496 | 0.13452828221987173 | No Hit |
| CTCGCTATGTTGCCCAGGCTGGAGTGCAGTGGCTATTCACAGGCGCGATC | 100972 | 0.12875928672466147 | No Hit |
| CCTTAGGCAACCTGGTGGTCCCCCGCTCCCGGGAGGTCACCATATTGATG | 95652 | 0.12197523366663351 | No Hit |
| AGGGGAGATACCATGATCACGAAGGTGGTTTTCCCAGGGCGAGGCTTATC | 86576 | 0.11040153713380235 | No Hit |
| CTCCTCTATCGGGGATGGTCGTCCTCTTCGACCGAGCGCGCAGCTTCGGG | 85587 | 0.10914036636793965 | No Hit |
| CTGGAGTGCAGTGGCTATTCACAGGCGCGATCCCACTACTGATCAGCACG | 83212 | 0.1061117712527486 | No Hit |
| GGGGAGATACCATGATCACGAAGGTGGTTTTCCCAGGGCGAGGCTTATCC | 82426 | 0.10510946566936327 | No Hit |
| CTCGCTATGTTGCTCAGGCTGGAGTGCAGTGGCTATTCACAGGCGCGATC | 78698 | 0.10035552773697075 | No Hit |

## Adapter Content

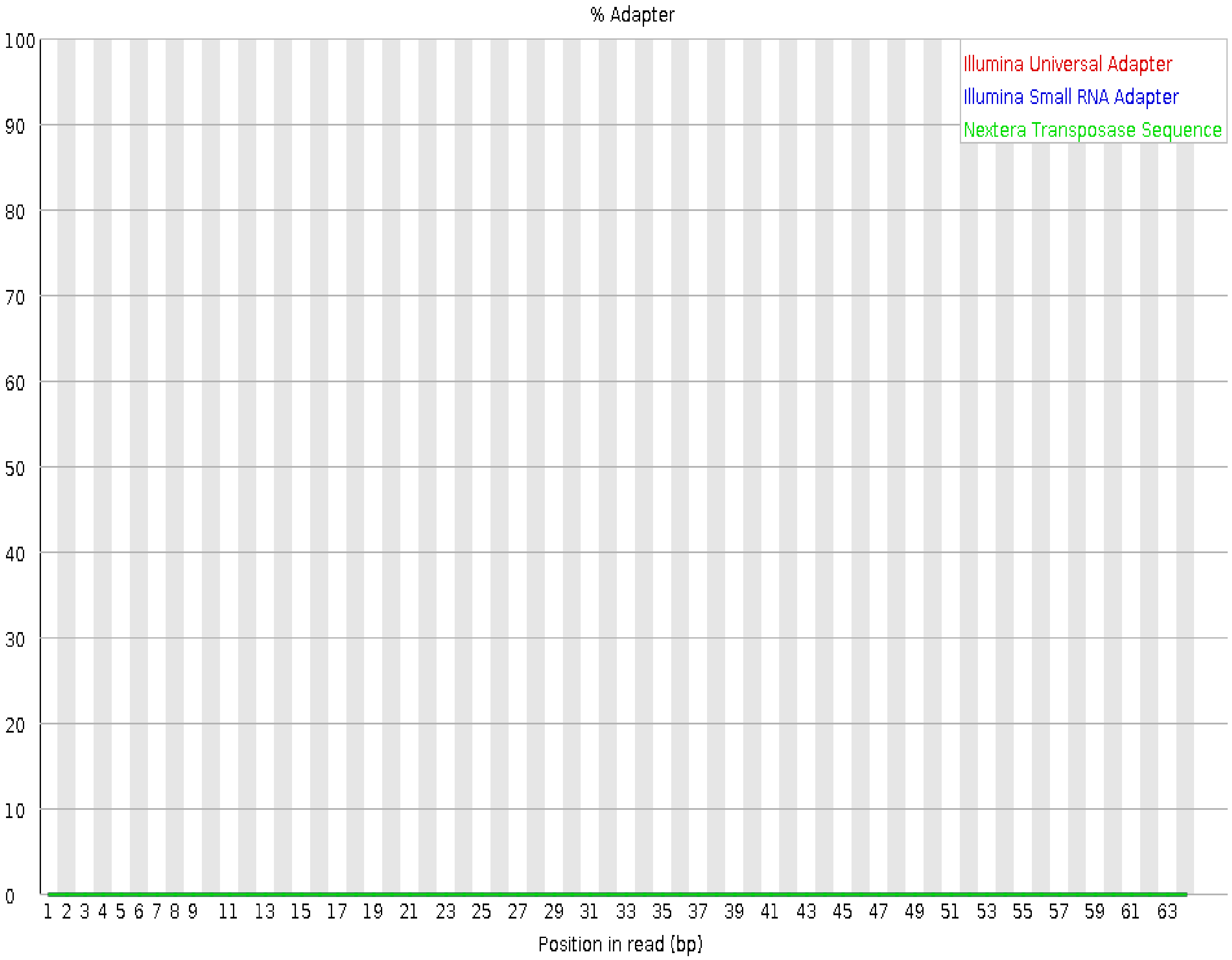


## Kmer Content

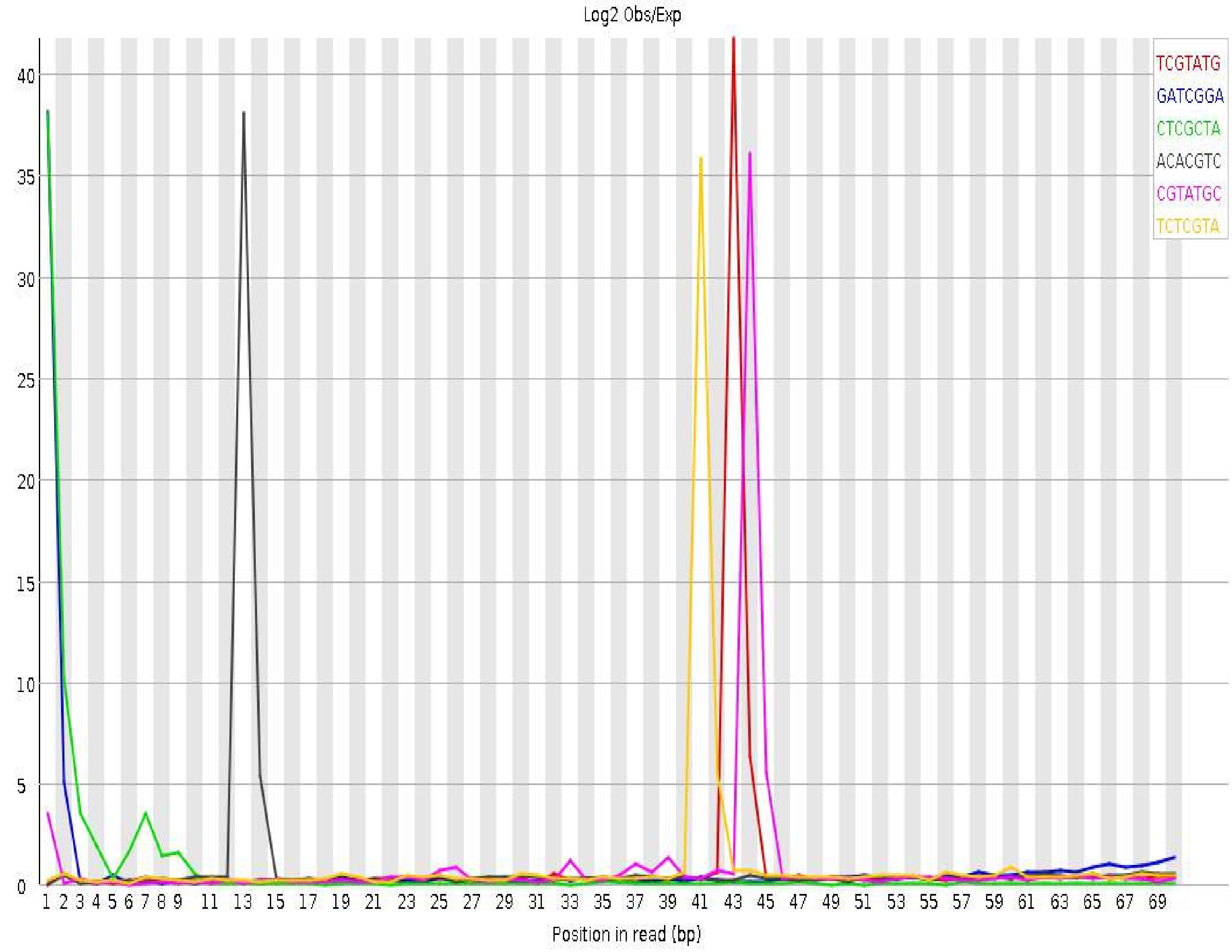


| Sequence | Count | PValue | Obs/Exp Max | Max Obs/Exp Position |
|---|---|---|---|---|
| TCGTATG | 23930 | 0.0 | 41.74011 | 43 |
| GATCGGA | 26695 | 0.0 | 38.19135 | 1 |
| CTCGCTA | 34595 | 0.0 | 38.118046 | 1 |
| ACACGTC | 26630 | 0.0 | 38.1099 | 13 |
| CGTATGC | 27770 | 0.0 | 36.06891 | 44 |
| TCTCGTA | 27230 | 0.0 | 35.846798 | 41 |
| CGTCTGA | 28350 | 0.0 | 35.550137 | 16 |
| ATCGGAA | 29630 | 0.0 | 34.40337 | 2 |
| TCGCTAT | 38740 | 0.0 | 34.262215 | 2 |
| ATCTCGT | 28450 | 0.0 | 34.17429 | 40 |
| CACGTCT | 30095 | 0.0 | 33.639954 | 14 |
| ACGTCTG | 30405 | 0.0 | 33.2049 | 15 |
| GCCGTCT | 30665 | 0.0 | 32.77392 | 49 |
| ATGCCGT | 31110 | 0.0 | 32.39506 | 47 |
| TGCCGTC | 31375 | 0.0 | 32.076817 | 48 |

| Sequence | Count | PValue | Obs/Exp Max | Max Obs/Exp Position |
|---|---|---|---|---|
| CACACGT | 32195 | 0.0 | 31.[illegible] | 12 |
| GCACACG | 32720 | 0.0 | 31.2[illegible] | 11 |
| TCGGAAG | 33345 | 0.0 | 30.884878 | 3 |
| TGGCGCG | 45695 | 0.0 | 29.951002 | 2 |
| GATCTCG | 33450 | 0.0 | 29.424053 | 39 |

**Produced by FastQC (version 0.11.2)**

# FastQC Report

Mon 4 May 2015
Biochain_Fetal_Colon_ACAGTG_L006_R2.fastq.gz

## Summary


- Basic Statistics
- Per base sequence quality
- Per tile sequence quality
- Per sequence quality scores
- Per base sequence content
- Per sequence GC content
- Per base N content
- Sequence Length Distribution
- Sequence Duplication Levels
- Overrepresented sequences
- Adapter Content
- Kmer Content


## Basic Statistics

| Measure | Value |
| --- | --- |
| Filename | Biochain_Fetal_Colon_ACAGTG_L006_R2.fastq.gz |
| File type | Conventional base calls |
| Encoding | Sanger / Illumina 1.9 |
| Total Sequences | 78419198 |
| Sequences flagged as poor quality | 0 |
| Sequence length | 76 |
| %GC | 48 |

## Per base sequence quality

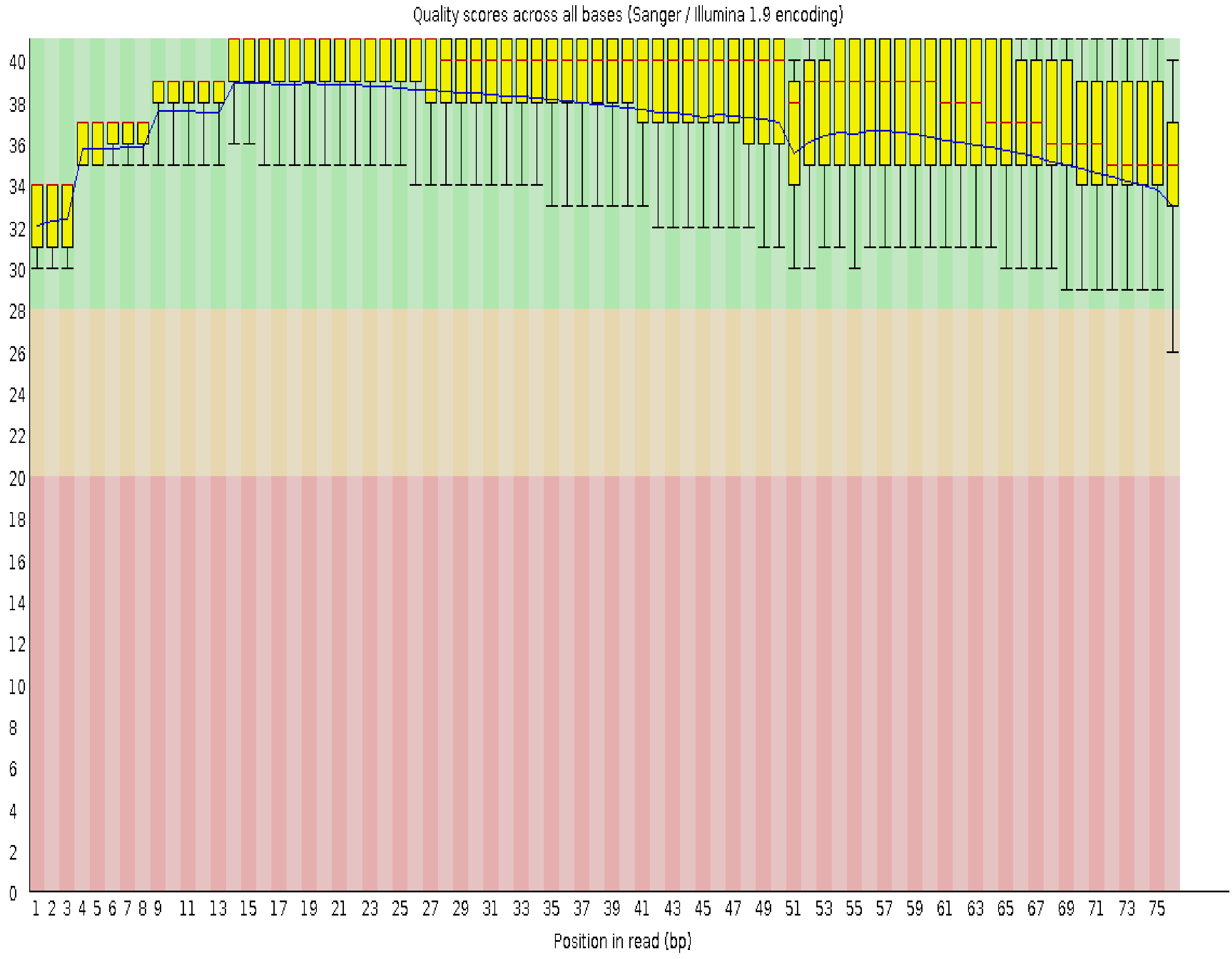


## Per tile sequence quality

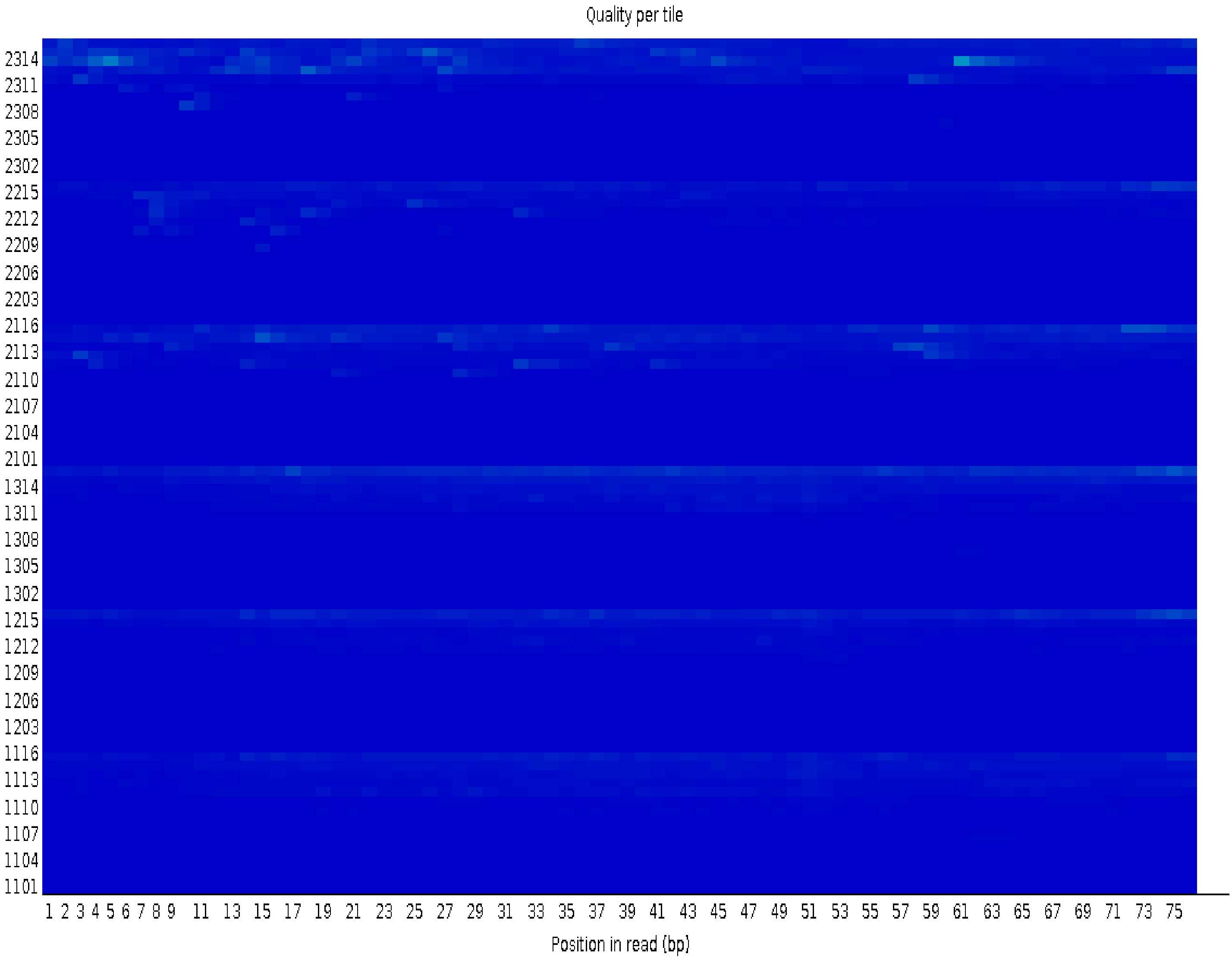


## Per sequence quality scores

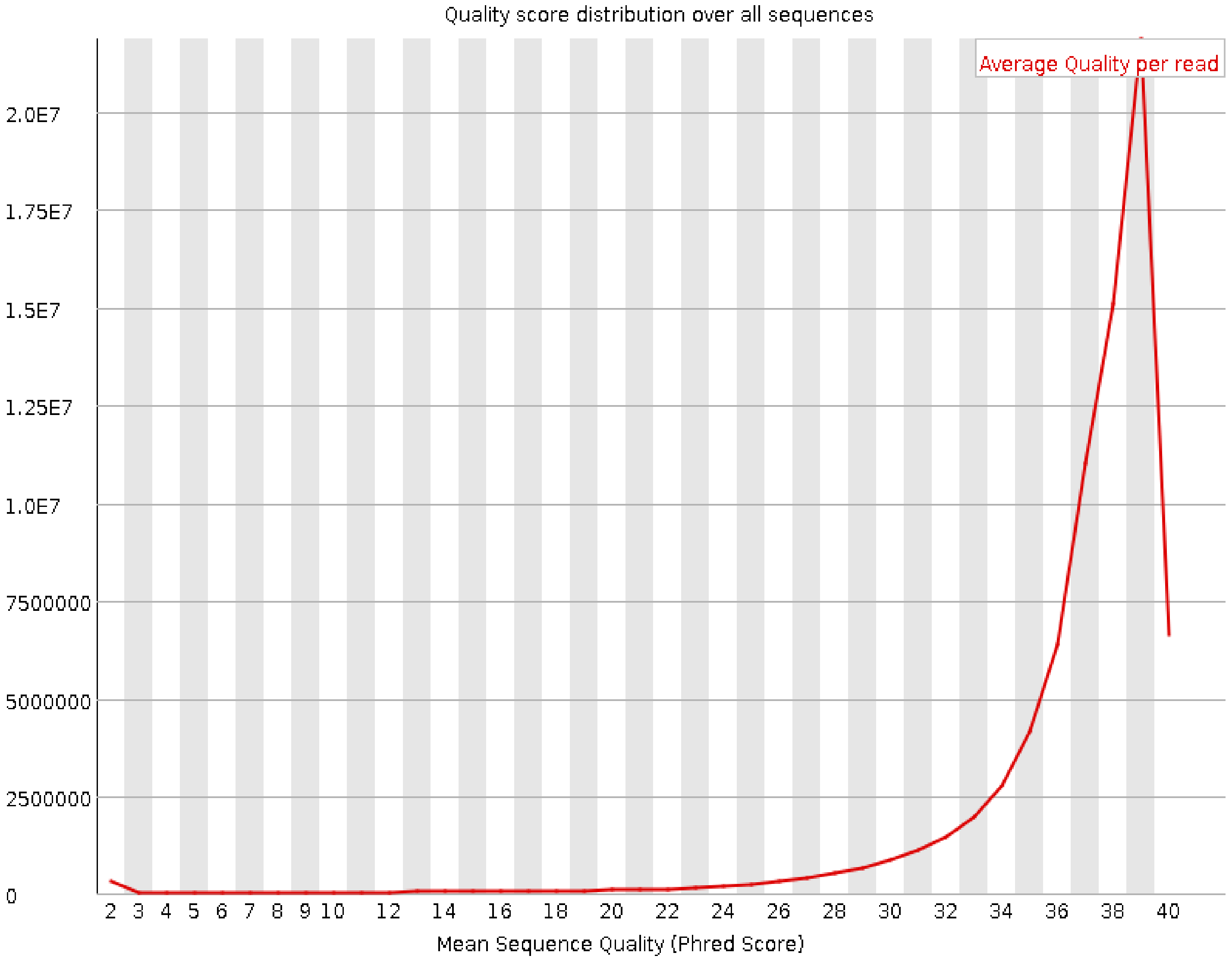


## Per base sequence content

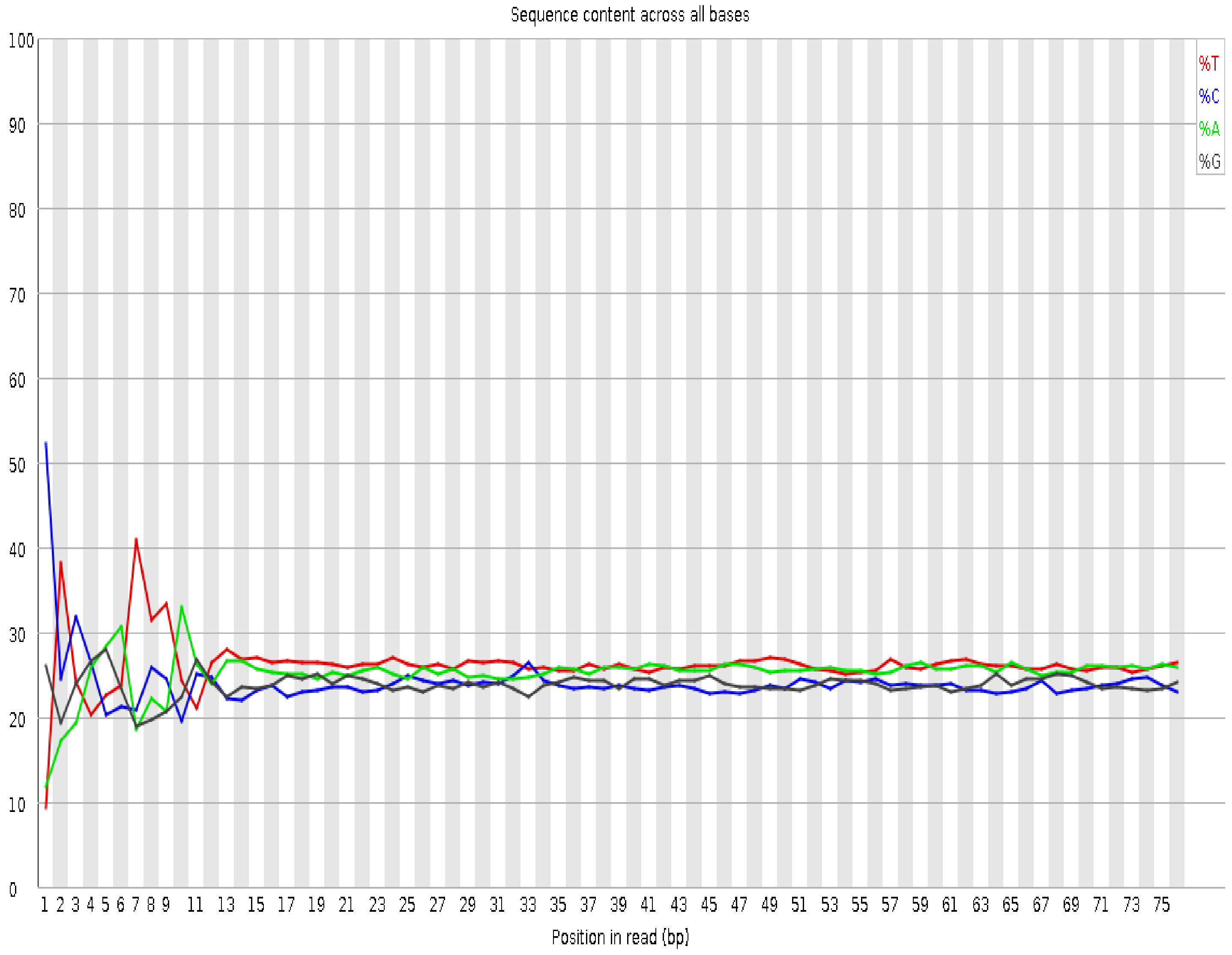


## Per sequence GC content

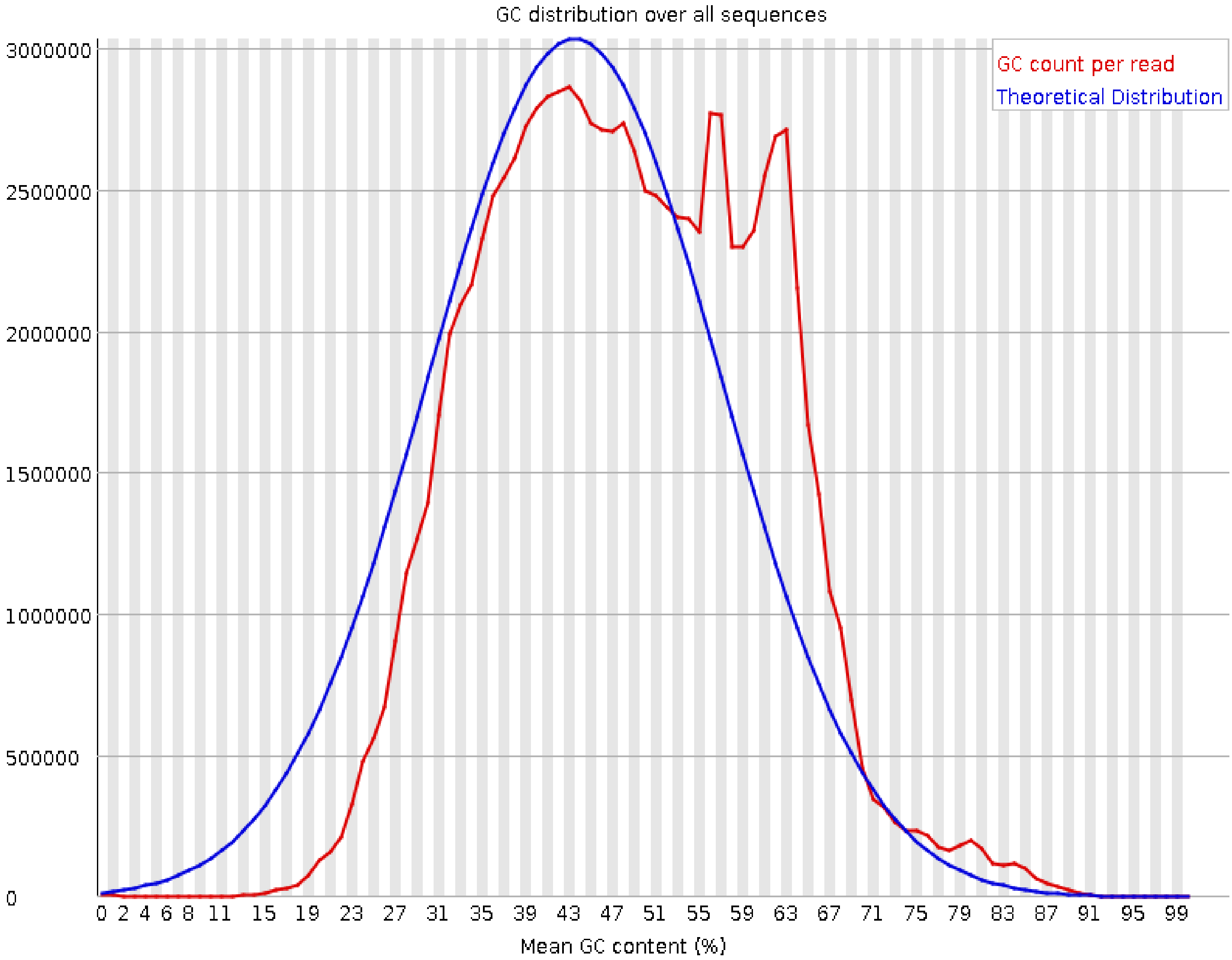


## Per base N content

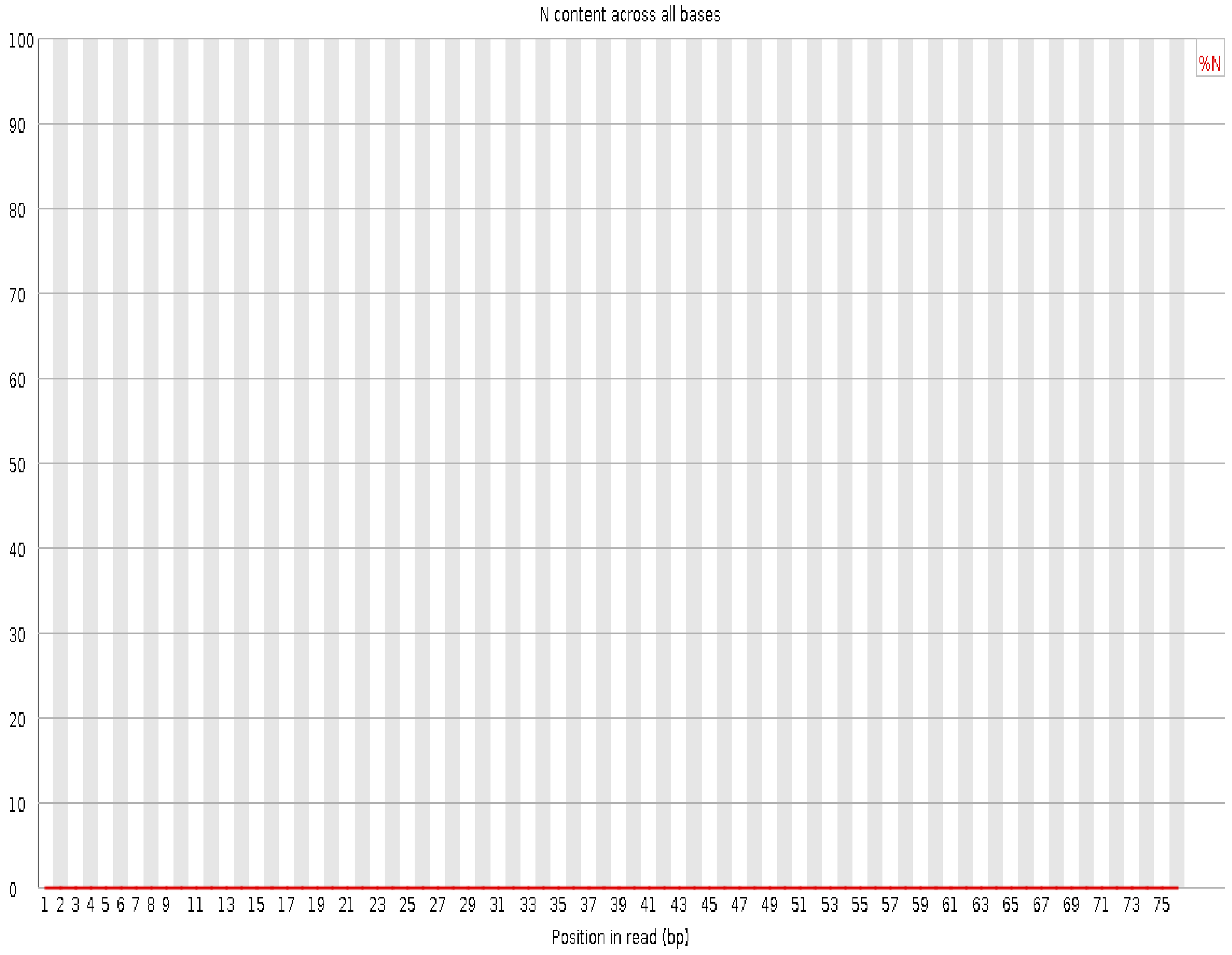


## Sequence Length Distribution

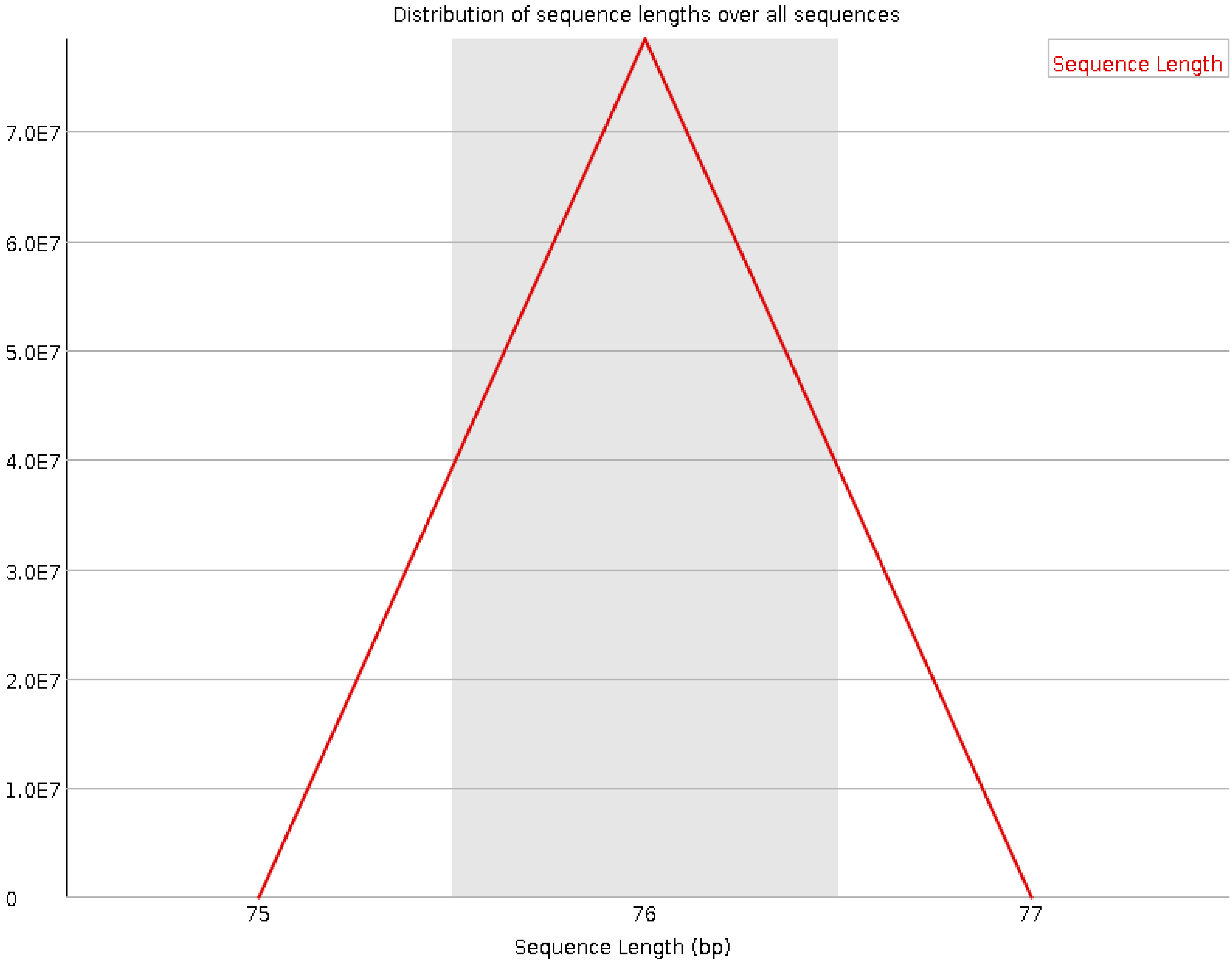


## Sequence Duplication Levels

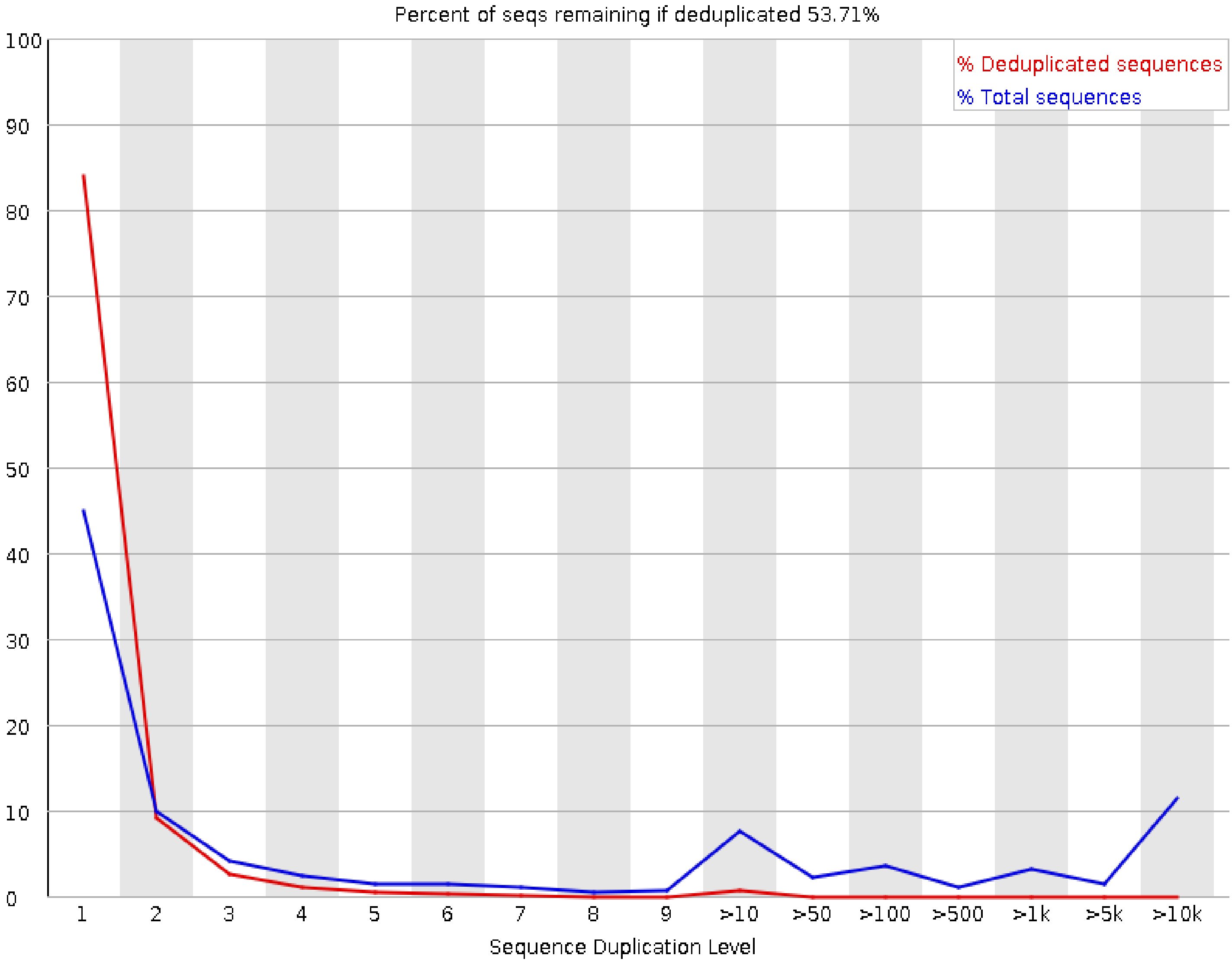


# Overrepresented sequences

| Sequence | Count | Percentage | Possible Source |
|---|---|---|---|
| CCCTCCTTAGGCAACCTGGTGGTCCCCCGCTCCCGGGAGGTCACCATATT | 290 76 | 0.370 41917  442334 | No Hit |
| CTCCGTTTCCGACCTGGGCCGGTTCACCCCTCCTTAGGCAACCTGGTGGT | 271876 | 0.34669 71601 8664 | No Hit |
| CCCCACTACCACAAATTATGCAGTCGAGTTTCCCACATTTGGGGAAATCG | 269308 | 0.3434210076976304 | No Hit |
| CTGGAGTCTTGGAAGCTTGACTACCCTACGTTCTCCTACAAATGGACCTT | 2382 1 | 0.30381718 1744773 | No Hit |
| GGGAGATACCATGATCACGAAGGTGGTTTTCCCAGGGCGAGGCTTATCCA | 2178 8 | 0.277812073 1801784 | No Hit |
| CCCCTCCTTAGGCAACCTGGTGGTCCCCCGCTCCCGGGAGGTCACCATAT | 200949 | 0.2 62497 149 8008 | No Hit |
| CCCCCACTACCACAAATTATGCAGTCGAGTTTCCCACATTTGGGGAAATC | 191903 | 0.2447143109012668 | No Hit |
| CCTCCTTAGGCAACCTGGTGGTCCCCCGCTCCCGGGAGGTCACCATATTG | 17   12 | 0.223 6897860648867 | No Hit |
| CCATGATCACGAAGGTGGTTTTCCCAGGGCGAGGCTTATCCATTGCACTC | 1 7787 | 0.2012096 786974768 | No Hit |
| CTGGCTGCGACATCTGTCACCCCATTGATCGCCAGGGTTGATTCGGCTGA | 1 4470 | 0.19697982629202612 | No Hit |
| GTGCGCTATGCCGATCGGGTGTCCGCACTAAGTTCGGCATCAATATGGTG | 14  22 | 0.18 18   021 0147 | No Hit |

| Sequence | Count | Percentage | Possible Source |
|---|---|---|---|
| CCCACTACCACAAATTATGCAGTCGAGTTTCCCACATTTGGGGAAATCGC | 13 16 | 0.1723621299780998 | No Hit |
| CCAGGCTGGAGTGCAGTGGCTATTCACAGGCGCGATCCCACTACTGATCA | 121233 | 0.1 4 960722 81346 | N[illegible] |
| GTGGCTATTCACAGGCGCGATCCCACTACTGATCAGCACGGGAGTTTTGA | 120647 | 0.1 8488062 787 78 | No Hit |
| CTCAGGCTGGAGTGCAGTGGCTATTCACAGGCGCGATCCCACTACTGATC | 111186 | 0.1417841 8 168978 | o Hit |
| CTCGCTATGTTGCCCAGGCTGGAGTGCAGTGGCTATTCACAGGCGCGATC | 101426 | 0.129338226 908917 | No Hit |
| GTCCGCACTAAGTTCGGCATCAATATGGTGACCTCCCGGGAGCGGGGGAC | 100686 | 0.128394 8011289 | o Hit |
| GTGGCGCGTGCCTGTAGTCCCAGCTACTCGGGAGGCTGAGGTGGGAGGAT | 100396 | 0.1280247727093 62 | No Hit |
| CCTTAGGCAACCTGGTGGTCCCCCGCTCCCGGGAGGTCACCATATTGATG | 92888 | 0.1184 0 86 1 22 | o Hit |
| CTCCTCTATCGGGGATGGTCGTCCTCTTCGACCGAGCGCGCAGCTTCGGG | 87181 | 0.111173031889461 7 | No Hit |
| CTGGAGTGCAGTGGCTATTCACAGGCGCGATCCCACTACTGATCAGCACG | 86894 | 0.1108070 00797 2 | o Hit |
| AGGGGAGATACCATGATCACGAAGGTGGTTTTCCCAGGGCGAGGCTTATC | 8 031 | 0.1084313 631149912 | No Hit |
| GGGGAGATACCATGATCACGAAGGTGGTTTTCCCAGGGCGAGGCTTATCC | 83 66 | 0.106 631913246 496 | No Hit |

# Adapter Content

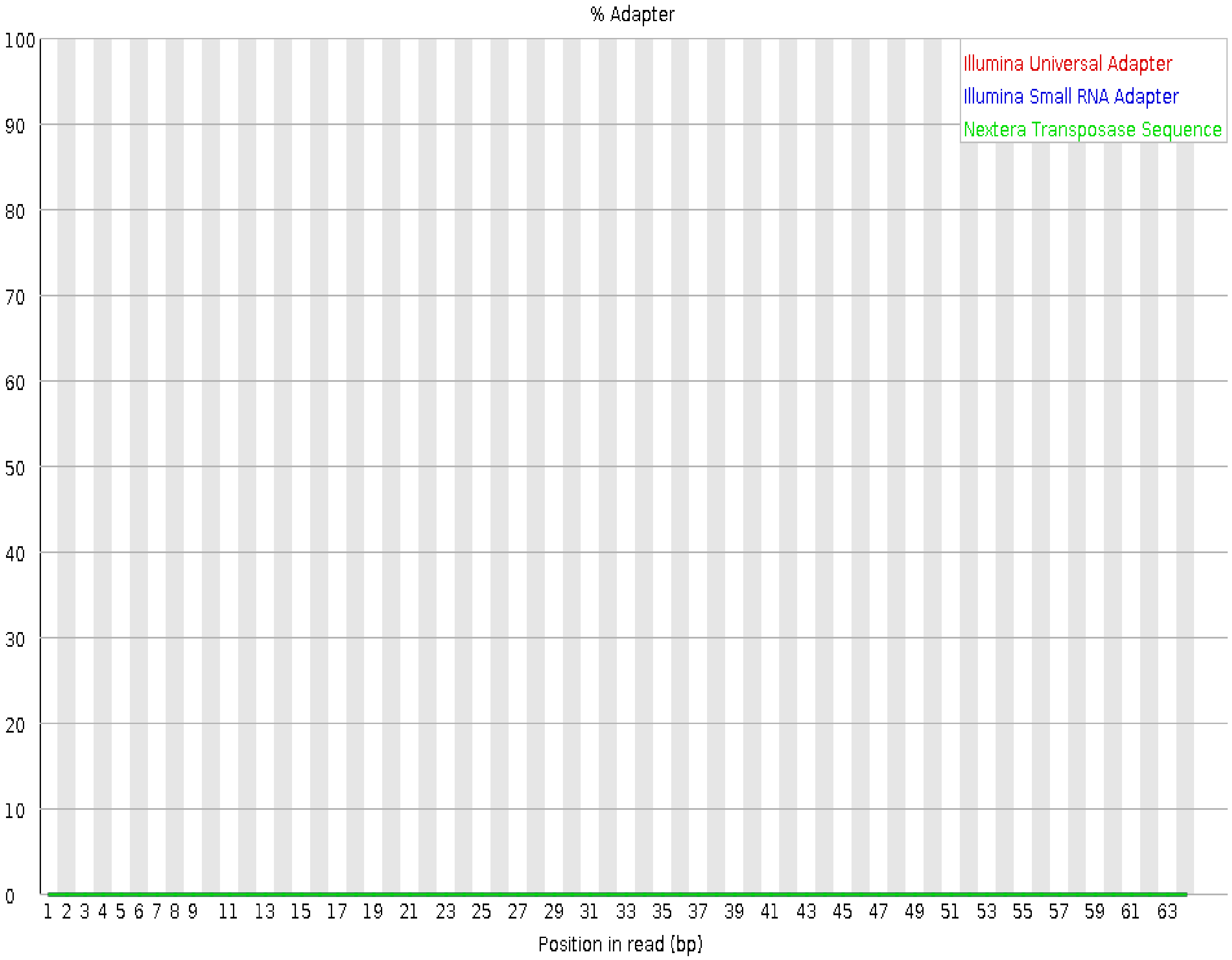

# Kmer Content

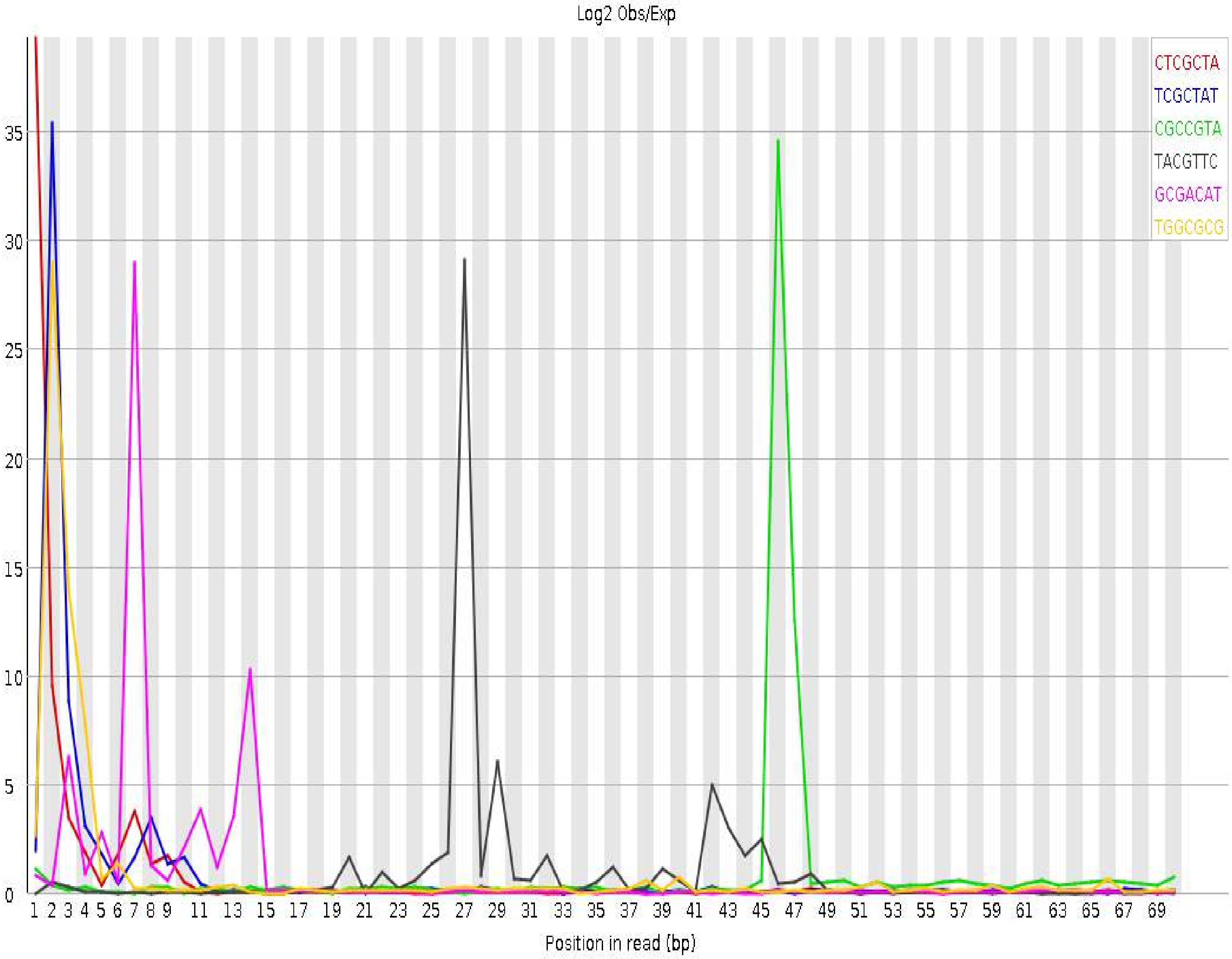


| Sequence | Count | PValue | Obs/Exp Max | Max Obs/Exp Position |
|---|---|---|---|---|
| CTCGCTA | 34660 | 0.0 | 39.2 941 | 1 |
| TCGCTAT | 38630 | 0.0 | 3 . 6926 | 2 |
| CGCCGTA | 103 | 0.0 | 34. 7313 | 46 |
| TACGTTC | 60490 | 0.0 | 29.083128 | 27 |
| GCGACAT | 4049 | 0.0 | 28.9 9808 | 7 |
| TGGCGCG | 4712 | 0.0 | 28.93872 | 2 |
| TGCGACA | 40 | 0.0 | 28.848 | 6 |
| CCTACGT | 6176 | 0.0 | 28. 1244 | 2 |
| CCCTACG | 62060 | 0.0 | 28.430347 | 24 |
| CTACGTT | 62180 | 0.0 | 28.366806 | 26 |
| GCTGCGA | 41 | 0.0 | 28.2228 7 | 4 |
| GGGCGAT | 1 0 | 0.0 | 28.087072 | 1 |
| | | | | |

| Sequence | Count | PValue | Obs/Exp Max | Max Obs/Exp Position |
|---|---|---|---|---|
| CTAGCAG | 63370 | 0.0 | 27.8078 8 | 70 |
| [illegible] | [illegible] | [illegible] | 27.380703 | [illegible] |
| GCCGTAT | 13290 | 0.0 | 27.363188 | 47 |
| GCGTCGT | 14 80 | 0.0 | 27.31 [illegible]8 | 11 |
| CTGCGAC | 4307 | 0.0 | 27.22 | |
| TCTAGCA | 6 660 | 0.0 | 26.8 273 | 69 |
| GGTTCTA | 6491 | 0.0 | 26.766 8 | 66 |
| ACGTTCT | 680 0 | 0.0 | 2 9396 | 82 |

**Produced by FastQC (version 0.11.2)**

# FastQC Report

Mon 4 May 2015
Biochain_Fetal_Stomach_GTGAAA_L006_R1.fastq.gz

## Summary


- Basic Statistics
- Per base sequence quality
- Per tile sequence quality
- Per sequence quality scores
- Per base sequence content
- Per sequence GC content
- Per base N content
- Sequence Length Distribution
- Sequence Duplication Levels
- Overrepresented sequences
- Adapter Content
- Kmer Content


## Basic Statistics

| Measure | Value |
|---|---|
| Filename | Biochain_Fetal_Stomach_GTGAAA_L006_R1.fastq.gz |
| File type | Conventional base calls |
| Encoding | Sanger / Illumina 1.9 |
| Total Sequences | 81875607 |
| Sequences flagged as poor quality | 0 |
| Sequence length | 76 |
| %GC | 45 |

## Per base sequence quality

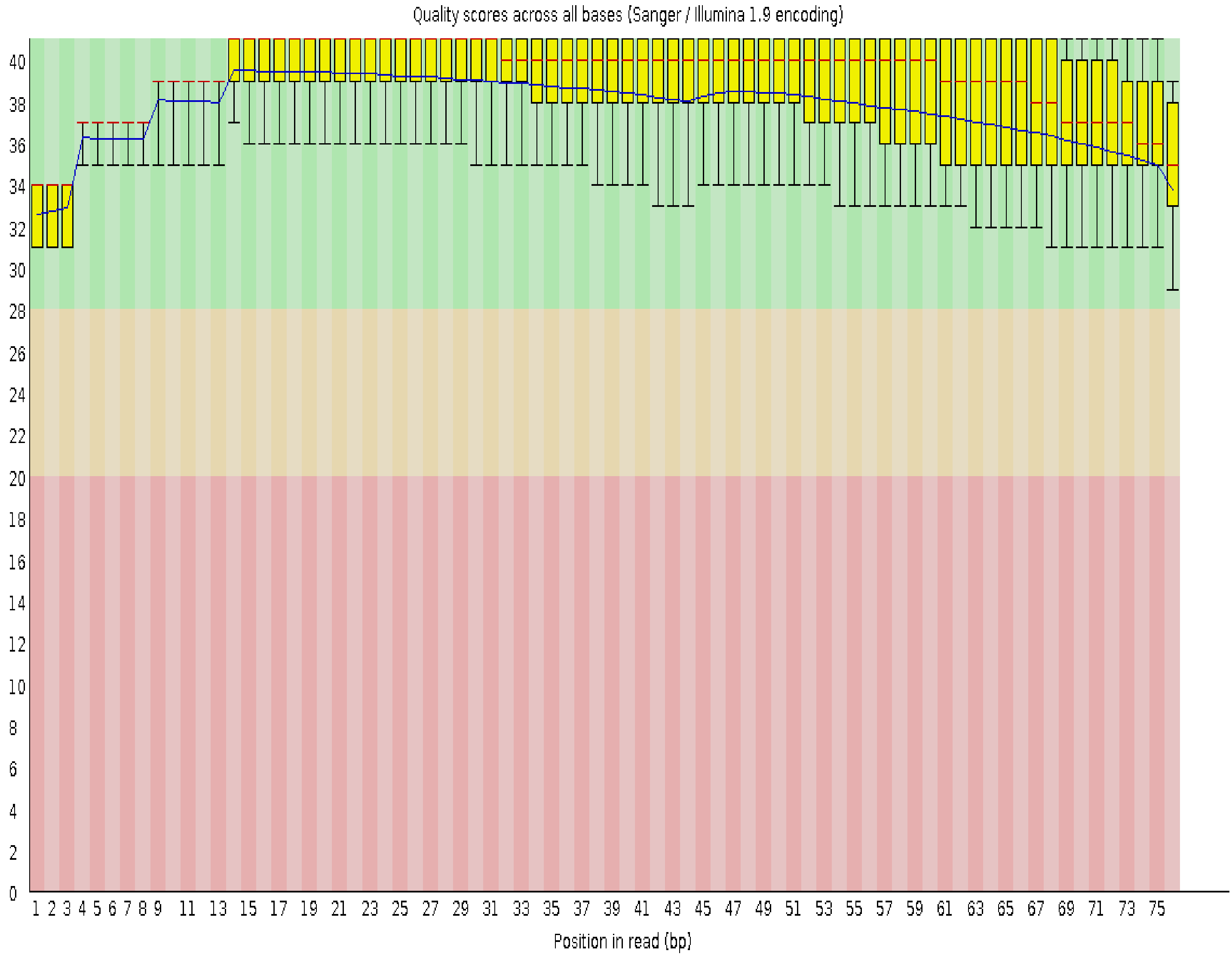


## Per tile sequence quality

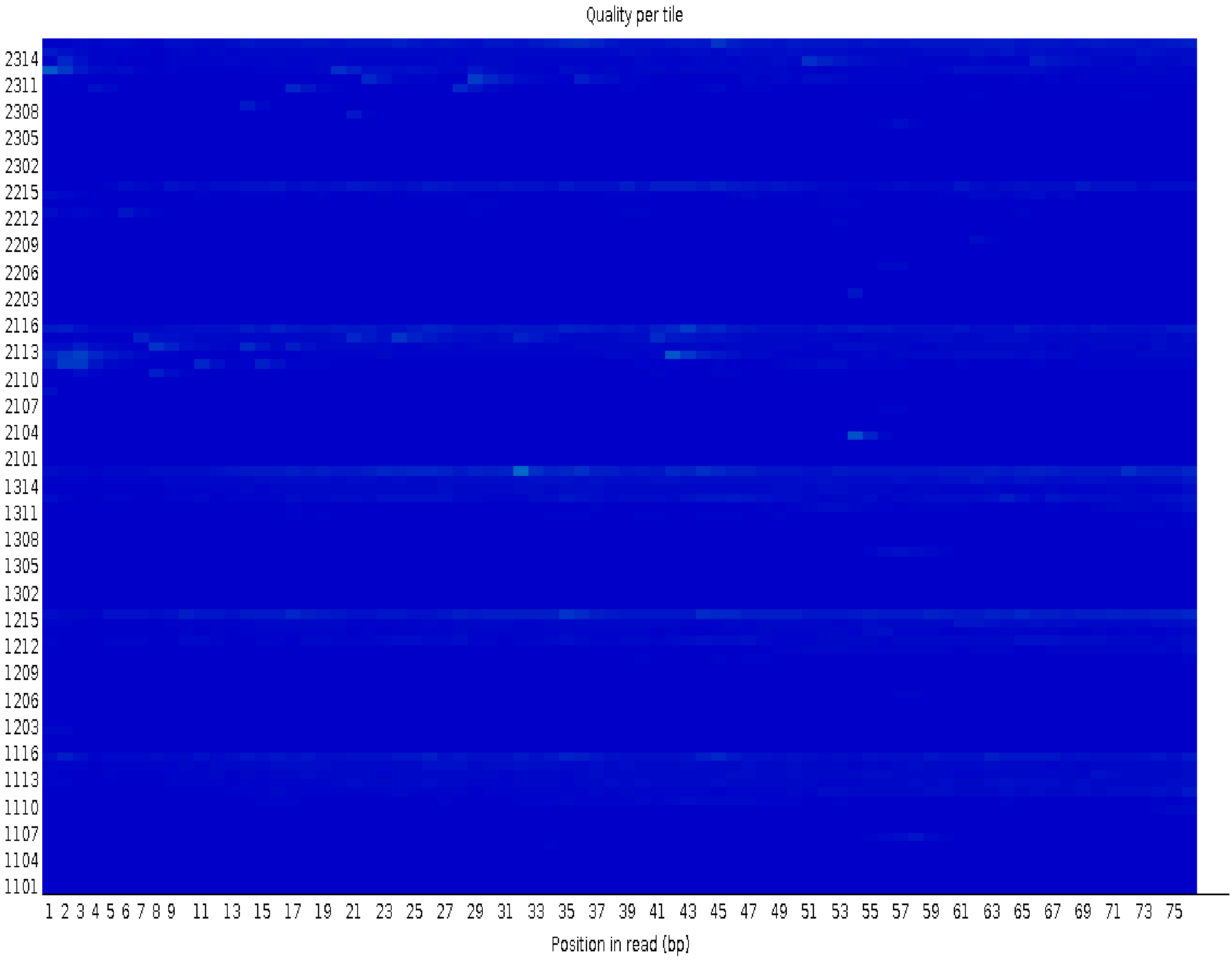


## Per sequence quality scores

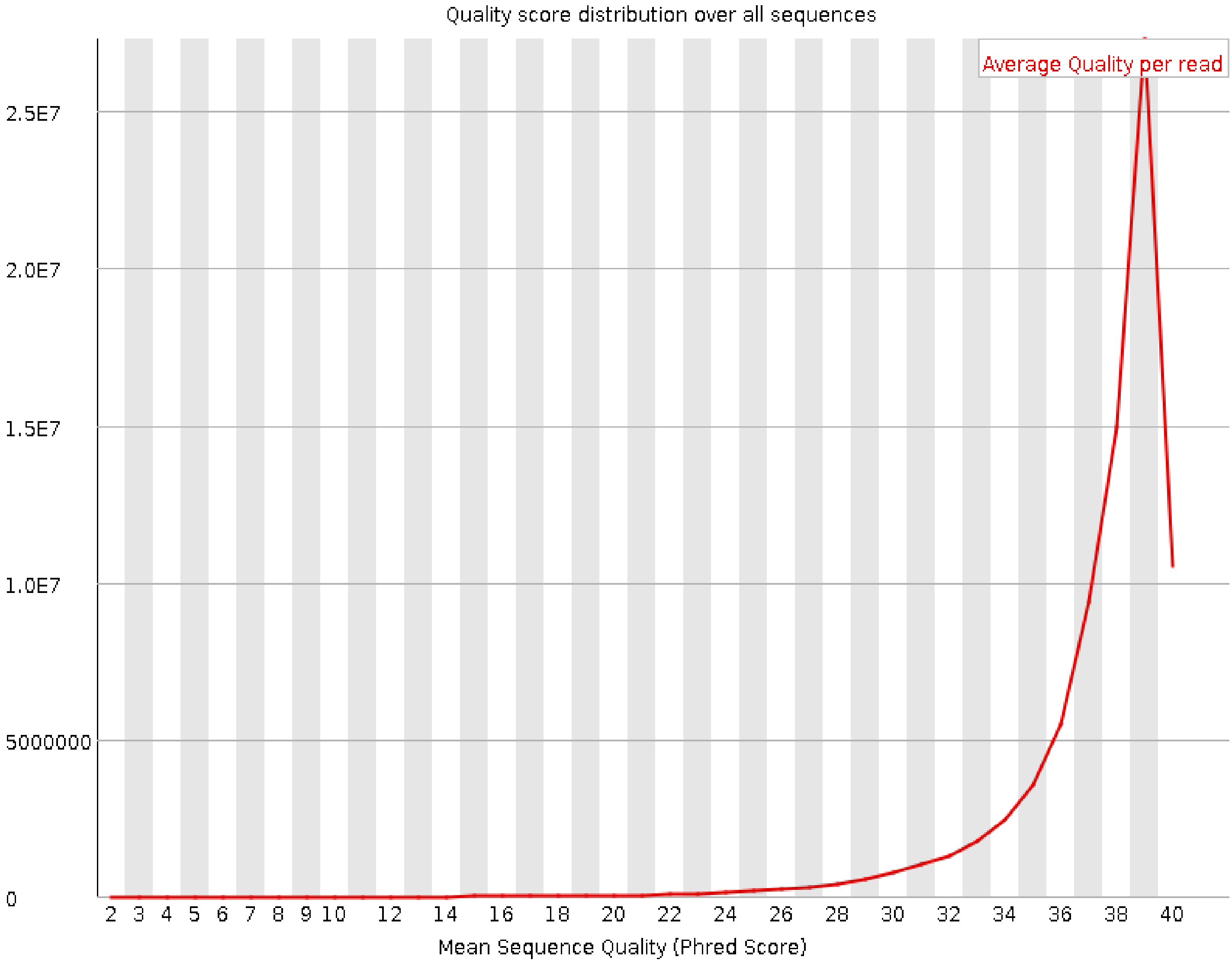


## Per base sequence content

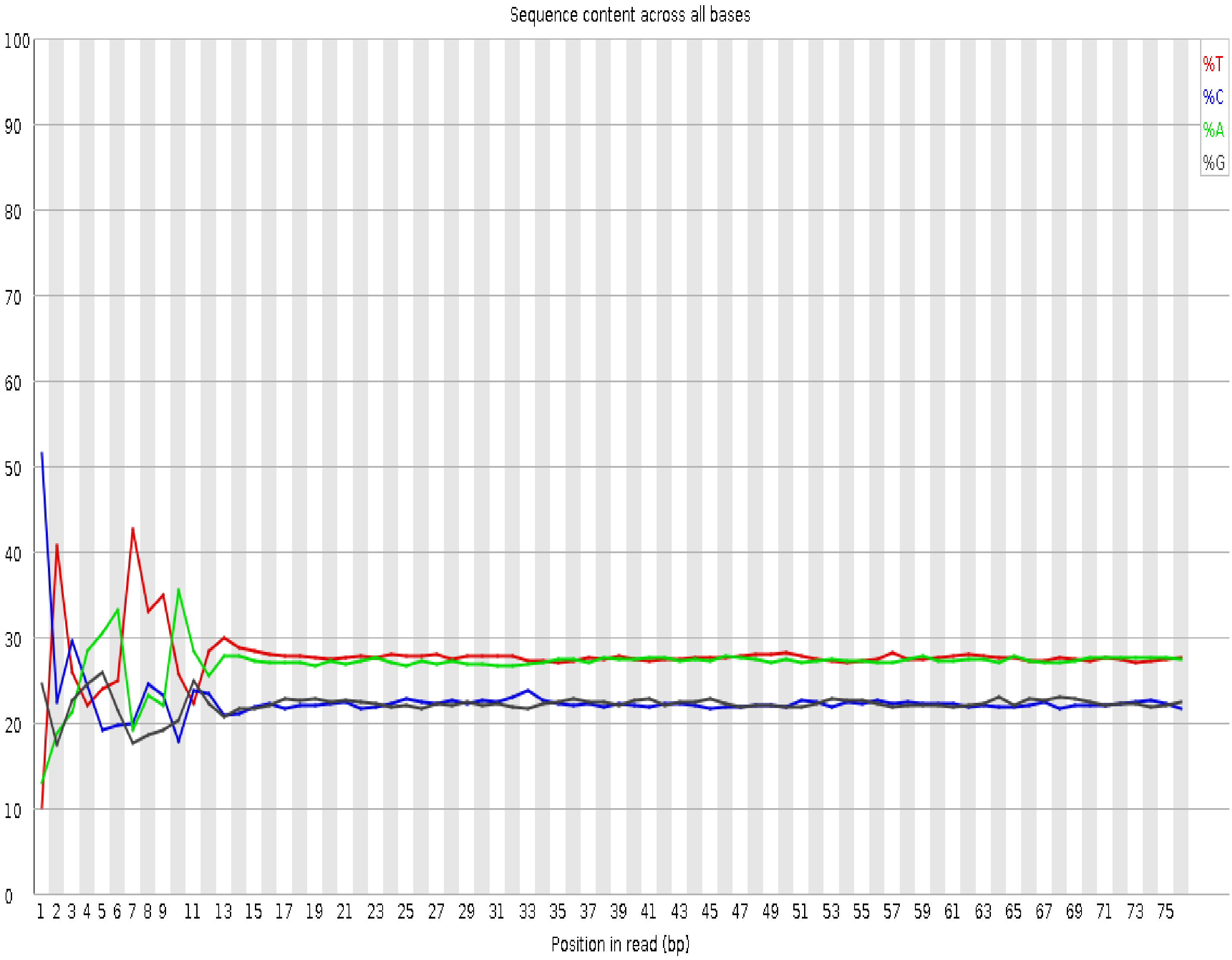


# Per sequence GC content

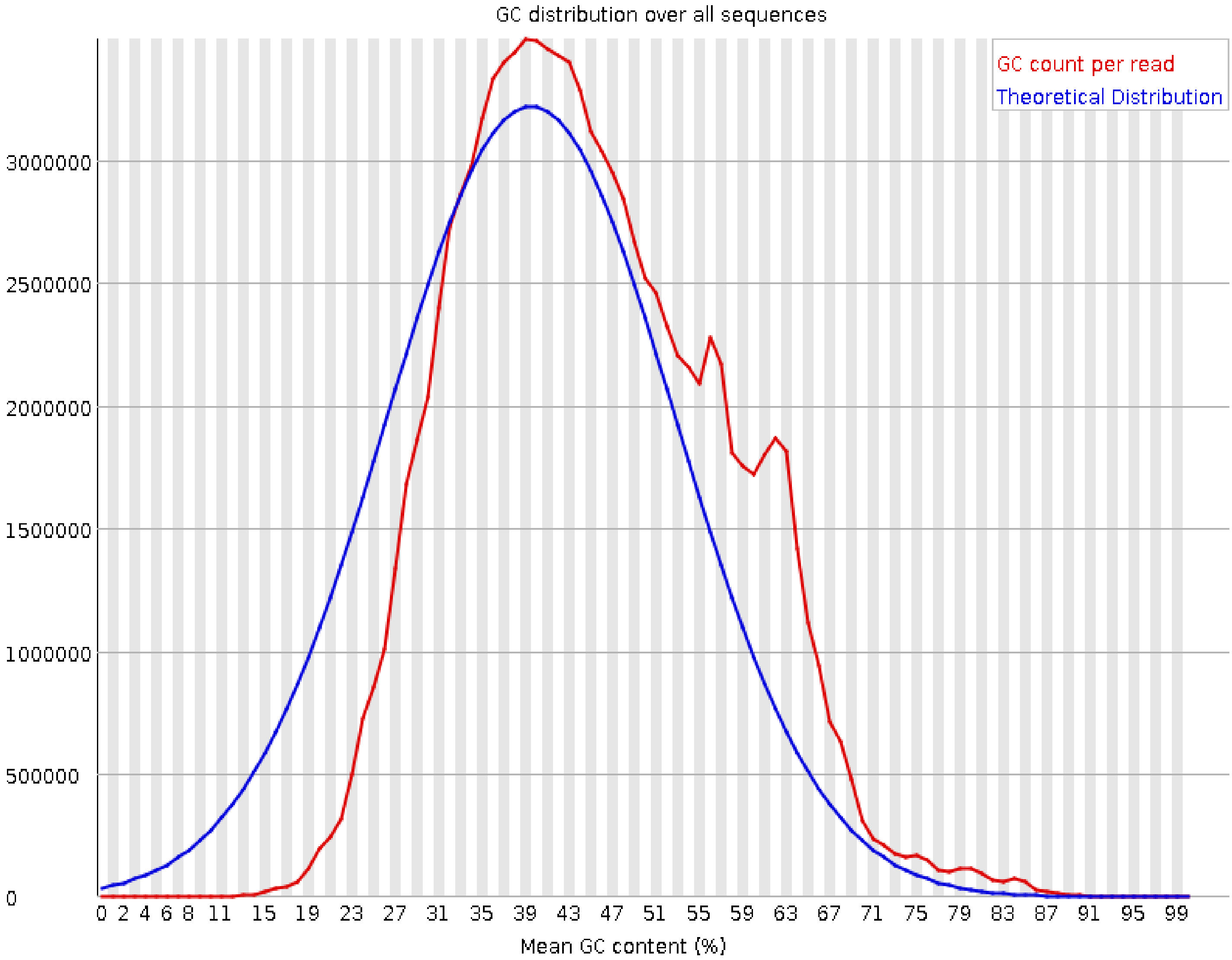


## Per base N content

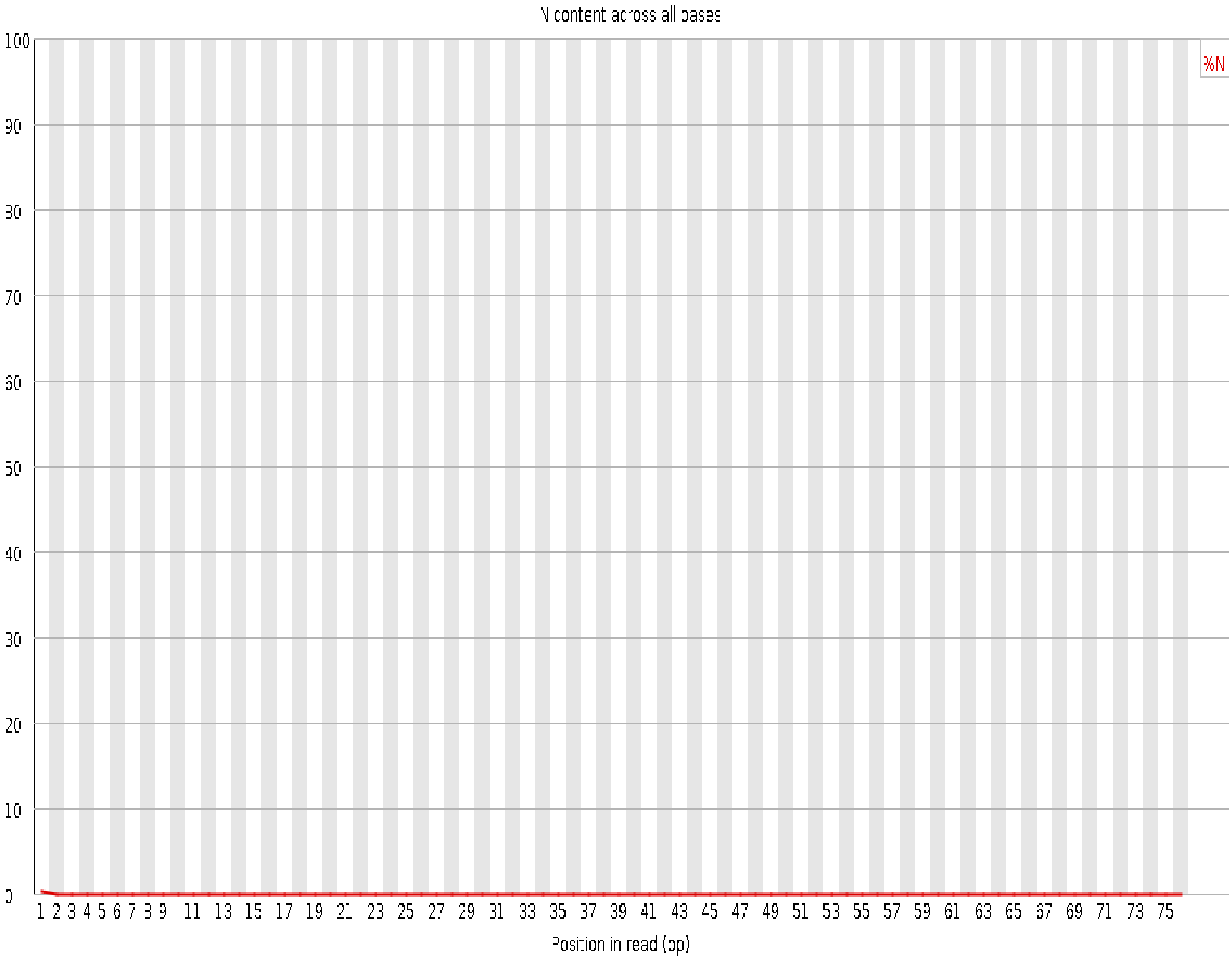


## Sequence Length Distribution

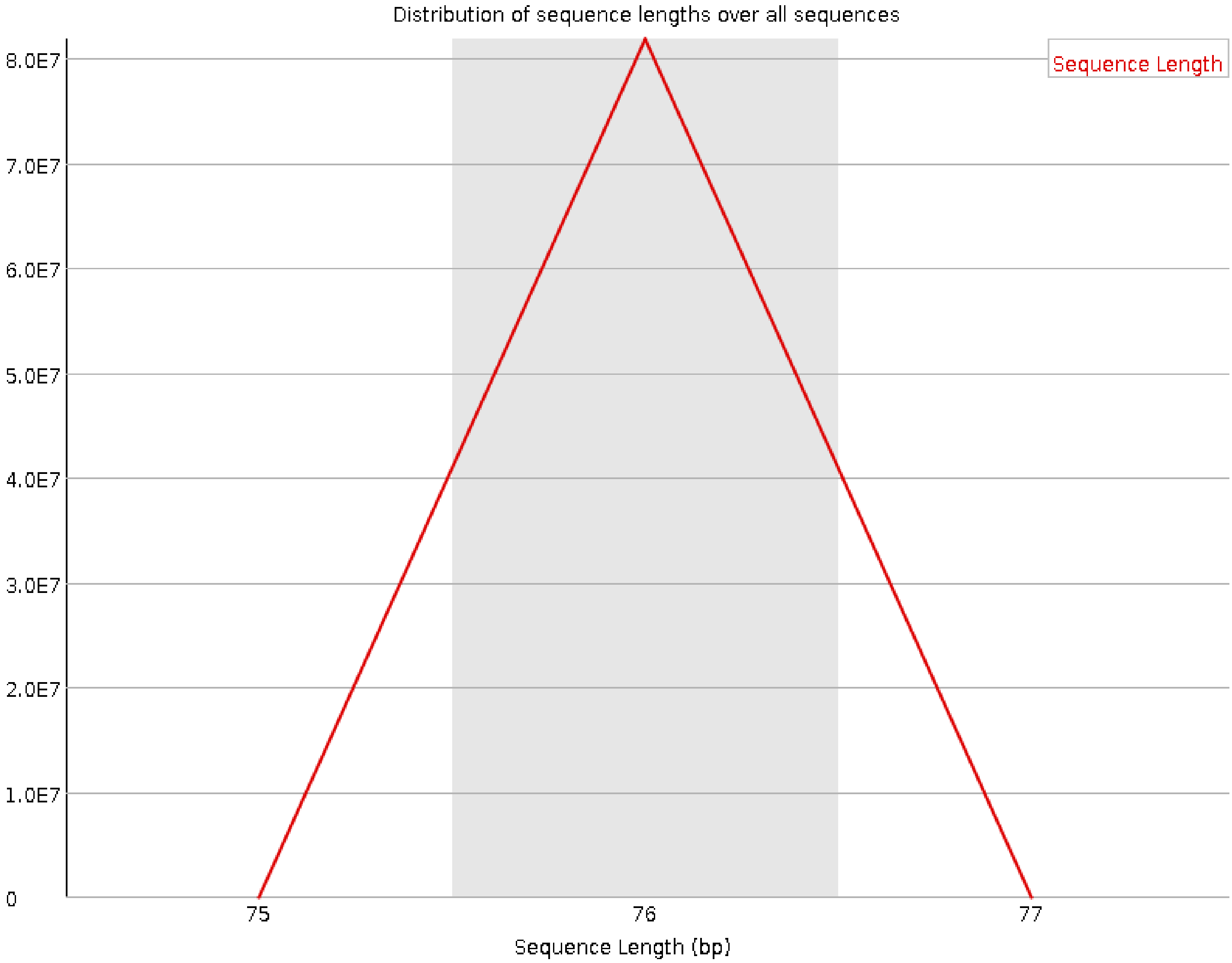


## Sequence Duplication Levels

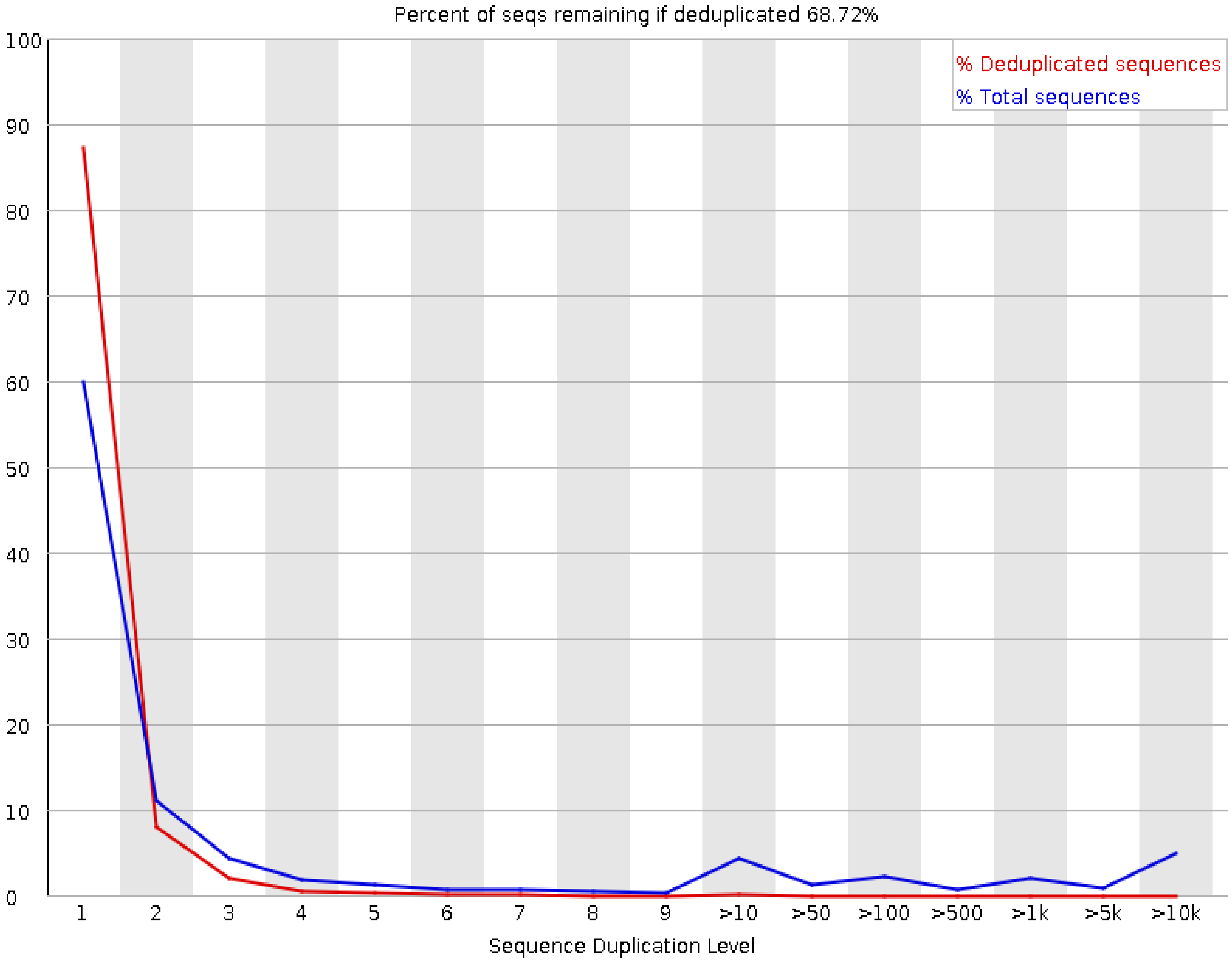


## Overrepresented sequences

| Sequence | Count | Percentage | Possible Source |
|---|---|---|---|
| CTGGAGTCTTGGAAGCTTGACTACCCTACGTTCTCCTACAAATGGACCTT | 173946 | 0.21245155471030586 | No Hit |
| CCCTCCTTAGGCAACCTGGTGGTCCCCCGCTCCCGGGAGGTCACCATATT | 160347 | 0.19584221219880543 | No Hit |
| CCCCACTACCACAAATTATGCAGTCGAGTTTCCCACATTTGGGGAAATCG | 157254 | 0.19206453028189457 | No Hit |
| GGGAGATACCATGATCACGAAGGTGGTTTTCCCAGGGCGAGGCTTATCCA | 135724 | 0.16576854202741972 | No Hit |
| CTCCGTTTCCGACCTGGGCCGGTTCACCCCTCCTTAGGCAACCTGGTGGT | 133080 | 0.16253925299143127 | No Hit |
| CTGGCTGCGACATCTGTCACCCCATTGATCGCCAGGGTTGATTCGGCTGA | 115694 | 0.14130460125932257 | No Hit |
| CCCCTCCTTAGGCAACCTGGTGGTCCCCCGCTCCCGGGAGGTCACCATAT | 106018 | 0.12948667360719535 | No Hit |
| CCCCCACTACCACAAATTATGCAGTCGAGTTTCCCACATTTGGGGAAATC | 105700 | 0.1290982795400833 | No Hit |
| CCATGATCACGAAGGTGGTTTTCCCAGGGCGAGGCTTATCCATTGCACTC | 101401 | 0.12384763144412474 | No Hit |
| CCCACTACCACAAATTATGCAGTCGAGTTTCCCACATTTGGGGAAATCGC | 90788 | 0.11088528479550692 | No Hit |
| CCTCCTTAGGCAACCTGGTGGTCCCCCGCTCCCGGGAGGTCACCATATTG | 87884 | 0.10733844086187964 | No Hit |

| Sequence | Count | Percentage | Possible Source |
| --- | --- | --- | --- |

## Adapter Content

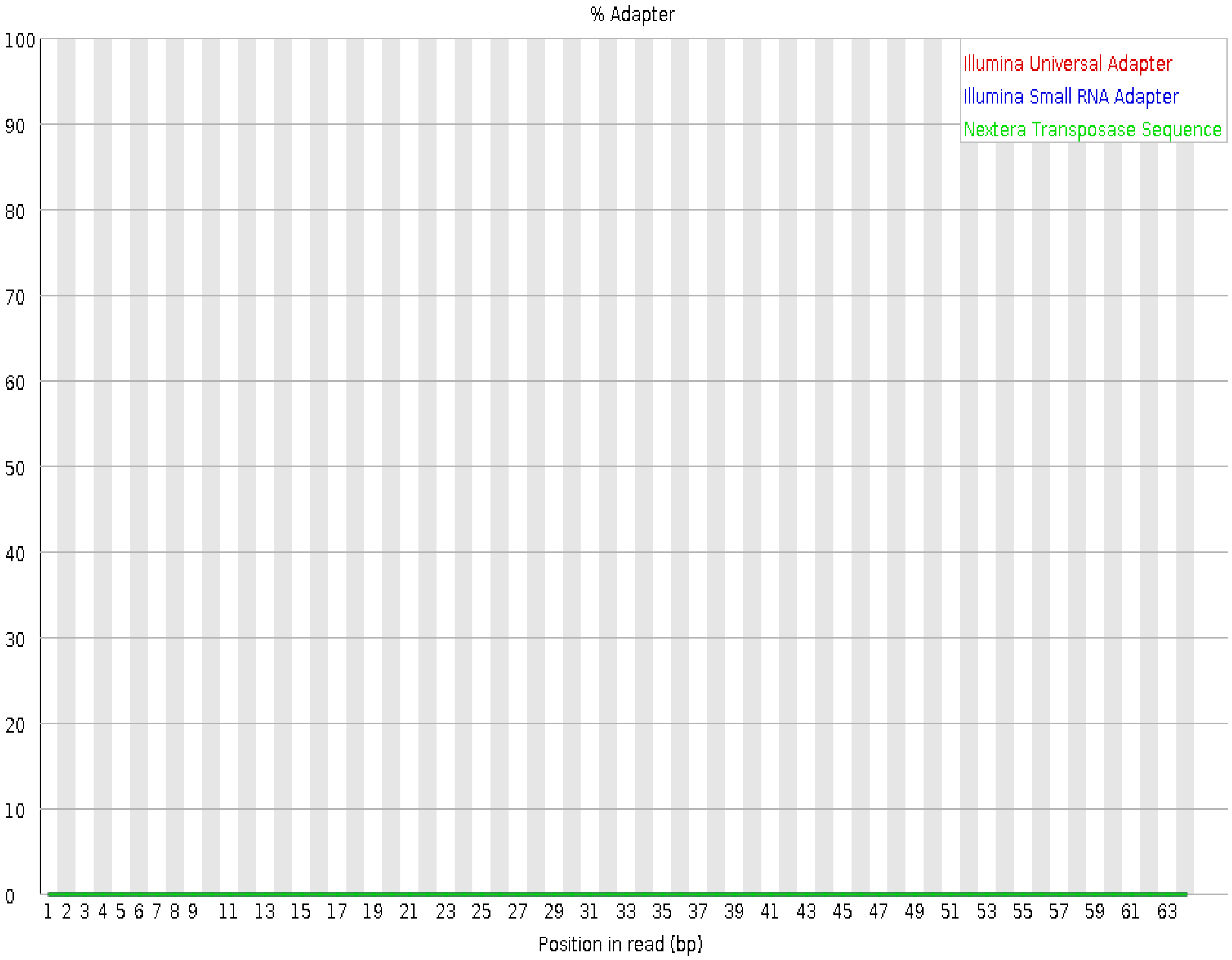


## Kmer Content

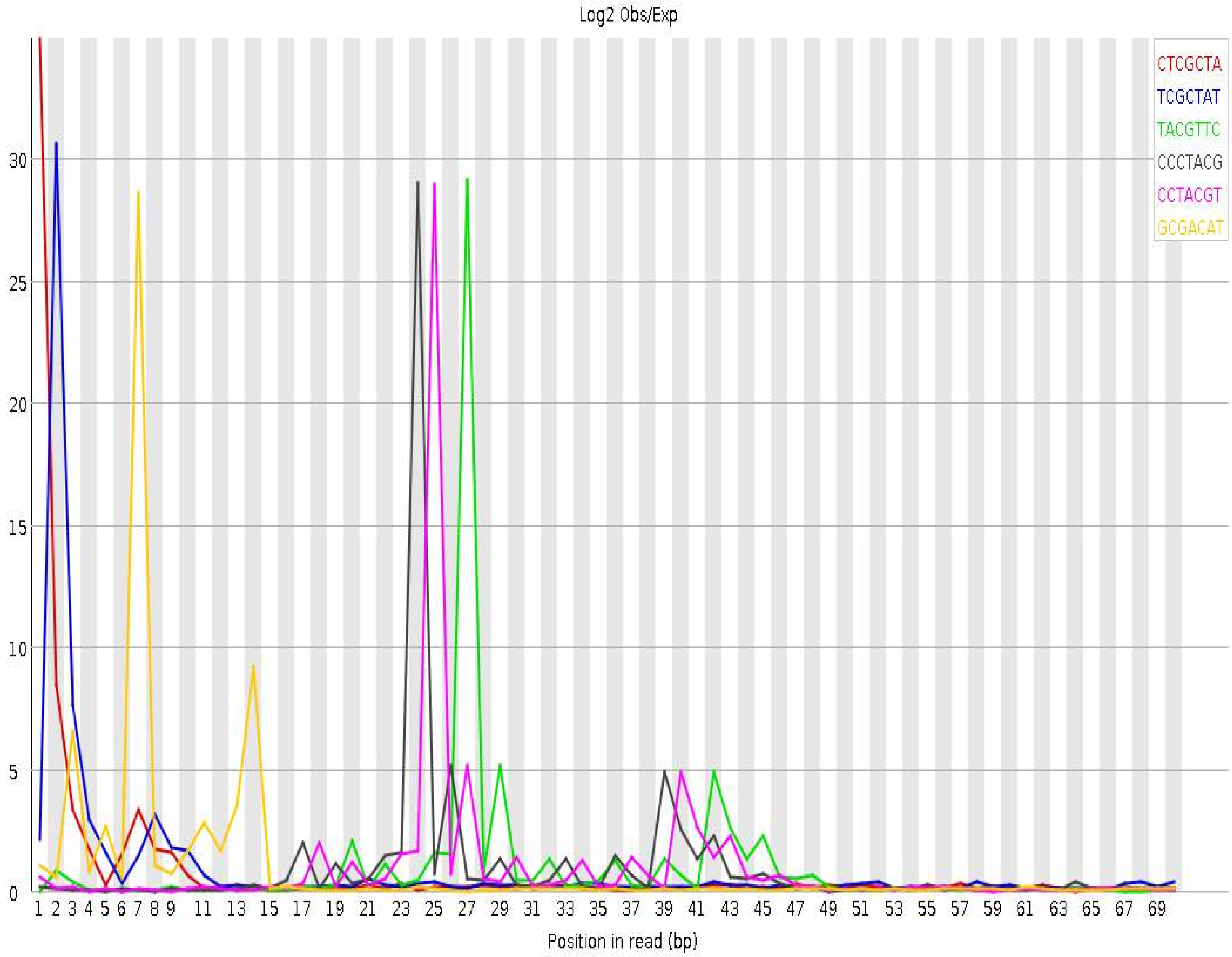


| Sequence | Count | PValue | Obs/Exp Max | Max Obs/Exp Position |
|---|---|---|---|---|
| CTCGCTA | 17890 | 0.0 | 34.884705 | 1 |
| TCGCTAT | 20300 | 0.0 | 30.615255 | 2 |
| TACGTTC | 43515 | 0.0 | 29.174522 | 27 |
| CCCTACG | 43835 | 0.0 | 28.98968 | 24 |
| CCTACGT | 43820 | 0.0 | 28.96875 | 25 |
| GCGACAT | 29085 | 0.0 | 28.623621 | 7 |
| CTACGTT | 44740 | 0.0 | 28.407005 | 26 |
| TGCGACA | 29575 | 0.0 | 28.11342 | 6 |
| TGGCGCG | 27165 | 0.0 | 27.348356 | 2 |
| GCTGCGA | 30525 | 0.0 | 27.318321 | 4 |
| GGCGCGT | 27215 | 0.0 | 27.29778 | 3 |
| CTGCGAC | 31560 | 0.0 | 26.44499 | 5 |
| CGACATC | 32980 | 0.0 | 25.359835 | 8 |
| GGGCGAT | 11180 | 0.0 | 24.938963 | 1 |
| ACGTTCT | 51140 | 0.0 | 24.927254 | 28 |

| Sequence | Count | PValue | Obs/Exp Max | Max Obs/Exp Position |
|---|---|---|---|---|
| GACTACC | 51505 | 0.0 | 24.[illegible] | 19 |
| ACTACCC | 53245 | 0.0 | 24.08[illegible] | 20 |
| ACCCTAC | 54830 | 0.0 | 23.463099 | 23 |
| TACCCTA | 55030 | 0.0 | 23.377254 | 22 |
| GGTTCTA | 55210 | 0.0 | 23.072493 | 66 |

**Produced by FastQC (version 0.11.2)**

# FastQC Report

Mon 4 May 2015
Biochain_Fetal_Stomach_GTGAAA_L006_R2.fastq.gz

## Summary


- Basic Statistics
- Per base sequence quality
- Per tile sequence quality
- Per sequence quality scores
- Per base sequence content
- Per sequence GC content
- Per base N content
- Sequence Length Distribution
- Sequence Duplication Levels
- Overrepresented sequences
- Adapter Content
- Kmer Content


## Basic Statistics

| Measure | Value |
|---|---|
| Filename | Biochain_Fetal_Stomach_GTGAAA_L006_R2.fastq.gz |
| File type | Conventional base calls |
| Encoding | Sanger / Illumina 1.9 |
| Total Sequences | 81875607 |
| Sequences flagged as poor quality | 0 |
| Sequence length | 76 |
| %GC | 45 |

## Per base sequence quality

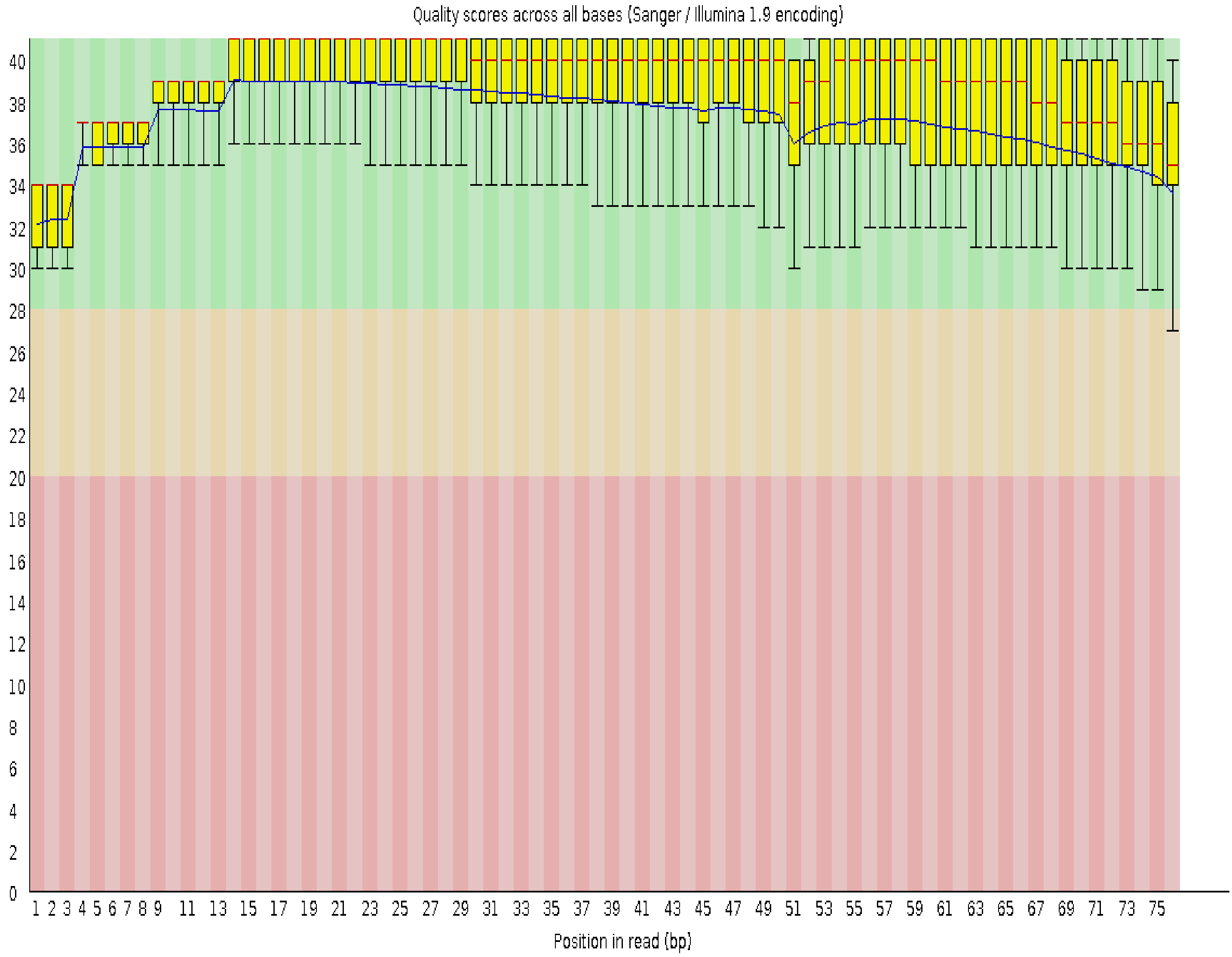


## Per tile sequence quality

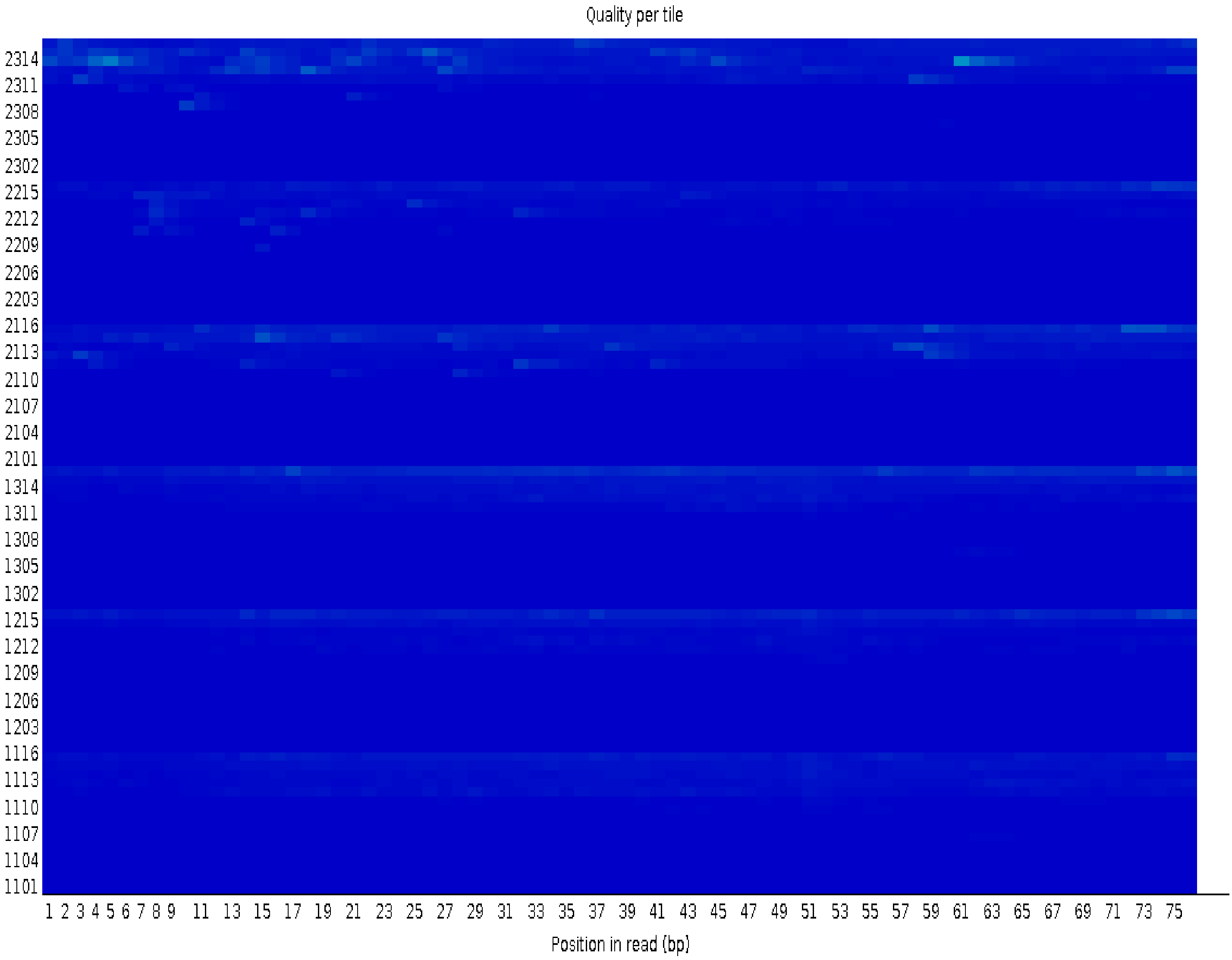


## Per sequence quality scores

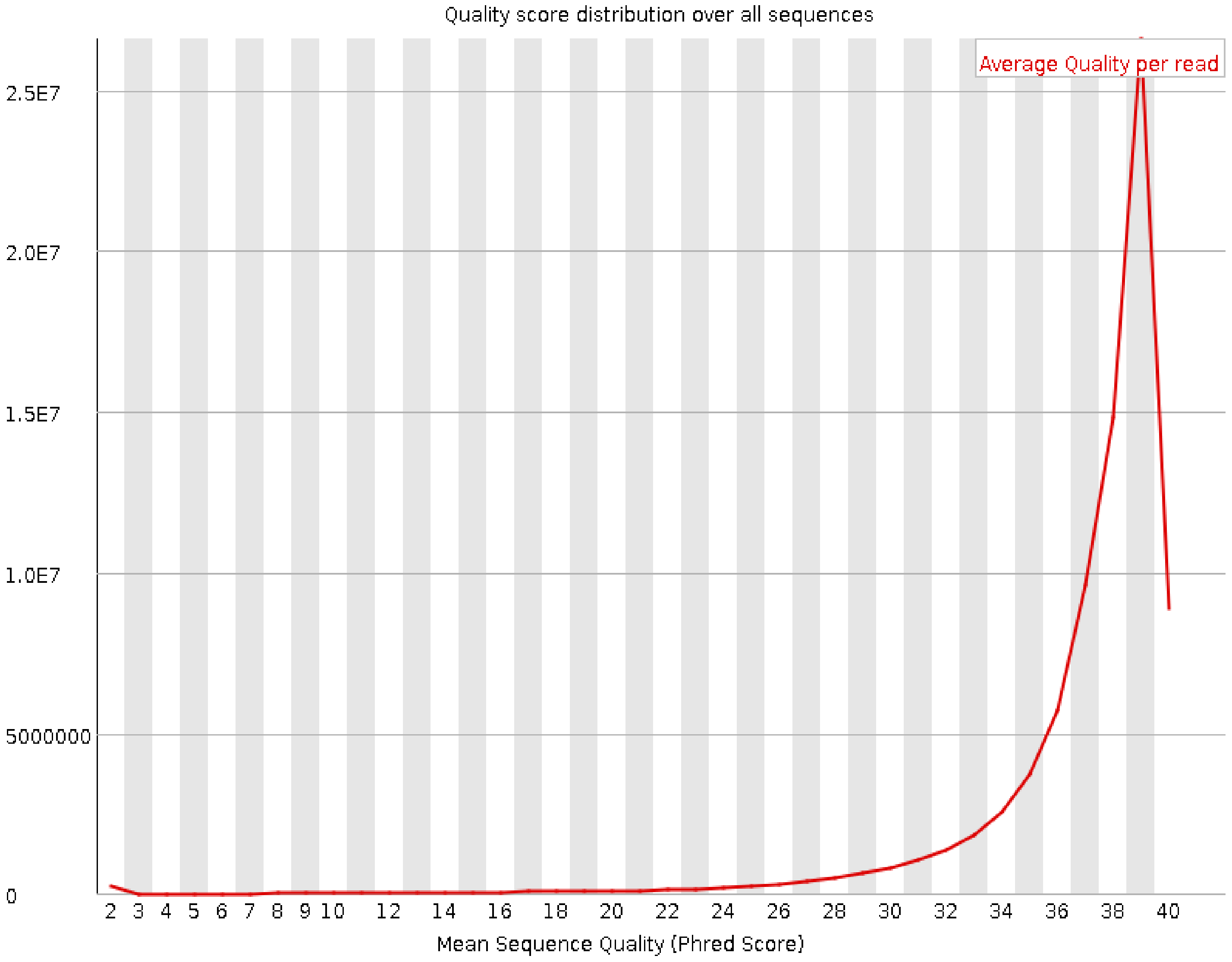


## Per base sequence content

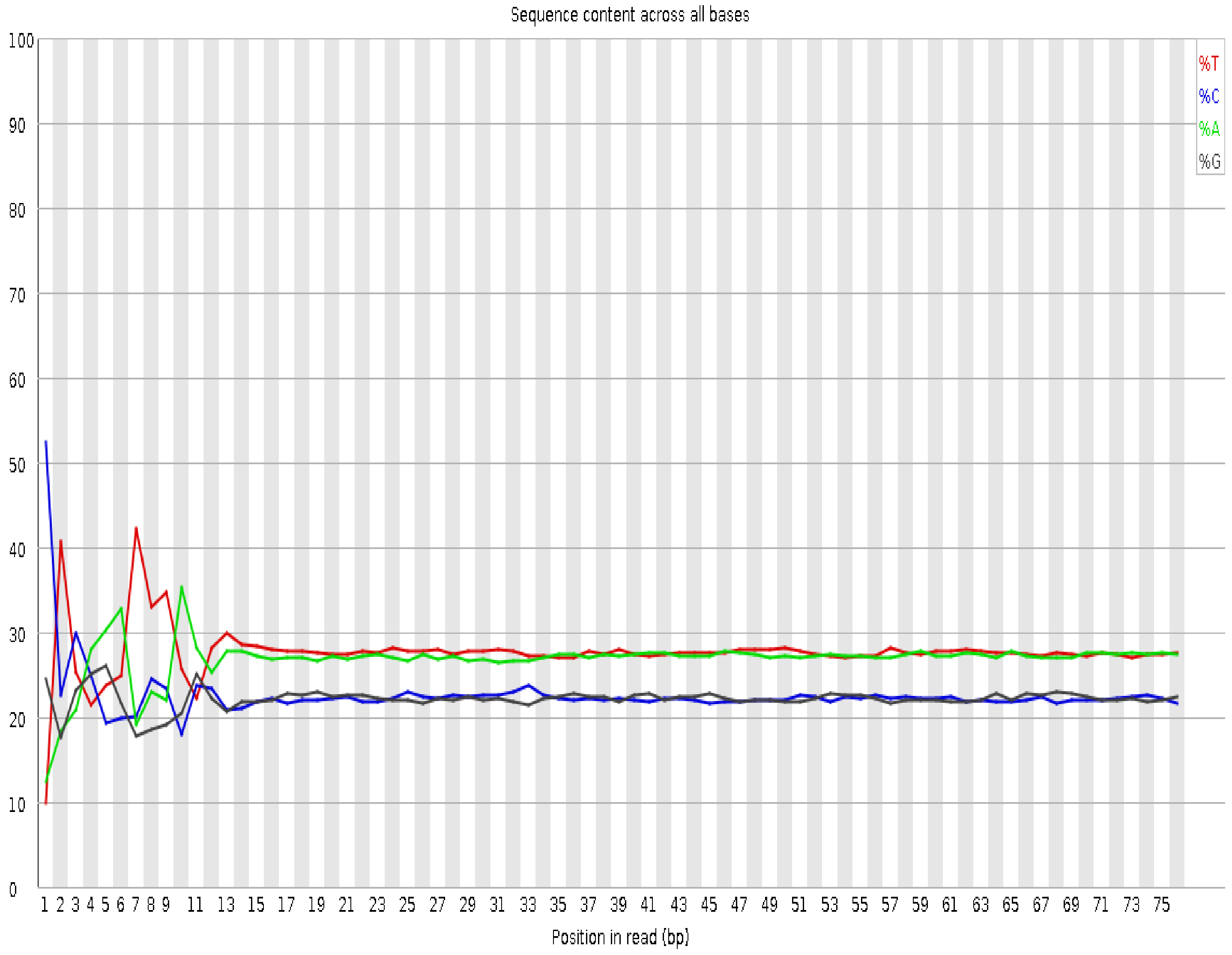


## Per sequence GC content

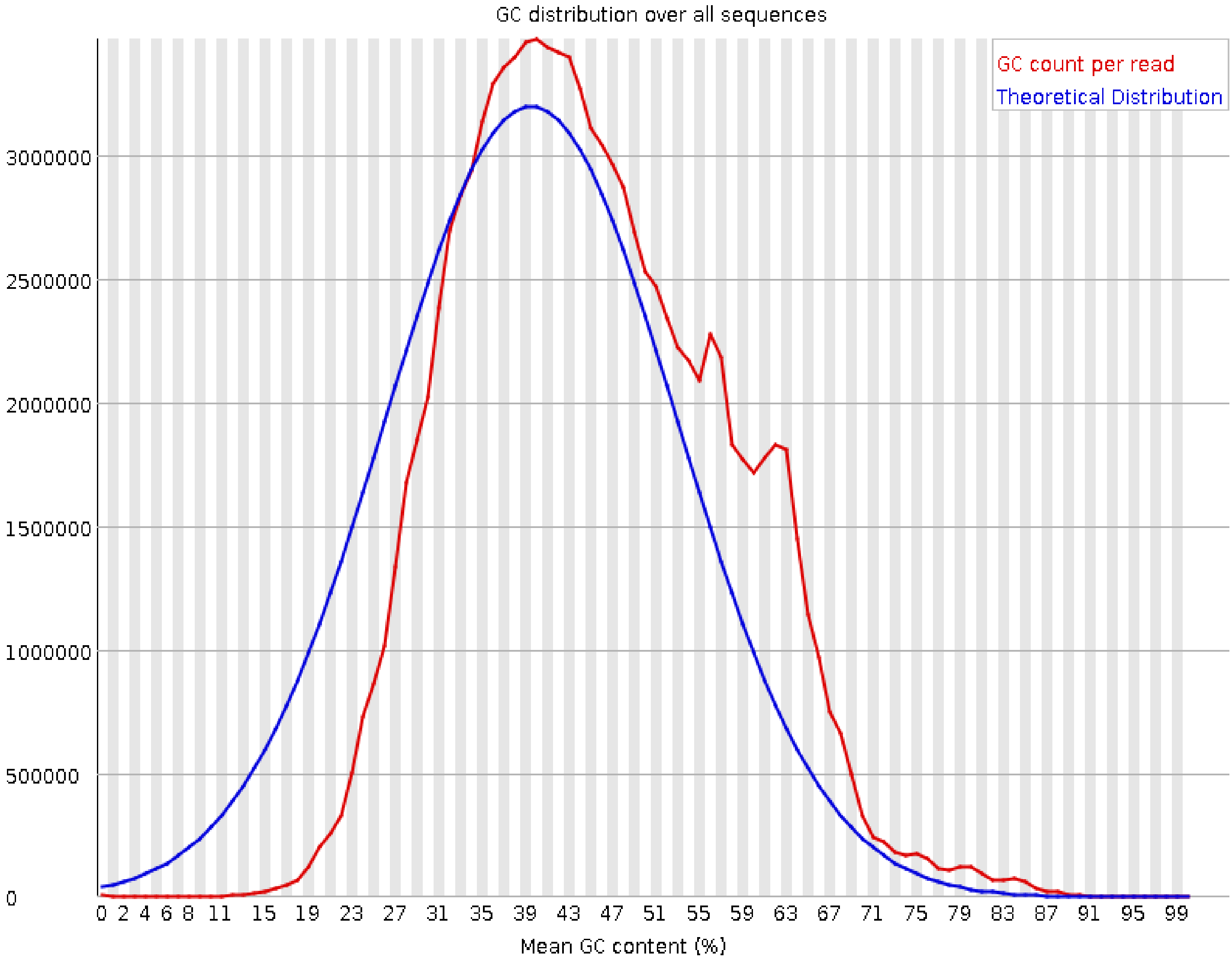


## Per base N content

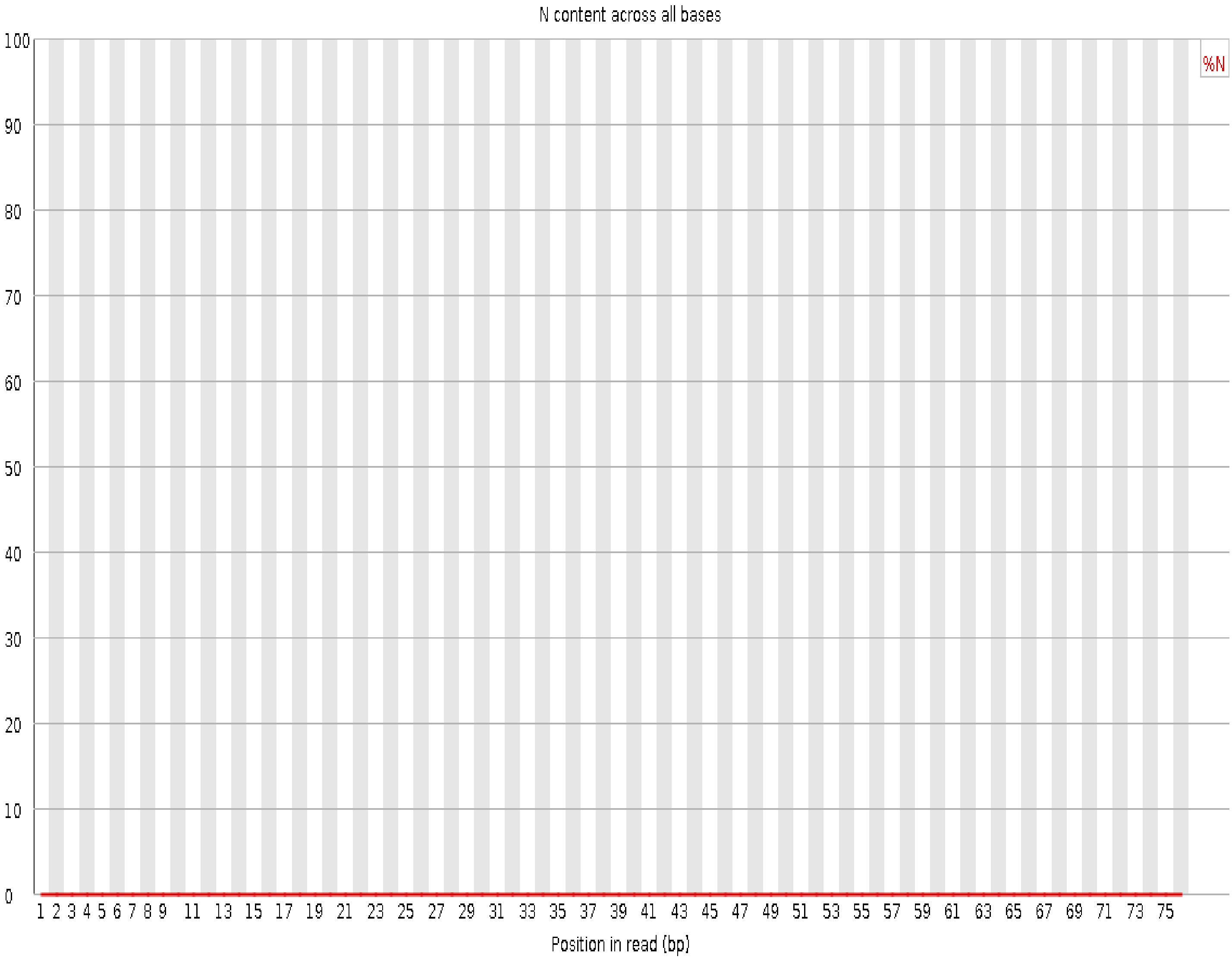


## Sequence Length Distribution

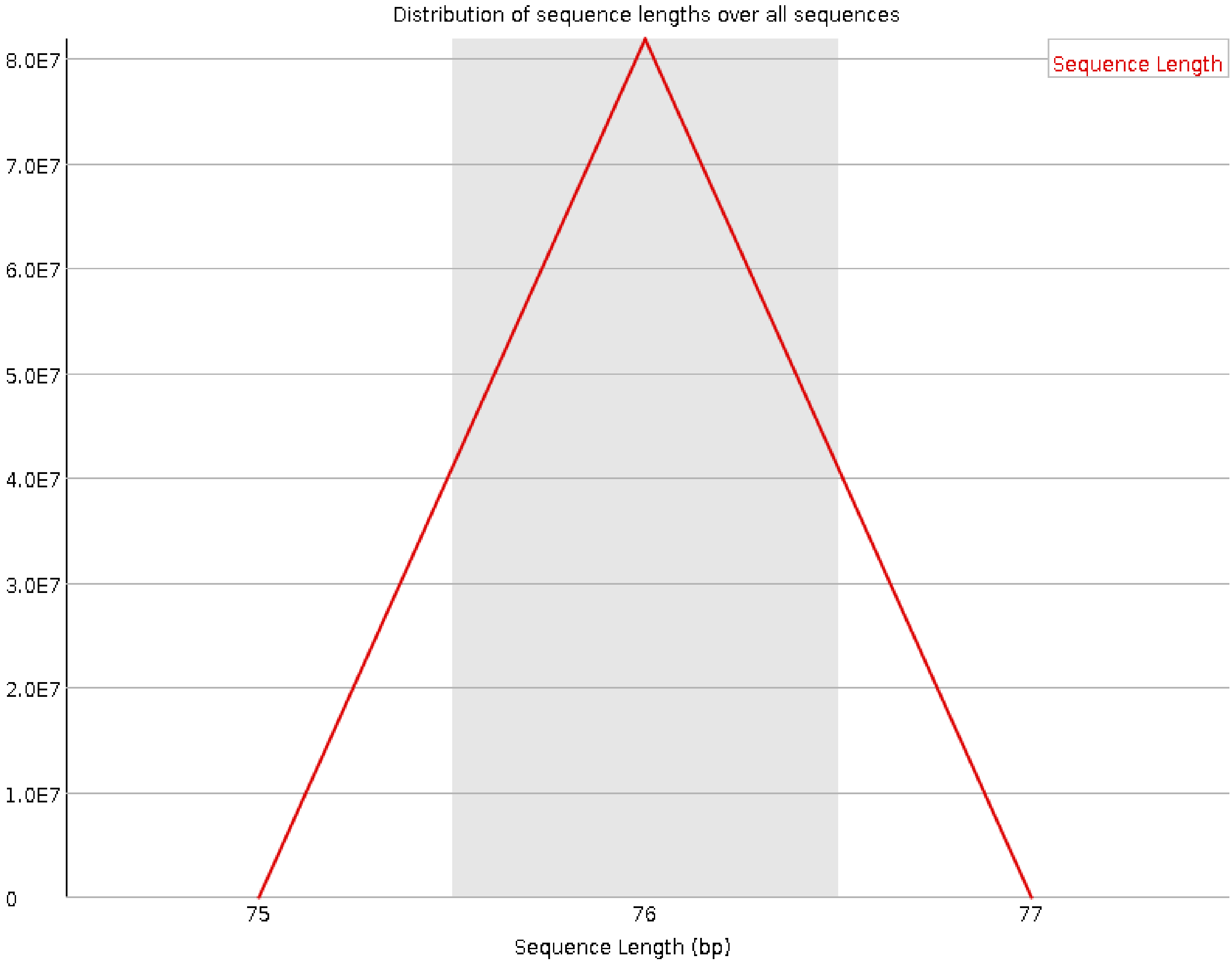


## Sequence Duplication Levels

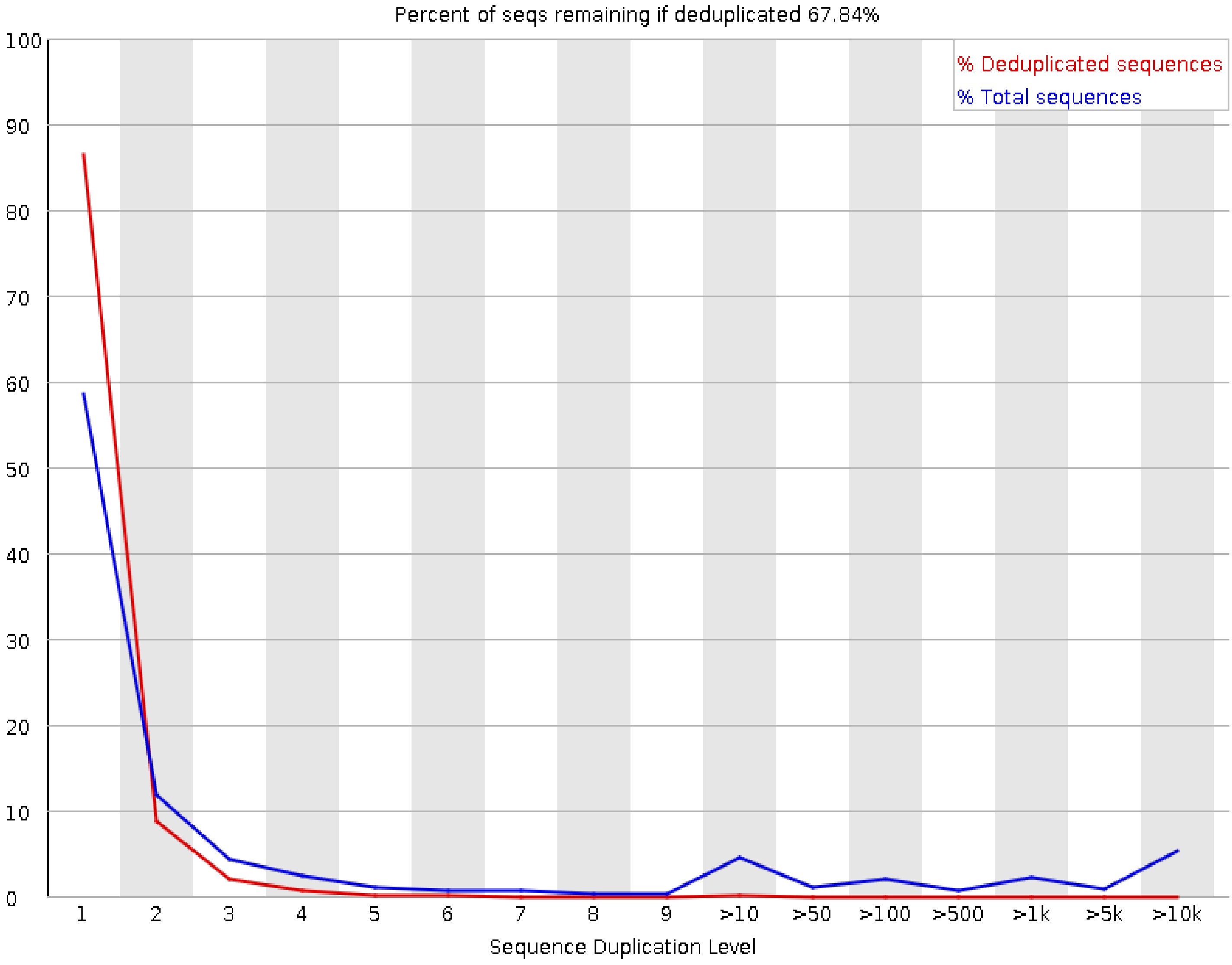


## Overrepresented sequences

| Sequence | Count | Percentage | Possible Source |
|---|---|---|---|
| CTGGAGTCTTGGAAGCTTGACTACCCTACGTTCTCCTACAAATGGACCTT | 185360 | 0.22639221471664936 | No Hit |
| CCCCACTACCACAAATTATGCAGTCGAGTTTCCCACATTTGGGGAAATCG | 161174 | 0.196852281046295 | No Hit |
| CCCTCCTTAGGCAACCTGGTGGTCCCCCGCTCCCGGGAGGTCACCATATT | 157508 | 0.19237475698958786 | No Hit |
| CTCCGTTTCCGACCTGGGCCGGTTCACCCCTCCTTAGGCAACCTGGTGGT | 145406 | 0.17759379786949245 | No Hit |
| GGGAGATACCATGATCACGAAGGTGGTTTTCCCAGGGCGAGGCTTATCCA | 135716 | 0.16575877110749238 | No Hit |
| CTGGCTGCGACATCTGTCACCCCATTGATCGCCAGGGTTGATTCGGCTGA | 120867 | 0.14762272235734386 | No Hit |
| CCCCTCCTTAGGCAACCTGGTGGTCCCCCGCTCCCGGGAGGTCACCATAT | 114087 | 0.13934186771891657 | No Hit |
| CCCCCACTACCACAAATTATGCAGTCGAGTTTCCCACATTTGGGGAAATC | 109733 | 0.1340240445484575 | No Hit |
| CCATGATCACGAAGGTGGTTTTCCCAGGGCGAGGCTTATCCATTGCACTC | 99392 | 0.12139390917736953 | No Hit |
| CCCACTACCACAAATTATGCAGTCGAGTTTCCCACATTTGGGGAAATCGC | 86184 | 0.10526212037731825 | No Hit |
| CCTCCTTAGGCAACCTGGTGGTCCCCCGCTCCCGGGAGGTCACCATATTG | 85702 | 0.10467342245169553 | No Hit |

| Sequence | Count | Percentage | Possible Source |
| --- | --- | --- | --- |

## Adapter Content

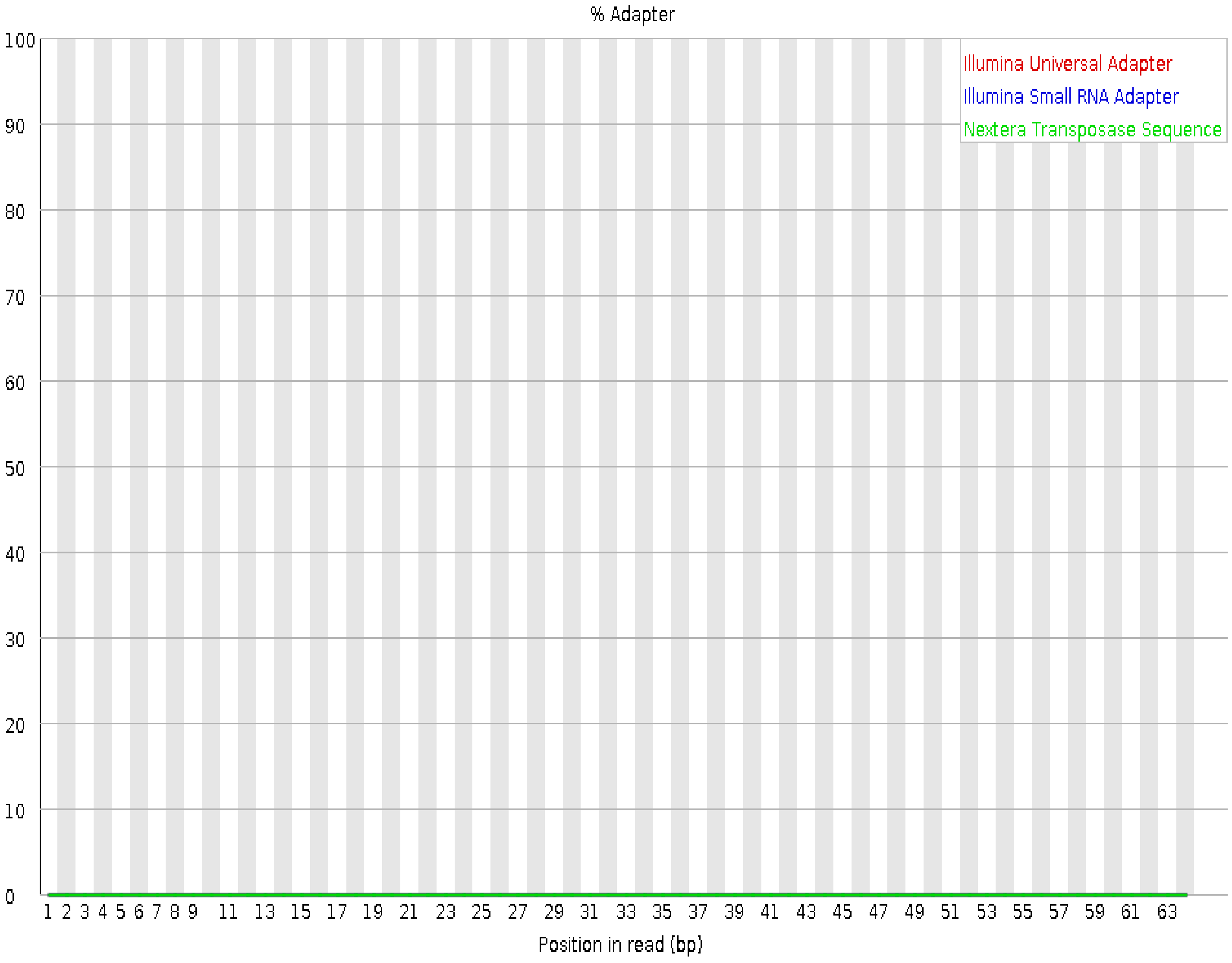


## Kmer Content

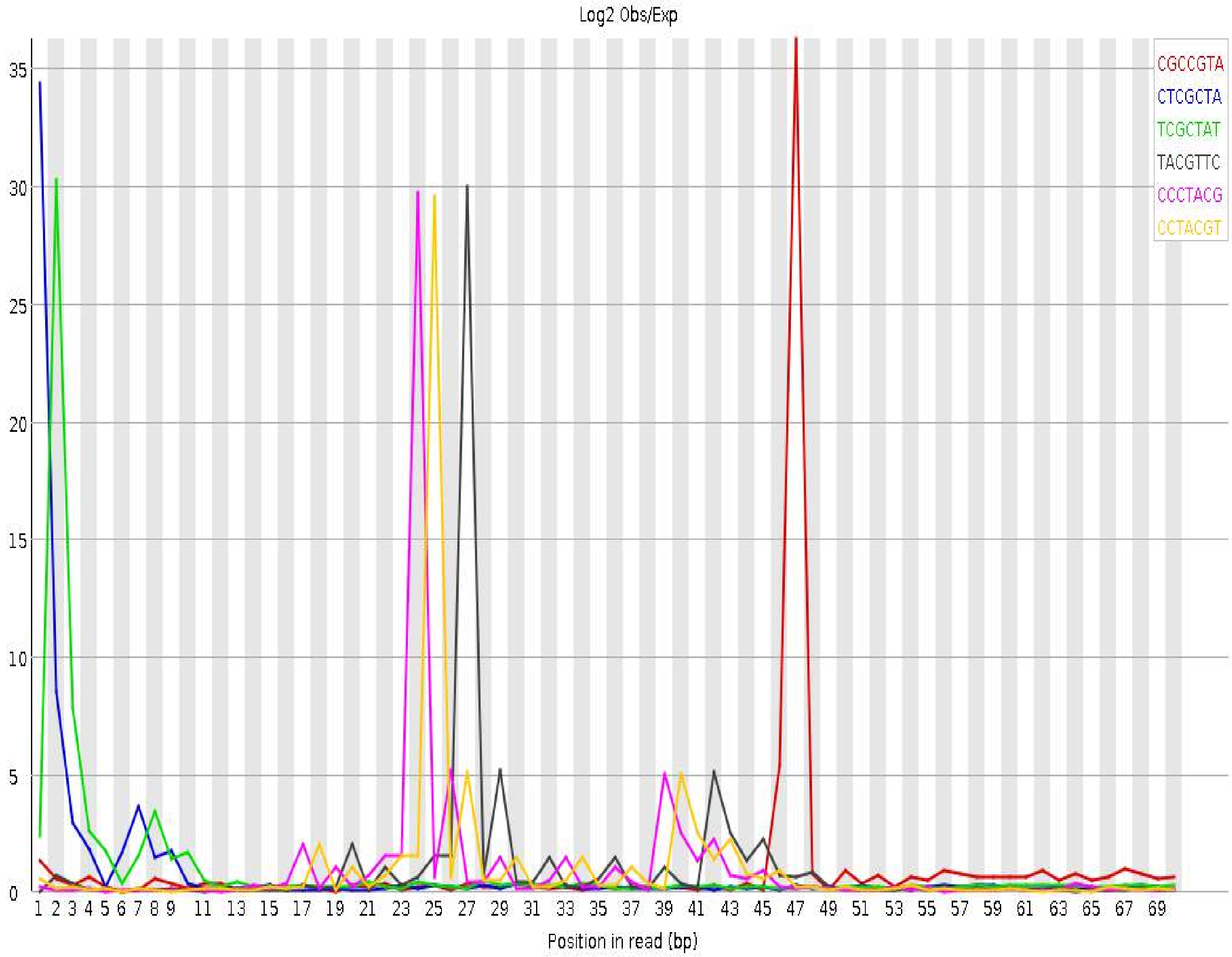


| Sequence | Count | PValue | Obs/Exp Max | Max Obs/Exp Position |
|---|---|---|---|---|
| CGCCGTA | 7580 | 0.0 | 36.24789 | 47 |
| CTCGCTA | 18555 | 0.0 | 34.431847 | 1 |
| TCGCTAT | 20790 | 0.0 | 30.29659 | 2 |
| TACGTTC | 44810 | 0.0 | 29.98537 | 27 |
| CCCTACG | 45230 | 0.0 | 29.72806 | 24 |
| CCTACGT | 45465 | 0.0 | 29.56999 | 25 |
| CTACGTT | 46355 | 0.0 | 29.047148 | 26 |
| TGCGACA | 31155 | 0.0 | 28.198025 | 6 |
| GCGACAT | 31165 | 0.0 | 28.129232 | 7 |
| GCGTCGT | 10025 | 0.0 | 27.85723 | 12 |
| GCTGCGA | 31795 | 0.0 | 27.647768 | 4 |
| GCCGTAT | 10400 | 0.0 | 26.621908 | 48 |
| GGCGCGT | 28795 | 0.0 | 26.373194 | 3 |
| CTGCGAC | 33300 | 0.0 | 26.32341 | 5 |
| GGGCGAT | 11850 | 0.0 | 26.321934 | 1 |

| Sequence | Count | PValue | Obs/Exp Max | Max Obs/Exp Position |
|---|---|---|---|---|
| ACGTTCT | 52190 | 0.0 | 25.[illegible] | 28 |
| TGGCGCG | 29425 | 0.0 | 25.76[illegible] | 2 |
| GACTACC | 53160 | 0.0 | 25.519478 | 19 |
| CGACATC | 34980 | 0.0 | 25.121422 | 8 |
| ACTACCC | 54560 | 0.0 | 24.859892 | 20 |

**Produced by FastQC (version 0.11.2)**

# FastQC Report

Mon 4 May 2015
Origene_Adult_Stomach_0288_GCCAAT_L006_R1.fastq.gz

## Summary


- Basic Statistics
- Per base sequence quality
- Per tile sequence quality
- Per sequence quality scores
- Per base sequence content
- Per sequence GC content
- Per base N content
- Sequence Length Distribution
- Sequence Duplication Levels
- Overrepresented sequences
- Adapter Content
- Kmer Content


## Basic Statistics

| Measure | Value |
| --- | --- |
| Filename | Origene_Adult_Stomach_0288_GCCAAT_L006_R1.fastq.gz |
| File type | Conventional base calls |
| Encoding | Sanger / Illumina 1.9 |
| Total Sequences | 88595062 |
| Sequences flagged as poor quality | 0 |
| Sequence length | 76 |
| %GC | 55 |

## Per base sequence quality

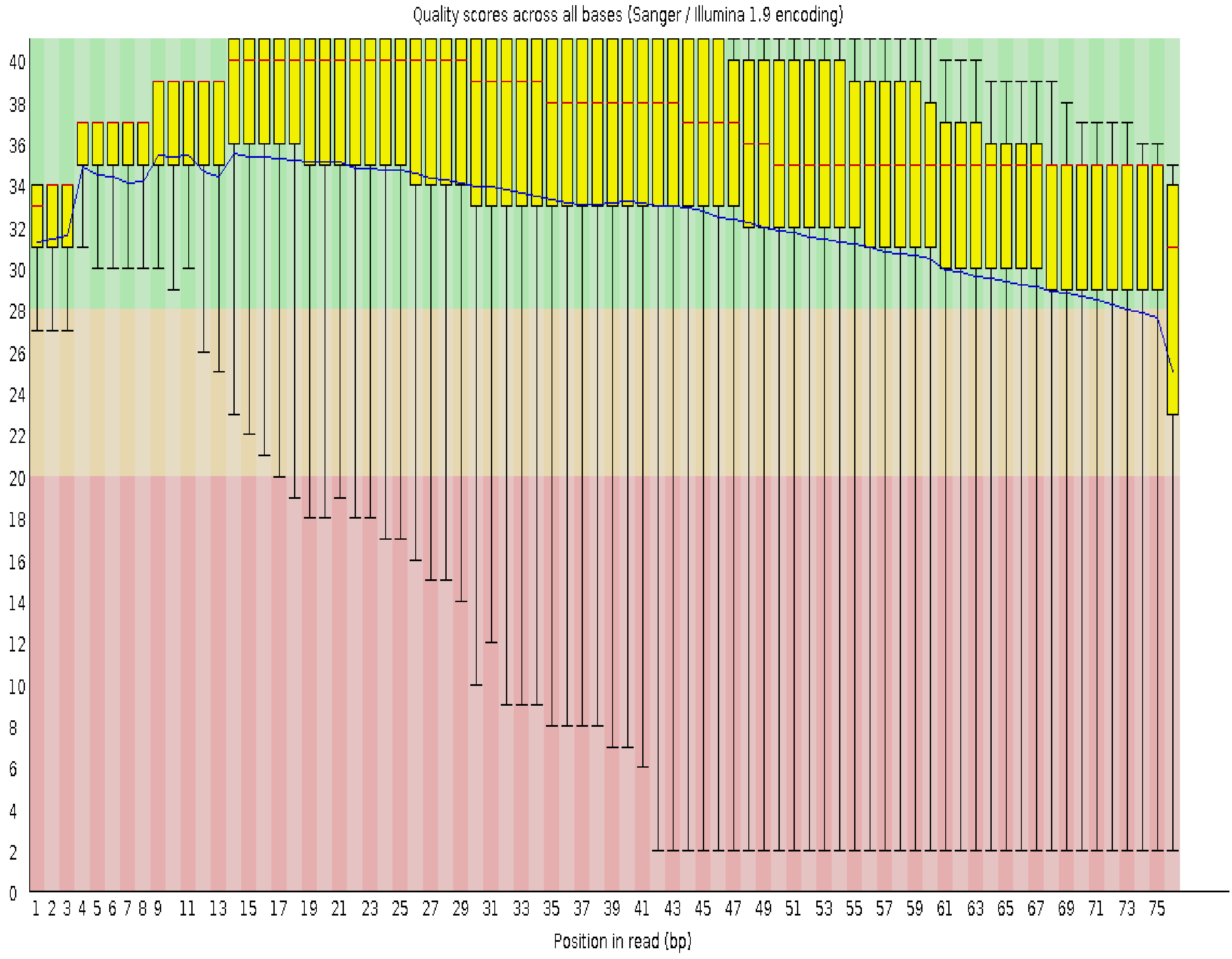


## Per tile sequence quality

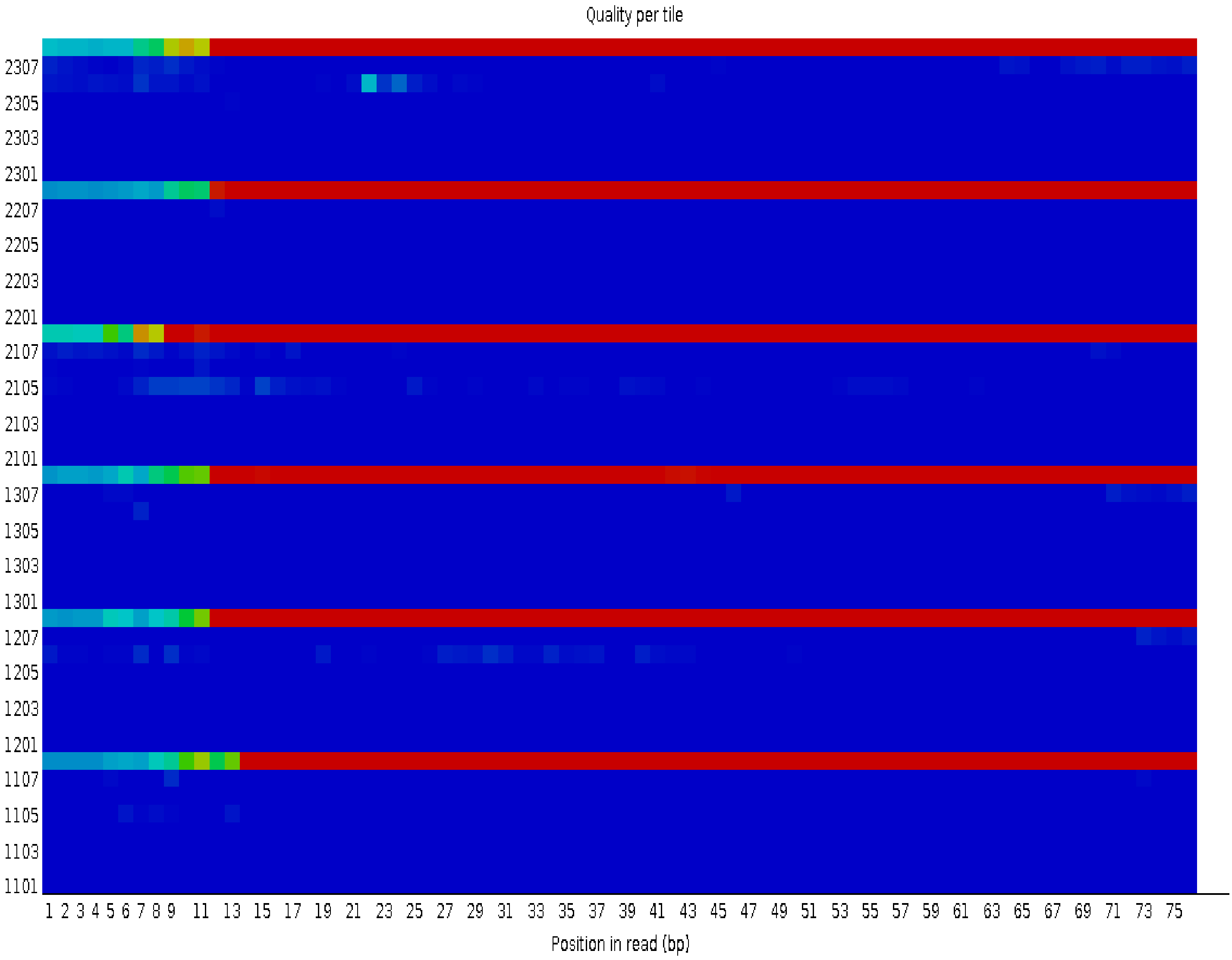


## Per sequence quality scores

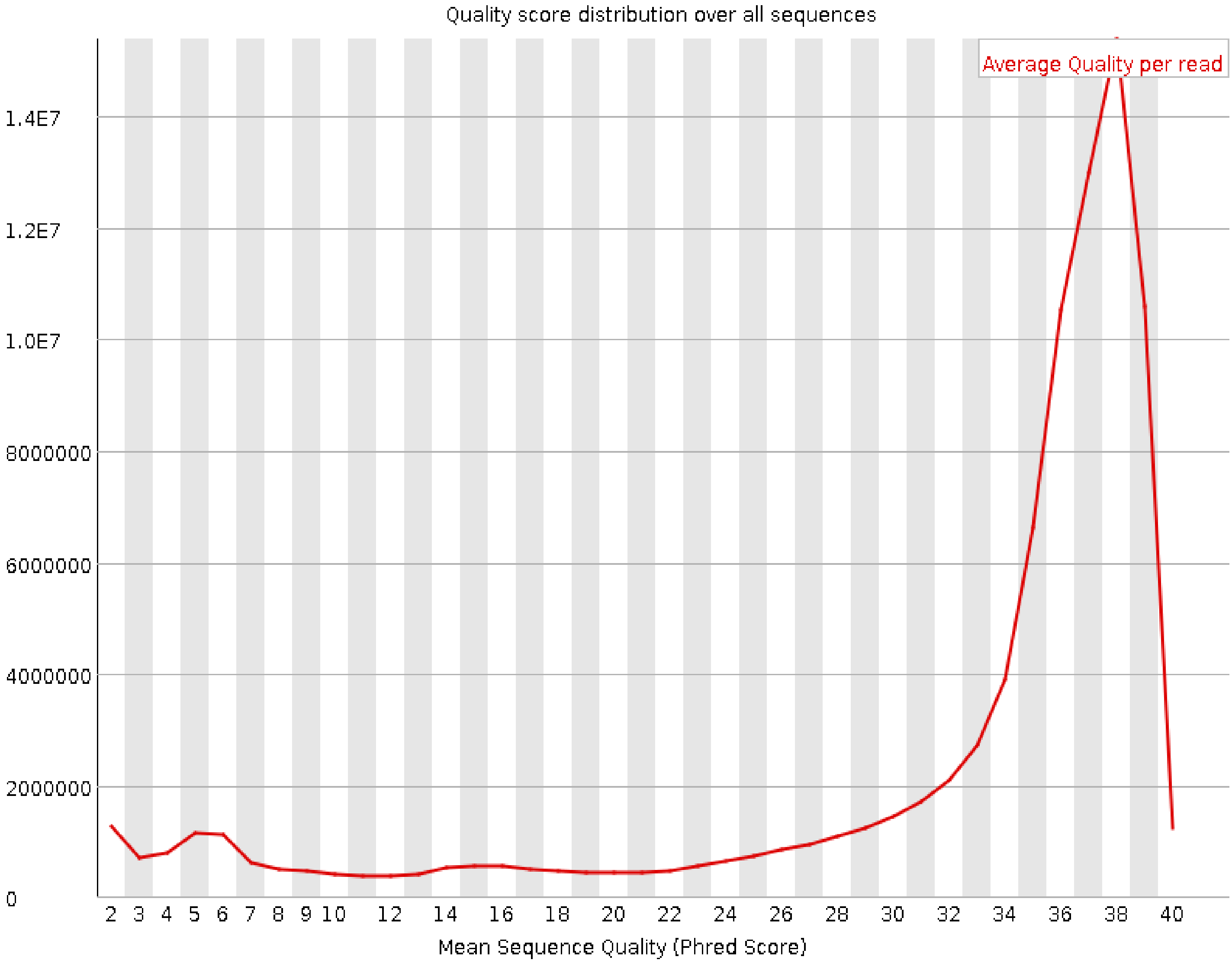


## Per base sequence content

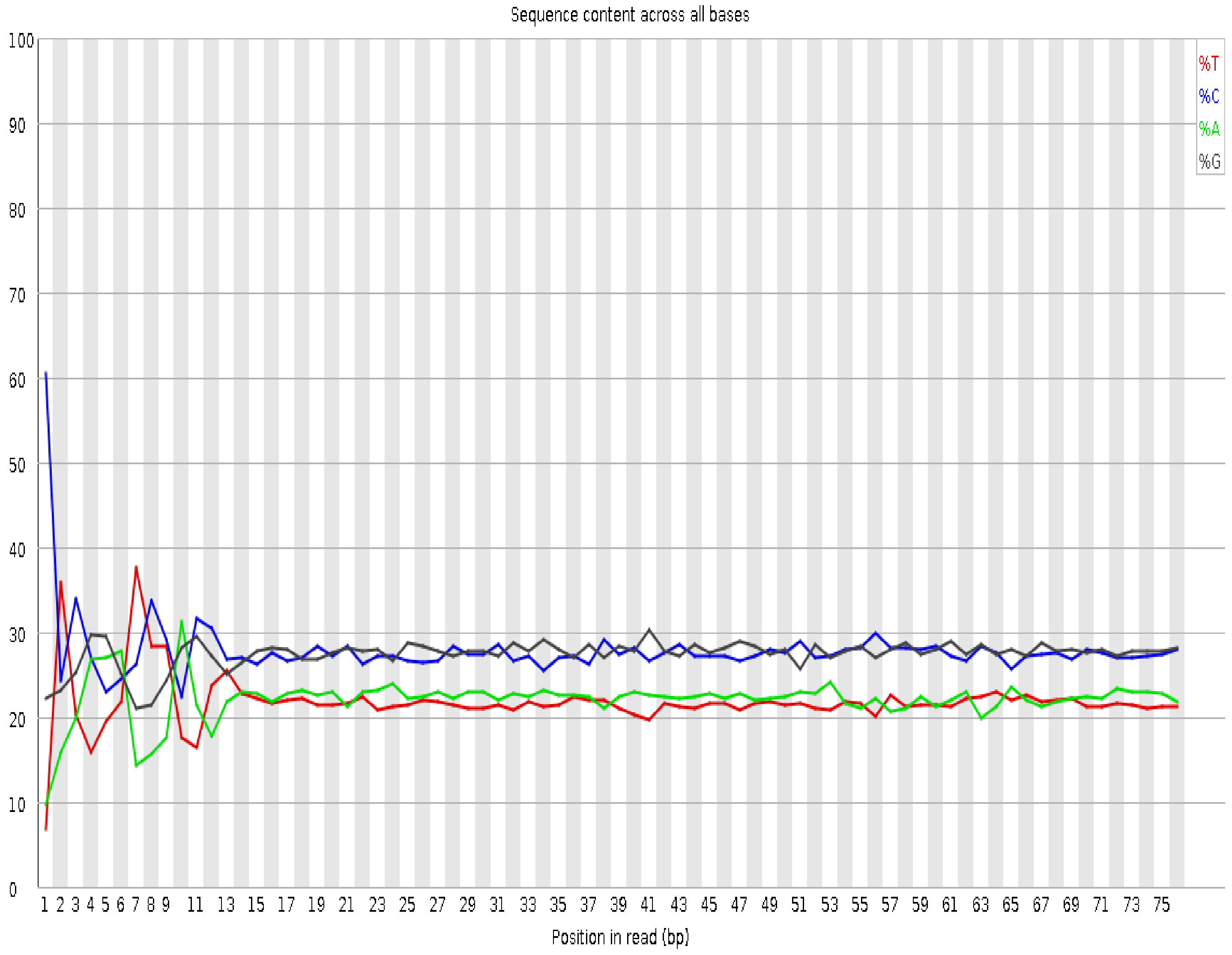


## Per sequence GC content

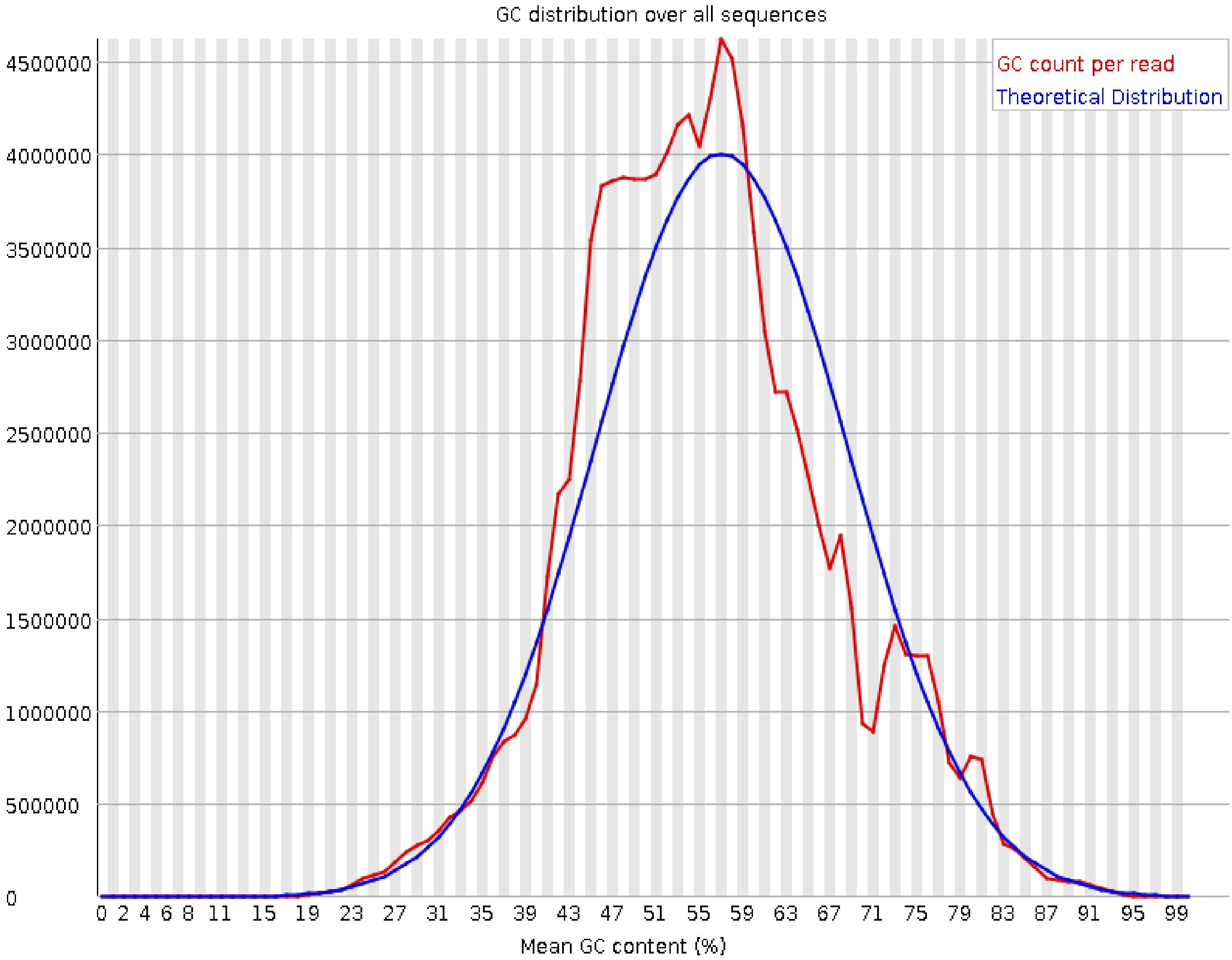


## Per base N content

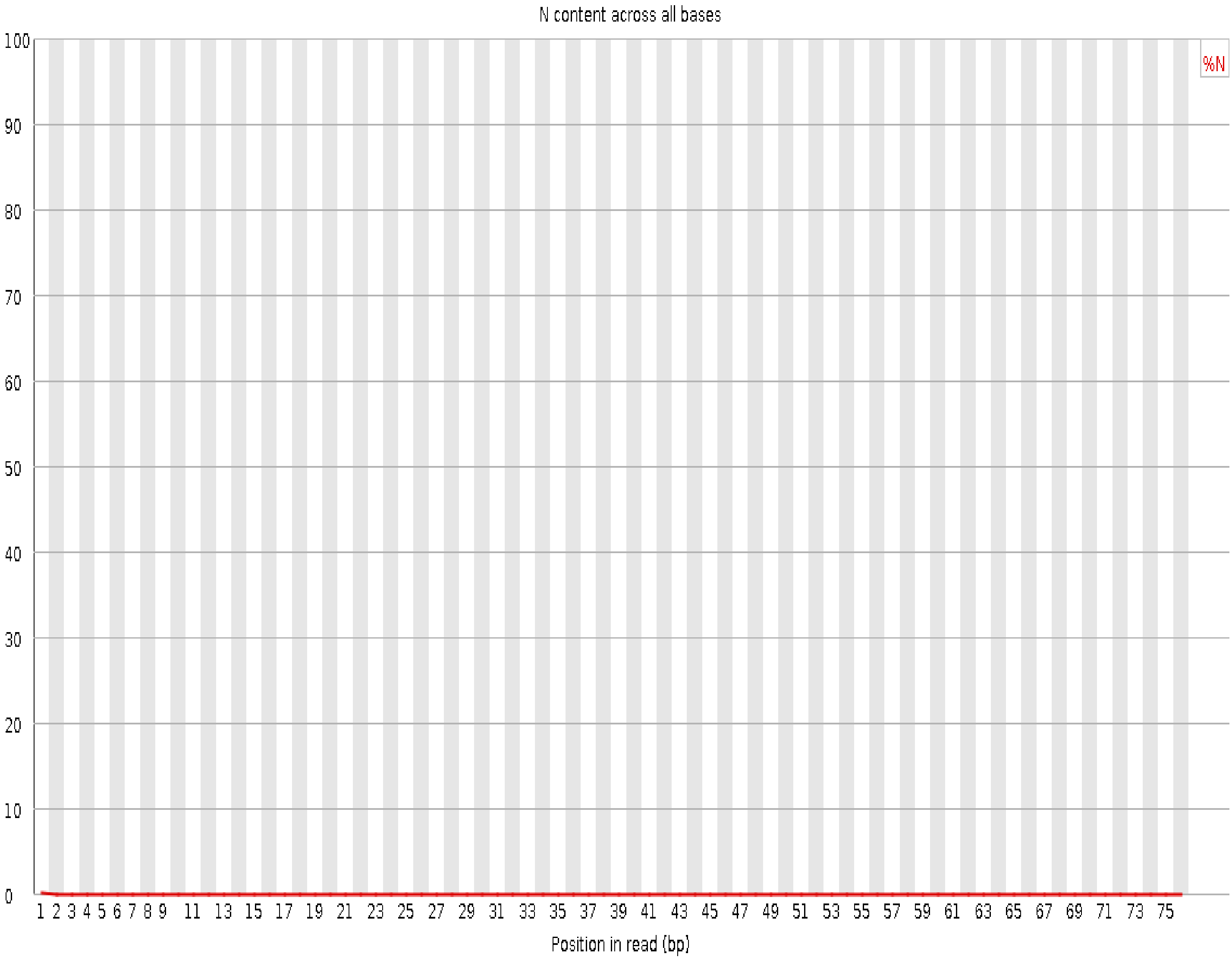


## Sequence Length Distribution

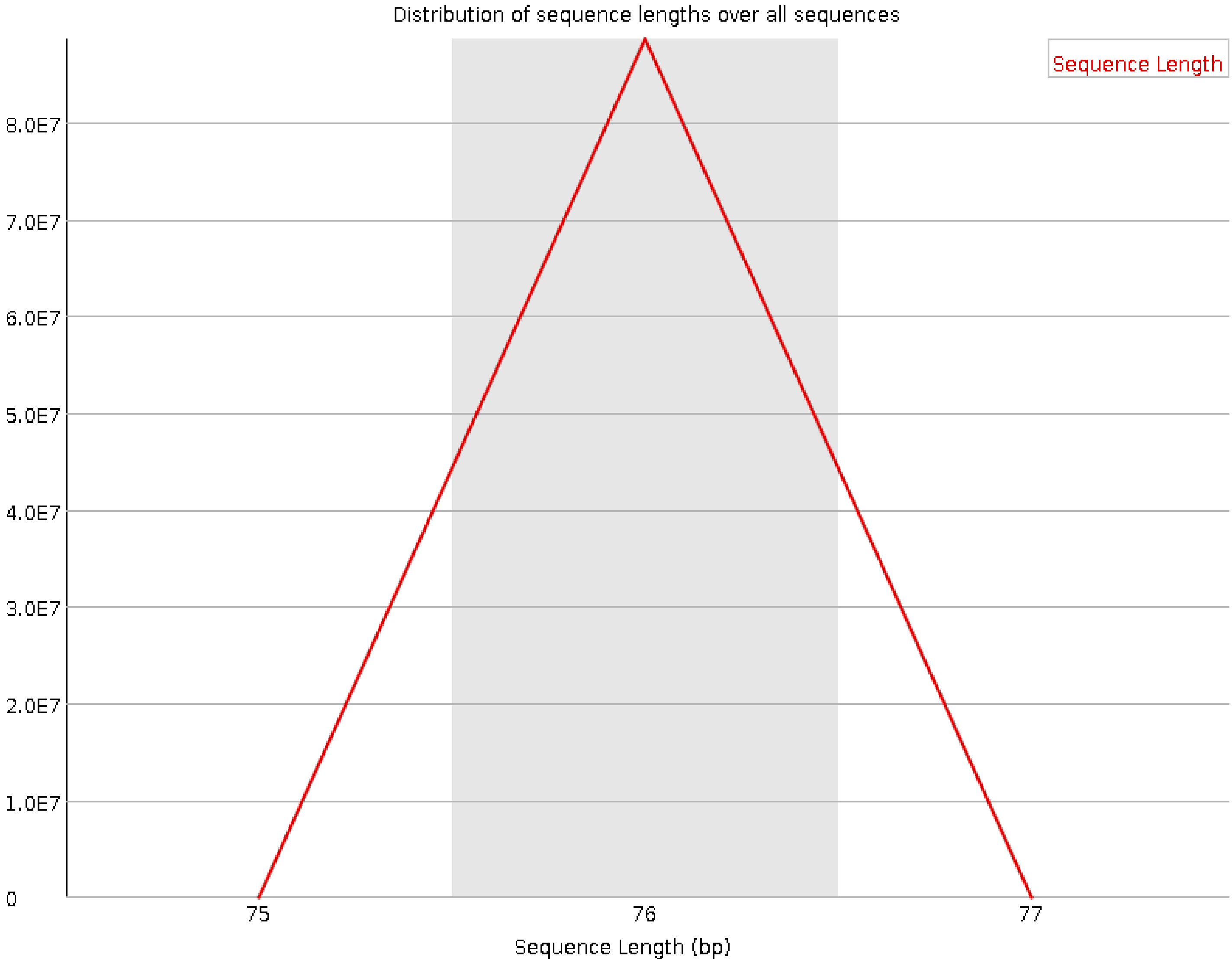


## Sequence Duplication Levels

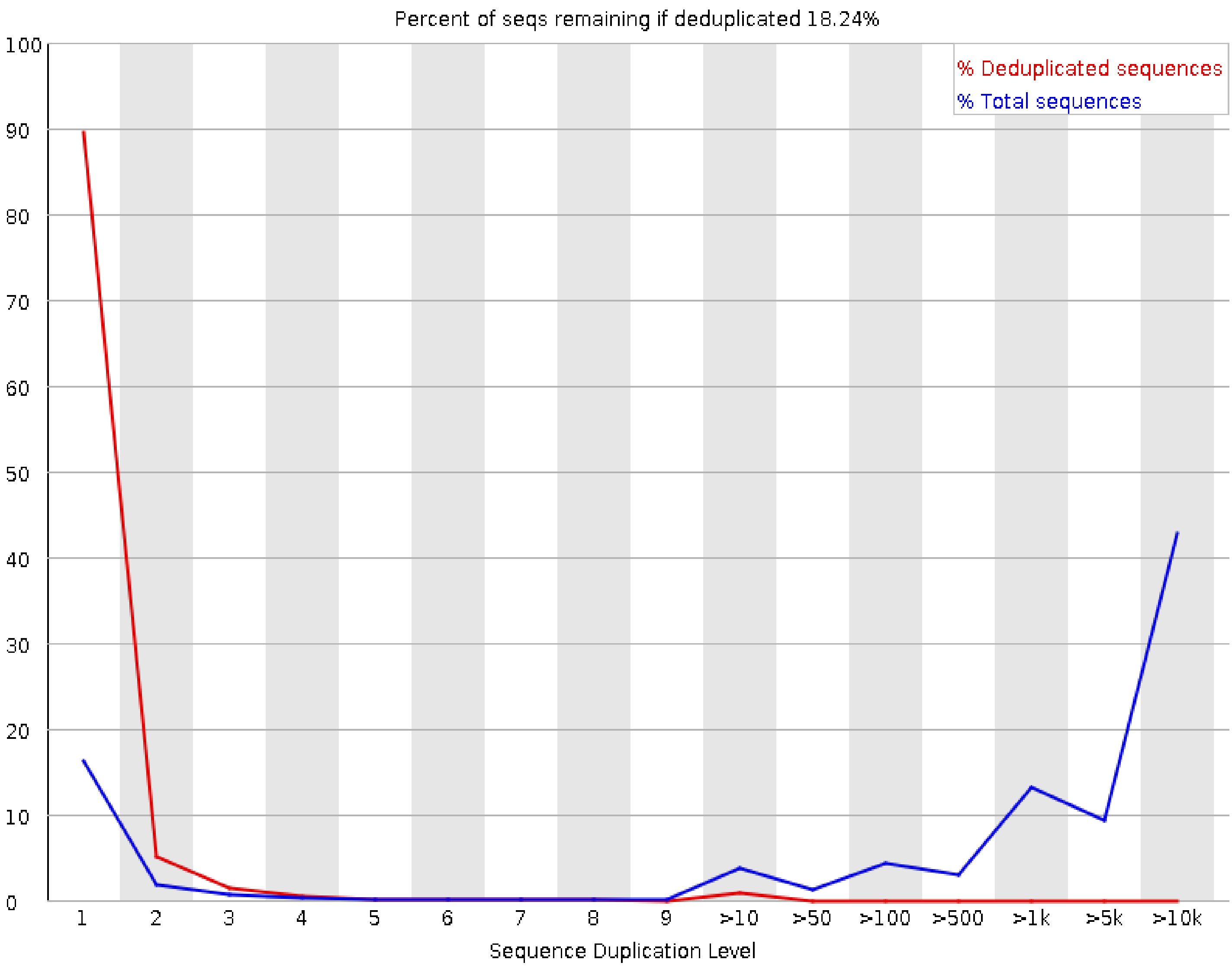


## Overrepresented sequences

| Sequence | Count | Percentage | Possible Source |
|---|---|---|---|
| CTTCCGTACGCCACATGTCCCGCGCCCCGCCGCGGGGCGGGGATTCGGCG | 328812 | 0.3711403238252714 | No Hit |
| CCGGTATTTAGCCTTAGATGGAGTTTACCACCCGCTTTGGGCTGCATTCC | 285216 | 0.321932163668446 | No Hit |
| CTGAATTTAAGCATATTAGTCAGCGGAGGAGAAGAAACTAACCAGGATTC | 250223 | 0.282434476991505 | No Hit |
| CTGGATAGTAGGTAGGGACAGTGGGAATCTCGTTCATCCATTCATGCGCG | 242562 | 0.2737872681888297 | No Hit |
| CGCAGTTTTATCCGGTAAAGCGAATGATTAGAGGTCTTGGGGCCGAAACG | 200241 | 0.2260182401588025 | No Hit |
| CTTCACCGTGCCAGACTAGAGTCAAGCTCAACAGGGTCTTCTTTCCCCGC | 195234 | 0.2203666836420296 | No Hit |
| CTCTCTTCAAAGTTCTTTTCAACTTTCCCTTACGGTACTTGTTGACTATC | 189962 | 0.2144160133890983 | No Hit |
| CTTGAACTCTCTCTTCAAAGTTCTTTTCAACTTTCCCTTACGGTACTTGT | 189375 | 0.2137534482452306 | No Hit |
| CTCCCTTTCGATCGGCCGAGGGCAACGGAGGCCATCGCCCGTCCCTTCGG | 180082 | 0.2032641503202514 | No Hit |
| CCCGAAGTTACGGATCCGGCTTGCCGACTTCCCTTACCTACATTGTTCCA | 178062 | 0.2009841135389690 | No Hit |
| GTCAAAGTGAAGAAATTCAATGAAGCGCGGGTAAACGGCGGGAGTAACTA | 176370 | 0.199074300551875 | No Hit |

| Sequence | Count | Percentage | Possible Source |
|---|---|---|---|
| CCCGTCGGCATGTATTAGCTCTAGAATTACCACAGTTATCCAAGTAGGAG | 167364 | 0.18890894844681072 | No Hit |
| CTGCCAGTAGCATATGCTTGTCTCAAAGATTAAGCCATGCATGTCTAAGT | 164803 | 0.1860182681513332 | N[illegible] |
| CCCATATCCGCAGCAGGTCTCCAAGGTGAACAGCCTCTGGCATGTTGGAA | 162888 | 0.18385674813343433 | No Hit |
| CGCGTAACTAGTTAGCATGCCAGAGTCTCGTTCGTTATCGGAATTAACCA | 162524 | 0.18344589002037157 | No Hit |
| CCTCACCCGGCCCGGACACGGACAGGATTGACAGATTGATAGCTCTTTCT | 158625 | 0.1790449675400645 | No Hit |
| CAGAAACCTCCCGTGGAGCAGAAGGGCAAAAGCTCGCTTGATCTTGATTT | 157962 | 0.1782966188341287 | No Hit |
| CTCTCATGTCTCTTCACCGTGCCAGACTAGAGTCAAGCTCAACAGGGTCT | 151996 | 0.17156260921178654 | No Hit |
| CGCGATGTGATTTCTGCCCAGTGCTCTGAATGTCAAAGTGAAGAAATTCA | 149698 | 0.16896878519030778 | No Hit |
| CGGGTCTTCCGTACGCCACATGTCCCGCGCCCCGCCGCGGGGCGGGGATT | 143914 | 0.16244020462449701 | No Hit |
| GTAAATCTCGCGCCGGGCCGTACCCATATCCGCAGCAGGTCTCCAAGGTG | 142469 | 0.16080918821412415 | No Hit |
| CTTGTCTCAAAGATTAAGCCATGCATGTCTAAGTACGCACGGCCGGTACA | 137636 | 0.1553540309052439 | No Hit |
| CTCGCATTCCACGCCCGGCTCCACGCCAGCGAGCCGGGCTTCTTACCCAT | 133962 | 0.15120707291790145 | No Hit |
| CCGACATCGAAGGATCAAAAAGCGACGTCGCTATGAACGCTTGGCCGCCA | 133876 | 0.15111000204503497 | No Hit |
| CTTAGAGCCAATCCTTATCCCGAAGTTACGGATCCGGCTTGCCGACTTCC | 133227 | 0.150377455574217 | No Hit |
| CTTAGATGGAGTTTACCACCCGCTTTGGGCTGCATTCCCAAGCAACCCGA | 122898 | 0.13871879225052067 | No Hit |
| CGAAGGCCCGCGGCGGGTGTTGACGCGATGTGATTTCTGCCCAGTGCTCT | 121548 | 0.1371950052927329 | No Hit |
| CACCCGTTTACCTCTTAACGGTTTCACGCCCTCTTGAACTCTCTCTTCAA | 119929 | 0.1353675896744674 | No Hit |
| CTCATGTCTCTTCACCGTGCCAGACTAGAGTCAAGCTCAACAGGGTCTTC | 119667 | 0.1350718621315486 | No Hit |
| CTTTAAATGGGTAAGAAGCCCGGCTCGCTGGCGTGGAGCCGGGCGTGGAA | 115962 | 0.130889913480731 | No Hit |
| CGCGTCACTAATTAGATGACGAGGCATTTGGCTACCTTAAGAGAGTCATA | 114113 | 0.12880288971410167 | No Hit |
| CTCACCCGGCCCGGACACGGACAGGATTGACAGATTGATAGCTCTTTCTC | 113971 | 0.128642609900764 | No Hit |
| CTCCGCCACTCCGGATTCGGGGATCTGAACCCGACTCCCTTTCGATCGGC | 113780 | 0.12842702226451402 | No Hit |
| CTGCTGTCTATATCAACCAACACCTTTTCTGGGGTCTGATGAGCGTCGGC | 113358 | 0.12795069774882037 | No Hit |
| CCCAGGCATAGTTCACCATCTTTCGGGTCCTAACACGTGCGCTCGTGCTC | 112767 | 0.12728361768063326 | No Hit |
| CCACTCTCGACTGCCGGCGACGGCCGGGTATGGGCCCGACGCTCCAGCGC | 112282 | 0.1267361831069095 | No Hit |
| CCCGCTTTGGGCTGCATTCCCAAGCAACCCGACTCCGGGAAGACCCGGGC | 111589 | 0.12595397246857842 | No Hit |
| CTCGATCAGAAGGACTTGGGCCCCCCACGAGCGGCGCCGGGGAGCGGGTC | 110361 | 0.12456789070253148 | No Hit |
| CAGACGTGGCGACCCGCTGAATTTAAGCATATTAGTCAGCGGAGGAGAAG | 109715 | 0.12383873042495304 | No Hit |
| CGAGAACTTTGAAGGCCGAAGTGGAGAAGGGTTCCATGTGAACAGCAGTT | 109604 | 0.12371344127509049 | No Hit |
| CGAGATTCCCACTGTCCCTACCTACTATCCAGCGAAACCACAGCCAAGGG | 107720 | 0.12158691192066663 | No Hit |
| CCTGTGGTAACTTTTCTGACACCTCCTGCTTAAAACCCAAAAGGTCAGAA | 103614 | 0.11695234210683209 | No Hit |
| GCTGAATTTAAGCATATTAGTCAGCGGAGGAGAAGAAACTAACCAGGATT | 103567 | 0.11689929174607948 | No Hit |
| CAAAGATTAAGCCATGCATGTCTAAGTACGCACGGCCGGTACAGTGAAAC | 102189 | 0.11534390031805611 | No Hit |
| CCCACTTATTCTACACCTCTCATGTCTCTTCACCGTGCCAGACTAGAGTC | 102108 | 0.11525247310058882 | No Hit |
| CTTGGCTGTGGTTTCGCTGGATAGTAGGTAGGGACAGTGGGAATCTCGTT | 99543 | 0.11235727788079204 | No Hit |
| GTCGGCATGTATTAGCTCTAGAATTACCACAGTTATCCAAGTAGGAGAGG | 94874 | 0.107087232469006 | No Hit |
| GCCCTCTTGAACTCTCTCTTCAAAGTTCTTTTCAACTTTCCCTTACGGTA | 93169 | 0.10516274597787403 | No Hit |
| CTCCCACTTATTCTACACCTCTCATGTCTCTTCACCGTGCCAGACTAGAG | 93165 | 0.10515823105355467 | No Hit |
| CTGAATGTCAAAGTGAAGAAATTCAATGAAGCGCGGGTAAACGGCGGGAG | 90586 | 0.10224723359864007 | No Hit |

| Sequence | Count | Percentage | Possible Source |
|---|---|---|---|
| CTCCCGTCCACTCTCGACTGCCGGCGACGGCCGGGTATGGGCCCGACGCT | 90296 | 0.10191990158548567 | No Hit |
| CTCCACTTCGGCCTTCAAAG[illegible]GAATATTTGCTACTACCACCA | [illegible] | 0.1012[illegible]9228 | No Hit |
| CCCGGGGCTCCCGCCGGCTTCTCCGGGATCGGTCGCGTTACCGCACTGGA | 89593 | 0.10112640363635617 | No Hit |
| GAACAATCCAACGCTTGGTGAATTCTGCTTCACAATGATAGGAAGAGCCG | 89359 | 0.10086228056367294 | No Hit |

## Adapter Content

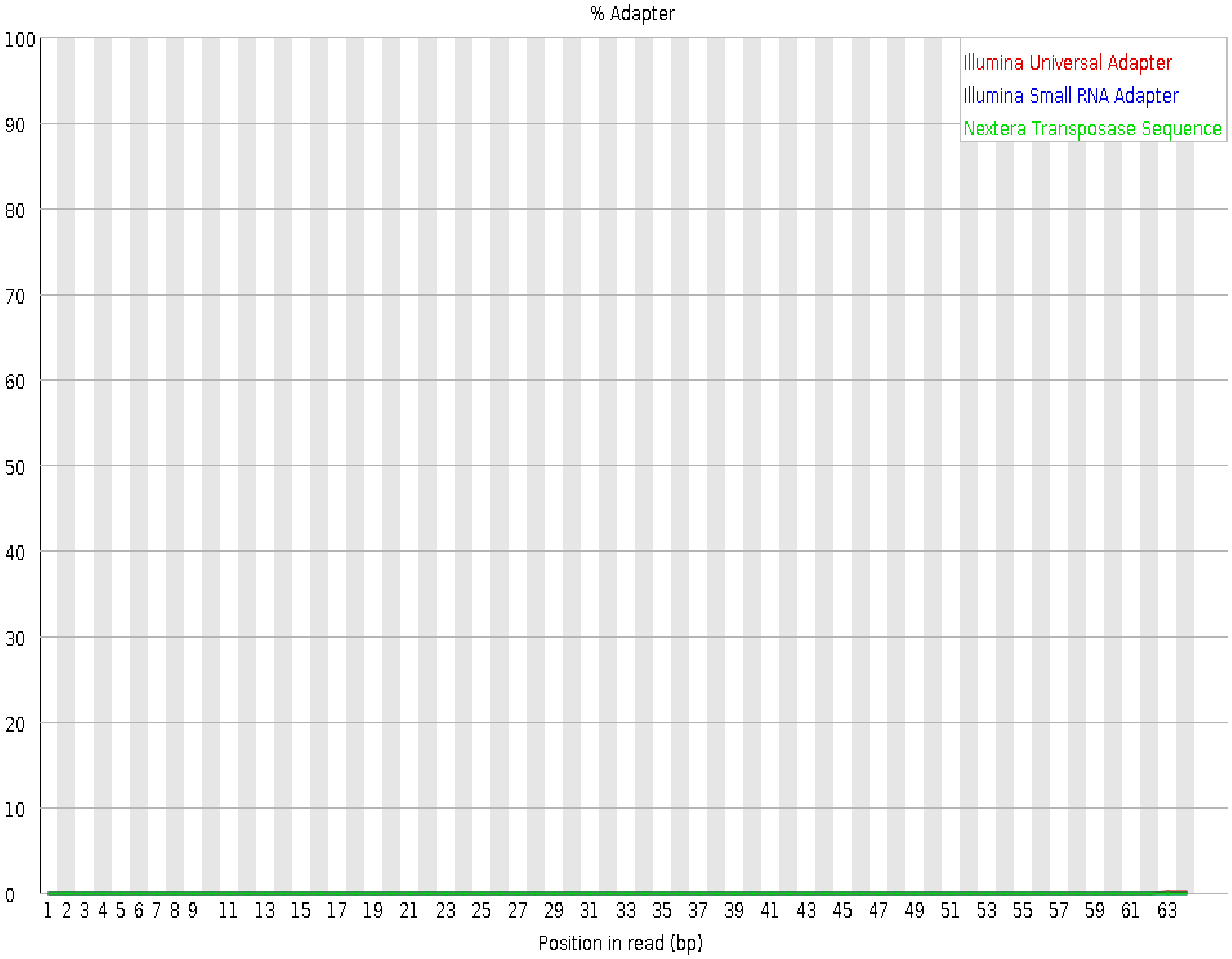


## Kmer Content

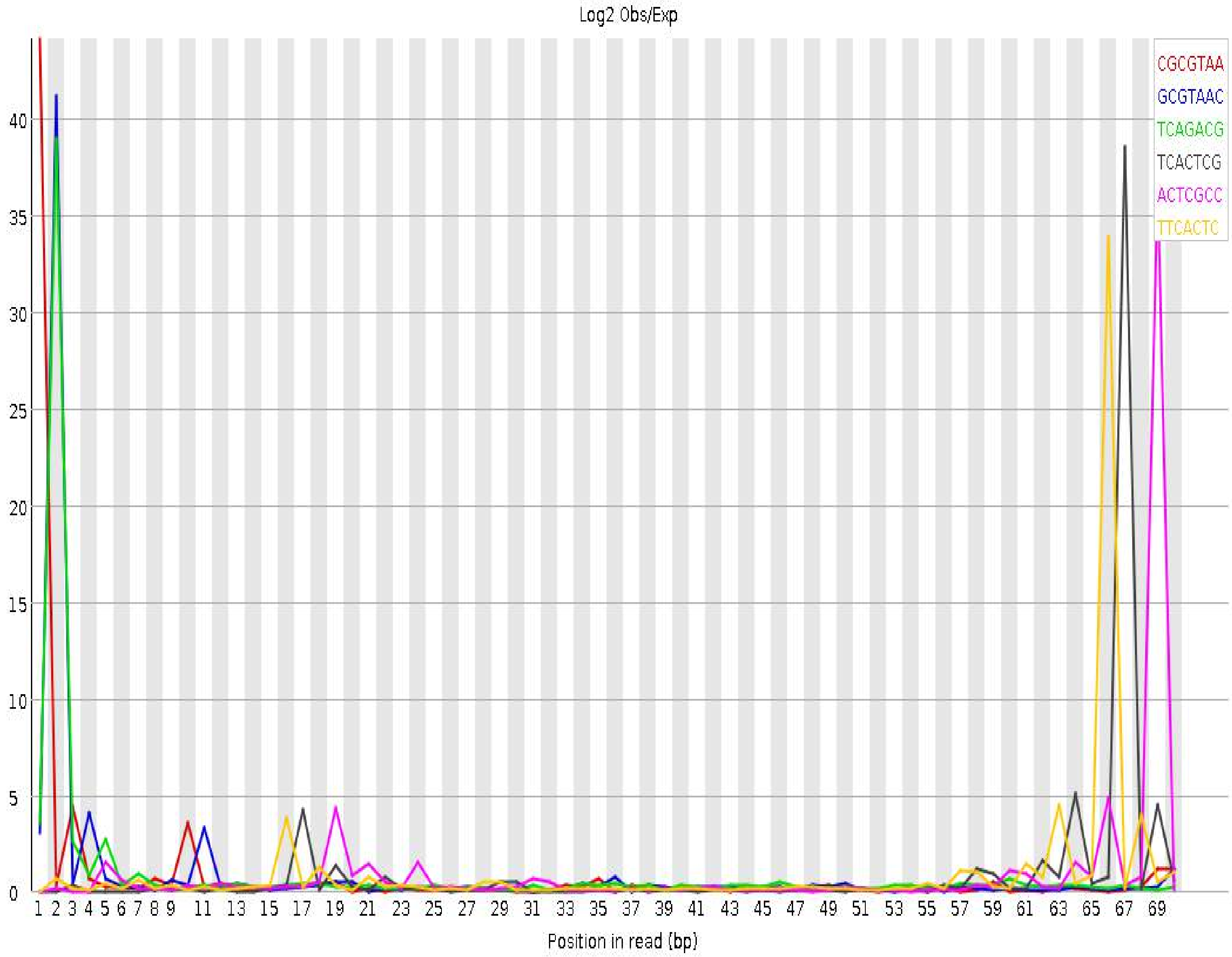


| Sequence | Count | PValue | Obs/Exp Max | Max Obs/Exp Position |
|---|---|---|---|---|
| CGCGTAA | 31895 | 0.0 | 44.11351 | 1 |
| GCGTAAC | 34315 | 0.0 | 41.18876 | 2 |
| TCAGACG | 19375 | 0.0 | 38.967705 | 2 |
| TCACTCG | 67485 | 0.0 | 38.55774 | 67 |
| ACTCGCC | 71375 | 0.0 | 36.677696 | 69 |
| TTCACTC | 76845 | 0.0 | 33.86794 | 66 |
| TATCGCC | 7035 | 0.0 | 32.831512 | 64 |
| TCGCCTA | 7095 | 0.0 | 32.454887 | 66 |
| CTATCGC | 7115 | 0.0 | 32.418793 | 63 |
| TCCAACG | 23195 | 0.0 | 32.093155 | 7 |
| CCAGTAG | 42825 | 0.0 | 32.026035 | 4 |
| GTTAGCA | 41605 | 0.0 | 31.979435 | 11 |
| AGTTAGC | 41795 | 0.0 | 31.917538 | 10 |
| TAGTTAG | 42295 | 0.0 | 31.88684 | 9 |
| CGCCTAT | 7430 | 0.0 | 31.130928 | 67 |

| Sequence | Count | PValue | Obs/Exp Max | Max Obs/Exp Position |
|---|---|---|---|---|
| ACAATCC | 25625 | 0.0 | 30.898603 | 3 |
| AACTAGT | 44930 | 0.0 | 30.62[illegible] | 6 |
| ACTAGTT | 45360 | 0.0 | 30.260342 | 7 |
| AGACGTG | 35595 | 0.0 | 30.021626 | 2 |
| TGTGTCG | 7090 | 0.0 | 29.918272 | 3 |

**Produced by FastQC (version 0.11.2)**

# FastQC Report

Mon 4 May 2015
Origene_Adult_Stomach_0288_GCCAAT_L006_R2.fastq.gz

## Summary


- Basic Statistics
- Per base sequence quality
- Per tile sequence quality
- Per sequence quality scores
- Per base sequence content
- Per sequence GC content
- Per base N content
- Sequence Length Distribution
- Sequence Duplication Levels
- Overrepresented sequences
- Adapter Content
- Kmer Content


## Basic Statistics

| Measure | Value |
|---|---|
| Filename | Origene_Adult_Stomach_0288_GCCAAT_L006_R2.fastq.gz |
| File type | Conventional base calls |
| Encoding | Sanger / Illumina 1.9 |
| Total Sequences | 88595062 |
| Sequences flagged as poor quality | 0 |
| Sequence length | 76 |
| %GC | 55 |

## Per base sequence quality

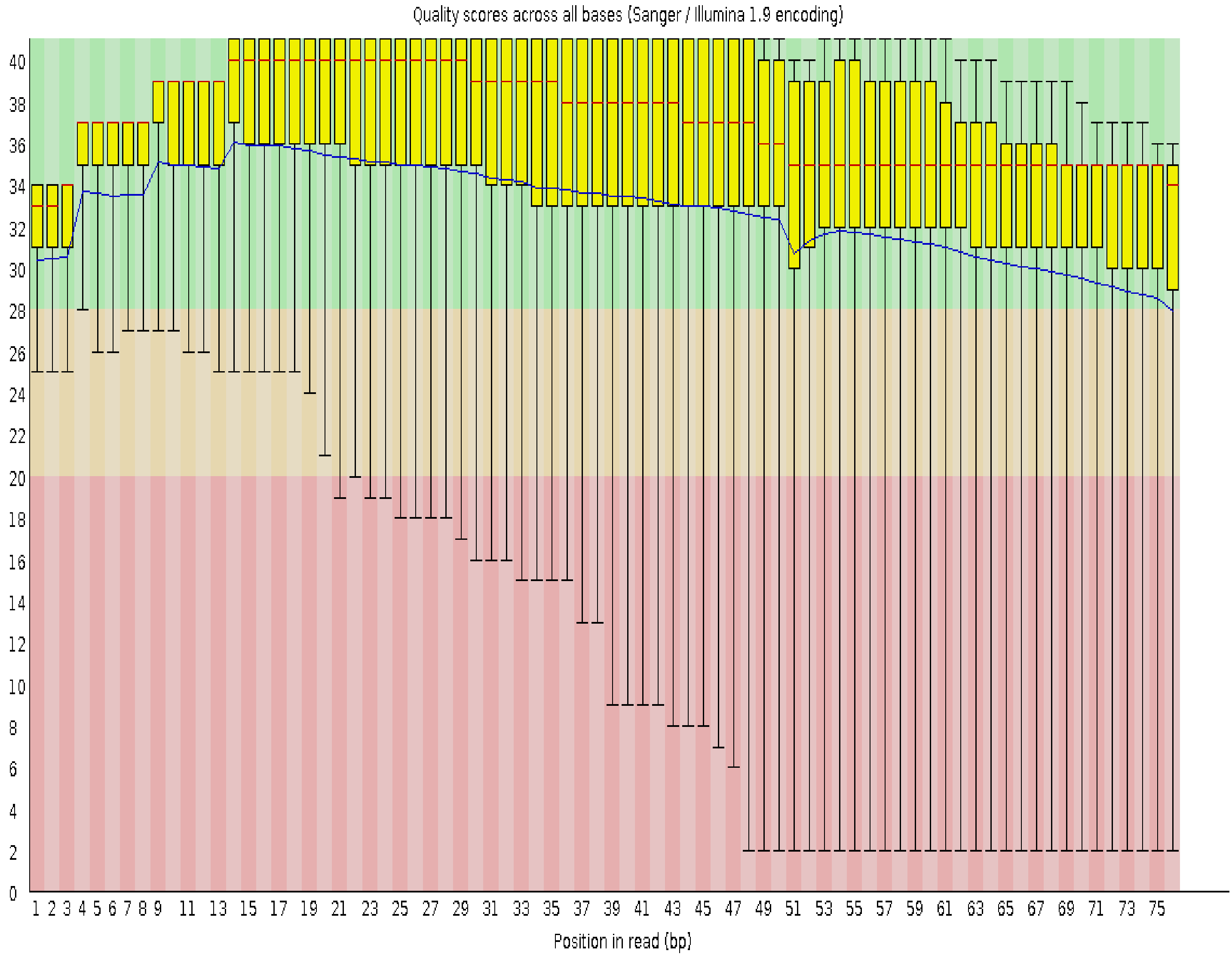


## Per tile sequence quality

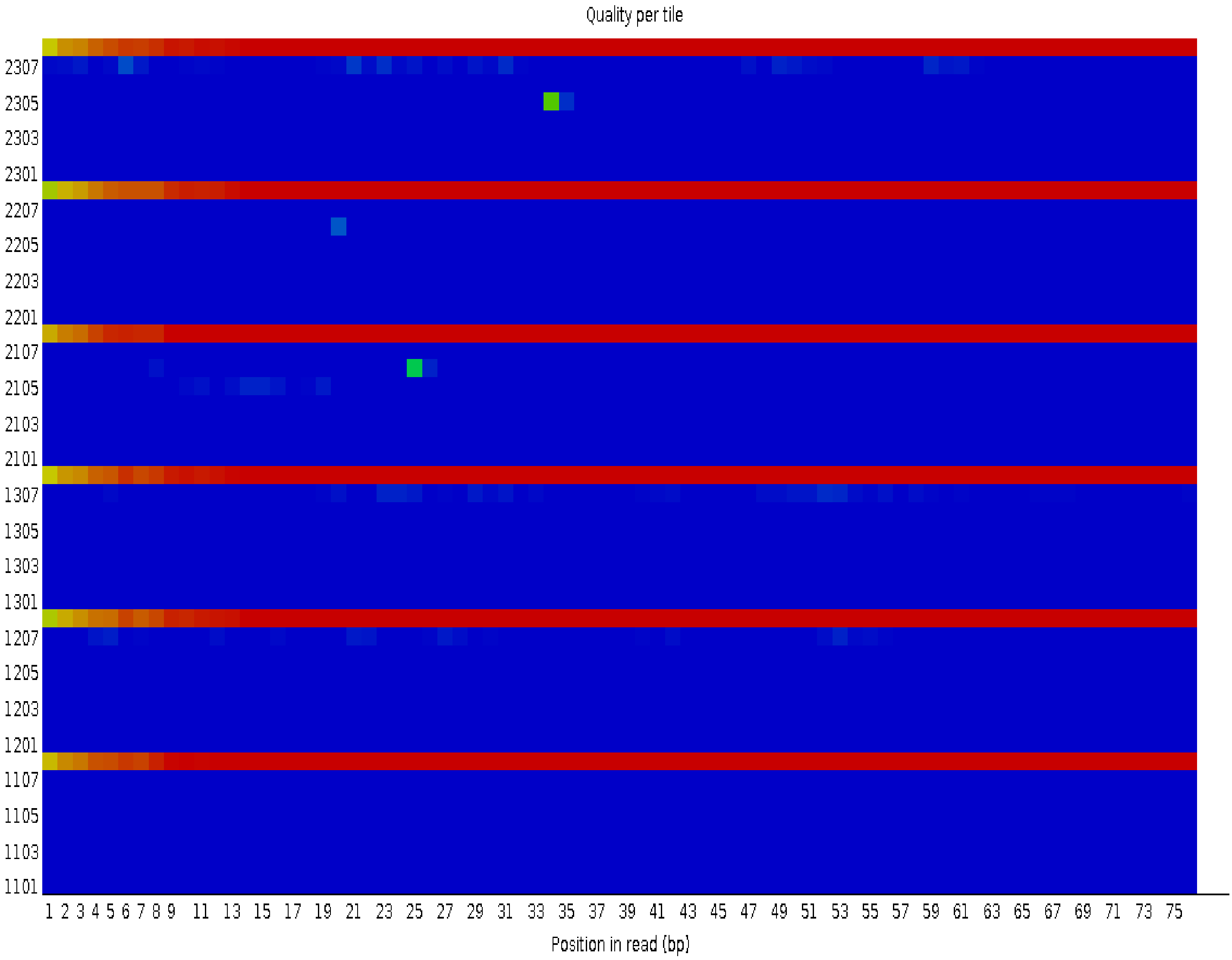


## Per sequence quality scores

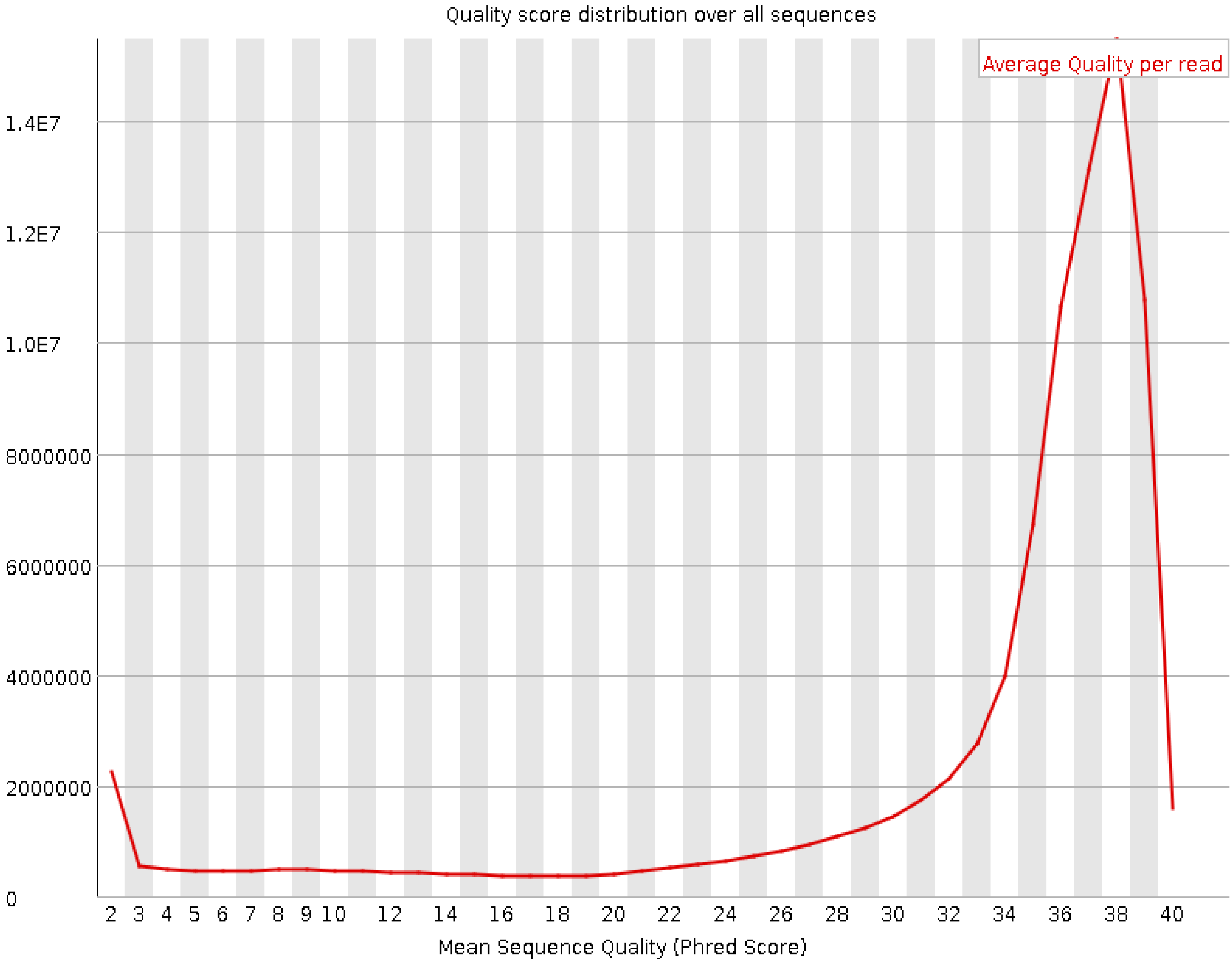


## Per base sequence content

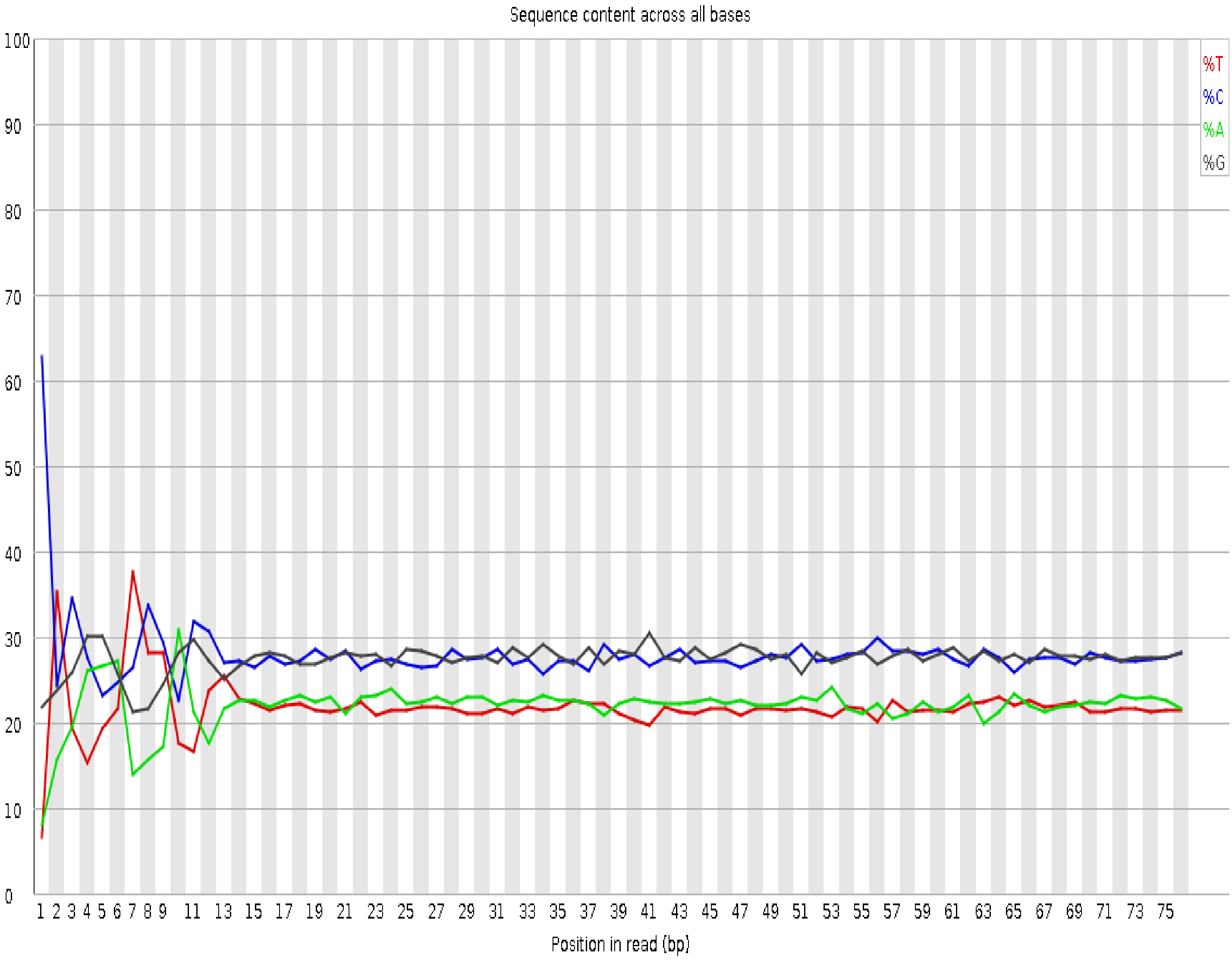


## Per sequence GC content

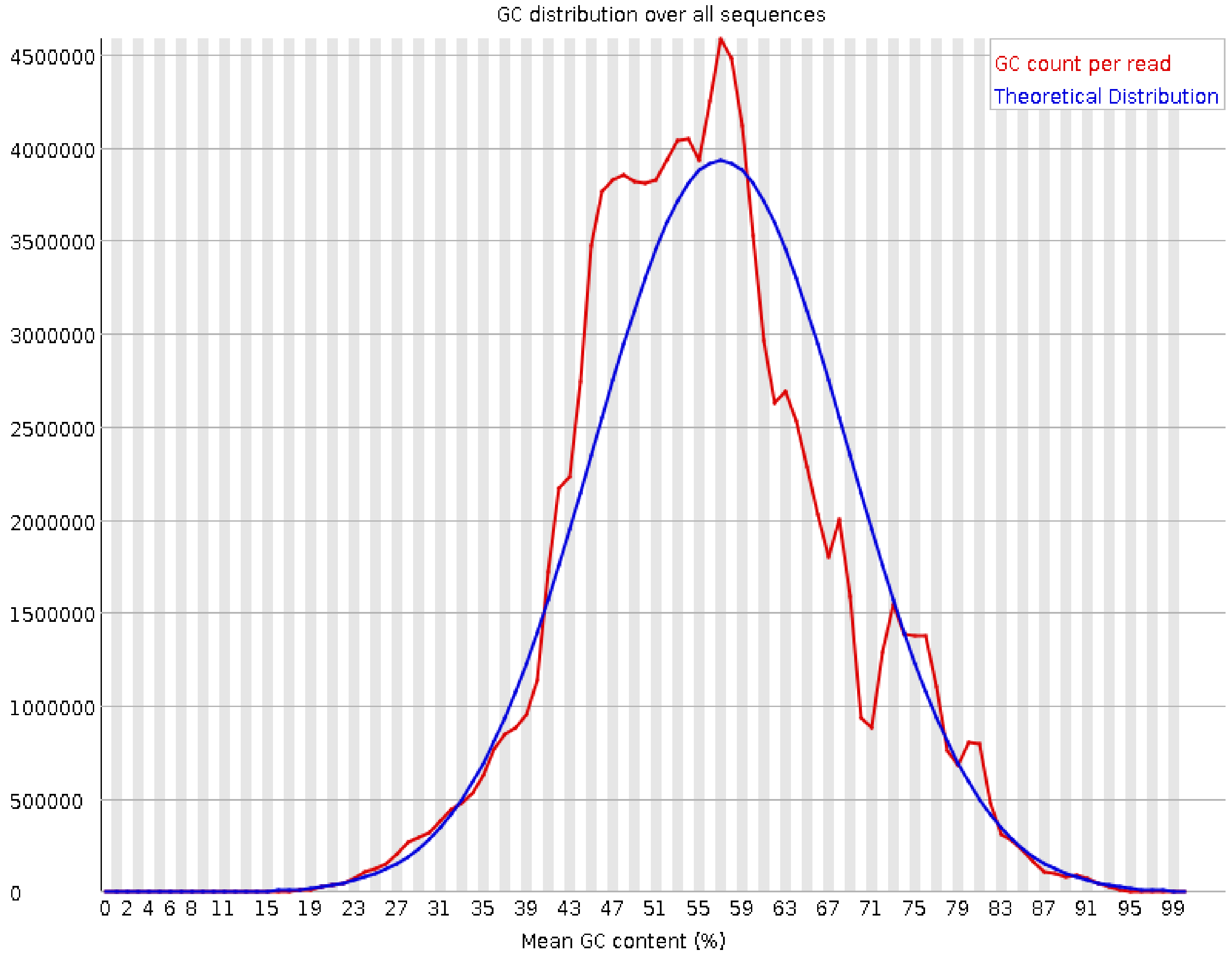


## Per base N content

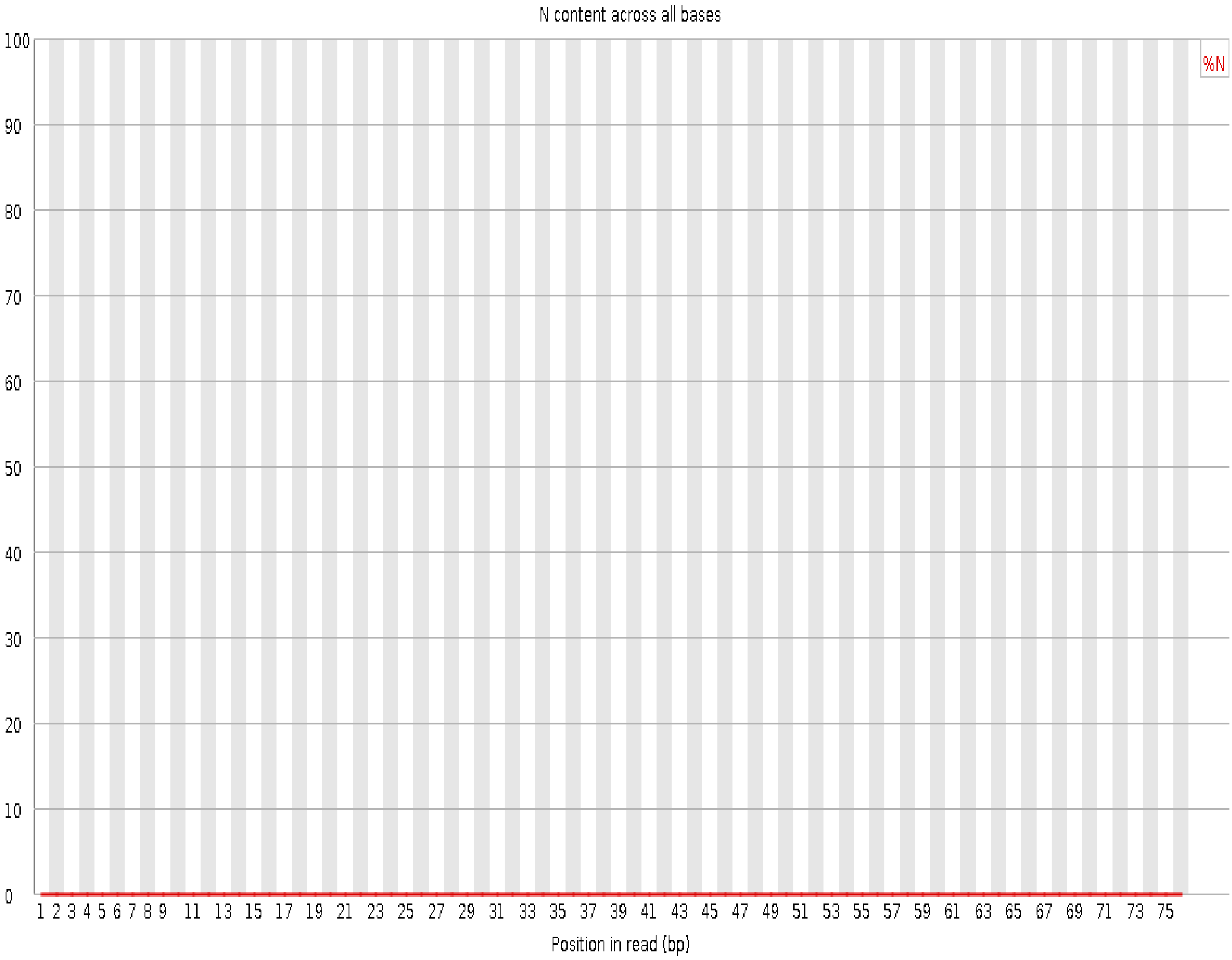


## Sequence Length Distribution

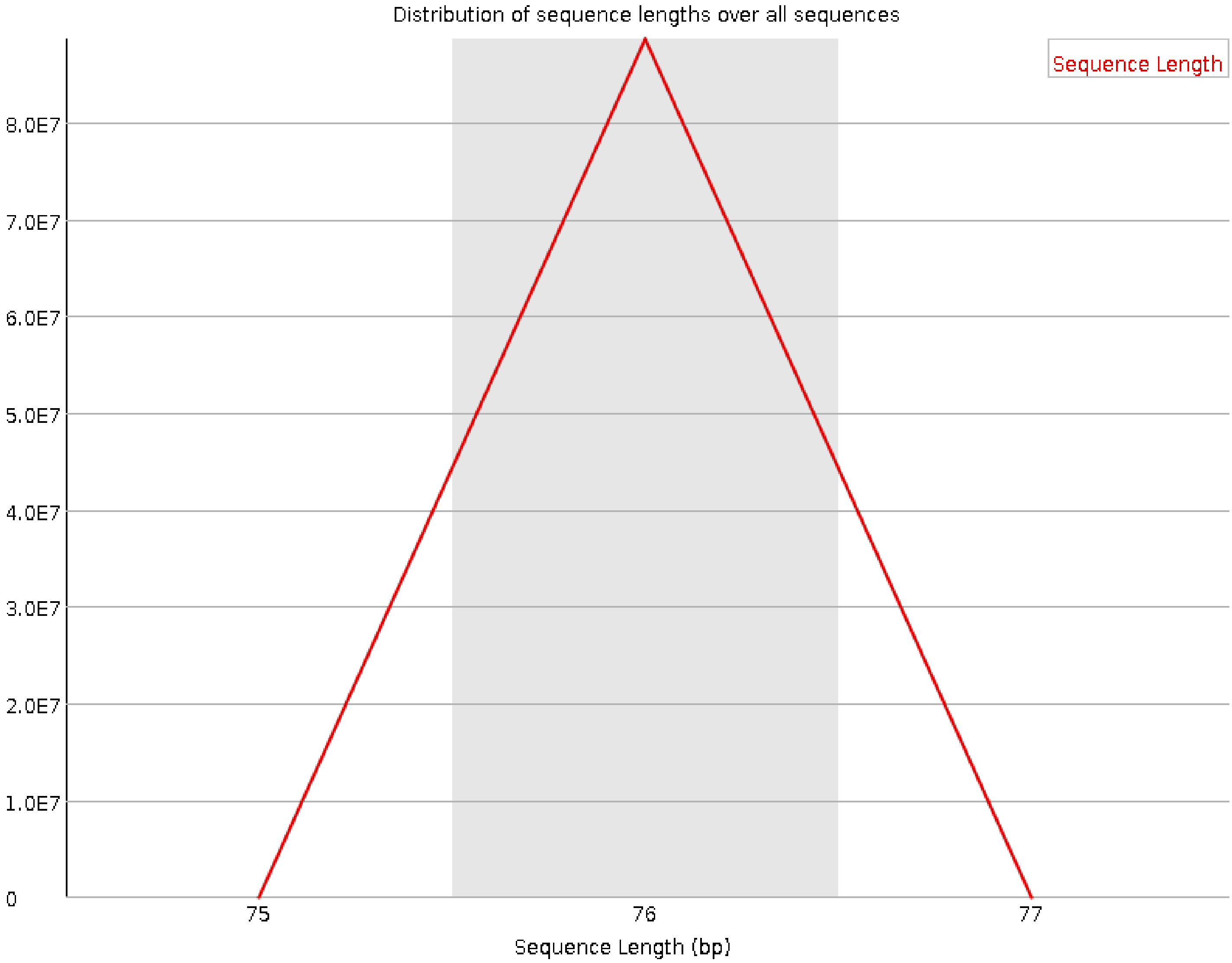


## Sequence Duplication Levels

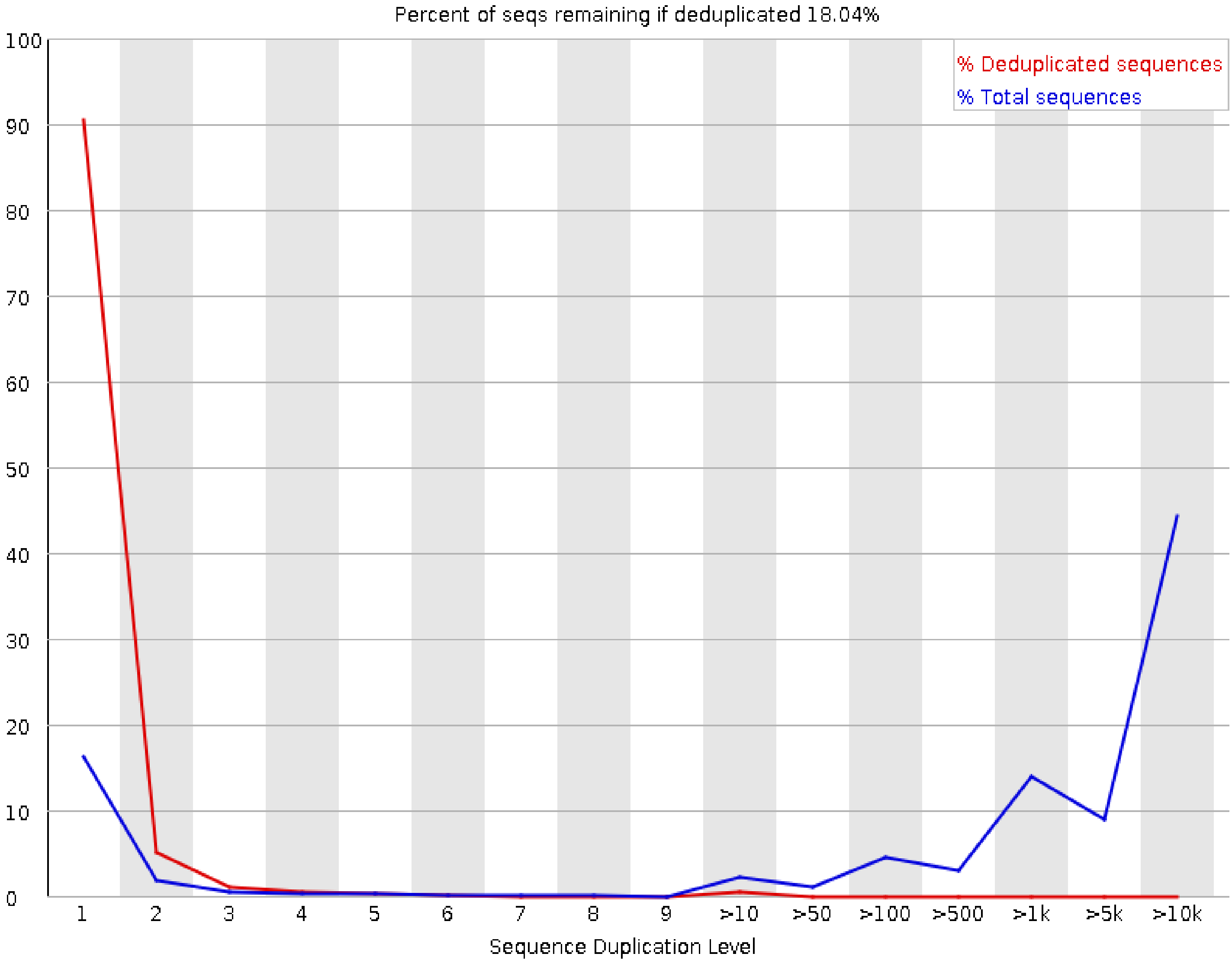


## Overrepresented sequences

| Sequence | Count | Percentage | Possible Source |
|---|---|---|---|
| CTTCCGTACGCCACATGTCCCGCGCCCCGCCGCGGGGCGGGGATTCGGCG | 354949 | 0.4006419680591227 | No Hit |
| CCGGTATTTAGCCTTAGATGGAGTTTACCACCCGCTTTGGGCTGCATTCC | 293542 | 0.3313299786392158 | No Hit |
| CTGGATAGTAGGTAGGGACAGTGGGAATCTCGTTCATCCATTCATGCGCG | 258172 | 0.2914067603451759 | No Hit |
| CTGAATTTAAGCATATTAGTCAGCGGAGGAGAAGAAACTAACCAGGATTC | 236612 | 0.2670713182637651 | No Hit |
| CTTCACCGTGCCAGACTAGAGTCAAGCTCAACAGGGTCTTCTTTCCCCGC | 217264 | 0.24523262933096654 | No Hit |
| CGCAGTTTTATCCGGTAAAGCGAATGATTAGAGGTCTTGGGGCCGAAACG | 216280 | 0.24412195794840125 | No Hit |
| CTCCCTTTCGATCGGCCGAGGGCAACGGAGGCCATCGCCCGTCCCTTCGG | 205904 | 0.23241024426395232 | No Hit |
| CCCGTCGGCATGTATTAGCTCTAGAATTACCACAGTTATCCAAGTAGGAG | 193191 | 0.21806068604591078 | No Hit |
| CTTGAACTCTCTCTTCAAAGTTCTTTTCAACTTTCCCTTACGGTACTTGT | 188785 | 0.21308749690812337 | No Hit |
| CTCTCTTCAAAGTTCTTTTCAACTTTCCCTTACGGTACTTGTTGACTATC | 188544 | 0.21281547271788126 | No Hit |
| CTGCCAGTAGCATATGCTTGTCTCAAAGATTAAGCCATGCATGTCTAAGT | 177051 | 0.19984296641724794 | No Hit |

| Sequence | Count | Percentage | Possible Source |
|---|---|---|---|
| CGGGTCTTCCGTACGCCACATGTCCCGCGCCCCGCCGCGGGGCGGGGATT | 176546 | 0.1992729572219273 | No Hit |
| GTCAAAGTGAAGAAATTCAATGAAGCGCGGGTAAACGGCGGGAGTAACTA | 174537 | 0.19700533648252314 | No Hit |
| CGCGTAACTAGTTAGCATGCCAGAGTCTCGTTCGTTATCGGAATTAACCA | 171674 | 0.19377377940093318 | No Hit |
| CAGAAACCTCCCGTGGAGCAGAAGGGCAAAAGCTCGCTTGATCTTGATTT | 167236 | 0.18876447086859086 | No Hit |
| CCTCACCCGGCCCGGACACGGACAGGATTGACAGATTGATAGCTCTTTCT | 166837 | 0.18831410716773359 | No Hit |
| CTCTCATGTCTCTTCACCGTGCCAGACTAGAGTCAAGCTCAACAGGGTCT | 163682 | 0.18475296061082955 | No Hit |
| CGCGATGTGATTTCTGCCCAGTGCTCTGAATGTCAAAGTGAAGAAATTCA | 160692 | 0.18137805468209955 | No Hit |
| CCCATATCCGCAGCAGGTCTCCAAGGTGAACAGCCTCTGGCATGTTGGAA | 159968 | 0.18056085338029337 | No Hit |
| CCCGAAGTTACGGATCCGGCTTGCCGACTTCCCTTACCTACATTGTTCCA | 156567 | 0.176722038977748 | No Hit |
| CCGACATCGAAGGATCAAAAAGCGACGTCGCTATGAACGCTTGGCCGCCA | 145199 | 0.163890624062095 | No Hit |
| CTCGCATTCCACGCCCGGCTCCACGCCAGCGAGCCGGGCTTCTTACCCAT | 144065 | 0.1626106430175533 | No Hit |
| GTAAATCTCGCGCCGGGCCGTACCCATATCCGCAGCAGGTCTCCAAGGTG | 138155 | 0.1559398423356823 | No Hit |
| CTCCGCCACTCCGGATTCGGGGATCTGAACCCGACTCCCTTTCGATCGGC | 136539 | 0.15411581291065635 | No Hit |
| CTTGTCTCAAAGATTAAGCCATGCATGTCTAAGTACGCACGGCCGGTACA | 135810 | 0.15329296795345096 | No Hit |
| CTCATGTCTCTTCACCGTGCCAGACTAGAGTCAAGCTCAACAGGGTCTTC | 129709 | 0.14640657963533002 | No Hit |
| CACCCGTTTACCTCTTAACGGTTTCACGCCCTCTTGAACTCTCTCTTCAA | 128526 | 0.14507129076787598 | No Hit |
| CGCGTCACTAATTAGATGACGAGGCATTTGGCTACCTTAAGAGAGTCATA | 125142 | 0.14125166479368795 | No Hit |
| CGAAGGCCCGCGGCGGGTGTTGACGCGATGTGATTTCTGCCCAGTGCTCT | 124961 | 0.1410473644682364 | No Hit |
| CTCACCCGGCCCGGACACGGACAGGATTGACAGATTGATAGCTCTTTCTC | 121152 | 0.13674802778511516 | No Hit |
| CTTAGAGCCAATCCTTATCCCGAAGTTACGGATCCGGCTTGCCGACTTCC | 121097 | 0.1366859475757238 | No Hit |
| CTCGATCAGAAGGACTTGGGCCCCCCACGAGCGGCGCCGGGGAGCGGGTC | 119681 | 0.1350876643666664 | No Hit |
| CTGCTGTCTATATCAACCAACACCTTTTCTGGGGTCTGATGAGCGTCGGC | 119226 | 0.1345740917253379 | No Hit |
| CTTAGATGGAGTTTACCACCCGCTTTGGGCTGCATTCCCAAGCAACCCGA | 116869 | 0.13191367257014844 | No Hit |
| CCACTCTCGACTGCCGGCGACGGCCGGGTATGGGCCCGACGCTCCAGCGC | 116631 | 0.13164503457314586 | No Hit |
| CCCGCTTTGGGCTGCATTCCCAAGCAACCCGACTCCGGGAAGACCCGGGC | 115525 | 0.1303966579988397 | No Hit |
| CCCAGGCATAGTTCACCATCTTTCGGGTCCTAACACGTGCGCTCGTGCTC | 115405 | 0.13026121026925858 | No Hit |
| CAGACGTGGCGACCCGCTGAATTTAAGCATATTAGTCAGCGGAGGAGAAG | 113574 | 0.1281945036620664 | No Hit |
| CTCCACTTCGGCCTTCAAAGTTCTCGTTTGAATATTTGCTACTACCACCA | 111060 | 0.1253568737273416 | No Hit |
| CGAGATTCCCACTGTCCCTACCTACTATCCAGCGAAACCACAGCCAAGGG | 110436 | 0.12465254553351969 | No Hit |
| CCCACTTATTCTACACCTCTCATGTCTCTTCACCGTGCCAGACTAGAGTC | 105748 | 0.11936105423121664 | No Hit |
| CTTTAAATGGGTAAGAAGCCCGGCTCGCTGGCGTGGAGCCGGGCGTGGAA | 105645 | 0.11924479492999283 | No Hit |
| CCTGTGGTAACTTTTCTGACACCTCCTGCTTAAAACCCAAAAGGTCAGAA | 105358 | 0.11892084911007794 | No Hit |
| CAAAGATTAAGCCATGCATGTCTAAGTACGCACGGCCGGTACAGTGAAAC | 105189 | 0.11873009355758452 | No Hit |
| CTTGGCTGTGGTTTCGCTGGATAGTAGGTAGGGACAGTGGGAATCTCGTT | 104388 | 0.11782597996263043 | No Hit |
| CGAGAACTTTGAAGGCCGAAGTGGAGAAGGGTTCCATGTGAACAGCAGTT | 104148 | 0.11755508450346816 | No Hit |
| CTCCCACTTATTCTACACCTCTCATGTCTCTTCACCGTGCCAGACTAGAG | 103578 | 0.11691170778795774 | No Hit |
| GTCGGCATGTATTAGCTCTAGAATTACCACAGTTATCCAAGTAGGAGAGG | 102882 | 0.11612611095638717 | No Hit |
| CTCCCGTCCACTCTCGACTGCCGGCGACGGCCGGGTATGGGCCCGACGCT | 101015 | 0.11401877003032064 | No Hit |
| CCCGGGGCTCCCGCCGGCTTCTCCGGGATCGGTCGCGTTACCGCACTGGA | 99584 | 0.1124035558550656 | No Hit |

| Sequence | Count | Percentage | Possible Source |
|---|---|---|---|
| GCCCTCTTGAACTCTCTCTTCAAAGTTCTTTTCAACTTTCCCTTACGGTA | 97943 | 0.11055130815304356 | No Hit |
| GCTGAATTTAAGCATATTAG[illegible]AGAAGAAACTAACCAGGATT | [illegible] | 0.10[illegible]2143 | No Hit |
| CCCCGCTTCGCGCCCCAGCCCGACCGACCCAGCCCTTAGAGCCAATCCTT | 94869 | 0.10708158881360678 | No Hit |
| CTGAATGTCAAAGTGAAGAAATTCAATGAAGCGCGGGTAAACGGCGGGAG | 93060 | 0.10503971429017116 | No Hit |
| CGAACGCCGGGTTAAGGCGCCCGATGCCGACGCTCATCAGACCCCAGAAA | 92755 | 0.1046954513108191 | No Hit |
| GAACAATCCAACGCTTGGTGAATTCTGCTTCACAATGATAGGAAGAGCCG | 92247 | 0.10412205592225897 | No Hit |
| CTCCGACTTTCGTTCTTGATTAATGAAAACATTCTTGGCAAATGCTTTCG | 90364 | 0.10199665529891497 | No Hit |
| CTCCATCTAAGGCTAAATACCGGCACGAGACCGATAGTCAACAAGTACCG | 88810 | 0.10024260720083926 | No Hit |

## Adapter Content

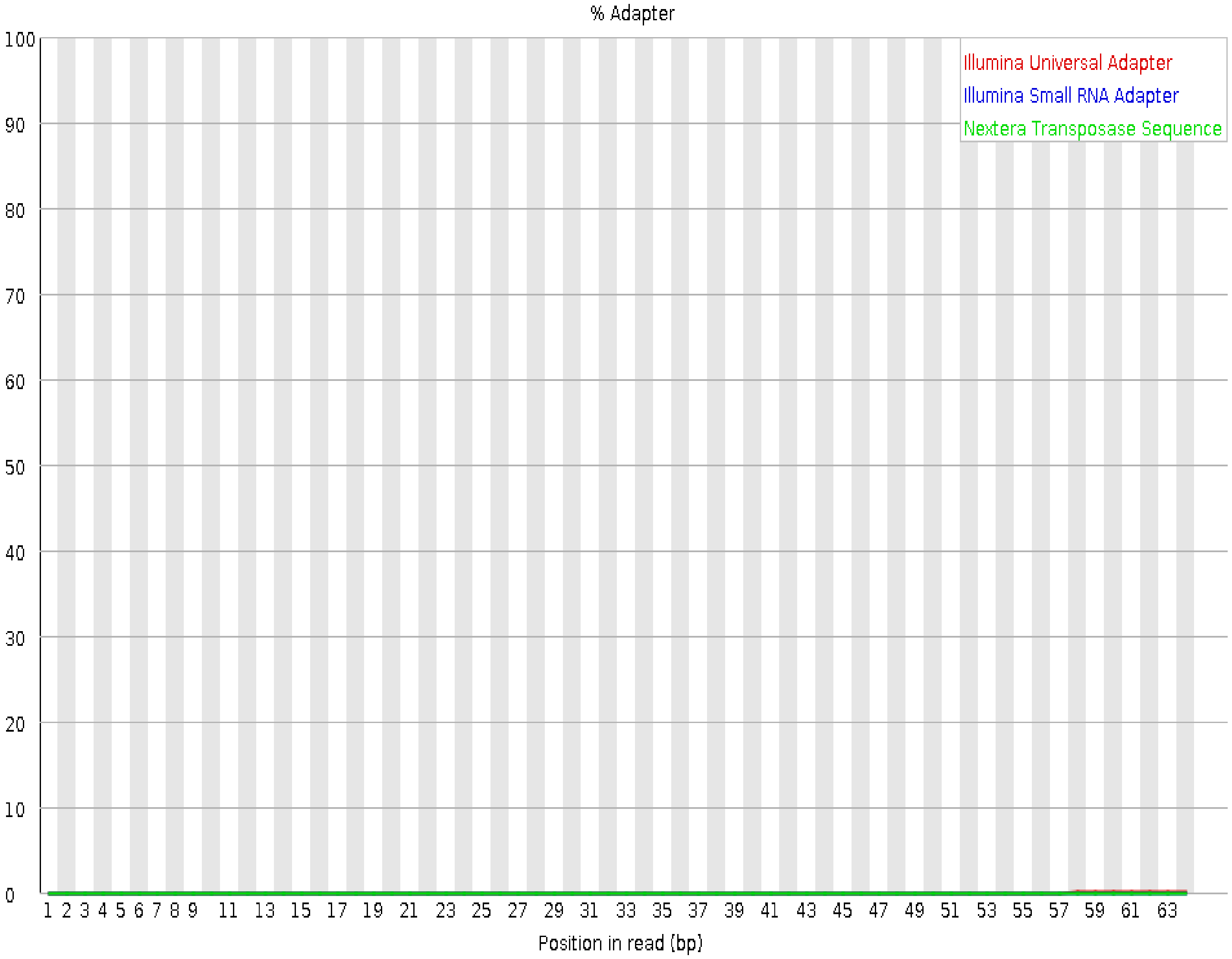


## Kmer Content

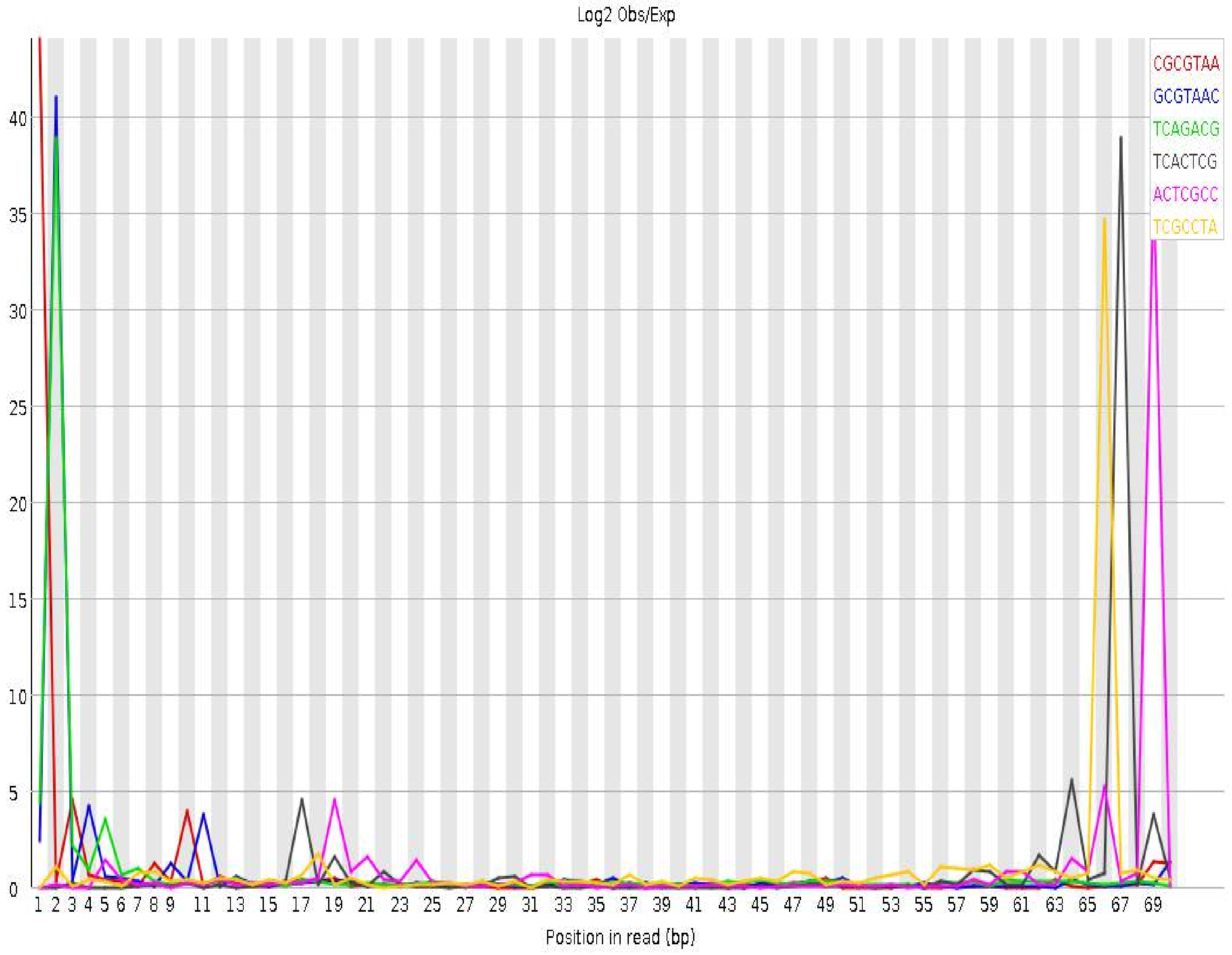


| Sequence | Count | PValue | Obs/Exp Max | Max Obs/Exp Position |
|---|---|---|---|---|
| CGCGTAA | 30545 | 0.0 | 44.053288 | 1 |
| GCGTAAC | 32780 | 0.0 | 41.06835 | 2 |
| TCAGACG | 16770 | 0.0 | 38.95816 | 2 |
| TCACTCG | 70425 | 0.0 | 38.902943 | 67 |
| ACTCGCC | 75110 | 0.0 | 36.57296 | 69 |
| TCGCCTA | 6040 | 0.0 | 34.64964 | 66 |
| TTCACTC | 79745 | 0.0 | 34.363132 | 66 |
| TATCGCC | 6130 | 0.0 | 34.311806 | 64 |
| CTATCGC | 6200 | 0.0 | 33.81211 | 63 |
| CCAGTAG | 41215 | 0.0 | 33.41123 | 4 |
| TCCAACG | 22075 | 0.0 | 33.37162 | 7 |
| CGCCTAT | 6310 | 0.0 | 33.05791 | 67 |
| AGTTAGC | 41185 | 0.0 | 32.972996 | 10 |
| TAGTTAG | 41950 | 0.0 | 32.661762 | 9 |
| GTTAGCA | 41465 | 0.0 | 32.549694 | 11 |

| Sequence | Count | PValue | Obs/Exp Max | Max Obs/Exp Position |
|---|---|---|---|---|
| AGACGTG | 32450 | 0.0 | 31.[illegible] | 2 |
| ACTAGTT | 44610 | 0.0 | 30.8[illegible] | 7 |
| AACTAGT | 44320 | 0.0 | 30.77369 | 6 |
| CAACGCT | 23815 | 0.0 | 30.618887 | 9 |
| CCAACGC | 24355 | 0.0 | 30.079035 | 8 |

# FastQC Report

Mon 4 May 2015
Origene_Adult_Stomach_0393_CAGATC_L006_R1.fastq.gz

## Summary


- [PASS] Basic Statistics
- [PASS] Per base sequence quality
- [FAIL] Per tile sequence quality
- [PASS] Per sequence quality scores
- [FAIL] Per base sequence content
- [WARN] Per sequence GC content
- [PASS] Per base N content
- [PASS] Sequence Length Distribution
- [FAIL] Sequence Duplication Levels
- [WARN] Overrepresented sequences
- [PASS] Adapter Content
- [FAIL] Kmer Content


## Basic Statistics

| Measure | Value |
|---|---|
| Filename | Origene_Adult_Stomach_0393_CAGATC_L006_R1.fastq.gz |
| File type | Conventional base calls |
| Encoding | Sanger / Illumina 1.9 |
| Total Sequences | 89447266 |
| Sequences flagged as poor quality | 0 |
| Sequence length | 76 |
| %GC | 54 |

## Per base sequence quality

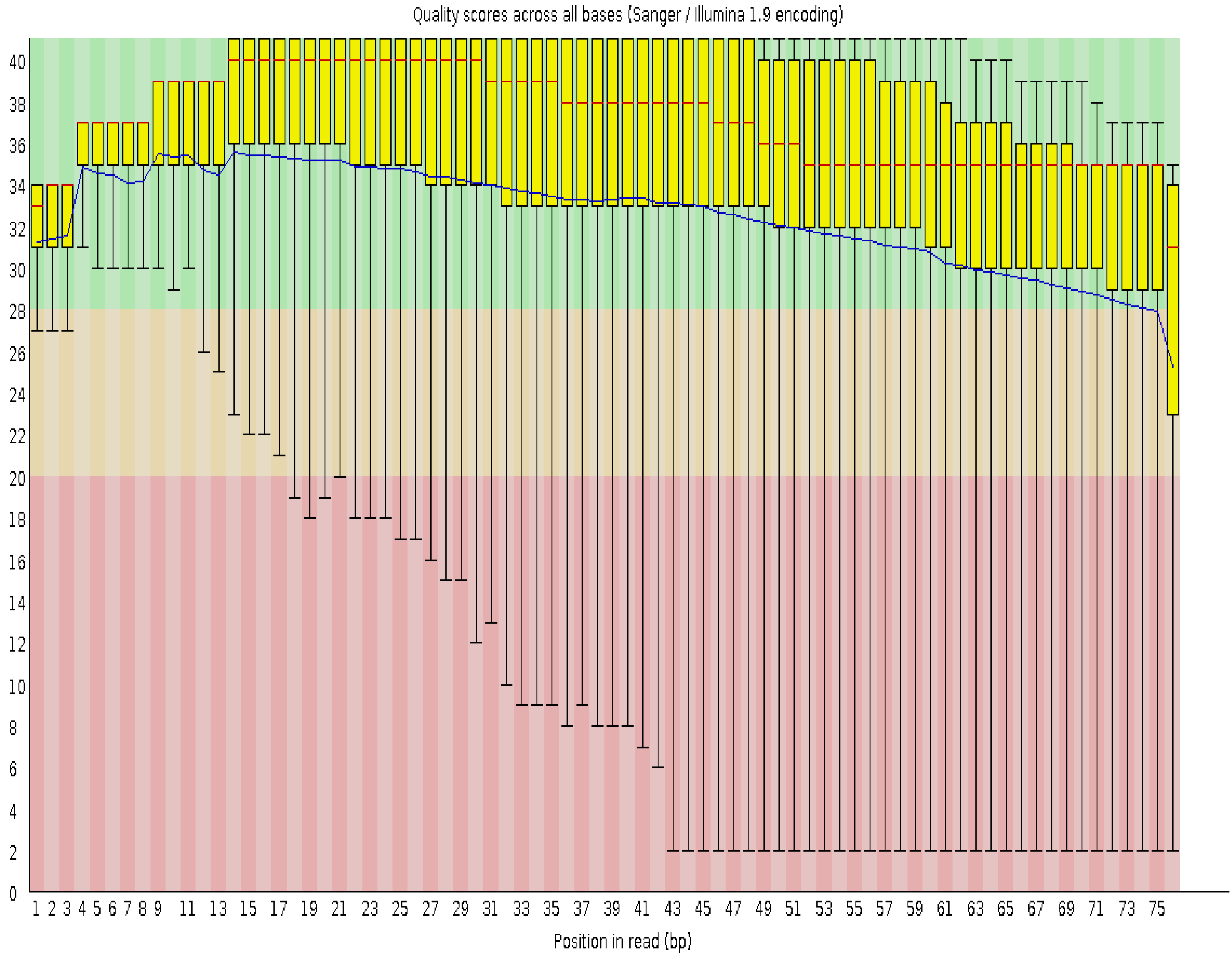


## Per tile sequence quality

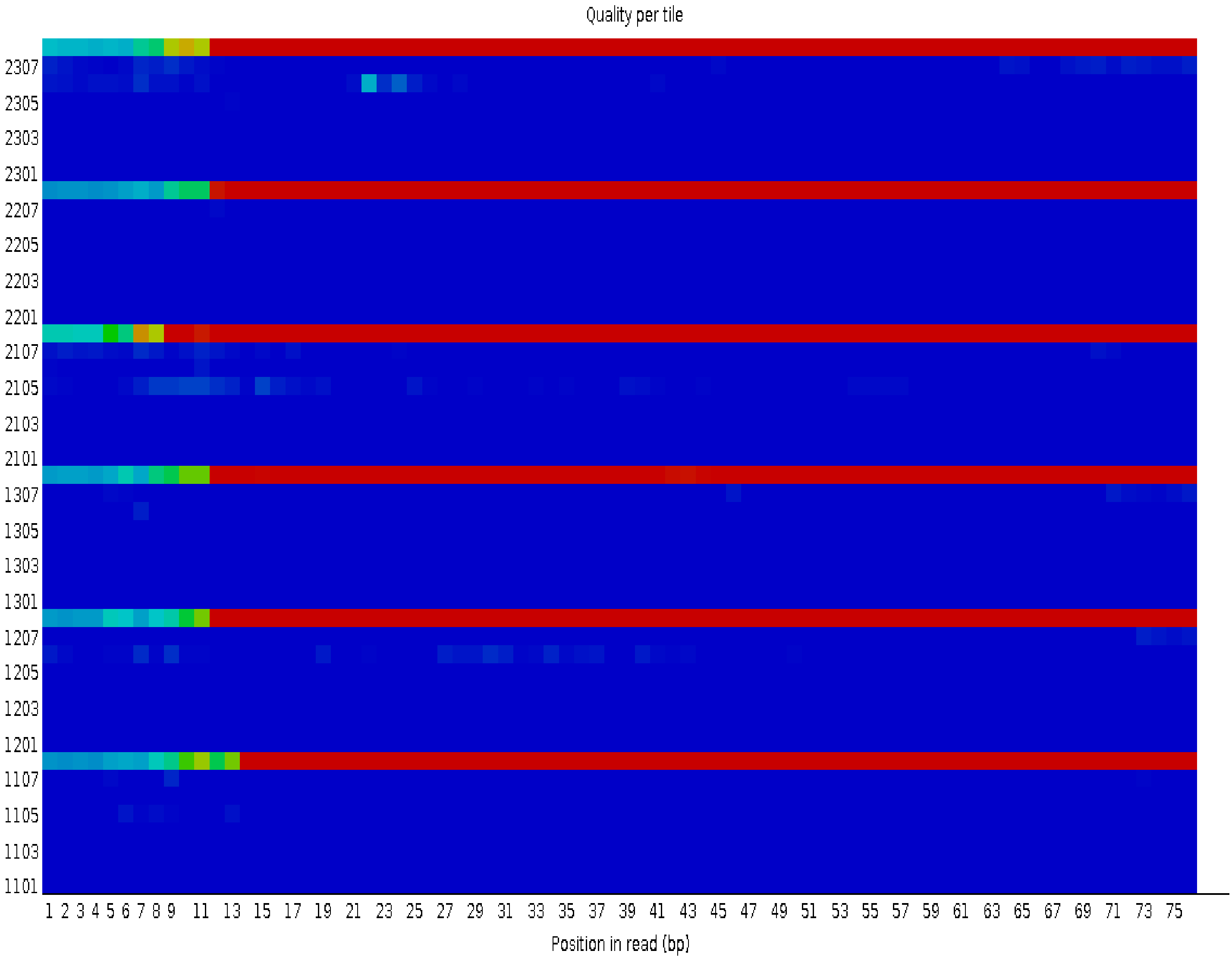


## Per sequence quality scores

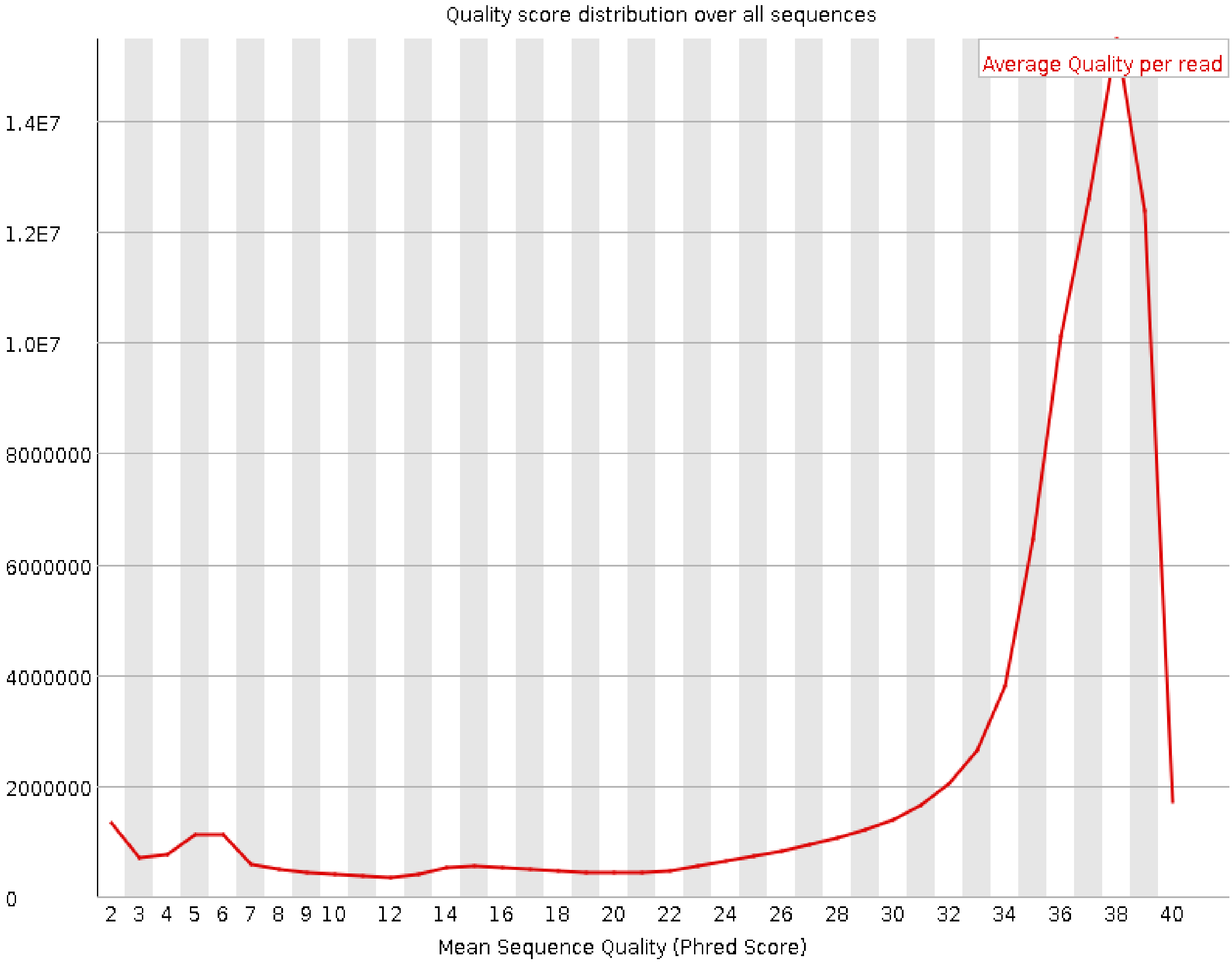


## Per base sequence content

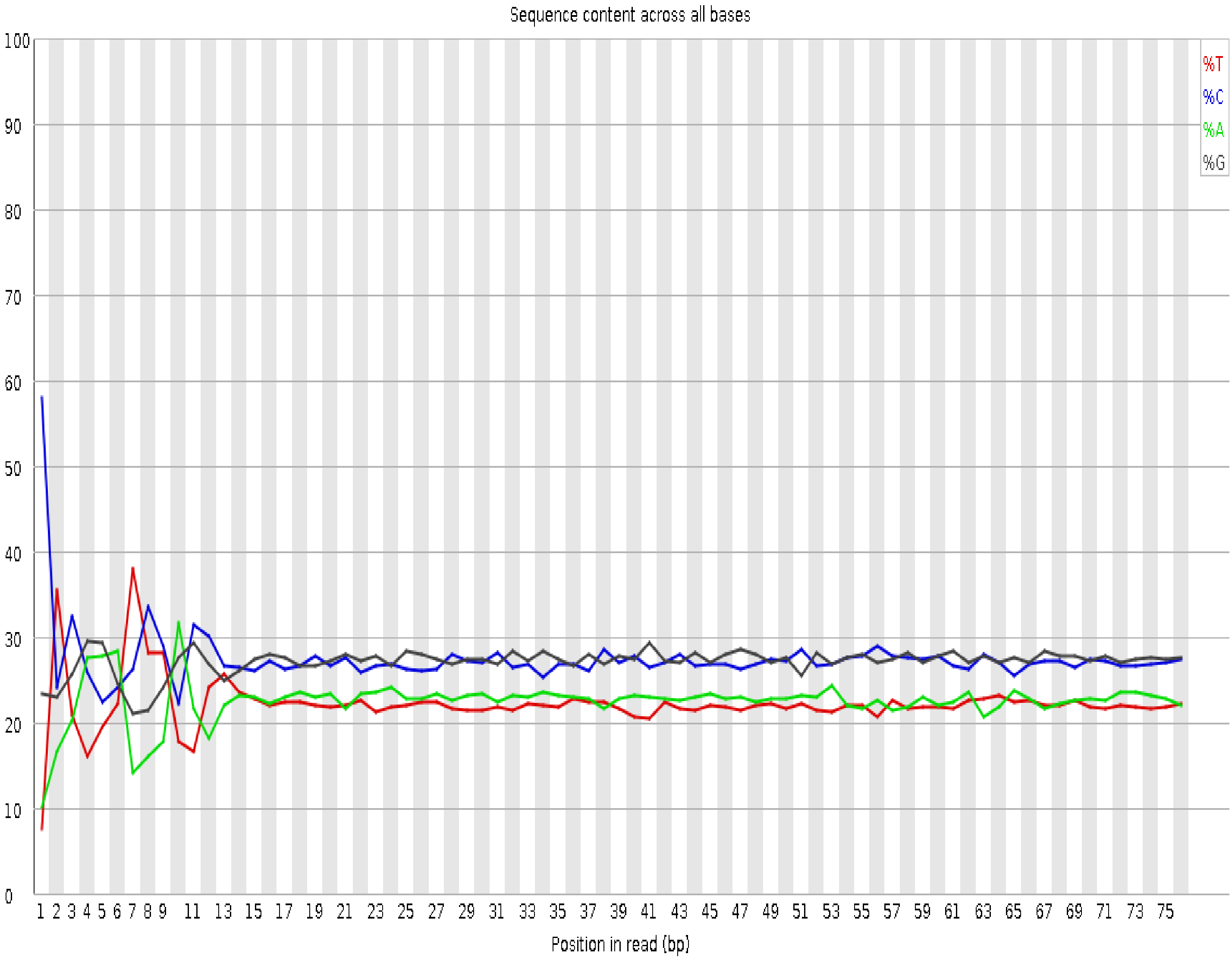


## Per sequence GC content

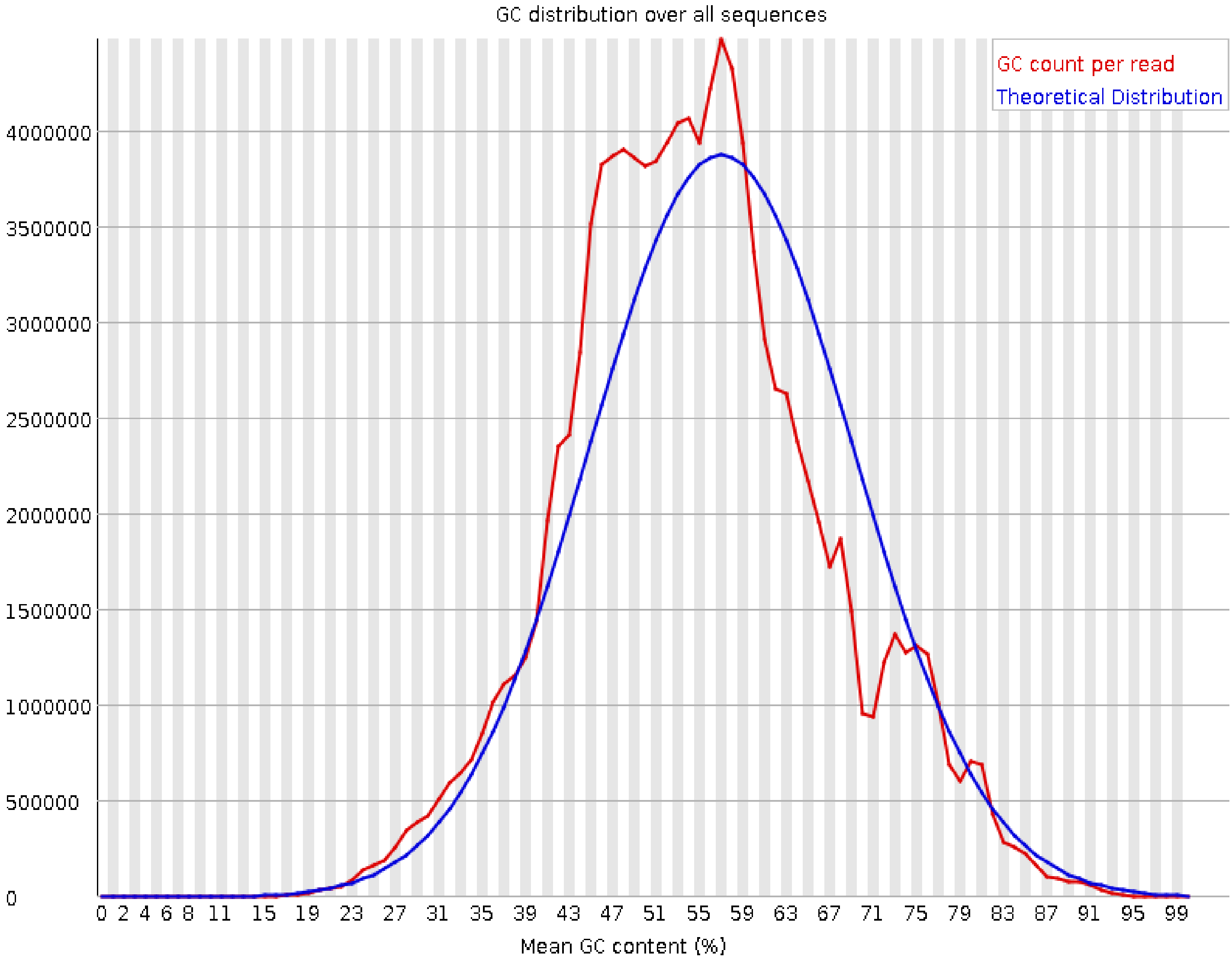


## Per base N content

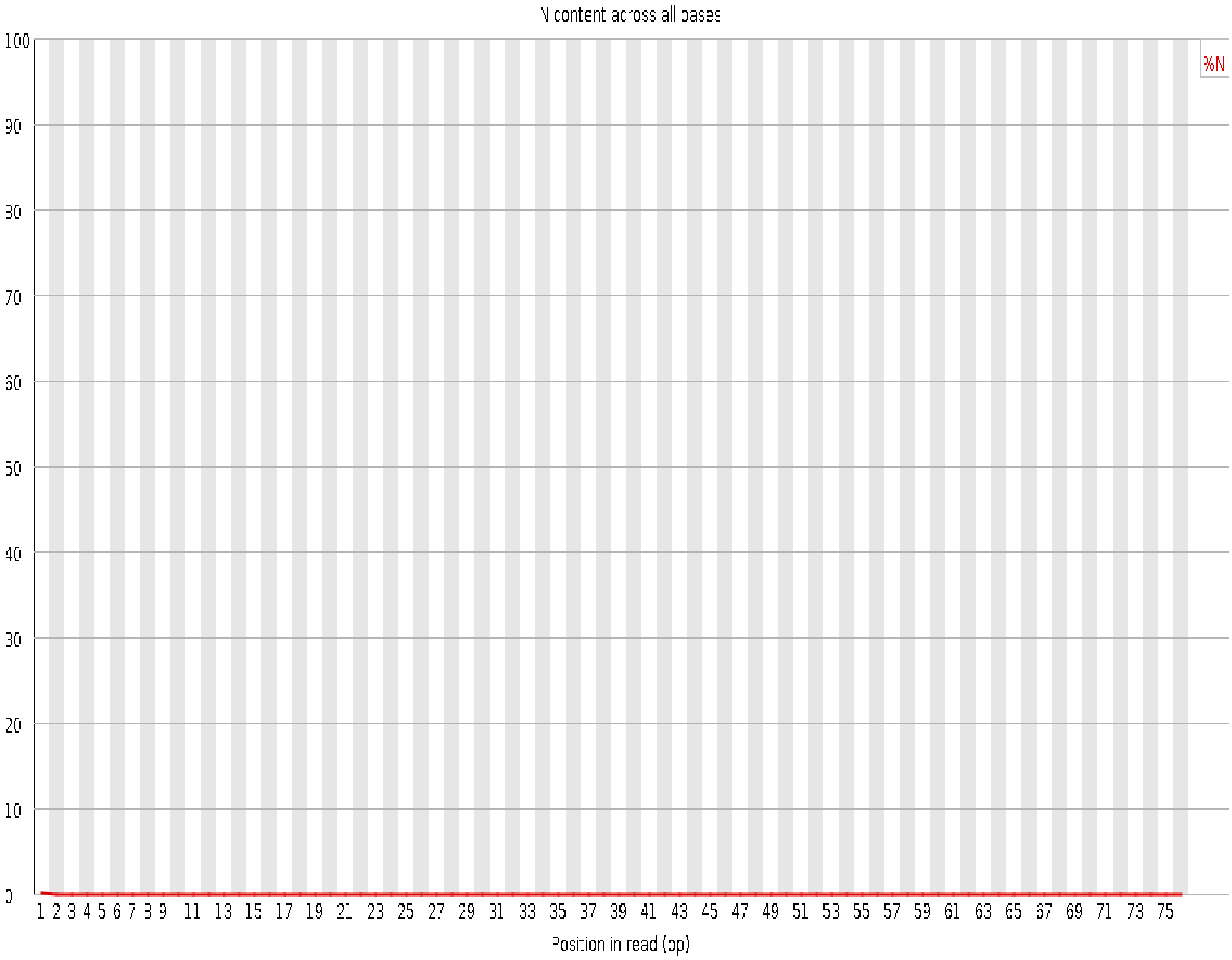


## Sequence Length Distribution

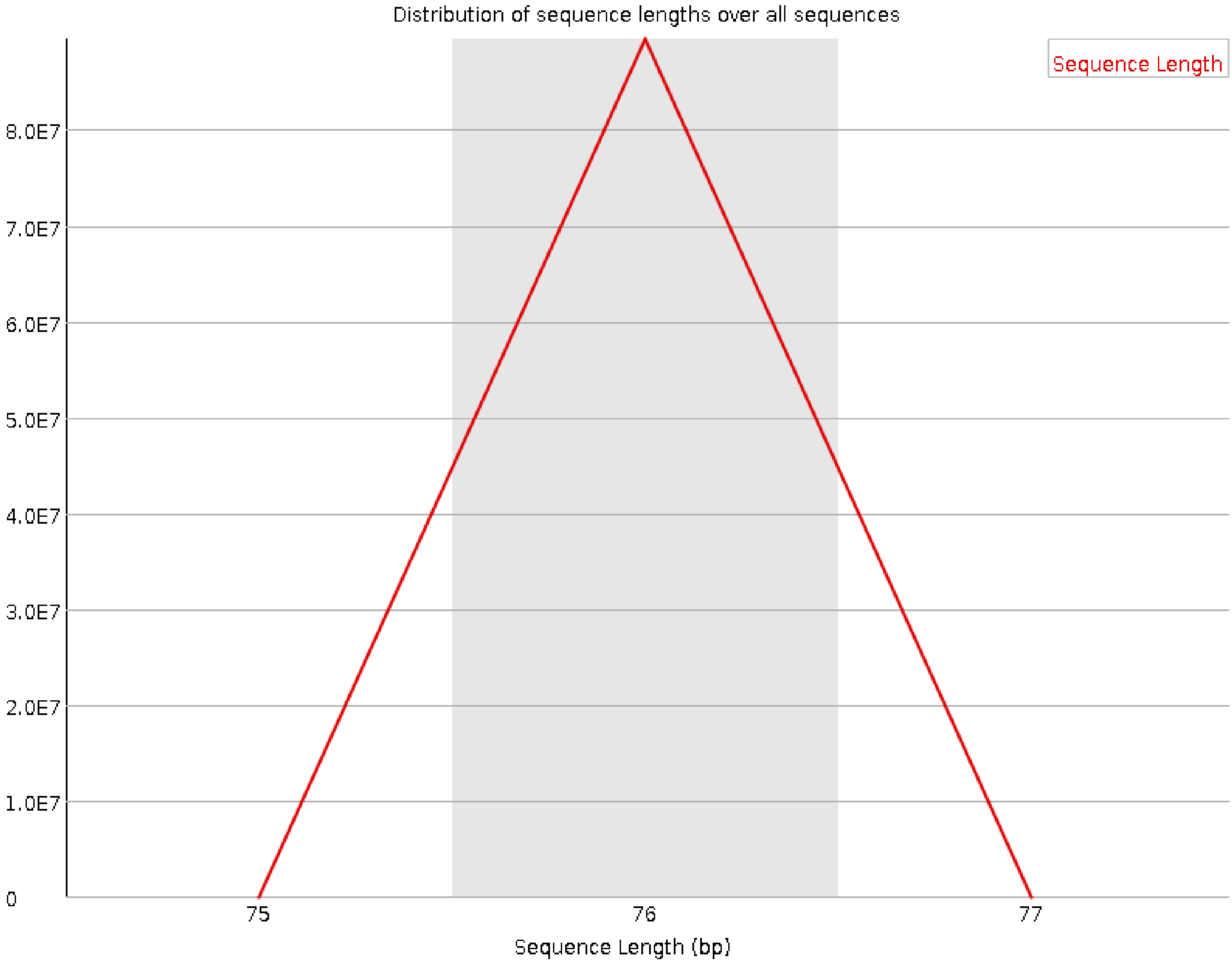


## Sequence Duplication Levels

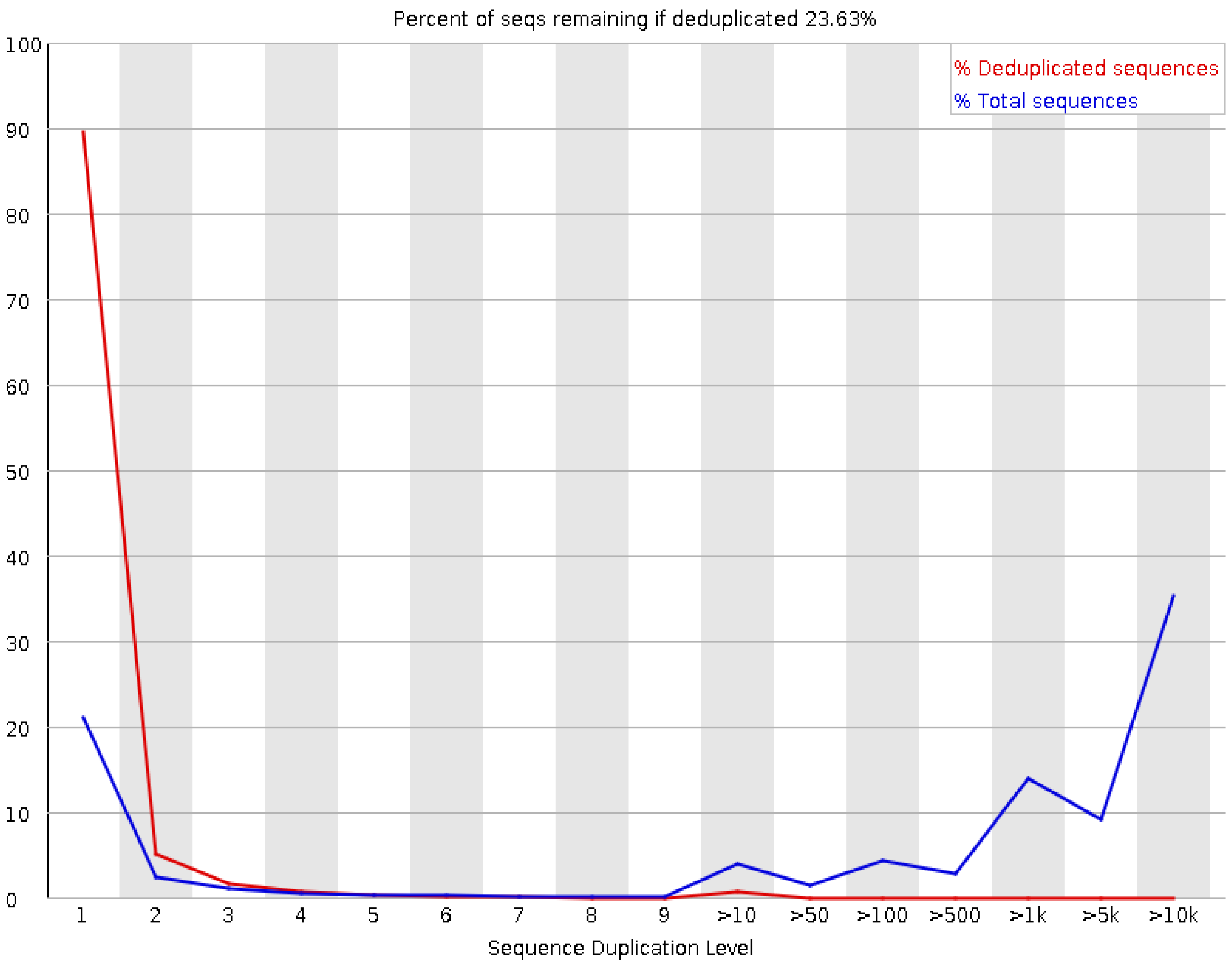


## Overrepresented sequences

| Sequence | Count | Percentage | Possible Source |
|---|---|---|---|
| CCGGTATTTAGCCTTAGATGGAGTTTACCACCCGCTTTGGGCTGCATTCC | 286623 | 0.320437966209051 | No Hit |
| CTTCCGTACGCCACATGTCCCGCGCCCCGCCGCGGGGCGGGGATTCGGCG | 243535 | 0.27226656653765136 | No Hit |
| CTGAATTTAAGCATATTAGTCAGCGGAGGAGAAGAAACTAACCAGGATTC | 220829 | 0.24688177724738955 | No Hit |
| CTGGATAGTAGGTAGGGACAGTGGGAATCTCGTTCATCCATTCATGCGCG | 215707 | 0.24115549825748728 | No Hit |
| CTTGAACTCTCTCTTCAAAGTTCTTTTCAACTTTCCCTTACGGTACTTGT | 191366 | 0.2139428163181645 | No Hit |
| GTCAAAGTGAAGAAATTCAATGAAGCGCGGGTAAACGGCGGGAGTAACTA | 172241 | 0.19256150322135057 | No Hit |
| CAGAAACCTCCCGTGGAGCAGAAGGGCAAAAGCTCGCTTGATCTTGATTT | 171161 | 0.19135408789353048 | No Hit |
| CTTCACCGTGCCAGACTAGAGTCAAGCTCAACAGGGTCTTCTTTCCCCGC | 154253 | 0.17245133015021388 | No Hit |
| CCCGTCGGCATGTATTAGCTCTAGAATTACCACAGTTATCCAAGTAGGAG | 150421 | 0.1681672416907633 6 | No Hit |
| CCCATATCCGCAGCAGGTCTCCAAGGTGAACAGCCTCTGGCATGTTGGAA | 145784 | 0.16298318162122474 | No Hit |
| CCCGAAGTTACGGATCCGGCTTGCCGACTTCCCTTACCTACATTGTTCCA | 144830 | 0.16191663141498366 | No Hit |

| Sequence | Count | Percentage | Possible Source |
|---|---|---|---|
| CGCAGTTTTATCCGGTAAAGCGAATGATTAGAGGTCTTGGGGCCGAAACG | 139106 | 0.15551733017753722 | No Hit |
| CCGACATCGAAGGATCAAAAAGCGACGTCGCTATGAACGCTTGGCCGCCA | 137692 | 0.15393651047981724 | N[illegible] |
| CTCTCTTCAAAGTTCTTTTCAACTTTCCCTTACGGTACTTGTTGACTATC | 137651 | 0.1538906734164463 | No Hit |
| GTAAATCTCGCGCCGGGCCGTACCCATATCCGCAGCAGGTCTCCAAGGTG | 137209 | 0.15339652751376437 | No Hit |
| CTCCCTTTCGATCGGCCGAGGGCAACGGAGGCCATCGCCCGTCCCTTCGG | 136666 | 0.15278946591838816 | No Hit |
| CGCGATGTGATTTCTGCCCAGTGCTCTGAATGTCAAAGTGAAGAAATTCA | 133779 | 0.14956186587078021 | No Hit |
| CTGCCAGTAGCATATGCTTGTCTCAAAGATTAAGCCATGCATGTCTAAGT | 132230 | 0.14783011925708273 | No Hit |
| CTTGTCTCAAAGATTAAGCCATGCATGTCTAAGTACGCACGGCCGGTACA | 126205 | 0.1410943068958642 | No Hit |
| CTCTCATGTCTCTTCACCGTGCCAGACTAGAGTCAAGCTCAACAGGGTCT | 119945 | 0.1340957699031293 | No Hit |
| CTTAGAGCCAATCCTTATCCCGAAGTTACGGATCCGGCTTGCCGACTTCC | 114516 | 0.1280262719265226 | No Hit |
| CGGGTCTTCCGTACGCCACATGTCCCGCGCCCCGCCGCGGGGCGGGGATT | 113026 | 0.12636048596499305 | No Hit |
| CTCGCATTCCACGCCCGGCTCCACGCCAGCGAGCCGGGCTTCTTACCCAT | 112941 | 0.12626545790678498 | No Hit |
| CTTAGATGGAGTTTACCACCCGCTTTGGGCTGCATTCCCAAGCAACCCGA | 110015 | 0.12299425675011688 | No Hit |
| CACCCGTTTACCTCTTAACGGTTTCACGCCCTCTTGAACTCTCTCTTCAA | 108675 | 0.12149616736189567 | No Hit |
| CCCGCTTTGGGCTGCATTCCCAAGCAACCCGACTCCGGGAAGACCCGGGC | 108644 | 0.1214615100700786 | No Hit |
| CCTCACCCGGCCCGGACACGGACAGGATTGACAGATTGATAGCTCTTTCT | 105334 | 0.11776100568574113 | No Hit |
| GTCGGCATGTATTAGCTCTAGAATTACCACAGTTATCCAAGTAGGAGAGG | 104891 | 0.11726574180590382 | No Hit |
| CTCATGTCTCTTCACCGTGCCAGACTAGAGTCAAGCTCAACAGGGTCTTC | 104764 | 0.11712375870716943 | No Hit |
| CGAAGGCCCGCGGCGGGTGTTGACGCGATGTGATTTCTGCCCAGTGCTCT | 103041 | 0.11519748406843425 | No Hit |
| CGAGATTCCCACTGTCCCTACCTACTATCCAGCGAAACCACAGCCAAGGG | 101521 | 0.11349815879224301 | No Hit |
| CTCGATCAGAAGGACTTGGGCCCCCCACGAGCGGCGCCGGGGAGCGGGTC | 97699 | 0.1092252501043464 | No Hit |
| CAAAGATTAAGCCATGCATGTCTAAGTACGCACGGCCGGTACAGTGAAAC | 97050 | 0.10849968293049897 | No Hit |
| CTCCGCCACTCCGGATTCGGGGATCTGAACCCGACTCCCTTTCGATCGGC | 95570 | 0.10684507674052329 | No Hit |
| CCTGTGGTAACTTTTCTGACACCTCCTGCTTAAAACCCAAAAGGTCAGAA | 92122 | 0.10299029150874214 | No Hit |
| GCTGAATTTAAGCATATTAGTCAGCGGAGGAGAAGAAACTAACCAGGATT | 91490 | 0.10228372994653633 | No Hit |
| CTTGGCTGTGGTTTCGCTGGATAGTAGGTAGGGACAGTGGGAATCTCGTT | 90668 | 0.10136475272480658 | No Hit |
| CCACTCTCGACTGCCGGCGACGGCCGGGTATGGGCCCGACGCTCCAGCGC | 90637 | 0.10133009543298953 | No Hit |
| CGAGAACTTTGAAGGCCGAAGTGGAGAAGGGTTCCATGTGAACAGCAGTT | 89876 | 0.10047931481773854 | No Hit |
| CGCGTCACTAATTAGATGACGAGGCATTTGGCTACCTTAAGAGAGTCATA | 89688 | 0.10026913511252541 | No Hit |
| CCCAGGCATAGTTCACCATCTTTCGGGTCCTAACACGTGCGCTCGTGCTC | 89561 | 0.10012715201379102 | No Hit |

## Adapter Content

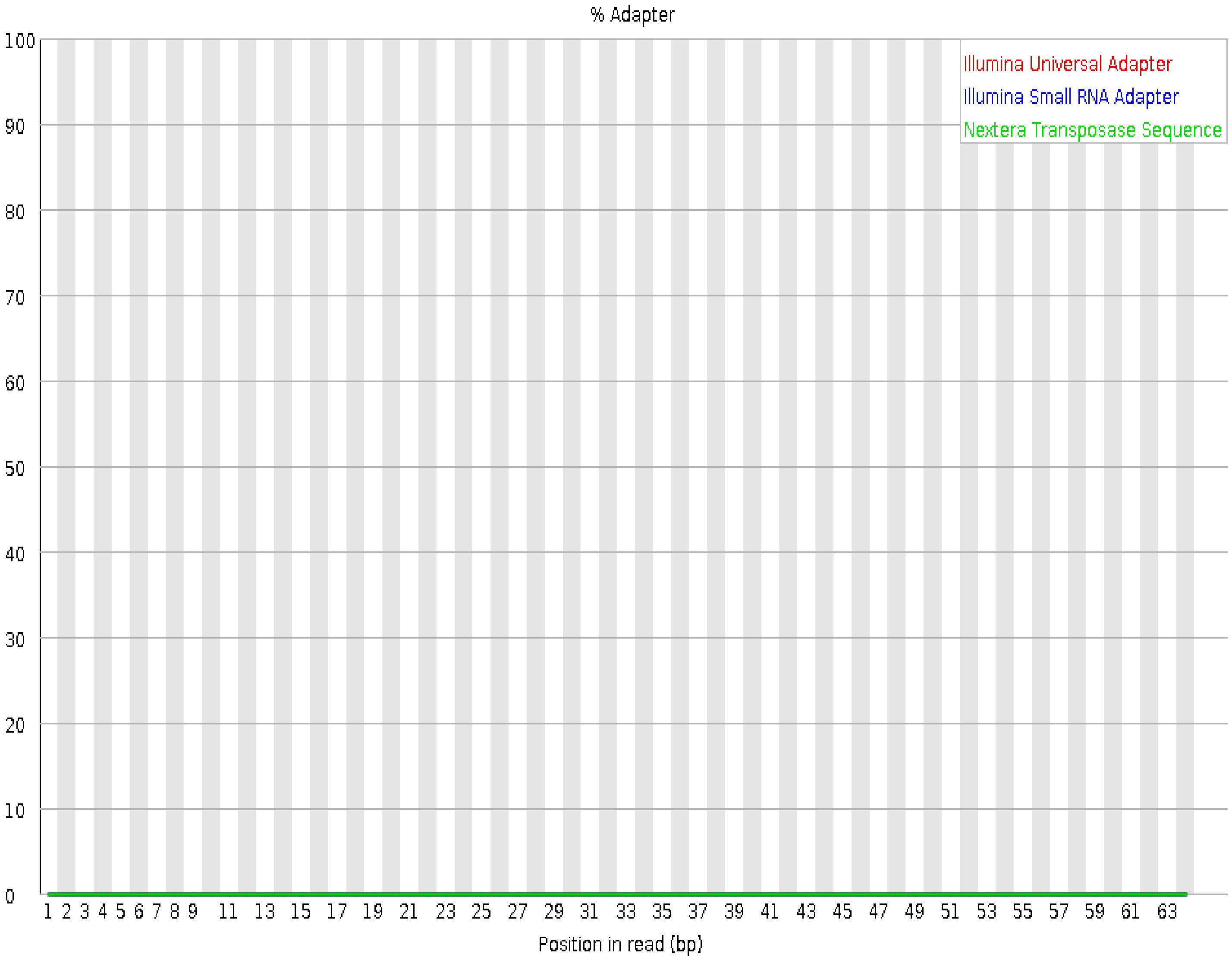


## Kmer Content

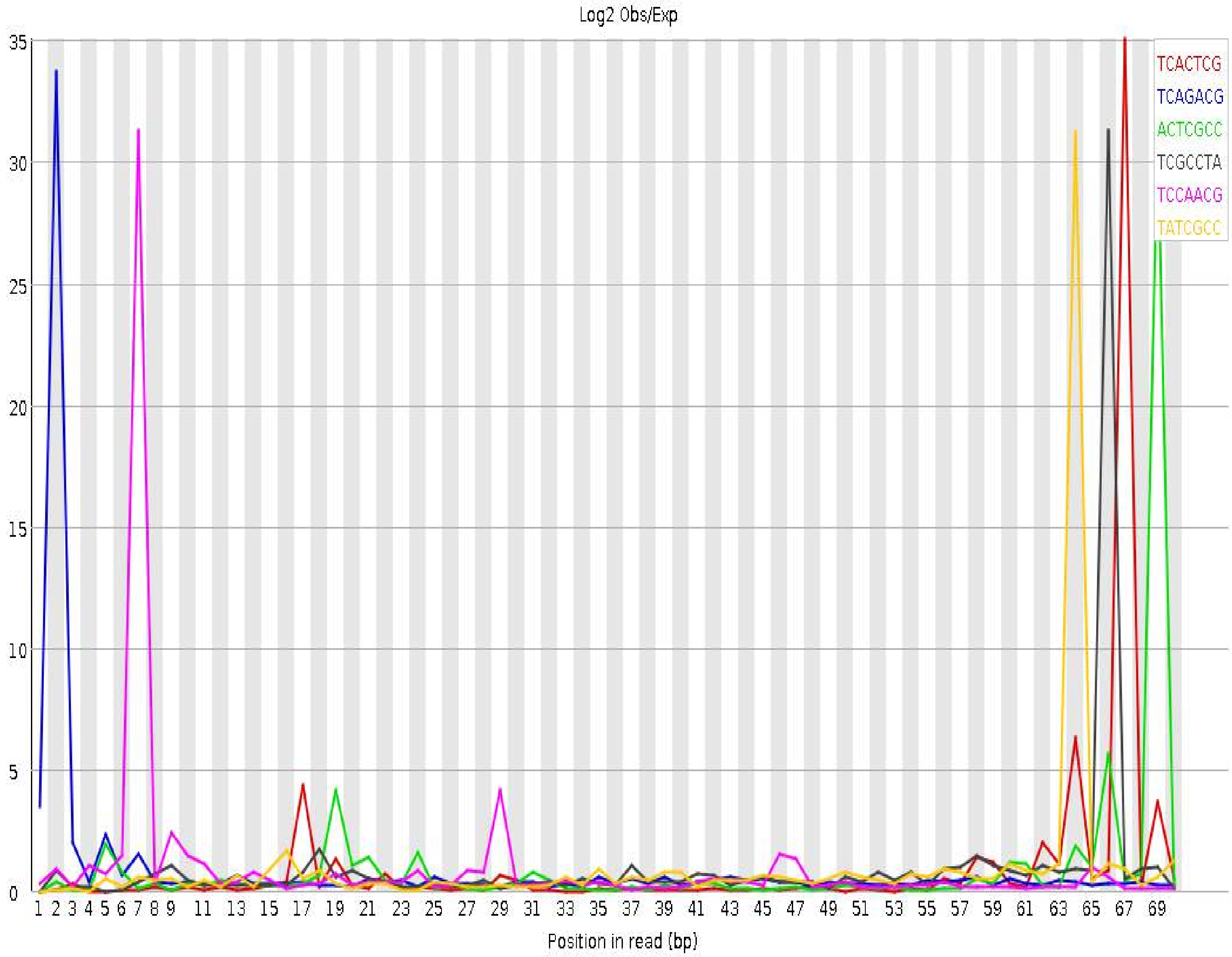


| Sequence | Count | PValue | Obs/Exp Max | Max Obs/Exp Position |
|---|---|---|---|---|
| TCACTCG | 56145 | 0.0 | 35.04593 | 67 |
| TCAGACG | 16420 | 0.0 | 33.702072 | 2 |
| ACTCGCC | 62775 | 0.0 | 31.300316 | 69 |
| TCGCCTA | 7760 | 0.0 | 31.29734 | 66 |
| TCCAACG | 22180 | 0.0 | 31.273716 | 7 |
| TATCGCC | 7815 | 0.0 | 31.256706 | 64 |
| CTATCGC | 7855 | 0.0 | 30.479086 | 63 |
| CGCCTAT | 8060 | 0.0 | 30.217237 | 67 |
| CGCGTAA | 26905 | 0.0 | 28.970371 | 1 |
| TTCACTC | 68080 | 0.0 | 28.914299 | 66 |
| AGACGTG | 29160 | 0.0 | 28.832571 | 2 |
| CAACGCT | 23225 | 0.0 | 28.363594 | 9 |
| CCAACGC | 23705 | 0.0 | 28.126392 | 8 |
| TGCGCGA | 16065 | 0.0 | 28.084686 | 9 |
| GCGTAAC | 28055 | 0.0 | 27.959501 | 2 |

| Sequence | Count | PValue | Obs/Exp Max | Max Obs/Exp Position |
|---|---|---|---|---|
| ACAATCC | 26845 | 0.0 | 27.[illegible] | 3 |
| CCAGTAG | 41045 | 0.0 | 27.49[illegible] | 4 |
| CAATCCA | 28265 | 0.0 | 26.850231 | 4 |
| AACAATC | 28525 | 0.0 | 26.787115 | 2 |
| TTGGCCG | 41510 | 0.0 | 26.567312 | 41 |

**Produced by FastQC (version 0.11.2)**

# FastQC Report

Mon 4 May 2015
Origene_Adult_Stomach_0393_CAGATC_L006_R2.fastq.gz

## Summary


- Basic Statistics
- Per base sequence quality
- Per tile sequence quality
- Per sequence quality scores
- Per base sequence content
- Per sequence GC content
- Per base N content
- Sequence Length Distribution
- Sequence Duplication Levels
- Overrepresented sequences
- Adapter Content
- Kmer Content


## Basic Statistics

| Measure | Value |
|---|---|
| Filename | Origene_Adult_Stomach_0393_CAGATC_L006_R2.fastq.gz |
| File type | Conventional base calls |
| Encoding | Sanger / Illumina 1.9 |
| Total Sequences | 89447266 |
| Sequences flagged as poor quality | 0 |
| Sequence length | 76 |
| %GC | 55 |

## Per base sequence quality

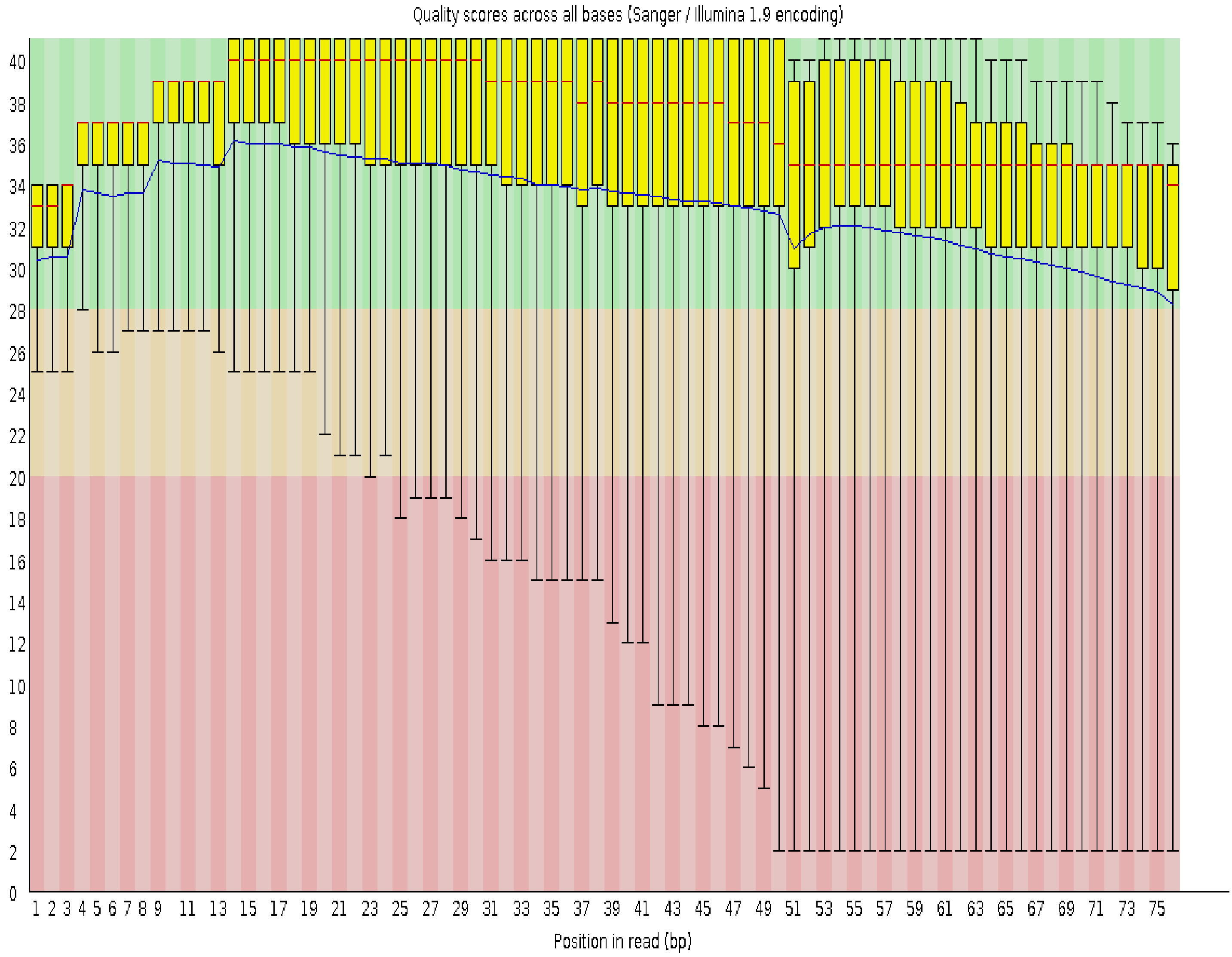


## Per tile sequence quality

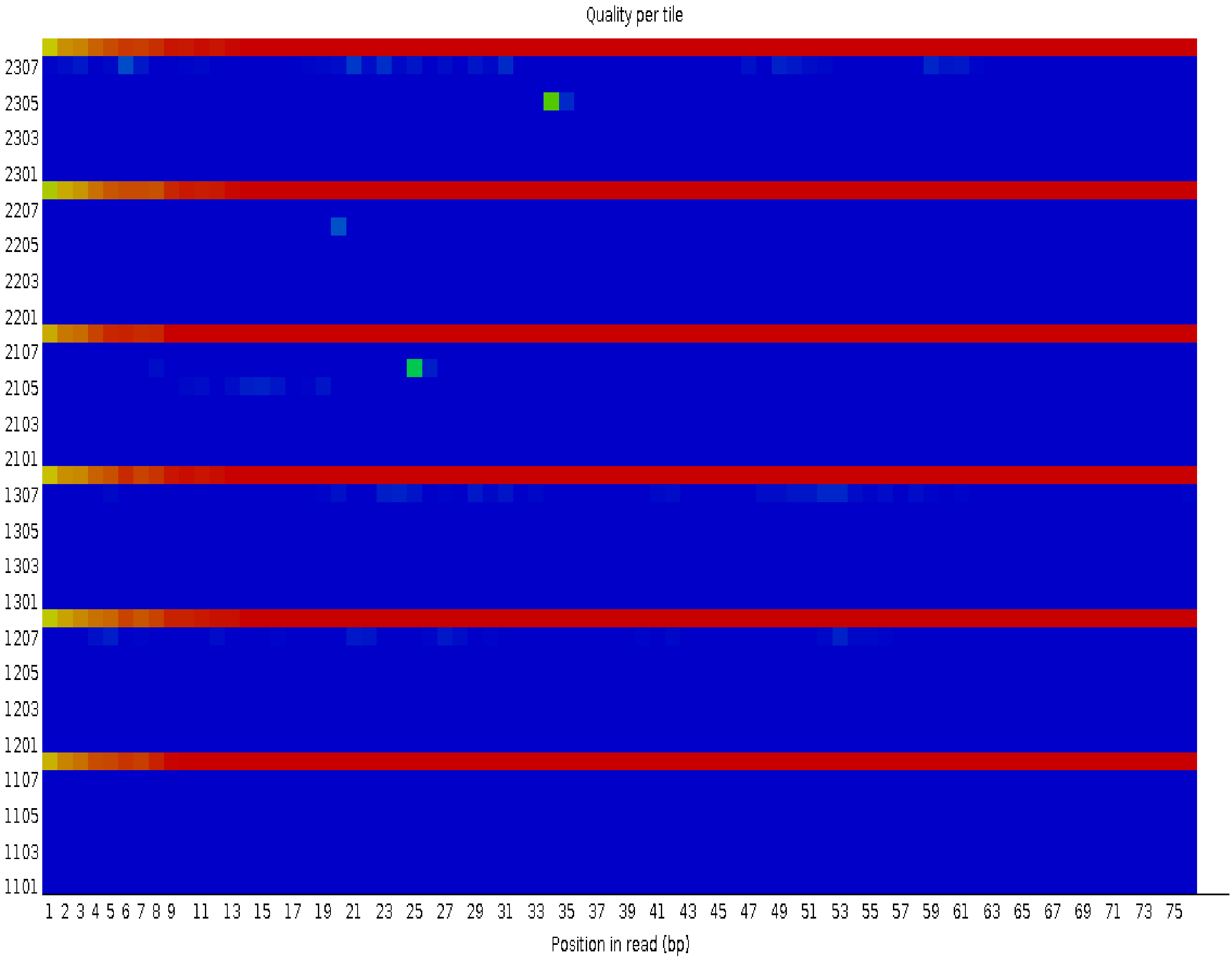


## Per sequence quality scores

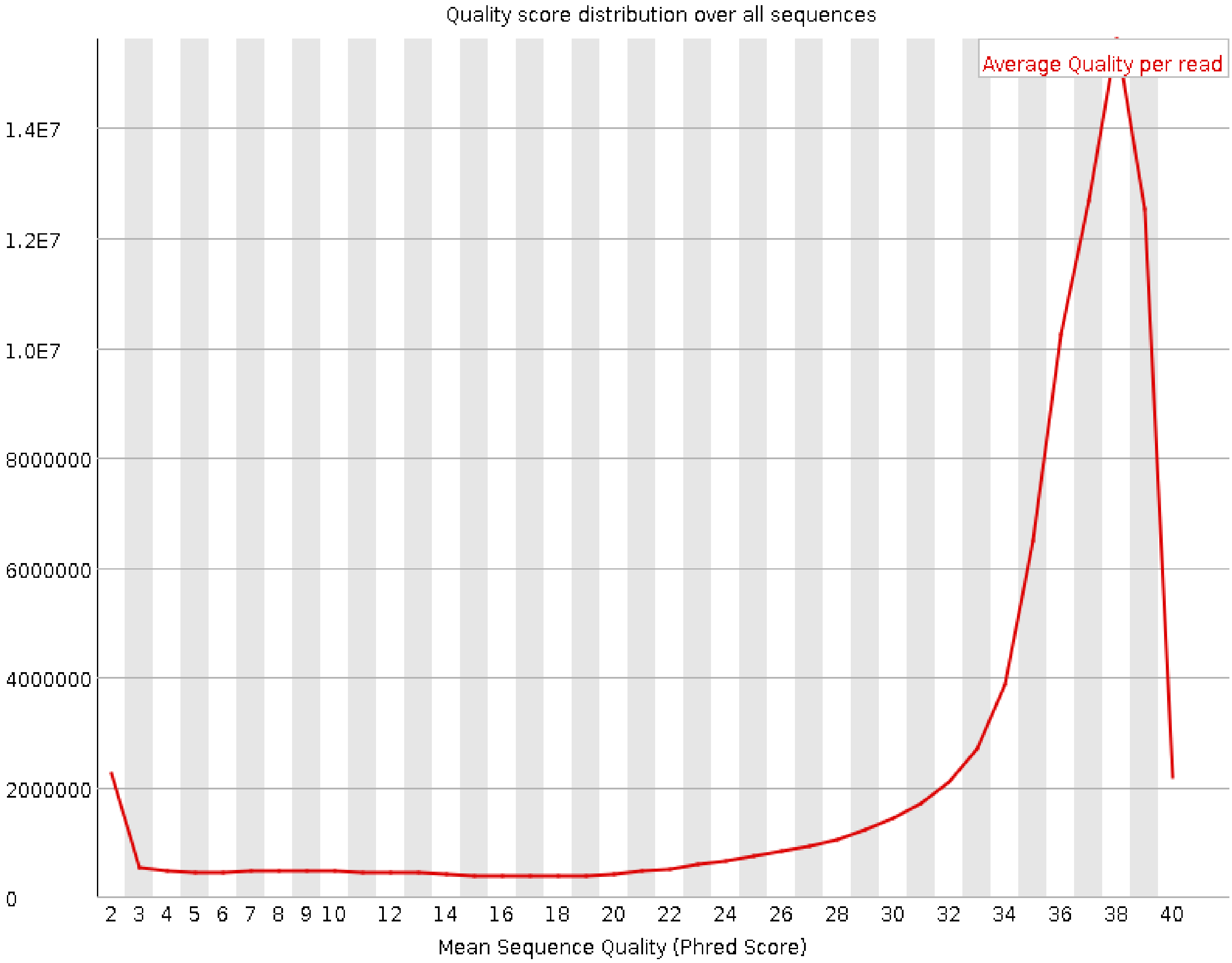


## Per base sequence content

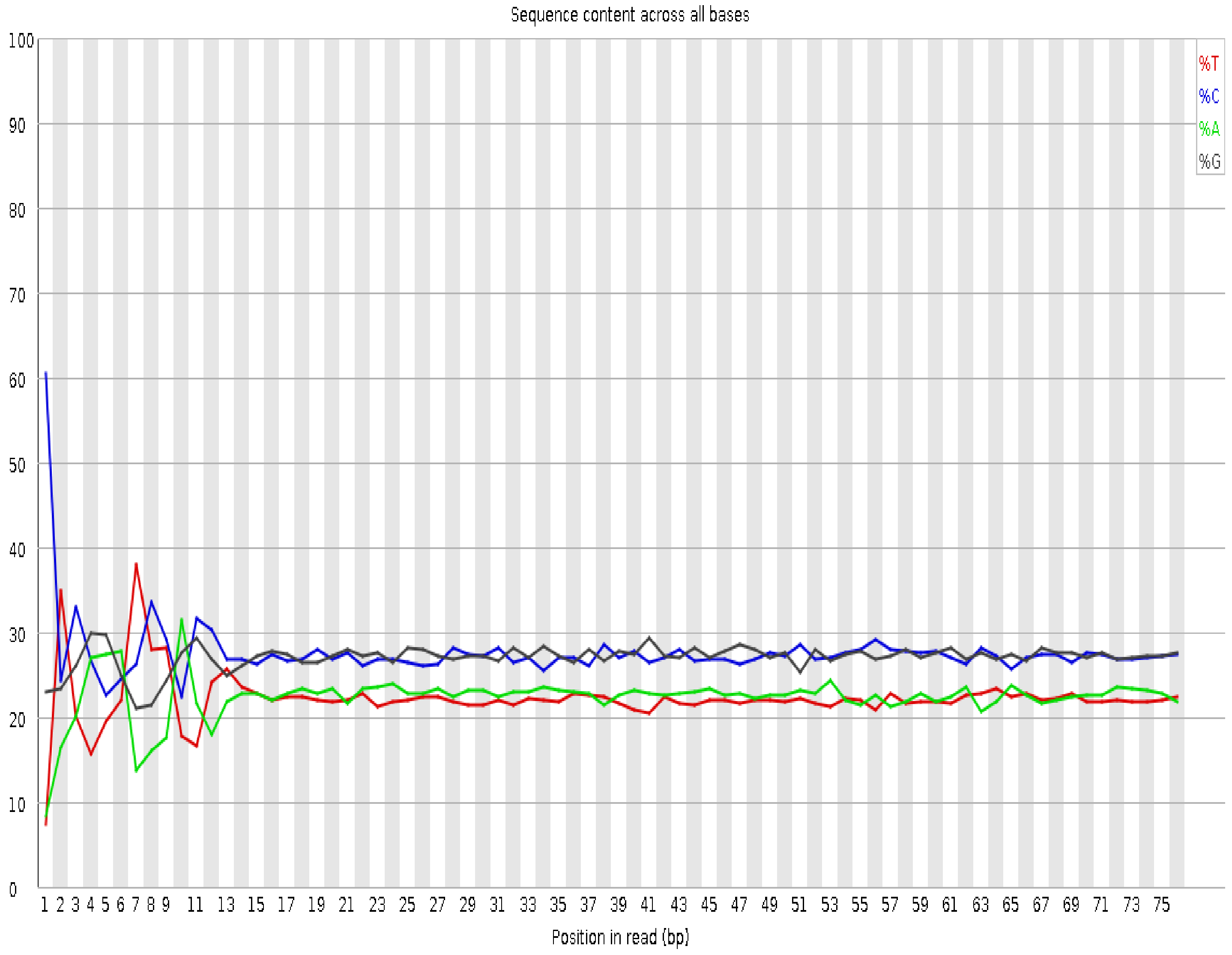


## Per sequence GC content

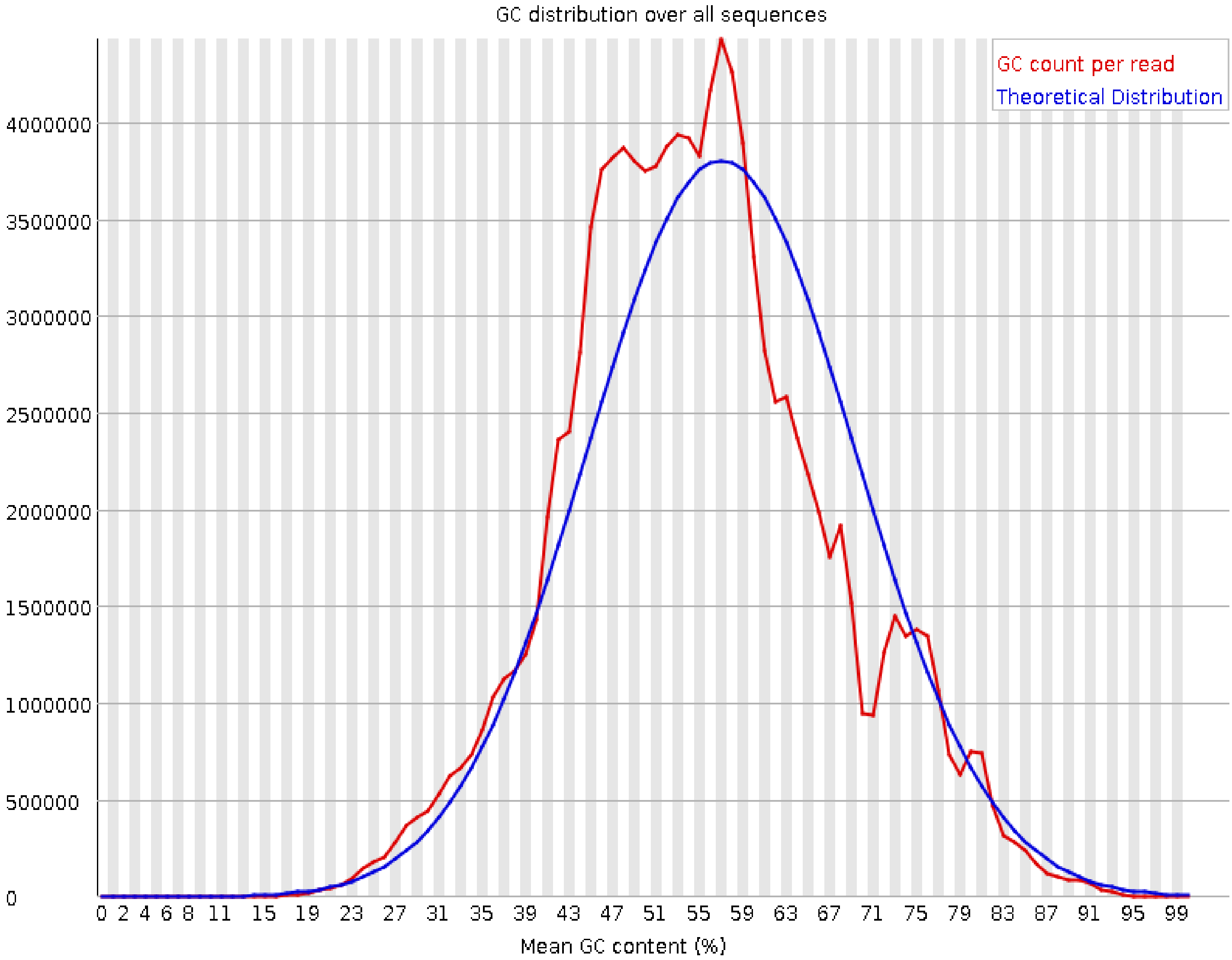


## Per base N content

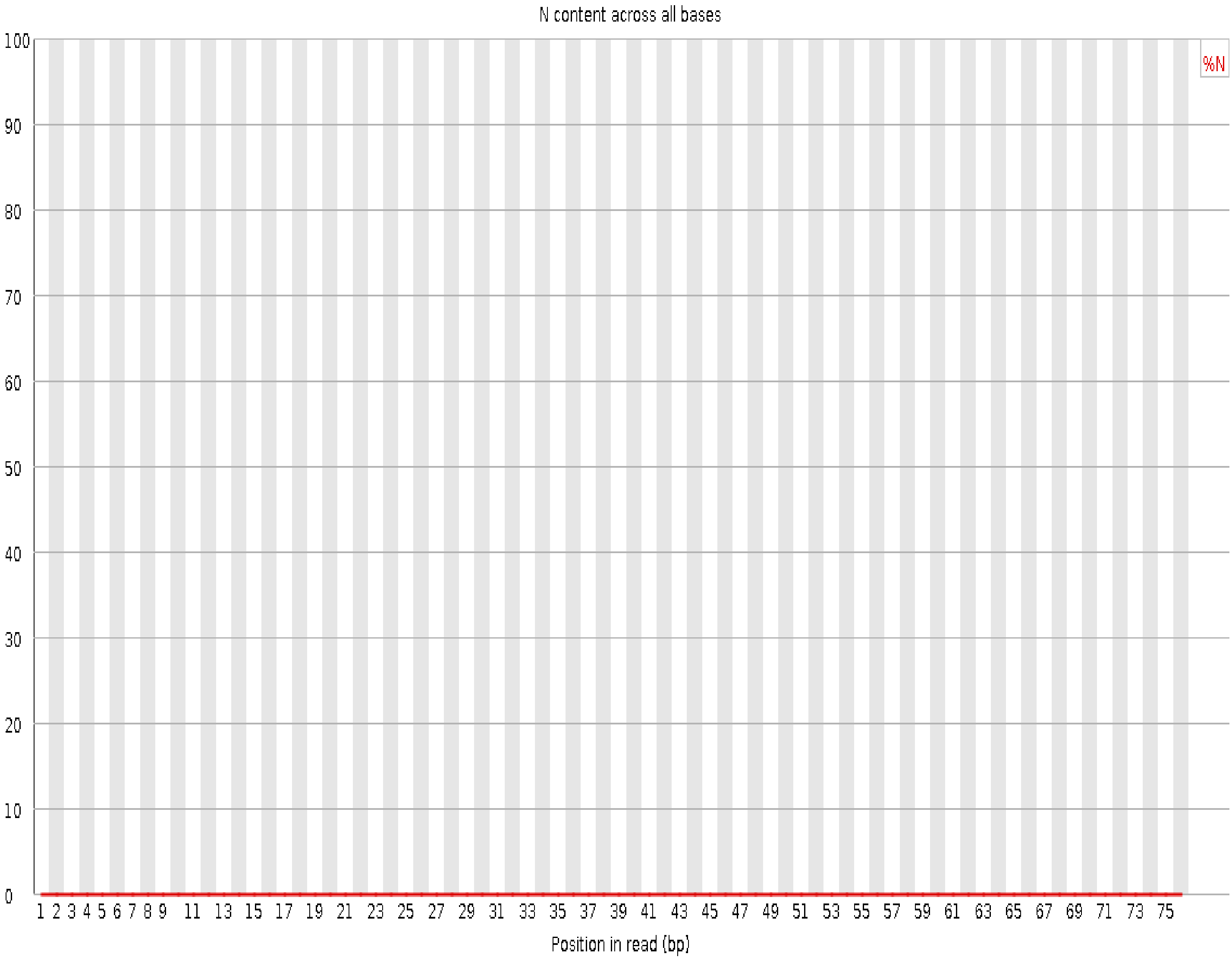


## Sequence Length Distribution

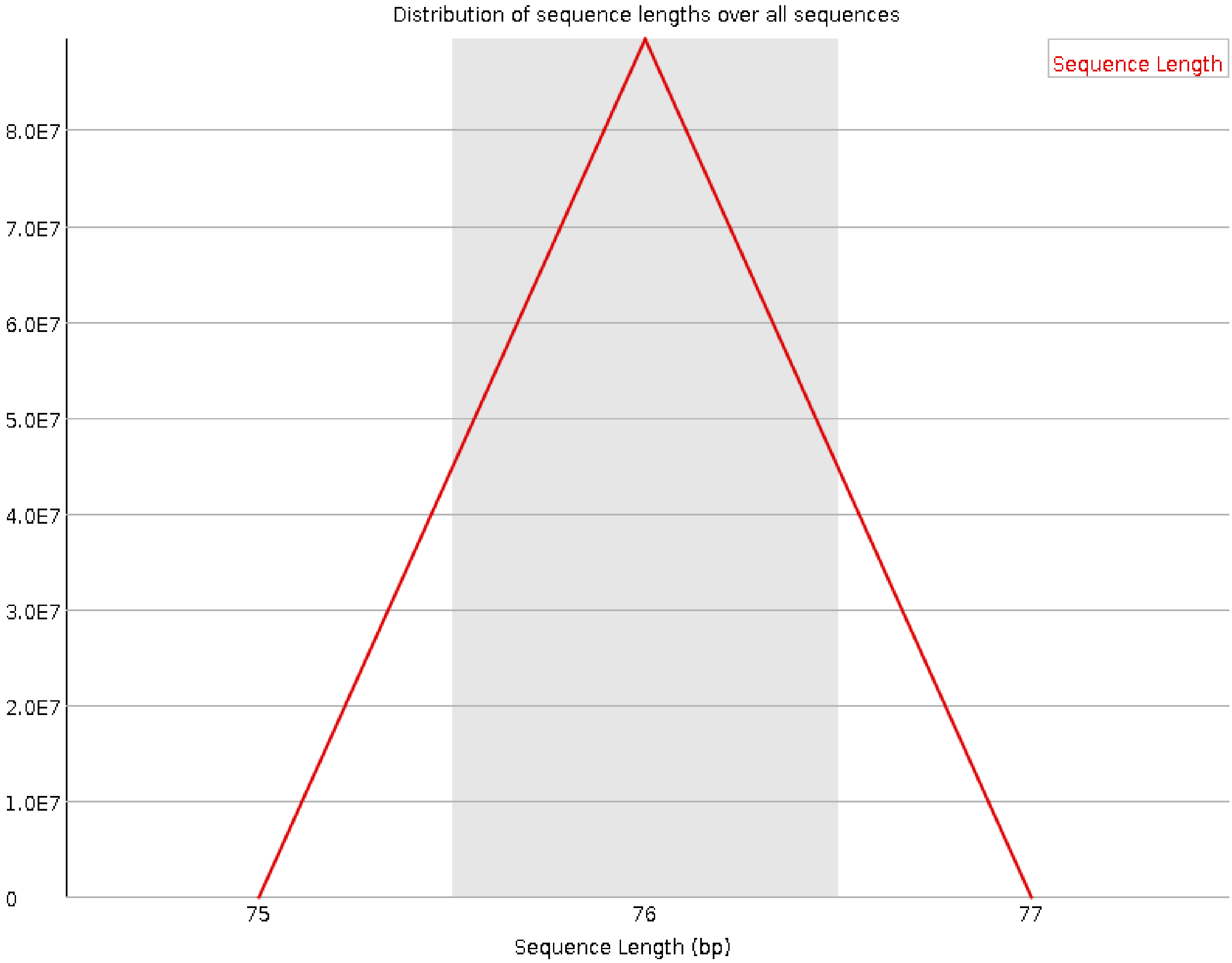


## Sequence Duplication Levels

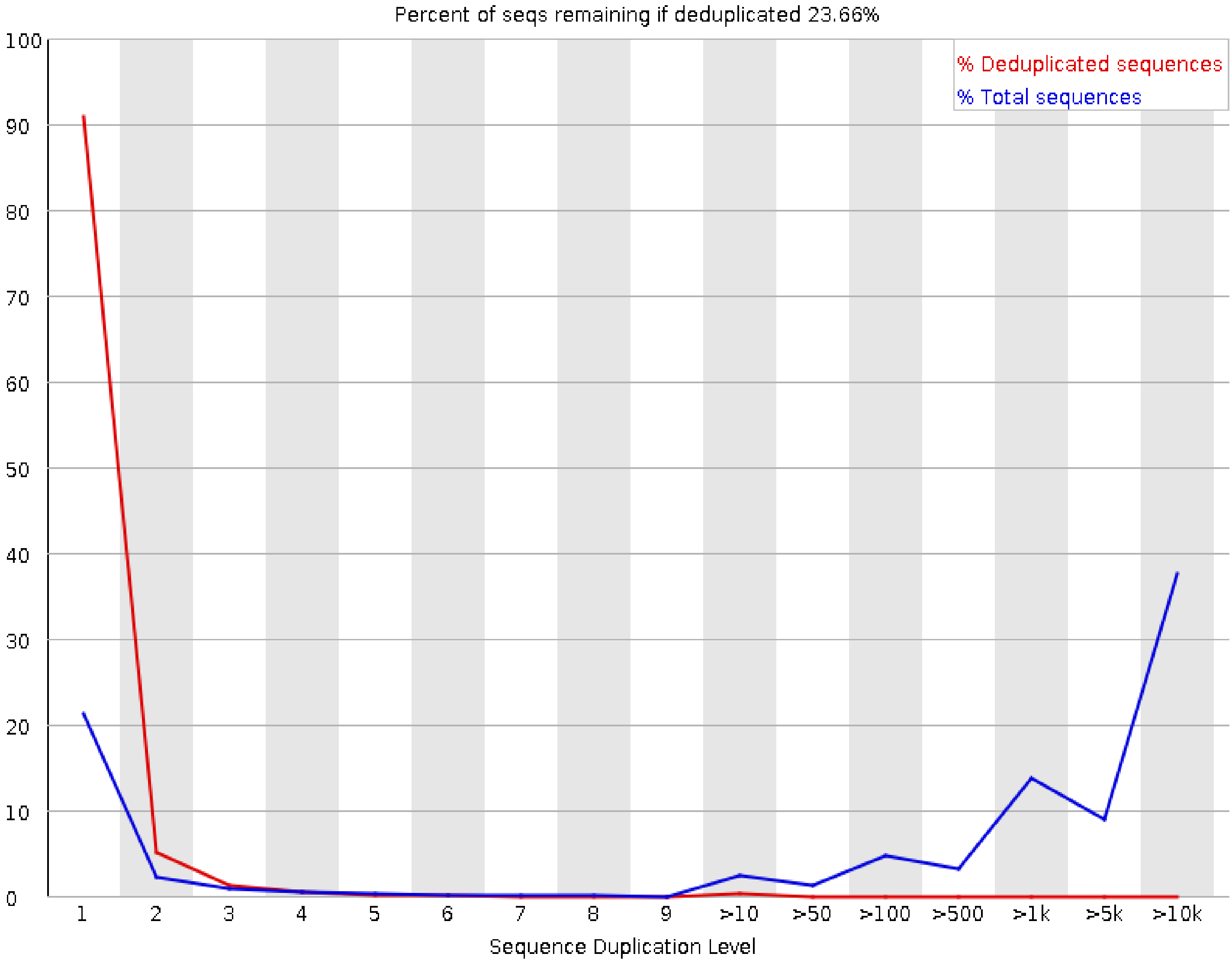


# Overrepresented sequences

| Sequence | Count | Percentage | Possible Source |
|---|---|---|---|
| CCGGTATTTAGCCTTAGATGGAGTTTACCACCCGCTTTGGGCTGCATTCC | 293398 | 0.3280122614368113 | No Hit |
| CTTCCGTACGCCACATGTCCCGCGCCCCGCCGCGGGGCGGGGATTCGGCG | 267137 | 0.2986530633591417 | No Hit |
| CTGGATAGTAGGTAGGGACAGTGGGAATCTCGTTCATCCATTCATGCGCG | 231699 | 0.25903418892646757 | No Hit |
| CTGAATTTAAGCATATTAGTCAGCGGAGGAGAAGAAACTAACCAGGATTC | 206315 | 0.23065545681407412 | No Hit |
| CTTGAACTCTCTCTTCAAAGTTCTTTTCAACTTTCCCTTACGGTACTTGT | 194107 | 0.21700719170108565 | No Hit |
| CTTCACCGTGCCAGACTAGAGTCAAGCTCAACAGGGTCTTCTTTCCCCGC | 174398 | 0.19497297994552457 | No Hit |
| GTCAAAGTGAAGAAATTCAATGAAGCGCGGGTAAACGGCGGGAGTAACTA | 169983 | 0.19003711080448227 | No Hit |
| CCCGTCGGCATGTATTAGCTCTAGAATTACCACAGTTATCCAAGTAGGAG | 168283 | 0.18813654964032103 | No Hit |
| CAGAAACCTCCCGTGGAGCAGAAGGGCAAAAGCTCGCTTGATCTTGATTT | 164332 | 0.18371942189937923 | No Hit |
| CTCCCTTTCGATCGGCCGAGGGCAACGGAGGCCATCGCCCGTCCCTTCGG | 157615 | 0.17620996934663158 | No Hit |
| CCGACATCGAAGGATCAAAAAGCGACGTCGCTATGAACGCTTGGCCGCCA | 151476 | 0.16934670758969872 | No Hit |

| Sequence | Count | Percentage | Possible Source |
|---|---|---|---|
| CGCAGTTTTATCCGGTAAAGCGAATGATTAGAGGTCTTGGGGCCGAAACG | 151041 | 0.1688603875271045З | No Hit |
| CTGCCAGTAGCATATGCTTGTCTCAAAGATTAAGCCATGCATGTCTAAGT | 145044 | 0.1621558785262369 | [illegible] |
| CGCGATGTGATTTCTGCCCAGTGCTCTGAATGTCAAAGTGAAGAAATTCA | 143054 | 0.15993110398701285 | No Hit |
| CCCATATCCGCAGCAGGTCTCCAAGGTGAACAGCCTCTGGCATGTTGGAA | 142756 | 0.15959794679470696 | No Hit |
| CTCTCTTCAAAGTTCTTTTCAACTTTCCCTTACGGTACTTGTTGACTATC | 137405 | 0.1536156510362206 | No Hit |
| CGGGTCTTCCGTACGCCACATGTCCCGCGCCCCGCCGCGGGGCGGGGATT | 136990 | 0.1531516905167342 | No Hit |
| CCCGAAGTTACGGATCCGGCTTGCCGACTTCCCTTACCTACATTGTTCCA | 134230 | 0.15006607356786064 | No Hit |
| GTAAATCTCGCGCCGGGCCGTACCCATATCCGCAGCAGGTCTCCAAGGTG | 131394 | 0.14689549035517754 | No Hit |
| CTCTCATGTCTCTTCACCGTGCCAGACTAGAGTCAAGCTCAACAGGGTCT | 130387 | 0.14576968735970086 | No Hit |
| CTTGTCTCAAAGATTAAGCCATGCATGTCTAAGTACGCACGGCCGGTACA | 128771 | 0.1439630362765923 | No Hit |
| CTCGCATTCCACGCCCGGCTCCACGCCAGCGAGCCGGGCTTCTTACCCAT | 120950 | 0.1352193369442952 | No Hit |
| CACCCGTTTACCTCTTAACGGTTTCACGCCCTCTTGAACTCTCTCTTCAA | 118948 | 0.1329811466792065 | No Hit |
| CTCATGTCTCTTCACCGTGCCAGACTAGAGTCAAGCTCAACAGGGTCTTC | 115778 | 0.12943715909662348 | No Hit |
| CTCCGCCACTCCGGATTCGGGGATCTGAACCCGACTCCCTTTCGATCGGC | 115287 | 0.1288882323133275 | No Hit |
| CCCGCTTTGGGCTGCATTCCCAAGCAACCCGACTCCGGGAAGACCCGGGC | 113852 | 0.12728393509534433 | No Hit |
| CCTCACCCGGCCCGGACACGGACAGGATTGACAGATTGATAGCTCTTTCT | 112178 | 0.1254124413372232 | No Hit |
| GTCGGCATGTATTAGCTCTAGAATTACCACAGTTATCCAAGTAGGAGAGG | 110644 | 0.12369746438085653 | No Hit |
| CTTAGAGCCAATCCTTATCCCGAAGTTACGGATCCGGCTTGCCGACTTCC | 107912 | 0.12064315079233388 | No Hit |
| CTCGATCAGAAGGACTTGGGCCCCCCACGAGCGGCGCCGGGGAGCGGGTC | 105803 | 0.11828533697161855 | No Hit |
| CTTAGATGGAGTTTACCACCCGCTTTGGGCTGCATTCCCAAGCAACCCGA | 105679 | 0.11814670780435034 | No Hit |
| CGAAGGCCCGCGGCGGGTGTTGACGCGATGTGATTTCTGCCCAGTGCTCT | 103564 | 0.11578218612070268 | No Hit |
| CGAGATTCCCACTGTCCCTACCTACTATCCAGCGAAACCACAGCCAAGGG | 103059 | 0.11521760765723126 | No Hit |
| CCTGTGGTAACTTTTCTGACACCTCCTGCTTAAAACCCAAAAGGTCAGAA | 102911 | 0.11505214703823369 | No Hit |
| CAAAGATTAAGCCATGCATGTCTAAGTACGCACGGCCGGTACAGTGAAAC | 101410 | 0.11337406332799485 | No Hit |
| CCCGGGGCTCCCGCCGGCTTCTCCGGGATCGGTCGCGTTACCGCACTGGA | 100210 | 0.1120324907415281 | No Hit |
| CGCGTCACTAATTAGATGACGAGGCATTTGGCTACCTTAAGAGAGTCATA | 97659 | 0.10918053101813083 | No Hit |
| CTTGGCTGTGGTTTCGCTGGATAGTAGGTAGGGACAGTGGGAATCTCGTT | 96954 | 0.10839235712358161 | No Hit |
| CCACTCTCGACTGCCGGCGACGGCCGGGTATGGGCCCGACGCTCCAGCGC | 96796 | 0.10821571673303015 | No Hit |
| CTCCACTTCGGCCTTCAAAGTTCTCGTTTGAATATTTGCTACTACCACCA | 91463 | 0.10225354456334082 | No Hit |
| CCCAGGCATAGTTCACCATCTTTCGGGTCCTAACACGTGCGCTCGTGCTC | 91405 | 0.10218870188832825 | No Hit |
| CGCGTAACTAGTTAGCATGCCAGAGTCTCGTTCGTTATCGGAATTAACCA | 90990 | 0.10172474136884184 | No Hit |
| GCCCTCTTGAACTCTCTCTTCAAAGTTCTTTTCAACTTTCCCTTACGGTA | 90174 | 0.10081247201004444 | No Hit |

## Adapter Content

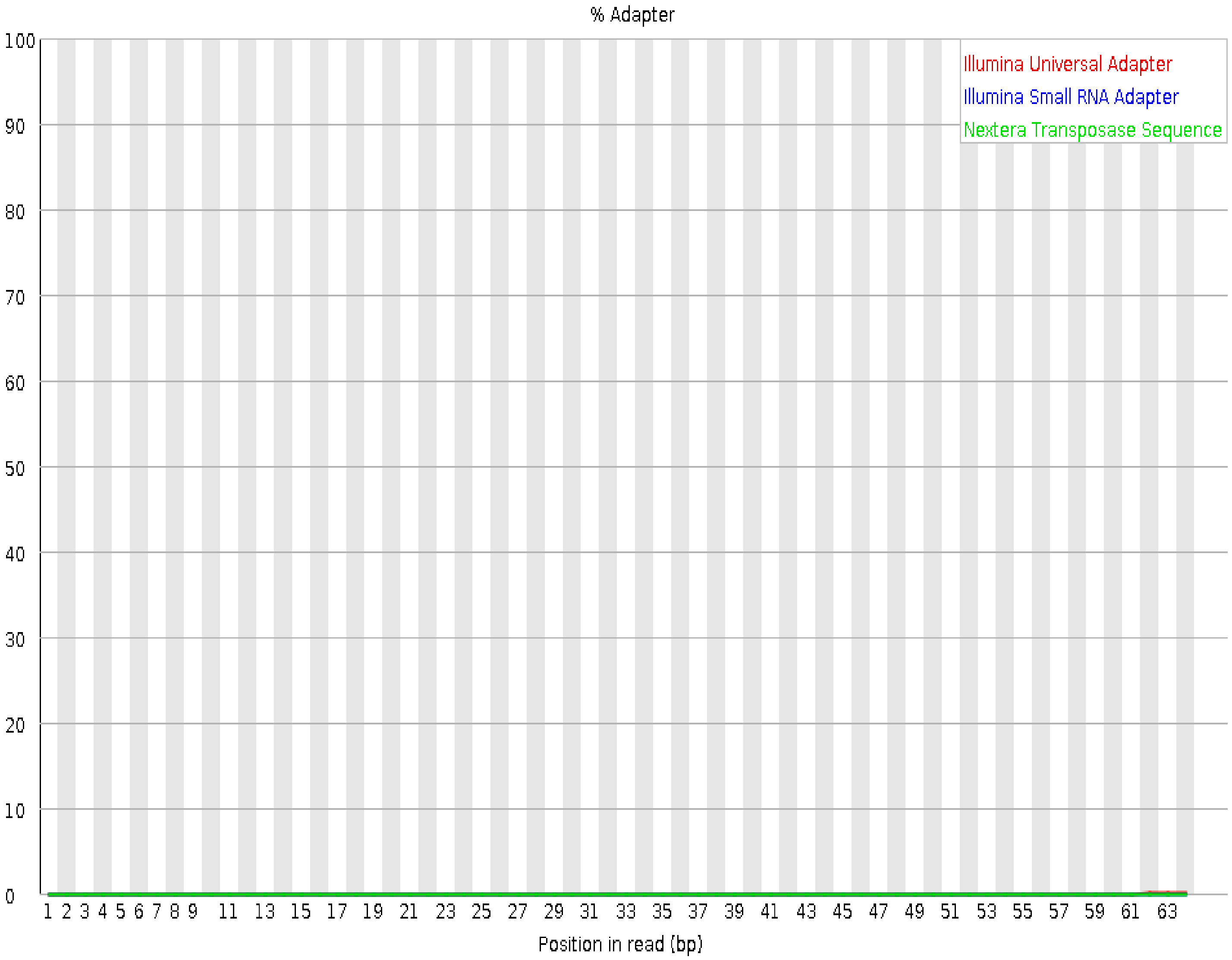


## Kmer Content

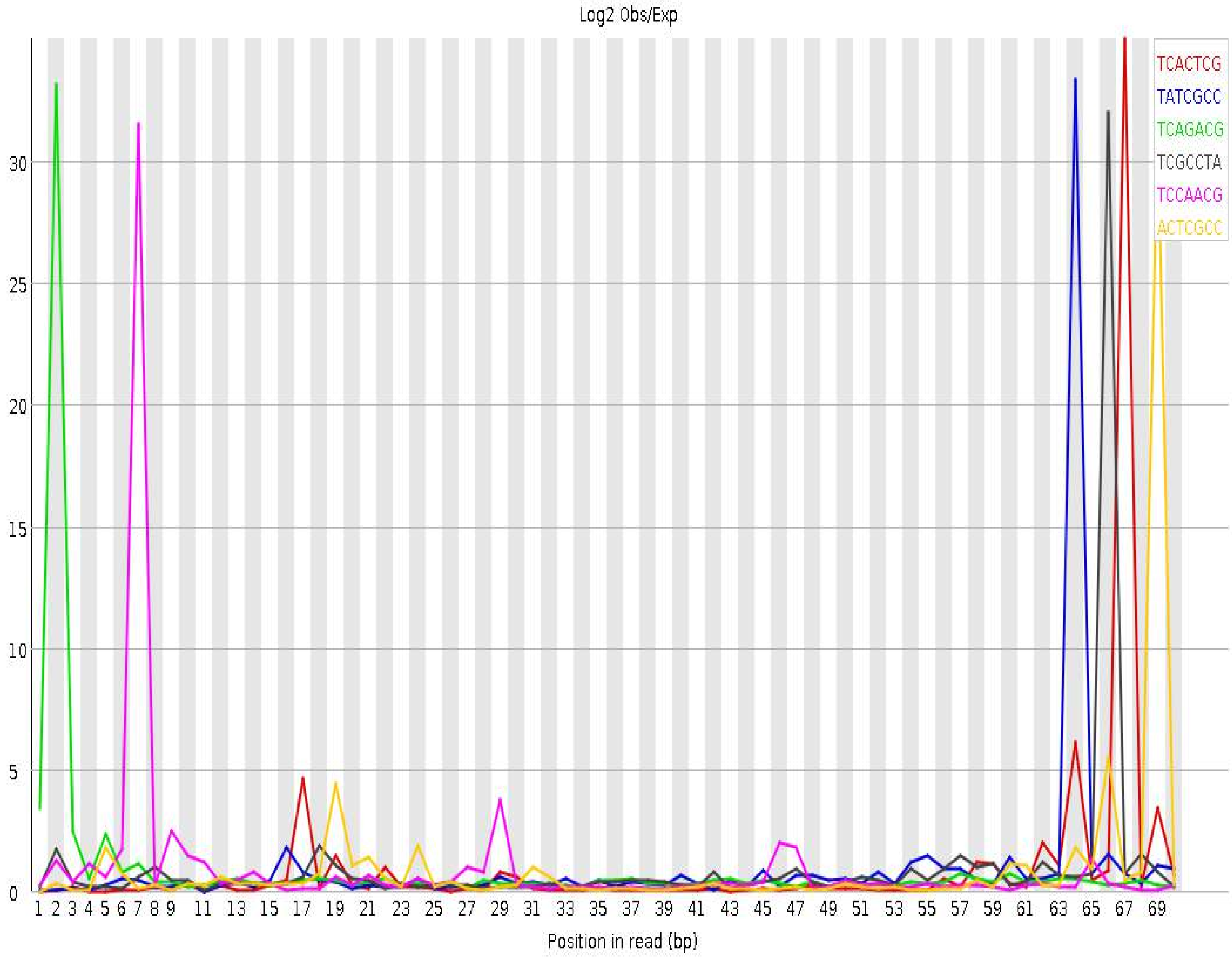


| Sequence | Count | PValue | Obs/Exp Max | Max Obs/Exp Position |
|---|---|---|---|---|
| TCACTCG | 59900 | 0.0 | 34.981514 | 67 |
| TATCGCC | 6640 | 0.0 | 33.310013 | 64 |
| TCAGACG | 13995 | 0.0 | 33.09813 | 2 |
| TCGCCTA | 6855 | 0.0 | 31.959587 | 66 |
| TCCAACG | 22295 | 0.0 | 31.503878 | 7 |
| ACTCGCC | 67100 | 0.0 | 31.420494 | 69 |
| CTATCGC | 6980 | 0.0 | 31.38709 | 63 |
| AGACGTG | 25900 | 0.0 | 30.118402 | 2 |
| CGCCTAT | 7280 | 0.0 | 29.951105 | 67 |
| TGCGCGA | 15330 | 0.0 | 29.617191 | 9 |
| CAACGCT | 23505 | 0.0 | 29.503279 | 9 |
| TTCACTC | 72095 | 0.0 | 29.184223 | 66 |
| CGCGTAA | 25440 | 0.0 | 28.958185 | 1 |
| CCAACGC | 24045 | 0.0 | 28.937899 | 8 |
| CCAGTAG | 39220 | 0.0 | 28.93281 | 4 |

| Sequence | Count | PValue | Obs/Exp Max | Max Obs/Exp Position |
| --- | --- | --- | --- | --- |
| TTGGCCG | 45170 | 0.0 | 27.[illegible] | 41 |
| GCGTAAC | 27305 | 0.0 | 27.19[illegible] | 2 |
| CGCGAGT | 16405 | 0.0 | 27.167624 | 11 |
| ACAATCC | 26765 | 0.0 | 26.973083 | 3 |
| GTGCGCG | 17205 | 0.0 | 26.710709 | 8 |

**Produced by FastQC (version 0.11.2)**

# FastQC Report

Mon 4 May 2015
Origene_Adult_Stomach_1840_CAGATC_L005_R1.fastq.gz

## Summary


- Basic Statistics
- Per base sequence quality
- Per tile sequence quality
- Per sequence quality scores
- Per base sequence content
- Per sequence GC content
- Per base N content
- Sequence Length Distribution
- Sequence Duplication Levels
- Overrepresented sequences
- Adapter Content
- Kmer Content


## Basic Statistics

| Measure | Value |
|---|---|
| Filename | Origene_Adult_Stomach_1840_CAGATC_L005_R1.fastq.gz |
| File type | Conventional base calls |
| Encoding | Sanger / Illumina 1.9 |
| Total Sequences | 81103684 |
| Sequences flagged as poor quality | 0 |
| Sequence length | 76 |
| %GC | 56 |

## Per base sequence quality

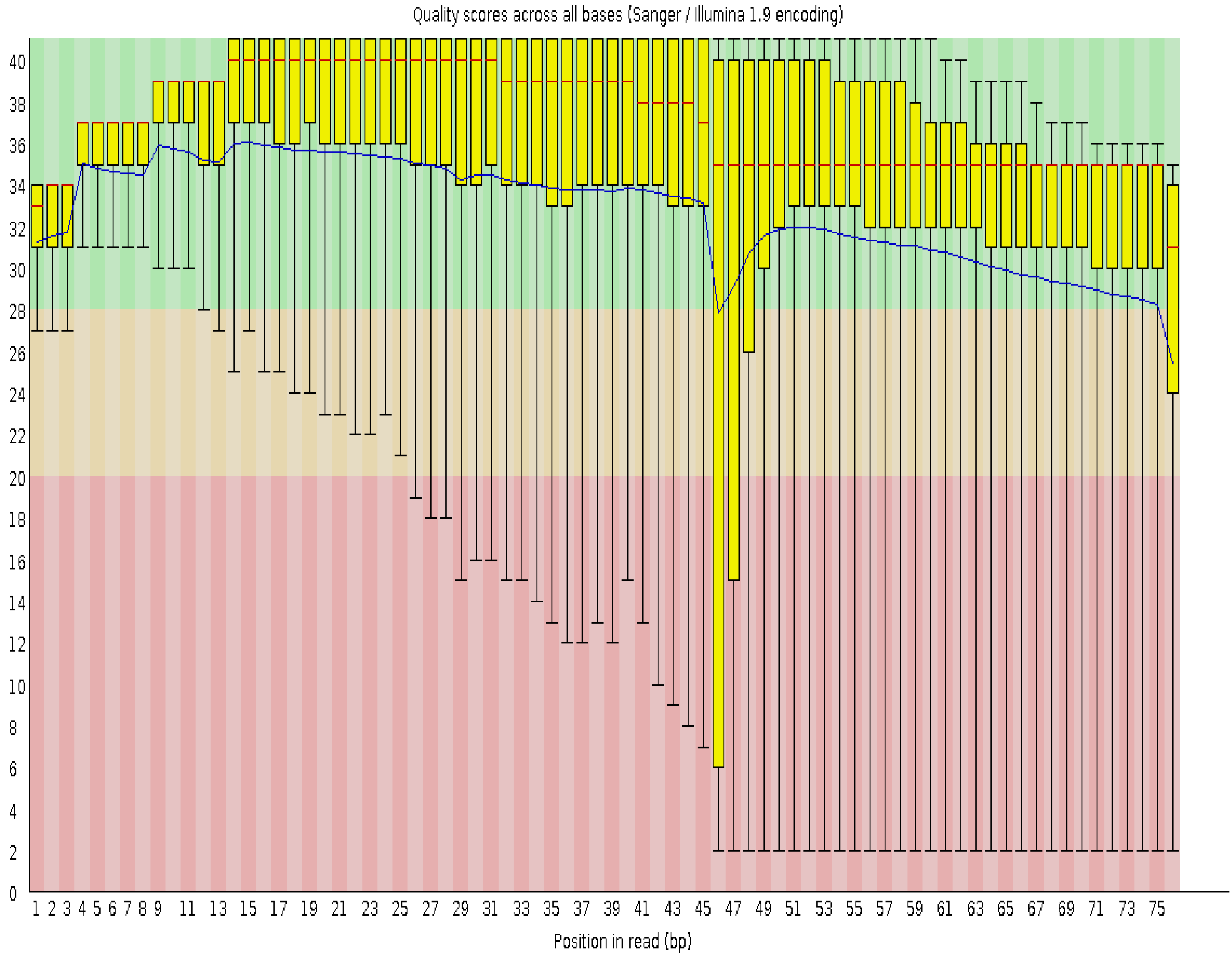


## Per tile sequence quality

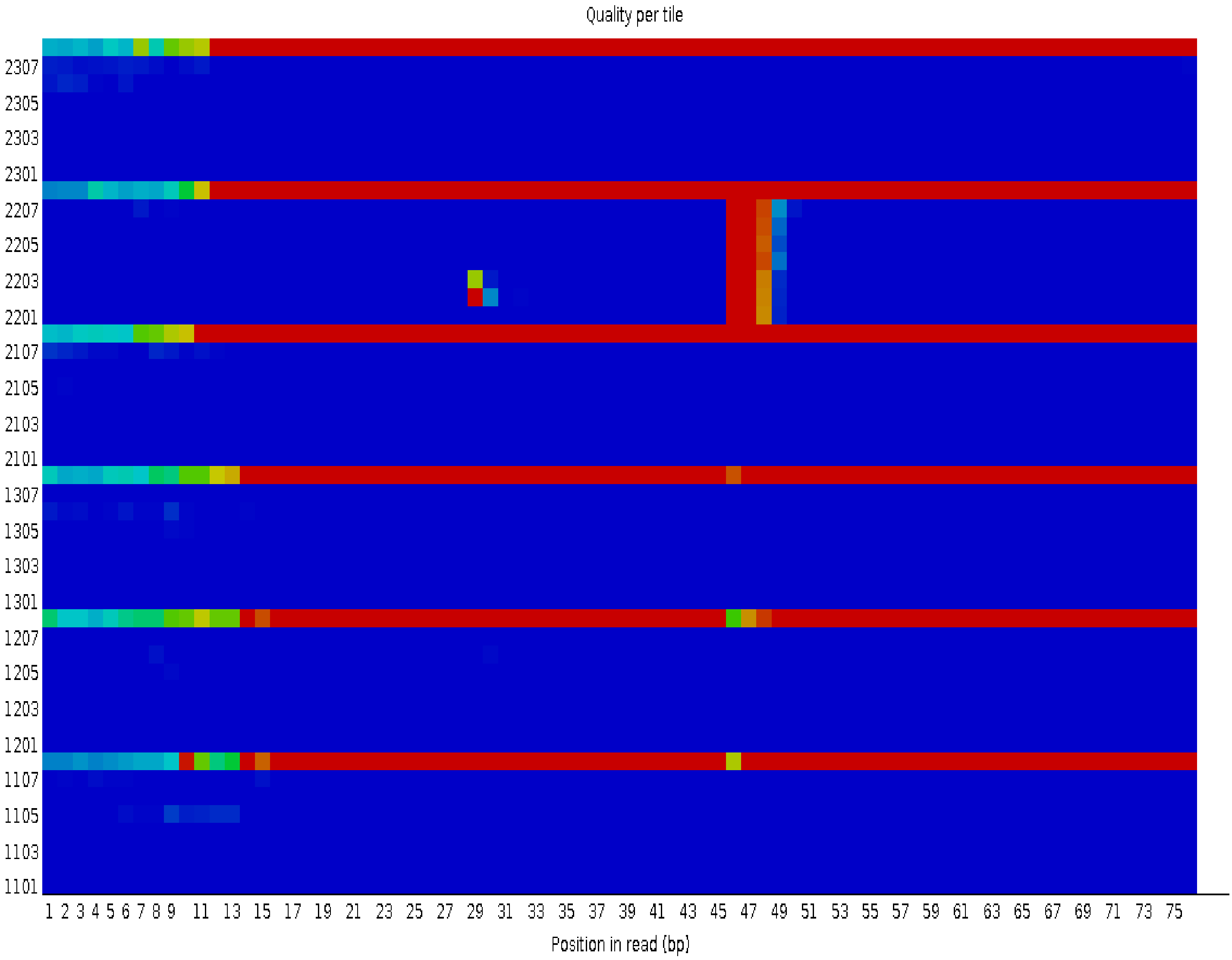


## Per sequence quality scores

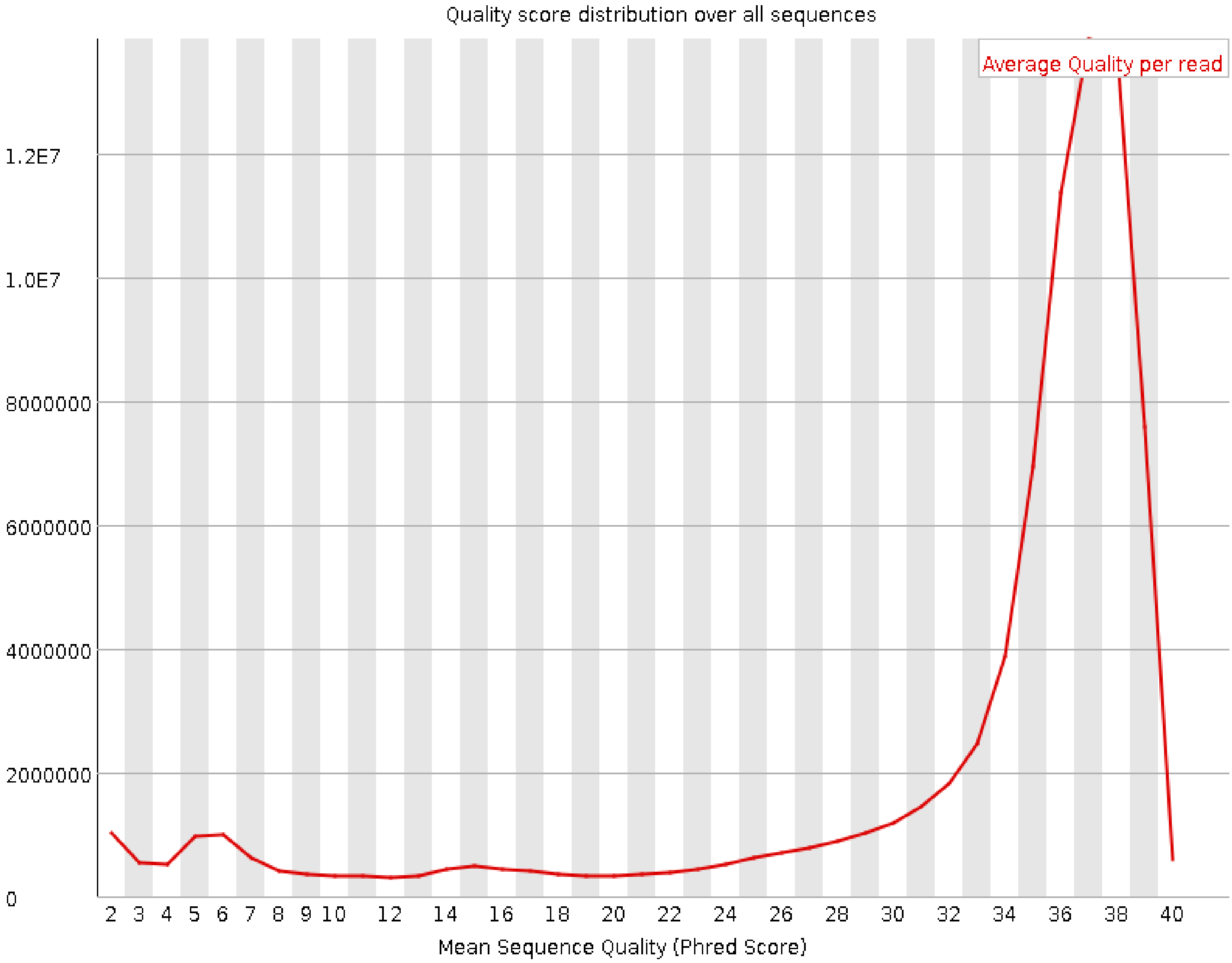


## Per base sequence content

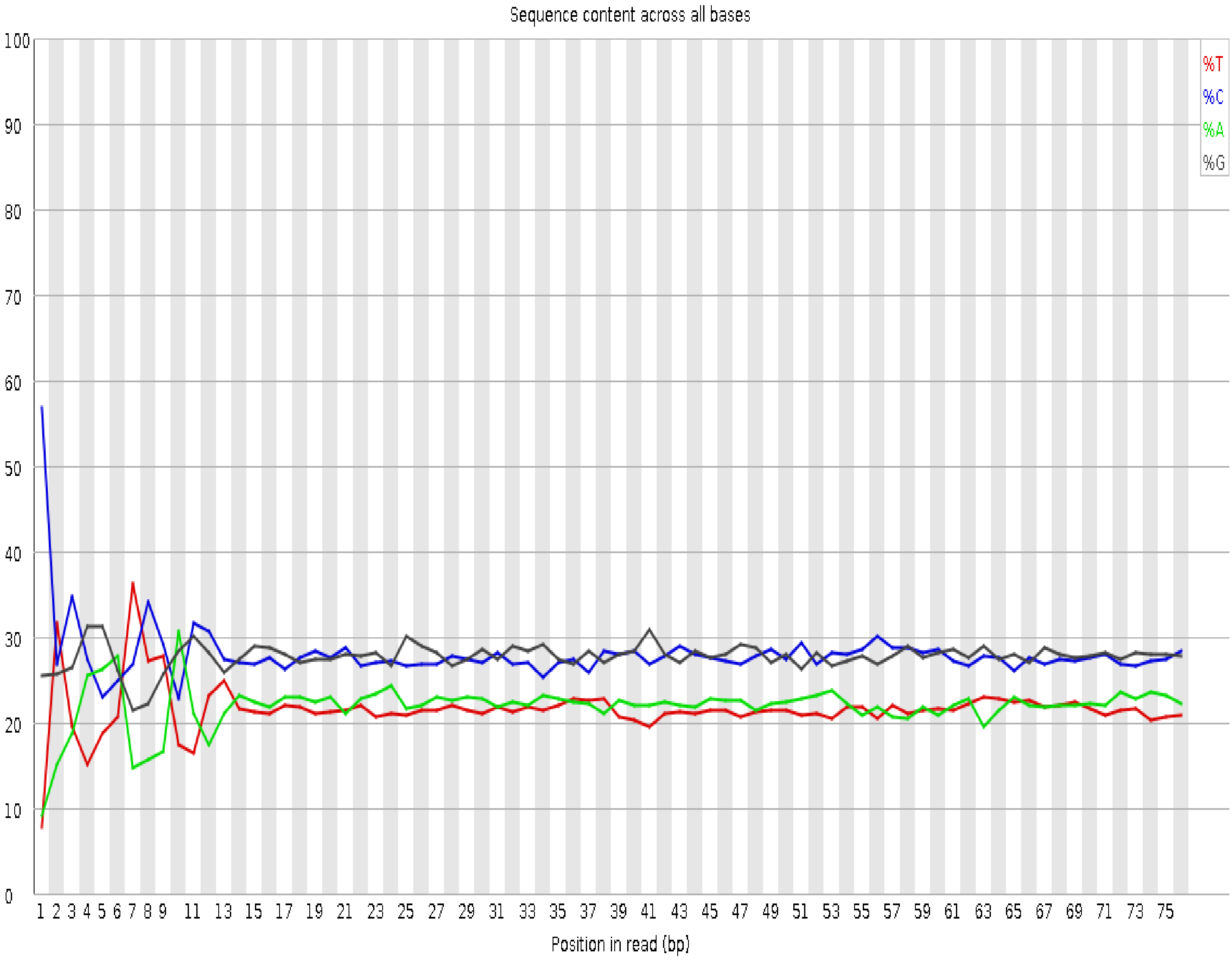


## Per sequence GC content

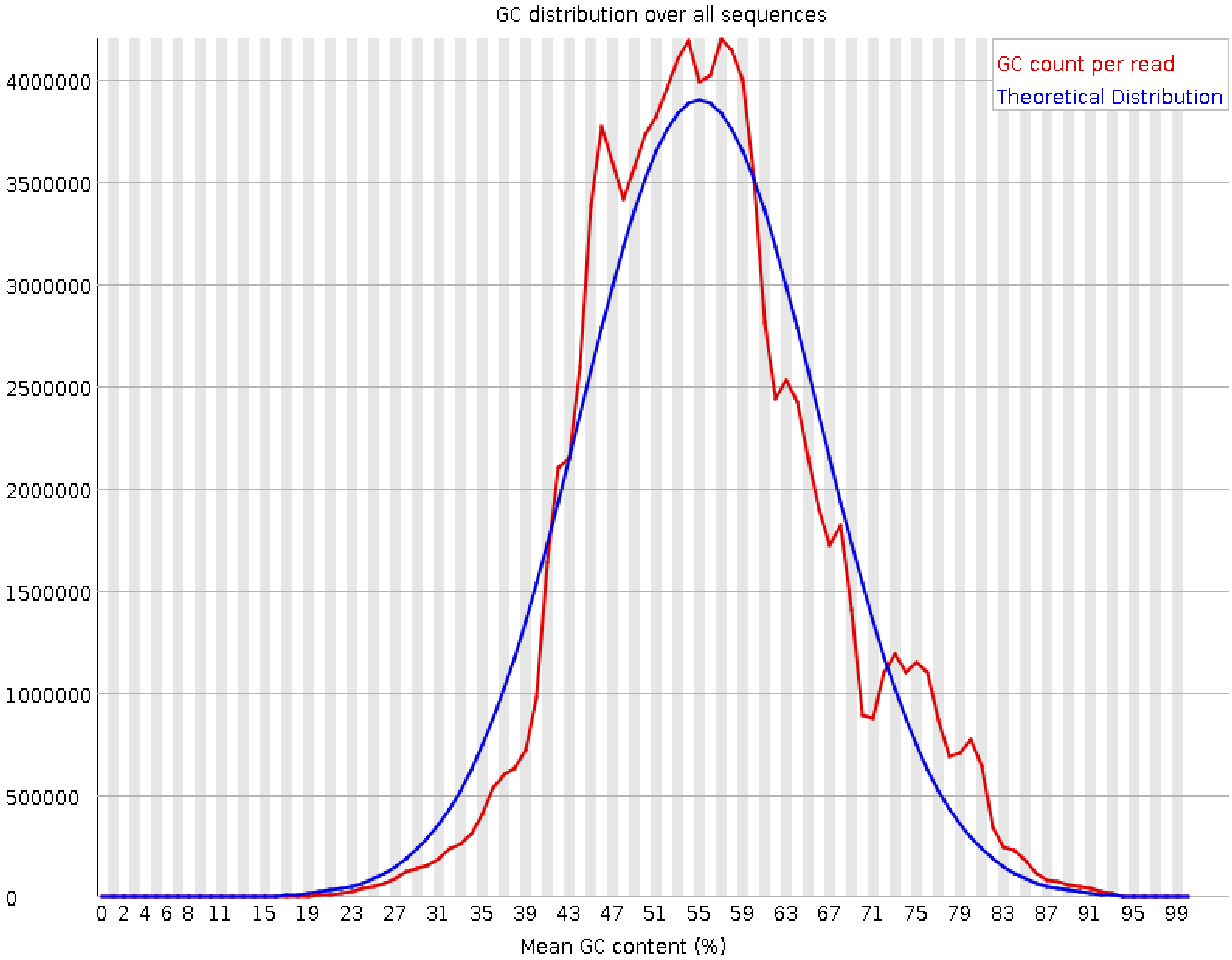


## Per base N content

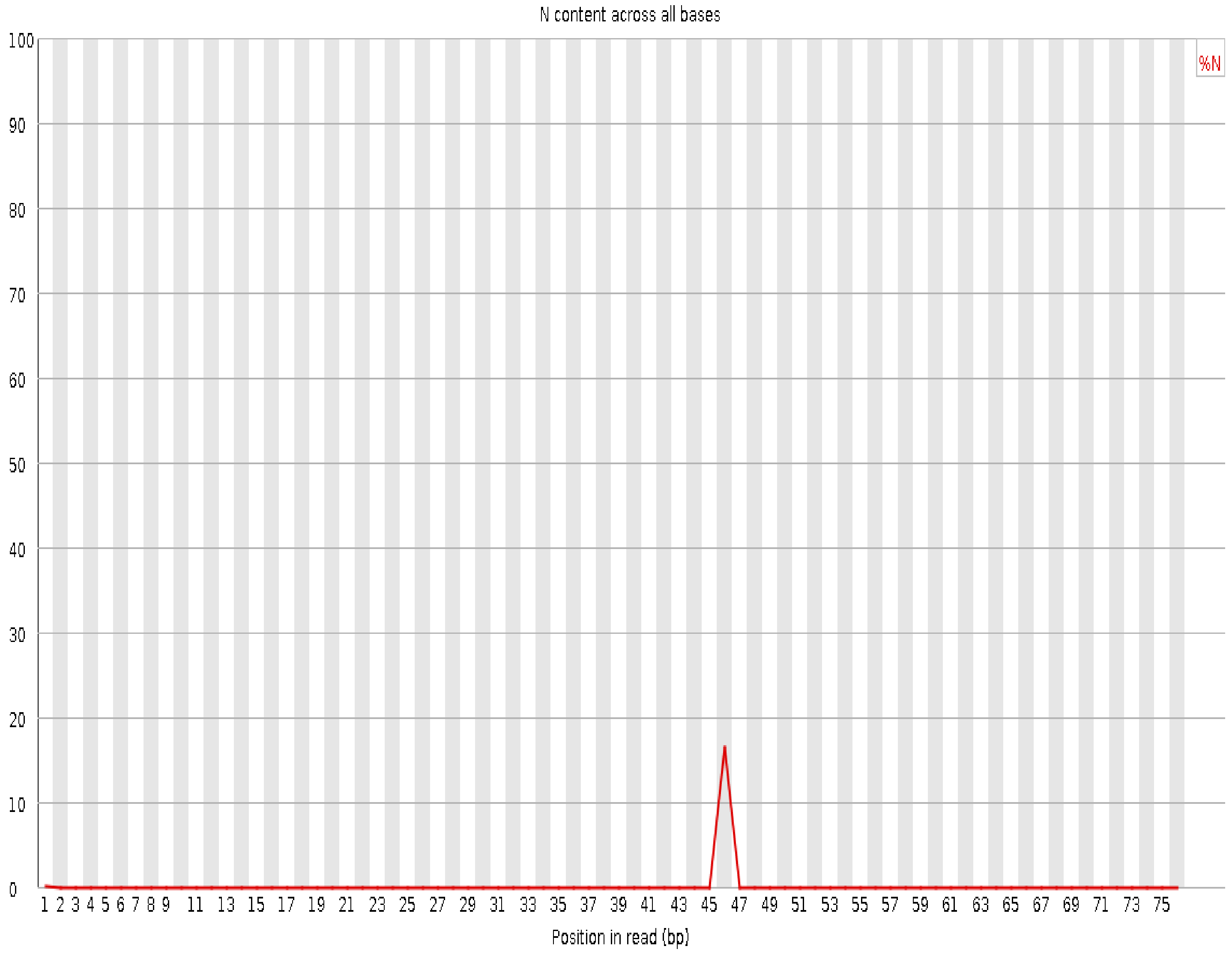


## Sequence Length Distribution

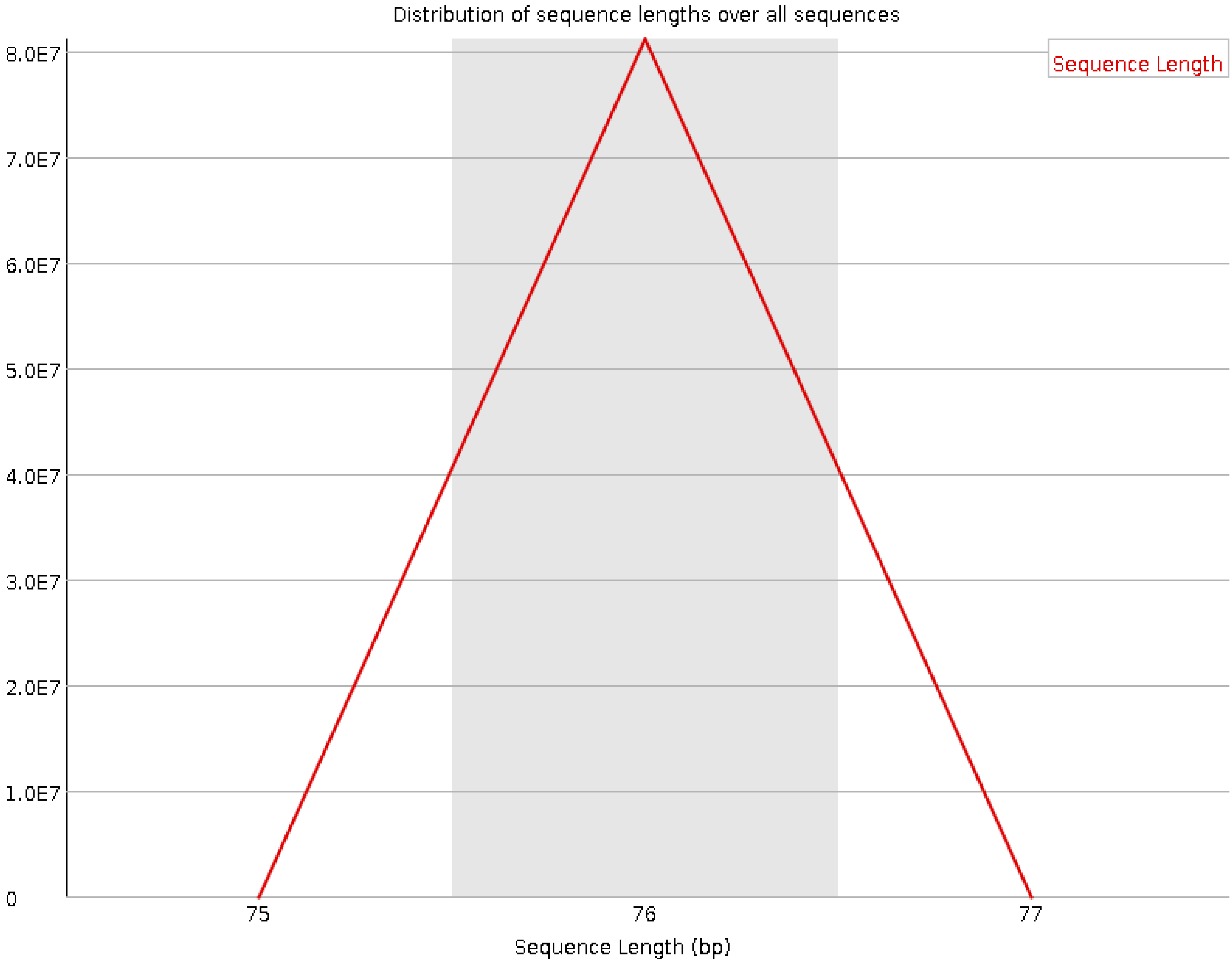


## Sequence Duplication Levels

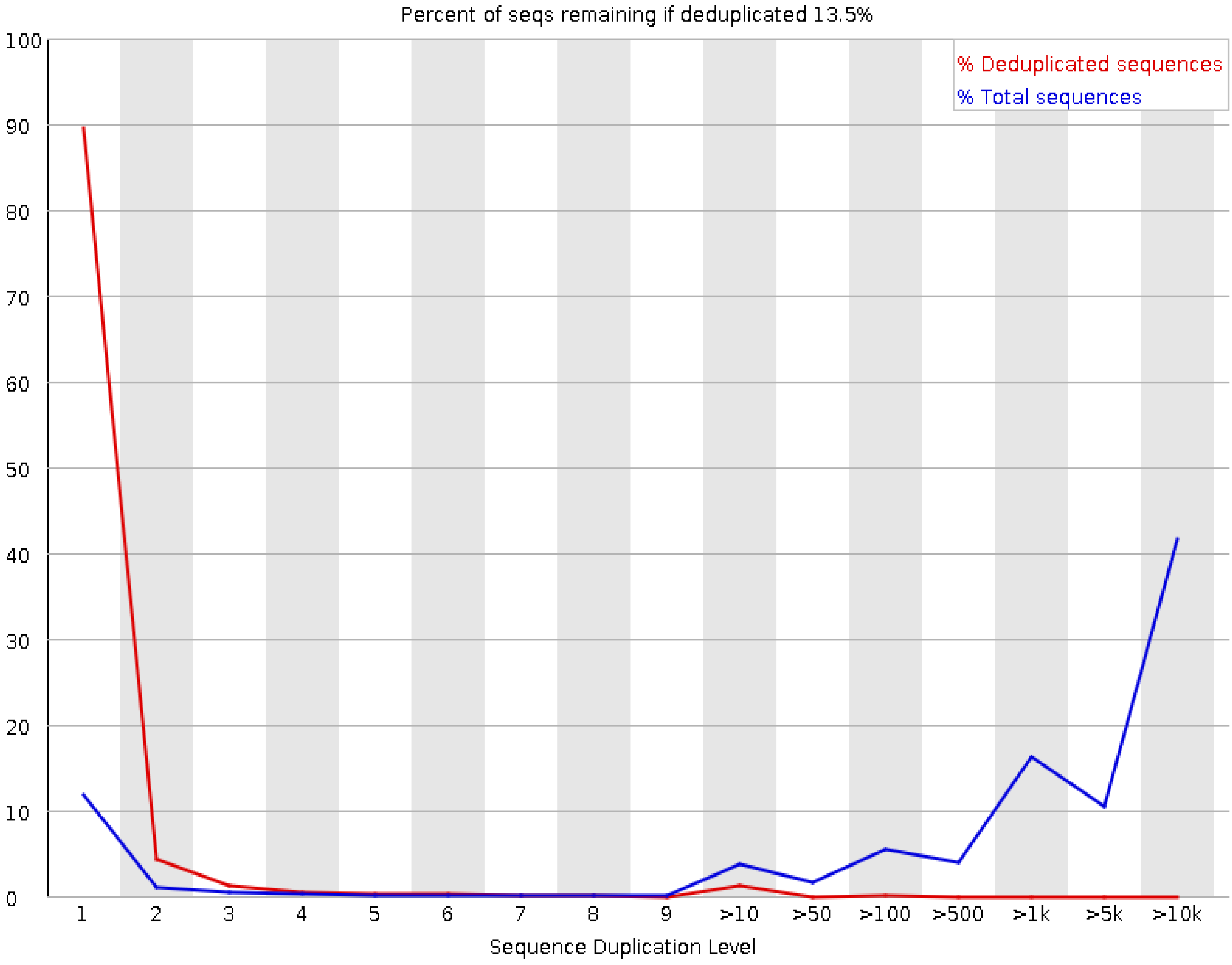


## Overrepresented sequences

| Sequence | Count | Percentage | Possible Source |
|---|---|---|---|
| CGCGTAACTAGTTAGCATGCCAGAGTCTCGTTCGTTATCGGAATTAACCA | 326713 | 0.4028337356414044 | No Hit |
| CCTCACCCGGCCCGGACACGGACAGGATTGACAGATTGATAGCTCTTTCT | 271595 | 0.33487381411675454 | No Hit |
| CGCAGTTTTATCCGGTAAAGCGAATGATTAGAGGTCTTGGGGCCGAAACG | 255166 | 0.31461702775425093 | No Hit |
| CTTCCGTACGCCACATGTCCCGCGCCCCGCCGCGGGGCGGGGATTCGGCG | 175117 | 0.21591744217192402 | No Hit |
| CCGGTATTTAGCCTTAGATGGAGTTTACCACCCGCTTTGGGCTGCATTCC | 153869 | 0.18971887886128574 | No Hit |
| GTCAAAGTGAAGAAATTCAATGAAGCGCGGGTAAACGGCGGGAGTAACTA | 147553 | 0.1819313164615309 | No Hit |
| CTCACCCGGCCCGGACACGGACAGGATTGACAGATTGATAGCTCTTTCTC | 146941 | 0.18117672681798278 | No Hit |
| CCCGAAGTTACGGATCCGGCTTGCCGACTTCCCTTACCTACATTGTTCCA | 141989 | 0.17507096225123386 | No Hit |
| CCCATATCCGCAGCAGGTCTCCAAGGTGAACAGCCTCTGGCATGTTGGAA | 138186 | 0.17038190275055817 | No Hit |
| CTTCACCGTGCCAGACTAGAGTCAAGCTCAACAGGGTCTTCTTTCCCCGC | 137516 | 0.16955579970941886 | No Hit |
| CTGGATAGTAGGTAGGGACAGTGGGAATCTCGTTCATCCATTCATGCGCG | 134711 | 0.16609726384315662 | No Hit |

| Sequence | Count | Percentage | Possible Source |
|---|---|---|---|
| CGCGATGTGATTTCTGCCCAGTGCTCTGAATGTCAAAGTGAAGAAATTCA | 130840 | 0.16132436104875333 | No Hit |
| GTAAATCTCGCGCCGGGCCGTACCCATATCCGCAGCAGGTCTCCAAGGTG | 116227 | 0.14330668382462133 | No Hit |
| CCCAGGCATAGTTCACCATCTTTCGGGTCCTAACACGTGCGCTCGTGCTC | 110082 | 0.13572996264879902 | No Hit |
| CTCTCATGTCTCTTCACCGTGCCAGACTAGAGTCAAGCTCAACAGGGTCT | 106002 | 0.13069936502514487 | No Hit |
| CTCGCATTCCACGCCCGGCTCCACGCCAGCGAGCCGGGCTTCTTACCCAT | 104250 | 0.1285391672220463 | No Hit |
| CTCTCTTCAAAGTTCTTTTCAACTTTCCCTTACGGTACTTGTTGACTATC | 100182 | 0.12352336547375578 | No Hit |
| CCCACTTATTCTACACCTCTCATGTCTCTTCACCGTGCCAGACTAGAGTC | 98639 | 0.12162086249990814 | No Hit |
| CTGAATTTAAGCATATTAGTCAGCGGAGGAGAAGAAACTAACCAGGATTC | 96740 | 0.1192794152236044 | No Hit |
| CTCCCTTTCGATCGGCCGAGGGCAACGGAGGCCATCGCCCGTCCCTTCGG | 95764 | 0.11807601736069104 | No Hit |
| CTCTGGTCCGTCTTGCGCCGGTCCAAGAATTTCACCTCTAGCGGCGCAAT | 94811 | 0.11690097825889142 | No Hit |
| CTTTAAATGGGTAAGAAGCCCGGCTCGCTGGCGTGGAGCCGGGCGTGGAA | 94791 | 0.11687631846661861 | No Hit |
| GGGATCCCGAGGCCTCTCCAGTCCGCCGAGGGCGCACCACCGGCCCGTCT | 94450 | 0.11645586900836712 | No Hit |
| CTCCGACTTTCGTTCTTGATTAATGAAAACATTCTTGGCAAATGCTTTCG | 94352 | 0.11633503602623034 | No Hit |
| CCCGGGGCTCCCGCCGGCTTCTCCGGGATCGGTCGCGTTACCGCACTGGA | 93017 | 0.11468899489201995 | No Hit |
| CGGGTCTTCCGTACGCCACATGTCCCGCGCCCCGCCGCGGGGCGGGGATT | 90890 | 0.11206642598380612 | No Hit |
| CTCGATCAGAAGGACTTGGGCCCCCCACGAGCGGCGCCGGGGAGCGGGTC | 90836 | 0.1119998445446695 | No Hit |
| CCACTCTCGACTGCCGGCGACGGCCGGGTATGGGCCCGACGCTCCAGCGC | 90646 | 0.11176557651807777 | No Hit |
| CTTGAACTCTCTCTTCAAAGTTCTTTTCAACTTTCCCTTACGGTACTTGT | 89542 | 0.1104043559846184 | No Hit |
| CGCGTCACTAATTAGATGACGAGGCATTTGGCTACCTTAAGAGAGTCATA | 87469 | 0.10784836851554117 | No Hit |
| CACGAGCGCACGTGTTAGGACCCGAAAGATGGTGAACTATGCCTGGGCAG | 85635 | 0.10558706556412407 | No Hit |
| CCGACATCGAAGGATCAAAAAGCGACGTCGCTATGAACGCTTGGCCGCCA | 85509 | 0.10543170887280533 | No Hit |
| CCTATACCCAGGTCGGACGACCGATTTGCACGTCAGGACCGCTACGGACC | 85247 | 0.10510866559403145 | No Hit |
| CGAAGGCCCGCGGCGGGTGTTGACGCGATGTGATTTCTGCCCAGTGCTCT | 84856 | 0.10462656665509794 | No Hit |
| CTTAGAGCCAATCCTTATCCCGAAGTTACGGATCCGGCTTGCCGACTTCC | 84268 | 0.10390156876227717 | No Hit |

## Adapter Content

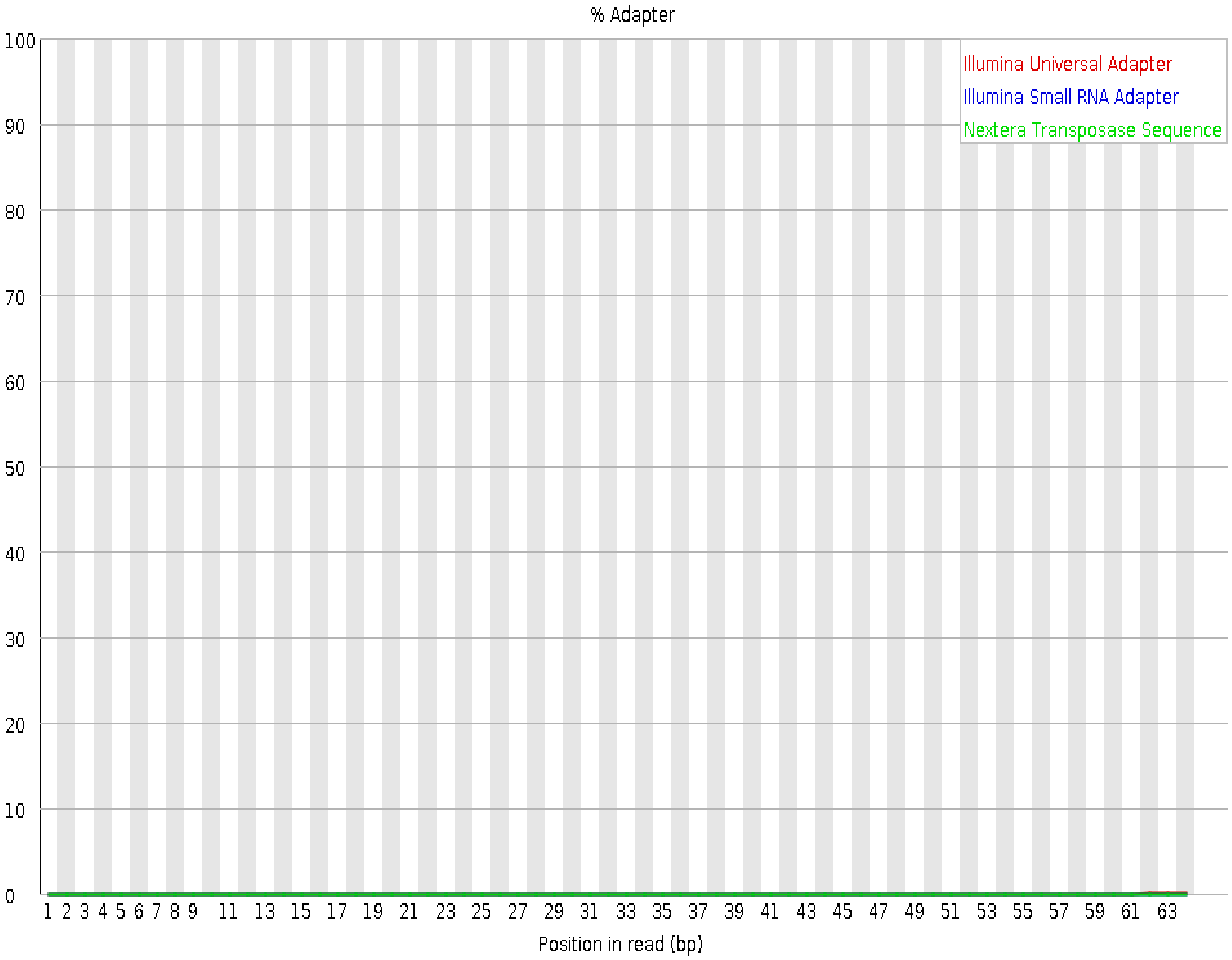


## Kmer Content

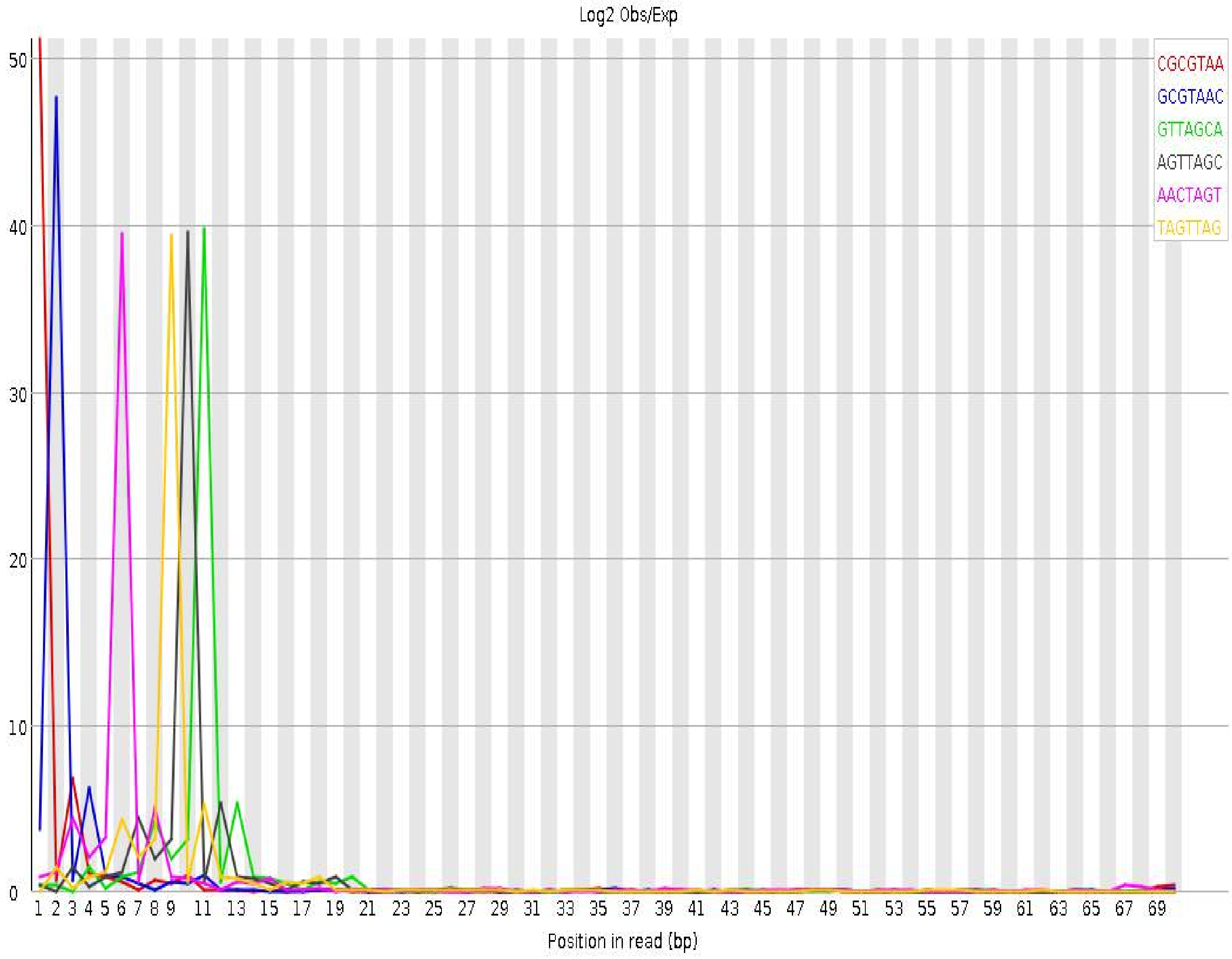


| Sequence | Count | PValue | Obs/Exp Max | Max Obs/Exp Position |
|---|---|---|---|---|
| CGCGTAA | 63715 | 0.0 | 51.15197 | 1 |
| GCGTAAC | 69015 | 0.0 | 47.612164 | 2 |
| GTTAGCA | 76715 | 0.0 | 39.77435 | 11 |
| AGTTAGC | 76620 | 0.0 | 39.562073 | 10 |
| AACTAGT | 80470 | 0.0 | 39.446747 | 6 |
| TAGTTAG | 77815 | 0.0 | 39.370296 | 9 |
| ACTAGTT | 80875 | 0.0 | 39.281128 | 7 |
| CGTAACT | 83730 | 0.0 | 39.216297 | 3 |
| TAGCATG | 84125 | 0.0 | 36.862682 | 13 |
| TTAGCAT | 84815 | 0.0 | 36.376637 | 12 |
| TCAGACG | 16420 | 0.0 | 36.326195 | 2 |
| AGCATGC | 85865 | 0.0 | 36.31653 | 14 |
| TAACTAG | 89745 | 0.0 | 36.21122 | 5 |
| GCATGCC | 87675 | 0.0 | 35.495396 | 15 |
| TGTGTCG | 7065 | 0.0 | 34.63831 | 3 |

| Sequence | Count | PValue | Obs/Exp Max | Max Obs/Exp Position |
|---|---|---|---|---|
| CTAGTTA | 90460 | 0.0 | 34.[illegible] | 8 |
| TCACTCG | 48740 | 0.0 | 33.9[illegible] | 67 |
| GTGTCGA | 7480 | 0.0 | 32.900593 | 4 |
| TCGCCTA | 6225 | 0.0 | 32.228024 | 66 |
| TCCAACG | 15975 | 0.0 | 32.206963 | 7 |

Produced by FastQC (version 0.11.2)

# FastQC Report

Mon 4 May 2015
Origene_Adult_Stomach_1840_CAGATC_L005_R2.fastq.gz

## Summary

- Basic Statistics
- Per base sequence quality
- Per tile sequence quality
- Per sequence quality scores
- Per base sequence content
- Per sequence GC content
- Per base N content
- Sequence Length Distribution
- Sequence Duplication Levels
- Overrepresented sequences
- Adapter Content
- Kmer Content

## Basic Statistics

| Measure | Value |
| --- | --- |
| Filename | Origene_Adult_Stomach_1840_CAGATC_L005_R2.fastq.gz |
| File type | Conventional base calls |
| Encoding | Sanger / Illumina 1.9 |
| Total Sequences | 81103684 |
| Sequences flagged as poor quality | 0 |
| Sequence length | 76 |
| %GC | 56 |

## Per base sequence quality

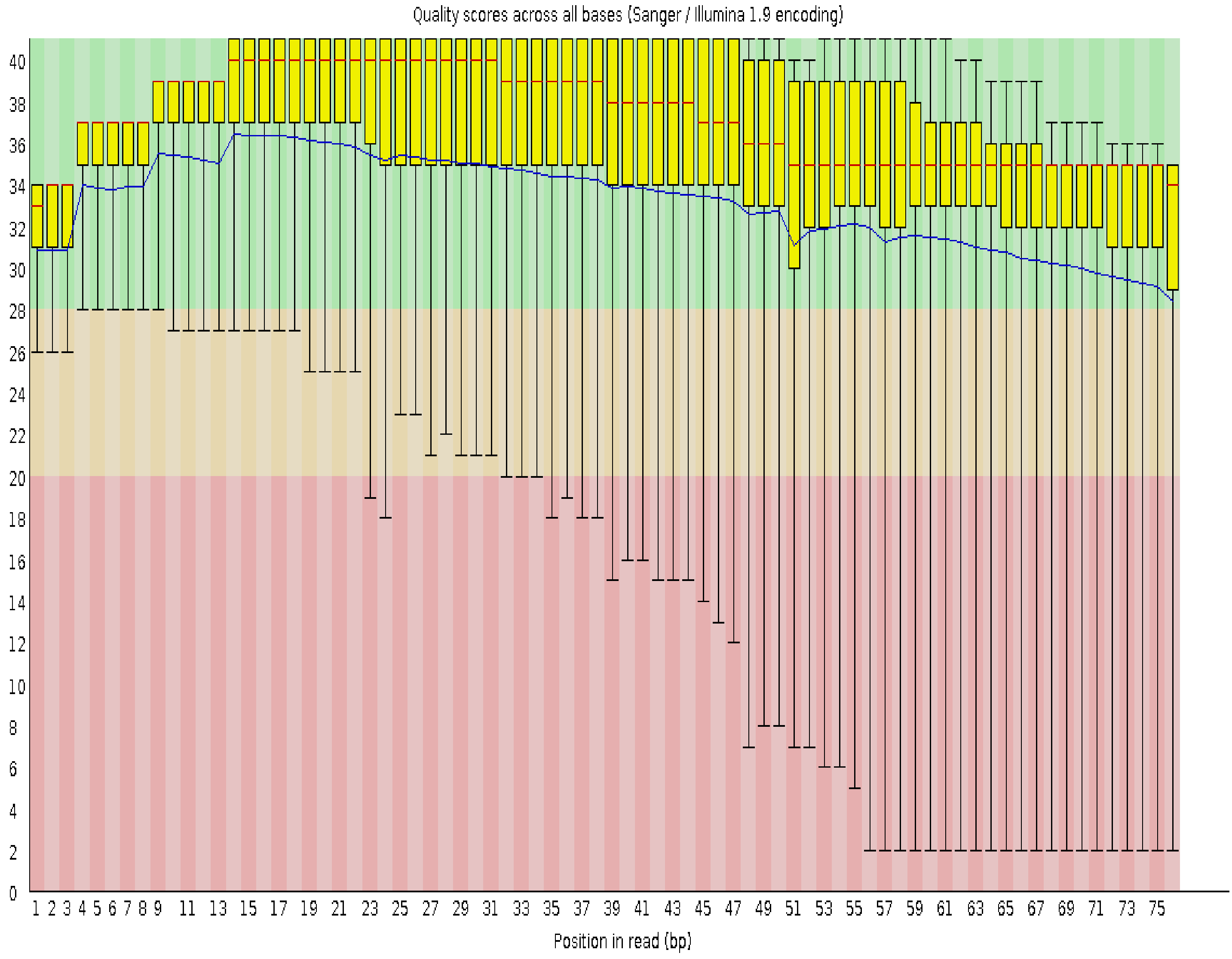


## Per tile sequence quality

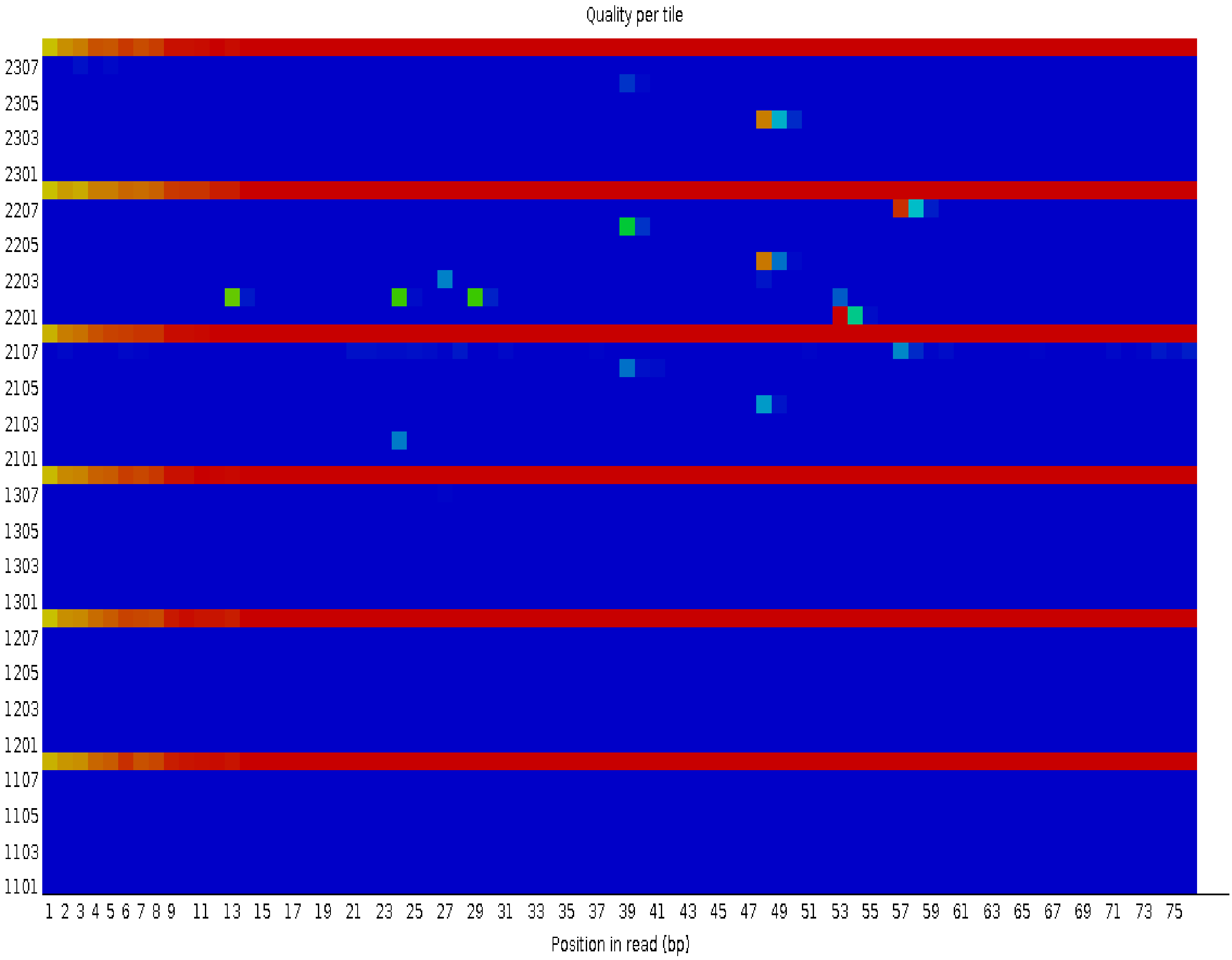


## Per sequence quality scores

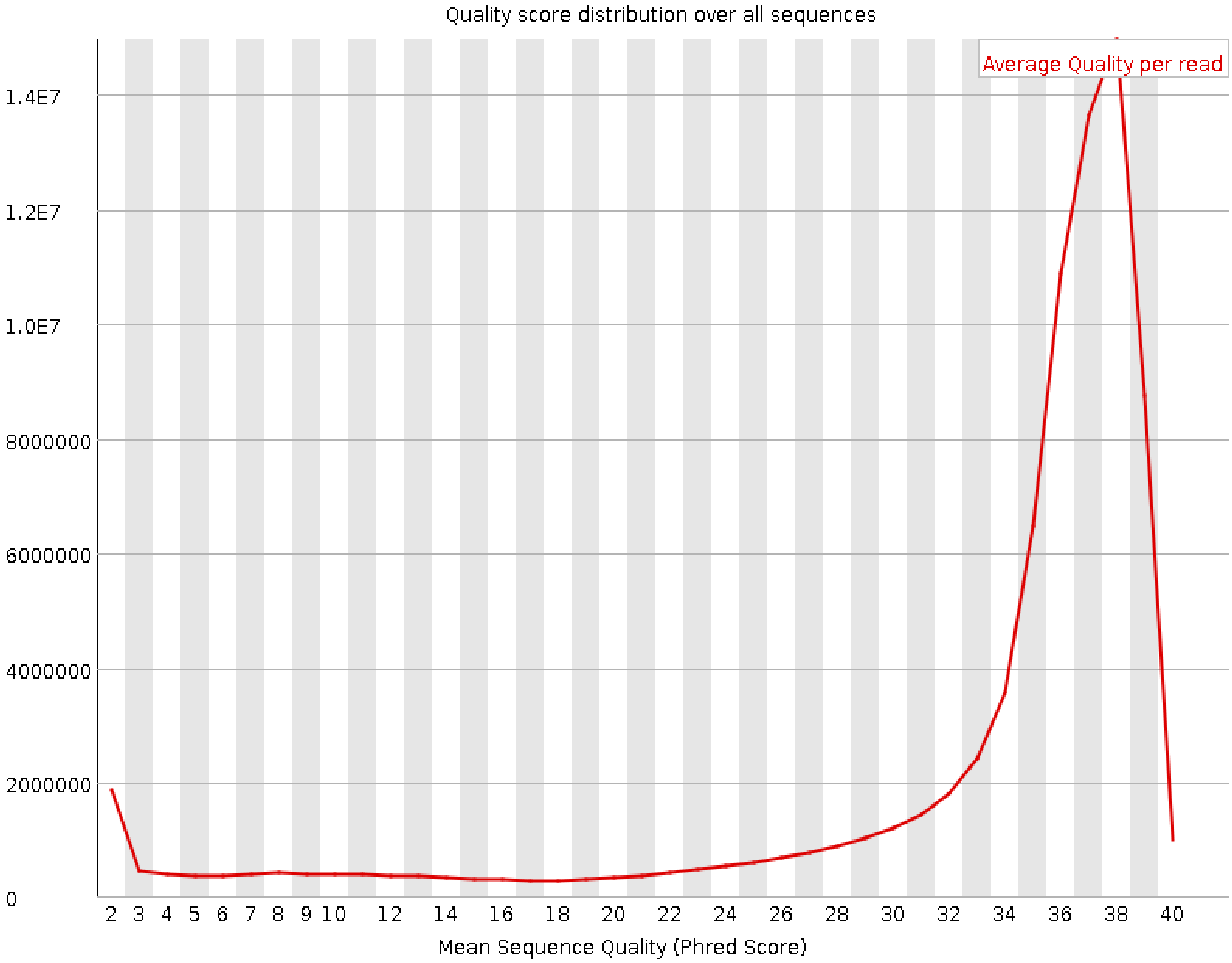


## Per base sequence content

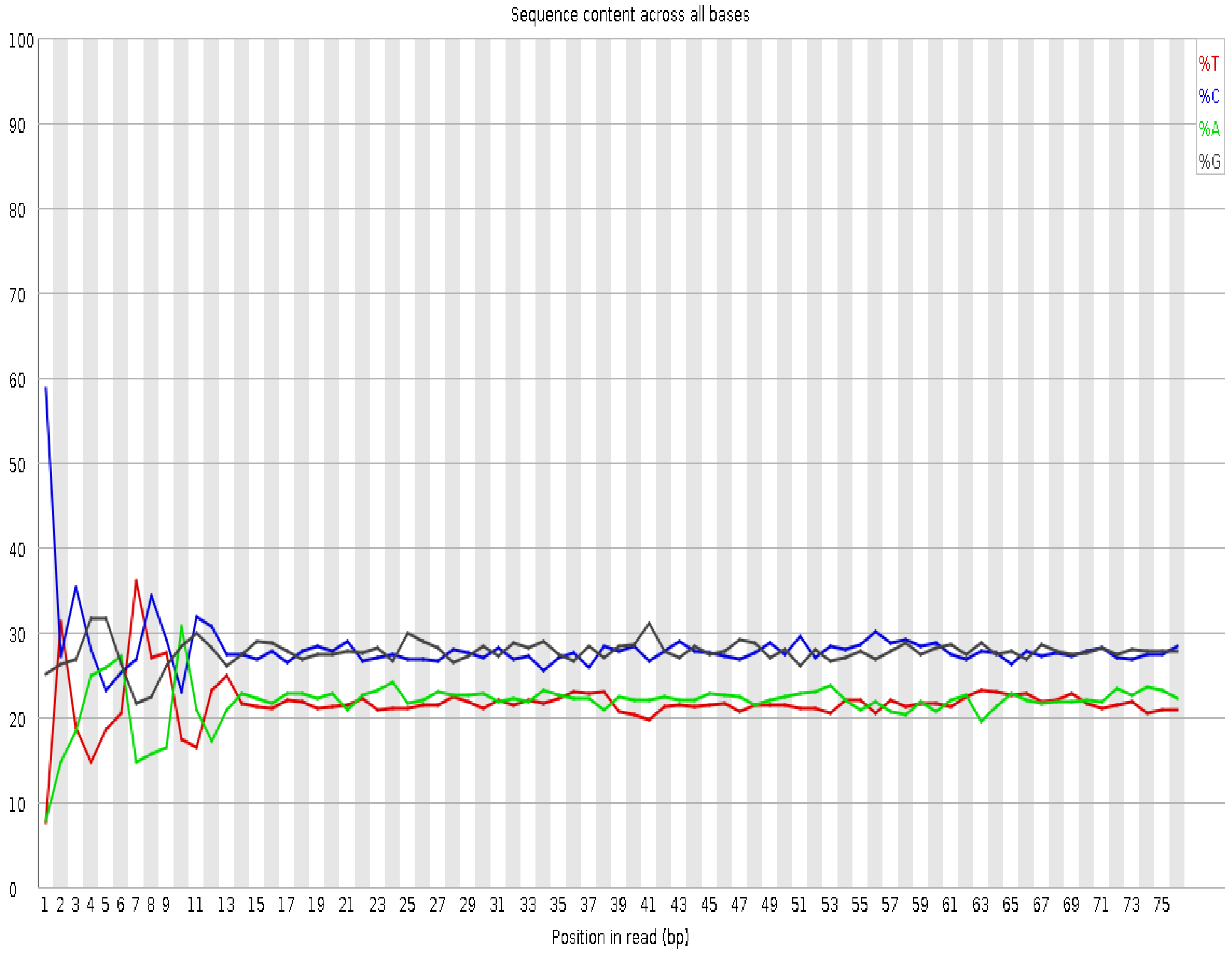


## Per sequence GC content

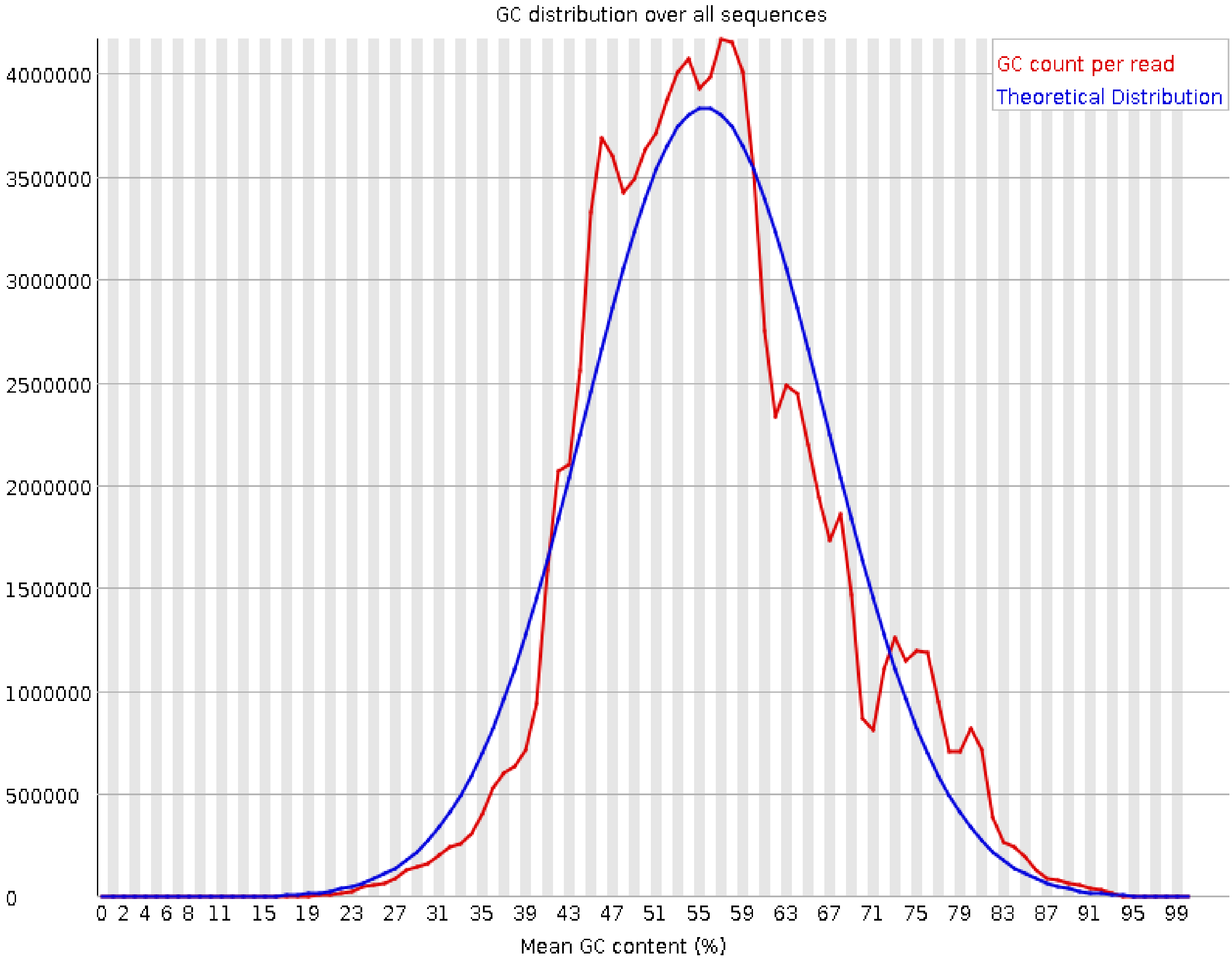


# Per base N content

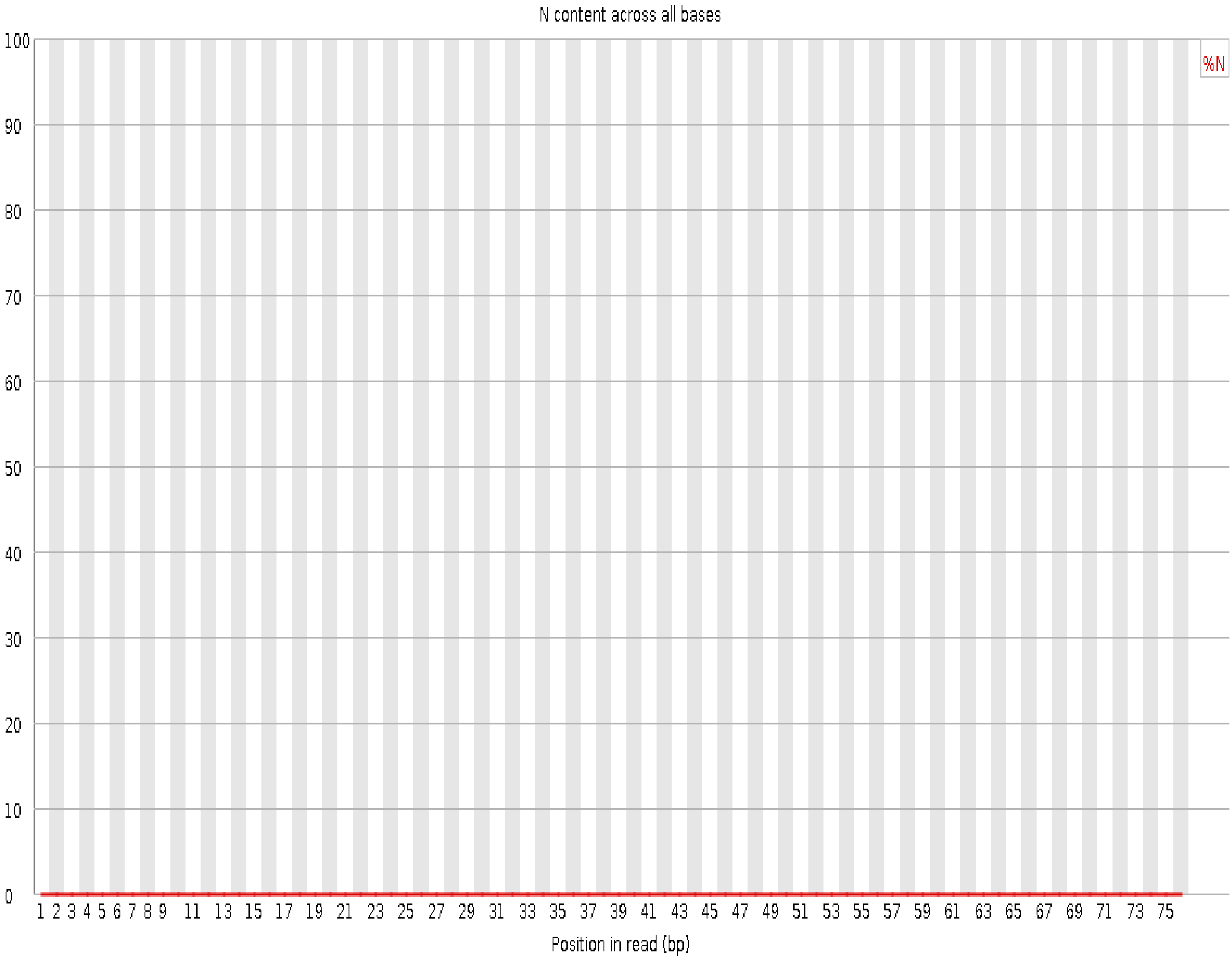


## Sequence Length Distribution

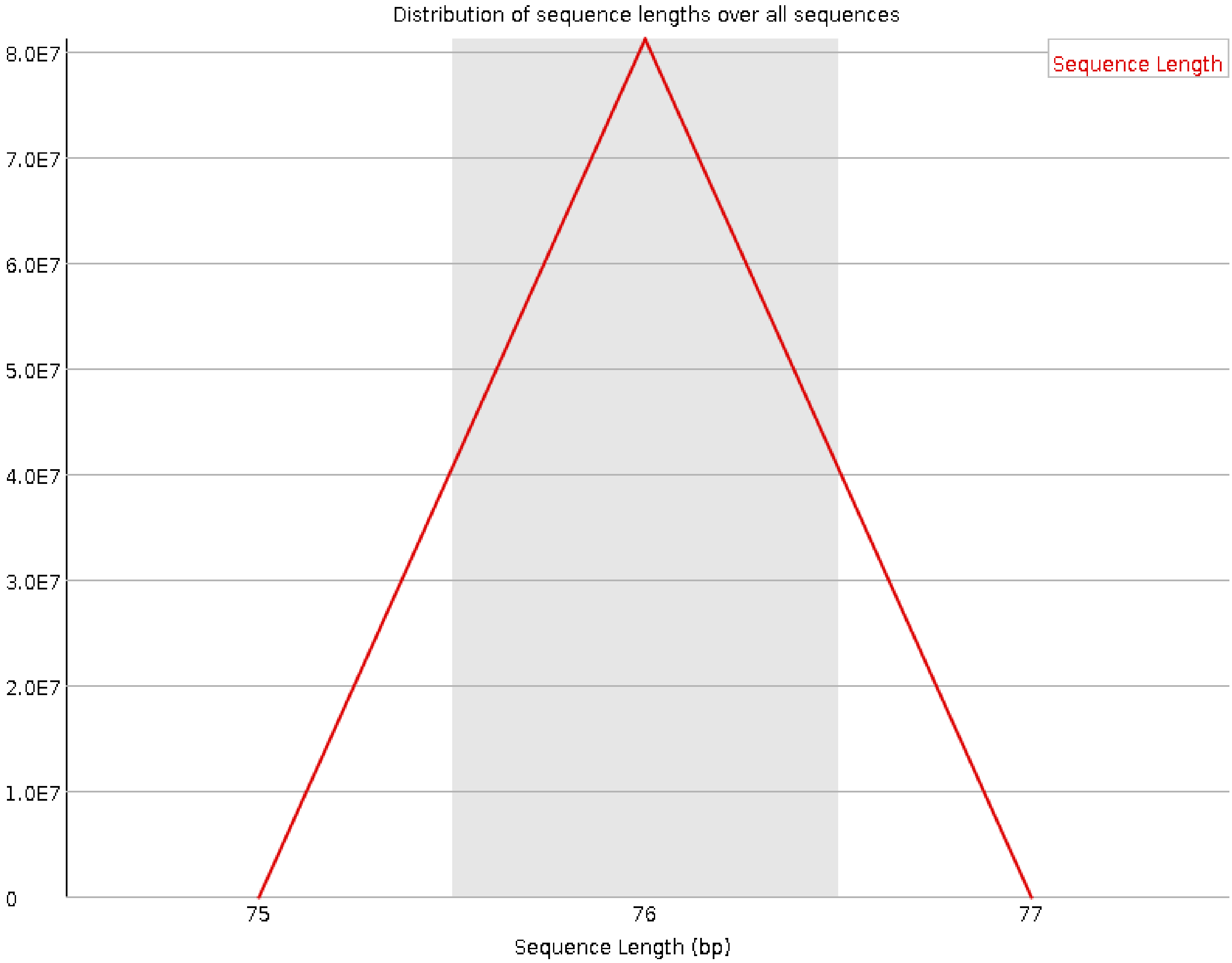


## Sequence Duplication Levels

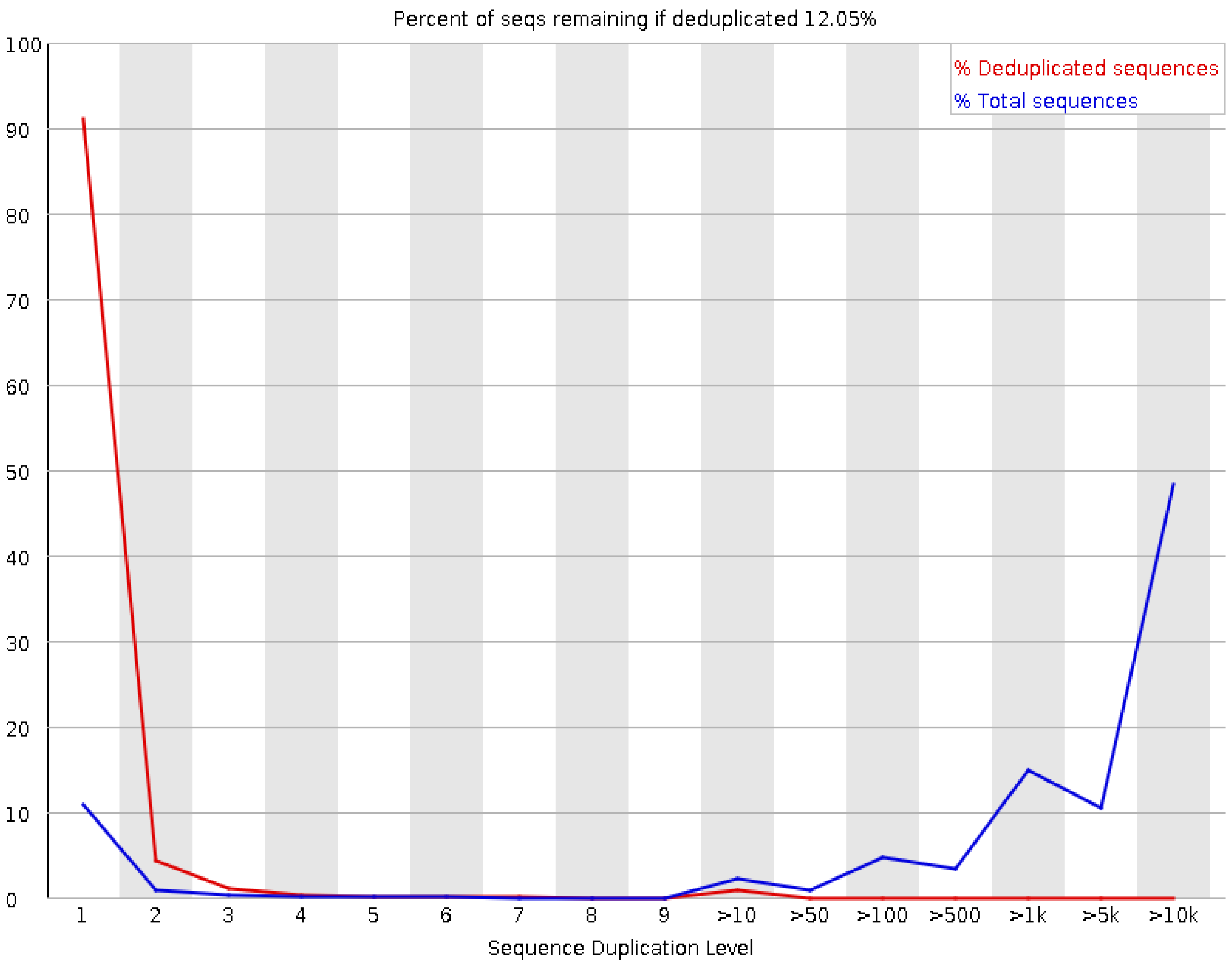


## Overrepresented sequences

| Sequence | Count | Percentage | Possible Source |
|---|---|---|---|
| CGCGTAACTAGTTAGCATGCCAGAGTCTCGTTCGTTATCGGAATTAACCA | 384673 | 0.4742978136480212 | No Hit |
| CCTCACCCGGCCCGGACACGGACAGGATTGACAGATTGATAGCTCTTTCT | 326751 | 0.40288058924672276 | No Hit |
| CGCAGTTTTATCCGGTAAAGCGAATGATTAGAGGTCTTGGGGCCGAAACG | 317092 | 0.39097114256856697 | No Hit |
| CTTCCGTACGCCACATGTCCCGCGCCCCGCCGCGGGGCGGGGATTCGGCG | 216583 | 0.2670445894911506 | No Hit |
| CCGGTATTTAGCCTTAGATGGAGTTTACCACCCGCTTTGGGCTGCATTCC | 179277 | 0.22104667896466948 | No Hit |
| CTCACCCGGCCCGGACACGGACAGGATTGACAGATTGATAGCTCTTTCTC | 178194 | 0.21971135121309657 | No Hit |
| CTTCACCGTGCCAGACTAGAGTCAAGCTCAACAGGGTCTTCTTTCCCCGC | 173803 | 0.21429729381960008 | No Hit |
| CTGGATAGTAGGTAGGGACAGTGGGAATCTCGTTCATCCATTCATGCGCG | 165391 | 0.20392538518965428 | No Hit |
| GTCAAAGTGAAGAAATTCAATGAAGCGCGGGTAAACGGCGGGAGTAACTA | 164687 | 0.203057360501651 2 | No Hit |
| CGCGATGTGATTTCTGCCCAGTGCTCTGAATGTCAAAGTGAAGAAATTCA | 161259 | 0.19883067210609076 | No Hit |
| CCCATATCCGCAGCAGGTCTCCAAGGTGAACAGCCTCTGGCATGTTGGAA | 153551 | 0.189326788164148 | No Hit |

| Sequence | Count | Percentage | Possible Source |
|---|---|---|---|
| CCCGAAGTTACGGATCCGGCTTGCCGACTTCCCTTACCTACATTGTTCCA | 149889 | 0.18481158019899566 | No Hit |
| CCCAGGCATAGTTCACCATCTTTCGGGTCCTAACACGTGCGCTCGTGCTC | 130587 | 0.16101241467650224 | [illegible] |
| CTCTCATGTCTCTTCACCGTGCCAGACTAGAGTCAAGCTCAACAGGGTCT | 130158 | 0.16048346213225037 | No Hit |
| CTCCGACTTTCGTTCTTGATTAATGAAAACATTCTTGGCAAATGCTTTCG | 128657 | 0.15863274472217564 | No Hit |
| CTCGCATTCCACGCCCGGCTCCACGCCAGCGAGCCGGGCTTCTTACCCAT | 127450 | 0.15714452625851125 | No Hit |
| GTAAATCTCGCGCCGGGCCGTACCCATATCCGCAGCAGGTCTCCAAGGTG | 126815 | 0.1563615778538494 | No Hit |
| CCCGGGGCTCCCGCCGGCTTCTCCGGGATCGGTCGCGTTACCGCACTGGA | 124673 | 0.15372051410143098 | No Hit |
| CTCCCTTTCGATCGGCCGAGGGCAACGGAGGCCATCGCCCGTCCCTTCGG | 124277 | 0.15323225021442924 | No Hit |
| CGGGTCTTCCGTACGCCACATGTCCCGCGCCCCGCCGCGGGGCGGGGATT | 122821 | 0.15143701733696832 | No Hit |
| CTCTGGTCCGTCTTGCGCCGGTCCAAGAATTTCACCTCTAGCGGCGCAAT | 120059 | 0.14803150002409263 | No Hit |
| CCCACTTATTCTACACCTCTCATGTCTCTTCACCGTGCCAGACTAGAGTC | 117420 | 0.14477764043369473 | No Hit |
| CTCTCTTCAAAGTTCTTTTCAACTTTCCCTTACGGTACTTGTTGACTATC | 116106 | 0.1431574920813708 | No Hit |
| CTCGATCAGAAGGACTTGGGCCCCCCACGAGCGGCGCCGGGGAGCGGGTC | 111870 | 0.13793454807798866 | No Hit |
| CCACTCTCGACTGCCGGCGACGGCCGGGTATGGGCCCGACGCTCCAGCGC | 111099 | 0.13698391308587166 | No Hit |
| CGCGTCACTAATTAGATGACGAGGCATTTGGCTACCTTAAGAGAGTCATA | 110049 | 0.1356892739915489 | No Hit |
| GGGATCCCGAGGCCTCTCCAGTCCGCCGAGGGCGCACCACCGGCCCGTCT | 108303 | 0.13353647412613218 | No Hit |
| CCGACATCGAAGGATCAAAAAGCGACGTCGCTATGAACGCTTGGCCGCCA | 107395 | 0.1324169195569464 | No Hit |
| CTGAATTTAAGCATATTAGTCAGCGGAGGAGAAGAAACTAACCAGGATTC | 104634 | 0.12901263523368434 | No Hit |
| CTTGAACTCTCTCTTCAAAGTTCTTTTCAACTTTCCCTTACGGTACTTGT | 102240 | 0.1260608580986284 | No Hit |
| CTTTAAATGGGTAAGAAGCCCGGCTCGCTGGCGTGGAGCCGGGCGTGGAA | 102139 | 0.1259363261476507 | No Hit |
| CACGAGCGCACGTGTTAGGACCCGAAAGATGGTGAACTATGCCTGGGCAG | 100075 | 0.12339143558509624 | No Hit |
| CTCCGCCACTCCGGATTCGGGGATCTGAACCCGACTCCCTTTCGATCGGC | 98495 | 0.12144331199554388 | No Hit |
| CGAAGGCCCGCGGCGGGTGTTGACGCGATGTGATTTCTGCCCAGTGCTCT | 97807 | 0.12059501514135905 | No Hit |
| CACCCGTTTACCTCTTAACGGTTTCACGCCCTCTTGAACTCTCTCTTCAA | 96713 | 0.1192461245040361 | No Hit |
| CTCCCACTTATTCTACACCTCTCATGTCTCTTCACCGTGCCAGACTAGAG | 94668 | 0.1167246607441408 | No Hit |
| CTGCTGTCTATATCAACCAACACCTTTTCTGGGGTCTGATGAGCGTCGGC | 94621 | 0.11666671023229967 | No Hit |
| GTCGGGTCTGCGAGAGCGCCAGCTATCCTGAGGGAAACTTCGGAGGGAAC | 93818 | 0.11567661957254617 | No Hit |
| CCTATACCCAGGTCGGACGACCGATTTGCACGTCAGGACCGCTACGGACC | 93789 | 0.11564086287375058 | No Hit |
| GCCCTCTTGAACTCTCTCTTCAAAGTTCTTTTCAACTTTCCCTTACGGTA | 93703 | 0.11553482576697749 | No Hit |
| CTGCCAGTAGCATATGCTTGTCTCAAAGATTAAGCCATGCATGTCTAAGT | 92652 | 0.11423895368304109 | No Hit |
| CTCCCGTCCACTCTCGACTGCCGGCGACGGCCGGGTATGGGCCCGACGCT | 92297 | 0.11380124237019862 | No Hit |
| CCCCGCTTCGCGCCCCAGCCCGACCGACCCAGCCCTTAGAGCCAATCCTT | 91950 | 0.11337339497426528 | No Hit |
| CGAACGCCGGGTTAAGGCGCCCGATGCCGACGCTCATCAGACCCCAGAAA | 90368 | 0.11142280540548566 | No Hit |
| CGCTGATTCCGCCAAGCCCGTTCCCTTGGCTGTGGTTTCGCTGGATAGTA | 90059 | 0.11104181161487067 | No Hit |
| CTTAGAGCCAATCCTTATCCCGAAGTTACGGATCCGGCTTGCCGACTTCC | 89306 | 0.11011337043579919 | No Hit |
| CTCATGTCTCTTCACCGTGCCAGACTAGAGTCAAGCTCAACAGGGTCTTC | 89063 | 0.1098137539596845 | No Hit |
| CTTCCGTCAATTCCTTTAAGTTTCAGCTTTGCAACCATACTCCCCCCGGA | 87981 | 0.10847965919772522 | No Hit |
| CCACCGTCCTGCTGTCTATATCAACCAACACCTTTTCTGGGGTCTGATGA | 87684 | 0.10811346128247391 | No Hit |
| CCCGTCGGCATGTATTAGCTCTAGAATTACCACAGTTATCCAAGTAGGAG | 87420 | 0.10778795202447276 | No Hit |

| Sequence | Count | Percentage | Possible Source |
|---|---|---|---|
| GGAAACTCTGGTGGAGGTCCGTAGCGGTCCTGACGTGCAAATCGGTCGTC | 87136 | 0.1074377829741988 | No Hit |
| CAGAAACCTCCCGTGGAGCA[illegible]AGCTCGCTTGATCTTGATTT | [illegible] | 0.10[illegible]8334 | No Hit |
| GTTTTATCCGGTAAAGCGAATGATTAGAGGTCTTGGGGCCGAAACGATCT | 86495 | 0.1066474366318551 | No Hit |
| GTGGGATCCCGAGGCCTCTCCAGTCCGCCGAGGGCGCACCACCGGCCCGT | 86324 | 0.10643659540792254 | No Hit |
| CGGGTCTGCGAGAGCGCCAGCTATCCTGAGGGAAACTTCGGAGGGAACCA | 85879 | 0.1058879150298524 | No Hit |
| CAAACTTTAAATGGGTAAGAAGCCCGGCTCGCTGGCGTGGAGCCGGGCGT | 82329 | 0.1015108019014278 | No Hit |

## Adapter Content

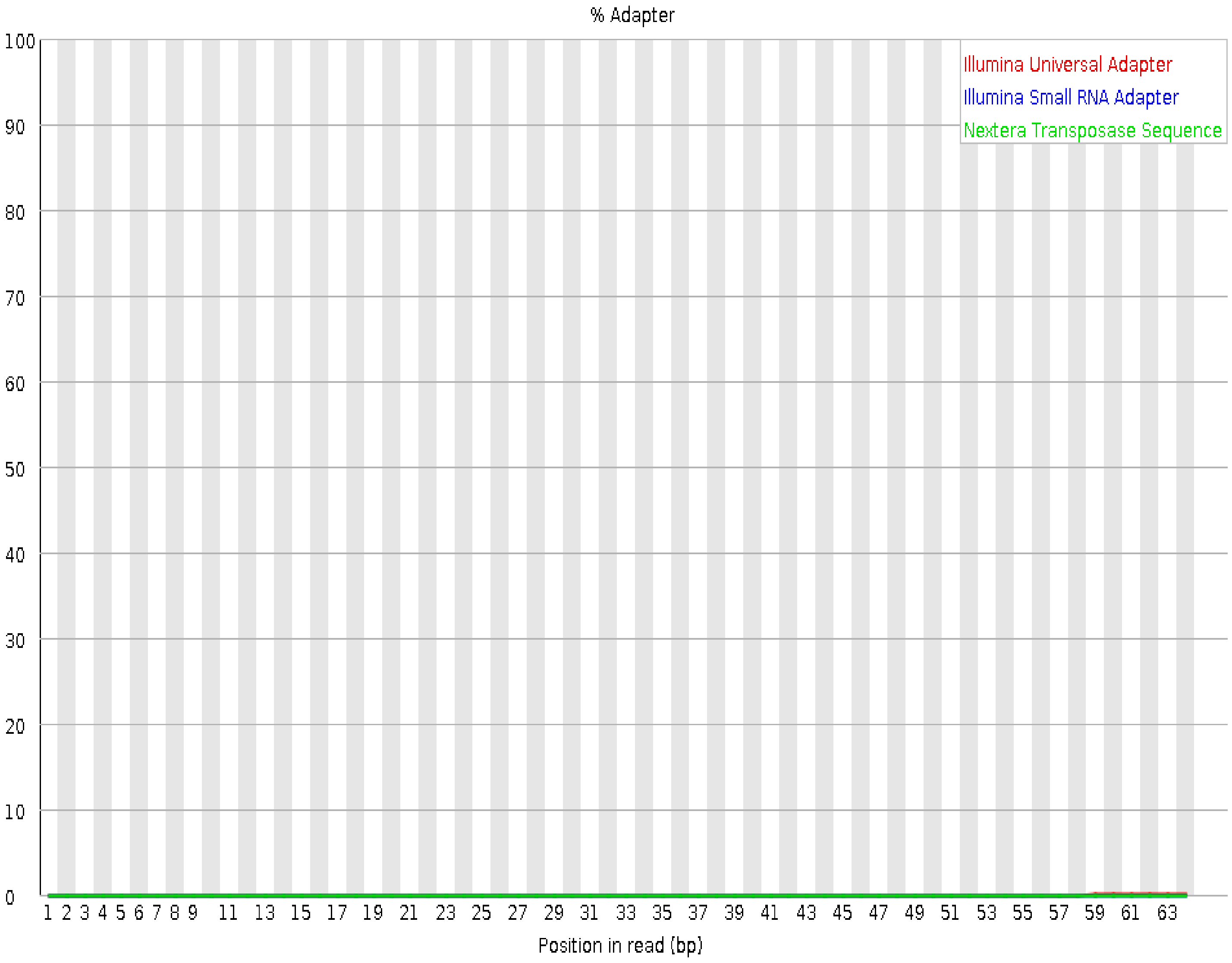


## Kmer Content

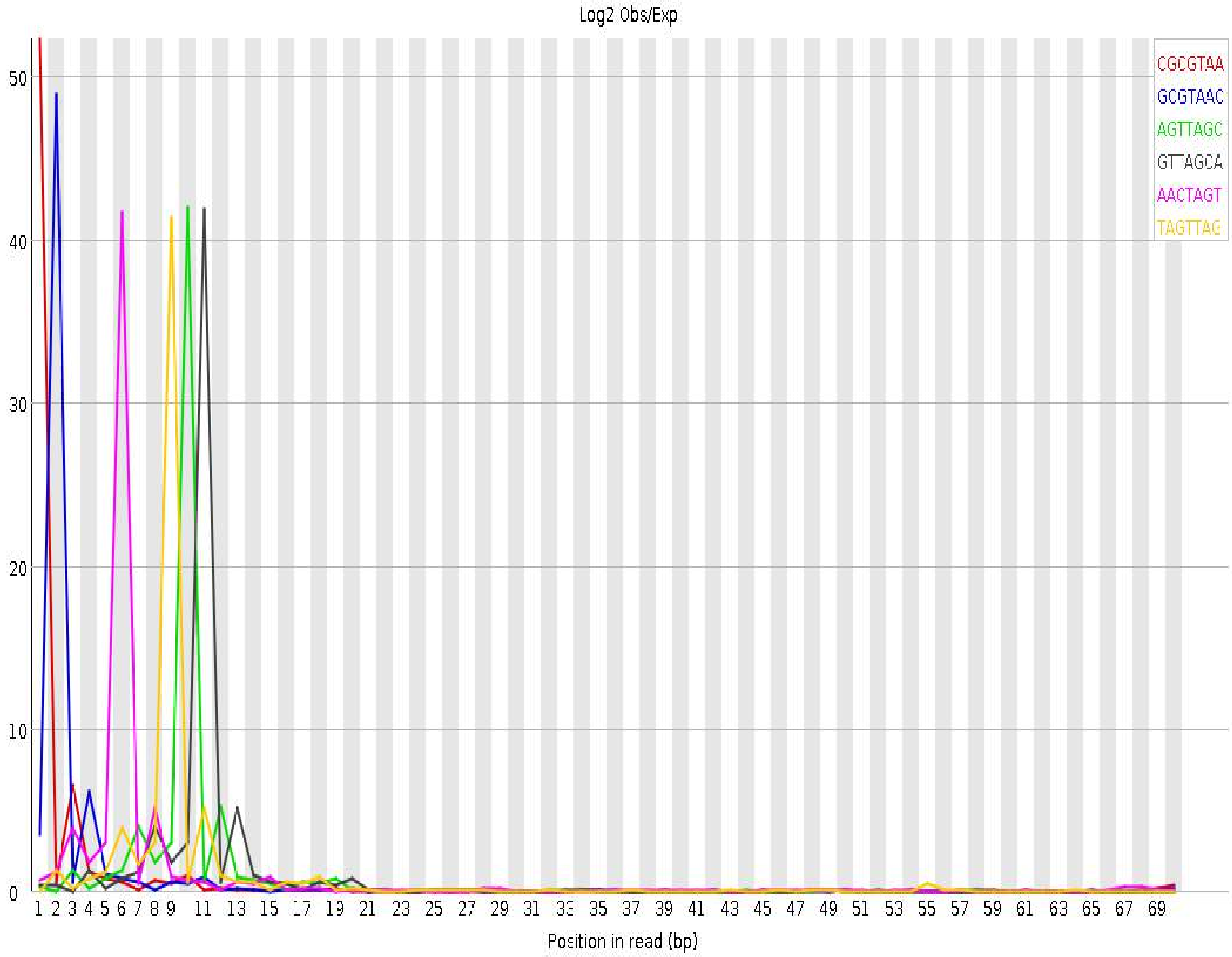


| Sequence | Count | PValue | Obs/Exp Max | Max Obs/Exp Position |
|---|---|---|---|---|
| CGCGTAA | 62830 | 0.0 | 52.306644 | 1 |
| GCGTAAC | 67555 | 0.0 | 48.9687 | 2 |
| AGTTAGC | 78440 | 0.0 | 42.028107 | 10 |
| GTTAGCA | 78675 | 0.0 | 41.894184 | 11 |
| AACTAGT | 80185 | 0.0 | 41.652546 | 6 |
| TAGTTAG | 80230 | 0.0 | 41.411804 | 9 |
| ACTAGTT | 81215 | 0.0 | 41.051834 | 7 |
| CGTAACT | 81160 | 0.0 | 40.806137 | 3 |
| TAGCATG | 85270 | 0.0 | 38.378105 | 13 |
| AGCATGC | 86115 | 0.0 | 38.262016 | 14 |
| TTAGCAT | 86925 | 0.0 | 37.928173 | 12 |
| TAACTAG | 88270 | 0.0 | 37.753674 | 5 |
| GCATGCC | 88140 | 0.0 | 37.307716 | 15 |
| TCAGACG | 15060 | 0.0 | 35.990025 | 2 |
| TGTGTCG | 6425 | 0.0 | 35.911583 | 3 |

| Sequence | Count | PValue | Obs/Exp Max | Max Obs/Exp Position |
|---|---|---|---|---|
| CTAGTTA | 93790 | 0.0 | 35.[illegible] | 8 |
| TCACTCG | 51315 | 0.0 | 35.04[illegible] | 67 |
| TCGCCTA | 5460 | 0.0 | 33.5847 | 66 |
| GTGTCGA | 7135 | 0.0 | 33.465813 | 4 |
| AGTCTCG | 96795 | 0.0 | 33.213882 | 24 |

**Produced by FastQC (version 0.11.2)**
