# Supplementary material for: A resource of ribosomal RNA-depleted RNA-Seq data from different normal adult and fetal human tissues
Source: Sci Data. 2015 Nov 10;2:150063. doi: 10.1038/sdata.2015.63 (PMC4640133; doi:10.1038/sdata.2015.63)
Supplement: Supplementary File 4 [file sdata201563-s5.pdf]

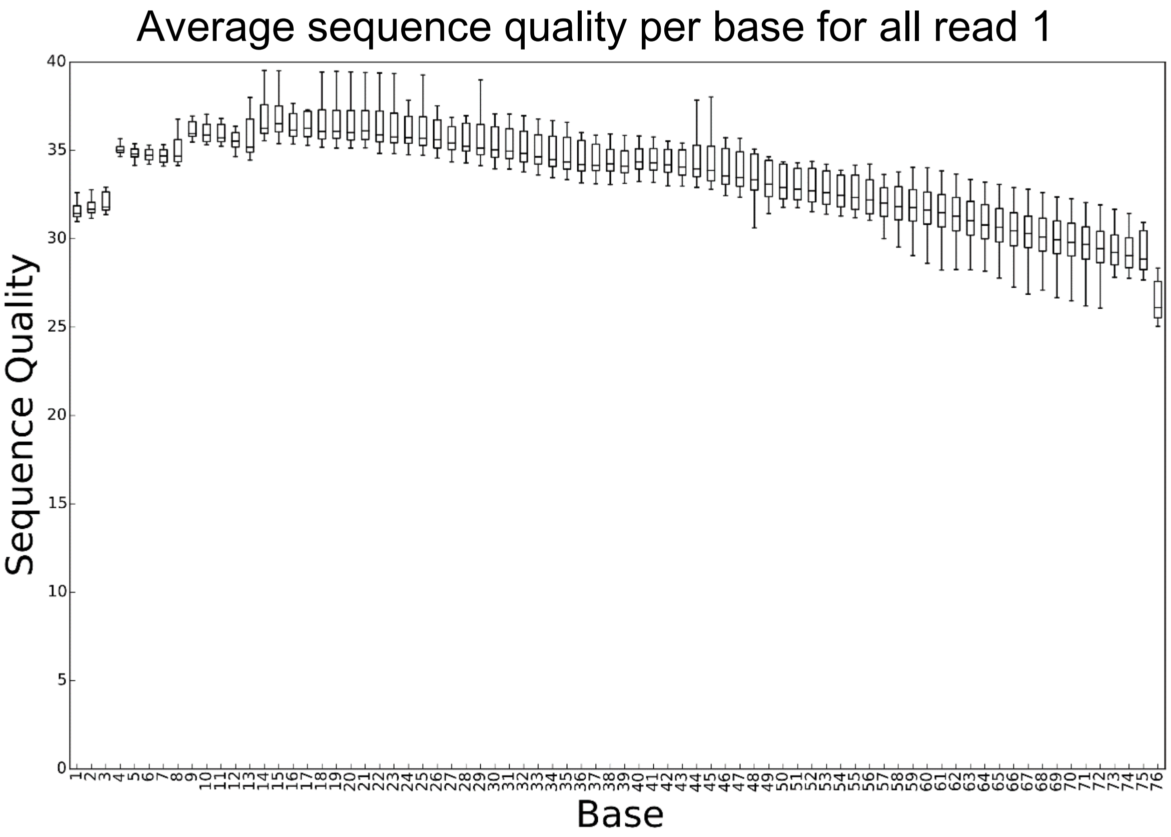

Figure S1: A boxplot of the average sequence quality per base for read 1 of all libraries. The average sequence quality was calculated from FastQC quality scores of all libraries.

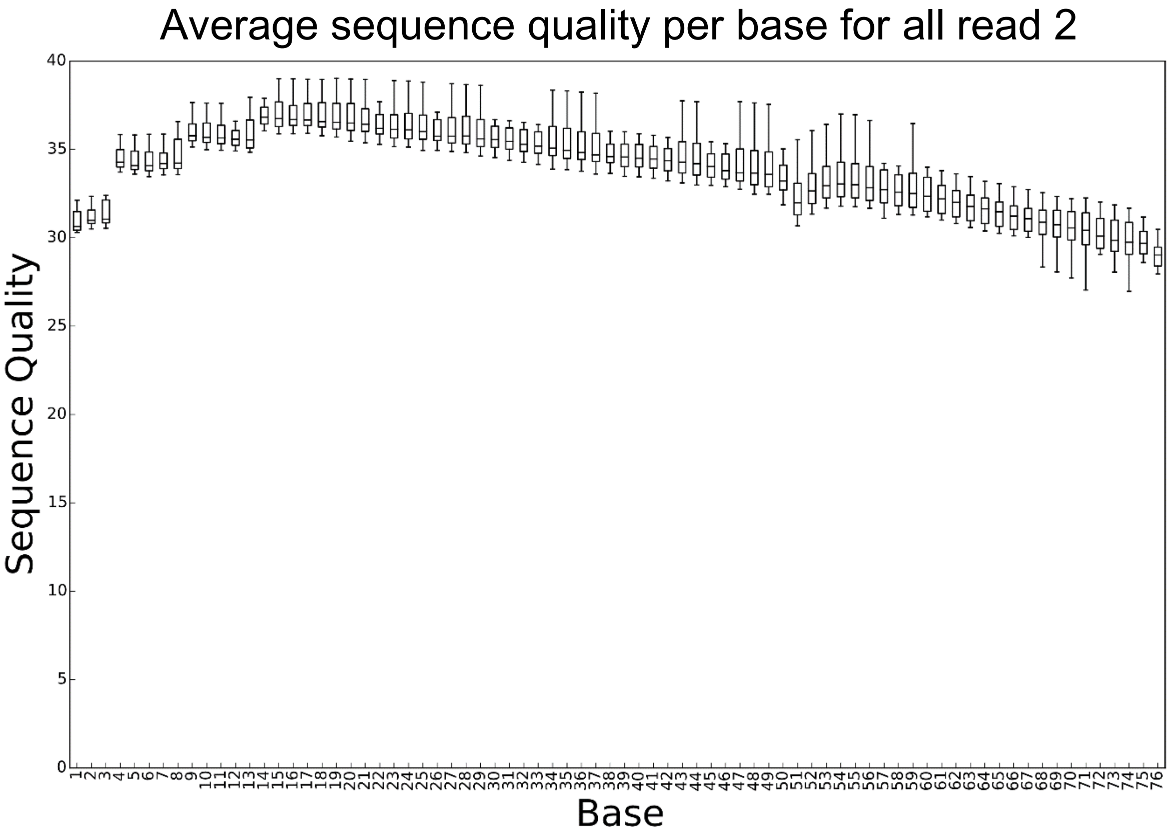

Figure S2: A boxplot of the average sequence quality per base for read 2 of all libraries. The average sequence quality was calculated from FastQC quality scores of all libraries.

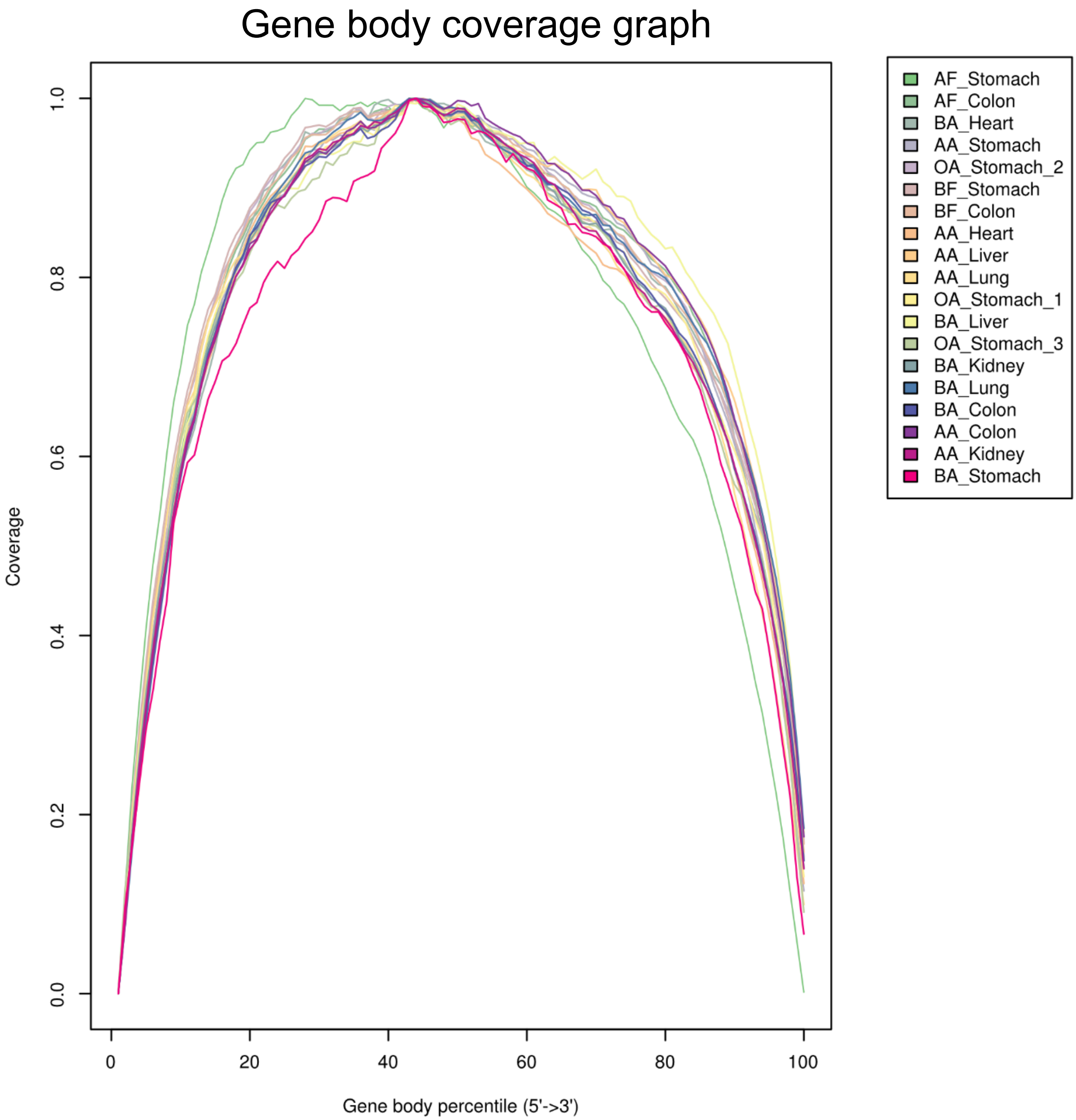

Figure S3: Graph of the gene body coverage of all libraries. The bulk of the reads map within the 20th to 80th percentile of all genes. Indicating an absence of 5' or 3' bias in the reads. The graph was generated using the aligned reads for each library and inputted into the geneBodyCoverage.py script from the RseQC package.

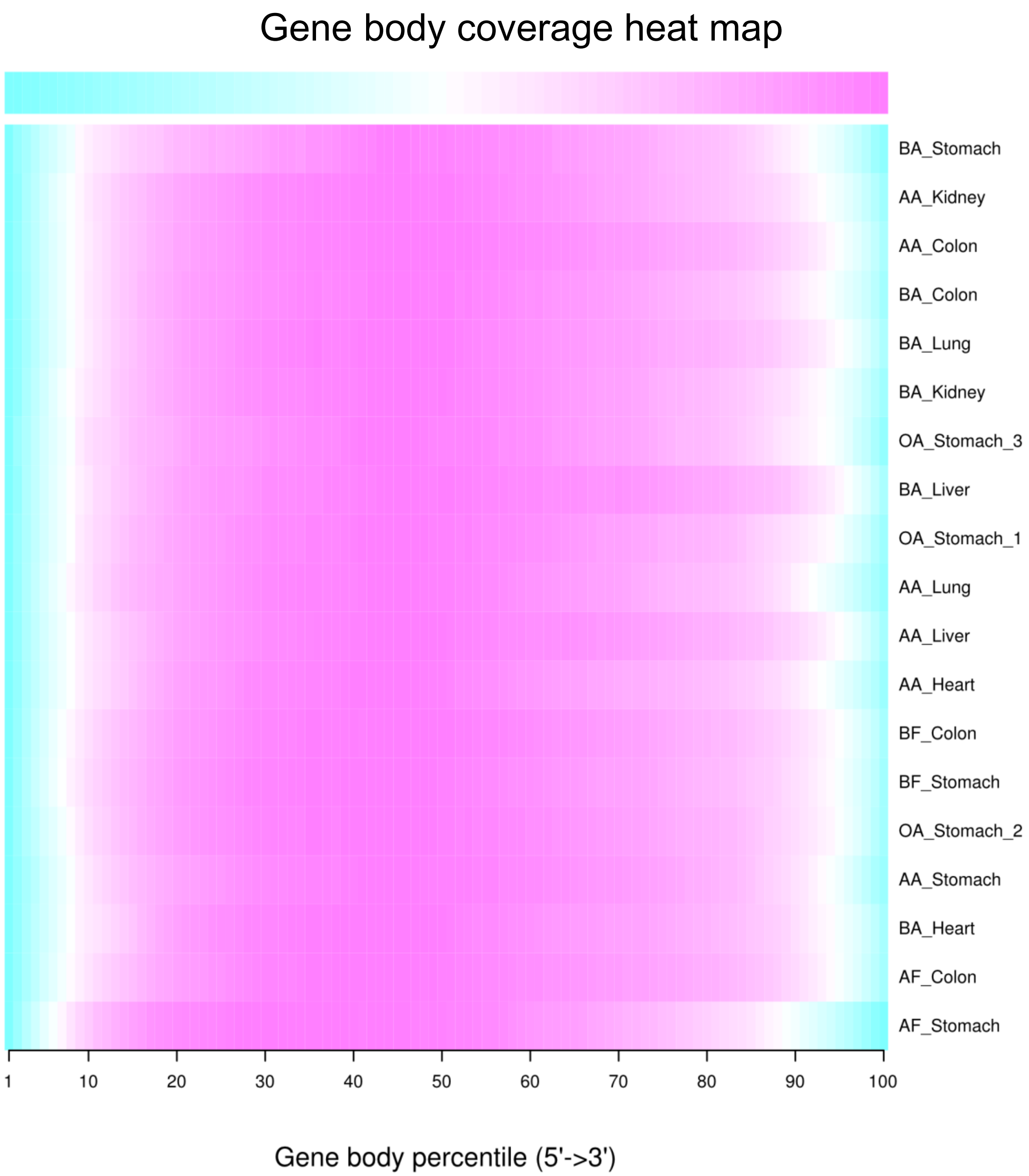

Figure S4: A heat map of the gene body coverage of all aligned reads from each library. The majority of the reads fall within the 40th to 60th percentile. The Agilent Fetal Stomach library shows a slight skew to the 5' end with the majority of the reads falling within the 30th to 40th percentile. The heat map was generated using the geneBodyCoverage.py script from the RseQC package.
